# Differentiating Staphylococcus infection-associated glomerulonephritis and primary IgA nephropathy: a mass spectrometry-based exploratory study

**DOI:** 10.1038/s41598-020-73847-x

**Published:** 2020-10-14

**Authors:** Anjali A. Satoskar, John P. Shapiro, Mikayla Jones, Cherri Bott, Samir V. Parikh, Sergey V. Brodsky, Lianbo Yu, Haikady N. Nagaraja, Daniel W. Wilkey, Michael L. Merchant, Jon B. Klein, Tibor Nadasdy, Brad H. Rovin

**Affiliations:** 1 Division of Renal and Transplant Pathology, Department of Pathology, The Ohio State University Wexner Medical Center, 320 W. 10th Avenue, M015/018 Starling Loving Hall, Columbus, OH 43210 USA; 2 Division of Nephrology, Department of Internal Medicine, Ground Floor, The Ohio State University Wexner Medical Center, 395 West 12th Avenue, Columbus, OH 43210 USA; 3 Center for Biomedical Informatics, The Ohio State University, Columbus, USA; 4 Division of Biostatistics, The Ohio State University College of Public Health, Columbus, USA; 5 Division of Nephrology and Hypertension, Department of Medicine, University of Louisville, Louisville, USA

**Keywords:** Biological techniques, Biomarkers, Diseases, Health care, Medical research, Nephrology

## Abstract

Staphylococcus infection-associated glomerulonephritis (SAGN) and primary IgA nephropathy (IgAN) are separate disease entities requiring different treatment approaches. However, overlapping histologic features may cause a diagnostic dilemma. An exploratory proteomic study to identify potential distinguishing biomarkers was performed on formalin fixed paraffin embedded kidney biopsy tissue, using mass spectrometry (HPLC–MS/MS) (n = 27) and immunohistochemistry (IHC) (n = 64), on four main diagnostic groups—SAGN, primary IgAN, acute tubular necrosis (ATN) and normal kidney (baseline transplant biopsies). Spectral counts modeled as a negative binomial distribution were used for statistical comparisons and in silico pathway analysis. Analysis of variance techniques were used to compare groups and the ROC curve to evaluate classification algorithms. The glomerular proteomes of SAGN and IgAN showed remarkable similarities, except for significantly higher levels of monocyte/macrophage proteins in SAGN—mainly lysozyme and S100A9. This finding was confirmed by IHC. In contrast, the tubulointerstitial proteomes were markedly different in IgAN and SAGN, with a lower abundance of metabolic pathway proteins and a higher abundance of extracellular matrix proteins in SAGN. The stress protein transglutaminase-2 (TGM2) was also significantly higher in SAGN. IHC of differentially-expressed glomerular and tubulointerstitial proteins can be used to help discriminate between SAGN and IgAN in ambiguous cases.

## Introduction

Staphylococcal infections have emerged as the most common cause of infection-associated glomerulonephritis in the elderly population and are also encountered with increasing frequency in young adults with a history of intravenous drug abuse^1–7^. Kidney biopsies from patients with Staphylococcal infection-associated glomerulonephritis (SAGN) frequently show glomerular IgA and co-dominant C3 staining with predominantly mesangial and a few capillary wall immune-type deposits. The pattern of staining is very similar to that seen in primary IgA nephropathy (IgAN), presenting a potential diagnostic pitfall. IgA vasculitis (Henoch–Schönlein purpura or HSP) can also show similar biopsy features, and 15–20% of patients with SAGN have a leukocytoclastic vasculitic rash, mimicking IgA vasculitis^8,9^. Making the correct diagnosis is important as different treatment approaches are warranted for SAGN and IgAN. Because of active infection, patients with SAGN should be treated with antibiotics, and immunosuppressive therapy should be avoided, at least in early stages of the disease. In contrast and depending on kidney function, level of proteinuria, and disease activity, primary IgAN is often treated with corticosteroids and/or immunosuppressive drugs. This is especially true for IgAN cases with crescents (Oxford score C1 or C2) and endocapillary hypercellularity (Oxford score E1)^10–13^. Histologically, such cases of IgAN can resemble SAGN, and while clinical presentation of SAGN such as older age, rapidity of disease onset, recent Staphylococcal infection, presence of risk factors for infection can together help in differentiating the two diseases, it can still be challenging since not all these features are consistently present in every case^7–9^.

We and others have previously demonstrated the diagnostic utility of mass spectrometry-based proteomic analysis on formalin-fixed paraffin-embedded (FFPE) tissue from kidney biopsies. Studies have also shown comparable peptide capture using both frozen and FFPE tissue^14–19^. In this exploratory study, we used liquid chromatography and tandem mass spectrometry (LC–MS/MS)-based proteomics on FFPE biopsy samples to identify differentially-expressed proteins between SAGN and primary IgAN in an effort to shed light on the pathogenesis and to identify potential diagnostic biomarkers to help differentiate SAGN from primary IgAN on a kidney biopsy. Laser microdissection was used to collect glomeruli and tubulointerstitial tissue separately for analysis.

## Results

### Classification of biopsies used for the study

Biopsies were identified from our Pathology database and from our previously published cohort of SAGN^3,6,9^. A total of 27 biopsies were used for LC–MS/MS (Tables 1, 2). These included SAGN (n = 4), IgAN (n = 7), living donor baseline transplant biopsies (n = 8) as healthy control kidney tissue, and biopsies with a primary diagnosis of acute tubular necrosis (ATN, n = 8). The ATN group was included for comparison since SAGN biopsies typically do show acute tubular injury^1,6^. For the SAGN group, we included biopsies from patients that had culture-proven Staphylococcal infection, and excluded biopsies that showed changes of underlying diabetic glomerulosclerosis. Also cases with more than moderate interstitial fibrosis and tubular atrophy were excluded to focus specifically on active SAGN as opposed to chronic renal injury. The IgAN group contained biopsies with MEST Score M1 E0 C0, (n = 3); MEST Score M1 E1 C0, (n = 2) and cases with MEST Score M1 E1 C1, (n = 2).

**Table 1:** SAGN and IgAN cases for mass spectrometry analysis.

Disease category	Age (years)	Ethnicity	Sex	S. cr	Urine protein	Urine RBCs/hpf	Crescents	Endocapillary proliferative lesions	IF/TA (%)	IgG	IgA	C3	EM deposits
SAGN	48	C	M	2.3	30 mg/dl	10–14	Present	Focal	30	Trace	1 + m	1 +	Paramesangial
SAGN	58	AA	M	1.5	140 mg/dl	10–14	Absent	Diffuse	20	1 + m, g	1 + m	2 +	Mesangial, few humps
SAGN	44	C	F	2	300 mg/dl	> 50	Present	Diffuse	< 10	1 + m, g	2 + m, g	1 + m, g	Mesangial, intra-membranous
SAGN	51	C	M	2.1	Trace	3–4	Absent	Focal	< 10	Trace	2 + m, g	2 + m	Mesangial
IgAN M1 E0 S1 T0 C0	24	Asian	M	1	1.6 g/d	15–19	Absent	Absent	Absent	Trace	2 + m	trace	Mesangial
IgAN M1 E0 S1 T1 C0	29	C	M	0.8	0.5 g/d	5–9	Absent	Absent	25	Trace m	2 + m	2 + m	Mesangial
IgAN M1 E0 S1 T0 C0	19	C	M	1.1	0.4 g/d	3+	Absent	Absent	10–15	1 + m	3 + m, g	2 + m, g	Mesangial, rare subendothelial
IgAN M1 E1S0 T0 C1	70	C	F	1.2	4 g/d	3+	Present	Focal	< 10	Absent	1 + m	2 + m	Mesangial
IgAN M1 E1S1 T0 C1	58	C	M	1	300 mg/dl	15–19	Present	Focal	< 10	Trace	2 + m	Trace	Mesangial
IgAN M1 E0 S1 T1 C1	42	Hispanic C	F	1	1.7 g/d	10–14	Present	Absent	25	Trace	3 + m	2 + m	Mesangial
IgAN M1 E0 S0 T1 C1	26	C	M	1.3	100 mg/dl	> 50	Present	Absent	< 10	1 +	3 + m	2 + m	Mesangial

*SAGN* Staphylococcus infection associated glomerulonephritis, *S.* *cr*. serum creatinine, *EM* electron microscopic, *m* mesangial, *g* glomerular capillary wall, *IF/TA* interstitial fibrosis and tubular atrophy, *HSP* Henoch–Schönlein purpura, *IgG* *IgA,* *C3* staining by immunofluorescence, *C* Caucasian, *AA* African American.

**Table 2:** Cases of vancomycin-induced ATN and ATN of other causes for mass spectrometry analysis. Baseline transplant biopsies are also shown.

Disease category	Age (years)	Ethnicity	Sex	Etiology of AKI	Antibotics	S. Cr	Urine protein	IF/TA
Vanc ATN	26	C	F	Cellulitis	Vancomycin, Keflex, Zosyn, Levofloxacin	8	2 g/d	Absent
Vanc ATN	31	C	M	Pneumonia, empyema	Vancomycin	4.5	1.6 g/d	Absent
Vanc ATN	60	AA	M	Osteomyelitis	Vancomycin, Zosyn	8.4	30 mg/dl	30%
Vanc ATN	63	C	M	Infected gangrenous toe	Vancomycin	2.7	Oligoanuria	Absent
ATN	58	Hispanic C	M	Cisplatin nephrotoxicity, dehydration	N/A	2.4	Oligoanuria	10%
ATN	72	AA	F	Gemzar, Cisplatin, intravenous contrast	N/A	4.4	100 mg/dl	10%
ATN	59	C	F	Hypotensive shock	N/A	5.7	Oligoanuria	Absent
ATN	45	AA	F	NSAIDs, antibiotics (Ciprofloxacin, Zosyn, Gentamycin)	N/A	9.9	Oligoanuria	Absent
Baseline Tx biopsy	58	C	F	LUD	N/A	N/A	N/A	N/A
Baseline Tx biopsy	63	C	F	LUD	N/A	N/A	N/A	N/A
Baseline Tx biopsy	60	C	M	LUD	N/A	N/A	N/A	N/A
Baseline Tx biopsy	45	C	M	LRD	N/A	N/A	N/A	N/A
Baseline Tx biopsy	51	A	M	LUD	N/A	N/A	N/A	N/A
Baseline Tx biopsy	62	C	F	LUD	N/A	N/A	N/A	N/A
Baseline Tx biopsy	63	AA	F	LUD	N/A	N/A	N/A	N/A
Baseline Tx biopsy	57	C	F	LUD	N/A	N/A	N/A	N/A

Vanc ATN- Vancomycin-induced acute tubular necrosis; S.Cr. = serum creatinine; IF/TA = interstitial fibrsis and tubular atrophy; AKI = acute kidney injury, C = Caucasian; AA = African American. C = Caucasian, AA = African American; LUD = living unrelated donor; LRD = living related donor.

As this study was done on tissue left-over after routine diagnostic processing, most biopsy samples were depleted after LC MS/MS analysis. Hence, for immunohistochemical (IHC) staining, additional biopsies were retrieved from our Pathology database, including SAGN (n = 28), IgAN (n = 36; 17 biopsies with MEST score E0 and 19 biopsies with MEST score E1) and ATN (n = 6). Among the 28 SAGN biopsies, 22 showed endocapillary proliferative lesions, 11 had focal crescents. Among the 19 biopsies with IgAN MEST score E1, 14 had focal glomerular crescents.

### LC–MS/MS

#### Glomerular compartment

**Figure 1:**
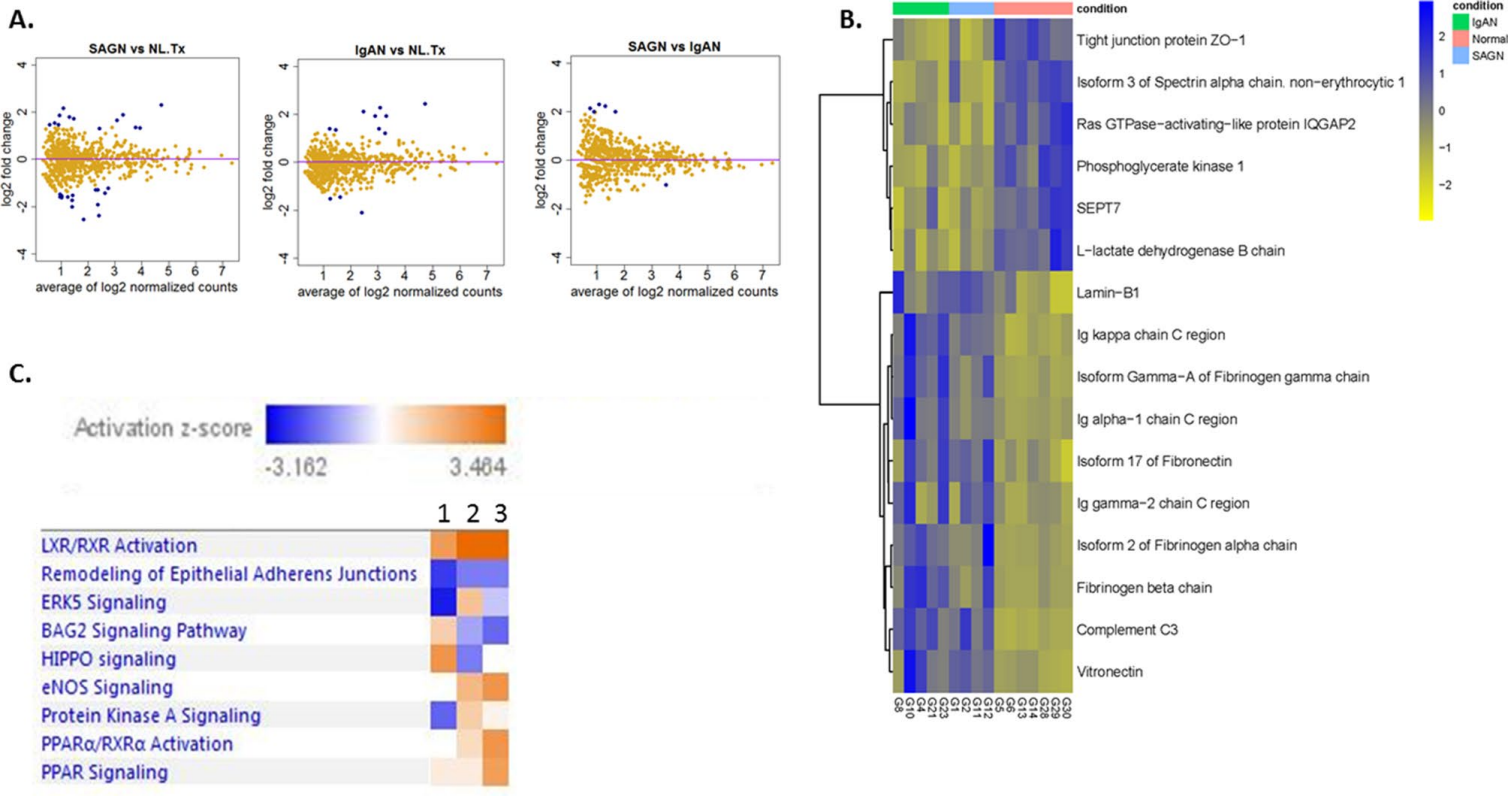
The glomerular proteome of SAGN and IgAN. (**A**) Mean protein expression using dispersion plots—proteins that are differentially expressed are in blue while proteins that are not are in yellow. Fold-change (in log2 scale) is plotted against the mean of normalized protein expression. Each dot represents an individual protein. A fold-change cut-off of ≥ 2 or ≤ -2 in combination with a p value cutoff of ≤ 0.01 was used for detecting differential expression. Only a few proteins are differentially expressed, indicating large overlap in the glomerular proteome of SAGN and primary IgAN. (**B**) Heat map with unsupervised hierarchical cluster analysis of the top similarly expressed proteins in diseased kidneys compared to normal control kidneys with at least 1.5 fold difference with p ≤ 0.05. Each column represents an individual biopsy sample, and each row represents an individual protein. Blue represents higher expression and yellow lower expression. The degrees of similarity in expression levels are presented in the dendrogram. R version 3.6.0. (**C**) Differentially-expressed glomerular signaling pathways between SAGN (heat map column 1), primary IgAN-Oxford E1 (heat map column 2), and primary IgAN-Oxford Class E0 (heat map column 3) using Ingenuity pathway analysis (IPA) and z scores (Qiagen Bioinformatics). Orange indicates pathways predicted to be activated, while blue indicates pathways predicted to be inhibited.

A total of 1063 proteins were identified across all samples in the glomerular compartment based on a 2-peptide hit criterion filtering the peptide identifications for 2 pmm mass accuracy and a peptide FDR less than 1%. Protein lists were filtered using a spectral counting rule to remove infrequently observed and low abundant proteins with four or less (≤ 4) spectral counts thus curating the list down to 320 proteins. Several proteins were similarly expressed in SAGN and IgAN glomeruli compared to normal kidney controls (Fig. 1A,B). Fibrinogen (alpha, beta and gamma chains), complement C3, IgA heavy chain, kappa and lambda light chains, and chymotrypsin (SERPINA3) showed high expression in both SAGN and IgAN. LC MS/MS is a semiquantitative method, looking for relative differences in protein expression based on spectral counts. It does not perform absolute protein quantification (Supplemental file 2). Therefore mild quantitative differences in protein molecules (such as IgA heavy chains, complement C3) between SAGN and IgAN may not be detectable by this method, even though C3 tends to show stronger staining in SAGN by immunofluorescence microscopy. Under-expressed proteins in diseased compared to normal glomeruli were mainly hydrolytic enzymes, including aminoacylase, fructose-1,6-biphosphatase 1, cytoplasmic aconitate hydratase, arginosuccinate synthase, carbonic anhydrase, and creatine kinase B. Many of these play important roles in energy pathways such as tricarboxylic acid cycle (Kreb’s cycle) in carbohydrate metabolism, acid base balance, and intracellular synthetic functions.

Only a few glomerular proteins showed differential expression between SAGN and primary IgAN (Table 3). Four were monocyte/macrophage-related proteins and were significantly higher in SAGN glomeruli compared to normal and IgAN glomeruli. Conversely, several cytoskeletal and adhesion proteins were down-regulated in SAGN compared to IgAN including alpha-actinin-4, laminin subunit alpha-5, isoform 6 of agrin, and basal cell adhesion molecule. Using in silico pathway analysis, the Hippo pathway was predicted to be activated in SAGN relative to IgAN, and conversely, peroxisome proliferator-activated receptor (PPAR), liver-X receptor/retinoid-X receptor (LXR/RXR) and eNOS signaling were increased in IgAN compared to SAGN (Fig. 1C).

**Table 3:** Proteins with statistically significant differential expression between SAGN and IgAN in the glomerular compartment.

Protein identification	avg.IgAN	avg.NL.Tx	avg.SAGN	SAGN vs IgAN	p value
				Fold change	
**Myeloid cell nuclear differentiation antigen**	0.20	0.35	0.81	3.70	0.0120
Thymidine phosphorylase	0.39	0.56	1.69	3.97	0.0104
**Protein S100-A9**	0.79	1.98	3.43	4.02	0.0055
Isoform 3 of Myoferlin	0.33	0.70	1.42	4.03	0.0095
Coronin-1A	0.26	0.51	1.22	4.40	0.0056
**Lysozyme C**	0.53	0.94	2.71	4.74	0.0027
Alpha-1-antichymotrypsin	0.40	0.59	2.18	5.00	0.0024
Basal cell adhesion molecule	12.17	12.90	6.47	− 2.03	0.0080
Alpha-actinin-4	89.96	93.46	66.15	− 1.34	0.0098
Laminin subunit alpha-5	55.83	56.31	39.14	− 1.42	0.008

Mean spectral counts, fold change and p values are shown.

Also, remodeling of epithelial adherens junctions was predicted to be inhibited in both SAGN and IgAN, but more prominently in SAGN (z-score was − 2.449 for SAGN and − 1.633 for IgAN E0 and E1).

#### Tubulointerstitial compartment

**Figure 2:**
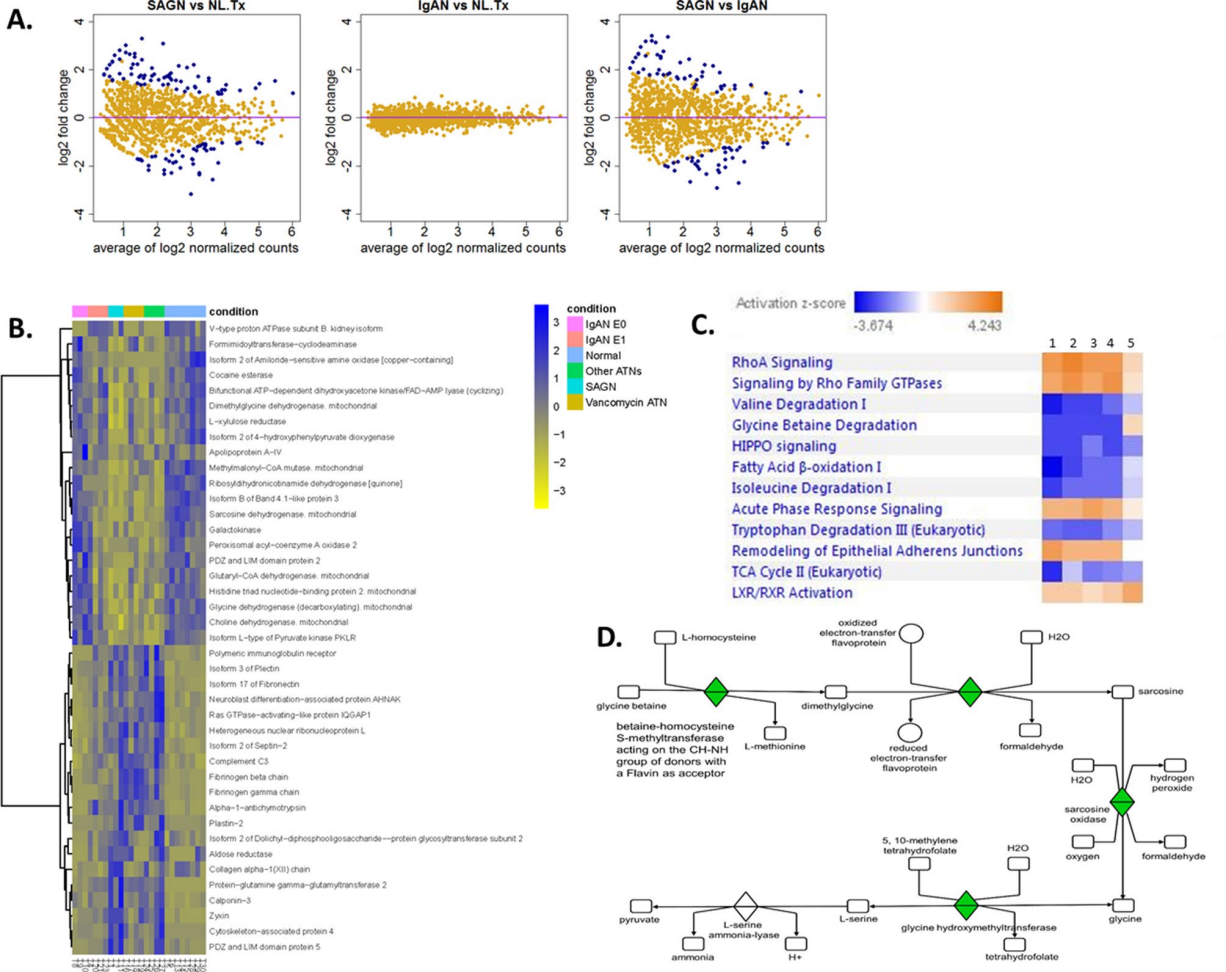
The tubulointerstitial proteome of SAGN and IgAN. (**A**) Mean protein expression using dispersion plots—proteins that are differentially expressed are in blue while proteins that are not are in yellow. Fold-change (in log2 scale) is plotted against the mean of normalized protein expression. Each dot represents an individual protein. A fold-change cut-off of ≥ 2 or ≤ -2 in combination with a p value cutoff of ≤ 0.01 was used for detecting differential expression. Many proteins were differentially expressed. (**B**) Heat map with unsupervised hierarchical clustering analysis of the top differentially expressed tubulointerstitial proteins between SAGN and IgAN (3 or greater fold difference, p < / = 0.01). Each column represents an individual biopsy sample, and each row represents an individual protein. Blue represents higher expression and yellow represents lower expression. The degrees of similarity in expression levels are presented in the dendrogram. R version 3.6.0. (**C**) Differentially-expressed tubulointerstitial signaling pathways between SAGN (heat map column 1), vancomycin-induced ATN (heat map column 2), ATN of other causes (heat map column 3), primary IgAN-Oxford Class E1 (heat map column 4), and primary IgAN-Oxford Class E0 (heat map column 5). Orange indicates pathways predicted to be activated while blue indicates pathways predicted to be inhibited. (**D**) Glycine betaine degradation pathway in SAGN as seen by IPA (Qiagen Bioinformatics). Sarcosine dehydrogenase is downregulated (shown in green and labeled as sarcosine oxidase). This correlates with the significantly low spectral counts for sarcosine dehydrogenase in SAGN biopsies by mass spectrometry.

A total of 1317 proteins were identified across all samples in the tubulointerstitial compartment and after filtering using the same spectral counting rule as for the glomerular compartment, we curated the list down to 622 proteins. In contrast to the glomerular compartment, the tubulointerstitial proteome of SAGN and primary IgAN showed many differentially-expressed proteins (Fig. 2A,B, Table 4). The top upregulated proteins in SAGN compared to IgAN included mainly extracellular matrix and epithelial cell junction proteins. Glutamine gamma-glutamyltransferase (TGM2), a tubular epithelial stress protein was one of the most highly expressed protein in SAGN. Compared to IgAN, SAGN showed marked downregulation of mitochondrial enzymes, enzymes involved in fatty acid oxidation and carbohydrate metabolism, and proteins involved in amino acid degradation. The differences in tubulointerstitial protein expression were generally more pronounced between SAGN and IgAN (E0, C0) than between SAGN and IgAN (E1, C1). Importantly, changes in the SAGN tubulointerstitium were similar to those in biopsies with ATN of other causes (Fig. 2B).

**Table 4:** Tubulointerstitial compartment proteins in SAGN, IgAN, Vancomycin-induced ATN and ATN of other causes, Mean peptide counts, fold changes and p values for selected proteins with highest fold changes between SAGN and IgAN are shown.

Identified proteins	Spectral counts (group means)						SAGN vs IgAN E0		SAGN vs IgAN E1		SAGN vs ATN		SAGN vs Vanc ATN	
	Normal Kidney	IgAN E0	IgAN E1	SAGN	ATN	Vanc ATN	Fold change	p value	Fold change	p value	Fold change	p value	Fold change	p value
**Higher expression in SAGN compared to IgAN**														
Alpha-1-antichymotrypsin	0.37	0	5.21	6.43	5.22	6.44	6.64	1.00E−04	1.21	0.6274	1.17	0.6855	1.04	0.9225
Isoform 17 of Fibronectin	1.84	1.72	7.06	21.42	25.09	17.75	6.09	1.00E−04	2.54	0.0219	− 1.2	0.6503	− 1.14	0.7487
PDZ and LIM domain protein 5	0.48	0	1.54	6.27	3.19	1.38	6	4.00E−04	2.63	0.0523	1.56	0.3659	− 2.95	0.0306
Ras GTPase-activating-like protein IQGAP1	2.08	0.37	3.54	7.56	14.34	9.23	5.71	1.00E−04	1.95	0.0657	− 1.89	0.0497	1.21	0.5642
Protein-glutamine gamma-glutamyltransferase 2	0	1.64	5.28	12.95	7.21	7.29	5.29	1.00E−04	2.19	0.0238	1.65	0.1425	− 1.64	0.1442
Fibrinogen gamma chain	0.36	0	1.39	5.23	7.14	10.75	5.13	9.00E−04	2.44	0.0544	− 1.37	0.4658	2	0.1063
Plastin-2	0.22	0	0.91	4.56	3.74	5.06	4.52	0.0033	2.78	0.0436	1.09	0.8611	1.14	0.7942
Cytoskeleton-associated protein 4	0	0.7	0.44	7.65	4.18	2.25	4.4	0.0039	5.42	0.001	1.46	0.4435	− 2.35	0.089
Isoform 2 of Septin-2	1.46	0.95	3.54	6.51	6.98	5.13	3.68	0.0038	1.68	0.1904	− 1.1	0.8045	− 1.22	0.6131
Zyxin	0.12	0.7	1.85	5.01	3.58	1.99	3.55	0.0114	2.09	0.1225	1.24	0.6403	− 2.09	0.1238
Fibrinogen beta chain	0.36	0.75	1.91	5.52	6.36	13.01	3.46	0.0106	2.35	0.0633	− 1.17	0.722	2.22	0.0613
Isoform 3 of Plectin	2.74	5.48	9.15	23.52	23.22	13.98	3.32	0.0029	2.23	0.0325	− 1.01	0.9682	− 1.54	0.2402
**Lower expression in SAGN compared to IgAN**														
Sarcosine dehydrogenase. mitochondrial	9.27	8.79	3.08	0	1.85	1.91	− 8.53	0	− 3.49	0.0072	− 2.28	0.0884	2.58	0.0473
Bifunctional ATP-dependent dihydroxyacetone kinase/FAD-AMP lyase (cyclizing)	18.28	16.78	18.89	1.65	9.41	9.6	− 5.66	0	− 6.47	0	− 3.43	0.001	3.45	0.001
Formimidoyltransferase-cyclodeaminase	9.56	13.76	7.99	1.34	5.41	8.24	− 5.36	1.00E−04	− 3.38	0.0029	− 2.36	0.0409	3.48	0.0024
L-xylulose reductase	9.94	11.07	9.6	1.27	6	7.63	− 4.7	1.00E−04	− 4.24	1.00E− 04	− 2.74	0.0105	3.42	0.0015
Isoform B of Band 4.1-like protein 3	11.27	9.33	5.62	1.27	2.76	2.97	− 4.31	3.00E−04	− 2.66	0.0146	− 1.5	0.3378	1.65	0.2337
Isoform 2 of 4-hydroxyphenylpyruvate dioxygenase	6.12	5.96	4	0	1.7	2.18	− 5.33	6.00E−04	− 4.08	0.0035	− 2.15	0.1232	2.62	0.0503
Dimethylglycine dehydrogenase. mitochondrial	17.8	15.06	12.64	3.17	6.84	9.38	− 3.42	6.00E−04	− 2.99	0.0015	− 1.69	0.1444	2.26	0.0207
Choline dehydrogenase. mitochondrial	12.25	13.67	10.42	3.65	5.27	6.8	− 3.16	7.00E−04	− 2.5	0.0056	− 1.36	0.3832	1.66	0.1423
Glutaryl-CoA dehydrogenase. mitochondrial	5.01	6.92	2.96	0.66	3.39	2.78	− 4.49	8.00E−04	− 2.29	0.0658	− 2.43	0.0501	2.16	0.0899
Ribosyldihydronicotinamide dehydrogenase [quinone]	6.33	5.38	2.28	0.31	1.01	2.17	− 4.75	9.00E−04	− 2.29	0.0803	− 1.34	0.5507	2.24	0.0906
Isoform L-type of Pyruvate kinase PKLR	7.58	9.48	6.73	1.69	1.54	3.42	− 3.81	9.00E−04	− 2.86	0.008	1.15	0.7569	1.57	0.2821
Glycine dehydrogenase (decarboxylating). mitochondrial	6.97	7.12	5.48	0.72	3.12	2	− 4.35	0.0012	− 3.59	0.004	− 2.18	0.0911	1.55	0.3473
Isoform 2 of Amiloride-sensitive amine oxidase [copper-containing]	7.6	5.49	2.21	0.66	0	0	− 4.07	0.0036	− 1.97	0.1617	1.86	0.2316	− 1.89	0.2185

Vanc *ATN* Vancomycin-induced acute tubular necrosis, *ATN* acute tubular necrosis of other causes.

Pathway analysis also predicted inhibition of metabolic and amino acid degradation pathways in SAGN compared to primary IgAN subgroups, primarily the Kreb’s cycle, fatty acid β-oxidation, glycine betaine degradation, isoleucine degradation, and valine degradation (Fig. 2C,D). In terms of activated and inhibited pathways, the IgAN E1 C1 subgroup appeared intermediate between SAGN and the IgAN E0 C0 subgroup. Vancomycin-induced ATN and ATN of other causes showed changes similar to those in SAGN. As in the glomerular compartment, LXR/RXR pathways were activated in primary IgAN, (especially the E0 C0 subgroup of IgAN) but not in SAGN. RhoA signaling, acute phase response signaling, remodeling of epithelial adherens junctions, BAG2 (Bcl2 associated athanogene 2) signaling were upregulated in SAGN and ATN subgroups as compared to the IgAN E0 C0 group (Fig. 2C). In contrast to the glomerular proteome, the Hippo pathway was inhibited in the tubulointerstitial compartment of all the diseased kidneys compared to normal kidney.

### Immunohistochemical Staining (IHC)

#### Glomerular compartment

**Figure 3:**
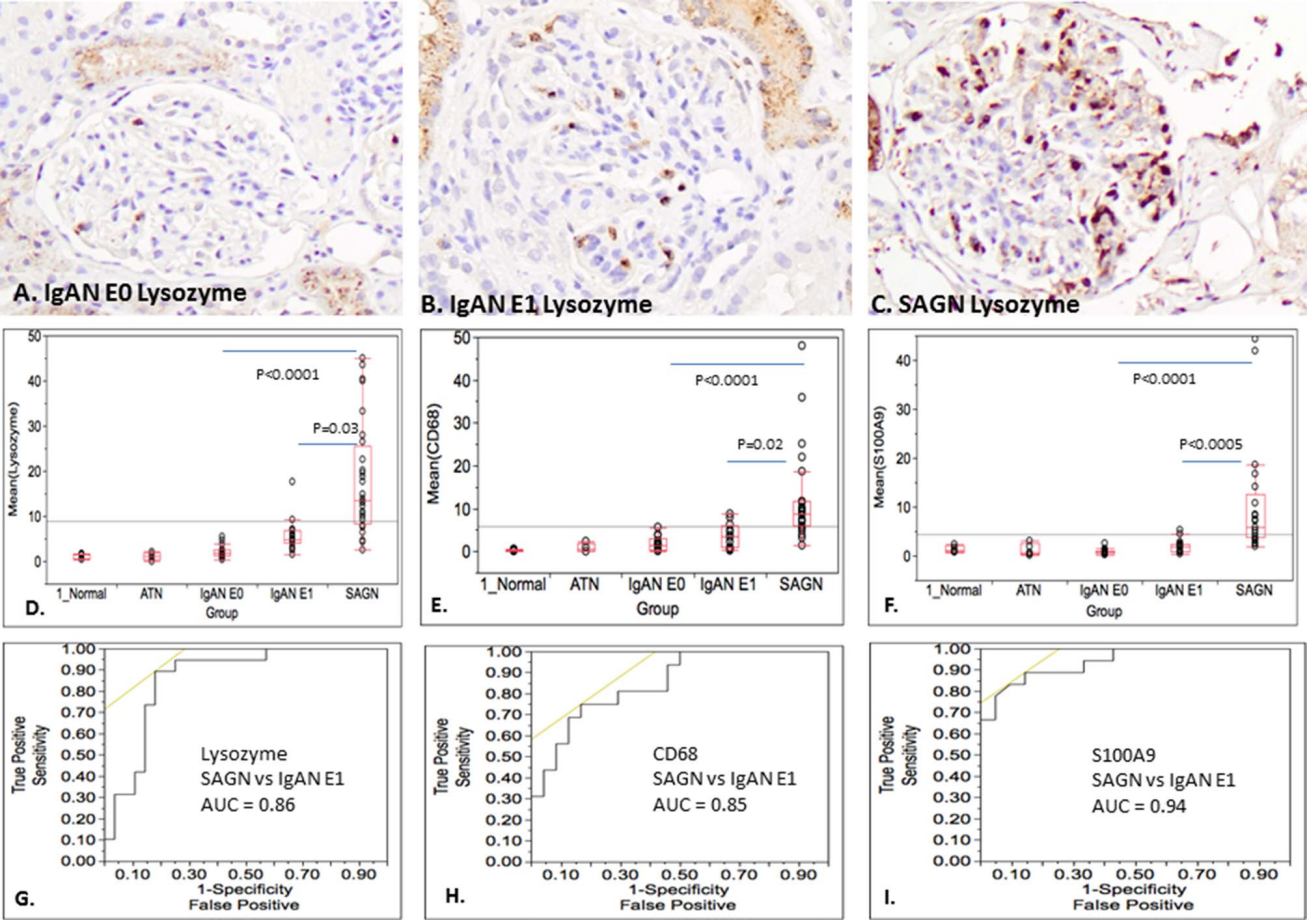
(**A**–**C**) Glomerular IHC staining for lysozyme in biopsy of IgAN E0; IgAN E1; and SAGN respectively. (**D**–**F**) Box and whisker plots showing average number of positively stained cells for lysozyme; CD68; and S100A9 respectively for each group—*baseline* *transplant* *biopsies,* *ATN,* IgAN E0, IgAN E1, and SAGN. Each dot indicates mean count for each biopsy. SAGN showed significantly higher counts compared to both subgroups of IgAN (p values shown) and also compared to normal kidney and ATN with all three antibodies (details not shown). (**G**–**I**) Receiver–operator characteristic curves for SAGN versus IgAN E1 for lysozyme; CD68; and S100A9, respectively.

**Figure 4:**
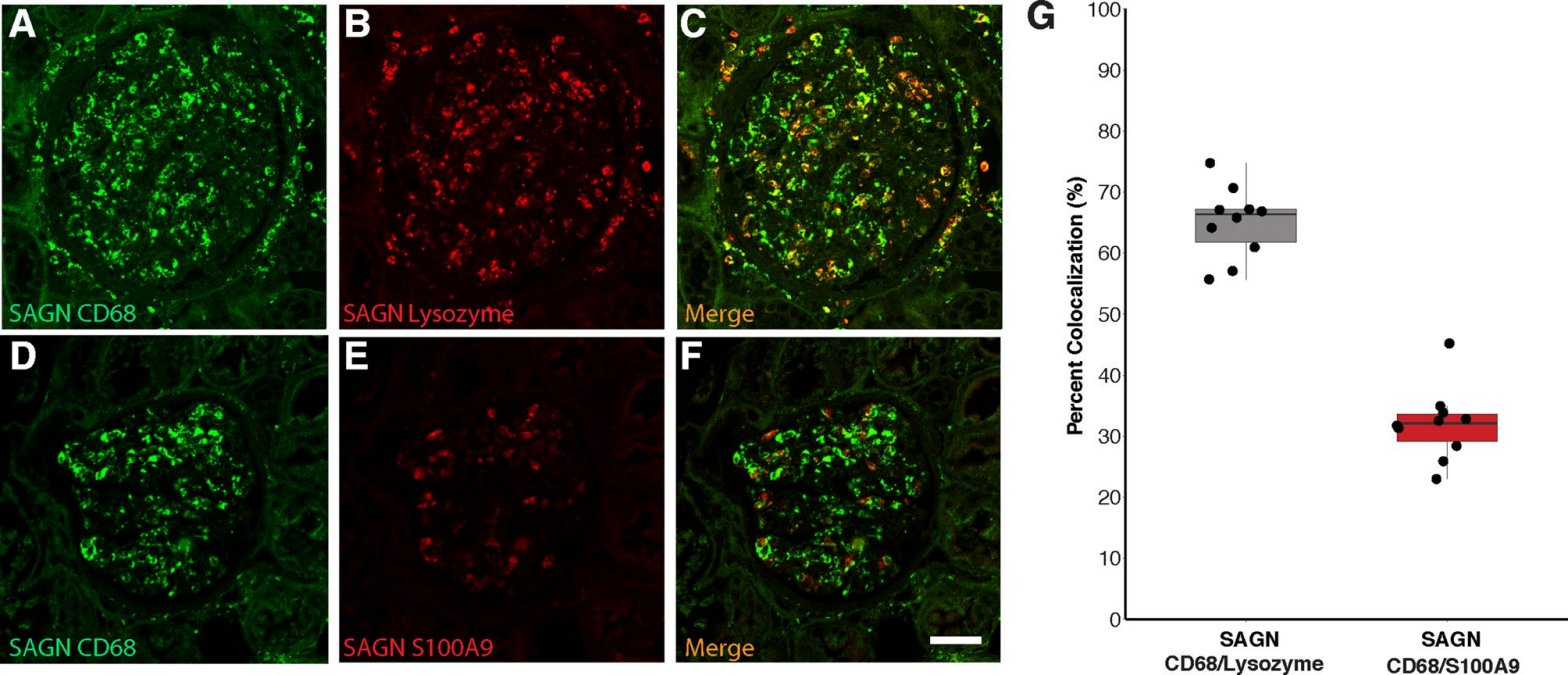
Glomerular macrophage expression of lysozyme and S100A9. Co-localization with CD68. Using kidney biopsy tissue from a case of SAGN, staining for macrophages was done using a FITC-conjugated antibody to CD68. Consecutive tissues sections were stained with rhodamine-conjugated antibodies to lysozyme and S100A9. The images were merged to determine the proportion of co-localization in macrophages. (**A**–**C**) CD68 and lysozyme co-localization. (**D**–**F**) CD68 and S100A9 co-localization (**G**). Graphical representation shows showed 65% co-localization of CD68 and lysozyme; and 31% co-localization of CD68 and S100A9. R Core Team. 2018. "A Language and Environment for Statistical Computing", R Foundation for Statistical Computing, Vienna Austria, https://www.R-project.org*.*

IHC staining for lysozyme (Fig. 3A–C), CD68 and S100A9 was performed on a total of 67 biopsies including subgroups SAGN, IgAN MEST E0 and IgAN MEST E1. Additionally 5 biopsies each of ATN and normal kidney were also stained. The number of cells that were positively stained for each marker was significantly higher in SAGN than IgAN E0 and E1, ATN and normal kidney (Fig. 3D–F, Table 5). The differences between IgAN E1 and IgAN E0 were not found to be statistically significant, except for lysozyme (p = 0.06 by non-parametric Dunn test for all pairwise comparisons; and p = 0.0015 using Tukey’s HSD parametric test applied to log-scale data, details not shown). Double immunofluorescence staining with antibody pairs performed on SAGN biopsies showed a 65% co-localization between lysozyme and CD68, but only a 31% co-localization between S100A9 and CD68 (Fig. 4A–G).

**Table 5:** Statistical analysis of CD68, lysozyme and S100A9 IHC staining results on training set and validation set. Non-parametric analysis (Dunn test).

Antibody for IHC	Differences in mean cell counts on IHC (p values)			Area under the curve (AUC)		Training set Maximum sensitivity; specificity (with cut-off values)		Validation set (n = 30) Maximum sensitivity; specificity (using cut-off value from training set)	
	IgAN E0 vs SAGN	IgAN E1 vs SAGN	IgAN E0 vs IgAN E1	IgAN E0 vs SAGN	IgAN E1 vs SAGN	IgAN E0 vs SAGN	IgAN E1 vs SAGN	IgAN E0 vs SAGN	IgAN E1 vs SAGN
Lysozyme (n = 64)	< 0.0001	0.03	0.06	0.98	0.86	100%; 85.7% (< / = 5.98)	89.5%; 82.14% (< / = 7.38)	100%; 60%	100%; 50%
CD68 (n = 57)	< 0.0001	0.02	0.8	0.96	0.85	94.1%; 87.5% (< / = 4.42)	75%; 83.3% (< / = 5.25)	100%; 80%	100%; 50%
S100A9 (n = 51)	< 0.0001	0.0005	0.7	0.99	0.94	100%; 91.48% (< / = 2.25)	83.33%; 88.89% (< / = 2.50)	100%; 80%	90%; 80%

*AUC* area under the curve.

To determine whether glomerular staining for lysozyme, CD68 or S100A9 could be used to distinguish between SAGN, IgAN E0 and IgAN E1, ROC analysis was done (Fig. 3G–I). S100A9 was the most robust marker with an area under the ROC curve (AUC) of 0.98 for IgAN E0 versus SAGN and 0.94 for IgAN E1 versus SAGN (Table 5). Using logarithms of lysozyme, S100A9 and CD68-positive cells, two variable-predictors in the logistic regression model were also evaluated, but the single predictor based on S100A9 gave the best results.

These histologic studies were validated in an independent set of 30 kidney biopsies. The achieved sum of sensitivity and specificity for the validation set was comparable to what was achieved for the training set (Table 5).

#### Tubulointerstitial compartment IHC

**Figure 5:**
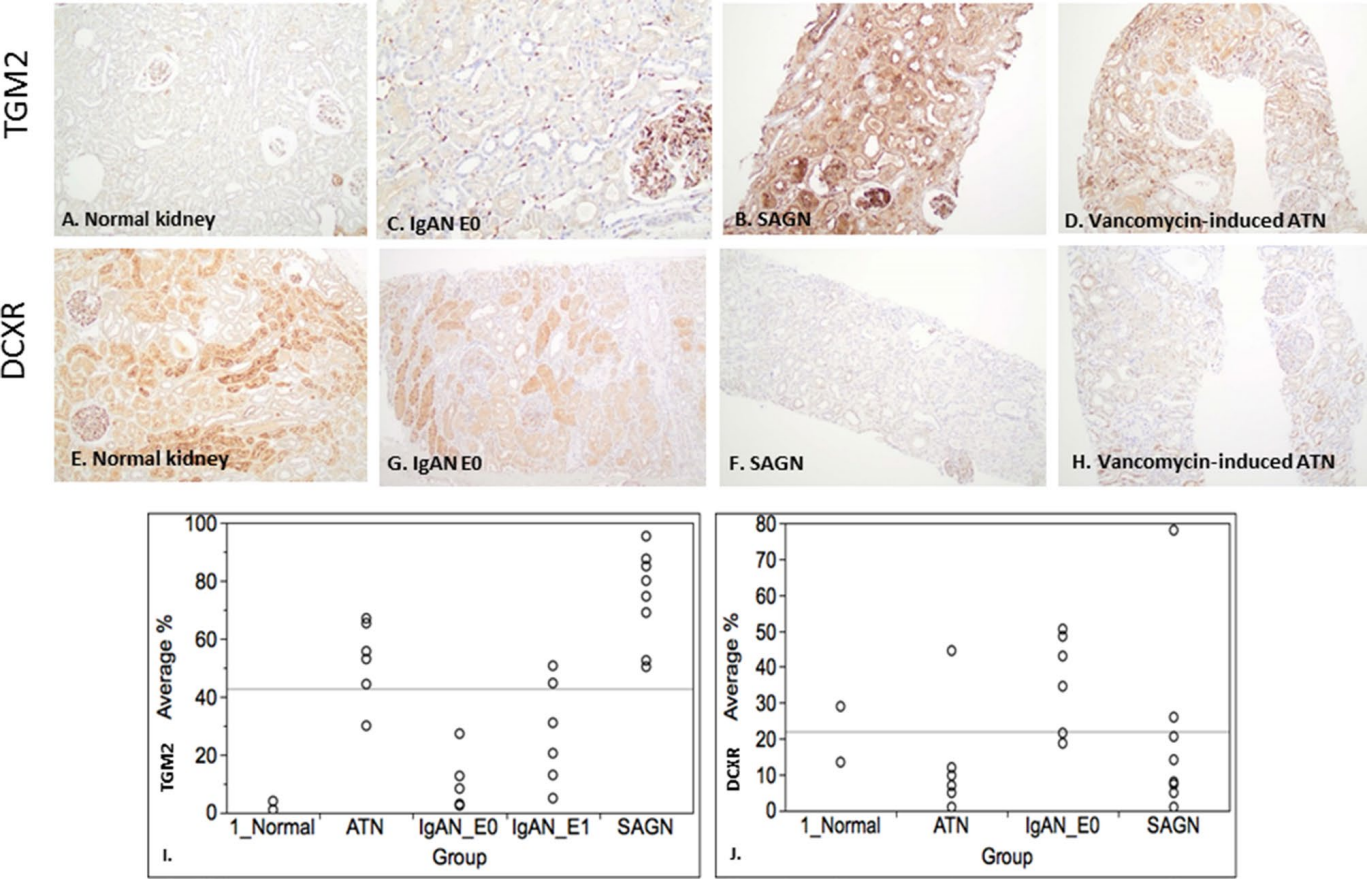
(**A**–**D**) Immunohistochemical staining for glutamine gamma-glutamyltransferase (TGM2), and (**E**–**H**). Xylulose reductase (DCXR) staining. Tissue from normal kidney (**A**,**E**), IgAN-E0 (**B**,**F**), SAGN (**C**,**G**), and vancomycin-induced ATN (**D**,**H**) was stained. Strong tubulointerstitial expression of TGM2 is seen in SAGN and ATN compared to normal and IgAN biopsies. Conversely, there is decreased expression of DCXR in SAGN and ATN compared to normal and IgAN biopsies. (**I**,**J**) Quantification of IHC staining for TGM2 and DCXR in each biopsy was performed using image analysis software, by calculating ratio of brown pixel area to total pixel area within the biopsy sample (Aperio ImageScope ver. 12.3.0.5056 (Leica Biosystems Inc., Buffalo Grove, IL, USA). These ratios were expressed as percentage. Distribution of the average percentages are shown by group for TGM2 (**I**) and DCXR (**J**).

IHC staining for two of the differentially expressed proteins—transglutaminase 2 (TGM2) and l-xylulose reductase (DCXR) was performed on 9 SAGN biopsies, 6 IgAN E0 biopsies, 6 IgAN E1 biopsies, and 6 ATN biopsies (etiologies being vancomycin toxicity, thrombotic microangiopathy, rhabdomyolysis, oxalate nephropathy, and minimal change disease), Fig. 5A–H. Both TGM2 and DCXR were found to be constitutively expressed in endothelial cells of the glomerular and peritubular capillaries, but not in the tubules or interstitium of normal kidney (Fig. 5A,E). Quantification was performed using image analysis software, calculating the ratio of brown pixel area to total pixel area within the biopsy sample (Aperio ImageScope ver. 12.3.0.5056 (Leica Biosystems Inc., Buffalo Grove, IL). These ratios were expressed as percentage. Distribution of the average percentage by group are shown in Fig. 5I,J for TGM2 and DCXR respectively. For TGM2 the highest mean ratio was seen for SAGN (74 ± 15.2), followed by ATN (52.5 ± 13), IgAN E1 (27.4 ± 17), IgA E0 (13.4 ± 11) and lowest was in the normal kidney samples (2.5 ± 2.1). Conversely, DCXR showed the highest ratios in normal kidney, followed by IgAN, as compared to the SAGN and ATN groups.

## Discussion

A comparison of the glomerular and tubulointerstitial proteomes from SAGN and primary IgAN kidney biopsies was carried out to identify differences and potential biopsy-based biomarkers that could help discriminate between these conditions. Our study found that the glomerular proteomes of IgAN and SAGN were very similar, except for a significant upregulation of inflammatory protein expression in SAGN glomeruli. The tubulointerstitial proteome of SAGN however showed marked differences from IgAN, and in fact resembled that of biopsies from patients with ATN of various causes. The tubulointerstitial proteome of IgAN was very similar to that of normal kidney.

The glomeruli from SAGN patients contained high levels of leukocyte-derived proteins known to be involved in the response to infection. Lysozyme in serum and other body fluids is antimicrobial and originates mostly from monocytes/macrophages and to a lesser extent from neutrophils^20^. S100A9 is a calcium-binding protein highly expressed in the cytosol of neutrophils and monocytes^21,22^, and participates in multiple inflammatory processes including bacterial infections, autoimmune diseases, complement activation and even aging processes^23–25^. The average age of SAGN patients in our cohort was higher than the IgAN patients (individual patient ages shown in Table 1), raising the possibility that aging contributed to inter-group differential expression of S100A9. However, even within the SAGN group, differential staining was observed (Fig. 3F), suggesting that the degree of glomerular inflammation was the main contributing factor. Myeloid nuclear differentiation antigen (higher in SAGN, shown in Table 3) is constitutively expressed by monocytes and neutrophils and can be upregulated by interferon-alpha, a cytokine increased during infections^26^.

IHC staining confirmed that lysozyme, CD68, and S100A9-positive cells were significantly more numerous in SAGN glomeruli compared to IgAN, and not only the E0, but also the E1 subgroup of IgAN. This is important because IgAN E1 is more inflammatory than E0 and therefore more likely to be confused with SAGN histologically.

This finding is also consistent with the higher frequency and severity of glomerular endocapillary hypercellularity seen in SAGN biopsies as compared to IgAN biopsies as we have shown previously (endocapillary hypercellularity in 60% of SAGN biopsies as compared to only 10% of IgAN)^6^. Endocapillary hypercellularity is thought to be due to infiltrating macrophages and admixed neutrophils in the glomerular capillaries. Counting endocapillary neutrophils and macrophages on routine hematoxylin and eosin stained slides is practically difficult. The immunostains help to better delineate the glomerular inflammatory cells. We further showed that lysozyme and S100A9 do co-localize with the macrophage marker CD68, suggesting that infiltrating macrophages were at least partially responsible for glomerular lysozyme and S100A9 expression. Co-localization was 65% for lysozyme but only 31% for S100A9, suggesting that lysozyme and S100A9 may be staining different macrophage populations or that S100A9 may also stain neutrophils.

It is conceivable that S100A9 staining could be used to differentiate SAGN from IgAN in ambiguous clinical situations. A single predictor model based on S100A9 showed the best ability to discriminate between SAGN and IgAN. However, it should be emphasized that occasional cases of active proliferative IgAN may show high numbers of S100A9 (and lysozyme, CD68) positive cells, but such cases are rare particularly in the Western population. Interpretation of staining results however, must be done within clinical context.

Actin-binding cytoskeletal proteins and cell adhesion proteins were downregulated in SAGN glomeruli compared to IgAN and normal glomeruli, correlating well with the predicted inhibition of epithelial adherens junction remodeling pathway on IPA. These changes may be the cause or effect of increased podocyte injury in SAGN glomeruli, and may facilitate the often encountered nephrotic-range proteinuria in SAGN. The Hippo pathway, also predicted to be activated in SAGN glomeruli may result in podocyte damage^27–29^, and contribute to the nephrotic range proteinuria.

The tubulointerstitial proteome of SAGN showed significantly higher expression of TGM2, compared to IgAN, also shown by IHC staining. TGM2, a marker of tubular injury^30–32^, is a member of the protein glutamine γ-glutamyltransferase enzyme family that catalyzes the calcium-dependent crosslinking of protein targets in the extracellular matrix (ECM)^33–35^. Secretion of TGM2 from cortical tubular epithelial cells into the surrounding interstitium has been shown to contribute to fibrotic remodeling of the kidney and the development of chronic kidney disease^31,32^. Hippo signaling was predicted to be decreased and Rho signaling was predicted to be increased in the SAGN tubulointerstitium, possibly predisposing to interstitial fibrosis^36–38^. In addition, the SAGN interstitium also showed lack of activation of pathways that could potentially protect against the development of chronic kidney damage, such as PPARα, LXR-RXR and eNOS signaling^39,40^. The latter were more prominently expressed in IgAN in our study as well in a study by Liu et al.^41^.

This is not to say that SAGN (and ATN) does not activate any protective mechanisms. For example, the predicted activation of BAG2 signaling and inhibition of ERK5 in SAGN glomeruli could possibly exert a protective effect through inhibition of apoptosis and glomerular fibrosis^42,43^. The interactions of these complex cellular pathways and their net effect likely depends on the surrounding milieu which may be different at different stages of the disease^44^.

The SAGN tubulointerstitial proteome also demonstrated a marked down-regulation of enzymes involved in energy generation and metabolism, likely contributing to tubular cell dysfunction and ATN. An interesting example is the marked decrease in DCXR expression in SAGN. DCXR is a multi-functional enzymatic protein playing a role in the uronate cycle of glucose metabolism and production of organic intracellular osmolytes needed in tissue osmoregulation and water absorption in the proximal tubule^45^. Loss of this enzyme in SAGN may predispose to tubular osmotic stress. However, this may be mitigated to some extent by the concomitant depression of specific amino acid degradation pathways like the glycine betaine degradation pathway. Glycine betaine is an osmoprotectant which accumulates in cells in response to osmotic stress^46^. The degradation of glycine betaine proceeds by sequential enzymatic demethylation (Fig. 2D) and the last step is catalyzed by enzyme sarcosine dehydrogenase. Sarcosine dehydrogenase was found to be markedly under-expressed in SAGN tubulointerstitial proteome.

Together these data suggest that SAGN is an aggressive form of glomerulonephritis accompanied by marked acute tubular injury as compared to primary IgAN. Interestingly, IgAN with endocapillary hypercellularity (Oxford MEST Score E1) had proteomic signatures in between SAGN and IgAN without endocapillary hypercellularity (Oxford MEST Score E0).

In summary, our study sheds light on the more inflammatory profile of SAGN compared to primary IgAN. These appear to be separate disease entities, but with plausible overlaps at some points in their down-stream pathogenetic pathways such as glomerular IgA deposition. The mean spectral counts for IgA heavy chain in SAGN biopsies however were lower than in IgAN (4.22 versus 13.9) comparing well with the consistently lower intensity of IgA staining in SAGN biopsies compared to primary IgAN^6,47^, and also the absence of IgA in up to 25% of SAGN biopsies^6,48^. It must be acknowledged that the analytic approach used here examined proteins observed in most tissue samples and compared only proteins with high spectral counts. While this approach improves confidence in the observed results, alternative approaches, for example assessment of mass intensities, would potentially increase proteomic depth and possibly identify disease-specific biomarkers. Also it is should be noted that glomerular lesions (especially crescents) can be focal in distribution. This can result in inter-glomerular proteome heterogeneity and subsequent analytical differences. We have combined the glomeruli present in each biopsy, therefore inter-glomerular heterogeneity was minimized. The proteomic differences between SAGN and IgAN emerging from this study appear more quantitative rather than qualitative with no disease-specific biomarkers. Nonetheless, our immunostaining results with the combination of inflammatory macrophage markers (S100A9 and lysozyme) and tubular stress marker TGM2 may aid diagnosis in ambiguous cases of SAGN.

## Methods

All methods were carried out in accordance with relevant guidelines and regulations. Archived tissue specimens remaining in paraffin embedded blocks after routine diagnostic work was already completed were used for this study. There was no prospective sample collection from human subjects and no direct patient interaction (approved waiver for informed consent). The study was approved by the Ohio State University Institutional Review Board (IRB Protocol number 2011H0364).

### Tissue collection for LC–MS/MS

Four 10 μm sections were cut from the paraffin-embedded tissue blocks and mounted on thermoplastic (polyethylenenapthalate covered) glass slides (Carl Zeiss MicroImaging). Blades and water bath were changed before each case. Slide processing, laser capture, tissue collection by catapulting, protein retrieval and preparation for LC MS are described in Supplemental File 1. Laser capture was used to separately collect the glomeruli and tubules.

Samples (1.5 μg) were analyzed with nanoLCMS using a 3 h 1D (C18) RP gradient and a LTQ-Orbitrap ELITE mass spectrometer collecting MS2 data by ETD/CID decision tree analysis. Details provided in Supplemental File 2. Data were annotated through ProteomeDiscoverer 1.4 using Mascot (v2.4) and Sequest HT algorithms with a merged human and Staphylococcus aureus protein databases. Results were loaded into Scaffold 4 for comparative proteomics. Protein and peptide FDR were minimized using Peptide/Protein Prophet, elimination of all decoy hits, and the 2-peptide hit rule^49^.

### Immunohistochemistry (IHC)

IHC was performed for representative proteins selected on the basis of their differential expression pattern between SAGN and primary IgAN and their potential applicability to diagnostic pathology. These included lysozyme, S100A9, protein-glutamine gamma-glutamyl transferase 2 (TGM2) and L-xylulose reductase (DCXR). CD68 staining was also performed, since it is a routinely used IHC macrophage maker. Appropriate secondary antibodies were used (Supplementary data file 3). Lysozyme and S100A9 are leukocyte, mainly macrophage-derived proteins and are thought to reflect inflammationTGM2 and DCXR are expressed by renal tubular epithelial cells. All IHC staining was performed using an indirect immunoperoxidase method on FFPE tissue sections cut at 3 microns after antibody validation (Supplemental data file 3). Appropriate positive (placental tissue for TGM2 and tonsillar lymphoid tissue for the inflammatory markers) and negative controls (both rabbit non-immune serum; and omission of primary antibody) were included.

Double staining and co-localization studies were done for lysozyme, S100A9 and CD68. Sections were cut at 3 microns, deparaffinized, and subjected to HIER using Target Retrieval Solution (Agilent). The slides were then treated with Protein Block, Serum-Free (Agilent) for 10 min prior to the first primary antibody, CD68 at 1:100 for 1 h. This was followed by a 1 h incubation using DyLight 488-conjugated, AffiniPure Donkey Anti Mouse IgG (H+L) (Jackson ImmunoResearch Laboratories, INC) at a dilution of 1:50. This was followed by staining with the second primary antibodies against lysozyme (diluted 1:100) or S100A9 (diluted 1:200) for 1 h, followed by a 30 min incubation with the secondary antibody, DyLight 594-conjugated, AfffiniPure Donkey Anti Rabbit IgG (H+L) at a dilution of 1:50. (Jackson ImmunoResearch Laboratories, INC). For each marker pair, images of ten different glomeruli within the biopsy sample were captured and percent co-localization was calculated using a custom image analysis workflow in R (Supplementary file 4)^50^.

Negative controls with secondary antibody alone were performed and showed no stain. Glomerular images were captured on a Carl Zeiss Axio Imager Z.1 with LSM700 confocal microscope. This microscope used a motorized stage at 20× objective with a pinhole of 0.5 Airy unit, scan speed of 8, and a resolution of 512 × 512 to capture images.

### Statistical analysis

#### LC–MS/MS data

Glomerular and tubulointerstitial proteins were studied separately. To ensure a robust comparison of proteomic data without imputation of missing values for proteins having low abundance (i.e. spectral counts ≤ 4) for at least 90% samples were excluded from further statistical analysis. Prior to statistical comparisons the protein spectral counts were normalized across samples. To compare protein abundance between groups, we applied negative binomial generalized linear models through a R package called *DESeq2* (R version 3.6.0)^51^. DESeq2 method was used for comparing protein mean expression between groups. Dispersion estimation and fold-change estimation were then improved by applying an Empirical Bayes shrinkage method. The significance level was determined by controlling the mean number of false positives at 1 out of every 100 proteins^52^. Top proteins were selected by both fold-change (≥ 2) and the significance level (≤ 0.01). After excluding three outliers (biopsies with overall low glomerular spectral counts), hierarchical clustering on heat maps as well as principal component analysis plots (PCA) were performed.

Data files for acquired LCMS data (.RAW), for peak lists (.mgf), and scaffold search results (.sf3) files along with a sample key and sequence database were deposited in MassIVE (https://massive.ucsd.edu/) data repository (MassIVE ID: MSV000085473) with the Center for Computational Mass Spectrometry at the University of California, San Diego and shared with the ProteomeXchange (www.proteomexchange.org) (Proteome Exchange ID: PXD019422). Data with uniprot numbers is provided in Supplemental files 5 and 6.

#### IHC data

Immunostaining for lysozyme, S100A9 and CD68 was performed on individual tissue sections. Positively stained cells within the glomerular capillary tuft were manually counted using criteria described by Soares et al.^53^. A positive cell was defined by clearly delimited aggregates of brown-stained cytoplasmic granules with or without a juxtaposed nucleus. Counts were performed on every non-globally sclerosed glomerulus in the biopsy sample at 400× magnification. An average count for each biopsy was calculated and used for statistical comparisons. Pairwise comparisons of mean counts for IgAN E0, IgAN E1 and SAGN groups were made using both non-parametric Dunn test and Tukey’s HSD test for the log-transformed counts that were found to be normally distributed. The normal kidney and ATN cases were also stained and counted. Classification rules based on each of the three IHC markers were developed and the associated sensitivity and specificity values were computed for distinguishing IgAN E0 and IgAN E1 from SAGN. Threshold values that yielded the maximum sum of sensitivity and specificity were used to determine optimal classification rules. Receiver operating characteristic (ROC) curves were also generated. Two predictor logistic regression models using combinations of the 3 IHC markers were also developed and ROC curves were generated. These analyses were done on JMP Version 13 (SAS Institute, Cary, NC, USA). Level of significance was set at 0.05 in all analyses.

#### Ingenuity pathway analysis

Qiagen’s Ingenuity Pathway Analysis (IPA, Qiagen Redwood City www.qiagen.com/ingenuity) was used to identify canonical pathways that were enriched in the various groups and separately in the glomerular and tubulointerstitial compartments. All proteins that were used for statistical analysis were used for IPA analysis. Significant pathways were determined by Fisher’s exact test, right tailed and presented as − log (*p* value). The statistically significant functional pathways in each disease state, and the predicted activation (or inhibition) state of these functional pathway end-points in each group was determined by the z score algorithm calculated by IPA software.

## Supplementary information

Supplementary Information

## References

[1] Satoskar AA, Nadasdy T, Silva FG, Jennette JC, Olson JL, D’Agati VD, Silva F (2014). Acute post-infectious glomerulonephritis and Glomerulonephritis caused by persistent bacterial infection. Chapter 10. Heptinstall’s Pathology of the Kidney.

[2] Nasr SH (2003). IgA-dominant acute poststaphylococcal glomerulonephritis complicating diabetic nephropathy. Hum. Pathol..

[3] Satoskar AA (2006). Staphylococcus infection-associated glomerulonephritis mimicking IgA nephropathy. Clin. J. Am. Soc. Nephrol..

[4] Haas M, Racusen LC, Bagnasco SM (2008). IgA-dominant postinfectious glomerulonephritis: A report of 13 cases with common ultrastructural features. Hum. Pathol..

[5] Worawichawong S (2011). Immunoglobulin A-dominant postinfectious glomerulonephritis: Frequent occurrence in nondiabetic patients with Staphylococcus aureus infection. Hum. Pathol..

[6] Satoskar AA (2017). Staphylococcus infection-associated GN—spectrum of IgA staining and prevalence of ANCA in a single-center cohort. Clin. J. Am. Soc. Nephrol..

[7] Satoskar AA, Parikh SV, Nadasdy T (2020). Epidemiology, pathogenesis, treatment and outcomes of infection-associated glomerulonephritis. Nat. Rev. Nephrol..

[8] Haas M, Jennette JC, Olson JL, D’Agati VD, Silva F (2014). IgA nephropathy and IgA vasculitis (Henoch-Schönlein purpura) nephritis. Chapcter 12. Heptinstall’s Pathology of the Kidney.

[9] Satoskar AA (2013). Henoch-Schonlein purpura-like presentation in IgA-dominant Staphylococcus infection-associated glomerulonephritis—a diagnostic pitfall. Clin. Nephrol..

[10] Working Group of the International Ig, A. N. N. (2009). The Oxford classification of IgA nephropathy: Rationale, clinicopathological correlations, and classification. Kidney Int..

[11] Trimarchi H (2017). Oxford Classification of IgA nephropathy 2016: An update from the IgA Nephropathy Classification Working Group. Kidney Int..

[12] Ballardie FW, Roberts IS (2002). Controlled prospective trial of prednisolone and cytotoxics in progressive IgA nephropathy. J. Am. Soc. Nephrol..

[13] Rauen T (2015). Intensive supportive care plus immunosuppression in IgA nephropathy. N. Engl. J. Med..

[14] Satoskar AA (2012). Characterization of glomerular diseases using proteomic analysis of laser capture microdissected glomeruli. Modern Pathol..

[15] Shapiro JP (2012). A quantitative proteomic workflow for characterization of frozen clinical biopsies: Laser capture microdissection coupled with label-free mass spectrometry. J. Proteom..

[16] Vrana JA (2009). Classification of amyloidosis by laser microdissection and mass spectrometry-based proteomic analysis in clinical biopsy specimens. Blood.

[17] Shapiro JP (2017). Laser capture microdissection of pancreatic acinar cells to identify proteomic alterations in a murine model of caerulein-induced pancreatitis. Clin. Transl. Gastroenterol..

[18] Sprung RW (2009). Equivalence of protein inventories obtained from formalin-fixed paraffin-embedded and frozen tissue in multidimensional liquid chromatography-tandem mass spectrometry shotgun proteomic analysis. Mol. Cell. Proteom..

[19] Kawata N (2020). Proteomics of human glomerulonephritis by laser microdissection and liquid chromatography-tandem mass spectrometry. Nephrology.

[20] Resnitzky P, Shaft D, Yaari A, Nir E (1994). Distinct intracellular lysozyme content in normal granulocytes and monocytes: A quantitative immunoperoxidase and ultrastructural immunogold study. J. Histochem. Cytochem..

[21] Zhao F (2012). S100A9 a new marker for monocytic human myeloid-derived suppressor cells. Immunology.

[22] Wang S (2018). S100A8/A9 in inflammation. Front. Immunol..

[23] Chimenti MS (2016). S100A8/A9 in psoriatic plaques from patients with psoriatic arthritis. J. Int. Med. Res..

[24] Tyden H (2017). Pro-inflammatory S100 proteins are associated with glomerulonephritis and anti-dsDNA antibodies in systemic lupus erythematosus. Lupus.

[25] Swindell WR (2013). Robust shifts in S100a9 expression with aging: A novel mechanism for chronic inflammation. Sci. Rep..

[26] Briggs RC (1994). The human myeloid cell nuclear differentiation antigen gene is one of at least two related interferon-inducible genes located on chromosome 1q that are expressed specifically in hematopoietic cells. Blood.

[27] Meliambro K (2017). The Hippo pathway regulator KIBRA promotes podocyte injury by inhibiting YAP signaling and disrupting actin cytoskeletal dynamics. J. Biol. Chem..

[28] Wennmann DO (2014). The Hippo pathway is controlled by Angiotensin II signaling and its reactivation induces apoptosis in podocytes. Cell Death Dis..

[29] Bonse J (2018). Nuclear YAP localization as a key regulator of podocyte function. Cell Death Dis..

[30] Folk JE, Finlayson JS (1977). The epsilon-(gamma-glutamyl) lysine crosslink and the catalytic role of transglutaminases. Adv. Protein Chem..

[31] Johnson TS (2003). Tissue transglutaminase and the progression of human renal scarring. J. Am. Soc. Nephrol..

[32] Johnson TS (2007). Transglutaminase inhibition reduces fibrosis and preserves function in experimental chronic kidney disease. J. Am. Soc. Nephrol..

[33] Stamnaes J, Fleckenstein B, Sollid LM (2008). The propensity for deamidation and transamidation of peptides by transglutaminase 2 is dependent on substrate affinity and reaction conditions. Biochem. Biophys. Acta..

[34] Stamnaes J, Pinkas DM, Fleckenstein B, Khosla C, Sollid LM (2010). Redox regulation of transglutaminase 2 activity. J. Biol. Chem..

[35] Skill NJ (2001). Increases in renal epsilon-(gamma-glutamyl)-lysine crosslinks result from compartment-specific changes in tissue transglutaminase in early experimental diabetic nephropathy: Pathologic implications. Lab. Investig. J. Tech. Methods Pathol..

[36] Seo E (2016). The Hippo-Salvador signaling pathway regulates renal tubulointerstitial fibrosis. Sci. Rep..

[37] Anorga S (2018). Deregulation of Hippo-TAZ pathway during renal injury confers a fibrotic maladaptive phenotype. FASEB J..

[38] Wong JS, Meliambro K, Ray J, Campbell KN (2016). Hippo signaling in the kidney: The good and the bad. Am. J. Physiol. Renal Physiol..

[39] Deng J (2019). Protective effect of rosiglitazone on chronic renal allograft dysfunction in rats. Transpl. Immunol..

[40] Caldas YA (2011). Liver X receptor-activating ligands modulate renal and intestinal sodium-phosphate transporters. Kidney Int..

[41] Liu P (2017). Transcriptomic and proteomic profiling provides insight into mesangial cell function in IgA nephropathy. J. Am. Soc. Nephrol..

[42] Qin L, Guo J, Zheng Q, Zhang H (2016). BAG2 structure, function and involvement in disease. Cell. Mol. Biol. Lett..

[43] Nagai T (2016). Differential regulation of angiotensin II-induced extracellular signal regulated kinase-1/2 and -5 in progressive glomerulonephritis. Nephrology.

[44] Luo F (2016). Mitogen-activated protein kinases and hypoxic/ischemic nephropathy. Cell. Physiol. Biochem..

[45] Nakagawa J (2002). Molecular characterization of mammalian dicarbonyl/L-xylulose reductase and its localization in kidney. J. Biol. Chem..

[46] Delgado-Reyes CV, Garrow TA (2005). High sodium chloride intake decreases betaine-homocysteine S-methyltransferase expression in guinea pig liver and kidney. Am. J. Physiol. Regul. Integrat. Comp. Physiol..

[47] Cassol CA (2019). Immunostaining for galactose-deficient immunoglobulin A is not specific for primary immunoglobulin A nephropathy. Nephrol. Dialysis Transplant..

[48] Boils CL, Nasr SH, Walker PD, Couser WG, Larsen CP (2015). Update on endocarditis-associated glomerulonephritis. Kidney Int..

[49] Hobeika L, Barati MT, Caster DJ, McLeish KR, Merchant ML (2017). Characterization of glomerular extracellular matrix by proteomic analysis of laser-captured microdissected glomeruli. Kidney Int..

[50] Kaya B (2017). Automated fluorescent miscroscopic image analysis of PTBP1 expression in glioma. PLoS One.

[51] Love MI, Huber W, Anders S (2014). Moderated estimation of fold change and dispersion for RNA-seq data with DESeq2. Genome Biol..

[52] Gordon AM, Schneider JA, Chinnan A, Charles JR (2007). Efficacy of a hand-arm bimanual intensive therapy (HABIT) in children with hemiplegic cerebral palsy: A randomized control trial. Dev. Med. Child Neurol..

[53] Soares MF (2019). Relationship between renal CD68(+) infiltrates and the Oxford Classification of IgA nephropathy. Histopathology.

