# Supplementary material for: Differentiating Staphylococcus infection-associated glomerulonephritis and primary IgA nephropathy: a mass spectrometry-based exploratory study
Source: Sci Rep. 2020 Oct 14;10:17179. doi: 10.1038/s41598-020-73847-x (PMC7560901; doi:10.1038/s41598-020-73847-x)
Supplement: Supplementary file 1 — Supplementary Information [file 41598_2020_73847_MOESM1_ESM.pdf]

Differentiating Staphylococcus infection-associated glomerulonephritis and primary IgA nephropathy – A mass spectrometry-based exploratory study.

Satoskar AA, Shapiro J, Jones, M, Bott C, Parikh S, Brodsky SV, Yu L, Nagaraja HN, Wilkey D, Merchant ML, Klein JB, Nadasdy T, Rovin BH.

Supplemental File 1.

Protocol for preparation of tissue for laser capture:

Four 10 micron sections were cut from the paraffin-embedded tissue blocks and mounted on thermoplastic (polyethylenenapthalate covered) glass slides (Carl Zeiss MicroImaging). Blades and water bath were changed before each case. Slides were processed as previously described (20). They were placed in a desiccator for at least one week, deparaffinized, in decreasing strengths of ethanol and stained with hematoxylin. Following hematoxylin staining slides were dehydrated in ethanol 70, 90 and 100%), air dried and immediately used for laser capture on the PALM technology microdissection system. Sections were microdissected under a 10x ocular lens. For each biopsy, glomerular and tubulointerstitial compartments were collected separately. The cut elements were catapulted into 25ul of 0.5% Rapigest (Waters Corporation, MA, USA), resuspended in 50 mM ammonium bicarbonate, collected in a 0.2 ml tube, and stored at -80◦C until the time of protein retrieval. Samples were thawed briefly, boiled for 20 minutes and then further heated at 60°C for 2 hours. Trypsin was added in a ratio of 1:30 trypsin:protein assuming ~2 µg retrieved protein/10,000 isolated cells (17). After overnight incubation at 37°C, formic acid was added to a final concentration of 30% and the suspension was incubated for 30 min at 37°C to degrade and render the Rapigest compatible with LC-MS/MS analysis. The extracts were dried and peptides were re-suspended in 20 µl of a solution of 2% acetonitrile with 0.1% formic acid and sonicated for 1 min in a water bath sonicator at 4°C to ensure peptide solubilization. Peptide concentration was obtained at 280 nm absorbance using 1 µl of sample on a Nanodrop ND-1000 spectrometer.

Differentiating Staphylococcus infection-associated glomerulonephritis and primary IgA nephropathy – A mass spectrometry-based exploratory study.

Satoskar AA, Shapiro J, Jones, M, Bott C, Parikh S, Brodsky SV, Yu L, Nagaraja HN, Wilkey D, Merchant ML, Klein JB, Nadasdy T, Rovin BH.

SupplementaL File 2

Proteomic Analysis of Laser Capture Tissue.

Tissue was digested and trap cleaned as described previously.[48] Peptide values were estimated using absorbance measurements at 205nm by NanoDrop (Thermo-Fisher Scientific, Waltham, MA). Peptide samples (1.5µg) were separated with a 3h 1D gradient using an Proxeon EASY n-LC (Thermo-Fisher Scientific) UHPLC system and Dionex (Sunnyvale, CA) Acclaim PepMap 100 75µm x 2cm, nanoViper (C18, 3µm, 100Å) trap, and a Dionex Acclaim PepMap RSLC 50µm x 15cm, nanoViper (C18, 2µm, 100Å) separating column. The sample was introduced at a flow rate of 300nL/min into an LTQ-Orbitrap ELITE (Thermo-Fisher Scientific) using a Nanospray Flex source with the ion transfer capillary temperature of the mass spectrometer was set at 225°C, and the spray voltage was set at 1.6kV. Data were acquired with an approach known as an Nth Order Double Play with electron transfer dissociation (ETD) Decision Tree method to exploit peptide fragmentation data acquisition by ETD and CID approaches. Scan event one of the method obtained an FTMS MS1 scan (normal mass range; 60,000 resolution, full scan type, positive polarity, profile data type) for the range 300-2000m/z. Scan event two obtained ITMS MS2 scans (normal mass range, rapid scan rate, centroid data type) on up to ten peaks that had a minimum signal threshold of 10,000 counts from scan event one. A decision tree was used to determine whether CID or ETD activation was used. An ETD scan was triggered if any of the following held: an ion had charge state 3 and m/z less than 650, an ion had charge state 4 and m/z less than 900, an ion had charge state 5 and m/z less than 950, or an ion had charge state greater than 5; a CID scan was triggered in all other cases. The lock mass option was enabled (0% lock mass abundance) using the 371.101236m/z polysiloxane peak as an internal calibrant. The collected data were analyzed by Proteome Discoverer v1.4.1.114 using Mascot v2.5.1 and SequestHT searches with the 4/16/2014 version of the UniprotKB Homo sapiens reference proteome canonical and isoform sequences. In order to estimate the false discovery rate, a

Target Decoy PSM Validator node was included in the Proteome Discoverer workflow. The resulting .msf files from Proteome Discoverer were loaded into Scaffold Q+S v4.4.1 for comparative proteomics. Scaffold was used to calculate the false discovery rate using the Peptide and Protein Prophet algorithms. The Scaffold delta-mass correction was enable during loading to accommodate instrumental mass measurement drift in Orbitrap data. The results were annotated with human gene ontology information from the Gene Ontology Annotations Database (ftp.ebi.ac.uk).

LC-MS/MS Data Collection

To ensure consistent performance of the LC-MS/MS system across sample collection periods, quality control standards for both LC retention times and MS1 peak characteristics were run prior to data collection on glomerular or tubulointerstitial samples. Lys-C (Promega, Madison, WI, USA) digests of equine skeletal muscle apomyoglobin (Sigma-Aldrich, St. Louis, MO, USA) were diluted 1:200 with 2% v/v acetonitrile / 0.1% v/v formic acid and 100fmol injected on column. Data were collected for five apomyoglobin peaks (501.5613m/z, z=+3; 650.3144m/z, z=+1; 454.2576m/z, z=+3; 689.9245m/z, z=+2; 908.4549m/z, z=+2) eluting across the LC gradient. The deviation of retention times and peak intensities more than 10% resulted in reconditioning and recalibration of the respective system.

Supplementary File 3. Staining protocols for Primary antibodies.						
Antibody	Dilution/Incubation	H2O2 Block	Retrieval	Detection	Chromogen	Hematoxylin
TGM2 Novus Biologicals, USA Rabbit polyclonal NBP2-24470	1:500/60min	Dako FLEX Perox Block 7 minutes	Low pH 25minutes	Dako FLEX HRP 30mins	FLEX DAB 10min	FLEX Hematoxylin 5minutes
DCXR ThermoFisher Scientific USA, Goat polyclonal Ab, PA1-31039	1:1000/60min	3% H2O2 7 minutes	Low pH 25minutes	Rabbit anti-goat secondary (1:200 w/2% normal rabbit serum) Vectastain Elite ABC HRP 30 min	DAKO DAB+ 10min	FLEX Hematoxylin 5minutes
CKBB	1:100/60min	Dako FLEX Perox Block 7 minutes	Low pH 25minutes	Dako FLEX HRP 30mins	FLEX DAB 10min	FLEX Hematoxylin 5minutes
Lysozyme DAKO rabbit polyclonal A0099	1:6,000/15min	Leice Bond Perox Block 5 minutes	Low pH 20minutes	Leica Bond Polymer Refine Post Primary 8minutes Polymer 8minutes	Leica Bond DAB 10min	Leica Bond Hematoxylin 3minutes
S100A9 Abcam Rabbit mAb ab92468	1:1,ooo/60min	Dako FLEX Perox Block 7 minutes	Low pH 25 minutes	Dako FLEX HRP 30mins	FLEX DAB 10min	FLEX Hematoxylin 5minutes

Differentiating Staphylococcus infection-associated glomerulonephritis and primary IgA nephropathy – A mass spectrometry-based exploratory study.

Satoskar AA, Shapiro J, Jones, M, Bott C, Parikh S, Brodsky SV, Yu L, Nagaraja HN, Wilkey, D, Merchant ML, Klein JB, Nadasdy T, Rovin BH.

Supplementary File 4.
Methods for Double staining and colocalization:

Tissue Collection

Immunohistochemistry

Before staining with the primary antibody human tissue samples were quenched with ____ to reduce autofluorescence and false secondary signal (Supplementary Figure 2).

Image Capture

Glomeruli images were captured on a Carl Zeiss Axio Imager Z.1 with LSM700 confocal microscope. This microscope used a motorized stage at 20X objective with a pinhole of 0.5 Airy unit, scan speed of 8, and a resolution of 512x512 to capture images.

Image Analysis

For each marker pair ten images captured and percent colocalization was calculated using a custom image analysis workflow in R (Supplementary 1).

Results:

Figure 1. Kidney inflammation was assessed utilizing two marker pairs: (A-C) CD68 and Lysozyme in contrast with (D-F) CD68 and S100A9. Quantification of (G) percent colocalization was calculated using randomly selected glomeruli (n=10 per group).

Supplementary Material:

Immunofluorescent Quenching

Figure 2. Quenching of human tissue autofluorescence to minimize background noise and false secondary signal.

1. *Custom image analysis workflow in R*

The following custom function was used to identify the colocalization of markers

CD68/Lysozyme and CD68/S100A9.

```
nuclei_count <- function(x, y)  {
  library(EBImage)
    CD68 <- readImage(x)
  CD68<- CD68 [,,1]
  CD68 <- na.omit(CD68)
  Lysozyme(S100A9)<- readImage(y)
  Lysozyme(S100A9)<- Lysozyme(S100A9) [,,1]
  Lysozyme(S100A9) <- na.omit(Lysozyme(S100A9))

  my_auto_thresh <- function(x) {
    library(imager)

    img <- threshold(x, thr = 'auto')
    detach(package:imager)
    return(img)
    }

  Lysozyme(S100A9)_thresh <- my_auto_thresh(Lysozyme(S100A9))
    Lysozyme(S100A9)_brush <- opening(  Lysozyme(S100A9)_thresh, makeBrush(5, shape =
'disc'))
  Lysozyme(S100A9)_bw <- bwlabel(  Lysozyme(S100A9)_brush)


    CD68_thresh <- my_auto_thresh(  CD68)
    CD68_brush <- opening(CD68_thresh, makeBrush(5, shape = 'disc'))
    CD68_bw <- bwlabel(CD68_brush)


  collage_2 <- computeFeatures.basic(CD68_bw,   Lysozyme(S100A9)_bw, na.rm = TRUE)
  df <- as.data.frame(collage_2)

  newvar <- (nrow(subset(df, df$b.mean > 0))/nrow(df))*100

  merged_image <- rgbImage(green = CD68_bw, red =   Lysozyme(S100A9)_bw)

  data <- paste("The percent of CD68 positive cells that are also   Lysozyme(S100A9) positive
equals", as.character(newvar))
```

```
  writeImage(merged_image, files = paste(x, "merged.jpg"))

  mylist = list("data" = data, "CD68" =  CD68_bw, "  Lysozyme(S100A9)" =
Lysozyme(S100A9)_bw, 'merged' = merged_image, 'rawLysozyme(S100A9)' =
Lysozyme(S100A9), 'rawCD68' = CD68)
  return(mylist)
}
```

Data searched using Mascot v2.5.1 and SequestHT as part of Proteome Discoverer 1.4.1.14. Data expo
Total Spectrum Count exported considering 1% FDR filtering for proteins and peptides.
Data are included for all proteins observed in at least one patient sample with a minimum of two pept
Data files for acquired LCMS data (.RAW), for peak lists (.mgf), and scaffold search results (.sf3) files alo

Sample Key

Sample	Cohort	Sample	Cohort
T24	ATN, other causes	G24	ATN, other causes
T25	ATN, other causes	G25	ATN, other causes
T26	ATN, other causes	G26	ATN, other causes
T27	ATN, other causes	G27	ATN, other causes
T08	IgAN E0C0	G08	IgAN E0C0
T09	IgAN E0C0	G09	IgAN E0C0
T10	IgAN E0C0	G10	IgAN E0C0
T04	IgAN E0C1	G04	IgAN E0C1
T20	IgAN E1C1	G23	IgAN E0C1
T21	IgAN E1C1	G20	IgAN E1C1
T23	IgANE0C1	G21	IgAN E1C1
T05	Normal kidney	G05	Normal kidney
T06	Normal kidney	G06	Normal kidney
T13	Normal kidney	G13	Normal kidney
T14	Normal kidney	G14	Normal kidney
T15	Normal kidney	G15	Normal kidney
T28	Normal kidney	G28	Normal kidney
T29	Normal kidney	G29	Normal kidney
T30	Normal kidney	G30	Normal kidney
T01	SAGN	G01	SAGN
T02	SAGN	G02	SAGN
T11	SAGN	G11	SAGN
T12	SAGN	G12	SAGN
T16	Vancomycin ATN	G16	Vancomycin ATN
T17	Vancomycin ATN	G17	Vancomycin ATN
T18	Vancomycin ATN	G18	Vancomycin ATN
T19	Vancomycin ATN	G19	Vancomycin ATN

ɔrted from exported from Scaffold v4.4.1.

ides.
ɔng with a sample key and sequence database were deposited in MassIVE (http://massive.ucsd.edu/) da

ıta repository (MassIVE ID: MSV000085473) with the Center for Computational Mass Spectrometry at th

ıe University of California, San Diego and shared with the ProteomeXchange (www.proteomexchange.oı

rg) (Proteome Exchange ID: PXD019422).

#Tubules_S

Tubules_Sa
#
1
2
3
4
5
6
7
8
9
10
11
12
13
14
15
16
17
18
19
20
21
22
23
24
25
26
27
28
29
30
31
32
33
34
35
36
37
38
39
40
41
42
43

1454
1455
1456
1457
1458
1459
1460
1461
1462
1463
1464
1465
1466
1467
1468
1469
1470
1471
1472
1473
1474
1475
1476
1477
1478
1479
1480
1481
1482
1483
1484
1485
1486
1487
1488
1489
1490
1491
1492
1493
1494
1495
1496
1497
1498
1499
1500

1501
1502
1503
1504
1505
1506
1507
1508
1509
1510

atoskar_141220, Samples Report created on 05/15/2020

toskar_141220
Identified Proteins
Myosin-9
Vimentin
Collagen alpha-3(VI) chain
Isoform 2 of Filamin-A
Isoform 2 of Keratin, type II cytoskeletal 8
Keratin, type I cytoskeletal 9
Actin, cytoplasmic 2
Keratin, type II cytoskeletal 1
Isoform 3 of Spectrin alpha chain, non-erythrocytic 1
Keratin, type II cytoskeletal 2 epidermal
Basement membrane-specific heparan sulfate proteoglycan core protein
Actin, alpha cardiac muscle 1
Prelamin-A/C
Alpha-enolase
Isoform 2 of Myosin-10
ATP synthase subunit beta, mitochondrial
Moesin
Keratin, type I cytoskeletal 10
Tubulin beta chain
Keratin, type I cytoskeletal 18
Tubulin beta-4B chain
ATP synthase subunit alpha, mitochondrial
Isoform 2 of Tropomyosin alpha-3 chain
Spectrin beta chain, non-erythrocytic 1
Low-density lipoprotein receptor-related protein 2
Isoform 3 of Tropomyosin alpha-1 chain
Pyruvate kinase PKM
Isoform 2 of Filamin-B
Alpha-actinin-4
Major vault protein
Isoform 3 of Plectin
Tropomyosin alpha-4 chain
Keratin, type I cytoskeletal 14
Tubulin beta-2A chain
Glyceraldehyde-3-phosphate dehydrogenase
Isoform 2 of Clathrin heavy chain 1
78 kDa glucose-regulated protein
Cytosolic non-specific dipeptidase
Tropomyosin 1 (Alpha), isoform CRA_m
Aconitate hydratase, mitochondrial
Talin-1
Isoform 4 of Sodium/potassium-transporting ATPase subunit alpha-1
Isoform 1 of Vinculin

Isoform 17 of Fibronectin
Keratin, type II cytoskeletal 5
Tubulin alpha-1B chain
Isoform 2 of Tropomyosin beta chain
Glycine amidinotransferase, mitochondrial
60 kDa heat shock protein, mitochondrial
Isoform 2 of Annexin A2
Heat shock protein HSP 90-beta
Isoform 2 of Alpha-actinin-1
Annexin A4
Heat shock cognate 71 kDa protein
Tubulin alpha-1C chain
Keratin, type II cytoskeletal 6C
Keratin, type II cytoskeletal 6B
L-lactate dehydrogenase B chain
Stress-70 protein, mitochondrial
Apoptosis-inducing factor 1, mitochondrial
Histone H4
Protein disulfide-isomerase A3
Heterogeneous nuclear ribonucleoproteins A2/B1
Ezrin
Delta-1-pyrroline-5-carboxylate dehydrogenase, mitochondrial
Trifunctional enzyme subunit alpha, mitochondrial
Alpha-crystallin B chain
Endoplasmin
Hemoglobin subunit alpha
Histone H2A type 2-A
Cytoplasmic dynein 1 heavy chain 1
Isoform 4 of Tenascin
Keratin, type I cytoskeletal 19
Histone H2A type 1
Glutamate dehydrogenase 1, mitochondrial
Acetyl-CoA acetyltransferase, mitochondrial
Isoform 2 of Heat shock protein HSP 90-alpha
Heat shock protein beta-1
Isoform 5 of Caldesmon
Heterogeneous nuclear ribonucleoprotein A1
Isoform 2 of Collagen alpha-1(XIV) chain
Keratin, type I cytoskeletal 16
Histone H2A type 1-C
3-ketoacyl-CoA thiolase, mitochondrial
Phosphoenolpyruvate carboxykinase [GTP], mitochondrial
Fructose-bisphosphate aldolase B
Methylmalonate-semialdehyde dehydrogenase [acylating], mitochondrial
Aldehyde dehydrogenase, mitochondrial
Elongation factor Tu, mitochondrial
Very long-chain specific acyl-CoA dehydrogenase, mitochondrial

14-3-3 protein epsilon
Hemoglobin subunit beta
Laminin subunit alpha-5
Heterogeneous nuclear ribonucleoprotein K
Ubiquitin-40S ribosomal protein S27a
Tubulin alpha-4A chain
Beta-actin-like protein 2
Aminopeptidase N
Ubiquitin-like modifier-activating enzyme 1
Annexin A6
Enoyl-CoA hydratase, mitochondrial
Transitional endoplasmic reticulum ATPase
Retinal dehydrogenase 1
Peptidyl-prolyl cis-trans isomerase A
Prohibitin
Junction plakoglobin
Acyl-coenzyme A synthetase ACSM2B, mitochondrial
Phosphoglycerate kinase 1
Cubilin
Isocitrate dehydrogenase [NADP], mitochondrial
Neuroblast differentiation-associated protein AHNAK
Peroxiredoxin-1
Malate dehydrogenase, mitochondrial
Isoform 2 of Transketolase
Calmodulin
Collagen alpha-2(IV) chain
Isoform Smooth muscle of Myosin light polypeptide 6
Heterogeneous nuclear ribonucleoprotein M
Profilin-1
14-3-3 protein zeta/delta
EMILIN-1
Protein-glutamine gamma-glutamyltransferase 2
Cytochrome b-c1 complex subunit 2, mitochondrial
Isoform 2 of Propionyl-CoA carboxylase alpha chain, mitochondrial
Heat shock 70 kDa protein 1A/1B
Aldehyde dehydrogenase X, mitochondrial
Carbonyl reductase [NADPH] 1
Collagen alpha-1(VI) chain
T-complex protein 1 subunit beta
Isoform 2 of Cytosol aminopeptidase
Dihydropyrimidinase-related protein 2
Ig gamma-1 chain C region
X-ray repair cross-complementing protein 6
Peroxisomal multifunctional enzyme type 2
Transgelin
Keratin, type II cytoskeletal 7
Acyl-coenzyme A synthetase ACSM2A, mitochondrial

Heterogeneous nuclear ribonucleoprotein A3
Keratin, type I cytoskeletal 13
Phosphatidylethanolamine-binding protein 1
Trifunctional enzyme subunit beta, mitochondrial
V-type proton ATPase subunit B, brain isoform
Collagen alpha-1(XVIII) chain
Annexin A5
Peroxiredoxin-2
Quinone oxidoreductase
Isoform 2 of Gelsolin
Laminin subunit gamma-1
Heterogeneous nuclear ribonucleoprotein U
Isoform Short of 14-3-3 protein beta/alpha
Cytochrome c oxidase subunit 2
Histone H3.3
Myosin regulatory light chain 12B
Radixin
Heterogeneous nuclear ribonucleoprotein H2
Isoform 2 of Histone H2B type 2-F
Argininosuccinate synthase
Pyruvate carboxylase, mitochondrial
Catalase
2-oxoglutarate dehydrogenase, mitochondrial
Cytoplasmic aconitate hydratase
Succinyl-CoA ligase [GDP-forming] subunit beta, mitochondrial
Propionyl-CoA carboxylase beta chain, mitochondrial
Fumarate hydratase, mitochondrial
Rab GDP dissociation inhibitor beta
Glutathione S-transferase P
NAD(P) transhydrogenase, mitochondrial
Elongation factor 1-alpha 1
Isoform 2 of Adenylate kinase 2, mitochondrial
Isoform 2 of Pyruvate dehydrogenase E1 component subunit beta, mitochondrial
Histone H2B type 1-O
Isoform 2 of Mitochondrial inner membrane protein
Cathepsin D
Cadherin-16
Nucleolin
Isoform 4 of Superoxide dismutase [Mn], mitochondrial
Annexin A1
Ig kappa chain C region
Collagen alpha-2(VI) chain
Heterogeneous nuclear ribonucleoprotein F
Isoform 1 of Ribosome-binding protein 1
LIM and SH3 domain protein 1
Collagen alpha-1(XII) chain
Isoform 2 of Nidogen-2

Heterogeneous nuclear ribonucleoprotein H
Serum albumin
Alcohol dehydrogenase [NADP(+)]
Isoform 2 of NADH-ubiquinone oxidoreductase 75 kDa subunit, mitochondrial
3-hydroxyisobutyryl-CoA hydrolase, mitochondrial
3-hydroxyacyl-CoA dehydrogenase type-2
Peroxiredoxin-6
ADP/ATP translocase 2
Protein disulfide-isomerase A6
Isoform 2 of Electron transfer flavoprotein subunit beta
Elongation factor 2
Isoform 2 of Fructose-bisphosphate aldolase A
Transgelin-2
Dihydrolipoyllysine-residue succinyltransferase component of 2-oxoglutarate dehydrogenase complex, mitochonc
Protein DJ-1
ATP synthase subunit O, mitochondrial
T-complex protein 1 subunit alpha
Ras GTPase-activating-like protein IQGAP1
Splicing factor, proline- and glutamine-rich
Unconventional myosin-VI
Dolichyl-diphosphooligosaccharide--protein glycosyltransferase subunit 1
Lamin-B2
Isoform 2 of Nidogen-1
Biglycan
Heterogeneous nuclear ribonucleoproteins C1/C2
Thrombospondin-1
Isoform 3 of Heterogeneous nuclear ribonucleoprotein D0
Uromodulin, secreted form
Hemoglobin subunit delta
14-3-3 protein eta
Aminoacylase-1
Peroxisomal bifunctional enzyme
Betaine--homocysteine S-methyltransferase 1
Isoform 2 of Medium-chain specific acyl-CoA dehydrogenase, mitochondrial
Delta(3,5)-Delta(2,4)-dienoyl-CoA isomerase, mitochondrial
Isoform 2 of Triosephosphate isomerase
Isoform 2 of Serine hydroxymethyltransferase, cytosolic
Methylcrotonoyl-CoA carboxylase beta chain, mitochondrial
Isoform 2 of Electron transfer flavoprotein subunit alpha, mitochondrial
Na(+)/H(+) exchange regulatory cofactor NHE-RF1
Fructose-1,6-bisphosphatase 1
Neutral alpha-glucosidase AB
Cytochrome b-c1 complex subunit 1, mitochondrial
Protein disulfide-isomerase
Carbonic anhydrase 2
Glutathione peroxidase 3
Nucleophosmin

Dihydrolipoyl dehydrogenase, mitochondrial
Gamma-butyrobetaine dioxygenase
3-hydroxybutyrate dehydrogenase type 2
Isoform 3 of L-lactate dehydrogenase A chain
Ester hydrolase C11orf54
40S ribosomal protein S3
Cytochrome c oxidase subunit 4 isoform 1, mitochondrial
Eukaryotic initiation factor 4A-I
Retinol-binding protein 4
Ras-related protein Rab-7a
Collagen alpha-2(I) chain
Transforming growth factor-beta-induced protein ig-h3
Lysozyme C
Calponin-3
Tensin-1
14-3-3 protein gamma
Isoform 6 of Agrin
RNA-binding motif protein, X chromosome
40S ribosomal protein S3a
Cytoskeleton-associated protein 4
PDZ and LIM domain protein 5
Alpha-centractin
Versican core protein
V-type proton ATPase catalytic subunit A
Choline dehydrogenase, mitochondrial
Villin-1
Aspartate aminotransferase, mitochondrial
6-phosphofructokinase, liver type
2,4-dienoyl-CoA reductase, mitochondrial
10 kDa heat shock protein, mitochondrial
Aldo-keto reductase family 1 member C3
Purine nucleoside phosphorylase
Keratin, type II cytoskeletal 4
Isoform 3 of Malate dehydrogenase, cytoplasmic
Histone H1.4
Elongation factor 1-gamma
Creatine kinase B-type
ADP-ribosylation factor 1
Peroxiredoxin-5, mitochondrial
Cytochrome c oxidase subunit 5A, mitochondrial
40S ribosomal protein SA (Fragment)
Poly(rC)-binding protein 1
Isoform 2 of Ras-related protein Rab-5C
Cathepsin B
Non-POU domain-containing octamer-binding protein
Ferritin heavy chain
Stomatin-like protein 2, mitochondrial

Peptidyl-prolyl cis-trans isomerase B
Matrin-3
Rho GDP-dissociation inhibitor 1
Src substrate cortactin
Guanine nucleotide-binding protein subunit beta-2-like 1
Isoform 2 of Septin-2
Cytochrome c oxidase subunit 5B, mitochondrial
Vitronectin
Laminin subunit beta-2
Erythrocyte band 7 integral membrane protein
F-actin-capping protein subunit alpha-2
60S ribosomal protein L18
Lamin-B1
Complement C3
Glycine N-acyltransferase
Succinate dehydrogenase [ubiquinone] flavoprotein subunit, mitochondrial
Isoform 3 of Cytosolic 10-formyltetrahydrofolate dehydrogenase
Adenylate kinase 4, mitochondrial
Long-chain-fatty-acid--CoA ligase 1
Prohibitin-2
Glucose-6-phosphate isomerase
Adenosylhomocysteinase
Calreticulin
Glycerol-3-phosphate dehydrogenase [NAD(+)], cytoplasmic
Amine oxidase [flavin-containing] B
Ferritin light chain
3-hydroxyisobutyrate dehydrogenase, mitochondrial
Isoform 2 of Calcium-binding mitochondrial carrier protein Aralar2
UDP-glucuronosyltransferase 1-6
Catenin alpha-1
ES1 protein homolog, mitochondrial
Isoform 3 of NADH-cytochrome b5 reductase 3
4-trimethylaminobutyraldehyde dehydrogenase
Macrophage-capping protein
Cytochrome b5
Collagen alpha-1(I) chain
2-oxoglutarate dehydrogenase-like, mitochondrial
ATP synthase subunit gamma, mitochondrial
Tubulointerstitial nephritis antigen-like
Protein NipSnap homolog 1
Synaptic vesicle membrane protein VAT-1 homolog
Transthyretin
6-phosphogluconate dehydrogenase, decarboxylating
T-complex protein 1 subunit zeta
UTP--glucose-1-phosphate uridylyltransferase
NADH dehydrogenase [ubiquinone] iron-sulfur protein 3, mitochondrial
Collagen alpha-1(IV) chain

Gamma-glutamyltranspeptidase 1
Protein AMBP
T-complex protein 1 subunit epsilon
Fatty acid-binding protein, heart
T-complex protein 1 subunit gamma
Heat shock protein 75 kDa, mitochondrial
Acylpyruvase FAHD1, mitochondrial
S-formylglutathione hydrolase
Isoform 2 of Glutathione reductase, mitochondrial
Clusterin
Isoform 2 of Elongation factor 1-delta
Isoform 1 of Voltage-dependent anion-selective channel protein 2
T-complex protein 1 subunit theta
D-3-phosphoglycerate dehydrogenase
Fibrinogen gamma chain
Cystatin-B
Ig alpha-1 chain C region
Flavin reductase (NADPH)
UDP-glucuronosyltransferase 1-9
Isoform 2 of ATP synthase-coupling factor 6, mitochondrial
Actin-related protein 2
Heterogeneous nuclear ribonucleoprotein Q
Coatomer subunit delta
40S ribosomal protein S4, X isoform
Proteasome subunit beta type-1
Calpain-1 catalytic subunit
tRNA-splicing ligase RtcB homolog
Transmembrane emp24 domain-containing protein 10
Coiled-coil-helix-coiled-coil-helix domain-containing protein 3, mitochondrial
Complement component C9
Coagulation factor XIII A chain
Far upstream element-binding protein 2
Aspartate--tRNA ligase, cytoplasmic
Ladinin-1
Zyxin
14-3-3 protein theta
Haptoglobin
Rab GDP dissociation inhibitor alpha
Isoform 2 of Poly(rC)-binding protein 2
Histone H2A type 2-B
ADP-ribosylation factor 4
V-type proton ATPase subunit B, kidney isoform
Peroxiredoxin-4
Na(+)/H(+) exchange regulatory cofactor NHE-RF3
Leucine-rich PPR motif-containing protein, mitochondrial
Acyl-coenzyme A thioesterase 1
Aromatic-L-amino-acid decarboxylase

Isoform 2 of Alpha-aminoadipic semialdehyde dehydrogenase
Acyl-CoA synthetase family member 2, mitochondrial
4-aminobutyrate aminotransferase, mitochondrial
Aflatoxin B1 aldehyde reductase member 3
Dipeptidyl peptidase 4
Aspartate aminotransferase, cytoplasmic
Dihydropyrimidinase
Agmatinase, mitochondrial
Prostaglandin reductase 1
Alpha-1-antitrypsin
Glutaminase kidney isoform, mitochondrial
Amine oxidase [flavin-containing] A
Isoform 2 of ATP synthase subunit d, mitochondrial
Lambda-crystallin homolog
Phosphoglycerate mutase 1
C-1-tetrahydrofolate synthase, cytoplasmic
Isoform 2 of Fibrinogen alpha chain
Glyoxylate reductase/hydroxypyruvate reductase
Alpha-methylacyl-CoA racemase
Isoform 3 of Disabled homolog 2
Isoform 3 of 4F2 cell-surface antigen heavy chain
Lon protease homolog, mitochondrial
Succinyl-CoA ligase [ADP/GDP-forming] subunit alpha, mitochondrial
Voltage-dependent anion-selective channel protein 1
Cofilin-1
ATP-dependent RNA helicase A
Probable N-acetyltransferase 8
Complement C4-B
Adenylyl cyclase-associated protein 1
WD repeat-containing protein 1
ATP synthase F(0) complex subunit B1, mitochondrial
X-ray repair cross-complementing protein 5
Cytochrome b-c1 complex subunit Rieske, mitochondrial
Isoform 2 of Enoyl-CoA delta isomerase 2, mitochondrial
Cytochrome c
Protein disulfide-isomerase A4
Prenylcysteine oxidase 1
Staphylococcal nuclease domain-containing protein 1
Catenin beta-1
V-type proton ATPase subunit H
Heterogeneous nuclear ribonucleoprotein L
Fibrinogen beta chain
Isoform 2 of HLA class I histocompatibility antigen, A-11 alpha chain
Membrane-associated progesterone receptor component 1
Alpha-1-antichymotrypsin
Isoform 2 of Acyl-CoA-binding protein
Serine hydroxymethyltransferase, mitochondrial

60S ribosomal protein L6
Isoform 2 of Phosphatidylinositol-binding clathrin assembly protein
Isoform 2 of Heterogeneous nuclear ribonucleoprotein H3
Cytochrome c oxidase subunit 6C
Lumican
Hepatoma-derived growth factor
Isoform 2 of Dolichyl-diphosphooligosaccharide--protein glycosyltransferase subunit 2
NADH dehydrogenase [ubiquinone] 1 alpha subcomplex subunit 13
Isoform 3 of Protein SET
Ras-related protein Rab-14
Isoform 2 of Alpha-adducin
40S ribosomal protein S9
Bifunctional purine biosynthesis protein PURH
Ribonuclease inhibitor
Serpin H1
Isoform 1 of Core histone macro-H2A.1
Coronin-1B
Isoform 2 of SRA stem-loop-interacting RNA-binding protein, mitochondrial
Isoform 3 of Periostin
Heat shock 70 kDa protein 4
Isoform 2 of Spliceosome RNA helicase DDX39B
Isoform sGi2 of Guanine nucleotide-binding protein G(i) subunit alpha-2
Citrate lyase subunit beta-like protein, mitochondrial
60S ribosomal protein L13
Isoform 2 of Sarcoplasmic/endoplasmic reticulum calcium ATPase 2
40S ribosomal protein S16
60S ribosomal protein L7
PRA1 family protein 3
Secretory carrier-associated membrane protein 2
Glutathione S-transferase theta-1
Isoform 2 of Coronin-1C
Serine/arginine-rich splicing factor 1
Coatomer subunit alpha
Far upstream element-binding protein 1
60S ribosomal protein L3
Isoform 2 of ATP-citrate synthase
Isoform 2 of Cysteine-rich protein 2
Tumor protein D52
Isoform 2 of Syntaxin-7
Protein S100-A9
40S ribosomal protein S6
Vacuolar protein sorting-associated protein 26A
Ras-related protein Rab-10
Heterogeneous nuclear ribonucleoprotein R
S-adenosylmethionine synthase isoform type-2
Cytochrome b-c1 complex subunit 8
60S ribosomal protein L31

Rho GTPase-activating protein 1
Isoform 2 of N-acetyl-D-glucosamine kinase
Adenine phosphoribosyltransferase
HLA class I histocompatibility antigen, B-7 alpha chain
Sideroflexin-3
60S ribosomal protein L7a
Ras-related protein Rab-1B
Aldose reductase
PDZ and LIM domain protein 1
Palladin
Histone H2A.Z
Prostaglandin-H2 D-isomerase (Fragment)
Isoform 3 of NSFL1 cofactor p47
Vesicular integral-membrane protein VIP36
Haptoglobin (Fragment)
Isoform 2 of RNA-binding protein 39
Isoform PML-4 of Protein PML
Eukaryotic initiation factor 4A-III
Isoform 2 of 116 kDa U5 small nuclear ribonucleoprotein component
Semenogelin-1
Isoform 2 of Hydroxyacyl-coenzyme A dehydrogenase, mitochondrial
Isocitrate dehydrogenase [NADP] cytoplasmic
Sideroflexin-1
Citrate synthase, mitochondrial
4-hydroxy-2-oxoglutarate aldolase, mitochondrial
Isoform 2 of Isochorismatase domain-containing protein 2, mitochondrial
Dimethylaniline monooxygenase [N-oxide-forming] 1
Mitochondrial carrier homolog 2
Fumarylacetoacetase
Tubulointerstitial nephritis antigen
Complement component 1 Q subcomponent-binding protein, mitochondrial
Aflatoxin B1 aldehyde reductase member 2
Guanine deaminase
Calnexin
Isovaleryl-CoA dehydrogenase, mitochondrial
Laminin subunit beta-1
Sepiapterin reductase
Sorbitol dehydrogenase
Isoform Non-brain of Clathrin light chain A
Methylcrotonoyl-CoA carboxylase subunit alpha, mitochondrial
Chloride intracellular channel protein 4
Alpha/beta hydrolase domain-containing protein 14B
Isoform 2 of Thioredoxin-dependent peroxide reductase, mitochondrial
Sideroflexin-2
Isoform 2 of Fatty aldehyde dehydrogenase
Ribonuclease UK114
Isoform B of Phosphate carrier protein, mitochondrial

Corticosteroid 11-beta-dehydrogenase isozyme 2
Phosphoglucomutase-1
S-methylmethionine--homocysteine S-methyltransferase BHMT2
Glutathione S-transferase kappa 1
Coactosin-like protein
Isoform 1AC of Catenin delta-1
Cytochrome b-c1 complex subunit 7
T-complex protein 1 subunit delta
Vacuolar protein sorting-associated protein 35
Probable ATP-dependent RNA helicase DDX17
Transforming protein RhoA
Aldehyde oxidase
Ras-related protein Rab-1A
40S ribosomal protein S7
Actin-related protein 3
Alcohol dehydrogenase 1B
Isoform 2 of Programmed cell death 6-interacting protein
Acyl-CoA synthetase short-chain family member 3, mitochondrial
Aquaporin-1
Histidine triad nucleotide-binding protein 1
Isoform 2 of Ras-related protein Rab-11A
LETM1 and EF-hand domain-containing protein 1, mitochondrial
Glycine N-acyltransferase-like protein 1
Isoform 2 of Ethylmalonyl-CoA decarboxylase
Polymeric immunoglobulin receptor
Asparagine--tRNA ligase, cytoplasmic
NADH dehydrogenase [ubiquinone] flavoprotein 2, mitochondrial
cAMP-dependent protein kinase type II-alpha regulatory subunit
Nuclease-sensitive element-binding protein 1
Isoform 2 of Membrane-associated progesterone receptor component 2
Pentatricopeptide repeat-containing protein 1, mitochondrial
Annexin A11
Interleukin enhancer-binding factor 2
Macrophage migration inhibitory factor
Coatomer protein complex, subunit beta 2 (Beta prime), isoform CRA_b
Retinol dehydrogenase 11
Epidermal growth factor receptor kinase substrate 8-like protein 2
Cysteine and glycine-rich protein 1
Single-stranded DNA-binding protein, mitochondrial
40S ribosomal protein S8
60S acidic ribosomal protein P0
Proteasome subunit alpha type-6
Isoform 2 of Enoyl-CoA delta isomerase 1, mitochondrial
ATP synthase subunit delta, mitochondrial
Vesicle-trafficking protein SEC22b
Peptidyl-prolyl cis-trans isomerase FKBP4
Sorting nexin-2

Plastin-3
Isoform 3 of T-complex protein 1 subunit eta
Transcriptional activator protein Pur-alpha
40S ribosomal protein S18
60S acidic ribosomal protein P2
Isoform 2 of B-cell receptor-associated protein 31
Eukaryotic translation initiation factor 4H
Isoform 2 of ATP-dependent RNA helicase DDX3X
Isoform 2 of Carnitine O-acetyltransferase
Isoform 2 of Polyadenylate-binding protein 1
Isoform 2 of Serine/arginine-rich splicing factor 7
Translocon-associated protein subunit delta
60S ribosomal protein L12
Capping protein (Actin filament) muscle Z-line, beta, isoform CRA_d
Coatomer subunit gamma-1
Protein ARPC4-TTLL3
Proteasome activator complex subunit 2
Tubulin-specific chaperone A
26S proteasome non-ATPase regulatory subunit 3
Transcription intermediary factor 1-beta
Ubiquitin-conjugating enzyme E2 N
Drebrin-like protein
Tropomodulin-3
A-kinase anchor protein 12
Integrin beta-1
60S ribosomal protein L23a
Isoform 2 of Annexin A7
Proteasome subunit alpha type-4
Cysteine and glycine-rich protein 2
Coronin-1A
26S protease regulatory subunit 6A
60S ribosomal protein L22
Fructose-bisphosphate aldolase C
Isoform 2 of Tripeptidyl-peptidase 1
Rho GDP-dissociation inhibitor 2
Isoform 2 of Diablo homolog, mitochondrial
Proteasome subunit beta type-4
Thymidine phosphorylase
Extended synaptotagmin-1
Guanine nucleotide-binding protein G(I)/G(S)/G(T) subunit beta-2
Lamina-associated polypeptide 2, isoform alpha
Glutathione peroxidase 1
Isoform 2 of Adipocyte plasma membrane-associated protein
Vesicle-associated membrane protein 2
Cathepsin Z
UPF0317 protein C14orf159, mitochondrial
Isoform 2 of Heterogeneous nuclear ribonucleoprotein A/B

Isoform Short of RNA-binding protein FUS
40S ribosomal protein S26
RuvB-like 2
Ras-related protein R-Ras2
6-phosphofructokinase, muscle type
Serine/arginine-rich splicing factor 3
Isoform 2 of Vacuolar protein sorting-associated protein 29
Cytochrome b5 type B
60S ribosomal protein L5
BTB/POZ domain-containing protein KCTD12
Eukaryotic translation initiation factor 3 subunit A
Flotillin-1
Isoform XLas-2 of Guanine nucleotide-binding protein G(s) subunit alpha isoforms XLas
40S ribosomal protein S21
Isoform 2 of 26S proteasome non-ATPase regulatory subunit 11
THO complex subunit 4
Epiplakin
Pro-cathepsin H
F-actin-capping protein subunit alpha-1
Isoform 3 of Myoferlin
Myosin-13
Isoform DeltaLf of Lactotransferrin
DNA-(apurinic or apyrimidinic site) lyase
Fibrillin-1
General vesicular transport factor p115
Isoform 3 of Integrin alpha-V
Histone H1.5
Fermitin family homolog 2
Dermcidin
RuvB-like 1
Brain acid soluble protein 1
Isoform B of Fibulin-1
Serine/arginine repetitive matrix protein 2
Deoxynucleoside triphosphate triphosphohydrolase SAMHD1
Heterogeneous nuclear ribonucleoprotein A0
Ras-related protein Rap-2b
Band 4.1-like protein 2
Isoform 2 of Laminin subunit alpha-4
Isoform 2 of Insulin-like growth factor-binding protein 7
Annexin A3
Glutathione S-transferase A2
Bifunctional ATP-dependent dihydroxyacetone kinase/FAD-AMP lyase (cyclizing)
Dimethylglycine dehydrogenase, mitochondrial
Isoform 2 of Electrogenic sodium bicarbonate cotransporter 1
Formimidoyltransferase-cyclodeaminase
3-ketoacyl-CoA thiolase, peroxisomal
Dipeptidase 1

UDP-glucuronosyltransferase 2B7
Thiosulfate sulfurtransferase
Isoform C of Ketohexokinase
Short/branched chain specific acyl-CoA dehydrogenase, mitochondrial
Short-chain specific acyl-CoA dehydrogenase, mitochondrial
Bifunctional epoxide hydrolase 2
V-type proton ATPase subunit E 1
Maltase-glucoamylase, intestinal
Selenium-binding protein 1
Phenazine biosynthesis-like domain-containing protein
Enoyl-CoA hydratase domain-containing protein 3, mitochondrial
Carnitine O-palmitoyltransferase 2, mitochondrial
Carboxymethylenebutenolidase homolog
Epoxide hydrolase 1
NADH dehydrogenase [ubiquinone] flavoprotein 1, mitochondrial
3-mercaptopyruvate sulfurtransferase
Isoform 2 of Pyruvate dehydrogenase E1 component subunit alpha, somatic form, mitochondrial
Isoform 2 of Polypyrimidine tract-binding protein 1
NADH dehydrogenase [ubiquinone] 1 alpha subcomplex subunit 9, mitochondrial
Nicotinate phosphoribosyltransferase (Fragment)
Mitochondrial amidoxime reducing component 2
Isoform 12 of Titin
N(G),N(G)-dimethylarginine dimethylaminohydrolase 1
UMP-CMP kinase
Dihydropteridine reductase
Calbindin
Aldose 1-epimerase
Isoform 2 of Valacyclovir hydrolase
Homogentisate 1,2-dioxygenase
Isoform 2 of Glucosidase 2 subunit beta
UDP-glucose 6-dehydrogenase
Sulfite oxidase, mitochondrial
Isoform 2 of V-type proton ATPase 116 kDa subunit a isoform 1
2'-deoxynucleoside 5'-phosphate N-hydrolase 1 (Fragment)
Xaa-Pro dipeptidase
PDZK1-interacting protein 1
Isoform 2 of Eukaryotic translation initiation factor 5A-1
Isoform 2 of Inorganic pyrophosphatase 2, mitochondrial
Isoform 3 of Nucleoside diphosphate kinase B
V-type proton ATPase subunit D
Glutamyl aminopeptidase
OCIA domain-containing protein 2
Dihydrolipoyllysine-residue acetyltransferase component of pyruvate dehydrogenase complex, mitochondrial
Proteasome subunit alpha type-1
Apolipoprotein E
Omega-amidase NIT2
Mitochondrial 2-oxoglutarate/malate carrier protein

Basal cell adhesion molecule
Microtubule-associated protein
NADH dehydrogenase [ubiquinone] 1 alpha subcomplex subunit 10, mitochondrial
2-oxoisovalerate dehydrogenase subunit alpha, mitochondrial
Proteasome activator complex subunit 1
NADH dehydrogenase [ubiquinone] 1 beta subcomplex subunit 4
Tricarboxylate transport protein, mitochondrial
Leukotriene A-4 hydrolase
Serine/threonine-protein phosphatase 2A 65 kDa regulatory subunit A alpha isoform
Thioredoxin
EH domain-containing protein 4
Haloacid dehalogenase-like hydrolase domain-containing protein 3
Alcohol dehydrogenase class-3
Pterin-4-alpha-carbinolamine dehydratase
Isoform 2 of Ras-related protein Rap-1b
CDGSH iron-sulfur domain-containing protein 1
Transaldolase
Actin-related protein 2/3 complex subunit 2
Chloride intracellular channel protein 1
40S ribosomal protein S17-like
Isoform 2 of Mitochondrial peptide methionine sulfoxide reductase
Isoform 2 of Enoyl-CoA hydratase domain-containing protein 2, mitochondrial
Calpain small subunit 1
Activated RNA polymerase II transcriptional coactivator p15
Isoform 2 of Succinyl-CoA ligase [ADP-forming] subunit beta, mitochondrial
Protein ERGIC-53
Isoform 2 of Acid ceramidase
Valine--tRNA ligase
Protein S100-A6
Phosphoserine aminotransferase
Isoform 2 of AP-2 complex subunit alpha-2
Isoform 3 of AP-2 complex subunit beta
Electron transfer flavoprotein-ubiquinone oxidoreductase, mitochondrial
Sodium/glucose cotransporter 2
Methyltransferase-like protein 7A
Isoform 2 of Plasminogen activator inhibitor 1 RNA-binding protein
Alpha-crystallin A chain
NADH dehydrogenase [ubiquinone] 1 beta subcomplex subunit 5, mitochondrial
Isoform 1 of Gamma-adducin
Nucleobindin-1
NADH dehydrogenase [ubiquinone] 1 alpha subcomplex subunit 2
Peroxisomal sarcosine oxidase
Plastin-2
Protein NDRG1
Peroxisomal acyl-coenzyme A oxidase 2
6-phosphogluconolactonase
Glutathione S-transferase Mu 3

Aldehyde dehydrogenase family 8 member A1
V-type proton ATPase subunit d 1
Nascent polypeptide-associated complex subunit alpha, muscle-specific form
60S ribosomal protein L4
Isoform 2 of Gamma-glutamyltransferase 5
Reticulon-4
Cytochrome c1, heme protein, mitochondrial
26S proteasome non-ATPase regulatory subunit 2
Isocitrate dehydrogenase [NAD] subunit alpha, mitochondrial
PDZ and LIM domain protein 2
Serpin B6
Cytochrome c oxidase subunit 6A1, mitochondrial
Cytochrome c oxidase subunit 7A2, mitochondrial
Endoplasmic reticulum resident protein 29
AFG3-like protein 2
40S ribosomal protein S2
Proteasome subunit alpha type-2
Isoform 2 of 40S ribosomal protein S20
Proteasome subunit alpha type-7
Sorting nexin-3
Lipoma-preferred partner
40S ribosomal protein S10
Lysosome-associated membrane glycoprotein 1
Puromycin-sensitive aminopeptidase
Glycogen phosphorylase, brain form
40S ribosomal protein S14
Eukaryotic translation initiation factor 6
ATP synthase subunit e, mitochondrial
Septin-7
40S ribosomal protein S19
NADH-cytochrome b5 reductase 1
Isoform 2 of Glyoxalase domain-containing protein 4
GTP-binding nuclear protein Ran
N-acylglucosamine 2-epimerase
Isoform 2 of Histone H1.0
cAMP-dependent protein kinase type I-alpha regulatory subunit
Cold-inducible RNA-binding protein
AP-2 complex subunit mu
40S ribosomal protein S28
Isoleucine--tRNA ligase, mitochondrial
Sialate O-acetylesterase
Acidic leucine-rich nuclear phosphoprotein 32 family member A
Nicotinate-nucleotide pyrophosphorylase [carboxylating]
Acylamino-acid-releasing enzyme
Actin-related protein 2/3 complex subunit 3
Ubiquinone biosynthesis protein COQ9, mitochondrial
26S proteasome non-ATPase regulatory subunit 14

60S ribosomal protein L9
Delta-aminolevulinic acid dehydratase
Isoform Short of Ubiquitin carboxyl-terminal hydrolase 5
High mobility group protein B1
Sodium-dependent neutral amino acid transporter B(0)AT1
Isobutyryl-CoA dehydrogenase, mitochondrial
Proteasome subunit alpha type-5
Isoform 3 of Dynamin-2
Isoform 2 of Protein NipSnap homolog 2
NADH dehydrogenase [ubiquinone] 1 beta subcomplex subunit 10
Isoform 2 of WASH complex subunit FAM21A
Toll-interacting protein
PDZ domain-containing protein GIPC2
Isoform 4 of Thiosulfate sulfurtransferase/rhodanese-like domain-containing protein 1
HLA class II histocompatibility antigen, DRB1-15 beta chain
Isoform 2 of Thioredoxin domain-containing protein 5
Cadherin-6
Isoform 2 of Ras suppressor protein 1
Isoform 2 of Dynactin subunit 2
Isoform 4 of Dipeptidyl peptidase 3
Isoform 3 of RNA-binding protein EWS
60S ribosomal protein L19
SH3 domain-binding glutamic acid-rich-like protein 3
ATP-dependent RNA helicase DDX1
Succinyl-CoA:3-ketoacid coenzyme A transferase 1, mitochondrial
40S ribosomal protein S15a
Coatomer subunit beta
Phosphoglucomutase-2
SH3 domain-binding glutamic acid-rich-like protein
TAR DNA-binding protein 43
Lipoamide acyltransferase component of branched-chain alpha-keto acid dehydrogenase complex, mitochondrial
60S ribosomal protein L29
Legumain
Uncharacterized protein (Fragment)
Small nuclear ribonucleoprotein Sm D3
Isoform 3 of Unconventional myosin-Ic
NADH dehydrogenase [ubiquinone] 1 beta subcomplex subunit 9
Isoform 2 of Myosin light chain kinase, smooth muscle
Glutamine--tRNA ligase
26S protease regulatory subunit 7
Serine/arginine-rich splicing factor 2
Heme oxygenase 1
26S protease regulatory subunit 4
Nuclear mitotic apparatus protein 1
Desmoglein-1
Eukaryotic translation initiation factor 2 subunit 1
Isoform CNPI of 2',3'-cyclic-nucleotide 3'-phosphodiesterase

Cytochrome c oxidase assembly factor 3 homolog, mitochondrial
Isoform D of Eukaryotic translation initiation factor 4 gamma 1
Alanine--tRNA ligase, cytoplasmic
Isoform C of Fibulin-1
Transmembrane protein 205
Persulfide dioxygenase ETHE1, mitochondrial
Ubiquitin thioesterase OTUB1
Dolichyl-diphosphooligosaccharide--protein glycosyltransferase 48 kDa subunit
Isoform 8 of Protein transport protein Sec31A
60S ribosomal protein L8
Isoform 2 of Heterochromatin protein 1-binding protein 3
Multidrug resistance protein 1
Cystatin-A
Tissue alpha-L-fucosidase
Isoform B of Ras-related C3 botulinum toxin substrate 1
Importin subunit beta-1
Hsp90 co-chaperone Cdc37
N(G),N(G)-dimethylarginine dimethylaminohydrolase 2
Thioredoxin domain-containing protein 17
Protein SCO1 homolog, mitochondrial
FERM, RhoGEF and pleckstrin domain-containing protein 1
40S ribosomal protein S13
Galectin-3-binding protein
60S ribosomal protein L24
ELAV-like protein 1
Isoform 2 of Short stature homeobox protein 2
Destrin
Chromobox protein homolog 3
Isoform 2 of DDRGK domain-containing protein 1
HLA class II histocompatibility antigen, DR alpha chain
Elongation factor 1-beta
Heterogeneous nuclear ribonucleoprotein U-like protein 2
Ragulator complex protein LAMTOR1
Stress-induced-phosphoprotein 1
Protein S100-A1
Isoform 4 of 40S ribosomal protein S24
Thymosin beta-4
UDP-glucuronosyltransferase 2A3
60S ribosomal protein L27a
Erlin-2
Lysosomal protective protein
Translationally-controlled tumor protein
Protein NEDD8-MDP1
Isoform 3 of Thioredoxin reductase 1, cytoplasmic
Isoform 2 of HLA class II histocompatibility antigen gamma chain
Xaa-Pro aminopeptidase 1
Protein FAM162A

Isoform 4 of Band 4.1-like protein 1
Fatty acid synthase
Filaggrin-2
Keratin, type II cytoskeletal 78
Long-chain-fatty-acid--CoA ligase 3
Isoform 2 of Beta-galactosidase
Malectin
Proline synthase co-transcribed bacterial homolog protein
Signal transducer and activator of transcription 1-alpha/beta
Lysosomal alpha-glucosidase
Integrin-linked protein kinase
Isoform Alpha-6X1B of Integrin alpha-6
Type-1 angiotensin II receptor-associated protein
Transformer-2 protein homolog beta
Ubiquilin-4
N-acetylglucosamine-6-sulfatase
Isoform 2 of Glutamine--fructose-6-phosphate aminotransferase [isomerizing] 1
Adseverin
Mitochondrial import receptor subunit TOM70
Cleavage and polyadenylation-specificity factor subunit 6
Estradiol 17-beta-dehydrogenase 12
Isoform 2 of DAZ-associated protein 1
Obg-like ATPase 1
Adaptin ear-binding coat-associated protein 2
Eukaryotic translation initiation factor 3 subunit G
39S ribosomal protein L1, mitochondrial (Fragment)
Asporin
GDP-L-fucose synthase
Isoform F of Solute carrier family 12 member 1
Flotillin-2
Ig kappa chain V-III region CLL
Isocitrate dehydrogenase [NAD] subunit gamma, mitochondrial
Cell cycle and apoptosis regulator protein 2
Isoform 4 of LIM domain and actin-binding protein 1
Actin-related protein 2/3 complex subunit 1B
Fascin
Programmed cell death protein 5
Arylsulfatase E
Isoform 2 of Lipopolysaccharide-responsive and beige-like anchor protein
Neprilysin
L-xylulose reductase
Phosphoenolpyruvate carboxykinase, cytosolic [GTP]
Isoform B of Band 4.1-like protein 3
Probable proline dehydrogenase 2
Desmoplakin
Isoform 2 of Peroxisomal acyl-coenzyme A oxidase 1
Sarcosine dehydrogenase, mitochondrial

Isoform L-type of Pyruvate kinase PKLR
Glycine dehydrogenase (decarboxylating), mitochondrial
Apolipoprotein A-I
Ketimine reductase mu-crystallin
Fatty acid-binding protein, liver
Isoform 2 of Succinate-semialdehyde dehydrogenase, mitochondrial
Apolipoprotein A-IV
Very long-chain acyl-CoA synthetase
Mitochondrial dicarboxylate carrier
Cocaine esterase
Isoform 2 of 4-hydroxyphenylpyruvate dioxygenase
Glutaryl-CoA dehydrogenase, mitochondrial
Hydroxysteroid dehydrogenase-like protein 2
Isoform 2 of Amiloride-sensitive amine oxidase [copper-containing]
Hydroxymethylglutaryl-CoA lyase, mitochondrial
Ribosyldihydronicotinamide dehydrogenase [quinone]
NADH dehydrogenase [ubiquinone] iron-sulfur protein 2, mitochondrial
Hypoxia up-regulated protein 1
GTP:AMP phosphotransferase AK3, mitochondrial
Pyridoxine-5'-phosphate oxidase
Isoform 2 of Sodium/potassium-transporting ATPase subunit beta-1
Putative L-aspartate dehydrogenase
Ankyrin-3
28S ribosomal protein S36, mitochondrial
Methylmalonyl-CoA mutase, mitochondrial
Dehydrogenase/reductase SDR family member 4
Periplakin
Isoform 2 of Argininosuccinate lyase
Acetolactate synthase-like protein
Succinate dehydrogenase [ubiquinone] iron-sulfur subunit, mitochondrial
Cornulin
Histidine triad nucleotide-binding protein 2, mitochondrial
Alanine--glyoxylate aminotransferase 2, mitochondrial
Isoform 2 of Myosin-11
Sodium-coupled monocarboxylate transporter 2
Gamma-glutamyl hydrolase
Galactokinase
Ras GTPase-activating-like protein IQGAP2
Cytochrome P450 4A11
Isoform 1 of Glycerol kinase
Xaa-Pro aminopeptidase 2
Glucosamine-6-phosphate isomerase 1
Tetratricopeptide repeat protein 38
Hydroxyacid oxidase 2
Phenylalanine-4-hydroxylase
Envoplakin
Pyridoxal kinase

Protein amnionless
Ornithine aminotransferase, mitochondrial
Serotransferrin
Keratin, type II cytoskeletal 3
Isoform 2 of Phosphotriesterase-related protein
Sulfide:quinone oxidoreductase, mitochondrial
Isoform 7 of Non-specific lipid-transfer protein
Mycophenolic acid acyl-glucuronide esterase, mitochondrial
Nesprin-1
Isoform 2 of Acyl-coenzyme A thioesterase 13
Isoform 2 of 26S protease regulatory subunit 6B
Ig gamma-2 chain C region
Protein transport protein Sec23A
Neutral and basic amino acid transport protein rBAT
Glutathione synthetase
Acyl-CoA dehydrogenase family member 11
Methylglutaconyl-CoA hydratase, mitochondrial
ATP synthase subunit g, mitochondrial
Hsc70-interacting protein
EH domain-containing protein 1
Protein FAM151A
Junction plakoglobin
Isoform 2 of Probable D-lactate dehydrogenase, mitochondrial
Small nuclear ribonucleoprotein Sm D1
Small integral membrane protein 24
1,4-alpha-glucan-branching enzyme
Isoform 2 of Interleukin enhancer-binding factor 3
NADH dehydrogenase [ubiquinone] 1 alpha subcomplex subunit 12
Plastin-1
Estradiol 17-beta-dehydrogenase 8
NADH dehydrogenase [ubiquinone] 1 alpha subcomplex subunit 6
Fructosamine-3-kinase
Microsomal glutathione S-transferase 1
FRAS1-related extracellular matrix protein 2
V-type proton ATPase subunit G 1
Carnitine O-palmitoyltransferase 1, liver isoform
Beta-2-microglobulin
Complement factor D
Proteasome subunit beta type-3
Proteasome subunit beta type-7
NADH dehydrogenase [ubiquinone] 1 alpha subcomplex subunit 5
Isoform 2 of V-type proton ATPase subunit F
Secernin-2
NADPH--cytochrome P450 reductase
Isoform Fetal-tau of Microtubule-associated protein tau
Alpha-2-macroglobulin
Glutaredoxin-1

Gamma-glutamylaminecyclotransferase
Microsomal glutathione S-transferase 3
Isoform 2 of Ig mu chain C region
NADH dehydrogenase [ubiquinone] 1 alpha subcomplex subunit 4
Bcl-2-like protein 13
Hydroxyacylglutathione hydrolase, mitochondrial
NADH dehydrogenase (Ubiquinone) 1 beta subcomplex, 8, 19kDa, isoform CRA_a
Isoform 2 of Acetyl-coenzyme A synthetase 2-like, mitochondrial
Elongation factor G, mitochondrial
Ras-related protein Rab-2A
D-dopachrome decarboxylase
ATP-binding cassette sub-family D member 3
Spectrin beta chain, non-erythrocytic 2
Isoform 2 of Bifunctional coenzyme A synthase
Calmodulin-like protein 3
Microtubule-actin cross-linking factor 1, isoforms 1/2/3/5
Serpin B3
Proteasome subunit beta type-5
Isoform 3 of Dynactin subunit 1
Zinc-alpha-2-glycoprotein
GTP-binding protein SAR1b
Probable 2-oxoglutarate dehydrogenase E1 component DHKTD1, mitochondrial
Protein AHNAK2
Isoform IIC1 of Myc box-dependent-interacting protein 1
Coatomer protein complex, subunit epsilon, isoform CRA_g
Adipogenesis regulatory factor
V-type proton ATPase 16 kDa proteolipid subunit
Very-long-chain (3R)-3-hydroxyacyl-[acyl-carrier protein] dehydratase 3
Aminopeptidase B
N-acyl-aromatic-L-amino acid amidohydrolase (carboxylate-forming)
Kinectin
Ganglioside GM2 activator
Isoform 2 of Coagulation factor IX
26S protease regulatory subunit 10B
D-amino-acid oxidase
Septin-9
Methionine--tRNA ligase, cytoplasmic
Isoform 2 of Solute carrier family 22 member 8
AP-1 complex subunit mu-1
Basigin
Ras-related protein Ral-B
Kynurenine 3-monooxygenase
Phospholysine phosphohistidine inorganic pyrophosphate phosphatase
Isoform 2 of Vigilin
Putative transferase CAF17, mitochondrial
GTP cyclohydrolase 1 feedback regulatory protein
Dipeptidyl peptidase 1

3-hydroxyanthranilate 3,4-dioxygenase
Apolipoprotein D
Alpha-2-macroglobulin-like protein 1
Isoform 2 of Glutathione S-transferase omega-1
Cell division control protein 42 homolog
Isoform 2C of Cytoplasmic dynein 1 intermediate chain 2
Non-secretory ribonuclease
Bifunctional glutamate/proline--tRNA ligase
Pigment epithelium-derived factor
Leucine--tRNA ligase, cytoplasmic
NADH dehydrogenase [ubiquinone] 1 beta subcomplex subunit 7
Putative pre-mRNA-splicing factor ATP-dependent RNA helicase DHX15
Involucrin
D-2-hydroxyglutarate dehydrogenase, mitochondrial
Isoform 2 of Tumor protein D54
Angiotensin-converting enzyme
DNA-dependent protein kinase catalytic subunit
Collagen alpha-2(V) chain
Ig lambda-2 chain C regions
Isoform 2 of Ferrochelatase, mitochondrial
POTE ankyrin domain family member I
Coiled-coil domain-containing protein 170
Tubulin beta-6 chain
Collagen alpha-1(III) chain
Plasma serine protease inhibitor
Isoform 2 of RNA-binding protein 47
Isoform 3 of Retinal dehydrogenase 2
Isoform 2 of Lactoylglutathione lyase
Kinesin-1 heavy chain
Acyl-coenzyme A synthetase ACSM3, mitochondrial
Keratin, type II cytoskeletal 2 oral
ADP/ATP translocase 3
Isoform 2 of 26S protease regulatory subunit 8
NADH dehydrogenase [ubiquinone] iron-sulfur protein 6, mitochondrial
Ig kappa chain V-III region SIE
Heat shock 70 kDa protein 12A
Inorganic pyrophosphatase
Protein-glutamine gamma-glutamyltransferase E
Up-regulated during skeletal muscle growth protein 5
Mitochondrial fission 1 protein
Cystatin-C
Guanine nucleotide-binding protein G(I)/G(S)/G(O) subunit gamma-12
60S ribosomal protein L10
Protein S100-A11
Isoform 2 of Inositol monophosphatase 2
Isoform 3 of Ubiquitin-conjugating enzyme E2 L3
Arginine--tRNA ligase, cytoplasmic

Tubulin beta-4A chain
Echinoderm microtubule-associated protein-like 4
Sorting nexin-5
Isoform 2 of Sodium-dependent phosphate transport protein 4
Isoform Non-brain of Clathrin light chain B
von Willebrand factor A domain-containing protein 1
Peptidyl-prolyl cis-trans isomerase FKBP1A
60S ribosomal protein L27
Isoform 2 of Cullin-associated NEDD8-dissociated protein 1
Guanine nucleotide-binding protein subunit alpha-11
NADH dehydrogenase [ubiquinone] iron-sulfur protein 4, mitochondrial
HLA class I histocompatibility antigen, A-68 alpha chain
SPRY domain-containing protein 4
Heme-binding protein 1
NADH dehydrogenase [ubiquinone] iron-sulfur protein 7, mitochondrial
5'(3')-deoxyribonucleotidase, cytosolic type
Aldehyde dehydrogenase, dimeric NADP-preferring
Isoform 2 of Niban-like protein 1
Isoform 3 of Hydroxymethylglutaryl-CoA synthase, mitochondrial
Superoxide dismutase [Cu-Zn]
Isoform A of Protein CutA
Protein transport protein Sec24C
Mitochondrial-processing peptidase subunit alpha
Isoform 3 of Solute carrier family 13 member 3
Ectonucleoside triphosphate diphosphohydrolase 5
KH domain-containing, RNA-binding, signal transduction-associated protein 1
26S proteasome non-ATPase regulatory subunit 12
60S ribosomal protein L13a (Fragment)
Protein transport protein Sec61 subunit alpha isoform 1
Tryptophan--tRNA ligase, cytoplasmic
Histidine-rich glycoprotein
Isoform 2 of 14-3-3 protein sigma
Guanylate-binding protein 6
Acyl-CoA synthetase family member 3, mitochondrial
Serine dehydratase-like
Isoform 3 of Serine protease HTRA2, mitochondrial
Isoform 2 of Gephyrin
Long-chain specific acyl-CoA dehydrogenase, mitochondrial
Calcium-binding mitochondrial carrier protein Aralar1
Cingulin-like protein 1
Isoform 2 of Golgin subfamily B member 1
AP-1 complex subunit sigma-1A (Fragment)
Carboxypeptidase Q
Isoform 4 of AP-3 complex subunit delta-1
Isoform 2 of Splicing factor U2AF 65 kDa subunit
DnaJ homolog subfamily A member 2
Isoform 2 of Carbonic anhydrase 12

Isoform 2 of Glucose-6-phosphate translocase
Histone deacetylase 6
60S ribosomal protein L15
NADH dehydrogenase [ubiquinone] 1 beta subcomplex subunit 6
Membrane primary amine oxidase
Isoform 2 of Ras-related protein Rab-6A
Proteasome subunit beta type-6
Sodium/potassium-transporting ATPase subunit gamma
Microtubule-associated protein RP/EB family member 1
Isoform 2 of NADH dehydrogenase [ubiquinone] 1 beta subcomplex subunit 11, mitochondrial
Receptor expression-enhancing protein 6
Beta-lactamase-like protein 2
60S ribosomal protein L14
GrpE protein homolog 1, mitochondrial
Isoform 2 of Protein-L-isoaspartate(D-aspartate) O-methyltransferase
Glycine--tRNA ligase
Prolargin
Sulfatase-modifying factor 2
Galectin-1
NADH-ubiquinone oxidoreductase chain 4
Isoform 2 of 5-formyltetrahydrofolate cyclo-ligase
Mitochondrial-processing peptidase subunit beta
Galectin-3
Cadherin-related family member 2
Leucine-rich repeat-containing protein 47
Calcineurin B homologous protein 1
Antithrombin-III
GTP-binding protein SAR1a
Isoform 1A of Sorting nexin-1
Isoform B of AP-1 complex subunit beta-1
Isoform 2 of Acyl-protein thioesterase 1
Hornerin
Plakophilin-1
Isoform 3 of Clathrin interactor 1
UPF0598 protein C8orf82 (Fragment)
Isoform 2 of Vesicle-associated membrane protein-associated protein A
Thy-1 membrane glycoprotein
HCLS1-binding protein 3
Lupus La protein
AP-2 complex subunit sigma
Isoform 2 of Programmed cell death protein 6
Protein phosphatase 1 regulatory subunit 7
Cadherin-2
Platelet-activating factor acetylhydrolase IB subunit beta
Eukaryotic translation initiation factor 4B
Isoform Epsilon of Apoptosis regulator BAX
Splicing factor 3B subunit 1

Leukocyte elastase inhibitor
ATPase family AAA domain-containing protein 2
Fibrous sheath-interacting protein 2
Isoform 2 of Collagen alpha-2(XI) chain
Isoform 2 of Sciellin
NADH dehydrogenase [ubiquinone] iron-sulfur protein 8, mitochondrial (Fragment)
Sushi domain-containing protein 2
Ras GTPase-activating protein-binding protein 1
Guanidinoacetate N-methyltransferase
Cytochrome c oxidase subunit 6B1
28S ribosomal protein S31, mitochondrial
Transmembrane protein 14C
OCIA domain-containing protein 1
NADH dehydrogenase [ubiquinone] 1 alpha subcomplex subunit 3
Isoform 2 of Solute carrier family 22 member 6
Complement C5
Isoform 2 of Voltage-dependent anion-selective channel protein 3
Complement factor H
ATPase ASNA1
Putative ATP-dependent Clp protease proteolytic subunit, mitochondrial
Copine-1
Isoform 2 of PDZ domain-containing protein 11
Ubiquitin-fold modifier 1
Phenylalanine--tRNA ligase alpha subunit
Proliferation-associated protein 2G4
Alpha-1-acid glycoprotein 1
Isoform 3 of Aminomethyltransferase, mitochondrial
Keratin, type I cytoskeletal 15
Isoform 2 of Mitogen-activated protein kinase 1
Proton-coupled amino acid transporter 2
L-2-hydroxyglutarate dehydrogenase, mitochondrial
Isoform 2 of Heterogeneous nuclear ribonucleoprotein D-like
NADH dehydrogenase [ubiquinone] 1 alpha subcomplex subunit 7
Sulfate anion transporter 1
Isoform 2 of Succinate--hydroxymethylglutarate CoA-transferase
Isoform 2 of Dynamin-like 120 kDa protein, mitochondrial
Ig gamma-4 chain C region
Methyltransferase-like protein 7B
Saccharopine dehydrogenase-like oxidoreductase
Isoform 2 of Eukaryotic initiation factor 4A-II
von Willebrand factor A domain-containing protein 8
Beta-hexosaminidase subunit beta
Putative hexokinase HKDC1
Isoform 2B of Desmocollin-2
Apolipoprotein B-100
Receptor expression-enhancing protein 5
NADH-ubiquinone oxidoreductase chain 5

Thymosin alpha-1 (Fragment)
Isoform B of AP-2 complex subunit alpha-1
Angiotensinogen
Isoform SM-B of Small nuclear ribonucleoprotein-associated proteins B and B'
U1 small nuclear ribonucleoprotein A
Splicing factor 3B subunit 2
Isoform 2 of Spectrin beta chain, erythrocytic
Suprabasin
Calmodulin-like protein 5
Serpin B5
Protein phosphatase 1F
Proteasome subunit beta type-2
Collectrin
Isoform 2 of Mitochondrial carrier homolog 1
ADP-ribosylation factor-like protein 8B
Paralemmin-3
Probable serine carboxypeptidase CPVL
Large neutral amino acids transporter small subunit 2
Isoform 2 of Protein TFG
Isoform 5 of Acyl-CoA dehydrogenase family member 10
Xylulose kinase
3'(2'),5'-bisphosphate nucleotidase 1
Cyclic AMP-responsive element-binding protein 3-like protein 1
Small proline-rich protein 3
Isoform LAMP-2B of Lysosome-associated membrane glycoprotein 2
ADP-ribosylation factor 5
Splicing factor 3B subunit 3
Vesicle-fusing ATPase
Rab11 family-interacting protein 5
Angiotensin-converting enzyme 2
Syndecan-1
Vitamin D-binding protein
Isoform 2 of Nuclear protein localization protein 4 homolog
Isoform 2 of Leucine-rich repeat flightless-interacting protein 1
Sedoheptulokinase
Cleft lip and palate transmembrane protein 1
Isoform 3 of Perilipin-3
Alpha-soluble NSF attachment protein
Selenoprotein O
Collagen alpha-5(VI) chain
6-phosphofructokinase type C
Nucleoprotein TPR
Succinate dehydrogenase cytochrome b560 subunit, mitochondrial
Nicotinamide phosphoribosyltransferase
Density-regulated protein
Serine--tRNA ligase, mitochondrial
Isoform 2 of Serine/threonine-protein phosphatase 2A 55 kDa regulatory subunit B alpha isoform

GH3 domain-containing protein
NADH dehydrogenase [ubiquinone] iron-sulfur protein 5
UDP-glucose 4-epimerase
Chromosome 2 open reading frame 18, isoform CRA_c
D-beta-hydroxybutyrate dehydrogenase, mitochondrial
Copine-3
60S ribosomal protein L28
Isoform 2 of Nucleosome assembly protein 1-like 4
Small nuclear ribonucleoprotein Sm D2
Ceruloplasmin
Complement factor B
Adenylosuccinate synthetase isozyme 1
Alanine aminotransferase 1
Beta-ureidopropionase
Tight junction protein 1 (Zona occludens 1), isoform CRA_a
Peroxisomal NADH pyrophosphatase NUDT12
Bis(5'-adenosyl)-triphosphatase
Isoform 2 of Sorting nexin-12
Protein phosphatase 1 regulatory subunit 12A
Solute carrier family 22 member 2
N-acetylneuraminate lyase
Paraspeckle component 1
60S ribosomal protein L21
Probable ATP-dependent RNA helicase DDX6
Isoform B of Osteopontin
Isoform 2 of Sodium-dependent phosphate transport protein 2B
Serum amyloid A-1 protein
Eukaryotic translation initiation factor 3 subunit F
ATP synthase subunit epsilon, mitochondrial
HEAT repeat-containing protein 5B
Pentraxin-related protein PTX3
Keratin, type II cuticular Hb5
Isoform Beta of Tripartite motif-containing protein 29
Desmoglein-3
Isochorismatase domain-containing protein 1
Isoform 3 of Ubiquitin-conjugating enzyme E2 K
Ubiquitin carboxyl-terminal hydrolase isozyme L1
Sorting and assembly machinery component 50 homolog
Isoform 6 of Poly(U)-binding-splicing factor PUF60
Isoform 2 of Dapper homolog 1
Ras-related protein Rab-21
Heat shock protein beta-6
Isoform 2 of Creatine kinase U-type, mitochondrial
ATPase inhibitor, mitochondrial
Aspartyl aminopeptidase
Isoform 2 of Harmonin
Cadherin-1

Isoform Short of Delta-1-pyrroline-5-carboxylate synthase
Isoform 3 of Cadherin-related family member 5
Thioredoxin-like protein 1 (Fragment)
Isoform 5 of Calpastatin
UV excision repair protein RAD23 homolog B
O-acetyl-ADP-ribose deacetylase MACROD1
Isoform 3 of Solute carrier family 22 member 12
Ig kappa chain V-III region VG (Fragment)
Isoform CYP4F3B of Leukotriene-B(4) omega-hydroxylase 2
Isoform 1 of Serine/threonine-protein phosphatase 2A activator
RNA-binding protein 14
Serine--tRNA ligase, cytoplasmic
26S proteasome non-ATPase regulatory subunit 7
Serine/threonine-protein phosphatase PP1-alpha catalytic subunit
Malonyl-CoA decarboxylase, mitochondrial
Complement C1q subcomponent subunit B
PRKC apoptosis WT1 regulator protein
60S ribosomal protein L35a
Vasodilator-stimulated phosphoprotein
Isoform 2 of Surfeit locus protein 4
Eukaryotic translation initiation factor 3 subunit H
Regucalcin
Isoform 2 of Fermitin family homolog 3
Isoform 2 of Myosin-14
Hydrocephalus-inducing protein homolog
Serpin B4
Immunoglobulin J chain
26S proteasome non-ATPase regulatory subunit 1
Isoform 7 of Tight junction protein ZO-2
Isoform 2 of Mitochondrial enolase superfamily member 1
Lipopolysaccharide-binding protein
Hemopexin
Isoform 2 of Spectrin beta chain, non-erythrocytic 1
Aldo-keto reductase family 1 member C1
5-oxoprolinase
Isoform 2 of E3 ubiquitin-protein ligase RNF213
Caspase-14
Filaggrin
Isoform 5 of NADPH:adrenodoxin oxidoreductase, mitochondrial
Galectin-7
Isoform 2 of Chromatin target of PRMT1 protein
Alpha-2-macroglobulin receptor-associated protein
G-protein-coupled receptor family C group 5 member C (Fragment)
Cytoplasmic dynein 1 light intermediate chain 2
Tyrosine-protein phosphatase non-receptor type 1
39S ribosomal protein L28, mitochondrial
Dolichyl-diphosphooligosaccharide--protein glycosyltransferase subunit STT3A

Cordon-bleu protein-like 1
Steroid 17-alpha-hydroxylase/17,20 lyase
Vinexin
Eosinophil peroxidase
Glycerol-3-phosphate dehydrogenase, mitochondrial
Protein-glutamine gamma-glutamyltransferase K
Ig lambda chain V-III region LOI
Mannosyl-oligosaccharide glucosidase
Serine/threonine-protein kinase R1
Twinfilin-1
Galactose-1-phosphate uridylyltransferase
Isoform 2 of Secernin-1
Protein THEM6
NADH dehydrogenase [ubiquinone] 1 alpha subcomplex assembly factor 3
Ubiquitin carboxyl-terminal hydrolase isozyme L3
Phosphatidylinositide phosphatase SAC1
Prostaglandin E synthase 2
Alpha-N-acetylglucosaminidase
Isoform 4 of Double-stranded RNA-specific adenosine deaminase
Neutral cholesterol ester hydrolase 1
Myristoylated alanine-rich C-kinase substrate
Prolow-density lipoprotein receptor-related protein 1
Serine/threonine-protein kinase Nek9
Keratin, type I cuticular Ha1
Interleukin-1 receptor antagonist protein
Isoform 3B of Desmocollin-3
Mitochondrial import receptor subunit TOM22 homolog
6-phosphofructo-2-kinase/fructose-2,6-bisphosphatase 2
Isoform Short of Beta-glucuronidase
Dual specificity protein phosphatase 23
Apolipoprotein O-like
Inosine-5'-monophosphate dehydrogenase 2
Isoform 3 of Iodotyrosine dehalogenase 1
Serum amyloid P-component
Cleavage and polyadenylation specificity factor subunit 5
ATP-dependent Clp protease ATP-binding subunit clpX-like, mitochondrial
DnaJ homolog subfamily B member 1
Isoform 2 of Interferon-induced GTP-binding protein Mx1
Isoform 3 of Zinc finger CCCH-type antiviral protein 1
Eukaryotic translation initiation factor 3 subunit D
Keratin, type II cytoskeletal 6A
Putative uncharacterized protein
Beta-hexosaminidase subunit alpha
Isoform PKP3b of Plakophilin-3
Isoform 1 of Protein POF1B
Ig kappa chain V-I region AG
NADH-ubiquinone oxidoreductase chain 2

Hematological and neurological-expressed 1-like protein (Fragment)
Isoform 3 of Mitochondrial fission factor
V-type proton ATPase subunit C 1
Isoform 2 of Scaffold attachment factor B1
Growth hormone-inducible transmembrane protein
Isoform 2 of 2',5'-phosphodiesterase 12
Isoform 3 of Glucose-6-phosphate 1-dehydrogenase
Copine-6
Fatty acid-binding protein, epidermal
Signal recognition particle subunit SRP72
F-box only protein 50
Voltage-dependent calcium channel gamma-8 subunit
Isoform 7 of Zinc finger protein 185
Alpha-amylase 1
Repetin
Atlastin-3 (Fragment)
Complement component C8 beta chain
Carbonyl reductase [NADPH] 3
Isoform 2 of Keratin, type I cuticular Ha6
Transferrin receptor protein 1
Isoform 2 of Serpin B13
Isoform 2 of Alcohol dehydrogenase class 4 mu/sigma chain
Extracellular superoxide dismutase [Cu-Zn]
Isoform 12 of CD44 antigen
Secretory carrier-associated membrane protein 1
HLA class I histocompatibility antigen, A-69 alpha chain
Alpha-1-acid glycoprotein 2
Isoform 6 of Protein inscuteable homolog
Integrin beta
NADP-dependent malic enzyme, mitochondrial
Isoform 2 of Inter-alpha-trypsin inhibitor heavy chain H4
Isoform 2 of Protein arginine N-methyltransferase 1
Ran GTPase-activating protein 1
Sulfotransferase 1A1
Annexin A8
SERPINB12 protein
Kallikrein-10
Leucine-rich alpha-2-glycoprotein
Carbonic anhydrase 4
Isoform 2 of NAD(P)H dehydrogenase [quinone] 1
Pyridoxal-dependent decarboxylase domain-containing protein 1
HLA class I histocompatibility antigen, B-40 alpha chain
Isoform 2 of Carbonic anhydrase 6
Isoform 2 of Kallikrein-7
Cystatin-S
Isoform 2 of Kallikrein-12
Carbonic anhydrase 3

Vacuolar protein sorting-associated protein 4B
Aldo-keto reductase family 1 member B10
Prolactin-inducible protein
Elafin
Isoform 2 of V-set and immunoglobulin domain-containing protein 10-like
Mucin-13
Kallikrein-14
Dynein light chain 1, cytoplasmic
AT-rich interactive domain-containing protein 3C
Nucleoside diphosphate-linked moiety X motif 19, mitochondrial

Gene Name	Accession Number	Gene Locus	Molecular \	Protein Grc	Total Spect SAGN T1	Total Spect SAGN T2
MYH9	sp|P35579	MYH9_HUMAN	227 kDa	TRUE	98	90
VIM	sp|P08670	VIME_HUMAN	54 kDa	TRUE	73	74
COL6A3	sp|P12111	CO6A3_HUMAN	344 kDa	TRUE	53	55
FLNA	sp|P21333-2	FLNA_HUMAN	280 kDa	TRUE	51	40
KRT8	sp|P05787-2	K2C8_HUMAN	57 kDa	TRUE	47	49
KRT9	sp|P35527	K1C9_HUMAN	62 kDa	TRUE	45	22
ACTG1	sp|P63261	ACTG_HUMAN	42 kDa	TRUE	42	55
KRT1	sp|P04264	K2C1_HUMAN	66 kDa	TRUE	42	15
SPTAN1	sp|Q13813-3	SPTN1_HUMAN	282 kDa		37	62
KRT2	sp|P35908	K22E_HUMAN	65 kDa	TRUE	37	11
HSPG2	sp|P98160	PGBM_HUMAN	469 kDa		36	56
ACTC1	sp|P68032	ACTC_HUMAN	42 kDa	TRUE	36	54
LMNA	sp|P02545	LMNA_HUMAN	74 kDa	TRUE	35	33
ENO1	sp|P06733	ENOA_HUMAN	47 kDa	TRUE	34	34
MYH10	sp|P35580-2	MYH10_HUMAN	231 kDa	TRUE	34	45
ATP5B	sp|P06576	ATPB_HUMAN	57 kDa		33	47
MSN	sp|P26038	MOES_HUMAN	68 kDa	TRUE	33	32
KRT10	sp|P13645	K1C10_HUMAN	59 kDa	TRUE	33	23
TUBB	sp|P07437	TBB5_HUMAN	50 kDa	TRUE	31	34
KRT18	sp|P05783	K1C18_HUMAN	48 kDa	TRUE	31	34
TUBB4B	sp|P68371	TBB4B_HUMAN	50 kDa	TRUE	31	37
ATP5A1	sp|P25705	ATPA_HUMAN	60 kDa		30	41
TPM3	sp|P06753-2	TPM3_HUMAN	29 kDa	TRUE	29	27
SPTBN1	sp|Q01082	SPTB2_HUMAN	275 kDa	TRUE	28	48
LRP2	sp|P98164	LRP2_HUMAN	522 kDa		27	52
TPM1	sp|P09493-3	TPM1_HUMAN	33 kDa	TRUE	27	21
PKM	sp|P14618	KPYM_HUMAN	58 kDa	TRUE	26	28
FLNB	sp|O75369-2	FLNB_HUMAN	276 kDa	TRUE	26	36
ACTN4	sp|O43707	ACTN4_HUMAN	105 kDa	TRUE	25	31
MVP	sp|Q14764	MVP_HUMAN	99 kDa		25	35
PLEC	sp|Q15149-3	PLEC_HUMAN	518 kDa	TRUE	24	12
TPM4	sp|P67936	TPM4_HUMAN	29 kDa	TRUE	24	20
KRT14	sp|P02533	K1C14_HUMAN	52 kDa	TRUE	24	6
TUBB2A	sp|Q13885	TBB2A_HUMAN	50 kDa	TRUE	24	26
GAPDH	sp|P04406	G3P_HUMAN	36 kDa	TRUE	23	38
CLTC	sp|Q00610-2	CLH1_HUMAN	188 kDa		23	27
HSPA5	sp|P11021	GRP78_HUMAN	72 kDa	TRUE	23	28
CNDP2	sp|Q96KP4	CNDP2_HUMAN	53 kDa		23	22
TPM1	tr|H7BYY1	H7BYY1_HUMAN	29 kDa	TRUE	23	20
ACO2	sp|Q99798	ACON_HUMAN	85 kDa		22	33
TLN1	sp|Q9Y490	TLN1_HUMAN	270 kDa	TRUE	22	15
ATP1A1	sp|P05023-4	AT1A1_HUMAN	113 kDa	TRUE	21	23
VCL	sp|P18206-2	VINC_HUMAN	117 kDa		21	19

FN1	sp|P02751-17	FINC_HUMAN	256 kDa		21	14
KRT5	sp|P13647	K2C5_HUMAN	62 kDa	TRUE	21	5
TUBA1B	sp|P68363	TBA1B_HUMAN	50 kDa	TRUE	21	22
TPM2	sp|P07951-2	TPM2_HUMAN	33 kDa	TRUE	21	15
GATM	sp|P50440	GATM_HUMAN	48 kDa		20	51
HSPD1	sp|P10809	CH60_HUMAN	61 kDa		20	31
ANXA2	sp|P07355-2	ANXA2_HUMAN	40 kDa		20	15
HSP90AB1	sp|P08238	HS90B_HUMAN	83 kDa	TRUE	19	17
ACTN1	sp|P12814-2	ACTN1_HUMAN	103 kDa	TRUE	19	15
ANXA4	sp|P09525	ANXA4_HUMAN	36 kDa	TRUE	18	18
HSPA8	sp|P11142	HSP7C_HUMAN	71 kDa	TRUE	17	19
TUBA1C	tr|F5H5D3	F5H5D3_HUMAN	58 kDa	TRUE	17	19
KRT6C	sp|P48668	K2C6C_HUMAN	60 kDa	TRUE	17	5
KRT6B	sp|P04259	K2C6B_HUMAN	60 kDa	TRUE	17	0
LDHB	sp|P07195	LDHB_HUMAN	37 kDa	TRUE	16	25
HSPA9	sp|P38646	GRP75_HUMAN	74 kDa	TRUE	16	18
AIFM1	sp|O95831	AIFM1_HUMAN	67 kDa		16	19
HIST1H4A	sp|P62805	H4_HUMAN	11 kDa		16	19
PDIA3	sp|P30101	PDIA3_HUMAN	57 kDa	TRUE	16	26
HNRNPA2B1	sp|P22626	ROA2_HUMAN	37 kDa	TRUE	16	19
EZR	sp|P15311	EZRI_HUMAN	69 kDa	TRUE	16	24
ALDH4A1	sp|P30038	AL4A1_HUMAN	62 kDa		15	11
HADHA	sp|P40939	ECHA_HUMAN	83 kDa		15	34
CRYAB	sp|P02511	CRYAB_HUMAN	20 kDa		15	11
HSP90B1	sp|P14625	ENPL_HUMAN	92 kDa	TRUE	15	16
HBA1	sp|P69905	HBA_HUMAN	15 kDa		15	10
HIST2H2AA3	sp|Q6FI13	H2A2A_HUMAN	14 kDa	TRUE	15	13
DYNC1H1	sp|Q14204	DYHC1_HUMAN	532 kDa		15	10
TNC	sp|P24821-4	TENA_HUMAN	231 kDa		15	12
KRT19	sp|P08727	K1C19_HUMAN	44 kDa	TRUE	15	19
HIST1H2AG	sp|P0C0S8	H2A1_HUMAN	14 kDa	TRUE	15	13
GLUD1	sp|P00367	DHE3_HUMAN	61 kDa		14	22
ACAT1	sp|P24752	THIL_HUMAN	45 kDa		14	24
HSP90AA1	sp|P07900-2	HS90A_HUMAN	98 kDa	TRUE	14	18
HSPB1	sp|P04792	HSPB1_HUMAN	23 kDa		14	16
CALD1	sp|Q05682-5	CALD1_HUMAN	61 kDa		14	11
HNRNPA1	tr|F8W6I7	F8W6I7_HUMAN	33 kDa	TRUE	14	15
COL14A1	sp|Q05707-2	COEA1_HUMAN	192 kDa		14	7
KRT16	sp|P08779	K1C16_HUMAN	51 kDa	TRUE	14	0
HIST1H2AC	sp|Q93077	H2A1C_HUMAN	14 kDa	TRUE	14	12
ACAA2	sp|P42765	THIM_HUMAN	42 kDa	TRUE	13	44
PCK2	sp|Q16822	PCKGM_HUMAN	71 kDa	TRUE	13	30
ALDOB	sp|P05062	ALDOB_HUMAN	39 kDa	TRUE	13	24
ALDH6A1	sp|Q02252	MMSA_HUMAN	58 kDa		13	23
ALDH2	sp|P05091	ALDH2_HUMAN	56 kDa	TRUE	13	15
TUFM	sp|P49411	EFTU_HUMAN	50 kDa		13	12
ACADVL	sp|P49748	ACADV_HUMAN	70 kDa		13	20

YWHAE	sp|P62258	1433E_HUMAN	29 kDa	TRUE	13	14
HBB	sp|P68871	HBB_HUMAN	16 kDa	TRUE	13	12
LAMA5	sp|O15230	LAMA5_HUMAN	400 kDa		13	16
HNRNPK	sp|P61978	HNRPK_HUMAN	51 kDa		13	16
RPS27A	sp|P62979	RS27A_HUMAN	18 kDa		13	11
TUBA4A	sp|P68366	TBA4A_HUMAN	50 kDa	TRUE	13	15
ACTBL2	sp|Q562R1	ACTBL_HUMAN	42 kDa	TRUE	13	15
ANPEP	sp|P15144	AMPN_HUMAN	110 kDa		12	23
UBA1	sp|P22314	UBA1_HUMAN	118 kDa		12	16
ANXA6	sp|P08133	ANXA6_HUMAN	76 kDa		12	23
ECHS1	sp|P30084	ECHM_HUMAN	31 kDa		12	19
VCP	sp|P55072	TERA_HUMAN	89 kDa		12	15
ALDH1A1	sp|P00352	AL1A1_HUMAN	55 kDa	TRUE	12	15
PPIA	sp|P62937	PPIA_HUMAN	18 kDa	TRUE	12	14
PHB	sp|P35232	PHB_HUMAN	30 kDa		12	11
JUP	tr|F5GWP8	F5GWP8_HUMAN	66 kDa	TRUE	12	5
ACSM2B	sp|Q68CK6	ACS2B_HUMAN	64 kDa	TRUE	11	20
PGK1	sp|P00558	PGK1_HUMAN	45 kDa	TRUE	11	19
CUBN	sp|O60494	CUBN_HUMAN	399 kDa		11	19
IDH2	sp|P48735	IDHP_HUMAN	51 kDa	TRUE	11	16
AHNAK	sp|Q09666	AHNK_HUMAN	629 kDa		11	6
PRDX1	sp|Q06830	PRDX1_HUMAN	22 kDa	TRUE	11	18
MDH2	sp|P40926	MDHM_HUMAN	36 kDa		11	13
TKT	sp|P29401-2	TKT_HUMAN	69 kDa		11	16
CALM2	tr|E7EMB3	E7EMB3_HUMAN	22 kDa	TRUE	11	15
COL4A2	sp|P08572	CO4A2_HUMAN	168 kDa		11	12
MYL6	sp|P60660-2	MYL6_HUMAN	17 kDa		11	6
HNRNPM	sp|P52272	HNRPM_HUMAN	78 kDa	TRUE	11	6
PFN1	sp|P07737	PROF1_HUMAN	15 kDa		11	8
YWHAZ	sp|P63104	1433Z_HUMAN	28 kDa	TRUE	11	13
EMILIN1	sp|Q9Y6C2	EMIL1_HUMAN	107 kDa		11	8
TGM2	sp|P21980	TGM2_HUMAN	77 kDa		11	6
UQCRC2	sp|P22695	QCR2_HUMAN	48 kDa		10	11
PCCA	sp|P05165-2	PCCA_HUMAN	77 kDa		10	12
HSPA1A	sp|P08107	HSP71_HUMAN	70 kDa	TRUE	10	12
ALDH1B1	sp|P30837	AL1B1_HUMAN	57 kDa	TRUE	10	12
CBR1	sp|P16152	CBR1_HUMAN	30 kDa	TRUE	10	14
COL6A1	sp|P12109	CO6A1_HUMAN	109 kDa		10	16
CCT2	sp|P78371	TCPB_HUMAN	57 kDa		10	15
LAP3	sp|P28838-2	AMPL_HUMAN	53 kDa		10	11
DPYSL2	sp|Q16555	DPYL2_HUMAN	62 kDa	TRUE	10	14
IGHG1	sp|P01857	IGHG1_HUMAN	36 kDa	TRUE	10	21
XRCC6	sp|P12956	XRCC6_HUMAN	70 kDa		10	6
HSD17B4	sp|P51659	DHB4_HUMAN	80 kDa		10	5
TAGLN	sp|Q01995	TAGL_HUMAN	23 kDa		10	9
KRT7	sp|P08729	K2C7_HUMAN	51 kDa	TRUE	10	13
ACSM2A	sp|Q08AH3	ACS2A_HUMAN	64 kDa	TRUE	10	20

HNRNPA3	sp|P51991	ROA3_HUMAN	40 kDa	TRUE	10	4
KRT13	sp|P13646	K1C13_HUMAN	50 kDa	TRUE	9	3
PEBP1	sp|P30086	PEBP1_HUMAN	21 kDa		9	16
HADHB	sp|P55084	ECHB_HUMAN	51 kDa		9	15
ATP6V1B2	sp|P21281	VATB2_HUMAN	57 kDa	TRUE	9	12
COL18A1	sp|P39060	COIA1_HUMAN	178 kDa		9	17
ANXA5	sp|P08758	ANXA5_HUMAN	36 kDa		9	16
PRDX2	sp|P32119	PRDX2_HUMAN	22 kDa	TRUE	9	12
CRYZ	sp|Q08257	QOR_HUMAN	35 kDa		9	12
GSN	sp|P06396-2	GELS_HUMAN	81 kDa		9	13
LAMC1	sp|P11047	LAMC1_HUMAN	178 kDa		9	15
HNRNPU	sp|Q00839	HNRPU_HUMAN	91 kDa		9	7
YWHAB	sp|P31946-2	1433B_HUMAN	28 kDa	TRUE	9	12
MT-CO2	sp|P00403	COX2_HUMAN	26 kDa		9	8
H3F3A	sp|P84243	H33_HUMAN	15 kDa	TRUE	9	8
MYL12B	sp|O14950	ML12B_HUMAN	20 kDa	TRUE	9	5
RDX	sp|P35241	RADI_HUMAN	69 kDa	TRUE	9	14
HNRNPH2	sp|P55795	HNRH2_HUMAN	49 kDa	TRUE	9	7
HIST2H2BF	sp|Q5QNW6-2	H2B2F_HUMAN	15 kDa	TRUE	9	0
ASS1	sp|P00966	ASSY_HUMAN	47 kDa		8	34
PC	sp|P11498	PYC_HUMAN	130 kDa	TRUE	8	17
CAT	sp|P04040	CATA_HUMAN	60 kDa		8	20
OGDH	sp|Q02218	ODO1_HUMAN	116 kDa	TRUE	8	17
ACO1	sp|P21399	ACOC_HUMAN	98 kDa		8	9
SUCLG2	sp|Q96I99	SUCB2_HUMAN	47 kDa		8	12
PCCB	tr|C9JQS9	C9JQS9_HUMAN	61 kDa		8	9
FH	sp|P07954	FUMH_HUMAN	55 kDa		8	6
GDI2	tr|E7EU23	E7EU23_HUMAN	51 kDa	TRUE	8	9
GSTP1	sp|P09211	GSTP1_HUMAN	23 kDa		8	16
NNT	sp|Q13423	NNTM_HUMAN	114 kDa		8	7
EEF1A1	sp|P68104	EF1A1_HUMAN	50 kDa		8	11
AK2	sp|P54819-2	KAD2_HUMAN	26 kDa		8	12
PDHB	sp|P11177-2	ODPB_HUMAN	37 kDa		8	5
HIST1H2BO	sp|P23527	H2B1O_HUMAN	14 kDa	TRUE	8	13
IMMT	sp|Q16891-2	IMMT_HUMAN	83 kDa		8	12
CTSD	sp|P07339	CATD_HUMAN	45 kDa		8	14
CDH16	sp|O75309	CAD16_HUMAN	90 kDa		8	5
NCL	sp|P19338	NUCL_HUMAN	77 kDa		8	9
SOD2	sp|P04179-4	SODM_HUMAN	20 kDa		8	6
ANXA1	sp|P04083	ANXA1_HUMAN	39 kDa		8	7
IGKC	sp|P01834	IGKC_HUMAN	12 kDa		8	9
COL6A2	sp|P12110	CO6A2_HUMAN	109 kDa		8	13
HNRNPF	sp|P52597	HNRPF_HUMAN	46 kDa	TRUE	8	6
RRBP1	sp|Q9P2E9-2	RRBP1_HUMAN	152 kDa	TRUE	8	7
LASP1	sp|Q14847	LASP1_HUMAN	30 kDa		8	6
COL12A1	tr|D6RGG3	D6RGG3_HUMAN	333 kDa		8	12
NID2	sp|Q14112-2	NID2_HUMAN	141 kDa	TRUE	8	4

HNRNPH1	tr|G8JLB6	G8JLB6_HUMAN	51 kDa	TRUE	8	7
ALB	sp|P02768	ALBU_HUMAN	69 kDa		7	17
AKR1A1	sp|P14550	AK1A1_HUMAN	37 kDa		7	12
NDUFS1	sp|P28331-2	NDUS1_HUMAN	81 kDa		7	6
HIBCH	sp|Q6NVY1	HIBCH_HUMAN	43 kDa		7	5
HSD17B10	sp|Q99714	HCD2_HUMAN	27 kDa		7	9
PRDX6	sp|P30041	PRDX6_HUMAN	25 kDa		7	10
SLC25A5	sp|P05141	ADT2_HUMAN	33 kDa	TRUE	7	7
PDIA6	tr|F8WA83	F8WA83_HUMAN	49 kDa		7	13
ETFB	sp|P38117-2	ETFB_HUMAN	38 kDa		7	9
EEF2	sp|P13639	EF2_HUMAN	95 kDa	TRUE	7	5
ALDOA	sp|P04075-2	ALDOA_HUMAN	45 kDa	TRUE	7	12
TAGLN2	sp|P37802	TAGL2_HUMAN	22 kDa		7	10
DLST	sp|P36957	ODO2_HUMAN	49 kDa		7	6
PARK7	sp|Q99497	PARK7_HUMAN	20 kDa		7	9
ATP5O	sp|P48047	ATPO_HUMAN	23 kDa		7	6
TCP1	sp|P17987	TCPA_HUMAN	60 kDa		7	12
IQGAP1	sp|P46940	IQGA1_HUMAN	189 kDa	TRUE	7	8
SFPQ	sp|P23246	SFPQ_HUMAN	76 kDa	TRUE	7	7
MYO6	tr|E7EW20	E7EW20_HUMAN	149 kDa		7	7
RPN1	sp|P04843	RPN1_HUMAN	69 kDa		7	8
LMNB2	sp|Q03252	LMNB2_HUMAN	68 kDa	TRUE	7	6
NID1	sp|P14543-2	NID1_HUMAN	122 kDa	TRUE	7	9
BGN	sp|P21810	PGS1_HUMAN	42 kDa		7	5
HNRNPC	tr|B4DY08	B4DY08_HUMAN	32 kDa		7	4
THBS1	sp|P07996	TSP1_HUMAN	129 kDa		7	6
HNRNPD	sp|Q14103-3	HNRPD_HUMAN	33 kDa	TRUE	7	6
UMOD	tr|E9PEA4	E9PEA4_HUMAN	74 kDa		7	6
HBD	sp|P02042	HBD_HUMAN	16 kDa	TRUE	7	0
YWHAH	sp|Q04917	1433F_HUMAN	28 kDa	TRUE	7	6
ACY1	sp|Q03154	ACY1_HUMAN	46 kDa		6	26
EHHADH	sp|Q08426	ECHP_HUMAN	79 kDa		6	18
BHMT	sp|Q93088	BHMT1_HUMAN	45 kDa	TRUE	6	17
ACADM	sp|P11310-2	ACADM_HUMAN	47 kDa		6	15
ECH1	sp|Q13011	ECH1_HUMAN	36 kDa		6	17
TPI1	sp|P60174-1	TPIS_HUMAN	27 kDa		6	13
SHMT1	sp|P34896-2	GLYC_HUMAN	49 kDa	TRUE	6	9
MCCC2	sp|Q9HCC0	MCCB_HUMAN	61 kDa		6	13
ETFA	sp|P13804-2	ETFA_HUMAN	30 kDa		6	16
SLC9A3R1	sp|O14745	NHRF1_HUMAN	39 kDa		6	9
FBP1	sp|P09467	F16P1_HUMAN	37 kDa	TRUE	6	10
GANAB	sp|Q14697	GANAB_HUMAN	107 kDa		6	9
UQCRC1	sp|P31930	QCR1_HUMAN	53 kDa	TRUE	6	11
P4HB	sp|P07237	PDIA1_HUMAN	57 kDa		6	12
CA2	sp|P00918	CAH2_HUMAN	29 kDa		6	8
GPX3	sp|P22352	GPX3_HUMAN	26 kDa		6	12
NPM1	sp|P06748	NPM_HUMAN	33 kDa		6	10

DLD	sp|P09622	DLDH_HUMAN	54 kDa		6	5
BBOX1	sp|O75936	BODG_HUMAN	45 kDa		6	6
BDH2	sp|Q9BUT1	BDH2_HUMAN	27 kDa		6	13
LDHA	sp|P00338-3	LDHA_HUMAN	40 kDa	TRUE	6	3
C11orf54	sp|Q9H0W9	CK054_HUMAN	35 kDa		6	7
RPS3	sp|P23396	RS3_HUMAN	27 kDa		6	6
COX4I1	sp|P13073	COX41_HUMAN	20 kDa		6	4
EIF4A1	sp|P60842	IF4A1_HUMAN	46 kDa	TRUE	6	4
RBP4	sp|P02753	RET4_HUMAN	23 kDa		6	5
RAB7A	sp|P51149	RAB7A_HUMAN	23 kDa		6	4
COL1A2	sp|P08123	CO1A2_HUMAN	129 kDa		6	4
TGFBI	sp|Q15582	BGH3_HUMAN	75 kDa		6	8
LYZ	sp|P61626	LYSC_HUMAN	17 kDa		6	5
CNN3	sp|Q15417	CNN3_HUMAN	36 kDa	TRUE	6	3
TNS1	tr|E9PGF5	E9PGF5_HUMAN	183 kDa	TRUE	6	2
YWHAG	sp|P61981	1433G_HUMAN	28 kDa	TRUE	6	6
AGRN	sp|O00468-6	AGRIN_HUMAN	215 kDa		6	0
RBMX	sp|P38159	RBMX_HUMAN	42 kDa		6	3
RPS3A	sp|P61247	RS3A_HUMAN	30 kDa		6	3
CKAP4	sp|Q07065	CKAP4_HUMAN	66 kDa		6	5
PDLIM5	sp|Q96HC4	PDLI5_HUMAN	64 kDa		6	4
ACTR1A	sp|P61163	ACTZ_HUMAN	43 kDa	TRUE	6	2
VCAN	sp|P13611	CSPG2_HUMAN	373 kDa		6	2
ATP6V1A	sp|P38606	VATA_HUMAN	68 kDa		5	11
CHDH	sp|Q8NE62	CHDH_HUMAN	65 kDa		5	5
VIL1	sp|P09327	VILI_HUMAN	93 kDa		5	19
GOT2	sp|P00505	AATM_HUMAN	48 kDa		5	9
PFKL	sp|P17858	K6PL_HUMAN	85 kDa	TRUE	5	8
DECR1	tr|B7Z6B8	B7Z6B8_HUMAN	35 kDa		5	7
HSPE1	sp|P61604	CH10_HUMAN	11 kDa		5	8
AKR1C3	sp|P42330	AK1C3_HUMAN	37 kDa	TRUE	5	9
PNP	sp|P00491	PNPH_HUMAN	32 kDa	TRUE	5	5
KRT4	sp|P19013	K2C4_HUMAN	57 kDa	TRUE	5	0
MDH1	sp|P40925-3	MDHC_HUMAN	39 kDa		5	9
HIST1H1E	sp|P10412	H14_HUMAN	22 kDa	TRUE	5	11
EEF1G	sp|P26641	EF1G_HUMAN	50 kDa		5	5
CKB	sp|P12277	KCRB_HUMAN	43 kDa		5	1
ARF1	sp|P84077	ARF1_HUMAN	21 kDa	TRUE	5	7
PRDX5	sp|P30044	PRDX5_HUMAN	22 kDa		5	8
COX5A	sp|P20674	COX5A_HUMAN	17 kDa		5	8
RPSA	tr|C9J9K3	C9J9K3_HUMAN	30 kDa		5	6
PCBP1	sp|Q15365	PCBP1_HUMAN	37 kDa	TRUE	5	7
RAB5C	sp|P51148-2	RAB5C_HUMAN	27 kDa	TRUE	5	7
CTSB	sp|P07858	CATB_HUMAN	38 kDa		5	9
NONO	sp|Q15233	NONO_HUMAN	54 kDa	TRUE	5	8
FTH1	sp|P02794	FRIH_HUMAN	21 kDa		5	6
STOML2	sp|Q9UJZ1	STML2_HUMAN	39 kDa		5	4

PPIB	sp|P23284	PPIB_HUMAN	24 kDa	TRUE	5	3
MATR3	sp|P43243	MATR3_HUMAN	95 kDa		5	7
ARHGDIA	sp|P52565	GDIR1_HUMAN	23 kDa		5	5
CTTN	sp|Q14247	SRC8_HUMAN	62 kDa		5	2
GNB2L1	sp|P63244	GBLP_HUMAN	35 kDa		5	2
SEPT2	sp|Q15019-2	SEPT2_HUMAN	45 kDa		5	5
COX5B	sp|P10606	COX5B_HUMAN	14 kDa		5	4
VTN	sp|P04004	VTNC_HUMAN	54 kDa		5	7
LAMB2	sp|P55268	LAMB2_HUMAN	196 kDa		5	4
STOM	sp|P27105	STOM_HUMAN	32 kDa		5	1
CAPZA2	sp|P47755	CAZA2_HUMAN	33 kDa	TRUE	5	3
RPL18	sp|Q07020	RL18_HUMAN	22 kDa		5	3
LMNB1	sp|P20700	LMNB1_HUMAN	66 kDa	TRUE	5	3
C3	sp|P01024	CO3_HUMAN	187 kDa		4	11
GLYAT	sp|Q6IB77	GLYAT_HUMAN	34 kDa		4	10
SDHA	sp|P31040	SDHA_HUMAN	73 kDa		4	10
ALDH1L1	sp|O75891-3	AL1L1_HUMAN	100 kDa	TRUE	4	11
AK4	sp|P27144	KAD4_HUMAN	25 kDa		4	12
ACSL1	tr|B7Z452	B7Z452_HUMAN	78 kDa		4	11
PHB2	sp|Q99623	PHB2_HUMAN	33 kDa		4	8
GPI	sp|P06744	G6PI_HUMAN	63 kDa		4	7
AHCY	sp|P23526	SAHH_HUMAN	48 kDa		4	10
CALR	sp|P27797	CALR_HUMAN	48 kDa		4	14
GPD1	sp|P21695	GPDA_HUMAN	38 kDa	TRUE	4	5
MAOB	sp|P27338	AOFB_HUMAN	59 kDa	TRUE	4	7
FTL	sp|P02792	FRIL_HUMAN	20 kDa		4	6
HIBADH	sp|P31937	3HIDH_HUMAN	35 kDa		4	9
SLC25A13	sp|Q9UJS0-2	CMC2_HUMAN	74 kDa	TRUE	4	7
UGT1A6	sp|P19224	UD16_HUMAN	61 kDa	TRUE	4	8
CTNNA1	sp|P35221	CTNA1_HUMAN	100 kDa	TRUE	4	6
C21orf33	sp|P30042	ES1_HUMAN	28 kDa		4	5
CYB5R3	sp|P00387-3	NB5R3_HUMAN	38 kDa		4	6
ALDH9A1	sp|P49189	AL9A1_HUMAN	54 kDa		4	4
CAPG	sp|P40121	CAPG_HUMAN	38 kDa		4	8
CYB5A	sp|P00167	CYB5_HUMAN	15 kDa		4	7
COL1A1	sp|P02452	CO1A1_HUMAN	139 kDa		4	7
OGDHL	sp|Q9ULD0	OGDHL_HUMAN	114 kDa	TRUE	4	5
ATP5C1	sp|P36542	ATPG_HUMAN	33 kDa		4	4
TINAGL1	sp|Q9GZM7	TINAL_HUMAN	52 kDa		4	6
NIPSNAP1	sp|Q9BPW8	NIPS1_HUMAN	33 kDa	TRUE	4	8
VAT1	sp|Q99536	VAT1_HUMAN	42 kDa		4	4
TTR	sp|P02766	TTHY_HUMAN	16 kDa		4	3
PGD	sp|P52209	6PGD_HUMAN	53 kDa		4	2
CCT6A	sp|P40227	TCPZ_HUMAN	58 kDa		4	2
UGP2	sp|Q16851	UGPA_HUMAN	57 kDa		4	4
NDUFS3	sp|O75489	NDUS3_HUMAN	30 kDa		4	4
COL4A1	sp|P02462	CO4A1_HUMAN	161 kDa		4	7

GGT1	sp|P19440	GGT1_HUMAN	61 kDa		4	5
AMBP	sp|P02760	AMBP_HUMAN	39 kDa		4	5
CCT5	sp|P48643	TCPE_HUMAN	60 kDa		4	4
FABP3	sp|P05413	FABPH_HUMAN	15 kDa		4	5
CCT3	tr|B4DUR8	B4DUR8_HUMAN	56 kDa		4	7
TRAP1	tr|F5H897	F5H897_HUMAN	74 kDa		4	6
FAHD1	sp|Q6P587	FAHD1_HUMAN	25 kDa		4	3
ESD	sp|P10768	ESTD_HUMAN	31 kDa		4	3
GSR	sp|P00390-3	GSHR_HUMAN	53 kDa		4	5
CLU	sp|P10909	CLUS_HUMAN	52 kDa		4	5
EEF1D	sp|P29692-2	EF1D_HUMAN	71 kDa		4	1
VDAC2	sp|P45880-1	VDAC2_HUMAN	33 kDa	TRUE	4	5
CCT8	sp|P50990	TCPQ_HUMAN	60 kDa		4	5
PHGDH	sp|O43175	SERA_HUMAN	57 kDa		4	5
FGG	tr|C9JC84	C9JC84_HUMAN	52 kDa		4	1
CSTB	sp|P04080	CYTB_HUMAN	11 kDa		4	5
IGHA1	sp|P01876	IGHA1_HUMAN	38 kDa	TRUE	4	4
BLVRB	sp|P30043	BLVRB_HUMAN	22 kDa		4	5
UGT1A9	sp|O60656	UD19_HUMAN	60 kDa	TRUE	4	10
ATP5J	sp|P18859-2	ATP5J_HUMAN	13 kDa		4	5
ACTR2	sp|P61160	ARP2_HUMAN	45 kDa		4	4
SYNCRIP	sp|O60506	HNRPQ_HUMAN	70 kDa	TRUE	4	4
ARCN1	sp|P48444	COPD_HUMAN	57 kDa		4	3
RPS4X	sp|P62701	RS4X_HUMAN	30 kDa		4	2
PSMB1	sp|P20618	PSB1_HUMAN	26 kDa		4	2
CAPN1	sp|P07384	CAN1_HUMAN	82 kDa		4	3
RTCB	sp|Q9Y3I0	RTCB_HUMAN	55 kDa		4	2
TMED10	sp|P49755	TMEDA_HUMAN	25 kDa		4	4
CHCHD3	sp|Q9NX63	CHCH3_HUMAN	26 kDa		4	3
C9	sp|P02748	CO9_HUMAN	63 kDa		4	3
F13A1	sp|P00488	F13A_HUMAN	83 kDa		4	3
KHSRP	sp|Q92945	FUBP2_HUMAN	73 kDa	TRUE	4	2
DARS	sp|P14868	SYDC_HUMAN	57 kDa		4	3
LAD1	tr|E9PDI4	E9PDI4_HUMAN	59 kDa		4	1
ZYX	sp|Q15942	ZYX_HUMAN	61 kDa		4	5
YWHAQ	sp|P27348	1433T_HUMAN	28 kDa	TRUE	4	3
HP	sp|P00738	HPT_HUMAN	45 kDa	TRUE	4	0
GDI1	sp|P31150	GDIA_HUMAN	51 kDa	TRUE	4	8
PCBP2	sp|Q15366-2	PCBP2_HUMAN	39 kDa	TRUE	4	5
HIST2H2AB	sp|Q8IUE6	H2A2B_HUMAN	14 kDa	TRUE	4	0
ARF4	sp|P18085	ARF4_HUMAN	21 kDa	TRUE	4	0
ATP6V1B1	sp|P15313	VATB1_HUMAN	57 kDa	TRUE	4	0
PRDX4	sp|Q13162	PRDX4_HUMAN	31 kDa	TRUE	4	0
PDZK1	sp|Q5T2W1	NHRF3_HUMAN	57 kDa		3	19
LRPPRC	sp|P42704	LPPRC_HUMAN	158 kDa		3	8
ACOT1	sp|Q86TX2	ACOT1_HUMAN	46 kDa	TRUE	3	12
DDC	sp|P20711	DDC_HUMAN	54 kDa		3	6

ALDH7A1	sp|P49419-2	AL7A1_HUMAN	55 kDa		3	8
ACSF2	tr|B4DHT5	B4DHT5_HUMAN	67 kDa		3	8
ABAT	sp|P80404	GABT_HUMAN	56 kDa		3	4
AKR7A3	sp|O95154	ARK73_HUMAN	37 kDa	TRUE	3	5
DPP4	sp|P27487	DPP4_HUMAN	88 kDa		3	6
GOT1	sp|P17174	AATC_HUMAN	46 kDa		3	6
DPYS	sp|Q14117	DPYS_HUMAN	57 kDa	TRUE	3	9
AGMAT	sp|Q9BSE5	SPEB_HUMAN	38 kDa		3	6
PTGR1	sp|Q14914	PTGR1_HUMAN	36 kDa		3	9
SERPINA1	sp|P01009	A1AT_HUMAN	47 kDa	TRUE	3	5
GLS	sp|O94925	GLSK_HUMAN	73 kDa		3	7
MAOA	sp|P21397	AOFA_HUMAN	60 kDa	TRUE	3	4
ATP5H	sp|O75947-2	ATP5H_HUMAN	16 kDa		3	11
CRYL1	sp|Q9Y2S2	CRYL1_HUMAN	35 kDa		3	8
PGAM1	sp|P18669	PGAM1_HUMAN	29 kDa		3	10
MTHFD1	sp|P11586	C1TC_HUMAN	102 kDa		3	5
FGA	sp|P02671-2	FIBA_HUMAN	70 kDa		3	6
GRHPR	sp|Q9UBQ7	GRHPR_HUMAN	36 kDa		3	5
AMACR	sp|Q9UHK6	AMACR_HUMAN	42 kDa		3	17
DAB2	sp|P98082-3	DAB2_HUMAN	80 kDa		3	7
SLC3A2	sp|P08195-3	4F2_HUMAN	62 kDa		3	10
LONP1	tr|K7EKE6	K7EKE6_HUMAN	95 kDa		3	5
SUCLG1	sp|P53597	SUCA_HUMAN	36 kDa		3	5
VDAC1	sp|P21796	VDAC1_HUMAN	31 kDa	TRUE	3	7
CFL1	sp|P23528	COF1_HUMAN	19 kDa		3	6
DHX9	sp|Q08211	DHX9_HUMAN	141 kDa		3	5
NAT8	sp|Q9UHE5	NAT8_HUMAN	26 kDa	TRUE	3	6
C4B	sp|P0C0L5	CO4B_HUMAN	193 kDa		3	3
CAP1	sp|Q01518	CAP1_HUMAN	52 kDa		3	5
WDR1	sp|O75083	WDR1_HUMAN	66 kDa		3	4
ATP5F1	sp|P24539	AT5F1_HUMAN	29 kDa		3	3
XRCC5	sp|P13010	XRCC5_HUMAN	83 kDa		3	6
UQCRFS1	sp|P47985	UCRI_HUMAN	30 kDa	TRUE	3	4
ECI2	sp|O75521-2	ECI2_HUMAN	40 kDa		3	4
CYCS	sp|P99999	CYC_HUMAN	12 kDa		3	5
PDIA4	sp|P13667	PDIA4_HUMAN	73 kDa		3	3
PCYOX1	sp|Q9UHG3	PCYOX_HUMAN	57 kDa		3	4
SND1	sp|Q7KZF4	SND1_HUMAN	102 kDa		3	7
CTNNB1	tr|B4DGU4	B4DGU4_HUMAN	85 kDa		3	3
ATP6V1H	sp|Q9UI12	VATH_HUMAN	56 kDa		3	4
HNRNPL	sp|P14866	HNRPL_HUMAN	64 kDa		3	4
FGB	sp|P02675	FIBB_HUMAN	56 kDa		3	1
HLA-A	sp|P13746-2	1A11_HUMAN	41 kDa	TRUE	3	3
PGRMC1	sp|O00264	PGRC1_HUMAN	22 kDa	TRUE	3	3
SERPINA3	sp|P01011	AACT_HUMAN	48 kDa	TRUE	3	4
DBI	sp|P07108-2	ACBP_HUMAN	12 kDa		3	3
SHMT2	sp|P34897	GLYM_HUMAN	56 kDa	TRUE	3	6

RPL6	sp|Q02878	RL6_HUMAN	33 kDa		3	4
PICALM	sp|Q13492-2	PICAL_HUMAN	69 kDa	TRUE	3	4
HNRNPH3	sp|P31942-2	HNRH3_HUMAN	35 kDa		3	4
COX6C	sp|P09669	COX6C_HUMAN	9 kDa		3	4
LUM	sp|P51884	LUM_HUMAN	38 kDa		3	1
HDGF	sp|P51858	HDGF_HUMAN	27 kDa		3	3
RPN2	sp|P04844-2	RPN2_HUMAN	68 kDa		3	6
NDUFA13	sp|Q9P0J0	NDUAD_HUMAN	17 kDa		3	3
SET	sp|Q01105-3	SET_HUMAN	31 kDa		3	3
RAB14	sp|P61106	RAB14_HUMAN	24 kDa	TRUE	3	3
ADD1	sp|P35611-2	ADDA_HUMAN	70 kDa		3	2
RPS9	sp|P46781	RS9_HUMAN	23 kDa		3	1
ATIC	sp|P31939	PUR9_HUMAN	65 kDa		3	5
RNH1	sp|P13489	RINI_HUMAN	50 kDa		3	4
SERPINH1	sp|P50454	SERPH_HUMAN	46 kDa		3	2
H2AFY	sp|O75367-2	H2AY_HUMAN	39 kDa	TRUE	3	3
CORO1B	sp|Q9BR76	COR1B_HUMAN	54 kDa		3	3
SLIRP	sp|Q9GZT3-2	SLIRP_HUMAN	12 kDa		3	3
POSTN	sp|Q15063-3	POSTN_HUMAN	87 kDa		3	4
HSPA4	sp|P34932	HSP74_HUMAN	94 kDa		3	4
DDX39B	sp|Q13838-2	DX39B_HUMAN	51 kDa		3	3
GNAI2	sp|P04899-4	GNAI2_HUMAN	42 kDa	TRUE	3	3
CLYBL	sp|Q8N0X4	CLYBL_HUMAN	37 kDa		3	1
RPL13	sp|P26373	RL13_HUMAN	24 kDa		3	2
ATP2A2	sp|P16615-2	AT2A2_HUMAN	110 kDa		3	2
RPS16	sp|P62249	RS16_HUMAN	16 kDa		3	1
RPL7	sp|P18124	RL7_HUMAN	29 kDa		3	1
ARL6IP5	sp|O75915	PRAF3_HUMAN	22 kDa		3	3
SCAMP2	sp|O15127	SCAM2_HUMAN	37 kDa		3	3
GSTT1	sp|P30711	GSTT1_HUMAN	27 kDa		3	2
CORO1C	sp|Q9ULV4-2	COR1C_HUMAN	54 kDa		3	3
SRSF1	sp|Q07955	SRSF1_HUMAN	28 kDa		3	1
COPA	sp|P53621	COPA_HUMAN	138 kDa		3	4
FUBP1	sp|Q96AE4	FUBP1_HUMAN	68 kDa	TRUE	3	0
RPL3	sp|P39023	RL3_HUMAN	46 kDa		3	3
ACLY	sp|P53396-2	ACLY_HUMAN	120 kDa		3	2
CRIP2	sp|P52943-2	CRIP2_HUMAN	30 kDa		3	1
TPD52	tr|F5H0B0	F5H0B0_HUMAN	31 kDa	TRUE	3	3
STX7	sp|O15400-2	STX7_HUMAN	27 kDa		3	3
S100A9	sp|P06702	S10A9_HUMAN	13 kDa		3	0
RPS6	sp|P62753	RS6_HUMAN	29 kDa		3	1
VPS26A	tr|F5H4L7	F5H4L7_HUMAN	37 kDa		3	3
RAB10	sp|P61026	RAB10_HUMAN	23 kDa	TRUE	3	2
HNRNPR	sp|O43390	HNRPR_HUMAN	71 kDa	TRUE	3	3
MAT2A	sp|P31153	METK2_HUMAN	44 kDa		3	2
UQCRQ	sp|O14949	QCR8_HUMAN	10 kDa		3	3
RPL31	sp|P62899	RL31_HUMAN	14 kDa		3	3

ARHGAP1	sp|Q07960	RHG01_HUMAN	50 kDa		3	0
NAGK	sp|Q9UJ70-2	NAGK_HUMAN	42 kDa		3	0
APRT	sp|P07741	APT_HUMAN	20 kDa		3	2
HLA-B	sp|P01889	1B07_HUMAN	40 kDa	TRUE	3	4
SFXN3	sp|Q9BWM7	SFXN3_HUMAN	36 kDa		3	2
RPL7A	sp|P62424	RL7A_HUMAN	30 kDa		3	0
RAB1B	sp|Q9H0U4	RAB1B_HUMAN	22 kDa	TRUE	3	6
AKR1B1	sp|P15121	ALDR_HUMAN	36 kDa		3	3
PDLIM1	sp|O00151	PDLI1_HUMAN	36 kDa		3	2
PALLD	sp|Q8WX93	PALLD_HUMAN	151 kDa		3	0
H2AFZ	sp|P0C0S5	H2AZ_HUMAN	14 kDa	TRUE	3	0
PTGDS	tr|H0Y5A1	H0Y5A1_HUMAN	23 kDa		3	2
NSFL1C	sp|Q9UNZ2-5	NSF1C_HUMAN	41 kDa		3	2
LMAN2	sp|Q12907	LMAN2_HUMAN	40 kDa		3	1
HP	tr|H3BS21	H3BS21_HUMAN	25 kDa	TRUE	3	0
RBM39	sp|Q14498-2	RBM39_HUMAN	59 kDa		3	0
PML	sp|P29590-5	PML_HUMAN	70 kDa		3	1
EIF4A3	sp|P38919	IF4A3_HUMAN	47 kDa	TRUE	3	0
EFTUD2	sp|Q15029-2	U5S1_HUMAN	105 kDa	TRUE	3	0
SEMG1	sp|P04279	SEMG1_HUMAN	52 kDa	TRUE	3	0
HADH	sp|Q16836-2	HCDH_HUMAN	42 kDa		2	9
IDH1	sp|O75874	IDHC_HUMAN	47 kDa	TRUE	2	7
SFXN1	sp|Q9H9B4	SFXN1_HUMAN	36 kDa		2	10
CS	sp|O75390	CISY_HUMAN	52 kDa	TRUE	2	2
HOGA1	sp|Q86XE5	HOGA1_HUMAN	35 kDa	TRUE	2	2
ISOC2	sp|Q96AB3-2	ISOC2_HUMAN	24 kDa		2	5
FMO1	sp|Q01740	FMO1_HUMAN	60 kDa		2	3
MTCH2	sp|Q9Y6C9	MTCH2_HUMAN	33 kDa		2	7
FAH	sp|P16930	FAAA_HUMAN	46 kDa		2	7
TINAG	sp|Q9UJW2	TINAG_HUMAN	55 kDa		2	3
C1QBP	sp|Q07021	C1QBP_HUMAN	31 kDa		2	5
AKR7A2	sp|O43488	ARK72_HUMAN	40 kDa	TRUE	2	5
GDA	sp|Q9Y2T3	GUAD_HUMAN	51 kDa		2	9
CANX	sp|P27824	CALX_HUMAN	68 kDa		2	6
IVD	sp|P26440	IVD_HUMAN	46 kDa		2	4
LAMB1	sp|P07942	LAMB1_HUMAN	198 kDa		2	8
SPR	sp|P35270	SPRE_HUMAN	28 kDa		2	6
SORD	sp|Q00796	DHSO_HUMAN	38 kDa		2	5
CLTA	sp|P09496-2	CLCA_HUMAN	24 kDa		2	4
MCCC1	sp|Q96RQ3	MCCA_HUMAN	80 kDa		2	1
CLIC4	sp|Q9Y696	CLIC4_HUMAN	29 kDa	TRUE	2	5
ABHD14B	sp|Q96IU4	ABHEB_HUMAN	22 kDa		2	3
PRDX3	sp|P30048-2	PRDX3_HUMAN	26 kDa		2	5
SFXN2	sp|Q96NB2	SFXN2_HUMAN	36 kDa		2	2
ALDH3A2	sp|P51648-2	AL3A2_HUMAN	58 kDa	TRUE	2	4
HRSP12	sp|P52758	UK114_HUMAN	14 kDa		2	3
SLC25A3	sp|Q00325-2	MPCP_HUMAN	40 kDa		2	2

HSD11B2	sp|P80365	DHI2_HUMAN	44 kDa		2	3
PGM1	sp|P36871	PGM1_HUMAN	61 kDa		2	2
BHMT2	sp|Q9H2M3	BHMT2_HUMAN	40 kDa	TRUE	2	4
GSTK1	sp|Q9Y2Q3	GSTK1_HUMAN	25 kDa		2	2
COTL1	sp|Q14019	COTL1_HUMAN	16 kDa		2	4
CTNND1	sp|O60716-3	CTND1_HUMAN	105 kDa		2	5
UQCRB	sp|P14927	QCR7_HUMAN	14 kDa		2	1
CCT4	sp|P50991	TCPD_HUMAN	58 kDa		2	4
VPS35	sp|Q96QK1	VPS35_HUMAN	92 kDa		2	5
DDX17	sp|Q92841	DDX17_HUMAN	80 kDa	TRUE	2	2
RHOA	sp|P61586	RHOA_HUMAN	22 kDa	TRUE	2	3
AOX1	sp|Q06278	AOXA_HUMAN	148 kDa	TRUE	2	2
RAB1A	sp|P62820	RAB1A_HUMAN	23 kDa	TRUE	2	4
RPS7	sp|P62081	RS7_HUMAN	22 kDa		2	4
ACTR3	sp|P61158	ARP3_HUMAN	47 kDa		2	3
ADH1B	sp|P00325	ADH1B_HUMAN	40 kDa		2	3
PDCD6IP	sp|Q8WUM4-2	PDC6I_HUMAN	97 kDa		2	3
ACSS3	sp|Q9H6R3	ACSS3_HUMAN	75 kDa		2	1
AQP1	sp|P29972	AQP1_HUMAN	29 kDa		2	3
HINT1	sp|P49773	HINT1_HUMAN	14 kDa		2	3
RAB11A	sp|P62491-2	RB11A_HUMAN	18 kDa	TRUE	2	4
LETM1	sp|O95202	LETM1_HUMAN	83 kDa		2	2
GLYATL1	sp|Q969I3	GLYL1_HUMAN	35 kDa		2	2
ECHDC1	sp|Q9NTX5-2	ECHD1_HUMAN	33 kDa		2	4
PIGR	sp|P01833	PIGR_HUMAN	83 kDa		2	8
NARS	sp|O43776	SYNC_HUMAN	63 kDa		2	2
NDUFV2	sp|P19404	NDUV2_HUMAN	27 kDa		2	1
PRKAR2A	sp|P13861	KAP2_HUMAN	46 kDa	TRUE	2	3
YBX1	sp|P67809	YBOX1_HUMAN	36 kDa	TRUE	2	2
PGRMC2	sp|O15173-2	PGRC2_HUMAN	26 kDa	TRUE	2	3
ATP5J2-PTCD1	tr|G3V325	G3V325_HUMAN	84 kDa		2	3
ANXA11	sp|P50995	ANX11_HUMAN	54 kDa		2	4
ILF2	sp|Q12905	ILF2_HUMAN	43 kDa		2	4
MIF	sp|P14174	MIF_HUMAN	12 kDa		2	4
COPB2	tr|B4DZI8	B4DZI8_HUMAN	99 kDa		2	2
RDH11	sp|Q8TC12	RDH11_HUMAN	35 kDa		2	2
EPS8L2	sp|Q9H6S3	ES8L2_HUMAN	81 kDa		2	1
CSRP1	sp|P21291	CSRP1_HUMAN	21 kDa		2	2
SSBP1	sp|Q04837	SSBP_HUMAN	17 kDa		2	2
RPS8	sp|P62241	RS8_HUMAN	24 kDa		2	3
RPLP0	sp|P05388	RLA0_HUMAN	34 kDa		2	2
PSMA6	sp|P60900	PSA6_HUMAN	27 kDa		2	3
ECI1	sp|P42126-2	ECI1_HUMAN	31 kDa		2	2
ATP5D	sp|P30049	ATPD_HUMAN	17 kDa		2	2
SEC22B	sp|O75396	SC22B_HUMAN	25 kDa		2	2
FKBP4	sp|Q02790	FKBP4_HUMAN	52 kDa		2	3
SNX2	sp|O60749	SNX2_HUMAN	58 kDa	TRUE	2	4

PLS3	sp|P13797	PLST_HUMAN	71 kDa	TRUE	2	2
CCT7	sp|Q99832-3	TCPH_HUMAN	55 kDa		2	2
PURA	sp|Q00577	PURA_HUMAN	35 kDa	TRUE	2	2
RPS18	sp|P62269	RS18_HUMAN	18 kDa		2	3
RPLP2	sp|P05387	RLA2_HUMAN	12 kDa		2	3
BCAP31	sp|P51572-2	BAP31_HUMAN	35 kDa		2	3
EIF4H	sp|Q15056	IF4H_HUMAN	27 kDa		2	5
DDX3X	sp|O00571-2	DDX3X_HUMAN	71 kDa	TRUE	2	2
CRAT	sp|P43155-2	CACP_HUMAN	69 kDa		2	0
PABPC1	sp|P11940-2	PABP1_HUMAN	61 kDa	TRUE	2	1
SRSF7	sp|Q16629-2	SRSF7_HUMAN	16 kDa	TRUE	2	2
SSR4	sp|P51571	SSRD_HUMAN	19 kDa		2	1
RPL12	sp|P30050	RL12_HUMAN	18 kDa		2	2
CAPZB	tr|B1AK88	B1AK88_HUMAN	34 kDa		2	2
COPG1	sp|Q9Y678	COPG1_HUMAN	98 kDa		2	2
ARPC4-TTLL3	tr|F8WCF6	F8WCF6_HUMAN	21 kDa		2	3
PSME2	sp|Q9UL46	PSME2_HUMAN	27 kDa		2	1
TBCA	sp|O75347	TBCA_HUMAN	13 kDa		2	5
PSMD3	sp|O43242	PSMD3_HUMAN	61 kDa		2	2
TRIM28	sp|Q13263	TIF1B_HUMAN	89 kDa		2	2
UBE2N	sp|P61088	UBE2N_HUMAN	17 kDa		2	3
DBNL	sp|Q9UJU6	DBNL_HUMAN	48 kDa		2	2
TMOD3	sp|Q9NYL9	TMOD3_HUMAN	40 kDa		2	4
AKAP12	sp|Q02952	AKA12_HUMAN	191 kDa		2	2
ITGB1	sp|P05556	ITB1_HUMAN	88 kDa		2	1
RPL23A	sp|P62750	RL23A_HUMAN	18 kDa		2	1
ANXA7	sp|P20073-2	ANXA7_HUMAN	50 kDa		2	2
PSMA4	sp|P25789	PSA4_HUMAN	29 kDa		2	0
CSRP2	sp|Q16527	CSRP2_HUMAN	21 kDa		2	2
CORO1A	sp|P31146	COR1A_HUMAN	51 kDa		2	3
PSMC3	tr|E9PM69	E9PM69_HUMAN	44 kDa		2	2
RPL22	sp|P35268	RL22_HUMAN	15 kDa		2	2
ALDOC	sp|P09972	ALDOC_HUMAN	39 kDa	TRUE	2	3
TPP1	sp|O14773-2	TPP1_HUMAN	34 kDa		2	2
ARHGDIB	sp|P52566	GDIR2_HUMAN	23 kDa		2	5
DIABLO	sp|Q9NR28-2	DBLOH_HUMAN	21 kDa		2	2
PSMB4	sp|P28070	PSB4_HUMAN	29 kDa		2	2
TYMP	sp|P19971	TYPH_HUMAN	50 kDa		2	3
ESYT1	sp|Q9BSJ8	ESYT1_HUMAN	123 kDa		2	0
GNB2	sp|P62879	GBB2_HUMAN	37 kDa	TRUE	2	1
TMPO	sp|P42166	LAP2A_HUMAN	75 kDa		2	1
GPX1	sp|P07203	GPX1_HUMAN	22 kDa		2	3
APMAP	sp|Q9HDC9-2	APMAP_HUMAN	32 kDa		2	2
VAMP2	sp|P63027	VAMP2_HUMAN	13 kDa	TRUE	2	2
CTSZ	sp|Q9UBR2	CATZ_HUMAN	34 kDa		2	2
C14orf159	sp|Q7Z3D6	CN159_HUMAN	66 kDa		2	0
HNRNPAB	sp|Q99729-2	ROAA_HUMAN	36 kDa		2	1

FUS	sp|P35637-2	FUS_HUMAN	53 kDa	TRUE	2	1
RPS26	sp|P62854	RS26_HUMAN	13 kDa		2	2
RUVBL2	sp|Q9Y230	RUVB2_HUMAN	51 kDa		2	1
RRAS2	sp|P62070	RRAS2_HUMAN	23 kDa	TRUE	2	2
PFKM	sp|P08237	K6PF_HUMAN	85 kDa	TRUE	2	2
SRSF3	sp|P84103	SRSF3_HUMAN	19 kDa	TRUE	2	2
VPS29	sp|Q9UBQ0-2	VPS29_HUMAN	21 kDa		2	1
CYB5B	sp|O43169	CYB5B_HUMAN	16 kDa		2	1
RPL5	sp|P46777	RL5_HUMAN	34 kDa		2	0
KCTD12	sp|Q96CX2	KCD12_HUMAN	36 kDa		2	1
EIF3A	tr|F5H335	F5H335_HUMAN	163 kDa		2	1
FLOT1	sp|O75955	FLOT1_HUMAN	47 kDa		2	0
GNAS	sp|Q5JWF2-2	GNAS1_HUMAN	110 kDa	TRUE	2	2
RPS21	sp|P63220	RS21_HUMAN	9 kDa		2	0
PSMD11	sp|O00231-2	PSD11_HUMAN	48 kDa		2	2
ALYREF	tr|E9PB61	E9PB61_HUMAN	28 kDa		2	0
EPPK1	sp|P58107	EPIPL_HUMAN	556 kDa	TRUE	2	0
CTSH	sp|P09668	CATH_HUMAN	37 kDa		2	1
CAPZA1	sp|P52907	CAZA1_HUMAN	33 kDa	TRUE	2	3
MYOF	sp|Q9NZM1-3	MYOF_HUMAN	233 kDa		2	0
MYH13	sp|Q9UKX3	MYH13_HUMAN	224 kDa	TRUE	2	0
LTF	sp|P02788-2	TRFL_HUMAN	73 kDa		2	0
APEX1	sp|P27695	APEX1_HUMAN	36 kDa		2	1
FBN1	sp|P35555	FBN1_HUMAN	312 kDa		2	2
USO1	tr|F5GYR8	F5GYR8_HUMAN	109 kDa		2	1
ITGAV	sp|P06756-3	ITAV_HUMAN	111 kDa		2	1
HIST1H1B	sp|P16401	H15_HUMAN	23 kDa	TRUE	2	2
FERMT2	sp|Q96AC1	FERM2_HUMAN	78 kDa		2	0
DCD	sp|P81605	DCD_HUMAN	11 kDa		2	1
RUVBL1	sp|Q9Y265	RUVB1_HUMAN	50 kDa		2	1
BASP1	sp|P80723	BASP1_HUMAN	23 kDa		2	1
FBLN1	sp|P23142-3	FBLN1_HUMAN	77 kDa	TRUE	2	3
SRRM2	sp|Q9UQ35	SRRM2_HUMAN	300 kDa		2	0
SAMHD1	sp|Q9Y3Z3	SAMH1_HUMAN	72 kDa		2	0
HNRNPA0	sp|Q13151	ROA0_HUMAN	31 kDa		2	0
RAP2B	sp|P61225	RAP2B_HUMAN	21 kDa		2	0
EPB41L2	tr|E9PHY5	E9PHY5_HUMAN	104 kDa		2	0
LAMA4	sp|Q16363-2	LAMA4_HUMAN	202 kDa		2	0
IGFBP7	sp|Q16270-2	IBP7_HUMAN	29 kDa		2	1
ANXA3	sp|P12429	ANXA3_HUMAN	36 kDa		2	0
GSTA2	sp|P09210	GSTA2_HUMAN	26 kDa	TRUE	1	2
DAK	sp|Q3LXA3	DHAK_HUMAN	59 kDa		1	4
DMGDH	sp|Q9UI17	M2GD_HUMAN	97 kDa		1	9
SLC4A4	sp|Q9Y6R1-2	S4A4_HUMAN	116 kDa		1	9
FTCD	sp|O95954	FTCD_HUMAN	59 kDa		1	3
ACAA1	sp|P09110	THIK_HUMAN	44 kDa		1	8
DPEP1	sp|P16444	DPEP1_HUMAN	46 kDa		1	11

UGT2B7	sp|P16662	UD2B7_HUMAN	61 kDa	TRUE	1	7
TST	sp|Q16762	THTR_HUMAN	33 kDa	TRUE	1	5
KHK	sp|P50053-2	KHK_HUMAN	33 kDa		1	5
ACADSB	sp|P45954	ACDSB_HUMAN	47 kDa		1	5
ACADS	sp|P16219	ACADS_HUMAN	44 kDa		1	4
EPHX2	sp|P34913	HYES_HUMAN	63 kDa		1	2
ATP6V1E1	sp|P36543	VATE1_HUMAN	26 kDa		1	5
MGAM	tr|E7ER45	E7ER45_HUMAN	312 kDa	TRUE	1	11
SELENBP1	tr|A6PVW9	A6PVW9_HUMAN	57 kDa		1	4
PBLD	sp|P30039	PBLD_HUMAN	32 kDa		1	5
ECHDC3	sp|Q96DC8	ECHD3_HUMAN	33 kDa		1	9
CPT2	sp|P23786	CPT2_HUMAN	74 kDa		1	5
CMBL	sp|Q96DG6	CMBL_HUMAN	28 kDa		1	6
EPHX1	sp|P07099	HYEP_HUMAN	53 kDa		1	4
NDUFV1	sp|P49821	NDUV1_HUMAN	51 kDa		1	5
MPST	sp|P25325	THTM_HUMAN	33 kDa	TRUE	1	5
PDHA1	sp|P08559-2	ODPA_HUMAN	44 kDa		1	5
PTBP1	sp|P26599-2	PTBP1_HUMAN	59 kDa	TRUE	1	3
NDUFA9	sp|Q16795	NDUA9_HUMAN	43 kDa		1	2
NAPRT1	tr|G5E977	G5E977_HUMAN	62 kDa		1	6
MARC2	sp|Q969Z3	MARC2_HUMAN	38 kDa		1	0
TTN	sp|Q8WZ42-12	TITIN_HUMAN	3994 kDa		1	0
DDAH1	sp|O94760	DDAH1_HUMAN	31 kDa	TRUE	1	5
CMPK1	sp|P30085	KCY_HUMAN	22 kDa		1	6
QDPR	sp|P09417	DHPR_HUMAN	26 kDa		1	3
CALB1	sp|P05937	CALB1_HUMAN	30 kDa		1	1
GALM	sp|Q96C23	GALM_HUMAN	38 kDa		1	2
BPHL	sp|Q86WA6-2	BPHL_HUMAN	31 kDa		1	3
HGD	sp|Q93099	HGD_HUMAN	50 kDa		1	1
PRKCSH	sp|P14314-2	GLU2B_HUMAN	59 kDa		1	3
UGDH	sp|O60701	UGDH_HUMAN	55 kDa		1	2
SUOX	sp|P51687	SUOX_HUMAN	60 kDa		1	6
ATP6V0A1	sp|Q93050-1	VPP1_HUMAN	96 kDa		1	1
DNPH1	tr|H0Y8X4	H0Y8X4_HUMAN	26 kDa		1	4
PEPD	sp|P12955	PEPD_HUMAN	55 kDa		1	2
PDZK1IP1	sp|Q13113	PDZ1I_HUMAN	12 kDa		1	4
EIF5A	sp|P63241-2	IF5A1_HUMAN	20 kDa		1	2
PPA2	sp|Q9H2U2-2	IPYR2_HUMAN	40 kDa		1	3
NME2	sp|P22392-2	NDKB_HUMAN	30 kDa	TRUE	1	1
ATP6V1D	sp|Q9Y5K8	VATD_HUMAN	28 kDa		1	3
ENPEP	sp|Q07075	AMPE_HUMAN	109 kDa		1	1
OCIAD2	sp|Q56VL3	OCAD2_HUMAN	17 kDa		1	3
DLAT	sp|P10515	ODP2_HUMAN	69 kDa		1	2
PSMA1	sp|P25786	PSA1_HUMAN	30 kDa		1	5
APOE	sp|P02649	APOE_HUMAN	36 kDa		1	1
NIT2	sp|Q9NQR4	NIT2_HUMAN	31 kDa		1	4
SLC25A11	sp|Q02978	M2OM_HUMAN	34 kDa		1	1

BCAM	sp|P50895	BCAM_HUMAN	67 kDa		1	1
MAP4	tr|E7EVA0	E7EVA0_HUMAN	245 kDa		1	2
NDUFA10	tr|E7ESZ7	E7ESZ7_HUMAN	45 kDa		1	1
BCKDHA	sp|P12694	ODBA_HUMAN	50 kDa		1	3
PSME1	sp|Q06323	PSME1_HUMAN	29 kDa		1	3
NDUFB4	sp|O95168	NDUB4_HUMAN	15 kDa		1	0
SLC25A1	sp|P53007	TXTP_HUMAN	34 kDa		1	2
LTA4H	sp|P09960	LKHA4_HUMAN	69 kDa		1	2
PPP2R1A	sp|P30153	2AAA_HUMAN	65 kDa	TRUE	1	3
TXN	sp|P10599	THIO_HUMAN	12 kDa		1	2
EHD4	sp|Q9H223	EHD4_HUMAN	61 kDa	TRUE	1	2
HDHD3	sp|Q9BSH5	HDHD3_HUMAN	28 kDa		1	2
ADH5	sp|P11766	ADHX_HUMAN	40 kDa		1	0
PCBD1	sp|P61457	PHS_HUMAN	12 kDa		1	3
RAP1B	sp|P61224-2	RAP1B_HUMAN	15 kDa		1	3
CISD1	sp|Q9NZ45	CISD1_HUMAN	12 kDa		1	1
TALDO1	sp|P37837	TALDO_HUMAN	38 kDa		1	1
ARPC2	sp|O15144	ARPC2_HUMAN	34 kDa		1	1
CLIC1	sp|O00299	CLIC1_HUMAN	27 kDa	TRUE	1	1
RPS17L	sp|P0CW22	RS17L_HUMAN	16 kDa	TRUE	1	2
MSRA	sp|Q9UJ68-2	MSRA_HUMAN	19 kDa		1	3
ECHDC2	sp|Q86YB7-2	ECHD2_HUMAN	28 kDa		1	1
CAPNS1	sp|P04632	CPNS1_HUMAN	28 kDa		1	3
SUB1	sp|P53999	TCP4_HUMAN	14 kDa		1	6
SUCLA2	sp|Q9P2R7-2	SUCB1_HUMAN	48 kDa		1	2
LMAN1	sp|P49257	LMAN1_HUMAN	58 kDa		1	2
ASAH1	sp|Q13510-2	ASAH1_HUMAN	47 kDa		1	1
VARS	sp|P26640	SYVC_HUMAN	140 kDa		1	2
S100A6	sp|P06703	S10A6_HUMAN	10 kDa		1	1
PSAT1	sp|Q9Y617	SERC_HUMAN	40 kDa		1	1
AP2A2	sp|O94973-2	AP2A2_HUMAN	104 kDa	TRUE	1	2
AP2B1	sp|P63010-3	AP2B1_HUMAN	98 kDa	TRUE	1	0
ETFDH	sp|Q16134	ETFD_HUMAN	68 kDa		1	2
SLC5A2	sp|P31639	SC5A2_HUMAN	73 kDa		1	1
METTL7A	sp|Q9H8H3	MET7A_HUMAN	28 kDa		1	2
SERBP1	sp|Q8NC51-2	PAIRB_HUMAN	44 kDa		1	1
CRYAA	sp|P02489	CRYAA_HUMAN	20 kDa		1	4
NDUFB5	sp|O43674	NDUB5_HUMAN	22 kDa		1	1
ADD3	sp|Q9UEY8-2	ADDG_HUMAN	76 kDa	TRUE	1	3
NUCB1	sp|Q02818	NUCB1_HUMAN	54 kDa		1	2
NDUFA2	sp|O43678	NDUA2_HUMAN	11 kDa		1	1
PIPOX	sp|Q9P0Z9	SOX_HUMAN	44 kDa		1	1
LCP1	sp|P13796	PLSL_HUMAN	70 kDa	TRUE	1	3
NDRG1	sp|Q92597	NDRG1_HUMAN	43 kDa		1	3
ACOX2	sp|Q99424	ACOX2_HUMAN	77 kDa		1	1
PGLS	sp|O95336	6PGL_HUMAN	28 kDa		1	2
GSTM3	sp|P21266	GSTM3_HUMAN	27 kDa	TRUE	1	0

ALDH8A1	sp|Q9H2A2	AL8A1_HUMAN	53 kDa		1	0
ATP6V0D1	tr|F5GYQ1	F5GYQ1_HUMAN	45 kDa		1	2
NACA	sp|E9PAV3	NACAM_HUMAN	205 kDa		1	2
RPL4	sp|P36578	RL4_HUMAN	48 kDa		1	4
GGT5	sp|P36269-2	GGT5_HUMAN	59 kDa		1	5
RTN4	sp|Q9NQC3	RTN4_HUMAN	130 kDa		1	1
CYC1	sp|P08574	CY1_HUMAN	35 kDa		1	0
PSMD2	sp|Q13200	PSMD2_HUMAN	100 kDa		1	1
IDH3A	sp|P50213	IDH3A_HUMAN	40 kDa		1	1
PDLIM2	sp|Q96JY6	PDLI2_HUMAN	37 kDa		1	0
SERPINB6	sp|P35237	SPB6_HUMAN	43 kDa		1	1
COX6A1	sp|P12074	CX6A1_HUMAN	12 kDa		1	3
COX7A2	sp|P14406	CX7A2_HUMAN	9 kDa		1	1
ERP29	sp|P30040	ERP29_HUMAN	29 kDa		1	2
AFG3L2	sp|Q9Y4W6	AFG32_HUMAN	89 kDa		1	3
RPS2	sp|P15880	RS2_HUMAN	31 kDa		1	0
PSMA2	sp|P25787	PSA2_HUMAN	26 kDa		1	0
RPS20	sp|P60866-2	RS20_HUMAN	16 kDa		1	2
PSMA7	sp|O14818	PSA7_HUMAN	28 kDa		1	2
SNX3	sp|O60493	SNX3_HUMAN	19 kDa	TRUE	1	2
LPP	sp|Q93052	LPP_HUMAN	66 kDa		1	1
RPS10	sp|P46783	RS10_HUMAN	19 kDa		1	2
LAMP1	sp|P11279	LAMP1_HUMAN	45 kDa		1	1
NPEPPS	sp|P55786	PSA_HUMAN	103 kDa		1	1
PYGB	sp|P11216	PYGB_HUMAN	97 kDa	TRUE	1	1
RPS14	sp|P62263	RS14_HUMAN	16 kDa		1	2
EIF6	sp|P56537	IF6_HUMAN	27 kDa		1	2
ATP5I	sp|P56385	ATP5I_HUMAN	8 kDa		1	1
SEPT7	tr|E7EPK1	E7EPK1_HUMAN	51 kDa		1	2
RPS19	sp|P39019	RS19_HUMAN	16 kDa		1	1
CYB5R1	sp|Q9UHQ9	NB5R1_HUMAN	34 kDa		1	0
GLOD4	sp|Q9HC38-2	GLOD4_HUMAN	33 kDa		1	2
RAN	sp|P62826	RAN_HUMAN	24 kDa	TRUE	1	0
RENBP	sp|P51606	RENBP_HUMAN	49 kDa		1	1
H1F0	sp|P07305-2	H10_HUMAN	19 kDa		1	1
PRKAR1A	sp|P10644	KAP0_HUMAN	43 kDa		1	1
CIRBP	sp|Q14011	CIRBP_HUMAN	19 kDa		1	4
AP2M1	tr|E9PFW3	E9PFW3_HUMAN	52 kDa		1	1
RPS28	sp|P62857	RS28_HUMAN	8 kDa		1	2
IARS2	sp|Q9NSE4	SYIM_HUMAN	114 kDa		1	1
SIAE	sp|Q9HAT2	SIAE_HUMAN	58 kDa		1	4
ANP32A	sp|P39687	AN32A_HUMAN	29 kDa		1	0
QPRT	sp|Q15274	NADC_HUMAN	31 kDa		1	1
APEH	tr|C9JIF9	C9JIF9_HUMAN	82 kDa		1	2
ARPC3	sp|O15145	ARPC3_HUMAN	21 kDa		1	2
COQ9	sp|O75208	COQ9_HUMAN	36 kDa		1	1
PSMD14	sp|O00487	PSDE_HUMAN	35 kDa		1	1

RPL9	sp|P32969	RL9_HUMAN	22 kDa		1	1
ALAD	sp|P13716	HEM2_HUMAN	36 kDa		1	1
USP5	sp|P45974-2	UBP5_HUMAN	93 kDa		1	0
HMGB1	sp|P09429	HMGB1_HUMAN	25 kDa		1	1
SLC6A19	sp|Q695T7	S6A19_HUMAN	71 kDa		1	1
ACAD8	sp|Q9UKU7	ACAD8_HUMAN	45 kDa		1	1
PSMA5	sp|P28066	PSA5_HUMAN	26 kDa		1	2
DNM2	sp|P50570-3	DYN2_HUMAN	98 kDa		1	2
GBAS	sp|O75323-2	NIPS2_HUMAN	29 kDa	TRUE	1	2
NDUFB10	sp|O96000	NDUBA_HUMAN	21 kDa		1	1
FAM21A	sp|Q641Q2-2	FA21A_HUMAN	145 kDa		1	2
TOLLIP	sp|Q9H0E2	TOLIP_HUMAN	30 kDa		1	1
GIPC2	sp|Q8TF65	GIPC2_HUMAN	34 kDa		1	1
TSTD1	sp|Q8NFU3-4	TSTD1_HUMAN	13 kDa		1	1
HLA-DRB1	sp|P01911	2B1F_HUMAN	30 kDa	TRUE	1	1
TXNDC5	sp|Q8NBS9-2	TXND5_HUMAN	36 kDa		1	0
CDH6	sp|P55285	CADH6_HUMAN	88 kDa		1	3
RSU1	sp|Q15404-2	RSU1_HUMAN	26 kDa		1	1
DCTN2	sp|Q13561-2	DCTN2_HUMAN	45 kDa		1	0
DPP3	sp|Q9NY33-4	DPP3_HUMAN	79 kDa		1	2
EWSR1	sp|Q01844-3	EWS_HUMAN	68 kDa		1	0
RPL19	sp|P84098	RL19_HUMAN	23 kDa		1	2
SH3BGRL3	sp|Q9H299	SH3L3_HUMAN	10 kDa		1	1
DDX1	sp|Q92499	DDX1_HUMAN	82 kDa		1	1
OXCT1	sp|P55809	SCOT1_HUMAN	56 kDa		1	1
RPS15A	sp|P62244	RS15A_HUMAN	15 kDa	TRUE	1	0
COPB1	sp|P53618	COPB_HUMAN	107 kDa		1	0
PGM2	sp|Q96G03	PGM2_HUMAN	68 kDa		1	3
SH3BGRL	sp|O75368	SH3L1_HUMAN	13 kDa		1	1
TARDBP	sp|Q13148	TADBP_HUMAN	45 kDa		1	1
DBT	sp|P11182	ODB2_HUMAN	53 kDa		1	1
RPL29	sp|P47914	RL29_HUMAN	18 kDa		1	1
LGMN	sp|Q99538	LGMN_HUMAN	49 kDa		1	1
4	tr|F8W031	F8W031_HUMAN	29 kDa	TRUE	1	2
SNRPD3	sp|P62318	SMD3_HUMAN	14 kDa		1	1
MYO1C	sp|O00159-3	MYO1C_HUMAN	120 kDa		1	1
NDUFB9	sp|Q9Y6M9	NDUB9_HUMAN	22 kDa		1	2
MYLK	sp|Q15746-2	MYLK_HUMAN	203 kDa		1	1
QARS	sp|P47897	SYQ_HUMAN	88 kDa		1	1
PSMC2	sp|P35998	PRS7_HUMAN	49 kDa		1	1
SRSF2	sp|Q01130	SRSF2_HUMAN	25 kDa		1	2
HMOX1	sp|P09601	HMOX1_HUMAN	33 kDa		1	0
PSMC1	sp|P62191	PRS4_HUMAN	49 kDa		1	1
NUMA1	sp|Q14980	NUMA1_HUMAN	238 kDa		1	0
DSG1	sp|Q02413	DSG1_HUMAN	114 kDa		1	0
EIF2S1	sp|P05198	IF2A_HUMAN	36 kDa		1	1
CNP	sp|P09543-2	CN37_HUMAN	45 kDa		1	1

COA3	sp|Q9Y2R0	COA3_HUMAN	12 kDa		1	1
EIF4G1	sp|Q04637-5	IF4G1_HUMAN	159 kDa		1	1
AARS	sp|P49588	SYAC_HUMAN	107 kDa		1	1
FBLN1	sp|P23142-4	FBLN1_HUMAN	74 kDa	TRUE	1	2
TMEM205	sp|Q6UW68	TM205_HUMAN	21 kDa		1	1
ETHE1	sp|O95571	ETHE1_HUMAN	28 kDa		1	1
OTUB1	sp|Q96FW1	OTUB1_HUMAN	31 kDa		1	2
DDOST	sp|P39656	OST48_HUMAN	51 kDa		1	1
SEC31A	sp|O94979-8	SC31A_HUMAN	135 kDa		1	2
RPL8	sp|P62917	RL8_HUMAN	28 kDa		1	0
HP1BP3	sp|Q5SSJ5-2	HP1B3_HUMAN	57 kDa		1	1
ABCB1	tr|B5AK60	B5AK60_HUMAN	134 kDa		1	1
CSTA	sp|P01040	CYTA_HUMAN	11 kDa		1	0
FUCA1	sp|P04066	FUCO_HUMAN	54 kDa		1	1
RAC1	sp|P63000-2	RAC1_HUMAN	23 kDa		1	1
KPNB1	sp|Q14974	IMB1_HUMAN	97 kDa		1	0
CDC37	sp|Q16543	CDC37_HUMAN	44 kDa		1	0
DDAH2	sp|O95865	DDAH2_HUMAN	30 kDa	TRUE	1	0
TXNDC17	sp|Q9BRA2	TXD17_HUMAN	14 kDa		1	0
SCO1	sp|O75880	SCO1_HUMAN	34 kDa		1	1
FARP1	sp|Q9Y4F1	FARP1_HUMAN	119 kDa		1	1
RPS13	sp|P62277	RS13_HUMAN	17 kDa		1	0
LGALS3BP	sp|Q08380	LG3BP_HUMAN	65 kDa		1	2
RPL24	sp|P83731	RL24_HUMAN	18 kDa		1	1
ELAVL1	sp|Q15717	ELAV1_HUMAN	36 kDa		1	1
SHOX2	sp|O60902-2	SHOX2_HUMAN	34 kDa		1	2
DSTN	sp|P60981	DEST_HUMAN	19 kDa		1	1
CBX3	sp|Q13185	CBX3_HUMAN	21 kDa		1	0
DDRGK1	sp|Q96HY6-2	DDRGK_HUMAN	35 kDa		1	1
HLA-DRA	tr|Q30118	Q30118_HUMAN	26 kDa	TRUE	1	0
EEF1B2	sp|P24534	EF1B_HUMAN	25 kDa		1	0
HNRNPUL2	sp|Q1KMD3	HNRL2_HUMAN	85 kDa		1	1
LAMTOR1	sp|Q6IAA8	LTOR1_HUMAN	18 kDa		1	1
STIP1	sp|P31948	STIP1_HUMAN	63 kDa		1	1
S100A1	sp|P23297	S10A1_HUMAN	11 kDa		1	1
RPS24	sp|P62847-4	RS24_HUMAN	32 kDa		1	1
TMSB4X	sp|P62328	TYB4_HUMAN	5 kDa		1	1
UGT2A3	sp|Q6UWM9	UD2A3_HUMAN	60 kDa	TRUE	1	1
RPL27A	sp|P46776	RL27A_HUMAN	17 kDa		1	0
ERLIN2	sp|O94905	ERLN2_HUMAN	38 kDa		1	1
CTSA	sp|P10619	PPGB_HUMAN	54 kDa		1	1
TPT1	tr|Q5W0H4	Q5W0H4_HUMAN	22 kDa		1	0
NEDD8-MDP1	tr|S4R3E9	S4R3E9_HUMAN	10 kDa		1	2
TXNRD1	sp|Q16881-3	TRXR1_HUMAN	66 kDa		1	1
CD74	sp|P04233-2	HG2A_HUMAN	26 kDa		1	0
XPNPEP1	tr|G8JLB2	G8JLB2_HUMAN	72 kDa		1	1
FAM162A	sp|Q96A26	F162A_HUMAN	17 kDa		1	0

EPB41L1	sp|Q9H4G0-4	E41L1_HUMAN	79 kDa		1	1
FASN	sp|P49327	FAS_HUMAN	273 kDa		1	0
FLG2	sp|Q5D862	FILA2_HUMAN	248 kDa		1	0
KRT78	sp|Q8N1N4	K2C78_HUMAN	57 kDa	TRUE	1	0
ACSL3	sp|O95573	ACSL3_HUMAN	80 kDa		1	0
GLB1	sp|P16278-2	BGAL_HUMAN	61 kDa		1	1
MLEC	tr|F5GX14	F5GX14_HUMAN	24 kDa		1	1
PROSC	sp|O94903	PROSC_HUMAN	30 kDa		1	1
STAT1	sp|P42224	STAT1_HUMAN	87 kDa		1	0
GAA	sp|P10253	LYAG_HUMAN	105 kDa		1	4
ILK	sp|Q13418	ILK_HUMAN	51 kDa		1	0
ITGA6	sp|P23229-3	ITA6_HUMAN	122 kDa		1	1
AGTRAP	sp|Q6RW13	ATRAP_HUMAN	17 kDa		1	0
TRA2B	sp|P62995	TRA2B_HUMAN	34 kDa		1	0
UBQLN4	sp|Q9NRR5	UBQL4_HUMAN	64 kDa		1	1
GNS	sp|P15586	GNS_HUMAN	62 kDa		1	1
GFPT1	sp|Q06210-2	GFPT1_HUMAN	77 kDa		1	0
SCIN	sp|Q9Y6U3	ADSV_HUMAN	80 kDa		1	0
TOMM70A	sp|O94826	TOM70_HUMAN	67 kDa		1	1
CPSF6	tr|F8WJN3	F8WJN3_HUMAN	52 kDa		1	1
HSD17B12	sp|Q53GQ0	DHB12_HUMAN	34 kDa		1	0
DAZAP1	sp|Q96EP5-2	DAZP1_HUMAN	41 kDa		1	0
OLA1	tr|J3KQ32	J3KQ32_HUMAN	47 kDa	TRUE	1	0
NECAP2	sp|Q9NVZ3	NECP2_HUMAN	28 kDa		1	0
EIF3G	sp|O75821	EIF3G_HUMAN	36 kDa		1	1
MRPL1	tr|H0Y8N7	H0Y8N7_HUMAN	27 kDa		1	1
ASPN	sp|Q9BXN1	ASPN_HUMAN	43 kDa		1	0
TSTA3	sp|Q13630	FCL_HUMAN	36 kDa		1	1
SLC12A1	sp|Q13621-3	S12A1_HUMAN	121 kDa		1	1
FLOT2	tr|J3QLD9	J3QLD9_HUMAN	47 kDa		1	0
1	sp|P04207	KV308_HUMAN	14 kDa		1	1
IDH3G	sp|P51553	IDH3G_HUMAN	43 kDa		1	0
CCAR2	sp|Q8N163	CCAR2_HUMAN	103 kDa		1	1
LIMA1	sp|Q9UHB6-4	LIMA1_HUMAN	85 kDa		1	0
ARPC1B	sp|O15143	ARC1B_HUMAN	41 kDa	TRUE	1	0
FSCN1	sp|Q16658	FSCN1_HUMAN	55 kDa		1	1
PDCD5	sp|O14737	PDCD5_HUMAN	14 kDa		1	0
ARSE	sp|P51690	ARSE_HUMAN	66 kDa		1	2
LRBA	sp|P50851-2	LRBA_HUMAN	318 kDa	TRUE	1	0
MME	sp|P08473	NEP_HUMAN	86 kDa		0	8
DCXR	sp|Q7Z4W1	DCXR_HUMAN	26 kDa	TRUE	0	3
PCK1	sp|P35558	PCKGC_HUMAN	69 kDa	TRUE	0	9
EPB41L3	sp|Q9Y2J2-2	E41L3_HUMAN	97 kDa	TRUE	0	3
PRODH2	sp|Q9UF12	PROD2_HUMAN	59 kDa		0	5
DSP	sp|P15924	DESP_HUMAN	332 kDa		0	0
ACOX1	sp|Q15067-2	ACOX1_HUMAN	75 kDa	TRUE	0	5
SARDH	sp|Q9UL12	SARDH_HUMAN	101 kDa		0	0

PKLR	sp|P30613-2	KPYR_HUMAN	58 kDa	TRUE	0	2
GLDC	sp|P23378	GCSP_HUMAN	113 kDa		0	0
APOA1	sp|P02647	APOA1_HUMAN	31 kDa		0	1
CRYM	sp|Q14894	CRYM_HUMAN	34 kDa		0	8
FABP1	sp|P07148	FABPL_HUMAN	14 kDa		0	5
ALDH5A1	sp|P51649-2	SSDH_HUMAN	59 kDa		0	2
APOA4	sp|P06727	APOA4_HUMAN	45 kDa		0	0
SLC27A2	sp|O14975	S27A2_HUMAN	70 kDa		0	6
SLC25A10	tr|B4DLN1	B4DLN1_HUMAN	48 kDa	TRUE	0	1
CES2	sp|O00748	EST2_HUMAN	62 kDa		0	4
HPD	sp|P32754-2	HPPD_HUMAN	40 kDa		0	0
GCDH	sp|Q92947	GCDH_HUMAN	48 kDa	TRUE	0	1
HSDL2	sp|Q6YN16	HSDL2_HUMAN	45 kDa		0	5
AOC1	sp|P19801-2	AOC1_HUMAN	87 kDa		0	1
HMGCL	sp|P35914	HMGCL_HUMAN	34 kDa		0	1
NQO2	sp|P16083	NQO2_HUMAN	26 kDa		0	1
NDUFS2	sp|O75306	NDUS2_HUMAN	53 kDa		0	2
HYOU1	sp|Q9Y4L1	HYOU1_HUMAN	111 kDa		0	3
AK3	sp|Q9UIJ7	KAD3_HUMAN	26 kDa		0	4
PNPO	sp|Q9NVS9	PNPO_HUMAN	30 kDa		0	1
ATP1B1	sp|P05026-2	AT1B1_HUMAN	35 kDa		0	2
ASPDH	sp|A6ND91	ASPD_HUMAN	30 kDa		0	0
ANK3	sp|Q12955	ANK3_HUMAN	480 kDa	TRUE	0	4
MRPS36	sp|P82909	RT36_HUMAN	11 kDa		0	2
MUT	sp|P22033	MUTA_HUMAN	83 kDa		0	0
DHRS4	sp|Q9BTZ2	DHRS4_HUMAN	30 kDa		0	2
PPL	sp|O60437	PEPL_HUMAN	205 kDa		0	0
ASL	sp|P04424-2	ARLY_HUMAN	50 kDa		0	2
ILVBL	sp|A1L0T0	ILVBL_HUMAN	68 kDa		0	3
SDHB	sp|P21912	SDHB_HUMAN	32 kDa		0	1
CRNN	sp|Q9UBG3	CRNN_HUMAN	54 kDa		0	0
HINT2	sp|Q9BX68	HINT2_HUMAN	17 kDa		0	2
AGXT2	sp|Q9BYV1	AGT2_HUMAN	57 kDa		0	2
MYH11	sp|P35749-2	MYH11_HUMAN	228 kDa	TRUE	0	14
SLC5A12	sp|Q1EHB4	SC5AC_HUMAN	68 kDa		0	3
GGH	sp|Q92820	GGH_HUMAN	36 kDa		0	5
GALK1	sp|P51570	GALK1_HUMAN	42 kDa		0	3
IQGAP2	sp|Q13576	IQGA2_HUMAN	181 kDa	TRUE	0	2
CYP4A11	sp|Q02928	CP4AB_HUMAN	59 kDa		0	7
GK	sp|P32189-1	GLPK_HUMAN	57 kDa		0	3
XPNPEP2	sp|O43895	XPP2_HUMAN	76 kDa		0	1
GNPDA1	sp|P46926	GNPI1_HUMAN	33 kDa		0	1
TTC38	sp|Q5R3I4	TTC38_HUMAN	53 kDa		0	2
HAO2	sp|Q9NYQ3	HAOX2_HUMAN	39 kDa		0	1
PAH	sp|P00439	PH4H_HUMAN	52 kDa		0	3
EVPL	sp|Q92817	EVPL_HUMAN	232 kDa	TRUE	0	0
PDXK	sp|O00764	PDXK_HUMAN	35 kDa		0	3

AMN	sp|Q9BXJ7	AMNLS_HUMAN	48 kDa		0	4
OAT	sp|P04181	OAT_HUMAN	49 kDa		0	2
TF	sp|P02787	TRFE_HUMAN	77 kDa		0	0
KRT3	sp|P12035	K2C3_HUMAN	64 kDa	TRUE	0	0
PTER	sp|Q96BW5-2	PTER_HUMAN	33 kDa		0	0
SQRDL	sp|Q9Y6N5	SQRD_HUMAN	50 kDa		0	1
SCP2	sp|P22307-7	NLTP_HUMAN	54 kDa		0	0
ABHD10	sp|Q9NUJ1	ABHDA_HUMAN	34 kDa		0	1
SYNE1	sp|Q8NF91	SYNE1_HUMAN	1011 kDa		0	0
ACOT13	sp|Q9NPJ3-2	ACO13_HUMAN	12 kDa		0	1
PSMC4	sp|P43686-2	PRS6B_HUMAN	44 kDa		0	0
IGHG2	sp|P01859	IGHG2_HUMAN	36 kDa	TRUE	0	9
SEC23A	sp|Q15436	SC23A_HUMAN	86 kDa	TRUE	0	0
SLC3A1	sp|Q07837	SLC31_HUMAN	79 kDa		0	3
GSS	sp|P48637	GSHB_HUMAN	52 kDa		0	2
ACAD11	sp|Q709F0	ACD11_HUMAN	87 kDa		0	0
AUH	sp|Q13825	AUHM_HUMAN	36 kDa		0	2
ATP5L	sp|O75964	ATP5L_HUMAN	11 kDa		0	1
ST13	sp|P50502	F10A1_HUMAN	41 kDa		0	1
EHD1	sp|Q9H4M9	EHD1_HUMAN	61 kDa	TRUE	0	2
FAM151A	sp|Q8WW52	F151A_HUMAN	64 kDa	TRUE	0	0
JUP	sp|P14923	PLAK_HUMAN	82 kDa	TRUE	0	3
LDHD	sp|Q86WU2-2	LDHD_HUMAN	52 kDa		0	0
SNRPD1	sp|P62314	SMD1_HUMAN	13 kDa		0	2
SMIM24	sp|O75264	SIM24_HUMAN	15 kDa		0	3
GBE1	sp|Q04446	GLGB_HUMAN	80 kDa		0	2
ILF3	sp|Q12906-2	ILF3_HUMAN	76 kDa		0	2
NDUFA12	sp|Q9UI09	NDUAC_HUMAN	17 kDa		0	0
PLS1	sp|Q14651	PLSI_HUMAN	70 kDa	TRUE	0	3
HSD17B8	sp|Q92506	DHB8_HUMAN	27 kDa		0	2
NDUFA6	sp|P56556	NDUA6_HUMAN	18 kDa		0	1
FN3K	sp|Q9H479	FN3K_HUMAN	35 kDa		0	0
MGST1	sp|P10620	MGST1_HUMAN	18 kDa		0	0
FREM2	sp|Q5SZK8	FREM2_HUMAN	351 kDa		0	0
ATP6V1G1	sp|O75348	VATG1_HUMAN	14 kDa		0	1
CPT1A	sp|P50416	CPT1A_HUMAN	88 kDa		0	3
B2M	sp|P61769	B2MG_HUMAN	14 kDa		0	0
CFD	sp|P00746	CFAD_HUMAN	27 kDa		0	0
PSMB3	sp|P49720	PSB3_HUMAN	23 kDa		0	1
PSMB7	sp|Q99436	PSB7_HUMAN	30 kDa		0	1
NDUFA5	sp|Q16718	NDUA5_HUMAN	13 kDa		0	0
ATP6V1F	sp|Q16864-2	VATF_HUMAN	16 kDa		0	1
SCRN2	tr|J3QL71	J3QL71_HUMAN	47 kDa		0	1
POR	sp|P16435	NCPR_HUMAN	77 kDa		0	1
MAPT	sp|P10636-2	TAU_HUMAN	37 kDa	TRUE	0	2
A2M	sp|P01023	A2MG_HUMAN	163 kDa		0	0
GLRX	sp|P35754	GLRX1_HUMAN	12 kDa		0	1

GGACT	sp|Q9BVM4	GGACT_HUMAN	17 kDa		0	2
MGST3	sp|O14880	MGST3_HUMAN	17 kDa		0	1
IGHM	sp|P01871-2	IGHM_HUMAN	52 kDa		0	0
NDUFA4	sp|O00483	NDUA4_HUMAN	9 kDa		0	1
BCL2L13	sp|Q9BXK5	B2L13_HUMAN	53 kDa		0	1
HAGH	sp|Q16775	GLO2_HUMAN	34 kDa		0	1
NDUFB8	tr|Q5W145	Q5W145_HUMAN	18 kDa		0	0
ACSS1	sp|Q9NUB1-2	ACS2L_HUMAN	75 kDa		0	0
GFM1	sp|Q96RP9	EFGM_HUMAN	83 kDa		0	2
RAB2A	sp|P61019	RAB2A_HUMAN	24 kDa		0	2
DDT	sp|P30046	DOPD_HUMAN	13 kDa		0	1
ABCD3	tr|E7EUE1	E7EUE1_HUMAN	78 kDa		0	1
SPTBN2	sp|O15020	SPTN2_HUMAN	271 kDa	TRUE	0	0
COASY	sp|Q13057-2	COASY_HUMAN	65 kDa		0	0
CALML3	sp|P27482	CALL3_HUMAN	17 kDa	TRUE	0	0
MACF1	tr|H3BPE1	H3BPE1_HUMAN	857 kDa	TRUE	0	0
SERPINB3	sp|P29508	SPB3_HUMAN	45 kDa	TRUE	0	0
PSMB5	sp|P28074	PSB5_HUMAN	28 kDa		0	1
DCTN1	sp|Q14203-3	DCTN1_HUMAN	137 kDa		0	1
AZGP1	sp|P25311	ZA2G_HUMAN	34 kDa		0	2
SAR1B	sp|Q9Y6B6	SAR1B_HUMAN	22 kDa	TRUE	0	0
DHTKD1	sp|Q96HY7	DHTK1_HUMAN	103 kDa		0	0
AHNAK2	sp|Q8IVF2	AHNK2_HUMAN	617 kDa		0	0
BIN1	sp|O00499-3	BIN1_HUMAN	55 kDa		0	1
COPE	tr|M0QXB4	M0QXB4_HUMAN	37 kDa		0	1
ADIRF	sp|Q15847	ADIRF_HUMAN	8 kDa		0	1
ATP6V0C	sp|P27449	VATL_HUMAN	16 kDa		0	0
PTPLAD1	sp|Q9P035	HACD3_HUMAN	43 kDa		0	2
RNPEP	sp|Q9H4A4	AMPB_HUMAN	73 kDa		0	0
ACY3	sp|Q96HD9	ACY3_HUMAN	35 kDa		0	1
KTN1	sp|Q86UP2	KTN1_HUMAN	156 kDa		0	2
GM2A	sp|P17900	SAP3_HUMAN	21 kDa		0	1
F9	sp|P00740-2	FA9_HUMAN	48 kDa		0	2
PSMC6	sp|P62333	PRS10_HUMAN	44 kDa		0	1
DAO	sp|P14920	OXDA_HUMAN	39 kDa		0	0
SEPT9	sp|Q9UHD8	SEPT9_HUMAN	65 kDa	TRUE	0	0
MARS	sp|P56192	SYMC_HUMAN	101 kDa		0	0
SLC22A8	sp|Q8TCC7-2	S22A8_HUMAN	61 kDa		0	1
AP1M1	sp|Q9BXS5	AP1M1_HUMAN	49 kDa		0	2
BSG	sp|P35613	BASI_HUMAN	42 kDa		0	1
RALB	sp|P11234	RALB_HUMAN	23 kDa	TRUE	0	1
KMO	sp|O15229	KMO_HUMAN	56 kDa		0	0
LHPP	sp|Q9H008	LHPP_HUMAN	29 kDa		0	0
HDLBP	sp|Q00341-2	VIGLN_HUMAN	138 kDa		0	1
IBA57	sp|Q5T440	CAF17_HUMAN	38 kDa		0	0
GCHFR	sp|P30047	GFRP_HUMAN	10 kDa		0	0
CTSC	sp|P53634	CATC_HUMAN	52 kDa		0	3

HAAO	sp|P46952	3HAO_HUMAN	33 kDa		0	2
APOD	sp|P05090	APOD_HUMAN	21 kDa		0	1
A2ML1	sp|A8K2U0	A2ML1_HUMAN	161 kDa		0	0
GSTO1	sp|P78417-2	GSTO1_HUMAN	24 kDa		0	1
CDC42	sp|P60953	CDC42_HUMAN	21 kDa		0	1
DYNC1I2	sp|Q13409-3	DC1I2_HUMAN	68 kDa		0	2
RNASE2	sp|P10153	RNAS2_HUMAN	18 kDa		0	1
EPRS	sp|P07814	SYEP_HUMAN	171 kDa		0	2
SERPINF1	sp|P36955	PEDF_HUMAN	46 kDa		0	0
LARS	tr|B4DER1	B4DER1_HUMAN	131 kDa		0	0
NDUFB7	sp|P17568	NDUB7_HUMAN	16 kDa		0	0
DHX15	sp|O43143	DHX15_HUMAN	91 kDa	TRUE	0	0
IVL	sp|P07476	INVO_HUMAN	68 kDa		0	0
D2HGDH	sp|Q8N465	D2HDH_HUMAN	56 kDa		0	1
TPD52L2	sp|O43399-2	TPD54_HUMAN	20 kDa		0	0
ACE	sp|P12821	ACE_HUMAN	150 kDa		0	0
PRKDC	sp|P78527	PRKDC_HUMAN	469 kDa		0	2
COL5A2	sp|P05997	CO5A2_HUMAN	145 kDa		0	0
IGLC2	sp|P0CG05	LAC2_HUMAN	11 kDa	TRUE	0	2
FECH	sp|P22830-2	HEMH_HUMAN	49 kDa		0	1
POTEI	sp|P0CG38	POTEI_HUMAN	121 kDa	TRUE	0	12
CCDC170	sp|Q8IYT3	CC170_HUMAN	82 kDa		0	1
TUBB6	sp|Q9BUF5	TBB6_HUMAN	50 kDa	TRUE	0	0
COL3A1	sp|P02461	CO3A1_HUMAN	139 kDa		0	0
SERPINA5	sp|P05154	IPSP_HUMAN	46 kDa		0	0
RBM47	sp|A0AV96-2	RBM47_HUMAN	57 kDa		0	0
ALDH1A2	sp|O94788-3	AL1A2_HUMAN	55 kDa	TRUE	0	1
GLO1	sp|Q04760-2	LGUL_HUMAN	19 kDa		0	3
KIF5B	sp|P33176	KINH_HUMAN	110 kDa	TRUE	0	1
ACSM3	sp|Q53FZ2	ACSM3_HUMAN	66 kDa		0	0
KRT76	sp|Q01546	K22O_HUMAN	66 kDa	TRUE	0	0
SLC25A6	sp|P12236	ADT3_HUMAN	33 kDa	TRUE	0	6
PSMC5	sp|P62195-2	PRS8_HUMAN	45 kDa	TRUE	0	2
NDUFS6	sp|O75380	NDUS6_HUMAN	14 kDa		0	1
1	sp|P01620	KV302_HUMAN	12 kDa	TRUE	0	3
HSPA12A	sp|O43301	HS12A_HUMAN	75 kDa		0	0
PPA1	sp|Q15181	IPYR_HUMAN	33 kDa		0	0
TGM3	sp|Q08188	TGM3_HUMAN	77 kDa		0	0
USMG5	sp|Q96IX5	USMG5_HUMAN	6 kDa		0	1
FIS1	sp|Q9Y3D6	FIS1_HUMAN	17 kDa		0	2
CST3	sp|P01034	CYTC_HUMAN	16 kDa		0	0
GNG12	sp|Q9UBI6	GBG12_HUMAN	8 kDa		0	0
RPL10	sp|P27635	RL10_HUMAN	25 kDa		0	1
S100A11	sp|P31949	S10AB_HUMAN	12 kDa		0	2
IMPA2	sp|O14732-2	IMPA2_HUMAN	29 kDa		0	0
UBE2L3	sp|P68036-3	UB2L3_HUMAN	24 kDa		0	1
RARS	sp|P54136	SYRC_HUMAN	75 kDa		0	0

TUBB4A	sp|P04350	TBB4A_HUMAN	50 kDa	TRUE	0	28
EML4	sp|Q9HC35	EMAL4_HUMAN	109 kDa		0	1
SNX5	sp|Q9Y5X3	SNX5_HUMAN	47 kDa		0	0
SLC17A3	sp|O00476-2	NPT4_HUMAN	54 kDa		0	1
CLTB	sp|P09497-2	CLCB_HUMAN	23 kDa		0	1
VWA1	sp|Q6PCB0	VWA1_HUMAN	47 kDa		0	1
FKBP1A	sp|P62942	FKB1A_HUMAN	12 kDa		0	2
RPL27	sp|P61353	RL27_HUMAN	16 kDa		0	2
CAND1	sp|Q86VP6-2	CAND1_HUMAN	118 kDa		0	1
GNA11	sp|P29992	GNA11_HUMAN	42 kDa	TRUE	0	1
NDUFS4	sp|O43181	NDUS4_HUMAN	20 kDa		0	0
HLA-A	sp|P01891	1A68_HUMAN	41 kDa	TRUE	0	5
SPRYD4	sp|Q8WW59	SPRY4_HUMAN	23 kDa		0	1
HEBP1	sp|Q9NRV9	HEBP1_HUMAN	21 kDa		0	1
NDUFS7	sp|O75251	NDUS7_HUMAN	24 kDa		0	0
NT5C	sp|Q8TCD5	NT5C_HUMAN	23 kDa		0	0
ALDH3A1	sp|P30838	AL3A1_HUMAN	50 kDa	TRUE	0	0
FAM129B	sp|Q96TA1-2	NIBL1_HUMAN	83 kDa		0	0
HMGCS2	sp|P54868-3	HMCS2_HUMAN	50 kDa		0	12
SOD1	sp|P00441	SODC_HUMAN	16 kDa		0	1
CUTA	sp|O60888-2	CUTA_HUMAN	21 kDa		0	2
SEC24C	tr|E7EP00	E7EP00_HUMAN	107 kDa		0	1
PMPCA	sp|Q10713	MPPA_HUMAN	58 kDa		0	0
SLC13A3	sp|Q8WWT9-3	S13A3_HUMAN	58 kDa		0	0
ENTPD5	sp|O75356	ENTP5_HUMAN	48 kDa		0	2
KHDRBS1	sp|Q07666	KHDR1_HUMAN	48 kDa		0	0
PSMD12	sp|O00232	PSD12_HUMAN	53 kDa		0	0
RPL13A	tr|M0QYS1	M0QYS1_HUMAN	24 kDa		0	0
SEC61A1	sp|P61619	S61A1_HUMAN	52 kDa		0	0
WARS	sp|P23381	SYWC_HUMAN	53 kDa		0	0
HRG	sp|P04196	HRG_HUMAN	60 kDa		0	0
SFN	sp|P31947-2	1433S_HUMAN	24 kDa	TRUE	0	0
GBP6	sp|Q6ZN66	GBP6_HUMAN	72 kDa		0	0
ACSF3	sp|Q4G176	ACSF3_HUMAN	64 kDa		0	1
SDSL	sp|Q96GA7	SDSL_HUMAN	35 kDa		0	0
HTRA2	sp|O43464-3	HTRA2_HUMAN	46 kDa		0	1
GPHN	sp|Q9NQX3-2	GEPH_HUMAN	83 kDa		0	0
ACADL	sp|P28330	ACADL_HUMAN	48 kDa		0	0
SLC25A12	sp|O75746	CMC1_HUMAN	75 kDa	TRUE	0	3
CGNL1	sp|Q0VF96	CGNL1_HUMAN	149 kDa		0	1
GOLGB1	sp|Q14789-2	GOGB1_HUMAN	377 kDa	TRUE	0	0
AP1S1	tr|H7C1E4	H7C1E4_HUMAN	22 kDa		0	1
CPQ	sp|Q9Y646	CBPQ_HUMAN	52 kDa		0	3
AP3D1	sp|O14617-4	AP3D1_HUMAN	115 kDa		0	2
U2AF2	sp|P26368-2	U2AF2_HUMAN	53 kDa		0	1
DNAJA2	sp|O60884	DNJA2_HUMAN	46 kDa		0	2
CA12	sp|O43570-2	CAH12_HUMAN	38 kDa		0	0

SLC37A4	sp|O43826-2	G6PT1_HUMAN	49 kDa		0	0
HDAC6	sp|Q9UBN7	HDAC6_HUMAN	131 kDa		0	0
RPL15	sp|P61313	RL15_HUMAN	24 kDa		0	0
NDUFB6	sp|O95139	NDUB6_HUMAN	15 kDa		0	1
AOC3	sp|Q16853	AOC3_HUMAN	85 kDa		0	1
RAB6A	sp|P20340-2	RAB6A_HUMAN	24 kDa		0	1
PSMB6	sp|P28072	PSB6_HUMAN	25 kDa		0	0
FXYD2	sp|P54710	ATNG_HUMAN	7 kDa		0	0
MAPRE1	sp|Q15691	MARE1_HUMAN	30 kDa		0	1
NDUFB11	sp|Q9NX14-2	NDUBB_HUMAN	18 kDa		0	0
REEP6	sp|Q96HR9	REEP6_HUMAN	21 kDa		0	2
LACTB2	sp|Q53H82	LACB2_HUMAN	33 kDa		0	0
RPL14	sp|P50914	RL14_HUMAN	23 kDa		0	1
GRPEL1	sp|Q9HAV7	GRPE1_HUMAN	24 kDa		0	0
PCMT1	sp|P22061-2	PIMT_HUMAN	25 kDa		0	1
GARS	sp|P41250	SYG_HUMAN	83 kDa		0	0
PRELP	sp|P51888	PRELP_HUMAN	44 kDa		0	1
SUMF2	sp|Q8NBJ7	SUMF2_HUMAN	34 kDa		0	0
LGALS1	sp|P09382	LEG1_HUMAN	15 kDa		0	1
MT-ND4	sp|P03905	NU4M_HUMAN	52 kDa		0	0
MTHFS	sp|P49914-2	MTHFS_HUMAN	21 kDa		0	0
PMPCB	sp|O75439	MPPB_HUMAN	54 kDa	TRUE	0	1
LGALS3	sp|P17931	LEG3_HUMAN	26 kDa		0	1
CDHR2	sp|Q9BYE9	CDHR2_HUMAN	142 kDa		0	0
LRRC47	sp|Q8N1G4	LRC47_HUMAN	63 kDa		0	2
CHP1	sp|Q99653	CHP1_HUMAN	22 kDa		0	0
SERPINC1	sp|P01008	ANT3_HUMAN	53 kDa		0	1
SAR1A	sp|Q9NR31	SAR1A_HUMAN	22 kDa	TRUE	0	0
SNX1	sp|Q13596-2	SNX1_HUMAN	52 kDa	TRUE	0	0
AP1B1	sp|Q10567-2	AP1B1_HUMAN	104 kDa	TRUE	0	0
LYPLA1	sp|O75608-2	LYPA1_HUMAN	23 kDa		0	0
HRNR	sp|Q86YZ3	HORN_HUMAN	282 kDa		0	0
PKP1	sp|Q13835	PKP1_HUMAN	83 kDa		0	0
CLINT1	sp|Q14677-3	EPN4_HUMAN	70 kDa		0	1
C8orf82	tr|H0YF29	H0YF29_HUMAN	29 kDa		0	0
VAPA	sp|Q9P0L0-2	VAPA_HUMAN	33 kDa	TRUE	0	0
THY1	tr|J3QRJ3	J3QRJ3_HUMAN	16 kDa		0	2
HS1BP3	sp|Q53T59	H1BP3_HUMAN	43 kDa		0	2
SSB	sp|P05455	LA_HUMAN	47 kDa		0	1
AP2S1	tr|M0QYZ2	M0QYZ2_HUMAN	19 kDa		0	1
PDCD6	sp|O75340-2	PDCD6_HUMAN	22 kDa		0	1
PPP1R7	sp|Q15435	PP1R7_HUMAN	42 kDa		0	1
CDH2	sp|P19022	CADH2_HUMAN	100 kDa		0	0
PAFAH1B2	sp|P68402	PA1B2_HUMAN	26 kDa		0	0
EIF4B	sp|P23588	IF4B_HUMAN	69 kDa		0	0
BAX	sp|Q07812-5	BAX_HUMAN	18 kDa		0	2
SF3B1	sp|O75533	SF3B1_HUMAN	146 kDa		0	0

SERPINB1	sp|P30740	ILEU_HUMAN	43 kDa		0	0
ATAD2	sp|Q6PL18	ATAD2_HUMAN	159 kDa		0	0
FSIP2	sp|Q5CZC0	FSIP2_HUMAN	781 kDa		0	0
COL11A2	sp|P13942-2	COBA2_HUMAN	169 kDa		0	1
SCEL	sp|O95171-2	SCEL_HUMAN	75 kDa		0	0
NDUFS8	tr|E9PN51	E9PN51_HUMAN	12 kDa		0	0
SUSD2	sp|Q9UGT4	SUSD2_HUMAN	90 kDa		0	0
G3BP1	sp|Q13283	G3BP1_HUMAN	52 kDa		0	0
GAMT	sp|Q14353	GAMT_HUMAN	26 kDa		0	0
COX6B1	sp|P14854	CX6B1_HUMAN	10 kDa		0	1
MRPS31	sp|Q92665	RT31_HUMAN	45 kDa		0	0
TMEM14C	sp|Q9P0S9	TM14C_HUMAN	12 kDa		0	0
OCIAD1	sp|Q9NX40	OCAD1_HUMAN	28 kDa		0	0
NDUFA3	sp|O95167	NDUA3_HUMAN	9 kDa		0	0
SLC22A6	sp|Q4U2R8-2	S22A6_HUMAN	60 kDa		0	1
C5	sp|P01031	CO5_HUMAN	188 kDa		0	0
VDAC3	sp|Q9Y277-2	VDAC3_HUMAN	31 kDa	TRUE	0	1
CFH	sp|P08603	CFAH_HUMAN	139 kDa		0	0
ASNA1	sp|O43681	ASNA_HUMAN	39 kDa		0	1
CLPP	sp|Q16740	CLPP_HUMAN	30 kDa		0	0
CPNE1	sp|Q99829	CPNE1_HUMAN	59 kDa		0	1
PDZD11	sp|Q5EBL8-2	PDZ11_HUMAN	19 kDa		0	1
UFM1	sp|P61960	UFM1_HUMAN	9 kDa		0	0
FARSA	sp|Q9Y285	SYFA_HUMAN	58 kDa		0	1
PA2G4	sp|Q9UQ80	PA2G4_HUMAN	44 kDa		0	0
ORM1	sp|P02763	A1AG1_HUMAN	24 kDa	TRUE	0	2
AMT	sp|P48728-3	GCST_HUMAN	39 kDa		0	0
KRT15	sp|P19012	K1C15_HUMAN	49 kDa	TRUE	0	0
MAPK1	sp|P28482-2	MK01_HUMAN	36 kDa	TRUE	0	0
SLC36A2	sp|Q495M3	S36A2_HUMAN	53 kDa		0	0
L2HGDH	sp|Q9H9P8	L2HDH_HUMAN	50 kDa		0	0
HNRNPDL	sp|O14979-2	HNRDL_HUMAN	34 kDa	TRUE	0	0
NDUFA7	sp|O95182	NDUA7_HUMAN	13 kDa		0	0
SLC26A1	sp|Q9H2B4	S26A1_HUMAN	75 kDa		0	0
SUGCT	sp|Q9HAC7-2	SUCHY_HUMAN	47 kDa		0	0
OPA1	sp|O60313-2	OPA1_HUMAN	116 kDa		0	0
IGHG4	sp|P01861	IGHG4_HUMAN	36 kDa	TRUE	0	4
METTL7B	sp|Q6UX53	MET7B_HUMAN	28 kDa		0	0
SCCPDH	sp|Q8NBX0	SCPDL_HUMAN	47 kDa		0	0
EIF4A2	sp|Q14240-2	IF4A2_HUMAN	46 kDa	TRUE	0	0
VWA8	sp|A3KMH1	VWA8_HUMAN	215 kDa		0	0
HEXB	sp|P07686	HEXB_HUMAN	63 kDa		0	2
HKDC1	sp|Q2TB90	HKDC1_HUMAN	103 kDa	TRUE	0	0
DSC2	sp|Q02487-2	DSC2_HUMAN	94 kDa	TRUE	0	0
APOB	sp|P04114	APOB_HUMAN	516 kDa		0	0
REEP5	sp|Q00765	REEP5_HUMAN	21 kDa		0	0
MT-ND5	sp|P03915	NU5M_HUMAN	67 kDa		0	0

PTMA	tr|H7C2N1	H7C2N1_HUMAN	16 kDa		0	0
AP2A1	sp|O95782-2	AP2A1_HUMAN	105 kDa	TRUE	0	1
AGT	sp|P01019	ANGT_HUMAN	53 kDa		0	0
SNRPB	sp|P14678-2	RSMB_HUMAN	24 kDa		0	0
SNRPA	sp|P09012	SNRPA_HUMAN	31 kDa	TRUE	0	0
SF3B2	sp|Q13435	SF3B2_HUMAN	100 kDa		0	0
SPTB	sp|P11277-2	SPTB1_HUMAN	268 kDa	TRUE	0	0
SBSN	sp|Q6UWP8	SBSN_HUMAN	61 kDa		0	0
CALML5	sp|Q9NZT1	CALL5_HUMAN	16 kDa		0	0
SERPINB5	sp|P36952	SPB5_HUMAN	42 kDa		0	0
PPM1F	sp|P49593	PPM1F_HUMAN	50 kDa		0	1
PSMB2	sp|P49721	PSB2_HUMAN	23 kDa		0	0
TMEM27	sp|Q9HBJ8	TMM27_HUMAN	25 kDa		0	0
MTCH1	sp|Q9NZJ7-2	MTCH1_HUMAN	40 kDa		0	1
ARL8B	sp|Q9NVJ2	ARL8B_HUMAN	22 kDa		0	1
PALM3	sp|A6NDB9	PALM3_HUMAN	72 kDa		0	0
CPVL	sp|Q9H3G5	CPVL_HUMAN	54 kDa		0	3
SLC7A8	sp|Q9UHI5	LAT2_HUMAN	58 kDa		0	0
TFG	sp|Q92734-2	TFG_HUMAN	43 kDa		0	1
ACAD10	sp|Q6JQN1-5	ACD10_HUMAN	122 kDa		0	0
XYLB	sp|O75191	XYLB_HUMAN	58 kDa		0	0
BPNT1	sp|O95861	BPNT1_HUMAN	33 kDa		0	0
CREB3L1	sp|Q96BA8	CR3L1_HUMAN	57 kDa		0	0
SPRR3	sp|Q9UBC9	SPRR3_HUMAN	18 kDa		0	0
LAMP2	sp|P13473-2	LAMP2_HUMAN	45 kDa		0	0
ARF5	sp|P84085	ARF5_HUMAN	21 kDa	TRUE	0	0
SF3B3	sp|Q15393	SF3B3_HUMAN	136 kDa	TRUE	0	0
NSF	sp|P46459	NSF_HUMAN	83 kDa		0	1
RAB11FIP5	sp|Q9BXF6	RFIP5_HUMAN	70 kDa		0	0
ACE2	sp|Q9BYF1	ACE2_HUMAN	92 kDa		0	0
SDC1	sp|P18827	SDC1_HUMAN	32 kDa		0	0
GC	tr|D6RF35	D6RF35_HUMAN	53 kDa		0	0
NPLOC4	sp|Q8TAT6-2	NPL4_HUMAN	69 kDa		0	1
LRRFIP1	sp|Q32MZ4-2	LRRF1_HUMAN	86 kDa	TRUE	0	0
SHPK	sp|Q9UHJ6	SHPK_HUMAN	51 kDa		0	1
CLPTM1	tr|F5H8J3	F5H8J3_HUMAN	75 kDa		0	1
PLIN3	sp|O60664-3	PLIN3_HUMAN	47 kDa		0	1
NAPA	sp|P54920	SNAA_HUMAN	33 kDa		0	0
SELO	sp|Q9BVL4	SELO_HUMAN	73 kDa		0	0
COL6A5	sp|A8TX70	CO6A5_HUMAN	290 kDa		0	0
PFKP	sp|Q01813	K6PP_HUMAN	86 kDa	TRUE	0	0
TPR	sp|P12270	TPR_HUMAN	267 kDa		0	0
SDHC	sp|Q99643	C560_HUMAN	19 kDa		0	0
NAMPT	sp|P43490	NAMPT_HUMAN	56 kDa		0	0
DENR	sp|O43583	DENR_HUMAN	22 kDa		0	0
SARS2	sp|Q9NP81	SYSM_HUMAN	58 kDa		0	0
PPP2R2A	sp|P63151-2	2ABA_HUMAN	53 kDa		0	0

GHDC	sp|Q8N2G8	GHDC_HUMAN	58 kDa		0	1
NDUFS5	sp|O43920	NDUS5_HUMAN	13 kDa		0	0
GALE	sp|Q14376	GALE_HUMAN	38 kDa		0	0
C2orf18	tr|B4DLH2	B4DLH2_HUMAN	31 kDa		0	0
BDH1	sp|Q02338	BDH_HUMAN	38 kDa		0	1
CPNE3	sp|O75131	CPNE3_HUMAN	60 kDa	TRUE	0	0
RPL28	tr|H0YKD8	H0YKD8_HUMAN	19 kDa		0	0
NAP1L4	sp|Q99733-2	NP1L4_HUMAN	44 kDa		0	0
SNRPD2	sp|P62316	SMD2_HUMAN	14 kDa		0	0
CP	sp|P00450	CERU_HUMAN	122 kDa		0	1
CFB	sp|P00751	CFAB_HUMAN	86 kDa		0	0
ADSSL1	sp|Q8N142	PURA1_HUMAN	50 kDa		0	0
GPT	sp|P24298	ALAT1_HUMAN	55 kDa		0	0
UPB1	sp|Q9UBR1	BUP1_HUMAN	43 kDa		0	0
TJP1	tr|G3V1L9	G3V1L9_HUMAN	197 kDa		0	0
NUDT12	sp|Q9BQG2	NUD12_HUMAN	52 kDa		0	0
FHIT	sp|P49789	FHIT_HUMAN	17 kDa		0	0
SNX12	sp|Q9UMY4-2	SNX12_HUMAN	19 kDa	TRUE	0	0
PPP1R12A	sp|O14974	MYPT1_HUMAN	115 kDa		0	0
SLC22A2	sp|O15244	S22A2_HUMAN	63 kDa		0	0
NPL	sp|Q9BXD5	NPL_HUMAN	35 kDa		0	1
PSPC1	sp|Q8WXF1	PSPC1_HUMAN	59 kDa		0	0
RPL21	sp|P46778	RL21_HUMAN	19 kDa		0	0
DDX6	sp|P26196	DDX6_HUMAN	54 kDa		0	0
SPP1	sp|P10451-2	OSTP_HUMAN	34 kDa		0	0
SLC34A2	sp|O95436-2	NPT2B_HUMAN	76 kDa		0	0
SAA1	sp|P0DJI8	SAA1_HUMAN	14 kDa	TRUE	0	0
EIF3F	sp|O00303	EIF3F_HUMAN	38 kDa		0	0
ATP5E	sp|P56381	ATP5E_HUMAN	6 kDa		0	0
HEATR5B	sp|Q9P2D3	HTR5B_HUMAN	224 kDa		0	0
PTX3	sp|P26022	PTX3_HUMAN	42 kDa		0	0
KRT85	sp|P78386	KRT85_HUMAN	56 kDa	TRUE	0	0
TRIM29	sp|Q14134-2	TRI29_HUMAN	64 kDa	TRUE	0	0
DSG3	sp|P32926	DSG3_HUMAN	108 kDa		0	0
ISOC1	sp|Q96CN7	ISOC1_HUMAN	32 kDa		0	0
UBE2K	sp|P61086-3	UBE2K_HUMAN	17 kDa		0	0
UCHL1	sp|P09936	UCHL1_HUMAN	25 kDa		0	1
SAMM50	sp|Q9Y512	SAM50_HUMAN	52 kDa		0	0
PUF60	sp|Q9UHX1-6	PUF60_HUMAN	55 kDa		0	0
DACT1	sp|Q9NYF0-2	DACT1_HUMAN	86 kDa		0	0
RAB21	sp|Q9UL25	RAB21_HUMAN	24 kDa		0	0
HSPB6	sp|O14558	HSPB6_HUMAN	17 kDa		0	2
CKMT1A	sp|P12532-2	KCRU_HUMAN	50 kDa		0	0
ATPIF1	sp|Q9UII2	ATIF1_HUMAN	12 kDa		0	0
DNPEP	tr|E7ETB3	E7ETB3_HUMAN	55 kDa		0	1
USH1C	sp|Q9Y6N9-2	USH1C_HUMAN	58 kDa		0	0
CDH1	sp|P12830	CADH1_HUMAN	97 kDa		0	0

ALDH18A1	sp|P54886-2	P5CS_HUMAN	87 kDa		0	0
CDHR5	sp|Q9HBB8-4	CDHR5_HUMAN	88 kDa		0	0
TXNL1	tr|K7ER96	K7ER96_HUMAN	31 kDa		0	0
CAST	sp|P20810-5	ICAL_HUMAN	81 kDa	TRUE	0	0
RAD23B	sp|P54727	RD23B_HUMAN	43 kDa	TRUE	0	0
MACROD1	sp|Q9BQ69	MACD1_HUMAN	36 kDa		0	0
SLC22A12	sp|Q96S37-3	S22AC_HUMAN	36 kDa		0	0
1	sp|P04433	KV309_HUMAN	13 kDa		0	1
CYP4F3	sp|Q08477-2	CP4F3_HUMAN	60 kDa	TRUE	0	1
PPP2R4	sp|Q15257-2	PTPA_HUMAN	37 kDa		0	0
RBM14	sp|Q96PK6	RBM14_HUMAN	69 kDa		0	1
SARS	sp|P49591	SYSC_HUMAN	59 kDa		0	2
PSMD7	sp|P51665	PSMD7_HUMAN	37 kDa		0	1
PPP1CA	sp|P62136	PP1A_HUMAN	38 kDa		0	1
MLYCD	sp|O95822	DCMC_HUMAN	55 kDa		0	0
C1QB	sp|P02746	C1QB_HUMAN	27 kDa		0	0
PAWR	sp|Q96IZ0	PAWR_HUMAN	37 kDa		0	0
RPL35A	sp|P18077	RL35A_HUMAN	13 kDa		0	0
VASP	sp|P50552	VASP_HUMAN	40 kDa		0	0
SURF4	sp|O15260-2	SURF4_HUMAN	18 kDa		0	0
EIF3S3	tr|B3KS98	B3KS98_HUMAN	42 kDa		0	0
RGN	sp|Q15493	RGN_HUMAN	33 kDa		0	0
FERMT3	sp|Q86UX7-2	URP2_HUMAN	75 kDa		0	0
MYH14	sp|Q7Z406-2	MYH14_HUMAN	232 kDa	TRUE	0	0
HYDIN	sp|Q4G0P3	HYDIN_HUMAN	576 kDa		0	0
SERPINB4	sp|P48594	SPB4_HUMAN	45 kDa	TRUE	0	0
IGJ	sp|P01591	IGJ_HUMAN	18 kDa		0	0
PSMD1	sp|Q99460	PSMD1_HUMAN	106 kDa		0	0
TJP2	sp|Q9UDY2-7	ZO2_HUMAN	137 kDa		0	0
ENOSF1	sp|Q7L5Y1-2	ENOF1_HUMAN	41 kDa		0	1
LBP	sp|P18428	LBP_HUMAN	53 kDa		0	0
HPX	sp|P02790	HEMO_HUMAN	52 kDa		0	0
SPTBN1	sp|Q01082-3	SPTB2_HUMAN	251 kDa	TRUE	0	0
AKR1C1	sp|Q04828	AK1C1_HUMAN	37 kDa	TRUE	0	0
OPLAH	sp|O14841	OPLA_HUMAN	137 kDa		0	0
RNF213	sp|Q63HN8-4	RN213_HUMAN	596 kDa		0	0
CASP14	sp|P31944	CASPE_HUMAN	28 kDa		0	0
FLG	sp|P20930	FILA_HUMAN	435 kDa		0	0
FDXR	sp|P22570-5	ADRO_HUMAN	53 kDa		0	0
LGALS7	sp|P47929	LEG7_HUMAN	15 kDa		0	0
CHTOP	sp|Q9Y3Y2-3	CHTOP_HUMAN	27 kDa		0	1
LRPAP1	sp|P30533	AMRP_HUMAN	41 kDa		0	1
GPRC5C	tr|A8MXZ4	A8MXZ4_HUMAN	50 kDa		0	0
DYNC1LI2	sp|O43237	DC1L2_HUMAN	54 kDa		0	0
PTPN1	sp|P18031	PTN1_HUMAN	50 kDa		0	0
MRPL28	sp|Q13084	RM28_HUMAN	30 kDa		0	0
STT3A	tr|E9PNQ1	E9PNQ1_HUMAN	70 kDa		0	0

COBLL1	tr|G8JL86	G8JL86_HUMAN	135 kDa		0	0
CYP17A1	sp|P05093	CP17A_HUMAN	57 kDa		0	0
SORBS3	sp|O60504	VINEX_HUMAN	75 kDa		0	3
EPX	sp|P11678	PERE_HUMAN	81 kDa	TRUE	0	0
GPD2	sp|P43304	GPDM_HUMAN	81 kDa		0	0
TGM1	sp|P22735	TGM1_HUMAN	90 kDa		0	0
1	sp|P80748	LV302_HUMAN	12 kDa		0	2
MOGS	sp|Q13724	MOGS_HUMAN	92 kDa		0	0
OXSR1	sp|O95747	OXSR1_HUMAN	58 kDa		0	0
TWF1	sp|Q12792	TWF1_HUMAN	40 kDa		0	0
GALT	sp|P07902	GALT_HUMAN	43 kDa		0	2
SCRN1	sp|Q12765-2	SCRN1_HUMAN	49 kDa		0	1
THEM6	sp|Q8WUY1	THEM6_HUMAN	24 kDa		0	0
NDUFAF3	sp|Q9BU61	NDUF3_HUMAN	20 kDa		0	0
UCHL3	sp|P15374	UCHL3_HUMAN	26 kDa		0	0
SACM1L	tr|B4DK71	B4DK71_HUMAN	61 kDa		0	0
PTGES2	sp|Q9H7Z7	PGES2_HUMAN	42 kDa		0	0
NAGLU	sp|P54802	ANAG_HUMAN	82 kDa		0	0
ADAR	sp|P55265-4	DSRAD_HUMAN	141 kDa		0	0
NCEH1	sp|Q6PIU2	NCEH1_HUMAN	46 kDa		0	0
MARCKS	sp|P29966	MARCS_HUMAN	32 kDa		0	0
LRP1	sp|Q07954	LRP1_HUMAN	505 kDa		0	0
NEK9	sp|Q8TD19	NEK9_HUMAN	107 kDa		0	0
KRT31	sp|Q15323	K1H1_HUMAN	47 kDa	TRUE	0	0
IL1RN	sp|P18510	IL1RA_HUMAN	20 kDa		0	0
DSC3	sp|Q14574-2	DSC3_HUMAN	93 kDa	TRUE	0	0
TOMM22	sp|Q9NS69	TOM22_HUMAN	16 kDa		0	1
PFKFB2	sp|O60825	F262_HUMAN	58 kDa		0	0
GUSB	sp|P08236-2	BGLR_HUMAN	69 kDa		0	2
DUSP23	sp|Q9BVJ7	DUS23_HUMAN	17 kDa		0	1
APOOL	sp|Q6UXV4	APOOL_HUMAN	29 kDa		0	0
IMPDH2	sp|P12268	IMDH2_HUMAN	56 kDa		0	0
IYD	sp|Q6PHW0-3	IYD1_HUMAN	29 kDa		0	0
APCS	sp|P02743	SAMP_HUMAN	25 kDa		0	2
NUDT21	sp|O43809	CPSF5_HUMAN	26 kDa		0	0
CLPX	sp|O76031	CLPX_HUMAN	69 kDa		0	0
DNAJB1	sp|P25685	DNJB1_HUMAN	38 kDa		0	0
MX1	sp|P20591-2	MX1_HUMAN	56 kDa		0	0
ZC3HAV1	sp|Q7Z2W4-3	ZCCHV_HUMAN	68 kDa		0	0
EIF3D	sp|O15371	EIF3D_HUMAN	64 kDa		0	0
KRT6A	sp|P02538	K2C6A_HUMAN	60 kDa	TRUE	0	0
SAOUHSC_02581	tr|Q2FVW0	Q2FVW0_STAA8	17 kDa		0	0
HEXA	sp|P06865	HEXA_HUMAN	61 kDa		0	3
PKP3	sp|Q9Y446-2	PKP3_HUMAN	89 kDa		0	0
POF1B	sp|Q8WVV4-1	POF1B_HUMAN	69 kDa		0	0
1	sp|P01593	KV101_HUMAN	12 kDa		0	1
MT-ND2	sp|P03891	NU2M_HUMAN	39 kDa		0	0

HN1L	tr|H3BMV3	H3BMV3_HUMAN	21 kDa		0	0
MFF	sp|Q9GZY8-3	MFF_HUMAN	28 kDa		0	0
ATP6V1C1	sp|P21283	VATC1_HUMAN	44 kDa		0	0
SAFB	sp|Q15424-2	SAFB1_HUMAN	95 kDa	TRUE	0	0
GHITM	sp|Q9H3K2	GHITM_HUMAN	37 kDa		0	0
PDE12	sp|Q6L8Q7-2	PDE12_HUMAN	59 kDa		0	0
G6PD	sp|P11413-3	G6PD_HUMAN	62 kDa		0	0
CPNE6	tr|F5GXN1	F5GXN1_HUMAN	68 kDa	TRUE	0	0
FABP5	sp|Q01469	FABP5_HUMAN	15 kDa		0	0
SRP72	sp|O76094	SRP72_HUMAN	75 kDa		0	0
NCCRP1	sp|Q6ZVX7	FBX50_HUMAN	31 kDa		0	0
CACNG8	sp|Q8WXS5	CCG8_HUMAN	43 kDa		0	0
ZNF185	sp|O15231-7	ZN185_HUMAN	70 kDa		0	0
AMY1A	sp|P04745	AMY1_HUMAN	58 kDa		0	0
RPTN	sp|Q6XPR3	RPTN_HUMAN	91 kDa		0	0
ATL3	tr|F5GWF8	F5GWF8_HUMAN	18 kDa		0	0
C8B	tr|F5GY80	F5GY80_HUMAN	60 kDa		0	0
CBR3	sp|O75828	CBR3_HUMAN	31 kDa	TRUE	0	0
KRT36	sp|O76013-2	KRT36_HUMAN	47 kDa	TRUE	0	0
TFRC	sp|P02786	TFR1_HUMAN	85 kDa		0	0
SERPINB13	sp|Q9UIV8-2	SPB13_HUMAN	38 kDa		0	0
ADH7	sp|P40394-2	ADH7_HUMAN	42 kDa		0	0
SOD3	sp|P08294	SODE_HUMAN	26 kDa		0	2
CD44	sp|P16070-12	CD44_HUMAN	39 kDa		0	0
SCAMP1	sp|O15126	SCAM1_HUMAN	38 kDa		0	0
HLA-A	sp|P10316	1A69_HUMAN	41 kDa	TRUE	0	0
ORM2	sp|P19652	A1AG2_HUMAN	24 kDa	TRUE	0	0
INSC	sp|Q1MX18-6	INSC_HUMAN	57 kDa		0	0
ITGB3	tr|B4DTY9	B4DTY9_HUMAN	84 kDa		0	0
ME3	sp|Q16798	MAON_HUMAN	67 kDa		0	0
ITIH4	sp|Q14624-2	ITIH4_HUMAN	101 kDa		0	0
PRMT1	sp|Q99873-2	ANM1_HUMAN	40 kDa		0	0
RANGAP1	tr|F8W7I9	F8W7I9_HUMAN	58 kDa		0	0
SULT1A1	sp|P50225	ST1A1_HUMAN	34 kDa		0	0
ANXA8	sp|P13928	ANXA8_HUMAN	37 kDa		0	0
SERPINB12	tr|Q3SYB4	Q3SYB4_HUMAN	48 kDa		0	0
KLK10	sp|O43240	KLK10_HUMAN	30 kDa		0	0
LRG1	sp|P02750	A2GL_HUMAN	38 kDa		0	0
CA4	sp|P22748	CAH4_HUMAN	35 kDa		0	0
NQO1	sp|P15559-2	NQO1_HUMAN	27 kDa		0	0
PDXDC1	sp|Q6P996	PDXD1_HUMAN	87 kDa		0	0
HLA-B	sp|Q04826	1B40_HUMAN	41 kDa	TRUE	0	0
CA6	sp|P23280-2	CAH6_HUMAN	35 kDa		0	0
KLK7	sp|P49862-2	KLK7_HUMAN	20 kDa		0	0
CST4	sp|P01036	CYTS_HUMAN	16 kDa		0	0
KLK12	sp|Q9UKR0-2	KLK12_HUMAN	27 kDa		0	0
CA3	sp|P07451	CAH3_HUMAN	30 kDa		0	0

VPS4B	sp|O75351	VPS4B_HUMAN	49 kDa	TRUE	0	0
AKR1B10	sp|O60218	AK1BA_HUMAN	36 kDa		0	0
PIP	sp|P12273	PIP_HUMAN	17 kDa		0	0
PI3	sp|P19957	ELAF_HUMAN	12 kDa		0	0
VSIG10L	sp|Q86VR7-2	VS10L_HUMAN	93 kDa		0	0
MUC13	sp|Q9H3R2	MUC13_HUMAN	55 kDa		0	0
KLK14	sp|Q9P0G3	KLK14_HUMAN	29 kDa		0	0
DYNLL1	sp|P63167	DYL1_HUMAN	10 kDa		0	0
ARID3C	sp|A6NKF2	ARI3C_HUMAN	44 kDa		0	0
NUDT19	sp|A8MXV4	NUD19_HUMAN	42 kDa		0	2

Total Spect SAGN T11	Total Spect SAGN T12	Total Spect IgAN E0C1 T4	Total Spect IgAN E0C0 T8	Total Spect IgAN E0C0 T9	Total Spect IgAN E0C0 T10	Total Spect Normal kid T5	Total Spect Normal kid T6	Total Spect Normal kid T13
107	64	67	46	61	48	48	50	75
93	18	52	18	53	39	28	23	44
58	4	23	18	56	31	14	15	33
50	15	32	23	35	23	19	17	34
59	34	45	48	34	45	39	34	44
19	19	33	9	8	5	34	36	6
48	39	45	41	52	41	49	38	46
15	166	22	9	12	29	39	28	11
54	34	56	54	45	49	47	51	58
8	37	15	6	8	37	31	15	8
53	8	42	48	52	48	26	30	49
47	24	34	34	39	31	34	23	38
37	29	27	20	27	24	23	25	29
40	33	36	43	37	28	28	39	39
39	12	31	31	26	21	22	27	34
29	28	46	46	47	40	40	50	48
35	23	38	39	30	32	29	28	33
14	127	23	15	21	44	38	27	15
37	20	24	36	30	31	26	23	30
41	9	20	21	15	14	16	20	23
40	23	31	41	35	33	28	27	35
30	26	43	55	44	37	43	47	49
28	14	26	17	21	15	16	20	23
36	15	49	43	39	34	46	43	45
36	11	49	47	32	35	36	38	42
19	6	15	9	15	12	8	9	13
35	31	24	19	22	10	21	19	23
21	10	18	4	15	10	14	10	11
28	27	22	25	24	27	21	22	33
33	13	0	17	24	8	9	9	7
27	4	8	2	7	7	0	0	7
22	10	17	9	19	11	7	8	15
3	68	5	2	3	10	16	13	2
30	17	20	28	23	24	18	18	25
36	37	40	41	39	27	31	37	39
31	17	25	31	21	15	28	21	22
31	30	34	18	19	19	23	27	28
18	15	23	20	22	21	15	20	21
20	0	15	10	15	13	10	9	12
24	19	37	45	37	44	37	41	39
23	4	11	9	11	9	6	3	11
20	7	39	35	30	27	27	34	36
23	6	13	5	21	10	11	9	15

23	2	7	0	3	2	1	0	5
0	71	6	0	0	12	18	8	3
31	18	19	20	22	19	16	16	20
0	0	0	0	14	8	0	0	0
22	26	54	62	57	54	51	63	52
20	25	39	34	27	32	35	41	49
25	21	10	6	10	9	8	6	10
22	15	17	18	15	10	16	18	18
16	6	9	6	7	10	5	5	10
19	8	16	15	12	15	12	17	14
21	18	17	18	15	19	18	17	21
27	17	16	19	18	15	16	14	18
7	173	6	6	5	9	13	7	5
0	199	0	0	0	0	0	0	0
23	15	26	27	24	16	27	25	25
16	10	28	23	19	26	22	29	26
14	13	22	24	24	22	23	20	24
16	11	17	13	13	13	12	14	13
19	15	19	11	15	13	15	18	19
21	8	17	16	20	17	12	14	15
19	21	27	26	21	21	21	20	22
10	12	22	21	23	22	23	27	25
20	15	27	35	25	17	27	36	26
16	7	11	7	7	9	6	4	12
26	8	14	17	14	12	8	11	13
11	8	10	11	17	16	17	1	7
12	8	12	9	12	11	12	9	9
15	9	7	5	6	7	5	7	7
27	0	1	0	7	3	0	0	7
26	91	12	11	12	13	13	10	10
0	0	12	0	13	0	12	10	0
23	15	34	44	30	34	41	37	37
14	15	27	30	26	20	29	32	27
22	14	17	16	14	11	17	17	22
15	29	20	17	16	12	19	17	16
15	3	14	11	13	18	6	9	13
19	6	16	12	13	6	8	15	10
5	0	11	0	5	3	2	6	10
0	57	0	0	0	0	10	7	0
11	8	12	10	13	10	12	8	9
19	20	47	46	44	31	32	42	34
14	18	38	50	40	35	37	40	41
13	25	33	36	35	37	32	34	40
14	16	25	32	30	25	31	34	32
17	13	24	29	25	32	28	25	26
12	6	20	19	15	18	21	19	20
11	5	14	13	7	10	9	13	11

16	17	19	22	18	11	14	16	14
15	9	10	12	18	13	10	1	6
14	2	12	11	12	10	12	10	15
14	9	8	14	15	9	8	8	15
12	13	10	12	9	11	11	8	10
23	12	12	15	15	14	12	12	15
0	0	12	12	0	14	12	11	0
16	16	35	44	29	28	26	35	33
19	13	16	17	14	12	16	14	16
18	3	15	16	19	13	14	13	10
8	10	19	23	17	17	17	21	22
18	17	11	17	15	5	12	12	15
16	2	13	12	14	13	9	9	10
12	14	14	17	17	11	11	11	13
7	6	15	13	13	11	11	14	11
0	32	3	0	0	0	8	6	0
11	12	20	33	22	29	25	22	27
16	19	20	19	18	18	20	23	20
12	4	16	24	14	15	20	23	23
15	7	16	16	14	14	14	16	17
16	98	5	1	1	4	3	1	7
16	15	17	19	14	14	17	20	17
11	8	13	15	12	10	15	13	14
14	7	10	6	7	7	7	9	11
14	12	14	17	14	14	12	13	19
8	5	10	10	11	10	5	3	7
8	8	4	5	7	9	7	6	9
9	6	5	7	1	4	5	1	2
13	5	6	7	7	7	6	7	4
15	20	10	10	9	10	7	9	9
7	1	3	4	4	4	2	2	3
18	0	4	1	3	1	0	0	0
8	5	16	18	11	15	14	14	14
5	4	16	18	20	16	18	17	14
13	14	14	11	10	11	10	10	14
10	6	17	15	18	15	14	9	7
11	7	8	12	8	9	7	9	9
7	1	3	3	13	4	3	2	6
14	6	10	14	11	7	8	12	10
14	2	13	11	7	8	10	12	10
13	1	9	10	11	8	11	7	9
11	3	8	3	6	8	7	3	3
7	3	5	6	7	3	3	4	9
1	5	4	7	5	6	7	5	4
6	2	1	2	4	3	2	2	3
7	0	3	5	8	9	6	2	4
0	0	16	25	18	29	25	19	21

6	4	5	2	3	4	4	2	2
4	552	3	2	4	5	5	2	3
10	13	20	24	22	14	18	20	21
9	6	14	18	11	10	16	19	15
11	8	15	21	18	13	14	16	17
12	4	11	11	9	13	6	13	12
15	6	9	10	12	7	11	12	11
11	10	13	15	17	10	16	15	11
7	5	13	12	11	10	13	12	8
11	12	6	4	13	10	5	5	10
8	1	6	5	9	10	6	7	8
12	4	6	5	7	6	7	8	7
14	13	12	10	9	9	4	11	10
5	5	8	8	7	8	7	7	8
11	6	9	6	8	8	8	7	10
9	3	4	4	4	4	2	3	2
13	0	18	20	9	12	17	14	16
10	4	9	9	8	7	5	5	8
11	0	9	0	8	11	7	9	0
10	19	31	57	50	22	32	44	40
6	12	22	27	31	27	22	26	18
11	13	20	23	23	15	17	23	26
7	5	18	22	16	10	13	15	23
16	9	11	20	13	10	14	12	15
8	4	14	15	9	11	15	17	15
7	6	12	15	14	10	11	10	11
8	7	8	12	9	16	10	17	14
9	7	8	13	7	6	9	7	8
21	17	7	9	11	5	9	10	9
12	2	8	9	9	8	10	9	10
10	15	8	10	12	8	9	11	10
6	4	10	17	18	10	11	10	13
7	2	7	9	4	7	5	11	11
9	7	9	10	7	11	8	9	8
8	5	8	11	8	7	11	11	10
10	3	7	4	6	4	6	5	7
12	1	14	9	4	11	8	12	14
11	9	12	7	7	4	13	8	9
9	2	7	6	4	1	4	5	4
13	35	4	0	4	5	1	1	5
9	4	7	4	8	9	5	3	2
10	0	2	1	9	5	2	2	3
7	4	5	6	5	6	4	5	7
10	2	9	2	2	4	2	3	3
7	2	8	6	4	7	4	7	4
10	0	2	1	3	2	3	1	7
8	2	3	1	3	4	2	1	5

9	5	7	6	7	4	5	5	6
24	12	11	12	14	18	17	12	9
9	9	11	16	18	17	12	16	13
2	2	10	13	8	10	11	12	12
7	3	11	14	15	6	12	14	14
9	5	11	12	13	12	9	14	13
10	12	10	11	10	8	10	15	12
4	3	7	11	9	8	10	10	8
9	4	5	6	7	4	2	7	7
5	4	9	12	9	9	10	11	8
15	18	4	5	8	5	4	6	7
15	15	6	6	10	7	9	8	9
13	5	5	7	8	7	4	6	6
7	4	9	9	12	13	9	7	8
9	3	7	6	9	5	6	8	7
5	4	7	6	6	5	10	8	6
4	7	9	5	7	3	1	6	5
6	12	3	0	0	1	3	1	1
6	4	5	6	9	3	5	5	6
7	4	11	7	8	4	6	7	6
6	4	6	6	4	1	2	6	2
12	1	9	6	9	6	9	4	6
10	3	7	4	7	5	2	3	7
3	0	4	0	4	3	6	5	6
8	2	5	5	4	4	3	4	6
5	0	2	1	3	2	2	3	4
6	0	3	3	2	3	3	3	4
4	0	0	1	0	3	0	0	1
6	0	0	0	0	7	0	0	0
8	7	6	0	5	6	3	5	3
9	14	29	37	32	23	22	19	25
6	12	27	34	15	20	17	24	24
10	11	16	28	30	21	20	25	23
7	14	16	16	18	16	15	14	13
4	6	13	27	19	8	11	16	13
11	7	16	14	9	11	13	11	13
7	10	13	20	17	15	16	18	17
4	5	14	14	11	10	10	12	11
6	11	13	17	16	9	9	15	11
9	10	17	13	14	12	18	12	14
5	6	14	19	16	11	17	10	11
8	2	7	12	6	3	6	7	6
9	6	12	13	11	8	10	7	11
12	27	11	8	10	5	6	10	7
6	4	15	11	8	5	12	12	12
7	6	9	11	9	9	10	11	12
13	2	6	2	4	6	7	9	9

6	2	5	12	7	10	8	8	10
4	3	8	9	10	9	4	8	9
5	4	11	9	11	8	9	10	5
9	9	5	4	3	5	4	6	3
6	7	7	8	7	8	8	8	9
8	4	4	6	6	3	5	5	3
5	2	6	7	6	8	6	7	9
10	6	6	2	3	4	4	2	5
7	3	7	8	5	6	5	6	7
7	5	6	2	1	3	5	4	3
4	4	5	6	4	6	3	4	6
11	0	4	0	7	3	2	3	3
9	5	9	5	2	8	4	5	4
10	0	4	3	2	0	1	0	1
1	0	3	3	2	6	1	4	2
8	8	0	6	0	0	0	5	0
2	0	0	0	0	1	0	0	0
3	0	5	1	1	2	1	1	2
2	2	1	1	1	2	2	0	2
10	9	1	0	1	1	0	0	0
7	2	1	0	0	0	0	1	2
3	1	0	0	3	1	1	1	1
5	0	0	0	0	0	0	0	0
12	10	15	20	19	15	13	14	17
0	5	14	14	11	16	9	15	12
8	4	13	14	8	5	12	9	14
9	4	10	9	6	8	11	9	11
5	6	9	13	9	5	7	8	9
3	4	9	5	6	6	9	8	8
3	4	6	7	7	8	10	8	8
3	6	9	8	6	5	10	7	6
4	4	7	9	8	5	8	6	6
0	171	0	0	0	0	0	0	4
4	3	7	6	5	6	6	7	4
8	5	8	7	5	6	8	8	8
7	8	3	3	4	4	3	5	5
6	1	4	5	5	3	4	10	6
6	2	6	4	5	2	3	4	3
4	3	7	6	5	4	7	9	5
7	2	7	6	5	5	9	5	6
8	5	7	5	4	3	5	3	6
6	3	6	5	6	3	7	5	6
5	2	7	2	4	4	3	4	3
5	2	5	4	7	2	3	5	4
7	1	6	3	4	4	4	3	6
7	1	3	8	6	4	3	2	6
2	1	5	3	5	5	7	7	5

7	2	4	3	3	1	2	3	3
6	0	5	5	5	1	3	3	3
6	3	5	5	5	4	4	6	5
5	3	6	2	3	4	4	1	5
4	2	1	2	1	4	1	4	4
8	0	3	1	2	0	1	2	3
3	4	5	5	6	2	4	5	3
6	2	4	3	3	5	3	2	4
1	0	2	0	4	2	0	1	4
5	0	0	1	0	3	0	0	1
3	1	4	3	4	2	3	1	2
5	4	2	1	2	0	2	2	0
8	0	3	3	4	1	0	2	1
35	8	10	0	3	9	11	3	8
2	6	14	17	21	5	19	17	14
7	8	17	21	13	15	15	16	22
8	7	12	16	16	10	12	16	16
6	6	10	11	12	11	10	12	9
5	5	10	13	9	6	8	11	7
7	5	11	11	7	10	5	8	11
8	1	8	7	5	3	4	4	6
5	3	9	8	8	8	12	6	9
10	9	9	5	5	7	9	11	12
2	3	7	10	9	5	10	12	9
3	6	9	8	11	10	12	8	11
14	4	5	19	12	5	7	6	14
5	3	11	12	10	8	7	9	9
3	2	7	9	6	6	2	7	7
6	4	6	7	6	3	4	7	8
6	5	6	7	9	8	7	3	6
4	3	8	8	7	13	8	6	7
9	2	4	6	5	3	8	4	5
6	5	6	6	6	6	6	7	7
10	8	9	10	7	9	9	3	4
3	5	9	11	7	5	11	12	11
8	3	5	4	7	6	8	8	10
4	0	9	14	11	7	12	11	12
3	0	5	5	5	3	6	5	4
9	2	4	4	2	4	6	3	3
4	2	6	7	5	8	5	7	7
4	4	3	3	3	2	3	4	4
6	2	8	15	9	6	4	11	6
5	7	3	3	2	3	3	4	5
4	2	4	1	1	3	5	3	4
7	2	5	5	4	2	2	6	5
4	2	11	6	4	5	5	7	6
6	2	6	5	5	3	3	5	6

2	1	6	5	5	4	6	6	5
5	3	5	5	4	7	4	7	6
3	5	4	3	4	4	1	4	2
6	3	5	3	5	1	6	4	9
6	5	4	6	4	2	5	3	3
3	3	5	6	4	4	6	5	4
3	2	5	5	4	5	6	3	4
4	1	3	4	5	5	4	4	4
6	0	3	4	5	3	4	4	3
2	1	3	4	4	4	3	3	3
4	3	2	3	1	2	2	0	2
5	3	5	4	5	2	4	6	6
5	5	3	4	3	4	4	5	1
3	3	3	5	5	6	3	4	1
9	0	0	0	0	0	0	0	0
4	14	3	2	2	2	3	3	1
6	3	3	2	6	6	3	0	1
3	3	3	5	5	1	3	4	3
7	6	6	8	0	4	0	8	10
3	3	4	8	3	2	5	5	6
5	4	4	4	3	2	2	4	2
5	1	3	2	3	2	2	1	2
5	2	4	2	2	3	2	2	2
4	2	1	0	2	1	1	1	1
3	0	2	1	2	2	2	3	3
4	4	2	2	0	2	1	0	3
3	2	5	4	2	3	3	3	2
5	2	4	2	3	1	1	3	2
3	1	2	3	2	2	4	2	3
2	0	1	2	2	4	2	2	3
9	0	1	1	3	2	0	0	0
2	1	1	1	1	2	0	0	1
5	2	3	2	1	0	2	1	1
2	0	2	1	0	2	0	1	1
5	1	1	0	1	1	0	0	1
4	4	3	4	0	4	2	0	2
6	2	3	0	2	0	2	0	0
5	0	6	8	5	2	5	4	5
5	2	4	4	5	2	0	2	3
0	0	0	0	6	0	0	0	4
4	0	0	2	0	0	2	0	0
7	0	6	0	0	0	9	6	6
0	0	0	0	0	0	0	0	0
12	13	24	26	21	16	27	22	22
6	4	9	11	7	9	9	13	12
2	8	19	7	17	13	14	18	14
4	8	13	20	14	9	10	11	14

4	4	9	9	12	4	9	15	9
2	4	11	13	14	14	10	13	11
3	4	13	8	9	10	9	9	5
3	6	13	18	9	9	9	12	8
5	6	7	9	5	9	8	9	8
4	5	6	9	9	5	9	6	7
3	2	9	16	10	11	11	10	12
5	7	9	15	12	12	9	12	10
6	7	10	10	9	8	9	9	8
16	4	2	1	4	1	4	2	1
7	1	8	8	9	5	7	8	8
7	5	9	11	8	12	7	10	14
3	4	7	11	7	6	9	10	11
3	3	6	11	8	5	4	7	7
5	8	5	4	3	6	4	5	6
5	5	6	8	9	6	8	10	5
15	5	5	1	4	4	5	1	0
2	5	6	11	9	7	3	9	4
4	5	6	7	5	1	2	4	4
6	0	6	5	4	3	6	7	11
2	2	9	9	7	8	6	12	8
1	1	4	5	6	6	5	4	6
2	1	8	8	5	6	12	12	7
2	2	4	5	3	5	5	4	8
5	7	4	3	5	3	2	5	4
10	1	3	4	4	4	1	6	2
2	1	7	7	7	3	5	6	5
8	3	2	0	2	3	1	0	3
5	7	4	3	4	4	4	3	3
9	7	3	4	3	3	4	4	5
2	0	3	3	3	4	3	4	3
8	2	2	0	4	3	2	4	6
3	1	4	5	4	7	3	2	6
1	3	6	8	5	6	8	6	5
4	4	8	6	4	2	7	7	7
6	2	4	1	1	2	4	2	7
5	2	6	6	7	6	4	7	4
6	4	4	4	2	2	1	2	6
3	5	4	3	1	4	6	4	2
4	3	3	6	5	4	5	4	5
8	2	2	0	1	1	2	0	2
11	0	2	0	0	2	1	0	0
7	3	3	2	4	3	4	2	2
2	2	3	5	3	2	3	3	4
11	1	4	0	0	0	1	0	0
3	3	4	3	4	2	5	5	5
3	0	5	8	8	7	9	5	6

3	3	3	1	2	1	2	2	1
4	1	4	2	2	1	3	2	1
5	2	3	2	3	5	3	2	2
0	1	7	3	5	2	6	5	6
1	0	2	0	3	3	1	3	2
6	2	6	4	5	4	4	5	4
6	3	4	1	1	1	0	4	3
1	1	4	2	4	2	3	3	3
4	2	3	3	3	1	3	3	3
5	4	4	2	3	1	1	1	1
2	1	3	2	2	3	3	2	3
2	1	1	1	1	0	1	1	1
6	1	5	2	3	1	3	3	3
4	3	4	4	4	3	3	3	2
3	0	1	0	1	1	1	1	1
3	1	3	2	1	1	2	3	1
5	3	3	4	6	2	1	1	5
2	0	4	4	3	2	4	4	2
3	0	0	0	11	0	0	0	0
4	5	4	2	4	2	3	2	4
3	0	1	2	2	2	2	2	3
3	0	2	2	2	2	2	2	2
1	2	2	2	2	7	3	4	3
6	4	2	2	4	1	2	2	1
3	2	4	1	1	3	1	2	4
2	4	2	2	0	2	1	2	1
5	2	1	0	2	3	1	1	3
4	1	2	1	4	2	2	2	1
4	0	2	2	2	4	1	2	3
1	0	3	2	2	0	3	3	1
1	2	2	1	1	1	2	1	3
4	3	1	1	0	2	0	1	2
4	3	2	0	1	1	1	1	2
3	0	3	1	2	1	0	2	2
4	5	3	0	3	1	2	1	0
1	1	0	0	0	0	0	1	1
2	1	2	1	2	2	2	2	2
2	0	1	1	1	1	2	2	2
2	1	2	1	1	0	1	5	2
3	1	0	0	0	0	1	0	0
3	2	2	1	1	1	1	1	2
3	1	4	0	1	1	2	1	2
5	5	4	1	1	0	2	2	2
3	1	2	2	3	0	3	1	2
2	2	2	2	3	3	1	2	1
1	0	4	3	2	2	5	3	2
3	0	4	1	1	2	1	1	0

3	2	0	0	0	1	0	0	0
2	2	0	0	0	1	2	0	1
2	1	2	2	2	1	2	2	2
6	2	3	1	2	0	3	1	1
2	0	3	3	2	3	5	2	3
1	0	1	1	1	1	1	1	2
3	4	3	3	2	3	0	3	0
3	0	2	0	0	0	2	0	0
9	0	0	0	0	0	0	0	1
1	0	0	0	0	0	0	0	0
0	0	0	0	0	0	0	0	0
1	0	2	0	1	1	1	1	3
4	2	2	3	2	0	2	2	1
1	0	0	0	0	1	0	0	0
3	0	2	0	1	0	1	0	0
2	0	0	0	0	0	0	0	1
2	0	0	0	0	0	0	0	0
4	0	0	0	0	0	0	0	0
0	0	0	1	0	0	0	0	0
0	0	0	0	0	0	0	0	0
3	3	9	15	10	8	9	13	8
6	7	7	13	11	10	9	12	12
3	3	8	8	7	6	8	12	8
3	2	4	6	2	2	3	2	3
1	2	9	8	5	3	6	6	8
0	1	7	7	10	3	5	7	7
2	0	4	9	5	4	3	4	4
3	1	5	7	6	6	8	8	5
2	2	6	7	7	6	4	8	8
2	0	2	2	1	1	3	1	2
1	0	5	4	3	5	5	5	5
4	2	6	8	5	5	6	5	6
6	2	6	8	5	4	6	6	6
7	4	3	2	2	2	2	4	3
1	0	4	4	3	6	8	8	5
2	1	0	0	3	3	0	2	2
5	2	3	3	3	3	4	3	6
2	4	4	7	7	3	5	5	5
7	1	4	5	3	3	4	4	3
4	4	6	8	6	6	10	8	7
1	1	3	4	2	2	3	4	5
2	2	5	4	5	4	7	5	3
3	1	4	5	4	3	5	5	4
0	2	3	8	5	5	6	6	6
2	3	2	5	3	5	4	5	6
2	2	2	3	3	4	3	2	2
2	1	2	2	1	2	1	2	3

2	1	2	4	3	2	5	1	2
3	0	4	5	3	3	5	4	3
2	2	4	10	10	6	8	7	7
2	2	2	3	3	3	3	4	3
3	1	4	3	2	2	5	5	3
4	3	3	3	2	5	4	1	6
2	1	2	5	2	5	5	3	5
3	2	6	4	3	2	3	5	3
1	2	1	0	0	2	1	0	2
3	0	1	0	1	1	1	1	1
7	2	2	4	3	3	2	2	5
0	1	2	3	2	2	2	1	1
2	6	5	2	2	3	3	4	1
5	3	2	2	3	2	1	4	3
3	0	2	3	3	2	0	1	3
1	0	3	0	2	1	0	0	1
3	4	3	3	2	2	2	3	4
0	0	6	4	1	5	3	4	5
2	2	3	3	3	4	2	3	5
2	1	4	5	5	2	2	7	2
1	3	3	0	3	1	2	2	1
2	0	2	5	2	1	4	2	3
2	2	4	3	3	6	2	3	3
1	1	1	3	1	4	3	5	4
4	0	0	0	0	0	1	1	0
4	2	2	2	0	2	2	3	2
3	0	2	3	1	3	4	3	4
0	1	3	3	3	3	4	5	3
4	1	5	4	2	3	1	2	0
3	1	2	3	4	2	2	3	3
2	2	3	3	3	3	3	3	3
4	1	2	2	2	3	1	2	2
3	1	3	0	2	2	1	1	4
1	3	2	3	3	2	3	3	7
7	0	1	3	1	1	1	2	2
3	0	2	3	0	1	3	2	1
1	3	1	2	0	1	2	1	1
1	1	1	1	2	3	2	2	4
5	1	4	2	4	1	2	5	4
2	6	1	4	1	2	1	1	1
2	2	2	2	1	1	2	2	1
3	2	1	2	3	2	2	3	2
1	0	2	2	2	3	2	2	2
1	3	2	1	2	2	1	1	2
5	1	2	1	2	2	3	1	1
2	3	3	1	2	4	2	2	2
1	0	3	4	2	2	3	0	1

3	7	2	2	1	1	2	2	1
2	1	1	1	1	0	0	1	1
1	1	3	4	2	1	2	4	6
1	2	1	0	0	1	1	1	0
4	1	2	1	1	0	2	2	1
2	3	2	0	1	1	1	2	2
2	1	1	3	2	0	1	2	2
2	1	0	0	0	2	2	0	0
0	0	5	2	4	3	2	2	1
4	1	1	0	2	0	0	2	0
2	2	1	2	1	1	2	1	1
3	1	1	1	0	2	0	1	1
2	1	2	2	2	1	2	2	2
3	1	0	3	1	2	2	1	0
5	0	2	0	0	1	0	0	0
2	1	2	2	1	1	2	2	2
2	2	2	2	2	0	1	1	1
4	1	3	4	4	2	2	4	4
2	1	2	0	1	0	1	1	1
4	0	3	0	1	1	1	0	3
2	1	2	2	2	1	3	2	2
2	3	1	2	2	1	2	2	4
2	5	2	1	3	1	1	3	5
3	0	2	0	1	0	0	0	0
1	1	2	1	2	1	0	1	1
1	2	1	1	1	1	1	1	1
2	1	2	1	2	1	2	2	2
1	0	1	2	2	1	1	2	1
3	1	4	0	3	1	0	1	2
4	0	1	0	4	0	0	0	2
4	3	0	2	1	1	1	3	0
2	1	2	2	1	2	2	1	1
4	4	0	3	3	2	2	2	3
2	1	2	3	1	2	1	1	1
2	0	0	1	2	0	0	0	0
1	1	2	2	1	1	2	4	3
4	1	1	2	1	4	1	2	0
8	1	2	0	2	0	0	0	1
1	0	0	0	0	0	0	0	0
1	0	1	1	0	1	1	1	1
3	0	2	0	1	1	1	1	2
3	0	1	0	0	0	1	2	0
5	2	1	1	2	1	1	1	0
2	1	1	1	2	1	1	1	1
2	0	1	2	2	1	2	1	2
0	0	1	2	2	1	4	1	1
2	0	0	0	0	1	0	1	1

2	1	1	1	1	1	0	2	1
2	0	0	1	0	0	1	1	1
0	0	0	0	1	0	0	0	0
2	1	2	2	2	0	1	0	1
2	0	2	4	0	1	2	2	2
3	3	3	2	2	2	2	2	0
1	1	1	1	1	1	1	1	1
1	1	1	1	1	2	1	1	1
1	3	1	0	0	0	0	1	1
6	0	2	0	1	0	1	0	1
1	0	0	0	0	0	0	0	0
1	0	0	0	0	0	0	0	0
2	0	3	2	2	2	3	2	2
1	1	1	0	1	1	1	0	0
2	1	1	1	0	0	0	1	2
2	1	1	1	1	0	1	0	0
0	1	0	0	0	0	0	0	0
1	0	1	0	0	1	0	2	0
5	3	0	0	0	0	0	0	0
2	0	0	0	1	0	0	0	0
1	1	1	0	0	1	0	0	0
3	0	0	0	0	0	0	0	0
1	0	1	0	0	1	0	0	1
2	0	1	0	2	1	0	0	1
2	0	0	2	0	0	0	1	0
4	0	0	0	1	0	0	0	0
3	0	2	0	0	2	2	0	0
1	0	0	0	1	0	0	0	1
0	3	0	0	0	0	1	1	0
2	0	0	0	0	0	0	0	0
2	0	1	0	1	1	0	1	1
3	0	0	0	0	0	0	0	0
0	0	0	0	0	0	0	0	0
2	0	0	0	0	0	0	0	0
1	0	0	0	0	0	0	0	0
2	0	0	0	0	0	0	0	1
1	0	1	0	1	1	0	0	1
0	0	0	0	0	0	0	0	0
1	0	0	0	0	0	0	0	0
0	0	0	0	0	0	0	0	0
2	3	7	10	6	10	14	17	10
0	4	16	23	17	12	13	18	13
0	3	18	20	11	15	20	15	19
2	5	10	12	10	5	10	10	7
0	5	5	15	23	5	7	11	7
3	5	11	12	10	9	8	10	12
2	7	7	23	13	5	10	10	12

1	3	3	8	9	8	7	5	7
3	2	7	9	8	7	8	8	9
4	4	12	14	9	8	13	10	8
3	1	7	9	4	8	7	7	7
2	2	2	9	9	2	5	7	6
1	4	6	12	4	5	4	5	3
3	3	5	11	5	3	7	6	8
1	1	6	9	5	3	4	6	7
3	2	7	11	8	9	6	6	8
1	4	5	6	6	4	3	6	7
0	2	7	8	7	6	5	7	1
1	0	4	9	5	3	1	6	4
2	1	5	8	6	5	5	3	3
2	1	3	4	5	3	5	7	8
3	1	5	5	3	4	8	2	3
3	4	4	8	6	4	5	5	8
2	2	4	7	3	2	3	5	4
3	1	2	1	0	1	1	4	0
3	2	4	3	3	3	3	4	4
3	3	7	2	8	3	1	7	7
1	1	1	4	3	2	1	4	3
1	1	0	0	0	0	0	0	0
3	0	3	7	6	5	5	7	5
4	6	3	4	1	1	2	3	3
3	1	5	6	6	5	7	3	5
0	2	3	0	1	2	3	5	1
3	1	4	3	4	2	3	3	3
1	1	6	7	5	3	3	5	8
2	0	3	3	3	2	2	3	2
4	1	3	2	3	6	4	4	3
3	1	3	3	2	4	4	3	6
1	1	5	7	6	0	1	6	5
1	3	5	5	5	3	3	2	2
1	1	2	6	2	1	1	1	3
0	1	2	6	4	1	1	1	2
3	3	4	5	5	3	5	5	2
2	4	1	3	1	0	0	1	4
2	1	4	5	2	1	6	4	5
3	2	1	2	1	2	1	1	2
2	1	2	2	4	3	4	3	1
0	1	2	2	1	3	1	3	2
1	0	2	4	5	2	4	4	2
0	0	2	2	4	1	3	2	2
4	0	6	5	5	1	1	4	1
1	0	2	2	1	3	1	1	3
1	2	4	4	4	3	6	5	2
1	0	1	3	1	1	2	2	1

2	1	1	0	1	2	0	1	2
3	3	2	1	3	1	2	1	0
1	0	5	4	2	2	2	4	2
1	1	6	6	5	1	9	4	3
4	0	2	0	1	0	3	1	2
1	1	3	3	1	3	4	2	4
1	0	2	3	3	2	3	3	3
3	1	3	2	0	0	2	1	1
5	2	3	2	4	0	1	1	0
0	4	1	1	2	1	1	1	1
2	0	2	2	2	4	3	2	1
1	1	3	5	4	3	3	4	4
0	1	2	0	1	2	1	3	1
1	2	2	3	5	2	2	3	1
2	0	1	2	3	2	2	1	1
1	1	3	2	2	1	3	2	4
4	2	2	2	0	0	2	2	4
5	0	0	0	1	0	1	1	1
1	0	1	1	1	0	0	1	0
5	2	2	3	2	2	2	3	3
1	1	5	3	3	4	2	2	2
1	1	3	4	3	3	3	2	3
3	0	1	1	1	1	0	0	1
3	2	3	2	2	1	2	2	3
0	1	2	4	2	3	3	2	1
0	2	4	2	3	1	3	3	2
1	0	1	2	1	1	0	1	0
3	0	1	2	0	0	0	1	1
2	1	1	1	0	2	2	1	2
1	0	2	6	2	1	2	4	2
3	0	4	2	2	1	0	1	0
1	1	0	0	0	0	0	2	1
1	0	3	3	5	2	2	3	3
1	2	2	2	1	3	4	1	3
1	1	3	1	1	1	1	3	2
3	1	2	1	1	2	1	1	2
3	1	2	1	2	1	2	0	3
2	1	6	3	1	3	2	5	2
1	1	3	4	2	3	2	2	4
4	0	2	0	0	0	1	4	4
1	2	3	3	2	4	3	3	2
0	2	5	1	6	3	2	4	2
9	1	0	0	0	0	1	0	0
1	1	3	4	2	1	3	5	3
1	0	5	7	6	3	2	5	4
3	1	1	2	3	1	2	1	4
0	0	0	0	0	0	0	0	0

1	1	1	1	1	4	1	1	2
2	0	1	1	2	2	2	1	3
4	2	1	2	1	1	2	1	1
5	4	3	2	1	1	1	1	2
1	0	3	1	3	2	1	3	3
3	0	1	1	0	1	1	1	1
1	0	2	1	2	3	2	0	3
2	1	1	1	1	1	0	0	2
1	0	1	1	1	3	2	0	3
0	3	3	5	1	5	3	3	3
4	0	2	4	2	0	2	1	1
1	2	4	1	1	1	3	4	2
1	1	1	1	1	1	1	1	1
2	1	1	1	1	0	1	2	2
0	0	3	1	1	0	2	1	1
2	1	1	1	0	0	0	1	0
1	1	0	0	0	0	0	1	2
2	1	1	1	1	1	1	1	1
2	1	1	2	1	0	1	1	1
2	0	2	2	1	0	1	2	2
2	0	1	1	1	1	0	1	1
3	0	1	3	1	1	3	3	2
2	0	1	1	0	1	0	1	1
2	1	0	1	1	0	1	1	2
3	1	0	2	0	1	2	0	2
2	4	1	1	1	2	3	1	1
2	3	1	2	2	2	3	2	1
1	0	0	1	1	1	0	1	1
2	1	2	0	1	0	0	1	1
1	2	1	1	1	1	0	0	1
0	0	0	1	0	0	0	1	0
1	2	1	1	2	1	2	2	1
2	1	0	0	0	1	0	1	1
1	0	1	4	2	1	2	2	2
1	0	1	1	2	3	1	1	1
2	0	2	1	1	3	0	1	1
1	0	3	2	2	1	1	2	4
2	0	1	0	1	1	3	2	1
2	2	1	1	2	1	1	1	2
0	0	0	1	1	1	1	1	1
1	0	2	1	2	1	0	2	4
1	0	0	2	1	1	0	0	0
1	0	0	1	2	0	1	0	0
1	0	0	3	0	1	2	2	0
3	0	3	1	2	1	2	1	2
1	0	3	3	2	2	2	1	2
2	0	1	1	1	2	1	0	0

1	2	2	0	0	0	1	1	0
1	1	2	2	1	2	2	1	2
2	1	1	1	1	0	1	2	1
2	1	1	0	0	0	1	1	0
0	0	3	1	1	1	3	0	3
0	0	2	2	3	2	1	3	2
3	2	2	1	2	0	2	1	1
0	1	2	1	1	0	0	0	1
1	0	3	1	1	1	1	2	1
1	0	1	0	0	2	1	1	0
3	0	0	0	0	2	1	0	2
1	0	2	2	1	1	1	1	1
1	0	2	2	2	1	1	3	1
1	0	1	2	1	0	1	1	1
1	0	2	0	2	1	0	1	1
2	0	0	0	0	0	0	1	0
3	0	2	0	0	1	0	0	0
1	0	1	1	1	1	1	1	1
1	0	0	0	0	0	1	0	0
5	1	1	1	4	0	0	0	1
1	0	0	1	0	1	1	0	0
2	1	1	1	1	1	1	1	1
2	1	1	2	1	0	1	1	0
1	0	2	0	0	0	1	0	1
0	0	1	0	1	0	1	1	0
0	0	0	1	0	0	1	0	0
1	0	1	0	1	0	0	0	0
1	0	1	0	2	1	1	1	1
2	1	2	1	2	1	1	1	1
2	0	1	1	1	1	1	1	2
1	0	1	1	0	1	1	2	0
2	2	1	1	1	1	1	1	1
1	1	1	1	1	2	2	1	1
1	1	2	1	1	1	1	1	2
1	1	1	0	0	0	1	0	0
3	0	0	1	0	0	1	1	1
1	0	2	1	1	3	4	1	2
1	0	1	0	1	2	0	0	0
1	0	1	0	1	1	0	2	1
1	0	1	0	1	0	1	1	0
1	1	0	0	0	0	0	0	0
1	0	1	0	0	0	0	0	0
1	0	0	0	0	2	1	0	0
1	0	0	2	1	3	0	0	0
0	21	0	0	0	0	0	0	0
1	2	1	1	1	1	1	1	1
1	0	1	1	1	1	1	2	1

0	0	2	1	1	1	0	1	1
2	1	1	1	1	0	0	1	1
1	0	1	1	1	0	1	1	1
4	0	0	0	0	0	0	0	0
1	1	1	1	1	1	1	1	1
2	1	2	0	1	1	1	1	1
1	4	0	1	0	0	1	1	1
2	0	2	0	0	0	0	0	0
0	0	1	1	0	0	0	2	0
1	3	1	0	1	0	1	0	0
1	0	1	0	0	1	0	2	1
0	0	0	0	0	1	0	0	0
0	14	0	0	0	0	3	0	0
1	0	1	1	0	1	0	1	1
1	2	2	0	0	1	1	1	0
0	1	0	0	0	0	0	0	0
3	1	1	2	0	1	0	0	2
2	0	2	1	1	1	1	1	1
0	0	1	1	1	0	1	1	1
1	0	2	2	2	1	1	2	1
0	0	2	1	1	0	1	2	1
1	0	1	0	1	0	1	0	0
2	0	0	0	0	1	0	1	1
1	2	1	1	1	0	0	1	0
1	0	1	0	1	0	0	0	1
1	1	1	1	1	1	1	1	4
1	1	1	0	1	1	1	1	1
1	0	1	0	1	0	1	1	0
1	2	1	1	0	1	1	1	2
2	0	1	0	0	0	0	0	0
1	1	0	0	0	0	0	0	1
2	0	0	0	0	0	0	0	1
1	0	0	0	0	0	1	1	1
0	2	0	0	0	0	0	0	1
1	0	0	0	0	0	0	0	0
1	1	0	0	0	0	1	1	0
4	0	2	1	1	0	1	1	1
1	1	1	0	0	0	0	1	1
1	0	1	0	0	0	0	0	1
2	0	1	0	1	1	1	0	0
0	0	0	0	0	0	0	0	0
0	3	0	0	1	0	1	1	1
1	1	1	1	2	0	1	1	1
0	0	0	2	1	0	0	1	0
4	0	0	0	0	0	0	0	0
2	0	1	1	1	1	1	1	1
0	1	0	1	1	0	2	1	0

1	0	1	0	0	1	2	1	0
0	2	0	0	0	0	0	0	0
0	7	1	0	0	0	1	1	0
1	19	0	0	0	0	0	0	0
1	1	1	1	0	0	0	1	1
1	0	1	1	0	0	1	1	1
1	0	1	0	0	1	0	1	1
1	0	2	2	2	0	2	3	0
3	0	0	0	0	0	0	0	0
1	0	1	0	1	1	1	0	0
1	0	1	0	0	0	0	0	1
1	0	1	1	0	2	1	1	1
2	0	1	0	0	1	0	1	2
0	0	0	0	0	0	0	1	0
1	0	1	0	1	0	0	1	1
0	0	0	0	0	2	0	0	1
1	0	0	0	0	0	0	0	0
2	0	1	0	1	0	1	0	1
0	0	0	0	0	0	0	0	0
1	0	0	1	0	0	0	0	0
1	0	0	0	0	0	0	0	1
1	0	0	0	0	1	0	0	1
0	0	0	0	0	1	0	0	0
3	0	0	0	0	0	0	0	0
2	0	1	0	0	0	0	1	0
1	0	2	0	0	0	1	2	1
0	0	0	0	0	0	0	3	1
1	2	0	0	0	0	1	1	1
0	0	1	1	0	0	0	0	1
1	0	0	0	0	0	0	0	0
1	0	1	0	1	1	0	0	0
0	0	1	1	0	0	1	0	0
2	0	1	0	0	0	0	0	0
2	0	0	0	0	0	0	0	0
2	0	0	0	1	0	0	0	0
0	0	0	0	0	0	0	0	0
0	0	0	0	0	0	0	1	0
0	0	0	0	0	1	1	0	0
0	0	0	0	0	0	0	0	0
5	5	11	12	10	10	13	11	12
1	2	8	12	14	8	10	6	8
0	18	7	6	10	13	10	7	9
1	2	8	14	8	7	10	11	11
1	1	8	7	8	8	8	9	11
0	157	1	0	1	0	3	1	1
3	3	7	7	5	5	5	5	8
0	0	5	12	7	8	9	10	14

3	4	12	14	5	10	11	10	7
2	1	11	9	8	5	7	10	4
5	0	5	0	2	9	6	8	2
2	3	6	8	12	7	4	4	2
1	3	4	7	4	4	3	4	3
4	0	2	6	3	3	3	5	5
3	1	0	8	6	18	3	10	4
0	2	4	2	4	1	2	5	2
1	1	5	3	2	4	4	4	3
0	4	3	10	8	4	4	7	3
0	3	3	6	6	6	4	2	5
1	0	5	8	6	7	4	8	4
1	1	5	5	3	2	1	4	5
1	1	3	6	8	3	4	3	4
0	1	1	2	2	4	1	2	4
0	0	2	8	7	2	7	7	8
1	0	2	3	1	4	1	2	3
5	0	4	2	1	0	0	1	1
1	0	3	7	1	3	5	3	4
0	2	2	2	5	0	3	2	1
2	1	7	5	2	3	3	4	8
0	1	1	5	4	1	2	2	4
0	0	2	0	1	0	4	2	1
1	0	8	4	7	3	4	8	5
1	1	3	4	3	4	7	5	4
0	1	4	1	2	3	1	4	1
1	71	0	0	0	0	0	0	0
1	1	1	4	3	1	4	1	3
1	0	4	3	4	1	2	4	3
2	1	2	3	2	2	2	1	1
0	78	0	0	0	0	0	0	0
0	2	4	5	5	4	4	3	4
1	2	7	0	5	2	8	6	1
12	11	7	0	10	7	10	7	9
0	1	3	2	3	3	5	4	4
0	0	1	2	0	1	0	1	2
0	0	5	7	6	3	3	5	5
0	0	3	2	2	3	2	4	4
0	1	2	5	5	4	1	3	3
0	1	3	4	2	4	3	3	2
0	1	0	1	3	4	2	3	4
2	0	1	2	3	2	2	4	1
1	0	2	2	0	1	0	4	2
0	0	5	3	1	4	3	3	3
0	0	3	3	3	2	3	5	2
0	59	0	0	0	0	0	0	0
3	2	2	3	3	0	1	3	3

1	0	6	6	4	4	1	4	4
0	1	2	2	2	1	1	3	0
2	0	0	1	1	1	0	0	0
0	83	0	0	0	0	0	0	1
0	0	0	2	3	0	0	1	0
2	0	2	0	1	0	0	0	2
1	0	0	1	0	1	0	0	1
0	0	2	3	1	2	4	4	2
1	1	0	0	0	1	0	1	0
0	0	2	6	2	4	2	4	2
1	2	0	2	0	0	0	1	0
16	3	5	2	3	2	3	0	0
1	0	0	0	0	2	0	0	1
1	0	3	4	2	2	3	4	2
1	0	4	2	2	0	3	2	1
0	0	1	5	2	1	0	2	4
0	1	2	2	2	1	1	3	1
0	1	1	1	0	0	2	1	1
1	0	1	1	1	0	3	1	2
0	2	3	3	4	0	5	3	3
0	0	0	1	1	1	2	1	0
0	29	0	1	0	0	5	2	1
0	0	4	4	1	3	2	0	3
0	2	1	2	1	0	0	0	1
1	0	3	4	2	3	4	4	4
2	2	1	4	1	0	0	1	0
1	0	0	0	2	1	0	0	0
0	0	3	2	1	3	3	4	4
0	0	1	1	0	0	1	2	0
1	1	2	3	2	1	3	2	2
1	0	2	1	1	2	2	2	1
0	1	2	4	3	1	2	3	3
0	0	0	0	0	0	0	0	0
1	0	1	0	0	3	2	1	1
0	3	1	2	1	2	2	2	1
0	2	1	1	1	0	1	1	1
3	1	1	0	1	3	1	1	1
2	1	2	1	0	4	0	3	2
1	0	1	1	2	1	1	1	1
2	0	1	2	1	2	0	1	1
0	0	1	0	1	1	1	1	1
0	1	1	1	1	0	1	1	0
0	0	2	2	2	1	1	1	1
1	1	1	0	1	0	0	1	1
0	0	0	1	0	2	2	4	3
17	0	0	0	0	0	0	0	0
1	1	1	1	1	1	1	0	1

0	0	2	3	2	1	1	2	3
0	0	0	0	0	0	0	0	0
9	0	0	0	2	0	0	0	0
1	1	1	2	1	1	2	1	2
1	0	2	1	0	1	2	1	1
1	0	2	3	4	2	1	2	2
1	1	4	2	1	1	2	2	3
2	0	4	3	2	1	4	2	2
1	0	3	3	2	1	1	4	1
0	1	1	2	0	1	2	0	0
0	0	2	1	1	0	1	0	0
1	0	1	0	0	1	1	0	0
1	5	0	1	0	0	1	0	0
0	1	1	0	2	0	0	2	1
0	15	0	2	2	0	2	1	2
0	1	0	0	0	0	0	0	0
0	37	0	0	0	0	0	0	0
1	0	1	4	1	0	1	2	1
2	0	1	1	2	0	1	1	1
1	0	3	2	1	2	2	2	0
0	0	0	2	0	0	0	0	0
0	0	1	2	1	0	0	2	1
0	2	0	0	0	1	0	0	0
2	0	1	2	2	1	1	1	2
0	0	1	0	0	0	0	1	2
1	0	2	3	2	2	3	3	2
0	0	0	1	0	1	0	0	0
1	1	1	1	0	1	1	1	1
0	1	1	1	1	0	1	1	2
0	0	2	7	2	1	1	2	1
0	0	1	1	1	0	0	0	0
0	0	0	0	0	0	2	0	0
1	0	1	1	3	0	1	1	2
1	4	1	1	1	1	2	2	1
0	0	1	4	3	1	1	1	3
0	0	0	0	0	1	0	0	0
0	0	0	0	1	0	0	0	0
0	1	1	1	1	1	2	2	1
1	0	0	1	1	0	2	1	0
0	0	0	3	0	1	1	0	0
0	2	1	1	0	0	0	0	2
0	0	1	3	1	1	2	0	1
0	0	1	1	1	0	0	0	1
3	1	0	0	0	0	0	0	0
0	0	1	1	0	1	2	2	0
0	0	3	2	3	2	2	2	2
1	0	1	0	0	2	2	0	1

0	0	1	6	1	1	3	1	2
0	0	1	1	0	0	0	3	0
0	33	0	0	0	0	0	0	0
1	0	1	1	1	1	1	1	1
1	1	1	0	0	2	1	1	0
1	3	0	1	0	0	0	1	2
1	0	1	2	1	2	2	2	2
3	2	1	1	1	1	2	0	1
2	0	1	2	1	2	2	2	0
1	0	0	0	0	0	0	0	0
0	0	2	0	0	0	1	3	1
1	1	0	0	1	0	1	0	0
0	33	0	0	0	0	0	0	0
0	0	1	0	1	3	2	3	1
0	1	0	0	0	0	1	0	0
0	0	2	1	1	3	1	0	0
2	0	1	0	0	0	0	0	0
0	0	0	0	0	0	1	1	1
2	1	0	0	0	1	1	0	1
0	0	2	1	0	0	1	0	0
0	0	0	9	10	0	0	0	5
2	0	2	2	1	2	1	0	0
15	0	6	8	0	0	0	0	9
0	0	0	1	0	1	0	0	0
0	0	3	3	1	2	2	4	0
1	1	2	1	1	1	1	0	0
0	0	0	0	1	1	1	1	1
0	1	1	2	0	0	0	0	2
2	2	1	0	0	1	0	1	2
0	0	0	1	1	0	0	0	0
0	38	0	0	0	0	0	0	0
5	3	7	9	8	7	8	8	0
1	2	1	0	0	0	0	1	0
0	1	3	4	2	2	3	2	2
2	1	1	1	3	1	0	0	0
0	0	0	3	0	0	2	1	1
2	2	1	1	0	0	0	0	1
0	29	0	0	0	0	0	0	0
1	0	2	0	0	0	2	1	1
0	0	1	0	1	1	0	0	1
2	2	2	1	0	4	1	2	1
0	0	0	0	0	3	0	1	1
0	2	1	0	0	0	1	0	0
1	0	0	0	0	2	0	0	0
0	1	0	1	0	0	1	1	2
0	0	1	0	1	0	0	0	0
1	0	0	0	0	0	0	0	0

0	0	24	0	27	0	0	20	0
0	0	0	0	0	0	0	0	0
0	1	0	1	0	1	1	1	1
0	0	0	1	0	0	2	1	1
1	1	0	1	0	1	0	1	2
1	0	0	0	0	0	0	0	0
3	1	1	2	1	0	1	1	0
1	2	1	0	0	0	0	0	0
0	0	0	0	0	1	0	1	0
0	1	1	0	1	0	1	1	0
1	0	1	1	0	0	1	1	0
9	0	3	0	0	3	3	0	0
0	1	0	1	1	1	2	2	1
0	0	0	0	1	0	1	2	0
0	0	1	1	0	2	1	2	1
0	0	0	2	0	0	0	0	0
0	14	0	1	0	0	1	1	0
0	3	1	0	0	1	0	0	0
0	0	0	0	0	0	0	0	0
0	1	0	1	0	0	2	1	0
1	1	0	1	1	0	0	1	1
1	0	0	1	1	0	0	0	0
0	0	2	0	2	0	1	1	0
0	1	1	1	2	1	1	3	1
0	0	0	1	2	1	4	2	1
1	0	0	0	0	1	0	0	0
1	0	0	0	0	0	0	0	0
0	0	1	0	0	0	0	0	0
0	0	0	0	0	0	0	0	0
3	0	2	1	1	0	1	0	1
0	0	0	0	1	0	0	0	1
0	21	0	0	0	0	0	0	0
0	14	0	0	0	0	0	0	0
1	0	1	1	1	0	0	3	1
1	0	0	4	1	1	1	1	1
0	0	1	0	1	0	0	1	1
1	1	1	2	1	0	1	0	0
0	0	1	2	0	0	2	0	0
0	0	4	4	0	0	0	0	2
0	0	0	2	1	0	1	0	0
0	0	0	0	0	0	0	0	1
1	0	1	1	1	1	1	1	1
0	0	1	1	1	1	1	1	1
0	0	1	0	1	0	0	2	1
1	0	1	0	0	0	1	1	0
0	0	0	0	0	0	0	0	1
0	0	2	0	0	0	0	1	1

0	0	0	0	0	1	0	0	0
0	0	0	1	1	0	2	1	1
0	2	0	0	0	0	0	0	0
0	0	2	0	0	0	3	1	1
1	0	0	0	0	1	0	0	0
3	2	0	0	0	0	0	0	0
1	1	1	1	1	1	1	0	2
0	0	1	1	0	0	1	2	0
1	0	1	1	0	0	1	1	0
2	0	1	0	0	1	1	0	1
0	0	0	1	0	0	0	0	1
0	0	0	1	0	0	0	1	0
1	0	0	1	0	0	0	0	0
0	0	1	1	1	1	0	1	1
1	2	0	0	0	0	3	1	1
2	1	0	1	0	0	0	0	1
0	0	0	0	2	0	0	2	0
1	0	0	1	0	0	1	0	0
0	0	0	0	0	0	0	0	0
0	0	0	0	0	0	0	0	0
0	0	1	1	1	1	1	1	1
0	0	1	2	0	0	0	1	1
0	2	0	0	1	0	2	0	1
0	0	0	2	1	1	1	1	0
2	0	1	1	1	1	2	1	0
0	0	0	0	0	1	0	1	0
2	0	0	0	0	0	1	0	0
0	0	0	0	0	0	0	0	0
1	1	0	1	1	0	2	1	0
1	0	1	0	0	1	0	2	0
0	0	0	0	0	0	0	0	0
0	4	2	0	0	0	2	4	0
0	18	0	0	0	0	0	0	0
1	0	1	0	1	2	2	1	0
1	0	1	3	1	1	1	1	2
0	1	2	0	1	1	0	1	1
0	0	1	1	0	1	2	2	1
1	0	0	0	0	1	0	1	1
0	0	0	0	0	0	1	0	1
0	0	0	0	0	0	0	1	0
1	0	1	0	0	0	0	0	1
0	1	0	1	1	0	0	0	1
0	0	1	2	1	1	2	2	1
1	0	0	0	0	0	0	0	0
0	0	0	0	0	0	0	0	0
3	0	1	2	0	0	0	0	0
0	0	0	1	0	1	1	0	0

1	2	0	0	0	0	0	0	1
0	0	0	0	0	1	0	0	0
0	0	0	0	0	0	0	2	0
0	0	0	0	1	0	0	0	0
0	15	0	0	0	0	0	0	0
0	1	0	1	0	1	2	1	1
0	0	1	2	2	2	1	1	1
2	0	0	0	0	0	1	0	0
0	0	1	3	1	0	1	1	1
0	0	0	1	0	1	1	0	0
0	0	0	0	0	0	0	0	0
0	0	0	0	0	0	0	0	0
0	0	1	0	0	1	2	1	0
0	0	1	0	0	0	1	0	0
0	1	0	0	1	2	0	1	0
1	0	0	0	1	0	0	0	0
1	1	1	1	0	0	2	1	1
2	0	0	0	1	1	0	1	2
0	1	2	1	1	0	1	1	1
0	0	0	1	1	0	2	1	1
0	0	0	0	0	0	0	0	0
0	0	2	0	0	0	0	2	1
0	0	0	0	0	0	0	0	0
1	0	0	1	0	0	0	0	1
1	1	0	0	0	0	0	0	0
2	1	0	0	0	0	1	0	0
0	0	1	1	0	0	1	0	0
0	51	0	0	0	0	0	0	0
1	0	1	1	0	0	0	1	1
0	0	1	1	1	0	1	1	1
0	0	2	0	1	0	0	0	1
0	0	0	0	0	0	0	0	0
1	0	2	0	1	1	2	1	1
0	0	2	1	0	1	1	2	2
0	0	0	0	1	0	0	1	0
0	0	0	0	1	0	0	0	0
0	1	0	0	0	0	0	0	0
0	0	0	0	0	1	1	0	0
0	0	0	0	1	0	0	1	0
11	0	6	0	0	0	0	3	6
0	0	0	1	0	0	2	0	0
1	0	0	0	0	0	0	0	0
0	0	0	0	0	0	0	0	0
0	11	0	1	0	0	0	0	0
0	0	0	0	0	0	0	0	0
1	0	0	0	1	0	0	0	1
0	0	0	0	0	0	0	0	0

0	0	0	0	0	0	0	0	0
0	1	0	1	1	0	0	0	2
0	0	0	0	0	0	0	0	0
0	0	0	0	0	0	0	0	0
0	0	0	0	0	0	0	0	0
1	0	0	0	0	0	0	0	0
0	0	0	2	0	0	0	0	1
0	11	0	0	0	0	0	0	0
0	16	0	0	0	0	0	0	0
0	16	0	0	0	0	0	0	0
2	0	1	1	0	0	0	0	0
0	0	0	0	0	0	0	0	0
0	0	0	1	0	1	2	0	1
0	0	1	1	2	0	1	0	3
0	0	1	0	0	0	1	0	0
0	0	0	0	0	0	1	0	0
1	0	0	0	0	0	0	0	0
0	0	0	2	0	0	1	1	0
0	0	0	0	0	0	0	0	0
0	0	0	0	2	0	2	0	0
0	0	1	1	1	0	0	1	3
1	0	0	0	0	1	0	1	0
0	0	0	0	0	1	0	0	0
0	13	0	0	0	0	0	0	0
0	0	0	0	0	0	0	0	0
5	0	0	0	0	2	0	0	0
0	0	0	0	0	0	0	0	0
0	0	0	0	0	0	0	0	1
0	0	0	0	0	0	0	0	0
1	0	0	0	0	0	1	0	0
0	0	0	1	0	1	0	1	0
0	0	0	0	0	0	0	0	0
3	0	1	0	0	0	0	1	2
0	1	1	0	0	0	0	1	0
0	1	0	0	2	0	1	1	0
0	1	3	0	0	1	0	0	1
2	0	0	0	0	0	0	0	0
0	0	0	0	0	0	0	0	0
0	0	0	2	2	0	1	0	2
0	0	0	0	0	0	0	0	0
0	0	0	0	0	0	0	0	0
0	0	0	0	0	0	0	0	0
0	0	0	0	0	0	0	0	0
1	7	0	0	0	0	0	0	0
2	0	0	0	0	0	0	0	0
0	0	0	0	0	1	0	0	0
0	0	0	0	0	0	0	0	0

0	0	1	1	1	0	1	2	1
0	0	0	0	0	0	0	0	0
0	0	1	0	1	1	0	0	0
1	0	1	0	0	0	0	1	0
0	0	0	0	0	1	0	0	0
0	0	0	0	0	0	0	0	0
1	1	0	0	0	0	0	0	0
2	1	1	1	0	0	1	0	0
1	0	0	0	0	0	0	0	0
5	0	1	0	0	0	0	0	0
2	1	0	0	0	0	0	0	0
0	0	0	2	0	1	0	0	2
0	0	2	1	0	0	3	0	0
0	0	0	4	3	0	0	1	0
1	0	0	0	0	0	0	0	0
0	0	0	0	0	0	0	0	0
0	0	1	2	1	1	1	1	0
0	0	0	0	0	0	0	0	0
0	0	0	0	0	0	0	0	0
0	0	0	1	0	1	1	0	0
2	0	1	2	1	1	1	1	0
2	1	1	0	2	0	0	1	0
0	3	0	0	0	0	0	0	0
0	1	0	0	0	0	0	0	0
1	1	0	0	0	0	0	0	0
0	0	0	0	0	0	0	0	0
0	1	2	0	0	0	0	0	0
0	0	0	0	0	0	0	0	0
0	0	0	0	0	0	1	0	0
0	0	0	0	1	0	0	0	0
3	0	0	0	0	0	0	0	0
0	0	0	0	0	0	8	0	0
0	10	0	0	0	0	0	0	0
0	12	0	0	0	0	0	0	0
1	0	1	1	0	0	1	1	0
0	0	0	1	1	0	0	0	0
2	0	1	0	0	0	0	0	0
0	0	2	0	0	0	0	0	0
1	0	1	0	0	0	0	0	0
1	0	0	0	0	0	0	0	0
0	1	0	0	0	1	0	0	0
2	0	1	0	0	0	0	0	0
0	2	0	0	0	0	0	0	0
0	0	1	0	0	1	0	1	1
0	0	0	0	0	0	0	0	0
0	0	1	0	0	0	1	0	1
0	2	2	0	0	0	1	1	1

0	0	0	0	0	0	0	0	0
0	0	0	3	1	0	0	2	0
2	0	0	0	0	0	0	0	0
1	3	0	0	0	0	1	0	0
0	0	0	0	0	0	0	0	0
0	0	0	1	0	1	0	0	0
0	0	1	0	0	0	1	0	0
2	0	0	0	1	0	0	0	0
0	0	1	0	1	0	1	2	1
0	0	0	0	0	1	0	0	0
2	0	0	0	0	1	1	0	1
2	2	1	0	0	0	0	1	0
0	0	0	1	1	0	0	0	0
2	0	0	0	0	0	0	0	0
0	0	2	2	0	0	2	0	0
0	0	0	0	0	0	0	0	0
0	0	0	0	0	0	0	0	0
1	1	0	0	0	0	0	0	0
2	0	0	0	0	0	0	0	0
0	2	0	0	0	0	1	1	0
0	0	0	0	0	0	0	0	0
0	0	0	2	4	0	0	0	1
4	0	0	0	0	0	0	0	0
0	8	0	0	0	0	0	0	0
0	0	0	0	0	0	0	0	0
0	30	0	0	0	0	0	0	0
1	0	0	0	0	1	0	0	0
0	0	0	0	0	0	0	0	0
1	0	0	0	0	0	0	0	1
0	0	0	1	0	1	3	0	0
0	0	0	0	0	0	0	0	0
1	0	0	0	0	0	0	0	0
0	0	48	0	0	0	46	43	43
0	9	0	0	0	0	0	0	0
0	0	0	0	0	0	0	0	0
0	0	0	0	1	0	0	0	0
0	2	0	0	0	0	1	0	0
0	6	1	0	0	0	0	0	0
0	0	0	0	0	0	0	0	0
0	10	0	0	0	0	0	0	0
0	0	0	0	0	1	0	0	0
0	0	0	1	0	0	0	0	0
0	0	0	0	0	0	0	0	0
0	0	0	0	0	0	0	0	0
0	0	0	0	0	0	0	0	0
0	0	0	0	0	0	0	0	0
0	0	0	0	0	1	0	0	0

0	0	0	0	0	0	1	0	2
0	0	0	0	0	0	0	0	2
0	0	1	0	0	0	0	0	0
0	0	0	0	0	0	0	0	0
0	0	0	0	0	0	0	0	0
0	7	0	0	0	0	0	0	0
1	0	0	0	1	0	0	0	0
0	0	0	0	0	0	0	0	0
0	0	0	0	1	0	0	0	0
0	3	0	1	1	0	0	0	0
0	0	1	2	0	0	1	1	0
1	0	1	1	0	0	0	0	0
1	0	0	1	2	1	0	1	0
0	0	0	0	0	0	0	0	0
0	2	0	0	0	0	1	0	0
0	0	0	0	0	0	0	0	0
0	0	0	0	0	0	0	0	0
0	0	0	0	0	0	0	0	0
0	0	0	0	0	0	0	0	0
0	0	0	0	0	0	0	0	0
0	0	0	0	0	0	0	0	0
0	0	1	0	0	0	0	0	0
0	0	0	0	0	0	0	0	0
0	5	0	0	0	0	5	0	0
0	6	0	0	0	0	0	0	0
0	6	0	0	0	0	0	0	0
0	0	1	0	0	0	0	1	1
0	0	0	0	0	0	0	0	1
0	0	0	0	0	0	0	0	0
0	0	0	0	0	0	0	0	0
0	0	0	1	0	0	1	1	0
0	0	0	0	0	0	0	0	0
0	0	0	0	0	0	1	2	0
0	0	0	0	0	0	0	0	0
0	0	0	0	0	0	0	0	0
0	0	0	0	0	0	0	0	1
1	2	0	0	0	0	0	0	0
0	0	0	0	0	0	2	0	0
2	0	0	0	0	0	0	0	0
0	3	0	0	0	0	0	0	0
0	174	0	0	0	0	0	0	0
0	0	0	0	0	0	0	0	0
0	0	0	0	0	0	0	0	0
0	3	0	0	0	0	0	0	0
0	5	0	0	0	0	0	0	0
1	0	0	0	0	0	0	0	0
0	0	0	0	0	0	0	0	0

1	0	0	0	0	0	0	0	0
0	0	1	0	0	0	1	2	0
0	0	0	0	0	0	1	1	0
0	0	0	0	0	0	0	0	0
0	0	0	0	0	0	1	0	0
0	0	0	0	0	0	0	1	0
1	2	0	0	0	0	0	0	0
0	0	0	0	0	0	0	0	0
0	3	0	0	0	0	0	0	0
0	0	0	0	0	0	0	0	0
0	4	0	0	0	0	0	1	0
0	0	0	0	0	0	0	0	0
0	4	0	0	0	0	0	0	0
0	3	0	0	0	0	0	0	0
0	6	0	0	0	0	0	0	0
2	0	0	0	0	0	0	0	0
0	0	0	0	0	0	0	0	0
0	7	0	0	0	0	0	0	0
0	0	0	0	0	0	4	0	0
0	0	0	0	0	0	0	0	0
0	3	0	0	0	0	0	0	0
0	3	0	0	0	0	0	0	0
0	0	0	0	1	0	0	0	0
1	0	0	0	0	0	0	0	0
1	0	0	0	0	0	0	0	0
0	0	0	0	0	0	0	0	0
0	0	0	0	0	0	0	0	0
0	0	0	0	0	0	0	0	0
2	0	0	0	0	0	0	0	0
0	0	0	0	0	0	0	0	0
2	0	0	0	0	0	0	0	0
2	0	0	0	0	0	0	0	0
0	0	0	0	0	0	0	0	0
0	0	0	0	0	0	0	0	0
0	3	0	0	0	0	0	0	0
0	3	0	0	0	0	0	0	0
0	3	0	0	0	0	0	0	0
1	0	0	0	0	0	0	0	0
0	0	0	0	0	0	0	1	0
0	0	0	0	0	0	0	0	0
0	0	0	0	0	0	0	0	0
0	0	0	0	0	0	0	0	0
0	3	0	0	0	0	0	0	0
0	2	0	0	0	0	0	0	0
0	3	0	0	0	0	0	0	0
0	3	0	0	0	0	0	0	0
0	0	0	0	0	0	0	0	0

0	2	0	0	0	0	0	0	0
0	2	0	0	0	0	0	0	0
0	2	0	0	0	0	0	0	0
0	2	0	0	0	0	0	0	0
0	2	0	0	0	0	0	0	0
0	0	0	0	0	0	0	2	0
0	2	0	0	0	0	0	0	0
0	2	0	0	0	0	0	0	0
0	0	0	0	0	0	0	0	0
0	0	0	0	0	0	0	0	0

Total Spect Normal kid T14	Total Spect Normal kid T15	Total Spect Normal kid T28	Total Spect Normal kid T29	Total Spect Normal kid T30	Total Spect Vancomyci T16	Total Spect Vancomyci T17	Total Spect Vancomyci T18	Total Spect Vancomyci T19
54	47	48	38	38	62	52	59	60
27	14	29	19	18	42	35	45	48
25	14	31	37	46	58	32	54	32
20	18	16	23	20	51	26	32	39
42	28	35	39	45	37	40	64	41
25	40	11	1	3	5	0	4	0
44	52	80	43	52	64	60	65	48
18	48	22	5	8	26	3	9	9
69	64	69	62	70	60	48	57	32
17	29	15	3	0	33	0	0	9
51	29	50	56	55	58	47	59	39
34	27	47	34	34	43	40	45	31
26	22	20	19	19	22	21	22	19
36	27	28	30	28	28	26	32	28
29	18	28	24	21	27	13	23	19
46	53	81	68	75	47	60	48	36
42	28	33	29	33	38	41	37	36
20	40	17	5	2	32	0	4	7
29	24	28	28	29	31	31	27	25
13	17	22	22	21	28	27	32	26
36	27	35	33	33	35	34	28	27
48	45	42	44	36	34	41	26	28
24	22	11	10	8	9	9	9	9
47	41	56	46	43	50	42	34	24
39	53	67	65	63	75	85	70	44
9	14	4	6	4	6	6	7	8
28	17	21	16	17	26	19	22	22
13	17	5	18	9	28	12	17	18
29	25	45	46	42	35	33	32	25
11	17	6	13	11	31	35	32	23
2	4	4	5	3	20	5	15	14
9	12	7	8	6	10	8	11	8
3	9	4	3	0	8	3	3	4
22	21	27	27	28	28	31	23	21
54	40	84	63	69	28	42	28	26
22	15	56	44	45	50	50	42	33
29	27	20	26	22	23	27	24	23
24	19	36	23	37	21	34	23	17
9	15	0	6	4	6	6	8	8
43	40	54	60	52	34	38	34	28
5	6	9	15	15	31	19	27	26
44	31	40	47	40	24	27	24	24
9	5	16	22	17	29	14	17	17

6	2	1	1	0	24	14	10	21
6	17	0	0	0	9	0	10	0
21	18	0	18	17	24	20	20	19
0	0	0	6	0	9	4	0	0
61	74	49	40	51	22	40	19	15
49	33	58	72	64	34	55	32	27
7	5	4	3	4	13	9	10	12
16	13	19	22	17	16	24	20	15
5	0	13	15	13	26	13	19	20
13	13	11	12	15	18	15	22	10
17	15	26	29	30	29	29	23	23
20	19	19	17	17	24	20	20	17
6	10	10	6	7	10	0	11	8
0	0	0	0	0	0	0	0	0
29	25	36	27	33	21	31	22	18
34	31	22	27	27	19	22	15	15
23	21	20	22	20	16	20	13	11
11	14	22	19	22	22	17	19	9
14	18	18	13	10	18	16	12	12
17	13	16	12	15	12	13	14	12
28	21	25	27	23	23	28	25	19
28	30	42	28	38	23	35	20	21
31	35	28	33	34	24	28	26	20
8	2	11	15	9	39	52	25	17
10	9	20	17	18	26	27	19	13
8	11	16	17	5	17	22	15	16
10	21	17	8	12	16	13	9	10
5	5	4	6	6	14	19	13	13
0	0	0	0	0	20	2	10	14
9	9	12	7	10	20	15	21	18
0	15	10	0	0	11	7	0	0
35	33	35	35	29	22	29	21	19
27	37	32	33	38	26	30	19	12
16	15	18	23	21	23	26	21	15
17	13	14	21	17	14	18	16	11
11	7	7	9	8	11	11	13	12
9	8	13	8	8	9	8	6	8
0	0	0	0	0	5	0	0	3
3	5	0	0	0	0	0	0	0
10	15	10	0	0	0	0	0	0
34	49	33	44	45	30	33	19	22
52	39	55	42	47	20	34	22	18
45	35	78	30	32	13	36	23	19
32	36	41	45	49	23	32	23	20
30	24	36	38	42	23	33	21	18
20	20	21	19	22	13	20	12	17
10	13	18	21	19	21	17	13	14

19	14	16	13	13	16	18	15	13
6	11	14	12	6	18	28	26	18
14	7	13	7	14	13	14	23	10
12	8	16	9	9	16	13	17	15
11	15	7	6	6	6	6	7	8
13	16	17	16	15	19	17	15	16
15	0	25	15	16	19	0	21	0
45	31	43	48	54	33	48	39	23
16	12	16	20	21	19	22	17	10
9	13	23	25	24	20	18	13	14
25	20	20	21	18	13	18	10	9
14	8	14	13	15	18	17	17	11
8	11	20	25	22	20	22	20	13
15	13	9	9	8	9	9	10	8
13	20	11	7	9	10	9	7	9
3	0	0	0	0	0	0	4	6
28	28	35	35	41	22	30	17	17
24	20	28	26	26	20	23	18	15
25	23	27	22	30	10	5	9	9
17	16	20	20	21	15	19	16	13
4	0	7	8	5	10	4	13	9
20	13	12	12	10	8	10	9	9
15	5	14	13	17	13	14	11	13
10	13	11	11	9	18	14	19	11
18	13	10	15	12	10	9	13	10
8	9	7	10	8	7	7	7	7
7	6	6	5	5	11	9	9	9
3	4	2	6	4	9	5	13	7
8	5	4	5	8	6	7	7	8
9	8	9	11	10	11	12	9	11
3	1	2	2	1	5	1	8	7
0	0	0	0	0	6	9	7	7
15	9	30	16	24	15	17	18	11
20	16	20	15	15	6	13	6	6
13	11	17	21	16	16	24	13	12
9	15	13	20	25	13	12	10	10
8	7	11	13	12	11	12	9	8
5	5	8	12	14	13	11	14	7
11	7	8	12	6	5	9	11	8
13	9	9	12	10	6	10	10	6
5	5	5	5	5	10	6	4	11
8	8	7	7	6	11	4	16	6
6	5	5	7	6	9	5	5	9
3	7	8	9	11	10	12	7	9
2	2	5	8	4	12	9	11	8
0	0	4	4	0	10	6	18	14
25	24	34	37	43	21	32	18	17

3	2	4	5	6	10	6	7	7
3	3	2	2	2	4	3	5	4
21	15	16	17	15	13	14	11	8
16	22	16	19	19	15	12	12	11
18	15	19	17	17	13	17	14	10
12	13	11	12	11	11	11	16	7
12	13	12	6	9	14	11	13	11
15	15	10	9	10	9	10	8	8
9	10	9	10	13	11	10	9	10
11	8	8	8	9	7	8	8	12
6	7	7	6	6	9	8	12	13
8	7	5	8	7	9	7	10	8
10	9	6	10	11	12	10	13	14
9	8	9	9	10	5	4	4	4
10	5	2	3	2	3	4	4	2
2	4	4	5	10	7	7	9	7
21	18	15	19	13	15	18	17	12
8	0	0	4	7	11	6	8	3
0	8	11	11	9	8	9	11	9
46	43	57	34	40	18	29	30	13
24	27	37	23	31	13	25	12	21
21	28	24	17	21	12	19	13	10
22	15	27	25	17	11	21	14	12
16	11	21	26	22	11	19	19	9
15	14	26	21	22	12	22	14	9
11	10	19	18	16	12	15	8	9
17	9	15	13	16	9	15	8	9
8	7	12	16	16	15	15	18	9
8	7	14	14	15	11	8	13	8
12	13	21	14	20	13	14	8	9
8	6	11	11	12	12	11	10	10
15	13	11	13	10	6	10	7	10
10	8	10	18	14	11	12	11	9
5	7	10	10	9	7	7	11	9
12	11	7	13	13	6	8	6	6
5	9	6	9	9	7	10	10	7
17	8	13	7	9	7	7	3	4
10	5	8	7	4	6	2	5	5
3	6	6	7	8	9	9	8	7
1	0	1	9	4	7	4	7	8
3	7	4	6	5	9	7	9	8
2	1	4	6	9	11	3	11	5
3	3	3	4	5	7	4	6	6
2	1	0	3	3	11	5	7	13
5	4	4	5	4	6	3	5	6
8	6	4	7	1	2	1	2	2
6	0	3	2	3	4	2	5	4

6	5	5	4	7	11	6	5	4
15	20	22	8	12	30	32	26	15
16	14	22	19	19	12	15	14	6
17	14	17	14	17	6	13	9	8
14	8	21	12	17	8	17	6	11
14	13	15	13	11	9	14	9	9
12	13	16	10	11	10	12	7	7
8	10	20	11	15	9	14	10	6
10	8	6	11	8	16	11	14	6
10	10	10	12	11	8	11	5	7
5	2	6	8	5	10	13	10	8
9	5	7	7	8	8	11	8	8
5	5	7	6	5	6	6	11	9
11	9	5	11	8	7	9	7	8
8	10	9	9	10	6	10	6	5
6	7	9	10	9	7	11	6	5
6	1	6	6	5	7	10	6	2
2	2	5	3	2	12	7	7	10
6	7	7	4	5	5	5	5	6
7	7	4	5	4	3	4	2	4
5	3	6	7	6	10	8	3	6
9	5	3	8	6	2	2	4	4
4	7	4	3	5	5	3	4	3
6	0	8	7	6	6	6	7	7
4	3	2	3	3	8	4	7	4
3	2	1	3	0	5	3	8	2
3	2	2	5	6	4	4	5	5
0	0	0	10	0	0	7	2	0
0	4	7	0	0	11	15	13	11
6	0	0	4	3	0	0	5	6
26	20	38	29	42	15	25	23	13
19	28	33	30	37	26	30	15	11
30	36	17	23	24	5	15	14	5
18	18	21	14	16	12	10	11	10
18	18	22	18	24	12	20	11	5
20	19	16	17	17	12	15	14	11
17	14	18	19	20	13	16	12	4
15	19	23	22	21	17	15	9	5
13	15	19	16	18	11	14	9	9
10	12	13	16	15	14	15	10	4
15	22	15	22	21	6	9	5	2
9	10	11	6	11	14	12	18	8
14	10	11	13	13	9	9	10	4
7	12	11	9	8	11	9	4	6
17	14	14	13	14	5	10	4	3
9	8	10	13	8	6	7	6	6
8	2	7	8	9	8	3	10	6

7	9	8	12	11	9	9	7	8
9	5	5	10	10	8	12	10	8
10	10	10	5	6	6	4	4	4
3	4	10	6	6	13	11	13	8
10	12	11	10	9	5	7	4	3
4	3	5	2	3	8	7	5	7
8	7	8	6	7	4	6	3	3
4	3	4	3	4	7	8	10	9
5	7	6	6	5	4	11	2	2
3	6	6	5	6	5	5	6	4
6	3	4	3	4	4	3	4	5
0	1	6	2	8	7	3	2	3
3	2	2	3	2	2	5	9	4
1	0	1	1	2	2	4	2	4
4	2	3	4	2	2	2	2	0
7	6	7	6	6	7	9	9	9
1	0	1	2	0	4	1	6	2
2	2	2	2	1	2	1	1	3
1	0	0	2	0	1	2	4	3
0	0	0	0	0	5	0	3	1
0	1	0	0	0	1	0	2	2
2	0	1	1	0	2	2	4	3
0	0	0	0	0	1	0	0	1
17	19	26	23	24	12	28	15	12
13	13	17	15	17	5	9	6	7
10	10	14	6	16	13	9	6	7
9	11	9	12	13	12	15	8	8
12	6	13	10	11	7	6	7	3
10	12	10	6	10	3	5	3	6
9	10	11	9	10	5	7	3	3
6	4	7	6	10	5	8	7	3
5	8	13	4	14	2	10	7	5
3	0	0	1	0	0	0	0	0
9	5	11	9	12	11	12	6	5
8	6	7	9	8	5	5	6	6
3	5	7	6	8	10	9	10	5
8	7	8	9	9	4	11	6	9
3	2	9	9	10	7	6	11	4
4	6	9	8	7	8	5	6	7
7	4	7	10	9	4	4	5	3
4	3	6	2	5	8	5	8	4
6	6	4	3	3	5	4	6	4
3	3	3	8	5	6	5	7	10
5	5	6	6	4	4	4	3	2
3	3	1	1	2	5	3	7	9
5	2	8	7	9	2	3	4	1
6	4	4	7	5	4	4	3	2

4	0	5	4	4	6	5	6	5
1	1	4	1	3	7	3	5	6
5	4	3	2	4	3	2	3	4
4	2	2	5	2	3	2	7	6
2	0	2	5	2	4	4	5	5
2	1	1	1	2	8	1	7	4
5	5	3	3	2	2	2	1	2
4	1	2	1	1	3	3	5	3
1	2	2	2	2	2	5	8	1
1	0	1	2	1	4	4	6	7
2	3	4	5	3	3	3	3	3
0	3	1	1	2	1	2	4	3
2	0	0	3	0	2	0	1	6
15	7	12	2	1	30	38	27	21
14	15	24	28	28	10	18	7	8
23	17	19	27	26	10	16	11	10
11	12	25	23	24	9	19	9	6
10	10	14	15	19	10	14	8	9
8	11	9	13	13	17	18	7	8
11	12	13	11	10	10	13	14	8
5	3	19	11	10	15	15	13	11
10	8	10	14	13	10	11	10	11
10	10	10	10	11	14	10	3	7
11	9	19	21	21	6	9	7	3
9	9	14	12	16	5	11	6	5
18	3	15	13	13	5	7	8	3
11	10	12	16	12	7	10	5	6
5	7	10	7	9	13	11	13	8
6	5	14	13	9	9	11	6	4
7	6	8	9	7	12	4	9	3
8	6	10	12	10	6	9	7	4
3	9	11	7	6	9	7	7	6
7	7	10	6	11	8	12	4	1
5	3	6	7	6	4	6	4	6
13	11	6	7	6	4	3	5	4
5	2	7	5	7	4	3	8	3
14	13	20	21	16	7	12	9	5
6	5	7	7	8	9	9	7	6
3	5	4	6	9	6	5	5	5
6	13	9	5	6	4	7	3	1
3	6	5	5	6	9	8	7	6
6	7	11	5	6	2	3	3	3
4	1	2	2	5	3	6	6	7
1	5	4	5	6	8	8	6	3
6	1	5	8	5	3	6	5	1
5	9	6	7	5	3	1	6	2
8	4	6	4	7	4	3	3	3

6	6	3	8	4	5	5	2	5
3	4	5	4	5	5	8	5	3
1	6	4	3	4	4	4	4	2
6	5	5	7	5	5	4	5	1
3	1	4	1	4	5	6	1	1
5	13	6	9	5	6	6	2	6
9	5	4	6	5	5	5	5	2
4	2	7	8	7	5	5	4	1
5	6	3	7	6	5	3	6	1
3	2	4	3	5	5	5	7	1
4	3	4	5	5	6	6	6	4
5	7	5	5	5	2	2	3	0
2	4	3	2	2	3	4	3	4
3	2	11	6	6	7	2	5	1
1	2	0	0	0	12	10	13	8
3	1	5	3	5	4	4	3	2
2	4	1	3	0	3	4	7	6
3	1	4	4	6	2	4	4	2
7	7	21	16	12	10	16	7	5
6	4	2	4	4	2	3	0	2
2	1	5	2	4	5	3	4	3
3	1	3	1	1	3	2	1	6
1	1	3	1	1	4	0	3	6
1	1	2	1	1	2	1	4	3
2	3	3	1	2	4	4	6	2
2	1	2	1	0	1	3	1	2
2	1	0	0	1	4	0	2	2
4	4	1	1	3	3	2	2	3
3	3	3	3	2	2	2	3	3
1	2	1	3	2	2	2	4	2
0	0	1	0	0	5	4	4	5
1	0	0	1	0	3	0	2	1
0	1	1	0	1	3	3	2	2
0	1	0	3	2	2	1	1	1
0	0	0	0	0	2	0	1	4
5	2	4	5	6	6	5	5	8
2	0	0	0	0	5	6	6	5
4	4	0	8	9	7	7	9	4
3	5	0	0	0	3	3	3	0
0	0	8	3	3	5	4	2	5
0	2	5	0	6	6	4	7	2
7	5	9	0	7	0	0	0	3
0	3	0	2	0	3	0	4	3
26	19	20	20	23	12	23	7	5
13	12	27	21	23	18	16	10	11
12	11	12	15	10	5	10	8	6
18	16	23	20	23	4	12	4	7

10	20	25	22	16	7	19	11	5
11	13	17	17	15	9	13	8	3
8	9	23	19	24	8	18	6	10
13	7	11	16	5	11	17	8	4
10	9	18	14	14	17	13	13	2
8	8	12	14	10	8	15	11	4
15	9	15	12	10	5	12	7	3
14	12	9	8	9	6	10	4	5
14	10	11	11	13	8	11	6	5
1	2	10	7	2	8	31	8	10
6	8	16	10	9	9	13	4	5
14	11	14	16	13	8	19	9	3
14	6	7	10	9	5	5	5	4
10	9	10	11	8	3	10	7	4
5	6	13	6	12	9	12	9	3
4	6	6	10	9	9	10	4	0
2	6	1	0	1	13	20	16	16
5	4	8	7	7	5	7	8	6
7	10	5	9	7	9	14	9	7
7	6	6	11	6	7	7	8	11
12	4	12	7	5	4	9	5	2
4	8	13	11	14	5	9	2	4
13	15	10	10	8	3	3	5	4
6	8	11	8	9	6	6	6	7
7	7	6	5	7	7	6	6	8
4	2	2	4	3	10	5	6	6
5	6	8	5	6	7	7	4	3
1	2	3	0	0	15	15	16	6
2	2	6	6	5	5	5	3	6
5	4	3	3	4	6	5	3	2
4	2	7	13	13	5	8	6	1
2	1	6	3	3	5	4	5	5
7	3	7	7	7	4	5	6	4
6	9	6	7	10	2	5	0	2
7	6	5	8	5	5	3	2	2
3	3	2	5	2	5	4	6	9
6	4	5	4	6	3	4	1	1
5	2	1	1	1	5	6	6	3
5	3	4	4	4	9	4	4	3
4	5	8	4	7	2	10	3	1
0	2	4	2	3	12	5	10	3
1	1	0	0	0	15	12	15	10
2	4	3	3	4	5	4	4	5
3	4	6	6	5	4	5	3	1
0	2	0	0	0	5	10	5	6
4	4	4	2	3	2	2	1	2
8	7	7	9	7	0	4	4	2

1	2	3	3	2	6	6	2	3
4	2	2	6	4	3	5	2	1
2	0	2	3	2	4	3	4	2
7	6	2	1	2	2	2	1	1
2	0	8	8	9	7	5	1	5
5	4	2	3	2	3	1	2	3
2	1	4	1	3	7	3	2	1
3	4	3	4	3	1	5	1	0
3	1	4	4	5	3	6	2	4
1	5	3	3	2	5	3	3	5
3	5	7	5	4	1	3	0	2
1	0	2	3	3	5	4	3	3
5	2	2	3	5	2	2	4	2
3	2	3	2	2	3	3	3	2
0	1	0	1	0	7	5	5	2
3	2	7	1	4	6	3	2	2
4	4	2	1	2	5	3	3	2
4	4	2	3	5	2	3	3	1
0	0	0	1	0	2	1	8	3
4	1	1	1	3	2	2	1	0
2	1	2	2	1	2	3	5	4
2	2	1	2	3	4	2	4	4
2	4	4	3	5	2	2	1	1
2	0	1	2	1	4	3	3	2
2	2	1	0	0	2	1	1	3
0	1	1	1	1	3	2	2	4
1	2	2	1	2	3	2	2	3
2	1	1	0	1	2	2	3	2
1	2	1	2	1	2	1	2	1
3	2	5	3	3	0	3	0	1
2	0	2	3	1	3	3	2	1
1	0	2	3	2	1	3	3	1
1	1	0	0	0	2	2	2	1
0	1	1	2	1	3	2	4	4
0	0	0	1	0	4	2	3	1
1	0	1	0	1	3	2	6	1
2	2	3	1	3	2	1	1	2
2	2	1	1	1	3	3	2	2
2	2	1	0	0	3	0	1	3
0	0	0	2	1	2	4	4	5
0	0	1	3	1	2	1	4	2
1	2	1	1	0	2	0	1	3
1	3	4	4	4	4	3	4	2
0	0	0	0	1	4	3	4	5
1	1	3	0	2	2	2	1	1
3	4	2	1	1	0	0	0	0
0	1	1	1	1	2	1	2	0

0	0	1	1	1	2	3	0	2
1	0	0	1	0	1	0	2	0
2	0	0	1	0	0	1	1	1
2	3	1	3	3	7	3	7	2
3	3	1	0	0	2	2	0	1
2	1	1	0	0	1	0	1	2
0	4	6	6	6	6	5	5	4
0	0	0	5	3	3	5	0	0
0	0	0	0	0	2	1	4	0
0	0	0	0	0	1	0	0	1
0	3	7	3	3	6	5	3	4
1	1	0	0	1	0	1	0	0
1	1	0	0	0	0	0	1	0
0	0	0	0	0	1	1	1	1
1	0	0	0	0	2	3	4	4
1	0	0	0	0	0	0	1	2
0	0	0	0	0	0	0	0	0
0	0	0	0	0	0	0	3	0
0	0	0	0	0	1	0	0	0
0	0	0	0	0	0	0	0	0
12	15	20	25	25	12	17	11	6
12	13	13	16	11	7	9	10	4
9	11	13	8	10	9	9	2	3
5	5	13	5	7	10	9	6	5
8	6	11	12	13	7	5	4	5
6	4	11	9	11	7	12	8	6
3	6	7	7	4	8	7	4	2
5	9	10	8	9	7	7	5	2
9	4	10	5	7	3	6	6	0
3	3	7	17	12	5	8	8	6
7	6	12	5	7	9	10	7	3
8	4	6	9	7	6	6	6	1
4	7	8	5	5	6	7	5	1
3	4	5	7	6	8	5	9	5
5	5	7	9	10	7	8	4	2
2	1	5	8	7	4	4	6	6
6	4	8	4	6	8	7	6	3
5	6	5	7	10	5	6	3	3
3	4	3	3	5	5	4	6	3
9	4	11	7	6	2	7	2	0
6	3	10	11	11	5	5	3	2
5	3	8	9	8	3	5	6	3
5	4	7	4	7	5	6	3	4
6	11	5	7	11	3	5	3	1
4	6	10	6	9	2	8	2	2
3	7	4	6	4	4	5	5	3
2	2	2	6	6	5	5	4	2

4	2	10	5	10	2	6	5	1
4	3	5	6	6	2	7	4	3
7	11	9	5	12	2	4	3	2
2	5	7	5	4	4	6	2	5
4	2	6	5	5	1	2	2	2
6	3	3	2	3	7	5	4	0
6	7	3	4	2	3	3	1	5
4	5	2	4	2	3	3	2	3
1	1	6	2	3	7	6	5	5
0	1	0	2	1	9	3	4	6
4	3	3	4	2	3	2	4	2
4	0	1	1	7	3	2	4	1
5	4	4	4	5	4	5	4	3
3	0	2	3	1	4	2	3	4
2	2	2	3	1	5	3	4	3
1	1	2	5	4	4	1	1	1
4	2	2	2	2	4	2	3	2
2	3	6	3	5	2	2	1	2
6	4	3	3	3	3	2	3	2
3	4	3	2	3	2	2	2	1
3	5	3	2	1	4	4	2	1
5	3	4	6	4	4	4	1	2
5	2	3	4	4	4	4	2	3
4	2	4	4	5	2	1	3	2
0	0	1	2	0	7	1	5	4
2	1	2	1	1	6	3	5	6
5	3	2	5	3	2	3	3	3
4	2	4	3	3	2	1	2	1
1	1	1	1	0	4	3	5	4
2	2	3	2	2	4	4	2	4
3	3	3	3	3	2	4	2	2
1	1	1	0	2	2	2	3	2
2	2	3	2	2	2	0	2	4
6	3	1	5	3	1	1	1	1
2	0	0	2	4	4	3	1	3
4	3	3	1	3	4	3	3	2
4	0	2	5	3	5	3	1	4
4	2	2	3	2	3	1	4	2
5	3	2	2	2	2	2	1	1
0	1	1	1	2	3	3	5	1
2	2	4	2	2	4	3	3	2
3	2	2	1	1	2	3	3	0
2	2	7	4	2	2	3	1	1
2	4	3	4	3	3	3	2	2
1	3	1	1	2	3	4	3	2
2	0	2	2	1	5	3	1	3
1	2	3	1	2	3	2	1	2

3	1	2	1	4	4	2	4	2
2	2	2	2	1	2	3	3	1
3	2	1	3	2	1	1	1	1
2	0	3	1	5	4	5	4	5
1	1	1	3	3	2	2	4	4
2	4	2	2	2	3	3	1	2
1	1	2	2	2	1	2	2	0
0	2	1	1	2	7	5	4	4
1	4	2	4	3	1	2	1	1
0	0	0	0	1	3	5	3	1
1	1	1	1	1	1	2	1	4
1	1	2	0	3	3	1	2	2
2	1	2	2	2	2	2	1	1
1	0	1	1	2	3	3	1	1
0	1	2	1	0	2	4	1	2
1	1	2	2	1	1	2	2	2
1	0	2	0	2	4	5	2	2
3	3	1	0	1	1	2	0	0
1	1	0	1	2	3	2	3	2
3	1	0	2	1	3	0	2	1
2	0	1	3	2	2	4	1	1
1	2	1	1	1	1	2	1	1
2	2	0	3	0	0	0	0	0
0	0	0	0	0	1	0	1	1
1	1	2	1	0	0	1	2	3
1	1	2	1	1	4	3	3	1
2	1	1	1	1	1	1	3	2
1	1	1	1	2	1	1	3	2
0	1	0	0	0	4	0	3	2
0	0	0	0	0	3	1	5	4
3	0	0	1	0	3	2	1	0
1	1	1	1	0	3	1	2	2
2	1	4	2	4	1	3	2	3
1	1	5	1	1	2	1	1	1
0	0	1	2	1	1	1	3	3
3	2	2	1	1	2	2	1	1
1	1	0	0	1	1	1	1	1
0	0	0	0	0	0	0	3	2
0	0	0	1	0	4	1	2	2
0	0	2	1	2	1	1	2	2
2	0	0	0	1	2	1	1	2
0	1	1	0	3	1	2	1	2
1	1	0	0	0	2	1	0	1
1	1	1	1	1	1	1	2	2
2	1	2	1	1	1	1	1	0
1	2	1	1	2	0	0	1	1
1	1	0	0	1	4	0	1	1

1	0	0	2	1	2	0	1	0
1	1	0	1	1	2	2	1	1
0	1	0	0	1	3	0	1	2
1	1	0	2	1	1	1	1	0
2	3	5	0	3	0	5	3	0
2	2	2	0	2	2	2	2	3
1	1	3	1	1	3	2	0	0
0	2	1	1	1	1	2	1	0
1	0	0	0	0	2	0	0	2
0	0	0	0	0	1	1	1	0
0	0	0	1	0	1	0	2	1
1	0	0	0	0	2	1	4	2
2	2	0	1	2	3	0	0	2
1	1	0	0	0	3	2	1	1
0	1	1	0	0	2	0	0	0
1	3	1	0	0	0	0	0	0
0	0	0	0	0	2	1	4	0
1	1	1	3	0	3	1	0	1
0	0	3	2	2	2	0	2	0
0	0	1	0	0	0	0	1	4
0	1	0	0	0	0	0	0	0
0	0	0	0	0	0	0	2	6
0	0	1	1	1	0	0	2	0
0	0	0	0	0	0	0	0	0
0	2	0	0	1	0	0	0	1
0	0	0	0	0	4	0	0	0
0	2	1	0	1	1	0	0	0
1	0	0	1	0	1	1	0	0
0	2	0	0	0	0	0	0	0
0	1	0	0	0	0	0	0	1
0	1	0	0	0	0	0	0	0
0	0	0	0	0	4	0	2	0
0	0	0	0	0	0	0	2	0
0	0	0	0	0	0	0	0	3
0	0	0	3	0	3	0	2	0
0	0	0	0	0	0	0	0	1
1	0	0	0	0	0	0	0	0
0	0	0	0	0	0	0	0	0
0	0	0	0	0	0	0	0	1
0	0	0	0	0	0	0	0	1
7	21	49	52	36	4	26	14	3
19	14	31	29	32	11	12	14	3
14	18	30	21	24	6	19	7	7
12	14	18	21	25	8	22	6	5
14	5	17	11	16	5	14	15	1
12	7	15	14	22	9	14	6	6
8	8	30	9	28	5	5	2	1

4	8	24	17	22	10	11	7	5
8	4	13	13	14	8	8	5	6
11	11	13	7	9	9	9	3	4
9	8	18	19	17	9	12	5	4
7	4	12	13	16	7	4	6	4
8	11	13	5	16	5	11	9	1
8	13	8	10	11	8	13	8	3
3	5	1	22	1	5	21	11	7
10	8	11	10	10	2	8	2	3
7	10	15	12	12	6	13	11	0
5	8	7	13	10	9	7	4	5
4	3	11	15	14	7	4	2	4
3	12	12	6	7	3	8	3	4
6	6	3	3	6	7	6	4	1
3	5	7	4	7	3	5	3	2
7	4	8	6	9	5	4	4	1
9	4	5	9	8	6	5	4	4
0	1	5	2	9	10	5	8	6
4	4	8	9	9	4	7	4	2
7	3	8	5	9	1	6	1	0
3	6	7	11	10	4	6	6	3
0	0	0	0	0	2	0	0	1
5	4	4	5	6	3	1	1	1
4	6	6	5	8	7	5	6	1
6	8	6	6	7	0	3	1	2
5	6	13	11	15	1	5	4	2
4	5	5	5	5	3	3	4	2
8	9	6	2	5	2	4	1	1
2	3	12	3	9	5	6	3	1
2	3	4	4	3	6	4	3	5
3	2	3	6	8	3	10	2	0
2	4	8	2	4	2	5	1	0
3	5	7	8	7	4	5	1	2
1	7	6	5	6	5	9	5	1
3	1	9	2	9	3	7	3	0
4	3	3	6	4	2	3	6	5
3	4	4	5	3	6	4	6	3
3	5	2	6	7	3	5	3	0
2	1	4	4	1	6	6	6	2
2	3	8	4	7	2	8	4	1
2	2	5	3	6	6	8	4	1
3	1	9	3	5	5	4	3	1
2	4	5	7	5	5	4	2	3
3	2	4	1	1	3	4	2	2
1	2	1	1	1	4	6	10	3
3	2	1	6	3	2	5	2	1
1	4	5	3	7	4	5	3	4

2	0	2	2	3	3	4	7	7
0	2	1	2	3	2	2	4	2
4	4	8	7	3	1	4	1	2
4	4	4	2	1	0	3	2	1
1	1	5	1	2	5	5	2	4
4	2	2	4	3	0	4	2	3
2	1	8	3	5	3	2	0	2
3	0	2	4	4	5	2	3	4
1	0	5	2	4	3	5	2	1
1	1	5	6	6	4	4	2	3
3	3	3	3	3	1	2	4	2
5	2	6	5	2	2	2	1	1
2	1	4	4	7	1	2	1	1
2	2	4	5	3	2	4	2	1
1	3	3	3	3	3	3	2	4
2	3	5	5	4	1	3	0	3
4	1	5	2	1	3	4	3	2
0	1	1	3	0	4	3	2	1
0	0	2	4	2	5	5	5	5
3	0	1	3	2	2	2	2	1
2	5	3	3	6	2	3	1	1
4	6	7	2	3	2	4	2	1
1	0	1	0	1	2	3	3	5
1	2	1	3	2	4	2	3	3
5	5	4	4	7	1	2	2	0
2	4	3	5	3	1	2	3	1
1	1	4	3	2	4	0	2	5
0	0	1	1	4	8	1	3	2
3	2	4	2	4	3	1	1	3
3	2	5	4	5	1	4	3	1
1	2	5	2	2	6	1	4	2
0	0	1	0	0	8	2	2	4
3	3	3	2	1	2	1	1	0
2	3	2	2	2	3	2	4	2
2	2	3	2	4	2	5	2	3
1	0	1	1	2	4	3	4	3
2	0	6	0	2	7	3	2	0
3	2	2	2	2	1	2	2	2
5	3	2	4	2	2	2	0	2
1	2	0	0	0	8	3	1	1
2	4	4	4	4	1	3	2	1
3	3	5	3	4	2	5	0	1
0	0	0	0	1	3	2	8	6
4	3	5	5	4	1	1	1	2
4	1	2	3	3	1	2	2	0
2	0	2	2	3	2	3	1	2
0	0	5	5	6	4	2	3	1

1	2	3	2	4	4	3	2	0
3	2	4	4	4	3	5	4	0
0	1	0	1	1	3	2	2	3
1	1	0	1	0	1	1	3	1
0	1	3	3	4	1	1	1	1
1	0	1	2	4	4	3	3	4
3	2	4	4	0	2	1	2	3
3	2	2	2	2	2	4	3	1
3	2	3	1	2	3	3	2	2
3	5	1	2	2	0	0	2	0
2	4	4	4	3	0	3	1	1
3	1	3	2	6	1	2	2	0
1	1	3	3	4	4	3	3	2
1	3	2	3	2	2	2	3	2
1	3	3	3	1	2	1	0	1
0	1	2	1	1	3	2	2	2
1	1	3	2	2	3	3	4	1
2	2	3	2	1	3	3	1	2
1	0	2	2	1	2	2	2	1
1	1	2	2	1	2	3	2	3
1	0	1	2	1	1	1	1	2
1	2	1	1	1	2	1	2	2
0	0	1	0	2	4	4	2	1
2	3	3	2	1	0	2	1	0
1	0	1	1	0	3	0	1	2
1	1	1	2	1	2	2	3	2
1	1	1	2	1	1	1	2	2
1	1	2	2	3	1	3	1	0
0	0	2	1	1	2	1	2	1
1	1	0	2	1	3	1	2	1
1	2	4	5	4	2	3	1	0
3	4	1	1	3	1	2	1	1
1	2	4	2	2	4	3	2	1
5	1	1	2	2	1	0	0	2
1	0	2	3	3	1	0	2	2
1	1	0	2	3	3	4	1	0
2	2	1	1	0	0	1	2	1
0	2	1	0	1	1	1	2	0
1	2	0	1	1	2	0	2	2
0	4	2	1	7	1	1	0	2
2	0	1	1	2	1	1	1	1
0	1	1	2	1	5	3	1	0
0	1	2	3	5	0	3	4	0
0	0	2	2	2	0	0	0	0
2	0	2	2	1	1	0	1	1
3	1	2	1	2	0	1	1	2
2	0	1	2	1	2	1	2	2

0	0	1	2	2	3	2	2	1
2	1	2	3	2	0	1	1	0
0	1	2	1	2	2	1	1	1
0	0	4	3	2	3	2	0	2
2	1	1	3	1	0	1	0	0
2	3	2	2	2	0	1	0	1
3	1	0	1	1	0	0	2	2
1	1	2	0	1	3	0	3	1
1	3	2	2	1	2	3	2	1
1	1	2	1	3	2	3	1	0
3	0	2	1	2	3	0	0	1
1	1	2	1	1	1	1	2	0
1	1	3	1	2	2	2	1	1
0	1	1	6	4	2	1	1	0
1	0	0	1	1	2	1	2	1
1	0	0	1	0	2	2	1	1
1	1	0	0	0	2	1	4	3
1	0	4	1	2	3	0	2	1
0	1	1	1	2	4	2	2	1
0	0	0	1	0	0	2	1	0
0	0	0	0	0	0	0	1	2
1	0	1	0	0	1	1	2	1
1	0	1	1	1	1	1	1	1
1	0	2	2	1	1	1	1	0
0	1	1	4	1	2	1	1	3
0	0	1	2	1	4	2	3	2
0	0	1	0	0	3	1	2	2
1	1	1	1	1	1	1	2	0
1	3	0	1	1	0	0	1	1
1	2	0	0	0	1	1	0	1
0	1	4	6	4	1	1	0	0
1	1	1	0	0	1	1	2	1
2	1	1	1	1	1	1	2	0
2	1	2	2	3	3	1	1	0
1	1	2	1	1	1	1	1	0
0	1	2	0	1	2	2	3	0
1	2	1	1	0	0	1	0	0
0	0	0	0	0	2	0	0	2
1	0	0	0	2	1	1	1	0
1	1	0	1	0	1	0	0	1
0	0	1	0	0	1	1	1	0
0	0	0	1	1	1	12	1	1
0	0	0	0	0	2	2	3	1
2	0	1	0	2	1	0	0	0
0	0	0	0	0	0	0	1	0
1	1	0	1	0	1	1	1	1
1	1	0	1	1	3	1	1	0

1	3	0	2	0	1	0	1	0
1	0	0	0	1	0	0	1	1
1	0	0	0	1	0	0	2	0
0	0	0	0	0	3	0	2	3
1	2	1	1	1	1	1	1	1
0	2	1	1	1	1	1	1	1
1	1	2	1	1	0	1	1	1
0	1	1	0	0	1	1	1	2
0	0	2	0	1	1	2	0	1
0	0	1	1	1	1	1	2	1
0	0	2	1	2	0	0	1	0
0	1	2	0	2	1	2	0	0
0	2	0	1	0	0	0	0	0
0	1	2	0	1	2	0	0	1
1	1	0	0	0	1	0	0	1
0	0	0	0	1	1	2	1	0
0	0	1	1	1	1	1	1	1
1	1	0	2	2	0	0	0	1
0	1	2	1	2	1	1	0	0
2	3	1	1	0	0	1	0	0
2	1	1	1	1	0	2	0	0
0	0	1	0	1	1	2	2	0
0	0	0	2	0	0	0	0	1
0	0	1	1	1	1	1	1	1
1	0	1	0	1	1	1	2	1
1	0	0	0	0	0	0	0	0
1	1	1	0	0	1	1	1	0
0	1	1	1	1	2	1	2	0
0	2	0	0	2	0	1	0	0
0	0	0	1	0	2	2	1	3
1	0	0	0	0	2	1	1	0
0	1	0	0	0	2	2	0	0
1	2	0	0	1	2	1	1	1
0	2	0	0	0	1	1	0	1
0	0	0	0	0	0	2	1	1
0	1	1	1	1	1	1	1	0
1	1	1	0	0	0	0	0	0
1	1	0	3	3	4	4	0	0
0	0	0	1	1	1	1	2	1
1	1	0	0	0	1	0	0	0
1	0	2	0	1	1	2	0	1
0	1	0	0	0	0	0	3	0
0	0	0	0	0	1	1	0	0
1	1	1	0	0	0	0	0	0
0	0	0	0	0	1	2	1	2
1	1	0	0	0	0	0	0	0
0	1	1	2	1	0	0	0	0

1	2	0	2	0	0	0	0	0
0	0	0	0	0	1	2	1	0
0	1	1	0	0	0	0	0	0
0	0	0	0	0	0	0	0	0
1	0	0	0	0	1	1	0	1
1	1	1	0	0	2	0	0	0
1	2	0	0	1	0	1	1	0
1	1	0	0	1	0	1	0	1
0	0	0	0	0	1	0	2	2
1	0	0	0	0	0	0	0	0
1	1	0	0	0	0	0	1	3
1	1	0	0	0	0	0	0	1
0	1	0	0	0	0	0	3	1
0	0	1	0	0	1	0	1	2
1	0	0	0	0	0	0	0	0
0	0	1	1	0	0	0	0	1
0	0	0	0	0	1	1	2	1
1	0	0	0	0	0	0	0	0
0	0	0	1	0	0	0	1	0
0	0	0	1	0	1	0	1	0
2	1	0	0	0	1	1	0	1
1	1	0	0	0	1	0	1	0
1	0	0	0	0	1	0	0	1
0	0	0	0	0	0	0	2	2
0	0	0	0	0	0	1	1	0
2	1	0	0	0	0	0	0	0
0	0	2	0	4	0	0	0	0
0	0	0	0	0	0	0	0	0
0	0	0	1	0	0	0	0	0
0	0	0	0	0	0	0	1	0
0	0	0	0	0	0	0	2	1
2	1	0	0	1	0	0	0	0
0	0	0	0	0	0	0	0	1
0	0	0	0	0	1	0	1	0
0	0	0	0	0	0	0	0	0
0	0	0	0	0	0	0	0	0
0	0	0	0	0	0	0	0	0
0	1	0	0	0	0	0	0	0
0	0	1	0	1	2	0	0	0
12	16	37	25	30	10	20	15	6
12	9	19	12	15	5	16	6	5
10	5	9	12	12	10	14	4	2
15	15	13	14	12	4	7	1	1
9	5	16	13	15	3	13	2	3
0	0	0	0	0	0	0	0	0
6	3	10	9	10	8	5	5	1
12	11	9	8	9	1	8	0	0

8	8	9	7	7	3	6	2	3
8	7	6	11	10	0	5	0	3
3	1	9	2	3	4	14	7	7
5	3	7	8	12	2	7	2	0
4	4	8	7	8	5	7	4	2
7	4	9	12	10	3	5	3	2
1	2	10	3	4	1	4	7	4
4	11	6	8	6	4	4	5	3
4	2	11	7	6	4	4	2	4
4	8	10	7	9	0	7	1	0
5	7	12	9	13	0	8	2	0
8	4	7	6	4	1	7	3	1
5	6	4	6	7	4	8	3	2
8	5	15	18	15	0	0	0	0
3	6	5	7	8	3	7	3	3
8	4	6	7	10	0	4	4	1
3	5	9	8	3	4	7	6	2
1	0	0	1	1	6	5	4	8
4	5	7	6	6	4	3	2	1
2	5	10	6	7	2	9	1	1
8	2	5	5	5	3	1	1	2
4	7	3	3	4	0	3	0	0
2	2	6	4	3	3	2	1	2
5	5	4	3	2	1	3	1	2
6	8	7	6	6	0	3	1	3
1	3	6	5	5	3	5	4	2
0	0	0	0	0	1	0	0	0
3	3	7	4	3	2	6	3	0
3	1	3	5	7	4	4	2	1
2	3	3	5	4	3	3	2	1
0	0	0	1	0	0	0	0	0
4	3	5	4	3	1	4	0	1
3	1	3	1	6	1	2	0	1
7	0	0	11	0	19	6	0	10
2	4	3	4	5	2	5	3	2
0	0	5	4	5	6	1	4	3
7	3	5	2	4	3	0	1	1
5	0	6	2	12	2	3	0	0
4	2	0	4	6	0	3	0	0
4	7	4	5	4	1	2	2	0
6	2	8	8	10	0	0	0	0
3	3	5	3	5	3	6	1	1
1	8	2	3	5	0	3	1	0
4	2	6	4	5	2	2	0	0
2	4	9	6	7	0	1	0	0
0	0	0	0	0	0	0	0	1
3	2	4	3	1	2	4	4	1

5	1	2	1	3	1	1	0	0
2	2	8	15	7	0	3	0	0
0	2	4	1	0	4	5	4	2
0	0	0	0	0	0	0	0	0
1	0	2	3	2	3	3	2	1
2	2	2	3	5	4	2	3	2
0	1	5	4	4	3	4	1	3
3	1	5	4	6	1	1	0	1
0	0	1	1	0	0	0	3	0
3	1	3	5	3	1	2	2	2
0	0	2	1	4	4	3	4	2
4	7	3	0	0	5	2	9	5
1	0	1	0	1	7	5	7	1
2	3	3	2	3	1	2	2	1
2	2	1	1	3	1	3	2	0
3	4	5	3	4	1	2	1	0
1	1	4	4	2	3	4	1	0
1	2	5	3	4	3	1	3	2
1	1	4	4	4	3	4	1	0
2	1	2	4	3	2	1	2	0
0	1	8	0	6	0	3	0	0
3	0	4	1	1	2	1	1	0
4	3	6	1	2	0	3	0	0
1	0	2	3	3	2	2	4	0
3	3	3	2	1	1	1	1	0
2	2	5	3	1	1	2	2	0
0	0	2	1	2	4	0	1	0
5	5	4	4	3	0	1	1	1
1	0	0	3	4	6	3	1	0
2	1	3	2	2	1	1	1	2
2	1	3	2	2	1	3	1	1
2	1	4	1	3	0	3	1	0
0	0	3	1	1	3	3	3	2
6	3	2	0	1	0	0	0	0
2	3	2	3	2	1	2	2	2
1	3	2	1	2	5	2	2	2
0	1	3	2	2	2	2	2	2
0	3	4	0	0	1	4	4	0
1	1	1	2	1	4	3	3	2
1	1	0	3	2	3	1	1	2
1	1	4	7	2	3	6	1	0
1	1	2	6	4	2	3	0	1
2	1	2	3	3	0	0	2	1
1	0	0	3	1	2	5	1	3
6	4	4	4	4	0	0	0	0
0	0	0	0	0	2	4	0	4
1	1	2	0	3	2	3	4	1

4	5	4	3	2	0	0	0	0
0	1	5	0	3	4	5	1	0
0	0	0	0	0	1	3	6	4
1	2	5	2	2	2	4	1	0
1	1	1	3	3	2	3	1	2
4	2	2	1	4	1	4	0	0
2	2	1	0	1	0	1	1	0
4	2	2	2	1	1	1	0	0
3	2	1	4	2	0	3	0	0
0	2	2	2	2	2	1	2	3
0	1	3	3	2	2	2	2	0
0	0	1	3	2	3	3	3	1
1	1	3	4	3	1	3	1	0
0	1	3	3	2	2	2	1	0
2	1	3	1	2	0	1	0	0
0	2	0	0	1	1	1	0	0
0	0	0	0	0	0	0	0	0
1	2	0	2	1	1	0	1	0
0	1	0	2	0	3	0	4	0
1	2	1	2	0	0	4	0	0
0	0	2	3	3	3	2	2	3
2	2	3	2	1	0	1	1	0
0	0	0	0	0	0	0	0	0
3	1	2	1	2	1	0	1	0
1	0	2	1	2	4	6	1	1
2	0	1	2	2	0	0	0	0
0	1	3	2	4	1	3	3	0
1	0	3	0	2	2	2	1	1
0	0	2	1	1	1	2	3	1
2	1	1	0	3	0	0	1	0
0	0	0	1	1	3	2	0	1
0	1	3	4	3	3	2	2	0
2	2	1	1	1	1	1	1	0
0	2	0	0	0	2	0	1	0
2	2	3	1	4	0	0	0	0
0	0	2	2	1	3	0	3	3
0	1	0	4	0	3	1	1	0
2	1	2	2	2	2	2	0	1
1	0	1	1	0	1	1	0	1
0	3	2	2	2	1	2	1	0
2	0	0	2	1	3	1	2	2
3	3	1	3	3	0	3	0	0
1	0	4	1	2	1	0	2	0
0	0	0	0	0	1	1	3	2
1	2	2	2	2	1	3	2	0
2	3	0	2	0	1	0	1	0
1	0	2	0	1	5	0	0	1

1	0	0	3	1	0	0	1	0
4	0	1	2	1	0	0	0	0
0	0	0	0	0	0	0	0	0
1	1	2	1	2	2	2	2	1
1	1	1	0	1	1	1	2	1
1	0	0	1	0	1	1	1	1
4	1	1	1	2	2	0	1	0
1	0	0	0	0	0	0	0	0
1	0	3	1	2	1	5	0	0
1	1	0	2	2	2	1	3	3
5	2	0	3	1	0	1	2	1
0	0	0	0	0	4	0	3	1
0	0	0	0	0	0	0	0	0
2	0	2	2	1	0	2	2	0
0	2	0	0	1	4	1	2	1
0	0	1	6	5	3	1	1	0
0	0	1	0	1	5	0	1	0
0	0	1	0	0	1	2	0	0
1	2	0	0	0	2	2	2	3
2	0	0	2	0	1	2	0	0
4	0	8	0	8	8	9	0	0
1	2	0	1	0	0	0	0	0
0	0	0	0	15	13	0	10	0
1	0	1	1	1	1	1	1	1
1	2	0	1	1	1	2	1	1
1	1	0	1	1	2	2	0	0
1	2	0	3	4	3	4	0	0
1	1	2	3	3	1	2	0	1
3	1	0	0	0	1	0	0	2
1	1	3	0	7	0	0	0	0
0	0	0	0	0	0	0	6	0
0	6	17	8	11	7	13	8	5
0	0	0	0	0	2	2	1	1
1	4	1	0	0	0	1	0	0
0	3	0	0	0	3	1	4	1
2	0	3	2	5	0	1	0	0
0	1	1	0	0	0	1	0	0
0	0	0	0	0	0	0	0	0
0	2	2	2	1	1	2	1	1
1	0	1	2	2	2	2	0	2
0	1	1	1	0	0	2	1	3
1	0	1	1	2	2	2	1	2
0	0	1	1	1	2	1	1	1
0	0	0	2	0	3	1	2	3
1	2	3	0	1	1	4	0	0
0	0	2	1	3	4	1	2	0
0	0	1	0	0	2	1	1	2

0	23	29	0	0	28	25	24	0
0	0	1	1	1	0	0	2	0
1	1	1	3	1	1	2	0	0
0	1	1	3	1	3	1	2	0
1	1	1	0	0	1	2	0	0
0	0	0	1	2	1	1	2	0
1	1	0	0	0	0	1	0	0
0	0	0	0	0	1	3	1	1
0	1	0	0	2	2	0	2	1
1	1	2	1	1	1	1	1	1
1	1	1	3	1	1	2	1	1
3	0	0	3	4	4	4	3	7
2	2	1	0	1	1	2	1	1
0	1	0	0	3	1	0	2	1
2	2	3	2	2	0	1	0	0
1	0	1	1	1	2	3	2	0
1	1	1	0	0	0	1	0	0
0	0	0	0	0	2	0	0	0
0	2	2	0	0	0	0	0	0
0	1	1	3	1	1	1	1	2
1	2	1	2	1	0	0	1	0
1	0	0	0	0	1	2	1	1
1	2	2	0	1	2	3	0	0
0	1	4	3	1	0	1	0	1
2	0	2	0	1	1	2	0	0
0	0	0	2	1	0	2	0	0
0	0	4	0	3	1	1	2	0
0	0	0	0	1	1	0	2	0
0	0	2	0	0	3	3	2	0
0	0	0	0	0	0	0	2	0
1	0	3	0	1	0	0	0	0
0	0	0	4	0	0	0	0	0
0	0	0	0	0	0	0	0	0
0	1	2	1	1	2	1	0	1
2	2	2	1	1	0	0	0	1
1	0	0	1	1	3	0	0	1
0	1	0	1	1	0	0	1	0
0	1	0	2	1	1	0	0	0
3	3	3	1	4	3	0	2	0
2	3	0	0	0	0	1	0	1
0	0	0	0	0	2	0	0	0
1	1	0	0	2	1	1	0	0
1	0	2	3	0	1	0	0	0
1	0	1	1	0	2	1	1	0
0	0	1	0	1	2	1	1	0
2	0	0	1	2	1	2	2	2
1	1	2	3	2	1	1	0	0

0	0	1	2	5	1	2	1	0
1	0	1	3	3	0	1	2	0
0	0	0	1	1	3	3	2	0
2	1	2	3	3	0	0	0	1
0	1	2	0	0	3	0	0	0
0	0	0	0	0	3	2	1	0
2	1	0	1	1	0	1	1	1
2	1	1	1	1	1	1	1	1
0	1	0	1	2	2	1	1	1
1	0	2	1	1	1	1	1	0
1	0	1	2	1	1	1	1	0
1	0	1	2	2	1	2	1	1
0	0	1	1	0	2	2	2	1
1	0	1	2	3	0	1	1	0
2	0	1	0	1	1	0	0	1
1	0	0	0	0	1	1	1	0
0	0	4	0	1	2	0	1	1
1	1	0	0	0	1	0	1	1
0	0	1	0	0	2	0	2	0
1	2	4	2	4	0	1	0	0
1	1	1	1	1	1	1	0	2
1	2	1	0	1	1	1	1	0
0	0	2	2	1	1	1	1	1
1	1	0	2	0	1	2	1	0
2	1	0	1	1	0	0	0	1
0	0	2	1	1	2	0	2	2
0	1	1	0	0	0	1	1	1
0	0	2	3	0	3	4	2	2
0	0	0	0	0	3	1	2	1
1	0	1	0	0	6	0	3	0
0	1	3	0	2	1	4	1	0
0	4	0	0	0	0	0	0	0
0	0	0	0	0	0	0	0	0
0	1	1	1	1	0	3	0	0
1	0	0	0	1	1	1	1	0
0	0	2	1	1	1	0	1	1
1	2	1	2	1	1	0	0	0
0	0	0	1	0	2	1	2	0
1	0	1	1	1	1	1	0	1
0	1	1	2	1	1	1	1	0
1	0	0	2	0	0	1	1	0
0	0	0	2	2	0	0	2	0
1	1	3	3	3	0	0	0	0
0	0	1	2	2	2	1	2	1
0	0	2	0	0	3	0	1	1
0	0	0	0	0	0	0	2	0
0	0	0	2	0	1	0	1	0

0	0	0	0	0	0	0	1	2
0	0	0	0	0	0	1	0	0
0	0	0	0	1	0	0	0	1
0	0	0	2	0	0	0	0	0
0	0	0	0	0	0	0	0	0
1	1	0	1	0	1	1	1	1
1	1	2	0	1	0	0	0	0
0	0	0	1	1	3	1	1	1
1	1	1	0	2	0	0	1	0
2	0	1	0	0	0	1	0	0
0	1	1	1	2	1	1	2	0
0	0	0	2	0	2	2	2	0
0	1	2	0	1	0	0	2	0
0	0	2	3	2	0	1	0	0
0	2	0	0	0	0	1	0	0
0	0	1	0	0	0	0	4	0
1	1	3	2	1	0	2	0	0
0	1	0	0	0	1	1	1	1
1	0	0	0	0	0	0	0	1
2	2	0	0	0	1	1	1	0
0	0	0	1	0	2	1	2	1
2	1	2	0	2	0	1	0	0
0	0	0	2	2	1	0	1	2
0	0	0	0	0	0	1	1	0
1	0	0	0	1	2	0	1	0
0	1	1	0	0	0	5	1	0
0	0	1	0	1	2	3	0	0
0	0	0	0	3	0	5	0	0
1	1	0	0	0	1	0	1	0
1	2	3	1	2	0	3	0	0
0	1	2	2	1	0	1	0	0
0	0	2	0	2	3	3	2	3
2	3	0	1	0	0	0	1	0
2	1	2	2	0	0	0	0	0
0	1	1	3	3	0	1	3	0
0	0	2	2	0	1	1	0	1
0	2	0	0	0	7	2	5	2
2	1	0	0	0	1	1	0	0
0	0	4	2	2	0	1	0	1
0	0	0	0	0	5	0	0	5
2	3	2	0	0	0	1	0	0
0	0	0	0	0	4	0	0	2
0	0	0	4	0	0	0	0	2
0	0	0	0	0	0	0	0	0
0	0	0	1	0	0	0	0	0
2	1	1	1	1	2	1	1	0
0	2	3	1	1	0	2	1	0

0	0	0	1	1	1	1	2	3
0	0	0	0	0	5	1	2	2
0	0	1	2	0	0	1	2	0
0	0	0	0	0	2	1	2	2
0	0	0	0	0	1	0	2	0
0	0	0	0	0	0	0	0	0
0	0	0	0	2	0	0	0	0
0	2	0	0	0	0	0	0	0
0	0	0	0	0	0	0	0	0
0	0	0	0	0	0	0	0	0
0	1	0	1	2	1	2	1	0
0	0	1	1	1	1	2	1	0
1	0	1	3	2	1	0	0	0
1	0	0	0	0	0	1	0	1
0	1	0	1	0	1	0	0	0
2	1	1	2	1	0	1	0	0
0	0	0	0	0	3	0	1	2
1	3	1	0	0	0	1	0	0
0	0	0	0	0	2	0	2	1
2	1	2	1	1	0	0	0	0
0	2	0	1	1	0	0	0	0
0	0	0	0	0	0	1	2	0
0	2	0	0	0	0	0	0	2
0	0	0	0	0	0	0	0	0
0	0	1	1	1	1	1	1	1
2	0	0	0	7	5	4	7	4
0	0	1	0	0	1	1	1	1
0	0	0	1	0	1	1	2	1
0	0	1	2	1	1	2	1	0
0	0	0	2	1	0	1	0	0
0	0	1	2	1	0	1	0	0
0	0	1	1	0	2	2	1	0
0	0	0	0	0	1	0	0	0
0	0	0	0	0	1	1	1	0
2	1	2	1	0	0	0	1	0
1	2	0	0	0	0	0	0	2
0	0	0	0	0	2	0	0	0
0	0	1	0	3	2	1	0	0
0	1	1	0	0	0	0	0	0
0	0	0	0	1	1	2	0	0
0	0	0	0	3	0	0	0	0
0	0	0	0	0	0	1	1	0
0	0	3	0	1	1	2	0	0
0	0	0	0	0	0	0	0	1
0	1	0	0	0	0	0	0	0
2	0	0	0	0	0	0	0	0
0	0	1	1	1	1	0	1	0

2	0	0	0	0	0	0	0	0
0	0	2	1	0	0	1	1	1
1	0	0	0	2	0	1	0	0
0	0	0	0	0	0	0	2	0
1	0	1	1	1	1	2	0	0
0	0	0	1	0	2	0	1	0
0	0	0	0	0	1	1	1	0
0	0	0	0	0	0	0	1	0
0	0	0	0	0	1	0	1	1
0	2	0	0	0	0	1	1	0
0	0	0	0	0	1	0	1	0
3	0	1	1	1	0	0	0	0
1	0	0	0	1	0	0	0	0
2	0	0	0	2	0	0	0	0
0	2	0	0	0	2	0	0	0
0	0	1	1	1	0	0	0	0
1	0	0	0	1	0	1	0	0
0	0	0	1	1	3	2	2	2
0	0	1	1	1	2	1	1	0
0	0	1	0	0	1	1	0	1
0	0	0	1	0	0	0	0	0
0	0	0	0	0	1	0	1	2
0	0	0	1	0	0	0	0	1
0	0	0	0	0	1	1	2	0
0	0	0	0	0	1	7	1	0
0	0	0	0	0	1	0	1	1
0	0	0	0	0	3	5	0	0
0	0	0	0	0	0	0	0	1
0	0	2	0	0	0	1	0	0
0	1	0	0	0	0	0	0	0
0	0	0	0	0	0	0	0	0
0	0	0	0	0	1	0	0	1
0	0	0	0	0	0	0	0	0
0	0	0	0	0	0	0	0	0
0	2	1	0	1	0	1	0	0
0	0	0	0	0	1	1	0	2
0	0	0	0	0	0	0	1	1
0	0	1	0	0	1	1	0	1
0	0	0	0	0	0	0	1	1
0	0	0	1	1	1	2	0	0
0	1	0	1	0	0	1	0	0
0	0	0	0	0	1	0	0	0
0	0	0	0	0	1	0	0	0
3	2	0	0	0	0	0	0	0
0	0	0	1	0	0	0	1	0
1	0	0	2	1	0	0	0	0
1	1	0	0	0	0	0	0	0

0	0	0	0	0	1	1	0	0
2	0	1	0	2	0	0	0	0
0	0	0	0	0	0	0	1	0
0	0	0	0	0	0	0	0	0
0	0	0	0	1	0	0	1	0
0	1	1	2	1	1	1	0	0
0	1	1	2	1	0	0	0	0
0	0	0	0	0	1	1	1	0
2	1	0	0	0	0	0	0	0
0	0	3	1	0	1	0	1	1
0	2	0	0	0	0	0	0	0
1	0	0	0	0	0	0	0	0
0	0	2	0	0	0	0	0	0
0	0	0	0	0	1	1	1	0
0	0	1	0	1	0	0	0	0
0	0	0	0	0	1	0	2	2
0	0	0	1	0	1	0	1	0
0	0	0	1	0	1	0	0	1
0	0	0	0	0	0	0	0	1
0	0	0	0	0	0	1	1	0
0	0	0	0	0	0	0	2	1
1	0	0	0	0	0	0	0	0
0	0	0	0	0	0	0	0	0
0	0	0	0	0	0	0	0	0
0	0	0	0	0	0	0	0	0
0	0	0	0	0	0	0	0	0
0	0	0	1	0	1	0	2	1
0	0	0	1	2	0	1	1	0
1	1	0	0	0	0	0	0	0
2	0	0	0	0	0	2	0	0
0	0	0	0	0	1	2	1	0
0	0	0	0	0	0	2	1	0
47	40	0	0	0	0	0	0	0
0	0	0	0	0	0	6	0	0
0	0	0	0	0	0	0	0	0
0	0	0	0	0	0	0	0	0
0	0	0	0	0	0	0	0	0
0	0	0	0	0	0	0	0	0
0	0	0	0	0	0	0	0	1
0	0	0	0	0	0	0	0	0
0	0	0	0	0	0	0	0	1
1	1	0	0	0	0	2	1	1
0	1	0	0	0	0	1	1	1
0	0	0	0	0	0	0	0	2
0	0	0	0	0	0	0	1	0
0	0	2	0	1	0	0	0	0
0	0	0	0	0	2	0	1	0

3	0	0	0	0	0	0	0	0
0	0	1	0	0	0	0	0	0
0	0	0	0	0	0	0	0	0
0	0	0	0	0	6	0	1	0
0	0	0	0	0	0	0	0	0
0	0	0	0	0	0	0	0	0
0	0	0	0	0	1	1	1	0
0	0	0	0	1	0	0	1	0
0	0	0	1	0	0	0	2	0
0	1	0	1	0	0	0	0	0
1	0	0	0	0	0	0	0	0
0	0	0	0	0	0	0	1	0
0	0	0	0	0	0	1	0	0
0	0	0	0	0	1	0	1	1
0	0	0	0	0	0	0	0	0
1	1	0	0	0	0	1	0	0
0	0	0	0	0	2	1	0	0
0	0	0	2	1	0	0	0	0
0	0	0	0	0	1	1	0	0
0	0	0	0	0	0	0	0	0
0	0	0	0	0	0	0	0	2
0	0	0	0	0	0	0	0	0
0	0	0	0	0	0	0	1	0
0	0	0	0	0	0	0	0	0
0	0	0	0	0	0	0	0	0
0	0	0	0	0	0	0	0	0
1	0	0	2	0	0	0	0	0
0	0	0	0	0	0	2	0	1
0	0	0	0	0	1	0	1	0
0	0	0	0	0	0	0	2	0
0	2	0	0	0	0	0	0	0
0	0	0	0	0	0	0	1	0
2	0	0	0	0	0	0	0	0
1	0	0	0	0	0	0	1	1
0	0	0	0	0	2	0	0	0
3	0	0	0	0	0	0	0	1
0	1	0	0	0	0	0	0	0
0	0	0	0	0	0	0	2	0
0	0	0	0	0	0	0	0	0
0	0	0	0	0	0	0	0	0
0	11	0	0	0	0	0	0	0
0	0	0	0	0	0	0	0	0
0	0	0	0	0	1	0	0	0
0	0	0	0	0	0	0	0	0
0	0	0	0	0	0	0	0	0
0	0	0	0	0	2	0	1	0
0	0	2	1	1	0	0	0	0

0	0	0	0	0	0	0	0	1
0	0	0	0	0	1	0	0	0
2	0	0	0	0	0	0	0	0
1	2	0	0	0	0	0	0	0
0	2	0	0	0	0	0	0	0
0	0	0	0	0	2	0	0	0
0	0	0	0	0	0	0	0	0
0	0	1	4	0	0	0	0	0
0	0	0	0	0	0	0	0	0
0	0	0	2	0	0	0	0	1
0	0	0	0	0	0	0	0	0
0	0	0	0	0	0	0	0	0
0	0	0	0	0	0	0	0	0
0	0	0	0	0	0	0	0	0
0	0	0	0	0	0	0	0	0
0	0	0	0	0	1	0	0	0
0	0	0	0	0	0	0	2	0
0	0	0	0	0	0	0	0	0
0	0	0	0	0	0	0	1	0
0	0	0	2	0	0	0	0	0
0	0	0	0	0	0	0	0	0
0	0	0	0	0	0	0	0	0
0	1	0	0	0	0	0	0	0
0	0	0	0	0	0	0	0	0
0	2	0	0	0	0	0	0	0
0	0	0	0	0	0	6	0	0
0	0	0	0	0	0	3	0	0
0	0	0	0	0	0	0	0	0
0	0	0	0	0	0	0	0	0
0	0	2	0	0	0	0	0	0
0	0	0	0	0	0	0	0	0
0	0	0	0	0	0	0	0	0
0	0	0	0	2	0	0	0	0
0	0	0	2	0	0	0	0	0
0	0	0	0	0	0	0	0	0
0	0	0	0	0	0	0	0	0
0	0	0	0	0	0	0	0	0
0	0	0	0	0	0	2	0	0
0	0	0	0	2	0	0	0	0
0	0	0	0	0	0	2	0	0
0	0	0	0	0	0	2	0	0
2	0	0	0	0	0	0	0	4
0	0	0	0	0	0	0	0	0
0	0	0	0	0	0	0	0	0
0	0	0	0	0	0	0	0	0
0	0	0	0	0	0	0	0	0
0	0	0	0	0	0	0	0	0

0	0	0	0	0	0	0	0	0
0	0	0	0	0	0	0	0	0
0	0	0	0	0	0	0	0	0
0	0	0	0	0	0	0	0	0
0	0	0	0	0	0	0	0	0
0	0	0	0	0	0	0	0	0
0	0	0	0	0	0	0	0	0
0	0	0	0	0	0	0	0	0
0	0	0	0	0	2	0	0	0
0	0	0	0	0	0	0	0	0

Total Spect IgAN E1C1 T20	Total Spect IgAN E1C1 T21	Total Spect IgAN E0C1 T23	Total Spect ATN, other T24	Total Spect ATN, other T25	Total Spect ATN, other T26	Total Spectrum Count ATN, other causes T27
69	63	75	42	55	91	91
40	34	39	45	37	55	40
48	48	52	40	45	67	72
47	39	44	27	33	66	60
47	53	57	45	38	50	42
18	0	6	8	7	6	1
46	68	79	36	54	66	59
29	9	18	23	16	11	7
41	58	65	42	56	64	57
31	15	12	34	20	11	6
39	51	47	43	46	53	49
36	43	57	25	38	50	53
19	23	29	20	22	25	25
30	34	32	27	23	23	24
18	27	36	20	31	32	32
35	62	70	34	44	47	43
36	33	30	26	35	35	38
26	18	15	25	23	17	6
21	30	33	23	26	27	31
26	42	35	33	22	28	30
20	37	37	21	27	29	28
30	33	35	28	34	33	30
7	9	11	7	8	11	11
30	44	51	36	33	44	41
46	64	70	56	56	57	59
9	7	10	4	8	15	12
18	22	20	24	18	24	21
26	18	28	19	23	30	35
35	44	50	28	35	51	40
19	12	20	12	23	24	22
10	14	7	30	5	28	22
9	11	10	8	12	14	13
8	5	0	7	6	5	3
13	29	30	20	22	23	24
25	47	52	17	22	35	20
37	47	47	34	40	47	47
18	22	26	20	22	30	27
15	34	34	14	26	19	16
10	7	9	4	9	14	13
25	46	38	29	35	30	27
27	21	26	28	21	38	39
22	38	35	24	25	21	21
19	21	27	14	21	39	30

13	4	3	19	5	39	33
18	0	0	13	7	0	0
19	0	0	0	16	25	24
0	8	9	0	6	12	10
17	39	46	18	29	20	18
21	42	48	24	34	29	30
10	7	10	13	9	15	23
16	26	23	14	22	23	22
20	20	30	21	17	35	30
13	16	18	14	18	20	21
18	34	25	22	21	30	24
19	21	19	20	15	25	24
18	9	9	12	9	0	7
0	0	0	0	0	0	0
15	30	32	14	26	24	19
15	14	18	16	22	15	16
20	18	21	11	16	14	13
17	25	19	12	13	20	19
11	16	15	8	10	17	14
19	18	16	15	10	14	13
21	25	20	15	22	24	21
22	36	31	24	27	27	23
20	29	27	19	20	18	17
13	59	16	39	29	47	38
14	24	23	13	22	24	24
16	18	23	7	9	20	11
7	14	20	7	12	15	12
8	6	7	16	14	28	15
16	0	20	13	1	33	25
15	15	14	24	22	23	15
0	10	14	0	0	0	12
16	32	26	19	22	15	19
19	30	30	16	18	23	18
20	19	27	17	27	30	23
17	21	18	14	14	17	17
15	11	13	6	13	13	16
12	12	12	8	6	12	13
0	0	2	0	1	0	0
0	0	0	0	0	0	0
0	0	13	0	0	0	0
18	39	33	22	30	28	28
15	34	33	17	21	14	16
21	49	52	16	26	15	10
22	42	33	17	27	21	22
22	35	37	18	23	21	25
12	18	22	11	13	12	14
17	25	21	19	25	13	21

10	19	17	9	15	12	12
18	21	20	12	14	22	15
8	17	15	21	9	20	17
11	14	12	23	14	20	18
6	8	7	7	8	6	6
14	17	17	18	14	20	19
0	20	25	13	0	19	14
23	41	31	24	37	27	26
10	26	23	8	22	30	18
13	28	18	8	16	22	18
10	12	18	8	17	13	11
15	17	17	11	21	22	19
13	29	26	12	17	25	13
8	12	11	8	9	11	12
7	9	9	10	10	6	7
4	0	4	0	5	0	5
20	22	30	17	20	19	18
19	28	26	10	19	21	22
14	27	33	8	10	3	1
11	18	19	17	15	13	14
11	2	12	9	14	27	28
10	13	11	8	9	10	9
10	12	19	9	12	10	11
9	14	12	7	14	14	12
10	13	11	8	8	7	8
7	9	9	5	7	6	8
10	7	6	5	8	8	9
12	4	5	5	7	9	11
9	6	8	7	7	7	8
12	9	13	7	9	14	7
7	7	5	7	8	13	14
5	6	8	5	5	7	11
14	18	21	10	17	13	12
6	11	16	5	12	6	6
15	17	17	11	17	19	14
9	14	15	10	17	11	13
11	17	12	8	12	13	10
9	17	15	9	13	11	14
6	8	7	10	11	15	11
5	11	11	4	9	8	7
8	9	9	4	7	11	12
8	6	7	1	8	8	14
8	4	11	7	10	7	15
7	12	7	10	10	8	8
6	10	12	5	10	11	14
10	7	8	25	16	18	16
18	30	29	17	19	17	21

5	7	9	9	8	9	7
15	2	3	5	5	5	4
9	16	12	7	12	8	12
12	18	17	11	15	14	11
13	18	15	11	12	10	8
12	8	13	11	13	15	10
11	12	11	12	14	14	13
11	10	9	12	12	10	10
9	11	10	8	14	6	7
14	11	11	5	9	16	16
7	8	13	13	9	10	15
12	6	7	8	6	14	11
16	11	12	5	9	12	8
3	8	7	5	5	6	5
3	3	2	3	4	5	3
4	9	11	6	10	9	8
0	14	11	9	16	14	12
5	6	5	6	5	10	8
6	11	11	6	6	6	8
15	41	43	14	22	16	15
13	34	24	10	20	5	10
8	24	14	8	14	5	8
11	21	13	9	18	10	13
10	34	26	12	20	13	12
9	16	18	11	14	14	13
9	18	14	18	17	12	14
12	12	12	7	8	10	12
10	15	15	10	16	14	12
14	14	13	7	10	9	11
12	9	15	7	8	9	9
11	11	12	11	10	12	13
7	10	9	5	6	5	7
7	11	11	11	10	10	10
5	12	11	5	6	6	8
6	9	10	7	7	5	8
9	9	7	7	10	8	10
9	12	9	7	5	3	2
4	5	8	7	7	7	6
7	6	8	7	7	13	15
6	5	6	5	12	9	10
11	7	5	3	9	6	9
8	6	5	6	6	13	15
7	5	7	5	4	8	8
5	1	5	4	3	12	12
9	3	4	2	5	6	4
2	0	2	7	1	3	7
4	3	4	5	4	8	6

6	6	6	5	4	9	7
17	32	24	12	26	31	21
10	18	19	12	11	11	13
8	13	13	11	14	5	12
12	13	19	5	7	8	8
6	12	16	10	8	11	11
9	15	13	6	8	6	8
5	12	18	10	12	9	13
7	9	9	9	12	17	16
7	12	11	7	7	8	6
6	7	9	10	11	10	9
6	9	9	5	7	11	10
11	9	10	12	9	12	9
7	6	7	9	9	11	8
2	13	9	4	9	6	7
5	11	9	7	9	9	7
4	10	7	5	6	11	9
4	3	5	6	11	19	21
8	6	7	8	5	6	8
3	7	5	7	6	4	4
2	5	8	4	6	7	7
5	5	6	6	7	6	7
1	8	6	5	5	8	6
6	4	4	5	1	6	9
3	3	3	9	7	8	8
4	3	4	7	3	18	14
1	4	3	4	5	5	5
6	8	11	1	10	0	5
12	11	9	5	6	11	11
6	5	5	4	3	5	3
12	43	41	10	27	12	17
11	45	30	18	16	9	16
7	25	19	11	14	10	10
9	17	10	9	12	10	12
10	19	16	8	12	6	11
13	16	17	7	16	14	15
8	16	20	7	14	8	10
11	14	20	8	17	9	5
9	16	14	12	15	10	11
7	11	11	9	12	8	6
5	16	17	2	7	3	5
11	20	13	7	14	13	17
5	7	8	8	8	7	6
4	11	11	5	6	12	9
7	10	12	2	5	5	6
5	10	10	4	7	6	4
6	10	9	6	2	9	8

8	9	8	7	10	7	8
6	9	5	3	13	7	6
4	7	9	2	6	3	4
6	10	9	4	13	13	13
7	12	10	4	5	3	5
7	7	9	6	6	7	9
5	6	6	3	4	3	4
5	6	5	9	8	6	9
1	4	9	3	3	5	6
5	7	4	4	5	4	6
7	2	3	4	2	4	4
3	3	6	4	3	8	6
0	2	3	5	2	4	3
2	3	3	4	2	6	7
0	2	1	0	3	3	4
8	7	9	6	8	7	6
2	3	1	11	5	5	5
4	2	1	4	3	2	5
4	0	2	5	3	5	5
0	0	1	0	1	6	10
1	1	4	0	1	6	6
2	2	2	3	1	1	2
1	0	0	0	0	4	3
11	24	25	11	15	9	13
7	18	7	3	8	4	6
9	17	11	2	17	3	10
7	15	11	9	9	8	9
4	11	12	7	10	6	8
7	6	8	5	5	3	5
5	6	11	3	5	5	4
5	10	9	5	5	7	6
2	14	11	3	7	3	4
11	0	0	4	0	0	0
3	12	9	3	10	6	5
7	4	7	5	7	6	5
1	10	9	8	7	9	11
9	8	6	7	10	7	9
4	10	10	7	9	9	7
6	7	7	5	5	5	4
5	6	7	3	4	6	4
4	7	7	6	3	7	9
6	4	5	2	4	5	6
5	6	6	8	5	7	6
4	12	5	2	6	3	4
9	2	3	6	6	9	11
4	8	2	4	5	3	4
0	4	4	5	5	5	5

6	7	9	6	4	7	8
5	3	4	6	2	5	9
3	3	4	2	3	4	4
4	2	1	5	4	3	6
3	3	6	3	4	7	6
4	2	6	5	4	10	8
1	3	3	2	2	4	2
3	2	2	2	4	2	3
1	4	6	3	3	1	5
3	4	3	5	5	9	9
3	3	3	2	2	2	3
1	3	3	3	2	4	4
2	2	6	1	2	5	6
14	29	16	12	21	42	22
6	26	22	6	12	7	11
10	15	11	7	16	7	10
3	18	18	6	24	7	7
5	15	13	7	11	9	10
6	16	11	9	10	7	10
11	13	14	9	13	11	10
13	17	19	11	12	14	11
9	14	12	5	10	10	9
8	13	12	2	6	12	13
5	15	15	2	9	3	2
4	11	11	6	7	6	7
9	10	8	5	10	4	6
5	10	7	5	7	6	7
7	7	9	7	10	10	9
8	13	12	5	7	7	6
2	8	7	6	13	9	11
4	8	9	5	6	4	5
8	8	8	10	8	8	7
1	11	9	5	6	4	3
3	8	5	3	7	7	6
4	4	5	3	4	3	5
3	4	4	4	3	2	3
8	15	19	9	7	8	6
3	8	9	4	6	3	6
5	12	8	3	5	6	6
4	5	4	2	2	2	3
8	13	9	4	5	7	8
4	5	10	0	0	3	2
3	6	7	7	5	9	7
2	5	6	4	5	8	6
3	3	4	5	3	7	4
3	6	4	8	5	4	4
2	6	4	2	3	4	3

5	6	5	4	9	2	4
5	5	5	4	3	2	6
2	3	4	5	2	6	5
3	5	7	0	6	4	2
4	5	3	1	5	5	2
5	6	2	6	7	5	2
2	6	4	3	5	4	3
5	7	5	1	5	3	3
1	4	5	5	4	5	4
3	3	3	2	4	6	5
2	7	4	6	5	6	6
4	3	6	3	1	2	3
2	2	7	2	4	4	5
2	3	3	2	2	1	3
0	3	4	7	4	13	3
1	4	6	0	5	3	3
5	0	1	0	5	5	6
2	7	5	1	4	4	3
8	17	15	5	10	8	8
1	2	1	1	3	4	2
2	4	5	2	1	3	4
3	4	3	3	7	5	5
3	3	1	3	2	9	5
4	2	4	4	2	6	6
2	2	4	2	3	7	6
2	2	3	2	5	4	1
1	1	1	1	0	6	4
0	2	2	4	3	3	3
2	3	2	3	3	3	2
2	4	4	3	3	2	3
3	0	2	1	3	9	5
2	1	1	5	2	8	6
1	2	1	3	1	4	5
4	2	3	3	3	4	4
2	1	4	2	2	4	6
6	6	8	0	9	7	5
2	0	0	1	0	3	1
5	7	7	5	0	7	8
0	0	4	4	0	5	4
2	6	8	3	2	2	7
2	8	6	5	8	6	6
6	7	8	0	0	0	0
3	0	0	0	2	2	3
9	18	24	1	21	4	8
7	23	18	13	15	11	19
5	17	10	7	12	10	8
5	21	16	2	6	6	2

7	14	20	3	9	9	6
5	16	12	6	12	9	9
4	19	15	4	11	3	7
5	20	19	2	15	6	4
2	16	12	7	10	7	9
4	20	10	5	13	7	5
4	13	12	4	11	5	4
6	6	7	3	5	4	2
3	13	9	7	9	5	8
9	8	16	2	15	25	8
3	7	14	5	8	7	6
6	11	9	5	9	11	8
4	8	9	2	6	6	8
3	13	8	2	10	6	3
1	16	9	0	5	14	8
6	11	10	5	5	4	7
6	3	5	8	3	14	12
3	12	7	7	9	4	4
5	11	5	4	7	8	7
8	6	5	4	10	6	4
4	5	7	3	6	3	2
0	10	9	5	10	7	5
1	6	9	4	4	3	4
3	6	8	6	7	9	6
5	7	9	3	6	7	6
2	3	7	9	8	11	11
2	9	6	2	3	3	3
3	2	5	6	1	11	6
3	8	5	5	3	7	5
5	3	6	4	2	5	6
4	7	6	2	7	6	8
3	8	7	2	4	6	8
5	4	5	7	7	6	6
1	6	4	1	3	0	1
3	4	3	2	4	5	3
2	6	3	5	7	8	6
2	4	4	3	4	2	4
2	5	3	2	4	8	9
3	2	4	4	7	4	5
4	3	4	2	5	3	1
3	2	7	5	3	7	9
4	1	0	6	2	11	5
8	3	3	5	3	8	5
3	6	5	2	2	4	3
8	4	5	5	6	4	5
3	5	4	0	2	2	2
2	6	5	2	4	1	0

2	4	4	4	3	7	7
1	3	3	3	3	5	6
0	2	3	6	3	6	3
2	3	2	2	1	1	2
4	7	4	1	3	3	2
3	2	2	3	3	1	2
1	4	4	1	2	8	7
3	4	5	2	3	4	3
3	4	4	2	3	4	3
1	4	4	4	3	3	2
4	3	6	1	2	0	0
2	4	4	3	4	7	7
0	3	3	3	3	2	4
2	3	3	3	3	3	3
2	3	3	5	2	12	10
2	6	3	1	1	3	3
0	1	2	0	2	4	5
2	2	3	3	2	1	3
4	0	2	2	0	6	10
1	2	2	1	3	3	1
0	3	4	2	3	4	4
2	2	3	3	3	3	4
0	2	3	1	3	1	0
2	2	2	3	3	4	3
1	1	2	3	0	1	3
2	2	3	3	2	3	3
1	2	2	1	0	3	3
2	3	1	2	0	3	2
0	1	1	2	2	4	4
1	0	3	0	1	1	0
0	2	3	1	2	4	3
3	3	1	1	2	4	3
3	1	2	1	2	2	4
0	2	1	4	3	3	4
1	0	1	1	3	3	2
0	2	1	3	4	4	5
1	2	2	1	2	3	3
0	1	2	4	1	2	3
4	1	2	1	1	4	0
6	0	0	0	6	6	4
2	1	3	1	1	3	2
2	0	2	1	1	3	4
2	4	4	3	4	4	4
4	3	4	2	2	4	5
1	2	1	1	1	1	1
0	1	1	1	0	0	0
0	2	2	3	2	2	1

1	2	2	1	1	4	2
0	2	2	0	1	3	2
1	2	2	0	1	1	1
5	3	3	3	3	4	6
0	0	0	1	3	1	2
1	0	1	3	1	1	2
0	5	6	5	5	6	6
0	3	3	0	2	3	5
2	0	0	1	1	4	3
3	0	2	1	1	3	5
3	4	5	2	0	0	5
0	0	0	0	2	0	2
0	0	0	0	0	0	0
0	0	0	3	2	0	2
2	1	1	0	0	3	0
0	0	0	0	0	1	0
0	0	0	1	0	2	2
2	0	0	1	0	3	3
0	0	0	0	0	1	1
0	0	0	0	0	0	0
9	20	19	5	11	7	11
5	12	10	4	11	10	5
3	8	6	2	6	4	5
4	7	10	6	8	6	8
3	7	6	2	5	4	4
3	10	11	2	5	4	7
7	12	14	2	7	6	8
3	6	10	3	8	3	6
2	7	7	3	9	3	3
1	7	7	5	6	6	5
1	6	12	5	6	8	8
4	6	7	3	5	4	5
2	8	8	1	6	6	4
8	5	8	6	5	8	10
1	7	7	2	5	4	4
1	8	9	6	7	9	10
2	9	4	6	9	4	6
4	6	6	4	4	4	4
4	4	7	3	5	5	5
0	3	4	2	2	2	1
7	7	5	1	7	3	3
4	9	5	2	5	3	5
4	6	6	2	5	4	3
1	6	4	2	2	1	1
1	11	12	2	4	3	1
5	6	6	6	5	4	4
2	2	4	5	3	4	4

2	7	6	4	4	2	1
2	9	4	1	3	1	3
2	7	8	2	2	4	4
3	7	6	0	2	4	5
3	2	4	0	0	2	3
1	1	0	2	2	3	6
5	3	3	1	2	2	3
4	2	2	2	4	5	3
5	2	6	4	4	6	7
5	3	4	5	5	8	8
1	0	1	3	3	3	3
0	10	1	5	11	1	1
3	3	4	4	4	5	5
3	2	5	1	3	3	5
4	3	2	4	5	3	6
3	7	8	1	5	9	6
2	3	1	3	3	4	1
1	4	3	3	3	0	1
4	3	3	3	3	3	2
1	4	5	3	2	3	3
3	3	4	3	3	2	3
4	2	3	3	3	3	3
1	5	2	2	3	3	3
0	2	4	2	3	2	2
5	2	2	7	1	12	3
3	2	3	4	5	3	6
1	3	2	1	2	3	2
2	5	2	1	3	3	3
3	0	2	5	2	8	6
2	3	4	3	3	3	3
1	3	3	3	2	2	2
6	2	2	3	3	4	5
4	1	4	3	2	6	4
2	3	1	1	1	1	3
0	1	2	1	1	4	7
0	4	3	3	3	3	2
0	4	0	4	6	5	3
2	2	2	2	3	4	2
1	2	3	3	3	1	3
2	2	1	3	3	4	4
2	4	3	2	2	3	4
1	3	3	2	2	1	2
0	3	5	2	1	2	1
2	2	3	1	2	3	3
3	1	1	2	4	4	3
2	1	1	1	5	4	2
2	3	2	1	2	3	3

3	3	3	2	4	2	4
3	5	5	3	3	6	4
1	3	2	0	1	4	4
4	3	0	2	2	3	4
2	2	2	1	3	5	3
3	2	2	2	2	2	2
1	1	2	1	0	2	4
0	0	4	5	4	10	8
3	2	3	2	2	1	1
2	1	1	6	4	3	5
3	1	1	2	1	4	4
0	3	3	2	2	2	3
2	2	2	2	2	2	2
3	1	2	1	4	3	5
2	3	0	3	3	2	3
3	2	2	3	2	1	1
3	1	1	2	2	3	4
0	2	2	0	0	0	0
0	3	1	1	1	4	4
2	0	0	2	1	2	3
1	1	1	0	2	2	1
2	2	3	1	2	2	2
2	0	2	0	0	0	1
1	2	0	8	6	4	3
2	1	2	2	1	3	3
1	2	3	1	0	4	4
2	1	1	2	3	0	1
1	2	1	2	2	2	3
2	0	2	2	3	2	2
2	0	1	1	1	3	5
2	0	0	2	0	3	4
2	1	1	2	1	3	2
3	2	3	3	2	2	2
1	2	3	1	0	0	1
1	1	2	0	1	4	4
0	0	1	1	1	1	1
2	0	0	0	3	0	3
0	0	0	0	0	4	6
0	1	0	5	1	6	8
3	3	1	1	2	2	3
0	0	1	1	0	2	2
1	2	1	3	2	2	1
1	1	0	3	0	1	0
1	1	1	1	1	1	1
2	2	1	1	1	1	1
0	1	1	0	1	2	1
0	2	0	3	3	4	4

2	1	0	1	1	2	2
1	2	1	1	1	3	4
2	0	1	1	2	3	2
1	1	0	1	1	1	1
0	3	4	0	2	1	2
3	2	2	2	2	3	3
1	2	1	0	1	1	1
1	1	1	2	1	1	1
0	0	1	0	0	4	1
2	0	0	1	1	1	2
1	0	0	2	1	1	2
1	0	0	3	2	4	1
2	2	1	2	2	0	2
1	1	0	1	1	3	3
0	1	2	2	0	2	3
0	1	1	0	0	0	1
0	4	1	5	1	0	5
0	2	1	0	2	0	1
4	0	2	0	0	4	3
0	0	0	1	0	4	2
0	1	0	0	0	0	0
1	0	0	2	0	2	0
1	1	1	1	1	2	2
0	0	1	0	1	1	0
0	2	1	0	3	0	0
0	0	1	0	0	2	5
2	0	1	0	0	0	1
0	1	1	0	0	1	2
0	0	0	0	0	0	0
1	0	0	0	0	0	0
1	1	0	0	0	0	1
0	0	0	1	1	4	4
3	0	0	0	0	0	0
1	0	0	0	0	0	0
0	0	0	0	0	3	3
0	0	0	0	0	1	1
0	0	0	0	0	1	1
1	0	0	0	0	1	2
0	0	0	0	0	0	0
0	0	0	1	0	0	0
9	27	34	6	8	8	9
9	28	34	2	21	8	8
4	16	20	5	12	2	8
4	14	18	3	12	4	5
4	14	14	5	9	4	3
6	17	6	6	11	5	8
2	19	8	3	11	2	3

10	15	14	7	5	2	2
8	15	8	6	10	6	3
4	11	10	1	3	2	5
2	13	13	5	10	2	5
4	12	11	6	8	9	5
0	13	11	2	12	4	3
5	8	9	6	8	6	5
0	9	15	1	18	0	0
4	9	9	2	4	1	5
1	13	13	1	8	6	3
5	6	8	7	8	1	7
0	11	5	5	10	6	8
3	11	7	2	6	2	2
3	4	3	2	6	2	4
1	6	6	9	5	4	6
2	8	5	2	4	3	4
1	4	3	4	3	5	5
5	9	12	7	4	9	11
2	9	10	5	4	4	5
1	8	11	0	4	0	0
3	6	7	3	6	5	4
0	0	0	0	0	1	0
1	4	5	0	1	2	2
1	6	5	1	8	6	4
3	5	4	3	3	0	0
5	4	4	1	6	4	1
2	7	5	1	4	4	1
1	5	3	2	2	1	1
1	7	8	3	7	3	1
4	4	4	3	3	3	4
1	6	2	3	3	3	0
1	5	1	0	2	1	0
1	2	2	2	4	1	3
2	8	6	0	5	1	1
0	5	5	0	6	1	0
3	3	4	2	5	1	3
2	4	4	4	4	6	7
1	2	5	2	4	4	2
4	4	4	3	6	10	7
0	4	4	2	3	3	3
0	6	5	1	2	1	3
2	3	4	1	4	3	3
2	2	1	3	4	3	3
2	4	3	1	4	4	3
3	9	7	3	5	5	3
1	3	1	3	4	1	4
2	3	4	4	6	5	5

4	2	2	7	5	5	5
5	4	4	0	2	7	5
1	2	5	2	3	1	2
2	1	2	3	4	0	2
1	5	3	2	5	3	4
1	2	3	2	2	2	2
0	5	4	2	5	4	3
1	5	4	0	4	2	3
2	1	3	0	2	5	2
4	6	3	2	6	3	4
1	1	0	2	3	2	4
2	2	1	0	2	0	1
2	5	8	0	3	2	1
1	7	4	1	2	4	1
2	4	4	3	3	2	3
3	3	5	1	1	4	1
4	3	5	2	3	2	3
3	1	2	1	1	6	8
4	2	3	3	5	6	8
1	2	2	3	2	3	4
2	4	2	0	3	1	2
0	2	2	0	1	1	1
3	4	2	4	1	5	3
3	3	1	2	3	2	2
1	3	2	0	1	1	1
0	2	2	0	0	2	2
4	6	2	0	6	3	2
2	4	3	6	3	9	5
1	5	5	2	3	2	1
0	5	4	1	2	1	1
2	0	4	2	1	5	3
3	2	2	4	2	6	4
1	3	1	1	2	2	2
2	2	2	2	1	2	2
1	4	5	2	2	1	2
2	1	1	4	2	4	1
2	4	3	1	0	3	1
2	1	3	2	2	1	2
0	1	2	1	2	1	2
3	3	4	4	2	4	3
0	3	1	1	3	1	2
0	7	1	1	1	0	0
1	0	3	1	2	2	10
0	1	1	1	2	0	1
1	2	0	0	2	0	1
1	3	3	2	3	4	1
0	7	8	2	4	3	7

1	4	3	1	3	5	3
0	1	2	2	2	1	1
4	1	2	3	2	4	3
2	2	2	1	1	3	5
3	5	3	1	2	1	1
4	2	2	3	2	3	3
2	1	1	0	4	1	1
1	3	2	3	1	5	4
0	2	3	2	5	2	4
0	2	1	1	2	1	1
1	2	2	0	2	1	1
0	2	2	0	0	0	2
2	3	3	2	3	3	2
2	2	2	1	2	1	3
1	3	2	1	2	1	3
1	3	2	3	1	4	5
0	1	2	5	5	4	3
2	3	1	2	2	3	2
1	4	2	1	2	3	1
2	2	3	1	1	2	3
2	3	1	2	2	6	3
1	2	1	2	2	3	2
1	3	3	2	4	3	4
0	3	5	0	3	4	1
2	2	0	1	2	2	0
2	1	1	2	3	3	2
1	3	1	0	2	3	2
1	2	3	0	1	1	2
3	2	3	3	0	3	5
4	1	2	2	1	5	4
2	2	4	1	2	2	2
0	2	1	0	1	2	0
2	2	1	1	1	2	3
1	2	2	0	3	0	0
2	2	2	3	3	2	1
0	1	1	1	2	2	3
0	0	1	2	0	2	3
0	1	0	1	0	2	1
2	2	1	2	2	2	2
0	3	0	1	0	0	0
1	1	2	1	1	1	1
2	2	3	0	2	4	2
1	5	3	0	4	2	0
2	2	1	1	1	0	0
0	1	2	2	1	1	2
0	1	1	0	2	0	1
0	2	1	1	1	1	4

2	2	1	2	1	3	4
0	2	2	1	1	1	2
0	3	2	1	1	2	2
0	2	2	0	0	0	2
0	1	2	0	0	0	0
0	2	1	1	1	1	0
0	0	1	0	2	1	0
0	2	0	0	0	3	3
1	2	2	1	1	1	1
1	1	1	1	0	2	3
0	1	3	0	3	0	1
0	2	2	2	2	2	1
0	3	1	0	1	0	1
1	3	0	0	2	1	1
2	3	3	0	1	2	1
0	2	1	2	1	2	2
0	1	0	3	3	1	3
0	0	3	0	0	1	3
0	1	2	3	3	3	3
0	1	2	0	4	3	2
3	0	0	2	0	0	3
1	1	1	2	3	2	2
1	1	1	0	1	1	1
1	1	3	1	1	1	1
0	1	2	2	0	0	2
1	2	2	2	1	3	2
1	1	0	1	0	0	3
0	2	2	1	2	1	2
1	2	1	0	0	0	1
0	1	0	1	1	2	3
0	0	3	0	0	1	0
1	1	1	1	1	2	1
0	1	1	0	1	1	1
0	1	1	0	2	3	1
1	2	2	2	1	2	2
2	1	3	2	1	0	2
1	1	1	0	0	0	0
1	1	1	0	0	7	4
0	2	1	1	1	1	3
2	0	0	3	2	1	1
2	1	1	1	1	1	1
1	1	0	1	1	1	2
0	0	1	1	3	2	3
2	0	1	0	0	1	0
1	0	0	0	0	0	0
2	1	0	1	1	1	1
0	2	0	0	1	2	0

2	0	0	1	1	1	1
0	0	0	1	0	3	3
3	1	0	0	0	1	0
0	0	0	0	0	5	6
1	1	1	1	1	1	1
1	0	0	1	1	1	1
0	2	0	0	0	2	1
2	0	2	2	0	2	2
0	2	1	1	0	0	0
0	1	1	2	2	2	1
0	2	1	1	1	2	1
1	2	1	0	3	2	3
0	0	0	0	3	0	0
0	2	3	0	2	1	1
0	0	1	0	0	0	0
1	1	2	2	2	3	2
0	1	1	1	0	1	1
1	1	0	0	2	1	0
0	2	1	0	1	0	1
0	0	1	1	0	0	0
0	0	0	0	2	0	0
0	1	1	1	0	3	3
0	1	0	0	3	2	0
1	1	0	1	1	1	1
1	1	1	2	1	1	1
0	0	0	0	0	0	0
1	1	1	0	0	1	2
0	0	0	0	0	2	3
0	1	1	0	1	0	0
1	0	2	1	1	2	1
2	0	1	1	1	4	3
1	1	1	1	1	1	2
0	1	0	1	1	1	1
1	0	0	0	1	1	1
0	3	0	1	4	1	1
0	1	2	1	1	1	1
1	0	1	0	0	1	1
0	0	0	2	1	0	0
1	1	1	1	1	2	1
0	0	1	0	0	2	1
0	2	1	0	1	1	1
2	0	1	0	1	1	1
0	0	0	0	0	0	0
0	2	0	1	0	1	2
1	0	2	1	0	3	3
0	1	1	0	0	0	0
1	0	0	0	0	0	1

0	0	0	0	0	0	0
0	0	0	0	0	2	1
0	0	0	0	0	0	0
0	0	0	0	0	0	0
0	1	0	2	2	2	1
0	1	1	0	1	0	1
0	0	0	0	1	2	1
0	0	0	0	0	0	0
0	0	0	0	0	2	4
0	0	0	0	2	1	1
2	0	0	0	2	0	1
0	1	1	0	0	0	0
0	0	0	1	0	1	0
0	3	1	3	0	1	1
0	0	0	1	0	1	2
1	1	1	0	1	0	0
0	0	0	2	2	2	2
2	0	0	0	0	3	0
0	1	0	0	1	1	3
0	2	0	1	1	1	1
0	1	0	0	1	1	1
0	1	0	0	0	2	2
3	0	0	1	2	0	0
0	0	0	1	0	1	2
1	0	1	1	0	1	1
0	0	0	0	0	0	0
0	0	0	0	0	0	0
0	0	1	0	0	1	0
0	2	1	0	0	0	0
0	0	0	0	0	2	1
1	0	0	0	0	0	0
0	0	0	0	0	0	0
1	0	0	0	0	0	0
2	0	0	0	0	1	0
0	0	1	0	0	1	0
0	0	0	0	0	3	3
0	0	0	1	1	0	2
0	0	0	0	0	0	0
0	0	0	0	0	1	0
7	22	25	4	22	2	9
5	15	16	3	9	5	7
5	22	13	9	29	2	2
5	4	7	2	6	1	2
3	10	10	3	4	2	3
0	0	0	0	1	0	0
3	18	5	1	5	0	3
1	5	3	2	2	1	2

3	8	7	1	1	1	3
1	9	4	2	0	5	5
3	7	12	5	3	10	6
0	8	7	1	9	3	1
4	12	11	4	4	2	1
1	10	9	2	8	6	4
4	8	8	0	0	8	3
2	8	5	3	14	2	5
3	7	6	1	6	2	2
0	10	3	0	2	2	1
1	9	6	1	6	0	0
0	6	3	3	3	3	4
1	7	1	4	6	2	4
1	2	4	0	0	0	0
4	4	9	4	5	2	7
2	3	3	1	3	0	0
2	4	3	6	6	5	7
3	4	4	8	5	7	9
2	6	6	4	4	2	1
2	10	10	0	6	4	2
4	3	4	1	3	2	1
1	2	1	0	0	1	0
4	3	5	2	2	3	1
3	3	3	3	2	1	2
3	3	2	3	3	0	1
2	5	6	3	3	3	4
0	1	0	0	0	0	0
1	6	4	1	3	1	4
1	6	5	2	2	2	3
1	4	3	3	4	2	3
0	0	0	0	2	0	0
1	4	1	2	2	0	1
2	4	5	2	1	1	1
0	7	13	0	11	14	16
1	3	2	2	2	0	0
0	5	4	1	5	5	3
0	1	1	0	1	1	2
0	4	5	0	0	0	0
1	7	1	1	6	0	0
0	1	4	0	1	0	0
0	8	1	0	2	0	0
0	4	6	0	2	2	1
1	1	3	1	6	1	1
1	7	1	1	1	0	0
0	3	3	0	1	0	0
0	0	0	0	0	0	0
1	3	2	0	4	2	5

1	3	3	3	4	0	0
0	5	3	0	0	2	0
7	8	10	0	4	5	3
0	0	0	0	0	0	0
0	10	2	0	4	6	0
1	2	3	1	3	6	5
3	5	3	1	2	1	1
0	3	1	1	3	0	1
0	0	0	0	0	1	1
0	3	1	1	4	3	1
0	5	3	1	3	6	4
0	3	4	0	2	5	5
2	2	3	1	2	5	5
1	1	1	0	1	0	1
0	3	4	2	3	1	2
0	4	1	1	1	1	0
3	1	3	1	2	0	2
1	3	4	1	3	3	1
2	4	3	0	1	4	1
2	0	1	0	0	0	2
1	4	4	0	3	0	0
0	2	4	0	3	2	4
0	6	0	0	0	0	0
0	2	2	3	4	4	3
0	2	1	0	2	0	1
0	0	3	2	2	3	3
3	5	2	1	1	5	4
0	0	1	0	0	0	0
0	4	2	0	7	5	7
2	2	2	1	2	2	2
1	2	3	1	2	2	2
2	2	3	0	1	1	0
1	5	4	3	3	3	3
1	0	1	0	0	0	0
1	2	2	2	2	1	1
1	1	2	2	3	2	3
1	2	2	0	2	2	2
0	0	2	0	0	2	5
2	2	2	2	3	2	2
2	2	1	2	2	1	1
1	1	1	1	3	1	1
0	2	3	3	1	2	3
0	3	2	0	4	1	2
1	1	0	4	2	4	2
0	0	1	0	0	0	0
0	0	0	1	0	8	3
1	5	3	0	2	1	1

0	4	1	0	1	0	0
0	6	6	3	1	0	3
2	0	0	1	1	4	4
0	2	3	1	1	1	1
1	0	1	0	2	1	1
0	0	0	1	3	0	0
2	0	0	0	1	0	1
0	0	1	0	0	0	1
0	0	0	1	0	0	0
3	2	2	2	1	1	3
2	3	2	2	1	2	1
1	5	2	3	3	3	2
0	0	2	1	0	3	0
0	3	1	0	1	0	1
0	0	0	0	1	0	0
0	1	0	0	0	0	0
0	0	0	0	0	0	0
0	2	3	2	2	1	1
0	1	0	2	2	4	3
0	4	3	0	2	0	0
3	2	1	1	2	3	1
0	3	1	0	2	0	0
0	0	1	0	0	0	1
0	4	1	0	1	1	0
0	3	1	1	2	3	4
0	2	3	1	0	0	0
2	1	2	1	2	1	3
3	1	2	1	0	2	1
0	2	2	0	0	2	2
0	3	3	0	3	0	0
1	0	2	1	1	1	3
0	5	4	0	2	1	2
1	2	1	1	1	1	1
1	0	0	2	2	2	2
0	4	2	1	0	0	0
2	1	1	7	2	2	3
1	1	2	0	0	1	0
0	1	2	0	2	0	0
1	1	2	0	2	2	1
1	2	3	1	2	2	1
1	0	0	3	4	1	2
0	3	0	0	0	0	1
0	5	5	0	0	1	1
0	0	0	3	1	3	2
0	3	2	2	1	1	0
2	1	0	0	2	0	0
0	2	2	0	2	0	0

0	4	0	0	0	0	0
1	3	1	1	0	0	0
0	0	0	0	0	0	0
1	2	1	1	1	1	1
2	1	1	2	0	1	1
4	0	1	0	2	2	1
0	0	3	0	0	0	0
2	0	1	0	1	3	3
0	0	0	1	0	4	0
1	1	0	3	2	2	4
2	0	0	0	1	0	0
0	1	1	2	0	3	4
0	0	0	0	0	0	0
0	1	1	0	0	0	0
1	1	0	2	3	6	4
0	0	0	0	3	0	0
1	0	1	0	0	3	0
0	0	0	1	0	1	1
4	0	0	0	3	1	2
1	1	0	0	0	0	2
0	9	9	11	9	8	9
0	0	0	1	0	0	0
0	12	12	11	12	13	18
1	2	2	1	1	1	1
0	0	2	0	0	0	1
0	2	0	5	0	0	1
1	0	3	0	0	2	2
1	0	0	0	1	0	0
3	1	0	0	1	1	2
0	6	2	0	4	0	0
0	4	0	0	0	6	0
4	10	14	8	10	9	11
1	0	3	2	2	3	2
0	1	0	0	0	0	1
0	0	1	0	0	0	3
0	3	1	1	0	0	2
0	1	2	0	1	0	1
0	0	0	0	0	0	0
1	1	2	0	1	2	1
2	2	2	0	1	2	2
0	1	0	0	0	1	1
2	1	1	2	1	0	2
0	0	1	1	1	3	5
2	1	1	2	2	2	2
0	2	2	0	2	0	0
0	2	1	0	2	2	4
2	0	1	1	0	5	2

0	30	30	0	24	22	0
0	0	0	0	0	0	0
0	1	1	0	1	0	1
0	1	0	1	3	0	2
0	1	1	0	0	1	1
2	0	1	1	1	2	2
1	0	0	1	2	2	2
0	0	1	1	1	4	1
0	2	0	0	1	3	3
0	2	2	0	1	1	1
0	1	1	0	1	2	1
3	3	3	0	3	5	8
0	1	1	0	0	0	0
1	1	2	1	1	0	1
0	1	1	0	0	0	0
0	2	1	1	4	1	2
0	1	1	0	0	0	0
0	0	0	1	1	1	1
0	0	0	0	0	0	1
1	1	1	0	2	2	1
0	2	3	0	1	2	1
1	1	1	3	3	1	2
0	1	2	1	1	1	0
0	0	0	0	1	0	0
0	0	2	0	0	0	0
1	1	0	1	0	1	1
0	4	2	1	1	0	2
1	1	1	1	1	1	2
0	2	3	1	2	2	3
0	0	0	0	1	3	5
0	3	1	0	0	1	4
0	0	0	0	4	0	0
0	0	1	0	0	0	0
0	2	1	0	1	1	1
0	2	1	0	0	0	1
0	1	0	1	1	2	3
3	1	1	0	2	1	0
1	1	1	2	1	0	2
0	1	0	0	0	4	1
1	0	0	0	0	0	0
0	0	0	1	0	1	0
0	1	1	0	2	2	1
1	1	1	0	1	0	1
0	3	1	0	2	1	1
0	1	1	3	0	1	1
0	1	0	1	2	2	2
0	1	2	1	1	0	1

1	1	1	1	3	1	2
0	1	1	0	1	0	0
1	1	2	2	2	1	2
0	0	1	0	0	0	0
2	1	6	0	0	0	1
0	0	0	2	3	2	3
0	1	1	1	1	1	0
2	1	1	1	1	1	1
0	1	1	0	1	1	1
2	1	0	0	2	1	1
0	2	1	1	2	1	2
0	1	1	1	1	1	1
2	0	1	1	1	2	2
0	1	0	0	1	0	0
0	1	0	0	0	0	3
1	0	0	2	2	3	1
1	1	1	0	0	0	1
1	0	0	2	1	2	2
1	1	1	0	1	2	3
0	0	1	1	0	2	0
1	1	0	1	1	1	0
0	1	1	2	1	2	2
0	0	1	2	1	1	1
0	2	1	1	1	0	0
2	0	1	0	0	0	0
0	2	1	1	2	2	1
0	7	1	1	1	2	1
0	3	2	2	3	2	2
0	0	0	0	2	4	2
0	3	0	0	4	4	5
0	2	2	1	0	0	0
1	0	0	0	0	0	0
0	0	0	0	0	0	0
1	1	1	0	0	0	2
0	1	0	1	1	0	0
1	2	0	0	0	1	1
0	1	0	0	0	1	0
0	1	1	2	2	1	1
0	2	1	1	0	2	2
0	1	1	1	1	2	1
0	2	1	2	1	2	1
1	1	0	0	1	3	1
0	0	0	0	0	0	0
0	0	1	0	2	3	2
1	1	2	1	1	1	2
1	0	0	1	3	2	1
1	0	0	0	3	1	2

1	0	0	0	2	3	2
0	0	0	2	1	1	1
1	0	0	0	0	0	0
0	0	0	0	0	0	0
0	0	0	0	0	0	0
2	0	0	1	1	1	1
0	2	0	0	0	0	0
2	0	0	2	0	2	2
0	0	0	0	1	0	0
2	0	1	1	0	0	1
1	1	0	0	1	1	1
0	1	2	0	3	2	2
0	1	0	0	0	0	0
1	0	1	0	1	0	0
0	1	0	0	0	0	0
0	0	0	0	1	2	0
2	0	2	0	0	2	0
1	2	1	1	0	1	0
0	1	1	0	0	0	1
0	0	0	1	1	0	0
1	0	1	1	3	2	2
0	1	1	0	0	0	1
1	2	1	1	1	0	2
0	1	1	2	0	2	1
0	1	1	0	2	2	3
0	3	1	0	0	0	1
0	3	2	0	2	0	0
8	0	0	0	0	0	0
0	0	0	1	1	2	0
0	0	0	0	0	0	0
0	2	0	1	1	0	0
1	2	0	0	2	2	2
0	0	0	0	0	0	0
0	0	0	0	0	0	0
0	0	0	1	3	0	0
0	1	0	1	2	0	0
0	0	0	0	1	2	4
1	2	0	0	1	0	0
1	0	2	0	0	0	0
6	0	0	7	4	0	0
0	1	0	0	1	0	0
0	1	2	0	2	0	0
0	0	0	0	0	5	3
0	0	0	0	0	0	0
0	0	0	0	0	2	0
0	1	1	0	0	0	1
0	1	1	1	1	1	2

3	0	1	1	1	1	1
2	0	1	3	0	2	4
0	2	1	0	1	1	2
1	0	0	1	1	1	2
1	0	1	1	0	2	1
0	0	0	0	0	2	1
0	1	0	0	0	0	0
0	0	0	0	0	0	0
0	0	0	0	0	0	0
0	0	0	0	0	0	0
0	0	0	0	1	1	1
0	1	2	1	1	1	1
0	0	1	0	1	1	0
1	0	0	1	0	0	0
0	1	0	0	1	2	3
0	1	0	0	1	1	0
0	0	1	0	1	1	1
2	0	0	0	0	0	0
5	0	0	0	1	1	1
0	0	0	0	0	0	0
0	0	0	0	0	0	0
0	1	0	0	0	3	0
1	0	0	0	0	0	0
0	0	0	0	0	0	0
0	1	2	1	1	2	1
0	8	0	6	7	6	6
0	1	1	1	2	1	3
0	0	0	1	1	2	1
0	1	1	0	1	1	1
0	2	1	0	2	2	1
0	1	1	0	1	0	0
0	1	2	0	1	1	1
0	1	0	1	0	1	3
0	0	2	1	0	3	1
0	0	0	0	0	0	0
0	0	0	1	0	0	0
1	1	0	0	1	2	2
0	1	2	0	0	0	1
0	0	0	0	0	0	0
0	1	0	0	0	0	1
0	0	0	3	4	4	4
0	0	1	0	0	3	1
0	0	0	0	1	0	0
0	0	0	0	0	0	2
0	0	0	0	0	0	0
0	0	1	0	0	0	0
0	1	1	0	2	2	1

0	0	0	1	1	0	0
1	1	1	1	1	1	0
0	1	1	0	3	0	0
0	2	1	1	1	0	1
0	0	2	1	0	0	0
1	1	1	1	1	0	1
0	0	0	1	1	2	1
1	0	0	0	1	2	0
0	0	0	1	1	1	4
1	0	0	0	1	0	0
0	1	0	0	0	2	2
0	0	0	0	0	0	0
0	2	1	0	0	0	0
0	0	0	0	0	0	0
0	0	0	0	0	0	2
2	0	0	0	0	0	0
0	1	0	0	1	0	0
0	0	2	1	0	1	2
0	0	1	0	1	3	1
1	0	0	2	0	0	0
0	0	0	0	0	0	0
0	0	0	0	0	0	2
1	0	1	0	1	1	2
2	0	0	0	0	1	1
0	0	0	0	0	0	1
0	0	0	0	0	4	2
0	0	0	1	0	0	0
1	0	0	2	0	4	1
0	0	0	0	0	1	0
0	2	0	0	0	0	0
0	0	0	0	0	3	6
0	0	0	0	0	0	0
0	0	0	0	0	0	0
0	0	0	0	0	0	0
0	1	0	1	0	0	0
1	1	0	0	1	1	1
0	1	0	1	1	1	0
0	0	0	0	2	1	1
1	0	0	2	0	1	1
1	0	0	0	2	1	0
0	2	0	0	1	0	0
0	2	0	0	0	1	1
0	2	1	0	1	3	1
1	0	0	0	0	0	0
0	1	0	0	2	1	1
0	0	0	0	1	0	0
0	1	0	0	0	0	0

1	0	0	2	1	2	1
0	0	0	0	0	0	0
2	0	0	0	1	2	0
0	0	1	0	0	2	0
0	0	0	2	1	2	0
0	0	0	0	2	0	0
1	1	0	0	1	0	0
0	0	0	0	1	0	1
0	0	0	0	0	0	0
0	1	0	0	0	0	2
0	0	0	1	0	0	1
0	0	0	0	0	1	0
0	0	1	0	0	1	1
0	0	0	0	0	2	2
0	2	0	0	0	0	0
1	0	0	0	0	1	1
0	1	0	0	0	1	2
0	0	0	1	0	1	2
1	0	0	1	0	0	1
0	0	0	1	0	0	2
1	0	0	1	0	0	0
0	0	0	0	0	0	0
0	0	0	0	0	0	3
0	0	0	0	0	6	0
0	0	0	0	0	2	0
0	0	0	0	0	0	0
0	0	0	0	1	1	1
0	1	0	1	1	1	0
1	0	0	1	2	0	0
0	0	0	0	0	0	0
0	0	0	1	1	0	3
0	1	0	0	0	2	0
0	0	0	0	0	0	0
0	8	8	0	0	0	4
2	1	0	0	2	0	0
0	0	0	0	0	1	3
0	0	0	0	0	0	0
0	0	0	0	0	0	0
0	0	0	5	0	0	0
0	0	0	0	0	0	0
1	0	0	1	2	1	1
0	1	0	0	0	0	0
0	0	0	0	2	1	1
3	0	0	1	0	1	1
0	0	0	2	0	2	2
0	1	0	0	0	1	0
0	0	0	0	0	1	1

0	0	0	0	0	1	0
0	2	0	0	0	0	0
0	0	0	0	0	0	0
0	0	0	0	0	0	0
0	0	0	2	1	0	0
0	0	0	0	0	0	0
0	0	0	0	0	1	0
0	0	1	2	1	1	1
1	0	0	1	1	1	0
0	0	0	0	1	0	0
0	0	0	0	0	0	0
0	0	0	0	2	0	0
0	0	0	0	0	0	0
0	0	0	2	1	0	0
0	1	0	0	0	1	1
0	0	0	0	2	0	0
0	1	0	1	0	0	0
0	0	0	0	1	0	2
0	0	0	0	0	1	2
0	0	0	1	0	2	1
1	0	0	0	0	1	4
0	0	0	0	0	2	0
0	0	0	0	0	0	2
0	0	0	0	0	0	0
0	0	0	0	0	0	0
0	0	0	0	0	0	0
0	0	0	0	0	0	0
0	1	0	0	2	0	0
0	1	0	0	0	0	2
1	0	0	0	0	1	1
0	0	0	0	0	0	0
0	0	2	0	0	1	1
0	0	1	0	0	0	0
0	0	0	0	0	0	0
0	0	0	1	0	1	2
0	0	0	0	0	0	0
0	0	0	0	0	0	0
0	0	0	0	0	0	0
1	0	0	0	0	0	0
0	0	0	0	0	2	0
0	0	0	0	0	0	0
0	2	1	1	0	0	0
0	0	0	0	0	0	0
0	0	0	0	0	0	0
0	0	0	0	0	0	0
0	0	0	0	0	0	1
0	0	0	1	0	1	0

0	0	0	1	0	2	0
0	0	0	0	0	0	0
0	0	0	0	0	0	0
0	0	0	0	0	0	1
0	0	0	0	0	0	0
0	0	0	0	0	0	0
0	0	0	0	0	0	0
0	0	0	0	0	0	0
0	0	0	0	1	0	0
0	0	0	0	0	0	0
0	0	0	0	0	0	0
0	0	0	0	0	0	2
0	0	0	0	0	0	0
0	0	0	0	0	0	0
0	0	0	0	0	0	0
0	0	0	0	0	1	1
0	0	0	1	1	0	0
0	0	0	0	0	7	0
0	0	1	0	0	0	0
0	0	0	0	0	0	0
0	0	0	0	0	0	0
0	0	0	0	0	0	0
0	0	0	0	0	0	0
0	0	0	0	0	0	2
0	0	0	0	0	0	0
0	0	0	0	0	0	9
0	2	0	0	0	0	0
0	0	0	0	0	2	0
0	0	0	0	0	0	0
0	0	0	0	0	0	0
0	0	0	0	0	0	0
0	0	0	0	0	0	0
0	0	0	0	0	0	0
0	0	0	0	0	0	0
0	0	0	0	0	0	0
0	0	0	0	0	0	0
0	0	0	0	0	0	0
0	0	0	0	0	0	0
0	0	0	0	0	0	0
0	0	0	0	0	0	0
0	0	0	0	0	0	0
0	0	0	0	0	0	0
0	0	0	0	0	0	0
0	0	0	0	0	0	0
0	0	0	0	0	0	0
0	0	0	0	0	0	0
0	0	0	0	0	2	0

0	0	0	0	0	0	0
0	0	0	0	0	0	0
0	0	0	0	0	0	0
0	0	0	0	0	0	0
0	0	0	0	0	0	0
0	0	0	0	0	0	0
0	0	0	0	0	0	0
0	0	0	0	0	0	0
0	0	0	0	0	0	0
0	0	0	0	0	0	0

#Glomeruli_Satoskar_141220, Samples Report created on 05/15/2020

Glomeruli_Satoskar_141220

#	Identified Proteins	Gene Name	Accession Number
1	Myosin-9	MYH9	sp|P35579
2	Vimentin	VIM	sp|P08670
8	Isoform 4 of Plectin	PLEC	sp|Q15149-4
10	Isoform 2 of Filamin-A	FLNA	sp|P21333-2
3	Alpha-actinin-4	ACTN4	sp|O43707
4	Actin, cytoplasmic 1	ACTB	sp|P60709
11	Nestin	NES	sp|P48681
6	Neuroblast differentiation-associated protein AH	AHNAK	sp|Q09666
15	Prelamin-A/C	LMNA	sp|P02545
5	Talin-1	TLN1	sp|Q9Y490
9	Collagen alpha-3(VI) chain	COL6A3	tr|E9PCV6
80	Actin, alpha cardiac muscle 1	ACTC1	sp|P68032
17	Keratin, type I cytoskeletal 10	KRT10	sp|P13645
18	Isoform 3 of Spectrin alpha chain, non-erythrocyt	SPTAN1	sp|Q13813-3
7	Laminin subunit alpha-5	LAMA5	sp|O15230
19	Isoform 1 of Vinculin	VCL	sp|P18206-2
12	Keratin, type II cytoskeletal 1	KRT1	sp|P04264
13	Laminin subunit beta-2	LAMB2	sp|P55268
21	Spectrin beta chain, non-erythrocytic 1	SPTBN1	sp|Q01082
23	Isoform 17 of Fibronectin	FN1	sp|P02751-17
14	Basement membrane-specific heparan sulfate pro	HSPG2	sp|P98160
22	Keratin, type II cytoskeletal 2 epidermal	KRT2	sp|P35908
20	Laminin subunit gamma-1	LAMC1	sp|P11047
38	Tropomyosin alpha-4 chain	TPM4	sp|P67936
39	Isoform 4 of Caldesmon	CALD1	sp|Q05682-4
16	Keratin, type I cytoskeletal 9	KRT9	sp|P35527
24	Moesin	MSN	sp|P26038
29	Complement C3	C3	sp|P01024
31	Isoform 2 of Annexin A2	ANXA2	sp|P07355-2
97	Alpha-actinin-1	ACTN1	sp|P12814
255	Isoform 2 of Tropomyosin beta chain	TPM2	sp|P07951-2
30	Isoform 2 of Synaptopodin	SYNPO	sp|Q8N3V7-2
76	Isoform 2 of Myosin-10	MYH10	sp|P35580-2
117	Isoform 2 of Tropomyosin alpha-3 chain	TPM3	sp|P06753-2
27	Tubulin beta-4B chain	TUBB4B	sp|P68371
26	Isoform 2 of Nidogen-1	NID1	sp|P14543-2
33	Tubulin alpha-1B chain	TUBA1B	sp|P68363
114	Tubulin beta chain	TUBB	sp|P07437
294	Tropomyosin 1 (Alpha), isoform CRA_f	TPM1	tr|Q6ZN40
436	Beta-actin-like protein 2	ACTBL2	sp|Q562R1
40	Tight junction protein ZO-1	TJP1	sp|Q07157
42	Isoform 5 of PDZ and LIM domain protein 2	PDLIM2	sp|Q96JY6-5
47	78 kDa glucose-regulated protein	HSPA5	sp|P11021

43	Histone H4	HIST1H4A	sp|P62805
58	Heat shock cognate 71 kDa protein	HSPA8	sp|P11142
449	Tropomyosin 1 (Alpha), isoform CRA_m	TPM1	tr|H7BYY1
25	Hemoglobin subunit beta	HBB	sp|P68871
32	Isoform 2 of Gelsolin	GSN	sp|P06396-2
49	Calmodulin	CALM1	sp|P62158
61	Keratin, type II cytoskeletal 5	KRT5	sp|P13647
111	Ezrin	EZR	sp|P15311
34	Ras GTPase-activating-like protein IQGAP2	IQGAP2	sp|Q13576
35	Isoform 6 of Agrin	AGRN	sp|O00468-6
46	ATP synthase subunit alpha, mitochondrial	ATP5A1	sp|P25705
78	Protein disulfide-isomerase A3	PDIA3	sp|P30101
206	Tubulin beta-2A chain	TUBB2A	sp|Q13885
37	Keratin, type I cytoskeletal 14	KRT14	sp|P02533
44	ATP synthase subunit beta, mitochondrial	ATP5B	sp|P06576
55	Collagen alpha-1(VI) chain	COL6A1	sp|P12109
56	EH domain-containing protein 3	EHD3	sp|Q9NZN3
63	Heat shock 70 kDa protein 1A/1B	HSPA1A	sp|P08107
67	Isoform 2 of Nebulette	NEBL	sp|O76041-2
71	Heat shock protein beta-1	HSPB1	sp|P04792
77	Histone H3.1	HIST1H3A	sp|P68431
95	Isoform 8 of Filamin-B	FLNB	sp|O75369-8
51	Histone H2A type 1	HIST1H2AG	sp|P0C0S8
70	Collagen alpha-2(VI) chain	COL6A2	sp|P12110
93	Lamin-B2	LMNB2	sp|Q03252
100	Myosin regulatory light chain 12B	MYL12B	sp|O14950
60	Ubiquitin-40S ribosomal protein S27a	RPS27A	sp|P62979
68	Heterogeneous nuclear ribonucleoproteins A2/B1	HNRNPA2B1	sp|P22626
69	Unconventional myosin-Ic	MYO1C	sp|O00159
87	Histone H1.4	HIST1H1E	sp|P10412
89	Transgelin-2	TAGLN2	sp|P37802
98	Isoform 3 of Zinc finger protein 185	ZNF185	sp|O15231-3
110	LIM and SH3 domain protein 1	LASP1	sp|Q14847
589	POTE ankyrin domain family member I	POTEI	sp|P0CG38
48	Dihydropyrimidinase-related protein 2	DPYSL2	sp|Q16555
50	Keratin, type II cytoskeletal 6A	KRT6A	sp|P02538
52	Annexin A1	ANXA1	sp|P04083
62	Isoform 2 of Heat shock protein HSP 90-alpha	HSP90AA1	sp|P07900-2
72	Transitional endoplasmic reticulum ATPase	VCP	sp|P55072
91	Isoform 2 of Heterogeneous nuclear ribonucleop	HNRNPK	sp|P61978-2
101	14-3-3 protein zeta/delta	YWHAZ	sp|P63104
113	Podocin	NPHS2	sp|Q9NP85
120	Heat shock protein HSP 90-beta	HSP90AB1	sp|P08238
171	LIM domain only protein 7	LMO7	tr|E9PMS6
235	Myosin regulatory light polypeptide 9	MYL9	sp|P24844
45	Isoform 2 of Clathrin heavy chain 1	CLTC	sp|Q00610-2
75	PDZ and LIM domain protein 5	PDLIM5	sp|Q96HC4

85	Basal cell adhesion molecule	BCAM	sp|P50895
86	Myosin light polypeptide 6	MYL6	tr|G8JLA2
92	Isoform 2 of Collagen alpha-1(XVIII) chain	COL18A1	sp|P39060-1
99	Isoform Gamma-A of Fibrinogen gamma chain	FGG	sp|P02679-2
102	Sodium/potassium-transporting ATPase subunit a	ATP1A1	sp|P05023
118	Isoform 2 of Fructose-bisphosphate aldolase A	ALDOA	sp|P04075-2
127	Profilin-1	PFN1	sp|P07737
141	Palladin	PALLD	sp|Q8WX93
208	Radixin	RDX	sp|P35241
439	Tubulin beta-6 chain	TUBB6	sp|Q9BUF5
28	Hemoglobin subunit alpha	HBA1	sp|P69905
36	Glyceraldehyde-3-phosphate dehydrogenase	GAPDH	sp|P04406
57	Alpha-enolase	ENO1	sp|P06733
79	Collagen alpha-2(IV) chain	COL4A2	sp|P08572
81	Peptidyl-prolyl cis-trans isomerase A	PPIA	sp|P62937
83	Cytoplasmic dynein 1 heavy chain 1	DYNC1H1	sp|Q14204
88	Na(+)/H(+) exchange regulatory cofactor NHE-RF:	SLC9A3R2	sp|Q15599
96	Isoform 2 of Septin-2	SEPT2	sp|Q15019-2
105	Peroxiredoxin-1	PRDX1	sp|Q06830
107	Endoplasmin	HSP90B1	sp|P14625
108	Keratin, type I cytoskeletal 16	KRT16	sp|P08779
124	Protein-glutamine gamma-glutamyltransferase 2	TGM2	sp|P21980
125	Vitronectin	VTN	sp|P04004
228	Cytoskeleton-associated protein 4	CKAP4	sp|Q07065
260	Hemoglobin subunit delta	HBD	sp|P02042
528	Histone H2B type 2-E	HIST2H2BE	sp|Q16778
53	Histone H2B type 1-C/E/F/G/I	HIST1H2BC	sp|P62807
84	Annexin A5	ANXA5	sp|P08758
94	Isoform 2 of Heterogeneous nuclear ribonucleopı	HNRNPM	sp|P52272-2
103	Fibrinogen beta chain	FGB	sp|P02675
104	Ig kappa chain C region	IGKC	sp|P01834
115	Tensin-1	TNS1	tr|E9PGF5
133	Na(+)/H(+) exchange regulatory cofactor NHE-RF:	SLC9A3R1	sp|O14745
149	Zyxin	ZYX	sp|Q15942
153	Isoform 4 of Tensin-like C1 domain-containing ph	TENC1	sp|Q63HR2-4
184	14-3-3 protein gamma	YWHAG	sp|P61981
192	Isoform 5 of Septin-9	SEPT9	sp|Q9UHD8-5
196	Erythrocyte band 7 integral membrane protein	STOM	sp|P27105
845	HLA class I histocompatibility antigen, B-49 alpha	HLA-B	sp|P30487
64	14-3-3 protein epsilon	YWHAE	sp|P62258
65	Pyruvate kinase PKM	PKM	sp|P14618
73	Isoform 2 of Fibrinogen alpha chain	FGA	sp|P02671-2
74	Ubiquitin-like modifier-activating enzyme 1	UBA1	sp|P22314
106	Ig gamma-1 chain C region	IGHG1	sp|P01857
112	Elongation factor 1-alpha 1	EEF1A1	sp|P68104
116	Src substrate cortactin	CTTN	sp|Q14247
119	Integrin-linked protein kinase	ILK	sp|Q13418

145	Transgelin	TAGLN	sp|Q01995
146	Adenylyl cyclase-associated protein 1	CAP1	sp|Q01518
147	Isoform LCRMP-4 of Dihydropyrimidinase-related	DPYSL3	sp|Q14195-2
151	Isoform 2 of Heterogeneous nuclear ribonucleopr	HNRNPA1	sp|P09651-3
154	Heterogeneous nuclear ribonucleoprotein H	HNRNPH1	sp|P31943
155	HLA class I histocompatibility antigen, B-67 alpha	HLA-B	sp|Q29836
167	Calreticulin	CALR	sp|P27797
175	Isoform 2 of HLA class I histocompatibility antiger	HLA-A	sp|P13746-2
178	Complement component C9	C9	sp|P02748
180	Calponin-3	CNN3	sp|Q15417
182	Apolipoprotein E	APOE	sp|P02649
201	Annexin A4	ANXA4	sp|P09525
209	PDZ and LIM domain protein 1	PDLIM1	sp|O00151
211	Capping protein (Actin filament) muscle Z-line, be	CAPZB	tr|B1AK88
212	Isoform 3 of Heterogeneous nuclear ribonucleopr	HNRNPH3	sp|P31942-3
251	Ras-related protein Rab-14	RAB14	sp|P61106
351	EMILIN-1	EMILIN1	sp|Q9Y6C2
608	HLA class I histocompatibility antigen, B-7 alpha c	HLA-B	sp|P01889
807	HLA class I histocompatibility antigen, A-68 alpha	HLA-A	sp|P01891
41	Serum albumin	ALB	sp|P02768
59	Phosphoglycerate kinase 1	PGK1	sp|P00558
82	Annexin A6	ANXA6	sp|P08133
90	Isoform Short of 14-3-3 protein beta/alpha	YWHAB	sp|P31946-2
109	Triosephosphate isomerase	TPI1	sp|P60174
126	Integrin beta-1	ITGB1	sp|P05556
132	Clusterin	CLU	sp|P10909
135	Microtubule-associated protein	MAP4	tr|E7EVA0
136	Glutamyl aminopeptidase	ENPEP	sp|Q07075
138	Heterogeneous nuclear ribonucleoprotein U	HNRNPU	sp|Q00839
142	Nucleolin	NCL	sp|P19338
144	Isoform 2 of Transketolase	TKT	sp|P29401-2
150	Ig alpha-1 chain C region	IGHA1	sp|P01876
156	Serum amyloid P-component	APCS	sp|P02743
169	Protein disulfide-isomerase	P4HB	sp|P07237
172	Splicing factor, proline- and glutamine-rich	SFPQ	sp|P23246
179	Isoform C1 of Heterogeneous nuclear ribonucleo	HNRNPC	sp|P07910-2
189	Protein S100-A6	S100A6	sp|P06703
204	Heterogeneous nuclear ribonucleoprotein A3	HNRNPA3	sp|P51991
207	Isoform 2 of Membrane-associated guanylate kin	MAGI2	sp|Q86UL8-2
217	Trifunctional enzyme subunit alpha, mitochondri	HADHA	sp|P40939
218	Stress-70 protein, mitochondrial	HSPA9	sp|P38646
220	Isoform 2 of NADH-cytochrome b5 reductase 3	CYB5R3	sp|P00387-2
223	Inverted formin-2	INF2	sp|Q27J81
229	Isoform 2 of Drebrin-like protein	DBNL	sp|Q9UJU6-2
243	Thymosin beta-4	TMSB4X	sp|P62328
252	Ribosome-binding protein 1	RRBP1	sp|Q9P2E9
254	Guanine nucleotide-binding protein G(I)/G(S)/G(T	GNB2	sp|P62879

257	EH domain-containing protein 4	EHD4	sp|Q9H223
275	Trifunctional enzyme subunit beta, mitochondria	HADHB	sp|P55084
278	Band 4.1-like protein 2	EPB41L2	sp|O43491
284	Cysteine and glycine-rich protein 2	CSRP2	sp|Q16527
312	Nuclease-sensitive element-binding protein 1	YBX1	sp|P67809
317	Lysozyme C	LYZ	sp|P61626
337	Isoform 2 of Transcription intermediary factor 1-l	TRIM28	sp|Q13263-2
362	Peroxisomal multifunctional enzyme type 2	HSD17B4	sp|P51659
411	Lamin-B1	LMNB1	sp|P20700
458	Probable ATP-dependent RNA helicase DDX17	DDX17	tr|H3BLZ8
479	Isoform Non-brain of Clathrin light chain A	CLTA	sp|P09496-2
499	Isoform 2 of A-kinase anchor protein 12	AKAP12	sp|Q02952-2
778	Plastin-2	LCP1	sp|P13796
970	Keratin, type I cytoskeletal 18	KRT18	sp|P05783
1056	Keratin, type II cytoskeletal 75	KRT75	sp|O95678
121	Ras GTPase-activating-like protein IQGAP1	IQGAP1	sp|P46940
123	Glutathione S-transferase P	GSTP1	sp|P09211
139	Plastin-3	PLS3	sp|P13797
143	Ras-related protein R-Ras	RRAS	sp|P10301
152	Lipoma-preferred partner	LPP	sp|Q93052
158	Protein DJ-1	PARK7	sp|Q99497
160	Isoform 2 of Podocalyxin	PODXL	sp|O00592-2
164	Integrin alpha-3	ITGA3	sp|P26006
165	Cysteine and glycine-rich protein 1	CSRP1	sp|P21291
168	Alpha-parvin	PARVA	sp|Q9NVD7
170	60 kDa heat shock protein, mitochondrial	HSPD1	sp|P10809
181	Cysteine-rich protein 2	CRIP2	sp|P52943
191	WD repeat-containing protein 1	WDR1	sp|O75083
200	40S ribosomal protein S3	RPS3	sp|P23396
202	Isoform 2 of Ras-related protein Rab-5C	RAB5C	sp|P51148-2
203	Protein AMBP	AMBP	sp|P02760
205	Poly(rC)-binding protein 1	PCBP1	sp|Q15365
214	Calnexin	CANX	tr|B4DGP8
216	F-actin-capping protein subunit alpha-1	CAPZA1	sp|P52907
221	Protein ARPC4-TTLL3	ARPC4-TTLL3	tr|F8WCF6
224	Aconitate hydratase, mitochondrial	ACO2	tr|A2A274
225	Glutamate dehydrogenase 1, mitochondrial	GLUD1	sp|P00367
233	Isoform 2 of Cytosol aminopeptidase	LAP3	sp|P28838-2
236	Actin-related protein 2	ACTR2	sp|P61160
237	Carbonyl reductase [NADPH] 1	CBR1	sp|P16152
238	Tensin-3	TNS3	sp|Q68CZ2
246	Fibulin-1	FBLN1	sp|P23142
249	X-ray repair cross-complementing protein 6	XRCC6	sp|P12956
250	Haptoglobin	HP	tr|H0Y300
253	Major vault protein	MVP	sp|Q14764
263	Rho GDP-dissociation inhibitor 1 (Fragment)	ARHGDIA	tr|J3KTF8
269	Protein eva-1 homolog B	EVA1B	sp|Q9NVM1

273	Nephrin	NPHS1	sp|O60500
280	Isoform 3 of Malate dehydrogenase, cytoplasmic	MDH1	sp|P40925-3
281	14-3-3 protein eta	YWHAH	sp|Q04917
282	Ras-related protein Rab-1A	RAB1A	sp|P62820
292	Isoform 2 of Elongation factor 1-delta	EEF1D	sp|P29692-2
295	Vesicle-associated membrane protein 2	VAMP2	sp|P63027
299	Dolichyl-diphosphooligosaccharide--protein glycc	RPN1	sp|P04843
311	Isoform 10 of Dysferlin	DYSF	sp|O75923-10
318	Non-POU domain-containing octamer-binding pr	NONO	sp|Q15233
323	Isoform B of Ras-related C3 botulinum toxin subs	RAC1	sp|P63000-2
325	ATP-dependent RNA helicase DDX3X	DDX3X	sp|O00571
326	Isoform 2 of Sarcoplasmic/endoplasmic reticulum	ATP2A2	sp|P16615-2
340	60S ribosomal protein L13	RPL13	sp|P26373
379	14-3-3 protein theta	YWHAQ	sp|P27348
380	Isoform 1 of Voltage-dependent anion-selective c	VDAC2	sp|P45880-1
383	Isoform 2 of Glucosidase 2 subunit beta	PRKCSH	sp|P14314-2
387	Isoform 1 of Core histone macro-H2A.1	H2AFY	sp|O75367-2
396	Complement C5	C5	sp|P01031
407	Kinesin-1 heavy chain	KIF5B	sp|P33176
422	Dihydrolipoyl dehydrogenase, mitochondrial	DLD	tr|E9PEX6
435	Aspartate aminotransferase, mitochondrial	GOT2	sp|P00505
440	Hepatoma-derived growth factor	HDGF	sp|P51858
444	Isoform 2 of Extended synaptotagmin-1	ESYT1	sp|Q9BSJ8-2
448	Histone H1.5	HIST1H1B	sp|P16401
451	Transforming growth factor-beta-induced proteir	TGFBI	sp|Q15582
565	Histone H2A.Z	H2AFZ	sp|P0C0S5
569	Isoform 2 of Dolichyl-diphosphooligosaccharide--	RPN2	sp|P04844-2
596	Protein phosphatase 1F	PPM1F	sp|P49593
749	SUN domain-containing protein 2	SUN2	sp|Q9UH99
761	Endonuclease domain-containing 1 protein	ENDOD1	sp|O94919
779	Isoform 4 of Uromodulin	UMOD	sp|P07911-4
851	Sideroflexin-3	SFXN3	sp|Q9BWM7
904	Thrombospondin-1	THBS1	sp|P07996
54	Neprilysin	MME	sp|P08473
128	Collagen alpha-4(IV) chain	COL4A4	tr|J3KNM7
130	Septin-7	SEPT7	sp|Q16181
157	Isoform 3 of L-lactate dehydrogenase A chain	LDHA	sp|P00338-3
159	Tenascin	TNC	sp|P24821
161	Complement C4-B	C4B	tr|F5GXS0
173	Rab GDP dissociation inhibitor beta	GDI2	sp|P50395
176	Elongation factor 2	EEF2	sp|P13639
183	Cathepsin D	CTSD	sp|P07339
186	Isoform 2 of Unconventional myosin-Ib	MYO1B	sp|O43795-2
187	Collagen alpha-1(IV) chain	COL4A1	sp|P02462
195	Cofilin-1	CFL1	tr|E9PK25
197	Nucleophosmin	NPM1	sp|P06748
198	Isoform 2 of Collagen alpha-3(IV) chain	COL4A3	sp|Q01955-2

199	ADP-ribosylation factor 1	ARF1	sp|P84077
213	Ras-related protein Rap-1A	RAP1A	sp|P62834
215	Isoform 3 of Tubulointerstitial nephritis antigen-li	TINAGL1	sp|Q9GZM7-3
222	Isoform sGi2 of Guanine nucleotide-binding prote	GNAI2	sp|P04899-4
227	Isoform 2 of Myosin light chain kinase, smooth m	MYLK	sp|Q15746-2
234	Actin-related protein 3	ACTR3	sp|P61158
239	Malate dehydrogenase, mitochondrial	MDH2	sp|P40926
242	Selenium-binding protein 1	SELENBP1	sp|Q13228
247	Galectin-1	LGALS1	sp|P09382
248	Ras-related protein Rab-7a	RAB7A	sp|P51149
258	Heterogeneous nuclear ribonucleoprotein R	HNRNPR	sp|O43390
264	Intercellular adhesion molecule 1	ICAM1	sp|P05362
265	Vacuolar protein sorting-associated protein 35	VPS35	sp|Q96QK1
272	F-actin-capping protein subunit alpha-2	CAPZA2	sp|P47755
274	Isoform 2 of Integrin alpha-V	ITGAV	sp|P06756-2
279	Isoform 3 of Heterogeneous nuclear ribonucleop	HNRNPD	sp|Q14103-3
286	Isoform 2 of Protein disulfide-isomerase A6	PDIA6	sp|Q15084-2
293	Eukaryotic initiation factor 4A-I	EIF4A1	sp|P60842
304	Fibrillin-1	FBN1	sp|P35555
305	Transforming protein RhoA	RHOA	sp|P61586
307	Fructose-bisphosphate aldolase C	ALDOC	sp|P09972
313	Transcriptional activator protein Pur-alpha	PURA	sp|Q00577
322	Flavin reductase (NADPH)	BLVRB	sp|P30043
324	Alpha-centractin	ACTR1A	sp|P61163
332	Rho GDP-dissociation inhibitor 2	ARHGDIB	sp|P52566
345	Isoform 2 of Nuclear mitotic apparatus protein 1	NUMA1	sp|Q14980-2
349	40S ribosomal protein S18	RPS18	sp|P62269
356	3-hydroxyacyl-CoA dehydrogenase type-2	HSD17B10	sp|Q99714
357	Ferritin light chain	FTL	sp|P02792
367	Tubulin polymerization-promoting protein family	TPPP3	sp|Q9BW30
368	Dihydrolipoyllysine-residue succinyltransferase co	DLST	sp|P36957
371	Vacuolar protein sorting-associated protein 26A	VPS26A	sp|O75436
373	Sulfide:quinone oxidoreductase, mitochondrial	SQRDL	sp|Q9Y6N5
374	Chloride intracellular channel protein 1	CLIC1	sp|O00299
376	Matrin-3	MATR3	tr|A8MXP9
378	Vinexin	SORBS3	sp|O60504
392	Isoform IIb of Profilin-2	PFN2	sp|P35080-2
394	Synaptic vesicle membrane protein VAT-1 homolo	VAT1	sp|Q99536
399	Guanine nucleotide-binding protein subunit beta-	GNB2L1	sp|P63244
400	Enoyl-CoA hydratase, mitochondrial	ECHS1	sp|P30084
401	Interleukin enhancer-binding factor 2	ILF2	sp|Q12905
404	Nascent polypeptide-associated complex subunit	NACA	sp|E9PAV3
408	Vasodilator-stimulated phosphoprotein	VASP	sp|P50552
412	Four and a half LIM domains protein 2	FHL2	sp|Q14192
418	Dermcidin	DCD	sp|P81605
419	Peptidyl-prolyl cis-trans isomerase FKBP1A	FKBP1A	sp|P62942
423	40S ribosomal protein S28	RPS28	sp|P62857

428	Cytoplasmic FMR1-interacting protein 1	CYFIP1	sp|Q7L576
430	40S ribosomal protein S14	RPS14	sp|P62263
432	Isoform 2 of Prothymosin alpha	PTMA	sp|P06454-2
433	Kinectin	KTN1	sp|Q86UP2
437	Isoform 2 of Very long-chain specific acyl-CoA del	ACADVL	sp|P49748-2
441	Vesicle-trafficking protein SEC22b	SEC22B	sp|O75396
450	ATP synthase subunit O, mitochondrial	ATP5O	sp|P48047
453	Heterogeneous nuclear ribonucleoprotein L	HNRNPL	sp|P14866
456	Protein NDRG1	NDRG1	sp|Q92597
457	Isoform 3 of Nucleoside diphosphate kinase B	NME2	sp|P22392-2
462	Protein disulfide-isomerase A4	PDIA4	sp|P13667
464	60S ribosomal protein L22	RPL22	sp|P35268
465	40S ribosomal protein S16	RPS16	sp|P62249
471	Lamina-associated polypeptide 2, isoforms beta/	TMPO	sp|P42167
473	Serine/arginine-rich-splicing factor 2 (Fragment)	SRSF2	tr|J3QL05
474	Isoform 2 of Alpha-adducin	ADD1	sp|P35611-2
476	Isoform 3 of KN motif and ankyrin repeat domain	KANK2	sp|Q63ZY3-3
488	Aspartate--tRNA ligase, cytoplasmic	DARS	sp|P14868
490	40S ribosomal protein S9	RPS9	sp|P46781
506	40S ribosomal protein S10	RPS10	sp|P46783
508	Mitogen-activated protein kinase 1	MAPK1	sp|P28482
509	Quinone oxidoreductase	CRYZ	sp|Q08257
512	Isoform 6 of Myoferlin	MYOF	sp|Q9NZM1-6
517	Isoform Short of Eukaryotic translation initiation	EIF4H	sp|Q15056-2
520	Complement factor H	CFH	sp|P08603
527	Protein phosphatase 1 regulatory subunit 12A	PPP1R12A	sp|O14974
529	Isoform 3 of Hexokinase-1	HK1	sp|P19367-3
540	Fatty acid-binding protein, adipocyte	FABP4	sp|P15090
542	von Willebrand factor A domain-containing prote	VWA1	sp|Q6PCB0
543	Delta(3,5)-Delta(2,4)-dienoyl-CoA isomerase, mit	ECH1	sp|Q13011
548	Isoform XLas-2 of Guanine nucleotide-binding pro	GNAS	sp|Q5JWF2-2
555	Lysosome-associated membrane glycoprotein 1	LAMP1	sp|P11279
566	Voltage-dependent anion-selective channel prote	VDAC1	sp|P21796
581	Actin-related protein 2/3 complex subunit 1A	ARPC1A	sp|Q92747
593	Serine/threonine-protein phosphatase PP1-beta	PPP1CB	sp|P62140
613	Tripeptidyl-peptidase 1	TPP1	sp|O14773
616	60S ribosomal protein L12	RPL12	sp|P30050
656	Isoform 1 of Gamma-adducin	ADD3	sp|Q9UEY8-2
663	Poly(rC)-binding protein 2 (Fragment)	PCBP2	tr|H3BRU6
676	Isoform Alpha of LIM domain and actin-binding p	LIMA1	sp|Q9UHB6-2
703	Isoform 2 of Fermitin family homolog 3	FERMT3	sp|Q86UX7-2
720	Isoform 1A of Sorting nexin-1	SNX1	sp|Q13596-2
723	Coronin-1A	CORO1A	sp|P31146
732	tRNA-splicing ligase RtcB homolog	RTCB	sp|Q9Y3I0
738	Isocitrate dehydrogenase [NADP]	IDH2	tr|B4DFL2
745	Fumarate hydratase, mitochondrial	FH	sp|P07954
772	Isoform Short of Endoglin	ENG	sp|P17813-2

811	Nucleobindin-1	NUCB1	sp|Q02818
812	Isoform 2 of Filamin-binding LIM protein 1	FBLIM1	sp|Q8WUP2-2
979	Isocitrate dehydrogenase [NAD] subunit alpha, m	IDH3A	sp|P50213
122	Isoform 3 of Fermitin family homolog 2	FERMT2	sp|Q96AC1-3
131	Peroxiredoxin-6	PRDX6	sp|P30041
134	L-lactate dehydrogenase B chain	LDHB	sp|P07195
137	Peroxiredoxin-2	PRDX2	sp|P32119
140	Phosphatidylethanolamine-binding protein 1	PEBP1	sp|P30086
148	Creatine kinase B-type	CKB	sp|P12277
162	Protein S100-A9	S100A9	sp|P06702
166	Cytosolic non-specific dipeptidase	CNDP2	sp|Q96KP4
190	Alpha-1-antitrypsin	SERPINA1	sp|P01009
193	Septin-11	SEPT11	sp|Q9NVA2
194	Ras suppressor protein 1	RSU1	sp|Q15404
210	Catalase	CAT	sp|P04040
230	Histone H1.0	H1F0	sp|P07305
240	Reticulon-4	RTN4	sp|Q9NQC3
241	Integrin alpha-1	ITGA1	sp|P56199
256	Galectin-3-binding protein	LGALS3BP	sp|Q08380
261	Isoform 2 of Transforming growth factor beta-1-i	TGFB1I1	sp|O43294-2
262	Isoform 2 of Utrophin	UTRN	sp|P46939-2
266	Isoform 2 of Kin of IRRE-like protein 1	KIRREL	sp|Q96J84-2
267	Tropomodulin-3	TMOD3	sp|Q9NYL9
270	Heterochromatin protein 1-binding protein 3	HP1BP3	sp|Q5SSJ5
271	Prohibitin-2	PHB2	sp|Q99623
276	Staphylococcal nuclease domain-containing prote	SND1	sp|Q7KZF4
283	Thioredoxin	TXN	sp|P10599
285	Cell division control protein 42 homolog	CDC42	sp|P60953
297	Rab GDP dissociation inhibitor alpha	GDI1	sp|P31150
298	Tryptophan--tRNA ligase, cytoplasmic	WARS	sp|P23381
303	Aquaporin-1	AQP1	tr|B4E220
309	Isoform 2 of Programmed cell death 6-interacting	PDCD6IP	sp|Q8WUM4-2
320	Beta-2-microglobulin	B2M	sp|P61769
321	Isoform 11 of Sorbin and SH3 domain-containing	SORBS2	sp|O94875-11
327	Retinal dehydrogenase 1	ALDH1A1	sp|P00352
329	Prohibitin	PHB	sp|P35232
331	Multimerin-2	MMRN2	sp|Q9H8L6
335	Peroxiredoxin-5, mitochondrial	PRDX5	sp|P30044
336	Isoform 3 of Nephronectin	NPNT	sp|Q6UXI9-3
339	Isoform 4 of Serine/arginine-rich splicing factor 7	SRSF7	sp|Q16629-4
341	Aldehyde dehydrogenase, mitochondrial	ALDH2	sp|P05091
343	Dendrin	DDN	sp|O94850
344	PDZ and LIM domain protein 7	PDLIM7	sp|Q9NR12
346	Protein-L-isoaspartate O-methyltransferase	PCMT1	tr|H7BY58
347	Chloride intracellular channel protein 4	CLIC4	sp|Q9Y696
350	Transaldolase	TALDO1	sp|P37837
352	Ferritin heavy chain	FTH1	sp|P02794

353	Alpha-1-antichymotrypsin	SERPINA3	sp|P01011
358	Fascin	FSCN1	sp|Q16658
369	Ras-related protein Rab-11A	RAB11A	sp|P62491
370	V-type proton ATPase catalytic subunit A	ATP6V1A	tr|B7Z1R5
372	Actin-related protein 2/3 complex subunit 3	ARPC3	sp|O15145
375	Isoform 1AC of Catenin delta-1	CTNND1	sp|O60716-3
381	AP-2 complex subunit beta	AP2B1	tr|K7EJT8
382	Acetyl-CoA acetyltransferase, mitochondrial	ACAT1	sp|P24752
386	Isoform 2 of Spliceosome RNA helicase DDX39B	DDX39B	sp|Q13838-2
388	40S ribosomal protein S4, X isoform	RPS4X	sp|P62701
390	Isoform 2 of Nidogen-2	NID2	sp|Q14112-2
393	T-complex protein 1 subunit zeta	CCT6A	sp|P40227
395	Elongation factor Tu, mitochondrial	TUFM	sp|P49411
397	Cytochrome c oxidase subunit 2	MT-CO2	sp|P00403
398	Guanine nucleotide-binding protein G(I)/G(S)/G((	GNG12	sp|Q9UBI6
402	Unconventional myosin-Ie	MYO1E	sp|Q12965
405	Ribonuclease inhibitor	RNH1	sp|P13489
406	HLA class II histocompatibility antigen, DR alpha c	HLA-DRA	sp|P01903
409	Proliferation-associated protein 2G4	PA2G4	sp|Q9UQ80
410	Serine/threonine-protein phosphatase 2A 65 kDa	PPP2R1A	sp|P30153
416	PRKC apoptosis WT1 regulator protein	PAWR	sp|Q96IZ0
417	Macrophage migration inhibitory factor	MIF	sp|P14174
420	40S ribosomal protein S7	RPS7	sp|P62081
424	Serine/arginine-rich splicing factor 1	SRSF1	sp|Q07955
425	Peptidyl-prolyl cis-trans isomerase B	PPIB	sp|P23284
431	4-trimethylaminobutyraldehyde dehydrogenase	ALDH9A1	sp|P49189
434	Alpha/beta hydrolase domain-containing protein	ABHD14B	sp|Q96IU4
443	Complement receptor type 1	CR1	sp|P17927
445	Isoform B of AP-2 complex subunit alpha-1	AP2A1	sp|O95782-2
446	Elongation factor 1-gamma	EEF1G	sp|P26641
455	Serpin H1	SERPINH1	sp|P50454
460	Leukocyte elastase inhibitor	SERPINB1	sp|P30740
469	Far upstream element-binding protein 1	FUBP1	tr|B4DT31
477	Phosphoglucomutase-2	PGM2	sp|Q96G03
483	Isoform 3 of Synaptopodin-2	SYNPO2	sp|Q9UMS6-3
486	Isoform 2 of Nuclear receptor-interacting protein	NRIP2	sp|Q9BQI9-2
487	Citrate synthase, mitochondrial	CS	sp|O75390
489	Unconventional myosin-Id	MYO1D	sp|O94832
491	Isoform 2 of KN motif and ankyrin repeat domain	KANK3	sp|Q6NY19-2
492	Isoform B of Methyl-CpG-binding protein 2	MECP2	sp|P51608-2
494	182 kDa tankyrase-1-binding protein	TNKS1BP1	sp|Q9C0C2
496	Cystatin-B	CSTB	sp|P04080
500	Isoform 2 of Receptor-type tyrosine-protein phos	PTPRO	sp|Q16827-2
503	SH3 domain-binding glutamic acid-rich-like protei	SH3BGRL3	sp|Q9H299
504	Isoform Short of RNA-binding protein FUS	FUS	sp|P35637-2
505	Isoform 2 of Collagen alpha-5(IV) chain	COL4A5	sp|P29400-2
507	MAGUK p55 subfamily member 5	MPP5	sp|Q8N3R9

514	Isoform 2B of Cytoplasmic dynein 1 intermediate	DYNC1I2	sp|Q13409-2
524	Isoform 2 of Syntaxin-7	STX7	sp|O15400-2
525	Isoform 4 of Cadherin-13	CDH13	sp|P55290-4
532	Catenin (Cadherin-associated protein), alpha 1, 1(	CTNNA1	tr|G3XAM7
534	Cytochrome b-c1 complex subunit 2, mitochondr	UQCRC2	sp|P22695
538	Isoform B of Phosphate carrier protein, mitochon	SLC25A3	sp|Q00325-2
539	40S ribosomal protein S26	RPS26	sp|P62854
541	Isoform 2 of Phosphatidylinositol-binding clathrir	PICALM	sp|Q13492-2
549	Isoform 3 of Drebrin	DBN1	sp|Q16643-3
551	Histone H1x	H1FX	sp|Q92522
559	Choline transporter-like protein 2	SLC44A2	sp|Q8IWA5
564	Ephrin-B1	EFNB1	sp|P98172
567	PRA1 family protein 3	ARL6IP5	sp|O75915
568	Chromobox protein homolog 3	CBX3	sp|Q13185
577	ATP synthase subunit delta, mitochondrial	ATP5D	sp|P30049
579	Activated RNA polymerase II transcriptional coact	SUB1	sp|P53999
580	Isoform 3 of RNA-binding protein EWS	EWSR1	sp|Q01844-3
584	Guanine nucleotide-binding protein subunit alpha	GNA11	sp|P29992
586	Protein S100-A10	S100A10	sp|P60903
587	T-complex protein 1 subunit delta	CCT4	sp|P50991
588	Thymidine phosphorylase	TYMP	sp|P19971
590	Isoform 3 of Protein AHNAK2	AHNAK2	sp|Q8IVF2-3
592	Ubiquitin-conjugating enzyme E2 N	UBE2N	sp|P61088
594	Isoform 2 of Membrane-associated progesterone	PGRMC2	sp|O15173-2
597	Hsp90 co-chaperone Cdc37	CDC37	sp|Q16543
600	60S ribosomal protein L4	RPL4	sp|P36578
601	Serpin B9	SERPINB9	sp|P50453
602	Proteasome subunit beta type-4	PSMB4	sp|P28070
609	Isoform 2 of Thioredoxin-dependent peroxide rec	PRDX3	sp|P30048-2
611	Isoform 2 of Plasminogen activator inhibitor 1 RN	SERBP1	sp|Q8NC51-2
612	Cytoplasmic dynein 1 light intermediate chain 2	DYNC1LI2	sp|O43237
618	Heat shock 70 kDa protein 4	HSPA4	sp|P34932
622	Protein transport protein Sec23A	SEC23A	sp|Q15436
628	Emerin	EMD	sp|P50402
630	60S ribosomal protein L7	RPL7	sp|P18124
632	60S ribosomal protein L6	RPL6	sp|Q02878
636	Isoform 2 of AP-2 complex subunit mu	AP2M1	sp|Q96CW1-2
641	Transmembrane protein 109	TMEM109	sp|Q9BVC6
643	Isoform 3 of Tumor protein D54	TPD52L2	sp|O43399-3
644	Isoform 2 of Cytochrome b5	CYB5A	sp|P00167-2
645	Isoform 3 of Protein crumbs homolog 2	CRB2	sp|Q5IJ48-3
646	cAMP-dependent protein kinase type I-alpha regu	PRKAR1A	sp|P10644
648	40S ribosomal protein S3a	RPS3A	sp|P61247
653	Actin-related protein 2/3 complex subunit 1B	ARPC1B	sp|O15143
654	Tubulin-folding cofactor B	TBCB	sp|Q99426
655	Electron transfer flavoprotein subunit alpha, mitc	ETFA	tr|H0YK49
658	Ras-related protein Ral-A	RALA	sp|P11233

671	Isoform 2 of Chromatin target of PRMT1 protein	CHTOP	sp|Q9Y3Y2-3
673	60S ribosomal protein L23a (Fragment)	RPL23A	tr|H7BY10
674	60S ribosomal protein L18	RPL18	sp|Q07020
675	Isoform 4 of T-complex protein 1 subunit eta	CCT7	sp|Q99832-4
677	Isoform 2 of Carnitine O-palmitoyltransferase 1, l	CPT1A	sp|P50416-2
680	Isoform Delta15 of Platelet endothelial cell adhes	PECAM1	sp|P16284-6
681	Inter-alpha-trypsin inhibitor heavy chain H5	ITIH5	sp|Q86UX2
682	Isoform 3 of Perilipin-3	PLIN3	sp|O60664-3
685	Far upstream element-binding protein 2	KHSRP	sp|Q92945
687	Isoform H14 of Myeloperoxidase	MPO	sp|P05164-2
688	LEM domain-containing protein 2	LEMD2	sp|Q8NC56
691	6-phosphofructokinase, liver type	PFKL	sp|P17858
695	Mitochondrial fission 1 protein	FIS1	sp|Q9Y3D6
696	Vesicle-associated membrane protein 5	VAMP5	sp|O95183
697	40S ribosomal protein S21	RPS21	sp|P63220
698	ATP synthase subunit gamma	ATP5C1	tr|B4DL14
699	Protein LYRIC	MTDH	sp|Q86UE4
701	Transmembrane emp24 domain-containing prote	TMED10	sp|P49755
702	Isoform 2 of Interleukin enhancer-binding factor :	ILF3	sp|Q12906-2
705	Translocon-associated protein subunit delta	SSR4	sp|P51571
707	GTP:AMP phosphotransferase AK3, mitochondria	AK3	sp|Q9UIJ7
709	Isoform 2 of Alpha-aminoadipic semialdehyde de	ALDH7A1	sp|P49419-2
713	Laminin subunit alpha-2	LAMA2	sp|P24043
714	26S proteasome non-ATPase regulatory subunit 2	PSMD2	sp|Q13200
715	Complement component C8 gamma chain	C8G	sp|P07360
716	Delta-1-pyrroline-5-carboxylate dehydrogenase, ı	ALDH4A1	sp|P30038
717	Rho guanine nucleotide exchange factor 17	ARHGEF17	sp|Q96PE2
721	40S ribosomal protein S8	RPS8	sp|P62241
725	60S ribosomal protein L7a	RPL7A	sp|P62424
726	Isoform 3 of Heterogeneous nuclear ribonucleopı	HNRNPAB	sp|Q99729-3
727	Apoptosis-inducing factor 1, mitochondrial	AIFM1	sp|O95831
740	Single-stranded DNA-binding protein, mitochondı	SSBP1	sp|Q04837
741	BTB/POZ domain-containing protein KCTD12	KCTD12	sp|Q96CX2
742	Bifunctional glutamate/proline--tRNA ligase	EPRS	sp|P07814
754	60S ribosomal protein L8	RPL8	sp|P62917
755	2,4-dienoyl-CoA reductase, mitochondrial	DECR1	tr|B7Z6B8
762	Transthyretin	TTR	sp|P02766
764	Gamma-interferon-inducible protein 16	IFI16	sp|Q16666
765	Uncharacterized protein (Fragment)	4	tr|H0YHG0
768	HLA class II histocompatibility antigen, DRB1-7 be	HLA-DRB1	sp|P13761
769	Putative RNA-binding protein 3	RBM3	sp|P98179
781	Platelet-activating factor acetylhydrolase IB subu	PAFAH1B1	sp|P43034
782	Platelet-derived growth factor receptor beta	PDGFRB	sp|P09619
785	Lipid phosphate phosphohydrolase 3	PPAP2B	sp|O14495
787	Coiled-coil-helix-coiled-coil-helix domain-containi	CHCHD3	sp|Q9NX63
789	Cytochrome b-c1 complex subunit 1, mitochondr	UQCRC1	sp|P31930
792	Mitochondrial inner membrane protein	IMMT	tr|B9A067

795	Eukaryotic translation initiation factor 3 subunit A	EIF3A	sp|Q14152
797	Isoform 4 of A-kinase anchor protein 2	AKAP2	sp|Q9Y2D5-6
820	Four and a half LIM domains protein 3	FHL3	sp|Q13643
823	Ig kappa chain V-II region Cum	1	sp|P01614
825	Isoform 2 of Ubiquitin carboxyl-terminal hydrolas	USP14	sp|P54578-2
826	Protein S100-A4	S100A4	sp|P26447
838	Cytochrome c	CYCS	sp|P99999
842	Neurogranin	NRGN	sp|Q92686
855	Putative pre-mRNA-splicing factor ATP-dependen	DHX15	sp|O43143
866	Sorting nexin-2	SNX2	sp|O60749
896	Isoform 2 of Electron transfer flavoprotein subun	ETFB	sp|P38117-2
897	Gap junction alpha-5 protein	GJA5	sp|P36382
900	Protein PML	PML	sp|P29590
907	Thyroid receptor-interacting protein 11	TRIP11	sp|Q15643
910	Isoform 3 of Probable palmitoyltransferase ZDHH	ZDHHC8	sp|Q9ULC8-3
911	Cathepsin Z	CTSZ	sp|Q9UBR2
916	Aldose reductase	AKR1B1	sp|P15121
934	60S ribosomal protein L31	RPL31	sp|P62899
938	Cathepsin G	CTSG	sp|P08311
950	Golgi integral membrane protein 4	GOLIM4	sp|O00461
954	Cell surface glycoprotein MUC18	MCAM	sp|P43121
991	Myeloid cell nuclear differentiation antigen	MNDA	sp|P41218
1038	Isoform 3 of Yorkie homolog	YAP1	sp|P46937-3
66	Desmoplakin	DSP	sp|P15924
129	Fructose-bisphosphate aldolase B	ALDOB	sp|P05062
163	Junction plakoglobin	JUP	tr|F5GWP8
174	Calpain small subunit 1	CAPNS1	sp|P04632
177	Aminoacylase-1	ACY1	sp|Q03154
185	Isoform 12 of Titin	TTN	sp|Q8WZ42-12
188	Phosphoglycerate mutase 1	PGAM1	sp|P18669
219	Serpin B4	SERPINB4	sp|P48594
226	Argininosuccinate synthase	ASS1	sp|P00966
231	Alpha-2-macroglobulin	A2M	sp|P01023
232	Desmoglein-1	DSG1	sp|Q02413
244	Isoform 2 of Ig mu chain C region	IGHM	sp|P01871-2
245	Keratin, type II cytoskeletal 3	KRT3	sp|P12035
259	T-complex protein 1 subunit beta	CCT2	sp|P78371
268	Neutral alpha-glucosidase AB	GANAB	sp|Q14697
277	Isoform 2 of Glucose-6-phosphate isomerase	GPI	sp|P06744-2
287	Alcohol dehydrogenase [NADP(+)]	AKR1A1	sp|P14550
288	Carbonic anhydrase 2	CA2	sp|P00918
289	Alpha-crystallin B chain	CRYAB	sp|P02511
290	6-phosphogluconate dehydrogenase, decarboxyla	PGD	sp|P52209
291	Actin-related protein 2/3 complex subunit 2	ARPC2	sp|O15144
296	X-ray repair cross-complementing protein 5	XRCC5	sp|P13010
300	Serotransferrin	TF	sp|P02787
301	Hornerin	HRNR	sp|Q86YZ3

306	Flotillin-1	FLOT1	tr|B4DVY7
308	Heat shock 70 kDa protein 12A	HSPA12A	sp|O43301
310	Serpin B6	SERPINB6	sp|P35237
314	Calpain-2 catalytic subunit	CAPN2	sp|P17655
315	Chloride intracellular channel protein 5	CLIC5	sp|Q9NZA1
316	Isoform 2 of Polypyrimidine tract-binding protein	PTBP1	sp|P26599-2
319	Cystatin-A	CSTA	sp|P01040
328	Isoform 2 of 14-3-3 protein sigma	SFN	sp|P31947-2
330	Protein S100-A11	S100A11	sp|P31949
333	S-formylglutathione hydrolase	ESD	sp|P10768
334	40S ribosomal protein SA	RPSA	sp|P08865
338	Coronin-1B	CORO1B	sp|Q9BR76
342	ADP-ribosylation factor 6	ARF6	sp|P62330
348	Apolipoprotein A-I	APOA1	sp|P02647
354	Fructose-1,6-bisphosphatase 1	FBP1	sp|P09467
355	Junction plakoglobin	JUP	sp|P14923
359	Protein-glutamine gamma-glutamyltransferase E	TGM3	sp|Q08188
360	60S acidic ribosomal protein P2	RPLP2	sp|P05387
361	Carbonic anhydrase 1	CA1	sp|P00915
363	Ig gamma-2 chain C region	IGHG2	sp|P01859
364	Isoform 2 of Prostaglandin reductase 1	PTGR1	sp|Q14914-2
365	Collagen alpha-2(I) chain	COL1A2	sp|P08123
366	Cytoplasmic aconitate hydratase	ACO1	sp|P21399
377	Microtubule-actin cross-linking factor 1, isoforms	MACF1	sp|Q9UPN3
384	40S ribosomal protein S2	RPS2	sp|P15880
385	Allograft inflammatory factor 1	AIF1	sp|P55008
389	ADP/ATP translocase 3	SLC25A6	sp|P12236
391	Heme-binding protein 1	HEBP1	sp|Q9NRV9
403	1-phosphatidylinositol 4,5-bisphosphate phospho	PLCG2	sp|P16885
413	cAMP-dependent protein kinase type II-alpha reg	PRKAR2A	sp|P13861
414	Keratin, type I cytoskeletal 13	KRT13	sp|P13646
415	Filaggrin-2	FLG2	sp|Q5D862
421	Isoform 2 of Pyruvate dehydrogenase E1 compor	PDHB	sp|P11177-2
426	Adenosylhomocysteinase	AHCY	sp|P23526
427	Succinate dehydrogenase [ubiquinone] flavoprot	SDHA	sp|P31040
429	High mobility group protein B1	HMGB1	sp|P09429
438	Glutathione S-transferase A2	GSTA2	sp|P09210
442	Ester hydrolase C11orf54	C11orf54	sp|Q9H0W9
447	Calmodulin-like protein 5	CALML5	sp|Q9NZT1
452	Isoform 5 of Sorbin and SH3 domain-containing p	SORBS1	sp|Q9BX66-5
454	Collagen alpha-1(I) chain	COL1A1	sp|P02452
459	Prenylcysteine oxidase 1	PCYOX1	sp|Q9UHG3
461	Ig lambda-2 chain C regions	IGLC2	sp|P0CG05
463	SH3 domain-binding glutamic acid-rich-like protei	SH3BGRL	sp|O75368
466	Annexin A11	ANXA11	sp|P50995
467	RNA-binding motif protein, X chromosome	RBMX	sp|P38159
468	N(G),N(G)-dimethylarginine dimethylaminohydro	DDAH2	sp|O95865

470	Proteasome subunit beta type-1	PSMB1	sp|P20618
472	Betaine--homocysteine S-methyltransferase 1	BHMT	sp|Q93088
475	Proteasome subunit alpha type-5	PSMA5	sp|P28066
478	Periaxin	PRX	sp|Q9BXM0
480	T-complex protein 1 subunit theta	CCT8	sp|P50990
481	Isoform 5 of Calpastatin	CAST	sp|P20810-5
484	Isoform 3 of NAD(P)H dehydrogenase [quinone] 1	NQO1	sp|P15559-3
485	Keratin, type II cytoskeletal 4	KRT4	sp|P19013
493	Microtubule-associated protein tau	MAPT	sp|P10636
495	T-complex protein 1 subunit epsilon	CCT5	tr|E9PCA1
497	Isocitrate dehydrogenase [NADP] cytoplasmic	IDH1	sp|O75874
498	Pentatricopeptide repeat-containing protein 1, m	ATP5J2-PTCD1	tr|G3V325
501	Methylmalonate-semialdehyde dehydrogenase [a	ALDH6A1	sp|Q02252
502	Filaggrin	FLG	sp|P20930
510	Signal transducer and activator of transcription 1-	STAT1	sp|P42224
511	Isoform 2 of Eukaryotic translation initiation facto	EIF5A	sp|P63241-2
513	Protein S100-A7	S100A7	sp|P31151
515	Molybdopterin molybdenumtransferase	GPHN	tr|F5H039
516	Integrin beta-3	ITGB3	sp|P05106
518	Adipogenesis regulatory factor	ADIRF	sp|Q15847
519	Rho GTPase-activating protein 1	ARHGAP1	sp|Q07960
521	Microtubule-associated protein 1B	MAP1B	sp|P46821
522	Isoform 2 of Nesprin-2	SYNE2	sp|Q8WXH0-2
523	Periplakin	PPL	sp|O60437
526	Catenin beta-1	CTNNB1	sp|P35222
530	60S ribosomal protein L3	RPL3	sp|P39023
531	Cullin-associated NEDD8-dissociated protein 1	CAND1	sp|Q86VP6
533	40S ribosomal protein S17-like	RPS17L	sp|P0CW22
535	Isoform 3 of Tumor protein D52	TPD52	sp|P55327-3
536	Band 4.1-like protein 5	EPB41L5	sp|Q9HCM4
537	Keratin, type II cuticular Hb5	KRT85	sp|P78386
544	Calpain-1 catalytic subunit	CAPN1	sp|P07384
545	Isoform 2 of TOM1-like protein 2	TOM1L2	sp|Q6ZVM7-2
546	Isoform 2 of Bifunctional purine biosynthesis prot	ATIC	sp|P31939-2
547	Plakophilin-1	PKP1	sp|Q13835
550	Isoform 2 of Protein SET	SET	sp|Q01105-2
552	Isoform 3 of Heterogeneous nuclear ribonucleopr	SYNCRIP	sp|O60506-3
553	Tubulin alpha-4A chain	TUBA4A	sp|P68366
554	Isoform 2 of Partitioning defective 3 homolog B	PARD3B	sp|Q8TEW8-2
556	6-phosphogluconolactonase	PGLS	sp|O95336
557	60S acidic ribosomal protein P0	RPLP0	sp|P05388
558	ATP-dependent RNA helicase A	DHX9	sp|Q08211
560	EH domain-containing protein 1	EHD1	sp|Q9H4M9
561	Aromatic-L-amino-acid decarboxylase	DDC	sp|P20711
562	Galectin-7	LGALS7	sp|P47929
563	Cornulin	CRNN	sp|Q9UBG3
570	Isoform 2 of Leukotriene A-4 hydrolase	LTA4H	sp|P09960-2

571	Isoform Short of Ubiquitin carboxyl-terminal hydı	USP5	sp|P45974-2
572	Fatty acid-binding protein, liver	FABP1	sp|P07148
573	Non-histone chromosomal protein HMG-17	HMGN2	sp|P05204
574	Isoform Long of Proteasome subunit alpha type-1	PSMA1	sp|P25786-2
575	Isoform 2 of Ras-related protein Rab-6A	RAB6A	sp|P20340-2
576	Protein S100-A8	S100A8	sp|P05109
578	Fibroblast growth factor 1	FGF1	sp|P05230
582	Isoform 2 of Annexin A7	ANXA7	sp|P20073-2
583	40S ribosomal protein S19	RPS19	sp|P39019
585	T-complex protein 1 subunit alpha	TCP1	sp|P17987
591	Small nuclear ribonucleoprotein Sm D3	SNRPD3	sp|P62318
595	Cold-inducible RNA-binding protein	CIRBP	sp|Q14011
598	MARCKS-related protein	MARCKSL1	sp|P49006
599	Histone H2A type 1-C	HIST1H2AC	sp|Q93077
603	Histone H2A type 2-A	HIST2H2AA3	sp|Q6FI13
604	Dystroglycan	DAG1	sp|Q14118
605	Isoform 2 of Myosin-11	MYH11	sp|P35749-2
606	Bifunctional ATP-dependent dihydroxyacetone ki	DAK	sp|Q3LXA3
607	Serpin B3	SERPINB3	sp|P29508
610	Serine/arginine-rich splicing factor 3	SRSF3	sp|P84103
614	Isoform XB of Plasma membrane calcium-transpc	ATP2B4	sp|P23634-6
615	Keratin, type II cytoskeletal 74	KRT74	sp|Q7RTS7
617	UV excision repair protein RAD23 homolog B	RAD23B	sp|P54727
619	Immunoglobulin lambda-like polypeptide 5	IGLL5	sp|B9A064
620	Isoform 2 of Dynactin subunit 2	DCTN2	sp|Q13561-2
621	Alcohol dehydrogenase class-3	ADH5	sp|P11766
623	Microtubule-associated protein RP/EB family mei	MAPRE1	sp|Q15691
624	Glyoxylate reductase/hydroxypyruvate reductase	GRHPR	sp|Q9UBQ7
626	Calbindin	CALB1	sp|P05937
627	Parathymosin	PTMS	sp|P20962
629	Cingulin-like protein 1	CGNL1	sp|Q0VF96
631	10 kDa heat shock protein, mitochondrial	HSPE1	sp|P61604
633	Isoform 2 of Proteasome activator complex subu	PSME1	sp|Q06323-2
634	Protein Shroom3	SHROOM3	sp|Q8TF72
635	Isoform 2 of STE20-like serine/threonine-protein	SLK	sp|Q9H2G2-2
637	Keratin, type II cytoskeletal 78	KRT78	sp|Q8N1N4
638	Keratin, type I cytoskeletal 17	KRT17	sp|Q04695
639	Interferon-induced GTP-binding protein Mx1	MX1	sp|P20591
640	Epiplakin	EPPK1	sp|P58107
642	Solute carrier organic anion transporter family m	SLCO2A1	sp|Q92959
647	Heterogeneous nuclear ribonucleoprotein U-like	HNRNPUL2	sp|Q1KMD3
649	Glycogen phosphorylase, brain form	PYGB	sp|P11216
650	Dihydropteridine reductase	QDPR	sp|P09417
651	Calmodulin-like protein 3	CALML3	sp|P27482
652	Envoplakin	EVPL	sp|Q92817
657	Apolipoprotein L2	APOL2	sp|Q9BQE5
659	Isoform 6 of Unconventional myosin-VI	MYO6	sp|Q9UM54-6

660	Isoform p135 of Dynactin subunit 1	DCTN1	sp|Q14203-2
661	Coactosin-like protein	COTL1	sp|Q14019
662	Microtubule-associated protein 6	MAP6	sp|Q96JE9
664	Isoform 2 of Coatomer subunit alpha	COPA	sp|P53621-2
665	Isoform 9 of Neurofascin	NFASC	sp|O94856-9
666	Histone H2AX	H2AFX	sp|P16104
667	Isoform 2 of Myosin-14	MYH14	sp|Q7Z406-2
668	Isoform 2 of Allograft inflammatory factor 1-like	AIF1L	sp|Q9BQI0-2
669	Isoform 2 of Glycine amidinotransferase, mitocho	GATM	sp|P50440-2
670	Isoform 3 of Thioredoxin reductase 1, cytoplasmi	TXNRD1	sp|Q16881-3
672	Lupus La protein	SSB	sp|P05455
678	Isoform 2 of 40S ribosomal protein S20	RPS20	sp|P60866-2
679	40S ribosomal protein S6	RPS6	sp|P62753
683	GTP-binding nuclear protein Ran	RAN	sp|P62826
684	SH3 domain-binding glutamic acid-rich-like protei	SH3BGRL2	sp|Q9UJC5
686	Zinc-alpha-2-glycoprotein	AZGP1	sp|P25311
689	Purine nucleoside phosphorylase	PNP	sp|P00491
690	Isoform 5 of Acyl-CoA-binding protein	DBI	sp|P07108-5
692	T-complex protein 1 subunit gamma	CCT3	tr|B4DUR8
693	Polyadenylate-binding protein 1	PABPC1	tr|E7ERJ7
694	Caspase-14	CASP14	sp|P31944
700	Isoform 2 of Ras-related protein Rab-2A	RAB2A	sp|P61019-2
704	Astrocytic phosphoprotein PEA-15	PEA15	sp|Q15121
706	Proteasome subunit alpha type-6	PSMA6	sp|P60900
708	Proteasome subunit alpha type-2	PSMA2	sp|P25787
710	Peptidyl-prolyl cis-trans isomerase FKBP4	FKBP4	sp|Q02790
711	Ig kappa chain V-III region SIE	1	sp|P01620
712	Isoform 2 of Coagulation factor IX	F9	sp|P00740-2
718	Heterogeneous nuclear ribonucleoprotein F	HNRNPF	sp|P52597
719	Isoform 4 of Signal recognition particle subunit SI	SRP68	sp|Q9UHB9-4
722	Extended synaptotagmin-2 (Fragment)	ESYT2	tr|H7BXI1
724	HLA class II histocompatibility antigen, DRB1-15 b	HLA-DRB1	sp|P01911
728	General vesicular transport factor p115	USO1	tr|F5H4X1
729	Importin subunit beta-1	KPNB1	sp|Q14974
730	Superoxide dismutase [Cu-Zn]	SOD1	sp|P00441
731	Keratin, type II cytoskeletal 6C	KRT6C	sp|P48668
733	Band 3 anion transport protein	SLC4A1	sp|P02730
734	Aminopeptidase N	ANPEP	sp|P15144
735	TRIO and F-actin-binding protein	TRIOBP	sp|Q9H2D6
736	Ig gamma-4 chain C region	IGHG4	sp|P01861
737	Ribosyldihydronicotinamide dehydrogenase [quir	NQO2	sp|P16083
739	Keratin, type II cytoskeletal 1b	KRT77	sp|Q7Z794
743	Isoform 2 of Nucleosome assembly protein 1-like	NAP1L4	sp|Q99733-2
744	Proteasome activator complex subunit 2	PSME2	sp|Q9UL46
746	ADP-ribosylation factor-like protein 8B	ARL8B	sp|Q9NVJ2
747	V-type proton ATPase subunit B, brain isoform	ATP6V1B2	sp|P21281
748	Isoform 2 of Eukaryotic initiation factor 4A-II	EIF4A2	sp|Q14240-2

750	Adenylate kinase isoenzyme 1	AK1	sp|P00568
751	Isoform 2 of Inositol-3-phosphate synthase 1	ISYNA1	sp|Q9NPH2-2
752	Keratin, type I cuticular Ha1	KRT31	sp|Q15323
753	Isoform 1 of Four and a half LIM domains protein	FHL1	sp|Q13642-1
756	Heat shock-related 70 kDa protein 2	HSPA2	sp|P54652
757	Adapter molecule crk	CRK	sp|P46108
758	Ras-related protein Rab-10	RAB10	sp|P61026
759	Serum deprivation-response protein	SDPR	sp|O95810
760	Coatomer subunit gamma-1	COPG1	sp|Q9Y678
763	Isoform 2 of Secernin-1	SCRN1	sp|Q12765-2
766	Isoform 2 of AP-2 complex subunit alpha-2	AP2A2	sp|O94973-2
767	Isoform 3 of NSFL1 cofactor p47	NSFL1C	sp|Q9UNZ2-5
770	Stress-induced-phosphoprotein 1	STIP1	tr|G3XAD8
771	Isoform 2 of Ankycorbin	RAI14	sp|Q9P0K7-2
773	SH3 and multiple ankyrin repeat domains protein	SHANK3	tr|M0QWZ9
774	Aspartate aminotransferase, cytoplasmic	GOT1	sp|P17174
775	60S ribosomal protein L13a (Fragment)	RPL13A	tr|M0QYS1
776	Ketimine reductase mu-crystallin	CRYM	sp|Q14894
777	Apolipoprotein B-100	APOB	sp|P04114
780	Isoform 2 of Arginase-1	ARG1	sp|P05089-2
783	Prolyl endopeptidase	PREP	sp|P48147
784	Sulfotransferase 1A1 (Fragment)	SULT1A1	tr|H3BRY5
786	26S protease regulatory subunit 10B	PSMC6	sp|P62333
788	Heterogeneous nuclear ribonucleoprotein D-like	HNRNPDL	sp|O14979
790	60S ribosomal protein L5	RPL5	sp|P46777
791	Isoform 2 of Protein TFG	TFG	sp|Q92734-2
793	Translationally-controlled tumor protein	TPT1	tr|Q5W0H4
794	Vigilin (Fragment)	HDLBP	tr|H0Y394
796	Isoform 2 of Inter-alpha-trypsin inhibitor heavy cl	ITIH4	sp|Q14624-2
798	Coatomer subunit delta	ARCN1	sp|P48444
799	Tubulin-specific chaperone A	TBCA	sp|O75347
800	Isoform 4 of Collagen alpha-1(XII) chain	COL12A1	sp|Q99715-4
801	Isoform 1 of Protein POF1B	POF1B	sp|Q8WVV4-1
802	Isoform 2 of 26S proteasome non-ATPase regulat	PSMD11	sp|O00231-2
803	Thioredoxin-like protein 1 (Fragment)	TXNL1	tr|K7ER96
804	Proteasome subunit beta type-3	PSMB3	sp|P49720
805	Papilin	PAPLN	sp|O95428
806	S-phase kinase-associated protein 1	SKP1	sp|P63208
808	2'-deoxynucleoside 5'-phosphate N-hydrolase 1 (	DNPH1	tr|H0Y8X4
809	3-ketoacyl-CoA thiolase, mitochondrial	ACAA2	sp|P42765
810	Ribonuclease UK114	HRSP12	sp|P52758
813	26S proteasome non-ATPase regulatory subunit 3	PSMD3	sp|O43242
814	Ketohexokinase (Fragment)	KHK	tr|C9JDL1
817	Glycerol-3-phosphate dehydrogenase [NAD(+)], c	GPD1	sp|P21695
818	Gasdermin-A	GSDMA	sp|Q96QA5
819	Isoform 2 of LIM and senescent cell antigen-like-c	LIMS1	sp|P48059-2
821	Isoform 2 of Bone marrow stromal antigen 2	BST2	sp|Q10589-2

822	Ubiquitin-conjugating enzyme E2 variant 2	UBE2V2	sp|Q15819
824	Isoform 2 of Glutathione S-transferase Mu 2	GSTM2	sp|P28161-2
827	Antithrombin-III	SERPINC1	sp|P01008
828	Pyridoxal kinase	PDXK	tr|F2Z2Y4
829	Retinol-binding protein 4	RBP4	sp|P02753
830	Isoform 3 of 6-phosphofructokinase, muscle type	PFKM	sp|P08237-3
831	ATP-citrate synthase	ACLY	sp|P53396
832	60S ribosomal protein L9	RPL9	sp|P32969
833	Fatty acid-binding protein, epidermal	FABP5	sp|Q01469
834	Fumarylacetoacetase	FAH	sp|P16930
835	Isoform 1B of Desmocollin-1	DSC1	sp|Q08554-2
836	Keratin, type II cytoskeletal 80	KRT80	sp|Q6KB66
839	ATP synthase subunit d, mitochondrial	ATP5H	sp|O75947
840	Aflatoxin B1 aldehyde reductase member 2 (Frag	AKR7A2	tr|H3BLU7
841	Eukaryotic translation initiation factor 4B	EIF4B	tr|E7EX17
843	Isoform 2 of Destrin	DSTN	sp|P60981-2
844	Eukaryotic initiation factor 4A-III	EIF4A3	sp|P38919
846	Charged multivesicular body protein 4b	CHMP4B	sp|Q9H444
847	Pterin-4-alpha-carbinolamine dehydratase	PCBD1	sp|P61457
848	Isoform C of Fibulin-1	FBLN1	sp|P23142-4
849	Copine-3	CPNE3	sp|O75131
850	Formin-binding protein 1-like	FNBP1L	sp|Q5T0N5
852	Isoform 2 of Coronin-2B	CORO2B	sp|Q9UQ03-2
853	Glutathione peroxidase 1	GPX1	sp|P07203
854	Isoform 4 of Acyl-coenzyme A thioesterase 9, mit	ACOT9	sp|Q9Y305-4
856	Chloride intracellular channel protein 2	CLIC2	sp|O15247
857	Ceruloplasmin	CP	sp|P00450
858	Apolipoprotein A-IV	APOA4	sp|P06727
859	Integrin alpha-8	ITGA8	sp|P53708
860	Isoform 4 of Deoxynucleoside triphosphate triph	SAMHD1	sp|Q9Y3Z3-4
861	Keratin, type II cytoskeletal 2 oral	KRT76	sp|Q01546
862	Glia maturation factor beta	GMFB	sp|P60983
863	Suprabasin	SBSN	sp|Q6UWP8
864	Alpha-2-macroglobulin-like protein 1	A2ML1	sp|A8K2U0
865	Isoform 2 of COP9 signalosome complex subunit	GPS1	sp|Q13098-7
867	2-oxoglutarate dehydrogenase, mitochondrial	OGDH	sp|Q02218
868	Leucine-rich repeat-containing protein 47	LRRC47	sp|Q8N1G4
869	Sorting nexin-3	SNX3	sp|O60493
870	Adenine phosphoribosyltransferase	APRT	sp|P07741
871	Secretory phospholipase A2 receptor	PLA2R1	sp|Q13018
872	Hsc70-interacting protein	ST13	sp|P50502
873	Valine--tRNA ligase	VARS	sp|P26640
874	Isoform 2 of Protein CDV3 homolog	CDV3	sp|Q9UKY7-2
875	SERPINB12 protein	SERPINB12	tr|Q3SYB4
876	EF-hand domain-containing protein D1	EFHD1	sp|Q9BUP0
877	Isoform 1B of Beta-arrestin-1	ARRB1	sp|P49407-2
878	Nicotinate-nucleotide pyrophosphorylase [carbo	QPRT	sp|Q15274

879	Acyl-coenzyme A synthetase ACSM2B, mitochonc	ACSM2B	sp|Q68CK6
880	Isoform DeltaLf of Lactotransferrin	LTF	sp|P02788-2
881	Phosphoenolpyruvate carboxykinase [GTP], mito	PCK2	sp|Q16822
882	Aspartyl aminopeptidase	DNPEP	sp|Q9ULA0
883	Isoform 2 of Calumenin	CALU	sp|O43852-2
884	1-phosphatidylinositol 4,5-bisphosphate phosphc	PLCB3	sp|Q01970
885	UTP--glucose-1-phosphate uridylyltransferase	UGP2	tr|E7EUC7
886	DCC-interacting protein 13-alpha	APPL1	sp|Q9UKG1
887	Isoform 3 of Afadin	MLLT4	sp|P55196-3
888	Protein SOGA2	SOGA2	sp|Q9Y4B5
889	Collagen alpha-1(III) chain	COL3A1	sp|P02461
890	Involucrin	IVL	sp|P07476
891	Isoform 3 of CD99 antigen	CD99	sp|P14209-3
892	Putative RNA-binding protein Luc7-like 2	LUC7L2	sp|Q9Y383
893	Serine/threonine-protein kinase R1	OXSR1	sp|O95747
894	Isoform 2 of Macrophage-capping protein	CAPG	sp|P40121-2
895	Transcription elongation factor A protein-like 3	TCEAL3	sp|Q969E4
898	Isoform 2 of Rho guanine nucleotide exchange fa	ARHGEF12	sp|Q9NZN5-2
899	3-hydroxybutyrate dehydrogenase type 2	BDH2	sp|Q9BUT1
901	Elongation factor 1-beta	EEF1B2	sp|P24534
902	26S protease regulatory subunit 6B	PSMC4	sp|P43686
903	Thrombospondin type-1 domain-containing prote	THSD7A	sp|Q9UPZ6
905	Keratinocyte proline-rich protein	KPRP	sp|Q5T749
908	Keratin, type I cuticular Ha5	KRT35	tr|C4AM86
909	60 kDa SS-A/Ro ribonucleoprotein	TROVE2	sp|P10155
912	Glutathione S-transferase Mu 3	GSTM3	sp|P21266
913	Proteasome subunit alpha type-4	PSMA4	sp|P25789
914	Aldose 1-epimerase	GALM	sp|Q96C23
915	Kelch repeat and BTB domain-containing protein	KBTBD11	sp|O94819
917	Isoform 2 of UDP-glucose:glycoprotein glucosyltr	UGGT1	sp|Q9NYU2-2
918	Eukaryotic translation initiation factor 3 subunit (	EIF3CL	sp|B5ME19
919	1,4-alpha-glucan-branching enzyme	GBE1	sp|Q04446
920	Isoform 2 of Keratin, type II cytoskeletal 8	KRT8	sp|P05787-2
921	Periostin	POSTN	sp|Q15063
922	Inorganic pyrophosphatase	PPA1	sp|Q15181
923	von Willebrand factor	VWF	sp|P04275
924	Ubiquilin-1	UBQLN1	sp|Q9UMX0
925	Protein phosphatase 1 regulatory subunit 7	PPP1R7	sp|Q15435
926	Desmoglein-3	DSG3	sp|P32926
927	L-xylulose reductase (Fragment)	DCXR	tr|J3QS36
928	F-box only protein 50	NCCRP1	sp|Q6ZVX7
929	Isoform PKP3b of Plakophilin-3	PKP3	sp|Q9Y446-2
930	Isoform 2 of Enoyl-CoA delta isomerase 1, mitoch	ECI1	sp|P42126-2
931	Serine/threonine-protein phosphatase 2A 55 kDa	PPP2R2A	sp|P63151
932	Peptidyl-prolyl cis-trans isomerase NIMA-interact	PIN1	sp|Q13526
933	Splicing factor 3B subunit 3	SF3B3	sp|Q15393
935	Isoform 2 of Uveal autoantigen with coiled-coil d	UACA	sp|Q9BZF9-2

936	Histidine triad nucleotide-binding protein 1	HINT1	sp|P49773
937	Endoplasmic reticulum resident protein 29	ERP29	sp|P30040
939	26S proteasome non-ATPase regulatory subunit 1	PSMD14	sp|O00487
940	Engulfment and cell motility protein 2	ELMO2	tr|B4DRL5
941	D-3-phosphoglycerate dehydrogenase	PHGDH	sp|O43175
942	Isoform 3 of Ubiquitin-conjugating enzyme E2 L3	UBE2L3	sp|P68036-3
943	Alpha-1-acid glycoprotein 1	ORM1	sp|P02763
944	Bleomycin hydrolase	BLMH	sp|Q13867
946	Eukaryotic translation initiation factor 3 subunit E	EIF3E	sp|P60228
947	Putative uncharacterized protein	SAOUHSC_01873	tr|Q2FXH4
948	UMP-CMP kinase	CMPK1	sp|P30085
949	HBV PreS1-transactivated protein 1	MXRA7	tr|Q6ZR64
951	Septin-8	SEPT8	sp|Q92599
952	Ig kappa chain V-I region EU	1	sp|P01598
953	Isoform 5 of 15-hydroxyprostaglandin dehydroge	HPGD	sp|P15428-5
955	26S proteasome non-ATPase regulatory subunit 1	PSMD12	sp|O00232
956	Isoform 3B of Desmocollin-3	DSC3	sp|Q14574-2
957	Tropomodulin-1	TMOD1	sp|P28289
958	D-dopachrome decarboxylase	DDT	sp|P30046
959	26S protease regulatory subunit 6A	PSMC3	sp|P17980
960	Acylamino-acid-releasing enzyme	APEH	tr|C9JIF9
961	Thymosin beta-10	TMSB10	sp|P63313
962	Isoform 4 of Tyrosine-protein phosphatase non-r	SIRPA	sp|P78324-4
963	Inhibin beta E chain	INHBE	sp|P58166
964	Complement component C7	C7	sp|P10643
965	Vascular endothelial growth factor receptor 1	FLT1	sp|P17948
966	Xaa-Pro dipeptidase	PEPD	sp|P12955
967	Small subunit processome component 20 homolo	UTP20	sp|O75691
968	Protein phosphatase 1 regulatory subunit 12C	PPP1R12C	tr|B4DME2
969	Arachidonate 12-lipoxygenase, 12R-type	ALOX12B	sp|O75342
972	Leucine--tRNA ligase, cytoplasmic	LARS	sp|Q9P2J5
973	Insulin-degrading enzyme	IDE	sp|P14735
974	Isoform 2 of 1-phosphatidylinositol 4,5-bisphospl	PLCD1	sp|P51178-2
975	Isoform 2 of Leucine-rich repeat-containing prote	LRRC15	sp|Q8TF66-2
976	Keratin, type II cuticular Hb2	KRT82	sp|Q9NSB4
977	Estradiol 17-beta-dehydrogenase 12	HSD17B12	sp|Q53GQ0
978	Keratin, type II cytoskeletal 6B	KRT6B	sp|P04259
980	Y-box-binding protein 3	YBX3	sp|P16989
981	Alpha-soluble NSF attachment protein	NAPA	sp|P54920
982	26S protease regulatory subunit 4	PSMC1	sp|P62191
983	40S ribosomal protein S15a	RPS15A	sp|P62244
984	DNA-(apurinic or apyrimidinic site) lyase	APEX1	sp|P27695
985	Cystatin-C	CST3	sp|P01034
986	Isoform 2 of 60S ribosomal protein L17	RPL17	sp|P18621-2
987	Dual specificity protein phosphatase 3	DUSP3	sp|P51452
988	40S ribosomal protein S13	RPS13	sp|P62277
989	Keratin, type I cuticular Ha6	KRT36	sp|O76013

990	CD2-associated protein	CD2AP	sp|Q9Y5K6
992	Dynein light chain 1, cytoplasmic	DYNLL1	sp|P63167
993	Retroviral-like aspartic protease 1	ASPRV1	sp|Q53RT3
994	Galactokinase	GALK1	sp|P51570
995	Keratin, type I cytoskeletal 15	KRT15	sp|P19012
996	Protein-glutamine gamma-glutamyltransferase K	TGM1	sp|P22735
997	Isoform 2 of Integrator complex subunit 3	INTS3	sp|Q68E01-2
998	Desmoglein-4	DSG4	sp|Q86SJ6
999	HLA class I histocompatibility antigen, B-44 alpha	HLA-B	sp|P30481
1000	Semenogelin-1	SEMG1	sp|P04279
1001	Serum amyloid A-1 protein	SAA1	sp|P0DJI8
1002	Dipeptidyl peptidase 4	DPP4	sp|P27487
1003	Histidine-rich glycoprotein	HRG	sp|P04196
1004	Charged multivesicular body protein 5	CHMP5	sp|Q9NZZ3
1005	Isoform 2 of Hydroxyacylglutathione hydrolase, n	HAGH	sp|Q16775-2
1006	5'(3')-deoxyribonucleotidase, cytosolic type	NT5C	sp|Q8TCD5
1007	Ubiquitin-fold modifier 1	UFM1	sp|P61960
1008	HLA class I histocompatibility antigen, A-34 alpha	HLA-A	sp|P30453
1009	Complement component C8 beta chain	C8B	sp|P07358
1010	Isoform 3 of PTB domain-containing engulfment a	GULP1	sp|Q9UBP9-3
1011	Isoform 2 of 40S ribosomal protein S24	RPS24	sp|P62847-2
1012	Isoform 2 of Plakophilin-4	PKP4	sp|Q99569-2
1013	Small nuclear ribonucleoprotein Sm D1	SNRPD1	sp|P62314
1014	Dipeptidyl aminopeptidase-like protein 6	DPP6	tr|E9PF59
1015	60S ribosomal protein L15	RPL15	sp|P61313
1016	Echinoderm microtubule-associated protein-like 4	EML4	tr|B5MBZ0
1017	Complement C1q subcomponent subunit B	C1QB	sp|P02746
1018	Lysosomal alpha-glucosidase	GAA	sp|P10253
1019	SUMO-activating enzyme subunit 1	SAE1	sp|Q9UBE0
1020	Putative protein RFPL3S	RFPL3S	sp|P0C7P2
1021	Eukaryotic translation initiation factor 3 subunit L	EIF3L	tr|B0QY89
1022	Isoform 3 of Guanine deaminase	GDA	sp|Q9Y2T3-3
1023	Myristoylated alanine-rich C-kinase substrate	MARCKS	sp|P29966
1024	Fatty acid synthase	FASN	sp|P49327
1025	3-hydroxyisobutyryl-CoA hydrolase, mitochondria	HIBCH	sp|Q6NVY1
1026	Isoform 2 of Interleukin-36 gamma	IL36G	sp|Q9NZH8-2
1027	NADH dehydrogenase [ubiquinone] flavoprotein 2	NDUFV2	sp|P19404
1028	Hepatoma-derived growth factor-related protein	HDGFRP3	sp|Q9Y3E1
1029	Asparagine--tRNA ligase, cytoplasmic	NARS	sp|O43776
1030	Proteasome subunit beta type-2	PSMB2	sp|P49721
1031	Lipopolysaccharide-binding protein	LBP	sp|P18428
1032	Protein NipSnap homolog 3A	NIPSNAP3A	sp|Q9UFN0
1033	Sepiapterin reductase	SPR	sp|P35270
1034	Neural Wiskott-Aldrich syndrome protein	WASL	sp|O00401
1035	ATP-dependent RNA helicase DDX1	DDX1	sp|Q92499
1036	Gamma-glutamylcyclotransferase	GGCT	sp|O75223
1037	Isoform 3 of Ran-binding protein 3	RANBP3	sp|Q9H6Z4-3

1039	Keratin, type II cuticular Hb3	KRT83	sp|P78385
1040	Fatty acid-binding protein, heart	FABP3	sp|P05413
1041	Keratin, type II cuticular Hb4	KRT84	sp|Q9NSB2
1042	Serpin B5	SERPINB5	sp|P36952
1043	Coatomer subunit epsilon	COPE	sp|O14579
1044	Putative keratin-87 protein	KRT87P	sp|A6NCN2
1045	Na(+)/H(+) exchange regulatory cofactor NHE-RF3	PDZK1	sp|Q5T2W1
1046	High mobility group protein B2	HMGB2	sp|P26583
1047	Beta-centractin	ACTR1B	sp|P42025
1048	Isoform 6 of Inosine-5'-monophosphate dehydro	IMPDH1	sp|P20839-6
1049	26S proteasome non-ATPase regulatory subunit 4	PSMD4	tr|Q5VWC4
1050	Protein S100-A2	S100A2	tr|R4GN49
1051	Heme oxygenase 1	HMOX1	sp|P09601
1052	Cytochrome b-c1 complex subunit Rieske, mitoch	UQCRFS1	sp|P47985
1053	Isoform A of Protein CutA	CUTA	sp|O60888-2
1054	Leucine-rich repeat-containing protein 40	LRRC40	sp|Q9H9A6
1055	Isoform 2 of Histidine ammonia-lyase	HAL	sp|P42357-2
1057	Keratin, type I cytoskeletal 23	KRT23	sp|Q9C075
1058	Isoform B of Osteopontin	SPP1	sp|P10451-2
1059	Nuclear ubiquitous casein and cyclin-dependent k	NUCKS1	sp|Q9H1E3
1060	Guanine nucleotide-binding protein subunit alpha	GNA13	sp|Q14344
1061	Isoform 2 of Stathmin	STMN1	sp|P16949-2
1062	Phenylalanine--tRNA ligase beta subunit	FARSB	sp|Q9NSD9
1063	Cytosolic 5'-nucleotidase 3A	NT5C3A	sp|Q9H0P0
1065	Isoform 2 of N-acetyl-D-glucosamine kinase	NAGK	sp|Q9UJ70-2
1066	Hemopexin	HPX	sp|P02790
1067	40S ribosomal protein S15	RPS15	sp|P62841
1068	V-set and immunoglobulin domain-containing pro	VSIG8	sp|Q5VU13
1069	Keratin, type I cuticular Ha3-II	KRT33B	sp|Q14525
1070	Isoform 2 of Ficolin-2	FCN2	sp|Q15485-2
1071	Three prime repair exonuclease 2	TREX2	sp|Q9BQ50
1072	Casein kinase I isoform epsilon (Fragment)	CSNK1E	tr|H0Y645
1073	Isoform 2 of Derlin-1	DERL1	sp|Q9BUN8-2

Gene Locus	Molecular '	Protein Grc	Total Spect SAGN G1	Total Spect SAGN G2	Total Spect SAGN G11	Total Spect SAGN G12	Total Spect IgAN E0C1 G4	Total Spect IgAN E0C0 G8
MYH9_HUMAN	227 kDa	TRUE	123	153	167	153	151	114
VIME_HUMAN	54 kDa	TRUE	117	94	131	145	136	103
PLEC_HUMAN	516 kDa	TRUE	73	50	44	40	40	57
FLNA_HUMAN	280 kDa	TRUE	60	63	49	59	54	43
ACTN4_HUMAN	105 kDa	TRUE	59	59	73	73	77	66
ACTB_HUMAN	42 kDa	TRUE	58	78	78	81	71	77
NEST_HUMAN	177 kDa	TRUE	54	47	50	54	51	48
AHNK_HUMAN	629 kDa		51	54	42	42	50	40
LMNA_HUMAN	74 kDa	TRUE	50	49	59	57	49	45
TLN1_HUMAN	270 kDa		49	54	52	43	55	55
E9PCV6_HUMAN	322 kDa	TRUE	46	80	47	31	44	45
ACTC_HUMAN	42 kDa	TRUE	44	67	58	65	53	63
K1C10_HUMAN	59 kDa	TRUE	42	41	79	94	48	36
SPTN1_HUMAN	282 kDa		42	27	30	26	32	24
LAMA5_HUMAN	400 kDa		39	44	45	26	46	43
VINC_HUMAN	117 kDa		37	38	44	29	32	43
K2C1_HUMAN	66 kDa	TRUE	36	40	71	59	53	29
LAMB2_HUMAN	196 kDa	TRUE	36	60	43	31	44	47
SPTB2_HUMAN	275 kDa	TRUE	36	32	32	36	38	32
FINC_HUMAN	256 kDa		36	43	39	61	38	23
PGBM_HUMAN	469 kDa		34	59	42	25	36	33
K22E_HUMAN	65 kDa	TRUE	31	38	83	110	41	24
LAMC1_HUMAN	178 kDa		30	39	44	21	34	35
TPM4_HUMAN	29 kDa	TRUE	30	31	29	30	29	25
CALD1_HUMAN	63 kDa		28	34	18	21	20	20
K1C9_HUMAN	62 kDa	TRUE	27	48	85	36	35	26
MOES_HUMAN	68 kDa	TRUE	26	28	35	32	36	21
CO3_HUMAN	187 kDa		25	52	26	45	34	31
ANXA2_HUMAN	40 kDa	TRUE	23	21	20	22	23	21
ACTN1_HUMAN	103 kDa	TRUE	22	26	17	19	18	13
TPM2_HUMAN	33 kDa	TRUE	22	21	0	22	20	18
SYNPO_HUMAN	96 kDa		21	23	25	18	26	25
MYH10_HUMAN	231 kDa	TRUE	20	27	20	14	17	12
TPM3_HUMAN	29 kDa	TRUE	20	25	21	26	24	22
TBB4B_HUMAN	50 kDa	TRUE	19	24	31	32	18	20
NID1_HUMAN	122 kDa	TRUE	18	24	25	22	25	23
TBA1B_HUMAN	50 kDa	TRUE	18	22	24	27	19	17
TBB5_HUMAN	50 kDa	TRUE	18	26	31	36	19	18
Q6ZN40_HUMAN	37 kDa	TRUE	18	17	14	19	19	15
ACTBL_HUMAN	42 kDa	TRUE	18	27	23	20	23	0
ZO1_HUMAN	195 kDa	TRUE	17	9	12	18	12	16
PDLI2_HUMAN	63 kDa		17	14	21	18	19	18
GRP78_HUMAN	72 kDa	TRUE	17	10	12	18	10	11

H4_HUMAN	11 kDa		16	16	21	16	20	13
HSP7C_HUMAN	71 kDa	TRUE	16	15	10	17	13	13
H7BYY1_HUMAN	29 kDa	TRUE	16	15	16	17	19	15
HBB_HUMAN	16 kDa	TRUE	15	20	16	24	20	22
GELS_HUMAN	81 kDa		15	18	21	27	16	14
CALM_HUMAN	17 kDa	TRUE	15	16	19	21	11	17
K2C5_HUMAN	62 kDa	TRUE	15	19	28	25	16	11
EZRI_HUMAN	69 kDa	TRUE	15	11	17	10	13	13
IQGA2_HUMAN	181 kDa	TRUE	14	9	16	10	16	11
AGRIN_HUMAN	215 kDa	TRUE	14	16	12	9	16	18
ATPA_HUMAN	60 kDa		14	17	9	14	14	13
PDIA3_HUMAN	57 kDa	TRUE	14	12	12	14	12	10
TBB2A_HUMAN	50 kDa	TRUE	14	22	23	28	16	17
K1C14_HUMAN	52 kDa	TRUE	13	14	34	21	19	13
ATPB_HUMAN	57 kDa	TRUE	13	13	19	18	14	11
CO6A1_HUMAN	109 kDa		13	15	13	4	9	9
EHD3_HUMAN	61 kDa	TRUE	13	7	10	9	8	8
HSP71_HUMAN	70 kDa	TRUE	12	11	15	13	12	9
NEBL_HUMAN	31 kDa	TRUE	12	6	11	10	11	13
HSPB1_HUMAN	23 kDa		12	13	9	13	14	10
H31_HUMAN	15 kDa	TRUE	12	13	13	12	12	10
FLNB_HUMAN	282 kDa	TRUE	12	18	6	13	13	7
H2A1_HUMAN	14 kDa	TRUE	11	11	19	22	10	9
CO6A2_HUMAN	109 kDa	TRUE	11	16	9	6	7	7
LMNB2_HUMAN	68 kDa	TRUE	11	11	12	6	7	9
ML12B_HUMAN	20 kDa	TRUE	11	9	11	9	8	9
RS27A_HUMAN	18 kDa		10	12	17	18	13	11
ROA2_HUMAN	37 kDa	TRUE	10	13	15	18	12	6
MYO1C_HUMAN	122 kDa	TRUE	10	8	8	7	12	13
H14_HUMAN	22 kDa	TRUE	10	10	10	8	10	10
TAGL2_HUMAN	22 kDa		10	11	8	10	8	8
ZN185_HUMAN	74 kDa		10	7	11	6	10	9
LASP1_HUMAN	30 kDa		10	12	7	9	9	11
POTEI_HUMAN	121 kDa	TRUE	10	0	0	0	0	0
DPYL2_HUMAN	62 kDa	TRUE	9	17	16	18	12	14
K2C6A_HUMAN	60 kDa	TRUE	9	11	23	21	13	0
ANXA1_HUMAN	39 kDa	TRUE	9	15	16	18	13	5
HS90A_HUMAN	98 kDa	TRUE	9	9	15	16	7	5
TERA_HUMAN	89 kDa		9	9	10	10	12	6
HNRPK_HUMAN	51 kDa		9	6	9	10	6	8
1433Z_HUMAN	28 kDa	TRUE	9	11	12	10	11	12
PODO_HUMAN	42 kDa		9	5	11	11	7	8
HS90B_HUMAN	83 kDa	TRUE	9	12	15	19	13	10
E9PMS6_HUMAN	145 kDa		9	3	3	2	6	9
MYL9_HUMAN	20 kDa	TRUE	9	8	9	5	6	10
CLH1_HUMAN	188 kDa		8	9	15	15	11	8
PDLI5_HUMAN	64 kDa		8	7	15	7	14	4

BCAM_HUMAN	67 kDa		8	4	6	7	13	12
G8JLA2_HUMAN	17 kDa		8	8	10	13	8	7
COIA1_HUMAN	154 kDa		8	11	8	5	6	7
FIBG_HUMAN	49 kDa	TRUE	8	4	10	21	13	9
AT1A1_HUMAN	113 kDa	TRUE	8	3	9	4	7	3
ALDOA_HUMAN	45 kDa	TRUE	8	4	11	7	2	5
PROF1_HUMAN	15 kDa		8	9	8	7	9	5
PALLD_HUMAN	151 kDa		8	1	3	7	5	6
RADI_HUMAN	69 kDa	TRUE	8	11	7	6	10	8
TBB6_HUMAN	50 kDa	TRUE	8	11	10	12	8	8
HBA_HUMAN	15 kDa		7	17	21	26	22	20
G3P_HUMAN	36 kDa		7	13	22	24	13	12
ENOA_HUMAN	47 kDa	TRUE	7	9	7	13	7	7
CO4A2_HUMAN	168 kDa		7	12	12	7	7	6
PPIA_HUMAN	18 kDa	TRUE	7	9	14	15	8	8
DYHC1_HUMAN	532 kDa		7	5	6	9	6	9
NHRF2_HUMAN	37 kDa	TRUE	7	7	8	10	8	8
SEPT2_HUMAN	45 kDa		7	8	8	9	6	16
PRDX1_HUMAN	22 kDa	TRUE	7	7	7	11	6	6
ENPL_HUMAN	92 kDa	TRUE	7	6	4	10	7	4
K1C16_HUMAN	51 kDa	TRUE	7	0	25	14	14	8
TGM2_HUMAN	77 kDa	TRUE	7	13	8	12	7	7
VTNC_HUMAN	54 kDa		7	8	7	8	9	4
CKAP4_HUMAN	66 kDa		7	5	3	3	5	2
HBD_HUMAN	16 kDa	TRUE	7	10	8	12	10	13
H2B2E_HUMAN	14 kDa	TRUE	7	13	0	0	0	7
H2B1C_HUMAN	14 kDa	TRUE	6	13	13	14	14	7
ANXA5_HUMAN	36 kDa		6	11	11	15	6	7
HNRPM_HUMAN	74 kDa		6	5	7	15	8	6
FIBB_HUMAN	56 kDa		6	2	7	23	23	5
IGKC_HUMAN	12 kDa		6	9	9	9	10	6
E9PGF5_HUMAN	183 kDa	TRUE	6	8	2	5	6	6
NHRF1_HUMAN	39 kDa		6	3	5	5	5	7
ZYX_HUMAN	61 kDa		6	6	6	8	5	5
TENC1_HUMAN	154 kDa	TRUE	6	3	7	9	9	6
1433G_HUMAN	28 kDa	TRUE	6	10	8	11	6	9
SEPT9_HUMAN	65 kDa	TRUE	6	4	3	4	3	2
STOM_HUMAN	32 kDa		6	4	1	0	2	2
1B49_HUMAN	41 kDa	TRUE	6	0	0	9	3	4
1433E_HUMAN	29 kDa	TRUE	5	11	11	6	7	7
KPYM_HUMAN	58 kDa	TRUE	5	9	15	19	11	4
FIBA_HUMAN	70 kDa		5	6	9	46	23	10
UBA1_HUMAN	118 kDa		5	12	16	11	14	9
IGHG1_HUMAN	36 kDa	TRUE	5	15	3	7	1	4
EF1A1_HUMAN	50 kDa		5	7	5	7	5	5
SRC8_HUMAN	62 kDa		5	6	0	9	4	5
ILK_HUMAN	51 kDa		5	3	11	3	5	5

TAGL_HUMAN	23 kDa		5	11	3	7	2	5
CAP1_HUMAN	52 kDa		5	10	3	5	6	4
DPYL3_HUMAN	74 kDa	TRUE	5	5	11	7	5	5
ROA1_HUMAN	29 kDa	TRUE	5	4	4	8	4	5
HNRH1_HUMAN	49 kDa	TRUE	5	5	7	8	7	2
1B67_HUMAN	40 kDa	TRUE	5	4	8	8	4	5
CALR_HUMAN	48 kDa		5	4	2	12	6	0
1A11_HUMAN	41 kDa	TRUE	5	3	7	8	7	5
CO9_HUMAN	63 kDa		5	7	3	3	5	4
CNN3_HUMAN	36 kDa	TRUE	5	1	4	4	5	4
APOE_HUMAN	36 kDa		5	4	1	3	6	2
ANXA4_HUMAN	36 kDa	TRUE	5	7	2	5	1	4
PDLI1_HUMAN	36 kDa		5	4	4	6	4	5
B1AK88_HUMAN	34 kDa		5	4	2	3	3	4
HNRH3_HUMAN	32 kDa		5	6	2	2	4	2
RAB14_HUMAN	24 kDa	TRUE	5	3	1	2	3	0
EMIL1_HUMAN	107 kDa		5	5	2	3	1	3
1B07_HUMAN	40 kDa	TRUE	5	0	6	6	4	5
1A68_HUMAN	41 kDa	TRUE	5	5	10	8	7	0
ALBU_HUMAN	69 kDa		4	14	21	14	12	13
PGK1_HUMAN	45 kDa	TRUE	4	7	9	8	5	6
ANXA6_HUMAN	76 kDa	TRUE	4	12	9	12	10	7
1433B_HUMAN	28 kDa	TRUE	4	10	11	9	8	9
TPIS_HUMAN	31 kDa		4	4	8	5	6	5
ITB1_HUMAN	88 kDa		4	3	6	2	2	3
CLUS_HUMAN	52 kDa		4	12	5	4	4	3
E7EVA0_HUMAN	245 kDa	TRUE	4	4	5	4	3	4
AMPE_HUMAN	109 kDa		4	0	2	2	3	4
HNRPU_HUMAN	91 kDa		4	4	3	4	2	5
NUCL_HUMAN	77 kDa		4	5	4	7	3	3
TKT_HUMAN	69 kDa		4	5	6	5	4	5
IGHA1_HUMAN	38 kDa	TRUE	4	2	5	6	5	7
SAMP_HUMAN	25 kDa		4	3	3	8	3	3
PDIA1_HUMAN	57 kDa		4	6	4	6	6	4
SFPQ_HUMAN	76 kDa	TRUE	4	1	1	5	2	4
HNRPC_HUMAN	32 kDa		4	6	2	6	3	2
S10A6_HUMAN	10 kDa		4	4	3	1	2	4
ROA3_HUMAN	40 kDa	TRUE	4	4	1	2	2	4
MAGI2_HUMAN	157 kDa		4	1	3	2	1	3
ECHA_HUMAN	83 kDa		4	1	2	1	4	1
GRP75_HUMAN	74 kDa		4	1	2	6	4	4
NB5R3_HUMAN	32 kDa		4	0	2	2	3	0
INF2_HUMAN	136 kDa		4	2	5	1	2	2
DBNL_HUMAN	48 kDa	TRUE	4	3	3	4	4	3
TYB4_HUMAN	5 kDa	TRUE	4	7	6	6	4	3
RRBP1_HUMAN	152 kDa	TRUE	4	2	4	6	5	1
GBB2_HUMAN	37 kDa	TRUE	4	2	2	3	2	1

EHD4_HUMAN	61 kDa	TRUE	4	4	5	5	3	3
ECHB_HUMAN	51 kDa		4	3	1	1	2	3
E41L2_HUMAN	113 kDa		4	3	0	3	1	2
CSRP2_HUMAN	21 kDa		4	3	1	2	3	5
YBOX1_HUMAN	36 kDa	TRUE	4	2	3	7	5	1
LYSC_HUMAN	17 kDa		4	3	2	7	1	0
TIF1B_HUMAN	79 kDa		4	3	1	0	1	2
DHB4_HUMAN	80 kDa		4	3	0	3	3	1
LMNB1_HUMAN	66 kDa	TRUE	4	5	5	4	2	6
H3BLZ8_HUMAN	80 kDa	TRUE	4	2	2	0	1	3
CLCA_HUMAN	24 kDa		4	2	0	2	0	1
AKA12_HUMAN	182 kDa		4	2	0	2	2	0
PLSL_HUMAN	70 kDa	TRUE	4	2	0	5	1	0
K1C18_HUMAN	48 kDa	TRUE	4	1	0	0	0	0
K2C75_HUMAN	60 kDa	TRUE	4	0	0	0	0	0
IQGA1_HUMAN	189 kDa	TRUE	3	4	8	11	4	5
GSTP1_HUMAN	23 kDa		3	3	8	8	3	2
PLST_HUMAN	71 kDa	TRUE	3	4	6	2	7	4
RRAS_HUMAN	23 kDa	TRUE	3	4	4	3	4	5
LPP_HUMAN	66 kDa	TRUE	3	7	3	1	3	4
PARK7_HUMAN	20 kDa		3	5	2	4	2	4
PODXL_HUMAN	55 kDa		3	5	3	1	3	4
ITA3_HUMAN	117 kDa		3	2	3	3	2	4
CSRP1_HUMAN	21 kDa		3	5	5	6	5	3
PARVA_HUMAN	42 kDa		3	2	3	1	2	2
CH60_HUMAN	61 kDa		3	3	4	5	5	1
CRIP2_HUMAN	22 kDa		3	2	4	4	5	4
WDR1_HUMAN	66 kDa		3	4	2	6	3	2
RS3_HUMAN	27 kDa		3	3	3	4	4	2
RAB5C_HUMAN	27 kDa	TRUE	3	3	2	1	2	2
AMBP_HUMAN	39 kDa		3	6	3	4	1	4
PCBP1_HUMAN	37 kDa	TRUE	3	2	6	6	4	1
B4DGP8_HUMAN	72 kDa		3	1	0	3	2	1
CAZA1_HUMAN	33 kDa	TRUE	3	2	4	4	6	3
F8WCF6_HUMAN	21 kDa		3	3	3	5	3	3
A2A274_HUMAN	88 kDa		3	2	2	2	1	3
DHE3_HUMAN	61 kDa		3	2	2	3	0	1
AMPL_HUMAN	53 kDa		3	1	4	4	2	4
ARP2_HUMAN	45 kDa	TRUE	3	3	3	3	3	3
CBR1_HUMAN	30 kDa		3	5	4	4	3	3
TENS3_HUMAN	155 kDa	TRUE	3	1	0	2	5	4
FBLN1_HUMAN	77 kDa	TRUE	3	5	5	3	3	1
XRCC6_HUMAN	70 kDa		3	1	4	0	2	0
H0Y300_HUMAN	49 kDa	TRUE	3	0	4	9	2	1
MVP_HUMAN	99 kDa		3	1	1	5	0	3
J3KTF8_HUMAN	22 kDa		3	4	3	2	3	3
EVA1B_HUMAN	18 kDa		3	3	5	2	3	1

NPHN_HUMAN	135 kDa		3	0	2	1	4	2
MDHC_HUMAN	39 kDa		3	2	2	3	2	1
1433F_HUMAN	28 kDa	TRUE	3	6	6	10	4	7
RAB1A_HUMAN	23 kDa	TRUE	3	2	4	6	5	2
EF1D_HUMAN	71 kDa		3	1	2	2	2	0
VAMP2_HUMAN	13 kDa	TRUE	3	2	3	2	1	2
RPN1_HUMAN	69 kDa		3	2	3	5	2	1
DYSF_HUMAN	239 kDa		3	3	0	0	3	1
NONO_HUMAN	54 kDa	TRUE	3	0	2	3	5	1
RAC1_HUMAN	23 kDa		3	3	1	1	1	2
DDX3X_HUMAN	73 kDa	TRUE	3	2	2	2	2	2
AT2A2_HUMAN	110 kDa		3	2	3	2	2	1
RL13_HUMAN	24 kDa		3	3	2	1	2	1
1433T_HUMAN	28 kDa	TRUE	3	4	4	3	3	5
VDAC2_HUMAN	33 kDa	TRUE	3	2	0	1	1	1
GLU2B_HUMAN	59 kDa		3	2	2	2	3	1
H2AY_HUMAN	39 kDa	TRUE	3	3	2	0	2	1
CO5_HUMAN	188 kDa		3	4	1	2	2	1
KINH_HUMAN	110 kDa	TRUE	3	1	1	1	1	1
E9PEX6_HUMAN	52 kDa		3	1	2	0	1	1
AATM_HUMAN	48 kDa		3	1	0	0	0	2
HDGF_HUMAN	27 kDa		3	4	3	3	0	2
ESYT1_HUMAN	124 kDa		3	2	0	1	0	0
H15_HUMAN	23 kDa	TRUE	3	3	1	4	3	3
BGH3_HUMAN	75 kDa		3	4	0	1	2	0
H2AZ_HUMAN	14 kDa	TRUE	3	1	0	0	0	3
RPN2_HUMAN	68 kDa		3	1	0	1	0	0
PPM1F_HUMAN	50 kDa		3	1	1	1	2	0
SUN2_HUMAN	80 kDa		3	0	0	0	0	0
ENDD1_HUMAN	55 kDa		3	1	2	1	1	0
UROM_HUMAN	67 kDa		3	0	0	0	0	0
SFXN3_HUMAN	36 kDa		3	0	0	2	0	0
TSP1_HUMAN	129 kDa		3	0	0	1	0	0
NEP_HUMAN	86 kDa		2	4	8	6	8	9
J3KNM7_HUMAN	164 kDa		2	4	6	3	5	2
SEPT7_HUMAN	51 kDa	TRUE	2	5	3	4	3	1
LDHA_HUMAN	40 kDa	TRUE	2	4	7	5	2	1
TENA_HUMAN	241 kDa		2	9	3	4	5	0
F5GXS0_HUMAN	188 kDa	TRUE	2	6	5	5	6	3
GDIB_HUMAN	51 kDa	TRUE	2	3	3	3	4	2
EF2_HUMAN	95 kDa	TRUE	2	0	1	5	3	3
CATD_HUMAN	45 kDa		2	6	2	4	3	2
MYO1B_HUMAN	125 kDa		2	4	1	4	0	2
CO4A1_HUMAN	161 kDa		2	5	4	2	3	2
E9PK25_HUMAN	23 kDa	TRUE	2	5	2	3	2	2
NPM_HUMAN	33 kDa		2	5	1	3	2	1
CO4A3_HUMAN	158 kDa		2	1	4	1	3	0

ARF1_HUMAN	21 kDa	TRUE	2	3	3	3	1	1
RAP1A_HUMAN	21 kDa	TRUE	2	1	2	8	2	3
TINAL_HUMAN	49 kDa		2	4	3	1	2	1
GNAI2_HUMAN	42 kDa	TRUE	2	4	3	3	3	3
MYLK_HUMAN	203 kDa		2	1	1	4	2	3
ARP3_HUMAN	47 kDa		2	2	3	2	1	3
MDHM_HUMAN	36 kDa		2	4	2	1	1	1
SBP1_HUMAN	52 kDa		2	2	2	1	2	4
LEG1_HUMAN	15 kDa		2	3	1	0	2	1
RAB7A_HUMAN	23 kDa	TRUE	2	2	1	1	2	2
HNRPR_HUMAN	71 kDa	TRUE	2	3	3	6	1	1
ICAM1_HUMAN	58 kDa		2	1	2	1	4	2
VPS35_HUMAN	92 kDa		2	1	4	3	3	2
CAZA2_HUMAN	33 kDa	TRUE	2	1	2	3	2	5
ITAV_HUMAN	112 kDa		2	3	2	2	4	1
HNRPD_HUMAN	33 kDa	TRUE	2	4	1	2	2	3
PDIA6_HUMAN	54 kDa		2	1	3	1	1	1
IF4A1_HUMAN	46 kDa	TRUE	2	2	1	3	2	2
FBN1_HUMAN	312 kDa	TRUE	2	3	1	1	1	2
RHOA_HUMAN	22 kDa	TRUE	2	4	2	1	1	1
ALDOC_HUMAN	39 kDa	TRUE	2	1	6	1	2	2
PURA_HUMAN	35 kDa	TRUE	2	3	2	2	3	1
BLVRB_HUMAN	22 kDa		2	1	2	3	2	1
ACTZ_HUMAN	43 kDa	TRUE	2	1	1	2	2	2
GDIR2_HUMAN	23 kDa		2	2	1	2	2	1
NUMA1_HUMAN	237 kDa		2	0	4	3	1	2
RS18_HUMAN	18 kDa		2	2	2	1	1	1
HCD2_HUMAN	27 kDa		2	3	2	1	4	1
FRIL_HUMAN	20 kDa		2	1	1	0	1	0
TPPP3_HUMAN	19 kDa		2	1	2	1	2	2
ODO2_HUMAN	49 kDa		2	2	1	2	1	2
VP26A_HUMAN	38 kDa	TRUE	2	1	2	1	0	1
SQRD_HUMAN	50 kDa		2	1	0	1	2	1
CLIC1_HUMAN	27 kDa	TRUE	2	1	0	0	1	1
A8MXP9_HUMAN	100 kDa		2	2	1	0	0	1
VINEX_HUMAN	75 kDa		2	3	1	0	0	1
PROF2_HUMAN	15 kDa		2	1	1	1	1	1
VAT1_HUMAN	42 kDa		2	0	1	2	2	2
GBLP_HUMAN	35 kDa		2	2	1	1	2	0
ECHM_HUMAN	31 kDa		2	0	0	1	1	1
ILF2_HUMAN	43 kDa		2	2	4	2	0	0
NACAM_HUMAN	205 kDa		2	1	2	1	2	1
VASP_HUMAN	40 kDa		2	2	1	1	2	0
FHL2_HUMAN	32 kDa		2	1	0	1	0	2
DCD_HUMAN	11 kDa		2	1	3	2	2	1
FKB1A_HUMAN	12 kDa		2	1	3	3	2	2
RS28_HUMAN	8 kDa		2	1	1	2	1	2

CYFP1_HUMAN	145 kDa	TRUE	2	1	1	2	0	0
RS14_HUMAN	16 kDa		2	2	1	2	1	1
PTMA_HUMAN	12 kDa		2	0	3	6	2	0
KTN1_HUMAN	156 kDa	TRUE	2	0	3	1	2	1
ACADV_HUMAN	68 kDa		2	0	2	1	0	0
SC22B_HUMAN	25 kDa		2	1	2	2	2	1
ATPO_HUMAN	23 kDa		2	1	1	1	0	1
HNRPL_HUMAN	64 kDa		2	0	0	1	0	0
NDRG1_HUMAN	43 kDa		2	1	2	1	2	0
NDKB_HUMAN	30 kDa		2	0	1	2	1	1
PDIA4_HUMAN	73 kDa		2	1	1	3	1	1
RL22_HUMAN	15 kDa		2	2	1	1	1	2
RS16_HUMAN	16 kDa		2	1	0	1	1	1
LAP2B_HUMAN	51 kDa		2	0	4	3	3	2
J3QL05_HUMAN	15 kDa		2	1	1	2	1	0
ADDA_HUMAN	70 kDa		2	1	0	0	1	1
KANK2_HUMAN	90 kDa		2	1	0	0	0	2
SYDC_HUMAN	57 kDa		2	1	1	1	2	0
RS9_HUMAN	23 kDa		2	2	0	0	1	1
RS10_HUMAN	19 kDa		2	0	0	0	0	1
MK01_HUMAN	41 kDa	TRUE	2	1	0	1	0	0
QOR_HUMAN	35 kDa		2	0	0	1	0	1
MYOF_HUMAN	233 kDa		2	4	0	2	0	0
IF4H_HUMAN	25 kDa		2	1	1	0	1	1
CFAH_HUMAN	139 kDa	TRUE	2	2	2	2	3	2
MYPT1_HUMAN	115 kDa	TRUE	2	1	2	1	2	1
HXK1_HUMAN	103 kDa		2	2	0	1	1	0
FABP4_HUMAN	15 kDa		2	3	1	4	1	1
VWA1_HUMAN	47 kDa		2	2	1	0	1	1
ECH1_HUMAN	36 kDa		2	1	0	0	0	1
GNAS1_HUMAN	110 kDa	TRUE	2	2	2	2	2	3
LAMP1_HUMAN	45 kDa		2	1	0	0	0	1
VDAC1_HUMAN	31 kDa	TRUE	2	1	0	0	1	1
ARC1A_HUMAN	42 kDa		2	0	2	0	1	1
PP1B_HUMAN	37 kDa	TRUE	2	0	0	1	1	1
TPP1_HUMAN	61 kDa		2	2	1	2	1	2
RL12_HUMAN	18 kDa		2	2	0	1	0	0
ADDG_HUMAN	76 kDa	TRUE	2	0	2	2	1	2
H3BRU6_HUMAN	32 kDa	TRUE	2	3	3	0	0	2
LIMA1_HUMAN	67 kDa		2	1	2	0	0	2
URP2_HUMAN	75 kDa		2	2	0	2	0	0
SNX1_HUMAN	52 kDa	TRUE	2	2	2	1	1	1
COR1A_HUMAN	51 kDa		2	2	1	2	0	0
RTCB_HUMAN	55 kDa		2	1	0	0	0	1
B4DFL2_HUMAN	45 kDa	TRUE	2	0	0	0	0	0
FUMH_HUMAN	55 kDa		2	0	0	0	0	0
EGLN_HUMAN	68 kDa		2	1	1	0	2	0

NUCB1_HUMAN	54 kDa		2	0	1	1	1	0
FBLI1_HUMAN	40 kDa		2	2	0	1	2	1
IDH3A_HUMAN	40 kDa		2	0	0	0	1	0
FERM2_HUMAN	79 kDa		1	2	4	5	5	2
PRDX6_HUMAN	25 kDa	TRUE	1	3	2	3	5	1
LDHB_HUMAN	37 kDa	TRUE	1	5	3	5	1	1
PRDX2_HUMAN	22 kDa	TRUE	1	5	9	3	8	5
PEBP1_HUMAN	21 kDa		1	4	2	4	2	2
KCRB_HUMAN	43 kDa		1	0	1	2	3	4
S10A9_HUMAN	13 kDa		1	6	4	8	1	0
CNDP2_HUMAN	53 kDa		1	2	2	3	2	1
A1AT_HUMAN	47 kDa		1	2	3	3	1	1
SEP11_HUMAN	49 kDa	TRUE	1	2	4	1	1	3
RSU1_HUMAN	32 kDa		1	3	0	0	1	2
CATA_HUMAN	60 kDa		1	2	3	7	3	1
H10_HUMAN	21 kDa	TRUE	1	3	4	1	3	3
RTN4_HUMAN	130 kDa		1	1	1	4	3	0
ITA1_HUMAN	131 kDa		1	2	0	0	0	2
LG3BP_HUMAN	65 kDa		1	4	0	1	0	1
TGFI1_HUMAN	48 kDa		1	1	2	0	2	1
UTRO_HUMAN	395 kDa	TRUE	1	0	4	0	1	6
KIRR1_HUMAN	85 kDa		1	1	1	2	4	1
TMOD3_HUMAN	40 kDa	TRUE	1	4	3	2	3	2
HP1B3_HUMAN	61 kDa		1	2	1	1	1	2
PHB2_HUMAN	33 kDa	TRUE	1	1	2	1	0	2
SND1_HUMAN	102 kDa		1	3	1	5	2	2
THIO_HUMAN	12 kDa		1	2	3	1	2	2
CDC42_HUMAN	21 kDa		1	2	5	2	1	1
GDIA_HUMAN	51 kDa	TRUE	1	2	2	2	3	1
SYWC_HUMAN	53 kDa		1	2	1	2	2	2
B4E220_HUMAN	36 kDa		1	2	2	2	4	2
PDC6I_HUMAN	97 kDa		1	2	2	1	2	2
B2MG_HUMAN	14 kDa		1	1	0	0	0	2
SRBS2_HUMAN	135 kDa		1	3	1	1	2	1
AL1A1_HUMAN	55 kDa	TRUE	1	0	1	1	0	2
PHB_HUMAN	30 kDa		1	2	2	2	4	2
MMRN2_HUMAN	104 kDa		1	2	1	1	3	1
PRDX5_HUMAN	22 kDa	TRUE	1	2	1	1	1	1
NPNT_HUMAN	65 kDa		1	0	0	1	0	0
SRSF7_HUMAN	26 kDa	TRUE	1	1	2	2	2	1
ALDH2_HUMAN	56 kDa	TRUE	1	1	1	1	1	0
DEND_HUMAN	76 kDa		1	0	0	0	3	2
PDLI7_HUMAN	50 kDa		1	2	2	0	2	3
H7BY58_HUMAN	30 kDa		1	0	0	0	0	2
CLIC4_HUMAN	29 kDa	TRUE	1	0	0	0	1	1
TALDO_HUMAN	38 kDa		1	1	2	2	1	1
FRIH_HUMAN	21 kDa		1	3	1	2	0	2

AACT_HUMAN	48 kDa		1	3	4	4	1	0
FSCN1_HUMAN	55 kDa		1	0	0	0	0	0
RB11A_HUMAN	24 kDa		1	2	1	1	1	1
B7Z1R5_HUMAN	65 kDa		1	2	1	2	1	1
ARPC3_HUMAN	21 kDa		1	1	1	1	1	0
CTND1_HUMAN	105 kDa		1	0	0	1	2	2
K7EJT8_HUMAN	101 kDa		1	1	1	1	2	0
THIL_HUMAN	45 kDa		1	1	1	2	1	0
DX39B_HUMAN	51 kDa		1	1	0	2	1	1
RS4X_HUMAN	30 kDa		1	1	1	1	1	0
NID2_HUMAN	141 kDa	TRUE	1	6	2	1	1	0
TCPZ_HUMAN	58 kDa		1	2	1	1	2	1
EFTU_HUMAN	50 kDa		1	1	0	3	0	1
COX2_HUMAN	26 kDa		1	1	1	1	1	1
GBG12_HUMAN	8 kDa		1	1	1	0	1	2
MYO1E_HUMAN	127 kDa	TRUE	1	0	0	1	0	0
RINI_HUMAN	50 kDa		1	1	2	1	2	2
DRA_HUMAN	29 kDa		1	1	0	2	0	3
PA2G4_HUMAN	44 kDa		1	0	1	1	0	0
2AAA_HUMAN	65 kDa		1	0	1	0	0	1
PAWR_HUMAN	37 kDa		1	0	2	1	4	1
MIF_HUMAN	12 kDa		1	1	2	2	0	1
RS7_HUMAN	22 kDa		1	0	1	1	2	0
SRSF1_HUMAN	28 kDa		1	3	3	0	0	1
PPIB_HUMAN	24 kDa	TRUE	1	1	2	2	3	1
AL9A1_HUMAN	54 kDa		1	1	0	1	1	1
ABHEB_HUMAN	22 kDa		1	2	5	1	1	1
CR1_HUMAN	224 kDa		1	1	1	0	0	1
AP2A1_HUMAN	105 kDa	TRUE	1	0	0	1	2	0
EF1G_HUMAN	50 kDa		1	1	1	0	1	1
SERPH_HUMAN	46 kDa		1	1	1	1	2	1
ILEU_HUMAN	43 kDa	TRUE	1	1	1	0	0	0
B4DT31_HUMAN	70 kDa	TRUE	1	0	2	1	3	0
PGM2_HUMAN	68 kDa		1	3	0	0	0	1
SYNP2_HUMAN	119 kDa		1	3	2	0	0	0
NRIP2_HUMAN	30 kDa		1	2	1	1	1	1
CISY_HUMAN	52 kDa		1	1	1	1	1	1
MYO1D_HUMAN	116 kDa		1	1	2	1	1	2
KANK3_HUMAN	86 kDa		1	0	2	2	1	2
MECP2_HUMAN	53 kDa		1	1	1	0	2	1
TB182_HUMAN	182 kDa		1	0	1	5	1	1
CYTB_HUMAN	11 kDa		1	2	1	0	0	0
PTPRO_HUMAN	135 kDa		1	1	0	0	0	1
SH3L3_HUMAN	10 kDa		1	2	0	2	1	1
FUS_HUMAN	53 kDa	TRUE	1	2	1	1	1	0
CO4A5_HUMAN	162 kDa		1	1	1	1	1	0
MPP5_HUMAN	77 kDa		1	0	1	2	0	0

DC1I2_HUMAN	71 kDa		1	0	2	1	0	1
STX7_HUMAN	27 kDa		1	1	0	1	1	1
CAD13_HUMAN	83 kDa		1	1	1	1	1	2
G3XAM7_HUMAN	93 kDa	TRUE	1	1	1	2	2	0
QCR2_HUMAN	48 kDa		1	0	0	1	0	0
MPCP_HUMAN	40 kDa		1	1	1	1	1	1
RS26_HUMAN	13 kDa		1	2	1	1	1	0
PICAL_HUMAN	69 kDa		1	0	0	0	2	1
DREB_HUMAN	76 kDa		1	1	1	1	2	1
H1X_HUMAN	22 kDa		1	3	3	0	3	2
CTL2_HUMAN	80 kDa		1	0	0	0	0	1
EFNB1_HUMAN	38 kDa	TRUE	1	0	0	1	0	1
PRAF3_HUMAN	22 kDa		1	1	1	1	0	0
CBX3_HUMAN	21 kDa		1	1	2	0	0	0
ATPD_HUMAN	17 kDa		1	1	1	1	2	2
TCP4_HUMAN	14 kDa		1	0	1	1	2	1
EWS_HUMAN	68 kDa		1	0	1	2	1	0
GNA11_HUMAN	42 kDa	TRUE	1	0	0	1	1	1
S10AA_HUMAN	11 kDa		1	2	0	2	1	0
TCPD_HUMAN	58 kDa		1	0	2	1	1	0
TYPH_HUMAN	50 kDa		1	4	1	5	0	1
AHNK2_HUMAN	606 kDa		1	0	0	0	0	0
UBE2N_HUMAN	17 kDa		1	1	1	0	2	1
PGRC2_HUMAN	26 kDa	TRUE	1	0	1	1	0	0
CDC37_HUMAN	44 kDa		1	1	0	0	1	0
RL4_HUMAN	48 kDa		1	0	0	1	3	0
SPB9_HUMAN	42 kDa	TRUE	1	0	0	0	0	1
PSB4_HUMAN	29 kDa		1	0	0	0	0	1
PRDX3_HUMAN	26 kDa		1	1	0	0	1	1
PAIRB_HUMAN	44 kDa		1	1	1	2	1	0
DC1L2_HUMAN	54 kDa		1	0	1	3	2	1
HSP74_HUMAN	94 kDa		1	1	0	0	1	0
SC23A_HUMAN	86 kDa		1	0	1	2	1	1
EMD_HUMAN	29 kDa		1	0	0	1	1	1
RL7_HUMAN	29 kDa		1	1	1	0	1	0
RL6_HUMAN	33 kDa		1	0	0	1	0	0
AP2M1_HUMAN	49 kDa		1	1	0	1	1	0
TM109_HUMAN	26 kDa		1	1	1	1	1	1
TPD54_HUMAN	23 kDa		1	1	1	0	0	0
CYB5_HUMAN	11 kDa		1	0	0	1	1	0
CRUM2_HUMAN	100 kDa		1	0	1	1	1	0
KAP0_HUMAN	43 kDa	TRUE	1	3	1	2	1	1
RS3A_HUMAN	30 kDa		1	0	0	0	1	0
ARC1B_HUMAN	41 kDa		1	0	1	2	1	1
TBCB_HUMAN	27 kDa		1	1	0	2	1	1
H0YK49_HUMAN	24 kDa		1	0	1	2	1	1
RALA_HUMAN	24 kDa	TRUE	1	0	0	0	0	0

CHTOP_HUMAN	27 kDa		1	0	1	1	1	1
H7BY10_HUMAN	18 kDa		1	1	1	1	1	1
RL18_HUMAN	22 kDa		1	2	1	0	1	0
TCPH_HUMAN	50 kDa		1	0	0	0	0	0
CPT1A_HUMAN	86 kDa		1	0	2	0	2	2
PECA1_HUMAN	81 kDa		1	2	2	0	3	2
ITIH5_HUMAN	105 kDa		1	0	0	0	0	1
PLIN3_HUMAN	47 kDa		1	1	2	1	0	0
FUBP2_HUMAN	73 kDa	TRUE	1	1	1	2	1	2
PERM_HUMAN	74 kDa		1	3	1	3	1	0
LEMD2_HUMAN	57 kDa		1	0	0	0	1	1
K6PL_HUMAN	85 kDa	TRUE	1	0	0	1	0	0
FIS1_HUMAN	17 kDa		1	1	1	1	1	1
VAMP5_HUMAN	13 kDa		1	1	0	2	0	1
RS21_HUMAN	9 kDa		1	0	1	2	0	1
B4DL14_HUMAN	28 kDa	TRUE	1	1	0	0	0	1
LYRIC_HUMAN	64 kDa		1	1	1	1	1	1
TMEDA_HUMAN	25 kDa		1	1	0	1	0	0
ILF3_HUMAN	76 kDa	TRUE	1	1	0	1	1	0
SSRD_HUMAN	19 kDa		1	1	1	2	0	0
KAD3_HUMAN	26 kDa		1	1	0	0	0	0
AL7A1_HUMAN	55 kDa		1	0	0	0	0	0
LAMA2_HUMAN	344 kDa		1	1	0	0	1	1
PSMD2_HUMAN	100 kDa		1	0	0	0	0	0
CO8G_HUMAN	22 kDa		1	5	0	0	1	1
AL4A1_HUMAN	62 kDa		1	0	0	0	1	0
ARHGH_HUMAN	222 kDa		1	0	1	1	0	0
RS8_HUMAN	24 kDa		1	1	1	0	1	1
RL7A_HUMAN	30 kDa		1	0	1	0	0	0
ROAA_HUMAN	31 kDa		1	0	1	2	0	0
AIFM1_HUMAN	67 kDa		1	0	0	0	0	0
SSBP_HUMAN	17 kDa		1	1	0	1	1	1
KCD12_HUMAN	36 kDa		1	1	0	1	0	0
SYEP_HUMAN	171 kDa		1	0	1	1	1	0
RL8_HUMAN	28 kDa		1	0	1	0	0	1
B7Z6B8_HUMAN	35 kDa		1	0	0	1	0	0
TTHY_HUMAN	16 kDa		1	0	1	0	0	0
IF16_HUMAN	88 kDa		1	0	1	1	2	0
H0YHG0_HUMAN	59 kDa		1	0	2	1	1	1
2B17_HUMAN	30 kDa	TRUE	1	1	0	3	1	0
RBM3_HUMAN	17 kDa		1	0	0	1	0	0
LIS1_HUMAN	47 kDa		1	0	2	1	2	0
PGFRB_HUMAN	124 kDa		1	1	0	0	1	1
LPP3_HUMAN	35 kDa		1	0	0	0	0	0
CHCH3_HUMAN	26 kDa		1	0	0	1	0	0
QCR1_HUMAN	53 kDa		1	1	0	1	0	0
B9A067_HUMAN	79 kDa		1	1	0	0	0	0

EIF3A_HUMAN	167 kDa		1	0	0	1	1	0
AKAP2_HUMAN	121 kDa	TRUE	1	1	0	0	0	0
FHL3_HUMAN	31 kDa		1	0	0	0	1	1
KV201_HUMAN	13 kDa		1	1	1	1	1	0
UBP14_HUMAN	52 kDa		1	0	1	0	1	0
S10A4_HUMAN	12 kDa		1	0	0	1	0	0
CYC_HUMAN	12 kDa		1	0	1	0	1	0
NEUG_HUMAN	8 kDa		1	1	0	1	0	1
DHX15_HUMAN	91 kDa		1	1	0	2	0	0
SNX2_HUMAN	58 kDa	TRUE	1	1	1	0	0	0
ETFB_HUMAN	38 kDa		1	1	0	0	1	0
CXA5_HUMAN	40 kDa		1	0	0	0	0	0
PML_HUMAN	98 kDa		1	0	0	0	0	0
TRIPB_HUMAN	228 kDa		1	0	0	0	0	0
ZDHC8_HUMAN	83 kDa		1	0	1	0	1	0
CATZ_HUMAN	34 kDa		1	0	0	0	0	1
ALDR_HUMAN	36 kDa		1	0	0	0	0	0
RL31_HUMAN	14 kDa		1	0	0	1	0	0
CATG_HUMAN	29 kDa		1	2	0	1	0	0
GOLI4_HUMAN	82 kDa		1	0	0	0	0	0
MUC18_HUMAN	72 kDa		1	0	1	2	0	0
MNDA_HUMAN	46 kDa		1	1	0	4	0	0
YAP1_HUMAN	49 kDa		1	0	0	0	0	0
DESP_HUMAN	332 kDa		0	1	6	1	2	0
ALDOB_HUMAN	39 kDa	TRUE	0	0	1	2	0	1
F5GWP8_HUMAN	66 kDa	TRUE	0	7	16	14	10	0
CPNS1_HUMAN	28 kDa		0	2	1	1	1	2
ACY1_HUMAN	46 kDa		0	0	0	0	0	2
TITIN_HUMAN	3994 kDa	TRUE	0	0	0	0	1	0
PGAM1_HUMAN	29 kDa		0	3	3	3	2	0
SPB4_HUMAN	45 kDa	TRUE	0	0	0	0	0	0
ASSY_HUMAN	47 kDa		0	0	1	0	0	0
A2MG_HUMAN	163 kDa		0	4	6	5	1	1
DSG1_HUMAN	114 kDa		0	0	3	5	3	0
IGHM_HUMAN	52 kDa		0	2	6	3	3	2
K2C3_HUMAN	64 kDa	TRUE	0	0	21	0	0	0
TCPB_HUMAN	57 kDa		0	3	3	2	4	0
GANAB_HUMAN	107 kDa		0	1	0	3	2	3
G6PI_HUMAN	64 kDa	TRUE	0	0	1	1	1	0
AK1A1_HUMAN	37 kDa		0	1	1	2	0	1
CAH2_HUMAN	29 kDa		0	0	0	0	1	1
CRYAB_HUMAN	20 kDa		0	0	0	1	0	1
6PGD_HUMAN	53 kDa		0	1	1	3	2	0
ARPC2_HUMAN	34 kDa		0	2	0	1	4	2
XRCC5_HUMAN	83 kDa		0	1	1	0	0	0
TRFE_HUMAN	77 kDa		0	2	0	1	1	3
HORN_HUMAN	282 kDa		0	0	11	6	2	0

B4DVY7_HUMAN	42 kDa		0	2	2	2	2	2
HS12A_HUMAN	75 kDa		0	1	2	3	3	1
SPB6_HUMAN	43 kDa		0	1	2	2	2	3
CAN2_HUMAN	80 kDa		0	0	0	1	0	0
CLIC5_HUMAN	47 kDa	TRUE	0	0	3	1	0	1
PTBP1_HUMAN	59 kDa	TRUE	0	1	2	1	1	0
CYTA_HUMAN	11 kDa		0	0	2	1	2	0
1433S_HUMAN	24 kDa	TRUE	0	0	0	0	0	0
S10AB_HUMAN	12 kDa		0	3	3	1	0	0
ESTD_HUMAN	31 kDa		0	2	3	1	0	1
RSSA_HUMAN	33 kDa		0	1	0	0	2	0
COR1B_HUMAN	54 kDa	TRUE	0	3	1	4	1	2
ARF6_HUMAN	20 kDa	TRUE	0	0	0	0	0	1
APOA1_HUMAN	31 kDa		0	1	0	0	0	0
F16P1_HUMAN	37 kDa	TRUE	0	0	0	1	0	0
PLAK_HUMAN	82 kDa	TRUE	0	0	3	0	0	0
TGM3_HUMAN	77 kDa		0	0	1	1	0	0
RLA2_HUMAN	12 kDa		0	0	0	1	1	0
CAH1_HUMAN	29 kDa		0	0	1	3	4	1
IGHG2_HUMAN	36 kDa	TRUE	0	5	4	7	0	4
PTGR1_HUMAN	33 kDa		0	2	1	1	0	0
CO1A2_HUMAN	129 kDa		0	1	0	0	2	0
ACOC_HUMAN	98 kDa		0	0	0	0	0	0
MACF1_HUMAN	838 kDa	TRUE	0	0	1	1	0	0
RS2_HUMAN	31 kDa		0	1	0	0	1	0
AIF1_HUMAN	17 kDa	TRUE	0	1	0	0	1	1
ADT3_HUMAN	33 kDa	TRUE	0	1	0	0	0	1
HEBP1_HUMAN	21 kDa		0	1	2	1	0	1
PLCG2_HUMAN	148 kDa		0	0	0	0	0	0
KAP2_HUMAN	46 kDa	TRUE	0	2	1	0	0	1
K1C13_HUMAN	50 kDa	TRUE	0	0	19	0	0	0
FILA2_HUMAN	248 kDa		0	0	3	4	0	0
ODPB_HUMAN	37 kDa		0	1	3	3	2	1
SAHH_HUMAN	48 kDa		0	0	0	1	0	1
SDHA_HUMAN	73 kDa		0	0	0	2	1	0
HMGB1_HUMAN	25 kDa	TRUE	0	1	0	0	1	0
GSTA2_HUMAN	26 kDa	TRUE	0	0	0	0	0	0
CK054_HUMAN	35 kDa		0	1	1	1	1	2
CALL5_HUMAN	16 kDa		0	0	0	0	0	0
SRBS1_HUMAN	112 kDa		0	1	1	2	1	1
CO1A1_HUMAN	139 kDa		0	1	1	0	0	0
PCYOX_HUMAN	57 kDa		0	1	1	0	3	0
LAC2_HUMAN	11 kDa	TRUE	0	1	4	4	4	1
SH3L1_HUMAN	13 kDa		0	4	2	2	1	0
ANX11_HUMAN	54 kDa	TRUE	0	0	1	1	1	1
RBMX_HUMAN	42 kDa	TRUE	0	2	1	1	0	0
DDAH2_HUMAN	30 kDa	TRUE	0	1	0	1	1	0

PSB1_HUMAN	26 kDa		0	1	1	0	1	1
BHMT1_HUMAN	45 kDa	TRUE	0	0	0	0	0	1
PSA5_HUMAN	26 kDa		0	1	1	3	1	1
PRAX_HUMAN	155 kDa		0	1	1	2	0	1
TCPQ_HUMAN	60 kDa		0	0	2	1	3	0
ICAL_HUMAN	81 kDa		0	0	0	1	0	0
NQO1_HUMAN	26 kDa		0	0	0	1	1	0
K2C4_HUMAN	57 kDa	TRUE	0	0	16	0	0	1
TAU_HUMAN	79 kDa		0	1	4	2	2	1
E9PCA1_HUMAN	57 kDa		0	1	0	1	0	1
IDHC_HUMAN	47 kDa	TRUE	0	0	1	1	0	0
G3V325_HUMAN	84 kDa		0	1	1	0	0	0
MMSA_HUMAN	58 kDa		0	0	0	0	0	0
FILA_HUMAN	435 kDa		0	0	0	0	0	0
STAT1_HUMAN	87 kDa		0	0	0	0	1	0
IF5A1_HUMAN	20 kDa		0	0	0	0	0	0
S10A7_HUMAN	11 kDa	TRUE	0	0	0	0	0	0
F5H039_HUMAN	85 kDa		0	0	0	0	2	1
ITB3_HUMAN	87 kDa		0	0	0	2	3	0
ADIRF_HUMAN	8 kDa		0	1	3	1	0	0
RHG01_HUMAN	50 kDa		0	2	0	1	1	1
MAP1B_HUMAN	271 kDa	TRUE	0	0	1	2	0	0
SYNE2_HUMAN	799 kDa	TRUE	0	0	1	0	0	0
PEPL_HUMAN	205 kDa		0	0	0	0	0	0
CTNB1_HUMAN	85 kDa		0	0	1	1	3	0
RL3_HUMAN	46 kDa		0	1	1	1	0	0
CAND1_HUMAN	136 kDa		0	1	1	1	0	0
RS17L_HUMAN	16 kDa		0	0	2	1	2	0
TPD52_HUMAN	26 kDa		0	1	1	2	0	0
E41L5_HUMAN	82 kDa	TRUE	0	0	0	0	0	0
KRT85_HUMAN	56 kDa	TRUE	0	0	0	0	0	0
CAN1_HUMAN	82 kDa		0	1	0	0	0	1
TM1L2_HUMAN	50 kDa		0	0	1	0	0	0
PUR9_HUMAN	65 kDa		0	1	0	0	0	1
PKP1_HUMAN	83 kDa		0	0	0	0	0	0
SET_HUMAN	32 kDa		0	0	0	1	0	0
HNRPQ_HUMAN	63 kDa	TRUE	0	2	1	2	0	0
TBA4A_HUMAN	50 kDa	TRUE	0	15	17	16	11	0
PAR3L_HUMAN	126 kDa		0	0	0	0	1	1
6PGL_HUMAN	28 kDa		0	2	3	0	2	0
RLA0_HUMAN	34 kDa		0	2	1	2	0	2
DHX9_HUMAN	141 kDa		0	1	0	1	0	0
EHD1_HUMAN	61 kDa	TRUE	0	0	1	0	0	0
DDC_HUMAN	54 kDa		0	0	0	0	0	0
LEG7_HUMAN	15 kDa		0	0	0	0	0	0
CRNN_HUMAN	54 kDa		0	0	1	0	0	0
LKHA4_HUMAN	60 kDa		0	1	1	1	1	0

UBP5_HUMAN	93 kDa		0	0	0	0	0	1
FABPL_HUMAN	14 kDa	TRUE	0	0	0	0	0	1
HMGN2_HUMAN	9 kDa		0	1	3	2	1	0
PSA1_HUMAN	30 kDa		0	0	0	0	1	0
RAB6A_HUMAN	24 kDa		0	0	0	0	2	0
S10A8_HUMAN	11 kDa		0	0	0	0	0	0
FGF1_HUMAN	17 kDa		0	1	0	1	2	1
ANXA7_HUMAN	50 kDa		0	1	1	2	1	2
RS19_HUMAN	16 kDa		0	0	0	1	0	0
TCPA_HUMAN	60 kDa		0	2	1	2	1	0
SMD3_HUMAN	14 kDa		0	0	1	1	0	2
CIRBP_HUMAN	19 kDa		0	0	1	1	2	0
MRP_HUMAN	20 kDa		0	1	1	2	1	0
H2A1C_HUMAN	14 kDa	TRUE	0	10	19	22	10	8
H2A2A_HUMAN	14 kDa	TRUE	0	0	0	0	0	0
DAG1_HUMAN	97 kDa		0	0	0	0	0	0
MYH11_HUMAN	228 kDa	TRUE	0	12	13	17	13	8
DHAK_HUMAN	59 kDa		0	0	0	0	0	0
SPB3_HUMAN	45 kDa	TRUE	0	0	0	0	0	0
SRSF3_HUMAN	19 kDa	TRUE	0	2	2	3	2	2
AT2B4_HUMAN	134 kDa	TRUE	0	1	1	1	0	1
K2C74_HUMAN	58 kDa	TRUE	0	0	11	0	1	0
RD23B_HUMAN	43 kDa		0	1	1	0	1	2
IGLL5_HUMAN	23 kDa	TRUE	0	1	2	3	1	1
DCTN2_HUMAN	45 kDa		0	1	0	0	0	1
ADHX_HUMAN	40 kDa		0	0	1	1	1	0
MARE1_HUMAN	30 kDa		0	0	1	0	0	1
GRHPR_HUMAN	36 kDa		0	0	0	0	0	1
CALB1_HUMAN	30 kDa		0	0	0	0	0	0
PTMS_HUMAN	12 kDa		0	1	2	3	1	0
CGNL1_HUMAN	149 kDa	TRUE	0	0	1	2	0	1
CH10_HUMAN	11 kDa		0	1	0	1	1	0
PSME1_HUMAN	29 kDa		0	0	0	0	0	1
SHRM3_HUMAN	217 kDa		0	0	0	0	1	0
SLK_HUMAN	139 kDa	TRUE	0	0	1	0	0	0
K2C78_HUMAN	57 kDa	TRUE	0	0	1	1	1	0
K1C17_HUMAN	48 kDa	TRUE	0	0	0	17	12	0
MX1_HUMAN	76 kDa		0	3	1	0	2	0
EPIPL_HUMAN	556 kDa	TRUE	0	0	1	1	0	2
SO2A1_HUMAN	70 kDa		0	0	2	1	2	0
HNRL2_HUMAN	85 kDa		0	3	1	2	2	1
PYGB_HUMAN	97 kDa	TRUE	0	1	0	1	1	0
DHPR_HUMAN	26 kDa		0	0	0	0	0	0
CALL3_HUMAN	17 kDa	TRUE	0	0	0	0	0	0
EVPL_HUMAN	232 kDa	TRUE	0	0	0	0	0	0
APOL2_HUMAN	37 kDa		0	0	1	1	1	1
MYO6_HUMAN	149 kDa		0	0	1	1	1	0

DCTN1_HUMAN	127 kDa		0	0	0	1	0	0
COTL1_HUMAN	16 kDa		0	1	0	1	0	1
MAP6_HUMAN	87 kDa		0	1	0	0	0	0
COPA_HUMAN	139 kDa		0	1	0	0	1	0
NFASC_HUMAN	138 kDa		0	1	0	0	0	0
H2AX_HUMAN	15 kDa	TRUE	0	0	0	0	0	0
MYH14_HUMAN	232 kDa	TRUE	0	0	0	0	0	0
AIF1L_HUMAN	20 kDa	TRUE	0	0	0	0	0	0
GATM_HUMAN	45 kDa		0	0	0	0	0	0
TRXR1_HUMAN	66 kDa		0	0	0	0	0	0
LA_HUMAN	47 kDa		0	0	1	0	1	1
RS20_HUMAN	16 kDa		0	1	1	0	0	0
RS6_HUMAN	29 kDa		0	1	0	0	1	0
RAN_HUMAN	24 kDa		0	1	0	0	0	0
SH3L2_HUMAN	12 kDa		0	0	2	1	0	1
ZA2G_HUMAN	34 kDa		0	0	1	1	1	0
PNPH_HUMAN	32 kDa		0	0	0	0	0	0
ACBP_HUMAN	16 kDa		0	0	0	0	0	0
B4DUR8_HUMAN	56 kDa		0	0	0	1	0	1
E7ERJ7_HUMAN	67 kDa	TRUE	0	0	0	1	0	0
CASPE_HUMAN	28 kDa		0	0	0	0	0	0
RAB2A_HUMAN	21 kDa		0	0	0	0	0	0
PEA15_HUMAN	15 kDa		0	1	0	1	0	0
PSA6_HUMAN	27 kDa		0	0	2	1	0	0
PSA2_HUMAN	26 kDa		0	0	0	0	0	0
FKBP4_HUMAN	52 kDa		0	0	0	1	0	0
KV302_HUMAN	12 kDa		0	3	1	0	1	1
FA9_HUMAN	48 kDa		0	2	0	0	0	0
HNRPF_HUMAN	46 kDa	TRUE	0	0	0	5	0	0
SRP68_HUMAN	66 kDa		0	0	0	0	0	0
H7BXI1_HUMAN	98 kDa		0	0	2	1	1	0
2B1F_HUMAN	30 kDa	TRUE	0	1	1	0	1	1
F5H4X1_HUMAN	106 kDa		0	0	1	2	1	0
IMB1_HUMAN	97 kDa		0	0	1	0	0	0
SODC_HUMAN	16 kDa		0	0	0	2	1	0
K2C6C_HUMAN	60 kDa	TRUE	0	11	23	0	13	0
B3AT_HUMAN	102 kDa		0	1	0	0	0	1
AMPN_HUMAN	110 kDa		0	0	0	0	0	0
TARA_HUMAN	261 kDa		0	0	1	0	2	0
IGHG4_HUMAN	36 kDa	TRUE	0	0	0	6	0	2
NQO2_HUMAN	26 kDa		0	0	0	0	0	0
K2C1B_HUMAN	62 kDa	TRUE	0	0	0	3	2	3
NP1L4_HUMAN	44 kDa		0	0	1	2	0	0
PSME2_HUMAN	27 kDa	TRUE	0	0	0	0	0	0
ARL8B_HUMAN	22 kDa		0	0	0	0	1	0
VATB2_HUMAN	57 kDa	TRUE	0	0	0	1	1	0
IF4A2_HUMAN	46 kDa	TRUE	0	0	0	0	0	0

KAD1_HUMAN	22 kDa		0	0	0	0	0	0
INO1_HUMAN	47 kDa		0	0	0	0	0	2
K1H1_HUMAN	47 kDa	TRUE	0	0	0	0	0	0
FHL1_HUMAN	32 kDa		0	2	1	1	1	0
HSP72_HUMAN	70 kDa	TRUE	0	0	0	11	0	0
CRK_HUMAN	34 kDa		0	0	1	0	1	1
RAB10_HUMAN	23 kDa	TRUE	0	0	0	0	0	0
SDPR_HUMAN	47 kDa		0	0	0	0	1	0
COPG1_HUMAN	98 kDa		0	0	0	1	0	0
SCRN1_HUMAN	49 kDa		0	0	1	0	1	1
AP2A2_HUMAN	104 kDa	TRUE	0	0	0	0	0	0
NSF1C_HUMAN	41 kDa		0	1	3	2	0	0
G3XAD8_HUMAN	68 kDa		0	0	0	1	1	0
RAI14_HUMAN	110 kDa		0	0	0	0	0	0
M0QWZ9_HUMAN	185 kDa		0	0	0	1	0	0
AATC_HUMAN	46 kDa		0	0	0	0	0	0
M0QYS1_HUMAN	24 kDa		0	0	0	0	1	0
CRYM_HUMAN	34 kDa		0	0	0	0	0	0
APOB_HUMAN	516 kDa		0	0	0	1	0	0
ARGI1_HUMAN	36 kDa		0	0	0	0	0	0
PPCE_HUMAN	81 kDa		0	1	1	0	0	1
H3BRY5_HUMAN	33 kDa		0	1	0	1	0	1
PRS10_HUMAN	44 kDa		0	0	1	1	1	0
HNRDL_HUMAN	46 kDa	TRUE	0	0	0	0	0	0
RL5_HUMAN	34 kDa		0	0	0	0	1	0
TFG_HUMAN	43 kDa		0	0	0	0	0	0
Q5W0H4_HUMAN	22 kDa		0	0	0	0	0	0
H0Y394_HUMAN	109 kDa		0	0	0	1	1	0
ITIH4_HUMAN	101 kDa		0	0	1	1	0	0
COPD_HUMAN	57 kDa		0	1	1	0	0	0
TBCA_HUMAN	13 kDa		0	3	0	0	0	0
COCA1_HUMAN	325 kDa		0	1	0	0	0	0
POF1B_HUMAN	69 kDa		0	0	0	0	0	0
PSD11_HUMAN	48 kDa		0	0	1	2	1	0
K7ER96_HUMAN	31 kDa		0	0	1	0	0	0
PSB3_HUMAN	23 kDa		0	1	0	1	0	0
PPN_HUMAN	138 kDa		0	1	0	0	0	0
SKP1_HUMAN	19 kDa	TRUE	0	0	0	0	0	0
H0Y8X4_HUMAN	26 kDa		0	0	0	1	0	0
THIM_HUMAN	42 kDa		0	0	0	0	0	0
UK114_HUMAN	14 kDa		0	0	0	0	0	0
PSMD3_HUMAN	61 kDa		0	1	0	0	0	0
C9JDL1_HUMAN	31 kDa		0	0	0	0	0	0
GPDA_HUMAN	38 kDa	TRUE	0	0	0	0	0	0
GSDMA_HUMAN	49 kDa		0	0	0	1	0	0
LIMS1_HUMAN	38 kDa		0	0	0	0	1	0
BST2_HUMAN	18 kDa		0	2	2	1	1	1

UB2V2_HUMAN	16 kDa		0	1	0	1	0	1
GSTM2_HUMAN	23 kDa	TRUE	0	0	0	0	0	0
ANT3_HUMAN	53 kDa		0	0	1	0	0	1
F2Z2Y4_HUMAN	31 kDa		0	0	0	0	0	0
RET4_HUMAN	23 kDa		0	0	0	1	0	1
K6PF_HUMAN	93 kDa	TRUE	0	0	0	0	0	0
ACLY_HUMAN	121 kDa		0	0	0	3	0	0
RL9_HUMAN	22 kDa		0	0	0	0	0	0
FABP5_HUMAN	15 kDa		0	0	0	0	0	0
FAAA_HUMAN	46 kDa		0	0	0	0	0	0
DSC1_HUMAN	94 kDa	TRUE	0	0	0	1	0	0
K2C80_HUMAN	51 kDa	TRUE	0	0	0	2	0	0
ATP5H_HUMAN	18 kDa		0	0	0	1	1	0
H3BLU7_HUMAN	35 kDa		0	0	0	0	0	0
E7EX17_HUMAN	70 kDa		0	0	1	1	1	0
DEST_HUMAN	17 kDa		0	1	0	0	0	0
IF4A3_HUMAN	47 kDa	TRUE	0	0	0	0	1	0
CHM4B_HUMAN	25 kDa		0	0	1	1	1	0
PHS_HUMAN	12 kDa		0	0	0	0	0	0
FBLN1_HUMAN	74 kDa	TRUE	0	2	0	2	0	0
CPNE3_HUMAN	60 kDa	TRUE	0	0	0	0	0	0
FBP1L_HUMAN	70 kDa		0	0	1	0	0	1
COR2B_HUMAN	54 kDa		0	0	0	0	0	0
GPX1_HUMAN	22 kDa		0	0	1	1	0	0
ACOT9_HUMAN	51 kDa		0	1	1	1	2	0
CLIC2_HUMAN	28 kDa	TRUE	0	0	0	0	3	0
CERU_HUMAN	122 kDa		0	0	1	2	0	0
APOA4_HUMAN	45 kDa		0	0	0	0	0	0
ITA8_HUMAN	117 kDa		0	0	0	0	0	0
SAMH1_HUMAN	68 kDa		0	1	0	4	0	0
K22O_HUMAN	66 kDa	TRUE	0	0	18	0	0	0
GMFB_HUMAN	17 kDa	TRUE	0	0	0	0	0	0
SBSN_HUMAN	61 kDa		0	0	0	0	0	0
A2ML1_HUMAN	161 kDa		0	0	0	0	0	0
CSN1_HUMAN	59 kDa		0	0	0	0	1	0
ODO1_HUMAN	116 kDa		0	1	0	0	1	0
LRC47_HUMAN	63 kDa		0	1	1	2	1	0
SNX3_HUMAN	19 kDa		0	0	0	0	0	0
APT_HUMAN	20 kDa		0	0	1	0	0	0
PLA2R_HUMAN	169 kDa		0	0	0	0	0	0
F10A1_HUMAN	41 kDa		0	0	2	0	0	0
SYVC_HUMAN	140 kDa		0	0	0	1	2	0
CDV3_HUMAN	22 kDa		0	0	1	3	0	0
Q3SYB4_HUMAN	48 kDa		0	0	1	0	1	0
EFHD1_HUMAN	27 kDa	TRUE	0	0	0	0	0	0
ARRB1_HUMAN	46 kDa		0	0	0	0	0	0
NADC_HUMAN	31 kDa		0	0	0	0	0	0

ACS2B_HUMAN	64 kDa		0	0	0	0	0	0
TRFL_HUMAN	73 kDa		0	2	0	1	0	0
PCKGM_HUMAN	71 kDa		0	0	0	0	0	0
DNPEP_HUMAN	52 kDa		0	0	0	0	0	0
CALU_HUMAN	37 kDa		0	2	0	1	0	0
PLCB3_HUMAN	139 kDa		0	0	0	1	0	0
E7EUC7_HUMAN	58 kDa		0	0	0	0	0	0
DP13A_HUMAN	80 kDa		0	0	0	0	0	0
AFAD_HUMAN	198 kDa		0	0	0	0	0	0
SOGA2_HUMAN	210 kDa	TRUE	0	0	0	0	0	0
CO3A1_HUMAN	139 kDa		0	0	0	0	0	0
INVO_HUMAN	68 kDa		0	0	0	0	0	0
CD99_HUMAN	17 kDa		0	0	1	0	1	0
LC7L2_HUMAN	47 kDa		0	0	0	2	1	0
OXSR1_HUMAN	58 kDa		0	0	0	1	1	0
CAPG_HUMAN	37 kDa		0	0	0	1	0	0
TCAL3_HUMAN	23 kDa	TRUE	0	1	3	0	0	0
ARHGC_HUMAN	171 kDa		0	0	0	0	0	1
BDH2_HUMAN	27 kDa		0	0	0	0	0	0
EF1B_HUMAN	25 kDa		0	0	0	0	1	0
PRS6B_HUMAN	47 kDa		0	0	0	0	0	0
THS7A_HUMAN	185 kDa		0	0	0	0	0	0
KPRP_HUMAN	64 kDa		0	0	0	0	0	0
C4AM86_HUMAN	48 kDa	TRUE	0	0	0	0	0	0
RO60_HUMAN	61 kDa		0	1	0	0	0	0
GSTM3_HUMAN	27 kDa	TRUE	0	0	0	0	0	1
PSA4_HUMAN	29 kDa		0	0	0	0	0	0
GALM_HUMAN	38 kDa		0	0	0	0	0	0
KBTBB_HUMAN	66 kDa		0	0	0	0	0	1
UGGG1_HUMAN	175 kDa		0	0	0	0	0	0
EIFCL_HUMAN	105 kDa		0	0	0	0	0	0
GLGB_HUMAN	80 kDa		0	0	0	0	0	0
K2C8_HUMAN	57 kDa	TRUE	0	0	0	0	6	0
POSTN_HUMAN	93 kDa		0	2	1	0	0	0
IPYR_HUMAN	33 kDa		0	0	0	0	0	0
VWF_HUMAN	309 kDa		0	6	0	1	1	0
UBQL1_HUMAN	63 kDa	TRUE	0	0	0	0	0	0
PP1R7_HUMAN	42 kDa		0	0	0	2	0	0
DSG3_HUMAN	108 kDa		0	0	0	1	0	0
J3QS36_HUMAN	24 kDa		0	0	0	0	0	0
FBX50_HUMAN	31 kDa		0	0	0	0	0	0
PKP3_HUMAN	89 kDa	TRUE	0	0	0	0	0	0
ECI1_HUMAN	31 kDa		0	0	0	0	0	0
2ABA_HUMAN	52 kDa		0	0	0	0	0	0
PIN1_HUMAN	18 kDa		0	0	1	0	0	0
SF3B3_HUMAN	136 kDa		0	0	0	0	0	0
UACA_HUMAN	162 kDa		0	0	0	1	0	0

HINT1_HUMAN	14 kDa		0	0	0	0	0	0
ERP29_HUMAN	29 kDa		0	1	0	2	0	0
PSDE_HUMAN	35 kDa		0	0	0	0	0	0
B4DRL5_HUMAN	84 kDa		0	0	0	0	0	0
SERA_HUMAN	57 kDa		0	0	0	1	0	0
UB2L3_HUMAN	24 kDa		0	0	0	0	0	0
A1AG1_HUMAN	24 kDa		0	0	0	0	0	0
BLMH_HUMAN	53 kDa		0	0	0	0	0	0
EIF3E_HUMAN	52 kDa		0	0	0	0	0	0
Q2FXH4_STAA8	238 kDa		0	0	0	0	0	0
KCY_HUMAN	22 kDa		0	0	0	0	0	0
Q6ZR64_HUMAN	23 kDa		0	0	0	0	0	0
SEPT8_HUMAN	56 kDa	TRUE	0	0	0	1	0	3
KV106_HUMAN	12 kDa		0	0	1	0	0	0
PGDH_HUMAN	22 kDa		0	0	0	1	0	0
PSD12_HUMAN	53 kDa		0	0	0	0	0	0
DSC3_HUMAN	93 kDa	TRUE	0	0	0	0	0	0
TMOD1_HUMAN	41 kDa		0	0	0	0	0	0
DOPD_HUMAN	13 kDa		0	0	0	0	0	0
PRS6A_HUMAN	49 kDa		0	0	0	0	0	0
C9JIF9_HUMAN	82 kDa		0	0	0	0	0	0
TYB10_HUMAN	5 kDa	TRUE	0	0	3	3	1	0
SHPS1_HUMAN	55 kDa		0	0	0	0	0	0
INHBE_HUMAN	39 kDa		0	0	0	0	0	0
CO7_HUMAN	94 kDa		0	2	0	0	3	0
VGFR1_HUMAN	151 kDa		0	0	0	0	1	0
PEPD_HUMAN	55 kDa		0	0	0	0	0	0
UTP20_HUMAN	318 kDa	TRUE	0	0	0	0	0	0
B4DME2_HUMAN	77 kDa		0	0	0	0	0	0
LX12B_HUMAN	80 kDa		0	0	0	0	0	0
SYLC_HUMAN	134 kDa		0	0	0	0	0	0
IDE_HUMAN	118 kDa		0	0	0	0	0	0
PLCD1_HUMAN	88 kDa		0	0	0	0	0	0
LRC15_HUMAN	65 kDa		0	0	0	0	0	0
KRT82_HUMAN	57 kDa	TRUE	0	0	0	0	0	0
DHB12_HUMAN	34 kDa		0	0	0	0	0	0
K2C6B_HUMAN	60 kDa	TRUE	0	0	0	22	15	0
YBOX3_HUMAN	40 kDa	TRUE	0	0	0	1	2	0
SNAA_HUMAN	33 kDa		0	0	1	0	0	0
PRS4_HUMAN	49 kDa		0	0	0	0	0	0
RS15A_HUMAN	15 kDa		0	0	0	0	0	0
APEX1_HUMAN	36 kDa		0	0	0	1	0	0
CYTC_HUMAN	16 kDa		0	0	0	0	0	0
RL17_HUMAN	17 kDa		0	0	0	0	0	0
DUS3_HUMAN	20 kDa		0	0	0	0	0	0
RS13_HUMAN	17 kDa		0	0	0	0	0	0
KRT36_HUMAN	52 kDa	TRUE	0	0	0	0	0	0

CD2AP_HUMAN	71 kDa		0	0	0	0	0	0
DYL1_HUMAN	10 kDa		0	0	0	0	0	0
APRV1_HUMAN	37 kDa		0	0	0	0	0	0
GALK1_HUMAN	42 kDa		0	0	0	0	0	0
K1C15_HUMAN	49 kDa	TRUE	0	0	0	0	0	0
TGM1_HUMAN	90 kDa		0	0	0	0	0	0
INT3_HUMAN	118 kDa		0	0	0	0	0	0
DSG4_HUMAN	114 kDa		0	0	0	0	0	0
1B44_HUMAN	40 kDa	TRUE	0	0	0	0	0	0
SEMG1_HUMAN	52 kDa		0	0	0	0	0	0
SAA1_HUMAN	14 kDa	TRUE	0	0	0	0	0	0
DPP4_HUMAN	88 kDa		0	0	0	0	0	0
HRG_HUMAN	60 kDa		0	0	1	1	0	0
CHMP5_HUMAN	25 kDa		0	0	0	0	0	0
GLO2_HUMAN	29 kDa		0	0	0	0	0	0
NT5C_HUMAN	23 kDa		0	0	0	0	0	0
UFM1_HUMAN	9 kDa		0	0	0	0	0	0
1A34_HUMAN	41 kDa	TRUE	0	0	0	0	0	0
CO8B_HUMAN	67 kDa		0	0	0	0	0	0
GULP1_HUMAN	23 kDa		0	0	0	0	1	0
RS24_HUMAN	15 kDa		0	0	0	0	0	0
PKP4_HUMAN	127 kDa		0	0	0	0	0	0
SMD1_HUMAN	13 kDa		0	0	0	0	0	0
E9PF59_HUMAN	91 kDa		0	0	0	0	0	0
RL15_HUMAN	24 kDa		0	0	0	1	0	0
B5MBZ0_HUMAN	110 kDa		0	0	0	1	0	0
C1QB_HUMAN	27 kDa		0	0	0	0	0	0
LYAG_HUMAN	105 kDa		0	2	0	0	0	0
SAE1_HUMAN	38 kDa		0	0	0	0	0	0
RFL3S_HUMAN	12 kDa		0	0	0	2	0	0
B0QY89_HUMAN	71 kDa		0	0	0	0	0	0
GUAD_HUMAN	53 kDa		0	0	0	0	0	0
MARCS_HUMAN	32 kDa		0	0	0	3	0	0
FAS_HUMAN	273 kDa		0	0	0	0	0	0
HIBCH_HUMAN	43 kDa		0	0	0	0	0	0
IL36G_HUMAN	15 kDa		0	0	0	0	0	0
NDUV2_HUMAN	27 kDa		0	0	0	0	0	0
HDGR3_HUMAN	23 kDa		0	0	0	0	0	0
SYNC_HUMAN	63 kDa		0	0	0	0	0	0
PSB2_HUMAN	23 kDa		0	0	0	0	0	0
LBP_HUMAN	53 kDa		0	1	0	0	0	0
NPS3A_HUMAN	28 kDa		0	0	0	0	0	0
SPRE_HUMAN	28 kDa		0	1	0	0	0	0
WASL_HUMAN	55 kDa		0	0	0	0	0	0
DDX1_HUMAN	82 kDa		0	1	0	0	0	0
GGCT_HUMAN	21 kDa		0	0	0	0	0	0
RANB3_HUMAN	53 kDa		0	0	0	0	2	0

KRT83_HUMAN	54 kDa	TRUE	0	0	0	0	0	0
FABPH_HUMAN	15 kDa		0	0	0	0	0	0
KRT84_HUMAN	65 kDa	TRUE	0	0	0	0	0	0
SPB5_HUMAN	42 kDa		0	0	0	0	0	0
COPE_HUMAN	34 kDa		0	0	0	0	0	0
KR87P_HUMAN	29 kDa	TRUE	0	0	0	0	0	0
NHRF3_HUMAN	57 kDa		0	0	0	0	0	0
HMGB2_HUMAN	24 kDa	TRUE	0	0	2	1	0	0
ACTY_HUMAN	42 kDa	TRUE	0	0	0	0	0	0
IMDH1_HUMAN	64 kDa		0	0	0	0	0	0
Q5VWC4_HUMAN	41 kDa		0	0	0	0	0	0
R4GN49_HUMAN	7 kDa		0	0	0	0	0	0
HMOX1_HUMAN	33 kDa		0	0	0	1	0	0
UCRI_HUMAN	30 kDa		0	0	0	0	0	0
CUTA_HUMAN	21 kDa		0	0	0	0	0	0
LRC40_HUMAN	68 kDa		0	0	0	0	0	0
HUTH_HUMAN	65 kDa		0	0	0	0	0	0
K1C23_HUMAN	48 kDa	TRUE	0	0	0	0	0	0
OSTP_HUMAN	34 kDa		0	0	0	0	0	0
NUCKS_HUMAN	27 kDa		0	0	0	0	0	0
GNA13_HUMAN	44 kDa	TRUE	0	0	0	0	0	0
STMN1_HUMAN	20 kDa		0	0	0	2	0	0
SYFB_HUMAN	66 kDa		0	0	0	0	0	0
5NT3A_HUMAN	38 kDa		0	0	0	0	0	0
NAGK_HUMAN	42 kDa		0	0	0	2	0	0
HEMO_HUMAN	52 kDa		0	0	0	0	0	0
RS15_HUMAN	17 kDa		0	0	0	0	0	0
VSIG8_HUMAN	44 kDa		0	0	0	0	0	0
KT33B_HUMAN	46 kDa	TRUE	0	0	0	0	0	0
FCN2_HUMAN	30 kDa		0	0	0	0	0	0
TREX2_HUMAN	31 kDa		0	0	0	0	0	0
H0Y645_HUMAN	31 kDa		0	0	0	0	0	0
DERL1_HUMAN	26 kDa		0	0	0	0	0	0

Total Spect IgAN E0C0 G9	Total Spect IgAN E0C0 G10	Total Spect Normal kid G5	Total Spect Normal kid G6	Total Spect Normal kid G13	Total Spect Normal kid G14	Total Spect Normal kid G15	Total Spect Normal kid G28	Total Spect Normal kid G29
142	173	171	240	162	211	196	175	151
112	146	108	128	113	159	94	227	97
37	45	67	88	39	52	56	97	72
54	57	52	67	49	60	52	75	71
67	90	94	122	91	106	87	204	127
56	81	60	71	68	73	63	191	84
42	47	62	56	59	62	48	71	56
26	40	80	100	42	72	69	107	80
51	70	51	55	57	63	45	41	45
50	56	72	89	61	60	63	102	103
35	30	54	77	43	62	48	100	56
37	60	44	59	52	45	43	73	50
85	59	20	9	46	66	90	26	53
24	30	50	67	52	53	53	83	77
60	56	62	77	65	84	59	80	66
36	50	32	52	40	47	37	49	60
221	31	22	19	34	35	85	16	39
55	57	54	55	49	58	53	66	57
22	35	45	54	41	43	39	60	59
33	57	30	44	26	42	32	46	32
35	46	41	59	43	50	46	83	72
52	49	15	4	29	49	47	13	32
35	44	35	54	40	61	46	50	42
21	27	26	26	27	28	24	13	15
25	31	20	26	22	22	17	19	21
322	3	20	21	24	39	48	3	36
22	28	29	37	29	36	24	39	36
28	54	7	6	7	6	14	17	9
18	25	21	18	28	26	23	31	24
17	27	26	40	17	26	22	55	54
15	19	19	16	19	17	14	10	9
21	23	29	34	32	35	26	30	28
18	27	23	35	12	16	25	31	28
16	18	24	25	22	22	18	9	11
26	25	30	38	26	36	29	36	34
27	34	25	50	26	39	39	36	25
20	21	23	21	26	26	18	49	30
26	27	30	39	24	39	27	41	36
13	18	20	15	19	19	13	7	8
11	0	0	16	0	15	14	36	16
12	15	34	32	24	33	29	37	28
16	20	19	25	28	30	18	16	16
7	10	23	20	8	13	25	21	14

13	16	11	18	16	16	16	18	14
12	11	18	13	13	14	15	21	23
0	19	0	14	22	18	0	7	6
32	29	14	28	12	12	12	26	29
23	32	27	32	28	36	24	39	33
12	22	14	15	23	18	14	12	18
79	13	5	5	9	16	67	0	10
13	18	21	18	19	15	16	27	27
11	20	29	29	27	23	23	39	43
12	17	24	30	16	22	23	25	26
10	13	14	23	16	19	19	22	14
7	8	14	11	6	12	15	14	16
24	22	32	32	22	28	27	32	32
104	6	5	1	7	12	79	3	22
6	16	17	24	13	19	17	28	29
9	7	12	21	12	14	10	25	18
5	9	13	25	11	15	11	24	31
9	8	13	12	14	15	11	24	29
5	13	20	17	17	13	10	14	17
18	10	10	14	13	9	16	13	17
9	12	9	14	9	11	8	5	5
3	5	5	12	8	10	6	17	11
13	14	15	25	21	21	16	11	14
2	5	8	17	5	13	8	21	16
9	10	15	19	8	9	9	15	15
8	8	12	14	5	8	9	10	6
9	16	12	11	18	16	16	10	8
7	16	14	13	9	20	9	17	13
6	7	17	19	9	13	13	31	20
4	13	8	9	15	19	11	15	10
10	7	8	10	5	6	10	10	7
2	6	15	10	13	10	8	13	6
10	6	9	13	9	7	12	9	9
0	0	10	0	0	0	8	16	18
14	15	19	20	18	22	14	21	18
195	13	0	0	7	12	152	0	10
15	13	13	22	13	17	17	14	20
6	8	12	12	13	11	13	19	15
13	6	15	14	8	11	23	17	18
7	7	8	13	9	9	6	18	13
11	16	12	14	14	19	16	16	15
5	9	6	8	13	10	7	9	5
6	8	14	17	14	14	15	16	12
3	11	5	11	2	4	2	4	4
7	8	9	15	6	5	9	11	7
10	11	18	24	11	14	18	43	37
9	11	15	20	18	13	10	15	16

7	14	10	16	15	11	8	13	12
8	9	9	14	14	17	13	12	10
8	6	7	13	4	9	11	20	11
10	28	3	1	0	1	1	7	1
1	7	9	13	6	7	8	17	12
5	8	9	10	10	7	13	10	8
7	5	9	7	6	6	7	9	9
2	2	10	7	4	7	3	6	9
8	9	20	18	15	10	14	26	24
0	8	10	12	9	0	14	11	18
50	35	13	43	9	24	15	31	42
18	19	20	22	23	32	34	76	29
13	7	15	15	14	22	27	17	18
4	14	9	14	10	10	15	14	11
8	7	9	19	12	14	15	16	14
11	4	13	15	6	10	14	22	12
8	8	14	13	8	10	6	17	16
9	11	10	11	15	12	11	12	13
8	6	8	10	11	11	17	6	5
4	6	9	8	4	6	7	14	11
100	0	0	0	0	0	77	0	16
3	5	6	6	8	7	4	8	4
9	13	5	6	5	6	5	6	5
2	2	6	5	2	1	6	3	2
19	18	6	11	7	0	4	13	14
0	0	7	8	0	20	12	12	9
9	28	6	9	10	18	9	12	8
11	8	12	9	9	18	12	21	16
5	12	6	7	10	12	5	8	6
2	24	2	0	0	0	0	7	1
11	19	6	2	2	5	6	5	3
4	10	10	12	5	8	6	6	11
6	3	8	7	6	7	5	8	3
5	6	5	6	5	5	4	6	7
3	5	5	8	8	7	6	11	7
4	15	9	13	13	16	13	11	10
1	2	2	4	5	6	2	8	8
3	4	3	5	2	0	2	5	9
0	7	0	0	8	0	5	0	0
8	11	18	14	14	18	21	22	13
10	10	15	15	15	13	24	19	17
12	20	1	0	1	1	2	8	2
10	5	19	18	12	13	16	24	18
5	9	3	1	1	2	3	8	7
5	6	7	6	6	6	9	11	11
8	2	11	13	8	6	12	10	9
5	8	3	10	5	8	5	8	7

5	6	3	5	3	3	2	11	7
5	4	1	6	4	2	6	14	6
5	7	16	15	12	13	4	16	9
3	6	13	7	5	3	9	7	6
5	5	10	9	4	3	6	6	6
3	4	9	6	4	11	5	6	7
2	2	8	7	5	6	13	10	6
5	8	7	3	9	11	3	6	5
5	12	3	3	3	4	4	3	4
4	4	3	7	5	4	2	5	6
0	5	1	1	3	5	4	5	3
4	1	4	5	3	6	3	5	3
1	3	3	13	5	5	6	5	2
1	4	3	6	3	3	2	5	3
3	5	5	5	4	3	4	3	2
4	0	3	4	3	2	5	5	3
2	2	3	3	0	1	1	1	2
4	0	8	10	0	10	6	0	7
0	0	0	0	7	0	0	0	8
20	26	13	7	9	10	9	45	12
8	7	16	21	12	11	14	27	22
4	10	7	15	7	14	8	19	23
5	12	14	15	14	20	18	18	16
5	5	10	9	5	7	13	14	12
5	6	8	6	4	5	5	7	17
7	6	5	5	3	3	3	8	7
3	7	7	5	5	6	5	10	6
2	1	11	7	5	2	4	16	12
4	5	11	5	3	6	5	10	10
2	2	6	9	7	6	8	5	6
3	5	6	7	5	5	5	9	7
6	21	3	0	0	1	0	2	0
3	5	7	9	4	6	4	6	5
2	1	6	5	2	4	11	6	5
2	6	7	7	3	3	5	5	9
2	1	4	7	5	3	4	5	5
3	6	4	3	4	2	1	7	2
3	1	6	4	2	3	3	8	5
3	4	4	5	3	3	3	6	3
1	1	4	9	2	2	5	5	5
1	0	4	4	1	2	2	5	6
1	3	7	2	4	1	3	12	10
1	2	7	4	5	4	1	4	7
3	5	7	5	6	5	4	6	4
3	4	6	5	3	5	3	0	1
1	1	3	2	1	1	0	2	0
0	1	1	1	2	1	1	3	3

3	4	7	7	5	2	3	10	7
1	2	3	2	1	2	2	3	0
3	3	2	5	4	3	3	2	5
4	2	4	4	2	0	2	3	3
0	3	3	0	1	1	2	1	2
0	0	1	0	1	1	1	0	0
2	0	3	5	0	0	2	4	5
1	0	2	0	0	0	2	4	2
0	3	3	5	1	2	4	3	0
2	2	0	3	0	0	0	3	2
0	0	3	1	3	0	3	2	0
0	1	0	3	0	0	0	1	0
0	0	0	0	0	1	0	0	0
0	0	0	0	0	0	0	0	0
0	0	0	0	0	0	0	0	0
5	4	8	5	9	10	8	13	11
7	4	11	10	5	7	12	11	10
3	3	5	4	6	3	6	10	12
4	4	6	9	6	4	3	8	10
3	5	9	9	3	3	3	7	5
3	2	7	7	4	3	7	12	6
3	3	4	3	3	3	5	8	4
2	4	7	7	4	5	2	10	11
5	4	6	5	5	5	5	7	7
1	4	1	2	3	4	1	6	9
0	4	10	6	5	6	5	10	5
2	4	4	4	5	4	4	7	3
1	2	4	2	4	5	6	5	4
1	2	3	4	3	5	3	3	5
1	4	3	2	2	3	4	3	7
2	4	3	6	1	6	4	4	2
3	5	5	4	4	6	2	3	3
1	0	4	4	3	2	4	5	6
4	2	2	3	3	4	4	8	5
1	3	1	2	4	4	1	6	3
0	1	5	5	0	1	3	5	2
0	1	0	3	3	2	3	7	4
0	2	5	3	4	7	3	7	4
4	3	3	3	3	3	4	8	2
3	3	5	3	1	3	5	4	4
3	4	5	12	3	7	3	5	2
3	4	3	3	1	3	3	2	2
1	2	2	4	2	2	2	6	4
1	0	2	0	0	0	0	2	0
0	1	5	1	3	4	2	2	0
2	3	5	5	2	2	4	6	2
3	5	4	6	4	4	4	2	1

1	2	4	2	4	3	0	8	6
3	2	4	4	4	3	4	6	4
1	9	8	8	10	12	8	9	5
2	3	1	3	2	5	6	2	2
0	1	2	0	4	3	4	5	2
0	2	3	1	2	4	3	1	2
0	0	5	4	4	2	3	4	4
1	2	0	4	0	1	0	3	2
2	0	3	0	2	2	0	9	5
1	1	2	2	3	3	4	2	2
1	1	1	1	0	2	2	4	3
0	2	4	4	0	2	2	1	1
0	1	1	1	1	1	0	2	3
2	0	4	4	6	10	6	7	8
2	1	1	3	3	1	2	2	2
0	0	1	0	1	0	0	2	2
0	2	1	1	1	1	1	4	1
1	4	1	1	0	0	0	0	0
0	1	3	2	2	2	0	1	1
0	0	3	0	2	2	2	3	1
1	0	0	0	0	0	1	2	4
0	1	1	1	3	1	1	1	1
1	0	4	2	1	1	1	6	4
1	8	3	2	7	0	3	1	1
0	0	0	1	0	0	0	4	1
0	6	0	0	0	0	0	6	6
1	0	1	2	1	2	1	2	2
0	1	2	1	2	2	0	2	1
0	0	0	0	0	0	1	1	2
0	0	1	2	1	0	2	0	0
0	4	5	0	0	0	0	0	0
0	0	1	0	0	0	1	1	0
0	0	0	1	0	0	0	0	0
7	9	10	18	11	9	14	35	29
10	3	5	6	5	6	6	10	8
2	4	7	8	7	7	8	13	14
5	3	5	4	4	3	6	12	7
1	2	7	9	3	6	7	6	1
4	7	3	5	1	6	6	5	5
2	4	4	4	3	6	6	11	7
2	1	5	3	0	1	10	4	6
1	2	2	5	2	2	6	3	5
3	1	6	7	4	4	4	11	11
3	5	2	6	2	4	8	9	8
2	2	3	4	2	6	4	11	7
1	2	7	6	2	5	4	6	4
3	4	4	3	3	3	3	3	4

2	2	2	3	2	4	2	6	6
1	3	2	3	8	5	2	9	5
3	3	3	5	1	2	6	10	7
2	1	5	3	2	2	3	6	6
2	3	7	0	2	3	1	9	5
1	1	3	4	3	2	3	7	5
1	3	3	5	1	3	3	8	7
5	2	4	4	3	4	3	5	3
3	1	2	3	1	1	1	4	6
4	1	3	2	1	0	6	4	4
1	2	6	3	3	4	4	3	4
0	3	3	2	3	2	1	2	6
1	1	1	1	2	3	2	4	3
2	3	5	7	5	3	5	6	7
0	0	4	5	5	5	4	4	6
1	1	7	5	1	1	2	5	4
0	0	2	3	0	1	6	7	5
0	1	1	4	0	2	5	4	1
1	3	1	2	1	1	1	3	1
4	0	2	5	2	1	2	4	4
1	0	8	4	1	1	3	3	1
1	1	4	4	3	3	3	4	3
1	3	2	0	3	5	0	3	0
1	1	3	6	3	3	4	6	2
0	0	4	4	3	1	3	6	3
1	4	2	0	3	5	0	2	1
1	1	1	1	1	1	3	3	3
1	1	3	2	3	2	2	7	2
1	1	1	1	1	3	2	8	3
1	2	0	1	2	2	2	2	2
1	0	1	1	2	1	1	3	3
3	2	2	3	2	3	4	1	3
0	0	1	2	2	2	1	4	4
0	0	1	1	1	0	1	5	5
3	0	5	3	0	2	1	7	2
0	0	1	8	3	2	2	4	2
1	0	1	2	1	1	1	4	7
0	2	2	3	1	0	2	1	2
2	0	1	1	0	0	4	3	0
0	0	2	3	2	1	1	3	4
2	1	2	3	4	3	2	4	3
1	2	3	2	2	1	3	1	1
0	2	1	2	3	1	0	2	3
1	0	2	2	2	0	1	2	0
2	0	0	0	0	1	3	0	2
1	1	2	3	2	1	2	3	1
1	1	2	0	1	2	2	2	2

0	0	1	0	3	0	2	6	0
1	1	1	1	1	1	1	1	2
0	3	0	0	3	3	0	3	1
1	2	3	2	1	2	2	1	3
0	0	1	2	0	0	1	3	5
1	0	4	1	2	2	0	4	0
1	1	2	1	0	0	2	2	4
0	0	3	1	0	0	2	6	3
0	2	3	2	2	1	1	2	1
1	0	1	0	1	1	2	2	2
0	0	1	0	0	1	1	1	1
1	2	1	1	1	1	2	1	2
0	0	2	1	1	1	0	2	1
0	1	2	2	2	2	1	0	0
0	1	0	1	4	2	1	0	1
2	0	1	2	3	0	1	1	2
1	1	0	1	0	0	2	1	2
0	2	2	2	3	1	2	1	1
0	0	1	0	1	0	1	1	2
0	0	1	1	1	0	0	2	2
0	0	3	3	1	1	2	4	2
1	0	5	0	0	0	4	3	2
0	0	0	0	1	1	0	0	0
1	1	1	3	0	1	1	1	2
1	2	0	0	0	0	0	0	0
0	2	0	0	1	1	0	3	1
0	0	2	1	1	1	0	2	1
0	1	0	1	1	3	1	1	0
0	2	0	1	0	1	0	2	1
0	0	1	1	0	0	1	3	2
1	1	2	2	2	2	2	2	2
0	0	1	2	0	0	0	2	1
0	0	0	2	1	0	1	2	2
1	1	1	1	1	1	1	3	1
0	1	1	1	0	2	0	1	2
0	0	1	1	0	1	1	0	2
1	0	2	1	1	0	2	2	2
0	0	0	1	1	1	1	1	1
0	4	2	2	3	0	1	3	2
1	0	0	0	2	0	0	0	1
1	0	1	1	0	0	1	2	0
0	0	2	1	0	1	0	2	2
1	0	0	1	0	0	0	1	0
0	1	0	3	0	1	1	0	0
0	0	2	1	0	0	0	0	1
0	0	0	3	0	0	2	1	2
0	0	0	0	0	0	0	0	0

0	0	1	0	0	0	0	2	0
0	2	0	2	0	0	2	0	0
0	0	0	0	0	0	0	0	0
5	2	9	10	10	16	7	15	16
5	5	11	9	9	11	11	13	12
4	6	10	10	7	9	10	10	18
6	6	10	11	3	5	13	7	6
8	6	6	8	4	9	4	14	8
1	1	10	11	3	14	6	17	18
0	0	4	4	1	0	33	3	6
0	3	10	8	10	5	9	9	9
2	0	2	0	0	0	1	6	0
2	3	5	10	8	5	5	9	8
1	2	3	5	0	2	3	16	7
5	4	2	8	4	2	3	5	2
1	3	2	2	3	4	2	3	3
2	1	1	4	2	4	3	2	2
1	1	2	3	0	0	1	4	9
0	1	9	5	2	3	7	1	7
1	4	3	3	3	5	0	5	8
2	2	3	4	5	2	3	7	1
3	3	7	5	2	1	5	5	2
3	2	3	4	3	3	4	4	4
0	3	4	6	1	2	4	3	4
1	1	3	2	2	3	1	7	4
1	1	4	8	0	0	7	5	2
2	0	3	2	2	3	1	5	5
1	2	3	3	0	4	2	7	3
1	6	3	7	2	6	4	11	6
1	2	6	4	2	3	2	2	6
3	2	2	2	2	4	2	1	2
2	0	2	3	3	1	3	2	2
1	1	3	2	2	5	1	2	2
2	2	1	4	4	1	1	0	0
0	0	1	4	2	3	5	6	7
2	1	2	4	1	2	2	3	3
3	2	5	6	3	1	2	2	2
0	0	1	1	1	0	2	4	4
1	0	1	2	0	1	1	4	5
1	1	1	1	2	1	1	1	4
1	1	4	1	1	1	2	4	4
2	1	4	6	1	2	1	4	2
1	0	2	6	2	1	4	1	2
0	1	5	5	1	1	2	8	6
0	0	1	0	0	1	0	7	3
1	1	2	2	2	0	2	5	3
0	0	1	4	1	1	2	5	4

0	0	0	0	0	0	0	1	0
0	0	2	4	3	0	2	5	3
1	2	1	2	2	3	2	2	1
1	1	1	1	2	1	1	2	2
2	0	3	3	1	0	4	4	2
0	1	2	5	2	2	2	2	5
0	1	2	1	1	1	1	3	4
1	0	2	1	1	3	0	3	4
0	1	2	3	2	2	1	4	1
0	1	1	2	0	0	2	3	2
3	2	3	5	1	3	2	4	2
1	0	1	2	1	1	3	5	4
1	0	3	3	2	1	2	3	1
1	0	3	1	1	1	2	2	2
0	0	1	0	2	0	0	2	2
0	0	2	3	0	1	2	4	5
0	3	1	1	3	2	1	2	2
1	0	2	4	2	4	3	3	4
0	1	2	1	1	2	2	3	1
1	1	1	2	1	3	5	5	3
1	1	3	3	3	1	1	2	1
1	2	1	2	1	4	3	2	1
1	0	2	2	0	2	2	3	3
1	1	3	1	0	1	2	2	1
1	0	1	2	1	0	0	1	2
0	1	1	1	1	2	4	3	5
0	0	1	1	3	2	0	5	4
0	1	3	1	3	2	2	5	4
0	1	0	1	1	1	0	3	3
1	1	3	3	0	0	5	4	1
1	1	1	1	1	1	1	1	2
0	0	2	2	2	2	1	4	3
0	0	4	1	1	2	0	1	0
1	0	2	1	0	0	2	3	0
1	2	1	3	3	3	1	1	2
1	2	3	4	1	1	3	0	1
1	1	2	1	1	1	1	0	1
0	1	1	0	2	1	2	0	0
0	2	0	2	2	4	1	1	2
1	2	4	1	1	4	1	0	0
1	0	0	2	3	2	2	2	0
0	0	2	1	0	0	3	3	6
0	0	1	0	0	0	0	7	4
2	1	2	1	1	1	2	1	2
0	1	2	1	1	1	1	1	1
0	1	2	3	1	1	2	2	0
0	0	3	3	1	1	1	2	3

0	5	0	2	1	2	1	4	1
1	0	2	2	2	0	1	2	3
0	4	2	2	1	1	0	1	1
0	0	0	0	2	0	0	0	1
0	0	4	2	2	2	2	3	1
0	1	1	1	1	1	1	0	2
1	0	2	2	1	0	1	1	1
2	0	2	3	1	1	1	3	1
1	2	1	1	1	1	1	3	1
0	1	0	0	2	0	1	0	2
0	0	0	1	0	0	0	3	2
2	1	3	1	1	1	1	2	1
1	0	2	3	0	0	3	2	1
2	0	3	4	0	0	0	2	0
0	0	2	0	1	1	1	2	1
1	1	1	2	1	1	1	2	1
1	3	1	1	1	1	0	2	1
0	1	0	2	1	2	0	0	0
1	0	1	1	2	0	0	4	1
0	0	0	2	0	0	1	2	0
0	0	1	0	0	0	9	0	0
0	2	0	0	0	0	0	0	0
1	1	0	2	1	1	1	0	1
0	1	0	0	1	2	1	2	0
0	1	2	1	0	0	1	1	1
0	0	0	0	0	1	2	0	0
0	1	2	0	1	3	0	5	4
0	0	3	1	1	3	4	2	1
1	0	2	0	0	0	1	2	1
0	3	1	1	1	1	0	1	1
0	1	2	0	1	3	0	3	1
0	0	2	1	0	0	2	2	2
0	0	0	0	0	0	0	2	1
0	2	0	0	2	1	0	0	0
1	0	2	0	0	0	2	1	1
0	1	0	1	0	0	1	0	2
0	0	0	2	0	0	1	3	0
1	0	1	0	3	3	0	0	0
0	0	1	1	1	0	1	3	1
1	0	1	1	0	0	1	2	1
0	1	1	0	1	1	0	4	2
0	0	0	1	1	0	0	1	1
0	1	0	0	0	0	0	1	1
1	0	1	1	1	2	1	1	1
0	0	0	0	1	1	1	1	1
0	1	0	0	2	0	1	2	2
0	1	1	1	1	1	1	3	1

1	1	1	0	1	2	0	1	1
0	1	1	1	0	0	1	1	1
0	0	2	0	0	0	0	1	1
1	0	2	1	0	1	0	2	1
0	1	0	0	0	0	1	1	1
0	0	1	1	0	0	1	1	2
0	0	1	0	1	1	0	2	1
0	0	0	1	0	1	0	1	2
0	0	0	0	2	3	0	0	0
0	0	1	0	0	0	0	0	0
0	1	0	0	0	0	0	1	0
0	1	1	2	1	0	0	2	0
0	1	0	0	1	1	0	1	0
0	2	1	1	2	1	0	1	0
2	1	1	1	1	0	0	1	0
0	0	1	2	0	0	1	1	1
0	0	0	0	1	1	0	1	1
1	0	1	2	0	0	2	3	2
0	1	1	1	0	0	0	1	1
0	0	1	0	1	2	0	1	0
0	0	0	0	0	0	1	1	1
0	0	1	0	1	0	0	2	2
0	3	0	0	0	2	0	0	0
0	1	2	1	0	0	1	5	1
1	7	0	0	0	0	0	0	0
0	0	0	1	0	0	1	0	0
0	2	0	0	0	0	0	0	0
1	0	2	2	0	0	0	0	1
0	0	0	1	1	0	0	1	2
0	2	1	1	0	0	0	1	0
0	0	0	0	2	1	0	1	0
0	1	2	2	1	1	0	0	0
0	1	2	3	0	0	0	1	2
0	1	0	0	0	0	0	0	2
0	0	1	1	0	0	0	1	1
0	1	1	1	1	0	1	0	1
0	0	2	3	1	1	0	3	0
0	0	1	1	1	0	0	0	1
0	2	0	0	1	1	0	0	0
0	1	0	3	0	0	1	0	1
1	0	1	1	3	1	0	0	1
0	1	1	1	1	1	1	1	1
1	0	1	1	0	1	1	0	0
0	0	0	0	0	0	0	1	1
0	0	0	0	1	0	0	1	1
1	0	0	1	0	0	0	0	2
0	0	0	1	0	0	0	0	1

1	0	0	0	0	0	1	0	0
0	2	0	1	1	0	0	0	0
0	0	0	2	1	0	1	0	0
0	3	0	0	0	0	0	0	0
1	0	2	1	1	0	1	0	0
0	1	0	0	0	0	0	1	0
0	1	1	1	0	0	0	1	1
0	0	0	1	1	1	0	2	0
0	0	0	0	0	1	0	1	0
0	0	1	0	1	0	0	1	0
0	0	0	0	0	0	0	2	0
1	4	0	0	1	0	0	1	1
0	0	0	1	0	0	0	0	0
0	0	0	0	0	0	0	0	0
0	1	0	0	0	0	0	0	0
0	0	0	0	0	0	0	1	0
0	0	3	1	0	0	1	3	0
0	0	0	0	0	0	0	2	0
0	0	0	0	0	0	0	0	0
0	1	0	0	0	1	0	1	0
0	1	0	1	0	0	0	0	0
0	0	0	0	0	0	0	0	0
0	0	0	2	0	0	0	0	0
115	0	0	0	0	0	82	0	0
0	1	14	9	11	11	13	16	17
70	0	0	0	0	3	55	0	6
2	1	2	4	2	2	2	11	6
0	1	10	7	4	9	8	20	12
1	1	1	0	2	0	1	2	1
2	2	6	4	4	8	7	16	5
3	0	0	0	0	0	59	0	4
0	1	4	6	3	4	2	17	9
6	4	0	0	0	0	0	3	0
25	0	0	0	0	0	19	0	1
8	2	1	0	0	2	3	2	0
38	11	0	0	0	0	28	0	0
1	2	6	6	3	3	4	3	2
1	0	2	4	2	2	1	6	3
0	0	3	1	1	1	3	6	8
2	2	4	2	2	4	5	7	6
0	2	6	4	3	2	5	8	5
1	0	2	1	5	3	0	4	3
1	2	4	4	4	2	4	4	5
0	1	2	3	2	3	0	9	6
0	1	6	5	1	2	2	8	8
2	1	0	0	0	1	0	7	0
8	0	0	0	0	1	2	0	3

0	2	2	3	2	2	1	2	5
2	1	1	1	4	3	1	4	4
0	1	4	2	2	1	2	9	2
1	2	2	1	1	1	5	7	9
0	1	1	2	1	4	2	7	9
1	0	1	1	2	0	2	12	4
7	0	0	0	0	0	13	0	2
12	0	0	0	0	0	16	0	6
2	1	1	1	2	2	1	2	6
1	2	3	5	1	2	3	4	2
1	1	2	3	1	1	7	2	2
4	0	4	4	2	2	3	3	2
0	1	1	2	0	0	1	7	5
4	0	0	0	0	0	0	5	0
0	0	5	4	3	3	5	7	9
22	0	0	0	0	0	11	0	0
3	0	0	0	0	0	12	0	8
0	2	1	2	0	0	3	4	4
4	2	1	3	0	0	0	1	3
4	9	2	0	0	2	3	3	2
2	1	4	5	5	4	2	3	5
0	0	0	0	0	0	0	1	1
0	0	4	2	1	3	5	11	5
0	0	3	2	2	2	2	6	1
1	0	1	0	0	0	2	1	2
0	0	5	3	1	0	1	4	9
1	0	1	2	0	1	2	2	4
0	1	4	2	1	0	2	4	4
0	4	12	3	0	2	1	5	1
0	3	4	0	1	1	2	2	3
11	0	0	0	0	4	8	0	5
1	0	0	0	0	0	19	0	0
2	0	3	4	3	2	3	2	1
0	1	3	3	1	1	3	2	2
0	2	1	3	1	0	0	2	2
0	0	3	3	0	1	2	4	6
0	0	2	0	0	0	0	7	10
1	0	3	5	2	1	3	2	3
12	0	0	0	0	0	18	0	1
0	1	0	0	1	3	0	0	1
0	0	0	0	0	0	0	0	0
1	1	2	4	0	0	1	2	2
0	5	0	0	1	0	0	0	0
1	0	2	1	2	2	2	4	5
1	1	1	2	4	0	2	2	2
1	1	2	1	0	1	1	1	1
1	2	2	0	3	2	1	2	2

1	1	2	1	0	0	3	4	3
0	0	1	3	3	4	3	1	2
1	0	2	0	1	0	3	3	4
0	1	0	1	4	4	1	0	1
0	0	0	2	2	2	2	0	2
0	0	3	0	1	2	5	3	1
0	0	1	2	5	2	5	3	3
5	0	0	0	0	0	0	0	4
1	0	2	0	2	1	0	0	0
0	1	5	2	0	0	1	5	1
0	0	4	0	1	1	2	7	4
0	0	2	1	0	0	2	2	2
0	0	1	1	0	0	0	0	0
0	0	0	0	0	0	25	0	0
0	1	9	1	0	1	1	2	2
0	0	0	2	0	2	1	3	3
0	0	0	0	0	0	28	0	0
1	1	2	0	0	0	0	3	1
0	0	2	2	2	2	1	2	2
1	0	1	2	2	2	2	1	2
1	1	2	2	1	0	2	3	3
0	1	0	3	3	2	0	2	0
0	1	0	0	2	0	0	1	0
0	0	0	0	0	0	20	0	0
1	1	2	0	1	0	2	3	1
0	0	3	0	0	0	3	1	1
0	2	2	1	1	1	2	6	5
1	0	2	3	3	0	2	4	0
0	0	1	3	1	2	2	4	3
0	0	2	1	1	2	0	6	7
0	0	0	0	0	0	0	0	0
0	0	0	0	0	1	1	4	2
1	0	2	3	0	2	1	3	1
0	0	2	4	0	1	2	5	3
8	0	0	0	0	1	2	0	0
0	1	3	3	2	1	3	2	3
1	2	6	2	0	3	3	4	4
10	0	15	15	0	18	14	45	23
0	0	1	3	1	2	0	2	2
0	0	1	0	2	1	1	1	1
0	0	0	1	0	1	0	3	3
0	0	4	2	1	0	1	4	2
0	0	5	8	0	0	3	10	14
0	0	6	1	1	4	2	6	1
9	0	0	0	0	0	11	0	1
0	0	0	0	0	0	0	0	9
0	1	0	0	1	1	0	2	0

1	0	1	1	1	1	2	2	3
0	0	2	1	1	3	2	3	2
0	3	2	1	4	3	1	0	0
0	0	2	1	0	0	2	2	1
0	0	0	0	0	1	0	1	2
0	0	0	0	0	0	11	0	2
1	0	3	1	1	1	1	1	1
0	0	2	1	1	1	2	2	2
0	1	1	0	0	1	1	2	3
0	0	1	0	0	0	3	2	1
1	1	1	0	1	1	1	2	2
0	1	1	2	0	3	1	0	3
0	2	0	0	2	2	0	2	1
12	16	14	25	20	21	15	0	0
12	15	15	24	0	26	16	16	15
0	0	3	2	3	3	0	4	2
0	18	0	11	0	0	15	12	0
0	0	0	0	1	0	1	7	5
5	0	0	0	0	0	56	0	0
1	1	1	2	2	0	2	2	3
0	0	0	1	1	1	0	2	1
2	2	0	0	1	0	2	0	1
0	0	4	1	0	0	1	2	1
3	2	0	0	0	0	0	0	1
0	0	0	1	0	1	1	2	3
0	0	1	2	0	1	1	3	2
0	0	1	1	0	1	1	1	2
0	0	1	0	0	0	1	3	3
0	0	1	0	0	0	1	11	4
1	2	0	1	2	1	1	0	1
0	1	1	0	3	3	1	1	1
0	0	2	2	1	1	2	2	3
0	2	3	1	0	0	1	0	2
0	0	0	0	0	0	0	0	0
0	2	1	0	2	1	0	2	0
5	0	0	0	0	0	9	0	0
70	0	0	0	0	0	58	0	8
0	0	3	0	0	0	1	0	0
7	0	0	0	0	0	13	0	3
0	1	0	0	2	1	0	0	1
0	2	2	0	3	0	0	1	1
0	0	1	1	1	0	2	0	0
0	0	2	1	0	1	1	2	3
5	0	0	0	0	0	5	0	2
0	0	0	0	0	0	9	0	0
0	1	2	0	1	1	0	1	1
1	3	1	1	1	1	0	1	1

2	1	1	4	1	1	0	4	1
0	0	2	0	1	0	0	1	1
0	0	0	1	1	3	0	1	0
0	0	0	1	1	0	2	0	1
0	0	0	6	0	0	1	1	1
0	0	0	0	0	0	0	10	9
0	5	0	0	5	0	0	7	10
0	0	4	0	0	0	1	0	5
0	0	2	1	0	0	0	1	0
0	0	0	3	0	0	3	2	2
0	1	1	1	1	1	0	2	1
1	0	1	1	0	0	1	1	0
0	1	3	0	0	0	0	1	1
0	0	0	1	0	0	1	2	1
0	1	3	2	3	2	1	3	0
2	0	0	0	0	1	5	0	0
1	0	1	0	0	0	3	3	2
0	0	3	2	1	1	4	2	1
0	0	2	2	0	0	3	1	0
0	1	0	0	0	1	1	1	0
0	0	0	0	0	0	13	0	0
1	0	1	1	1	0	2	2	1
1	1	1	1	0	0	1	2	2
0	1	2	1	0	2	1	1	0
1	1	1	1	0	1	1	1	3
0	0	0	0	1	1	0	2	2
1	1	0	0	0	0	0	0	0
1	1	3	0	0	0	0	4	1
0	0	8	5	0	3	0	0	5
0	1	0	0	0	0	0	0	0
0	1	0	0	1	1	0	0	1
0	2	2	3	2	1	4	0	0
0	1	0	1	1	0	1	2	0
0	0	0	0	0	0	1	3	0
0	0	0	0	2	2	0	2	2
196	0	0	0	7	0	149	0	9
2	0	0	0	0	0	0	0	0
0	0	2	0	0	0	0	0	0
0	2	1	1	1	0	0	0	0
2	0	0	0	0	0	0	0	0
0	0	3	1	1	4	0	4	0
0	0	0	0	1	2	0	0	0
0	1	1	0	2	2	0	0	0
0	0	3	1	0	0	2	1	1
0	0	1	0	0	0	0	1	2
0	0	0	0	0	0	0	1	1
0	0	3	0	1	0	0	5	2

0	0	1	3	0	0	1	4	2
0	0	0	1	0	0	0	2	0
0	0	0	0	0	0	0	0	0
0	1	1	1	1	1	0	1	0
0	0	12	0	10	0	0	9	12
1	1	1	2	1	0	0	0	2
0	0	1	5	0	0	3	3	2
0	1	1	1	2	0	0	2	1
0	1	1	0	1	1	0	0	1
0	1	2	1	0	0	0	2	1
0	0	2	2	0	0	0	2	2
0	0	3	0	0	1	2	0	0
0	0	0	1	1	0	1	0	2
0	1	2	1	1	1	0	1	1
0	0	0	1	0	0	0	0	1
0	0	2	1	0	1	2	2	2
0	0	0	0	0	0	0	1	0
0	0	1	0	0	2	1	4	2
0	0	0	0	0	1	0	0	0
4	0	0	0	0	0	10	0	0
1	0	1	1	0	0	0	2	0
0	0	1	1	1	0	1	0	2
1	0	0	0	0	0	2	0	0
1	0	0	0	0	0	0	3	3
0	0	1	1	1	1	0	2	0
0	0	0	0	0	0	0	1	2
0	0	0	0	0	0	3	1	1
0	1	0	0	0	2	1	0	0
0	0	1	0	0	0	0	0	0
0	0	1	0	0	0	0	4	0
0	0	3	0	0	0	1	0	0
0	0	0	0	1	0	3	0	0
0	0	0	0	0	0	13	0	0
1	1	1	1	1	1	1	2	0
0	2	1	0	1	1	1	2	0
0	0	1	0	0	0	1	1	0
1	0	1	0	1	0	0	0	0
0	0	1	0	0	0	0	2	0
0	0	1	2	2	1	1	3	1
0	0	1	2	1	1	1	1	0
1	0	0	1	0	0	1	1	1
0	0	1	0	0	1	1	2	1
0	0	0	0	2	2	0	1	0
0	0	1	0	0	0	1	2	0
0	0	0	0	0	0	8	0	0
1	2	1	1	1	0	1	1	0
0	1	0	2	1	0	0	1	0

0	0	0	0	0	0	0	0	1
0	0	0	0	1	0	0	1	2
0	0	0	0	0	0	0	1	0
1	0	3	1	0	1	1	0	1
0	0	0	0	0	1	0	1	0
0	0	3	3	0	0	1	2	2
1	0	1	0	0	1	0	0	0
0	0	0	0	0	0	0	2	2
3	0	0	0	0	0	3	0	0
0	0	1	0	0	0	0	4	0
5	0	0	0	0	0	4	0	0
4	0	0	0	0	0	5	0	0
0	0	1	3	0	0	0	1	1
0	0	0	1	0	0	0	2	2
0	2	0	0	2	2	0	1	0
0	0	1	1	0	0	1	2	2
0	2	2	3	0	0	0	0	0
0	1	0	0	1	0	0	0	0
0	0	0	1	1	2	0	2	1
0	5	0	2	0	0	0	0	0
0	0	0	0	0	0	0	2	1
0	0	1	1	1	2	0	0	0
0	0	2	0	0	0	1	1	2
0	0	0	0	1	1	0	0	0
0	0	0	1	2	1	0	0	0
1	0	0	2	1	0	2	0	1
0	0	0	0	0	0	0	0	0
0	0	0	0	0	0	0	2	0
0	0	1	2	0	0	1	4	2
0	0	0	0	0	0	0	0	0
14	12	0	0	0	0	10	0	0
0	0	0	0	0	0	0	1	3
1	0	0	0	0	0	10	0	0
0	0	0	0	0	0	11	0	0
0	1	1	0	2	1	2	2	0
0	0	0	0	0	0	0	2	0
0	1	1	1	0	1	0	0	0
0	0	1	0	0	0	0	0	1
1	0	1	1	0	0	2	0	0
0	0	1	0	0	0	0	2	1
0	0	1	0	1	0	0	1	0
0	0	0	0	0	0	0	0	0
0	1	1	0	2	1	0	0	0
2	0	0	0	0	0	1	0	0
0	0	1	0	0	0	0	2	1
0	0	0	2	0	0	0	1	0
0	0	1	0	0	0	0	1	1

0	1	2	0	0	0	0	0	0
0	0	0	0	0	0	0	1	0
0	0	3	1	0	0	0	1	0
0	0	0	0	1	0	0	2	2
0	0	0	0	0	0	0	0	0
0	0	0	0	2	1	1	0	0
0	0	2	2	0	0	0	0	0
0	0	0	2	0	0	0	2	1
0	0	0	1	0	0	0	0	0
0	0	0	0	0	0	2	0	0
0	0	0	0	0	0	0	0	0
5	0	0	0	0	0	6	0	0
0	1	0	0	0	1	0	0	1
0	1	0	0	0	1	1	0	1
0	0	0	0	1	1	0	1	1
1	0	0	0	1	1	1	0	1
0	0	1	0	0	0	1	2	0
0	1	1	2	0	1	1	0	0
0	0	1	2	0	0	1	1	2
0	0	1	0	1	0	0	0	0
0	0	1	1	0	0	2	3	1
0	0	1	2	0	0	0	0	1
3	0	0	0	0	0	1	0	0
0	0	0	0	0	0	0	0	0
0	0	0	0	0	0	1	2	2
0	0	0	0	0	0	0	3	3
0	0	1	1	0	0	1	1	1
0	0	1	0	2	3	1	2	1
0	1	0	0	0	0	0	2	0
0	0	1	0	0	0	0	2	0
0	1	0	0	0	0	0	0	0
0	0	0	0	0	0	0	2	1
0	0	0	0	0	0	0	0	0
0	0	0	0	0	0	0	0	2
0	0	1	0	0	1	0	2	1
0	0	0	0	0	0	0	0	0
0	0	0	0	0	0	0	0	0
0	0	0	0	0	0	0	0	0
0	0	0	0	0	0	0	0	2
0	0	0	0	0	0	0	3	0
1	0	0	0	0	0	5	0	0
0	0	0	0	0	0	0	0	0
0	0	1	1	0	0	0	2	1
0	1	0	0	0	0	0	2	1
0	0	2	1	0	0	0	1	1
0	0	2	1	0	0	0	1	0
0	0	1	1	0	0	0	1	0

0	0	2	1	0	0	1	2	0
0	0	0	0	0	2	0	1	0
0	0	2	0	0	0	1	0	2
0	0	2	1	0	0	0	0	0
3	0	0	0	0	0	3	1	0
0	0	0	0	0	0	0	1	3
0	0	0	0	0	0	0	0	0
0	0	0	0	0	0	5	0	0
0	0	0	0	0	0	0	1	0
0	0	0	0	0	0	0	0	0
0	0	1	0	0	0	2	0	0
0	0	0	0	1	0	0	0	1
0	1	0	3	0	1	0	0	0
0	1	0	0	0	0	0	0	0
0	0	0	0	1	0	0	1	1
0	0	0	0	0	0	0	2	1
3	0	0	0	0	0	1	0	0
1	0	2	2	0	0	0	0	0
0	0	1	0	0	0	0	0	0
0	0	2	1	0	0	0	2	0
0	0	2	0	1	0	0	2	0
0	0	0	0	0	0	0	0	0
0	0	0	0	0	0	0	0	0
0	0	1	2	0	0	0	0	0
0	1	0	0	0	0	0	0	0
0	0	0	0	0	0	0	0	0
0	0	0	0	0	0	0	2	0
0	0	0	0	0	0	0	0	0
0	0	0	0	0	0	0	0	0
0	0	0	0	0	0	2	0	0
0	0	0	0	0	0	0	2	0
0	0	0	0	0	0	6	0	0
0	0	0	0	0	0	0	0	0
0	0	0	0	0	0	0	0	0
0	0	0	0	0	0	0	0	0
0	0	0	2	0	0	0	1	1
246	0	0	0	0	0	154	0	0
0	1	0	0	2	1	0	0	0
0	0	0	0	0	0	0	1	0
0	0	1	0	0	0	0	3	2
0	0	0	0	0	0	0	2	0
0	1	0	0	0	0	0	0	0
0	0	0	0	0	0	0	0	0
0	0	0	0	0	0	0	0	0
0	0	0	0	0	0	0	2	1
0	0	0	0	0	0	0	0	0
0	0	0	0	0	0	0	0	0

0	0	0	1	1	0	0	3	0
1	0	0	2	0	0	2	0	0
1	0	0	0	0	0	4	0	0
0	0	0	0	0	0	0	0	0
0	0	0	0	0	0	14	0	0
0	0	0	0	0	0	4	0	0
0	0	0	0	0	0	0	0	0
0	0	0	0	0	0	0	0	0
0	8	0	0	0	0	0	0	5
0	0	0	0	0	0	0	0	0
0	0	0	0	0	0	0	0	0
0	0	0	0	0	0	0	0	0
1	0	0	0	1	0	0	0	0
0	0	2	1	1	0	1	0	0
0	0	2	2	0	1	0	0	1
0	0	2	0	0	0	0	1	2
0	0	0	0	0	0	0	1	2
0	8	0	0	0	10	0	0	0
1	3	0	0	0	0	0	0	0
0	1	0	0	2	0	0	0	0
0	0	0	0	0	0	0	0	0
0	0	0	0	2	0	0	1	0
0	0	0	0	0	0	0	1	3
0	0	0	1	0	0	0	1	2
0	0	0	0	0	0	0	0	0
0	0	0	0	0	0	0	2	0
0	0	0	0	0	0	0	0	0
0	0	0	0	0	0	0	0	0
0	0	0	0	0	1	0	3	0
0	0	0	0	0	0	0	0	0
0	0	0	0	0	0	1	0	0
0	0	0	0	0	0	2	1	0
0	0	0	0	0	0	0	0	0
0	0	0	0	0	0	2	0	0
0	0	0	0	0	0	0	0	0
0	0	0	0	0	0	4	0	0
0	0	0	1	0	0	0	1	0
0	0	0	2	0	0	1	0	1
0	0	0	0	0	0	0	2	1
0	0	0	0	0	0	1	1	0
0	0	0	0	0	0	0	0	0
0	0	0	0	0	0	0	2	1
0	0	0	0	0	0	0	3	0
0	0	0	0	0	0	0	1	0
0	0	0	0	0	0	0	2	0
0	0	0	0	0	0	2	0	0
0	0	0	0	1	0	0	0	0

0	0	0	0	0	0	0	0	0
0	0	0	0	0	4	0	0	0
0	0	0	0	0	0	0	0	0
0	0	0	0	0	0	0	0	0
0	0	0	0	0	0	0	4	0
0	0	0	0	0	0	0	0	0
0	0	0	0	0	0	0	0	0
0	0	0	0	0	0	0	0	0
0	0	0	0	0	0	0	2	3
0	0	0	0	0	0	0	1	0
0	0	0	0	0	1	2	0	0
0	0	0	0	0	0	2	0	0
0	0	0	0	0	0	3	0	0
0	0	0	0	2	0	0	2	0
0	0	0	0	0	0	0	3	0
0	2	0	0	0	0	0	0	0
0	0	0	0	0	0	2	0	0
0	0	0	0	0	0	2	0	0
0	0	0	0	0	0	0	0	0
0	0	0	1	0	0	0	0	2
0	0	0	0	0	0	0	0	2
0	0	0	0	0	0	0	0	0
0	0	0	0	0	0	2	0	0
0	0	0	0	0	0	2	0	0
0	0	0	0	0	0	0	0	0
0	0	0	0	0	0	0	0	0
0	0	0	0	0	0	0	3	0
0	0	0	0	0	0	0	0	0
0	0	0	0	0	0	0	0	0
0	0	0	0	0	0	0	0	0
0	0	0	0	0	0	2	0	0
0	0	0	0	0	0	0	0	0
0	0	0	0	0	0	0	0	0

Total Spect	Total Spect	Total Spect	Total Spect	Total Spect	Total Spect	Total Spect	Total Spect	Total Spect
Normal kid	Vancomyci	Vancomyci	Vancomyci	Vancomyci	IgAN E1C1	IgAN E1C1	IgAN E0C1	ATN
G30	G16	G17	G18	G19	G20	G21	G23	ATN, other
144	108	128	91	62	32	141	100	62
76	70	66	71	79	57	74	97	55
51	51	63	54	6	0	50	28	51
64	40	48	46	12	6	79	34	17
116	69	103	81	55	38	95	80	48
67	63	65	73	50	43	73	59	38
56	51	61	44	25	9	60	38	34
68	35	43	25	5	3	58	24	21
45	29	39	33	20	16	49	44	29
84	66	80	59	22	5	72	35	37
64	61	44	84	31	12	59	46	30
46	37	45	54	33	32	57	42	27
15	127	14	4	12	88	21	55	32
62	29	60	31	9	1	39	17	14
71	39	71	65	29	13	66	45	31
44	39	42	35	17	5	44	32	24
25	119	15	3	38	103	23	79	39
62	51	50	50	27	14	59	36	36
54	28	48	23	5	0	40	17	16
18	32	32	29	10	4	38	36	10
41	41	50	50	23	9	45	34	22
18	184	7	0	7	78	17	40	17
48	31	46	37	24	9	48	31	18
10	11	12	11	12	5	15	14	10
21	19	18	16	7	2	25	13	6
19	119	5	0	34	69	9	70	36
30	21	29	20	9	5	19	17	14
9	27	52	35	7	4	50	16	5
28	17	21	15	14	12	24	20	12
40	30	38	27	13	10	36	19	18
8	5	8	6	8	0	6	10	6
24	22	25	22	6	3	22	13	17
22	15	15	11	0	0	17	8	7
9	9	7	7	5	4	16	7	7
25	20	24	19	12	8	27	16	25
20	15	31	25	10	3	25	13	6
23	18	20	19	16	9	25	13	22
23	20	27	20	12	9	29	14	22
7	6	0	6	0	0	6	0	0
0	0	19	20	0	0	21	26	12
25	17	23	14	4	2	12	7	10
17	11	8	15	10	9	13	18	16
11	11	17	9	3	3	9	10	29

29	14	17	22	12	11	24	26	13
18	11	21	15	4	8	13	4	15
6	6	5	8	6	0	6	8	4
5	35	39	43	33	22	52	34	9
21	11	23	17	10	1	27	17	9
17	11	11	7	12	4	19	12	3
6	61	5	0	2	42	6	18	10
24	13	22	9	5	0	6	10	10
41	21	26	20	6	2	17	6	12
28	21	27	26	11	3	26	20	16
15	15	13	14	5	5	12	8	14
11	12	11	8	1	0	10	3	5
20	18	23	16	0	0	21	11	24
4	57	7	1	4	44	6	24	12
19	14	24	14	8	7	17	13	17
13	14	20	16	6	0	12	12	4
18	12	21	10	5	1	16	6	5
22	16	22	13	5	8	16	9	12
18	6	10	9	7	2	8	6	8
10	9	11	11	2	13	9	7	7
5	4	2	1	5	7	3	11	6
5	9	7	7	1	0	14	2	2
11	7	9	10	10	7	12	14	10
17	7	12	19	7	3	12	11	2
13	7	10	5	2	0	13	5	7
10	9	8	5	5	5	9	7	4
8	7	8	5	6	5	7	8	6
13	12	13	9	1	2	14	10	4
12	11	20	13	2	1	9	3	6
8	9	9	7	9	5	9	10	8
10	9	10	9	5	6	8	7	4
7	6	7	8	0	0	6	2	3
7	5	8	6	0	1	5	4	3
0	11	9	0	0	0	16	0	9
18	8	19	13	1	0	10	4	7
6	50	8	0	0	32	6	19	7
12	14	16	13	4	24	12	4	3
18	12	19	9	5	2	10	7	12
17	14	15	12	1	0	10	4	5
11	10	14	9	1	1	5	4	8
16	12	11	12	4	9	15	11	9
6	6	5	8	0	0	8	3	5
17	11	14	10	6	0	14	6	12
11	0	3	1	0	0	4	2	0
10	11	8	5	4	0	11	7	4
19	17	36	17	2	0	13	12	14
16	8	13	8	3	0	7	3	5

9	7	13	11	4	3	9	4	6
10	12	9	4	5	6	9	6	5
10	9	13	13	2	1	12	4	2
1	10	22	13	1	1	12	16	0
14	8	13	6	0	0	11	3	5
8	3	9	5	2	4	4	2	3
9	5	7	7	2	2	8	3	4
8	6	9	5	0	0	6	1	5
18	10	23	0	0	0	6	8	5
11	9	12	10	0	0	12	0	0
3	43	35	30	17	13	42	18	5
20	9	19	17	9	7	14	14	6
17	8	16	8	4	8	11	7	12
11	7	8	8	9	6	10	12	5
12	7	12	5	2	1	8	3	8
13	12	20	12	0	0	3	1	7
10	6	12	5	4	2	9	4	6
12	6	11	7	1	0	8	1	5
9	5	6	4	4	4	6	3	3
11	11	10	7	3	0	7	4	5
0	47	6	0	0	25	0	25	0
5	6	8	5	1	0	9	1	0
4	2	3	9	3	2	7	4	1
6	2	5	4	0	0	2	0	0
0	20	23	30	19	12	37	18	5
11	9	9	0	14	0	15	24	11
12	11	9	9	14	12	15	26	11
11	8	13	9	3	1	12	5	6
11	10	5	9	0	1	3	3	8
1	10	15	13	6	1	14	11	0
5	8	8	12	10	9	11	9	1
9	5	7	9	1	0	7	6	3
8	2	6	5	3	3	4	6	4
4	5	3	3	0	0	3	4	4
10	0	4	9	2	0	6	4	2
12	7	9	10	4	0	14	9	7
6	2	4	6	1	0	3	0	1
4	6	8	5	1	0	3	1	2
0	0	0	0	0	0	0	0	2
14	11	15	12	6	5	13	10	13
15	14	16	9	2	3	10	5	5
0	16	23	25	5	2	16	8	3
14	12	19	8	1	0	8	4	5
3	10	8	18	3	5	8	5	0
8	7	7	7	4	3	8	8	12
10	6	10	6	1	0	11	3	2
9	4	11	7	2	1	12	2	3

5	8	5	6	0	0	9	3	1
5	2	4	4	0	0	6	2	1
10	4	14	9	0	0	14	0	3
9	5	4	4	0	0	3	5	7
4	3	6	2	0	0	3	0	4
4	4	5	6	3	2	4	6	2
5	3	8	1	0	0	3	3	0
6	5	5	5	3	2	4	5	5
2	5	5	6	0	0	4	5	2
7	5	4	5	0	0	5	1	2
5	3	8	8	0	0	7	6	2
5	4	7	7	0	0	6	0	2
1	2	5	2	0	0	0	1	1
2	1	3	3	1	0	2	1	1
4	3	3	3	0	0	3	0	1
2	3	5	2	2	0	4	0	2
1	0	3	4	0	0	2	0	0
4	5	0	7	0	3	6	7	0
0	0	6	0	0	0	0	0	0
13	26	29	29	22	12	27	24	6
21	10	21	10	7	3	12	5	2
15	11	16	9	0	0	10	4	2
17	7	13	12	4	5	17	10	8
14	3	9	4	1	3	10	3	2
6	6	8	8	3	2	9	6	3
8	8	5	8	1	0	7	5	5
10	3	7	6	1	0	7	1	2
8	5	14	3	1	0	3	1	2
6	5	7	7	0	0	9	2	3
5	3	6	2	0	0	3	1	2
6	5	5	8	0	0	5	1	3
2	4	6	5	10	2	5	8	0
5	3	4	7	4	1	6	2	3
5	4	7	2	0	0	6	1	2
5	3	6	4	1	0	1	3	2
3	5	5	3	3	2	3	2	1
2	2	3	4	4	4	1	4	2
6	7	4	2	1	0	6	5	4
7	2	6	4	0	0	2	0	3
7	1	8	3	0	0	4	2	1
6	3	7	5	0	0	5	0	4
6	4	5	5	0	0	3	1	1
4	3	4	1	0	0	4	0	1
4	1	3	2	0	0	2	0	0
1	0	0	0	0	0	2	1	0
4	2	2	1	0	0	2	0	0
3	2	2	2	1	1	3	2	1

6	4	7	4	0	0	5	0	3
3	2	4	3	0	0	3	1	4
4	1	2	2	0	0	1	0	0
3	3	1	2	0	0	3	1	2
2	1	4	1	0	0	4	0	1
0	2	2	2	5	2	0	0	2
4	3	0	3	0	0	1	0	0
2	3	4	2	0	0	2	0	2
0	0	2	1	0	0	4	3	6
2	1	3	1	0	0	3	1	3
1	0	2	0	0	0	2	2	1
2	0	0	1	0	0	6	0	0
0	0	0	0	0	0	0	0	0
0	3	0	0	0	0	0	0	0
0	0	0	0	0	0	0	0	6
14	8	8	6	0	0	10	1	2
11	7	7	4	3	3	9	4	2
11	5	9	7	0	0	11	1	2
7	3	7	5	1	0	8	6	3
4	3	6	5	0	0	7	2	1
5	2	5	4	1	0	4	0	1
8	5	6	4	6	5	4	7	4
8	3	8	4	3	1	3	1	1
4	3	4	2	4	0	3	6	1
8	6	7	4	0	0	6	5	4
9	3	12	4	0	0	3	4	2
6	3	5	3	0	0	3	2	3
3	4	5	3	0	0	3	2	0
4	3	5	3	0	1	2	1	7
4	7	6	4	2	0	4	2	3
4	4	5	6	1	3	5	3	4
4	3	4	3	0	1	2	1	4
5	4	4	3	2	0	5	2	3
7	2	4	3	0	1	4	1	1
3	3	1	4	2	2	2	1	0
9	1	7	4	0	0	5	2	0
8	3	6	5	0	0	5	0	0
4	2	6	2	0	0	1	0	4
4	2	3	2	0	1	3	3	1
7	2	4	4	0	0	3	1	1
5	2	6	4	0	0	4	4	0
3	2	1	3	0	0	5	1	1
4	2	4	2	0	0	3	0	0
0	8	8	4	3	0	3	2	0
4	2	9	1	0	0	3	1	0
3	1	5	2	1	0	2	1	0
2	1	0	5	0	0	3	0	0

3	2	6	1	0	0	1	0	0
4	1	4	1	0	0	2	0	1
8	3	5	8	0	0	7	8	5
3	1	5	1	0	0	5	2	5
3	1	3	2	0	0	3	1	1
2	2	2	2	1	0	2	1	2
1	5	2	1	0	0	0	1	1
1	1	3	3	0	0	1	0	0
4	4	4	2	0	0	3	0	2
3	1	1	3	0	0	1	0	1
3	3	2	1	0	0	3	0	1
2	2	2	2	0	0	3	0	1
1	2	1	2	0	0	3	1	2
11	6	7	7	3	0	11	10	6
1	0	1	0	0	0	2	1	1
4	2	1	3	0	0	2	1	0
3	3	2	1	0	0	2	1	2
0	1	0	1	0	0	4	0	1
3	1	3	0	0	0	2	1	0
2	1	2	0	0	0	3	0	0
2	3	6	3	0	0	2	0	2
1	2	1	1	0	0	2	0	0
0	2	3	1	0	0	1	0	1
3	1	1	1	4	0	2	4	4
2	1	1	0	0	0	2	1	0
5	3	5	5	4	4	4	5	2
0	1	2	0	0	0	0	0	0
0	1	3	0	0	0	0	0	0
1	0	2	0	0	0	0	0	2
0	0	0	0	0	0	1	0	0
0	0	0	0	0	0	0	0	0
0	0	0	0	0	0	1	0	0
0	0	0	0	0	0	0	0	0
22	10	25	10	7	4	12	5	9
10	6	8	7	1	1	13	4	5
12	7	10	6	2	1	8	1	4
4	3	7	5	0	0	4	4	3
4	0	2	0	0	0	5	2	0
4	8	12	9	0	0	2	1	2
5	6	10	4	0	0	4	1	1
4	0	5	0	0	0	3	0	9
4	3	6	5	1	4	2	7	1
4	4	5	4	0	0	3	1	1
3	0	2	4	0	1	4	2	0
6	4	5	3	1	0	5	1	3
4	4	5	3	0	1	4	0	3
3	2	4	2	1	2	3	2	2

6	4	8	4	3	1	4	4	2
3	3	5	2	1	0	3	2	2
6	2	7	2	0	0	7	1	0
3	6	3	3	0	0	2	3	3
5	1	6	3	0	0	2	1	1
2	2	5	3	1	0	2	1	2
3	2	6	0	0	1	1	2	1
5	1	4	2	0	0	2	0	8
5	3	4	4	0	0	7	4	1
4	5	6	2	0	0	1	0	5
3	3	4	1	0	0	1	0	2
2	3	2	6	0	0	3	1	0
5	4	1	1	0	0	1	1	2
5	3	5	2	0	0	3	1	0
0	1	5	2	0	0	2	0	1
4	5	4	1	0	0	1	1	5
4	3	8	2	0	0	5	1	1
2	3	2	0	1	0	3	0	13
3	0	1	0	1	0	2	2	1
2	1	1	1	1	0	1	1	3
2	2	3	3	0	0	3	0	2
4	3	2	0	0	0	0	0	0
2	2	3	3	1	0	3	2	1
4	0	3	3	0	0	2	0	0
4	1	3	1	0	0	3	0	0
2	0	1	1	0	0	0	1	0
1	2	4	3	1	0	1	2	5
2	0	3	1	0	0	1	1	1
5	0	5	1	0	0	1	0	1
4	1	3	2	1	0	2	0	1
1	2	4	3	0	0	2	1	1
2	2	1	2	0	0	2	0	0
6	2	4	3	0	0	2	1	0
3	2	5	2	0	0	2	1	4
3	1	3	0	0	0	1	0	0
1	0	0	0	0	0	0	0	0
1	1	4	1	0	0	1	0	0
5	1	3	2	0	0	1	3	0
2	1	3	3	0	0	1	0	3
3	1	3	1	0	0	0	2	1
3	1	0	0	0	0	0	1	0
2	2	1	3	0	0	2	0	2
2	0	2	3	0	0	2	0	0
3	0	2	1	0	0	2	0	0
1	2	1	2	4	2	1	2	1
1	0	3	0	0	0	1	1	1
3	1	2	2	0	0	1	0	2

1	1	2	2	0	0	0	0	2
2	2	3	1	0	1	1	0	2
2	2	3	1	0	0	2	1	1
1	1	0	0	0	0	0	0	1
0	0	3	2	0	0	2	0	1
1	1	3	2	0	0	2	0	2
1	2	4	0	0	0	0	0	0
2	0	3	1	0	0	1	0	0
2	1	3	0	0	0	1	0	2
2	2	2	1	0	0	2	0	2
1	2	0	1	0	0	1	0	0
2	1	1	1	0	0	1	1	2
1	2	3	0	0	0	1	1	3
1	0	0	0	0	0	1	0	0
2	1	1	2	0	0	2	0	1
1	2	5	1	0	0	1	1	0
5	2	1	1	0	0	3	0	0
1	1	3	1	0	0	1	0	0
4	1	2	1	0	0	1	0	4
2	1	2	2	0	0	2	1	4
3	0	4	1	0	0	0	0	0
2	1	3	0	0	0	1	0	0
0	1	0	0	0	0	2	1	1
1	0	1	0	0	0	0	0	0
0	0	1	1	0	0	3	2	1
1	0	2	1	0	0	1	0	0
0	1	1	0	0	0	0	0	0
1	0	0	0	0	0	1	1	0
1	0	1	2	1	0	0	0	0
4	3	4	1	0	0	1	0	1
2	2	0	2	0	0	1	1	2
3	2	2	2	0	0	3	0	0
1	1	4	0	0	0	2	0	2
1	0	1	0	0	0	0	0	0
2	1	1	0	0	0	1	0	1
1	3	1	0	0	0	0	0	0
0	2	1	0	0	0	0	1	2
1	0	2	1	0	0	0	0	0
3	0	2	3	0	0	0	0	2
1	1	0	1	0	0	2	1	0
0	0	0	1	0	0	1	0	1
1	1	2	0	0	0	0	0	1
0	1	1	1	0	0	0	1	0
0	0	0	0	0	0	0	0	0
2	0	5	0	0	0	1	0	0
2	0	0	0	0	0	2	0	1
1	1	0	3	0	0	1	0	0

1	0	1	0	0	0	0	0	0
0	0	0	0	0	0	0	0	0
0	1	0	0	0	0	1	0	0
11	2	9	3	1	0	5	0	1
9	5	10	3	0	0	6	2	6
13	4	6	5	0	0	5	2	2
6	9	6	4	4	1	6	4	6
8	2	5	4	1	2	7	4	2
10	0	11	3	0	0	2	0	0
1	7	5	3	1	8	3	0	0
7	1	6	1	1	0	3	0	1
0	3	24	13	0	0	2	3	0
5	4	7	0	2	0	6	0	0
6	6	7	3	0	0	5	1	2
2	6	5	3	3	0	4	1	2
4	3	3	2	1	2	3	4	3
4	4	4	1	0	1	2	2	2
7	2	5	4	0	0	5	1	1
6	3	3	2	0	0	3	1	2
5	4	3	4	0	0	3	0	1
5	0	1	0	1	0	2	0	0
2	3	3	2	0	0	1	1	0
2	1	3	3	0	0	0	0	0
3	1	4	1	0	0	1	2	1
4	2	5	3	1	0	4	1	2
2	2	3	2	0	0	1	0	1
3	4	4	0	0	3	2	1	2
3	1	3	3	0	0	3	0	1
5	5	14	5	1	0	7	0	1
4	2	4	2	0	0	1	0	0
2	3	3	3	2	0	3	0	1
3	1	3	1	0	0	1	0	2
1	2	2	2	0	0	2	0	0
0	0	0	0	0	0	0	0	0
5	4	2	1	0	0	0	0	0
2	3	2	2	0	0	3	0	4
0	1	3	1	0	0	2	0	1
6	2	3	3	0	0	5	0	0
5	4	1	1	1	0	4	0	0
2	1	2	2	1	0	2	1	2
7	3	5	0	0	0	2	1	1
0	1	3	1	0	0	1	0	0
2	0	2	2	0	0	2	0	0
3	1	2	1	0	0	2	0	0
7	2	4	4	0	0	3	1	2
3	1	0	0	0	2	1	0	0
4	1	5	1	0	0	1	0	0

0	5	7	4	1	0	2	1	0
4	2	4	1	0	0	3	0	1
1	2	1	1	0	0	0	1	2
5	3	4	3	0	0	0	0	3
2	2	1	3	0	0	3	1	0
1	0	1	1	0	0	0	1	1
4	0	3	2	0	0	3	0	1
4	4	6	1	0	0	2	1	0
2	0	1	2	0	0	3	1	1
4	3	1	2	0	0	1	1	2
4	1	3	2	0	0	4	0	0
2	2	3	1	0	0	4	0	0
2	1	3	0	1	0	2	0	1
3	1	2	0	0	0	1	0	0
3	2	2	2	0	0	2	1	0
3	2	3	2	0	0	1	0	0
2	2	2	4	0	0	2	1	0
1	1	2	1	0	0	2	1	1
2	0	2	3	0	0	0	1	2
2	1	3	2	0	0	1	0	1
2	2	1	0	0	0	1	1	1
2	1	2	0	0	1	1	0	1
2	1	4	2	0	0	1	0	1
3	1	1	0	0	0	1	1	2
3	2	2	1	0	1	5	0	0
3	1	1	1	0	0	1	0	1
0	0	1	0	0	0	0	1	0
2	1	2	1	0	0	0	0	2
3	0	4	1	0	0	1	1	3
0	1	3	0	0	0	0	0	1
0	1	3	1	0	0	1	0	0
2	2	2	1	0	1	0	0	1
1	1	1	2	0	0	1	0	1
2	0	1	3	1	0	2	0	1
0	0	0	0	0	0	0	1	0
1	1	1	1	0	0	2	0	1
1	2	1	1	0	0	1	0	1
1	2	0	2	0	0	0	1	1
0	0	2	0	0	0	1	1	1
2	0	0	0	0	0	3	0	0
1	0	0	0	0	0	2	0	0
1	0	1	1	0	8	1	0	0
6	4	3	0	0	0	0	0	0
1	1	2	1	0	0	1	1	0
2	0	0	0	0	0	2	0	0
2	0	1	0	0	0	1	0	0
1	0	3	1	0	0	0	0	0

2	1	1	1	0	0	1	0	1
1	3	1	1	0	0	1	0	2
1	1	0	1	0	0	1	0	1
1	0	2	0	0	0	1	0	0
1	0	4	0	0	0	1	0	0
1	1	1	1	0	0	2	1	1
1	1	2	1	0	0	1	0	2
0	1	1	0	0	0	0	1	0
0	1	0	1	0	0	1	1	0
2	1	1	1	0	0	2	0	0
3	4	2	1	0	0	1	0	2
1	1	1	1	0	0	1	0	1
0	2	2	2	0	0	1	0	0
2	1	1	1	0	0	1	0	1
1	1	1	1	1	0	1	0	1
2	0	1	0	0	0	2	0	0
1	0	2	1	0	0	0	0	1
1	0	1	1	0	0	2	0	1
2	0	0	0	0	0	1	1	0
1	0	0	2	0	0	0	0	0
0	0	0	1	0	0	0	0	0
0	0	0	0	0	0	0	1	0
1	2	1	1	0	0	2	0	1
1	2	1	0	0	0	2	0	2
0	1	1	1	0	0	0	0	0
2	0	1	0	0	0	0	0	2
1	0	3	0	0	0	0	0	0
1	0	1	0	0	0	0	0	0
1	2	3	1	0	0	1	0	1
2	0	3	0	0	0	0	2	1
2	0	1	0	0	0	1	1	0
1	0	2	2	0	0	2	0	0
0	0	2	1	0	0	1	1	0
0	0	1	0	0	1	1	1	0
0	0	0	1	0	0	0	0	4
2	2	2	0	0	0	0	0	0
0	0	0	0	0	0	0	0	0
1	0	0	1	0	1	1	1	1
0	1	1	1	0	0	0	0	1
1	0	2	0	0	0	2	0	0
0	1	3	0	0	0	0	0	0
0	0	1	1	0	0	1	0	0
1	1	2	0	0	0	1	0	4
0	0	1	0	0	0	1	1	0
2	0	1	2	0	0	1	0	0
0	1	2	0	0	0	1	1	0
0	2	2	0	0	0	0	0	1

1	2	1	0	0	0	1	0	0
1	0	0	0	0	0	1	0	2
1	2	2	1	0	0	1	0	1
1	1	2	1	0	0	0	0	1
1	0	2	2	0	0	1	0	0
0	0	0	1	0	0	0	1	0
2	1	1	1	0	0	0	0	0
0	0	0	1	0	0	1	0	0
0	1	0	0	0	0	1	0	4
0	0	1	1	0	0	1	0	0
3	0	1	2	0	0	2	0	0
1	0	0	0	0	0	0	0	0
1	1	0	1	0	0	2	0	2
1	0	1	1	0	0	0	0	1
0	0	1	0	0	0	1	0	1
1	1	1	0	0	0	2	1	1
0	0	2	1	0	0	1	0	1
0	1	2	0	0	0	0	0	1
2	1	0	0	0	0	0	1	0
0	1	0	0	0	0	2	0	1
1	1	1	1	0	0	1	0	0
2	1	1	2	0	0	1	0	0
1	0	1	0	0	0	0	1	0
0	0	1	0	0	0	0	0	0
0	1	1	0	0	0	0	0	0
2	0	4	0	0	0	0	0	0
0	0	0	0	0	0	0	1	0
0	1	0	1	0	0	0	0	4
0	1	1	0	0	0	0	1	3
1	0	1	0	0	0	0	0	1
2	1	2	1	0	0	2	1	0
1	1	1	1	0	0	0	0	0
0	0	1	1	0	0	0	0	0
1	1	0	1	0	0	0	0	0
1	0	1	2	0	0	0	0	3
2	0	2	0	0	0	1	1	0
1	0	1	0	0	0	1	0	0
1	1	0	0	0	0	0	0	0
0	0	0	0	0	0	1	0	0
0	0	1	0	0	0	0	0	0
1	0	0	0	0	0	0	0	0
1	0	0	0	0	0	1	0	0
1	0	0	0	0	0	2	0	0
1	2	1	1	0	0	0	0	1
0	1	2	0	0	0	0	0	0
2	0	2	0	0	0	0	0	0
2	0	2	0	0	0	0	0	0

0	0	0	0	0	0	0	0	3
0	0	0	0	0	0	0	0	0
1	1	0	1	0	0	1	0	0
0	1	0	0	1	0	0	1	0
0	0	0	0	0	0	0	0	0
0	0	1	2	0	0	1	0	1
1	0	2	0	0	0	1	0	0
0	0	0	0	0	0	1	0	0
1	0	0	0	0	0	0	0	0
0	0	2	0	0	0	1	0	0
0	0	1	1	0	0	0	0	0
0	0	2	0	0	0	0	0	0
0	0	0	1	0	0	0	0	0
0	0	1	0	0	0	2	0	0
0	0	0	0	0	0	2	0	1
0	1	0	0	0	0	2	0	0
0	0	2	0	0	0	0	0	0
0	0	1	0	0	0	0	0	1
0	0	0	0	0	0	0	0	0
1	0	0	2	0	0	0	0	0
0	0	0	0	0	0	0	0	0
0	0	0	0	0	0	0	0	0
0	0	0	0	0	0	0	0	0
0	22	0	0	0	3	0	1	44
14	2	12	2	0	0	7	3	1
3	20	3	0	0	13	0	8	14
8	4	7	4	0	0	4	4	2
15	0	6	0	0	0	1	0	0
1	1	0	2	1	0	2	1	1
2	0	4	1	0	0	4	1	1
0	0	0	0	0	9	0	0	1
8	2	10	0	0	0	0	0	0
0	6	12	5	1	0	7	0	0
0	8	0	0	0	6	0	1	1
1	4	6	7	1	0	3	0	0
0	31	0	0	0	14	0	11	0
4	1	5	1	0	0	3	1	0
6	3	9	5	0	0	1	1	0
4	1	6	3	0	0	3	0	0
8	0	7	2	0	0	3	0	0
5	2	7	0	0	0	2	0	0
6	0	7	3	0	0	4	2	3
1	2	6	0	1	0	0	0	1
5	3	6	2	0	0	2	0	0
6	1	3	2	0	0	1	1	0
2	2	4	5	3	0	9	1	0
1	12	0	0	0	2	0	2	2

3	2	4	4	0	0	1	1	2
3	2	0	2	1	0	1	1	2
3	0	7	3	0	0	4	1	0
5	1	7	3	0	0	2	0	1
4	2	5	2	2	1	2	2	1
3	3	3	2	0	0	1	3	1
0	5	0	0	1	8	0	0	1
0	0	0	0	0	8	0	0	18
2	3	2	3	0	1	1	2	1
2	0	3	1	0	0	3	0	1
1	2	1	1	1	1	1	1	6
0	0	4	0	0	0	0	0	0
3	2	6	4	2	1	1	1	4
1	3	8	8	1	0	5	1	0
3	0	1	0	0	0	0	0	0
0	9	0	1	0	0	0	0	27
0	1	0	0	0	14	0	0	3
3	1	2	2	0	4	4	1	7
0	4	1	3	4	0	3	3	0
0	4	3	12	0	0	1	5	0
4	0	3	0	0	0	0	0	0
3	1	3	3	2	0	2	16	0
5	0	5	0	0	2	0	0	0
1	0	1	0	0	0	2	0	1
2	0	2	1	0	0	1	0	4
5	3	2	1	0	0	1	0	0
3	3	3	3	0	0	2	2	2
1	2	2	0	0	0	2	0	0
0	0	2	0	0	0	0	0	0
1	4	2	0	0	0	0	0	2
0	18	0	0	0	21	0	0	3
0	8	0	0	0	5	0	0	0
0	0	3	1	0	0	0	0	0
2	2	2	0	0	0	1	0	5
2	1	3	1	0	0	2	0	1
2	1	2	0	0	0	0	0	0
8	0	1	0	0	0	2	0	0
0	1	3	0	0	0	1	1	0
0	1	0	0	0	4	0	0	0
0	0	1	0	0	0	2	1	0
1	1	0	1	0	0	0	17	0
2	2	1	2	0	0	1	1	1
0	1	1	6	2	2	3	2	0
1	0	1	0	0	0	1	0	0
2	1	2	0	0	0	2	0	1
1	1	3	0	0	0	1	0	1
2	3	3	0	0	0	1	1	0

3	2	3	3	0	0	1	0	0
7	0	1	1	0	0	2	0	0
1	2	1	1	0	0	0	0	1
0	0	4	0	2	0	1	0	0
2	1	1	2	0	0	1	0	0
1	1	2	0	0	0	0	1	1
0	0	5	0	0	0	0	0	0
0	18	0	0	2	9	0	0	0
3	0	1	1	0	0	1	0	0
0	1	2	0	0	0	1	1	0
2	0	4	1	0	0	1	0	0
0	2	2	1	0	0	0	2	2
9	0	6	0	0	0	0	1	0
0	0	0	0	0	0	0	0	0
1	0	1	2	0	0	0	0	0
0	2	5	1	0	0	0	0	3
0	3	0	0	0	2	0	0	0
3	1	3	1	0	0	1	0	1
1	0	2	2	0	0	2	1	0
3	0	1	0	1	0	2	0	0
0	1	5	0	0	0	1	0	0
0	0	0	0	0	0	0	0	0
0	0	1	0	0	0	0	0	0
0	0	0	0	0	0	0	0	1
1	1	2	1	0	0	0	0	6
1	0	2	0	0	0	0	0	1
0	0	2	1	0	0	0	0	0
4	0	1	0	0	0	1	0	0
1	0	2	0	0	0	0	0	0
1	0	1	2	0	0	1	0	0
0	0	0	0	0	0	0	0	25
1	1	2	1	0	0	0	0	0
1	0	2	1	0	0	0	0	0
1	0	3	0	0	0	0	0	0
0	1	0	0	0	1	0	0	7
2	1	1	0	0	0	1	1	1
1	3	3	0	0	0	1	0	0
15	11	14	0	0	0	0	0	19
2	0	1	1	0	0	1	0	1
3	0	1	2	0	0	2	0	0
0	2	4	0	0	0	0	1	5
0	2	3	1	0	0	1	0	1
6	0	11	5	0	0	2	0	2
4	0	0	0	0	0	0	0	0
0	1	0	0	0	2	0	0	0
0	0	0	0	0	7	0	0	0
3	1	1	0	0	0	2	0	0

3	0	2	1	0	0	0	0	0
3	1	2	0	0	0	0	1	0
0	0	0	0	0	0	0	1	1
2	1	1	1	0	0	1	0	1
2	0	3	2	0	0	4	0	2
0	2	1	0	0	2	0	0	0
0	1	2	1	0	0	0	0	0
0	0	2	0	0	0	0	1	0
1	1	2	2	0	0	5	1	2
1	1	1	0	0	0	1	0	1
2	1	1	2	0	0	1	0	1
3	0	2	1	0	0	0	1	0
2	1	1	0	0	0	0	0	0
0	0	0	0	0	0	0	0	0
0	0	10	12	0	0	0	0	0
2	0	0	0	0	0	2	0	0
0	13	0	8	0	0	0	0	0
5	0	0	0	0	0	1	0	0
0	0	0	0	0	11	0	0	0
3	0	1	2	0	0	3	1	3
2	0	0	1	0	0	1	0	1
1	6	0	0	0	3	1	1	2
2	1	2	1	0	0	0	0	0
0	0	0	3	0	1	1	1	0
2	0	4	1	0	0	2	0	0
3	0	1	0	0	0	1	0	0
2	0	0	0	0	0	3	0	0
2	1	2	0	0	0	0	0	0
6	0	0	0	0	0	0	0	0
2	0	0	0	0	0	2	0	0
1	0	0	1	0	0	0	0	0
3	0	1	0	0	0	0	0	0
1	0	0	1	0	0	0	0	0
0	1	0	2	5	0	0	1	0
0	0	3	0	0	0	0	0	0
0	8	0	0	0	0	0	0	0
0	22	0	0	0	0	0	0	0
0	0	0	0	0	0	0	0	0
3	0	3	0	0	0	0	0	0
0	2	1	1	0	0	1	1	2
0	1	1	0	0	0	0	0	0
2	1	0	0	0	0	1	0	0
4	0	2	1	0	0	0	0	0
1	0	0	0	0	7	0	0	0
0	0	0	0	0	0	0	0	0
0	1	1	3	0	0	1	0	0
0	2	1	0	0	0	0	0	0

0	0	2	1	0	0	0	0	0
1	0	1	1	0	0	0	0	0
2	0	1	0	0	0	1	0	0
0	0	0	0	0	0	1	0	0
1	0	1	1	0	0	0	0	0
5	5	3	5	0	0	4	0	2
9	7	7	6	0	0	0	0	4
3	1	2	0	0	0	0	0	0
7	0	3	0	0	0	0	1	0
0	0	3	0	0	0	1	0	1
2	1	1	0	0	0	0	0	1
1	1	2	0	0	0	1	0	2
1	1	1	1	0	0	1	0	2
2	0	2	2	0	0	1	0	1
0	0	1	0	0	0	0	0	0
0	3	1	0	0	2	1	0	0
3	1	4	0	0	0	0	0	0
1	0	0	0	0	0	0	0	0
0	0	0	0	0	0	1	0	0
0	1	0	0	0	0	0	0	11
0	3	0	0	0	2	0	0	0
2	1	1	0	0	0	1	0	1
0	2	5	0	0	0	0	0	0
0	1	1	0	0	0	0	0	0
0	2	1	0	0	0	0	0	0
1	0	2	1	0	0	0	0	0
0	3	1	2	0	0	0	0	0
0	1	2	0	0	0	1	0	0
0	0	6	0	0	0	2	0	3
1	0	1	0	0	0	0	2	0
1	0	1	1	1	0	1	0	0
0	2	0	0	0	0	0	2	0
0	0	1	1	0	0	1	0	0
1	0	1	1	0	0	1	0	1
3	0	3	0	0	0	2	0	0
5	51	0	0	0	31	0	0	0
0	3	3	3	0	0	2	0	0
2	1	5	0	0	0	0	0	0
0	0	0	0	0	0	0	0	0
0	7	5	5	0	0	0	0	0
3	0	0	0	0	0	0	0	0
0	13	0	0	0	14	0	0	0
1	0	0	1	0	0	2	0	0
0	0	2	1	0	0	1	0	0
1	0	2	1	0	0	0	0	2
1	0	2	1	0	0	0	0	0
3	4	3	1	0	0	0	0	0

1	0	0	0	0	0	0	0	0
2	0	0	0	0	0	0	0	0
0	7	0	0	0	0	0	0	13
0	0	1	0	1	0	0	1	0
0	7	9	9	0	0	10	0	7
2	1	1	0	0	0	0	0	0
4	1	4	1	0	0	5	0	0
0	1	1	0	0	0	3	0	0
1	0	2	0	0	0	1	0	0
1	0	2	0	0	0	0	0	0
2	2	3	1	0	0	0	0	0
1	0	0	0	0	0	1	0	0
1	0	0	2	0	0	0	0	0
1	0	0	0	0	0	0	0	0
1	0	0	2	0	0	1	1	0
1	0	1	0	0	0	0	0	0
0	0	1	0	0	0	0	0	2
2	0	0	0	0	0	0	0	0
1	1	3	0	0	0	0	0	0
0	0	0	0	0	0	0	0	0
0	1	1	0	0	0	0	0	1
1	0	0	0	0	0	0	0	0
1	0	0	0	0	0	0	0	1
3	2	3	0	0	0	1	0	0
0	1	1	0	0	0	0	0	0
1	0	1	2	0	0	2	0	0
1	0	1	1	0	0	0	0	5
0	0	0	0	0	0	0	0	0
0	0	4	0	0	0	1	0	0
0	0	2	0	0	0	0	0	0
0	0	1	0	0	0	0	0	0
1	0	0	0	0	0	0	0	0
0	1	0	0	0	0	0	0	0
0	0	2	0	0	0	0	0	0
1	0	0	0	0	0	0	0	2
2	1	2	1	0	0	0	0	0
1	0	1	1	0	0	0	0	0
3	1	2	2	0	0	2	0	1
1	0	0	0	0	0	0	0	0
1	1	4	0	0	0	0	0	0
1	0	2	0	0	0	0	0	0
0	0	0	1	0	0	0	0	0
1	0	0	1	0	0	0	0	0
4	0	1	0	0	0	0	0	0
0	0	0	0	0	0	0	0	1
0	0	1	0	0	0	1	0	0
0	0	0	0	0	0	0	1	0

1	1	1	2	0	0	0	0	1
3	0	1	0	0	0	1	0	1
0	0	3	1	0	0	1	0	0
1	0	1	0	0	0	0	0	0
1	0	2	0	0	0	0	0	0
0	0	3	0	0	0	0	0	1
0	0	0	0	0	0	0	0	0
0	2	2	0	0	0	0	0	3
0	0	1	0	0	2	0	0	0
1	0	0	0	0	0	1	0	0
0	1	0	0	0	1	0	0	0
0	10	0	0	0	0	0	0	3
1	0	1	0	0	0	1	0	0
2	1	1	1	0	0	1	0	0
1	0	0	0	0	0	0	0	0
0	1	1	0	0	0	1	0	0
3	1	0	0	0	0	2	1	0
2	0	0	0	0	0	1	0	0
1	0	1	0	0	0	0	0	0
3	0	1	3	0	0	0	0	0
1	1	1	1	0	0	0	0	1
0	0	0	1	0	0	0	0	0
0	0	1	1	0	0	0	0	0
0	0	1	2	0	0	0	0	0
0	0	0	0	0	0	0	0	0
0	0	3	0	0	0	0	0	0
0	0	3	3	0	0	0	0	0
0	0	0	1	0	0	2	1	0
0	0	0	0	0	0	0	0	0
0	0	1	0	0	0	0	0	0
0	0	0	0	0	0	0	0	0
0	0	3	0	0	0	0	0	0
0	1	0	0	0	0	0	0	0
0	0	0	0	0	0	0	0	0
1	0	0	0	0	0	0	0	0
1	0	0	1	0	0	1	1	0
0	0	0	0	0	0	0	0	0
1	1	2	1	0	0	1	0	0
1	0	1	0	0	0	0	0	0
2	0	1	1	0	0	1	0	0
2	0	0	0	0	0	0	1	0
0	0	1	1	0	0	1	0	0
0	0	0	0	0	0	0	0	0
0	2	0	0	0	2	0	0	0
1	0	0	0	0	0	3	0	2
1	0	2	0	0	0	0	0	0
2	0	1	0	0	0	0	0	0

1	0	2	0	0	0	0	0	0
0	0	0	0	0	0	0	0	0
3	0	3	0	0	0	0	0	0
0	0	1	0	0	0	0	0	0
0	1	0	0	0	0	0	0	0
0	0	0	0	0	0	0	0	0
0	0	0	0	0	0	1	0	0
0	0	0	0	0	0	0	0	0
0	0	0	0	0	0	0	0	2
0	0	0	0	0	0	0	0	0
0	0	0	0	0	0	0	2	0
0	0	0	0	0	0	0	0	0
0	1	0	2	0	0	1	1	0
0	0	1	1	0	0	1	0	0
1	0	1	0	0	0	0	0	0
2	0	0	0	0	0	0	0	0
1	0	0	0	0	0	2	0	0
0	0	0	0	0	0	0	0	0
0	0	1	0	0	0	0	0	0
0	0	0	0	0	0	0	0	2
0	0	0	0	0	0	0	0	0
2	0	0	0	0	0	0	0	0
0	1	0	0	0	0	0	0	0
0	0	0	0	0	0	0	0	12
0	1	1	0	0	0	0	0	0
0	1	0	0	0	0	2	0	1
0	0	3	0	0	0	0	0	0
0	0	0	0	0	0	0	0	0
1	0	2	1	0	0	0	0	0
0	0	0	2	0	0	1	0	1
0	0	0	1	0	0	0	1	2
0	1	1	0	0	0	0	0	0
6	32	8	3	0	0	0	9	0
0	0	0	0	0	0	2	0	0
0	0	0	0	0	0	0	0	0
0	1	0	0	0	0	0	0	0
2	0	0	0	0	0	1	0	0
1	0	1	0	0	0	0	0	0
0	0	0	0	0	3	0	0	0
3	0	1	0	0	0	0	0	0
0	0	0	0	0	0	0	0	0
0	0	0	0	0	0	0	0	8
0	1	1	0	0	0	0	0	0
1	0	2	0	0	0	0	0	1
0	0	1	0	0	0	0	0	0
0	0	1	0	0	0	0	0	1
0	0	2	0	0	0	0	0	0

0	0	2	0	0	0	0	0	0
2	0	0	0	0	0	0	0	0
0	0	0	0	0	0	0	0	1
0	0	0	1	0	0	0	0	0
0	0	0	0	0	0	0	0	0
1	0	2	0	0	0	0	0	0
0	0	3	0	2	0	0	0	0
0	1	0	0	0	0	0	0	2
0	0	0	0	0	0	0	0	3
2	1	0	0	0	0	0	0	0
0	0	0	0	0	0	0	0	0
1	0	2	1	0	0	1	0	0
0	3	0	0	0	0	0	0	0
0	1	0	2	0	0	0	0	0
0	0	2	0	0	0	0	0	0
0	1	1	0	0	0	0	0	0
0	0	0	0	0	0	0	0	2
0	0	1	0	0	0	0	0	0
2	0	2	0	0	0	1	0	0
0	0	0	0	0	0	0	0	0
0	0	0	0	0	0	0	0	0
0	0	0	0	0	0	0	0	0
1	0	2	0	0	0	0	0	0
0	0	0	1	0	0	0	0	0
0	0	0	0	0	0	0	0	0
0	0	0	0	0	0	0	0	0
0	0	1	0	0	0	0	0	0
0	0	0	0	0	0	2	0	0
0	0	2	0	0	0	0	0	0
0	0	0	0	0	0	0	0	0
0	0	0	0	0	0	0	0	0
0	0	0	0	0	0	0	0	0
0	0	0	0	0	0	0	0	3
0	0	0	0	0	0	0	0	6
0	0	0	0	0	0	0	0	11
0	1	1	0	0	0	0	0	0
0	0	0	0	0	31	0	0	0
0	0	0	0	0	0	0	0	0
0	0	1	0	0	0	0	0	2
0	0	1	0	0	0	0	0	0
0	0	1	0	0	0	0	0	1
0	0	0	2	0	0	0	0	0
0	0	2	1	0	0	0	0	0
1	0	0	0	0	0	0	0	2
0	1	0	0	0	0	0	0	0
1	0	0	0	0	0	0	0	2
0	4	0	0	0	0	0	0	8

0	0	0	0	0	0	0	0	0
0	0	0	0	0	0	0	0	0
0	0	0	0	0	0	0	0	1
0	1	2	0	0	0	0	0	0
1	0	3	0	0	0	0	0	0
0	0	0	0	0	1	0	0	0
0	0	0	0	0	0	0	2	0
0	0	0	0	0	0	0	0	5
0	0	0	0	0	0	0	0	0
0	4	0	0	0	0	0	0	0
0	0	5	0	0	0	0	0	0
0	0	0	0	0	0	0	7	0
0	0	0	0	0	0	2	0	0
0	0	0	0	0	0	0	0	0
0	0	0	0	0	0	0	0	0
0	0	1	0	0	0	0	0	0
0	0	0	0	0	0	0	0	0
0	0	0	0	0	0	7	6	0
0	1	0	0	0	0	0	0	0
0	0	0	0	0	0	0	0	0
0	1	1	0	0	0	0	0	2
0	0	1	0	0	0	0	0	0
0	1	0	0	0	0	0	0	0
0	0	0	0	0	0	0	0	0
0	0	1	0	0	0	0	0	3
0	0	0	0	0	0	0	0	0
0	0	0	2	0	0	0	0	0
0	0	0	0	0	0	0	0	0
0	0	0	0	0	0	0	0	0
0	0	0	0	0	0	0	0	0
0	0	0	0	0	0	0	0	2
0	0	0	0	0	0	0	0	0
0	0	0	0	0	0	0	0	0
0	0	0	0	0	0	0	0	0
0	0	3	0	0	0	0	0	0
0	0	0	0	0	0	0	0	0
0	0	2	0	0	0	0	0	0
0	0	0	0	0	0	0	0	0
0	0	1	0	0	0	0	0	0
0	0	2	0	0	0	0	0	0
0	1	2	0	0	0	0	0	0
0	0	1	0	0	0	0	0	0
0	0	1	0	0	0	0	0	0
2	0	0	0	0	0	0	0	0
0	0	0	0	0	0	0	0	0
0	1	0	0	0	0	0	0	0
0	0	0	0	0	0	0	0	0

0	1	0	0	0	0	0	0	23
0	0	0	0	0	0	1	0	0
0	0	0	0	0	0	2	0	5
0	0	0	0	0	0	0	0	2
0	0	0	0	0	0	0	0	0
0	0	0	0	0	0	0	0	20
2	0	1	0	0	0	0	0	1
0	0	0	0	0	0	1	0	0
0	0	2	0	0	0	0	0	0
0	0	0	0	0	0	0	0	2
0	0	0	0	0	0	0	0	0
0	0	0	0	0	0	0	0	2
0	0	0	0	0	0	0	0	0
0	0	0	0	0	0	0	0	0
0	0	1	0	0	0	0	0	0
0	0	0	0	0	0	0	0	0
0	0	0	0	0	0	0	0	0
0	1	0	0	0	0	0	0	0
0	0	4	0	0	0	0	0	0
0	0	0	0	0	0	0	0	0
0	0	2	0	0	0	0	0	0
0	0	0	0	0	0	0	0	0
0	0	0	0	0	0	0	0	0
0	0	0	0	0	0	0	0	0
0	0	0	0	0	0	0	0	0
0	0	2	0	0	0	0	0	0
0	0	0	0	0	0	0	0	0
0	0	0	0	0	0	0	0	3
0	0	0	0	0	0	0	0	9
0	0	2	0	0	0	0	0	0
0	0	0	0	0	0	0	0	0
0	0	0	0	0	0	0	0	2
0	0	0	0	0	0	0	0	2

Total Spect	Total Spect	Total Spectrum Count
ATN	ATN	ATN
ATN, other	ATN, other	ATN, other causes
125	122	23
67	92	33
62	24	10
39	33	9
80	82	25
64	68	34
62	44	11
47	31	2
34	35	15
80	33	8
84	45	22
41	45	0
31	9	9
35	12	4
52	50	21
40	35	15
23	5	19
54	46	28
34	23	6
25	25	10
61	15	11
29	9	9
41	37	14
16	12	4
16	19	2
12	0	10
31	18	9
21	20	2
22	22	11
31	17	9
10	7	2
22	19	6
19	12	0
11	11	2
24	15	9
24	17	4
28	18	5
25	15	9
0	0	0
0	19	12
17	10	4
13	18	12
12	11	2

19	16	14
22	11	6
6	6	0
21	37	13
24	17	4
12	14	1
12	0	4
18	10	4
21	10	2
30	17	4
12	7	2
10	7	1
22	0	0
7	2	6
18	9	5
21	13	7
8	6	3
15	8	7
11	14	4
12	5	1
3	6	9
14	0	0
8	14	5
20	11	3
11	5	3
10	10	3
7	7	5
11	9	2
7	9	2
7	5	7
8	9	5
12	4	2
5	1	2
15	14	0
11	11	2
9	0	0
20	11	2
15	9	1
14	7	0
11	8	2
12	12	2
7	3	2
14	10	0
1	1	0
11	9	0
17	9	2
8	5	1

8	10	2
7	9	4
10	4	3
9	9	0
9	5	0
5	5	0
8	3	0
6	1	1
14	8	0
13	4	0
19	17	9
15	12	3
18	5	0
15	7	10
6	1	1
12	2	0
7	9	2
10	1	0
8	3	3
9	5	0
0	0	0
10	2	0
5	5	0
3	0	0
15	18	4
8	17	0
10	16	6
13	2	0
11	2	0
7	9	0
13	11	4
5	1	1
6	8	2
2	3	1
5	6	0
7	11	0
5	2	0
8	4	0
0	2	0
12	13	2
16	11	0
11	12	3
13	5	0
11	12	7
6	6	4
6	5	0
7	7	2

7	4	0
4	2	1
7	10	0
4	4	0
3	0	0
2	1	1
1	2	0
5	6	0
3	3	2
4	2	1
4	2	3
6	3	0
1	1	0
3	3	0
4	0	0
2	1	1
1	0	1
0	2	0
5	0	0
25	36	10
12	7	3
12	6	0
10	14	2
10	6	2
7	9	1
4	1	0
7	5	1
6	1	0
7	2	0
4	0	0
5	4	0
3	8	4
5	3	3
6	2	0
3	2	0
4	2	1
2	9	3
3	3	0
3	0	0
6	1	0
5	0	0
4	2	0
6	5	0
3	1	0
2	0	0
2	1	0
2	3	1

6	2	0
3	1	0
0	0	0
2	1	0
3	0	0
1	5	1
5	0	0
3	0	0
5	0	0
3	1	0
0	0	0
1	0	0
0	0	0
0	0	0
0	0	0
12	1	0
9	3	2
6	1	0
8	2	1
4	2	0
2	2	1
6	8	4
2	2	1
2	4	1
9	3	0
7	1	1
7	1	0
2	1	1
3	2	0
6	2	1
3	3	0
3	4	1
3	2	0
3	1	0
4	2	1
7	1	0
8	2	0
3	0	1
2	2	0
6	0	0
4	3	1
3	3	0
3	1	0
1	7	0
3	1	0
1	3	0
4	0	0

1	1	0
2	0	0
4	8	0
3	2	0
2	2	0
2	1	0
1	0	0
4	0	0
3	0	0
1	0	0
4	0	0
2	0	0
2	0	0
6	9	0
0	0	0
3	3	0
1	0	0
1	0	0
1	0	0
2	0	0
3	0	0
2	1	0
1	0	0
2	0	0
4	1	0
3	4	0
1	0	0
0	0	0
0	0	0
0	0	0
0	0	0
1	0	0
0	0	0
10	10	4
7	3	1
9	2	1
8	6	0
6	0	0
4	1	0
8	0	0
3	2	1
8	5	1
3	0	0
4	1	0
5	2	1
5	1	1
0	2	5

5	4	1
3	3	1
3	0	0
5	2	0
2	2	0
3	2	2
3	2	1
1	1	0
4	1	0
2	1	0
1	1	0
5	0	2
2	1	0
2	0	0
1	0	0
1	0	0
6	1	0
3	1	0
2	2	1
1	0	1
2	2	0
0	1	0
1	4	0
3	2	0
2	1	0
1	0	0
3	0	2
1	0	0
4	0	0
3	2	0
2	2	0
1	2	0
1	1	0
3	3	0
2	0	0
1	0	0
1	2	0
2	0	0
2	1	0
2	0	0
2	0	0
3	1	0
2	1	1
3	0	0
2	1	1
0	2	0
2	1	0

2	1	0
3	2	2
2	0	0
0	0	0
3	0	0
1	0	0
1	1	0
1	0	0
1	3	0
2	1	0
2	0	0
2	2	0
1	1	0
1	0	0
1	1	0
0	2	0
3	1	0
0	1	0
1	0	0
2	1	0
1	0	0
2	0	0
0	0	0
0	0	0
1	0	0
1	0	0
0	0	0
1	0	0
3	0	0
4	0	0
2	1	0
3	1	0
1	0	0
0	0	0
1	0	0
0	0	0
0	0	0
0	0	0
3	4	0
1	0	0
1	0	0
2	0	0
1	0	0
0	0	0
0	0	0
0	1	0
0	0	0

0	0	0
0	0	0
1	0	0
7	2	0
6	5	0
6	3	0
7	6	1
7	3	1
5	5	0
10	1	1
1	2	0
7	15	0
2	2	0
4	2	0
4	3	0
3	3	1
3	4	2
1	0	0
5	2	0
3	1	0
1	0	0
2	1	0
2	1	0
1	3	1
2	1	0
2	0	0
3	1	0
3	0	0
6	2	0
1	1	0
4	1	0
2	1	0
1	0	0
0	0	0
0	0	0
2	0	0
1	0	0
6	0	0
4	0	0
4	1	0
2	0	0
0	0	0
2	0	0
3	0	0
4	0	0
1	1	1
4	0	0

5	4	0
1	0	0
0	0	0
5	1	0
3	1	0
0	0	0
2	0	0
3	0	0
2	1	0
2	0	0
0	0	0
2	0	0
2	0	0
3	0	0
3	1	2
1	1	0
3	2	0
0	1	0
1	2	0
1	0	0
1	1	0
1	0	1
3	0	0
1	1	0
3	1	0
4	2	0
1	0	0
2	0	0
2	0	0
0	0	0
2	2	0
1	0	0
1	0	0
2	0	1
0	0	0
1	0	0
1	0	0
0	1	0
0	0	0
1	0	0
1	0	0
2	0	0
0	0	0
1	1	1
2	1	0
0	0	0
1	0	0

1	0	0
1	0	0
0	1	0
0	0	0
0	0	0
1	1	1
1	0	0
1	0	0
1	1	0
1	0	0
2	0	0
1	0	0
0	0	0
2	0	0
1	1	0
1	0	0
1	1	0
1	0	0
0	0	0
2	1	0
0	0	0
0	0	0
0	1	0
1	1	0
2	1	0
2	2	0
1	0	0
0	0	0
1	1	0
0	0	0
0	0	0
2	0	0
1	0	0
1	0	0
1	0	0
3	0	0
0	0	0
1	1	1
3	0	0
1	0	0
0	0	0
0	0	0
0	0	0
0	0	0
1	0	0
1	0	0
0	0	0

1	0	0
0	1	0
1	1	0
1	1	0
3	0	0
0	0	0
1	0	0
1	0	0
1	0	0
5	0	0
0	0	0
0	0	0
1	0	0
1	0	0
1	1	0
1	0	0
1	1	0
0	1	0
1	0	0
0	0	0
3	0	0
1	0	0
0	0	0
1	0	0
0	0	0
1	0	0
0	0	1
1	0	0
0	1	0
1	0	0
2	0	0
0	1	0
1	0	0
1	0	0
2	1	0
1	0	0
0	0	0
0	0	0
0	0	0
0	0	0
0	0	0
0	0	0
0	0	0
1	0	0
1	0	0
0	0	0
0	0	0

0	0	0
0	0	0
1	0	0
0	0	0
0	0	0
0	0	0
0	0	0
0	0	0
0	0	0
1	0	0
1	0	0
0	0	0
2	0	0
0	0	0
0	1	0
1	0	0
0	0	0
0	0	0
1	0	0
0	0	0
0	0	0
0	0	0
0	0	0
0	0	1
9	3	0
3	0	0
9	0	1
4	0	0
3	1	1
2	1	0
1	0	0
0	0	0
3	2	0
0	0	0
3	3	0
0	0	0
4	1	0
3	1	0
5	2	0
2	0	0
2	0	0
6	0	0
2	2	0
3	2	0
1	0	0
3	4	0
0	0	0

2	1	0
5	1	1
1	0	0
2	0	0
1	4	0
3	1	0
4	1	0
4	0	0
3	0	1
3	0	0
4	1	0
1	0	0
3	2	1
2	6	0
0	0	0
1	0	0
1	0	0
2	3	1
1	2	1
0	0	0
6	0	0
0	1	0
0	0	0
0	0	0
1	0	0
1	1	0
2	1	1
0	1	0
1	0	0
0	1	0
5	0	0
0	1	0
1	1	0
2	1	0
3	1	0
1	0	0
0	0	0
2	0	0
1	0	0
0	0	0
0	0	0
1	0	0
7	3	1
1	0	0
1	1	0
1	1	0
1	0	0

2	0	0
1	0	0
2	0	0
1	1	0
2	0	0
1	1	0
3	0	0
3	0	0
1	1	0
1	0	0
1	0	0
0	0	0
1	0	0
0	0	0
1	0	0
2	1	0
0	0	0
1	1	0
2	1	0
1	1	0
0	0	0
1	0	0
0	0	0
0	0	0
1	0	0
1	0	0
0	0	0
1	0	0
0	0	0
1	0	0
0	0	0
3	0	0
2	0	0
1	1	0
0	1	0
1	1	0
1	0	0
17	0	0
1	0	0
1	0	0
1	1	0
1	0	0
0	0	0
0	0	0
1	0	0
11	0	0
3	0	0

2	0	0
2	0	0
0	0	0
0	0	0
2	0	0
1	0	0
1	1	0
2	0	0
1	0	0
1	0	0
1	1	0
1	0	0
1	0	0
0	0	0
0	0	0
0	0	0
0	0	0
0	0	0
0	0	0
3	0	0
0	0	0
2	0	0
1	0	0
2	0	0
1	0	0
0	0	0
0	0	0
1	1	0
0	0	0
1	0	0
1	0	0
0	0	0
2	0	0
1	3	1
0	0	0
0	0	0
0	0	0
0	0	0
0	0	0
2	0	1
0	0	0
1	0	0
2	0	0
3	0	0
0	0	0
0	0	0
0	0	0

0	0	0
1	0	0
1	0	0
1	0	0
1	0	0
4	0	0
8	7	0
2	0	0
1	0	0
0	0	0
1	1	0
1	0	0
2	0	0
2	0	0
0	0	0
0	0	0
2	0	0
0	0	0
1	0	0
0	0	0
0	0	0
1	0	0
1	0	0
0	0	0
1	0	0
1	1	0
3	0	0
1	0	0
0	0	0
0	0	0
1	0	0
0	0	0
0	0	0
3	1	0
0	0	0
9	0	0
0	2	0
1	1	0
0	0	0
3	3	0
0	0	0
3	0	2
1	0	0
2	0	0
1	0	0
2	0	0
0	0	0

1	0	0
0	0	0
0	0	0
0	1	0
14	9	0
0	0	0
3	0	0
1	0	0
1	0	0
0	0	0
1	0	0
1	0	0
0	0	0
0	0	0
0	0	0
0	0	0
0	0	0
0	0	0
0	0	0
0	0	0
0	0	0
1	0	0
1	0	0
1	0	0
0	0	0
2	0	0
0	0	0
0	0	0
0	1	0
0	0	0
0	0	0
0	0	0
0	0	0
0	0	0
1	0	0
2	0	0
2	0	0
2	0	0
0	0	0
0	0	0
2	0	0
0	0	0
0	0	0
0	0	0
0	0	0
0	1	1
0	1	0

1	0	0
1	1	0
1	0	0
1	0	0
0	1	0
0	0	0
0	0	0
2	0	0
1	0	0
0	0	0
0	0	0
0	0	0
0	0	0
1	0	0
1	0	0
0	0	0
3	0	0
1	0	0
1	0	0
2	0	0
1	0	0
0	0	0
0	0	0
0	0	0
0	0	0
0	0	0
1	0	0
0	0	0
0	0	0
0	0	0
0	0	0
0	0	0
0	0	0
0	0	0
1	0	0
1	0	0
0	0	0
1	0	0
1	0	0
0	0	0
1	0	0
1	0	0
0	0	0
0	0	0
0	0	0
1	0	0
0	0	0

0	0	0
2	0	0
0	0	0
0	0	0
0	0	0
0	0	0
0	0	0
0	0	0
0	0	0
0	0	0
0	0	0
0	0	0
1	0	0
0	0	0
2	0	0
0	0	0
0	0	0
0	0	0
0	0	0
0	0	0
0	0	0
0	0	0
0	0	0
2	0	0
1	0	0
0	0	0
0	0	0
0	0	0
0	0	0
1	0	0
0	0	0
1	0	0
7	0	0
0	0	0
0	0	0
0	0	0
1	0	0
0	0	0
0	0	0
0	0	0
0	0	0
0	0	0
1	0	0
0	0	0
0	0	0
0	0	0
0	0	0

0	0	0
0	0	0
0	0	0
0	0	0
0	0	0
0	0	0
1	1	0
0	0	0
0	0	0
0	0	0
0	0	0
1	0	0
0	0	0
0	2	1
0	0	0
0	0	0
0	1	0
0	0	0
0	0	0
0	0	0
0	0	0
0	0	0
2	0	0
0	0	0
0	0	0
3	0	0
0	0	0
0	0	0
0	0	0
0	0	0
0	0	0
0	0	0
0	0	0
0	0	0
0	0	0
0	0	0
0	0	0
0	0	0
1	0	0
0	0	0
0	0	0
2	0	0
1	0	0
1	0	0
0	0	0
0	0	0
2	0	0

0	0	0
0	0	0
0	0	0
0	0	0
0	0	0
0	0	0
0	0	0
0	0	0
0	0	0
0	0	0
0	0	0
0	0	0
0	0	0
0	0	0
0	0	0
0	0	0
2	0	0
0	0	0
0	0	0
0	0	0
0	0	0
0	0	0
0	0	0
0	0	0
0	0	0
0	0	0
0	0	0
1	0	0
0	0	0
0	0	0
0	0	0
0	0	0
0	0	0
0	0	0
0	0	0
0	0	0
0	0	0
0	0	0
0	0	0
0	0	0
0	0	0
0	0	0
0	0	0
0	0	0
0	0	0
0	0	0
0	0	0

0	0	0
0	0	0
0	4	0
0	0	0
0	0	0
0	0	0
0	0	0
0	0	0
0	0	0
0	0	0
0	0	0
0	0	0
0	0	0
0	0	0
0	0	0
0	0	0
0	0	0
0	0	0
0	0	0
0	0	0
0	0	0
0	0	0
0	0	0
0	0	0
0	0	0
0	0	0
0	0	0
0	0	0
0	0	0
0	0	0
0	0	0
0	0	0
0	0	0

					ATN			IgAN E0
order	Identified Proteins	GeneName	Accession No	Molecular Weight	G24	G25	G26	G8
1	Myosin-9	MYH9	P35579	227 kDa	62	125	122	114
2	Vimentin	VIM	P08670	54 kDa	55	67	92	103
3	Alpha-actir	ACTN4	O43707	105 kDa	48	80	82	66
4	Actin. cyto	ACTB	P60709	42 kDa	38	64	68	77
5	Talin-1	TLN1	Q9Y490	270 kDa	37	80	33	55
6	Neuroblast	AHNAK	Q09666	629 kDa	21	47	31	40
7	Laminin sul	LAMA5	O15230	400 kDa	31	52	50	43
8	Isoform 4 c	PLEC	Q15149-4	516 kDa	51	62	24	57
9	Collagen al	COL6A3	E9PCV6	322 kDa	30	84	45	45
10	Isoform 2 c	FLNA	P21333-2	280 kDa	17	39	33	43
11	Nestin	NES	P48681	177 kDa	34	62	44	48
12	Keratin. ty	KRT1	P04264	66 kDa	39	23	5	29
13	Laminin sul	LAMB2	P55268	196 kDa	36	54	46	47
14	Basement	HSPG2	P98160	469 kDa	22	61	15	33
15	Prelamin-A	LMNA	P02545	74 kDa	29	34	35	45
16	Keratin. ty	KRT9	P35527	62 kDa	36	12	0	26
17	Keratin. ty	KRT10	P13645	59 kDa	32	31	9	36
18	Isoform 3 c	SPTAN1	Q13813-3	282 kDa	14	35	12	24
19	Isoform 1 c	VCL	P18206-2	117 kDa	24	40	35	43
20	Laminin sul	LAMC1	P11047	178 kDa	18	41	37	35
21	Spectrin be	SPTBN1	Q01082	275 kDa	16	34	23	32
22	Keratin. ty	KRT2	P35908	65 kDa	17	29	9	24
23	Isoform 17	FN1	P02751-17	256 kDa	10	25	25	23
24	Moesin	MSN	P26038	68 kDa	14	31	18	21
25	Hemoglobi	HBB	P68871	16 kDa	9	21	37	22
26	Isoform 2 c	NID1	P14543-2	122 kDa	6	24	17	23
27	Tubulin bet	TUBB4B	P68371	50 kDa	25	24	15	20
28	Hemoglobi	HBA1	P69905	15 kDa	5	19	17	20
29	Compleme	C3	P01024	187 kDa	5	21	20	31
30	Isoform 2 c	SYNPO	Q8N3V7-2	96 kDa	17	22	19	25
31	Isoform 2 c	ANXA2	P07355-2	40 kDa	12	22	22	21
32	Isoform 2 c	GSN	P06396-2	81 kDa	9	24	17	14
33	Tubulin alp	TUBA1B	P68363	50 kDa	22	28	18	17
34	Ras GTPase	IQGAP2	Q13576	181 kDa	12	21	10	11
35	Isoform 6 c	AGRN	O00468-6	215 kDa	16	30	17	18
36	Glyceraldel	GAPDH	P04406	36 kDa	6	15	12	12
37	Keratin. ty	KRT14	P02533	52 kDa	12	7	2	13
38	Tropomyos	TPM4	P67936	29 kDa	10	16	12	25
39	Isoform 4 c	CALD1	Q05682-4	63 kDa	6	16	19	20
40	Tight juncti	TJP1	Q07157	195 kDa	10	17	10	16
41	Serum albu	ALB	P02768	69 kDa	6	25	36	13

42	Isoform 5 c	PDLIM2	Q96JY6-5	63 kDa	16	13	18	18
43	Histone H4	HIST1H4A	P62805	11 kDa	13	19	16	13
44	ATP syntha	ATP5B	P06576	57 kDa	17	18	9	11
45	Isoform 2 c	CLTC	Q00610-2	188 kDa	14	17	9	8
46	ATP syntha	ATP5A1	P25705	60 kDa	14	12	7	13
47	78 kDa gluc	HSPA5	P11021	72 kDa	29	12	11	11
48	Dihydropyr	DPYSL2	Q16555	62 kDa	7	11	11	14
49	Calmodulin	CALM1	P62158	17 kDa	3	12	14	17
50	Keratin. typ	KRT6A	P02538	60 kDa	7	9	0	0
51	Histone H2	HIST1H2AG	P0C0S8	14 kDa	10	8	14	9
52	Annexin A1	ANXA1	P04083	39 kDa	3	20	11	5
53	Histone H2	HIST1H2BC	P62807	14 kDa	11	10	16	7
54	Neprilysin	MME	P08473	86 kDa	9	10	10	9
55	Collagen al	COL6A1	P12109	109 kDa	4	21	13	9
56	EH domain	EHD3	Q9NZN3	61 kDa	5	8	6	8
57	Alpha-enol	ENO1	P06733	47 kDa	12	18	5	7
58	Heat shock	HSPA8	P11142	71 kDa	15	22	11	13
59	Phosphogly	PGK1	P00558	45 kDa	2	12	7	6
60	Ubiquitin-4	RPS27A	P62979	18 kDa	6	7	7	11
61	Keratin. typ	KRT5	P13647	62 kDa	10	12	0	11
62	Isoform 2 c	HSP90AA1	P07900-2	98 kDa	12	15	9	5
63	Heat shock	HSPA1A	P08107	70 kDa	12	15	8	9
64	14-3-3 prot	YWHAE	P62258	29 kDa	13	12	13	7
65	Pyruvate ki	PKM	P14618	58 kDa	5	16	11	4
66	Desmoplak	DSP	P15924	332 kDa	44	0	0	0
67	Isoform 2 c	NEBL	O76041-2	31 kDa	8	11	14	13
68	Heterogen	HNRNPA2B	P22626	37 kDa	4	11	9	6
69	Unconvent	MYO1C	O00159	122 kDa	6	7	9	13
70	Collagen al	COL6A2	P12110	109 kDa	2	20	11	7
71	Heat shock	HSPB1	P04792	23 kDa	7	12	5	10
72	Transitiona	VCP	P55072	89 kDa	5	14	7	6
73	Isoform 2 c	FGA	P02671-2	70 kDa	3	11	12	10
74	Ubiquitin-li	UBA1	P22314	118 kDa	5	13	5	9
75	PDZ and LII	PDLIM5	Q96HC4	64 kDa	5	8	5	4
76	Isoform 2 c	MYH10	P35580-2	231 kDa	7	19	12	12
77	Histone H3	HIST1H3A	P68431	15 kDa	6	3	6	10
78	Protein disı	PDIA3	P30101	57 kDa	5	10	7	10
79	Collagen al	COL4A2	P08572	168 kDa	5	15	7	6
80	Actin. alph	ACTC1	P68032	42 kDa	27	41	45	63
81	Peptidyl-pr	PPIA	P62937	18 kDa	8	6	1	8
82	Annexin A6	ANXA6	P08133	76 kDa	2	12	6	7
83	Cytoplasmi	DYNC1H1	Q14204	532 kDa	7	12	2	9
84	Annexin A5	ANXA5	P08758	36 kDa	6	13	2	7
85	Basal cell a	BCAM	P50895	67 kDa	6	8	10	12
86	Myosin ligh	MYL6	G8JLA2	17 kDa	5	7	9	7
87	Histone H1	HIST1H1E	P10412	22 kDa	8	7	5	10
88	Na(+)/H(+)	SLC9A3R2	Q15599	37 kDa	6	7	9	8

89	Transgelin-	TAGLN2	P37802	22 kDa	4	8	9	8
90	Isoform Sh	YWHAB	P31946-2	28 kDa	8	10	14	9
91	Isoform 2 c	HNRNPK	P61978-2	51 kDa	8	11	8	8
92	Isoform 2 c	COL18A1	P39060-1	154 kDa	2	10	4	7
93	Lamin-B2	LMNB2	Q03252	68 kDa	7	11	5	9
94	Isoform 2 c	HNRNPM	P52272-2	74 kDa	8	11	2	6
95	Isoform 8 c	FLNB	O75369-8	282 kDa	2	14	0	7
96	Isoform 2 c	2-Sep	Q15019-2	45 kDa	5	10	1	16
97	Alpha-actir	ACTN1	P12814	103 kDa	18	31	17	13
98	Isoform 3 c	ZNF185	O15231-3	74 kDa	3	12	4	9
99	Isoform Ga	FGG	P02679-2	49 kDa	0	9	9	9
100	Myosin reg	MYL12B	O14950	20 kDa	4	10	10	9
101	14-3-3 prot	YWHAZ	P63104	28 kDa	9	12	12	12
102	Sodium/po	ATP1A1	P05023	113 kDa	5	9	5	3
103	Fibrinogen	FGB	P02675	56 kDa	0	7	9	5
104	Ig kappa ch	IGKC	P01834	12 kDa	1	13	11	6
105	Peroxiredo	PRDX1	Q06830	22 kDa	3	8	3	6
106	Ig gamma-:	IGHG1	P01857	36 kDa	0	11	12	4
107	Endoplasm	HSP90B1	P14625	92 kDa	5	9	5	4
108	Keratin. typ	KRT16	P08779	51 kDa	0	0	0	8
109	Triosephos	TPI1	P60174	31 kDa	2	10	6	5
110	LIM and SH	LASP1	Q14847	30 kDa	3	5	1	11
111	Ezrin	EZR	P15311	69 kDa	10	18	10	13
112	Elongation	EEF1A1	P68104	50 kDa	12	6	6	5
113	Podocin	NPHS2	Q9NP85	42 kDa	5	7	3	8
114	Tubulin bet	TUBB	P07437	50 kDa	22	25	15	18
115	Tensin-1	TNS1	E9PGF5	183 kDa	3	5	1	6
116	Src substra	CTTN	Q14247	62 kDa	2	6	5	5
117	Isoform 2 c	TPM3	P06753-2	29 kDa	7	11	11	22
118	Isoform 2 c	ALDOA	P04075-2	45 kDa	3	5	5	5
119	Integrin-lin	ILK	Q13418	51 kDa	3	7	7	5
120	Heat shock	HSP90AB1	P08238	83 kDa	12	14	10	10
121	Ras GTPase	IQGAP1	P46940	189 kDa	2	12	1	5
122	Isoform 3 c	FERMT2	Q96AC1-3	79 kDa	1	7	2	2
123	Glutathion	GSTP1	P09211	23 kDa	2	9	3	2
124	Protein-glu	TGM2	P21980	77 kDa	0	10	2	7
125	Vitronectin	VTN	P04004	54 kDa	1	5	5	4
126	Integrin be	ITGB1	P05556	88 kDa	3	7	9	3
127	Profilin-1	PFN1	P07737	15 kDa	4	8	3	5
128	Collagen al	COL4A4	J3KNM7	164 kDa	5	7	3	2
129	Fructose-bi	ALDOB	P05062	39 kDa	1	9	3	1
130	Septin-7	7-Sep	Q16181	51 kDa	4	9	2	1
131	Peroxiredo	PRDX6	P30041	25 kDa	6	6	5	1
132	Clusterin	CLU	P10909	52 kDa	5	4	1	3
133	Na(+)/H(+)	SLC9A3R1	O14745	39 kDa	4	6	8	7
134	L-lactate de	LDHB	P07195	37 kDa	2	6	3	1
135	Microtubul	MAP4	E7EVA0	245 kDa	2	7	5	4

136	Glutamyl a	ENP	Q07075	109 kDa	2	6	1	4
137	Peroxiredo	PRDX2	P32119	22 kDa	6	7	6	5
138	Heterogen	HNRNPU	Q00839	91 kDa	3	7	2	5
139	Plastin-3	PLS3	P13797	71 kDa	2	6	1	4
140	Phosphatid	BP1	P30086	21 kDa	2	7	3	2
141	Palladin	PALLD	Q8WX93	151 kDa	5	6	1	6
142	Nucleolin	NCL	P19338	77 kDa	2	4	0	3
143	Ras-relatec	RRAS	P10301	23 kDa	3	8	2	5
144	Isoform 2 c	TKT	P29401-2	69 kDa	3	5	4	5
145	Transgelin	TAGLN	Q01995	23 kDa	1	7	4	5
146	Adenylyl cy	CAP1	Q01518	52 kDa	1	4	2	4
147	Isoform LC	DPYSL3	Q14195-2	74 kDa	3	7	10	5
148	Creatine ki	CKB	P12277	43 kDa	0	5	5	4
149	Zyxin	ZYX	Q15942	61 kDa	4	2	3	5
150	Ig alpha-1 c	IGHA1	P01876	38 kDa	0	3	8	7
151	Isoform 2 c	HNRNPA1	P09651-3	29 kDa	7	4	4	5
152	Lipoma-pre	LPP	Q93052	66 kDa	1	4	2	4
153	Isoform 4 c	TENC1	Q63HR2-4	154 kDa	2	5	6	6
154	Heterogen	HNRNPH1	P31943	49 kDa	4	3	0	2
155	HLA class I	HLA-B	Q29836	40 kDa	2	2	1	5
156	Serum amy	APCS	P02743	25 kDa	3	5	3	3
157	Isoform 3 c	LDHA	P00338-3	40 kDa	3	8	6	1
158	Protein DJ-	PARK7	Q99497	20 kDa	1	2	2	4
159	Tenascin	TNC	P24821	241 kDa	0	6	0	0
160	Isoform 2 c	PODXL	O00592-2	55 kDa	4	6	8	4
161	Compleme	C4B	F5GXS0	188 kDa	2	4	1	3
162	Protein S10	S100A9	P06702	13 kDa	0	10	1	0
163	Junction pl	JUP	F5GWP8	66 kDa	14	3	0	0
164	Integrin alp	ITGA3	P26006	117 kDa	1	2	2	4
165	Cysteine ar	CSRP1	P21291	21 kDa	1	2	4	3
166	Cytosolic n	CNDP2	Q96KP4	53 kDa	1	1	2	1
167	Calreticulin	CALR	P27797	48 kDa	0	1	2	0
168	Alpha-parv	PARVA	Q9NVD7	42 kDa	4	9	3	2
169	Protein dis	P4HB	P07237	57 kDa	2	6	2	4
170	60 kDa hea	HSPD1	P10809	61 kDa	2	7	1	1
171	LIM domai	LMO7	E9PMS6	145 kDa	0	1	1	9
172	Splicing fac	SFPQ	P23246	76 kDa	2	3	2	4
173	Rab GDP di	GDI2	P50395	51 kDa	1	8	0	2
174	Calpain sm	CAPNS1	P04632	28 kDa	2	9	0	2
175	Isoform 2 c	HLA-A	P13746-2	41 kDa	5	5	6	5
176	Elongation	EEF2	P13639	95 kDa	9	3	2	3
177	Aminoacyla	ACY1	Q03154	46 kDa	0	4	0	2
178	Compleme	C9	P02748	63 kDa	2	3	3	4
179	Isoform C1	HNRNPC	P07910-2	32 kDa	1	4	2	2
180	Calponin-3	CNN3	Q15417	36 kDa	2	4	2	4
181	Cysteine-ri	CRIP2	P52943	22 kDa	3	7	1	4
182	Apolipopro	APOE	P02649	36 kDa	2	4	2	2

183	Cathepsin I	CTSD	P07339	45 kDa	1	8	5	2
184	14-3-3 prot	YWHAG	P61981	28 kDa	7	7	11	9
186	Isoform 2 c	MYO1B	O43795-2	125 kDa	1	3	0	2
187	Collagen al	COL4A1	P02462	161 kDa	0	4	1	2
188	Phosphogly	PGAM1	P18669	29 kDa	1	2	1	0
189	Protein S1(	S100A6	P06703	10 kDa	2	2	9	4
190	Alpha-1-an	SERPINA1	P01009	47 kDa	0	7	15	1
191	WD repeat	WDR1	O75083	66 kDa	0	2	1	2
192	Isoform 5 c	9-Sep	Q9UHD8-5	65 kDa	1	5	2	2
193	Septin-11	11-Sep	Q9NVA2	49 kDa	0	2	2	3
194	Ras suppre	RSU1	Q15404	32 kDa	2	4	2	2
195	Cofilin-1	CFL1	E9PK25	23 kDa	3	5	2	2
196	Erythrocyte	STOM	P27105	32 kDa	2	8	4	2
197	Nucleopho	NPM1	P06748	33 kDa	3	5	1	1
199	ADP-ribosy	ARF1	P84077	21 kDa	2	5	4	1
200	40S ribosor	RPS3	P23396	27 kDa	7	3	2	2
201	Annexin A4	ANXA4	P09525	36 kDa	2	6	3	4
202	Isoform 2 c	RAB5C	P51148-2	27 kDa	3	6	2	2
203	Protein AN	AMBP	P02760	39 kDa	4	3	3	4
204	Heterogen	HNRNPA3	P51991	40 kDa	4	3	3	4
205	Poly(rC)-bi	PCBP1	Q15365	37 kDa	4	3	4	1
206	Tubulin bet	TUBB2A	Q13885	50 kDa	24	22	0	17
207	Isoform 2 c	MAGI2	Q86UL8-2	157 kDa	3	3	0	3
208	Radixin	RDX	P35241	69 kDa	5	14	8	8
209	PDZ and LII	PDLIM1	O00151	36 kDa	1	1	1	5
210	Catalase	CAT	P04040	60 kDa	2	4	3	1
211	Capping pr	CAPZB	B1AK88	34 kDa	1	3	3	4
212	Isoform 3 c	HNRNPH3	P31942-3	32 kDa	1	4	0	2
213	Ras-relatec	RAP1A	P62834	21 kDa	2	3	3	3
214	Calnexin	CANX	B4DGP8	72 kDa	3	3	2	1
215	Isoform 3 c	TINAGL1	Q9GZM7-3	49 kDa	0	3	0	1
216	F-actin-cap	CAPZA1	P52907	33 kDa	1	3	1	3
217	Trifunction	HADHA	P40939	83 kDa	1	6	1	1
218	Stress-70 p	HSPA9	P38646	74 kDa	4	5	0	4
220	Isoform 2 c	CYB5R3	P00387-2	32 kDa	1	4	2	0
222	Isoform sG	GNAI2	P04899-4	42 kDa	3	5	2	3
223	Inverted fo	INF2	Q27J81	136 kDa	1	6	5	2
224	Aconitate h	ACO2	A2A274	88 kDa	0	7	1	3
225	Glutamate	GLUD1	P00367	61 kDa	0	8	2	1
226	Argininosu	ASS1	P00966	47 kDa	0	0	0	0
227	Isoform 2 c	MYLK	Q15746-2	203 kDa	1	2	2	3
228	Cytoskelet	CKAP4	Q07065	66 kDa	0	3	0	2
229	Isoform 2 c	DBNL	Q9UJU6-2	48 kDa	0	3	1	3
231	Alpha-2-ma	A2M	P01023	163 kDa	0	3	2	1
233	Isoform 2 c	LAP3	P28838-2	53 kDa	4	3	0	4
234	Actin-relat	ACTR3	P61158	47 kDa	2	3	2	3
235	Myosin reg	MYL9	P24844	20 kDa	4	11	9	10

237	Carbonyl re	CBR1	P16152	30 kDa	1	6	0	3
238	Tensin-3	TNS3	Q68CZ2	155 kDa	0	4	3	4
239	Malate deh	MDH2	P40926	36 kDa	1	3	2	1
241	Integrin alp	ITGA1	P56199	131 kDa	1	1	0	2
242	Selenium-b	SELENBP1	Q13228	52 kDa	8	1	1	4
243	Thymosin b	TMSB4X	P62328	5 kDa	0	2	0	3
244	Isoform 2 c	IGHM	P01871-2	52 kDa	0	3	3	2
245	Keratin. typ	KRT3	P12035	64 kDa	0	0	0	0
246	Fibulin-1	FBLN1	P23142	77 kDa	1	3	3	1
247	Galectin-1	LGALS1	P09382	15 kDa	1	4	1	1
248	Ras-relatec	RAB7A	P51149	23 kDa	5	2	1	2
250	Haptoglobi	HP	H0Y300	49 kDa	0	1	7	1
251	Ras-relatec	RAB14	P61106	24 kDa	2	2	1	0
253	Major vaul	MVP	Q14764	99 kDa	0	3	1	3
255	Isoform 2 c	TPM2	P07951-2	33 kDa	6	10	7	18
256	Galectin-3-	LGALS3BP	Q08380	65 kDa	2	5	2	1
257	EH domain	EHD4	Q9H223	61 kDa	3	6	2	3
259	T-complex	CCT2	P78371	57 kDa	0	4	1	0
260	Hemoglobi	HBD	P02042	16 kDa	5	15	18	13
261	Isoform 2 c	TGFB1I1	O43294-2	48 kDa	1	3	1	1
262	Isoform 2 c	UTRN	P46939-2	395 kDa	0	1	0	6
263	Rho GDP-d	ARHGDIA	J3KTF8	22 kDa	0	1	3	3
264	Intercellula	ICAM1	P05362	58 kDa	0	5	0	2
266	Isoform 2 c	KIRREL	Q96J84-2	85 kDa	0	2	1	1
268	Neutral alp	GANAB	Q14697	107 kDa	0	3	1	3
269	Protein eva	EVA1B	Q9NVM1	18 kDa	0	4	0	1
272	F-actin-cap	CAPZA2	P47755	33 kDa	0	2	0	5
273	Nephrin	NPHS1	O60500	135 kDa	0	1	1	2
274	Isoform 2 c	ITGAV	P06756-2	112 kDa	1	1	0	1
276	Staphyloco	SND1	Q7KZF4	102 kDa	1	2	0	2
277	Isoform 2 c	GPI	P06744-2	64 kDa	0	5	2	0
279	Isoform 3 c	HNRNPD	Q14103-3	33 kDa	5	1	0	3
281	14-3-3 prot	YWHAH	Q04917	28 kDa	5	4	8	7
282	Ras-relatec	RAB1A	P62820	23 kDa	5	3	2	2
286	Isoform 2 c	PDIA6	Q15084-2	54 kDa	1	6	1	1
287	Alcohol del	AKR1A1	P14550	37 kDa	0	2	0	1
288	Carbonic ar	CA2	P00918	29 kDa	0	2	0	1
289	Alpha-cryst	CRYAB	P02511	20 kDa	3	6	0	1
291	Actin-relat	ARPC2	O15144	34 kDa	0	3	2	2
294	Tropomyos	TPM1	Q6ZN40	37 kDa	0	0	0	15
296	X-ray repai	XRCC5	P13010	83 kDa	0	1	0	0
297	Rab GDP di	GDI1	P31150	51 kDa	1	6	2	1
299	Dolichyl-di	RPN1	P04843	69 kDa	1	1	0	1
300	Serotransfe	TF	P02787	77 kDa	0	3	4	3
301	Hornerin	HRNR	Q86YZ3	282 kDa	2	0	0	0
313	Calpain-2 c	CAPN2	P17655	80 kDa	1	2	0	0
314	Chloride in	CLIC5	Q9NZA1	47 kDa	1	1	4	1

317	Non-POU d	NONO	Q15233	54 kDa	2	3	0	1
326	Retinal deh	ALDH1A1	P00352	55 kDa	0	0	0	2
334	Peroxiredo	PRDX5	P30044	22 kDa	0	6	0	1
336	Isoform 2 c	TRIM28	Q13263-2	79 kDa	0	5	0	2
341	ADP-ribosy	ARF6	P62330	20 kDa	4	3	2	1
345	Protein-L-is	PCMT1	H7BY58	30 kDa	0	3	0	2
347	Apolipopro	APOA1	P02647	31 kDa	0	2	6	0
352	Alpha-1-an	SERPINA3	P01011	48 kDa	0	5	4	0
353	Fructose-1.	FBP1	P09467	37 kDa	0	0	0	0
356	Ferritin ligh	FTL	P02792	20 kDa	1	4	0	0
362	Ig gamma-2	IGHG2	P01859	36 kDa	0	0	0	4
363	Isoform 2 c	PTGR1	Q14914-2	33 kDa	0	6	0	0
365	Cytoplasmi	ACO1	P21399	98 kDa	0	0	0	0
373	Chloride in	CLIC1	O00299	27 kDa	4	3	3	1
378	14-3-3 prot	YWHAQ	P27348	28 kDa	6	6	9	5
384	Allograft in	AIF1	P55008	17 kDa	0	1	1	1
410	Lamin-B1	LMNB1	P20700	66 kDa	6	5	0	6
413	Keratin. typ	KRT13	P13646	50 kDa	3	5	0	0
435	Beta-actin-	ACTBL2	Q562R1	42 kDa	12	0	19	0
437	Glutathione	GSTA2	P09210	26 kDa	0	0	0	0
438	Tubulin bet	TUBB6	Q9BUF5	50 kDa	0	13	4	8
448	Tropomyos	TPM1	H7BYY1	29 kDa	4	6	6	15
460	Ig lambda-2	IGLC2	P0CG05	11 kDa	0	7	3	1
526	Histone H2	HIST2H2BE	Q16778	14 kDa	11	8	17	7
551	Tubulin alp	TUBA4A	P68366	50 kDa	19	17	0	0
558	EH domain	EHD1	Q9H4M9	61 kDa	2	0	0	0
563	Histone H2	H2AFZ	P0C0S5	14 kDa	2	3	4	3
587	POTE ankyr	POTEI	P0CG38	121 kDa	9	15	14	0
597	Histone H2	HIST1H2AC	Q93077	14 kDa	0	0	0	8
601	Histone H2	HIST2H2AA	Q6FI13	14 kDa	0	0	0	0
603	Isoform 2 c	MYH11	P35749-2	228 kDa	0	0	0	8
604	Bifunctiona	DAK	Q3LXA3	59 kDa	0	0	0	0
606	HLA class I	HLA-B	P01889	40 kDa	0	0	2	5
635	Keratin. typ	KRT17	Q04695	48 kDa	0	0	0	0
663	Histone H2	H2AFX	P16104	15 kDa	2	4	0	0
664	Isoform 2 c	MYH14	Q7Z406-2	232 kDa	4	8	7	0
715	Heterogene	HNRNPF	P52597	46 kDa	3	0	0	0
728	Keratin. typ	KRT6C	P48668	60 kDa	0	9	0	0
733	Ig gamma-4	IGHG4	P01861	36 kDa	0	3	3	2
753	Heat shock	HSPA2	P54652	70 kDa	7	14	9	0
804	HLA class I	HLA-A	P01891	41 kDa	0	5	0	0
839	HLA class I	HLA-B	P30487	41 kDa	2	0	2	4
913	Isoform 2 c	KRT8	P05787-2	57 kDa	0	7	0	0
999	HLA class I	HLA-A	P30453	41 kDa	0	0	0	0

Raw Counts								
	IgA E1			NLTx				
G10	G4	G21	G23	G5	G6	G13	G14	G28
173	151	141	100	171	240	162	211	175
146	136	74	97	108	128	113	159	227
90	77	95	80	94	122	91	106	204
81	71	73	59	60	71	68	73	191
56	55	72	35	72	89	61	60	102
40	50	58	24	80	100	42	72	107
56	46	66	45	62	77	65	84	80
45	40	50	28	67	88	39	52	97
30	44	59	46	54	77	43	62	100
57	54	79	34	52	67	49	60	75
47	51	60	38	62	56	59	62	71
31	53	23	79	22	19	34	35	16
57	44	59	36	54	55	49	58	66
46	36	45	34	41	59	43	50	83
70	49	49	44	51	55	57	63	41
3	35	9	70	20	21	24	39	3
59	48	21	55	20	9	46	66	26
30	32	39	17	50	67	52	53	83
50	32	44	32	32	52	40	47	49
44	34	48	31	35	54	40	61	50
35	38	40	17	45	54	41	43	60
49	41	17	40	15	4	29	49	13
57	38	38	36	30	44	26	42	46
28	36	19	17	29	37	29	36	39
29	20	52	34	14	28	12	12	26
34	25	25	13	25	50	26	39	36
25	18	27	16	30	38	26	36	36
35	22	42	18	13	43	9	24	31
54	34	50	16	7	6	7	6	17
23	26	22	13	29	34	32	35	30
25	23	24	20	21	18	28	26	31
32	16	27	17	27	32	28	36	39
21	19	25	13	23	21	26	26	49
20	16	17	6	29	29	27	23	39
17	16	26	20	24	30	16	22	25
19	13	14	14	20	22	23	32	76
6	19	6	24	5	1	7	12	3
27	29	15	14	26	26	27	28	13
31	20	25	13	20	26	22	22	19
15	12	12	7	34	32	24	33	37
26	12	27	24	13	7	9	10	45

20	19	13	18	19	25	28	30	16
16	20	24	26	11	18	16	16	18
16	14	17	13	17	24	13	19	28
11	11	13	12	18	24	11	14	43
13	14	12	8	14	23	16	19	22
10	10	9	10	23	20	8	13	21
15	12	10	4	19	20	18	22	21
22	11	19	12	14	15	23	18	12
13	13	6	19	0	0	7	12	0
14	10	12	14	15	25	21	21	11
13	13	12	4	13	22	13	17	14
28	14	15	26	6	9	10	18	12
9	8	12	5	10	18	11	9	35
7	9	12	12	12	21	12	14	25
9	8	16	6	13	25	11	15	24
7	7	11	7	15	15	14	22	17
11	13	13	4	18	13	13	14	21
7	5	12	5	16	21	12	11	27
16	13	7	8	12	11	18	16	10
13	16	6	18	5	5	9	16	0
8	7	10	7	12	12	13	11	19
8	12	16	9	13	12	14	15	24
11	7	13	10	18	14	14	18	22
10	11	10	5	15	15	15	13	19
0	2	0	1	0	0	0	0	0
13	11	8	6	20	17	17	13	14
16	12	14	10	14	13	9	20	17
7	12	9	3	17	19	9	13	31
5	7	12	11	8	17	5	13	21
10	14	9	7	10	14	13	9	13
6	12	10	4	15	14	8	11	17
20	23	16	8	1	0	1	1	8
5	14	8	4	19	18	12	13	24
11	14	7	3	15	20	18	13	15
27	17	17	8	23	35	12	16	31
12	12	3	11	9	14	9	11	5
8	12	10	3	14	11	6	12	14
14	7	10	12	9	14	10	10	14
60	53	57	42	44	59	52	45	73
7	8	8	3	9	19	12	14	16
10	10	10	4	7	15	7	14	19
4	6	3	1	13	15	6	10	22
8	6	12	5	12	9	9	18	21
14	13	9	4	10	16	15	11	13
9	8	9	6	9	14	14	17	12
13	10	9	10	8	9	15	19	15
8	8	9	4	14	13	8	10	17

7	8	8	7	8	10	5	6	10
12	8	17	10	14	15	14	20	18
7	6	5	4	8	13	9	9	18
6	6	12	4	7	13	4	9	20
10	7	13	5	15	19	8	9	15
12	8	3	3	6	7	10	12	8
5	13	14	2	5	12	8	10	17
11	6	8	1	10	11	15	12	12
27	18	36	19	26	40	17	26	55
6	10	6	2	15	10	13	10	13
28	13	12	16	3	1	0	1	7
8	8	9	7	12	14	5	8	10
16	11	15	11	12	14	14	19	16
7	7	11	3	9	13	6	7	17
24	23	14	11	2	0	0	0	7
19	10	11	9	6	2	2	5	5
6	6	6	3	8	10	11	11	6
9	1	8	5	3	1	1	2	8
6	7	7	4	9	8	4	6	14
0	14	0	25	0	0	0	0	0
5	6	10	3	10	9	5	7	14
6	9	5	4	9	13	9	7	9
18	13	6	10	21	18	19	15	27
6	5	8	8	7	6	6	6	11
9	7	8	3	6	8	13	10	9
27	19	29	14	30	39	24	39	41
10	6	7	6	10	12	5	8	6
2	4	11	3	11	13	8	6	10
18	24	16	7	24	25	22	22	9
8	2	4	2	9	10	10	7	10
8	5	12	2	3	10	5	8	8
8	13	14	6	14	17	14	14	16
4	4	10	1	8	5	9	10	13
2	5	5	0	9	10	10	16	15
4	3	9	4	11	10	5	7	11
5	7	9	1	6	6	8	7	8
13	9	7	4	5	6	5	6	6
6	2	9	6	8	6	4	5	7
5	9	8	3	9	7	6	6	9
3	5	13	4	5	6	5	6	10
1	0	7	3	14	9	11	11	16
4	3	8	1	7	8	7	7	13
5	5	6	2	11	9	9	11	13
6	4	7	5	5	5	3	3	8
3	5	4	6	8	7	6	7	8
6	1	5	2	10	10	7	9	10
7	3	7	1	7	5	5	6	10

1	3	3	1	11	7	5	2	16
6	8	6	4	10	11	3	5	7
5	2	9	2	11	5	3	6	10
3	7	11	1	5	4	6	3	10
6	2	7	4	6	8	4	9	14
2	5	6	1	10	7	4	7	6
2	3	3	1	6	9	7	6	5
4	4	8	6	6	9	6	4	8
5	4	5	1	6	7	5	5	9
6	2	9	3	3	5	3	3	11
4	6	6	2	1	6	4	2	14
7	5	14	0	16	15	12	13	16
1	3	2	0	10	11	3	14	17
6	5	3	4	5	6	5	5	6
21	5	5	8	3	0	0	1	2
6	4	3	5	13	7	5	3	7
5	3	7	2	9	9	3	3	7
5	9	6	4	5	8	8	7	11
5	7	3	0	10	9	4	3	6
4	4	4	6	9	6	4	11	6
5	3	6	2	7	9	4	6	6
3	2	4	4	5	4	4	3	12
2	2	4	0	7	7	4	3	12
2	5	5	2	7	9	3	6	6
3	3	4	7	4	3	3	3	8
7	6	2	1	3	5	1	6	5
0	1	3	0	4	4	1	0	3
0	10	0	8	0	0	0	3	0
4	2	3	1	7	7	4	5	10
4	5	3	6	6	5	5	5	7
3	2	3	0	10	8	10	5	9
2	6	3	3	8	7	5	6	10
4	2	6	5	1	2	3	4	6
1	6	6	1	6	5	2	4	6
4	5	3	4	10	6	5	6	10
11	6	4	2	5	11	2	4	4
6	2	1	3	7	7	3	3	5
4	4	4	1	4	4	3	6	11
1	1	4	4	2	4	2	2	11
8	7	4	5	7	3	9	11	6
1	3	3	0	5	3	0	1	4
1	0	1	0	10	7	4	9	20
12	5	4	5	3	3	3	4	3
1	3	3	2	4	7	5	3	5
4	5	5	1	3	7	5	4	5
4	5	3	2	4	4	5	4	7
5	6	7	6	1	1	3	5	5

2	3	2	7	2	5	2	2	3
15	6	14	9	9	13	13	16	11
1	0	3	1	6	7	4	4	11
5	3	4	2	2	6	2	4	9
2	2	4	1	6	4	4	8	16
6	2	1	4	4	3	4	2	7
0	1	2	3	2	0	0	0	6
2	3	3	2	4	2	4	5	5
2	3	3	0	2	4	5	6	8
3	1	6	0	5	10	8	5	9
2	1	5	1	3	5	0	2	16
2	2	5	1	3	4	2	6	11
4	2	3	1	3	5	2	0	5
2	2	4	0	7	6	2	5	6
2	1	4	4	2	3	2	4	6
2	4	2	1	3	4	3	5	3
1	1	6	0	4	5	3	6	5
4	2	4	2	3	2	2	3	3
4	1	5	3	3	6	1	6	4
1	2	6	5	6	4	2	3	8
5	4	2	1	5	4	4	6	3
22	16	21	11	32	32	22	28	32
4	1	2	0	4	5	3	3	6
9	10	6	8	20	18	15	10	26
3	4	0	1	3	13	5	5	5
4	3	4	1	2	8	4	2	5
4	3	2	1	3	6	3	3	5
5	4	3	0	5	5	4	3	3
3	2	3	2	2	3	8	5	9
0	2	5	2	4	4	3	2	5
3	2	7	1	3	5	1	2	10
2	6	4	1	2	3	3	4	8
1	4	4	2	4	9	2	2	5
0	4	5	0	4	4	1	2	5
3	3	3	1	7	2	4	1	12
1	3	2	3	5	3	2	2	6
2	2	4	0	7	4	5	4	4
1	1	5	2	5	5	0	1	5
1	0	5	0	0	3	3	2	7
1	0	0	0	4	6	3	4	17
3	2	2	1	7	0	2	3	9
2	5	2	0	6	5	2	1	3
5	4	2	0	7	5	6	5	6
4	1	7	0	0	0	0	0	3
2	2	1	0	5	3	4	7	7
1	1	2	1	3	4	3	2	7
8	6	11	7	9	15	6	5	11

3	3	3	1	5	3	1	3	4
4	5	4	4	5	12	3	7	5
3	1	1	2	3	5	1	3	8
1	0	5	1	2	3	0	0	4
2	2	2	0	4	4	3	4	5
4	4	2	1	6	5	3	5	0
2	3	3	0	1	0	0	2	2
11	0	0	11	0	0	0	0	0
4	3	5	1	3	3	1	3	2
1	2	7	4	2	3	1	1	4
1	2	1	0	3	2	1	0	4
0	2	3	2	2	0	0	0	2
0	3	4	0	3	4	3	2	5
1	0	3	1	5	1	3	4	2
19	20	6	10	19	16	19	17	10
1	0	3	1	9	5	2	3	1
4	3	5	0	7	7	5	2	10
2	4	3	1	6	6	3	3	3
18	10	37	18	6	11	7	0	13
4	2	3	0	3	3	3	5	5
2	1	2	0	3	4	5	2	7
3	3	2	1	5	5	2	2	6
3	4	3	1	3	2	3	2	2
3	4	1	1	7	5	2	1	5
0	2	1	1	2	4	2	2	6
5	3	3	0	4	6	4	4	2
3	2	3	1	5	7	5	3	6
2	4	1	0	4	2	4	3	8
0	4	2	0	4	5	5	5	4
1	2	1	0	4	8	0	0	5
0	1	3	0	3	1	1	1	6
1	2	1	1	7	5	1	1	5
9	4	7	8	8	8	10	12	9
3	5	5	2	1	3	2	5	2
0	1	5	1	2	3	0	1	7
2	0	3	0	4	2	2	4	7
2	1	2	0	6	4	3	2	8
0	0	4	2	2	1	5	3	4
1	4	2	0	2	3	2	3	9
18	19	6	0	20	15	19	19	7
1	0	1	1	6	5	1	2	8
6	3	7	0	3	7	2	6	11
0	2	0	1	5	4	4	2	4
1	1	9	1	0	0	0	1	7
0	2	0	2	0	0	0	1	0
2	0	2	0	2	1	1	1	7
1	0	2	2	1	2	1	4	7

0	5	3	0	3	0	2	2	9
0	0	0	0	1	4	2	3	6
0	1	5	0	1	1	1	0	4
0	1	1	0	3	5	0	0	4
1	0	1	1	1	2	0	0	7
1	0	2	0	5	5	1	1	8
0	0	5	1	0	0	0	0	5
0	1	2	1	0	0	0	0	1
0	0	0	0	5	4	3	3	7
1	1	1	0	1	1	1	3	8
9	0	1	5	2	0	0	2	3
1	0	0	0	4	5	5	4	3
0	0	0	0	4	2	1	3	11
0	1	2	1	1	1	1	0	5
0	3	11	10	4	4	6	10	7
0	1	1	0	5	3	1	0	4
3	2	4	3	3	5	1	2	3
0	0	0	0	0	0	0	4	0
0	23	21	26	0	16	0	15	36
0	0	2	0	2	0	0	0	7
8	8	12	0	10	12	9	0	11
19	19	6	8	0	14	22	18	7
5	4	3	2	0	0	1	0	0
0	0	15	24	7	8	0	20	12
0	11	0	0	15	15	0	18	45
0	0	2	0	5	8	0	0	10
6	0	4	5	0	0	0	0	6
0	0	16	0	10	0	0	0	16
16	10	0	0	14	25	20	21	0
15	0	0	0	15	24	0	26	16
18	13	0	0	0	11	0	0	12
0	0	1	0	0	0	1	0	7
0	4	6	7	8	10	0	10	0
0	12	0	0	0	0	0	0	0
0	0	4	0	0	0	0	0	10
5	0	0	0	0	0	5	0	7
0	0	2	0	8	5	0	3	0
0	13	0	0	0	0	7	0	0
0	0	0	0	0	0	0	0	0
0	0	10	0	12	0	10	0	9
0	7	0	0	0	0	7	0	0
7	3	0	0	0	0	8	0	0
0	6	0	9	0	0	0	0	0
8	0	7	6	0	0	0	10	0

		S.a.GN				VancATN		
G29	G30	G1	G2	G11	G12	G16	G17	G18
151	144	123	153	167	153	108	128	91
97	76	117	94	131	145	70	66	71
127	116	59	59	73	73	69	103	81
84	67	58	78	78	81	63	65	73
103	84	49	54	52	43	66	80	59
80	68	51	54	42	42	35	43	25
66	71	39	44	45	26	39	71	65
72	51	73	50	44	40	51	63	54
56	64	46	80	47	31	61	44	84
71	64	60	63	49	59	40	48	46
56	56	54	47	50	54	51	61	44
39	25	36	40	71	59	119	15	3
57	62	36	60	43	31	51	50	50
72	41	34	59	42	25	41	50	50
45	45	50	49	59	57	29	39	33
36	19	27	48	85	36	119	5	0
53	15	42	41	79	94	127	14	4
77	62	42	27	30	26	29	60	31
60	44	37	38	44	29	39	42	35
42	48	30	39	44	21	31	46	37
59	54	36	32	32	36	28	48	23
32	18	31	38	83	110	184	7	0
32	18	36	43	39	61	32	32	29
36	30	26	28	35	32	21	29	20
29	5	15	20	16	24	35	39	43
25	20	18	24	25	22	15	31	25
34	25	19	24	31	32	20	24	19
42	3	7	17	21	26	43	35	30
9	9	25	52	26	45	27	52	35
28	24	21	23	25	18	22	25	22
24	28	23	21	20	22	17	21	15
33	21	15	18	21	27	11	23	17
30	23	18	22	24	27	18	20	19
43	41	14	9	16	10	21	26	20
26	28	14	16	12	9	21	27	26
29	20	7	13	22	24	9	19	17
22	4	13	14	34	21	57	7	1
15	10	30	31	29	30	11	12	11
21	21	28	34	18	21	19	18	16
28	25	17	9	12	18	17	23	14
12	13	4	14	21	14	26	29	29

16	17	17	14	21	18	11	8	15
14	29	16	16	21	16	14	17	22
29	19	13	13	19	18	14	24	14
37	19	8	9	15	15	17	36	17
14	15	14	17	9	14	15	13	14
14	11	17	10	12	18	11	17	9
18	18	9	17	16	18	8	19	13
18	17	15	16	19	21	11	11	7
10	6	9	11	23	21	50	8	0
14	11	11	11	19	22	7	9	10
20	12	9	15	16	18	14	16	13
8	12	6	13	13	14	11	9	9
29	22	2	4	8	6	10	25	10
18	13	13	15	13	4	14	20	16
31	18	13	7	10	9	12	21	10
18	17	7	9	7	13	8	16	8
23	18	16	15	10	17	11	21	15
22	21	4	7	9	8	10	21	10
8	8	10	12	17	18	7	8	5
10	6	15	19	28	25	61	5	0
15	18	9	9	15	16	12	19	9
29	22	12	11	15	13	16	22	13
13	14	5	11	11	6	11	15	12
17	15	5	9	15	19	14	16	9
0	0	0	1	6	1	22	0	0
17	18	12	6	11	10	6	10	9
13	13	10	13	15	18	12	13	9
20	12	10	8	8	7	11	20	13
16	17	11	16	9	6	7	12	19
17	10	12	13	9	13	9	11	11
18	17	9	9	10	10	14	15	12
2	0	5	6	9	46	16	23	25
18	14	5	12	16	11	12	19	8
16	16	8	7	15	7	8	13	8
28	22	20	27	20	14	15	15	11
5	5	12	13	13	12	4	2	1
16	11	14	12	12	14	12	11	8
11	11	7	12	12	7	7	8	8
50	46	44	67	58	65	37	45	54
14	12	7	9	14	15	7	12	5
23	15	4	12	9	12	11	16	9
12	13	7	5	6	9	12	20	12
16	11	6	11	11	15	8	13	9
12	9	8	4	6	7	7	13	11
10	10	8	8	10	13	12	9	4
10	8	10	10	10	8	9	9	7
16	10	7	7	8	10	6	12	5

7	10	10	11	8	10	9	10	9
16	17	4	10	11	9	7	13	12
13	11	9	6	9	10	10	14	9
11	10	8	11	8	5	9	13	13
15	13	11	11	12	6	7	10	5
6	11	6	5	7	15	10	5	9
11	5	12	18	6	13	9	7	7
13	12	7	8	8	9	6	11	7
54	40	22	26	17	19	30	38	27
6	7	10	7	11	6	6	7	8
1	1	8	4	10	21	10	22	13
6	10	11	9	11	9	9	8	5
15	16	9	11	12	10	12	11	12
12	14	8	3	9	4	8	13	6
1	1	6	2	7	23	10	15	13
3	5	6	9	9	9	8	8	12
5	9	7	7	7	11	5	6	4
7	3	5	15	3	7	10	8	18
11	11	7	6	4	10	11	10	7
16	0	7	0	25	14	47	6	0
12	14	4	4	8	5	3	9	4
9	7	10	12	7	9	5	8	6
27	24	15	11	17	10	13	22	9
11	8	5	7	5	7	7	7	7
5	6	9	5	11	11	6	5	8
36	23	18	26	31	36	20	27	20
11	9	6	8	2	5	5	7	9
9	10	5	6	0	9	6	10	6
11	9	20	25	21	26	9	7	7
8	8	8	4	11	7	3	9	5
7	9	5	3	11	3	4	11	7
12	17	9	12	15	19	11	14	10
11	14	3	4	8	11	8	8	6
16	11	1	2	4	5	2	9	3
10	11	3	3	8	8	7	7	4
4	5	7	13	8	12	6	8	5
5	4	7	8	7	8	2	3	9
17	6	4	3	6	2	6	8	8
9	9	8	9	8	7	5	7	7
8	10	2	4	6	3	6	8	7
17	14	0	0	1	2	2	12	2
14	12	2	5	3	4	7	10	6
12	9	1	3	2	3	5	10	3
7	8	4	12	5	4	8	5	8
3	8	6	3	5	5	2	6	5
18	13	1	5	3	5	4	6	5
6	10	4	4	5	4	3	7	6

12	8	4	0	2	2	5	14	3
6	6	1	5	9	3	9	6	4
10	6	4	4	3	4	5	7	7
12	11	3	4	6	2	5	9	7
8	8	1	4	2	4	2	5	4
9	8	8	1	3	7	6	9	5
6	5	4	5	4	7	3	6	2
10	7	3	4	4	3	3	7	5
7	6	4	5	6	5	5	5	8
7	5	5	11	3	7	8	5	6
6	5	5	10	3	5	2	4	4
9	10	5	5	11	7	4	14	9
18	10	1	0	1	2	0	11	3
7	4	6	6	6	8	5	3	3
0	2	4	2	5	6	4	6	5
6	9	5	4	4	8	5	4	4
5	4	3	7	3	1	3	6	5
7	10	6	3	7	9	0	4	9
6	4	5	5	7	8	3	6	2
7	4	5	4	8	8	4	5	6
5	5	4	3	3	8	3	4	7
7	4	2	4	7	5	3	7	5
6	5	3	5	2	4	2	5	4
1	4	2	9	3	4	0	2	0
4	8	3	5	3	1	5	6	4
5	4	2	6	5	5	8	12	9
6	1	1	6	4	8	7	5	3
6	3	0	7	16	14	20	3	0
11	8	3	2	3	3	3	8	4
7	4	3	5	5	6	3	4	2
9	7	1	2	2	3	1	6	1
6	5	5	4	2	12	3	8	1
9	8	3	2	3	1	6	7	4
5	5	4	6	4	6	4	7	2
5	9	3	3	4	5	3	12	4
4	11	9	3	3	2	0	3	1
9	5	4	1	1	5	3	6	4
7	5	2	3	3	3	6	10	4
6	8	0	2	1	1	4	7	4
5	6	5	3	7	8	5	5	5
6	4	2	0	1	5	0	5	0
12	15	0	0	0	0	0	6	0
4	2	5	7	3	3	5	5	6
5	3	4	6	2	6	5	5	3
6	7	5	1	4	4	5	4	5
3	6	3	2	4	4	3	5	3
3	5	5	4	1	3	3	8	8

5	4	2	6	2	4	3	6	5
10	12	6	10	8	11	7	9	10
11	4	2	4	1	4	4	5	4
8	3	2	5	4	2	0	2	4
5	2	0	3	3	3	0	4	1
2	2	4	4	3	1	2	3	4
0	0	1	2	3	3	3	24	13
4	3	3	4	2	6	4	5	3
8	6	6	4	3	4	2	4	6
8	5	1	2	4	1	4	7	0
7	6	1	3	0	0	6	7	3
7	6	2	5	2	3	4	5	3
9	4	6	4	1	0	6	8	5
4	4	2	5	1	3	4	5	3
6	6	2	3	3	3	4	8	4
5	4	3	3	3	4	3	5	3
3	5	5	7	2	5	4	7	7
7	4	3	3	2	1	7	6	4
2	4	3	6	3	4	4	5	6
5	6	4	4	1	2	7	4	2
3	4	3	2	6	6	3	4	3
32	20	14	22	23	28	18	23	16
3	7	4	1	3	2	2	6	4
24	18	8	11	7	6	10	23	0
2	1	5	4	4	6	2	5	2
2	2	1	2	3	7	6	5	3
3	2	5	4	2	3	1	3	3
2	4	5	6	2	2	3	3	3
5	3	2	1	2	8	3	5	2
6	5	3	1	0	3	4	4	3
7	6	2	4	3	1	2	7	2
5	7	3	2	4	4	2	4	3
5	7	4	1	2	1	1	8	3
6	6	4	1	2	6	3	7	5
10	6	4	0	2	2	4	5	5
6	3	2	4	3	3	6	3	3
7	4	4	2	5	1	3	4	1
2	9	3	2	2	2	1	7	4
4	8	3	2	2	3	3	6	5
9	8	0	0	1	0	2	10	0
5	5	2	1	1	4	1	6	3
2	6	7	5	3	3	2	5	4
4	4	4	3	3	4	1	3	2
0	0	0	4	6	5	6	12	5
4	4	3	1	4	4	2	6	2
5	2	2	2	3	2	2	5	3
7	10	9	8	9	5	11	8	5

4	7	3	5	4	4	2	4	4
2	5	3	1	0	2	2	6	4
7	3	2	4	2	1	2	6	0
9	7	1	2	0	0	2	5	4
3	5	2	2	2	1	1	4	2
1	1	4	7	6	6	0	0	0
0	1	0	2	6	3	4	6	7
0	0	0	0	21	0	31	0	0
2	3	3	5	5	3	2	1	3
6	5	2	3	1	0	3	4	4
4	4	2	2	1	1	5	6	2
0	0	3	0	4	9	8	8	4
3	2	5	3	1	2	3	5	2
0	4	3	1	1	5	2	9	1
9	8	22	21	0	22	5	8	6
7	6	1	4	0	1	3	3	2
7	6	4	4	5	5	4	7	4
2	4	0	3	3	2	1	5	1
14	0	7	10	8	12	20	23	30
8	5	1	1	2	0	4	3	4
1	5	1	0	4	0	0	1	0
2	3	3	4	3	2	1	5	2
6	2	2	1	2	1	3	2	6
2	2	1	1	1	2	3	3	2
3	6	0	1	0	3	3	9	5
1	2	3	3	5	2	1	0	5
7	5	2	1	2	3	3	5	2
6	3	3	0	2	1	2	6	1
6	0	2	3	2	2	1	5	2
2	2	1	3	1	5	2	3	2
8	4	0	0	1	1	1	6	3
4	4	2	4	1	2	5	4	1
5	8	3	6	6	10	3	5	8
2	3	3	2	4	6	1	5	1
5	4	2	1	3	1	3	8	2
6	8	0	1	1	2	0	7	2
5	5	0	0	0	0	2	7	0
3	6	0	0	0	1	0	7	3
6	5	0	2	0	1	3	6	2
8	7	18	17	14	19	6	0	6
8	6	0	1	1	0	1	3	2
6	5	1	2	2	2	5	14	5
4	1	3	2	3	5	5	2	1
0	2	0	2	0	1	2	4	5
3	1	0	0	11	6	12	0	0
9	5	0	0	0	1	1	7	3
9	4	0	0	3	1	2	5	2

5	4	3	0	2	3	4	4	2
7	5	1	0	1	1	4	2	1
4	6	1	2	1	1	2	3	3
5	4	4	3	1	0	3	0	3
5	3	0	0	0	0	2	6	4
6	3	1	0	0	0	1	2	1
0	1	0	1	0	0	3	8	8
0	0	1	3	4	4	5	7	4
9	3	0	0	0	1	0	1	0
3	5	2	1	1	0	0	5	1
2	0	0	5	4	7	4	3	12
5	4	0	2	1	1	0	3	0
5	5	0	0	0	0	0	5	0
5	3	2	1	0	0	2	5	2
8	11	3	4	4	3	6	7	7
9	5	0	1	0	0	3	2	1
0	0	4	5	5	4	0	2	1
5	0	0	0	19	0	18	0	0
16	0	18	27	23	20	0	19	20
10	8	0	0	0	0	0	1	0
18	11	8	11	10	12	9	12	10
6	6	16	15	16	17	6	5	8
0	0	0	1	4	4	1	1	6
9	11	7	13	0	0	9	9	0
23	15	0	15	17	16	11	14	0
14	6	0	0	1	0	0	11	5
6	5	3	1	0	0	3	5	5
18	0	10	0	0	0	11	9	0
0	0	0	10	19	22	0	0	0
15	0	0	0	0	0	0	10	12
0	0	0	12	13	17	13	0	8
5	5	0	0	0	0	0	0	0
7	4	5	0	6	6	5	0	7
8	0	0	0	0	17	22	0	0
9	5	0	0	0	0	5	3	5
10	9	0	0	0	0	7	7	6
5	0	0	0	0	5	0	6	0
9	5	0	11	23	0	51	0	0
0	0	0	0	0	6	7	5	5
12	0	0	0	0	11	7	9	9
8	0	5	5	10	8	0	6	0
0	0	6	0	0	9	0	0	0
0	6	0	0	0	0	32	8	3
0	0	0	0	0	0	0	0	0

ATN			IgAN E0		IgA E1			
G24.norm	G25.norm	G26.norm	G8.norm	G10.norm	G4.norm	G21.norm	G23.norm	G5.norm
102.35	114.42	159.03	128.35	164.8	162.22	129.06	150.09	145.21
90.8	61.33	119.93	115.97	139.08	146.11	67.73	145.58	91.71
79.24	73.23	106.89	74.31	85.73	82.72	86.96	120.07	79.82
62.73	58.58	88.64	86.69	77.16	76.28	66.82	88.55	50.95
61.08	73.23	43.02	61.92	53.35	59.09	65.9	52.53	61.14
34.67	43.02	40.41	45.04	38.1	53.72	53.09	36.02	67.93
51.18	47.6	65.18	48.41	53.35	49.42	60.41	67.54	52.65
84.19	56.75	31.29	64.18	42.87	42.97	45.77	42.02	56.89
49.53	76.89	58.66	50.66	28.58	47.27	54	69.04	45.85
28.06	35.7	43.02	48.41	54.3	58.01	72.31	51.03	44.16
56.13	56.75	57.36	54.04	44.77	54.79	54.92	57.03	52.65
64.38	21.05	6.52	32.65	29.53	56.94	21.05	118.57	18.68
59.43	49.43	59.96	52.92	54.3	47.27	54	54.03	45.85
36.32	55.84	19.55	37.15	43.82	38.68	41.19	51.03	34.82
47.87	31.12	45.62	50.66	66.68	52.64	44.85	66.04	43.31
59.43	10.98	0	29.27	2.86	37.6	8.24	105.06	16.98
52.83	28.38	11.73	40.53	56.2	51.57	19.22	82.55	16.98
23.11	32.04	15.64	27.02	28.58	34.38	35.7	25.51	42.46
39.62	36.62	45.62	48.41	47.63	34.38	40.27	48.03	27.17
29.72	37.53	48.23	39.41	41.91	36.53	43.94	46.53	29.72
26.41	31.12	29.98	36.03	33.34	40.82	36.61	25.51	38.21
28.06	26.55	11.73	27.02	46.68	44.05	15.56	60.03	12.74
16.51	22.88	32.59	25.9	54.3	40.82	34.78	54.03	25.47
23.11	28.38	23.46	23.64	26.67	38.68	17.39	25.51	24.63
14.86	19.22	48.23	24.77	27.63	21.49	47.6	51.03	11.89
9.91	21.97	22.16	25.9	32.39	26.86	22.88	19.51	21.23
41.27	21.97	19.55	22.52	23.82	19.34	24.71	24.01	25.47
8.25	17.39	22.16	22.52	33.34	23.63	38.44	27.02	11.04
8.25	19.22	26.07	34.9	51.44	36.53	45.77	24.01	5.94
28.06	20.14	24.77	28.15	21.91	27.93	20.14	19.51	24.63
19.81	20.14	28.68	23.64	23.82	24.71	21.97	30.02	17.83
14.86	21.97	22.16	15.76	30.48	17.19	24.71	25.51	22.93
36.32	25.63	23.46	19.14	20	20.41	22.88	19.51	19.53
19.81	19.22	13.04	12.38	19.05	17.19	15.56	9.01	24.63
26.41	27.46	22.16	20.27	16.19	17.19	23.8	30.02	20.38
9.91	13.73	15.64	13.51	18.1	13.97	12.81	21.01	16.98
19.81	6.41	2.61	14.64	5.72	20.41	5.49	36.02	4.25
16.51	14.65	15.64	28.15	25.72	31.16	13.73	21.01	22.08
9.91	14.65	24.77	22.52	29.53	21.49	22.88	19.51	16.98
16.51	15.56	13.04	18.01	14.29	12.89	10.98	10.51	28.87
9.91	22.88	46.93	14.64	24.77	12.89	24.71	36.02	11.04

26.41	11.9	23.46	20.27	19.05	20.41	11.9	27.02	16.13
21.46	17.39	20.86	14.64	15.24	21.49	21.97	39.02	9.34
28.06	16.48	11.73	12.38	15.24	15.04	15.56	19.51	14.44
23.11	15.56	11.73	9.01	10.48	11.82	11.9	18.01	15.28
23.11	10.98	9.12	14.64	12.38	15.04	10.98	12.01	11.89
47.87	10.98	14.34	12.38	9.53	10.74	8.24	15.01	19.53
11.56	10.07	14.34	15.76	14.29	12.89	9.15	6	16.13
4.95	10.98	18.25	19.14	20.96	11.82	17.39	18.01	11.89
11.56	8.24	0	0	12.38	13.97	5.49	28.52	0
16.51	7.32	18.25	10.13	13.34	10.74	10.98	21.01	12.74
4.95	18.31	14.34	5.63	12.38	13.97	10.98	6	11.04
18.16	9.15	20.86	7.88	26.67	15.04	13.73	39.02	5.09
14.86	9.15	13.04	10.13	8.57	8.59	10.98	7.5	8.49
6.6	19.22	16.95	10.13	6.67	9.67	10.98	18.01	10.19
8.25	7.32	7.82	9.01	8.57	8.59	14.65	9.01	11.04
19.81	16.48	6.52	7.88	6.67	7.52	10.07	10.51	12.74
24.76	20.14	14.34	14.64	10.48	13.97	11.9	6	15.28
3.3	10.98	9.12	6.76	6.67	5.37	10.98	7.5	13.59
9.91	6.41	9.12	12.38	15.24	13.97	6.41	12.01	10.19
16.51	10.98	0	12.38	12.38	17.19	5.49	27.02	4.25
19.81	13.73	11.73	5.63	7.62	7.52	9.15	10.51	10.19
19.81	13.73	10.43	10.13	7.62	12.89	14.65	13.51	11.04
21.46	10.98	16.95	7.88	10.48	7.52	11.9	15.01	15.28
8.25	14.65	14.34	4.5	9.53	11.82	9.15	7.5	12.74
72.64	0	0	0	0	2.15	0	1.5	0
13.21	10.07	18.25	14.64	12.38	11.82	7.32	9.01	16.98
6.6	10.07	11.73	6.76	15.24	12.89	12.81	15.01	11.89
9.91	6.41	11.73	14.64	6.67	12.89	8.24	4.5	14.44
3.3	18.31	14.34	7.88	4.76	7.52	10.98	16.51	6.79
11.56	10.98	6.52	11.26	9.53	15.04	8.24	10.51	8.49
8.25	12.82	9.12	6.76	5.72	12.89	9.15	6	12.74
4.95	10.07	15.64	11.26	19.05	24.71	14.65	12.01	0.85
8.25	11.9	6.52	10.13	4.76	15.04	7.32	6	16.13
8.25	7.32	6.52	4.5	10.48	15.04	6.41	4.5	12.74
11.56	17.39	15.64	13.51	25.72	18.26	15.56	12.01	19.53
9.91	2.75	7.82	11.26	11.43	12.89	2.75	16.51	7.64
8.25	9.15	9.12	11.26	7.62	12.89	9.15	4.5	11.89
8.25	13.73	9.12	6.76	13.34	7.52	9.15	18.01	7.64
44.57	37.53	58.66	70.93	57.16	56.94	52.17	63.04	37.36
13.21	5.49	1.3	9.01	6.67	8.59	7.32	4.5	7.64
3.3	10.98	7.82	7.88	9.53	10.74	9.15	6	5.94
11.56	10.98	2.61	10.13	3.81	6.45	2.75	1.5	11.04
9.91	11.9	2.61	7.88	7.62	6.45	10.98	7.5	10.19
9.91	7.32	13.04	13.51	13.34	13.97	8.24	6	8.49
8.25	6.41	11.73	7.88	8.57	8.59	8.24	9.01	7.64
13.21	6.41	6.52	11.26	12.38	10.74	8.24	15.01	6.79
9.91	6.41	11.73	9.01	7.62	8.59	8.24	6	11.89

6.6	7.32	11.73	9.01	6.67	8.59	7.32	10.51	6.79
13.21	9.15	18.25	10.13	11.43	8.59	15.56	15.01	11.89
13.21	10.07	10.43	9.01	6.67	6.45	4.58	6	6.79
3.3	9.15	5.21	7.88	5.72	6.45	10.98	6	5.94
11.56	10.07	6.52	10.13	9.53	7.52	11.9	7.5	12.74
13.21	10.07	2.61	6.76	11.43	8.59	2.75	4.5	5.09
3.3	12.82	0	7.88	4.76	13.97	12.81	3	4.25
8.25	9.15	1.3	18.01	10.48	6.45	7.32	1.5	8.49
29.72	28.38	22.16	14.64	25.72	19.34	32.95	28.52	22.08
4.95	10.98	5.21	10.13	5.72	10.74	5.49	3	12.74
0	8.24	11.73	10.13	26.67	13.97	10.98	24.01	2.55
6.6	9.15	13.04	10.13	7.62	8.59	8.24	10.51	10.19
14.86	10.98	15.64	13.51	15.24	11.82	13.73	16.51	10.19
8.25	8.24	6.52	3.38	6.67	7.52	10.07	4.5	7.64
0	6.41	11.73	5.63	22.86	24.71	12.81	16.51	1.7
1.65	11.9	14.34	6.76	18.1	10.74	10.07	13.51	5.09
4.95	7.32	3.91	6.76	5.72	6.45	5.49	4.5	6.79
0	10.07	15.64	4.5	8.57	1.07	7.32	7.5	2.55
8.25	8.24	6.52	4.5	5.72	7.52	6.41	6	7.64
0	0	0	9.01	0	15.04	0	37.52	0
3.3	9.15	7.82	5.63	4.76	6.45	9.15	4.5	8.49
4.95	4.58	1.3	12.38	5.72	9.67	4.58	6	7.64
16.51	16.48	13.04	14.64	17.15	13.97	5.49	15.01	17.83
19.81	5.49	7.82	5.63	5.72	5.37	7.32	12.01	5.94
8.25	6.41	3.91	9.01	8.57	7.52	7.32	4.5	5.09
36.32	22.88	19.55	20.27	25.72	20.41	26.54	21.01	25.47
4.95	4.58	1.3	6.76	9.53	6.45	6.41	9.01	8.49
3.3	5.49	6.52	5.63	1.91	4.3	10.07	4.5	9.34
11.56	10.07	14.34	24.77	17.15	25.78	14.65	10.51	20.38
4.95	4.58	6.52	5.63	7.62	2.15	3.66	3	7.64
4.95	6.41	9.12	5.63	7.62	5.37	10.98	3	2.55
19.81	12.82	13.04	11.26	7.62	13.97	12.81	9.01	11.89
3.3	10.98	1.3	5.63	3.81	4.3	9.15	1.5	6.79
1.65	6.41	2.61	2.25	1.91	5.37	4.58	0	7.64
3.3	8.24	3.91	2.25	3.81	3.22	8.24	6	9.34
0	9.15	2.61	7.88	4.76	7.52	8.24	1.5	5.09
1.65	4.58	6.52	4.5	12.38	9.67	6.41	6	4.25
4.95	6.41	11.73	3.38	5.72	2.15	8.24	9.01	6.79
6.6	7.32	3.91	5.63	4.76	9.67	7.32	4.5	7.64
8.25	6.41	3.91	2.25	2.86	5.37	11.9	6	4.25
1.65	8.24	3.91	1.13	0.95	0	6.41	4.5	11.89
6.6	8.24	2.61	1.13	3.81	3.22	7.32	1.5	5.94
9.91	5.49	6.52	1.13	4.76	5.37	5.49	3	9.34
8.25	3.66	1.3	3.38	5.72	4.3	6.41	7.5	4.25
6.6	5.49	10.43	7.88	2.86	5.37	3.66	9.01	6.79
3.3	5.49	3.91	1.13	5.72	1.07	4.58	3	8.49
3.3	6.41	6.52	4.5	6.67	3.22	6.41	1.5	5.94

3.3	5.49	1.3	4.5	0.95	3.22	2.75	1.5	9.34
9.91	6.41	7.82	5.63	5.72	8.59	5.49	6	8.49
4.95	6.41	2.61	5.63	4.76	2.15	8.24	3	9.34
3.3	5.49	1.3	4.5	2.86	7.52	10.07	1.5	4.25
3.3	6.41	3.91	2.25	5.72	2.15	6.41	6	5.09
8.25	5.49	1.3	6.76	1.91	5.37	5.49	1.5	8.49
3.3	3.66	0	3.38	1.91	3.22	2.75	1.5	5.09
4.95	7.32	2.61	5.63	3.81	4.3	7.32	9.01	5.09
4.95	4.58	5.21	5.63	4.76	4.3	4.58	1.5	5.09
1.65	6.41	5.21	5.63	5.72	2.15	8.24	4.5	2.55
1.65	3.66	2.61	4.5	3.81	6.45	5.49	3	0.85
4.95	6.41	13.04	5.63	6.67	5.37	12.81	0	13.59
0	4.58	6.52	4.5	0.95	3.22	1.83	0	8.49
6.6	1.83	3.91	5.63	5.72	5.37	2.75	6	4.25
0	2.75	10.43	7.88	20	5.37	4.58	12.01	2.55
11.56	3.66	5.21	5.63	5.72	4.3	2.75	7.5	11.04
1.65	3.66	2.61	4.5	4.76	3.22	6.41	3	7.64
3.3	4.58	7.82	6.76	4.76	9.67	5.49	6	4.25
6.6	2.75	0	2.25	4.76	7.52	2.75	0	8.49
3.3	1.83	1.3	5.63	3.81	4.3	3.66	9.01	7.64
4.95	4.58	3.91	3.38	4.76	3.22	5.49	3	5.94
4.95	7.32	7.82	1.13	2.86	2.15	3.66	6	4.25
1.65	1.83	2.61	4.5	1.91	2.15	3.66	0	5.94
0	5.49	0	0	1.91	5.37	4.58	3	5.94
6.6	5.49	10.43	4.5	2.86	3.22	3.66	10.51	3.4
3.3	3.66	1.3	3.38	6.67	6.45	1.83	1.5	2.55
0	9.15	1.3	0	0	1.07	2.75	0	3.4
23.11	2.75	0	0	0	10.74	0	12.01	0
1.65	1.83	2.61	4.5	3.81	2.15	2.75	1.5	5.94
1.65	1.83	5.21	3.38	3.81	5.37	2.75	9.01	5.09
1.65	0.92	2.61	1.13	2.86	2.15	2.75	0	8.49
0	0.92	2.61	0	1.91	6.45	2.75	4.5	6.79
6.6	8.24	3.91	2.25	3.81	2.15	5.49	7.5	0.85
3.3	5.49	2.61	4.5	0.95	6.45	5.49	1.5	5.09
3.3	6.41	1.3	1.13	3.81	5.37	2.75	6	8.49
0	0.92	1.3	10.13	10.48	6.45	3.66	3	4.25
3.3	2.75	2.61	4.5	5.72	2.15	0.92	4.5	5.94
1.65	7.32	0	2.25	3.81	4.3	3.66	1.5	3.4
3.3	8.24	0	2.25	0.95	1.07	3.66	6	1.7
8.25	4.58	7.82	5.63	7.62	7.52	3.66	7.5	5.94
14.86	2.75	2.61	3.38	0.95	3.22	2.75	0	4.25
0	3.66	0	2.25	0.95	0	0.92	0	8.49
3.3	2.75	3.91	4.5	11.43	5.37	3.66	7.5	2.55
1.65	3.66	2.61	2.25	0.95	3.22	2.75	3	3.4
3.3	3.66	2.61	4.5	3.81	5.37	4.58	1.5	2.55
4.95	6.41	1.3	4.5	3.81	5.37	2.75	3	3.4
3.3	3.66	2.61	2.25	4.76	6.45	6.41	9.01	0.85

1.65	7.32	6.52	2.25	1.91	3.22	1.83	10.51	1.7
11.56	6.41	14.34	10.13	14.29	6.45	12.81	13.51	7.64
1.65	2.75	0	2.25	0.95	0	2.75	1.5	5.09
0	3.66	1.3	2.25	4.76	3.22	3.66	3	1.7
1.65	1.83	1.3	0	1.91	2.15	3.66	1.5	5.09
3.3	1.83	11.73	4.5	5.72	2.15	0.92	6	3.4
0	6.41	19.55	1.13	0	1.07	1.83	4.5	1.7
0	1.83	1.3	2.25	1.91	3.22	2.75	3	3.4
1.65	4.58	2.61	2.25	1.91	3.22	2.75	0	1.7
0	1.83	2.61	3.38	2.86	1.07	5.49	0	4.25
3.3	3.66	2.61	2.25	1.91	1.07	4.58	1.5	2.55
4.95	4.58	2.61	2.25	1.91	2.15	4.58	1.5	2.55
3.3	7.32	5.21	2.25	3.81	2.15	2.75	1.5	2.55
4.95	4.58	1.3	1.13	1.91	2.15	3.66	0	5.94
3.3	4.58	5.21	1.13	1.91	1.07	3.66	6	1.7
11.56	2.75	2.61	2.25	1.91	4.3	1.83	1.5	2.55
3.3	5.49	3.91	4.5	0.95	1.07	5.49	0	3.4
4.95	5.49	2.61	2.25	3.81	2.15	3.66	3	2.55
6.6	2.75	3.91	4.5	3.81	1.07	4.58	4.5	2.55
6.6	2.75	3.91	4.5	0.95	2.15	5.49	7.5	5.09
6.6	2.75	5.21	1.13	4.76	4.3	1.83	1.5	4.25
39.62	20.14	0	19.14	20.96	17.19	19.22	16.51	27.17
4.95	2.75	0	3.38	3.81	1.07	1.83	0	3.4
8.25	12.82	10.43	9.01	8.57	10.74	5.49	12.01	16.98
1.65	0.92	1.3	5.63	2.86	4.3	0	1.5	2.55
3.3	3.66	3.91	1.13	3.81	3.22	3.66	1.5	1.7
1.65	2.75	3.91	4.5	3.81	3.22	1.83	1.5	2.55
1.65	3.66	0	2.25	4.76	4.3	2.75	0	4.25
3.3	2.75	3.91	3.38	2.86	2.15	2.75	3	1.7
4.95	2.75	2.61	1.13	0	2.15	4.58	3	3.4
0	2.75	0	1.13	2.86	2.15	6.41	1.5	2.55
1.65	2.75	1.3	3.38	1.91	6.45	3.66	1.5	1.7
1.65	5.49	1.3	1.13	0.95	4.3	3.66	3	3.4
6.6	4.58	0	4.5	0	4.3	4.58	0	3.4
1.65	3.66	2.61	0	2.86	3.22	2.75	1.5	5.94
4.95	4.58	2.61	3.38	0.95	3.22	1.83	4.5	4.25
1.65	5.49	6.52	2.25	1.91	2.15	3.66	0	5.94
0	6.41	1.3	3.38	0.95	1.07	4.58	3	4.25
0	7.32	2.61	1.13	0.95	0	4.58	0	0
0	0	0	0	0.95	0	0	0	3.4
1.65	1.83	2.61	3.38	2.86	2.15	1.83	1.5	5.94
0	2.75	0	2.25	1.91	5.37	1.83	0	5.09
0	2.75	1.3	3.38	4.76	4.3	1.83	0	5.94
0	2.75	2.61	1.13	3.81	1.07	6.41	0	0
6.6	2.75	0	4.5	1.91	2.15	0.92	0	4.25
3.3	2.75	2.61	3.38	0.95	1.07	1.83	1.5	2.55
6.6	10.07	11.73	11.26	7.62	6.45	10.07	10.51	7.64

1.65	5.49	0	3.38	2.86	3.22	2.75	1.5	4.25
0	3.66	3.91	4.5	3.81	5.37	3.66	6	4.25
1.65	2.75	2.61	1.13	2.86	1.07	0.92	3	2.55
1.65	0.92	0	2.25	0.95	0	4.58	1.5	1.7
13.21	0.92	1.3	4.5	1.91	2.15	1.83	0	3.4
0	1.83	0	3.38	3.81	4.3	1.83	1.5	5.09
0	2.75	3.91	2.25	1.91	3.22	2.75	0	0.85
0	0	0	0	10.48	0	0	16.51	0
1.65	2.75	3.91	1.13	3.81	3.22	4.58	1.5	2.55
1.65	3.66	1.3	1.13	0.95	2.15	6.41	6	1.7
8.25	1.83	1.3	2.25	0.95	2.15	0.92	0	2.55
0	0.92	9.12	1.13	0	2.15	2.75	3	1.7
3.3	1.83	1.3	0	0	3.22	3.66	0	2.55
0	2.75	1.3	3.38	0.95	0	2.75	1.5	4.25
9.91	9.15	9.12	20.27	18.1	21.49	5.49	15.01	16.13
3.3	4.58	2.61	1.13	0.95	0	2.75	1.5	7.64
4.95	5.49	2.61	3.38	3.81	3.22	4.58	0	5.94
0	3.66	1.3	0	1.91	4.3	2.75	1.5	5.09
8.25	13.73	23.46	14.64	17.15	10.74	33.87	27.02	5.09
1.65	2.75	1.3	1.13	3.81	2.15	2.75	0	2.55
0	0.92	0	6.76	1.91	1.07	1.83	0	2.55
0	0.92	3.91	3.38	2.86	3.22	1.83	1.5	4.25
0	4.58	0	2.25	2.86	4.3	2.75	1.5	2.55
0	1.83	1.3	1.13	2.86	4.3	0.92	1.5	5.94
0	2.75	1.3	3.38	0	2.15	0.92	1.5	1.7
0	3.66	0	1.13	4.76	3.22	2.75	0	3.4
0	1.83	0	5.63	2.86	2.15	2.75	1.5	4.25
0	0.92	1.3	2.25	1.91	4.3	0.92	0	3.4
1.65	0.92	0	1.13	0	4.3	1.83	0	3.4
1.65	1.83	0	2.25	0.95	2.15	0.92	0	3.4
0	4.58	2.61	0	0	1.07	2.75	0	2.55
8.25	0.92	0	3.38	0.95	2.15	0.92	1.5	5.94
8.25	3.66	10.43	7.88	8.57	4.3	6.41	12.01	6.79
8.25	2.75	2.61	2.25	2.86	5.37	4.58	3	0.85
1.65	5.49	1.3	1.13	0	1.07	4.58	1.5	1.7
0	1.83	0	1.13	1.91	0	2.75	0	3.4
0	1.83	0	1.13	1.91	1.07	1.83	0	5.09
4.95	5.49	0	1.13	0	0	3.66	3	1.7
0	2.75	2.61	2.25	0.95	4.3	1.83	0	1.7
0	0	0	16.89	17.15	20.41	5.49	0	16.98
0	0.92	0	0	0.95	0	0.92	1.5	5.09
1.65	5.49	2.61	1.13	5.72	3.22	6.41	0	2.55
1.65	0.92	0	1.13	0	2.15	0	1.5	4.25
0	2.75	5.21	3.38	0.95	1.07	8.24	1.5	0
3.3	0	0	0	0	2.15	0	3	0
1.65	1.83	0	0	1.91	0	1.83	0	1.7
1.65	0.92	5.21	1.13	0.95	0	1.83	3	0.85

3.3	2.75	0	1.13	0	5.37	2.75	0	2.55
0	0	0	2.25	0	0	0	0	0.85
0	5.49	0	1.13	0	1.07	4.58	0	0.85
0	4.58	0	2.25	0	1.07	0.92	0	2.55
6.6	2.75	2.61	1.13	0.95	0	0.92	1.5	0.85
0	2.75	0	2.25	0.95	0	1.83	0	4.25
0	1.83	7.82	0	0	0	4.58	1.5	0
0	4.58	5.21	0	0	1.07	1.83	1.5	0
0	0	0	0	0	0	0	0	4.25
1.65	3.66	0	0	0.95	1.07	0.92	0	0.85
0	0	0	4.5	8.57	0	0.92	7.5	1.7
0	5.49	0	0	0.95	0	0	0	3.4
0	0	0	0	0	0	0	0	3.4
6.6	2.75	3.91	1.13	0	1.07	1.83	1.5	0.85
9.91	5.49	11.73	5.63	0	3.22	10.07	15.01	3.4
0	0.92	1.3	1.13	0	1.07	0.92	0	4.25
9.91	4.58	0	6.76	2.86	2.15	3.66	4.5	2.55
4.95	4.58	0	0	0	0	0	0	0
19.81	0	24.77	0	0	24.71	19.22	39.02	0
0	0	0	0	0	0	1.83	0	1.7
0	11.9	5.21	9.01	7.62	8.59	10.98	0	8.49
6.6	5.49	7.82	16.89	18.1	20.41	5.49	12.01	0
0	6.41	3.91	1.13	4.76	4.3	2.75	3	0
18.16	7.32	22.16	7.88	0	0	13.73	36.02	5.94
31.37	15.56	0	0	0	11.82	0	0	12.74
3.3	0	0	0	0	0	1.83	0	4.25
3.3	2.75	5.21	3.38	5.72	0	3.66	7.5	0
14.86	13.73	18.25	0	0	0	14.65	0	8.49
0	0	0	9.01	15.24	10.74	0	0	11.89
0	0	0	0	14.29	0	0	0	12.74
0	0	0	9.01	17.15	13.97	0	0	0
0	0	0	0	0	0	0.92	0	0
0	0	2.61	5.63	0	4.3	5.49	10.51	6.79
0	0	0	0	0	12.89	0	0	0
3.3	3.66	0	0	0	0	3.66	0	0
6.6	7.32	9.12	0	4.76	0	0	0	0
4.95	0	0	0	0	0	1.83	0	6.79
0	8.24	0	0	0	13.97	0	0	0
0	2.75	3.91	2.25	0	0	0	0	0
11.56	12.82	11.73	0	0	0	9.15	0	10.19
0	4.58	0	0	0	7.52	0	0	0
3.3	0	2.61	4.5	6.67	3.22	0	0	0
0	6.41	0	0	0	6.45	0	13.51	0
0	0	0	0	7.62	0	6.41	9.01	0

Normalized Counts								
NLTx						S.a.GN		
G6.norm	G13.norm	G14.norm	G28.norm	G29.norm	G30.norm	G1.norm	G2.norm	G11.norm
172.91	157.87	173.42	110.37	105.65	115.66	139.49	157.89	159.82
92.22	110.12	130.68	143.16	67.87	61.04	132.69	97	125.37
87.89	88.68	87.12	128.66	88.85	93.17	66.91	60.88	69.86
51.15	66.27	60	120.46	58.77	53.81	65.78	80.49	74.65
64.12	59.45	49.31	64.33	72.06	67.47	55.57	55.72	49.76
72.04	40.93	59.18	67.48	55.97	54.62	57.84	55.72	40.19
55.47	63.34	69.04	50.45	46.18	57.03	44.23	45.41	43.07
63.4	38.01	42.74	61.18	50.37	40.96	82.79	51.6	42.11
55.47	41.9	50.96	63.07	39.18	51.4	52.17	82.56	44.98
48.27	47.75	49.31	47.3	49.67	51.4	68.04	65.01	46.89
40.34	57.5	50.96	44.78	39.18	44.98	61.24	48.5	47.85
13.69	33.13	28.77	10.09	27.29	20.08	40.83	41.28	67.95
39.62	47.75	47.67	41.62	39.88	49.8	40.83	61.92	41.15
42.51	41.9	41.09	52.35	50.37	32.93	38.56	60.88	40.19
39.62	55.55	51.78	25.86	31.48	36.14	56.7	50.57	56.46
15.13	23.39	32.05	1.89	25.19	15.26	30.62	49.53	81.35
6.48	44.83	54.24	16.4	37.08	12.05	47.63	42.31	75.6
48.27	50.68	43.56	52.35	53.87	49.8	47.63	27.86	28.71
37.46	38.98	38.63	30.9	41.98	35.34	41.96	39.21	42.11
38.9	38.98	50.14	31.53	29.38	38.55	34.02	40.25	42.11
38.9	39.96	35.34	37.84	41.28	43.37	40.83	33.02	30.62
2.88	28.26	40.27	8.2	22.39	14.46	35.16	39.21	79.43
31.7	25.34	34.52	29.01	22.39	14.46	40.83	44.37	37.32
26.66	28.26	29.59	24.6	25.19	24.1	29.49	28.89	33.5
20.17	11.69	9.86	16.4	20.29	4.02	17.01	20.64	15.31
36.02	25.34	32.05	22.7	17.49	16.06	20.41	24.77	23.93
27.38	25.34	29.59	22.7	23.79	20.08	21.55	24.77	29.67
30.98	8.77	19.73	19.55	29.38	2.41	7.94	17.54	20.1
4.32	6.82	4.93	10.72	6.3	7.23	28.35	53.66	24.88
24.49	31.18	28.77	18.92	19.59	19.28	23.82	23.73	23.93
12.97	27.29	21.37	19.55	16.79	22.49	26.08	21.67	19.14
23.05	27.29	29.59	24.6	23.09	16.87	17.01	18.57	20.1
15.13	25.34	21.37	30.9	20.99	18.47	20.41	22.7	22.97
20.89	26.31	18.9	24.6	30.08	32.93	15.88	9.29	15.31
21.61	15.59	18.08	15.77	18.19	22.49	15.88	16.51	11.48
15.85	22.41	26.3	47.93	20.29	16.06	7.94	13.42	21.05
0.72	6.82	9.86	1.89	15.39	3.21	14.74	14.45	32.54
18.73	26.31	23.01	8.2	10.49	8.03	34.02	31.99	27.75
18.73	21.44	18.08	11.98	14.69	16.87	31.75	35.09	17.23
23.05	23.39	27.12	23.33	19.59	20.08	19.28	9.29	11.48
5.04	8.77	8.22	28.38	8.4	10.44	4.54	14.45	20.1

18.01	27.29	24.66	10.09	11.19	13.65	19.28	14.45	20.1
12.97	15.59	13.15	11.35	9.79	23.29	18.15	16.51	20.1
17.29	12.67	15.62	17.66	20.29	15.26	14.74	13.42	18.18
17.29	10.72	11.51	27.12	25.89	15.26	9.07	9.29	14.36
16.57	15.59	15.62	13.87	9.79	12.05	15.88	17.54	8.61
14.41	7.8	10.68	13.24	9.79	8.83	19.28	10.32	11.48
14.41	17.54	18.08	13.24	12.59	14.46	10.21	17.54	15.31
10.81	22.41	14.79	7.57	12.59	13.65	17.01	16.51	18.18
0	6.82	9.86	0	7	4.82	10.21	11.35	22.01
18.01	20.47	17.26	6.94	9.79	8.83	12.47	11.35	18.18
15.85	12.67	13.97	8.83	13.99	9.64	10.21	15.48	15.31
6.48	9.75	14.79	7.57	5.6	9.64	6.8	13.42	12.44
12.97	10.72	7.4	22.07	20.29	17.67	2.27	4.13	7.66
15.13	11.69	11.51	15.77	12.59	10.44	14.74	15.48	12.44
18.01	10.72	12.33	15.14	21.69	14.46	14.74	7.22	9.57
10.81	13.64	18.08	10.72	12.59	13.65	7.94	9.29	6.7
9.37	12.67	11.51	13.24	16.09	14.46	18.15	15.48	9.57
15.13	11.69	9.04	17.03	15.39	16.87	4.54	7.22	8.61
7.92	17.54	13.15	6.31	5.6	6.43	11.34	12.38	16.27
3.6	8.77	13.15	0	7	4.82	17.01	19.61	26.8
8.65	12.67	9.04	11.98	10.49	14.46	10.21	9.29	14.36
8.65	13.64	12.33	15.14	20.29	17.67	13.61	11.35	14.36
10.09	13.64	14.79	13.87	9.1	11.24	5.67	11.35	10.53
10.81	14.62	10.68	11.98	11.89	12.05	5.67	9.29	14.36
0	0	0	0	0	0	0	1.03	5.74
12.25	16.57	10.68	8.83	11.89	14.46	13.61	6.19	10.53
9.37	8.77	16.44	10.72	9.1	10.44	11.34	13.42	14.36
13.69	8.77	10.68	19.55	13.99	9.64	11.34	8.26	7.66
12.25	4.87	10.68	13.24	11.19	13.65	12.47	16.51	8.61
10.09	12.67	7.4	8.2	11.89	8.03	13.61	13.42	8.61
10.09	7.8	9.04	10.72	12.59	13.65	10.21	9.29	9.57
0	0.97	0.82	5.05	1.4	0	5.67	6.19	8.61
12.97	11.69	10.68	15.14	12.59	11.24	5.67	12.38	15.31
14.41	17.54	10.68	9.46	11.19	12.85	9.07	7.22	14.36
25.22	11.69	13.15	19.55	19.59	17.67	22.68	27.86	19.14
10.09	8.77	9.04	3.15	3.5	4.02	13.61	13.42	12.44
7.92	5.85	9.86	8.83	11.19	8.83	15.88	12.38	11.48
10.09	9.75	8.22	8.83	7.7	8.83	7.94	12.38	11.48
42.51	50.68	36.99	46.04	34.98	36.95	49.9	69.14	55.51
13.69	11.69	11.51	10.09	9.79	9.64	7.94	9.29	13.4
10.81	6.82	11.51	11.98	16.09	12.05	4.54	12.38	8.61
10.81	5.85	8.22	13.87	8.4	10.44	7.94	5.16	5.74
6.48	8.77	14.79	13.24	11.19	8.83	6.8	11.35	10.53
11.53	14.62	9.04	8.2	8.4	7.23	9.07	4.13	5.74
10.09	13.64	13.97	7.57	7	8.03	9.07	8.26	9.57
6.48	14.62	15.62	9.46	7	6.43	11.34	10.32	9.57
9.37	7.8	8.22	10.72	11.19	8.03	7.94	7.22	7.66

7.2	4.87	4.93	6.31	4.9	8.03	11.34	11.35	7.66
10.81	13.64	16.44	11.35	11.19	13.65	4.54	10.32	10.53
9.37	8.77	7.4	11.35	9.1	8.83	10.21	6.19	8.61
9.37	3.9	7.4	12.61	7.7	8.03	9.07	11.35	7.66
13.69	7.8	7.4	9.46	10.49	10.44	12.47	11.35	11.48
5.04	9.75	9.86	5.05	4.2	8.83	6.8	5.16	6.7
8.65	7.8	8.22	10.72	7.7	4.02	13.61	18.57	5.74
7.92	14.62	9.86	7.57	9.1	9.64	7.94	8.26	7.66
28.82	16.57	21.37	34.69	37.78	32.13	24.95	26.83	16.27
7.2	12.67	8.22	8.2	4.2	5.62	11.34	7.22	10.53
0.72	0	0.82	4.41	0.7	0.8	9.07	4.13	9.57
10.09	4.87	6.58	6.31	4.2	8.03	12.47	9.29	10.53
10.09	13.64	15.62	10.09	10.49	12.85	10.21	11.35	11.48
9.37	5.85	5.75	10.72	8.4	11.24	9.07	3.1	8.61
0	0	0	4.41	0.7	0.8	6.8	2.06	6.7
1.44	1.95	4.11	3.15	2.1	4.02	6.8	9.29	8.61
7.2	10.72	9.04	3.78	3.5	7.23	7.94	7.22	6.7
0.72	0.97	1.64	5.05	4.9	2.41	5.67	15.48	2.87
5.76	3.9	4.93	8.83	7.7	8.83	7.94	6.19	3.83
0	0	0	0	11.19	0	7.94	0	23.93
6.48	4.87	5.75	8.83	8.4	11.24	4.54	4.13	7.66
9.37	8.77	5.75	5.68	6.3	5.62	11.34	12.38	6.7
12.97	18.52	12.33	17.03	18.89	19.28	17.01	11.35	16.27
4.32	5.85	4.93	6.94	7.7	6.43	5.67	7.22	4.79
5.76	12.67	8.22	5.68	3.5	4.82	10.21	5.16	10.53
28.1	23.39	32.05	25.86	25.19	18.47	20.41	26.83	29.67
8.65	4.87	6.58	3.78	7.7	7.23	6.8	8.26	1.91
9.37	7.8	4.93	6.31	6.3	8.03	5.67	6.19	0
18.01	21.44	18.08	5.68	7.7	7.23	22.68	25.8	20.1
7.2	9.75	5.75	6.31	5.6	6.43	9.07	4.13	10.53
7.2	4.87	6.58	5.05	4.9	7.23	5.67	3.1	10.53
12.25	13.64	11.51	10.09	8.4	13.65	10.21	12.38	14.36
3.6	8.77	8.22	8.2	7.7	11.24	3.4	4.13	7.66
7.2	9.75	13.15	9.46	11.19	8.83	1.13	2.06	3.83
7.2	4.87	5.75	6.94	7	8.83	3.4	3.1	7.66
4.32	7.8	5.75	5.05	2.8	4.02	7.94	13.42	7.66
4.32	4.87	4.93	3.78	3.5	3.21	7.94	8.26	6.7
4.32	3.9	4.11	4.41	11.89	4.82	4.54	3.1	5.74
5.04	5.85	4.93	5.68	6.3	7.23	9.07	9.29	7.66
4.32	4.87	4.93	6.31	5.6	8.03	2.27	4.13	5.74
6.48	10.72	9.04	10.09	11.89	11.24	0	0	0.96
5.76	6.82	5.75	8.2	9.79	9.64	2.27	5.16	2.87
6.48	8.77	9.04	8.2	8.4	7.23	1.13	3.1	1.91
3.6	2.92	2.47	5.05	4.9	6.43	4.54	12.38	4.79
5.04	5.85	5.75	5.05	2.1	6.43	6.8	3.1	4.79
7.2	6.82	7.4	6.31	12.59	10.44	1.13	5.16	2.87
3.6	4.87	4.93	6.31	4.2	8.03	4.54	4.13	4.79

5.04	4.87	1.64	10.09	8.4	6.43	4.54	0	1.91
7.92	2.92	4.11	4.41	4.2	4.82	1.13	5.16	8.61
3.6	2.92	4.93	6.31	7	4.82	4.54	4.13	2.87
2.88	5.85	2.47	6.31	8.4	8.83	3.4	4.13	5.74
5.76	3.9	7.4	8.83	5.6	6.43	1.13	4.13	1.91
5.04	3.9	5.75	3.78	6.3	6.43	9.07	1.03	2.87
6.48	6.82	4.93	3.15	4.2	4.02	4.54	5.16	3.83
6.48	5.85	3.29	5.05	7	5.62	3.4	4.13	3.83
5.04	4.87	4.11	5.68	4.9	4.82	4.54	5.16	5.74
3.6	2.92	2.47	6.94	4.9	4.02	5.67	11.35	2.87
4.32	3.9	1.64	8.83	4.2	4.02	5.67	10.32	2.87
10.81	11.69	10.68	10.09	6.3	8.03	5.67	5.16	10.53
7.92	2.92	11.51	10.72	12.59	8.03	1.13	0	0.96
4.32	4.87	4.11	3.78	4.9	3.21	6.8	6.19	5.74
0	0	0.82	1.26	0	1.61	4.54	2.06	4.79
5.04	4.87	2.47	4.41	4.2	7.23	5.67	4.13	3.83
6.48	2.92	2.47	4.41	3.5	3.21	3.4	7.22	2.87
5.76	7.8	5.75	6.94	4.9	8.03	6.8	3.1	6.7
6.48	3.9	2.47	3.78	4.2	3.21	5.67	5.16	6.7
4.32	3.9	9.04	3.78	4.9	3.21	5.67	4.13	7.66
6.48	3.9	4.93	3.78	3.5	4.02	4.54	3.1	2.87
2.88	3.9	2.47	7.57	4.9	3.21	2.27	4.13	6.7
5.04	3.9	2.47	7.57	4.2	4.02	3.4	5.16	1.91
6.48	2.92	4.93	3.78	0.7	3.21	2.27	9.29	2.87
2.16	2.92	2.47	5.05	2.8	6.43	3.4	5.16	2.87
3.6	0.97	4.93	3.15	3.5	3.21	2.27	6.19	4.79
2.88	0.97	0	1.89	4.2	0.8	1.13	6.19	3.83
0	0	2.47	0	4.2	2.41	0	7.22	15.31
5.04	3.9	4.11	6.31	7.7	6.43	3.4	2.06	2.87
3.6	4.87	4.11	4.41	4.9	3.21	3.4	5.16	4.79
5.76	9.75	4.11	5.68	6.3	5.62	1.13	2.06	1.91
5.04	4.87	4.93	6.31	4.2	4.02	5.67	4.13	1.91
1.44	2.92	3.29	3.78	6.3	6.43	3.4	2.06	2.87
3.6	1.95	3.29	3.78	3.5	4.02	4.54	6.19	3.83
4.32	4.87	4.93	6.31	3.5	7.23	3.4	3.1	3.83
7.92	1.95	3.29	2.52	2.8	8.83	10.21	3.1	2.87
5.04	2.92	2.47	3.15	6.3	4.02	4.54	1.03	0.96
2.88	2.92	4.93	6.94	4.9	4.02	2.27	3.1	2.87
2.88	1.95	1.64	6.94	4.2	6.43	0	2.06	0.96
2.16	8.77	9.04	3.78	3.5	4.82	5.67	3.1	6.7
2.16	0	0.82	2.52	4.2	3.21	2.27	0	0.96
5.04	3.9	7.4	12.61	8.4	12.05	0	0	0
2.16	2.92	3.29	1.89	2.8	1.61	5.67	7.22	2.87
5.04	4.87	2.47	3.15	3.5	2.41	4.54	6.19	1.91
5.04	4.87	3.29	3.15	4.2	5.62	5.67	1.03	3.83
2.88	4.87	3.29	4.41	2.1	4.82	3.4	2.06	3.83
0.72	2.92	4.11	3.15	2.1	4.02	5.67	4.13	0.96

3.6	1.95	1.64	1.89	3.5	3.21	2.27	6.19	1.91
9.37	12.67	13.15	6.94	7	9.64	6.8	10.32	7.66
5.04	3.9	3.29	6.94	7.7	3.21	2.27	4.13	0.96
4.32	1.95	3.29	5.68	5.6	2.41	2.27	5.16	3.83
2.88	3.9	6.58	10.09	3.5	1.61	0	3.1	2.87
2.16	3.9	1.64	4.41	1.4	1.61	4.54	4.13	2.87
0	0	0	3.78	0	0	1.13	2.06	2.87
1.44	3.9	4.11	3.15	2.8	2.41	3.4	4.13	1.91
2.88	4.87	4.93	5.05	5.6	4.82	6.8	4.13	2.87
7.2	7.8	4.11	5.68	5.6	4.02	1.13	2.06	3.83
3.6	0	1.64	10.09	4.9	4.82	1.13	3.1	0
2.88	1.95	4.93	6.94	4.9	4.82	2.27	5.16	1.91
3.6	1.95	0	3.15	6.3	3.21	6.8	4.13	0.96
4.32	1.95	4.11	3.78	2.8	3.21	2.27	5.16	0.96
2.16	1.95	3.29	3.78	4.2	4.82	2.27	3.1	2.87
2.88	2.92	4.11	1.89	3.5	3.21	3.4	3.1	2.87
3.6	2.92	4.93	3.15	2.1	4.02	5.67	7.22	1.91
1.44	1.95	2.47	1.89	4.9	3.21	3.4	3.1	1.91
4.32	0.97	4.93	2.52	1.4	3.21	3.4	6.19	2.87
2.88	1.95	2.47	5.05	3.5	4.82	4.54	4.13	0.96
2.88	3.9	4.93	1.89	2.1	3.21	3.4	2.06	5.74
23.05	21.44	23.01	20.18	22.39	16.06	15.88	22.7	22.01
3.6	2.92	2.47	3.78	2.1	5.62	4.54	1.03	2.87
12.97	14.62	8.22	16.4	16.79	14.46	9.07	11.35	6.7
9.37	4.87	4.11	3.15	1.4	0.8	5.67	4.13	3.83
5.76	3.9	1.64	3.15	1.4	1.61	1.13	2.06	2.87
4.32	2.92	2.47	3.15	2.1	1.61	5.67	4.13	1.91
3.6	3.9	2.47	1.89	1.4	3.21	5.67	6.19	1.91
2.16	7.8	4.11	5.68	3.5	2.41	2.27	1.03	1.91
2.88	2.92	1.64	3.15	4.2	4.02	3.4	1.03	0
3.6	0.97	1.64	6.31	4.9	4.82	2.27	4.13	2.87
2.16	2.92	3.29	5.05	3.5	5.62	3.4	2.06	3.83
6.48	1.95	1.64	3.15	3.5	5.62	4.54	1.03	1.91
2.88	0.97	1.64	3.15	4.2	4.82	4.54	1.03	1.91
1.44	3.9	0.82	7.57	7	4.82	4.54	0	1.91
2.16	1.95	1.64	3.78	4.2	2.41	2.27	4.13	2.87
2.88	4.87	3.29	2.52	4.9	3.21	4.54	2.06	4.79
3.6	0	0.82	3.15	1.4	7.23	3.4	2.06	1.91
2.16	2.92	1.64	4.41	2.8	6.43	3.4	2.06	1.91
4.32	2.92	3.29	10.72	6.3	6.43	0	0	0.96
0	1.95	2.47	5.68	3.5	4.02	2.27	1.03	0.96
3.6	1.95	0.82	1.89	1.4	4.82	7.94	5.16	2.87
3.6	5.85	4.11	3.78	2.8	3.21	4.54	3.1	2.87
0	0	0	1.89	0	0	0	4.13	5.74
2.16	3.9	5.75	4.41	2.8	3.21	3.4	1.03	3.83
2.88	2.92	1.64	4.41	3.5	1.61	2.27	2.06	2.87
10.81	5.85	4.11	6.94	4.9	8.03	10.21	8.26	8.61

2.16	0.97	2.47	2.52	2.8	5.62	3.4	5.16	3.83
8.65	2.92	5.75	3.15	1.4	4.02	3.4	1.03	0
3.6	0.97	2.47	5.05	4.9	2.41	2.27	4.13	1.91
2.16	0	0	2.52	6.3	5.62	1.13	2.06	0
2.88	2.92	3.29	3.15	2.1	4.02	2.27	2.06	1.91
3.6	2.92	4.11	0	0.7	0.8	4.54	7.22	5.74
0	0	1.64	1.26	0	0.8	0	2.06	5.74
0	0	0	0	0	0	0	0	20.1
2.16	0.97	2.47	1.26	1.4	2.41	3.4	5.16	4.79
2.16	0.97	0.82	2.52	4.2	4.02	2.27	3.1	0.96
1.44	0.97	0	2.52	2.8	3.21	2.27	2.06	0.96
0	0	0	1.26	0	0	3.4	0	3.83
2.88	2.92	1.64	3.15	2.1	1.61	5.67	3.1	0.96
0.72	2.92	3.29	1.26	0	3.21	3.4	1.03	0.96
11.53	18.52	13.97	6.31	6.3	6.43	24.95	21.67	0
3.6	1.95	2.47	0.63	4.9	4.82	1.13	4.13	0
5.04	4.87	1.64	6.31	4.9	4.82	4.54	4.13	4.79
4.32	2.92	2.47	1.89	1.4	3.21	0	3.1	2.87
7.92	6.82	0	8.2	9.79	0	7.94	10.32	7.66
2.16	2.92	4.11	3.15	5.6	4.02	1.13	1.03	1.91
2.88	4.87	1.64	4.41	0.7	4.02	1.13	0	3.83
3.6	1.95	1.64	3.78	1.4	2.41	3.4	4.13	2.87
1.44	2.92	1.64	1.26	4.2	1.61	2.27	1.03	1.91
3.6	1.95	0.82	3.15	1.4	1.61	1.13	1.03	0.96
2.88	1.95	1.64	3.78	2.1	4.82	0	1.03	0
4.32	3.9	3.29	1.26	0.7	1.61	3.4	3.1	4.79
5.04	4.87	2.47	3.78	4.9	4.02	2.27	1.03	1.91
1.44	3.9	2.47	5.05	4.2	2.41	3.4	0	1.91
3.6	4.87	4.11	2.52	4.2	0	2.27	3.1	1.91
5.76	0	0	3.15	1.4	1.61	1.13	3.1	0.96
0.72	0.97	0.82	3.78	5.6	3.21	0	0	0.96
3.6	0.97	0.82	3.15	2.8	3.21	2.27	4.13	0.96
5.76	9.75	9.86	5.68	3.5	6.43	3.4	6.19	5.74
2.16	1.95	4.11	1.26	1.4	2.41	3.4	2.06	3.83
2.16	0	0.82	4.41	3.5	3.21	2.27	1.03	2.87
1.44	1.95	3.29	4.41	4.2	6.43	0	1.03	0.96
2.88	2.92	1.64	5.05	3.5	4.02	0	0	0
0.72	4.87	2.47	2.52	2.1	4.82	0	0	0
2.16	1.95	2.47	5.68	4.2	4.02	0	2.06	0
10.81	18.52	15.62	4.41	5.6	5.62	20.41	17.54	13.4
3.6	0.97	1.64	5.05	5.6	4.82	0	1.03	0.96
5.04	1.95	4.93	6.94	4.2	4.02	1.13	2.06	1.91
2.88	3.9	1.64	2.52	2.8	0.8	3.4	2.06	2.87
0	0	0.82	4.41	0	1.61	0	2.06	0
0	0	0.82	0	2.1	0.8	0	0	10.53
0.72	0.97	0.82	4.41	6.3	4.02	0	0	0
1.44	0.97	3.29	4.41	6.3	3.21	0	0	2.87

0	1.95	1.64	5.68	3.5	3.21	3.4	0	1.91
2.88	1.95	2.47	3.78	4.9	4.02	1.13	0	0.96
0.72	0.97	0	2.52	2.8	4.82	1.13	2.06	0.96
3.6	0	0	2.52	3.5	3.21	4.54	3.1	0.96
1.44	0	0	4.41	3.5	2.41	0	0	0
3.6	0.97	0.82	5.05	4.2	2.41	1.13	0	0
0	0	0	3.15	0	0.8	0	1.03	0
0	0	0	0.63	0	0	1.13	3.1	3.83
2.88	2.92	2.47	4.41	6.3	2.41	0	0	0
0.72	0.97	2.47	5.05	2.1	4.02	2.27	1.03	0.96
0	0	1.64	1.89	1.4	0	0	5.16	3.83
3.6	4.87	3.29	1.89	3.5	3.21	0	2.06	0.96
1.44	0.97	2.47	6.94	3.5	4.02	0	0	0
0.72	0.97	0	3.15	3.5	2.41	2.27	1.03	0
2.88	5.85	8.22	4.41	5.6	8.83	3.4	4.13	3.83
2.16	0.97	0	2.52	6.3	4.02	0	1.03	0
3.6	0.97	1.64	1.89	0	0	4.54	5.16	4.79
0	0	3.29	0	3.5	0	0	0	18.18
11.53	0	12.33	22.7	11.19	0	20.41	27.86	22.01
0	0	0	4.41	7	6.43	0	0	0
8.65	8.77	0	6.94	12.59	8.83	9.07	11.35	9.57
10.09	21.44	14.79	4.41	4.2	4.82	18.15	15.48	15.31
0	0.97	0	0	0	0	0	1.03	3.83
5.76	0	16.44	7.57	6.3	8.83	7.94	13.42	0
10.81	0	14.79	28.38	16.09	12.05	0	15.48	16.27
5.76	0	0	6.31	9.79	4.82	0	0	0.96
0	0	0	3.78	4.2	4.02	3.4	1.03	0
0	0	0	10.09	12.59	0	11.34	0	0
18.01	19.49	17.26	0	0	0	0	10.32	18.18
17.29	0	21.37	10.09	10.49	0	0	0	0
7.92	0	0	7.57	0	0	0	12.38	12.44
0	0.97	0	4.41	3.5	4.02	0	0	0
7.2	0	8.22	0	4.9	3.21	5.67	0	5.74
0	0	0	0	5.6	0	0	0	0
0	0	0	6.31	6.3	4.02	0	0	0
0	4.87	0	4.41	7	7.23	0	0	0
3.6	0	2.47	0	3.5	0	0	0	0
0	6.82	0	0	6.3	4.02	0	11.35	22.01
0	0	0	0	0	0	0	0	0
0	9.75	0	5.68	8.4	0	0	0	0
0	6.82	0	0	5.6	0	5.67	5.16	9.57
0	7.8	0	0	0	0	6.8	0	0
0	0	0	0	0	4.82	0	0	0
0	0	8.22	0	0	0	0	0	0

	VancATN				Group Mean				
G12.norm	G16.norm	G17.norm	G18.norm	mean.ATN	mean.IgAN	mean.IgAE1	mean.NLTx	mean.S.a.GN
140.31	117.51	102.52	93.21	125.27	146.58	147.12	140.15	149.38
132.98	76.16	52.86	72.72	90.68	127.52	119.81	99.54	122.01
66.95	75.08	82.49	82.97	86.45	80.02	96.58	93.46	66.15
74.28	68.55	52.06	74.77	69.99	81.93	77.22	65.92	73.8
39.43	71.81	64.07	60.43	59.11	57.63	59.17	62.55	50.12
38.52	38.08	34.44	25.61	39.37	41.57	47.61	59.74	48.07
23.84	42.43	56.86	66.58	54.65	50.88	59.12	56.31	39.14
36.68	55.49	50.46	55.31	57.41	53.52	43.59	50.51	53.29
28.43	66.37	35.24	86.04	61.69	39.62	56.77	49.69	52.03
54.11	43.52	38.44	47.12	35.59	51.36	60.45	48.27	58.51
49.52	55.49	48.86	45.07	56.75	49.41	55.58	47.2	51.78
54.11	129.48	12.01	3.07	30.65	31.09	65.52	21.68	51.04
28.43	55.49	40.05	51.21	56.28	53.61	51.77	44.6	43.08
22.93	44.61	40.05	51.21	37.24	40.49	43.63	42.28	40.64
52.27	31.55	31.24	33.8	41.54	58.67	54.51	40.53	54
33.01	129.48	4	0	23.47	16.07	50.3	18.56	48.63
86.21	138.18	11.21	4.1	30.98	48.37	51.11	26.87	62.94
23.84	31.55	48.05	31.75	23.6	27.8	31.86	48.71	32.01
26.6	42.43	33.64	35.85	40.62	48.02	40.89	35.78	37.47
19.26	33.73	36.84	37.9	38.49	40.66	42.33	36.74	33.91
33.01	30.47	38.44	23.56	29.17	34.68	34.32	39.27	34.37
100.88	200.2	5.61	0	22.11	36.85	39.88	18.46	63.67
55.94	34.82	25.63	29.7	23.99	40.1	43.21	26.13	44.62
29.35	22.85	23.23	20.49	24.98	25.16	27.19	26.14	30.31
22.01	38.08	31.24	44.04	27.44	26.2	40.04	13.47	18.74
20.18	16.32	24.83	25.61	18.01	29.14	23.08	24.41	22.32
29.35	21.76	19.22	19.46	27.6	23.17	22.69	24.91	26.33
23.84	46.79	28.03	30.73	15.94	27.93	29.7	17.41	17.36
41.27	29.38	41.65	35.85	17.85	43.17	35.44	6.61	37.04
16.51	23.94	20.02	22.53	24.32	25.03	22.53	23.84	22
20.18	18.5	16.82	15.36	22.88	23.73	25.56	19.76	21.77
24.76	11.97	18.42	17.41	19.66	23.12	22.47	23.92	20.11
24.76	19.59	16.02	19.46	28.47	19.57	20.94	21.68	22.71
9.17	22.85	20.82	20.49	17.36	15.72	13.92	25.48	12.41
8.25	22.85	21.62	26.63	25.35	18.23	23.67	18.87	13.03
22.01	9.79	15.22	17.41	13.09	15.81	15.93	23.69	16.1
19.26	62.02	5.61	1.02	9.61	10.18	20.64	6.02	20.25
27.51	11.97	9.61	11.27	15.6	26.93	21.97	16.69	30.32
19.26	20.67	14.42	16.39	16.44	26.02	21.29	16.97	25.83
16.51	18.5	18.42	14.34	15.04	16.15	11.46	23.63	14.14
12.84	28.29	23.23	29.7	26.57	19.7	24.54	11.47	12.98

16.51	11.97	6.41	15.36	20.59	19.66	19.78	17.29	17.58
14.67	15.23	13.62	22.53	19.9	14.94	27.49	13.64	17.36
16.51	15.23	19.22	14.34	18.76	13.81	16.7	16.17	15.71
13.76	18.5	28.83	17.41	16.8	9.74	13.91	17.58	11.62
12.84	16.32	10.41	14.34	14.41	13.51	12.68	13.63	13.72
16.51	11.97	13.62	9.22	24.4	10.96	11.33	12.04	14.4
16.51	8.7	15.22	13.32	11.99	15.03	9.35	15.21	14.89
19.26	11.97	8.81	7.17	11.4	20.05	15.74	13.39	17.74
19.26	54.4	6.41	0	6.6	6.19	15.99	4.07	15.71
20.18	7.62	7.21	10.24	14.03	11.73	14.25	13.43	15.55
16.51	15.23	12.81	13.32	12.53	9.01	10.32	12.28	14.38
12.84	11.97	7.21	9.22	16.06	17.28	22.6	8.42	11.37
5.5	10.88	20.02	10.24	12.35	9.35	9.03	14.23	4.89
3.67	15.23	16.02	16.39	14.26	8.4	12.89	12.47	11.58
8.25	13.06	16.82	10.24	7.8	8.79	10.75	14.77	9.95
11.92	8.7	12.81	8.19	14.27	7.27	9.37	13.18	8.96
15.59	11.97	16.82	15.36	19.75	12.56	10.62	13.23	14.7
7.34	10.88	16.82	10.24	7.8	6.71	7.95	14.11	6.93
16.51	7.62	6.41	5.12	8.48	13.81	10.79	9.59	14.13
22.93	66.37	4	0	9.16	12.38	16.57	5.94	21.59
14.67	13.06	15.22	9.22	15.09	6.63	9.06	11.07	12.13
11.92	17.41	17.62	13.32	14.66	8.88	13.68	14.11	12.81
5.5	11.97	12.01	12.29	16.46	9.18	11.48	12.57	8.26
17.42	15.23	12.81	9.22	12.41	7.01	9.49	12.11	11.68
0.92	23.94	0	0	24.21	0	1.22	0	1.92
9.17	6.53	8.01	9.22	13.84	13.51	9.38	13.09	9.87
16.51	13.06	10.41	9.22	9.47	11	13.57	10.96	13.9
6.42	11.97	16.02	13.32	9.35	10.65	8.54	12.97	8.42
5.5	7.62	9.61	19.46	11.98	6.32	11.67	10.38	10.78
11.92	9.79	8.81	11.27	9.69	10.39	11.26	9.54	11.89
9.17	15.23	12.01	12.29	10.06	6.24	9.35	10.95	9.56
42.19	17.41	18.42	25.61	10.22	15.16	17.12	1.3	15.67
10.09	13.06	15.22	8.19	8.89	7.45	9.46	12.92	10.86
6.42	8.7	10.41	8.19	7.37	7.49	8.65	12.7	9.27
12.84	16.32	12.01	11.27	14.86	19.62	15.28	18.06	20.63
11	4.35	1.6	1.02	6.82	11.35	10.72	6.6	12.62
12.84	13.06	8.81	8.19	8.84	9.44	8.85	9.2	13.15
6.42	7.62	6.41	8.19	10.37	10.05	11.56	8.72	9.56
59.61	40.26	36.04	55.31	46.92	64.04	57.38	40.79	58.54
13.76	7.62	9.61	5.12	6.67	7.84	6.81	10.58	11.1
11	11.97	12.81	9.22	7.37	8.7	8.63	10.74	9.13
8.25	13.06	16.02	12.29	8.38	6.97	3.56	9.8	6.77
13.76	8.7	10.41	9.22	8.14	7.75	8.31	10.5	10.61
6.42	7.62	10.41	11.27	10.09	13.42	9.4	9.64	6.34
11.92	13.06	7.21	4.1	8.8	8.23	8.61	9.71	9.71
7.34	9.79	7.21	7.17	8.71	11.82	11.33	9.48	9.64
9.17	6.53	9.61	5.12	9.35	8.31	7.61	9.6	8

9.17	9.79	8.01	9.22	8.55	7.84	8.81	6.15	9.88
8.25	7.62	10.41	12.29	13.54	10.78	13.05	12.71	8.41
9.17	10.88	11.21	9.22	11.23	7.84	5.68	8.8	8.55
4.59	9.79	10.41	13.32	5.89	6.8	7.81	7.85	8.17
5.5	7.62	8.01	5.12	9.38	9.83	8.97	10.29	10.2
13.76	10.88	4	9.22	8.63	9.09	5.28	6.83	8.1
11.92	9.79	5.61	7.17	5.37	6.32	9.93	7.33	12.46
8.25	6.53	8.81	7.17	6.24	14.25	5.09	9.6	8.03
17.42	32.64	30.43	27.66	26.75	20.18	26.94	27.63	21.37
5.5	6.53	5.61	8.19	7.05	7.92	6.41	8.41	8.65
19.26	10.88	17.62	13.32	6.66	18.4	16.32	1.43	10.51
8.25	9.79	6.41	5.12	9.6	8.88	9.11	7.18	10.14
9.17	13.06	8.81	12.29	13.83	14.38	14.02	11.85	10.55
3.67	8.7	10.41	6.15	7.67	5.02	7.36	8.42	6.11
21.09	10.88	12.01	13.32	6.05	14.25	18.01	1.09	9.17
8.25	8.7	6.41	12.29	9.3	12.43	11.44	3.12	8.24
10.09	5.44	4.81	4.1	5.4	6.24	5.48	6.9	7.99
6.42	10.88	6.41	18.44	8.57	6.54	5.3	2.61	7.61
9.17	11.97	8.01	7.17	7.67	5.11	6.64	6.8	6.78
12.84	51.14	4.81	0	0	4.5	17.52	1.6	11.18
4.59	3.26	7.21	4.1	6.76	5.2	6.7	7.72	5.23
8.25	5.44	6.41	6.15	3.61	9.05	6.75	7.02	9.67
9.17	14.14	17.62	9.22	15.34	15.89	11.49	16.69	13.45
6.42	7.62	5.61	7.17	11.04	5.67	8.23	6.01	6.02
10.09	6.53	4	8.19	6.19	8.79	6.45	6.53	9
33.01	21.76	21.62	20.49	26.25	22.99	22.66	25.5	27.48
4.59	5.44	5.61	9.22	3.61	8.14	7.29	6.76	5.39
8.25	6.53	8.01	6.15	5.1	3.77	6.29	7.44	5.03
23.84	9.79	5.61	7.17	11.99	20.96	16.98	14.07	23.11
6.42	3.26	7.21	5.12	5.35	6.63	2.94	6.95	7.54
2.75	4.35	8.81	7.17	6.83	6.63	6.45	5.48	5.51
17.42	11.97	11.21	10.24	15.22	9.44	11.93	11.63	13.59
10.09	8.7	6.41	6.15	5.2	4.72	4.98	7.79	6.32
4.59	2.18	7.21	3.07	3.56	2.08	3.32	9.6	2.9
7.34	7.62	5.61	4.1	5.15	3.03	5.82	7.13	5.37
11	6.53	6.41	5.12	3.92	6.32	5.75	4.98	10
7.34	2.18	2.4	9.22	4.25	8.44	7.36	4.12	7.56
1.83	6.53	6.41	8.19	7.7	4.55	6.46	5.75	3.8
6.42	5.44	5.61	7.17	5.95	5.2	7.16	6.1	8.11
2.75	6.53	6.41	7.17	6.19	2.55	7.76	5.47	3.72
1.83	2.18	9.61	2.05	4.6	1.04	3.64	10.19	0.7
3.67	7.62	8.01	6.15	5.82	2.47	4.02	7.42	3.49
2.75	5.44	8.01	3.07	7.31	2.94	4.62	8.21	2.22
3.67	8.7	4	8.19	4.41	4.55	6.07	4.23	6.34
4.59	2.18	4.81	5.12	7.51	5.37	6.01	5.29	4.82
4.59	4.35	4.81	5.12	4.23	3.42	2.88	8.47	3.44
3.67	3.26	5.61	6.15	5.41	5.59	3.71	5.41	4.28

1.83	5.44	11.21	3.07	3.37	2.73	2.49	6.54	2.07
2.75	9.79	4.81	4.1	8.04	5.67	6.7	5.27	4.41
3.67	5.44	5.61	7.17	4.66	5.2	4.46	5.56	3.8
1.83	5.44	7.21	7.17	3.37	3.68	6.36	5.57	3.78
3.67	2.18	4	4.1	4.54	3.98	4.85	6.14	2.71
6.42	6.53	7.21	5.12	5.02	4.33	4.12	5.67	4.85
6.42	3.26	4.81	2.05	2.32	2.64	2.49	4.96	4.99
2.75	3.26	5.61	5.12	4.96	4.72	6.88	5.48	3.53
4.59	5.44	4	8.19	4.91	5.2	3.46	4.93	5.01
6.42	8.7	4	6.15	4.42	5.67	4.96	3.91	6.58
4.59	2.18	3.2	4.1	2.64	4.16	4.98	3.97	5.86
6.42	4.35	11.21	9.22	8.13	6.15	6.06	10.17	6.94
1.83	0	8.81	3.07	3.7	2.73	1.68	8.88	0.98
7.34	5.44	2.4	3.07	4.11	5.67	4.71	4.21	6.52
5.5	4.35	4.81	5.12	4.39	13.94	7.32	0.89	4.22
7.34	5.44	3.2	4.1	6.81	5.67	4.85	5.61	5.24
0.92	3.26	4.81	5.12	2.64	4.63	4.21	4.38	3.6
8.25	0	3.2	9.22	5.23	5.76	7.05	6.2	6.21
7.34	3.26	4.81	2.05	3.12	3.51	3.42	4.65	6.22
7.34	4.35	4	6.15	2.15	4.72	5.65	5.26	6.2
7.34	3.26	3.2	7.17	4.48	4.07	3.91	4.65	4.46
4.59	3.26	5.61	5.12	6.7	1.99	3.94	4.17	4.42
3.67	2.18	4	4.1	2.03	3.2	1.94	4.73	3.54
3.67	0	1.6	0	1.83	0.95	4.32	4	4.52
0.92	5.44	4.81	4.1	7.51	3.68	5.8	3.6	3.09
4.59	8.7	9.61	9.22	2.76	5.02	3.26	3.13	4.46
7.34	7.62	4	3.07	3.49	0	1.27	2.02	4.62
12.84	21.76	2.4	0	8.62	0	7.58	1.3	8.84
2.75	3.26	6.41	4.1	2.03	4.16	2.13	5.63	2.77
5.5	3.26	3.2	2.05	2.9	3.59	5.71	4.31	4.71
2.75	1.09	4.81	1.02	1.72	1.99	1.63	6.53	1.97
11	3.26	6.41	1.02	1.17	0.95	4.56	5.17	5.68
0.92	6.53	5.61	4.1	6.25	3.03	5.05	3.57	2.31
5.5	4.35	5.61	2.05	3.8	2.73	4.48	3.6	5.01
4.59	3.26	9.61	4.1	3.67	2.47	4.71	5.66	3.73
1.83	0	2.4	1.02	0.74	10.31	4.37	4.51	4.5
4.59	3.26	4.81	4.1	2.88	5.11	2.52	4.26	2.78
2.75	6.53	8.01	4.1	2.99	3.03	3.15	4.28	2.75
0.92	4.35	5.61	4.1	3.85	1.6	3.58	3.68	0.98
7.34	5.44	4	5.12	6.88	6.63	6.23	5.43	5.7
4.59	0	4	0	6.74	2.17	1.99	2.45	1.95
0	0	4.81	0	1.22	1.6	0.31	8.27	0
2.75	5.44	4	6.15	3.32	7.97	5.51	2.46	4.63
5.5	5.44	4	3.07	2.64	1.6	2.99	3.55	4.54
3.67	5.44	3.2	5.12	3.19	4.16	3.82	4.1	3.55
3.67	3.26	4	3.07	4.22	4.16	3.71	3.68	3.24
2.75	3.26	6.41	8.19	3.19	3.51	7.29	2.55	3.38

3.67	3.26	4.81	5.12	5.16	2.08	5.19	2.5	3.51
10.09	7.62	7.21	10.24	10.77	12.21	10.92	9.49	8.72
3.67	4.35	4	4.1	1.47	1.6	1.42	5.02	2.76
1.83	0	1.6	4.1	1.66	3.51	3.3	3.56	3.27
2.75	0	3.2	1.02	1.6	0.95	2.44	4.81	2.18
0.92	2.18	2.4	4.1	5.62	5.11	3.02	2.65	3.11
2.75	3.26	19.22	13.32	8.65	0.56	2.47	0.78	2.21
5.5	4.35	4	3.07	1.04	2.08	2.99	3.03	3.74
3.67	2.18	3.2	6.15	2.94	2.08	1.99	4.26	4.37
0.92	4.35	5.61	0	1.48	3.12	2.19	5.52	1.99
0	6.53	5.61	3.07	3.19	2.08	2.38	3.94	1.06
2.75	4.35	4	3.07	4.05	2.08	2.74	4.14	3.02
0	6.53	6.41	5.12	5.28	3.03	2.13	2.97	2.97
2.75	4.35	4	3.07	3.61	1.52	1.94	3.73	2.78
2.75	4.35	6.41	4.1	4.36	1.52	3.58	3.13	2.75
3.67	3.26	4	3.07	5.64	2.08	2.54	3.01	3.26
4.59	4.35	5.61	7.17	4.23	2.73	2.19	3.45	4.85
0.92	7.62	4.81	4.1	4.35	3.03	2.94	2.63	2.33
3.67	4.35	4	6.15	4.42	4.16	3.38	2.84	4.03
1.83	7.62	3.2	2.05	4.42	2.73	5.05	3.68	2.86
5.5	3.26	3.2	3.07	4.85	2.94	2.54	3.31	4.18
25.68	19.59	18.42	16.39	19.92	20.05	17.64	21.9	21.57
1.83	2.18	4.81	4.1	2.57	3.59	0.97	3.41	2.57
5.5	10.88	18.42	0	10.5	8.79	9.41	14.35	8.16
5.5	2.18	4	2.05	1.29	4.24	1.93	3.75	4.78
6.42	6.53	4	3.07	3.62	2.47	2.8	2.74	3.12
2.75	1.09	2.4	3.07	2.77	4.16	2.18	2.73	3.62
1.83	3.26	2.4	3.07	1.77	3.51	2.35	2.96	3.9
7.34	3.26	4	2.05	3.32	3.12	2.63	3.91	3.14
2.75	4.35	3.2	3.07	3.44	0.56	3.24	3.17	1.8
0.92	2.18	5.61	2.05	0.92	1.99	3.35	3.54	2.55
3.67	2.18	3.2	3.07	1.9	2.64	3.87	3.46	3.24
0.92	1.09	6.41	3.07	2.82	1.04	3.65	3.68	2.1
5.5	3.26	5.61	5.12	3.73	2.25	2.96	3.01	3.25
1.83	4.35	4	5.12	2.64	1.43	2.49	4.5	2.07
2.75	6.53	2.4	3.07	4.05	2.17	3.19	2.91	3
0.92	3.26	3.2	1.02	4.55	2.08	1.94	3.95	3.08
1.83	1.09	5.61	4.1	2.57	2.17	2.88	2.92	2.3
2.75	3.26	4.81	5.12	3.31	1.04	1.53	2.91	2.53
0	2.18	8.01	0	0	0.48	0	5.34	0.24
3.67	1.09	4.81	3.07	2.03	3.12	1.83	3.36	1.98
2.75	2.18	4	4.1	0.92	2.08	2.4	2.8	4.68
3.67	1.09	2.4	2.05	1.35	4.07	2.04	4.19	3.54
4.59	6.53	9.61	5.12	1.78	2.47	2.49	0.27	3.61
3.67	2.18	4.81	2.05	3.12	3.2	1.02	3.78	2.98
1.83	2.18	4	3.07	2.88	2.17	1.47	2.79	2.26
4.59	11.97	6.41	5.12	9.47	9.44	9.01	6.9	7.92

3.67	2.18	3.2	4.1	2.38	3.12	2.49	2.97	4.01
1.83	2.18	4.81	4.1	2.52	4.16	5.01	4.31	1.57
0.92	2.18	4.81	0	2.33	1.99	1.66	3.13	2.31
0	2.18	4	4.1	0.86	1.6	2.03	2.61	0.8
0.92	1.09	3.2	2.05	5.14	3.2	1.33	3.11	1.79
5.5	0	0	0	0.61	3.59	2.54	2.46	5.75
2.75	4.35	4.81	7.17	2.22	2.08	1.99	0.65	2.64
0	33.73	0	0	0	5.24	5.5	0	5.02
2.75	2.18	0.8	3.07	2.77	2.47	3.1	1.89	4.02
0	3.26	3.2	4.1	2.21	1.04	4.85	2.34	1.58
0.92	5.44	4.81	2.05	3.8	1.6	1.02	1.93	1.55
8.25	8.7	6.41	4.1	3.35	0.56	2.63	0.42	3.87
1.83	3.26	4	2.05	2.15	0	2.29	2.41	2.89
4.59	2.18	7.21	1.02	1.35	2.17	1.42	2.24	2.49
20.18	5.44	6.41	6.15	9.39	19.18	14	11.31	16.7
0.92	3.26	2.4	2.05	3.5	1.04	1.42	3.72	1.54
4.59	4.35	5.61	4.1	4.35	3.59	2.6	4.79	4.51
1.83	1.09	4	1.02	1.66	0.95	2.85	3.04	1.95
11	21.76	18.42	30.73	15.15	15.89	23.88	5.41	9.23
0	4.35	2.4	4.1	1.9	2.47	1.63	3.5	1.02
0	0	0.8	0	0.31	4.33	0.97	3.01	1.24
1.83	1.09	4	2.05	1.61	3.12	2.18	2.72	3.06
0.92	3.26	1.6	6.15	1.53	2.55	2.85	2.23	1.53
1.83	3.26	2.4	2.05	1.04	1.99	2.24	2.64	1.24
2.75	3.26	7.21	5.12	1.35	1.69	1.52	2.7	0.95
1.83	1.09	0	5.12	1.22	2.94	1.99	2.64	3.28
2.75	3.26	4	2.05	0.61	4.24	2.13	4.19	1.99
0.92	2.18	4.81	1.02	0.74	2.08	1.74	3.26	1.56
1.83	1.09	4	2.05	0.86	0.56	2.04	3.24	2.28
4.59	2.18	2.4	2.05	1.16	1.6	1.02	2.19	2.44
0.92	1.09	4.81	3.07	2.39	0	1.27	2.52	0.47
1.83	5.44	3.2	1.02	3.06	2.17	1.52	2.93	2.3
9.17	3.26	4	8.19	7.45	8.23	7.57	6.82	6.13
5.5	1.09	4	1.02	4.54	2.55	4.32	2.02	3.7
0.92	3.26	6.41	2.05	2.82	0.56	2.38	2.26	1.77
1.83	0	5.61	2.05	0.61	1.52	0.92	3.59	0.96
0	2.18	5.61	0	0.61	1.52	0.97	3.59	0
0.92	0	5.61	3.07	3.48	0.56	2.22	2.74	0.23
0.92	3.26	4.81	2.05	1.78	1.6	2.04	3.17	0.75
17.42	6.53	0	6.15	0	17.02	8.63	11.08	17.19
0	1.09	2.4	2.05	0.31	0.48	0.81	3.83	0.5
1.83	5.44	11.21	5.12	3.25	3.42	3.21	4.23	1.74
4.59	5.44	1.6	1.02	0.86	0.56	1.22	2.68	3.23
0.92	2.18	3.2	5.12	2.65	2.17	3.6	0.98	0.75
5.5	13.06	0	0	1.1	0	1.72	0.53	4.01
0.92	1.09	5.61	3.07	1.16	0.95	0.61	2.71	0.23
0.92	2.18	4	2.05	2.59	1.04	1.61	2.93	0.95

2.75	4.35	3.2	2.05	2.02	0.56	2.71	2.65	2.02
0.92	4.35	1.6	1.02	0	1.13	0	2.98	0.75
0.92	2.18	2.4	3.07	1.83	0.56	1.88	1.81	1.27
0	3.26	0	3.07	1.53	1.13	0.66	2.2	2.15
0	2.18	4.81	4.1	3.99	1.04	0.81	1.8	0
0	1.09	1.6	1.02	0.92	1.6	0.61	3.04	0.28
0	3.26	6.41	8.19	3.22	0	2.03	0.57	0.26
3.67	5.44	5.61	4.1	3.26	0	1.47	0.09	2.93
0.92	0	0.8	0	0	0	0	3.66	0.23
0	0	4	1.02	1.77	0.48	0.66	2.31	1.06
6.42	4.35	2.4	12.29	0	6.54	2.81	0.95	3.85
0.92	0	2.4	0	1.83	0.48	0	3.39	0.98
0	0	4	0	0	0	0	3.25	0
0	2.18	4	2.05	4.42	0.56	1.47	1.66	0.83
2.75	6.53	5.61	7.17	9.04	2.81	9.43	5.6	3.53
0	3.26	1.6	1.02	0.74	0.56	0.66	2.89	0.26
3.67	0	1.6	1.02	4.83	4.81	3.44	1.52	4.54
0	19.59	0	0	3.18	0	0	0.97	4.55
18.34	0	15.22	20.49	14.86	0	27.65	8.25	22.16
0	0	0.8	0	0	0	0.61	2.79	0
11	9.79	9.61	10.24	5.7	8.31	6.53	7.75	10.25
15.59	6.53	4	8.19	6.64	17.49	12.64	8.54	16.13
3.67	1.09	0.8	6.15	3.44	2.94	3.35	0.14	2.13
0	9.79	7.21	0	15.88	3.94	16.58	7.26	5.34
14.67	11.97	11.21	0	15.64	0	3.94	13.55	11.61
0	0	8.81	5.12	1.1	0	0.61	4.42	0.24
0	3.26	4	5.12	3.75	4.55	3.72	1.71	1.11
0	11.97	7.21	0	15.61	0	4.88	4.45	2.84
20.18	0	0	0	0	12.12	3.58	9.52	12.17
0	0	8.01	12.29	0	7.14	0	10.28	0
15.59	14.14	0	8.19	0	13.08	4.66	2.21	10.1
0	0	0	0	0	0	0.31	1.84	0
5.5	5.44	0	7.17	0.87	2.81	6.77	4.33	4.23
15.59	23.94	0	0	0	0	4.3	0.8	3.9
0	5.44	2.4	5.12	2.32	0	1.22	2.37	0
0	7.62	5.61	6.15	7.68	2.38	0	3.36	0
4.59	0	4.81	0	1.65	0	0.61	2.34	1.15
0	55.49	0	0	2.75	0	4.66	2.45	8.34
5.5	7.62	4	5.12	2.22	1.13	0	0	1.38
10.09	7.62	7.21	9.22	12.03	0	3.05	4.86	2.52
7.34	0	4.81	0	1.53	0	2.51	1.77	6.93
8.25	0	0	0	1.97	5.59	1.07	1.11	3.76
0	34.82	6.41	3.07	2.14	0	6.65	0.69	0
0	0	0	0	0	3.81	5.14	1.17	0

mean.VancATN	mean.pooled.IgA E0 and E1	ATN.vs.NLTx.log2FC	ATN.vs.NLTx.FC	ATN.vs.NLTx.Pvalue	IgAN.vs.NLTx.log2FC	IgAN.vs.NLTx.FC	IgAN.vs.NLTx.Pvalue	IgA E1.vs.NLTx.log2FC
104.41		-0.15	-1.11	0.3847	0.07	1.05	0.7282	0.07
67.25		-0.14	-1.1	0.6164	0.34	1.26	0.2665	0.25
80.18		-0.13	-1.09	0.4396	-0.22	-1.17	0.2299	0.02
65.13		0.08	1.05	0.7536	0.3	1.23	0.2715	0.21
65.44		-0.06	-1.04	0.7109	-0.13	-1.09	0.4924	-0.07
32.71		-0.58	-1.5	0.0043	-0.53	-1.44	0.0194	-0.31
55.29		-0.05	-1.03	0.8124	-0.13	-1.1	0.5335	0.06
53.75		0.16	1.11	0.5471	0.07	1.05	0.8156	-0.21
62.55		0.31	1.24	0.2745	-0.31	-1.24	0.3509	0.17
43.03		-0.41	-1.33	0.026	0.09	1.07	0.6188	0.35
49.8		0.28	1.21	0.0848	0.07	1.05	0.6952	0.24
48.19		0.47	1.39	0.4299	0.52	1.44	0.4157	1.33
48.92		0.32	1.25	0.0777	0.27	1.2	0.1871	0.22
45.29		-0.15	-1.11	0.5773	-0.06	-1.04	0.8348	0.03
32.2		0.02	1.02	0.9165	0.54	1.46	0.0204	0.42
44.49		0.32	1.25	0.6722	0.15	1.11	0.841	0.94
51.16		0.24	1.18	0.696	0.72	1.64	0.27	0.79
37.12		-0.98	-1.97	0	-0.79	-1.73	0.0017	-0.58
37.31		0.17	1.13	0.3745	0.42	1.34	0.0417	0.17
36.16		0.08	1.06	0.7051	0.15	1.11	0.5404	0.2
30.82		-0.4	-1.32	0.0469	-0.18	-1.13	0.3966	-0.16
68.6		0.34	1.26	0.6195	0.83	1.77	0.2458	0.9
30.05		-0.09	-1.07	0.7391	0.61	1.53	0.0353	0.68
22.19		-0.03	-1.02	0.8805	-0.04	-1.03	0.8632	0.04
37.79		0.97	1.96	0.0095	0.91	1.88	0.0292	1.47
22.25		-0.36	-1.29	0.2047	0.25	1.19	0.4013	-0.07
20.15		0.08	1.06	0.7335	-0.1	-1.07	0.7155	-0.13
35.18		-0.07	-1.05	0.8703	0.61	1.52	0.2156	0.7
35.62		1.41	2.66	1.00E-04	2.57	5.95	0	2.33
22.16		0.01	1.01	0.9644	0.07	1.05	0.7807	-0.05
16.89		0.22	1.16	0.3802	0.27	1.21	0.313	0.35
15.93		-0.24	-1.18	0.3577	-0.03	-1.02	0.928	-0.1
18.35		0.34	1.27	0.1522	-0.14	-1.1	0.6191	-0.04
21.39		-0.54	-1.46	0.046	-0.66	-1.58	0.0315	-0.8
23.7		0.42	1.34	0.0836	-0.05	-1.04	0.8543	0.29
14.14		-0.8	-1.75	0.0362	-0.57	-1.48	0.1721	-0.59
22.88		0.61	1.53	0.3664	0.71	1.64	0.3123	1.39
10.95		-0.06	-1.05	0.8547	0.67	1.59	0.0685	0.39
17.16		0	-1	0.9909	0.62	1.53	0.0393	0.35
17.09		-0.64	-1.56	0.0212	-0.55	-1.46	0.0676	-1
27.07		1.11	2.15	0.016	0.72	1.65	0.1588	0.98

11.25	0.21	1.16	0.5406	0.19	1.14	0.6246	0.16
17.13	0.52	1.44	0.0829	0.16	1.11	0.6552	0.95
16.26	0.13	1.1	0.6295	-0.22	-1.16	0.4967	0
21.58	-0.13	-1.09	0.7083	-0.79	-1.73	0.0492	-0.38
13.69	0	1	0.9922	-0.01	-1.01	0.9658	-0.1
11.6	0.87	1.83	0.0215	-0.11	-1.08	0.8051	-0.11
12.41	-0.34	-1.26	0.2804	-0.02	-1.01	0.9556	-0.61
9.32	-0.14	-1.11	0.6687	0.58	1.5	0.0818	0.25
20.27	0.58	1.5	0.4528	0.61	1.53	0.4289	1.23
8.36	0.02	1.01	0.9588	-0.15	-1.11	0.7128	0.05
13.79	0.12	1.09	0.7103	-0.37	-1.29	0.3478	-0.19
9.47	0.85	1.8	0.0446	0.98	1.97	0.033	1.29
13.72	-0.27	-1.21	0.489	-0.6	-1.51	0.1776	-0.62
15.88	0.24	1.18	0.4775	-0.53	-1.44	0.1957	-0.02
13.37	-0.9	-1.86	0.0159	-0.73	-1.65	0.0661	-0.43
9.9	0.09	1.07	0.7688	-0.78	-1.72	0.057	-0.47
14.72	0.54	1.45	0.0587	-0.08	-1.06	0.822	-0.24
12.65	-0.74	-1.67	0.0439	-1.01	-2.01	0.0167	-0.78
6.38	-0.16	-1.11	0.6972	0.52	1.44	0.1918	0.17
23.46	0.59	1.5	0.4109	0.87	1.82	0.2376	1.12
12.5	0.39	1.31	0.2142	-0.63	-1.55	0.1251	-0.28
16.11	0.01	1	0.9842	-0.63	-1.54	0.1007	-0.05
12.09	0.31	1.24	0.3248	-0.4	-1.32	0.301	-0.15
12.42	0.08	1.06	0.8098	-0.67	-1.59	0.1042	-0.31
7.98	2.5	5.65	NA	0.82	1.76	NA	0.91
7.92	0.05	1.04	0.8665	0.04	1.03	0.9109	-0.45
10.9	-0.16	-1.11	0.655	0.05	1.03	0.9014	0.28
13.77	-0.51	-1.43	0.1678	-0.33	-1.25	0.4083	-0.55
12.23	0.24	1.18	0.5677	-0.61	-1.53	0.2166	0.1
9.96	0.04	1.03	0.9173	0.12	1.09	0.753	0.23
13.18	-0.08	-1.06	0.8188	-0.74	-1.67	0.0802	-0.19
20.48	2.4	5.26	1.00E-04	2.81	6.99	0	2.99
12.16	-0.47	-1.39	0.196	-0.76	-1.69	0.0702	-0.41
9.1	-0.74	-1.67	0.0615	-0.67	-1.59	0.116	-0.49
13.2	-0.25	-1.19	0.4217	0.11	1.08	0.7317	-0.23
2.33	0	-1	0.9979	0.69	1.62	0.1654	0.59
10.02	-0.04	-1.03	0.904	0.02	1.01	0.9643	0
7.41	0.29	1.22	0.4177	0.22	1.16	0.5813	0.31
43.87	0.18	1.14	0.3272	0.64	1.55	0.0011	0.48
7.45	-0.72	-1.65	0.0785	-0.43	-1.34	0.3145	-0.56
11.33	-0.43	-1.35	0.2725	-0.3	-1.23	0.4768	-0.29
13.79	-0.24	-1.18	0.5552	-0.51	-1.42	0.2603	-1.24
9.44	-0.31	-1.24	0.4163	-0.41	-1.33	0.3365	-0.3
9.77	0.04	1.03	0.9092	0.45	1.37	0.2274	0.01
8.12	-0.15	-1.11	0.7028	-0.2	-1.15	0.6374	-0.15
8.06	-0.16	-1.11	0.6911	0.31	1.24	0.4431	0.21
7.09	-0.11	-1.08	0.7708	-0.22	-1.16	0.5867	-0.3

9.01	0.45	1.36	0.2442	0.33	1.25	0.4444	0.46
10.11	0.05	1.03	0.8821	-0.21	-1.16	0.5643	0.03
10.44	0.28	1.22	0.4137	-0.18	-1.13	0.6603	-0.6
11.17	-0.3	-1.23	0.4783	-0.22	-1.16	0.6323	0
6.92	-0.15	-1.11	0.6879	-0.08	-1.06	0.8363	-0.16
8.03	0.31	1.24	0.5029	0.39	1.31	0.4304	-0.29
7.52	-0.25	-1.19	0.6253	-0.17	-1.12	0.7629	0.42
7.5	-0.51	-1.43	0.2118	0.51	1.42	0.1877	-0.71
30.24	-0.07	-1.05	0.8007	-0.43	-1.35	0.1693	-0.05
6.78	-0.14	-1.1	0.7465	-0.08	-1.06	0.8569	-0.29
13.94	1.89	3.71	0.0015	3.02	8.09	0	2.9
7.11	0.41	1.33	0.2752	0.28	1.21	0.5035	0.31
11.39	0.19	1.14	0.5348	0.28	1.22	0.3972	0.22
8.42	-0.15	-1.11	0.7076	-0.64	-1.56	0.1766	-0.15
12.07	1.93	3.81	0.0041	2.77	6.84	1.00E-04	3.1
9.13	1.53	2.89	8.00E-04	1.85	3.6	1.00E-04	1.72
4.78	-0.24	-1.18	0.5929	-0.11	-1.08	0.812	-0.25
11.91	1.45	2.74	0.0169	1.12	2.17	0.086	0.88
9.05	0.14	1.1	0.727	-0.35	-1.28	0.4584	-0.04
18.65	0.2	1.15	NA	0.61	1.53	NA	1.17
4.86	-0.13	-1.1	0.7504	-0.54	-1.45	0.2651	-0.17
6	-0.79	-1.72	0.0986	0.3	1.23	0.491	-0.05
13.66	-0.12	-1.09	0.6961	-0.07	-1.05	0.8323	-0.58
6.8	0.69	1.61	0.0854	-0.05	-1.04	0.9137	0.36
6.24	-0.04	-1.03	0.9259	0.42	1.33	0.3567	0.06
21.29	-0.01	-1.01	0.9616	-0.14	-1.1	0.6031	-0.15
6.76	-0.75	-1.69	0.1208	0.23	1.18	0.6077	0.06
6.89	-0.47	-1.38	0.3207	-0.86	-1.81	0.1085	-0.19
7.52	-0.19	-1.14	0.6303	0.53	1.45	0.196	0.29
5.2	-0.35	-1.28	0.4242	-0.06	-1.05	0.8879	-1.03
6.78	0.3	1.23	0.5189	0.26	1.19	0.6095	0.29
11.14	0.34	1.26	0.2687	-0.27	-1.21	0.4696	0.08
7.09	-0.41	-1.32	0.4035	-0.63	-1.54	0.2424	-0.49
4.15	-1.23	-2.34	0.0121	-1.87	-3.65	0.0011	-1.32
5.77	-0.36	-1.28	0.4271	-1.01	-2.01	0.0588	-0.28
6.02	-0.04	-1.03	0.939	0.33	1.26	0.5157	0.31
4.6	0.15	1.11	0.7675	0.97	1.96	0.0439	0.79
7.04	0.36	1.28	0.4422	-0.26	-1.2	0.6246	0.11
6.07	0	1	0.9957	-0.19	-1.14	0.6951	0.26
6.7	0.12	1.09	0.7816	-0.83	-1.77	0.1338	0.49
4.61	-1.04	-2.06	0.0463	-2.45	-5.45	1.00E-04	-1.41
7.26	-0.33	-1.25	0.4605	-1.27	-2.41	0.0213	-0.72
5.51	-0.26	-1.2	0.5282	-1.23	-2.35	0.0191	-0.74
6.97	0	-1	0.9936	0.13	1.1	0.8089	0.44
4.03	0.44	1.36	0.3188	0	1	0.9943	0.11
4.76	-0.91	-1.88	0.0557	-1.14	-2.21	0.0307	-1.37
5.01	0.04	1.03	0.9316	0.04	1.03	0.9364	-0.37

6.58	-0.83	-1.77	0.1468	-1.1	-2.15	0.0748	-1.16
6.23	0.51	1.42	0.2589	0.1	1.07	0.8381	0.31
6.07	-0.2	-1.15	0.6696	-0.12	-1.08	0.818	-0.22
6.61	-0.55	-1.46	0.2987	-0.51	-1.42	0.3678	0.22
3.43	-0.38	-1.3	0.4232	-0.57	-1.48	0.2788	-0.37
6.29	-0.19	-1.14	0.7031	-0.36	-1.28	0.5118	-0.33
3.37	-0.87	-1.83	0.1102	-0.78	-1.72	0.1739	-0.8
4.66	-0.08	-1.06	0.8548	-0.22	-1.16	0.6672	0.25
5.88	-0.02	-1.02	0.9625	0.05	1.03	0.9226	-0.36
6.28	0.24	1.18	0.6435	0.45	1.37	0.4003	0.33
3.16	-0.42	-1.34	0.4638	0.02	1.01	0.9785	0.27
8.26	-0.3	-1.24	0.4814	-0.64	-1.56	0.1929	-0.55
3.96	-1.15	-2.22	0.0523	-1.54	-2.91	0.0164	-1.92
3.64	-0.13	-1.09	0.7976	0.38	1.3	0.4441	0.1
4.76	1.88	3.68	0.0032	3.2	9.19	0	2.41
4.25	0.15	1.11	0.7473	0.01	1.01	0.9808	-0.25
4.4	-0.53	-1.44	0.3361	0.04	1.03	0.9431	-0.02
4.14	-0.21	-1.15	0.6634	-0.11	-1.08	0.8267	0.16
3.37	-0.56	-1.47	0.3172	-0.32	-1.25	0.5812	-0.31
4.83	-1.05	-2.07	0.0576	-0.16	-1.11	0.7636	0.01
4.55	-0.06	-1.04	0.9091	-0.15	-1.11	0.7776	-0.17
4.66	0.6	1.51	0.1994	-0.73	-1.66	0.214	-0.15
3.43	-1.04	-2.06	0.0678	-0.59	-1.5	0.3035	-0.98
0.53	-0.73	-1.65	0.2582	-1.27	-2.41	0.0648	0.02
4.78	0.91	1.88	0.0569	0.05	1.04	0.9282	0.51
9.18	-0.07	-1.05	0.8935	0.61	1.53	0.2516	0.11
4.9	0.69	1.62	0.2992	-1.02	-2.03	0.1585	-0.27
8.05	1.34	2.54	0.0885	0.35	1.27	0.6493	1.26
4.59	-1.26	-2.4	0.0219	-0.49	-1.4	0.3533	-1.19
2.84	-0.49	-1.41	0.3533	-0.22	-1.17	0.6821	0.25
2.31	-1.72	-3.3	0.0034	-1.49	-2.81	0.0135	-1.63
3.57	-1.54	-2.91	0.0118	-1.57	-2.96	0.0144	-0.24
5.41	0.71	1.63	0.1634	-0.14	-1.1	0.8188	0.38
4	0.13	1.09	0.8004	-0.32	-1.25	0.5784	0.34
5.66	-0.47	-1.39	0.3546	-0.91	-1.88	0.1131	-0.31
1.14	-1.56	-2.94	0.0161	0.91	1.88	0.1096	-0.07
4.06	-0.51	-1.43	0.3496	0.17	1.12	0.7525	-0.75
6.21	-0.29	-1.22	0.5883	-0.41	-1.33	0.4765	-0.35
4.69	0.06	1.04	0.9192	-0.86	-1.82	0.1952	-0.16
4.86	0.3	1.23	0.5178	0.3	1.23	0.5435	0.18
1.33	0.97	1.95	0.1609	-0.12	-1.08	0.8722	-0.14
1.6	-2.26	-4.78	7.00E-04	-2.22	-4.67	0.0013	-3.13
5.2	0.44	1.35	0.4242	1.54	2.91	0.0028	1.01
4.17	-0.28	-1.22	0.6082	-0.81	-1.75	0.1906	-0.22
4.59	-0.28	-1.22	0.5921	0	1	0.9955	-0.02
3.45	0.23	1.17	0.6482	0.15	1.11	0.7853	0.03
5.96	0.35	1.28	0.525	0.48	1.39	0.4114	1.33

4.4	0.98	1.97	0.0675	-0.07	-1.05	0.9092	0.78
8.36	0.14	1.1	0.7058	0.37	1.29	0.3397	0.2
4.15	-1.41	-2.65	0.0182	-1.39	-2.62	0.026	-1.49
1.9	-0.72	-1.65	0.2294	-0.09	-1.07	0.8771	-0.16
1.41	-1.34	-2.54	0.0354	-1.64	-3.11	0.0153	-0.9
2.89	0.89	1.85	0.1158	0.78	1.72	0.196	0.09
11.93	2.33	5.01	0.0018	0.48	1.4	0.5268	1.11
3.81	-0.9	-1.86	0.1479	-0.4	-1.32	0.5175	-0.02
3.84	-0.4	-1.32	0.4596	-0.82	-1.77	0.1713	-0.8
3.32	-1.45	-2.74	0.0168	-0.8	-1.74	0.1827	-1.03
5.07	-0.34	-1.27	0.5861	-0.75	-1.68	0.2652	-0.6
3.81	-0.1	-1.07	0.8486	-0.8	-1.74	0.1817	-0.48
6.02	0.71	1.64	0.2048	0.06	1.04	0.9226	-0.29
3.81	-0.08	-1.06	0.877	-0.92	-1.9	0.1405	-0.65
4.95	0.41	1.33	0.4303	-0.66	-1.58	0.288	0.08
3.45	0.65	1.57	0.2105	-0.34	-1.27	0.5763	-0.15
5.71	0.31	1.24	0.5496	-0.25	-1.19	0.6741	-0.32
5.51	0.65	1.57	0.2225	0.22	1.17	0.7069	0.17
4.83	0.48	1.39	0.3661	0.47	1.39	0.4024	0.23
4.29	0.13	1.09	0.8117	-0.36	-1.29	0.5465	0.33
3.18	0.42	1.34	0.4145	-0.04	-1.03	0.9448	-0.23
18.13	-0.18	-1.13	0.6199	-0.12	-1.09	0.7557	-0.29
3.69	-0.42	-1.34	0.4645	0	1	0.9995	-1.16
9.77	-0.41	-1.33	0.3328	-0.66	-1.58	0.1694	-0.61
2.74	-1.1	-2.14	0.089	0.05	1.03	0.9392	-0.76
4.54	0.35	1.27	0.5379	-0.03	-1.02	0.9662	0.1
2.19	0.04	1.03	0.94	0.47	1.38	0.4095	-0.22
2.91	-0.4	-1.32	0.5125	0.23	1.17	0.706	-0.14
3.11	-0.23	-1.17	0.6837	-0.29	-1.22	0.6348	-0.48
3.54	-0.02	-1.01	0.9733	-1.39	-2.62	0.0369	0
3.28	-1.16	-2.23	0.0739	-0.66	-1.58	0.312	-0.08
2.82	-0.63	-1.55	0.2778	-0.36	-1.28	0.5455	0.15
3.52	-0.26	-1.2	0.6518	-1.17	-2.25	0.0755	-0.06
4.66	0.21	1.16	0.7199	-0.3	-1.24	0.6395	0.07
4.49	-0.65	-1.57	0.2788	-1.16	-2.23	0.0761	-0.73
4	0.38	1.3	0.4786	-0.33	-1.26	0.5918	0.03
2.5	0.21	1.16	0.6834	-0.71	-1.64	0.2453	-0.69
3.6	-0.02	-1.02	0.9714	-0.31	-1.24	0.6569	-0.02
4.4	0.22	1.17	0.7266	-0.83	-1.78	0.233	-0.54
3.4	-3.07	-8.38	0	-2.48	-5.57	6.00E-04	-3.12
2.99	-0.6	-1.52	0.3321	-0.18	-1.13	0.7774	-0.7
3.43	-0.8	-1.74	0.2204	-0.29	-1.22	0.6604	-0.1
1.85	-1.09	-2.13	0.0663	-0.09	-1.06	0.8753	-0.78
7.09	1.65	3.14	0.0298	1.88	3.67	0.0146	1.96
3.01	-0.37	-1.29	0.5197	-0.28	-1.22	0.6327	-1.27
3.08	-0.03	-1.02	0.9525	-0.36	-1.29	0.5593	-0.71
7.83	0.45	1.37	0.2523	0.4	1.32	0.3526	0.35

3.16	-0.09	-1.06	0.8805	0.05	1.04	0.9303	-0.15
3.69	-0.54	-1.46	0.3407	-0.1	-1.07	0.8577	0.12
2.33	-0.4	-1.32	0.5109	-0.56	-1.47	0.3879	-0.85
3.43	-1.02	-2.03	0.1606	-0.57	-1.49	0.435	-0.33
2.11	0.38	1.3	0.507	-0.05	-1.03	0.9414	-0.79
0	-0.96	-1.94	0.1635	0.37	1.29	0.5719	0.05
5.44	1.41	2.66	0.0418	1.28	2.42	0.076	1.32
11.24	0.16	1.12	NA	0.32	1.25	NA	0.34
2.02	0.52	1.43	0.3798	0.41	1.33	0.5146	0.7
3.52	-0.01	-1	0.9915	-0.66	-1.58	0.3227	0.83
4.1	0.62	1.54	0.3163	-0.17	-1.12	0.807	-0.49
6.4	1.94	3.83	0.0095	0.82	1.76	0.2879	1.71
3.11	-0.22	-1.16	0.7238	-1.51	-2.85	0.0287	0.02
3.47	-0.31	-1.24	0.6571	-0.02	-1.02	0.9734	-0.35
6	-0.2	-1.15	0.7044	0.63	1.54	0.2612	0.27
2.57	-0.14	-1.1	0.8162	-1.24	-2.36	0.0689	-1.06
4.69	-0.13	-1.09	0.7922	-0.37	-1.29	0.4984	-0.62
2.04	-0.54	-1.45	0.3904	-1.02	-2.02	0.1326	-0.09
23.64	1.35	2.55	0.0069	1.39	2.62	0.0103	1.92
3.62	-0.71	-1.64	0.2323	-0.46	-1.38	0.4477	-0.81
0.27	-1.72	-3.29	0.0165	0.12	1.09	0.8626	-1.17
2.38	-0.55	-1.46	0.3748	0.1	1.07	0.8636	-0.25
3.67	-0.13	-1.1	0.8362	0.17	1.12	0.7974	0.33
2.57	-0.84	-1.79	0.2049	-0.35	-1.28	0.5942	-0.25
5.2	-0.63	-1.55	0.3282	-0.6	-1.52	0.3692	-0.68
2.07	-0.53	-1.44	0.4357	0.11	1.08	0.8684	-0.21
3.11	-1.66	-3.15	0.0085	-0.12	-1.09	0.8274	-0.83
2.67	-1.39	-2.62	0.0378	-0.62	-1.54	0.3408	-0.74
2.38	-1.38	-2.6	0.0393	-1.51	-2.85	0.0285	-0.55
2.21	-0.62	-1.54	0.3749	-0.39	-1.31	0.5847	-0.7
2.99	-0.11	-1.08	0.8732	-1.67	-3.19	0.0201	-0.72
3.22	-0.16	-1.12	0.7984	-0.38	-1.3	0.5737	-0.7
5.15	0.07	1.05	0.8712	0.25	1.19	0.5873	0.09
2.04	0.92	1.89	0.1035	0.38	1.3	0.5497	0.99
3.91	0.27	1.21	0.6635	-0.95	-1.94	0.1733	0.06
2.55	-1.58	-2.99	0.0223	-1.06	-2.09	0.1254	-1.38
2.59	-1.7	-3.25	0.0136	-1.17	-2.25	0.0882	-1.47
2.89	0.18	1.13	0.796	-1.18	-2.27	0.1035	-0.31
3.37	-0.64	-1.56	0.3066	-0.84	-1.79	0.2051	-0.53
4.22	-2.79	-6.93	0	0.38	1.3	0.533	-0.32
1.85	-2.27	-4.82	0.0012	-2.09	-4.25	0.003	-1.95
7.26	-0.26	-1.2	0.6354	-0.25	-1.19	0.6692	-0.25
2.69	-1.16	-2.24	0.0841	-1.31	-2.47	0.0596	-0.94
3.5	0.95	1.93	0.2107	0.73	1.66	0.3425	1.24
4.35	0.5	1.41	NA	0.26	1.19	NA	0.68
3.26	-0.97	-1.96	0.1777	-1.07	-2.1	0.1455	-1.33
2.74	-0.3	-1.23	0.6412	-1.02	-2.03	0.1447	-0.79

3.2	-0.35	-1.27	0.6008	-1.12	-2.17	0.113	0.02
2.33	-2.42	-5.37	7.00E-04	-1.4	-2.64	0.049	-2.48
2.55	0.11	1.08	0.8768	-0.72	-1.64	0.3329	0.08
2.11	-0.3	-1.23	0.6921	-0.57	-1.49	0.4499	-0.86
3.69	0.69	1.61	0.2973	-0.56	-1.47	0.4371	-0.82
1.24	-1.26	-2.39	0.0713	-1.01	-2.01	0.1541	-1.6
5.96	1.39	2.63	0.0759	0.04	1.03	0.9572	1.03
5.05	2.94	7.67	0	0.92	1.89	0.2396	1.98
0.27	-3.26	-9.59	0	-3.15	-8.9	0	-3.3
1.68	-0.36	-1.29	0.6067	-1.2	-2.29	0.102	-1.12
6.35	-0.35	-1.28	0.6403	1.89	3.71	0.0108	1.04
0.8	-0.72	-1.64	0.2816	-1.82	-3.52	0.0109	-2.47
1.33	-2.76	-6.79	2.00E-04	-2.64	-6.25	3.00E-04	-2.8
2.74	0.99	1.98	0.117	-0.73	-1.65	0.3101	-0.15
6.43	0.58	1.49	0.2173	-0.76	-1.69	0.1882	0.63
1.96	-1.47	-2.76	0.0422	-1.64	-3.11	0.0247	-1.53
0.88	1.3	2.45	0.0373	1.29	2.44	0.0493	0.94
6.53	0.46	1.38	NA	0.11	1.08	NA	0
11.9	0.55	1.46	0.4678	-0.71	-1.64	0.3534	1.1
0.27	-2.02	-4.05	0.0072	-1.91	-3.76	0.0086	-1.47
9.88	-0.25	-1.19	0.6441	0.08	1.06	0.8878	-0.13
6.24	-0.24	-1.18	0.6622	0.87	1.83	0.1245	0.51
2.68	2.7	6.52	4.00E-04	2.44	5.42	0.0017	2.61
5.67	0.66	1.58	0.3913	-0.27	-1.2	0.7266	0.69
7.73	-0.03	-1.02	0.9675	-1.46	-2.75	0.0576	-1
4.64	-1.43	-2.69	0.0659	-1.87	-3.66	0.0136	-1.61
4.13	0.76	1.69	0.3005	0.89	1.86	0.2331	0.73
6.39	0.7	1.63	0.3614	-0.32	-1.24	0.6667	0.07
0	-1.45	-2.74	0.0635	-0.13	-1.1	0.8614	-0.77
6.77	-2.32	-5	0.0029	-0.82	-1.76	0.291	-2.34
7.45	-0.13	-1.1	0.8636	0.99	1.98	0.1878	0.51
0	-1.9	-3.73	0.0093	-1.82	-3.52	0.0099	-1.54
4.2	-1.05	-2.07	0.1638	-0.37	-1.29	0.6243	0.33
7.98	0.06	1.04	NA	0.1	1.07	NA	0.25
4.32	-0.19	-1.14	0.804	-1.08	-2.12	0.1539	-0.56
6.46	0.71	1.64	0.3244	-0.42	-1.34	0.5778	-1.75
1.6	-0.35	-1.28	0.6386	-0.75	-1.68	0.2923	-0.64
18.5	0.19	1.14	NA	0.1	1.07	NA	0.31
5.58	2.19	4.57	0.0059	1.64	3.11	0.0348	0.83
8.01	0.63	1.55	0.4236	-0.76	-1.69	0.3221	-0.28
1.6	0.03	1.02	0.9655	-0.24	-1.18	0.7407	0.22
0	0.33	1.25	0.663	0.76	1.69	0.2927	0.13
14.77	0.72	1.65	NA	0.29	1.22	NA	1.34
0	-0.31	-1.24	0.6469	0.33	1.26	0.6063	0.6

IgA E1.vs.NLTx.FC	IgA E1.vs.NLTx.Pvalue	S.a.GN.vs.NLTx.log2FC	S.a.GN.vs.NLTx.FC	S.a.GN.vs.NLTx.Pvalue	VancATN.vs.NLTx.log2FC	VancATN.vs.NLTx.FC	VancATN.vs.NLTx.Pvalue
1.05	0.6904	0.09	1.07	0.5432	-0.42	-1.33	0.0177
1.19	0.3529	0.28	1.22	0.2417	-0.55	-1.46	0.0453
1.01	0.9108	-0.5	-1.41	8.00E-04	-0.22	-1.17	0.1593
1.16	0.3788	0.15	1.11	0.4705	-0.03	-1.02	0.9082
-1.05	0.6735	-0.33	-1.26	0.0259	0.06	1.04	0.7182
-1.24	0.1081	-0.33	-1.26	0.0523	-0.86	-1.82	0
1.05	0.7301	-0.52	-1.43	0.0032	-0.02	-1.01	0.9328
-1.15	0.4339	0.06	1.04	0.7966	0.08	1.06	0.7538
1.13	0.5405	0.06	1.04	0.8237	0.31	1.24	0.2708
1.27	0.0256	0.26	1.2	0.0578	-0.17	-1.13	0.2897
1.18	0.1231	0.14	1.1	0.3247	0.09	1.06	0.5729
2.52	0.0251	1.07	2.1	0.0564	0.98	1.98	0.0984
1.16	0.2294	-0.05	-1.04	0.7634	0.12	1.09	0.4829
1.02	0.9177	-0.07	-1.05	0.777	0.09	1.06	0.7348
1.33	0.047	0.43	1.34	0.0233	-0.3	-1.23	0.1715
1.92	0.2123	0.96	1.94	0.1951	0.84	1.79	0.2674
1.72	0.1946	1.05	2.07	0.0661	0.79	1.73	0.1926
-1.5	0.0064	-0.62	-1.54	0.001	-0.37	-1.29	0.0628
1.13	0.3608	0.06	1.04	0.7289	0.05	1.04	0.7815
1.15	0.3533	-0.11	-1.08	0.5681	-0.01	-1.01	0.95
-1.12	0.3954	-0.2	-1.15	0.2294	-0.32	-1.25	0.0825
1.87	0.1855	1.42	2.68	0.0286	1.46	2.74	0.0318
1.61	0.0075	0.76	1.69	0.001	0.19	1.14	0.4659
1.03	0.84	0.22	1.16	0.2522	-0.22	-1.17	0.3156
2.77	1.00E-04	0.46	1.37	0.1901	1.4	2.63	1.00E-04
-1.05	0.8092	-0.12	-1.09	0.6056	-0.12	-1.09	0.6546
-1.09	0.5812	0.09	1.07	0.6425	-0.3	-1.23	0.1947
1.63	0.1113	0.01	1.01	0.9839	0.91	1.88	0.0358
5.04	0	2.38	5.2	0	2.32	5.01	0
-1.04	0.8255	-0.1	-1.07	0.6289	-0.09	-1.07	0.6861
1.28	0.138	0.15	1.11	0.5015	-0.2	-1.15	0.4318
-1.07	0.6985	-0.23	-1.17	0.3	-0.55	-1.46	0.0344
-1.03	0.8689	0.07	1.05	0.7637	-0.25	-1.19	0.3163
-1.75	0.004	-1.02	-2.03	1.00E-04	-0.26	-1.2	0.2847
1.22	0.2358	-0.53	-1.45	0.032	0.31	1.24	0.1776
-1.51	0.1115	-0.54	-1.45	0.1077	-0.72	-1.64	0.0513
2.61	0.0387	1.41	2.66	0.0275	1.5	2.83	0.0248
1.31	0.2375	0.85	1.8	0.0036	-0.53	-1.45	0.1354
1.27	0.2092	0.59	1.51	0.0134	0.02	1.02	0.9349
-2	6.00E-04	-0.73	-1.66	0.0023	-0.45	-1.37	0.0673
1.98	0.0323	0.19	1.14	0.6674	1.12	2.17	0.0135

1.12	0.6314	0.04	1.03	0.8915	-0.58	-1.49	0.1062
1.93	7.00E-04	0.35	1.27	0.1957	0.32	1.25	0.2732
1	0.9858	-0.05	-1.04	0.8364	0.01	1.01	0.9738
-1.3	0.2611	-0.58	-1.5	0.0599	0.27	1.2	0.3865
-1.07	0.7597	0	1	0.9973	-0.01	-1.01	0.9662
-1.08	0.7956	0.24	1.18	0.5088	-0.04	-1.03	0.9254
-1.53	0.0577	-0.01	-1.01	0.9756	-0.25	-1.19	0.392
1.19	0.4301	0.43	1.34	0.117	-0.46	-1.38	0.1704
2.34	0.1119	1.27	2.41	0.0968	1.43	2.7	0.0638
1.03	0.8979	0.23	1.17	0.4864	-0.61	-1.53	0.1104
-1.14	0.5846	0.23	1.17	0.4158	0.15	1.11	0.623
2.45	0.0015	0.44	1.35	0.2671	0.19	1.14	0.6651
-1.54	0.1197	-1.41	-2.66	4.00E-04	-0.07	-1.05	0.8542
-1.01	0.9643	-0.14	-1.1	0.6548	0.32	1.25	0.3115
-1.35	0.1971	-0.59	-1.51	0.0523	-0.15	-1.11	0.6244
-1.39	0.1732	-0.52	-1.43	0.0933	-0.36	-1.29	0.2661
-1.18	0.4545	0.14	1.1	0.6035	0.16	1.12	0.5642
-1.72	0.0308	-0.98	-1.98	0.0029	-0.16	-1.12	0.6155
1.12	0.6537	0.58	1.49	0.0729	-0.49	-1.4	0.2206
2.18	0.1126	1.43	2.7	0.0354	1.47	2.77	0.0373
-1.22	0.4135	0.14	1.1	0.6132	0.19	1.14	0.5419
-1.03	0.8759	-0.14	-1.11	0.5994	0.18	1.13	0.5293
-1.11	0.6439	-0.56	-1.48	0.071	-0.05	-1.04	0.8713
-1.24	0.374	-0.02	-1.01	0.9565	0.03	1.02	0.9115
1.88	NA	1.01	2.01	NA	1.7	3.24	NA
-1.36	0.196	-0.38	-1.3	0.2046	-0.66	-1.58	0.0553
1.22	0.3691	0.34	1.27	0.2144	-0.01	-1.01	0.9772
-1.46	0.136	-0.63	-1.55	0.0546	0.06	1.04	0.8506
1.08	0.8021	0.02	1.01	0.9594	0.19	1.14	0.6357
1.17	0.4988	0.3	1.23	0.303	0.06	1.04	0.8579
-1.14	0.5772	-0.2	-1.15	0.5028	0.23	1.17	0.4455
7.94	0	2.92	7.55	0	3.2	9.2	0
-1.33	0.2422	-0.24	-1.18	0.4386	-0.08	-1.06	0.7955
-1.4	0.1873	-0.43	-1.34	0.1892	-0.44	-1.36	0.2118
-1.17	0.4563	0.16	1.11	0.5394	-0.46	-1.37	0.1312
1.5	0.2005	0.88	1.84	0.0325	-1.15	-2.23	0.0302
1	0.9986	0.48	1.39	0.0999	0.1	1.07	0.7748
1.24	0.3702	0.13	1.09	0.6936	-0.22	-1.16	0.5498
1.39	0.007	0.52	1.44	9.00E-04	0.1	1.07	0.5885
-1.47	0.1491	0.07	1.05	0.8197	-0.46	-1.38	0.2091
-1.22	0.4436	-0.23	-1.17	0.4841	0.05	1.04	0.8835
-2.37	0.0068	-0.53	-1.44	0.1491	0.44	1.36	0.2066
-1.23	0.4252	0.02	1.01	0.9567	-0.15	-1.11	0.6749
1.01	0.9731	-0.56	-1.47	0.1117	0.04	1.03	0.9032
-1.11	0.6856	0.02	1.02	0.9418	-0.24	-1.18	0.5081
1.16	0.56	0.03	1.02	0.9213	-0.21	-1.15	0.5852
-1.23	0.4134	-0.26	-1.2	0.4193	-0.39	-1.31	0.2753

1.37	0.2229	0.64	1.56	0.052	0.51	1.42	0.1568
1.02	0.9123	-0.54	-1.46	0.0783	-0.3	-1.23	0.3502
-1.52	0.1299	-0.06	-1.04	0.8537	0.22	1.16	0.5037
1	0.9924	0.01	1	0.9876	0.44	1.35	0.2326
-1.12	0.6502	-0.04	-1.03	0.8986	-0.53	-1.44	0.1475
-1.22	0.5543	0.26	1.2	0.5283	0.21	1.15	0.6484
1.34	0.3815	0.68	1.6	0.1185	0.02	1.01	0.969
-1.64	0.0877	-0.23	-1.17	0.5047	-0.3	-1.23	0.4215
-1.04	0.8405	-0.39	-1.31	0.1094	0.1	1.07	0.6789
-1.22	0.5003	0.04	1.03	0.9072	-0.28	-1.21	0.5118
7.45	0	2.4	5.26	0	2.73	6.64	0
1.24	0.4151	0.47	1.38	0.1585	-0.01	-1.01	0.9702
1.17	0.454	-0.15	-1.11	0.6093	-0.06	-1.04	0.839
-1.11	0.7031	-0.47	-1.38	0.2081	-0.01	-1.01	0.9711
8.58	0	2.36	5.15	2.00E-04	2.65	6.27	1.00E-04
3.29	1.00E-04	1.31	2.48	0.002	1.42	2.68	0.0014
-1.19	0.5664	0.23	1.18	0.5214	-0.44	-1.36	0.3063
1.83	0.1573	1.29	2.45	0.0238	1.8	3.49	0.0024
-1.03	0.9199	-0.03	-1.02	0.9451	0.35	1.27	0.3507
2.25	NA	0.98	1.97	NA	1.21	2.32	NA
-1.12	0.6835	-0.53	-1.45	0.1693	-0.58	-1.5	0.1667
-1.04	0.9007	0.43	1.35	0.2149	-0.19	-1.14	0.6397
-1.49	0.0708	-0.31	-1.24	0.2447	-0.26	-1.2	0.3755
1.28	0.3788	0	1	0.9939	0.15	1.11	0.7181
1.04	0.8841	0.47	1.39	0.198	-0.04	-1.03	0.9176
-1.11	0.5191	0.11	1.08	0.5612	-0.26	-1.19	0.2647
1.04	0.8852	-0.32	-1.25	0.4193	-0.02	-1.01	0.9695
-1.14	0.6756	-0.53	-1.44	0.209	-0.11	-1.08	0.8025
1.23	0.4356	0.7	1.62	0.0352	-0.8	-1.74	0.0516
-2.04	0.0339	0.11	1.08	0.7548	-0.34	-1.27	0.4143
1.22	0.522	0.01	1.01	0.9748	0.3	1.23	0.4922
1.06	0.7921	0.25	1.19	0.3588	-0.04	-1.03	0.8879
-1.4	0.3131	-0.26	-1.2	0.5419	-0.15	-1.11	0.7445
-2.5	0.0066	-1.57	-2.97	5.00E-04	-1.1	-2.15	0.0151
-1.21	0.5235	-0.37	-1.29	0.3459	-0.31	-1.24	0.4565
1.24	0.5005	0.96	1.95	0.0138	0.29	1.22	0.5236
1.73	0.0781	0.82	1.77	0.0437	0.16	1.12	0.7371
1.08	0.8129	-0.54	-1.45	0.2435	0.24	1.18	0.5961
1.2	0.5117	0.38	1.3	0.275	0	-1	0.9999
1.41	0.2358	-0.49	-1.41	0.2542	0.25	1.19	0.5485
-2.65	0.0087	-2.95	-7.7	0	-1.06	-2.09	0.0346
-1.65	0.1182	-1	-2.01	0.0204	-0.07	-1.05	0.8717
-1.67	0.092	-1.66	-3.15	3.00E-04	-0.53	-1.44	0.1966
1.36	0.3591	0.52	1.43	0.2353	0.61	1.52	0.186
1.08	0.8086	-0.11	-1.08	0.7921	-0.29	-1.22	0.531
-2.59	0.006	-1.2	-2.3	0.0059	-0.81	-1.75	0.0666
-1.3	0.4314	-0.31	-1.24	0.4608	-0.09	-1.06	0.8361

-2.24	0.0451	-1.45	-2.74	0.0085	-0.05	-1.03	0.9241
1.24	0.4935	-0.2	-1.15	0.6486	0.19	1.14	0.6631
-1.17	0.6365	-0.51	-1.43	0.2483	0.09	1.06	0.8419
1.17	0.6375	-0.51	-1.42	0.2803	0.2	1.15	0.6644
-1.29	0.4273	-1.06	-2.09	0.0228	-0.77	-1.7	0.1073
-1.26	0.5124	-0.22	-1.16	0.6255	0.13	1.09	0.7805
-1.75	0.13	0.01	1.01	0.9863	-0.47	-1.38	0.336
1.19	0.561	-0.58	-1.49	0.1835	-0.2	-1.15	0.6518
-1.28	0.4494	0.02	1.01	0.9702	0.19	1.14	0.6456
1.26	0.51	0.65	1.57	0.1436	0.56	1.47	0.24
1.21	0.6028	0.45	1.37	0.3416	-0.29	-1.22	0.5911
-1.47	0.2085	-0.5	-1.42	0.1997	-0.26	-1.2	0.529
-3.79	0.002	-2.5	-5.67	0	-1.07	-2.09	0.0628
1.07	0.8315	0.59	1.51	0.1384	-0.2	-1.15	0.6737
5.33	1.00E-04	1.82	3.52	0.0024	1.95	3.87	0.0017
-1.19	0.6128	-0.08	-1.05	0.8606	-0.36	-1.28	0.4543
-1.01	0.972	-0.28	-1.22	0.5551	0	-1	0.9955
1.12	0.7158	0.01	1.01	0.9835	-0.5	-1.42	0.2834
-1.24	0.565	0.37	1.3	0.4102	-0.37	-1.29	0.4764
1.01	0.9873	0.23	1.17	0.5712	-0.12	-1.09	0.7916
-1.13	0.7227	-0.04	-1.03	0.9201	-0.05	-1.04	0.9129
-1.11	0.7663	0.07	1.05	0.8776	0.13	1.1	0.7768
-1.97	0.0791	-0.44	-1.36	0.3488	-0.46	-1.37	0.3624
1.02	0.9714	0.1	1.07	0.8552	-1.72	-3.28	0.0104
1.42	0.3042	-0.2	-1.15	0.6882	0.36	1.28	0.4596
1.08	0.8382	0.46	1.38	0.3092	1.4	2.64	0.0013
-1.21	0.6965	0.95	1.94	0.1251	0.95	1.94	0.143
2.4	0.1096	1.44	2.72	0.0689	1.31	2.48	0.0975
-2.27	0.0266	-0.97	-1.96	0.0374	-0.3	-1.23	0.5078
1.19	0.5883	0.12	1.09	0.7759	-0.5	-1.42	0.3155
-3.08	0.0044	-1.56	-2.94	0.0025	-1.3	-2.46	0.0138
-1.18	0.6444	0.1	1.07	0.8317	-0.47	-1.38	0.3696
1.3	0.4636	-0.53	-1.44	0.3174	0.51	1.42	0.3085
1.27	0.4819	0.42	1.34	0.3361	0.16	1.12	0.7417
-1.24	0.5282	-0.54	-1.46	0.2346	0.03	1.02	0.9506
-1.05	0.9001	-0.07	-1.05	0.8949	-1.35	-2.55	0.0284
-1.68	0.1746	-0.57	-1.48	0.2498	-0.08	-1.06	0.8702
-1.27	0.5098	-0.58	-1.49	0.2469	0.45	1.37	0.337
-1.11	0.7937	-1.39	-2.62	0.0255	0.22	1.17	0.6952
1.13	0.6923	0.12	1.09	0.7768	-0.11	-1.08	0.8174
-1.1	0.8394	-0.23	-1.17	0.7414	-0.46	-1.37	0.5204
-8.76	0	-3.8	-13.97	0	-2.08	-4.24	0.0012
2.01	0.0446	0.82	1.77	0.0808	0.97	1.96	0.0477
-1.16	0.684	0.31	1.24	0.4914	0.19	1.14	0.7045
-1.01	0.9672	-0.19	-1.14	0.6746	0.11	1.08	0.8126
1.02	0.9566	-0.14	-1.1	0.7693	-0.05	-1.04	0.9184
2.51	0.0072	0.36	1.28	0.4792	1.11	2.16	0.0244

1.72	0.1462	0.43	1.35	0.3982	0.72	1.64	0.175
1.15	0.5756	-0.08	-1.06	0.7995	-0.15	-1.11	0.6708
-2.81	0.0119	-0.85	-1.8	0.086	-0.36	-1.28	0.4728
-1.12	0.7775	-0.17	-1.13	0.7382	-0.77	-1.7	0.1867
-1.86	0.1401	-1.03	-2.05	0.0697	-1.41	-2.65	0.0237
1.07	0.877	0.2	1.15	0.7164	0.14	1.1	0.8072
2.15	0.1421	1.02	2.03	0.165	2.68	6.43	3.00E-04
-1.01	0.9691	0.28	1.22	0.5563	0.29	1.22	0.5728
-1.75	0.1498	-0.02	-1.01	0.9625	-0.18	-1.13	0.722
-2.04	0.071	-1.29	-2.45	0.0169	-0.67	-1.59	0.2086
-1.51	0.3448	-1.46	-2.75	0.0213	0.19	1.14	0.7522
-1.39	0.3729	-0.45	-1.37	0.3507	-0.16	-1.12	0.7403
-1.23	0.6294	-0.06	-1.04	0.917	0.84	1.79	0.1207
-1.57	0.2508	-0.4	-1.32	0.4229	-0.01	-1.01	0.9796
1.05	0.8848	-0.17	-1.13	0.7315	0.58	1.5	0.2267
-1.11	0.7952	0.11	1.08	0.8183	0.19	1.14	0.7118
-1.25	0.5639	0.43	1.35	0.3481	0.65	1.57	0.1758
1.13	0.7559	-0.15	-1.11	0.7768	0.92	1.9	0.0608
1.17	0.6711	0.45	1.36	0.3502	0.66	1.59	0.1802
1.25	0.5276	-0.35	-1.28	0.4938	0.14	1.1	0.7852
-1.18	0.6747	0.35	1.27	0.4567	-0.01	-1.01	0.9861
-1.22	0.4189	-0.02	-1.01	0.955	-0.26	-1.2	0.4571
-2.23	0.0613	-0.4	-1.32	0.4426	0.09	1.06	0.8611
-1.53	0.1539	-0.78	-1.72	0.0483	-0.49	-1.41	0.2343
-1.7	0.2194	0.27	1.21	0.602	-0.37	-1.29	0.5322
1.07	0.8593	0.21	1.15	0.6921	0.61	1.52	0.2534
-1.17	0.7006	0.32	1.25	0.5176	-0.23	-1.18	0.6772
-1.1	0.8101	0.34	1.26	0.5047	-0.01	-1.01	0.9891
-1.39	0.402	-0.25	-1.19	0.6301	-0.27	-1.21	0.6192
1	0.9955	-0.73	-1.66	0.1831	0.08	1.06	0.8797
-1.06	0.8866	-0.48	-1.39	0.3957	-0.14	-1.1	0.8137
1.11	0.773	-0.11	-1.08	0.8244	-0.27	-1.2	0.6128
-1.04	0.9166	-0.74	-1.68	0.1804	-0.05	-1.04	0.9253
1.05	0.908	0.08	1.06	0.8801	0.52	1.43	0.3583
-1.66	0.2202	-1	-2	0.0759	-0.09	-1.07	0.8678
1.02	0.9555	0.03	1.02	0.9588	0.33	1.26	0.5189
-1.62	0.2239	-0.34	-1.26	0.4974	-0.54	-1.46	0.319
-1.02	0.9733	-0.29	-1.22	0.6417	0.23	1.17	0.7152
-1.45	0.4135	-0.19	-1.14	0.7521	0.44	1.36	0.4707
-8.67	0	-2.99	-7.97	0	-0.8	-1.74	0.2167
-1.62	0.2608	-0.65	-1.57	0.2586	-0.15	-1.11	0.7914
-1.07	0.8712	0.62	1.53	0.2447	0.25	1.19	0.6668
-1.72	0.1636	-0.23	-1.18	0.6188	-0.95	-1.93	0.0877
3.88	0.0093	2.31	4.94	0.0014	3	7.98	0
-2.41	0.0404	-0.31	-1.24	0.5383	-0.27	-1.2	0.6183
-1.63	0.2454	-0.3	-1.23	0.5761	0.1	1.07	0.8518
1.27	0.3746	0.17	1.13	0.637	0.15	1.11	0.698

-1.11	0.7892	0.38	1.3	0.4393	0.08	1.06	0.8805		
1.09	0.8199	-1.21	-2.31	0.031	-0.19	-1.14	0.7118		
-1.81	0.173	-0.46	-1.38	0.4057	-0.37	-1.29	0.5282		
-1.26	0.644	-1.18	-2.27	0.0964	0.17	1.12	0.8084		
-1.73	0.2118	-0.66	-1.58	0.2597	-0.42	-1.34	0.4873		
1.04	0.9348	1.08	2.12	0.0488	-1.79	-3.47	0.0106		
2.49	0.0572	1.58	2.98	0.0149	2.39	5.26	2.00E-04		
1.27	NA	0.34	1.27	NA	0.5	1.41	NA		
1.62	0.2208	0.97	1.96	0.0583	0.1	1.07	0.8712		
1.78	0.1314	-0.5	-1.41	0.3982	0.45	1.36	0.4271		
-1.4	0.463	-0.26	-1.2	0.6692	0.87	1.83	0.1397		
3.26	0.0228	2.16	4.47	0.0026	2.65	6.27	3.00E-04		
1.02	0.9695	0.15	1.11	0.7744	0.29	1.22	0.6064		
-1.28	0.6133	0.15	1.11	0.8158	0.53	1.44	0.4223		
1.2	0.6068	0.5	1.42	0.2867	-0.71	-1.64	0.1812		
-2.08	0.1022	-1.07	-2.09	0.0783	-0.51	-1.43	0.3986		
-1.53	0.2296	-0.1	-1.07	0.8158	-0.04	-1.03	0.9321		
-1.07	0.8774	-0.52	-1.44	0.3626	-0.43	-1.35	0.4757		
3.78	1.00E-04	0.72	1.65	0.1297	1.9	3.74	1.00E-04		
-1.76	0.1727	-1.42	-2.67	0.0168	-0.06	-1.04	0.91		
-2.25	0.0933	-1.07	-2.1	0.1069	-1.88	-3.69	0.0082		
-1.19	0.6736	0.11	1.08	0.8353	-0.12	-1.09	0.8311		
1.25	0.5908	-0.4	-1.32	0.5092	0.56	1.47	0.334		
-1.19	0.6863	-0.86	-1.81	0.1632	-0.1	-1.07	0.8693		
-1.6	0.2888	-1.1	-2.14	0.0793	0.77	1.71	0.159		
-1.16	0.7509	0.25	1.19	0.6735	-0.29	-1.22	0.6601		
-1.77	0.1404	-0.95	-1.94	0.0662	-0.42	-1.33	0.4164		
-1.67	0.2386	-0.95	-1.93	0.1104	-0.28	-1.21	0.6325		
-1.46	0.3758	-0.49	-1.41	0.3864	-0.4	-1.32	0.5042		
-1.62	0.3183	0.07	1.05	0.909	-0.07	-1.05	0.9173		
-1.65	0.2981	-1.55	-2.92	0.0261	0.08	1.06	0.8966		
-1.63	0.2851	-0.32	-1.25	0.5876	0.05	1.04	0.9352		
1.07	0.8273	-0.1	-1.07	0.8081	-0.34	-1.27	0.4446		
1.99	0.0721	0.8	1.74	0.1229	0.18	1.13	0.7652		
1.04	0.9215	-0.32	-1.25	0.5995	0.6	1.52	0.3147		
-2.6	0.0428	-1.49	-2.81	0.0218	-0.47	-1.39	0.4498		
-2.76	0.0294	-2.79	-6.9	1.00E-04	-0.5	-1.42	0.4096		
-1.24	0.6563	-1.79	-3.47	0.0117	0.02	1.02	0.9726		
-1.45	0.3836	-1.54	-2.91	0.0145	0	-1	0.9965		
-1.25	0.5815	0.52	1.43	0.3213	-1.16	-2.24	0.0491		
-3.86	0.0044	-2.29	-4.89	6.00E-04	-1.1	-2.14	0.0789		
-1.19	0.6444	-1.05	-2.07	0.0559	0.66	1.58	0.1731		
-1.92	0.151	0.2	1.15	0.7098	-0.11	-1.08	0.8509		
2.37	0.0983	-0.06	-1.04	0.9369	1.19	2.28	0.1133		
1.61	NA	1.26	2.4	NA	1.22	2.33	NA		
-2.52	0.0671	-1.94	-3.83	0.0075	0.05	1.03	0.9448		
-1.73	0.2378	-1.21	-2.31	0.0647	-0.17	-1.12	0.7905		

1.01	0.9789	-0.36	-1.28	0.5642	0.14	1.1	0.8234
-5.56	5.00E-04	-1.69	-3.22	0.0122	-0.58	-1.5	0.3627
1.06	0.9087	-0.37	-1.3	0.5924	0.3	1.23	0.6734
-1.82	0.2559	-0.12	-1.08	0.8731	-0.16	-1.12	0.8299
-1.76	0.2502	-1.84	-3.59	0.0106	0.71	1.64	0.2707
-3.03	0.023	-2.23	-4.69	0.0015	-1.17	-2.25	0.0839
2.04	0.1902	-0.14	-1.1	0.8615	2	4	0.0106
3.93	0.0089	2.8	6.96	1.00E-04	3.44	10.87	0
-9.86	0	-3.02	-8.14	0	-2.87	-7.33	1.00E-04
-2.18	0.1212	-0.93	-1.91	0.1802	-0.41	-1.33	0.5583
2.05	0.1566	1.49	2.81	0.0324	1.96	3.9	0.0058
-5.54	5.00E-04	-1.5	-2.83	0.0229	-1.56	-2.95	0.0231
-6.97	2.00E-04	-3.01	-8.07	1.00E-04	-1.31	-2.47	0.0772
-1.11	0.8276	-0.75	-1.68	0.2656	0.53	1.45	0.4013
1.55	0.1682	-0.56	-1.48	0.2343	0.18	1.13	0.7048
-2.9	0.0332	-2.17	-4.49	0.0026	-0.69	-1.61	0.3143
1.93	0.1341	1.32	2.49	0.0227	-0.23	-1.18	0.7272
1	NA	0.67	1.59	NA	0.78	1.72	NA
2.14	0.1468	0.96	1.94	0.1965	0.38	1.3	0.6154
-2.76	0.0565	-2.2	-4.6	0.0045	-1.77	-3.42	0.0208
-1.1	0.8046	0.35	1.28	0.4662	0.29	1.23	0.5695
1.42	0.335	0.84	1.79	0.0841	-0.32	-1.25	0.5513
6.09	6.00E-04	2.2	4.59	0.0032	2.36	5.15	0.0018
1.62	0.3661	-0.23	-1.18	0.7571	-0.14	-1.1	0.8512
-2	0.1948	-0.23	-1.17	0.762	-0.58	-1.49	0.4558
-3.05	0.0378	-2.08	-4.23	0.0073	-0.24	-1.18	0.7565
1.66	0.3206	-0.3	-1.23	0.6785	0.87	1.82	0.2345
1.05	0.9239	-0.18	-1.13	0.8185	0.19	1.14	0.8051
-1.71	0.3255	0.08	1.05	0.9236	-1.48	-2.8	0.0589
-5.07	0.0027	-2.61	-6.12	9.00E-04	-0.72	-1.65	0.3582
1.43	0.5082	1.01	2.01	0.2028	0.75	1.68	0.3341
-2.91	0.0393	-2.05	-4.14	0.0071	-1.95	-3.86	0.0086
1.26	0.6556	-0.05	-1.04	0.9401	-0.09	-1.06	0.9049
1.19	NA	0.25	1.19	NA	0.39	1.31	NA
-1.47	0.4754	-1.53	-2.9	0.0511	0.3	1.23	0.7063
-3.35	0.02	-2.07	-4.19	0.0056	0.54	1.46	0.4524
-1.55	0.3954	-0.48	-1.39	0.538	-0.33	-1.26	0.6599
1.24	NA	0.53	1.45	NA	0.87	1.83	NA
1.78	0.2886	1.87	3.65	0.0192	3.05	8.3	1.00E-04
-1.22	0.7182	-0.45	-1.36	0.5702	0.32	1.25	0.6869
1.17	0.7724	0.92	1.89	0.2438	0.04	1.03	0.9591
1.1	0.8573	0.72	1.64	0.3545	-0.21	-1.15	0.7817
2.54	NA	-0.09	-1.06	NA	1.98	3.93	NA
1.51	0.3818	-0.41	-1.33	0.5565	-0.32	-1.25	0.6373

			Log2 (Fold Change), Fol		
pooled.IgAN.vs.NLTx.log2FoldChange	pooled.IgAN.vs.NLTx.FoldChange	pooled.IgAN.vs.NLTx.pvalue	IgAN E0.vs	IgAN E0.vs	IgAN E0.vs
0.07	1.05	0.6210	0.22	1.17	0.3343
0.29	1.23	0.1897	0.48	1.39	0.1749
-0.08	-1.05	0.5704	-0.1	-1.07	0.6559
0.25	1.19	0.2094	0.22	1.16	0.479
-0.09	-1.07	0.4889	-0.07	-1.05	0.7624
-0.40	-1.32	0.0146	0.05	1.04	0.8436
-0.01	-1.01	0.9274	-0.09	-1.06	0.7247
-0.09	-1.06	0.6816	-0.09	-1.06	0.7953
-0.01	-1.01	0.9765	-0.62	-1.54	0.0991
0.25	1.19	0.0653	0.5	1.42	0.0308
0.18	1.13	0.1915	-0.21	-1.15	0.3423
1.11	2.16	0.0412	0.05	1.03	0.944
0.24	1.18	0.1073	-0.05	-1.04	0.8271
-0.01	-1.01	0.9647	0.09	1.06	0.8022
0.48	1.39	0.0062	0.52	1.43	0.0628
0.72	1.65	0.3326	-0.17	-1.12	0.834
0.80	1.74	0.1436	0.48	1.39	0.5026
-0.67	-1.59	0.0002	0.19	1.14	0.552
0.28	1.22	0.0724	0.25	1.19	0.3155
0.18	1.13	0.3065	0.07	1.05	0.8186
-0.17	-1.12	0.2789	0.22	1.17	0.403
0.88	1.84	0.1825	0.49	1.4	0.5256
0.66	1.58	0.0019	0.71	1.63	0.0431
0.01	1.01	0.9640	-0.01	-1.01	0.9756
1.29	2.44	0.0001	-0.06	-1.04	0.8922
0.08	1.05	0.7310	0.61	1.53	0.0887
-0.12	-1.08	0.5475	-0.17	-1.13	0.5723
0.69	1.61	0.0653	0.68	1.6	0.2244
2.47	5.53	0.0000	1.16	2.24	0.0033
0.00	1.00	0.9902	0.06	1.04	0.8419
0.33	1.25	0.1041	0.06	1.04	0.8627
-0.06	-1.04	0.7576	0.21	1.16	0.5231
-0.08	-1.06	0.6843	-0.49	-1.4	0.1389
-0.75	-1.68	0.0009	-0.12	-1.09	0.747
0.16	1.11	0.4557	-0.47	-1.39	0.1534
-0.59	-1.50	0.0580	0.24	1.18	0.6292
1.21	2.31	0.0534	0.1	1.07	0.8934
0.53	1.44	0.0576	0.73	1.66	0.0925
0.47	1.39	0.0388	0.62	1.54	0.088
-0.80	-1.74	0.0005	0.09	1.06	0.807
0.90	1.87	0.0230	-0.39	-1.31	0.4971

0.18	1.13		0.5223	-0.02	-1.02	0.9572
0.67	1.59		0.0100	-0.37	-1.29	0.3555
-0.09	-1.06		0.7052	-0.35	-1.28	0.3465
-0.56	-1.47		0.0559	-0.67	-1.59	0.1471
-0.06	-1.04		0.8084	-0.02	-1.01	0.965
-0.13	-1.09		0.7138	-0.98	-1.98	0.0494
-0.34	-1.26		0.1871	0.32	1.25	0.4213
0.41	1.33		0.1144	0.73	1.65	0.0768
1.10	2.15		0.1520	0.03	1.02	0.9697
-0.03	-1.02		0.9151	-0.17	-1.13	0.7199
-0.27	-1.21		0.3459	-0.49	-1.4	0.2748
1.21	2.31		0.0006	0.13	1.1	0.8002
-0.62	-1.54		0.0617	-0.33	-1.26	0.5212
-0.22	-1.17		0.4521	-0.77	-1.7	0.0974
-0.55	-1.47		0.0517	0.17	1.13	0.7255
-0.61	-1.53		0.0396	-0.88	-1.84	0.0585
-0.18	-1.13		0.4969	-0.62	-1.53	0.114
-0.89	-1.85		0.0036	-0.27	-1.21	0.591
0.34	1.27		0.2738	0.68	1.6	0.162
1.08	2.12		0.1025	0.28	1.21	0.7197
-0.44	-1.36		0.1384	-1.02	-2.03	0.0256
-0.27	-1.21		0.3140	-0.63	-1.55	0.1476
-0.26	-1.19		0.3585	-0.71	-1.63	0.1047
-0.46	-1.38		0.1170	-0.75	-1.68	0.1092
0.71	1.64	NA		-1.68	-3.21	NA
-0.22	-1.17		0.4279	-0.01	-1.01	0.9722
0.19	1.14		0.4769	0.2	1.15	0.6497
-0.46	-1.37		0.1261	0.19	1.14	0.6979
-0.17	-1.12		0.6533	-0.85	-1.8	0.1259
0.18	1.14		0.5150	0.08	1.06	0.8541
-0.41	-1.33		0.1693	-0.66	-1.58	0.1721
3.06	8.36		0.0000	0.41	1.33	0.5514
-0.56	-1.48		0.0634	-0.29	-1.22	0.5611
-0.57	-1.48		0.0688	0.07	1.05	0.8954
-0.07	-1.05		0.7659	0.36	1.28	0.358
0.67	1.59		0.0817	0.69	1.62	0.2351
0.00	1.00		0.9887	0.06	1.04	0.8952
0.28	1.21		0.3445	-0.07	-1.05	0.8745
0.55	1.46		0.0002	0.45	1.37	0.0543
-0.51	-1.43		0.1111	0.29	1.22	0.5739
-0.30	-1.23		0.3410	0.13	1.1	0.7936
-0.94	-1.91		0.0115	-0.27	-1.21	0.6031
-0.35	-1.28		0.2634	-0.1	-1.07	0.8472
0.22	1.17		0.4490	0.41	1.33	0.3611
-0.17	-1.13		0.5815	-0.05	-1.04	0.9159
0.27	1.20		0.3838	0.47	1.38	0.3375
-0.27	-1.21		0.3798	-0.11	-1.08	0.8111

0.41	1.32	0.2124	-0.12	-1.09	0.801
-0.06	-1.04	0.8204	-0.26	-1.2	0.5442
-0.43	-1.35	0.1846	-0.46	-1.38	0.3216
-0.09	-1.07	0.7892	0.08	1.06	0.8778
-0.13	-1.09	0.6715	0.06	1.05	0.8898
0.03	1.02	0.9332	0.08	1.06	0.8839
0.21	1.16	0.6161	0.08	1.06	0.8936
-0.08	-1.06	0.8200	1.02	2.03	0.0366
-0.20	-1.15	0.3739	-0.36	-1.29	0.3171
-0.21	-1.15	0.5694	0.06	1.04	0.919
3.10	8.60	0.0000	1.12	2.18	0.0839
0.30	1.23	0.3494	-0.13	-1.1	0.7839
0.26	1.19	0.3100	0.09	1.06	0.8192
-0.35	-1.27	0.3173	-0.49	-1.4	0.3707
3.21	9.24	0.0000	0.84	1.79	0.2511
1.82	3.54	0.0000	0.32	1.24	0.5404
-0.19	-1.14	0.5988	0.13	1.09	0.823
0.99	1.99	0.0731	-0.33	-1.26	0.6363
-0.18	-1.13	0.6144	-0.49	-1.41	0.3625
1.01	2.01	NA	0.4	1.32	NA
-0.31	-1.24	0.3801	-0.4	-1.32	0.4677
0.12	1.08	0.7335	1.08	2.12	0.053
-0.35	-1.27	0.1821	0.05	1.03	0.9024
0.20	1.15	0.5808	-0.74	-1.67	0.1638
0.23	1.17	0.5195	0.46	1.37	0.3959
-0.15	-1.11	0.4479	-0.13	-1.09	0.6858
0.15	1.11	0.6609	0.99	1.98	0.0869
-0.44	-1.36	0.2665	-0.39	-1.31	0.5291
0.42	1.34	0.1829	0.73	1.65	0.1378
-0.57	-1.48	0.1430	0.29	1.22	0.6007
0.29	1.22	0.4596	-0.04	-1.03	0.942
-0.06	-1.04	0.8232	-0.61	-1.52	0.1508
-0.57	-1.48	0.1711	-0.22	-1.17	0.7211
-1.58	-2.99	0.0003	-0.64	-1.56	0.3397
-0.56	-1.48	0.1478	-0.65	-1.57	0.2873
0.32	1.25	0.4105	0.37	1.29	0.5387
0.90	1.87	0.0184	0.82	1.77	0.1514
-0.04	-1.03	0.9210	-0.62	-1.54	0.3027
0.09	1.06	0.8015	-0.19	-1.14	0.7333
0.07	1.05	0.8657	-0.95	-1.93	0.1233
-1.83	-3.54	0.0003	-1.41	-2.65	0.0488
-0.97	-1.96	0.0183	-0.94	-1.92	0.1317
-0.96	-1.94	0.0139	-0.97	-1.96	0.1025
0.32	1.25	0.4478	0.14	1.1	0.8298
0.07	1.05	0.8625	-0.44	-1.36	0.4421
-1.32	-2.49	0.0019	-0.24	-1.18	0.7072
-0.19	-1.14	0.6318	0	1	0.9999

-1.21	-2.32	0.0180	-0.28	-1.21	0.6983
0.23	1.18	0.5521	-0.4	-1.32	0.4807
-0.18	-1.13	0.6557	0.09	1.06	0.8845
-0.03	-1.02	0.9470	0.04	1.03	0.948
-0.45	-1.37	0.2648	-0.19	-1.14	0.7594
-0.36	-1.29	0.4010	-0.17	-1.12	0.7898
-0.84	-1.79	0.0715	0.09	1.07	0.8912
0.08	1.06	0.8294	-0.13	-1.1	0.8212
-0.19	-1.14	0.6393	0.07	1.05	0.9043
0.39	1.31	0.3682	0.21	1.16	0.7284
0.19	1.14	0.6855	0.43	1.35	0.5251
-0.60	-1.52	0.1038	-0.33	-1.26	0.556
-1.85	-3.61	0.0007	-0.39	-1.31	0.5912
0.24	1.18	0.5563	0.51	1.42	0.3951
2.97	7.82	0.0000	1.32	2.5	0.0468
-0.14	-1.10	0.7439	-0.14	-1.1	0.8138
0.02	1.02	0.9600	0.57	1.48	0.3885
0.06	1.05	0.8663	0.1	1.07	0.8719
-0.33	-1.25	0.4799	0.24	1.18	0.7256
-0.05	-1.04	0.8966	0.89	1.85	0.1755
-0.17	-1.13	0.6839	-0.09	-1.07	0.8786
-0.43	-1.34	0.3539	-1.33	-2.51	0.0381
-0.83	-1.78	0.0804	0.45	1.37	0.5139
-0.38	-1.31	0.5100	-0.54	-1.46	0.4808
0.33	1.26	0.4518	-0.86	-1.82	0.1615
0.34	1.27	0.4469	0.68	1.61	0.2825
-0.81	-1.75	0.2422	-1.72	-3.29	0.0262
0.91	1.88	0.2801	-1	-1.99	0.187
-0.87	-1.83	0.0487	0.78	1.71	0.2433
0.08	1.06	0.8444	0.27	1.21	0.6799
-1.62	-3.07	0.0012	0.23	1.17	0.7515
-0.70	-1.63	0.1565	-0.02	-1.02	0.9742
0.18	1.13	0.6980	-0.84	-1.8	0.193
0.09	1.06	0.8393	-0.45	-1.37	0.4914
-0.56	-1.48	0.1974	-0.44	-1.36	0.5076
0.52	1.43	0.2895	2.46	5.52	6.00E-04
-0.29	-1.22	0.5360	0.68	1.6	0.2951
-0.40	-1.32	0.3879	-0.12	-1.09	0.8556
-0.45	-1.36	0.4197	-0.92	-1.9	0.2089
0.24	1.18	0.5301	0	1	0.9943
-0.17	-1.13	0.7932	-1.08	-2.12	0.1622
-2.83	-7.13	0.0000	0.03	1.02	0.9652
1.31	2.48	0.0027	1.1	2.15	0.0683
-0.50	-1.41	0.3113	-0.53	-1.44	0.4552
0.00	-1.00	0.9917	0.28	1.22	0.6572
0.09	1.06	0.8450	-0.08	-1.06	0.8956
1.06	2.09	0.0202	0.12	1.09	0.851

0.46	1.38	0.3521	-1.05	-2.07	0.1204
0.28	1.22	0.3378	0.23	1.17	0.6134
-1.56	-2.94	0.0035	0.02	1.01	0.9799
-0.11	-1.08	0.8258	0.63	1.55	0.3747
-1.22	-2.32	0.0320	-0.29	-1.22	0.705
0.45	1.37	0.3880	-0.11	-1.08	0.8739
0.75	1.68	0.3201	-1.84	-3.59	0.0193
-0.18	-1.14	0.7113	0.5	1.41	0.4992
-0.88	-1.85	0.0752	-0.42	-1.34	0.5394
-0.94	-1.92	0.0556	0.65	1.57	0.3696
-0.71	-1.64	0.2180	-0.41	-1.33	0.5871
-0.65	-1.57	0.1756	-0.7	-1.63	0.3
-0.19	-1.14	0.7253	-0.65	-1.57	0.3422
-0.84	-1.79	0.1052	-0.84	-1.79	0.2319
-0.24	-1.18	0.6222	-1.07	-2.1	0.1181
-0.27	-1.21	0.5868	-0.99	-1.99	0.1396
-0.35	-1.27	0.4762	-0.56	-1.48	0.3968
0.18	1.13	0.7096	-0.42	-1.34	0.5159
0.34	1.26	0.4719	-0.01	-1	0.992
0.08	1.05	0.8722	-0.49	-1.41	0.4686
-0.18	-1.13	0.7167	-0.46	-1.38	0.4769
-0.22	-1.17	0.4448	0.05	1.04	0.9054
-0.60	-1.51	0.2518	0.42	1.34	0.5398
-0.65	-1.57	0.0703	-0.24	-1.18	0.6585
-0.37	-1.29	0.5001	1.15	2.21	0.1223
0.03	1.02	0.9599	-0.38	-1.3	0.5879
0.12	1.09	0.8037	0.43	1.34	0.5205
0.04	1.03	0.9437	0.62	1.54	0.3785
-0.42	-1.33	0.4053	-0.06	-1.04	0.9356
-0.50	-1.41	0.3411	-1.37	-2.58	0.0618
-0.29	-1.22	0.5835	0.5	1.42	0.5106
-0.03	-1.02	0.9430	0.27	1.21	0.7001
-0.47	-1.38	0.3811	-0.91	-1.88	0.217
-0.11	-1.08	0.8394	-0.52	-1.43	0.4732
-0.97	-1.95	0.0754	-0.51	-1.43	0.49
-0.15	-1.11	0.7706	-0.71	-1.63	0.2987
-0.76	-1.70	0.1347	-0.92	-1.9	0.1744
-0.16	-1.11	0.7920	-0.28	-1.22	0.71
-0.79	-1.73	0.2014	-1.05	-2.07	0.1659
-3.18	-9.07	0.0000	0.59	1.51	0.4524
-0.47	-1.38	0.3915	0.43	1.34	0.5616
-0.20	-1.15	0.7154	0.51	1.43	0.4998
-0.44	-1.35	0.3523	1	2	0.1491
2.07	4.21	0.0041	0.23	1.17	0.774
-0.84	-1.79	0.1147	0.08	1.06	0.9074
-0.61	-1.53	0.2579	-0.33	-1.26	0.6408
0.38	1.30	0.2535	-0.05	-1.04	0.9195

-0.07	-1.05	0.8803	0.14	1.1	0.841
0.06	1.05	0.8870	0.44	1.36	0.5205
-0.78	-1.72	0.1654	-0.16	-1.12	0.83
-0.43	-1.35	0.5227	0.45	1.37	0.5714
-0.46	-1.38	0.4084	-0.43	-1.35	0.5397
0.28	1.21	0.6221	1.33	2.51	0.0834
1.33	2.52	0.0406	-0.13	-1.1	0.8594
0.50	1.41	NA	0.16	1.12	NA
0.60	1.52	0.2450	-0.11	-1.08	0.8751
0.39	1.31	0.4742	-0.66	-1.58	0.3793
-0.45	-1.37	0.4674	-0.79	-1.72	0.2883
1.36	2.57	0.0686	-1.12	-2.18	0.1539
-0.57	-1.48	0.3345	-1.29	-2.45	0.0907
-0.25	-1.19	0.6966	0.29	1.22	0.7144
0.47	1.39	0.2875	0.83	1.77	0.193
-1.26	-2.40	0.0367	-1.1	-2.14	0.1432
-0.54	-1.45	0.2195	-0.24	-1.18	0.7044
-0.44	-1.36	0.4309	-0.48	-1.39	0.5323
1.78	3.44	0.0000	0.04	1.03	0.945
-0.67	-1.59	0.1995	0.25	1.19	0.73
-0.37	-1.29	0.5598	1.84	3.57	0.0195
-0.08	-1.06	0.8741	0.65	1.57	0.3653
0.29	1.22	0.5950	0.3	1.23	0.6865
-0.28	-1.21	0.6187	0.49	1.4	0.5269
-0.71	-1.64	0.2235	0.03	1.02	0.9679
-0.05	-1.03	0.9394	0.64	1.56	0.4038
-0.46	-1.38	0.3198	1.54	2.9	0.033
-0.70	-1.63	0.2126	0.76	1.7	0.324
-0.95	-1.94	0.1073	-0.13	-1.09	0.8695
-0.64	-1.56	0.3278	0.23	1.17	0.768
-1.22	-2.33	0.0768	-1.57	-2.96	0.0436
-0.63	-1.55	0.2912	-0.22	-1.16	0.7737
0.18	1.13	0.6254	0.18	1.14	0.7385
0.79	1.73	0.1206	-0.54	-1.46	0.425
-0.36	-1.28	0.5565	-1.23	-2.34	0.1066
-1.31	-2.48	0.0367	0.52	1.43	0.5112
-1.36	-2.57	0.0272	0.53	1.44	0.504
-0.68	-1.60	0.3122	-1.36	-2.56	0.0814
-0.68	-1.60	0.2235	-0.2	-1.15	0.7915
0.10	1.07	0.8378	3.17	9.01	0
-2.17	-4.49	0.0011	0.18	1.13	0.8197
-0.26	-1.20	0.5843	0.01	1.01	0.9869
-1.26	-2.39	0.0460	-0.14	-1.1	0.8579
1.19	2.28	0.1028	-0.22	-1.17	0.7811
0.42	1.34	NA	-0.24	-1.18	NA
-1.35	-2.56	0.0550	-0.09	-1.07	0.9062
-0.99	-1.98	0.1165	-0.72	-1.64	0.3528

-0.41	-1.32		0.5191	-0.77	-1.71	0.321
-2.13	-4.39		0.0025	1.03	2.04	0.1949
-0.26	-1.19		0.7152	-0.83	-1.77	0.2948
-0.93	-1.90		0.2172	-0.27	-1.21	0.7298
-0.84	-1.79		0.2176	-1.25	-2.37	0.1046
-1.36	-2.57		0.0383	0.25	1.19	0.7517
0.65	1.57		0.4321	-1.35	-2.56	0.0798
1.48	2.79		0.0534	-2.02	-4.07	0.0073
-3.66	-12.65		0.0000	0.11	1.08	0.8823
-1.36	-2.57		0.0563	-0.83	-1.78	0.2909
1.65	3.14		0.0179	2.24	4.73	0.0047
-2.52	-5.75		0.0005	-1.1	-2.15	0.1585
-3.20	-9.16		0.0001	0.12	1.09	0.8701
-0.49	-1.40		0.4569	-1.71	-3.28	0.0235
0.21	1.15		0.6467	-1.34	-2.53	0.0345
-1.73	-3.32		0.0144	-0.17	-1.12	0.8308
1.18	2.27		0.0359	-0.01	-1.01	0.9898
-0.15	-1.11	NA		-0.35	-1.28	NA
0.60	1.52		0.4677	-1.26	-2.4	0.1122
-1.76	-3.38		0.0358	0.11	1.08	0.8698
-0.05	-1.03		0.9174	0.33	1.26	0.6124
0.73	1.66		0.1100	1.11	2.16	0.0873
2.81	7.01		0.0001	-0.27	-1.2	0.7316
0.46	1.37		0.5498	-0.93	-1.9	0.2433
-1.47	-2.76		0.0672	-1.43	-2.69	0.0699
-2.07	-4.20		0.0102	-0.45	-1.36	0.5599
0.93	1.90		0.1753	0.13	1.1	0.8667
-0.19	-1.14		0.8190	-1.02	-2.02	0.1417
-0.35	-1.28		0.6685	1.32	2.5	0.0779
-1.20	-2.30		0.1514	1.5	2.84	0.0484
0.99	1.99		0.2373	1.12	2.17	0.1163
-1.82	-3.53		0.0285	0.08	1.06	0.8934
0.15	1.11		0.8326	0.68	1.6	0.3924
0.20	1.15	NA		0.04	1.03	NA
-0.92	-1.89		0.2697	-0.89	-1.85	0.2372
-1.14	-2.21		0.1347	-1.13	-2.19	0.1516
-0.95	-1.93		0.2565	-0.4	-1.32	0.545
0.09	1.07	NA		-0.09	-1.07	NA
1.13	2.19		0.1789	-0.55	-1.47	0.4726
-0.67	-1.59		0.4204	-1.39	-2.62	0.0697
-0.05	-1.03		0.9572	-0.28	-1.21	0.6902
0.66	1.58		0.4326	0.43	1.35	0.5193
1.04	2.06	NA		-0.43	-1.34	NA
0.91	1.88		0.2636	0.64	1.56	0.2558

ld Change, P value for each group versus NL.Tx								
IgA E1.vs.A	IgA E1.vs.A	IgA E1.vs.A	S.a.GN.vs.A	S.a.GN.vs.A	S.a.GN.vs.A	VancATN.v	VancATN.v	VancATN.v
0.22	1.17	0.2877	0.25	1.19	0.2038	-0.26	-1.2	0.2151
0.39	1.31	0.2274	0.42	1.34	0.1592	-0.41	-1.33	0.2055
0.14	1.11	0.4598	-0.37	-1.29	0.0481	-0.1	-1.07	0.621
0.13	1.1	0.6399	0.08	1.06	0.7659	-0.1	-1.07	0.7169
-0.01	-1	0.9725	-0.27	-1.2	0.1561	0.12	1.08	0.5471
0.28	1.21	0.2619	0.25	1.19	0.2684	-0.28	-1.21	0.2723
0.11	1.08	0.6321	-0.48	-1.39	0.0315	0.03	1.02	0.8937
-0.36	-1.28	0.2457	-0.1	-1.07	0.7381	-0.08	-1.05	0.8017
-0.14	-1.1	0.6837	-0.25	-1.19	0.4217	0	-1	0.9922
0.76	1.69	3.00E-04	0.68	1.6	7.00E-04	0.24	1.18	0.2717
-0.03	-1.02	0.861	-0.14	-1.1	0.4437	-0.19	-1.14	0.3211
0.86	1.81	0.1986	0.6	1.51	0.3516	0.51	1.42	0.4454
-0.1	-1.07	0.6357	-0.37	-1.29	0.0722	-0.2	-1.15	0.3607
0.18	1.13	0.578	0.08	1.06	0.7813	0.23	1.18	0.4502
0.39	1.31	0.1303	0.4	1.32	0.0974	-0.33	-1.25	0.2254
0.62	1.54	0.4328	0.64	1.56	0.4152	0.52	1.43	0.5139
0.55	1.46	0.42	0.81	1.76	0.2133	0.55	1.46	0.4175
0.4	1.32	0.1615	0.36	1.28	0.1756	0.61	1.52	0.0259
0	1	0.9972	-0.11	-1.08	0.6023	-0.12	-1.09	0.5988
0.12	1.08	0.6614	-0.2	-1.15	0.4304	-0.1	-1.07	0.7105
0.24	1.18	0.3178	0.2	1.15	0.3693	0.08	1.06	0.7349
0.56	1.48	0.4506	1.08	2.12	0.1331	1.12	2.17	0.1327
0.78	1.71	0.0154	0.85	1.8	0.0047	0.28	1.22	0.382
0.08	1.06	0.7744	0.25	1.19	0.3187	-0.19	-1.14	0.4966
0.5	1.41	0.2363	-0.51	-1.43	0.2105	0.43	1.34	0.3104
0.3	1.23	0.3791	0.24	1.18	0.4529	0.25	1.19	0.4619
-0.21	-1.15	0.466	0.01	1.01	0.9582	-0.38	-1.3	0.1805
0.78	1.71	0.1363	0.08	1.06	0.8684	0.99	1.98	0.0559
0.92	1.89	0.0128	0.97	1.95	0.0056	0.91	1.88	0.0127
-0.06	-1.05	0.8291	-0.11	-1.08	0.6782	-0.1	-1.08	0.7175
0.13	1.1	0.6468	-0.07	-1.05	0.7946	-0.42	-1.34	0.1703
0.14	1.1	0.6463	0.01	1.01	0.979	-0.31	-1.24	0.3402
-0.38	-1.3	0.1947	-0.28	-1.21	0.3027	-0.59	-1.51	0.0449
-0.26	-1.2	0.4537	-0.48	-1.39	0.1446	0.28	1.22	0.3739
-0.13	-1.1	0.6486	-0.95	-1.94	0.0012	-0.11	-1.08	0.705
0.21	1.16	0.6391	0.27	1.2	0.5292	0.09	1.06	0.8442
0.77	1.71	0.2931	0.8	1.74	0.2626	0.89	1.85	0.2263
0.46	1.37	0.2618	0.91	1.88	0.0153	-0.47	-1.38	0.273
0.35	1.27	0.3103	0.6	1.51	0.0605	0.03	1.02	0.9399
-0.36	-1.28	0.3217	-0.09	-1.07	0.7766	0.18	1.14	0.5754
-0.12	-1.09	0.8133	-0.92	-1.89	0.0692	0.01	1.01	0.9779

-0.05	-1.03	0.908	-0.17	-1.12	0.6598	-0.79	-1.72	0.0605
0.42	1.34	0.2107	-0.17	-1.13	0.6035	-0.2	-1.15	0.568
-0.13	-1.09	0.7022	-0.18	-1.13	0.5554	-0.12	-1.09	0.7029
-0.26	-1.19	0.53	-0.46	-1.38	0.2333	0.39	1.31	0.3069
-0.1	-1.07	0.7959	0	-1	0.9952	-0.02	-1.01	0.9662
-0.98	-1.97	0.034	-0.64	-1.55	0.1301	-0.91	-1.88	0.044
-0.27	-1.21	0.4908	0.33	1.26	0.3393	0.09	1.07	0.803
0.39	1.31	0.32	0.57	1.49	0.1158	-0.32	-1.24	0.4436
0.65	1.57	0.4168	0.69	1.61	0.3873	0.85	1.8	0.286
0.03	1.02	0.9499	0.21	1.15	0.6129	-0.63	-1.55	0.1645
-0.31	-1.24	0.4476	0.11	1.08	0.7646	0.03	1.02	0.9372
0.44	1.36	0.3514	-0.41	-1.33	0.3723	-0.66	-1.58	0.1796
-0.35	-1.28	0.4605	-1.14	-2.21	0.0161	0.2	1.15	0.6543
-0.25	-1.19	0.5321	-0.37	-1.3	0.3221	0.08	1.06	0.8325
0.47	1.38	0.2915	0.31	1.24	0.4636	0.75	1.68	0.0779
-0.57	-1.48	0.1663	-0.61	-1.53	0.1057	-0.46	-1.37	0.2441
-0.78	-1.71	0.0334	-0.4	-1.32	0.21	-0.37	-1.3	0.2672
-0.04	-1.03	0.9301	-0.24	-1.18	0.5751	0.58	1.5	0.1662
0.32	1.25	0.4863	0.73	1.66	0.0852	-0.33	-1.26	0.4921
0.54	1.45	0.482	0.85	1.8	0.2557	0.88	1.85	0.2469
-0.67	-1.59	0.093	-0.25	-1.19	0.4826	-0.2	-1.15	0.5797
-0.05	-1.04	0.8855	-0.15	-1.11	0.6673	0.17	1.13	0.6291
-0.46	-1.37	0.2358	-0.87	-1.83	0.0197	-0.36	-1.28	0.3375
-0.38	-1.31	0.3484	-0.1	-1.07	0.7964	-0.04	-1.03	0.9081
-1.58	-3	NA	-1.49	-2.81	NA	-0.8	-1.74	NA
-0.5	-1.41	0.2219	-0.44	-1.35	0.244	-0.71	-1.64	0.081
0.44	1.35	0.2757	0.5	1.41	0.1818	0.15	1.11	0.7174
-0.03	-1.02	0.9432	-0.12	-1.09	0.7799	0.57	1.49	0.1753
-0.13	-1.1	0.785	-0.22	-1.16	0.6357	-0.05	-1.03	0.9232
0.19	1.14	0.6476	0.27	1.2	0.4876	0.02	1.02	0.9564
-0.11	-1.08	0.7894	-0.12	-1.09	0.7493	0.31	1.24	0.4257
0.59	1.51	0.3614	0.52	1.43	0.4039	0.81	1.75	0.2129
0.06	1.04	0.8948	0.24	1.18	0.5633	0.39	1.31	0.3566
0.25	1.19	0.5984	0.31	1.24	0.4839	0.29	1.23	0.5234
0.02	1.02	0.9493	0.41	1.33	0.2311	-0.21	-1.15	0.5802
0.59	1.5	0.2875	0.88	1.84	0.0888	-1.15	-2.22	0.059
0.04	1.03	0.9185	0.52	1.44	0.1783	0.14	1.1	0.7402
0.02	1.02	0.9565	-0.16	-1.12	0.6813	-0.51	-1.42	0.2407
0.29	1.22	0.1841	0.34	1.26	0.0981	-0.09	-1.06	0.6946
0.16	1.12	0.7484	0.79	1.73	0.0739	0.26	1.2	0.5914
0.14	1.1	0.7611	0.2	1.15	0.651	0.48	1.4	0.2824
-1.01	-2.01	0.0588	-0.29	-1.22	0.5263	0.67	1.6	0.1305
0.01	1.01	0.9774	0.33	1.26	0.4354	0.16	1.12	0.7143
-0.03	-1.02	0.9461	-0.6	-1.51	0.1636	0	1	0.9995
-0.01	-1	0.9899	0.17	1.13	0.688	-0.1	-1.07	0.8312
0.37	1.29	0.4181	0.19	1.14	0.6624	-0.05	-1.03	0.9169
-0.2	-1.14	0.6634	-0.16	-1.11	0.7071	-0.29	-1.22	0.5174

0.01	1.01	0.982	0.19	1.14	0.6408	0.06	1.04	0.8877
-0.01	-1.01	0.9731	-0.59	-1.51	0.1205	-0.35	-1.27	0.3732
-0.88	-1.85	0.0527	-0.34	-1.27	0.3845	-0.06	-1.04	0.8754
0.31	1.24	0.5395	0.31	1.24	0.5113	0.74	1.67	0.1192
-0.02	-1.01	0.9717	0.11	1.08	0.7924	-0.38	-1.3	0.3907
-0.6	-1.51	0.2857	-0.05	-1.03	0.9267	-0.1	-1.07	0.8442
0.67	1.59	0.247	0.93	1.91	0.0885	0.27	1.21	0.6447
-0.2	-1.15	0.7008	0.29	1.22	0.5331	0.21	1.16	0.658
0.01	1.01	0.9633	-0.32	-1.25	0.2918	0.17	1.13	0.582
-0.15	-1.11	0.7696	0.18	1.14	0.6996	-0.14	-1.1	0.7904
1.01	2.01	0.1023	0.51	1.42	0.3953	0.84	1.79	0.1717
-0.11	-1.08	0.8125	0.05	1.04	0.8961	-0.43	-1.34	0.3427
0.03	1.02	0.9286	-0.34	-1.26	0.3409	-0.25	-1.19	0.4921
0	1	0.9989	-0.32	-1.25	0.4972	0.14	1.1	0.768
1.17	2.25	0.0952	0.43	1.35	0.5246	0.72	1.65	0.3069
0.18	1.14	0.7034	-0.22	-1.17	0.6363	-0.11	-1.08	0.8256
-0.01	-1.01	0.9813	0.47	1.39	0.3269	-0.21	-1.15	0.6984
-0.58	-1.49	0.3928	-0.16	-1.12	0.8041	0.35	1.28	0.5918
-0.18	-1.13	0.7084	-0.17	-1.12	0.7136	0.2	1.15	0.6548
0.97	1.96	NA	0.78	1.71	NA	1.01	2.01	NA
-0.03	-1.02	0.9445	-0.4	-1.32	0.4059	-0.45	-1.37	0.375
0.73	1.66	0.1786	1.21	2.32	0.0155	0.59	1.51	0.2719
-0.46	-1.38	0.2314	-0.2	-1.15	0.5705	-0.14	-1.1	0.699
-0.33	-1.25	0.4886	-0.69	-1.61	0.1312	-0.54	-1.45	0.2513
0.1	1.07	0.8423	0.51	1.43	0.2796	0	-1	0.9964
-0.14	-1.1	0.6269	0.13	1.09	0.6266	-0.24	-1.18	0.3891
0.81	1.76	0.1408	0.43	1.35	0.4232	0.74	1.67	0.177
0.28	1.21	0.6106	-0.06	-1.04	0.9111	0.36	1.28	0.5041
0.48	1.4	0.2915	0.89	1.85	0.0366	-0.6	-1.52	0.2139
-0.68	-1.6	0.2391	0.47	1.38	0.3331	0.01	1.01	0.9786
-0.01	-1	0.9899	-0.28	-1.22	0.58	0.01	1	0.9914
-0.26	-1.19	0.4914	-0.09	-1.06	0.7886	-0.38	-1.3	0.2986
-0.08	-1.06	0.8888	0.15	1.11	0.7874	0.26	1.2	0.644
-0.1	-1.07	0.8755	-0.34	-1.27	0.5552	0.12	1.09	0.8321
0.08	1.06	0.8823	-0.02	-1.01	0.9753	0.04	1.03	0.9357
0.35	1.27	0.535	1	2	0.0497	0.32	1.25	0.5573
0.64	1.56	0.2409	0.67	1.6	0.1925	0.01	1.01	0.9836
-0.25	-1.19	0.6532	-0.9	-1.86	0.0973	-0.12	-1.08	0.8256
0.26	1.2	0.5988	0.38	1.3	0.405	0	-1	0.9962
0.37	1.29	0.4681	-0.62	-1.53	0.2366	0.13	1.09	0.8047
-0.36	-1.29	0.5687	-1.9	-3.74	0.005	-0.02	-1.02	0.9703
-0.39	-1.31	0.476	-0.68	-1.6	0.203	0.26	1.2	0.6077
-0.48	-1.4	0.3614	-1.39	-2.63	0.0103	-0.27	-1.2	0.5963
0.44	1.36	0.445	0.52	1.43	0.3432	0.61	1.53	0.2802
-0.33	-1.26	0.5298	-0.56	-1.47	0.2706	-0.74	-1.67	0.1714
-0.47	-1.38	0.4418	-0.29	-1.22	0.6011	0.1	1.07	0.8631
-0.41	-1.33	0.4608	-0.35	-1.28	0.4992	-0.13	-1.09	0.8071

-0.34	-1.26	0.6216	-0.63	-1.54	0.3455	0.78	1.71	0.2231
-0.2	-1.15	0.7071	-0.71	-1.63	0.1682	-0.31	-1.24	0.5474
-0.02	-1.01	0.9743	-0.31	-1.24	0.5757	0.29	1.22	0.5926
0.77	1.71	0.195	0.04	1.03	0.9443	0.75	1.68	0.2027
0.01	1.01	0.9842	-0.68	-1.6	0.233	-0.39	-1.31	0.5026
-0.14	-1.1	0.8179	-0.03	-1.02	0.9593	0.32	1.25	0.573
0.07	1.05	0.9164	0.88	1.84	0.1351	0.4	1.32	0.5215
0.33	1.26	0.5294	-0.5	-1.41	0.3535	-0.12	-1.08	0.8308
-0.34	-1.27	0.5492	0.04	1.03	0.9426	0.22	1.16	0.681
0.09	1.07	0.8758	0.41	1.33	0.4518	0.32	1.25	0.5722
0.69	1.61	0.2816	0.87	1.82	0.1523	0.13	1.09	0.8456
-0.25	-1.19	0.6385	-0.2	-1.15	0.6852	0.05	1.03	0.9287
-0.77	-1.71	0.281	-1.35	-2.55	0.0564	0.08	1.06	0.9023
0.23	1.17	0.6932	0.72	1.64	0.1723	-0.07	-1.05	0.9002
0.54	1.45	0.4079	-0.06	-1.04	0.9214	0.07	1.05	0.9095
-0.4	-1.32	0.4834	-0.23	-1.17	0.6648	-0.51	-1.43	0.3639
0.51	1.43	0.4169	0.25	1.19	0.6844	0.53	1.44	0.3954
0.37	1.29	0.5007	0.21	1.16	0.6802	-0.3	-1.23	0.599
0.25	1.19	0.6989	0.93	1.91	0.1167	0.19	1.14	0.7728
1.05	2.08	0.0892	1.28	2.42	0.0289	0.93	1.9	0.1328
-0.12	-1.08	0.8407	0.01	1.01	0.9817	0.01	1	0.9928
-0.75	-1.68	0.1898	-0.53	-1.44	0.3098	-0.46	-1.38	0.3923
0.07	1.05	0.9233	0.6	1.52	0.3369	0.59	1.5	0.3676
0.75	1.68	0.2956	0.83	1.78	0.2273	-0.99	-1.99	0.1922
-0.41	-1.32	0.4651	-1.11	-2.16	0.0445	-0.55	-1.47	0.3167
0.18	1.13	0.775	0.54	1.45	0.3556	1.47	2.78	0.0093
-0.97	-1.95	0.2001	0.26	1.2	0.71	0.26	1.2	0.7195
-0.08	-1.06	0.9171	0.1	1.07	0.8996	-0.04	-1.03	0.9636
0.08	1.06	0.9059	0.3	1.23	0.6371	0.97	1.96	0.1175
0.74	1.67	0.2128	0.61	1.53	0.2835	-0.01	-1.01	0.9833
0.1	1.07	0.8931	0.16	1.12	0.8104	0.42	1.34	0.5414
1.3	2.47	0.0571	1.64	3.12	0.0115	1.08	2.11	0.1155
-0.33	-1.26	0.5764	-1.24	-2.36	0.0378	-0.2	-1.15	0.7271
0.21	1.16	0.7145	0.29	1.23	0.5928	0.03	1.02	0.9582
0.16	1.12	0.7863	-0.07	-1.05	0.9013	0.5	1.41	0.3855
1.49	2.8	0.0375	1.49	2.81	0.0308	0.21	1.15	0.7829
-0.24	-1.18	0.7154	-0.06	-1.04	0.9292	0.43	1.35	0.4863
-0.06	-1.04	0.9249	-0.29	-1.22	0.639	0.74	1.67	0.2098
-0.22	-1.16	0.7513	-1.45	-2.73	0.039	0.16	1.12	0.8055
-0.12	-1.08	0.8299	-0.18	-1.13	0.7251	-0.4	-1.32	0.456
-1.11	-2.16	0.145	-1.19	-2.28	0.1084	-1.42	-2.68	0.0621
-0.87	-1.83	0.2672	-1.55	-2.92	0.0497	0.17	1.13	0.8211
0.57	1.49	0.3359	0.39	1.31	0.4985	0.54	1.45	0.3606
0.06	1.05	0.9211	0.59	1.51	0.3133	0.47	1.38	0.4463
0.26	1.2	0.6686	0.09	1.06	0.8805	0.39	1.31	0.5064
-0.2	-1.15	0.734	-0.37	-1.29	0.516	-0.28	-1.22	0.6321
0.97	1.96	0.1014	0	1	0.9967	0.76	1.69	0.2026

-0.2	-1.15	0.7438	-0.54	-1.46	0.3488	-0.26	-1.2	0.6601
0.06	1.04	0.8882	-0.22	-1.17	0.59	-0.29	-1.22	0.5037
-0.08	-1.06	0.9095	0.56	1.47	0.4023	1.05	2.07	0.1134
0.57	1.48	0.4087	0.55	1.47	0.3993	-0.04	-1.03	0.9502
0.45	1.36	0.541	0.31	1.24	0.6629	-0.06	-1.05	0.931
-0.79	-1.73	0.2273	-0.69	-1.61	0.2661	-0.74	-1.68	0.2487
-1.22	-2.33	0.1188	-1.3	-2.47	0.0894	0.36	1.28	0.6424
0.88	1.83	0.2083	1.18	2.26	0.072	1.18	2.27	0.0785
-0.41	-1.32	0.5383	0.38	1.3	0.5219	0.22	1.17	0.7199
0.42	1.34	0.5535	0.16	1.12	0.8159	0.78	1.72	0.2562
-0.26	-1.19	0.723	-1.12	-2.17	0.122	0.53	1.44	0.446
-0.38	-1.3	0.5473	-0.35	-1.28	0.5493	-0.07	-1.05	0.9123
-1.01	-2.01	0.1332	-0.77	-1.71	0.2175	0.13	1.09	0.8365
-0.57	-1.48	0.3869	-0.32	-1.25	0.6002	0.07	1.05	0.909
-0.33	-1.26	0.5876	-0.58	-1.49	0.3229	0.18	1.13	0.7587
-0.79	-1.73	0.2055	-0.53	-1.45	0.353	-0.45	-1.37	0.4449
-0.63	-1.55	0.3174	0.12	1.09	0.828	0.34	1.27	0.5556
-0.47	-1.39	0.4454	-0.8	-1.74	0.1862	0.28	1.21	0.6303
-0.25	-1.19	0.6842	-0.03	-1.02	0.9563	0.19	1.14	0.7495
0.2	1.15	0.7451	-0.48	-1.39	0.4313	0.01	1.01	0.9839
-0.66	-1.58	0.2965	-0.07	-1.05	0.8964	-0.43	-1.35	0.4736
-0.11	-1.08	0.7952	0.16	1.12	0.6858	-0.08	-1.06	0.846
-0.74	-1.67	0.3035	0.02	1.02	0.9701	0.51	1.43	0.4264
-0.2	-1.15	0.6986	-0.37	-1.29	0.4522	-0.08	-1.06	0.8731
0.34	1.26	0.6508	1.37	2.59	0.0443	0.73	1.66	0.3104
-0.25	-1.19	0.7059	-0.14	-1.1	0.8179	0.26	1.2	0.6787
-0.26	-1.2	0.6935	0.28	1.21	0.6503	-0.28	-1.21	0.6752
0.26	1.2	0.7111	0.74	1.67	0.254	0.39	1.31	0.5664
-0.25	-1.19	0.7099	-0.02	-1.01	0.9794	-0.04	-1.03	0.9501
0.02	1.02	0.9732	-0.71	-1.64	0.2716	0.1	1.07	0.8764
1.08	2.11	0.1357	0.68	1.61	0.3337	1.02	2.03	0.1524
0.78	1.72	0.2324	0.52	1.44	0.4094	0.36	1.29	0.5831
0.2	1.15	0.7601	-0.48	-1.4	0.466	0.21	1.16	0.7481
-0.14	-1.11	0.8317	-0.13	-1.09	0.8408	0.3	1.24	0.6425
-0.08	-1.06	0.907	-0.36	-1.28	0.6003	0.56	1.47	0.4064
-0.35	-1.27	0.5804	-0.35	-1.28	0.5523	-0.05	-1.03	0.9375
-0.91	-1.87	0.1612	-0.55	-1.46	0.3515	-0.75	-1.69	0.2282
0	1	0.9981	-0.27	-1.2	0.7094	0.25	1.19	0.7243
-0.76	-1.69	0.2995	-0.41	-1.33	0.5517	0.22	1.16	0.7532
-0.05	-1.03	0.9508	0.07	1.05	0.9263	2.27	4.82	0.0032
-0.09	-1.07	0.8973	-0.04	-1.03	0.9491	0.45	1.37	0.5174
0.7	1.63	0.3371	1.42	2.67	0.0379	1.05	2.07	0.1406
0.31	1.24	0.6585	0.86	1.81	0.1845	0.14	1.1	0.8398
0.31	1.24	0.6944	0.66	1.57	0.3842	1.35	2.54	0.0771
-0.9	-1.87	0.2029	0.05	1.04	0.9331	0.1	1.07	0.8805
-0.67	-1.59	0.3339	-0.26	-1.2	0.6808	0.13	1.1	0.8349
-0.1	-1.07	0.8241	-0.28	-1.21	0.5214	-0.3	-1.23	0.5152

-0.07	-1.05	0.9216	0.46	1.38	0.4479	0.17	1.12	0.7962
0.66	1.58	0.304	-0.66	-1.58	0.3233	0.35	1.27	0.59
-0.45	-1.37	0.5297	-0.06	-1.05	0.9239	0.03	1.02	0.9639
0.7	1.62	0.3779	-0.16	-1.11	0.8432	1.19	2.29	0.1258
-1.18	-2.26	0.0942	-1.04	-2.06	0.1164	-0.8	-1.74	0.2364
1.01	2.01	0.1808	2.04	4.11	0.0038	-0.84	-1.79	0.2904
-0.1	-1.07	0.8958	0.16	1.12	0.8116	0.98	1.98	0.1527
0.18	1.14	NA	0.19	1.14	NA	0.34	1.27	NA
0.18	1.13	0.7834	0.45	1.37	0.4591	-0.42	-1.34	0.53
0.84	1.79	0.2049	-0.49	-1.41	0.4759	0.45	1.37	0.499
-1.11	-2.16	0.1283	-0.88	-1.84	0.2009	0.25	1.19	0.7092
-0.23	-1.17	0.7643	0.22	1.17	0.7663	0.71	1.64	0.3496
0.24	1.18	0.7312	0.37	1.29	0.575	0.51	1.42	0.4553
-0.04	-1.03	0.9573	0.46	1.38	0.5345	0.84	1.79	0.2635
0.47	1.38	0.439	0.71	1.63	0.2161	-0.51	-1.43	0.4062
-0.91	-1.88	0.2078	-0.93	-1.9	0.1844	-0.37	-1.29	0.5952
-0.49	-1.4	0.4187	0.03	1.02	0.9583	0.09	1.06	0.8733
0.45	1.36	0.5281	0.02	1.01	0.9818	0.11	1.08	0.8758
0.57	1.48	0.3029	-0.63	-1.55	0.2414	0.55	1.47	0.3131
-0.1	-1.07	0.8906	-0.7	-1.63	0.3215	0.65	1.57	0.3266
0.55	1.46	0.4914	0.65	1.57	0.4015	-0.17	-1.12	0.8353
0.3	1.23	0.6721	0.66	1.58	0.3227	0.43	1.35	0.5372
0.46	1.37	0.519	-0.27	-1.2	0.7063	0.69	1.62	0.3168
0.59	1.5	0.4293	-0.02	-1.01	0.9789	0.74	1.67	0.3106
-0.05	-1.03	0.9481	-0.46	-1.38	0.5277	1.4	2.64	0.0415
0.32	1.25	0.6727	0.78	1.72	0.2759	0.24	1.18	0.7483
0.83	1.78	0.2517	0.7	1.63	0.3177	1.24	2.36	0.0754
0.65	1.57	0.3914	0.44	1.35	0.5556	1.11	2.15	0.132
0.83	1.78	0.2683	0.89	1.85	0.2217	0.98	1.98	0.1842
-0.08	-1.05	0.9232	0.69	1.62	0.3506	0.55	1.47	0.4694
-0.61	-1.53	0.4235	-1.44	-2.71	0.0608	0.19	1.14	0.7947
-0.54	-1.45	0.4667	-0.16	-1.12	0.8172	0.21	1.16	0.7641
0.02	1.02	0.9649	-0.17	-1.12	0.7323	-0.41	-1.33	0.4358
0.07	1.05	0.9036	-0.11	-1.08	0.8463	-0.74	-1.67	0.2507
-0.21	-1.16	0.7667	-0.6	-1.51	0.3933	0.33	1.26	0.6335
0.21	1.15	0.794	0.09	1.06	0.9074	1.11	2.15	0.1443
0.23	1.18	0.7662	-1.09	-2.12	0.1725	1.2	2.29	0.1121
-0.48	-1.4	0.5247	-1.97	-3.91	0.0108	-0.15	-1.11	0.8381
0.11	1.08	0.8822	-0.9	-1.87	0.2191	0.64	1.55	0.3585
2.47	5.56	7.00E-04	3.31	9.93	0	1.63	3.09	0.027
0.32	1.25	0.6872	-0.02	-1.02	0.9777	1.17	2.25	0.1297
0.01	1.01	0.9864	-0.79	-1.72	0.2261	0.93	1.9	0.1258
0.22	1.17	0.7741	1.37	2.58	0.0545	1.05	2.07	0.1536
0.3	1.23	0.7075	-1.01	-2.01	0.2022	0.24	1.18	0.7601
0.18	1.14	NA	0.76	1.7	NA	0.72	1.65	NA
-0.36	-1.28	0.653	-0.96	-1.95	0.2263	1.02	2.03	0.1873
-0.48	-1.4	0.5193	-0.9	-1.87	0.2245	0.14	1.1	0.8509

0.36	1.29	0.6217	-0.01	-1.01	0.9887	0.49	1.4	0.5035
-0.05	-1.04	0.9491	0.74	1.67	0.3469	1.84	3.59	0.0164
-0.03	-1.02	0.97	-0.49	-1.4	0.5276	0.18	1.14	0.8104
-0.56	-1.48	0.481	0.18	1.13	0.8173	0.14	1.1	0.8624
-1.51	-2.84	0.0484	-2.53	-5.77	0.001	0.02	1.02	0.9749
-0.34	-1.27	0.6636	-0.97	-1.96	0.2187	0.08	1.06	0.9141
-0.36	-1.29	0.6472	-1.53	-2.89	0.0547	0.61	1.52	0.4467
-0.96	-1.95	0.1823	-0.14	-1.1	0.8296	0.5	1.42	0.4418
-0.04	-1.03	0.9581	0.24	1.18	0.7578	0.39	1.31	0.6126
-0.76	-1.69	0.3366	-0.57	-1.48	0.4642	-0.04	-1.03	0.9549
1.39	2.62	0.0783	1.84	3.59	0.0164	2.32	4.98	0.0029
-1.75	-3.37	0.0247	-0.79	-1.73	0.2979	-0.84	-1.79	0.274
-0.04	-1.03	0.9601	-0.25	-1.19	0.7447	1.46	2.75	0.0595
-1.13	-2.19	0.119	-1.73	-3.33	0.0165	-0.46	-1.37	0.5112
0.06	1.04	0.9168	-1.14	-2.2	0.0366	-0.4	-1.32	0.4603
-0.07	-1.05	0.9321	-0.7	-1.62	0.3803	0.78	1.71	0.3206
-0.35	-1.28	0.607	0.02	1.02	0.9723	-1.53	-2.89	0.0328
-0.46	-1.38	NA	0.2	1.15	NA	0.31	1.24	NA
0.55	1.46	0.4897	0.41	1.33	0.6032	-0.17	-1.12	0.8307
0.55	1.47	0.4312	-0.18	-1.13	0.7967	0.24	1.18	0.7274
0.12	1.09	0.8492	0.61	1.52	0.3035	0.55	1.46	0.3726
0.75	1.68	0.2268	1.08	2.11	0.0666	-0.08	-1.06	0.8965
-0.1	-1.07	0.8966	-0.51	-1.42	0.4963	-0.34	-1.27	0.654
0.04	1.02	0.9644	-0.89	-1.85	0.2608	-0.8	-1.74	0.3153
-0.97	-1.96	0.2238	-0.2	-1.15	0.8017	-0.54	-1.46	0.4951
-0.18	-1.14	0.8149	-0.65	-1.57	0.4096	1.19	2.28	0.1351
-0.03	-1.02	0.967	-1.06	-2.09	0.172	0.11	1.08	0.8921
-0.63	-1.54	0.3881	-0.88	-1.84	0.2378	-0.51	-1.43	0.4823
0.68	1.6	0.3769	1.53	2.89	0.0505	-0.03	-1.02	0.9701
-0.02	-1.01	0.9778	-0.29	-1.23	0.708	1.6	3.03	0.0408
0.65	1.57	0.3836	1.14	2.21	0.1333	0.88	1.85	0.2344
0.36	1.28	0.5858	-0.15	-1.11	0.8209	-0.05	-1.04	0.9374
1.39	2.61	0.0811	1	2	0.2041	0.96	1.95	0.2254
0.2	1.14	NA	0.2	1.15	NA	0.33	1.26	NA
-0.37	-1.29	0.6402	-1.34	-2.53	0.0877	0.49	1.41	0.5319
-2.46	-5.5	0.0018	-2.78	-6.87	4.00E-04	-0.17	-1.13	0.8247
-0.28	-1.22	0.6825	-0.12	-1.09	0.8637	0.02	1.01	0.9764
0.12	1.08	NA	0.34	1.27	NA	0.68	1.6	NA
-1.36	-2.57	0.0791	-0.32	-1.25	0.6834	0.86	1.82	0.279
-0.91	-1.88	0.2467	-1.08	-2.11	0.1751	-0.31	-1.24	0.6924
0.19	1.14	0.7952	0.89	1.85	0.2384	0.01	1	0.9931
-0.19	-1.14	0.7831	0.39	1.31	0.59	-0.53	-1.45	0.4433
0.62	1.54	NA	-0.81	-1.75	NA	1.26	2.39	NA
0.91	1.87	0.1332	-0.11	-1.08	0.8658	-0.01	-1.01	0.9856

IgA E1.vs.I	IgA_HSP_A	IgA_HSP_A	S.a.GN.vs.I	S.a.GN.vs.I	S.a.GN.vs.I	VancATN.v	VancATN.v	VancATN.v
0	-1	0.9997	0.03	1.02	0.9053	-0.48	-1.4	0.0346
-0.09	-1.06	0.7955	-0.05	-1.04	0.8702	-0.88	-1.85	0.0116
0.24	1.18	0.26	-0.27	-1.21	0.1876	0	1	0.9971
-0.09	-1.06	0.777	-0.14	-1.1	0.6286	-0.32	-1.25	0.2937
0.06	1.04	0.7839	-0.2	-1.15	0.331	0.18	1.14	0.3868
0.22	1.17	0.4017	0.2	1.15	0.4228	-0.33	-1.26	0.2213
0.2	1.15	0.4259	-0.39	-1.31	0.1122	0.12	1.09	0.6282
-0.27	-1.21	0.4203	-0.01	-1.01	0.9789	0.01	1.01	0.9733
0.48	1.4	0.1985	0.37	1.29	0.305	0.62	1.53	0.0975
0.26	1.19	0.2267	0.17	1.13	0.3879	-0.27	-1.2	0.2216
0.17	1.13	0.4231	0.07	1.05	0.74	0.02	1.01	0.9427
0.81	1.75	0.2492	0.55	1.46	0.4194	0.46	1.38	0.5122
-0.05	-1.04	0.8278	-0.32	-1.25	0.1578	-0.14	-1.11	0.5361
0.09	1.06	0.7947	0	-1	0.9887	0.15	1.11	0.6605
-0.13	-1.09	0.6342	-0.12	-1.09	0.638	-0.85	-1.8	0.0024
0.79	1.73	0.3208	0.81	1.75	0.3063	0.68	1.61	0.389
0.07	1.05	0.9221	0.33	1.26	0.6272	0.07	1.05	0.9193
0.21	1.16	0.481	0.17	1.13	0.5389	0.42	1.34	0.1441
-0.24	-1.18	0.3101	-0.36	-1.28	0.114	-0.37	-1.29	0.1248
0.05	1.03	0.8621	-0.26	-1.2	0.3308	-0.16	-1.12	0.5622
0.02	1.02	0.9299	-0.02	-1.01	0.9427	-0.14	-1.1	0.5793
0.07	1.05	0.9223	0.6	1.51	0.4231	0.63	1.55	0.4096
0.07	1.05	0.8273	0.14	1.11	0.6399	-0.42	-1.34	0.2061
0.09	1.06	0.7656	0.26	1.2	0.3422	-0.18	-1.13	0.5469
0.56	1.48	0.2196	-0.45	-1.37	0.3149	0.49	1.4	0.2842
-0.31	-1.24	0.3673	-0.37	-1.29	0.2529	-0.36	-1.29	0.2849
-0.03	-1.02	0.9153	0.19	1.14	0.5112	-0.2	-1.15	0.5098
0.09	1.07	0.8636	-0.6	-1.51	0.259	0.31	1.24	0.5746
-0.24	-1.18	0.5177	-0.19	-1.14	0.5774	-0.25	-1.19	0.4987
-0.13	-1.09	0.6861	-0.18	-1.13	0.5477	-0.17	-1.12	0.586
0.08	1.06	0.8007	-0.13	-1.09	0.668	-0.47	-1.39	0.1429
-0.07	-1.05	0.8279	-0.21	-1.15	0.5034	-0.52	-1.43	0.1192
0.1	1.07	0.7617	0.21	1.16	0.5054	-0.11	-1.08	0.7513
-0.14	-1.1	0.7077	-0.36	-1.28	0.3155	0.4	1.32	0.2489
0.34	1.27	0.3034	-0.48	-1.39	0.1534	0.37	1.29	0.2581
-0.02	-1.02	0.9616	0.03	1.02	0.9444	-0.15	-1.11	0.7571
0.67	1.59	0.3757	0.7	1.62	0.345	0.79	1.73	0.2987
-0.27	-1.21	0.5149	0.18	1.14	0.6372	-1.2	-2.3	0.0061
-0.27	-1.21	0.4364	-0.02	-1.02	0.9396	-0.59	-1.51	0.0894
-0.45	-1.36	0.2367	-0.18	-1.13	0.5958	0.09	1.07	0.7867
0.26	1.2	0.6445	-0.53	-1.45	0.334	0.4	1.32	0.4779

-0.02	-1.02	0.9575	-0.14	-1.1	0.7282	-0.76	-1.7	0.0892
0.79	1.73	0.0384	0.19	1.14	0.604	0.17	1.12	0.6707
0.22	1.17	0.5483	0.17	1.12	0.6325	0.23	1.17	0.5328
0.41	1.33	0.3749	0.21	1.16	0.6385	1.06	2.08	0.0168
-0.08	-1.06	0.8428	0.02	1.01	0.9668	0	1	0.9954
0.01	1	0.9903	0.35	1.27	0.4737	0.07	1.05	0.884
-0.59	-1.51	0.1426	0.01	1.01	0.977	-0.23	-1.17	0.5474
-0.33	-1.26	0.3897	-0.15	-1.11	0.6691	-1.04	-2.06	0.0107
0.62	1.53	0.4349	0.66	1.58	0.4055	0.82	1.77	0.2993
0.2	1.15	0.6744	0.38	1.3	0.3979	-0.46	-1.38	0.3495
0.18	1.13	0.6887	0.6	1.51	0.1525	0.52	1.43	0.2325
0.31	1.24	0.5394	-0.54	-1.46	0.2728	-0.79	-1.73	0.1301
-0.02	-1.02	0.9651	-0.81	-1.76	0.1164	0.53	1.44	0.2855
0.52	1.43	0.269	0.39	1.31	0.3774	0.85	1.8	0.0595
0.29	1.23	0.5221	0.14	1.1	0.7558	0.58	1.49	0.1943
0.31	1.24	0.5176	0.26	1.2	0.5616	0.42	1.34	0.3692
-0.16	-1.12	0.6992	0.21	1.16	0.5668	0.24	1.18	0.5344
0.23	1.17	0.6443	0.03	1.02	0.9541	0.85	1.81	0.0684
-0.35	-1.28	0.4473	0.06	1.04	0.8953	-1.01	-2.01	0.0366
0.26	1.2	0.7414	0.57	1.48	0.4573	0.6	1.52	0.4381
0.35	1.28	0.4633	0.78	1.71	0.0774	0.82	1.76	0.0704
0.58	1.49	0.1824	0.48	1.4	0.2449	0.8	1.75	0.0552
0.25	1.19	0.5811	-0.17	-1.12	0.7033	0.35	1.27	0.423
0.36	1.29	0.4467	0.65	1.57	0.1402	0.7	1.63	0.1231
0.1	1.07	NA	0.19	1.14	NA	0.88	1.84	NA
-0.49	-1.4	0.2637	-0.42	-1.34	0.2943	-0.7	-1.62	0.1073
0.24	1.18	0.5703	0.3	1.23	0.4448	-0.05	-1.04	0.8967
-0.22	-1.16	0.6454	-0.31	-1.24	0.4958	0.39	1.31	0.3819
0.72	1.64	0.197	0.63	1.55	0.235	0.8	1.75	0.1421
0.11	1.08	0.8073	0.18	1.14	0.6527	-0.06	-1.04	0.8893
0.55	1.46	0.2553	0.54	1.45	0.2418	0.97	1.95	0.035
0.18	1.14	0.7866	0.11	1.08	0.866	0.4	1.32	0.5585
0.35	1.27	0.4771	0.52	1.44	0.2512	0.68	1.6	0.1485
0.18	1.13	0.7159	0.24	1.18	0.6071	0.23	1.17	0.642
-0.34	-1.26	0.3836	0.05	1.03	0.897	-0.57	-1.48	0.1417
-0.11	-1.08	0.849	0.19	1.14	0.7244	-1.85	-3.6	0.0028
-0.02	-1.01	0.9705	0.46	1.38	0.2659	0.08	1.06	0.8608
0.1	1.07	0.8329	-0.09	-1.07	0.8324	-0.44	-1.36	0.3461
-0.16	-1.12	0.4767	-0.11	-1.08	0.5843	-0.54	-1.45	0.0181
-0.13	-1.1	0.7909	0.5	1.41	0.2751	-0.04	-1.02	0.9424
0.01	1.01	0.9822	0.07	1.05	0.886	0.35	1.27	0.4584
-0.73	-1.66	0.1994	-0.02	-1.01	0.9746	0.95	1.93	0.0546
0.11	1.08	0.8247	0.43	1.34	0.3537	0.26	1.2	0.5888
-0.44	-1.35	0.3223	-1.01	-2.01	0.0211	-0.41	-1.33	0.344
0.05	1.03	0.9243	0.22	1.17	0.6268	-0.04	-1.03	0.9272
-0.1	-1.07	0.837	-0.28	-1.21	0.5332	-0.51	-1.43	0.2755
-0.08	-1.06	0.8663	-0.04	-1.03	0.9265	-0.17	-1.13	0.7178

0.13	1.1	0.7823	0.31	1.24	0.4829	0.18	1.14	0.6948
0.25	1.19	0.5602	-0.33	-1.26	0.4323	-0.09	-1.06	0.8395
-0.42	-1.34	0.4018	0.12	1.09	0.7871	0.4	1.32	0.3796
0.22	1.17	0.6712	0.22	1.17	0.6512	0.66	1.57	0.1911
-0.08	-1.06	0.862	0.04	1.03	0.923	-0.45	-1.36	0.3399
-0.68	-1.6	0.2465	-0.13	-1.09	0.8093	-0.19	-1.14	0.7381
0.59	1.5	0.3364	0.85	1.8	0.1445	0.18	1.14	0.7637
-1.22	-2.32	0.0133	-0.73	-1.66	0.0907	-0.81	-1.75	0.0785
0.38	1.3	0.2929	0.04	1.03	0.9073	0.53	1.45	0.1276
-0.21	-1.16	0.7041	0.13	1.09	0.8006	-0.19	-1.14	0.7223
-0.12	-1.09	0.8505	-0.62	-1.54	0.3094	-0.28	-1.22	0.6505
0.03	1.02	0.9564	0.19	1.14	0.6783	-0.3	-1.23	0.5422
-0.06	-1.04	0.8827	-0.43	-1.34	0.2541	-0.34	-1.27	0.3768
0.49	1.41	0.3652	0.17	1.13	0.7414	0.63	1.55	0.2342
0.33	1.26	0.6489	-0.41	-1.33	0.5616	-0.12	-1.09	0.8637
-0.13	-1.1	0.7951	-0.54	-1.45	0.2716	-0.42	-1.34	0.4036
-0.14	-1.1	0.8037	0.35	1.27	0.4936	-0.33	-1.26	0.549
-0.24	-1.18	0.7306	0.17	1.13	0.7969	0.68	1.61	0.3238
0.31	1.24	0.5675	0.33	1.25	0.5255	0.7	1.62	0.1789
0.56	1.48	NA	0.37	1.29	NA	0.6	1.52	NA
0.37	1.29	0.5037	0	1	0.9969	-0.05	-1.03	0.9314
-0.35	-1.27	0.4937	0.13	1.09	0.7783	-0.49	-1.4	0.3349
-0.51	-1.42	0.2106	-0.24	-1.18	0.509	-0.19	-1.14	0.6251
0.41	1.33	0.443	0.06	1.04	0.9163	0.2	1.15	0.7103
-0.35	-1.28	0.5025	0.06	1.04	0.9076	-0.46	-1.37	0.3782
-0.01	-1.01	0.9659	0.25	1.19	0.3789	-0.12	-1.09	0.7036
-0.17	-1.13	0.7436	-0.56	-1.47	0.2773	-0.25	-1.19	0.6324
0.67	1.59	0.266	0.33	1.26	0.5734	0.75	1.68	0.2053
-0.24	-1.18	0.6104	0.17	1.12	0.7053	-1.33	-2.51	0.0074
-0.97	-1.96	0.0986	0.18	1.13	0.7205	-0.28	-1.21	0.6059
0.03	1.02	0.9509	-0.24	-1.18	0.6582	0.05	1.03	0.9324
0.35	1.28	0.4098	0.52	1.43	0.195	0.23	1.17	0.5919
0.14	1.1	0.8201	0.37	1.29	0.5288	0.48	1.39	0.4242
0.54	1.46	0.4151	0.3	1.23	0.6494	0.76	1.7	0.24
0.73	1.66	0.2258	0.64	1.55	0.2726	0.69	1.62	0.2439
-0.02	-1.01	0.976	0.64	1.55	0.2244	-0.04	-1.03	0.9419
-0.18	-1.13	0.738	-0.15	-1.11	0.7704	-0.81	-1.75	0.1433
0.37	1.3	0.5366	-0.28	-1.21	0.6459	0.5	1.42	0.3969
0.45	1.36	0.4018	0.57	1.48	0.2572	0.19	1.14	0.7284
1.32	2.49	0.0275	0.33	1.26	0.582	1.08	2.11	0.0721
1.04	2.06	0.1482	-0.5	-1.41	0.5065	1.38	2.61	0.0493
0.54	1.46	0.3905	0.26	1.2	0.6702	1.2	2.3	0.0454
0.49	1.41	0.4205	-0.42	-1.34	0.4994	0.71	1.63	0.2323
0.31	1.24	0.6093	0.38	1.31	0.5026	0.48	1.39	0.4196
0.11	1.08	0.8538	-0.12	-1.08	0.8358	-0.3	-1.23	0.6141
-0.23	-1.17	0.7208	-0.06	-1.04	0.925	0.33	1.26	0.5832
-0.41	-1.33	0.4814	-0.35	-1.28	0.5227	-0.13	-1.09	0.8167

-0.06	-1.04	0.9313	-0.35	-1.27	0.6172	1.05	2.08	0.1203
0.21	1.15	0.7199	-0.3	-1.23	0.5903	0.09	1.06	0.8739
-0.11	-1.08	0.859	-0.4	-1.32	0.4922	0.21	1.15	0.7199
0.73	1.66	0.2423	0	-1	0.998	0.71	1.63	0.2518
0.2	1.15	0.7428	-0.49	-1.41	0.4197	-0.2	-1.15	0.7463
0.03	1.02	0.9605	0.14	1.1	0.8146	0.49	1.4	0.4209
-0.02	-1.02	0.9707	0.79	1.72	0.1992	0.31	1.24	0.6341
0.46	1.38	0.4113	-0.36	-1.29	0.5247	0.02	1.01	0.9757
-0.41	-1.33	0.4875	-0.03	-1.02	0.9516	0.15	1.11	0.7902
-0.12	-1.09	0.8403	0.2	1.15	0.7306	0.11	1.08	0.8557
0.26	1.19	0.6926	0.43	1.35	0.4807	-0.31	-1.24	0.6433
0.09	1.06	0.8793	0.13	1.1	0.8027	0.38	1.3	0.4932
-0.38	-1.3	0.6119	-0.96	-1.94	0.1959	0.48	1.39	0.5077
-0.28	-1.21	0.6283	0.21	1.16	0.6876	-0.58	-1.5	0.3173
-0.79	-1.72	0.223	-1.38	-2.61	0.0281	-1.25	-2.37	0.0553
-0.26	-1.2	0.6678	-0.09	-1.06	0.8758	-0.37	-1.29	0.5347
-0.06	-1.04	0.9278	-0.32	-1.25	0.5976	-0.04	-1.03	0.9459
0.27	1.21	0.634	0.12	1.09	0.8281	-0.39	-1.31	0.5052
0.01	1.01	0.9856	0.69	1.62	0.2579	-0.05	-1.04	0.9349
0.16	1.12	0.7829	0.39	1.31	0.4867	0.04	1.02	0.9519
-0.02	-1.02	0.9701	0.11	1.08	0.8538	0.1	1.07	0.8684
0.58	1.5	0.3813	0.8	1.74	0.2027	0.87	1.82	0.1791
-0.39	-1.31	0.5714	0.15	1.11	0.8122	0.13	1.1	0.8401
1.29	2.44	0.0844	1.37	2.58	0.0581	-0.45	-1.36	0.5683
0.46	1.37	0.4654	-0.25	-1.19	0.6907	0.31	1.24	0.6175
-0.5	-1.42	0.4157	-0.15	-1.11	0.7953	0.79	1.73	0.154
0.75	1.68	0.3393	1.98	3.93	0.0081	1.97	3.93	0.0096
0.91	1.88	0.2257	1.09	2.14	0.1518	0.96	1.94	0.2037
-0.7	-1.62	0.2861	-0.48	-1.4	0.4281	0.19	1.14	0.7498
0.47	1.39	0.4354	0.34	1.27	0.5554	-0.28	-1.22	0.6561
-0.14	-1.1	0.8505	-0.07	-1.05	0.9201	0.19	1.14	0.7879
1.33	2.51	0.0601	1.66	3.17	0.0134	1.1	2.15	0.1185
0.52	1.43	0.4312	-0.39	-1.31	0.5534	0.64	1.56	0.316
0.67	1.59	0.2986	0.75	1.68	0.2215	0.48	1.4	0.4504
0.6	1.52	0.355	0.37	1.29	0.5597	0.94	1.92	0.1364
-0.98	-1.97	0.1344	-0.98	-1.97	0.1161	-2.26	-4.78	0.0012
-0.92	-1.89	0.1596	-0.73	-1.66	0.2283	-0.25	-1.19	0.6829
0.06	1.04	0.9258	-0.16	-1.12	0.7987	0.86	1.82	0.1691
0.71	1.63	0.3379	-0.53	-1.44	0.4841	1.09	2.13	0.1317
-0.12	-1.09	0.833	-0.18	-1.13	0.7351	-0.41	-1.33	0.475
-0.03	-1.02	0.9732	-0.11	-1.08	0.8887	-0.34	-1.27	0.6663
-0.91	-1.88	0.2534	-1.58	-2.99	0.0469	0.14	1.1	0.8581
-0.53	-1.44	0.3504	-0.72	-1.64	0.1864	-0.57	-1.48	0.3105
0.59	1.51	0.3965	1.12	2.17	0.0847	0.99	1.99	0.1383
-0.02	-1.02	0.9698	-0.2	-1.15	0.7416	0.11	1.08	0.8569
-0.12	-1.09	0.8475	-0.29	-1.22	0.6333	-0.2	-1.15	0.7475
0.85	1.8	0.1688	-0.12	-1.09	0.8451	0.63	1.55	0.3053

0.85	1.81	0.208	0.51	1.42	0.4449	0.79	1.73	0.2403
-0.17	-1.12	0.7046	-0.45	-1.37	0.2924	-0.52	-1.43	0.2488
-0.1	-1.07	0.8916	0.54	1.45	0.4334	1.03	2.05	0.1323
-0.07	-1.05	0.9236	-0.08	-1.06	0.9033	-0.67	-1.6	0.3326
0.74	1.67	0.3274	0.6	1.52	0.4135	0.23	1.17	0.7651
-0.69	-1.61	0.3169	-0.58	-1.5	0.3724	-0.64	-1.56	0.3452
0.62	1.54	0.432	0.54	1.45	0.4922	2.2	4.59	0.005
0.38	1.3	0.5864	0.68	1.6	0.296	0.68	1.61	0.3056
0.02	1.01	0.9793	0.8	1.74	0.2127	0.65	1.56	0.3331
-0.23	-1.17	0.7416	-0.49	-1.41	0.4724	0.13	1.09	0.8522
0.15	1.11	0.8409	-0.71	-1.64	0.3453	0.94	1.91	0.1989
0.33	1.25	0.6372	0.35	1.27	0.5957	0.64	1.56	0.3389
-0.35	-1.28	0.6217	-0.12	-1.09	0.8616	0.78	1.72	0.2465
0.27	1.21	0.7083	0.52	1.43	0.4455	0.91	1.88	0.1843
0.74	1.67	0.2861	0.49	1.4	0.4675	1.24	2.37	0.0607
0.2	1.15	0.777	0.46	1.37	0.4868	0.54	1.45	0.4261
-0.07	-1.05	0.9169	0.68	1.61	0.2739	0.9	1.87	0.157
-0.05	-1.04	0.9401	-0.37	-1.29	0.5685	0.7	1.62	0.2643
-0.24	-1.18	0.7042	-0.02	-1.02	0.967	0.19	1.14	0.7533
0.69	1.61	0.2991	0.01	1.01	0.9854	0.5	1.42	0.4477
-0.19	-1.14	0.7761	0.39	1.31	0.5287	0.03	1.02	0.9619
-0.17	-1.12	0.7179	0.1	1.08	0.8061	-0.14	-1.1	0.7631
-1.16	-2.23	0.1068	-0.4	-1.32	0.5392	0.09	1.06	0.8893
0.05	1.03	0.9346	-0.12	-1.09	0.8191	0.16	1.12	0.7631
-0.81	-1.75	0.2636	0.23	1.17	0.7309	-0.41	-1.33	0.5548
0.13	1.09	0.8544	0.23	1.18	0.7252	0.63	1.55	0.344
-0.69	-1.61	0.3038	-0.15	-1.11	0.8043	-0.7	-1.63	0.2873
-0.37	-1.29	0.5959	0.11	1.08	0.8606	-0.23	-1.18	0.7286
-0.19	-1.14	0.7815	0.04	1.03	0.9518	0.02	1.01	0.9821
1.39	2.62	0.0559	0.66	1.58	0.3666	1.47	2.76	0.0406
0.57	1.49	0.4265	0.18	1.13	0.7974	0.52	1.44	0.4662
0.51	1.42	0.4436	0.25	1.19	0.6969	0.09	1.07	0.8919
1.11	2.16	0.1251	0.43	1.34	0.5551	1.12	2.17	0.1186
0.37	1.3	0.6052	0.39	1.31	0.5766	0.82	1.77	0.2414
0.43	1.35	0.5611	0.16	1.11	0.8298	1.07	2.09	0.1345
0.36	1.28	0.6022	0.36	1.28	0.5911	0.66	1.58	0.3228
0.02	1.01	0.9808	0.37	1.3	0.5759	0.17	1.12	0.8077
0.28	1.22	0.7072	0.02	1.01	0.9814	0.54	1.45	0.4724
0.29	1.22	0.7063	0.64	1.56	0.3892	1.27	2.41	0.0886
-0.64	-1.56	0.4172	-0.52	-1.43	0.5141	1.68	3.2	0.0296
-0.52	-1.43	0.4779	-0.47	-1.39	0.5028	0.03	1.02	0.9708
0.19	1.14	0.7964	0.9	1.87	0.1858	0.54	1.45	0.4519
-0.69	-1.62	0.3017	-0.15	-1.11	0.8088	-0.86	-1.82	0.1972
0.08	1.06	0.9204	0.43	1.35	0.5745	1.12	2.17	0.1464
-0.99	-1.98	0.1761	-0.03	-1.02	0.9663	0.02	1.01	0.9795
-0.34	-1.27	0.6393	0.07	1.05	0.9224	0.46	1.38	0.4986
-0.05	-1.04	0.9148	-0.23	-1.17	0.6243	-0.25	-1.19	0.6116

-0.2	-1.15	0.7662	0.33	1.25	0.6064	0.03	1.02	0.9656
0.22	1.17	0.7323	-1.1	-2.15	0.1033	-0.09	-1.07	0.8896
-0.3	-1.23	0.6919	0.09	1.07	0.8945	0.19	1.14	0.7929
0.24	1.18	0.7574	-0.61	-1.52	0.4415	0.74	1.67	0.3424
-0.75	-1.68	0.3126	-0.61	-1.53	0.3856	-0.37	-1.29	0.6047
-0.32	-1.25	0.6631	0.71	1.64	0.2933	-2.17	-4.49	0.0052
0.04	1.03	0.9598	0.3	1.23	0.6781	1.12	2.17	0.1183
0.03	1.02	NA	0.03	1.02	NA	0.18	1.13	NA
0.29	1.22	0.6722	0.56	1.47	0.384	-0.31	-1.24	0.6557
1.5	2.83	0.0359	0.17	1.12	0.8211	1.11	2.16	0.1228
-0.32	-1.25	0.6744	-0.1	-1.07	0.8955	1.03	2.05	0.1515
0.89	1.85	0.2585	1.34	2.54	0.08	1.83	3.56	0.0184
1.54	2.9	0.0414	1.67	3.18	0.0216	1.8	3.49	0.0145
-0.33	-1.26	0.6747	0.17	1.13	0.8167	0.55	1.47	0.4663
-0.36	-1.28	0.5637	-0.12	-1.09	0.8387	-1.34	-2.53	0.0358
0.19	1.14	0.8108	0.17	1.13	0.8163	0.73	1.66	0.3315
-0.25	-1.19	0.6952	0.27	1.2	0.6512	0.33	1.25	0.5889
0.92	1.9	0.2141	0.49	1.41	0.5019	0.59	1.5	0.4313
0.53	1.44	0.3708	-0.67	-1.59	0.2438	0.51	1.43	0.3823
-0.35	-1.27	0.6301	-0.95	-1.94	0.1852	0.41	1.32	0.5505
-1.29	-2.45	0.0972	-1.19	-2.27	0.1173	-2	-4.01	0.0108
-0.35	-1.28	0.6152	0	1	0.9954	-0.22	-1.17	0.7427
0.16	1.12	0.8257	-0.57	-1.48	0.4307	0.39	1.31	0.5751
0.1	1.07	0.8913	-0.5	-1.42	0.4946	0.25	1.19	0.7279
-0.08	-1.06	0.917	-0.5	-1.41	0.5097	1.37	2.59	0.0524
-0.32	-1.25	0.6702	0.14	1.1	0.8441	-0.4	-1.32	0.5949
-0.71	-1.63	0.2937	-0.83	-1.78	0.1948	-0.3	-1.23	0.6434
-0.11	-1.08	0.8792	-0.33	-1.25	0.655	0.34	1.27	0.6358
0.96	1.95	0.2075	1.02	2.02	0.1693	1.11	2.16	0.1394
-0.31	-1.24	0.6957	0.46	1.38	0.5395	0.32	1.25	0.6783
0.95	1.94	0.2237	0.13	1.09	0.8709	1.76	3.39	0.0217
-0.32	-1.25	0.673	0.05	1.04	0.9401	0.43	1.35	0.5601
-0.16	-1.12	0.7675	-0.35	-1.28	0.4965	-0.59	-1.51	0.2793
0.62	1.53	0.3582	0.43	1.35	0.5108	-0.2	-1.15	0.774
1.02	2.02	0.1829	0.63	1.55	0.4004	1.56	2.94	0.0364
-0.31	-1.24	0.6886	-0.43	-1.35	0.5765	0.59	1.5	0.439
-0.3	-1.23	0.7068	-1.61	-3.06	0.0423	0.67	1.59	0.3743
0.88	1.83	0.2642	-0.61	-1.53	0.4412	1.21	2.31	0.1201
0.31	1.24	0.6814	-0.7	-1.63	0.3535	0.84	1.78	0.2466
-0.7	-1.62	0.3075	0.14	1.1	0.8286	-1.54	-2.92	0.0262
0.14	1.1	0.8615	-0.2	-1.15	0.7965	0.99	1.98	0.2008
0	-1	0.9998	-0.8	-1.74	0.2379	0.92	1.89	0.1494
0.36	1.29	0.6423	1.51	2.84	0.0378	1.19	2.29	0.1116
0.52	1.43	0.5142	-0.79	-1.72	0.3214	0.46	1.38	0.5599
0.43	1.34	NA	1.01	2.01	NA	0.96	1.95	NA
-0.26	-1.2	0.7405	-0.87	-1.82	0.2758	1.12	2.17	0.1524
0.23	1.18	0.7654	-0.18	-1.14	0.8124	0.85	1.81	0.2611

1.14	2.2	0.1382	0.76	1.7	0.3126	1.26	2.4	0.0969
-1.08	-2.11	0.1748	-0.29	-1.22	0.7121	0.82	1.76	0.2886
0.8	1.74	0.3114	0.34	1.27	0.6619	1.01	2.02	0.1959
-0.29	-1.22	0.7165	0.46	1.37	0.5617	0.41	1.33	0.6031
-0.26	-1.2	0.7455	-1.28	-2.43	0.108	1.27	2.41	0.0949
-0.6	-1.51	0.4541	-1.22	-2.34	0.1234	-0.17	-1.12	0.8318
0.99	1.98	0.2003	-0.18	-1.13	0.8174	1.96	3.89	0.0113
1.06	2.08	0.1727	1.88	3.69	0.0104	2.53	5.77	6.00E-04
-0.15	-1.11	0.8409	0.13	1.09	0.864	0.28	1.21	0.7098
0.08	1.05	0.9247	0.27	1.2	0.7323	0.79	1.73	0.3143
-0.85	-1.81	0.2741	-0.4	-1.32	0.5977	0.07	1.05	0.9241
-0.65	-1.57	0.4113	0.32	1.24	0.6862	0.26	1.2	0.7433
-0.16	-1.11	0.8307	-0.37	-1.29	0.623	1.34	2.53	0.0779
0.58	1.49	0.4591	-0.02	-1.02	0.9777	1.26	2.39	0.0972
1.39	2.63	0.0263	0.2	1.15	0.7579	0.94	1.92	0.1382
0.1	1.07	0.8981	-0.53	-1.44	0.5046	0.95	1.93	0.2257
-0.34	-1.27	0.6295	0.03	1.02	0.9629	-1.52	-2.87	0.0398
-0.11	-1.08	NA	0.56	1.47	NA	0.67	1.59	NA
1.81	3.5	0.0226	1.67	3.18	0.0339	1.09	2.13	0.1693
0.44	1.36	0.5111	-0.29	-1.22	0.6711	0.14	1.1	0.8399
-0.21	-1.16	0.7425	0.27	1.21	0.6591	0.21	1.16	0.7383
-0.36	-1.29	0.5657	-0.04	-1.03	0.9525	-1.2	-2.29	0.0636
0.17	1.12	0.8288	-0.24	-1.18	0.7537	-0.07	-1.05	0.9248
0.96	1.95	0.2256	0.04	1.02	0.9646	0.12	1.09	0.8749
0.46	1.37	0.5605	1.23	2.35	0.1178	0.89	1.85	0.2616
0.26	1.2	0.7322	-0.21	-1.15	0.788	1.63	3.1	0.0345
-0.17	-1.12	0.8345	-1.19	-2.29	0.1289	-0.03	-1.02	0.9725
0.39	1.31	0.5734	0.14	1.1	0.8485	0.5	1.42	0.4652
-0.64	-1.56	0.3955	0.21	1.16	0.7835	-1.35	-2.55	0.0722
-1.53	-2.88	0.0456	-1.8	-3.48	0.0201	0.09	1.07	0.9033
-0.47	-1.39	0.5094	0.02	1.02	0.9764	-0.23	-1.18	0.7434
0.27	1.21	0.662	-0.23	-1.18	0.7167	-0.13	-1.1	0.8312
0.7	1.63	0.3747	0.32	1.25	0.6869	0.28	1.22	0.7222
0.16	1.11	NA	0.16	1.12	NA	0.29	1.22	NA
0.52	1.44	0.4854	-0.45	-1.37	0.5485	1.38	2.6	0.0679
-1.33	-2.51	0.0953	-1.65	-3.13	0.0388	0.96	1.95	0.2231
0.11	1.08	0.8624	0.27	1.21	0.6873	0.42	1.34	0.5266
0.21	1.16	NA	0.44	1.35	NA	0.77	1.71	NA
-0.81	-1.75	0.2832	0.23	1.17	0.7676	1.41	2.67	0.0681
0.48	1.39	0.5328	0.31	1.24	0.6843	1.08	2.11	0.1597
0.47	1.38	0.5032	1.16	2.24	0.1052	0.28	1.22	0.6847
-0.62	-1.54	0.3507	-0.04	-1.03	0.9548	-0.96	-1.95	0.1474
1.05	2.07	NA	-0.38	-1.3	NA	1.68	3.21	NA
0.27	1.2	0.64	-0.75	-1.68	0.2085	-0.65	-1.57	0.2509

S.a.GN.vs.I	S.a.GN.vs.I	S.a.GN.vs.I	VancATN.v	VancATN.v	VancATN.v	VancATN.v	VancATN.v	VancATN.v
0.03	1.02	0.8937	-0.48	-1.4	0.0198	-0.51	-1.42	0.0085
0.04	1.03	0.9026	-0.79	-1.73	0.0128	-0.83	-1.78	0.0055
-0.52	-1.43	0.0049	-0.24	-1.18	0.207	0.28	1.21	0.1339
-0.05	-1.04	0.8378	-0.24	-1.18	0.4005	-0.18	-1.13	0.4871
-0.26	-1.2	0.1602	0.12	1.09	0.5171	0.39	1.31	0.0315
-0.02	-1.02	0.92	-0.55	-1.47	0.0229	-0.53	-1.44	0.019
-0.58	-1.5	0.0068	-0.08	-1.06	0.7191	0.51	1.42	0.0175
0.27	1.2	0.3602	0.29	1.22	0.3531	0.02	1.01	0.9446
-0.12	-1.08	0.7119	0.13	1.1	0.687	0.25	1.19	0.421
-0.08	-1.06	0.6316	-0.52	-1.43	0.007	-0.44	-1.35	0.0151
-0.1	-1.08	0.5573	-0.16	-1.11	0.4088	-0.05	-1.04	0.7677
-0.26	-1.2	0.6833	-0.35	-1.27	0.5999	-0.09	-1.06	0.8901
-0.27	-1.2	0.1929	-0.09	-1.07	0.6636	0.17	1.13	0.3885
-0.09	-1.07	0.7471	0.06	1.04	0.8461	0.15	1.11	0.5917
0.01	1.01	0.9682	-0.72	-1.65	0.0054	-0.73	-1.66	0.0025
0.02	1.01	0.9831	-0.1	-1.08	0.8952	-0.12	-1.09	0.8773
0.26	1.2	0.6841	0	1	0.9971	-0.26	-1.2	0.6866
-0.04	-1.03	0.8799	0.21	1.16	0.4076	0.25	1.19	0.2898
-0.11	-1.08	0.5928	-0.12	-1.09	0.5902	-0.01	-1.01	0.9715
-0.31	-1.24	0.1998	-0.21	-1.16	0.4068	0.1	1.07	0.6812
-0.04	-1.03	0.8541	-0.16	-1.12	0.4808	-0.12	-1.09	0.5636
0.52	1.44	0.4676	0.56	1.47	0.4535	0.03	1.02	0.9639
0.07	1.05	0.7919	-0.49	-1.41	0.1043	-0.57	-1.48	0.0448
0.17	1.13	0.4801	-0.27	-1.2	0.3213	-0.44	-1.35	0.0727
-1.01	-2.02	0.0113	-0.07	-1.05	0.857	0.94	1.92	0.0182
-0.06	-1.04	0.8441	-0.05	-1.04	0.8696	0.01	1	0.981
0.22	1.17	0.3944	-0.17	-1.13	0.5492	-0.39	-1.31	0.1275
-0.69	-1.62	0.1542	0.21	1.16	0.6739	0.9	1.87	0.0603
0.05	1.03	0.8873	-0.01	-1.01	0.9801	-0.05	-1.04	0.8647
-0.05	-1.04	0.8536	-0.04	-1.03	0.8867	0.01	1.01	0.9719
-0.21	-1.15	0.4399	-0.55	-1.47	0.0617	-0.34	-1.27	0.2175
-0.14	-1.1	0.6318	-0.45	-1.37	0.148	-0.31	-1.24	0.2831
0.11	1.08	0.7023	-0.21	-1.15	0.4932	-0.31	-1.24	0.2572
-0.22	-1.16	0.5127	0.54	1.46	0.0942	0.76	1.7	0.0122
-0.82	-1.77	0.0054	0.03	1.02	0.9276	0.85	1.8	0.0032
0.05	1.04	0.895	-0.12	-1.09	0.7778	-0.18	-1.13	0.6629
0.03	1.02	0.971	0.12	1.08	0.8746	0.09	1.06	0.8994
0.46	1.37	0.2029	-0.93	-1.9	0.0242	-1.38	-2.61	3.00E-04
0.25	1.19	0.4095	-0.32	-1.25	0.3268	-0.57	-1.48	0.0585
0.27	1.2	0.4255	0.54	1.46	0.1112	0.28	1.21	0.356
-0.79	-1.73	0.1156	0.14	1.1	0.7902	0.93	1.91	0.0622

-0.12	-1.09	0.7493	-0.74	-1.67	0.0757	-0.62	-1.54	0.1153
-0.6	-1.51	0.0566	-0.62	-1.54	0.0608	-0.03	-1.02	0.9314
-0.06	-1.04	0.8583	0	1	0.9908	0.06	1.04	0.8438
-0.2	-1.15	0.6025	0.65	1.57	0.0945	0.85	1.8	0.0191
0.1	1.07	0.7821	0.08	1.06	0.8212	-0.01	-1.01	0.9678
0.34	1.27	0.4419	0.07	1.05	0.8853	-0.27	-1.21	0.5288
0.6	1.52	0.0879	0.37	1.29	0.3325	-0.24	-1.18	0.4606
0.18	1.13	0.5937	-0.71	-1.63	0.0707	-0.89	-1.85	0.014
0.04	1.03	0.9617	0.2	1.15	0.7987	0.17	1.12	0.8349
0.18	1.13	0.6565	-0.66	-1.58	0.1421	-0.84	-1.79	0.0448
0.42	1.33	0.261	0.34	1.26	0.3866	-0.08	-1.06	0.8204
-0.85	-1.81	0.057	-1.1	-2.15	0.0218	-0.25	-1.19	0.5947
-0.79	-1.73	0.1013	0.55	1.47	0.2279	1.35	2.54	0.0033
-0.12	-1.09	0.7493	0.33	1.26	0.3907	0.45	1.37	0.2077
-0.16	-1.12	0.6839	0.28	1.21	0.4739	0.44	1.36	0.2314
-0.05	-1.03	0.9079	0.11	1.08	0.7926	0.15	1.11	0.6859
0.37	1.3	0.2833	0.4	1.32	0.2706	0.03	1.02	0.9325
-0.2	-1.15	0.6357	0.62	1.54	0.1341	0.83	1.77	0.0342
0.41	1.33	0.3097	-0.66	-1.58	0.1572	-1.07	-2.09	0.012
0.31	1.24	0.6767	0.35	1.27	0.6491	0.04	1.03	0.9608
0.43	1.34	0.2601	0.47	1.38	0.2341	0.04	1.03	0.9012
-0.1	-1.07	0.7802	0.23	1.17	0.5221	0.32	1.25	0.3247
-0.41	-1.33	0.2878	0.1	1.07	0.7927	0.51	1.43	0.1686
0.29	1.22	0.4475	0.34	1.27	0.3921	0.05	1.04	0.8864
0.09	1.07	NA	0.78	1.72	NA	0.69	1.61	NA
0.06	1.04	0.8719	-0.21	-1.16	0.6167	-0.28	-1.21	0.4814
0.06	1.04	0.8561	-0.29	-1.22	0.4389	-0.35	-1.28	0.3082
-0.09	-1.06	0.8367	0.61	1.52	0.1472	0.69	1.62	0.0735
-0.09	-1.06	0.8539	0.09	1.06	0.8558	0.17	1.13	0.7025
0.08	1.06	0.8331	-0.17	-1.12	0.6732	-0.24	-1.18	0.502
-0.01	-1.01	0.9743	0.42	1.34	0.2775	0.43	1.35	0.2223
-0.07	-1.05	0.9045	0.21	1.16	0.7398	0.28	1.22	0.6394
0.18	1.13	0.6562	0.33	1.26	0.423	0.15	1.11	0.6816
0.06	1.04	0.8868	0.05	1.03	0.9182	-0.01	-1.01	0.9716
0.38	1.3	0.2499	-0.23	-1.17	0.5307	-0.61	-1.53	0.0648
0.29	1.22	0.549	-1.74	-3.34	0.0031	-2.03	-4.09	3.00E-04
0.48	1.39	0.2058	0.09	1.07	0.8174	-0.38	-1.31	0.2875
-0.19	-1.14	0.633	-0.53	-1.45	0.2129	-0.35	-1.27	0.3939
0.05	1.03	0.8135	-0.38	-1.3	0.0745	-0.42	-1.34	0.0304
0.63	1.55	0.1362	0.1	1.07	0.8311	-0.53	-1.45	0.1885
0.06	1.04	0.8961	0.34	1.26	0.4351	0.28	1.22	0.4766
0.72	1.64	0.16	1.68	3.21	8.00E-04	0.97	1.95	0.0205
0.32	1.24	0.4452	0.15	1.11	0.732	-0.17	-1.12	0.6754
-0.57	-1.48	0.1802	0.03	1.02	0.9435	0.6	1.51	0.147
0.18	1.13	0.6729	-0.09	-1.07	0.839	-0.27	-1.2	0.5144
-0.18	-1.13	0.6604	-0.42	-1.34	0.3436	-0.24	-1.18	0.5691
0.04	1.03	0.9249	-0.09	-1.06	0.8387	-0.13	-1.09	0.7516

0.18	1.13	0.652	0.05	1.04	0.9045	-0.13	-1.09	0.7368
-0.58	-1.49	0.1235	-0.33	-1.26	0.385	0.24	1.18	0.5219
0.54	1.46	0.2146	0.82	1.77	0.0646	0.28	1.21	0.461
0	1	0.9968	0.43	1.35	0.337	0.43	1.35	0.3001
0.12	1.09	0.7592	-0.37	-1.29	0.4043	-0.49	-1.4	0.228
0.55	1.46	0.295	0.49	1.41	0.3706	-0.06	-1.04	0.9068
0.26	1.2	0.6171	-0.4	-1.32	0.4714	-0.66	-1.58	0.2057
0.48	1.4	0.2966	0.41	1.33	0.398	-0.07	-1.05	0.8647
-0.34	-1.26	0.2632	0.16	1.11	0.6098	0.49	1.41	0.0899
0.34	1.26	0.4808	0.02	1.01	0.9735	-0.32	-1.25	0.4921
-0.5	-1.41	0.3793	-0.17	-1.12	0.7788	0.33	1.26	0.5558
0.16	1.12	0.6962	-0.32	-1.25	0.4741	-0.48	-1.4	0.2441
-0.37	-1.29	0.2852	-0.29	-1.22	0.4285	0.08	1.06	0.8108
-0.32	-1.25	0.4898	0.14	1.1	0.7658	0.46	1.37	0.3034
-0.74	-1.67	0.2689	-0.45	-1.37	0.511	0.28	1.22	0.6705
-0.41	-1.33	0.3724	-0.29	-1.22	0.5388	0.11	1.08	0.8033
0.48	1.4	0.3067	-0.19	-1.14	0.7125	-0.68	-1.6	0.1506
0.42	1.34	0.5172	0.93	1.9	0.1622	0.51	1.42	0.415
0.02	1.01	0.9722	0.39	1.31	0.4013	0.37	1.29	0.3816
-0.19	-1.14	NA	0.04	1.03	NA	0.23	1.17	NA
-0.37	-1.29	0.4429	-0.42	-1.34	0.4084	-0.05	-1.04	0.9179
0.48	1.4	0.279	-0.14	-1.1	0.7769	-0.62	-1.54	0.1578
0.26	1.2	0.4631	0.32	1.25	0.3939	0.06	1.04	0.8672
-0.36	-1.28	0.4389	-0.21	-1.16	0.6562	0.14	1.11	0.7539
0.41	1.33	0.3744	-0.11	-1.08	0.8331	-0.51	-1.43	0.2563
0.27	1.2	0.3051	-0.1	-1.08	0.7134	-0.37	-1.29	0.1457
-0.38	-1.3	0.4278	-0.08	-1.05	0.8767	0.31	1.24	0.5189
-0.34	-1.27	0.5058	0.08	1.06	0.8785	0.42	1.34	0.399
0.41	1.33	0.3166	-1.09	-2.13	0.0201	-1.49	-2.82	6.00E-04
1.14	2.21	0.0281	0.69	1.61	0.2148	-0.45	-1.37	0.3237
-0.28	-1.21	0.5846	0.01	1.01	0.9809	0.29	1.22	0.5594
0.17	1.12	0.6305	-0.13	-1.09	0.7359	-0.29	-1.22	0.39
0.23	1.17	0.6728	0.34	1.26	0.5418	0.11	1.08	0.8256
-0.25	-1.19	0.6681	0.22	1.16	0.7069	0.47	1.38	0.4009
-0.09	-1.07	0.8476	-0.04	-1.03	0.9429	0.06	1.04	0.9043
0.65	1.57	0.1758	-0.02	-1.02	0.9639	-0.68	-1.6	0.1511
0.03	1.02	0.9462	-0.63	-1.55	0.2313	-0.66	-1.58	0.1805
-0.65	-1.57	0.2329	0.13	1.09	0.8111	0.78	1.72	0.1421
0.12	1.09	0.7787	-0.26	-1.2	0.5799	-0.38	-1.3	0.3784
-0.98	-1.98	0.0471	-0.24	-1.18	0.6146	0.74	1.67	0.1349
-1.54	-2.91	0.0249	0.34	1.27	0.5855	1.88	3.68	0.0048
-0.28	-1.22	0.6055	0.66	1.58	0.2117	0.94	1.92	0.0614
-0.91	-1.88	0.1034	0.21	1.16	0.6808	1.13	2.19	0.0362
0.08	1.05	0.8842	0.17	1.12	0.755	0.09	1.07	0.8533
-0.22	-1.17	0.6649	-0.4	-1.32	0.4611	-0.18	-1.13	0.7299
0.17	1.13	0.7648	0.56	1.48	0.3347	0.39	1.31	0.4656
0.06	1.04	0.9083	0.28	1.22	0.6057	0.22	1.17	0.6624

-0.29	-1.22	0.6681	1.12	2.17	0.0849	1.4	2.65	0.0245
-0.51	-1.42	0.3232	-0.12	-1.08	0.8251	0.39	1.31	0.4402
-0.29	-1.22	0.5945	0.31	1.24	0.5649	0.6	1.52	0.2455
-0.73	-1.66	0.1831	-0.02	-1.02	0.9674	0.71	1.64	0.19
-0.69	-1.61	0.2191	-0.4	-1.32	0.4845	0.29	1.22	0.6095
0.11	1.08	0.8452	0.46	1.37	0.4206	0.35	1.27	0.5075
0.81	1.75	0.1585	0.33	1.26	0.588	-0.48	-1.39	0.3753
-0.83	-1.77	0.1043	-0.45	-1.36	0.3842	0.38	1.3	0.4644
0.38	1.3	0.4737	0.56	1.47	0.3019	0.18	1.13	0.7078
0.32	1.25	0.5509	0.23	1.17	0.6808	-0.09	-1.06	0.8636
0.18	1.13	0.7535	-0.56	-1.48	0.3618	-0.74	-1.67	0.2015
0.05	1.03	0.9225	0.29	1.22	0.5668	0.24	1.18	0.6061
-0.58	-1.49	0.4261	0.86	1.81	0.2239	1.44	2.71	0.039
0.49	1.4	0.3288	-0.3	-1.23	0.5916	-0.79	-1.73	0.118
-0.6	-1.51	0.3275	-0.46	-1.38	0.4656	0.14	1.1	0.8248
0.17	1.13	0.7516	-0.11	-1.08	0.8451	-0.28	-1.22	0.5952
-0.26	-1.2	0.6454	0.02	1.01	0.9794	0.28	1.21	0.6191
-0.15	-1.11	0.7569	-0.67	-1.59	0.2203	-0.51	-1.43	0.3219
0.68	1.6	0.2341	-0.07	-1.05	0.916	-0.75	-1.68	0.1849
0.22	1.17	0.6594	-0.13	-1.09	0.8138	-0.35	-1.28	0.4826
0.13	1.09	0.8106	0.12	1.09	0.8282	-0.01	-1.01	0.9888
0.22	1.16	0.6935	0.28	1.22	0.6213	0.07	1.05	0.9015
0.54	1.45	0.383	0.52	1.43	0.4157	-0.02	-1.01	0.9745
0.08	1.06	0.902	-1.74	-3.33	0.0182	-1.82	-3.52	0.0104
-0.7	-1.63	0.2117	-0.15	-1.11	0.7953	0.56	1.47	0.3185
0.36	1.28	0.5248	1.29	2.45	0.0178	0.94	1.92	0.052
1.22	2.34	0.0897	1.22	2.34	0.0987	0	-1	0.9987
0.18	1.13	0.8188	0.05	1.03	0.9534	-0.14	-1.1	0.8641
0.22	1.16	0.7243	0.89	1.85	0.1415	0.67	1.59	0.2211
-0.13	-1.09	0.8014	-0.76	-1.69	0.1882	-0.63	-1.54	0.2533
0.07	1.05	0.9199	0.32	1.25	0.6315	0.26	1.2	0.6862
0.34	1.26	0.552	-0.23	-1.17	0.7123	-0.56	-1.48	0.3201
-0.91	-1.88	0.1324	0.13	1.09	0.8247	1.04	2.05	0.0782
0.08	1.06	0.8803	-0.18	-1.14	0.7447	-0.26	-1.2	0.6163
-0.23	-1.18	0.676	0.34	1.26	0.547	0.57	1.48	0.2839
0	1	0.9973	-1.28	-2.43	0.0639	-1.28	-2.43	0.0531
0.19	1.14	0.7653	0.67	1.59	0.2821	0.49	1.4	0.3984
-0.23	-1.17	0.7087	0.8	1.74	0.1722	1.03	2.04	0.0659
-1.23	-2.35	0.0803	0.38	1.3	0.5685	1.61	3.06	0.0187
-0.06	-1.04	0.9014	-0.29	-1.22	0.5944	-0.22	-1.17	0.6586
-0.08	-1.06	0.9142	-0.31	-1.24	0.6865	-0.23	-1.17	0.7601
-0.67	-1.59	0.399	1.05	2.07	0.1778	1.72	3.29	0.0269
-0.19	-1.14	0.726	-0.04	-1.03	0.9471	0.15	1.11	0.7736
0.53	1.44	0.3576	0.4	1.32	0.5025	-0.13	-1.09	0.8149
-0.17	-1.13	0.7574	0.13	1.1	0.8159	0.3	1.24	0.5716
-0.17	-1.12	0.771	-0.08	-1.06	0.894	0.09	1.06	0.8762
-0.97	-1.96	0.0757	-0.22	-1.16	0.6857	0.75	1.69	0.1673

-0.35	-1.27	0.5512	-0.06	-1.05	0.9138	0.28	1.22	0.6226
-0.28	-1.22	0.4828	-0.35	-1.28	0.4099	-0.07	-1.05	0.8647
0.64	1.56	0.3343	1.13	2.19	0.0865	0.5	1.41	0.3978
-0.01	-1.01	0.9823	-0.61	-1.53	0.3622	-0.6	-1.51	0.35
-0.14	-1.1	0.8402	-0.51	-1.43	0.4778	-0.37	-1.3	0.5921
0.11	1.08	0.8688	0.05	1.03	0.9411	-0.06	-1.04	0.928
-0.09	-1.06	0.9126	1.58	2.98	0.0427	1.66	3.17	0.0295
0.3	1.23	0.6077	0.31	1.24	0.6146	0.01	1	0.9927
0.78	1.72	0.196	0.63	1.54	0.3213	-0.16	-1.11	0.7785
-0.26	-1.2	0.6955	0.36	1.28	0.5861	0.62	1.54	0.3309
-0.86	-1.82	0.2354	0.79	1.72	0.2608	1.65	3.14	0.0185
0.02	1.02	0.9678	0.31	1.24	0.6112	0.29	1.22	0.6166
0.24	1.18	0.7221	1.14	2.2	0.0836	0.9	1.87	0.1394
0.25	1.19	0.6931	0.64	1.56	0.3164	0.39	1.31	0.5062
-0.25	-1.19	0.6772	0.51	1.42	0.3857	0.75	1.69	0.1762
0.26	1.2	0.6712	0.34	1.26	0.5911	0.08	1.06	0.8916
0.76	1.69	0.2017	0.97	1.96	0.1076	0.22	1.16	0.6792
-0.32	-1.25	0.6045	0.75	1.68	0.2059	1.07	2.1	0.0608
0.22	1.16	0.7039	0.44	1.35	0.4591	0.22	1.16	0.6873
-0.68	-1.6	0.2526	-0.19	-1.14	0.7534	0.49	1.41	0.4054
0.58	1.5	0.3264	0.22	1.17	0.7233	-0.36	-1.28	0.5257
0.27	1.21	0.4919	0.03	1.02	0.9445	-0.24	-1.18	0.5323
0.76	1.69	0.2628	1.25	2.38	0.0658	0.49	1.4	0.4136
-0.17	-1.12	0.7327	0.12	1.09	0.814	0.29	1.22	0.5491
1.04	2.05	0.1166	0.4	1.32	0.5734	-0.64	-1.56	0.31
0.11	1.08	0.8656	0.51	1.42	0.4217	0.4	1.32	0.4952
0.54	1.45	0.3816	-0.01	-1.01	0.9859	-0.55	-1.46	0.3605
0.48	1.39	0.4426	0.13	1.1	0.8413	-0.35	-1.27	0.5658
0.23	1.18	0.7123	0.21	1.16	0.7504	-0.02	-1.02	0.9679
-0.73	-1.66	0.2513	0.08	1.05	0.9023	0.81	1.75	0.1943
-0.39	-1.31	0.5489	-0.05	-1.04	0.9376	0.34	1.27	0.599
-0.26	-1.2	0.6572	-0.42	-1.34	0.4957	-0.16	-1.12	0.7865
-0.69	-1.61	0.2891	0.01	1.01	0.9908	0.69	1.62	0.2762
0.01	1.01	0.9821	0.45	1.37	0.4939	0.43	1.35	0.4842
-0.27	-1.21	0.6855	0.64	1.56	0.3392	0.91	1.88	0.1568
0	-1	0.9938	0.3	1.23	0.6243	0.31	1.24	0.596
0.36	1.28	0.5737	0.15	1.11	0.8189	-0.2	-1.15	0.7384
-0.27	-1.2	0.706	0.25	1.19	0.7246	0.52	1.43	0.4547
0.35	1.27	0.6258	0.98	1.97	0.1723	0.63	1.55	0.3493
0.12	1.09	0.878	2.32	4.99	0.0027	2.2	4.58	0.0043
0.05	1.03	0.9432	0.55	1.46	0.4332	0.5	1.41	0.4526
0.71	1.64	0.2653	0.35	1.27	0.6075	-0.37	-1.29	0.5497
0.55	1.46	0.3759	-0.17	-1.12	0.8025	-0.71	-1.64	0.2436
0.35	1.27	0.6386	1.04	2.06	0.1676	0.69	1.61	0.3408
0.96	1.94	0.1554	1	2	0.1472	0.04	1.03	0.9408
0.41	1.33	0.542	0.81	1.75	0.2311	0.4	1.32	0.5167
-0.18	-1.13	0.6834	-0.2	-1.15	0.6679	-0.02	-1.01	0.9647

0.53	1.44	0.3831	0.23	1.17	0.7173	-0.3	-1.23	0.6085
-1.33	-2.51	0.0371	-0.31	-1.24	0.6072	1.01	2.02	0.1133
0.39	1.31	0.5725	0.49	1.4	0.4922	0.1	1.07	0.884
-0.85	-1.8	0.2735	0.5	1.41	0.517	1.35	2.55	0.0785
0.13	1.1	0.8497	0.38	1.3	0.6011	0.24	1.18	0.7231
1.03	2.04	0.1163	-1.85	-3.6	0.0156	-2.88	-7.35	1.00E-04
0.26	1.2	0.7059	1.08	2.11	0.1159	0.82	1.76	0.2014
0	1	NA	0.16	1.11	NA	0.15	1.11	NA
0.27	1.21	0.6458	-0.6	-1.52	0.3595	-0.87	-1.83	0.1542
-1.33	-2.52	0.0391	-0.39	-1.31	0.5326	0.94	1.92	0.1478
0.23	1.17	0.7559	1.36	2.56	0.0557	1.13	2.19	0.0898
0.45	1.37	0.5442	0.94	1.92	0.2155	0.49	1.4	0.5048
0.13	1.1	0.8378	0.27	1.2	0.6864	0.13	1.1	0.8273
0.5	1.42	0.497	0.88	1.84	0.2393	0.38	1.3	0.5931
0.24	1.18	0.6675	-0.98	-1.97	0.1061	-1.22	-2.33	0.0337
-0.01	-1.01	0.9883	0.54	1.46	0.4535	0.55	1.47	0.4259
0.52	1.43	0.3594	0.58	1.49	0.3209	0.06	1.04	0.9055
-0.43	-1.35	0.5197	-0.34	-1.26	0.6256	0.1	1.07	0.8874
-1.2	-2.3	0.0231	-0.01	-1.01	0.9783	1.18	2.27	0.024
-0.61	-1.52	0.3923	0.75	1.68	0.2582	1.36	2.56	0.0406
0.11	1.08	0.8905	-0.71	-1.64	0.3691	-0.82	-1.76	0.2915
0.36	1.28	0.5773	0.13	1.09	0.8492	-0.23	-1.17	0.7118
-0.72	-1.65	0.2891	0.24	1.18	0.7233	0.96	1.94	0.1486
-0.61	-1.52	0.3934	0.15	1.11	0.8276	0.76	1.69	0.2748
-0.42	-1.33	0.5701	1.45	2.73	0.0339	1.87	3.65	0.0054
0.46	1.38	0.5096	-0.08	-1.06	0.9159	-0.54	-1.46	0.4377
-0.13	-1.09	0.8423	0.41	1.33	0.5248	0.54	1.45	0.3797
-0.21	-1.16	0.7644	0.46	1.37	0.5159	0.67	1.59	0.3253
0.05	1.04	0.9377	0.15	1.11	0.8316	0.1	1.07	0.8859
0.77	1.7	0.2997	0.63	1.54	0.4101	-0.14	-1.1	0.8434
-0.83	-1.77	0.2881	0.81	1.75	0.2846	1.63	3.1	0.0306
0.38	1.3	0.5977	0.75	1.68	0.2987	0.37	1.3	0.5814
-0.19	-1.14	0.6933	-0.43	-1.35	0.4046	-0.24	-1.18	0.6249
-0.19	-1.14	0.7443	-0.82	-1.76	0.1996	-0.63	-1.55	0.3059
-0.38	-1.31	0.5835	0.54	1.45	0.4347	0.93	1.9	0.1703
-0.12	-1.08	0.8796	0.9	1.87	0.2291	1.02	2.02	0.1633
-1.32	-2.49	0.0948	0.96	1.95	0.1949	2.28	4.87	0.0027
-1.49	-2.8	0.056	0.33	1.26	0.6615	1.82	3.52	0.018
-1.01	-2.02	0.1639	0.53	1.44	0.4376	1.54	2.91	0.0273
0.84	1.79	0.1755	-0.85	-1.8	0.2064	-1.68	-3.21	0.0077
-0.34	-1.27	0.6632	0.85	1.8	0.2671	1.19	2.28	0.1156
-0.8	-1.74	0.215	0.92	1.89	0.1256	1.71	3.28	0.0044
1.14	2.21	0.0999	0.83	1.78	0.2518	-0.31	-1.24	0.6249
-1.3	-2.47	0.0976	-0.06	-1.04	0.9436	1.25	2.38	0.1122
0.58	1.49	NA	0.54	1.45	NA	-0.04	-1.03	NA
-0.6	-1.52	0.4482	1.38	2.6	0.076	1.98	3.95	0.0106
-0.42	-1.33	0.58	0.62	1.54	0.3999	1.04	2.05	0.1533

-0.37	-1.3	0.5962	0.12	1.09	0.8628	0.5	1.41	0.4743
0.79	1.72	0.3158	1.89	3.72	0.0138	1.11	2.16	0.1395
-0.46	-1.37	0.5518	0.21	1.16	0.7806	0.67	1.59	0.3768
0.74	1.67	0.3453	0.7	1.62	0.3792	-0.04	-1.03	0.9553
-1.02	-2.03	0.1976	1.53	2.88	0.0427	2.55	5.87	8.00E-04
-0.63	-1.55	0.4283	0.43	1.35	0.5844	1.06	2.08	0.1768
-1.17	-2.25	0.1432	0.97	1.96	0.2229	2.14	4.4	0.0073
0.82	1.77	0.2368	1.47	2.77	0.0346	0.64	1.56	0.297
0.28	1.21	0.7207	0.43	1.34	0.5791	0.15	1.11	0.8496
0.19	1.14	0.8053	0.71	1.64	0.3621	0.52	1.44	0.4959
0.45	1.37	0.5433	0.93	1.9	0.2223	0.47	1.39	0.5179
0.97	1.95	0.2148	0.91	1.88	0.2489	-0.06	-1.04	0.9415
-0.21	-1.16	0.7832	1.49	2.82	0.0543	1.7	3.26	0.0308
-0.6	-1.52	0.4268	0.68	1.6	0.3523	1.28	2.43	0.0781
-1.2	-2.29	0.0264	-0.45	-1.37	0.394	0.74	1.67	0.1745
-0.63	-1.55	0.4282	0.85	1.8	0.2798	1.48	2.78	0.0589
0.37	1.29	0.5641	-1.18	-2.27	0.1029	-1.55	-2.93	0.0237
0.67	1.59	NA	0.78	1.71	NA	0.11	1.08	NA
-0.14	-1.1	0.8581	-0.72	-1.65	0.3655	-0.58	-1.49	0.4615
-0.73	-1.66	0.3132	-0.31	-1.24	0.6684	0.43	1.34	0.5573
0.49	1.4	0.4025	0.43	1.35	0.4805	-0.06	-1.04	0.918
0.33	1.26	0.5622	-0.83	-1.78	0.1746	-1.16	-2.23	0.0453
-0.41	-1.33	0.5834	-0.24	-1.18	0.7501	0.17	1.12	0.8227
-0.93	-1.9	0.2422	-0.84	-1.78	0.2943	0.09	1.06	0.9097
0.77	1.71	0.3315	0.43	1.34	0.5932	-0.35	-1.27	0.664
-0.47	-1.38	0.5538	1.37	2.59	0.0841	1.84	3.58	0.0211
-1.03	-2.04	0.1849	0.14	1.1	0.8592	1.17	2.25	0.1311
-0.25	-1.19	0.7339	0.12	1.08	0.8731	0.37	1.29	0.6205
0.85	1.8	0.2797	-0.71	-1.64	0.3581	-1.56	-2.95	0.0467
-0.27	-1.21	0.7289	1.62	3.07	0.0384	1.89	3.71	0.0169
0.49	1.41	0.5173	0.24	1.18	0.7505	-0.26	-1.19	0.7371
-0.51	-1.42	0.4596	-0.41	-1.33	0.5412	0.1	1.07	0.8829
-0.39	-1.31	0.6191	-0.42	-1.34	0.5932	-0.03	-1.02	0.9648
0	1	NA	0.14	1.1	NA	0.13	1.1	NA
-0.97	-1.97	0.213	0.86	1.81	0.2752	1.83	3.56	0.0202
-0.32	-1.25	0.6886	2.29	4.89	0.0037	2.61	6.1	9.00E-04
0.16	1.12	0.8232	0.3	1.24	0.6624	0.14	1.11	0.8419
0.23	1.17	NA	0.56	1.48	NA	0.34	1.26	NA
1.04	2.05	0.1837	2.22	4.67	0.0044	1.19	2.27	0.1374
-0.16	-1.12	0.8367	0.6	1.52	0.4461	0.76	1.7	0.3359
0.7	1.62	0.3555	-0.18	-1.14	0.8026	-0.88	-1.84	0.2434
0.58	1.5	0.4211	-0.34	-1.27	0.6232	-0.93	-1.9	0.2009
-1.43	-2.7	NA	0.63	1.55	NA	2.07	4.19	NA
-1.01	-2.02	0.1102	-0.92	-1.89	0.131	0.09	1.07	0.8802

netiated proteins (FC > 2 and P<.01)

flag.ATN.v:	flag.IgAN E	flag.IgA_H!	flag.S.a.GN	flag.VancA'	flag.pooled.IgAN E0.IgAN E1.vs.NLTx	flag.IgAN E	flag.IgAN E
0	0	0	0	0	0	0	0
0	0	0	0	0	0	0	0
0	0	0	0	0	0	0	0
0	0	0	0	0	0	0	0
0	0	0	0	0	0	0	0
0	0	0	0	0	0	0	0
0	0	0	0	0	0	0	0
0	0	0	0	0	0	0	0
0	0	0	0	0	0	0	0
0	0	0	0	0	0	0	0
0	0	0	0	0	0	0	0
0	0	0	0	0	0	0	0
0	0	0	0	0	0	0	0
0	0	0	0	0	0	0	0
0	0	0	0	0	0	0	0
0	0	0	0	0	0	0	0
0	0	0	0	0	0	0	0
0	0	0	0	0	0	0	0
0	0	0	0	0	0	0	0
0	0	0	0	0	0	0	0
0	0	0	0	0	0	0	0
0	0	0	0	0	0	0	0
0	0	0	0	0	0	0	0
0	0	0	0	0	0	0	0
0	0	1	0	1	0	0	0
0	0	0	0	0	0	0	0
0	0	0	0	0	0	0	0
0	0	0	0	0	0	0	0
1	1	1	1	1	1	1	0
0	0	0	0	0	0	0	0
0	0	0	0	0	0	0	0
0	0	0	0	0	0	0	0
0	0	0	0	0	0	0	0
0	0	0	1	0	0	0	0
0	0	0	0	0	0	0	0
0	0	0	0	0	0	0	0
0	0	0	0	0	0	0	0
0	0	0	0	0	0	0	0
0	0	0	0	0	0	0	0
0	0	1	0	0	0	0	0
0	0	0	0	0	0	0	0

0	0	0	0	0		0	0	0
0	0	0	0	0		0	0	0
0	0	0	0	0		0	0	0
0	0	0	0	0		0	0	0
0	0	0	0	0		0	0	0
0	0	0	0	0		0	0	0
0	0	0	0	0		0	0	0
0	0	0	0	0		0	0	0
0	0	0	0	0		0	0	0
0	0	0	0	0		0	0	0
0	0	0	0	0		0	0	0
0	0	1	0	0		0	0	0
0	0	0	1	0		0	0	0
0	0	0	0	0		0	0	0
0	0	0	0	0		0	0	0
0	0	0	0	0		0	0	0
0	0	0	0	0		0	0	0
0	0	0	0	0		0	0	0
0	0	0	0	0		0	0	0
0	0	0	0	0		0	0	0
0	0	0	0	0		0	0	0
0	0	0	0	0		0	0	0
0	0	0	0	0		0	0	0
0	0	0	0	0		0	0	0
0	0	0	0	0		0	0	0
0	0	0	0	0		0	0	0
0	0	0	0	0		0	0	0
0	0	0	0	0		0	0	0
0	0	0	0	0		0	0	0
0	0	0	0	0		0	0	0
0	0	0	0	0		0	0	0
1	1	1	1	1		1	0	0
0	0	0	0	0		0	0	0
0	0	0	0	0		0	0	0
0	0	0	0	0		0	0	0
0	0	0	0	0		0	0	0
0	0	0	0	0		0	0	0
0	0	0	0	0		0	0	0
0	0	0	0	0		0	0	0
0	0	0	0	0		0	0	0
0	0	0	0	0		0	0	0
0	0	1	0	0		0	0	0
0	0	0	0	0		0	0	0
0	0	0	0	0		0	0	0
0	0	0	0	0		0	0	0
0	0	0	0	0		0	0	0
0	0	0	0	0		0	0	0

0	0	0	0	0	0	0	0
0	0	0	0	0	0	0	0
0	0	0	0	0	0	0	0
0	0	0	0	0	0	0	0
0	0	0	0	0	0	0	0
0	0	0	0	0	0	0	0
0	0	0	0	0	0	0	0
0	0	0	0	0	0	0	0
0	0	0	0	0	0	0	0
0	0	0	0	0	0	0	0
1	1	1	1	1	1	0	0
0	0	0	0	0	0	0	0
0	0	0	0	0	0	0	0
0	0	0	0	0	0	0	0
1	1	1	1	1	1	0	0
1	1	1	1	1	0	0	0
0	0	0	0	0	0	0	0
0	0	0	0	1	0	0	0
0	0	0	0	0	0	0	0
0	0	0	0	0	0	0	0
0	0	0	0	0	0	0	0
0	0	0	0	0	0	0	0
0	0	0	0	0	0	0	0
0	0	0	0	0	0	0	0
0	0	0	0	0	0	0	0
0	0	0	0	0	0	0	0
0	0	0	0	0	0	0	0
0	0	0	0	0	0	0	0
0	0	0	0	0	0	0	0
0	0	0	0	0	0	0	0
0	0	0	0	0	0	0	0
0	0	0	0	0	0	0	0
0	0	0	0	0	0	0	0
0	1	1	1	0	0	0	0
0	0	0	0	0	0	0	0
0	0	0	0	0	0	0	0
0	0	0	0	0	0	0	0
0	0	0	0	0	0	0	0
0	0	0	0	0	0	0	0
0	0	0	0	0	0	0	0
0	1	1	1	0	0	0	0
0	0	0	0	0	0	0	0
0	0	0	1	0	0	0	0
0	0	0	0	0	0	0	0
0	0	0	0	0	0	0	0
0	0	1	1	0	0	0	0
0	0	0	0	0	0	0	0

0	0	0	1	0	0	0	0
0	0	0	0	0	0	0	0
0	0	0	0	0	0	0	0
0	0	0	0	0	0	0	0
0	0	0	0	0	0	0	0
0	0	0	0	0	0	0	0
0	0	0	0	0	0	0	0
0	0	0	0	0	0	0	0
0	0	0	0	0	0	0	0
0	0	0	0	0	0	0	0
0	0	0	0	0	0	0	0
0	0	0	0	0	0	0	0
0	0	1	1	0	0	0	0
0	0	0	0	0	0	0	0
1	1	1	1	1	1	0	0
0	0	0	0	0	0	0	0
0	0	0	0	0	0	0	0
0	0	0	0	0	0	0	0
0	0	0	0	0	0	0	0
0	0	0	0	0	0	0	0
0	0	0	0	0	0	0	0
0	0	0	0	0	0	0	0
0	0	0	0	0	0	0	0
0	0	0	0	0	0	0	0
0	0	0	0	0	0	0	0
0	0	0	0	1	0	0	0
0	0	0	0	0	0	0	0
0	0	0	0	0	0	0	0
0	0	0	0	0	0	0	0
0	0	0	0	0	0	0	0
1	0	1	1	0	0	0	0
0	0	0	0	0	0	0	0
0	0	0	0	0	0	0	0
0	0	0	0	0	0	0	0
0	0	0	0	0	0	0	0
0	0	0	0	0	0	1	0
0	0	0	0	0	0	0	0
0	0	0	0	0	0	0	0
0	0	0	0	0	0	0	0
0	0	0	0	0	0	0	0
0	0	0	0	0	0	0	0
1	1	1	1	1	1	0	0
0	1	0	0	0	0	0	0
0	0	0	0	0	0	0	0
0	0	0	0	0	0	0	0
0	0	0	0	0	0	0	0
0	0	1	0	0	0	0	0

0	0	0	0	0	0	0	0
0	0	0	0	0	0	0	0
0	0	0	0	0	0	0	0
0	0	0	0	0	0	0	0
0	0	0	0	0	0	0	0
0	0	0	0	0	0	0	0
1	0	0	0	1	0	0	0
0	0	0	0	0	0	0	0
0	0	0	0	0	0	0	0
0	0	0	0	0	0	0	0
0	0	0	0	0	0	0	0
0	0	0	0	0	0	0	0
0	0	0	0	0	0	0	0
0	0	0	0	0	0	0	0
0	0	0	0	0	0	0	0
0	0	0	0	0	0	0	0
0	0	0	0	0	0	0	0
0	0	0	0	0	0	0	0
0	0	0	0	0	0	0	0
0	0	0	0	0	0	0	0
0	0	0	0	0	0	0	0
0	0	0	0	0	0	0	0
0	0	0	0	0	0	0	0
0	0	0	0	0	0	0	0
0	0	0	0	0	0	0	0
0	0	0	0	0	0	0	0
0	0	0	0	0	0	0	0
0	0	0	0	0	0	0	0
0	0	0	0	0	0	0	0
0	0	0	0	0	0	0	0
0	0	0	0	0	0	0	0
0	0	0	0	0	0	0	0
0	0	0	0	0	0	0	0
0	0	0	0	0	0	0	0
0	0	0	0	0	0	0	0
0	0	0	0	0	0	0	0
0	0	0	0	0	0	0	0
0	0	0	0	0	0	0	0
0	0	0	0	0	0	0	0
1	1	1	1	0	1	0	0
0	0	0	0	0	0	0	0
0	0	0	0	0	0	0	0
0	0	0	0	0	0	0	0
0	0	1	1	1	1	0	0
0	0	0	0	0	0	0	0
0	0	0	0	0	0	0	0
0	0	0	0	0	0	0	0

0	0	0	0	0	0	0	0
0	0	0	0	0	0	0	0
0	0	0	0	0	0	0	0
0	0	0	0	0	0	0	0
0	0	0	0	0	0	0	0
0	0	0	0	0	0	0	0
0	0	0	0	1	0	0	0
0	0	0	0	0	0	0	0
0	0	0	0	0	0	0	0
0	0	0	0	0	0	0	0
0	0	0	0	0	0	0	0
1	0	0	1	1	0	0	0
0	0	0	0	0	0	0	0
0	0	0	0	0	0	0	0
0	0	0	0	0	0	0	0
0	0	0	0	0	0	0	0
0	0	0	0	0	0	0	0
0	0	0	0	0	0	0	0
1	0	1	0	1	0	0	0
0	0	0	0	0	0	0	0
0	0	0	0	1	0	0	0
0	0	0	0	0	0	0	0
0	0	0	0	0	0	0	0
0	0	0	0	0	0	0	0
0	0	0	0	0	0	0	0
0	0	0	0	0	0	0	0
1	0	0	0	0	0	0	0
0	0	0	0	0	0	0	0
0	0	0	0	0	0	0	0
0	0	0	0	0	0	0	0
0	0	0	0	0	0	0	0
0	0	0	0	0	0	0	0
0	0	0	0	0	0	0	0
0	0	0	0	0	0	0	0
0	0	0	0	0	0	0	0
0	0	0	0	0	0	0	0
0	0	0	1	0	0	0	0
0	0	0	0	0	0	0	0
0	0	0	0	0	0	0	0
1	0	0	0	0	0	1	1
1	1	1	1	0	1	0	0
0	0	0	0	0	0	0	0
0	0	0	0	0	0	0	0
0	0	0	0	0	0	0	0
0	0	0	0	0	0	0	0
0	0	0	1	0	0	0	0
0	0	0	0	0	0	0	0

0	0	0	0	0	0	0	0
1	0	1	0	0	1	0	0
0	0	0	0	0	0	0	0
0	0	0	0	0	0	0	0
0	0	0	0	0	0	0	0
0	0	0	1	0	0	0	0
0	0	0	0	0	0	0	0
1	0	1	1	1	0	1	0
1	1	1	1	1	1	0	0
0	0	0	0	0	0	0	0
0	0	0	0	1	0	1	0
0	0	1	0	0	1	0	0
1	1	1	1	0	1	0	0
0	0	0	0	0	0	0	0
0	0	0	0	0	0	0	0
0	0	0	1	0	0	0	0
0	0	0	0	0	0	0	0
0	0	0	0	0	0	0	0
0	0	0	0	0	0	0	0
1	1	0	1	0	0	0	0
0	0	0	0	0	0	0	0
0	0	0	0	0	0	0	0
1	1	1	1	1	1	0	0
0	0	0	0	0	0	0	0
0	0	0	0	0	0	0	0
0	0	0	1	0	0	0	0
0	0	0	0	0	0	0	0
0	0	0	0	0	0	0	0
0	0	0	0	0	0	0	0
1	0	1	1	0	0	0	0
0	0	0	0	0	0	0	0
1	1	0	1	1	0	0	0
0	0	0	0	0	0	0	0
0	0	0	0	0	0	0	0
0	0	0	0	0	0	0	0
0	0	0	1	0	0	0	1
0	0	0	0	0	0	0	0
0	0	0	0	0	0	0	0
1	0	0	0	1	0	0	0
0	0	0	0	0	0	0	0
0	0	0	0	0	0	0	0
0	0	0	0	0	0	0	0
0	0	0	0	0	0	0	0
0	0	0	0	0	0	0	0

flag.S.a.GN	flag.VancA	flag.IgAN E	flag.S.a.GN	flag.VancA	flag.S.a.GN	flag.VancA	flag.VancATN.vs.S.a.G
0	0	0	0	0	0	0	0
0	0	0	0	0	0	0	0
0	0	0	0	0	0	0	0
0	0	0	0	0	0	0	0
0	0	0	0	0	0	0	0
0	0	0	0	0	0	0	0
0	0	0	0	0	0	0	0
0	0	0	0	0	0	0	0
0	0	0	0	0	0	0	0
0	0	0	0	0	0	0	0
0	0	0	0	0	0	0	0
0	0	0	0	0	0	0	0
0	0	0	0	0	0	0	0
0	0	0	0	0	0	0	0
0	0	0	0	0	0	0	0
0	0	0	0	0	0	0	0
0	0	0	0	0	0	0	0
0	0	0	0	0	0	0	0
0	0	0	0	0	0	0	0
0	0	0	0	0	0	0	0
0	0	0	0	0	0	0	0
0	0	0	0	0	0	0	0
0	0	0	0	0	0	0	0
0	0	0	0	0	0	0	0
0	0	0	0	0	0	0	0
0	0	0	0	0	0	0	0
0	0	0	0	0	0	0	0
0	0	0	0	0	0	0	0
0	0	0	0	0	0	0	0
0	0	0	0	0	0	0	0
0	0	0	0	0	0	0	0
0	0	0	0	0	0	0	0
0	0	0	0	0	0	0	0
0	0	0	0	0	0	0	0
0	0	0	0	0	0	0	0
0	0	0	0	0	0	0	0
0	0	0	0	0	0	0	0
0	0	0	0	1	0	0	1
0	0	0	0	0	0	0	0
0	0	0	0	0	0	0	0
0	0	0	0	0	0	0	0

0	0	0	0	0	0	0	0
0	0	0	0	0	0	0	0
0	0	0	0	0	0	0	0
0	0	0	0	0	0	0	0
0	0	0	0	0	0	0	0
0	0	0	0	0	0	0	0
0	0	0	0	0	0	0	0
0	0	0	0	0	0	0	0
0	0	0	0	0	0	0	0
0	0	0	0	0	0	0	0
0	0	0	0	0	0	0	0
0	0	0	0	0	0	0	0
0	0	0	0	0	0	0	1
0	0	0	0	0	0	0	0
0	0	0	0	0	0	0	0
0	0	0	0	0	0	0	0
0	0	0	0	0	0	0	0
0	0	0	0	0	0	0	0
0	0	0	0	0	0	0	0
0	0	0	0	0	0	0	0
0	0	0	0	0	0	0	0
0	0	0	0	0	0	0	0
0	0	0	0	0	0	0	0
0	0	0	0	0	0	0	0
0	0	0	0	0	0	0	0
0	0	0	0	0	0	0	0
0	0	0	0	0	0	0	0
0	0	0	0	0	0	0	0
0	0	0	0	0	0	0	0
0	0	0	0	0	0	0	0
0	0	0	0	0	0	0	0
0	0	0	0	0	0	0	0
0	0	0	0	0	0	0	0
0	0	0	0	0	0	0	0
0	0	0	0	0	0	0	0
0	0	0	0	1	0	1	1
0	0	0	0	0	0	0	0
0	0	0	0	0	0	0	0
0	0	0	0	0	0	0	0
0	0	0	0	0	0	0	0
0	0	0	0	0	0	0	0
0	0	0	0	0	0	1	0
0	0	0	0	0	0	0	0
0	0	0	0	0	0	0	0
0	0	0	0	0	0	0	0
0	0	0	0	0	0	0	0
0	0	0	0	0	0	0	0

0	0	0	0	0	0	0	0
0	0	0	0	0	0	0	0
0	0	0	0	0	0	0	0
0	0	0	0	0	0	0	0
0	0	0	0	0	0	0	0
0	0	0	0	0	0	0	0
0	0	0	0	0	0	0	0
0	0	0	0	0	0	0	0
0	0	0	0	0	0	0	0
0	0	0	0	0	0	0	0
0	0	0	0	0	0	0	0
0	0	0	0	0	0	0	0
0	0	0	0	0	0	0	0
0	0	0	0	0	0	0	0
0	0	0	0	0	0	0	0
0	0	0	0	0	0	0	0
0	0	0	0	0	0	0	0
0	0	0	0	0	0	0	0
0	0	0	0	0	0	0	0
0	0	0	0	0	0	0	0
0	0	0	0	0	0	0	0
0	0	0	0	0	0	0	0
0	0	0	0	0	0	0	0
0	0	0	0	0	0	0	0
0	0	0	0	0	0	0	0
0	0	0	0	0	0	0	0
0	0	0	0	0	0	0	0
0	0	0	0	0	0	0	0
0	0	0	0	1	0	0	1
0	0	0	0	0	0	0	0
0	0	0	0	0	0	0	0
0	0	0	0	0	0	0	0
0	0	0	0	0	0	0	0
0	0	0	0	0	0	0	0
0	0	0	0	0	0	0	0
0	0	0	0	0	0	0	0
0	0	0	0	0	0	0	0
0	0	0	0	0	0	0	0
0	0	0	0	0	0	0	0
0	0	0	0	0	0	0	0
1	0	0	0	0	0	0	1
0	0	0	0	0	0	0	0
0	0	0	0	0	0	0	0
0	0	0	0	0	0	0	0
0	0	0	0	0	0	0	0
0	0	0	0	0	0	0	0
0	0	0	0	0	0	0	0

0	0	0	0	0	0	0	0
0	0	0	0	0	0	0	0
0	0	0	0	0	0	0	0
0	0	0	0	0	0	0	0
0	0	0	0	0	0	0	0
0	0	0	0	0	0	0	0
0	0	0	0	0	0	0	0
0	0	0	0	0	0	0	0
0	0	0	0	0	0	0	0
0	0	0	0	0	0	0	0
0	0	0	0	0	0	0	0
0	0	0	0	0	0	0	0
0	0	0	0	0	0	0	0
0	0	0	0	0	0	0	0
0	0	0	0	0	0	0	0
0	0	0	0	0	0	0	0
0	0	0	0	0	0	0	0
0	0	0	0	0	0	0	0
0	0	0	0	0	0	0	0
0	0	0	0	0	0	0	0
0	0	0	0	0	0	0	0
0	0	0	0	0	0	0	0
0	0	0	0	0	0	0	0
0	0	0	0	0	0	0	0
0	0	0	0	0	0	0	0
0	1	0	0	0	0	0	0
0	0	0	1	1	0	0	0
0	0	0	0	0	0	0	0
0	0	0	0	0	0	0	0
0	0	0	0	0	0	0	0
0	0	0	0	0	0	0	0
0	0	0	0	0	0	0	0
0	0	0	0	0	0	0	0
0	0	0	0	0	0	0	0
0	0	0	0	0	0	0	0
0	0	0	0	1	0	0	0
0	0	0	0	0	0	0	0
0	0	0	0	0	0	0	0
0	0	0	0	0	0	0	0
0	0	0	0	0	0	0	0
0	0	0	0	0	0	0	0
0	0	0	0	0	0	0	0
0	0	0	0	0	0	0	0
0	0	0	0	0	0	0	0
0	0	0	0	0	0	0	0
0	0	0	0	0	0	0	0
0	0	0	0	0	0	0	0

0	0	0	0	0	0	0	0
0	0	0	0	0	0	0	0
0	0	0	0	0	0	0	0
0	0	0	0	0	0	0	0
0	0	0	0	0	0	0	0
0	0	0	0	0	0	0	0
0	0	0	0	1	0	0	0
0	0	0	0	0	0	0	0
0	0	0	0	0	0	0	0
0	0	0	0	0	0	0	0
0	0	0	0	0	0	0	0
0	0	0	0	0	0	0	0
0	0	0	0	0	0	0	0
0	0	0	0	0	0	0	0
0	0	0	0	0	0	0	0
0	0	0	0	0	0	0	0
0	0	0	0	0	0	0	0
0	0	0	0	0	0	0	0
0	0	0	0	0	0	0	0
0	0	0	0	0	0	0	0
0	0	0	0	0	0	0	0
0	0	0	0	0	0	0	0
0	0	0	0	0	0	0	0
0	0	0	0	0	0	0	0
0	0	0	0	0	0	0	0
0	0	0	0	0	0	0	0
0	0	0	0	0	0	0	0
0	0	0	0	0	0	0	0
0	0	0	0	0	0	0	0
0	0	0	0	0	0	0	0
0	0	0	0	0	0	0	0
0	0	0	0	0	0	0	0
0	0	0	0	0	0	0	0
0	0	0	0	0	0	0	0
0	0	0	0	0	0	0	0
0	0	0	0	0	0	0	0
0	0	0	0	0	0	0	0
0	0	0	0	0	0	0	0
0	0	0	0	0	0	0	0
0	1	0	0	0	0	1	1
0	0	0	0	0	0	0	0
0	0	0	0	0	0	0	0
0	0	0	0	0	0	0	0
0	0	0	0	0	0	0	0
0	0	0	0	0	0	0	0
0	0	0	0	0	0	0	0
0	0	0	0	0	0	0	0

0	0	0	0	0	0	0	0
0	0	0	0	0	0	0	0
0	0	0	0	0	0	0	0
0	0	0	0	0	0	0	0
0	0	0	0	0	0	0	0
1	0	0	0	1	0	0	1
0	0	0	0	0	0	0	0
0	0	0	0	0	0	0	0
0	0	0	0	0	0	0	0
0	0	0	0	0	0	0	0
0	0	0	0	0	0	0	0
0	0	0	0	0	0	0	0
0	0	0	0	0	0	0	0
0	0	0	0	0	0	0	0
0	0	0	0	0	0	0	0
0	0	0	0	0	0	0	0
0	0	0	0	0	0	0	0
0	0	0	0	0	0	0	0
0	0	0	0	0	0	0	0
0	0	0	0	0	0	0	0
0	0	0	0	0	0	0	0
0	0	0	0	0	0	0	0
0	0	0	0	0	0	0	0
0	0	0	0	0	0	0	0
0	0	0	0	0	0	0	1
0	0	0	0	0	0	0	0
0	0	0	0	0	0	0	0
0	0	0	0	0	0	0	0
0	0	0	0	0	0	0	0
0	0	0	0	0	0	0	0
0	0	0	0	0	0	0	0
0	0	0	0	0	0	0	0
0	0	0	0	0	0	0	0
0	0	0	0	0	0	0	0
0	0	0	0	0	0	0	0
0	0	0	0	0	0	0	0
0	0	0	0	0	0	0	1
0	0	0	0	0	0	0	0
0	0	0	0	0	0	0	0
1	0	0	0	0	0	0	1
0	0	0	0	0	0	0	0
0	0	0	0	0	0	0	1
0	0	0	0	0	0	0	0
0	0	0	0	0	0	0	0
0	0	0	0	0	0	0	0
0	0	0	0	0	0	0	0
0	0	0	0	0	0	0	0

0	0	0	0	0	0	0	0
0	0	0	0	0	0	0	0
0	0	0	0	0	0	0	0
0	0	0	0	0	0	0	0
1	0	0	0	0	0	0	1
0	0	0	0	0	0	0	0
0	0	0	0	0	0	0	1
0	0	0	0	1	0	0	0
0	0	0	0	0	0	0	0
0	0	0	0	0	0	0	0
0	1	0	0	0	0	0	0
0	0	0	0	0	0	0	0
0	0	0	0	0	0	0	0
0	0	0	0	0	0	0	0
0	0	0	0	0	0	0	0
0	0	0	0	0	0	0	0
0	0	0	0	0	0	0	0
0	0	0	0	0	0	0	0
0	0	0	0	0	0	0	0
0	0	0	0	0	0	0	0
0	0	0	0	0	0	0	0
0	0	0	0	0	0	0	0
0	0	0	0	0	0	0	0
0	0	0	0	0	0	0	0
0	0	0	0	0	0	0	0
0	0	0	0	0	0	0	0
0	0	0	0	0	0	0	0
0	0	0	0	0	0	0	0
0	0	0	0	0	0	0	0
0	0	0	0	0	0	0	0
0	0	0	0	0	0	0	0
0	0	0	0	0	0	0	0
0	0	0	0	0	0	0	0
0	0	0	0	0	0	0	0
1	0	0	0	0	0	1	1
0	0	0	0	0	0	0	0
0	0	0	0	0	0	0	0
0	0	0	0	0	0	1	0
0	0	0	0	0	0	0	0
0	0	0	0	0	0	0	0
0	0	0	0	0	0	0	0
0	0	0	0	0	0	0	0
0	0	0	0	0	0	0	0

N

order	Identified.I	Gene.Nam	Accession.I	Molecular.	ATN T24	ATN T25	ATN T26	ATN T27
1	Myosin-9	MYH9	P35579	227 kDa	42	55	91	91
2	Isoform 3 c	SPTAN1	Q13813-3	282 kDa	42	56	64	57
3	Actin. cyto	ACTG1	P63261	42 kDa	36	54	66	59
4	Low-densit	LRP2	P98164	522 kDa	56	56	57	59
5	ATP syntha	ATP5B	P06576	57 kDa	34	44	47	43
6	Basement i	HSPG2	P98160	469 kDa	43	46	53	49
7	Glycine am	GATM	P50440	48 kDa	18	29	20	18
8	Collagen al	COL6A3	P12111	344 kDa	40	45	67	72
9	Spectrin be	SPTBN1	Q01082	275 kDa	36	33	44	41
10	Glyceraldel	GAPDH	P04406	36 kDa	17	22	35	20
11	Vimentin	VIM	P08670	54 kDa	45	37	55	40
12	Isoform 2 c	KRT8	P05787-2	57 kDa	45	38	50	42
13	ATP syntha	ATP5A1	P25705	60 kDa	28	34	33	30
14	60 kDa hea	HSPD1	P10809	61 kDa	24	34	29	30
15	Aconitate h	ACO2	Q99798	85 kDa	29	35	30	27
16	Isoform 2 c	CLTC	Q00610-2	188 kDa	34	40	47	47
17	Isoform 2 c	FLNA	P21333-2	280 kDa	27	33	66	60
18	3-ketoacyl-	ACAA2	P42765	42 kDa	22	30	28	28
19	Moesin	MSN	P26038	68 kDa	26	35	35	38
20	Aminopept	ANP	P15144	110 kDa	24	37	27	26
21	Alpha-actir	ACTN4	O43707	105 kDa	28	35	51	40
22	Alpha-enol	ENO1	P06733	47 kDa	27	23	23	24
23	Phosphoen	PCK2	Q16822	71 kDa	17	21	14	16
24	Argininosu	ASS1	P00966	47 kDa	14	22	16	15
25	Fructose-bi	ALDOB	P05062	39 kDa	16	26	15	10
26	Isoform 4 c	ATP1A1	P05023-4	113 kDa	24	25	21	21
27	Glutamate	GLUD1	P00367	61 kDa	19	22	15	19
28	Methylmal	ALDH6A1	Q02252	58 kDa	17	27	21	22
29	Tubulin bet	TUBB	P07437	50 kDa	23	26	27	31
30	Aldehyde d	ALDH2	P05091	56 kDa	18	23	21	25
31	Delta-1-pyr	ALDH4A1	P30038	62 kDa	24	27	27	23
32	Acetyl-CoA	ACAT1	P24752	45 kDa	16	18	23	18
33	Trifunction	HADHA	P40939	83 kDa	19	20	18	17
34	Keratin. typ	KRT13	P13646	50 kDa	5	5	5	4
35	Keratin. typ	KRT18	P05783	48 kDa	33	22	28	30
36	Aminoacyla	ACY1	Q03154	46 kDa	10	27	12	17
37	78 kDa gluc	HSPA5	P11021	72 kDa	20	22	30	27
38	Prelamin-A	LMNA	P02545	74 kDa	20	22	25	25
39	Keratin. typ	KRT1	P04264	66 kDa	23	16	11	7
40	L-lactate de	LDHB	P07195	37 kDa	14	26	24	19
41	Acyl-coenz	ACSM2B	Q68CK6	64 kDa	17	20	19	18
42	Cytosolic n	CNDP2	Q96KP4	53 kDa	14	26	19	16
43	Keratin. typ	KRT10	P13645	59 kDa	25	23	17	6

44	Pyruvate ki	PKM	P14618	58 kDa	24	18	24	21
45	Isoform 2 c	MYH10	P35580-2	231 kDa	20	31	32	32
46	Peroxisoma	EHHADH	Q08426	79 kDa	18	16	9	16
47	Pyruvate ca	PC	P11498	130 kDa	10	20	5	10
48	Phosphogly	PGK1	P00558	45 kDa	10	19	21	22
49	Stress-70 p	HSPA9	P38646	74 kDa	16	22	15	16
50	Isoform 2 c	HSP90AA1	P07900-2	98 kDa	17	27	30	23
51	Heat shock	HSPA8	P11142	71 kDa	22	21	30	24
52	Isoform 2 c	FLNB	O75369-2	276 kDa	19	23	30	35
53	Apoptosis-i	AIFM1	O95831	67 kDa	11	16	14	13
54	Alpha-cryst	CRYAB	P02511	20 kDa	39	29	47	38
55	Serum albu	ALB	P02768	69 kDa	12	26	31	21
56	Tubulin alp	TUBA1C	F5H5D3	58 kDa	20	15	25	24
57	Major vault	MVP	Q14764	99 kDa	12	23	24	22
58	Talin-1	TLN1	Q9Y490	270 kDa	28	21	38	39
59	Isoform 1 c	VCL	P18206-2	117 kDa	14	21	39	30
60	Betaine--hc	BHMT	Q93088	45 kDa	11	14	10	10
61	Elongation	TUFM	P49411	50 kDa	11	13	12	14
62	Heat shock	HSPB1	P04792	23 kDa	14	14	17	17
63	Na(+)/H(+)	PDZK1	Q5T2W1	57 kDa	1	21	4	8
64	Ubiquitin-li	UBA1	P22314	118 kDa	8	22	30	18
65	Cubilin	CUBN	O60494	399 kDa	8	10	3	1
66	Annexin A6	ANXA6	P08133	76 kDa	8	16	22	18
67	V-type prot	ATP6V1A	P38606	68 kDa	11	15	9	13
68	Endoplasm	HSP90B1	P14625	92 kDa	13	22	24	24
69	Catalase	CAT	P04040	60 kDa	8	14	5	8
70	Enoyl-CoA	ECHS1	P30084	31 kDa	8	17	13	11
71	Compleme	C3	P01024	187 kDa	12	21	42	22
72	2-oxoglutaı	OGDH	Q02218	116 kDa	9	18	10	13
73	Very long-c	ACADVL	P49748	70 kDa	19	25	13	21
74	Histone H4	HIST1H4A	P62805	11 kDa	12	13	20	19
75	Cytoplasmi	ACO1	P21399	98 kDa	12	20	13	12
76	Protein dis	PDIA3	P30101	57 kDa	8	10	17	14
77	Transitiona	VCP	P55072	89 kDa	11	21	22	19
78	14-3-3 prot	YWHAE	P62258	29 kDa	9	15	12	12
79	Heterogen	HNRNPA2B	P22626	37 kDa	15	10	14	13
80	Isocitrate d	IDH2	P48735	51 kDa	17	15	13	14
81	Glutathion	GSTA2	P09210	26 kDa	6	8	8	9
82	Phosphatid	BP1	P30086	21 kDa	7	12	8	12
83	Bifunctiona	DAK	Q3LXA3	59 kDa	2	21	8	8
84	Trifunction	HADHB	P55084	51 kDa	11	15	14	11
85	Succinyl-Cc	SUCLG2	Q96I99	47 kDa	11	14	14	13
86	Annexin A4	ANXA4	P09525	36 kDa	14	18	20	21
87	Neuroblast	AHNAK	Q09666	629 kDa	9	14	27	28
88	Alcohol del	AKR1A1	P14550	37 kDa	12	11	11	13
89	Glycine N-a	GLYAT	Q6IB77	34 kDa	6	12	7	11
90	Succinate c	SDHA	P31040	73 kDa	7	16	7	10

91	Retinal deh	ALDH1A1	P00352	55 kDa	12	17	25	13
92	V-type prot	ATP6V1B2	P21281	57 kDa	11	12	10	8
93	Cytochrom	UQCRC2	P22695	48 kDa	10	17	13	12
94	Hemoglobi	HBB	P68871	16 kDa	12	14	22	15
95	Keratin. typ	KRT9	P35527	62 kDa	8	7	6	1
96	Isoform 2 c	ACADM	P11310-2	47 kDa	9	12	10	12
97	Delta(3.5)-I	ECH1	Q13011	36 kDa	8	12	6	11
98	Neprilysin	MME	P08473	86 kDa	4	22	2	9
99	Isoform 2 c	TPI1	P60174-1	27 kDa	7	16	14	15
100	Leucine-ric	LRPPRC	P42704	158 kDa	13	15	11	19
101	Laminin sul	LAMA5	O15230	400 kDa	21	9	20	17
102	Isoform 2 c	SHMT1	P34896-2	49 kDa	7	14	8	10
103	Peroxiredo	PRDX1	Q06830	22 kDa	8	9	10	9
104	Heterogen	HNRNPK	P61978	51 kDa	23	14	20	18
105	Isoform 3 c	ALDH1L1	O75891-3	100 kDa	6	24	7	7
106	Isoform 2 c	PCCA	P05165-2	77 kDa	5	12	6	6
107	Propionyl-C	PCCB	C9JQS9	61 kDa	18	17	12	14
108	Hemoglobi	HBA1	P69905	15 kDa	7	9	20	11
109	Isoform 3 c	PLEC	Q15149-3	518 kDa	30	5	28	22
110	Heat shock	HSPA1A	P08107	70 kDa	11	17	19	14
111	Dimethylgl	DMGDH	Q9UI17	97 kDa	5	12	2	8
112	Malate deh	MDH2	P40926	36 kDa	9	12	10	11
113	Methylcrot	MCCC2	Q9HCC0	61 kDa	8	17	9	5
114	Peptidyl-pr	PPIA	P62937	18 kDa	8	9	11	12
115	Isoform 2 c	ETFA	P13804-2	30 kDa	12	15	10	11
116	Na(+)/H(+)	SLC9A3R1	O14745	39 kDa	9	12	8	6
117	Aldehyde d	ALDH1B1	P30837	57 kDa	10	17	11	13
118	Actin. alpha	ACTC1	P68032	42 kDa	25	38	50	53
119	Isoform 2 c	TKT	P29401-2	69 kDa	7	14	14	12
120	Ezrin	EZR	P15311	69 kDa	15	22	24	21
121	Calmodulin	CALM2	E7EMB3	22 kDa	8	8	7	8
122	Histone H2	HIST2H2AA	Q6FI13	14 kDa	7	12	15	12
123	Collagen al	COL18A1	P39060	178 kDa	11	13	15	10
124	Isoform 2 c	HADH	Q16836-2	42 kDa	5	11	7	11
125	Acyl-coenz	ACOT1	Q86TX2	46 kDa	7	12	10	8
126	Fumarate h	FH	P07954	55 kDa	7	8	10	12
127	Keratin. typ	KRT2	P35908	65 kDa	34	20	11	6
128	Fructose-1.	FBP1	P09467	37 kDa	2	7	3	5
129	Isoform 2 c	NDUFS1	P28331-2	81 kDa	11	14	5	12
130	Cytoplasmi	DYNC1H1	Q14204	532 kDa	16	14	28	15
131	Adenylate I	AK4	P27144	25 kDa	7	11	9	10
132	Rab GDP di	GDI2	E7EU23	51 kDa	10	16	14	12
133	Aromatic-L	DDC	P20711	54 kDa	2	6	6	2
134	Glutathion	GSTP1	P09211	23 kDa	7	10	9	11
135	Annexin A5	ANXA5	P08758	36 kDa	12	14	14	13
136	Isoform 2 c	ANXA2	P07355-2	40 kDa	13	9	15	23
137	Isoform 5 c	CALD1	Q05682-5	61 kDa	6	13	13	16

138	NAD(P) tra	NNT	Q13423	114 kDa	7	8	9	9
139	3-hydroxyi	HIBCH	Q6NVY1	43 kDa	5	7	8	8
140	3-hydroxya	HSD17B10	Q99714	27 kDa	10	8	11	11
141	Isoform 17	FN1	P02751-17	256 kDa	19	5	39	33
142	Isoform 2 c	ALDH7A1	P49419-2	55 kDa	3	9	9	6
143	Heat shock	HSP90AB1	P08238	83 kDa	14	22	23	22
144	Long-chain	ACSL1	B7Z452	78 kDa	9	10	7	10
145	Elongation	EEF1A1	P68104	50 kDa	11	10	12	13
146	Isoform 2 c	SLC4A4	Q9Y6R1-2	116 kDa	3	12	4	5
147	Choline de	CHDH	Q8NE62	65 kDa	3	8	4	6
148	Acyl-CoA s	ACSF2	B4DHT5	67 kDa	6	12	9	9
149	Isoform 3 c	TPM1	P09493-3	33 kDa	4	8	15	12
150	Peroxiredo	PRDX6	P30041	25 kDa	6	8	6	8
151	Neutral alp	GANAB	Q14697	107 kDa	7	14	13	17
152	Peroxiredo	PRDX2	P32119	22 kDa	12	12	10	10
153	Prohibitin	PHB	P35232	30 kDa	10	10	6	7
154	Prohibitin-2	PHB2	Q99623	33 kDa	9	13	11	10
155	Ubiquitin-4	RPS27A	P62979	18 kDa	7	8	6	6
156	ADP/ATP tr	SLC25A5	P05141	33 kDa	10	12	9	13
157	Isoform 4 c	TNC	P24821-4	231 kDa	13	1	33	25
158	Carbonyl re	CBR1	P16152	30 kDa	8	12	13	10
159	4-aminobu	ABAT	P80404	56 kDa	4	11	3	7
160	Keratin. typ	KRT19	P08727	44 kDa	24	22	23	15
161	Quinone o	CRYZ	Q08257	35 kDa	8	14	6	7
162	Formimido	FTCD	O95954	59 kDa	5	9	4	3
163	Cytochrom	UQCRC1	P31930	53 kDa	8	8	7	6
164	Isoform 2 c	AK2	P54819-2	26 kDa	5	6	5	7
165	Glucose-6-	GPI	P06744	63 kDa	11	12	14	11
166	Isoform 2 c	GSN	P06396-2	81 kDa	5	9	16	16
167	Villin-1	VIL1	P09327	93 kDa	2	17	3	10
168	Aflatoxin B	AKR7A3	O95154	37 kDa	2	15	6	4
169	Dipeptidyl	DPP4	P27487	88 kDa	7	10	7	9
170	Collagen al	COL6A1	P12109	109 kDa	9	13	11	14
171	Isoform 2 c	PDHB	P11177-2	37 kDa	11	10	10	10
172	T-complex	CCT2	P78371	57 kDa	10	11	15	11
173	3-ketoacyl-	ACAA1	P09110	44 kDa	6	11	5	8
174	Tubulin bet	TUBB4B	P68371	50 kDa	21	27	29	28
175	Isoform 2 c	TPM3	P06753-2	29 kDa	7	8	11	11
176	Isocitrate d	IDH1	O75874	47 kDa	4	11	10	5
177	Aspartate a	GOT2	P00505	48 kDa	9	9	8	9
178	Protein dis	P4HB	P07237	57 kDa	5	6	12	9
179	Adenosylho	AHCY	P23526	48 kDa	5	10	10	9
180	Calreticulin	CALR	P27797	48 kDa	2	6	12	13
181	Isoform 2 c	LAP3	P28838-2	53 kDa	4	9	8	7
182	Histone H2	HIST1H2BC	P23527	14 kDa	5	6	6	8
183	Protein dis	PDIA6	F8WA83	49 kDa	9	12	17	16
184	Laminin su	LAMC1	P11047	178 kDa	13	9	10	15

185	Dipeptidas	DP1	P16444	46 kDa	3	11	2	3
186	Aspartate a	GOT1	P17174	46 kDa	5	13	7	5
187	Carbonic ar	CA2	P00918	29 kDa	2	5	5	6
188	Isoform 2 c	ETFB	P38117-2	38 kDa	7	7	8	6
189	L-xylulose r	DCXR	Q7Z4W1	26 kDa	3	9	5	7
190	Isoform 2 c	IMMT	Q16891-2	83 kDa	7	7	5	8
191	Glycerol-3-	GPD1	P21695	38 kDa	2	9	3	2
192	Dihydropyr	DPYS	Q14117	57 kDa	4	11	5	4
193	Amine oxid	MAOB	P27338	59 kDa	6	7	6	7
194	Keratin. typ	KRT6C	P48668	60 kDa	12	9	0	7
195	Elongation	EEF2	P13639	95 kDa	10	11	10	9
196	Agmatinase	AGMAT	Q9BSE5	38 kDa	3	5	4	2
197	Prostagland	PTGR1	Q14914	36 kDa	7	9	5	8
198	UDP-glucur	UGT2B7	P16662	61 kDa	7	5	2	2
199	Thiosulfate	TST	Q16762	33 kDa	6	10	6	3
200	Isoform 2 c	ALDOA	P04075-2	45 kDa	5	7	11	10
201	Glutathione	GPX3	P22352	26 kDa	4	7	6	4
202	Ferritin ligh	FTL	P02792	20 kDa	5	10	4	6
203	Transgelin-	TAGLN2	P37802	22 kDa	12	9	12	9
204	6-phosphof	PFKL	P17858	85 kDa	7	10	6	8
205	Heterogene	HNRNPU	Q00839	91 kDa	8	6	14	11
206	Collagen al	COL4A2	P08572	168 kDa	5	7	6	8
207	Cathepsin I	CTSD	P07339	45 kDa	7	10	8	10
208	Cadherin-1	CDH16	O75309	90 kDa	7	5	3	2
209	Dihydrolipo	DLST	P36957	49 kDa	9	9	11	8
210	Heterogene	HNRNPA1	F8W6I7	33 kDa	8	6	12	13
211	Dihydropyr	DPYSL2	Q16555	62 kDa	4	7	11	12
212	Nucleopho	NPM1	P06748	33 kDa	6	2	9	8
213	3-hydroxyis	HIBADH	P31937	35 kDa	5	7	6	7
214	Isoform 2 c	SLC25A13	Q9UJS0-2	74 kDa	7	10	10	9
215	Alpha-1-an	SERPINA1	P01009	47 kDa	2	15	25	8
216	Protein DJ-	PARK7	Q99497	20 kDa	4	9	6	7
217	Isoform C c	KHK	P50053-2	33 kDa	1	3	2	5
218	Ig gamma-1	IGHG1	P01857	36 kDa	1	8	8	14
219	Dihydrolipo	DLD	P09622	54 kDa	7	10	7	8
220	Isoform Sh	YWHAB	P31946-2	28 kDa	5	9	12	8
221	X-ray repai	XRCC6	P12956	70 kDa	7	10	7	15
222	Glutaminas	GLS	O94925	73 kDa	5	8	7	6
223	Short/bran	ACADSB	P45954	47 kDa	5	10	2	5
224	ATP syntha	ATP5O	P48047	23 kDa	7	9	9	7
225	Short-chair	ACADS	P16219	44 kDa	6	8	9	5
226	Bifunctiona	EPHX2	P34913	63 kDa	2	12	4	3
227	Phosphoen	PCK1	P35558	69 kDa	9	29	2	2
228	Gamma-bu	BBOX1	O75936	45 kDa	3	13	7	6
229	UDP-glucur	UGT1A6	P19224	61 kDa	5	7	7	6
230	Catenin alp	CTNNA1	P35221	100 kDa	6	13	9	11
231	V-type prot	ATP6V1E1	P36543	26 kDa	6	8	6	5

232	Isoform Sm	MYL6	P60660-2	17 kDa	5	8	8	9
233	Amine oxid	MAOA	P21397	60 kDa	5	9	11	8
234	Nucleolin	NCL	P19338	77 kDa	7	7	7	6
235	Isoform 2 c	ATP5H	O75947-2	16 kDa	2	6	6	8
236	Lambda-cry	CRYL1	Q9Y2S2	35 kDa	2	10	6	3
237	3-hydroxyb	BDH2	Q9BUT1	27 kDa	2	6	3	4
238	Isoform 3 c	LDHA	P00338-3	40 kDa	4	13	13	13
239	2.4-dienoyl	DECR1	B7Z6B8	35 kDa	5	5	3	5
240	Ester hydro	C11orf54	Q9H0W9	35 kDa	4	5	3	5
241	Heterogen	HNRNPM	P52272	78 kDa	5	7	9	11
242	Peroxisoma	HSD17B4	P51659	80 kDa	10	10	8	8
243	Phosphogly	PGAM1	P18669	29 kDa	0	5	14	8
244	Maltase-glu	MGAM	E7ER45	312 kDa	1	18	0	0
245	10 kDa hea	HS1	P61604	11 kDa	3	5	5	4
246	Isoform 2 c	ACTN1	P12814-2	103 kDa	21	17	35	30
247	ES1 protein	C21orf33	P30042	28 kDa	5	6	4	5
248	C-1-tetrahy	MTHFD1	P11586	102 kDa	5	5	4	7
249	Isoform 2 c	FGA	P02671-2	70 kDa	8	3	14	12
250	Aldo-keto r	AKR1C3	P42330	37 kDa	5	5	7	6
251	T-complex	TCP1	P17987	60 kDa	5	6	11	9
252	Isoform B c	EPB41L3	Q9Y2J2-2	97 kDa	2	6	1	2
253	Sideroflexi	SFXN1	Q9H9B4	36 kDa	2	6	4	5
254	Profilin-1	PFN1	P07737	15 kDa	7	7	7	8
255	Purine nucl	PNP	P00491	32 kDa	3	7	3	4
257	Isoform 4 c	SOD2	P04179-4	20 kDa	7	7	13	15
258	Ras GTPase	IQGAP1	P46940	189 kDa	6	11	19	21
259	Isoform 3 c	MDH1	P40925-3	39 kDa	3	10	6	5
260	Glyoxylate	GRHPR	Q9UBQ7	36 kDa	7	9	4	4
261	Alpha-meth	AMACR	Q9UHK6	42 kDa	4	7	8	7
262	Cytochrom	MT-CO2	P00403	26 kDa	5	5	6	5
263	14-3-3 prot	YWHAZ	P63104	28 kDa	7	9	14	7
264	Histone H1	HIST1H1E	P10412	22 kDa	5	7	6	5
265	Selenium-b	SELENBP1	A6PVW9	57 kDa	2	4	1	5
266	Transgelin	TAGLN	Q01995	23 kDa	5	10	11	14
267	Isoform 3 c	DAB2	P98082-3	80 kDa	4	10	6	4
268	Probable p	PRODH2	Q9UF12	59 kDa	3	4	2	3
269	Isoform 3 c	CYB5R3	P00387-3	38 kDa	10	8	8	7
271	Elongation	EEF1G	P26641	50 kDa	8	7	9	11
272	Creatine ki	CKB	P12277	43 kDa	7	10	7	9
273	Histone H3	H3F3A	P84243	15 kDa	3	4	5	3
274	Phenazine	PBLD	P30039	32 kDa	1	8	6	3
275	Annexin A1	ANXA1	P04083	39 kDa	5	12	9	10
276	Ig kappa ch	IGKC	P01834	12 kDa	3	9	6	9
277	Isoform 3 c	SLC3A2	P08195-3	62 kDa	3	6	3	2
278	Lon protea	LONP1	K7EKE6	95 kDa	5	10	7	5
279	Collagen al	COL6A2	P12110	109 kDa	6	6	13	15
280	Succinyl-Co	SUCLG1	P53597	36 kDa	4	4	3	4

281	4-trimethyl	ALDH9A1	P49189	54 kDa	5	6	4	3
282	Tropomyos	TPM4	P67936	29 kDa	8	12	14	13
283	Heterogen	HNRNPF	P52597	46 kDa	5	4	8	8
284	ADP-ribosy	ARF1	P84077	21 kDa	7	9	9	7
285	Citrate syn	CS	O75390	52 kDa	6	8	6	8
286	Enoyl-CoA	ECHDC3	Q96DC8	33 kDa	7	8	1	7
287	Macrophag	CAPG	P40121	38 kDa	3	7	7	6
288	Peroxiredo	PRDX5	P30044	22 kDa	5	5	5	4
289	Cytochrom	CYB5A	P00167	15 kDa	3	4	3	5
290	Carnitine O	CPT2	P23786	74 kDa	5	10	6	8
291	Collagen al	COL1A1	P02452	139 kDa	4	3	2	3
292	4-hydroxy-	HOGA1	Q86XE5	35 kDa	2	5	4	4
293	Isoform 2 c	ISOC2	Q96AB3-2	24 kDa	2	5	4	7
294	Voltage-de	VDAC1	P21796	31 kDa	6	7	9	6
295	Splicing fac	SFPQ	P23246	76 kDa	8	5	6	8
296	Cytochrom	COX5A	P20674	17 kDa	3	4	6	4
297	40S ribosor	RPS3	P23396	27 kDa	6	6	7	9
298	Myosin reg	MYL12B	O14950	20 kDa	6	10	9	8
299	Cytochrom	COX4I1	P13073	20 kDa	3	4	3	4
300	Eukaryotic	EIF4A1	P60842	46 kDa	9	8	6	9
301	2-oxoglutai	OGDHL	Q9ULD0	114 kDa	9	7	8	6
302	Unconvent	MYO6	E7EW20	149 kDa	7	6	4	4
303	Dimethylar	FMO1	Q01740	60 kDa	2	7	6	8
304	Isoform 2 c	ACOX1	Q15067-2	75 kDa	1	5	0	3
305	Dolichyl-di	RPN1	P04843	69 kDa	4	6	7	7
306	Mitochond	MTCH2	Q9Y6C9	33 kDa	3	8	3	6
307	ATP syntha	ATP5C1	P36542	33 kDa	4	6	3	6
308	Tubulointe	TINAGL1	Q9GZM7	52 kDa	3	5	6	6
309	Lamin-B2	LMNB2	Q03252	68 kDa	6	7	6	7
310	Fumarylace	FAH	P16930	46 kDa	3	9	3	3
311	Tubulointe	TINAG	Q9UJW2	55 kDa	5	6	6	5
312	Keratin. typ	KRT7	P08729	51 kDa	25	16	18	16
313	Sarcosine c	SARDH	Q9UL12	101 kDa	2	2	1	2
314	Protein Nip	NIPSNAP1	Q9BPW8	33 kDa	2	2	2	3
315	Isoform 2 c	NID1	P14543-2	122 kDa	5	5	8	6
316	Isoform 1 c	RRBP1	Q9P2E9-2	152 kDa	4	3	12	12
317	Compleme	C1QBP	Q07021	31 kDa	5	6	8	8
318	Biglycan	BGN	P21810	42 kDa	5	1	6	9
319	Aflatoxin B	AKR7A2	O43488	40 kDa	3	5	4	5
320	Guanine de	GDA	Q9Y2T3	51 kDa	1	6	6	4
321	Synaptic ve	VAT1	Q99536	42 kDa	4	5	7	8
322	Calnexin	CANX	P27824	68 kDa	6	5	8	10
323	Cofilin-1	CFL1	P23528	19 kDa	3	6	7	6
324	Isoform L-t	PKLR	P30613-2	58 kDa	1	1	1	3
325	Retinol-bin	RBP4	P02753	23 kDa	3	3	5	6
326	Acyl-coenz	ACSM2A	Q08AH3	64 kDa	17	19	17	21
327	Heterogen	HNRNPC	B4DY08	32 kDa	9	7	8	8

328	EMILIN-1	EMILIN1	Q9Y6C2	107 kDa	7	8	13	14
329	Transthyre	TTR	P02766	16 kDa	0	0	3	2
330	Carboxyme	CMBL	Q96DG6	28 kDa	2	6	2	2
331	40S ribosor	RPSA	C9J9K3	30 kDa	6	3	7	9
332	ATP-depen	DHX9	Q08211	141 kDa	9	8	11	11
333	Isovaleryl-(	IVD	P26440	46 kDa	2	5	4	4
334	LIM and SH	LASP1	Q14847	30 kDa	2	5	6	4
335	Glycine del	GLDC	P23378	113 kDa	2	0	5	5
336	6-phospho	PGD	P52209	53 kDa	7	5	9	7
337	Ras-relatec	RAB7A	P51149	23 kDa	4	5	4	6
338	T-complex	CCT6A	P40227	58 kDa	4	5	8	6
339	Apolipopro	APOA1	P02647	31 kDa	5	3	10	6
340	UTP--gluco	UGP2	Q16851	57 kDa	5	3	7	4
341	Probable N	NAT8	Q9UHE5	26 kDa	2	3	3	3
342	Ketimine re	CRYM	Q14894	34 kDa	1	9	3	1
343	Compleme	C4B	P0C0L5	193 kDa	6	1	11	6
344	Heterogen	HNRNPA3	P51991	40 kDa	9	8	9	7
345	Poly(rC)-bi	PCBP1	Q15365	37 kDa	2	4	5	6
346	NADH deh	NDUFS3	O75489	30 kDa	8	5	4	4
347	Collagen al	COL4A1	P02462	161 kDa	2	3	4	3
348	Epoxide hy	EPHX1	P07099	53 kDa	2	6	2	4
349	Laminin sul	LAMB1	P07942	198 kDa	6	7	9	10
350	Adenylyl cy	CAP1	Q01518	52 kDa	5	3	7	5
351	NADH deh	NDUFV1	P49821	51 kDa	9	5	4	6
352	WD repeat	WDR1	O75083	66 kDa	4	2	5	6
353	3-mercapt	MPST	P25325	33 kDa	2	4	3	4
354	Fatty acid-l	FABP1	P07148	14 kDa	4	4	2	1
355	Isoform 2 c	RAB5C	P51148-2	27 kDa	8	5	7	6
356	Isoform 2 c	PDHA1	P08559-2	44 kDa	4	3	5	5
357	Protein-glu	TGM2	P21980	77 kDa	5	5	7	11
358	Sepiapterir	SPR	P35270	28 kDa	6	9	4	6
359	Sorbitol de	SORD	Q00796	38 kDa	4	4	4	4
360	Isoform No	CLTA	P09496-2	24 kDa	3	5	5	5
361	ATP syntha	ATP5F1	P24539	29 kDa	2	7	6	8
362	X-ray repai	XRCC5	P13010	83 kDa	2	4	6	8
363	Methylcrot	MCCC1	Q96RQ3	80 kDa	2	2	2	1
364	Collagen al	COL1A2	P08123	129 kDa	4	2	4	4
365	Cytochrom	UQCRFS1	P47985	30 kDa	7	7	6	6
366	Gamma-glu	GGT1	P19440	61 kDa	4	9	2	4
367	Isoform 2 c	ALDH5A1	P51649-2	59 kDa	2	8	6	4
368	Isoform 2 c	ECI2	O75521-2	40 kDa	1	3	0	1
369	Keratin. typ	KRT14	P02533	52 kDa	7	6	5	3
370	Collagen al	COL12A1	D6RGG3	333 kDa	7	1	3	7
371	Cytochrom	CYCS	P99999	12 kDa	2	4	5	3
372	Protein AN	AMBP	P02760	39 kDa	4	3	2	6
373	Chloride in	CLIC4	Q9Y696	29 kDa	1	7	3	3
374	Apolipopro	APOA4	P06727	45 kDa	0	0	8	3

375	Isoform 2 c	PTBP1	P26599-2	59 kDa	7	4	9	11
376	T-complex	CCT5	P48643	60 kDa	5	2	6	5
377	Cathepsin I	CTSB	P07858	38 kDa	2	6	3	4
378	Non-POU d	NONO	Q15233	54 kDa	6	6	9	11
379	Ferritin hea	FTH1	P02794	21 kDa	4	5	3	4
380	Fatty acid-l	FABP3	P05413	15 kDa	0	6	4	2
381	Stomatin-li	STOML2	Q9UJZ1	39 kDa	5	5	5	5
382	Transformi	TGFBI	Q15582	75 kDa	4	3	8	6
383	Protein disı	PDIA4	P13667	73 kDa	5	7	8	6
384	Lysozyme (	LYZ	P61626	17 kDa	5	2	4	3
385	Peptidyl-pr	PPIB	P23284	24 kDa	6	4	7	8
386	Alpha/beta	ABHD14B	Q96IU4	22 kDa	2	5	3	5
387	NADH dehy	NDUFA9	Q16795	43 kDa	5	4	4	5
388	T-complex	CCT3	B4DUR8	56 kDa	1	5	5	2
389	Heat shock	TRAP1	F5H897	74 kDa	6	7	5	2
390	Very long-c	SLC27A2	O14975	70 kDa	3	14	2	5
391	Isoform 2 c	PRDX3	P30048-2	26 kDa	2	5	4	3
392	Prenylcyste	PCYOX1	Q9UHG3	57 kDa	3	4	2	4
393	Staphyloco	SND1	Q7KZF4	102 kDa	2	4	8	9
394	Nicotinate	NAPRT1	G5E977	62 kDa	0	4	0	0
395	Catenin be	CTNNB1	B4DGU4	85 kDa	4	7	4	5
396	Mitochond	SLC25A10	B4DLN1	48 kDa	1	6	2	2
397	Mitochond	2-Mar	Q969Z3	38 kDa	3	6	5	4
398	Acylpyruva	FAHD1	Q6P587	25 kDa	3	5	4	3
399	Thrombosp	THBS1	P07996	129 kDa	7	3	18	14
400	Sideroflexi	SFXN2	Q96NB2	36 kDa	2	2	1	1
401	Cocaine est	CES2	O00748	62 kDa	0	2	2	1
402	Isoform 2 c	ALDH3A2	P51648-2	58 kDa	2	4	3	1
403	Isoform 2 c	HPD	P32754-2	40 kDa	1	6	0	0
405	S-formylglu	ESD	P10768	31 kDa	1	5	3	3
406	N(G).N(G)-c	DDAH1	O94760	31 kDa	0	1	2	2
407	V-type prot	ATP6V1H	Q9UI12	56 kDa	2	5	3	1
408	Matrin-3	MATR3	P43243	95 kDa	6	2	5	9
409	Isoform 2 c	GSR	P00390-3	53 kDa	5	4	5	4
410	Clusterin	CLU	P10909	52 kDa	2	4	6	5
411	Isoform 2 c	EEF1D	P29692-2	71 kDa	6	5	6	6
412	UMP-CMP	CMPK1	P30085	22 kDa	1	8	6	4
413	Dihydropte	QDPR	P09417	26 kDa	3	3	0	0
414	Keratin. typ	KRT5	P13647	62 kDa	13	7	0	0
415	Isoform 1 c	VDAC2	P45880-1	33 kDa	3	1	2	3
416	Heterogen	HNRNPL	P14866	64 kDa	5	3	7	9
417	Glutaryl-Co	GCDH	Q92947	48 kDa	3	3	3	4
418	Ribonuclea	HRSP12	P52758	14 kDa	6	5	4	4
419	T-complex	CCT8	P50990	60 kDa	2	4	4	5
420	Isoform B c	SLC25A3	Q00325-2	40 kDa	5	3	4	4
421	Fibrinogen	FGB	P02675	56 kDa	6	2	11	5
422	Corticoster	HSD11B2	P80365	44 kDa	4	4	2	1

423	Hydroxyste	HSDL2	Q6YN16	45 kDa	4	6	2	4
424	Isoform 2 c	AOC1	P19801-2	87 kDa	0	0	0	0
425	Calbindin	CALB1	P05937	30 kDa	1	6	4	1
426	Rho GDP-d	ARHGDIA	P52565	23 kDa	2	3	4	4
427	Aldose 1-e	GALM	Q96C23	38 kDa	1	4	4	1
428	Isoform 3 c	HNRNPD	Q14103-3	33 kDa	4	5	5	5
429	Isoform 2 c	NID2	Q14112-2	141 kDa	5	4	8	6
430	Isoform 2 c	HLA-A	P13746-2	41 kDa	5	3	8	5
431	D-3-phospl	PHGDH	O43175	57 kDa	2	2	1	3
432	Src substra	CTTN	Q14247	62 kDa	5	4	3	6
433	Isoform 2 c	BPHL	Q86WA6-2	31 kDa	2	2	1	1
434	Homogenti	HGD	Q93099	50 kDa	3	7	3	1
435	Hydroxyme	HMGCL	P35914	34 kDa	4	5	2	7
436	Ribosyldihy	NQO2	P16083	26 kDa	1	3	0	0
437	Fibrinogen	FGG	C9JC84	52 kDa	7	4	13	3
438	Membrane	PGRMC1	O00264	22 kDa	2	2	4	3
439	Radixin	RDX	P35241	69 kDa	9	16	14	12
440	NADH dehy	NDUFS2	O75306	53 kDa	6	6	5	7
441	Hypoxia up	HYOU1	Q9Y4L1	111 kDa	8	5	7	9
442	Phosphoglu	PGM1	P36871	61 kDa	1	3	1	3
443	GTP:AMP p	AK3	Q9UIJ7	26 kDa	4	4	2	1
444	Isoform 2 c	PRKCSH	P14314-2	59 kDa	3	3	3	4
445	S-methylm	BHMT2	Q9H2M3	40 kDa	2	2	4	4
446	Glutathion	GSTK1	Q9Y2Q3	25 kDa	0	2	4	5
447	Guanine nu	GNB2L1	P63244	35 kDa	3	4	7	6
448	Alpha-1-an	SERPINA3	P01011	48 kDa	5	6	4	5
449	Uromoduli	UMOD	E9PEA4	74 kDa	1	10	0	5
450	Coactosin-l	COTL1	Q14019	16 kDa	0	0	2	3
451	Isoform 1A	CTNND1	O60716-3	105 kDa	2	2	3	6
452	Isoform 2 c	2-Sep	Q15019-2	45 kDa	5	4	10	8
453	UDP-glucos	UGDH	O60701	55 kDa	3	3	3	0
454	Cystatin-B	CSTB	P04080	11 kDa	0	5	3	3
455	Pyridoxine-	PNPO	Q9NVS9	30 kDa	0	6	4	2
456	Ig alpha-1 c	IGHA1	P01876	38 kDa	0	5	5	6
457	Flavin redu	BLVRB	P30043	22 kDa	1	4	4	3
458	Cytochrom	COX5B	P10606	14 kDa	2	2	4	2
459	Vitronectin	VTN	P04004	54 kDa	2	4	2	3
460	Sulfite oxid	SUOX	P51687	60 kDa	0	2	1	0
461	Isoform 2 c	ATP6V0A1	Q93050-1	96 kDa	2	4	1	3
462	Isoform 2 c	ATP1B1	P05026-2	35 kDa	1	3	2	1
463	2'-deoxynu	DNPH1	H0Y8X4	26 kDa	0	5	1	1
464	Xaa-Pro dip	PD	P12955	55 kDa	0	6	1	0
466	PDZK1-inte	PDZK1IP1	Q13113	12 kDa	2	5	1	3
467	Isoform 2 c	DBI	P07108-2	12 kDa	0	2	2	2
468	Serine hydr	SHMT2	P34897	56 kDa	2	4	1	0
469	Cytochrom	UQCRB	P14927	14 kDa	1	2	2	3
470	Isoform 2 c	EIF5A	P63241-2	20 kDa	4	4	6	7

472	60S ribosor	RPL6	Q02878	33 kDa	4	3	7	7
473	T-complex	CCT4	P50991	58 kDa	2	4	5	3
474	Isoform 2 c	PICALM	Q13492-2	69 kDa	3	3	5	6
475	Isoform 2 c	HNRNPH3	P31942-2	35 kDa	6	3	6	3
476	28S ribosor	MRPS36	P82909	11 kDa	3	2	1	2
477	Cytochrom	COX6C	P09669	9 kDa	2	1	1	2
478	UDP-glucur	UGT1A9	O60656	60 kDa	5	10	8	8
479	Lumican	LUM	P51884	38 kDa	1	3	3	2
480	Hepatoma-	HDGF	P51858	27 kDa	3	3	1	2
481	Tubulin bet	TUBB2A	Q13885	50 kDa	20	22	23	24
482	Isoform 2 c	RPN2	P04844-2	68 kDa	1	2	8	7
483	Isoform 2 c	PPA2	Q9H2U2-2	40 kDa	2	4	4	2
484	Isoform 2 c	ATP5J	P18859-2	13 kDa	1	3	4	2
485	Vacuolar pı	VPS35	Q96QK1	92 kDa	4	4	6	7
486	Laminin sul	LAMB2	P55268	196 kDa	3	3	1	5
487	Probable A	DDX17	Q92841	80 kDa	5	5	8	8
488	Isoform 2 c	COL14A1	Q05707-2	192 kDa	0	1	0	0
489	Actin-relate	ACTR2	P61160	45 kDa	2	1	3	4
492	Aldehyde o	AOX1	Q06278	148 kDa	5	11	1	1
493	Ras-relatec	RAB1A	P62820	23 kDa	4	4	5	5
494	Heterogen	SYNCRIP	O60506	70 kDa	3	7	5	5
495	Isoform 3 c	NME2	P22392-2	30 kDa	3	6	10	7
496	V-type prot	ATP6V1D	Q9Y5K8	28 kDa	2	3	3	3
497	Glutamyl a	ENP	Q07075	109 kDa	1	2	1	3
498	40S ribosor	RPS7	P62081	22 kDa	1	3	3	5
499	OCIA doma	OCIAD2	Q56VL3	17 kDa	1	4	3	3
500	Dihydrolip	DLAT	P10515	69 kDa	3	4	3	3
501	Actin-relate	ACTR3	P61158	47 kDa	4	5	3	6
502	Methylmal	MUT	P22033	83 kDa	3	3	0	1
504	Dehydroge	DHRS4	Q9BTZ2	30 kDa	3	3	3	4
505	Alcohol del	ADH1B	P00325	40 kDa	1	5	9	6
507	Proteasom	PSMA1	P25786	30 kDa	1	4	4	3
508	Apolipopro	APOE	P02649	36 kDa	3	5	5	3
509	Acyl-CoA sy	ACSS3	Q9H6R3	75 kDa	3	3	0	1
511	Omega-am	NIT2	Q9NQR4	31 kDa	3	4	1	4
513	Ras-relatec	RAB14	P61106	24 kDa	4	3	3	2
514	Histidine tr	HINT1	P49773	14 kDa	3	2	3	3
516	Coatomer s	ARCN1	P48444	57 kDa	3	2	9	5
517	LETM1 and	LETM1	O95202	83 kDa	3	3	3	3
518	Mitochond	SLC25A11	Q02978	34 kDa	4	6	5	5
519	Isoform 2 c	ASL	P04424-2	50 kDa	1	3	1	4
520	Acetolactat	ILVBL	A1L0T0	68 kDa	2	2	2	3
521	Isoform 2 c	ADD1	P35611-2	70 kDa	1	2	0	0
522	40S ribosor	RPS9	P46781	23 kDa	3	4	7	7
524	Basal cell a	BCAM	P50895	67 kDa	7	5	5	5
525	Microtubul	MAP4	E7EVA0	245 kDa	0	2	7	5
526	Calponin-3	CNN3	Q15417	36 kDa	4	2	6	7

527	Erythrocyte	STOM	P27105	32 kDa	5	5	9	9
529	Glycine N-a	GLYATL1	Q969I3	35 kDa	2	3	3	3
531	Bifunctiona	ATIC	P31939	65 kDa	3	3	2	4
532	Heterogen	HNRNPH2	P55795	49 kDa	6	5	10	8
533	Tubulin alp	TUBA4A	P68366	50 kDa	18	14	20	19
534	Proteasom	PSMB1	P20618	26 kDa	2	3	7	6
535	NADH dehy	NDUFA10	E7ESZ7	45 kDa	2	3	1	2
536	2-oxoisova	BCKDHA	P12694	50 kDa	3	4	0	2
539	Proteasom	PSME1	Q06323	29 kDa	2	5	3	4
540	Serpin H1	SERPINH1	P50454	46 kDa	5	2	12	10
541	Polymeric i	PIGR	P01833	83 kDa	7	1	12	3
542	Asparagine	NARS	O43776	63 kDa	4	5	3	6
544	Tricarboxyl	SLC25A1	P53007	34 kDa	2	5	4	3
546	Isoform 1 c	H2AFY	O75367-2	39 kDa	1	1	3	3
550	Histidine tr	HINT2	Q9BX68	17 kDa	2	2	0	1
552	Alanine--gl	AGXT2	Q9BYV1	57 kDa	2	1	1	1
553	Isoform 2 c	MYH11	P35749-2	228 kDa	0	11	14	16
555	Nuclease-s	YBX1	P67809	36 kDa	5	2	8	6
557	Serine/thre	PPP2R1A	P30153	65 kDa	0	2	5	2
558	Thioredoxi	TXN	P10599	12 kDa	2	6	3	4
560	Haloacid de	HDHD3	Q9BSH5	28 kDa	0	2	0	1
561	Coronin-1B	CORO1B	Q9BR76	54 kDa	0	2	4	5
562	Sodium-co	SLC5A12	Q1EHB4	68 kDa	2	2	0	0
563	Alcohol del	ADH5	P11766	40 kDa	0	3	2	1
564	Gamma-glu	GGH	Q92820	36 kDa	1	5	5	3
566	Heterogen	HNRNPH1	G8JLB6	51 kDa	5	4	9	7
567	Pterin-4-al	PCBD1	P61457	12 kDa	1	2	4	1
571	CDGSH iror	CISD1	Q9NZ45	12 kDa	1	1	4	1
573	14-3-3 prot	YWHAG	P61981	28 kDa	6	8	7	6
574	Actin-relate	ARPC2	O15144	34 kDa	1	1	6	8
575	Isoform 3 c	POSTN	Q15063-3	87 kDa	2	0	6	10
580	Galactokina	GALK1	P51570	42 kDa	0	1	1	2
581	Ras GTPase	IQGAP2	Q13576	181 kDa	0	0	0	0
585	Chloride in	CLIC1	O00299	27 kDa	3	5	6	8
586	Cytochrom	CYP4A11	Q02928	59 kDa	1	6	0	0
589	Macrophag	MIF	P14174	12 kDa	1	1	1	3
590	Isoform 2 c	MSRA	Q9UJ68-2	19 kDa	0	3	1	2
596	Isoform 2 c	SUCLA2	Q9P2R7-2	48 kDa	0	1	1	1
599	Coagulatio	F13A1	P00488	83 kDa	1	3	9	5
600	Xaa-Pro am	XPNP2	O43895	76 kDa	0	2	0	0
603	Epidermal	EPS8L2	Q9H6S3	81 kDa	4	6	5	3
604	Isoform 2 c	ASAH1	Q13510-2	47 kDa	0	6	3	2
605	Valine--tRN	VARS	P26640	140 kDa	6	3	9	5
607	Single-stra	SSBP1	Q04837	17 kDa	3	3	1	3
609	Phosphose	PSAT1	Q9Y617	40 kDa	1	2	1	1
610	Isoform 2 c	AP2A2	O94973-2	104 kDa	2	1	5	3
613	Glucosamir	GNPDA1	P46926	33 kDa	0	2	2	1

614	Tetratricop	TTC38	Q5R3I4	53 kDa	1	6	1	1
615	Hydroxyaci	HAO2	Q9NYQ3	39 kDa	1	1	0	0
616	Isoform 6 c	AGRN	O00468-6	215 kDa	11	5	5	5
617	Phenylalan	PAH	P00439	52 kDa	0	1	0	0
627	Protein am	AMN	Q9BXJ7	48 kDa	3	4	0	0
633	Ornithine a	OAT	P04181	49 kDa	0	0	2	0
634	Serotransfe	TF	P02787	77 kDa	0	4	5	3
635	RNA-bindin	RBMX	P38159	42 kDa	4	3	2	5
637	40S ribosor	RPS3A	P61247	30 kDa	5	3	5	5
638	Far upstrea	KHSRP	Q92945	73 kDa	5	2	8	6
640	Cytoskelet	CKAP4	Q07065	66 kDa	0	1	6	10
649	Peroxisoma	PIPOX	Q9P0Z9	44 kDa	1	1	0	0
650	Plastin-2	LCP1	P13796	70 kDa	1	2	2	10
653	Protein ND	NDRG1	Q92597	43 kDa	1	2	0	1
654	Isoform 3 c	CCT7	Q99832-3	55 kDa	3	3	6	4
655	Peroxisoma	ACOX2	Q99424	77 kDa	0	2	0	1
659	40S ribosor	RPS18	P62269	18 kDa	2	2	3	4
660	Sulfide:qui	SQRDL	Q9Y6N5	50 kDa	1	3	6	5
662	Glutathion	GSTM3	P21266	27 kDa	2	4	3	7
674	Isoform 2 c	DDX3X	O00571-2	71 kDa	5	4	10	8
685	Lamin-B1	LMNB1	P20700	66 kDa	1	2	5	6
686	Ig gamma-2	IGHG2	P01859	36 kDa	0	2	5	5
692	Isoform 2 c	PABPC1	P11940-2	61 kDa	6	4	3	5
693	Protein tra	SEC23A	Q15436	86 kDa	1	2	5	5
699	PDZ and LII	PDLIM2	Q96JY6	37 kDa	1	2	1	1
701	Zyxin	ZYX	Q15942	61 kDa	2	2	4	6
719	PDZ and LII	PDLIM5	Q96HC4	64 kDa	0	1	6	6
720	Protein S1(	S100A9	P06702	13 kDa	0	6	6	4
735	Tubulin alp	TUBA1B	P68363	50 kDa	0	16	25	24
738	14-3-3 prot	YWHAQ	P27348	28 kDa	0	9	7	5
751	Plastin-1	PLS1	Q14651	70 kDa	0	7	5	7
774	Isoform 2 c	HIST2H2BF	Q5QNW6-2	15 kDa	6	6	6	8
793	Microsoma	MGST3	O14880	17 kDa	3	1	0	3
795	Keratin. typ	KRT16	P08779	51 kDa	0	0	0	0
817	Beta-actin-	ACTBL2	Q562R1	42 kDa	13	0	19	14
818	Haptoglobi	HP	P00738	45 kDa	1	0	3	1
820	HLA class I	HLA-B	P01889	40 kDa	3	3	4	6
830	Junction pl	JUP	F5GWP8	66 kDa	0	5	0	5
843	Extended s	ESYT1	Q9BSJ8	123 kDa	5	1	6	8
845	Tropomyos	TPM1	H7BYY1	29 kDa	4	9	14	13
875	Rab GDP di	GDI1	P31150	51 kDa	5	0	7	8
876	Ras-relatec	RAB1B	Q9H0U4	22 kDa	5	5	6	6
886	Aldose red	AKR1B1	P15121	36 kDa	0	2	3	5
938	POTE ankyı	POTEI	P0CG38	121 kDa	11	9	8	9
940	Tubulin bet	TUBB6	Q9BUF5	50 kDa	11	12	13	18
961	ADP/ATP tı	SLC25A6	P12236	33 kDa	8	10	9	11
964	Hemoglobi	HBD	P02042	16 kDa	5	6	11	11

982	14-3-3 prot	YWHAH	Q04917	28 kDa	4	3	5	3
986	Isoform 2 c	PCBP2	Q15366-2	39 kDa	4	0	5	4
988	Histone H2	HIST1H2AC	Q93077	14 kDa	0	0	0	0
990	Histone H2	HIST2H2AB	Q8IUE6	14 kDa	3	2	2	7
992	Histone H2	H2AFZ	P0C0S5	14 kDa	2	0	0	5
995	Histone H2	HIST1H2AG	P0C0S8	14 kDa	0	0	0	12
997	Tubulin bet	TUBB4A	P04350	50 kDa	0	24	22	0
1006	ADP-ribosy	ARF4	P18085	21 kDa	5	8	6	6
1021	HLA class I	HLA-A	P01891	41 kDa	0	3	5	8
1064	V-type prot	ATP6V1B1	P15313	57 kDa	0	0	0	0
1126	Isoform 2 c	TPM2	P07951-2	33 kDa	0	6	12	10
1210	Isoform 2 c	EIF4A2	Q14240-2	46 kDa	7	4	0	0
1250	ADP-ribosy	ARF5	P84085	21 kDa	6	7	6	6
1380	Isoform 2 c	SPTBN1	Q01082-3	251 kDa	0	0	0	0
1381	Aldo-keto r	AKR1C1	Q04828	37 kDa	0	0	0	4

Raw C								
IgAN E0	IgAN E0	IgAN E0	IgAN E1	IgAN E1	IgAN E1	IgAN E1	NLTx	NLTx
T8	T9	T10	T4	T20	T21	T23	T5	T6
46	61	48	67	69	63	75	48	50
54	45	49	56	41	58	65	47	51
41	52	41	45	46	68	79	49	38
47	32	35	49	46	64	70	36	38
46	47	40	46	35	62	70	40	50
48	52	48	42	39	51	47	26	30
62	57	54	54	17	39	46	51	63
18	56	31	23	48	48	52	14	15
43	39	34	49	30	44	51	46	43
41	39	27	40	25	47	52	31	37
18	53	39	52	40	34	39	28	23
48	34	45	45	47	53	57	39	34
55	44	37	43	30	33	35	43	47
34	27	32	39	21	42	48	35	41
45	37	44	37	25	46	38	37	41
31	21	15	25	37	47	47	28	21
23	35	23	32	47	39	44	19	17
46	44	31	47	18	39	33	32	42
39	30	32	38	36	33	30	29	28
44	29	28	35	23	41	31	26	35
25	24	27	22	35	44	50	21	22
43	37	28	36	30	34	32	28	39
50	40	35	38	15	34	33	37	40
57	50	22	31	15	41	43	32	44
36	35	37	33	21	49	52	32	34
35	30	27	39	22	38	35	27	34
44	30	34	34	16	32	26	41	37
32	30	25	25	22	42	33	31	34
36	30	31	24	21	30	33	26	23
29	25	32	24	22	35	37	28	25
21	23	22	22	22	36	31	23	27
30	26	20	27	19	30	30	29	32
35	25	17	27	20	29	27	27	36
2	4	5	3	15	2	3	5	2
21	15	14	20	26	42	35	16	20
37	32	23	29	12	43	41	22	19
18	19	19	34	18	22	26	23	27
20	27	24	27	19	23	29	23	25
9	12	29	22	29	9	18	39	28
27	24	16	26	15	30	32	27	25
33	22	29	20	20	22	30	25	22
20	22	21	23	15	34	34	15	20
15	21	44	23	26	18	15	38	27

19	22	10	24	18	22	20	21	19
31	26	21	31	18	27	36	22	27
34	15	20	27	11	45	30	17	24
27	31	27	22	13	34	24	22	26
19	18	18	20	19	28	26	20	23
23	19	26	28	15	14	18	22	29
16	14	11	17	20	19	27	17	17
18	15	19	17	18	34	25	18	17
4	15	10	18	26	18	28	14	10
24	24	22	22	20	18	21	23	20
7	7	9	11	13	59	16	6	4
12	14	18	11	17	32	24	17	12
19	18	15	16	19	21	19	16	14
17	24	8	0	19	12	20	9	9
9	11	9	11	27	21	26	6	3
5	21	10	13	19	21	27	11	9
28	30	21	16	7	25	19	20	25
19	15	18	20	12	18	22	21	19
17	16	12	20	17	21	18	19	17
26	21	16	24	9	18	24	27	22
17	14	12	16	10	26	23	16	14
24	14	15	16	14	27	33	20	23
16	19	13	15	13	28	18	14	13
20	19	15	15	11	24	25	13	14
17	14	12	14	14	24	23	8	11
23	23	15	20	8	24	14	17	23
23	17	17	19	10	12	18	17	21
0	3	9	10	14	29	16	11	3
22	16	10	18	11	21	13	13	15
13	7	10	14	17	25	21	9	13
13	13	13	17	17	25	19	12	14
20	13	10	11	10	34	26	14	12
11	15	13	19	11	16	15	15	18
17	15	5	11	15	17	17	12	12
22	18	11	19	10	19	17	14	16
16	20	17	17	19	18	16	12	14
16	14	14	16	11	18	19	14	16
10	6	10	7	9	27	34	14	17
24	22	14	20	9	16	12	18	20
23	17	12	16	9	28	34	13	18
18	11	10	14	12	18	17	16	19
15	9	11	14	9	16	18	15	17
15	12	15	16	13	16	18	12	17
1	1	4	5	11	2	12	3	1
16	18	17	11	10	18	19	12	16
17	21	5	14	6	26	22	19	17
21	13	15	17	10	15	11	15	16

12	14	13	13	13	29	26	9	9
21	18	13	15	13	18	15	14	16
18	11	15	16	14	18	21	14	14
12	18	13	10	18	21	20	10	1
9	8	5	33	18	0	6	34	36
16	18	16	16	9	17	10	15	14
27	19	8	13	10	19	16	11	16
12	10	10	11	7	22	25	13	11
14	9	11	16	13	16	17	13	11
11	7	9	9	7	23	18	9	13
11	12	10	12	8	17	15	12	10
20	17	15	13	8	16	20	16	18
19	14	14	17	10	13	11	17	20
14	15	9	8	11	14	12	8	8
16	16	10	12	3	18	18	12	16
18	20	16	16	6	11	16	18	17
15	14	10	12	9	18	14	11	10
11	17	16	10	16	18	23	17	1
2	7	7	8	10	14	7	0	0
11	10	11	14	15	17	17	10	10
20	11	15	18	4	16	20	20	15
15	12	10	13	10	12	19	15	13
14	11	10	14	11	14	20	10	12
17	17	11	14	8	12	11	11	11
17	16	9	13	9	16	14	9	15
13	14	12	17	7	11	11	18	12
15	18	15	17	9	14	15	14	9
34	39	31	34	36	43	57	34	23
6	7	7	10	9	14	12	7	9
26	21	21	27	21	25	20	21	20
17	14	14	14	10	13	11	12	13
9	12	11	12	7	14	20	12	9
11	9	13	11	12	8	13	6	13
15	10	8	9	9	20	19	9	13
7	17	13	19	5	17	10	14	18
12	9	16	8	12	12	12	10	17
6	8	37	15	31	15	12	31	15
19	16	11	14	5	16	17	17	10
13	8	10	10	8	13	13	11	12
5	6	7	7	8	6	7	5	7
11	12	11	10	5	15	13	10	12
13	7	6	8	10	15	15	9	7
20	14	9	13	5	21	16	10	11
9	11	5	7	14	14	13	9	10
10	12	7	9	11	12	11	11	12
6	10	9	10	10	7	10	8	6
11	13	18	14	15	11	13	6	9

9	9	8	8	12	9	15	10	9
14	15	6	11	12	13	19	12	14
12	13	12	11	6	12	16	9	14
0	3	2	7	13	4	3	1	0
9	12	4	9	7	14	20	9	15
18	15	10	17	16	26	23	16	18
13	9	6	10	6	16	11	8	11
10	12	8	8	11	11	12	9	11
12	10	5	10	4	14	18	10	10
14	11	16	14	7	18	7	9	15
13	14	14	11	5	16	12	10	13
9	15	12	15	9	7	10	8	9
11	10	8	10	9	15	13	10	15
12	6	3	7	11	20	13	6	7
15	17	10	13	11	10	9	16	15
13	13	11	15	7	9	9	11	14
11	7	10	11	11	13	14	5	8
12	9	11	10	6	8	7	11	8
11	9	8	7	5	12	18	10	10
0	7	3	1	16	0	20	0	0
12	8	9	8	11	17	12	7	9
8	9	10	13	4	19	15	9	9
11	12	13	12	15	15	14	13	10
12	11	10	13	9	11	10	13	12
15	23	5	5	4	14	14	7	11
13	11	8	12	5	7	8	10	7
17	18	10	10	7	10	9	11	10
7	5	3	8	13	17	19	4	4
4	13	10	6	14	11	11	5	5
14	8	5	13	9	17	11	12	9
18	9	9	13	5	20	19	9	12
9	5	9	7	2	16	12	8	9
3	13	4	3	9	17	15	3	2
9	4	7	7	7	11	11	5	11
14	11	7	10	6	8	7	8	12
12	10	9	11	6	17	6	8	10
41	35	33	31	20	37	37	28	27
17	21	15	26	7	9	11	16	20
13	11	10	7	5	12	10	9	12
9	6	8	10	7	15	11	11	9
8	10	5	11	4	11	11	6	10
8	8	8	9	9	14	12	12	6
5	5	7	9	8	13	12	9	11
11	7	8	13	5	11	11	10	12
10	7	11	9	5	12	11	8	9
6	7	4	5	7	9	9	2	7
5	9	10	6	7	8	13	6	7

23	13	5	7	2	19	8	10	10
9	9	5	6	4	20	10	9	6
11	8	5	15	7	10	12	12	12
12	9	9	9	7	12	11	10	11
12	14	8	8	5	15	16	10	6
11	8	7	8	6	9	10	11	11
10	9	5	7	5	15	15	10	12
16	10	11	9	4	13	12	11	10
8	11	10	9	4	11	11	12	8
6	5	9	6	18	9	9	13	7
5	8	5	4	6	7	9	4	6
15	12	12	9	6	6	7	9	12
10	9	8	10	3	13	9	9	9
8	9	8	3	10	15	14	7	5
9	8	7	7	8	15	8	8	8
6	10	7	6	6	9	9	9	8
11	9	9	9	5	10	10	10	11
19	12	5	5	9	10	8	7	6
7	8	7	5	11	9	10	4	6
13	9	5	9	4	11	12	7	8
5	7	6	6	12	6	7	7	8
10	11	10	10	7	9	9	5	3
4	6	4	7	9	9	7	6	5
9	4	11	14	9	12	9	8	12
9	12	13	9	7	6	7	9	7
12	13	6	16	12	12	12	8	15
10	11	8	9	8	9	9	11	7
2	4	6	6	6	10	9	7	9
12	10	8	11	5	10	7	7	9
9	6	6	7	7	7	9	2	7
1	4	1	2	9	8	16	4	2
6	9	5	7	2	13	9	6	8
14	9	8	12	4	11	10	13	10
3	6	8	8	8	6	7	7	3
12	7	10	5	8	9	8	8	8
10	9	9	12	16	11	12	4	11
6	7	3	5	8	4	11	3	4
8	9	5	8	3	7	14	7	8
9	4	8	7	2	13	13	7	7
6	6	5	7	5	11	9	10	8
9	9	2	2	4	12	11	5	7
12	4	5	6	0	13	11	4	5
6	10	13	7	5	22	13	10	7
9	10	9	8	6	9	5	4	8
7	6	3	6	8	13	12	4	7
7	9	8	6	2	8	7	7	3
11	5	3	5	5	8	9	7	6

5	7	9	4	10	7	6	7	6
11	8	12	9	6	11	9	7	10
7	7	4	12	4	5	8	13	8
11	7	6	7	4	8	9	9	10
11	8	5	6	3	13	8	4	7
9	11	8	11	4	7	9	9	10
4	3	5	5	6	10	9	4	6
5	6	6	9	7	6	8	9	8
8	7	8	7	7	12	10	8	8
7	1	4	5	12	4	5	5	1
7	5	6	4	7	12	7	7	5
4	3	6	5	1	16	9	4	5
9	5	3	6	0	9	15	4	6
7	7	8	6	5	6	11	10	8
6	7	10	9	20	20	30	5	5
8	7	13	8	4	8	9	8	6
8	9	6	6	6	11	10	8	10
1	4	4	5	6	3	5	5	1
8	6	5	9	5	10	9	10	7
5	7	3	9	4	10	7	1	6
14	8	7	8	5	4	7	10	11
8	7	6	8	3	8	6	8	12
7	7	7	6	9	6	8	6	7
9	8	5	7	2	14	11	8	6
6	4	1	7	7	6	8	4	5
0	0	1	3	4	3	5	3	1
6	5	6	7	3	12	9	6	7
11	9	7	6	3	12	7	3	9
7	5	1	6	5	11	5	2	4
8	7	8	8	3	8	7	7	7
10	9	10	10	12	9	13	7	9
7	5	6	8	7	4	7	8	8
11	8	9	7	4	9	9	6	6
2	4	3	1	6	10	12	2	2
5	4	3	6	8	6	5	6	7
7	8	8	8	3	10	10	8	9
6	5	3	4	8	8	8	8	4
3	4	4	3	1	10	9	3	5
5	5	3	4	9	8	6	4	10
6	8	8	9	3	3	2	8	7
6	6	4	5	1	13	13	3	6
0	4	5	4	6	5	6	1	1
4	8	9	7	11	7	5	5	3
9	7	8	9	4	5	7	6	12
5	6	6	4	0	10	9	5	4
1	9	5	2	8	6	5	2	2
8	5	6	8	1	6	9	12	12

6	6	6	6	1	11	9	6	7
9	19	11	17	9	11	10	7	8
6	5	6	5	7	5	7	4	5
4	5	2	6	4	10	10	3	4
6	2	2	4	4	7	10	3	2
8	7	6	7	5	6	8	5	7
10	7	9	9	3	8	5	9	3
6	5	4	7	6	7	7	7	9
11	7	5	9	4	4	5	11	12
9	5	3	4	0	11	5	1	6
4	7	6	5	3	4	4	8	8
8	5	3	9	3	7	6	6	6
7	10	3	7	3	10	11	5	7
5	3	5	4	3	6	8	5	4
6	9	3	5	8	6	7	5	5
6	5	5	7	5	6	7	9	5
6	6	3	4	7	7	9	5	5
4	4	4	4	4	9	11	2	3
7	6	8	6	5	6	6	6	7
2	3	4	6	5	6	5	4	2
14	11	7	9	8	15	19	12	11
7	8	4	11	3	7	5	6	7
9	5	4	4	7	12	14	3	4
7	5	5	7	3	18	5	5	5
6	4	1	6	2	5	8	2	6
7	6	6	5	3	6	10	8	8
5	5	3	5	3	8	9	6	5
4	2	4	4	5	12	8	6	3
6	9	6	9	5	5	6	9	4
7	7	6	6	2	7	7	4	8
2	1	1	2	1	7	7	3	1
5	8	9	3	10	7	8	6	2
12	7	8	5	1	5	3	9	10
7	5	8	6	4	5	4	5	7
4	7	5	7	1	8	6	2	3
2	2	4	9	5	1	5	2	3
4	3	5	5	1	6	12	5	5
0	4	3	4	6	4	4	6	5
8	5	5	6	4	6	7	6	5
8	5	4	6	2	8	8	6	6
3	3	2	3	8	13	9	3	4
2	2	2	3	8	5	8	2	4
3	5	3	4	5	7	9	2	5
14	5	10	12	3	8	7	11	10
8	5	6	7	1	4	9	5	6
25	18	29	16	18	30	29	25	19
5	4	4	5	3	3	3	3	4

4	4	4	3	7	7	5	2	2
15	9	6	8	4	5	10	4	11
8	6	5	5	3	11	7	5	3
5	4	3	7	4	7	7	5	3
4	4	4	3	2	3	7	1	6
4	3	6	4	1	7	7	8	8
6	4	7	8	9	3	4	4	7
9	8	5	11	1	9	4	7	10
3	2	3	3	3	6	7	3	4
2	1	3	6	5	7	4	5	4
1	1	3	4	2	5	6	5	3
0	2	9	5	3	7	12	6	8
5	4	2	5	3	3	4	2	6
7	7	3	7	2	9	6	5	6
8	12	7	6	0	8	7	4	4
0	2	3	2	3	2	5	1	0
2	3	4	5	5	7	9	4	2
5	6	3	6	6	4	5	7	5
6	4	5	11	3	6	4	5	7
5	5	3	6	2	6	4	3	5
4	5	3	3	3	4	3	5	7
0	3	3	0	1	8	9	0	2
3	4	4	4	3	8	5	4	3
5	3	4	5	1	6	6	8	2
4	3	3	3	5	3	6	4	4
8	6	4	4	2	8	5	5	5
7	4	4	4	4	12	11	3	4
2	4	4	7	5	6	6	3	4
7	3	2	4	1	4	3	3	5
1	3	1	4	5	6	8	0	0
3	3	3	3	2	9	4	4	3
7	7	3	4	4	6	6	5	5
5	3	3	4	4	4	7	4	4
3	3	4	3	4	7	6	3	4
0	4	3	2	3	8	7	2	4
8	6	6	6	0	3	4	10	8
6	4	6	5	7	2	3	3	4
5	4	7	4	5	4	5	3	2
5	5	4	6	5	6	5	6	6
6	3	3	2	1	10	9	3	5
8	5	6	6	1	6	4	8	6
2	3	10	5	8	5	0	16	13
1	3	2	2	2	0	2	3	1
6	4	2	8	3	4	3	7	7
5	4	7	5	5	5	5	4	7
4	2	2	3	7	7	5	3	4
8	6	18	0	4	8	8	3	10

1	0	1	2	5	9	12	1	4
3	4	4	4	2	3	4	1	4
4	7	2	5	4	12	5	3	5
3	4	4	6	9	2	3	4	3
8	6	4	3	4	8	2	3	2
3	5	1	5	3	5	7	6	4
3	5	5	5	0	4	4	7	7
0	7	3	4	3	3	6	2	3
1	1	2	4	2	6	3	4	2
5	2	8	9	0	2	3	4	5
3	3	1	4	6	7	9	2	3
4	5	4	5	4	9	5	7	5
3	3	3	4	2	9	10	3	4
6	4	2	4	4	5	3	5	3
6	4	4	5	5	6	2	6	5
2	4	1	4	2	8	5	2	5
5	4	3	4	4	6	6	5	5
6	7	6	6	2	4	4	4	7
4	2	2	4	2	5	3	1	2
2	8	3	7	1	8	11	1	7
3	1	4	4	3	2	4	6	4
3	2	4	5	3	7	6	4	4
4	3	2	1	3	6	7	1	4
5	4	5	5	2	6	4	6	3
1	3	2	2	4	3	4	2	3
8	5	5	3	1	6	4	6	6
10	8	4	3	0	10	3	4	7
5	3	5	2	1	11	12	4	5
6	6	6	3	1	9	6	4	2
4	5	5	3	5	7	5	4	4
7	6	5	3	1	4	5	5	7
6	5	4	3	4	3	4	5	4
5	5	1	5	5	3	4	3	3
4	5	3	3	1	4	5	4	4
4	4	4	3	3	3	3	3	3
3	1	2	2	2	7	4	2	0
4	1	1	3	1	6	5	2	3
6	6	5	5	3	5	4	7	3
0	0	12	6	18	0	0	18	8
4	5	2	5	4	3	6	4	6
0	1	1	2	3	2	7	2	0
8	6	7	5	0	6	3	4	8
3	3	4	2	5	6	6	3	2
4	3	4	3	2	2	7	4	5
2	1	2	2	2	2	4	1	2
0	0	2	2	4	1	0	1	0
4	3	2	2	2	7	6	5	1

5	3	2	5	1	7	1	1	4
6	8	3	3	1	2	4	4	3
0	1	2	3	5	4	4	3	5
5	5	4	5	3	3	4	4	6
3	4	2	4	2	7	5	3	3
3	2	3	3	1	4	3	3	3
1	3	4	3	4	3	4	2	1
2	4	3	3	8	3	3	4	2
5	5	6	3	2	3	3	3	4
2	3	4	6	4	2	1	4	1
7	5	3	6	1	5	3	3	5
3	3	2	3	1	7	8	2	3
2	2	4	1	4	4	9	1	2
8	7	2	2	2	3	3	7	7
0	0	0	0	0	3	4	0	0
5	3	2	3	3	6	5	3	3
20	9	12	18	0	14	11	17	14
3	1	4	2	2	4	3	1	2
2	1	0	4	3	4	4	0	1
5	3	3	4	2	9	4	5	4
7	1	3	3	2	6	6	5	3
2	3	6	3	4	4	4	4	4
10	10	6	4	2	7	8	8	7
3	3	3	2	3	7	6	3	4
2	1	4	1	3	3	6	1	4
0	0	0	4	8	4	5	1	0
1	0	3	0	6	8	11	0	0
3	2	2	4	3	2	4	5	5
3	2	5	3	1	1	0	4	1
1	2	0	3	4	2	6	1	2
3	2	4	3	1	6	2	4	3
2	2	2	3	1	4	6	3	3
2	5	0	2	2	10	10	3	2
2	6	6	3	5	0	1	3	0
5	5	1	3	2	7	5	3	4
5	6	2	5	1	3	3	4	5
3	3	5	4	3	2	2	3	2
7	6	0	5	1	5	1	1	6
5	5	3	5	1	2	2	3	2
5	2	3	7	4	3	4	3	4
6	2	1	2	2	8	6	1	1
6	4	1	2	0	5	5	1	1
5	5	3	4	3	3	4	5	5
3	4	2	4	3	5	4	5	5
8	8	7	5	2	6	5	9	5
5	2	5	2	5	3	3	5	3
3	1	0	1	2	4	4	0	1

1	2	1	3	2	4	4	2	2
4	3	2	6	4	2	2	3	5
2	2	1	4	1	3	3	3	2
2	3	5	3	0	2	3	3	2
4	7	3	8	3	3	3	4	8
3	5	2	7	2	3	2	6	5
8	0	4	6	8	17	15	0	8
0	3	3	2	4	7	4	1	3
4	5	4	6	3	2	2	4	5
28	23	24	20	13	29	30	18	18
1	1	1	4	1	4	4	0	4
5	2	1	4	1	2	5	6	4
8	3	2	4	1	2	1	5	5
0	0	2	1	5	2	6	1	0
0	4	2	2	1	4	6	0	1
0	1	1	1	5	3	4	1	1
0	5	3	11	0	0	2	2	6
4	3	2	4	2	4	5	2	4
3	2	2	2	0	10	1	2	1
2	2	3	5	3	3	4	3	4
2	3	2	3	3	4	3	2	1
2	1	2	1	4	4	4	1	1
2	4	3	2	0	4	4	4	3
2	1	3	2	0	6	5	1	3
2	3	2	2	3	2	5	1	4
4	5	2	2	2	3	4	4	4
2	4	1	2	2	2	1	3	2
3	3	2	2	4	3	2	0	1
4	3	4	3	3	3	2	7	5
1	2	3	4	2	5	6	1	4
0	2	1	3	3	7	8	0	0
5	5	1	6	2	4	3	1	4
2	1	3	2	3	9	7	1	1
4	1	5	6	1	4	3	3	4
4	4	3	4	1	3	1	6	5
2	3	1	4	1	4	4	1	1
5	5	2	4	1	4	5	2	7
2	2	3	4	3	3	1	2	2
5	2	1	2	4	2	3	4	2
3	1	1	1	2	3	4	2	2
4	3	1	1	1	6	4	4	1
3	4	1	4	1	6	5	2	4
2	2	3	3	4	3	6	3	2
1	1	0	1	2	4	4	1	1
0	1	2	1	4	2	2	0	1
1	3	1	2	5	4	4	2	1
3	2	0	4	2	3	3	1	0

1	0	3	0	3	4	3	0	0
3	3	6	4	1	5	2	2	3
2	3	1	5	0	3	3	3	3
9	8	7	9	5	6	5	5	5
15	15	14	12	14	17	17	12	12
1	2	2	2	2	2	4	2	3
4	2	2	5	1	2	5	2	4
6	5	1	6	2	1	2	9	4
0	1	0	2	1	5	3	3	1
0	1	1	1	2	3	3	1	1
0	0	0	0	5	2	2	1	1
2	0	2	2	3	2	3	2	3
3	3	2	2	0	5	4	3	3
2	1	1	3	2	6	3	2	3
5	5	4	4	1	4	1	4	3
0	5	2	7	2	4	5	8	6
0	10	7	7	0	7	13	10	7
4	2	3	5	3	0	2	1	2
2	4	0	3	2	1	3	1	1
1	2	1	1	4	6	3	1	1
5	4	3	3	2	2	1	3	4
4	6	2	3	0	1	2	1	1
2	3	3	3	1	3	2	5	4
0	1	2	2	2	5	8	1	3
2	0	1	1	0	5	4	0	1
6	7	4	7	6	6	6	5	5
3	5	2	2	1	7	4	2	3
2	2	1	3	3	3	5	3	2
6	0	0	0	8	7	9	0	5
0	1	0	0	3	1	2	1	1
0	11	0	0	4	0	2	0	0
7	6	3	5	0	1	1	3	5
2	2	3	3	0	4	5	2	4
1	1	0	1	4	2	3	0	1
5	5	4	2	1	7	1	1	3
3	3	2	2	2	3	1	3	3
3	3	4	5	2	4	2	2	2
4	2	3	2	1	3	2	3	2
1	3	2	1	3	0	2	0	0
1	3	4	0	0	8	1	2	3
2	0	1	1	0	4	0	2	1
2	1	1	1	4	6	2	0	1
2	0	0	1	2	4	3	0	1
2	4	1	4	1	2	3	2	5
6	2	1	2	0	5	4	2	4
2	2	1	4	2	0	4	0	1
2	3	2	1	0	4	6	2	4

2	0	1	2	1	1	3	0	4
3	1	4	5	1	7	1	3	3
0	0	1	0	2	3	1	0	0
3	3	2	3	0	3	3	3	5
6	4	4	6	1	3	3	1	4
2	2	1	2	0	5	3	1	3
1	1	1	0	7	8	10	0	0
1	1	2	5	4	2	1	1	1
1	1	2	1	4	0	2	2	0
1	1	2	1	2	1	1	0	0
0	1	1	1	0	0	1	0	0
1	6	3	5	0	7	1	2	4
0	0	0	0	1	0	3	1	0
4	2	1	3	0	1	1	3	5
1	1	0	1	3	5	5	0	1
7	6	3	5	1	2	0	2	5
0	0	1	1	4	3	0	1	1
0	1	0	2	1	2	3	0	0
0	0	0	0	0	7	8	0	0
0	0	2	0	0	0	4	2	0
3	4	1	3	2	2	6	0	2
2	3	2	5	0	3	4	3	0
0	2	0	1	2	1	1	0	2
0	0	2	0	2	2	3	0	0
5	1	5	3	0	2	1	3	3
0	1	1	1	2	1	4	0	0
0	0	0	1	1	1	4	0	1
0	0	0	0	6	0	0	1	0
20	22	19	19	19	0	0	16	16
4	0	4	3	6	6	8	2	0
1	0	0	1	0	4	2	1	2
0	8	11	9	6	11	11	7	9
0	0	0	0	0	6	6	0	0
0	0	0	0	0	0	0	10	7
12	0	14	12	0	20	25	12	11
0	2	0	3	2	0	0	2	0
1	2	0	3	5	3	3	3	1
0	0	0	3	4	0	4	8	6
0	0	0	0	0	1	0	0	0
10	15	13	15	10	7	9	10	9
8	5	2	6	5	7	7	5	4
3	2	3	3	0	5	6	0	3
0	0	0	2	0	3	3	2	0
9	10	0	0	0	9	9	0	0
8	0	0	6	0	12	12	0	0
9	8	7	7	4	10	14	8	8
0	0	7	0	12	11	9	0	0

0	5	6	6	6	5	5	3	5
4	5	2	4	0	0	4	0	2
10	13	10	12	0	0	13	12	8
0	6	0	0	2	6	8	0	0
0	0	0	0	3	4	5	0	0
0	13	0	12	0	10	14	12	10
0	27	0	24	0	30	30	0	20
2	0	0	0	2	8	6	2	0
0	0	3	3	3	3	3	3	0
0	0	0	6	6	7	8	9	6
0	14	8	0	0	8	9	0	0
0	0	0	6	6	0	0	0	3
0	0	2	0	0	8	0	0	0
0	0	0	48	0	0	0	46	43
0	0	0	0	0	8	8	0	0

ounts

NLTx	NLTx	NLTx	NLTx	NLTx	NLTx	S.a.GN	S.a.GN	S.a.GN
T13	T14	T15	T28	T29	T30	T1	T2	T11
75	54	47	48	38	38	98	90	107
58	69	64	69	62	70	37	62	54
46	44	52	80	43	52	42	55	48
42	39	53	67	65	63	27	52	36
48	46	53	81	68	75	33	47	29
49	51	29	50	56	55	36	56	53
52	61	74	49	40	51	20	51	22
33	25	14	31	37	46	53	55	58
45	47	41	56	46	43	28	48	36
39	54	40	84	63	69	23	38	36
44	27	14	29	19	18	73	74	93
44	42	28	35	39	45	47	49	59
49	48	45	42	44	36	30	41	30
49	49	33	58	72	64	20	31	20
39	43	40	54	60	52	22	33	24
22	22	15	56	44	45	23	27	31
34	20	18	16	23	20	51	40	50
34	34	49	33	44	45	13	44	19
33	42	28	33	29	33	33	32	35
33	45	31	43	48	54	12	23	16
33	29	25	45	46	42	25	31	28
39	36	27	28	30	28	34	34	40
41	52	39	55	42	47	13	30	14
40	46	43	57	34	40	8	34	10
40	45	35	78	30	32	13	24	13
36	44	31	40	47	40	21	23	20
37	35	33	35	35	29	14	22	23
32	32	36	41	45	49	13	23	14
30	29	24	28	28	29	31	34	37
26	30	24	36	38	42	13	15	17
25	28	30	42	28	38	15	11	10
27	27	37	32	33	38	14	24	14
26	31	35	28	33	34	15	34	20
3	3	3	2	2	2	9	3	4
23	13	17	22	22	21	31	34	41
25	26	20	38	29	42	6	26	9
28	29	27	20	26	22	23	28	31
29	26	22	20	19	19	35	33	37
11	18	48	22	5	8	42	15	15
25	29	25	36	27	33	16	25	23
27	28	28	35	35	41	11	20	11
21	24	19	36	23	37	23	22	18
15	20	40	17	5	2	33	23	14

23	28	17	21	16	17	26	28	35
34	29	18	28	24	21	34	45	39
24	19	28	33	30	37	6	18	6
18	24	27	37	23	31	8	17	6
20	24	20	28	26	26	11	19	16
26	34	31	22	27	27	16	18	16
22	16	15	18	23	21	14	18	22
21	17	15	26	29	30	17	19	21
11	13	17	5	18	9	26	36	21
24	23	21	20	22	20	16	19	14
12	8	2	11	15	9	15	11	16
9	15	20	22	8	12	7	17	24
18	20	19	19	17	17	17	19	27
7	11	17	6	13	11	25	35	33
11	5	6	9	15	15	22	15	23
15	9	5	16	22	17	21	19	23
23	30	36	17	23	24	6	17	10
20	20	20	21	19	22	13	12	12
16	17	13	14	21	17	14	16	15
22	26	19	20	20	23	3	19	12
16	16	12	16	20	21	12	16	19
23	25	23	27	22	30	11	19	12
10	9	13	23	25	24	12	23	18
17	17	19	26	23	24	5	11	12
13	10	9	20	17	18	15	16	26
26	21	28	24	17	21	8	20	11
22	25	20	20	21	18	12	19	8
8	15	7	12	2	1	4	11	35
23	22	15	27	25	17	8	17	7
11	10	13	18	21	19	13	20	11
13	11	14	22	19	22	16	19	16
15	16	11	21	26	22	8	9	16
19	14	18	18	13	10	16	26	19
15	14	8	14	13	15	12	15	18
14	19	14	16	13	13	13	14	16
15	17	13	16	12	15	16	19	21
17	17	16	20	20	21	11	16	15
10	7	21	49	52	36	1	2	2
21	21	15	16	17	15	9	16	10
13	19	14	31	29	32	1	4	0
15	16	22	16	19	19	9	15	9
15	15	14	26	21	22	8	12	8
14	13	13	11	12	15	18	18	19
7	4	0	7	8	5	11	6	16
13	16	14	22	19	19	7	12	9
14	14	15	24	28	28	4	10	2
22	23	17	19	27	26	4	10	7

10	8	11	20	25	22	12	15	16
17	18	15	19	17	17	9	12	11
14	15	9	30	16	24	10	11	8
6	6	11	14	12	6	13	12	15
6	25	40	11	1	3	45	22	19
13	18	18	21	14	16	6	15	7
13	18	18	22	18	24	6	17	4
12	12	16	37	25	30	0	8	5
13	20	19	16	17	17	6	13	11
12	13	12	27	21	23	3	8	6
15	14	7	13	7	14	13	16	14
17	17	14	18	19	20	6	9	7
17	20	13	12	12	10	11	18	16
15	12	8	16	9	9	13	16	14
16	11	12	25	23	24	4	11	8
14	20	16	20	15	15	10	12	5
11	11	10	19	18	16	8	9	7
7	8	11	16	17	5	15	10	11
7	2	4	4	5	3	24	12	27
14	13	11	17	21	16	10	12	13
19	14	18	30	21	24	1	9	0
14	15	5	14	13	17	11	13	11
11	15	19	23	22	21	6	13	4
13	15	13	9	9	8	12	14	12
11	13	15	19	16	18	6	16	6
14	10	12	13	16	15	6	9	9
7	9	15	13	20	25	10	12	10
38	34	27	47	34	34	36	54	47
11	10	13	11	11	9	11	16	14
22	28	21	25	27	23	16	24	19
19	18	13	10	15	12	11	15	14
9	10	21	17	8	12	15	13	12
12	12	13	11	12	11	9	17	12
8	12	15	20	25	25	2	9	3
14	12	11	12	15	10	3	12	2
14	17	9	15	13	16	8	6	8
8	17	29	15	3	0	37	11	8
11	15	22	15	22	21	6	10	5
12	17	14	17	14	17	7	6	2
7	5	5	4	6	6	15	10	15
9	10	10	14	15	19	4	12	6
8	8	7	12	16	16	8	9	9
14	18	16	23	20	23	3	6	4
9	8	7	14	14	15	8	16	21
11	12	13	12	6	9	9	16	15
10	7	5	4	3	4	20	15	25
13	11	7	7	9	8	14	11	15

10	12	13	21	14	20	8	7	12
14	14	8	21	12	17	7	5	7
13	14	13	15	13	11	7	9	9
5	6	2	1	1	0	21	14	23
9	10	20	25	22	16	3	8	4
18	16	13	19	22	17	19	17	22
7	8	11	9	13	13	4	11	5
10	8	6	11	11	12	8	11	10
7	12	14	18	21	25	1	9	2
12	13	13	17	15	17	5	5	0
11	11	13	17	17	15	3	8	2
13	9	14	4	6	4	27	21	19
12	12	13	16	10	11	7	10	10
6	9	10	11	6	11	6	9	8
11	15	15	10	9	10	9	12	11
11	13	20	11	7	9	12	11	7
11	11	12	13	11	10	4	8	7
10	11	15	7	6	6	13	11	12
8	8	10	20	11	15	7	7	4
7	0	0	0	0	0	15	12	27
9	8	7	11	13	12	10	14	11
5	8	9	23	19	24	3	4	3
10	9	9	12	7	10	15	19	26
8	9	10	9	10	13	9	12	7
7	14	5	17	11	16	1	3	0
11	14	10	11	13	13	6	11	9
13	15	13	11	13	10	8	12	6
6	5	3	19	11	10	4	7	8
10	11	8	8	8	9	9	13	11
14	10	10	14	6	16	5	19	8
8	13	7	11	16	5	3	5	3
8	10	9	18	14	14	3	6	5
6	5	5	8	12	14	10	16	7
11	10	8	10	18	14	8	5	7
10	11	7	8	12	6	10	15	14
12	12	7	15	14	22	1	8	3
35	36	27	35	33	33	31	37	40
23	24	22	11	10	8	29	27	28
12	12	13	13	16	11	2	7	6
11	9	11	9	12	13	5	9	9
7	7	12	11	9	8	6	12	12
9	10	8	10	14	13	4	10	5
12	10	10	10	10	11	4	14	10
10	13	9	9	12	10	10	11	14
8	5	7	10	10	9	8	13	9
7	10	8	6	11	8	7	13	9
8	6	7	7	6	6	9	15	8

12	8	8	30	9	28	1	11	2
7	8	8	12	14	10	3	6	4
12	17	14	14	13	14	6	8	6
8	10	10	10	12	11	7	9	5
8	12	9	19	12	15	0	3	1
10	12	11	7	13	13	8	12	8
9	11	9	19	21	21	4	5	2
12	15	9	15	12	10	3	9	3
11	9	9	14	12	16	4	7	3
5	6	10	10	6	7	17	5	7
7	5	2	6	8	5	7	5	15
10	14	12	9	8	9	3	6	5
8	14	10	11	11	13	3	9	6
7	4	8	24	17	22	1	7	1
9	8	4	13	13	14	1	5	3
9	9	5	7	7	8	7	12	15
12	9	8	10	13	8	6	12	7
14	18	3	15	13	13	4	6	14
6	5	5	7	6	5	7	10	13
9	12	6	13	10	11	5	8	5
7	8	7	5	8	7	9	7	12
7	8	9	7	10	8	11	12	8
7	5	9	6	9	9	8	14	10
14	17	8	13	7	9	8	5	12
8	11	9	5	11	8	7	6	7
10	9	8	13	8	8	14	15	19
9	5	5	5	5	5	10	14	13
9	8	2	7	8	9	6	10	13
9	11	10	12	16	12	4	9	5
7	5	7	10	7	9	4	7	3
1	1	2	10	7	2	3	5	16
7	8	10	9	9	10	7	9	9
8	11	11	13	7	9	1	5	4
3	8	8	7	7	6	10	21	11
10	7	9	8	12	11	6	5	6
10	10	9	6	10	11	9	12	14
9	6	5	5	7	6	10	6	7
8	6	8	16	10	9	3	7	7
7	9	8	18	19	17	1	5	3
6	6	7	9	10	9	7	6	5
6	7	4	12	13	16	1	4	2
3	8	11	13	5	16	1	2	1
9	10	5	9	12	12	0	9	0
9	9	5	5	10	10	6	6	4
8	6	5	14	13	9	4	8	6
6	7	6	8	9	7	4	6	6
8	8	13	8	10	11	1	5	3

9	7	6	6	5	5	11	6	8
14	14	11	14	16	13	3	4	7
9	10	5	8	7	4	8	9	11
11	14	6	7	10	9	3	11	3
7	10	9	10	11	8	3	8	3
5	10	10	10	5	6	6	13	5
3	3	4	10	6	6	6	3	9
8	10	12	10	6	10	5	7	3
9	10	12	11	10	9	6	7	6
2	3	4	2	6	4	11	6	9
4	3	7	8	9	11	10	5	1
6	5	6	13	6	12	3	10	5
7	3	5	1	22	1	1	11	1
8	9	10	11	9	10	5	8	3
10	5	0	13	15	13	19	15	16
7	8	6	10	12	10	4	5	4
5	4	6	6	10	9	3	5	5
0	2	6	1	0	1	3	6	15
6	6	4	7	6	10	5	9	3
5	6	1	6	6	5	7	12	4
11	15	15	13	14	12	0	3	1
8	9	11	13	8	10	2	10	3
4	8	5	4	5	8	11	8	13
6	5	8	13	4	14	5	5	4
4	3	6	6	7	8	8	6	9
1	2	2	5	3	2	7	8	6
4	9	5	11	9	12	5	9	4
4	5	4	8	7	7	3	5	2
4	7	10	5	9	7	3	17	4
8	9	8	9	9	10	9	8	5
9	9	8	9	11	10	11	13	15
8	8	6	7	9	8	5	11	8
8	10	8	11	10	10	1	4	3
3	2	2	5	8	4	10	9	6
11	7	6	6	11	6	3	7	6
11	9	5	16	13	15	0	5	1
5	3	9	11	7	6	4	6	9
5	3	5	7	6	8	5	5	7
6	8	7	8	9	9	5	1	6
10	10	5	2	3	2	9	8	11
7	7	10	15	12	12	1	5	1
5	1	0	1	9	4	8	7	13
2	3	7	4	6	5	8	9	9
8	12	4	12	7	5	3	10	2
6	4	8	13	11	14	3	5	1
3	2	1	4	6	9	8	13	10
7	13	15	10	10	8	3	5	2

7	7	7	10	6	11	4	4	6
15	9	12	7	8	6	24	20	22
7	3	3	3	4	5	8	6	7
3	3	2	9	9	10	5	7	6
3	5	5	13	5	7	2	2	3
1	5	8	7	13	10	1	9	0
4	5	3	6	7	6	4	8	10
5	4	6	9	8	7	5	8	4
11	13	11	6	7	6	4	7	3
4	4	3	11	15	14	1	5	1
10	5	2	7	5	7	4	7	8
8	8	6	11	12	13	2	2	1
7	6	4	11	9	11	2	5	0
8	6	8	11	8	9	3	7	2
6	6	7	7	4	5	7	7	6
6	7	4	7	10	9	5	8	7
3	4	3	5	2	3	6	6	8
2	2	4	4	5	10	9	5	9
9	8	7	8	6	7	6	4	5
5	4	3	4	3	4	6	4	10
12	14	13	20	21	16	4	5	4
6	7	7	4	5	4	7	7	7
4	3	6	7	7	4	2	3	2
8	6	3	10	9	10	0	5	3
2	5	3	6	7	6	7	8	6
5	5	9	10	8	9	2	7	3
4	6	5	7	7	8	4	4	3
3	3	5	4	6	9	4	6	9
6	9	5	3	8	6	7	6	12
8	9	4	10	5	7	2	7	2
2	3	3	7	17	12	2	3	2
4	0	0	4	4	0	10	13	7
14	12	11	9	8	9	0	0	0
7	6	13	9	5	6	4	8	4
7	4	7	4	3	5	7	9	10
3	2	1	0	3	3	8	7	10
5	7	6	12	5	7	2	5	1
6	6	0	8	7	6	7	5	3
6	8	4	6	9	7	2	5	4
6	4	7	8	5	5	2	9	6
4	3	6	5	5	6	4	4	4
3	3	4	5	7	6	2	6	7
4	7	7	6	5	7	3	6	5
7	8	8	9	7	7	0	2	3
7	5	7	6	6	5	6	5	7
21	25	24	34	37	43	10	20	0
6	4	3	2	3	3	7	4	8

3	3	1	2	2	1	11	8	7
6	6	7	11	5	6	4	3	6
3	3	12	12	6	7	1	6	2
6	4	3	6	2	5	5	6	8
2	4	2	2	4	3	3	5	10
5	5	5	7	9	10	2	4	1
4	5	4	4	5	4	8	6	7
4	8	7	6	11	10	0	0	2
5	4	1	2	2	5	4	2	5
3	3	6	6	5	6	6	4	7
4	1	5	4	5	6	4	2	4
2	3	1	9	2	3	0	1	5
5	6	1	5	8	5	4	4	7
5	5	6	8	5	6	3	6	2
2	5	3	7	8	12	0	8	2
3	1	2	3	0	0	3	3	8
2	3	2	4	5	6	10	4	6
6	6	6	4	3	3	5	7	6
6	5	9	6	7	5	4	4	4
6	8	4	6	4	7	4	7	6
8	6	6	3	3	6	1	4	2
2	2	1	5	8	7	2	8	2
3	2	2	6	6	5	3	5	5
3	3	5	7	4	7	1	5	3
5	5	4	3	3	4	3	4	9
8	7	4	8	6	9	1	5	3
3	4	4	8	7	8	0	5	1
3	3	3	3	8	5	5	7	5
4	9	4	5	9	8	1	5	2
0	0	0	0	0	0	11	6	18
6	6	4	8	4	6	2	6	5
5	5	6	5	7	10	2	5	2
3	3	4	3	3	5	2	4	7
3	4	2	7	13	13	3	3	2
6	2	1	6	3	3	3	6	8
7	9	4	11	7	6	2	1	4
6	6	3	4	3	4	6	4	4
6	7	3	7	7	7	3	4	3
5	6	6	3	8	4	4	5	2
5	7	4	9	12	10	0	2	4
5	6	9	6	7	10	3	4	1
2	3	9	4	3	0	24	6	3
7	8	6	4	7	1	8	12	10
7	7	6	5	8	5	3	5	4
6	3	4	5	4	5	4	5	5
5	6	3	10	11	11	2	5	1
4	1	2	10	3	4	0	0	3

0	0	1	5	2	9	1	3	3
2	1	6	4	3	4	4	4	3
4	5	5	6	6	4	5	9	5
6	3	3	1	1	2	5	8	7
6	5	2	8	7	9	5	6	7
9	6	5	5	7	5	4	5	6
5	6	4	4	7	5	5	4	2
3	0	1	6	2	8	6	8	11
7	3	3	2	5	2	3	3	6
4	3	2	2	3	2	6	5	9
3	4	0	5	4	4	5	3	7
3	5	3	8	9	8	2	3	2
4	4	4	8	9	9	1	2	3
3	3	1	4	1	4	4	7	6
4	5	13	6	9	5	4	6	3
2	4	11	6	8	6	0	6	0
4	5	4	7	4	7	2	5	3
4	6	4	5	4	6	3	4	5
6	5	2	1	1	1	3	7	6
7	7	3	8	5	9	1	6	3
2	5	3	4	4	4	3	3	3
3	4	2	11	7	6	0	1	1
3	3	6	7	11	10	1	0	1
4	9	5	4	6	5	4	3	3
4	3	2	1	3	0	7	6	5
6	6	11	5	7	11	2	2	0
3	4	8	10	7	9	0	4	0
6	4	6	10	6	9	2	4	2
5	5	7	12	9	13	0	0	0
4	4	2	7	8	7	4	3	4
5	5	4	4	5	6	1	5	3
5	4	5	8	4	7	3	4	4
3	1	1	4	1	3	5	7	6
3	5	6	3	7	6	4	5	6
3	3	2	4	3	5	4	5	2
2	4	3	4	5	5	4	1	4
3	4	6	6	5	8	1	6	4
5	6	8	6	6	7	1	3	3
3	6	17	0	0	0	21	5	0
6	5	7	5	5	5	4	5	5
2	0	2	4	2	3	3	4	8
4	8	4	7	6	4	0	1	1
2	3	7	4	6	4	2	3	2
1	2	4	3	2	2	4	5	5
3	2	2	2	6	6	2	2	2
0	1	1	0	0	0	3	1	11
2	4	2	10	5	10	2	3	2

5	5	6	4	6	7	0	5	1
4	8	5	15	18	15	0	1	1
1	5	6	13	11	15	1	1	0
5	5	4	3	2	4	5	5	6
3	4	5	5	5	5	1	2	3
4	3	2	2	5	6	7	6	6
5	6	0	3	2	3	8	4	8
2	2	4	3	3	4	3	3	7
1	3	2	11	6	6	4	5	3
5	4	2	2	5	2	5	2	5
8	8	9	6	2	5	1	3	1
2	2	3	12	3	9	1	1	2
4	3	6	5	7	8	0	1	0
8	8	4	6	7	10	0	1	0
0	1	2	0	0	0	4	1	9
4	3	4	6	6	5	3	3	2
16	21	18	15	19	13	9	14	13
3	3	5	9	8	3	0	2	1
1	1	0	0	1	1	0	3	5
3	4	3	5	6	6	2	2	3
4	4	5	7	6	6	0	4	1
3	2	3	4	4	3	1	3	4
7	7	11	9	5	12	2	4	2
3	2	5	7	5	4	2	2	2
4	2	0	2	5	2	5	2	4
0	0	2	0	0	0	3	4	11
1	0	0	0	10	0	7	6	4
3	4	2	6	5	5	2	4	3
6	6	3	3	2	3	2	5	4
3	2	1	1	1	2	5	5	8
6	3	2	3	6	8	1	2	3
1	3	1	5	3	5	4	5	4
1	2	5	10	6	7	0	1	0
1	2	4	1	3	0	4	4	6
3	3	1	4	4	6	4	5	3
3	5	5	3	3	2	5	4	3
4	4	1	2	1	1	5	7	6
5	2	4	8	2	4	1	6	1
2	3	5	7	8	7	1	1	1
8	8	2	5	5	5	0	2	2
3	1	7	6	5	6	1	4	1
2	3	1	9	2	9	1	2	0
2	4	3	3	6	4	1	4	3
5	4	4	4	2	3	3	3	3
6	8	7	7	9	7	3	6	3
5	6	7	3	4	2	2	1	2
4	3	4	4	5	3	1	2	2

1	1	2	3	3	2	3	4	3
3	4	5	2	4	2	2	4	3
1	4	2	2	6	4	3	4	4
2	2	0	2	3	2	3	4	5
5	5	5	4	3	2	0	2	1
6	7	6	2	1	2	3	4	0
10	7	7	21	16	12	4	10	7
2	2	0	8	8	9	3	1	1
4	5	4	2	3	2	3	3	6
25	22	21	27	27	28	24	26	30
3	2	1	4	1	3	3	6	6
5	3	5	2	6	7	1	3	2
6	6	4	2	4	4	4	5	3
2	1	1	6	2	3	2	5	1
4	1	2	2	2	2	5	4	1
1	0	1	0	2	1	2	2	3
10	0	0	0	0	0	14	7	5
2	2	1	5	2	4	4	4	5
1	4	0	1	1	7	2	2	0
1	5	4	4	4	5	2	4	2
2	3	1	3	1	1	4	4	5
2	2	1	4	4	1	1	1	3
1	2	3	8	4	7	1	3	2
2	2	2	5	3	6	1	1	0
3	3	0	2	3	1	2	4	5
2	3	1	9	3	5	1	3	1
2	2	4	5	7	5	1	2	0
3	2	2	2	3	1	2	3	3
4	6	8	7	6	6	0	0	1
1	1	3	6	5	5	0	2	0
1	1	1	2	5	4	2	3	1
1	3	2	4	1	1	1	5	4
3	1	2	1	1	1	1	1	1
5	2	3	6	3	5	2	1	0
2	3	2	1	6	3	1	4	1
1	1	5	3	3	2	3	3	5
2	3	4	3	2	3	2	3	2
2	1	1	3	1	1	4	3	5
3	5	3	4	6	4	2	2	2
1	1	4	5	3	7	1	1	1
3	3	3	7	4	3	0	2	1
3	3	1	3	5	7	0	3	1
3	3	5	7	5	4	3	2	2
1	1	0	2	3	3	3	1	2
2	2	0	2	2	3	1	1	2
0	0	2	1	2	3	1	2	3
1	1	0	1	1	2	6	3	10

1	1	0	1	2	1	5	1	5
3	5	2	3	4	4	2	2	2
3	5	2	2	3	5	3	5	6
8	8	0	0	4	7	9	7	10
15	13	16	17	16	15	13	15	23
3	2	3	3	1	2	4	2	3
2	4	4	8	7	3	1	1	1
3	4	4	4	2	1	1	3	1
2	1	1	5	1	2	1	3	4
1	0	1	0	1	0	3	2	3
0	0	0	1	2	0	2	8	4
2	2	1	2	1	1	2	2	4
3	2	1	8	3	5	1	2	1
1	3	2	7	1	4	3	3	3
4	4	3	5	4	3	0	2	0
1	3	1	3	1	6	0	2	1
9	7	0	0	11	0	0	14	12
0	1	1	1	1	0	2	2	4
0	1	0	5	2	4	1	3	5
1	1	1	5	6	6	1	2	0
4	5	2	6	5	2	1	2	1
5	4	4	2	1	2	3	3	5
4	2	4	3	4	5	0	3	0
1	2	1	4	4	7	1	0	0
2	0	0	5	4	5	0	5	0
6	6	5	5	4	7	8	7	9
1	2	2	4	5	3	1	3	1
4	2	3	5	5	4	1	1	1
0	7	6	7	6	6	6	6	8
1	0	1	1	3	0	1	1	5
0	0	0	0	1	0	3	4	3
5	7	3	5	2	4	0	3	0
4	5	0	6	2	12	0	2	0
0	0	0	2	4	2	1	1	1
3	4	2	0	4	6	0	7	0
7	6	3	1	5	3	2	4	1
2	2	5	3	3	6	1	3	1
1	5	5	4	4	7	1	2	0
0	0	0	1	0	0	4	3	9
4	6	2	8	8	10	0	1	0
1	4	0	2	5	3	2	1	1
0	1	1	4	3	2	1	1	1
1	0	0	1	1	4	1	2	3
4	5	3	2	2	2	2	2	5
2	3	2	5	4	5	1	1	1
0	1	2	5	2	2	1	2	3
1	3	3	5	3	5	0	1	2

2	1	8	2	3	5	0	2	1
3	4	2	6	4	5	0	1	0
0	1	0	1	2	0	6	0	2
2	2	4	9	6	7	0	3	0
4	5	1	2	1	3	0	4	1
0	2	2	8	15	7	0	2	0
0	0	2	4	1	0	0	0	2
2	2	2	2	2	1	6	3	3
2	1	0	0	2	0	6	3	2
1	1	0	0	1	0	4	2	2
0	0	0	0	0	0	6	5	10
2	3	3	5	3	4	1	1	0
0	0	0	0	0	1	1	3	9
3	4	3	5	5	4	1	3	1
1	2	2	2	2	1	2	2	2
4	4	1	2	3	3	1	1	1
0	2	0	3	1	5	2	3	1
2	2	2	2	3	5	0	1	2
0	0	0	5	5	6	1	0	0
0	0	2	1	1	2	2	2	2
1	2	0	0	3	0	5	3	8
0	4	7	3	0	0	0	9	16
0	0	0	0	0	1	2	1	4
1	1	0	1	0	1	0	0	1
3	3	5	1	2	2	1	0	0
1	0	0	0	0	0	4	5	5
2	0	1	0	0	0	6	4	7
0	0	0	0	2	1	3	0	3
20	21	18	0	18	17	21	22	31
2	5	2	4	5	6	4	3	4
0	1	0	0	3	4	0	3	0
0	0	8	11	11	9	9	0	11
0	0	1	5	0	3	0	1	0
0	3	5	0	0	0	14	0	0
0	15	0	25	15	16	13	15	0
0	2	0	0	0	0	4	0	6
1	2	3	1	3	3	3	4	6
0	3	0	0	0	0	12	5	0
0	0	0	0	1	0	2	0	1
12	9	15	0	6	4	23	20	20
5	4	4	0	8	9	4	8	5
0	0	4	6	6	6	3	6	3
0	0	0	0	5	3	3	3	3
5	4	0	8	0	8	0	12	0
9	0	0	0	0	15	0	0	15
0	0	6	17	8	11	0	6	5
0	0	4	7	0	0	7	0	6

3	6	0	0	4	3	7	6	8
3	3	5	0	0	0	4	5	5
9	10	15	10	0	0	14	12	11
4	0	0	8	3	3	4	0	0
0	0	3	7	3	3	3	0	0
0	0	15	10	0	0	15	13	0
0	0	23	29	0	0	0	28	0
0	0	2	5	0	6	4	0	4
0	3	0	0	3	4	0	5	9
6	7	5	9	0	7	4	0	7
0	0	0	0	6	0	21	15	0
6	0	0	0	0	0	0	0	11
0	2	0	0	0	7	0	0	5
43	47	40	0	0	0	0	0	0
0	0	0	0	0	0	0	0	0

VancATN T16	VancATN T17	VancATN T18	VancATN T19	ATN T24.norme	ATN T25.norme	ATN T26.norme	ATN T27.norme	IgAN E0 T8.normed
62	52	59	60	54	50	90	90	41
60	48	57	32	54	51	63	56	48
64	60	65	48	47	49	65	58	36
75	85	70	44	73	51	56	58	41
47	60	48	36	44	40	46	42	41
58	47	59	39	56	42	52	48	42
22	40	19	15	23	27	20	18	55
58	32	54	32	52	41	66	71	16
50	42	34	24	47	30	43	40	38
28	42	28	26	22	20	35	20	36
42	35	45	48	58	34	54	39	16
37	40	64	41	58	35	49	41	42
34	41	26	28	36	31	33	30	49
34	55	32	27	31	31	29	30	30
34	38	34	28	38	32	30	27	40
50	50	42	33	44	37	46	46	27
51	26	32	39	35	30	65	59	20
30	33	19	22	29	27	28	28	41
38	41	37	36	34	32	35	38	34
33	48	39	23	31	34	27	26	39
35	33	32	25	36	32	50	39	22
28	26	32	28	35	21	23	24	38
20	34	22	18	22	19	14	16	44
18	29	30	13	18	20	16	15	50
13	36	23	19	21	24	15	10	32
24	27	24	24	31	23	21	21	31
22	29	21	19	25	20	15	19	39
23	32	23	20	22	25	21	22	28
31	31	27	25	30	24	27	31	32
23	33	21	18	23	21	21	25	26
23	35	20	21	31	25	27	23	19
26	30	19	12	21	16	23	18	26
24	28	26	20	25	18	18	17	31
4	3	5	4	6	5	5	4	2
28	27	32	26	43	20	28	30	19
15	25	23	13	13	25	12	17	33
23	27	24	23	26	20	30	27	16
22	21	22	19	26	20	25	25	18
26	3	9	9	30	15	11	7	8
21	31	22	18	18	24	24	19	24
22	30	17	17	22	18	19	18	29
21	34	23	17	18	24	19	16	18
32	0	4	7	32	21	17	6	13

26	19	22	22	31	16	24	21	17
27	13	23	19	26	28	32	32	27
26	30	15	11	23	15	9	16	30
13	25	12	21	13	18	5	10	24
20	23	18	15	13	17	21	22	17
19	22	15	15	21	20	15	16	20
23	26	21	15	22	25	30	23	14
29	29	23	23	29	19	30	24	16
28	12	17	18	25	21	30	35	4
16	20	13	11	14	15	14	13	21
39	52	25	17	51	27	46	38	6
30	32	26	15	16	24	31	21	11
24	20	20	17	26	14	25	24	17
31	35	32	23	16	21	24	22	15
31	19	27	26	36	19	38	38	8
29	14	17	17	18	19	39	30	4
5	15	14	5	14	13	10	10	25
13	20	12	17	14	12	12	14	17
14	18	16	11	18	13	17	17	15
12	23	7	5	1	19	4	8	23
19	22	17	10	10	20	30	18	15
10	5	9	9	10	9	3	1	21
20	18	13	14	10	15	22	18	14
12	28	15	12	14	14	9	13	18
26	27	19	13	17	20	24	24	15
12	19	13	10	10	13	5	8	20
13	18	10	9	10	16	13	11	20
30	38	27	21	16	19	42	22	0
11	21	14	12	12	16	10	13	19
21	17	13	14	25	23	13	21	11
22	17	19	9	16	12	20	19	11
11	19	19	9	16	18	13	12	18
18	16	12	12	10	9	17	14	10
18	17	17	11	14	19	22	19	15
16	18	15	13	12	14	12	12	19
12	13	14	12	19	9	14	13	14
15	19	16	13	22	14	13	14	14
4	26	14	3	8	7	8	9	9
13	14	11	8	9	11	8	12	21
11	12	14	3	3	19	8	8	20
15	12	12	11	14	14	14	11	16
12	22	14	9	14	13	14	13	13
18	15	22	10	18	16	20	21	13
10	4	13	9	12	13	27	28	1
12	15	14	6	16	10	11	13	14
10	18	7	8	8	11	7	11	15
10	16	11	10	9	15	7	10	19

20	22	20	13	16	16	25	13	11
13	17	14	10	14	11	10	8	19
15	17	18	11	13	16	13	12	16
18	28	26	18	16	13	22	15	11
5	0	4	0	10	6	6	1	8
12	10	11	10	12	11	10	12	14
12	20	11	5	10	11	6	11	24
10	20	15	6	5	20	2	9	11
12	15	14	11	9	15	14	15	12
18	16	10	11	17	14	11	19	10
13	14	23	10	27	8	20	17	10
13	16	12	4	9	13	8	10	18
8	10	9	9	10	8	10	9	17
16	13	17	15	30	13	20	18	12
9	19	9	6	8	22	7	7	14
6	13	6	6	6	11	6	6	16
12	15	8	9	23	16	12	14	13
17	22	15	16	9	8	20	11	10
20	5	15	14	39	5	28	22	2
16	24	13	12	14	16	19	14	10
6	19	7	7	6	11	2	8	18
13	14	11	13	12	11	10	11	13
17	15	9	5	10	16	9	5	12
9	9	10	8	10	8	11	12	15
11	14	9	9	16	14	10	11	15
14	15	10	4	12	11	8	6	11
13	12	10	10	13	16	11	13	13
43	40	45	31	32	35	49	52	30
18	14	19	11	9	13	14	12	5
23	28	25	19	19	20	24	21	23
10	9	13	10	10	7	7	8	15
16	13	9	10	9	11	15	12	8
11	11	16	7	14	12	15	10	10
12	17	11	6	6	10	7	11	13
5	10	8	6	9	11	10	8	6
9	15	8	9	9	7	10	12	11
33	0	0	9	44	18	11	6	5
6	9	5	2	3	6	3	5	17
6	13	9	8	14	13	5	12	11
14	19	13	13	21	13	28	15	4
10	14	8	9	9	10	9	10	10
15	15	18	9	13	15	14	12	11
4	12	4	7	3	5	6	2	18
11	8	13	8	9	9	9	11	8
14	11	13	11	16	13	14	13	9
13	9	10	12	17	8	15	23	5
11	11	13	12	8	12	13	16	10

13	14	8	9	9	7	9	9	8
8	17	6	11	6	6	8	8	12
9	14	9	9	13	7	11	11	11
24	14	10	21	25	5	39	33	0
7	19	11	5	4	8	9	6	8
16	24	20	15	18	20	23	22	16
17	18	7	8	12	9	7	10	11
12	11	10	10	14	9	12	13	9
8	22	6	5	4	11	4	5	11
5	9	6	7	4	7	4	6	12
9	13	8	3	8	11	9	9	11
6	6	7	8	5	7	15	12	8
10	12	7	7	8	7	6	8	10
14	12	18	8	9	13	13	17	11
9	10	8	8	16	11	10	10	13
10	9	7	9	13	9	6	7	11
10	13	14	8	12	12	11	10	10
6	6	7	8	9	7	6	6	11
9	14	10	6	13	11	9	13	10
20	2	10	14	17	1	33	25	0
11	12	9	8	10	11	13	10	11
8	18	6	10	5	10	3	7	7
20	15	21	18	31	20	23	15	10
11	10	9	10	10	13	6	7	11
5	14	15	1	6	8	4	3	13
9	9	10	4	10	7	7	6	11
6	10	7	10	6	5	5	7	15
15	15	13	11	14	11	14	11	6
7	8	8	12	6	8	16	16	4
13	9	6	7	3	16	3	10	12
11	17	8	4	3	14	6	4	16
17	13	13	2	9	9	7	9	8
13	11	14	7	12	12	11	14	3
11	12	11	9	14	9	10	10	8
5	9	11	8	13	10	15	11	12
9	14	6	6	8	10	5	8	11
35	34	28	27	27	25	29	28	36
9	9	9	9	9	7	11	11	15
7	9	10	4	5	10	10	5	11
12	15	8	8	12	8	8	9	8
11	9	4	6	6	5	12	9	7
10	11	10	11	6	9	10	9	7
14	10	3	7	3	5	12	13	4
6	10	10	6	5	8	8	7	10
7	7	11	9	6	5	6	8	9
16	11	14	6	12	11	17	16	5
9	8	12	13	17	8	10	15	4

5	5	2	1	4	10	2	3	20
8	15	11	4	6	12	7	5	8
5	10	4	3	3	5	5	6	10
8	11	5	7	9	6	8	6	11
5	16	6	5	4	8	5	7	11
6	8	6	6	9	6	5	8	10
6	9	7	3	3	8	3	2	9
5	12	7	3	5	10	5	4	14
5	11	6	5	8	6	6	7	7
10	0	11	8	16	8	0	7	5
10	13	10	8	13	10	10	9	4
6	10	4	5	4	5	4	2	13
8	11	6	5	9	8	5	8	9
10	11	7	5	9	5	2	2	7
8	8	5	6	8	9	6	3	8
8	11	8	8	6	6	11	10	5
6	7	6	6	5	6	6	4	10
5	7	8	3	6	9	4	6	17
6	6	11	9	16	8	12	9	6
7	6	7	3	9	9	6	8	11
9	7	10	8	10	5	14	11	4
7	7	7	7	6	6	6	8	9
7	10	10	7	9	9	8	10	4
7	7	3	4	9	5	3	2	8
7	9	7	8	12	8	11	8	8
9	8	6	8	10	5	12	13	11
10	6	4	11	5	6	11	12	9
8	3	10	6	8	2	9	8	2
7	10	5	6	6	6	6	7	11
13	11	13	8	9	9	10	9	8
8	31	8	10	3	14	25	8	1
6	10	6	5	5	8	6	7	5
9	9	3	4	1	3	2	5	12
11	4	16	6	1	7	8	14	3
9	9	7	8	9	9	7	8	11
12	10	13	14	6	8	12	8	9
9	5	5	9	9	9	7	15	5
9	13	4	5	6	7	7	6	7
9	12	5	4	6	9	2	5	8
7	11	6	5	9	8	9	7	5
7	4	6	4	8	7	9	5	8
5	11	9	1	3	11	4	3	11
10	14	4	2	12	27	2	2	5
8	12	10	8	4	12	7	6	8
9	11	6	4	6	6	7	6	6
12	4	9	3	8	12	9	11	6
8	13	8	3	8	7	6	5	10

11	9	9	9	6	7	8	9	4
8	19	9	3	6	8	11	8	10
6	2	5	5	9	6	7	6	6
5	5	5	4	3	5	6	8	10
3	10	7	4	3	9	6	3	10
6	4	4	4	3	5	3	4	8
13	11	13	8	5	12	13	13	4
3	5	3	6	6	5	3	5	4
5	7	4	3	5	5	3	5	7
9	5	13	7	6	6	9	11	6
10	12	7	9	13	9	8	8	6
9	12	9	3	0	5	14	8	4
5	21	11	7	1	16	0	0	8
5	7	3	3	4	5	5	4	6
26	13	19	20	27	16	35	30	5
6	9	7	4	6	5	4	5	7
9	10	4	0	6	5	4	7	7
13	20	16	16	10	3	14	12	1
5	8	7	3	6	5	7	6	7
7	10	6	2	6	5	11	9	4
4	7	1	1	3	5	1	2	12
9	9	2	3	3	5	4	5	7
6	7	7	8	9	6	7	8	6
2	10	7	5	4	6	3	4	8
9	9	8	7	9	6	13	15	5
12	7	7	10	8	10	19	21	0
11	12	6	5	4	9	6	5	5
5	7	8	6	9	8	4	4	10
9	14	9	7	5	6	8	7	6
5	4	4	4	6	5	6	5	7
11	12	9	11	9	8	14	7	9
5	5	6	6	6	6	6	5	6
2	8	2	3	3	4	1	5	10
12	9	11	8	6	9	11	14	2
7	7	8	11	5	9	6	4	4
3	13	2	3	4	4	2	3	6
9	7	7	6	13	7	8	7	5
10	9	10	5	10	6	9	11	3
4	11	6	9	9	9	7	9	4
3	4	4	2	4	4	5	3	5
6	13	11	0	1	7	6	3	5
7	4	7	8	6	11	9	10	0
9	7	9	8	4	8	6	9	4
4	9	5	2	4	5	3	2	8
5	9	2	4	6	9	7	5	4
11	3	11	5	8	5	13	15	1
3	3	5	4	5	4	3	4	7

8	12	4	1	6	5	4	3	5
10	8	11	8	10	11	14	13	8
7	4	6	6	6	4	8	8	5
7	6	11	4	9	8	9	7	4
10	9	6	5	8	7	6	8	5
9	7	4	5	9	7	1	7	7
4	6	4	6	4	6	7	6	9
8	5	6	7	6	5	5	4	5
4	3	5	4	4	4	3	5	10
7	4	2	4	6	9	6	8	8
4	3	8	3	5	3	2	3	4
7	5	4	5	3	5	4	4	7
7	12	8	6	3	5	4	7	6
6	6	6	7	8	6	9	6	4
5	5	5	6	10	5	6	8	5
4	4	5	3	4	4	6	4	5
8	7	5	7	8	5	7	9	5
7	7	9	7	8	9	9	8	4
4	6	3	3	4	4	3	4	6
7	8	10	9	12	7	6	9	2
7	12	9	5	12	6	8	6	12
3	4	2	4	9	5	4	4	6
8	7	4	2	3	6	6	8	8
8	5	5	1	1	5	0	3	6
10	8	3	6	5	5	7	7	5
7	7	5	2	4	7	3	6	6
9	9	7	6	5	5	3	6	4
6	5	5	5	4	5	6	6	4
2	2	4	4	8	6	6	7	5
3	6	6	0	4	8	3	3	6
5	8	8	6	6	5	6	5	2
10	6	18	14	32	15	18	16	4
1	8	0	0	3	2	1	2	11
4	7	3	1	3	2	2	3	6
5	3	4	3	6	5	8	6	4
11	5	7	13	5	3	12	12	2
9	10	7	3	6	5	8	8	4
6	6	7	7	6	1	6	9	0
6	6	6	1	4	5	4	5	7
6	7	5	1	1	5	6	4	7
9	8	7	6	5	5	7	8	3
8	5	9	5	8	5	8	10	2
7	6	6	8	4	5	7	6	3
3	6	2	3	1	1	1	3	12
4	11	2	2	4	3	5	6	7
21	32	18	17	22	17	17	21	22
8	4	7	4	12	6	8	8	4

5	1	8	7	9	7	13	14	4
2	3	3	3	0	0	3	2	13
3	8	3	4	3	5	2	2	7
8	5	8	4	8	3	7	9	4
10	5	6	6	12	7	11	11	4
7	8	4	2	3	5	4	4	4
6	3	5	6	3	5	6	4	5
0	5	0	3	3	0	5	5	8
3	6	6	7	9	5	9	7	3
5	5	6	4	5	5	4	6	2
8	8	6	3	5	5	8	6	1
4	14	7	7	6	3	10	6	0
3	6	5	1	6	3	7	4	4
7	7	4	3	3	3	3	3	6
2	7	2	0	1	8	3	1	7
15	15	16	6	8	1	11	6	0
10	6	7	7	12	7	9	7	2
5	4	6	4	3	4	5	6	4
3	1	6	2	10	5	4	4	5
4	3	3	3	3	3	4	3	4
7	6	4	1	3	5	2	4	4
4	4	6	6	8	6	9	10	0
5	5	3	6	6	3	7	5	3
3	5	3	2	12	5	4	6	4
6	5	3	2	5	2	5	6	4
5	4	4	1	3	4	3	4	7
5	7	4	2	5	4	2	1	6
6	5	7	10	10	5	7	6	2
6	5	4	4	5	3	5	5	6
6	9	7	7	6	5	7	11	1
8	7	6	3	8	8	4	6	3
5	6	3	3	5	4	4	4	6
5	4	6	3	4	5	5	5	4
5	8	6	1	3	6	6	8	3
5	4	5	5	3	4	6	8	0
2	7	2	0	3	2	2	1	7
4	3	4	5	5	2	4	4	5
4	5	6	4	9	6	6	6	4
5	5	2	5	5	8	2	4	4
3	5	3	2	3	7	6	4	5
2	5	0	2	1	3	0	1	7
8	3	3	4	9	5	5	3	2
2	1	2	2	9	1	3	7	1
5	3	2	2	3	4	5	3	5
5	8	5	3	5	3	2	6	4
5	5	3	2	1	6	3	3	4
1	4	7	4	0	0	8	3	7

10	5	8	6	9	4	9	11	1
4	4	4	2	6	2	6	5	3
4	4	3	2	3	5	3	4	4
5	3	7	9	8	5	9	11	3
2	3	4	1	5	5	3	4	7
5	4	5	1	0	5	4	2	3
4	4	3	2	6	5	5	5	3
7	3	2	3	5	3	8	6	0
5	4	6	9	6	6	8	6	1
2	5	9	4	6	2	4	3	4
6	5	6	5	8	4	7	8	3
3	5	6	3	3	5	3	5	4
4	7	4	2	6	4	4	5	3
5	6	1	1	1	5	5	2	5
6	6	2	6	8	6	5	2	5
4	4	5	3	4	13	2	5	2
5	6	3	4	3	5	4	3	4
3	4	1	1	4	4	2	4	5
5	6	6	3	3	4	8	9	4
1	6	1	0	0	4	0	0	2
9	4	4	3	5	6	4	5	3
4	4	2	4	1	5	2	2	3
4	6	6	3	4	5	5	4	4
5	5	5	2	4	5	4	3	4
5	3	8	2	9	3	18	14	1
3	5	3	1	3	2	1	1	7
0	7	1	0	0	2	2	1	9
2	8	2	2	3	4	3	1	4
0	8	2	0	1	5	0	0	5
5	5	4	1	1	5	3	3	4
3	1	1	1	0	1	2	2	6
2	10	3	1	3	5	3	1	5
7	3	5	6	8	2	5	9	4
5	3	6	1	6	4	5	4	4
5	5	7	1	3	4	6	5	4
6	6	6	4	8	5	6	6	3
7	5	6	1	1	7	6	4	4
0	3	1	2	4	3	0	0	5
9	0	10	0	17	6	0	0	0
2	2	3	0	4	1	2	3	4
12	5	10	3	6	3	7	9	0
1	7	3	1	4	3	3	4	7
4	5	5	3	8	5	4	4	3
3	4	3	4	3	4	4	5	4
5	5	4	2	6	3	4	4	2
15	12	15	10	8	2	11	5	0
2	6	5	1	5	4	2	1	4

4	8	3	2	5	5	2	4	4
0	0	0	0	0	0	0	0	5
1	5	4	2	1	5	4	1	0
3	2	3	4	3	3	4	4	4
3	3	4	2	1	4	4	1	3
4	4	5	5	5	5	5	5	3
4	2	5	4	6	4	8	6	1
5	4	4	5	6	3	8	5	2
7	2	5	1	3	2	1	3	4
3	2	7	6	6	4	3	6	2
2	4	1	1	3	2	1	1	6
5	6	3	1	4	6	3	1	3
3	7	3	3	5	5	2	7	2
0	4	4	1	1	3	0	0	7
12	10	13	8	9	4	13	3	0
4	5	3	1	3	2	4	3	4
15	18	17	12	12	15	14	12	18
4	7	6	2	8	5	5	7	3
6	5	4	8	10	5	7	9	2
2	7	4	3	1	3	1	3	4
4	3	2	1	5	4	2	1	6
6	4	3	5	4	3	3	4	2
2	4	3	2	3	2	4	4	9
4	6	2	5	0	2	4	5	3
4	4	5	5	4	4	7	6	2
5	10	5	6	6	5	4	5	0
0	7	2	0	1	9	0	5	1
1	2	2	2	0	0	2	3	3
7	5	4	0	3	2	3	6	3
8	1	7	4	6	4	10	8	1
3	10	2	0	4	3	3	0	3
4	4	3	2	0	5	3	3	2
2	9	1	1	0	5	4	2	2
3	4	7	6	0	5	5	6	2
2	4	4	2	1	4	4	3	4
2	2	1	2	3	2	4	2	4
3	3	5	3	3	4	2	3	3
2	5	1	0	0	2	1	0	6
4	5	1	2	3	4	1	3	4
3	1	1	2	1	3	2	1	4
5	9	5	1	0	5	1	1	5
3	7	3	0	0	5	1	0	5
2	3	6	5	3	5	1	3	4
2	2	1	2	0	2	2	2	3
0	4	4	2	3	4	1	0	7
3	3	1	5	1	2	2	3	4
6	4	6	3	5	4	6	7	3

6	6	2	3	5	3	7	7	1
3	3	2	3	3	4	5	3	4
3	5	2	1	4	3	5	6	2
4	3	4	2	8	3	6	3	2
1	3	1	2	4	2	1	2	4
2	2	1	1	3	1	1	2	3
10	16	7	5	6	9	8	8	7
7	5	1	5	1	3	3	2	0
3	1	2	3	4	3	1	2	4
28	31	23	21	26	20	23	24	25
7	3	2	1	1	2	8	7	1
3	5	3	0	3	4	4	2	4
2	3	0	2	1	3	4	2	7
7	6	5	5	5	4	6	7	0
2	5	8	1	4	3	1	5	0
9	3	4	6	6	5	8	8	0
5	0	0	3	0	1	0	0	0
5	3	4	3	3	1	3	4	4
3	2	4	1	6	10	1	1	3
4	5	4	3	5	4	5	5	2
3	2	1	6	4	6	5	5	2
6	6	6	2	4	5	10	7	2
2	8	4	1	3	3	3	3	2
6	8	4	1	1	2	1	3	2
4	2	3	4	1	3	3	5	2
5	4	3	1	1	4	3	3	4
5	4	2	3	4	4	3	3	2
5	3	4	3	5	5	3	6	3
0	3	1	3	4	3	0	1	4
3	5	4	2	4	3	3	4	1
4	1	1	1	1	5	9	6	0
3	4	2	2	1	4	4	3	4
4	6	10	3	4	5	5	3	2
2	2	1	2	4	3	0	1	4
2	5	2	1	4	4	1	4	4
5	3	3	5	5	3	3	2	2
2	2	2	1	4	2	3	3	4
4	0	3	6	4	2	9	5	2
4	4	1	2	4	3	3	3	4
4	5	3	4	5	5	5	5	3
2	6	3	0	1	3	1	4	4
4	4	2	1	3	2	2	3	3
1	3	0	2	1	2	0	0	2
5	4	3	3	4	4	7	7	1
3	4	7	7	9	5	5	5	0
2	2	4	2	0	2	7	5	1
2	4	2	4	5	2	6	7	3

4	4	6	7	6	5	9	9	1
4	4	2	3	3	3	3	3	3
2	2	4	2	4	3	2	4	2
11	6	8	3	8	5	10	8	8
19	17	15	16	23	13	20	19	13
4	4	6	2	3	3	7	6	1
1	4	1	2	3	3	1	2	4
0	3	2	1	4	4	0	2	5
5	5	2	4	3	5	3	4	0
7	5	5	2	6	2	12	10	0
7	1	5	4	9	1	12	3	0
6	3	5	6	5	5	3	6	2
3	2	0	2	3	5	4	3	3
6	3	2	2	1	1	3	3	2
1	4	0	1	3	2	0	1	4
1	2	0	1	3	1	1	1	0
19	6	0	10	0	10	14	16	0
4	3	5	4	6	2	8	6	4
3	5	2	1	0	2	5	2	2
4	4	2	3	3	5	3	4	1
2	2	1	1	0	2	0	1	4
5	3	3	2	0	2	4	5	4
2	5	3	2	3	2	0	0	2
1	2	1	1	0	3	2	1	0
6	1	4	3	1	5	5	3	2
11	6	5	4	6	4	9	7	5
2	4	2	1	1	2	4	1	3
1	3	0	3	1	1	4	1	2
7	9	9	9	8	7	7	6	5
4	3	2	1	1	1	6	8	0
2	1	8	3	3	0	6	10	0
3	0	1	1	0	1	1	2	6
2	3	0	0	0	0	0	0	2
5	5	5	5	4	5	6	8	1
0	3	0	0	1	5	0	0	4
1	1	1	1	1	1	1	3	3
2	3	1	1	0	3	1	2	3
1	2	2	0	0	1	1	1	4
5	4	4	5	1	3	9	5	1
0	0	0	0	0	2	0	0	1
5	3	1	4	5	5	5	3	2
4	0	2	5	0	5	3	2	2
8	1	3	2	8	3	9	5	2
2	2	1	1	4	3	1	3	2
1	4	3	1	1	2	1	1	5
6	1	4	2	3	1	5	3	2
3	6	1	1	0	2	2	1	2

0	3	1	0	1	5	1	1	2
2	2	0	0	1	1	0	0	3
4	1	6	2	14	5	5	5	0
0	1	0	0	0	1	0	0	3
1	1	0	0	4	4	0	0	5
0	3	0	0	0	0	2	0	2
4	5	4	2	0	4	5	3	1
2	1	1	3	5	3	2	5	1
1	2	4	3	6	3	5	5	1
3	0	2	1	6	2	8	6	1
5	0	3	1	0	1	6	10	0
2	5	0	1	1	1	0	0	1
3	2	8	6	1	2	2	10	0
1	1	1	2	1	2	0	1	4
2	3	3	1	4	3	6	4	1
1	2	2	0	0	2	0	1	6
4	5	4	5	3	2	3	4	0
4	2	3	2	1	3	6	5	0
4	2	3	1	3	4	3	7	0
7	5	4	4	6	4	10	8	0
2	0	1	6	1	2	5	6	3
5	2	9	5	0	2	5	5	2
3	5	3	1	8	4	3	5	0
7	5	7	1	1	2	5	5	0
0	0	2	0	1	2	1	1	4
2	0	1	4	3	2	4	6	0
1	0	2	2	0	1	6	6	0
2	4	4	5	0	5	6	4	0
24	20	20	19	0	15	25	24	18
6	5	5	8	0	8	7	5	4
6	3	1	0	0	6	5	7	1
8	9	11	9	8	5	6	8	0
4	5	1	0	4	1	0	3	0
0	0	0	0	0	0	0	0	0
19	0	21	0	17	0	19	14	11
5	6	6	5	1	0	3	1	0
7	3	7	2	4	3	4	6	1
0	0	4	6	0	5	0	5	0
4	1	2	2	6	1	6	8	0
6	6	8	8	5	8	14	13	9
7	7	9	4	6	0	7	8	7
6	5	5	4	6	5	6	6	3
3	5	0	0	0	2	3	5	0
8	9	0	0	14	8	8	9	8
13	0	10	0	14	11	13	18	7
7	13	8	5	10	9	9	11	8
11	15	13	11	6	5	11	11	0

0	0	5	6	5	3	5	3	0
3	3	3	0	5	0	5	4	4
0	0	0	0	0	0	0	0	9
5	4	2	5	4	2	2	7	0
6	5	3	4	3	0	0	5	0
11	7	0	0	0	0	0	12	0
28	25	24	0	0	22	22	0	0
6	4	7	2	6	7	6	6	2
4	4	3	7	0	3	5	8	0
0	0	0	3	0	0	0	0	0
9	4	0	0	0	5	12	10	0
5	0	0	5	9	4	0	0	0
5	4	7	4	8	6	6	6	0
0	0	0	0	0	0	0	0	0
0	6	0	0	0	0	0	4	0

								Normalized Counts
IgAN E0	IgAN E0	IgAN E1	IgAN E1	IgAN E1	IgAN E1	NLTx	NLTx	NLTx
T9.normed	T10.norme	T4.normed	T20.norme	T21.norme	T23.norme	T5.normed	T6.normed	T13.norme
60	54	63	85	50	60	49	48	72
44	55	53	51	46	52	48	49	55
51	46	42	57	54	63	50	36	44
31	39	46	57	51	56	37	36	40
46	45	43	43	49	56	41	48	46
51	54	40	48	40	38	26	29	47
56	60	51	21	31	37	52	60	50
55	35	22	59	38	42	14	14	32
38	38	46	37	35	41	47	41	43
38	30	38	31	37	42	32	35	37
52	44	49	50	27	31	29	22	42
33	50	42	58	42	46	40	32	42
43	41	40	37	26	28	44	45	47
26	36	37	26	33	38	36	39	47
36	49	35	31	36	30	38	39	37
21	17	24	46	37	38	29	20	21
34	26	30	58	31	35	19	16	32
43	35	44	22	31	26	33	40	32
29	36	36	45	26	24	30	27	32
28	31	33	28	32	25	26	33	32
23	30	21	43	35	40	21	21	32
36	31	34	37	27	26	29	37	37
39	39	36	19	27	26	38	38	39
49	25	29	19	32	34	33	42	38
34	41	31	26	39	42	33	32	38
29	30	37	27	30	28	28	32	34
29	38	32	20	25	21	42	35	35
29	28	24	27	33	26	32	32	31
29	35	23	26	24	26	26	22	29
24	36	23	27	28	30	29	24	25
22	25	21	27	28	25	23	26	24
25	22	25	24	24	24	30	31	26
24	19	25	25	23	22	28	34	25
4	6	3	19	2	2	5	2	3
15	16	19	32	33	28	16	19	22
31	26	27	15	34	33	22	18	24
19	21	32	22	17	21	23	26	27
26	27	25	24	18	23	23	24	28
12	32	21	36	7	14	40	27	11
23	18	24	19	24	26	28	24	24
21	32	19	25	17	24	25	21	26
21	24	22	19	27	27	15	19	20
21	49	22	32	14	12	39	26	14

21	11	23	22	17	16	21	18	22
25	24	29	22	21	29	22	26	32
15	22	25	14	36	24	17	23	23
30	30	21	16	27	19	22	25	17
18	20	19	24	22	21	20	22	19
19	29	26	19	11	14	22	28	25
14	12	16	25	15	22	17	16	21
15	21	16	22	27	20	18	16	20
15	11	17	32	14	22	14	10	11
23	25	21	25	14	17	23	19	23
7	10	10	16	47	13	6	4	11
14	20	10	21	25	19	17	11	9
18	17	15	24	17	15	16	13	17
23	9	0	24	9	16	9	9	7
11	10	10	33	17	21	6	3	11
21	11	12	24	17	22	11	9	14
29	24	15	9	20	15	20	24	22
15	20	19	15	14	18	21	18	19
16	13	19	21	17	14	19	16	15
21	18	23	11	14	19	28	21	21
14	13	15	12	21	18	16	13	15
14	17	15	17	21	26	20	22	22
19	15	14	16	22	14	14	12	10
19	17	14	14	19	20	13	13	16
14	13	13	17	19	18	8	10	12
22	17	19	10	19	11	17	22	25
17	19	18	12	9	14	17	20	21
3	10	9	17	23	13	11	3	8
16	11	17	14	17	10	13	14	22
7	11	13	21	20	17	9	12	11
13	15	16	21	20	15	12	13	12
13	11	10	12	27	21	14	11	14
15	15	18	14	13	12	15	17	18
15	6	10	19	13	14	12	11	14
18	12	18	12	15	14	14	15	13
20	19	16	24	14	13	12	13	14
14	16	15	14	14	15	14	15	16
6	11	7	11	21	27	14	16	10
21	16	19	11	13	10	18	19	20
17	13	15	11	22	27	13	17	12
11	11	13	15	14	14	16	18	14
9	12	13	11	13	14	15	16	14
12	17	15	16	13	14	12	16	13
1	4	5	14	2	10	3	1	7
18	19	10	12	14	15	12	15	12
21	6	13	7	21	18	19	16	13
13	17	16	12	12	9	15	15	21

14	15	12	16	23	21	9	9	10
18	15	14	16	14	12	14	15	16
11	17	15	17	14	17	14	13	13
18	15	9	22	17	16	10	1	6
8	6	31	22	0	5	35	34	6
18	18	15	11	13	8	15	13	12
19	9	12	12	15	13	11	15	12
10	11	10	9	17	20	13	10	11
9	12	15	16	13	14	13	10	12
7	10	8	9	18	14	9	12	11
12	11	11	10	13	12	12	10	14
17	17	12	10	13	16	16	17	16
14	16	16	12	10	9	17	19	16
15	10	8	14	11	10	8	8	14
16	11	11	4	14	14	12	15	15
20	18	15	7	9	13	18	16	13
14	11	11	11	14	11	11	10	11
17	18	9	20	14	18	17	1	7
7	8	8	12	11	6	0	0	7
10	12	13	19	13	14	10	10	13
11	17	17	5	13	16	20	14	18
12	11	12	12	9	15	15	12	13
11	11	13	14	11	16	10	11	11
17	12	13	10	9	9	11	10	12
16	10	12	11	13	11	9	14	11
14	13	16	9	9	9	18	11	13
18	17	16	11	11	12	14	9	7
38	35	32	45	34	46	35	22	36
7	8	9	11	11	10	7	9	11
21	24	25	26	20	16	21	19	21
14	16	13	12	10	9	12	12	18
12	12	11	9	11	16	12	9	9
9	15	10	15	6	10	6	12	11
10	9	8	11	16	15	9	12	8
17	15	18	6	13	8	14	17	13
9	18	8	15	9	10	10	16	13
8	41	14	38	12	10	32	14	8
16	12	13	6	13	14	17	10	11
8	11	9	10	10	10	11	11	11
6	8	7	10	5	6	5	7	7
12	12	9	6	12	10	10	11	9
7	7	8	12	12	12	9	7	8
14	10	12	6	17	13	10	10	13
11	6	7	17	11	10	9	10	9
12	8	8	14	9	9	11	11	11
10	10	9	12	6	8	8	6	10
13	20	13	19	9	10	6	9	12

9	9	8	15	7	12	10	9	10
15	7	10	15	10	15	12	13	13
13	13	10	7	9	13	9	13	12
3	2	7	16	3	2	1	0	5
12	4	8	9	11	16	9	14	9
15	11	16	20	21	18	16	17	17
9	7	9	7	13	9	8	10	7
12	9	8	14	9	10	9	10	10
10	6	9	5	11	14	10	10	7
11	18	13	9	14	6	9	14	11
14	16	10	6	13	10	10	12	11
15	13	14	11	6	8	8	9	12
10	9	9	11	12	10	10	14	11
6	3	7	14	16	10	6	7	6
17	11	12	14	8	7	16	14	11
13	12	14	9	7	7	11	13	11
7	11	10	14	10	11	5	8	11
9	12	9	7	6	6	11	8	10
9	9	7	6	9	14	10	10	8
7	3	1	20	0	16	0	0	7
8	10	8	14	13	10	7	9	9
9	11	12	5	15	12	9	9	5
12	15	11	19	12	11	13	10	10
11	11	12	11	9	8	13	11	8
22	6	5	5	11	11	7	10	7
11	9	11	6	6	6	10	7	11
18	11	9	9	8	7	11	10	12
5	3	8	16	13	15	4	4	6
13	11	6	17	9	9	5	5	10
8	6	12	11	13	9	12	9	13
9	10	12	6	16	15	9	11	8
5	10	7	2	13	10	8	9	8
13	4	3	11	13	12	3	2	6
4	8	7	9	9	9	5	10	11
11	8	9	7	6	6	8	11	10
10	10	10	7	13	5	8	10	11
34	37	29	25	29	30	29	26	33
21	17	24	9	7	9	16	19	22
11	11	7	6	9	8	9	11	11
6	9	9	9	12	9	11	9	11
10	6	10	5	9	9	6	10	7
8	9	8	11	11	10	12	6	9
5	8	8	10	10	10	9	10	11
7	9	12	6	9	9	10	11	10
7	12	8	6	9	9	8	9	8
7	4	5	9	7	7	2	7	7
9	11	6	9	6	10	6	7	8

13	6	7	2	15	6	10	10	11
9	6	6	5	16	8	9	6	7
8	6	14	9	8	10	12	11	11
9	10	8	9	9	9	10	10	8
14	9	8	6	12	13	10	6	8
8	8	8	7	7	8	11	10	10
9	6	7	6	12	12	10	11	9
10	12	8	5	10	10	11	10	11
11	11	8	5	9	9	12	8	11
5	10	6	22	7	7	13	7	5
8	6	4	7	6	7	4	6	7
12	13	8	7	5	6	9	11	10
9	9	9	4	10	7	9	9	8
9	9	3	12	12	11	7	5	7
8	8	7	10	12	6	8	8	9
10	8	6	7	7	7	9	8	9
9	10	8	6	8	8	10	10	11
12	6	5	11	8	6	7	6	13
8	8	5	14	7	8	4	6	6
9	6	8	5	9	10	7	8	9
7	7	6	15	5	6	7	8	7
11	11	9	9	7	7	5	3	7
6	4	7	11	7	6	6	5	7
4	12	13	11	9	7	8	11	13
12	15	8	9	5	6	9	7	8
13	7	15	15	9	10	8	14	10
11	9	8	10	7	7	11	7	9
4	7	6	7	8	7	7	9	9
10	9	10	6	8	6	7	9	9
6	7	7	9	6	7	2	7	7
4	1	2	11	6	13	4	2	1
9	6	7	2	10	7	6	8	7
9	9	11	5	9	8	13	10	8
6	9	8	10	5	6	7	3	3
7	11	5	10	7	6	8	8	10
9	10	11	20	9	10	4	10	10
7	3	5	10	3	9	3	4	9
9	6	8	4	6	11	7	8	8
4	9	7	2	10	10	7	7	7
6	6	7	6	9	7	10	8	6
9	2	2	5	9	9	5	7	6
4	6	6	0	10	9	4	5	3
10	15	7	6	17	10	10	7	9
10	10	8	7	7	4	4	8	9
6	3	6	10	10	10	4	7	8
9	9	6	2	6	6	7	3	6
5	3	5	6	6	7	7	6	8

7	10	4	12	6	5	7	6	9
8	13	8	7	9	7	7	10	13
7	4	11	5	4	6	13	8	9
7	7	7	5	6	7	9	10	11
8	6	6	4	10	6	4	7	7
11	9	10	5	6	7	9	10	5
3	6	5	7	8	7	4	6	3
6	7	8	9	5	6	9	8	8
7	9	7	9	9	8	8	8	9
1	4	5	15	3	4	5	1	2
5	7	4	9	9	6	7	5	4
3	7	5	1	13	7	4	5	6
5	3	6	0	7	12	4	6	7
7	9	6	6	5	9	10	8	8
7	11	8	25	16	24	5	5	10
7	15	8	5	6	7	8	6	7
9	7	6	7	9	8	8	10	5
4	4	5	7	2	4	5	1	0
6	6	8	6	8	7	10	7	6
7	3	8	5	8	6	1	6	5
8	8	8	6	3	6	10	10	11
7	7	8	4	6	5	8	11	8
7	8	6	11	5	6	6	7	4
8	6	7	2	11	9	8	6	6
4	1	7	9	5	6	4	5	4
0	1	3	5	2	4	3	1	1
5	7	7	4	9	7	6	7	4
9	8	6	4	9	6	3	9	4
5	1	6	6	9	4	2	4	4
7	9	8	4	6	6	7	7	8
9	11	9	15	7	10	7	9	9
5	7	8	9	3	6	8	8	8
8	10	7	5	7	7	6	6	8
4	3	1	7	8	10	2	2	3
4	3	6	10	5	4	6	7	11
8	9	8	4	8	8	8	9	11
5	3	4	10	6	6	8	4	5
4	4	3	1	8	7	3	5	5
5	3	4	11	6	5	4	10	6
8	9	8	4	2	2	8	7	10
6	4	5	1	10	10	3	6	7
4	6	4	7	4	5	1	1	5
8	10	7	14	6	4	5	3	2
7	9	8	5	4	6	6	11	8
6	7	4	0	8	7	5	4	6
9	6	2	10	5	4	2	2	3
5	7	8	1	5	7	12	11	7

6	7	6	1	9	7	6	7	7
19	12	16	11	9	8	7	8	14
5	7	5	9	4	6	4	5	7
5	2	6	5	8	8	3	4	3
2	2	4	5	6	8	3	2	3
7	7	7	6	5	6	5	7	1
7	10	8	4	6	4	9	3	4
5	4	7	7	6	6	7	9	5
7	6	8	5	3	4	11	11	11
5	3	4	0	9	4	1	6	4
7	7	5	4	3	3	8	8	10
5	3	8	4	6	5	6	6	8
10	3	7	4	8	9	5	7	7
3	6	4	4	5	6	5	4	8
9	3	5	10	5	6	5	5	6
5	6	7	6	5	6	9	5	6
6	3	4	9	6	7	5	5	3
4	4	4	5	7	9	2	3	2
6	9	6	6	5	5	6	7	9
3	4	6	6	5	4	4	2	5
11	8	8	10	12	15	12	10	11
8	4	10	4	6	4	6	7	6
5	4	4	9	9	11	3	4	4
5	6	7	4	14	4	5	5	8
4	1	6	2	4	6	2	6	2
6	7	5	4	5	8	8	8	5
5	3	5	4	6	7	6	5	4
2	4	4	6	9	6	6	3	3
9	7	8	6	4	5	9	4	6
7	7	6	2	6	6	4	8	8
1	1	2	1	6	6	3	1	2
8	10	3	12	6	6	6	2	4
7	9	5	1	4	2	9	10	13
5	9	6	5	4	3	5	7	7
7	6	7	1	6	5	2	3	7
2	4	8	6	1	4	2	3	3
3	6	5	1	5	10	5	5	5
4	3	4	7	3	3	6	5	6
5	6	6	5	5	6	6	5	6
5	4	6	2	6	6	6	6	6
3	2	3	10	10	7	3	4	4
2	2	3	10	4	6	2	4	3
5	3	4	6	6	7	2	5	4
5	11	11	4	6	6	11	10	7
5	7	7	1	3	7	5	6	7
18	32	15	22	24	23	25	18	20
4	4	5	4	2	2	3	4	6

4	4	3	9	6	4	2	2	3
9	7	8	5	4	8	4	10	6
6	6	5	4	9	6	5	3	3
4	3	7	5	6	6	5	3	6
4	4	3	2	2	6	1	6	2
3	7	4	1	6	6	8	8	5
4	8	8	11	2	3	4	7	4
8	6	10	1	7	3	7	10	4
2	3	3	4	5	6	3	4	5
1	3	6	6	6	3	5	4	3
1	3	4	2	4	5	5	3	4
2	10	5	4	6	10	6	8	2
4	2	5	4	2	3	2	6	5
7	3	7	2	7	5	5	6	5
12	8	6	0	6	6	4	4	2
2	3	2	4	2	4	1	0	3
3	4	5	6	6	7	4	2	2
6	3	6	7	3	4	7	5	6
4	6	10	4	5	3	5	7	6
5	3	6	2	5	3	3	5	6
5	3	3	4	3	2	5	7	8
3	3	0	1	6	7	0	2	2
4	4	4	4	6	4	4	3	3
3	4	5	1	5	5	8	2	3
3	3	3	6	2	5	4	4	5
6	4	4	2	6	4	5	5	8
4	4	4	5	9	9	3	4	3
4	4	7	6	5	5	3	4	3
3	2	4	1	3	2	3	5	4
3	1	4	6	5	6	0	0	0
3	3	3	2	7	3	4	3	6
7	3	4	5	5	5	5	5	5
3	3	4	5	3	6	4	4	3
3	4	3	5	6	5	3	4	3
4	3	2	4	6	6	2	4	6
6	7	6	0	2	3	10	8	7
4	7	5	9	2	2	3	4	6
4	8	4	6	3	4	3	2	6
5	4	6	6	5	4	6	6	5
3	3	2	1	8	7	3	5	5
5	7	6	1	5	3	8	6	5
3	11	5	10	4	0	16	12	2
3	2	2	2	0	2	3	1	7
4	2	8	4	3	2	7	7	7
4	8	5	6	4	4	4	7	6
2	2	3	9	6	4	3	4	5
6	20	0	5	6	6	3	10	4

0	1	2	6	7	10	1	4	0
4	4	4	2	2	3	1	4	2
7	2	5	5	9	4	3	5	4
4	4	6	11	2	2	4	3	6
6	4	3	5	6	2	3	2	6
5	1	5	4	4	6	6	4	9
5	6	5	0	3	3	7	7	5
7	3	4	4	2	5	2	3	3
1	2	4	2	5	2	4	2	7
2	9	8	0	2	2	4	5	4
3	1	4	7	6	7	2	3	3
5	4	5	5	7	4	7	5	3
3	3	4	2	7	8	3	4	4
4	2	4	5	4	2	5	3	3
4	4	5	6	5	2	6	5	4
4	1	4	2	6	4	2	5	2
4	3	4	5	5	5	5	5	4
7	7	6	2	3	3	4	7	4
2	2	4	2	4	2	1	2	6
8	3	7	1	6	9	1	7	7
1	4	4	4	2	3	6	4	2
2	4	5	4	6	5	4	4	3
3	2	1	4	5	6	1	4	3
4	6	5	2	5	3	6	3	4
3	2	2	5	2	3	2	3	4
5	6	3	1	5	3	6	6	6
8	4	3	0	8	2	4	7	3
3	6	2	1	9	10	4	5	6
6	7	3	1	7	5	4	2	5
5	6	3	6	6	4	4	4	4
6	6	3	1	3	4	5	7	5
5	4	3	5	2	3	5	4	5
5	1	5	6	2	3	3	3	3
5	3	3	1	3	4	4	4	3
4	4	3	4	2	2	3	3	3
1	2	2	2	6	3	2	0	2
1	1	3	1	5	4	2	3	3
6	6	5	4	4	3	7	3	5
0	13	6	22	0	0	18	8	3
5	2	5	5	2	5	4	6	6
1	1	2	4	2	6	2	0	2
6	8	5	0	5	2	4	8	4
3	4	2	6	5	5	3	2	2
3	4	3	2	2	6	4	5	1
1	2	2	2	2	3	1	2	3
0	2	2	5	1	0	1	0	0
3	2	2	2	6	5	5	1	2

3	2	5	1	6	1	1	4	5
8	3	3	1	2	3	4	3	4
1	2	3	6	3	3	3	5	1
5	4	5	4	2	3	4	6	5
4	2	4	2	6	4	3	3	3
2	3	3	1	3	2	3	3	4
3	4	3	5	2	3	2	1	5
4	3	3	10	2	2	4	2	2
5	7	3	2	2	2	3	4	1
3	4	6	5	2	1	4	1	5
5	3	6	1	4	2	3	5	8
3	2	3	1	6	6	2	3	2
2	4	1	5	3	7	1	2	4
7	2	2	2	2	2	7	7	8
0	0	0	0	2	3	0	0	0
3	2	3	4	5	4	3	3	4
9	13	17	0	11	9	17	13	15
1	4	2	2	3	2	1	2	3
1	0	4	4	3	3	0	1	1
3	3	4	2	7	3	5	4	3
1	3	3	2	5	5	5	3	4
3	7	3	5	3	3	4	4	3
10	7	4	2	6	6	8	7	7
3	3	2	4	6	5	3	4	3
1	4	1	4	2	5	1	4	4
0	0	4	10	3	4	1	0	0
0	3	0	7	6	9	0	0	1
2	2	4	4	2	3	5	5	3
2	6	3	1	1	0	4	1	6
2	0	3	5	2	5	1	2	3
2	4	3	1	5	2	4	3	6
2	2	3	1	3	5	3	3	1
5	0	2	2	8	8	3	2	1
6	7	3	6	0	1	3	0	1
5	1	3	2	6	4	3	4	3
6	2	5	1	2	2	4	5	3
3	6	4	4	2	2	3	2	4
6	0	5	1	4	1	1	6	5
5	3	5	1	2	2	3	2	2
2	3	7	5	2	3	3	4	8
2	1	2	2	6	5	1	1	3
4	1	2	0	4	4	1	1	2
5	3	4	4	2	3	5	5	2
4	2	4	4	4	3	5	5	5
8	8	5	2	5	4	9	5	6
2	6	2	6	2	2	5	3	5
1	0	1	2	3	3	0	1	4

2	1	3	2	3	3	2	2	1
3	2	6	5	2	2	3	5	3
2	1	4	1	2	2	3	2	1
3	6	3	0	2	2	3	2	2
7	3	8	4	2	2	4	8	5
5	2	7	2	2	2	6	5	6
0	4	6	10	13	12	0	8	10
3	3	2	5	6	3	1	3	2
5	4	6	4	2	2	4	5	4
22	27	19	16	23	24	18	17	24
1	1	4	1	3	3	0	4	3
2	1	4	1	2	4	6	4	5
3	2	4	1	2	1	5	5	6
0	2	1	6	2	5	1	0	2
4	2	2	1	3	5	0	1	4
1	1	1	6	2	3	1	1	1
5	3	10	0	0	2	2	6	10
3	2	4	2	3	4	2	4	2
2	2	2	0	8	1	2	1	1
2	3	5	4	2	3	3	4	1
3	2	3	4	3	2	2	1	2
1	2	1	5	3	3	1	1	2
4	3	2	0	3	3	4	3	1
1	3	2	0	5	4	1	3	2
3	2	2	4	2	4	1	4	3
5	2	2	2	2	3	4	4	2
4	1	2	2	2	1	3	2	2
3	2	2	5	2	2	0	1	3
3	4	3	4	2	2	7	5	4
2	3	4	2	4	5	1	4	1
2	1	3	4	6	6	0	0	1
5	1	6	2	3	2	1	4	1
1	3	2	4	7	6	1	1	3
1	6	6	1	3	2	3	4	5
4	3	4	1	2	1	6	5	2
3	1	4	1	3	3	1	1	1
5	2	4	1	3	4	2	7	2
2	3	4	4	2	1	2	2	2
2	1	2	5	2	2	4	2	3
1	1	1	2	2	3	2	2	1
3	1	1	1	5	3	4	1	3
4	1	4	1	5	4	2	4	3
2	3	3	5	2	5	3	2	3
1	0	1	2	3	3	1	1	1
1	2	1	5	2	2	0	1	2
3	1	2	6	3	3	2	1	0
2	0	4	2	2	2	1	0	1

0	3	0	4	3	2	0	0	1
3	7	4	1	4	2	2	3	3
3	1	5	0	2	2	3	3	3
8	8	8	6	5	4	5	5	8
15	16	11	17	13	14	12	11	14
2	2	2	2	2	3	2	3	3
2	2	5	1	2	4	2	4	2
5	1	6	2	1	2	9	4	3
1	0	2	1	4	2	3	1	2
1	1	1	2	2	2	1	1	1
0	0	0	6	2	2	1	1	0
0	2	2	4	2	2	2	3	2
3	2	2	0	4	3	3	3	3
1	1	3	2	5	2	2	3	1
5	4	4	1	3	1	4	3	4
5	2	7	2	3	4	8	6	1
10	8	7	0	6	10	10	7	9
2	3	5	4	0	2	1	2	0
4	0	3	2	1	2	1	1	0
2	1	1	5	5	2	1	1	1
4	3	3	2	2	1	3	4	4
6	2	3	0	1	2	1	1	5
3	3	3	1	2	2	5	4	4
1	2	2	2	4	6	1	3	1
0	1	1	0	4	3	0	1	2
7	4	7	7	5	5	5	5	6
5	2	2	1	6	3	2	3	1
2	1	3	4	2	4	3	2	4
0	0	0	10	6	7	0	5	0
1	0	0	4	1	2	1	1	1
11	0	0	5	0	2	0	0	0
6	3	5	0	1	1	3	5	5
2	3	3	0	3	4	2	4	4
1	0	1	5	2	2	0	1	0
5	4	2	1	6	1	1	3	3
3	2	2	2	2	1	3	3	7
3	4	5	2	3	2	2	2	2
2	3	2	1	2	2	3	2	1
3	2	1	4	0	2	0	0	0
3	4	0	0	6	1	2	3	4
0	1	1	0	3	0	2	1	1
1	1	1	5	5	2	0	1	0
0	0	1	2	3	2	0	1	1
4	1	4	1	2	2	2	5	4
2	1	2	0	4	3	2	4	2
2	1	4	2	0	3	0	1	0
3	2	1	0	3	5	2	4	1

0	1	2	1	1	2	0	4	2
1	4	5	1	6	1	3	3	3
0	1	0	2	2	1	0	0	0
3	2	3	0	2	2	3	5	2
4	4	6	1	2	2	1	4	4
2	1	2	0	4	2	1	3	0
1	1	0	9	6	8	0	0	0
1	2	5	5	2	1	1	1	2
1	2	1	5	0	2	2	0	2
1	2	1	2	1	1	0	0	1
1	1	1	0	0	1	0	0	0
6	3	5	0	6	1	2	4	2
0	0	0	1	0	2	1	0	0
2	1	3	0	1	1	3	5	3
1	0	1	4	4	4	0	1	1
6	3	5	1	2	0	2	5	4
0	1	1	5	2	0	1	1	0
1	0	2	1	2	2	0	0	2
0	0	0	0	6	6	0	0	0
0	2	0	0	0	3	2	0	0
4	1	3	2	2	5	0	2	1
3	2	5	0	2	3	3	0	0
2	0	1	2	1	1	0	2	0
0	2	0	2	2	2	0	0	1
1	6	3	0	2	1	3	3	3
1	1	1	2	1	3	0	0	1
0	0	1	1	1	3	0	1	2
0	0	0	7	0	0	1	0	0
21	21	18	24	0	0	16	15	19
0	4	3	7	5	6	2	0	2
0	0	1	0	3	2	1	2	0
8	12	8	7	9	9	7	9	0
0	0	0	0	5	5	0	0	0
0	0	0	0	0	0	10	7	0
0	16	11	0	16	20	12	10	0
2	0	3	2	0	0	2	0	0
2	0	3	6	2	2	3	1	1
0	0	3	5	0	3	8	6	0
0	0	0	0	1	0	0	0	0
15	15	14	12	6	7	10	9	11
5	2	6	6	6	6	5	4	5
2	3	3	0	4	5	0	3	0
0	0	2	0	2	2	2	0	0
10	0	0	0	7	7	0	0	5
0	0	6	0	9	10	0	0	9
8	8	7	5	8	11	8	8	0
0	8	0	15	9	7	0	0	0

5	7	6	7	4	4	3	5	3
5	2	4	0	0	3	0	2	3
13	11	11	0	0	10	12	8	9
6	0	0	2	5	6	0	0	4
0	0	0	4	3	4	0	0	0
13	0	11	0	8	11	12	10	0
26	0	23	0	24	24	0	19	0
0	0	0	2	6	5	2	0	0
0	3	3	4	2	2	3	0	0
0	0	6	7	6	6	9	6	6
14	9	0	0	6	7	0	0	0
0	0	6	7	0	0	0	3	6
0	2	0	0	6	0	0	0	0
0	0	45	0	0	0	47	41	41
0	0	0	0	6	6	0	0	0

NLTx T14.norme	NLTx T15.norme	NLTx T28.norme	NLTx T29.norme	NLTx T30.norme	S.a.GN T1.normed	S.a.GN T2.normed	S.a.GN T11.norme	VancATN T16.norme
48	46	39	30	30	125	82	115	58
61	63	55	49	54	47	57	58	56
39	51	64	34	40	53	50	52	60
35	52	54	52	49	34	48	39	70
41	52	65	54	58	42	43	31	44
45	28	40	44	43	46	51	57	54
54	72	39	32	40	25	47	24	21
22	14	25	29	36	67	50	62	54
42	40	45	36	33	36	44	39	47
48	39	67	50	54	29	35	39	26
24	14	23	15	14	93	68	100	39
37	27	28	31	35	60	45	63	35
43	44	34	35	28	38	38	32	32
44	32	47	57	50	25	28	21	32
38	39	43	48	40	28	30	26	32
20	15	45	35	35	29	25	33	47
18	18	13	18	16	65	37	54	48
30	48	27	35	35	17	40	20	28
37	27	27	23	26	42	29	38	35
40	30	35	38	42	15	21	17	31
26	24	36	36	33	32	28	30	33
32	26	22	24	22	43	31	43	26
46	38	44	33	37	17	27	15	19
41	42	46	27	31	10	31	11	17
40	34	63	24	25	17	22	14	12
39	30	32	37	31	27	21	21	22
31	32	28	28	23	18	20	25	21
29	35	33	36	38	17	21	15	21
26	24	22	22	23	39	31	40	29
27	24	29	30	33	17	14	18	21
25	29	34	22	30	19	10	11	21
24	36	26	26	30	18	22	15	24
28	34	22	26	26	19	31	21	22
3	3	2	2	2	11	3	4	4
12	17	18	17	16	39	31	44	26
23	20	31	23	33	8	24	10	14
26	26	16	21	17	29	26	33	21
23	22	16	15	15	45	30	40	21
16	47	18	4	6	53	14	16	24
26	24	29	21	26	20	23	25	20
25	27	28	28	32	14	18	12	21
21	19	29	18	29	29	20	19	20
18	39	14	4	2	42	21	15	30

25	17	17	13	13	33	26	38	24
26	18	22	19	16	43	41	42	25
17	27	27	24	29	8	16	6	24
21	26	30	18	24	10	16	6	12
21	20	22	21	20	14	17	17	19
30	30	18	21	21	20	16	17	18
14	15	14	18	16	18	16	24	21
15	15	21	23	23	22	17	23	27
12	17	4	14	7	33	33	23	26
20	21	16	17	16	20	17	15	15
7	2	9	12	7	19	10	17	36
13	20	18	6	9	9	16	26	28
18	19	15	13	13	22	17	29	22
10	17	5	10	9	32	32	35	29
4	6	7	12	12	28	14	25	29
8	5	13	17	13	27	17	25	27
27	35	14	18	19	8	16	11	5
18	20	17	15	17	17	11	13	12
15	13	11	17	13	18	15	16	13
23	19	16	16	18	4	17	13	11
14	12	13	16	16	15	15	20	18
22	23	22	17	23	14	17	13	9
8	13	18	20	19	15	21	19	19
15	19	21	18	19	6	10	13	11
9	9	16	13	14	19	15	28	24
19	27	19	13	16	10	18	12	11
22	20	16	17	14	15	17	9	12
13	7	10	2	1	5	10	38	28
20	15	22	20	13	10	16	8	10
9	13	14	17	15	17	18	12	20
10	14	18	15	17	20	17	17	21
14	11	17	21	17	10	8	17	10
12	18	14	10	8	20	24	20	17
12	8	11	10	12	15	14	19	17
17	14	13	10	10	17	13	17	15
15	13	13	10	12	20	17	23	11
15	16	16	16	16	14	15	16	14
6	21	39	41	28	1	2	2	4
19	15	13	13	12	11	15	11	12
17	14	25	23	25	1	4	0	10
14	22	13	15	15	11	14	10	14
13	14	21	17	17	10	11	9	11
12	13	9	10	12	23	16	20	17
4	0	6	6	4	14	5	17	9
14	14	18	15	15	9	11	10	11
12	15	19	22	22	5	9	2	9
20	17	15	21	20	5	9	8	9

7	11	16	20	17	15	14	17	19
16	15	15	13	13	11	11	12	12
13	9	24	13	19	13	10	9	14
5	11	11	10	5	17	11	16	17
22	39	9	1	2	57	20	20	5
16	18	17	11	12	8	14	8	11
16	18	18	14	19	8	16	4	11
11	16	30	20	23	0	7	5	9
18	19	13	13	13	8	12	12	11
12	12	22	17	18	4	7	6	17
12	7	10	6	11	17	15	15	12
15	14	14	15	16	8	8	8	12
18	13	10	10	8	14	16	17	7
11	8	13	7	7	17	15	15	15
10	12	20	18	19	5	10	9	8
18	16	16	12	12	13	11	5	6
10	10	15	14	12	10	8	8	11
7	11	13	13	4	19	9	12	16
2	4	3	4	2	31	11	29	19
12	11	14	17	12	13	11	14	15
12	18	24	17	19	1	8	0	6
13	5	11	10	13	14	12	12	12
13	19	18	17	16	8	12	4	16
13	13	7	7	6	15	13	13	8
12	15	15	13	14	8	15	6	10
9	12	10	13	12	8	8	10	13
8	15	10	16	19	13	11	11	12
30	26	38	27	26	46	49	50	40
9	13	9	9	7	14	15	15	17
25	21	20	21	18	20	22	20	21
16	13	8	12	9	14	14	15	9
9	21	14	6	9	19	12	13	15
11	13	9	10	9	11	16	13	10
11	15	16	20	19	3	8	3	11
11	11	10	12	8	4	11	2	5
15	9	12	10	12	10	5	9	8
15	28	12	2	0	47	10	9	31
13	22	12	17	16	8	9	5	6
15	14	14	11	13	9	5	2	6
4	5	3	5	5	19	9	16	13
9	10	11	12	15	5	11	6	9
7	7	10	13	12	10	8	10	14
16	16	18	16	18	4	5	4	4
7	7	11	11	12	10	15	23	10
11	13	10	5	7	11	15	16	13
6	5	3	2	3	25	14	27	12
10	7	6	7	6	18	10	16	10

11	13	17	11	16	10	6	13	12
12	8	17	10	13	9	5	8	7
12	13	12	10	9	9	8	10	8
5	2	1	1	0	27	13	25	22
9	20	20	17	12	4	7	4	7
14	13	15	17	13	24	16	24	15
7	11	7	10	10	5	10	5	16
7	6	9	9	9	10	10	11	11
11	14	14	17	19	1	8	2	7
12	13	14	12	13	6	5	0	5
10	13	14	13	12	4	7	2	8
8	14	3	5	3	34	19	20	6
11	13	13	8	9	9	9	11	9
8	10	9	5	9	8	8	9	13
13	15	8	7	8	11	11	12	8
12	20	9	6	7	15	10	8	9
10	12	10	9	8	5	7	8	9
10	15	6	5	5	17	10	13	6
7	10	16	9	12	9	6	4	8
0	0	0	0	0	19	11	29	19
7	7	9	10	9	13	13	12	10
7	9	18	15	19	4	4	3	7
8	9	10	6	8	19	17	28	19
8	10	7	8	10	11	11	8	10
12	5	14	9	12	1	3	0	5
12	10	9	10	10	8	10	10	8
13	13	9	10	8	10	11	6	6
4	3	15	9	8	5	6	9	14
10	8	6	6	7	11	12	12	7
9	10	11	5	12	6	17	9	12
12	7	9	13	4	4	5	3	10
9	9	14	11	11	4	5	5	16
4	5	6	10	11	13	15	8	12
9	8	8	14	11	10	5	8	10
10	7	6	10	5	13	14	15	5
11	7	12	11	17	1	7	3	8
32	26	28	26	26	39	34	43	33
21	22	9	8	6	37	25	30	8
11	13	10	13	9	3	6	6	7
8	11	7	10	10	6	8	10	11
6	12	9	7	6	8	11	13	10
9	8	8	11	10	5	9	5	9
9	10	8	8	9	5	13	11	13
12	9	7	10	8	13	10	15	6
4	7	8	8	7	10	12	10	7
9	8	5	9	6	9	12	10	15
5	7	6	5	5	11	14	9	8

7	8	24	7	22	1	10	2	5
7	8	10	11	8	4	5	4	7
15	14	11	10	11	8	7	6	5
9	10	8	10	9	9	8	5	7
11	9	15	10	12	0	3	1	5
11	11	6	10	10	10	11	9	6
10	9	15	17	16	5	5	2	6
13	9	12	10	8	4	8	3	5
8	9	11	10	12	5	6	3	5
5	10	8	5	5	22	5	8	9
4	2	5	6	4	9	5	16	9
12	12	7	6	7	4	5	5	6
12	10	9	9	10	4	8	6	7
4	8	19	13	17	1	6	1	9
7	4	10	10	11	1	5	3	7
8	5	6	6	6	9	11	16	7
8	8	8	10	6	8	11	8	6
16	3	12	10	10	5	5	15	5
4	5	6	5	4	9	9	14	6
11	6	10	8	9	6	7	5	7
7	7	4	6	5	11	6	13	8
7	9	6	8	6	14	11	9	7
4	9	5	7	7	10	13	11	7
15	8	10	6	7	10	5	13	7
10	9	4	9	6	9	5	8	7
8	8	10	6	6	18	14	20	8
4	5	4	4	4	13	13	14	9
7	2	6	6	7	8	9	14	7
10	10	10	13	9	5	8	5	7
4	7	8	6	7	5	6	3	12
1	2	8	6	2	4	5	17	7
7	10	7	7	8	9	8	10	6
10	11	10	6	7	1	5	4	8
7	8	6	6	5	13	19	12	10
6	9	6	10	9	8	5	6	8
9	9	5	8	9	11	11	15	11
5	5	4	6	5	13	5	8	8
5	8	13	8	7	4	6	8	8
8	8	14	15	13	1	5	3	8
5	7	7	8	7	9	5	5	7
6	4	10	10	12	1	4	2	7
7	11	10	4	12	1	2	1	5
9	5	7	10	9	0	8	0	9
8	5	4	8	8	8	5	4	7
5	5	11	10	7	5	7	6	8
6	6	6	7	5	5	5	6	11
7	13	6	8	9	1	5	3	7

6	6	5	4	4	14	5	9	10
12	11	11	13	10	4	4	8	7
9	5	6	6	3	10	8	12	6
12	6	6	8	7	4	10	3	5
9	9	8	9	6	4	7	3	3
9	10	8	4	5	8	12	5	6
3	4	8	5	5	8	3	10	12
9	12	8	5	8	6	6	3	3
9	12	9	8	7	8	6	6	5
3	4	2	5	3	14	5	10	8
3	7	6	7	9	13	5	1	9
4	6	10	5	9	4	9	5	8
3	5	1	17	1	1	10	1	5
8	10	9	7	8	6	7	3	5
4	0	10	12	10	24	14	17	24
7	6	8	10	8	5	5	4	6
4	6	5	8	7	4	5	5	8
2	6	1	0	1	4	5	16	12
5	4	6	5	8	6	8	3	5
5	1	5	5	4	9	11	4	7
13	15	10	11	9	0	3	1	4
8	11	10	6	8	3	9	3	8
7	5	3	4	6	14	7	14	6
4	8	10	3	11	6	5	4	2
3	6	5	6	6	10	5	10	8
2	2	4	2	2	9	7	6	11
8	5	9	7	9	6	8	4	10
4	4	6	6	5	4	5	2	5
6	10	4	7	5	4	16	4	8
8	8	7	7	8	11	7	5	5
8	8	7	9	8	14	12	16	10
7	6	6	7	6	6	10	9	5
9	8	9	8	8	1	4	3	2
2	2	4	6	3	13	8	6	11
6	6	5	9	5	4	6	6	7
8	5	13	10	12	0	5	1	3
3	9	9	6	5	5	5	10	8
3	5	6	5	6	6	5	8	9
7	7	6	7	7	6	1	6	4
9	5	2	2	2	11	7	12	3
6	10	12	10	9	1	5	1	6
1	0	1	7	3	10	6	14	7
3	7	3	5	4	10	8	10	8
11	4	10	6	4	4	9	2	4
4	8	10	9	11	4	5	1	5
2	1	3	5	7	10	12	11	10
12	15	8	8	6	4	5	2	3

6	7	8	5	9	5	4	6	7
8	12	6	6	5	31	18	24	9
3	3	2	3	4	10	5	8	7
3	2	7	7	8	6	6	6	7
4	5	10	4	5	3	2	3	9
4	8	6	10	8	1	8	0	8
4	3	5	6	5	5	7	11	4
4	6	7	6	5	6	7	4	7
12	11	5	6	5	5	6	3	4
4	3	9	12	11	1	5	1	7
4	2	6	4	5	5	6	9	4
7	6	9	10	10	3	2	1	7
5	4	9	7	9	3	5	0	7
5	8	9	6	7	4	6	2	6
5	7	6	3	4	9	6	6	5
6	4	6	8	7	6	7	8	4
4	3	4	2	2	8	5	9	7
2	4	3	4	8	11	5	10	7
7	7	6	5	5	8	4	5	4
4	3	3	2	3	8	4	11	7
12	13	16	17	12	5	5	4	7
6	7	3	4	3	9	6	8	3
3	6	6	6	3	3	3	2	7
5	3	8	7	8	0	5	3	7
4	3	5	6	5	9	7	6	9
4	9	8	6	7	3	6	3	7
5	5	6	6	6	5	4	3	8
3	5	3	5	7	5	5	10	6
8	5	2	6	5	9	5	13	2
8	4	8	4	5	3	6	2	3
3	3	6	13	9	3	3	2	5
0	0	3	3	0	13	12	8	9
11	11	7	6	7	0	0	0	1
5	13	7	4	5	5	7	4	4
4	7	3	2	4	9	8	11	5
2	1	0	2	2	10	6	11	10
6	6	10	4	5	3	5	1	8
5	0	6	6	5	9	5	3	6
7	4	5	7	5	3	5	4	6
4	7	6	4	4	3	8	6	6
3	6	4	4	5	5	4	4	8
3	4	4	6	5	3	5	8	7
6	7	5	4	5	4	5	5	7
7	8	7	6	5	0	2	3	3
4	7	5	5	4	8	5	8	4
22	24	27	29	33	13	18	0	20
4	3	2	2	2	9	4	9	7

3	1	2	2	1	14	7	8	5
5	7	9	4	5	5	3	6	2
3	12	10	5	5	1	5	2	3
4	3	5	2	4	6	5	9	7
4	2	2	3	2	4	5	11	9
4	5	6	7	8	3	4	1	7
4	4	3	4	3	10	5	8	6
7	7	5	9	8	0	0	2	0
4	1	2	2	4	5	2	5	3
3	6	5	4	5	8	4	8	5
1	5	3	4	5	5	2	4	7
3	1	7	2	2	0	1	5	4
5	1	4	6	4	5	4	8	3
4	6	6	4	5	4	5	2	7
4	3	6	6	9	0	7	2	2
1	2	2	0	0	4	3	9	14
3	2	3	4	5	13	4	6	9
5	6	3	2	2	6	6	6	5
4	9	5	6	4	5	4	4	3
7	4	5	3	5	5	6	6	4
5	6	2	2	5	1	4	2	7
2	1	4	6	5	3	7	2	4
2	2	5	5	4	4	5	5	5
3	5	6	3	5	1	5	3	3
4	4	2	2	3	4	4	10	6
6	4	6	5	7	1	5	3	5
4	4	6	6	6	0	5	1	5
3	3	2	6	4	6	6	5	6
8	4	4	7	6	1	5	2	6
0	0	0	0	0	14	5	19	6
5	4	6	3	5	3	5	5	7
4	6	4	6	8	3	5	2	5
3	4	2	2	4	3	4	8	5
4	2	6	10	10	4	3	2	5
2	1	5	2	2	4	5	9	5
8	4	9	6	5	3	1	4	2
5	3	3	2	3	8	4	4	4
6	3	6	6	5	4	4	3	4
5	6	2	6	3	5	5	2	5
6	4	7	10	8	0	2	4	3
5	9	5	6	8	4	4	1	2
3	9	3	2	0	31	5	3	7
7	6	3	6	1	10	11	11	2
6	6	4	6	4	4	5	4	5
3	4	4	3	4	5	5	5	5
5	3	8	9	9	3	5	1	5
1	2	8	2	3	0	0	3	1

0	1	4	2	7	1	3	3	9
1	6	3	2	3	5	4	3	4
4	5	5	5	3	6	8	5	4
3	3	1	1	2	6	7	8	5
4	2	6	6	7	6	5	8	2
5	5	4	6	4	5	5	6	5
5	4	3	6	4	6	4	2	4
0	1	5	2	6	8	7	12	7
3	3	2	4	2	4	3	6	5
3	2	2	2	2	8	5	10	2
4	0	4	3	3	6	3	8	6
4	3	6	7	6	3	3	2	3
4	4	6	7	7	1	2	3	4
3	1	3	1	3	5	6	6	5
4	13	5	7	4	5	5	3	6
4	11	5	6	5	0	5	0	4
4	4	6	3	5	3	5	3	5
5	4	4	3	5	4	4	5	3
4	2	1	1	1	4	6	6	5
6	3	6	4	7	1	5	3	1
4	3	3	3	3	4	3	3	8
4	2	9	6	5	0	1	1	4
3	6	6	9	8	1	0	1	4
8	5	3	5	4	5	3	3	5
3	2	1	2	0	9	5	5	5
5	11	4	6	9	3	2	0	3
4	8	8	6	7	0	4	0	0
4	6	8	5	7	3	4	2	2
4	7	10	7	10	0	0	0	0
4	2	6	6	5	5	3	4	5
4	4	3	4	5	1	5	3	3
4	5	6	3	5	4	4	4	2
1	1	3	1	2	6	6	6	7
4	6	2	6	5	5	5	6	5
3	2	3	2	4	5	5	2	5
4	3	3	4	4	5	1	4	6
4	6	5	4	6	1	5	4	7
5	8	5	5	5	1	3	3	0
5	17	0	0	0	27	5	0	8
4	7	4	4	4	5	5	5	2
0	2	3	2	2	4	4	9	11
7	4	6	5	3	0	1	1	1
3	7	3	5	3	3	3	2	4
2	4	2	2	2	5	5	5	3
2	2	2	5	5	3	2	2	5
1	1	0	0	0	4	1	12	14
4	2	8	4	8	3	3	2	2

4	6	3	5	5	0	5	1	4
7	5	12	14	12	0	1	1	0
4	6	10	9	12	1	1	0	1
4	4	2	2	3	6	5	6	3
4	5	4	4	4	1	2	3	3
3	2	2	4	5	9	5	6	4
5	0	2	2	2	10	4	9	4
2	4	2	2	3	4	3	8	5
3	2	9	5	5	5	5	3	7
4	2	2	4	2	6	2	5	3
7	9	5	2	4	1	3	1	2
2	3	10	2	7	1	1	2	5
3	6	4	6	6	0	1	0	3
7	4	5	6	8	0	1	0	0
1	2	0	0	0	5	1	10	11
3	4	5	5	4	4	3	2	4
19	18	12	15	10	11	13	14	14
3	5	7	6	2	0	2	1	4
1	0	0	1	1	0	3	5	6
4	3	4	5	5	3	2	3	2
4	5	6	5	5	0	4	1	4
2	3	3	3	2	1	3	4	6
6	11	7	4	9	3	4	2	2
2	5	6	4	3	3	2	2	4
2	0	2	4	2	6	2	4	4
0	2	0	0	0	4	4	12	5
0	0	0	8	0	9	5	4	0
4	2	5	4	4	3	4	3	1
5	3	2	2	2	3	5	4	7
2	1	1	1	2	6	5	9	7
3	2	2	5	6	1	2	3	3
3	1	4	2	4	5	5	4	4
2	5	8	5	5	0	1	0	2
2	4	1	2	0	5	4	6	3
3	1	3	3	5	5	5	3	2
4	5	2	2	2	6	4	3	2
4	1	2	1	1	6	6	6	3
2	4	6	2	3	1	5	1	2
3	5	6	6	5	1	1	1	4
7	2	4	4	4	0	2	2	3
1	7	5	4	5	1	4	1	5
3	1	7	2	7	1	2	0	3
4	3	2	5	3	1	4	3	2
4	4	3	2	2	4	3	3	2
7	7	6	7	5	4	5	3	0
5	7	2	3	2	3	1	2	3
3	4	3	4	2	1	2	2	6

1	2	2	2	2	4	4	3	6
4	5	2	3	2	3	4	3	3
4	2	2	5	3	4	4	4	3
2	0	2	2	2	4	4	5	4
4	5	3	2	2	0	2	1	1
6	6	2	1	2	4	4	0	2
6	7	17	13	9	5	9	8	9
2	0	6	6	7	4	1	1	7
4	4	2	2	2	4	3	6	3
20	21	22	21	22	31	24	32	26
2	1	3	1	2	4	5	6	7
3	5	2	5	5	1	3	2	3
5	4	2	3	3	5	5	3	2
1	1	5	2	2	3	5	1	7
1	2	2	2	2	6	4	1	2
0	1	0	2	1	3	2	3	8
0	0	0	0	0	18	6	5	5
2	1	4	2	3	5	4	5	5
4	0	1	1	5	3	2	0	3
4	4	3	3	4	3	4	2	4
3	1	2	1	1	5	4	5	3
2	1	3	3	1	1	1	3	6
2	3	6	3	5	1	3	2	2
2	2	4	2	5	1	1	0	6
3	0	2	2	1	3	4	5	4
3	1	7	2	4	1	3	1	5
2	4	4	6	4	1	2	0	5
2	2	2	2	1	3	3	3	5
5	8	6	5	5	0	0	1	0
1	3	5	4	4	0	2	0	3
1	1	2	4	3	3	3	1	4
3	2	3	1	1	1	5	4	3
1	2	1	1	1	1	1	1	4
2	3	5	2	4	3	1	0	2
3	2	1	5	2	1	4	1	2
1	5	2	2	2	4	3	5	5
3	4	2	2	2	3	3	2	2
1	1	2	1	1	5	3	5	4
4	3	3	5	3	3	2	2	4
1	4	4	2	5	1	1	1	4
3	3	6	3	2	0	2	1	2
3	1	2	4	5	0	3	1	4
3	5	6	4	3	4	2	2	1
1	0	2	2	2	4	1	2	5
2	0	2	2	2	1	1	2	3
0	2	1	2	2	1	2	3	2
1	0	1	1	2	8	3	11	2

1	0	1	2	1	6	1	5	4
4	2	2	3	3	3	2	2	4
4	2	2	2	4	4	5	6	2
7	0	0	3	5	11	6	11	10
12	16	14	13	12	17	14	25	18
2	3	2	1	2	5	2	3	4
4	4	6	6	2	1	1	1	1
4	4	3	2	1	1	3	1	0
1	1	4	1	2	1	3	4	5
0	1	0	1	0	4	2	3	7
0	0	1	2	0	3	7	4	7
2	1	2	1	1	3	2	4	6
2	1	6	2	4	1	2	1	3
3	2	6	1	3	4	3	3	6
4	3	4	3	2	0	2	0	1
3	1	2	1	5	0	2	1	1
6	0	0	9	0	0	13	13	18
1	1	1	1	0	3	2	4	4
1	0	4	2	3	1	3	5	3
1	1	4	5	5	1	2	0	4
4	2	5	4	2	1	2	1	2
4	4	2	1	2	4	3	5	5
2	4	2	3	4	0	3	0	2
2	1	3	3	5	1	0	0	1
0	0	4	3	4	0	5	0	6
5	5	4	3	5	10	6	10	10
2	2	3	4	2	1	3	1	2
2	3	4	4	3	1	1	1	1
6	6	6	5	5	8	5	9	7
0	1	1	2	0	1	1	5	4
0	0	0	1	0	4	4	3	2
6	3	4	2	3	0	3	0	3
4	0	5	2	9	0	2	0	2
0	0	2	3	2	1	1	1	5
4	2	0	3	5	0	6	0	0
5	3	1	4	2	3	4	1	1
2	5	2	2	5	1	3	1	2
4	5	3	3	5	1	2	0	1
0	0	1	0	0	5	3	10	5
5	2	6	6	8	0	1	0	0
4	0	2	4	2	3	1	1	5
1	1	3	2	2	1	1	1	4
0	0	1	1	3	1	2	3	7
4	3	2	2	2	3	2	5	2
3	2	4	3	4	1	1	1	1
1	2	4	2	2	1	2	3	6
3	3	4	2	4	0	1	2	3

1	8	2	2	4	0	2	1	0
4	2	5	3	4	0	1	0	2
1	0	1	2	0	8	0	2	4
2	4	7	5	5	0	3	0	0
4	1	2	1	2	0	4	1	1
2	2	6	12	5	0	2	0	0
0	2	3	1	0	0	0	2	4
2	2	2	2	1	8	3	3	2
1	0	0	2	0	8	3	2	1
1	0	0	1	0	5	2	2	3
0	0	0	0	0	8	5	11	5
3	3	4	2	3	1	1	0	2
0	0	0	0	1	1	3	10	3
4	3	4	4	3	1	3	1	1
2	2	2	2	1	3	2	2	2
4	1	2	2	2	1	1	1	1
2	0	2	1	4	3	3	1	4
2	2	2	2	4	0	1	2	4
0	0	4	4	5	1	0	0	4
0	2	1	1	2	3	2	2	7
2	0	0	2	0	6	3	9	2
4	7	2	0	0	0	8	17	5
0	0	0	0	1	3	1	4	3
1	0	1	0	1	0	0	1	7
3	5	1	2	2	1	0	0	0
0	0	0	0	0	5	5	5	2
0	1	0	0	0	8	4	8	1
0	0	0	2	1	4	0	3	2
19	18	0	14	13	27	20	33	22
4	2	3	4	5	5	3	4	6
1	0	0	2	3	0	3	0	6
0	8	9	9	7	11	0	12	7
0	1	4	0	2	0	1	0	4
3	5	0	0	0	18	0	0	0
13	0	20	12	12	17	14	0	18
2	0	0	0	0	5	0	6	5
2	3	1	2	2	4	4	6	7
3	0	0	0	0	15	5	0	0
0	0	0	1	0	3	0	1	4
8	15	0	5	3	29	18	21	6
4	4	0	6	7	5	7	5	7
0	4	5	5	5	4	5	3	6
0	0	0	4	2	4	3	3	3
4	0	6	0	6	0	11	0	7
0	0	0	0	12	0	0	16	12
0	6	14	6	9	0	5	5	7
0	4	6	0	0	9	0	6	10

5	0	0	3	2	9	5	9	0
3	5	0	0	0	5	5	5	3
9	15	8	0	0	18	11	12	0
0	0	6	2	2	5	0	0	5
0	3	6	2	2	4	0	0	6
0	15	8	0	0	19	12	0	10
0	23	23	0	0	0	26	0	26
0	2	4	0	5	5	0	4	6
3	0	0	2	3	0	5	10	4
6	5	7	0	5	5	0	8	0
0	0	0	5	0	27	14	0	8
0	0	0	0	0	0	0	12	5
2	0	0	0	5	0	0	5	5
42	39	0	0	0	0	0	0	0
0	0	0	0	0	0	0	0	0

VancATN T17.norme	VancATN T18.norme	VancATN T19.norme	Group Mean mean.ATN	mean.IgAN	mean.IgAN	mean.NLTx	mean.S.a.(	mean.Vanc
44	60	76	71.17	51.3	64.59	45.08	107.38	59.45
40	58	41	56.33	48.82	50.34	54.39	53.97	48.74
50	66	61	54.92	44.29	54.09	44.88	51.81	59.31
71	71	56	59.64	37.3	52.43	44.26	40.23	67.11
50	49	46	43.33	43.76	47.93	50.57	38.74	47.2
39	60	50	49.67	48.96	41.45	37.89	51.36	50.8
33	19	19	21.87	56.94	34.88	49.91	31.94	23.11
27	55	41	57.61	35.09	40.17	23.26	60.05	44.16
35	35	30	40.23	38.03	39.72	40.98	39.43	36.75
35	29	33	24.14	34.83	36.85	45.32	34.26	30.72
29	46	61	46.53	37.1	39.14	22.83	86.88	43.84
33	65	52	46.02	41.98	47.03	34.14	56.03	46.34
34	27	36	32.43	44.31	32.93	39.87	35.99	32.04
46	33	34	30.14	30.73	33.59	43.88	25.11	36.18
32	35	36	31.5	41.7	33.15	40.37	28.01	33.45
42	43	42	43.4	21.55	36.04	27.34	29.11	43.32
22	33	50	47.43	26.74	38.6	18.77	51.76	37.89
28	19	28	27.84	39.42	30.95	34.98	25.76	25.74
34	38	46	34.48	33.18	32.62	28.47	36.31	38.31
40	40	29	29.35	32.83	29.67	34.56	17.85	34.99
28	33	32	39.57	25.24	34.72	28.69	30.1	31.17
22	33	36	25.63	35.14	30.88	28.7	39.13	29.02
28	22	23	17.73	40.79	26.91	39.2	19.69	23.11
24	31	17	17.24	41.25	28.65	37.47	17.36	22.05
30	23	24	17.32	35.79	34.36	36.12	17.5	22.47
23	24	30	23.88	30.14	30.5	33.05	23.1	24.99
24	21	24	19.6	35.4	24.48	31.79	20.9	22.59
27	23	25	22.32	28.51	27.6	33.14	17.55	24.28
26	28	32	27.74	31.92	24.68	24.21	36.79	28.54
28	21	23	22.47	28.61	26.78	27.4	16.18	23.34
29	20	27	26.32	21.87	25.31	26.61	13.3	24.46
25	19	15	19.44	24.75	24.17	28.45	18.28	21
23	27	25	19.39	24.78	23.68	27.96	23.91	24.44
3	5	5	4.99	3.76	6.35	2.53	6.17	4.11
23	33	33	30.07	16.28	28.07	17.13	38.22	28.6
21	23	17	16.59	29.88	27.25	24.17	13.71	18.72
23	24	29	25.6	18.57	23.12	22.75	29.41	24.44
18	22	24	23.87	23.63	22.59	20.7	38.18	21.17
3	9	11	15.57	17.38	19.53	20.98	27.77	11.85
26	22	23	21.12	21.73	23.1	25.19	22.66	22.71
25	17	22	19.23	27.69	21.25	26.55	14.71	21.15
28	23	22	19.14	20.88	23.58	21.29	22.92	23.28
0	4	9	19.06	27.67	20.02	19.37	26.04	10.71

16	22	28	23.02	16.48	19.57	18.23	32.12	22.64
11	23	24	29.39	25.42	25.41	22.75	42.13	20.92
25	15	14	15.68	22.35	24.66	23.32	10.19	19.67
21	12	27	11.53	28.11	20.73	23.04	10.73	17.99
19	18	19	18.21	18.17	21.33	20.72	16.2	18.83
18	15	19	17.88	22.65	17.6	24.46	18.02	17.63
22	21	19	24.79	13.37	19.35	16.57	19.32	20.93
24	23	29	25.28	17.27	21.3	18.96	20.54	26
10	17	23	27.48	9.79	21.45	10.98	29.55	19.1
17	13	14	13.9	23.08	19.13	19.45	17.6	14.73
44	25	22	40.28	7.7	21.49	7.28	15.45	31.76
27	27	19	22.69	14.8	18.98	12.96	16.76	25.09
17	20	22	22.02	17.05	17.6	15.66	22.68	20.28
29	33	29	20.52	15.8	12.26	9.32	33.11	30.02
16	28	33	32.91	9.59	20.3	7.58	22.15	26.35
12	17	22	26.39	12.04	18.5	11.32	22.95	19.43
13	14	6	11.71	25.84	14.68	22.34	11.32	9.46
17	12	22	12.97	17.19	16.38	18.14	13.48	15.68
15	16	14	16.14	14.69	17.72	14.98	16.2	14.61
19	7	6	8.1	20.45	16.79	20.14	11.37	10.99
18	17	13	19.49	14.04	16.61	14.5	16.78	16.55
4	9	11	5.87	17.21	20.04	21.44	14.77	8.53
15	13	18	16.14	15.74	16.69	14.24	18.56	16.2
23	15	15	12.44	17.67	16.68	16.8	9.78	16.3
23	19	17	21.11	14.04	16.98	11.54	20.56	20.69
16	13	13	9.01	19.85	14.73	19.91	13.44	13.27
15	10	11	12.42	18.64	13.54	18.37	13.76	12.21
32	28	27	24.51	4.34	15.63	6.74	17.59	28.51
18	14	15	12.72	15.41	14.4	17.32	11.09	14.34
14	13	18	20.28	9.83	17.7	12.45	15.56	16.22
14	19	11	16.5	12.91	18.01	13.92	18.32	16.39
16	19	11	14.65	13.85	17.61	14.96	11.87	14.25
13	12	15	12.54	12.97	14.04	14.16	21.53	14.42
14	17	14	18.5	11.75	14	11.44	16.12	15.59
15	15	17	12.28	16.44	14.73	13.35	15.52	15.45
11	14	15	13.82	17.56	16.64	12.73	20.11	12.9
16	16	17	15.62	14.49	14.53	15.61	14.92	15.68
22	14	4	7.98	8.63	16.58	21.93	1.75	10.9
12	11	10	9.96	19.45	13.06	16.11	12.29	11.31
10	14	4	9.41	16.78	18.89	18.28	1.65	9.6
10	12	14	13.18	12.61	13.97	15.91	11.62	12.56
18	14	11	13.44	11.45	12.85	15.94	9.92	13.83
13	22	13	18.79	13.92	14.55	12.02	19.94	16.12
3	13	11	19.71	2.11	7.38	3.77	12.23	9.34
13	14	8	12.34	16.91	13.05	14.42	9.86	11.41
15	7	10	9.14	13.7	14.7	17.42	5.47	10.43
13	11	13	10.13	16.01	12.26	18.2	7.26	11.66

18	20	17	17.17	12.94	18.02	12.28	15.4	18.5
14	14	13	10.76	16.89	14.12	14.81	11.42	13.34
14	18	14	13.31	14.47	15.86	14.83	10.47	15.14
23	27	23	16.24	14.24	16.08	7.3	14.55	22.41
0	4	0	5.93	7.12	14.53	18.51	32.61	2.19
8	11	13	11.1	16.54	11.91	14.39	9.63	10.87
17	11	6	9.54	17.11	13.11	15.4	9.17	11.38
17	15	8	9.05	10.52	14.11	16.81	4.23	12.25
13	14	14	13.1	11.15	14.35	14.02	10.46	13
13	10	14	15.06	8.87	12.44	14.08	5.86	13.59
12	23	13	18.01	10.87	11.66	10.29	15.42	15
13	12	5	9.92	17.01	12.7	15.46	7.8	10.71
8	9	11	9.35	15.37	11.87	13.76	15.89	9.11
11	17	19	20.05	12.36	10.46	9.45	15.42	15.55
16	9	8	10.9	13.65	10.91	15.16	7.92	10.28
11	6	8	7.33	17.78	11	15.13	9.7	7.56
13	8	11	16.15	12.7	11.97	11.6	8.65	10.84
18	15	20	11.99	14.74	15.47	9.14	13.36	17.48
4	15	18	23.22	5.48	9.15	2.74	23.52	13.98
20	13	15	15.61	10.6	14.7	12.28	12.56	15.88
16	7	9	6.84	15.06	12.64	17.8	3.17	9.38
12	11	17	10.85	12.05	12.33	11.76	12.58	12.9
13	9	6	9.95	11.43	13.47	14.55	7.95	10.99
8	10	10	10.34	14.64	10.34	10.1	13.66	9.07
12	9	11	12.51	13.57	11.81	12.78	9.58	10.65
13	10	5	9.13	12.86	10.54	12.33	8.52	10.23
10	10	13	13.06	15.87	12.56	12.25	11.49	11.27
33	46	39	42.25	34.27	39.05	30.1	48.6	39.72
12	19	14	11.9	6.66	10.31	9.05	14.57	15.47
23	25	24	21.02	22.32	21.8	20.8	20.92	23.63
8	13	13	8.13	14.78	11.16	12.6	14.26	10.71
11	9	13	11.69	10.66	11.76	11.03	14.63	11.93
9	16	9	12.72	11.02	10.49	10.04	13.31	11.17
14	11	8	8.59	10.65	12.66	13.74	4.67	11.07
8	8	8	9.46	12.44	11.38	11.95	5.65	7.2
13	8	11	9.54	12.43	10.37	12.32	8.09	10.14
0	0	11	19.81	18.18	18.5	13.94	21.92	10.56
8	5	3	4.23	14.9	11.41	14.76	7.39	5.19
11	9	10	10.97	10.16	10	12.62	5.52	8.96
16	13	17	19.02	6.04	6.71	5.06	14.79	14.69
12	8	11	9.48	11.25	9.47	10.86	7.51	10.16
13	18	11	13.33	8.34	10.95	9.03	9.37	14.09
10	4	9	4	13.8	11.96	14.75	4.54	6.69
7	13	10	9.5	8.09	11.35	9.41	15.8	10.1
9	13	14	13.77	9.46	10.1	9.75	14.08	12.38
8	10	15	15.66	8.38	8.83	5.41	22.02	11.28
9	13	15	12.09	14.19	12.71	7.84	14.67	11.99

12	8	11	8.55	8.56	10.38	11.91	9.83	10.86
14	6	14	7.18	11.24	12.68	12.36	7	10.45
12	9	11	10.51	12.24	10.02	11.38	8.94	10.18
12	10	27	25.09	1.72	7.06	1.84	21.42	17.75
16	11	6	6.74	8.05	11.06	13.82	5.15	10
20	20	19	20.69	13.91	18.7	15.45	21.13	18.62
15	7	10	9.41	8.99	9.58	8.86	6.85	12.06
9	10	13	12.03	9.83	9.86	8.64	10.33	10.83
18	6	6	5.94	8.65	9.96	12.67	3.89	9.59
8	6	9	5.27	13.67	10.42	12.25	3.65	6.8
11	8	4	9.14	13.61	9.7	11.8	4.43	7.81
5	7	10	9.8	12.01	9.7	7.75	24.67	6.98
10	7	9	7.24	9.48	10.71	11.09	9.6	8.85
10	18	10	12.89	6.6	11.61	7.31	8.16	12.91
8	8	10	11.58	13.68	10.24	11.51	11.42	8.77
8	7	11	8.74	12.16	9.28	10.95	10.96	8.86
11	14	10	11.08	9.25	11.37	8.97	6.65	11.16
5	7	10	7.07	10.56	7.19	8.49	13.17	6.98
12	10	8	11.42	9.15	9.17	10.09	6.54	9.49
2	10	18	18.77	3.4	9.19	0.84	19.7	12.08
10	9	10	11.02	9.49	11.05	8.35	12.46	9.91
15	6	13	6.29	9.01	11.06	11.34	3.57	10.34
13	21	23	22.21	11.99	13.24	9.02	21.48	18.88
8	9	13	9.01	10.84	10.02	9.43	9.99	10.13
12	15	1	5.41	13.76	7.99	9.56	1.34	8.24
8	10	5	7.64	10.39	7.36	9.86	9.13	7.8
8	7	13	5.96	14.59	8.3	10.77	9.21	8.45
13	13	14	12.49	4.81	13.07	6.6	6.7	13.45
7	8	15	11.59	9.14	10.12	7.1	11.73	9.16
8	6	9	7.75	8.59	11.41	10.17	10.79	8.67
14	8	5	6.55	11.58	12.36	9.01	3.87	9.44
11	13	3	8.51	7.63	7.83	9.82	4.9	10.64
9	14	9	12.07	6.61	9.86	5.86	11.64	11.13
10	11	11	10.8	6.56	8.19	9.5	7.43	10.74
8	11	10	12.18	10.31	7.19	8.3	13.84	8.39
12	6	8	7.68	10.14	9.01	10.87	3.94	8.47
28	29	34	27.07	35.77	28.21	28.27	38.78	31
8	9	11	9.54	17.43	12.26	15.41	30.58	9.14
8	10	5	7.52	11.14	7.57	10.9	5.14	7.34
13	8	10	9.18	7.59	9.69	9.49	8.09	10.52
8	4	8	8.18	7.47	8.2	7.82	10.51	7.38
9	10	14	8.6	7.94	10.07	9.07	6.54	10.68
8	3	9	8.2	5.71	9.57	9.29	9.55	8.35
8	10	8	7.06	8.5	8.98	9.51	12.62	7.95
6	11	11	6.45	9.33	8.24	7.33	10.59	8.76
9	14	8	13.82	5.54	6.92	6.49	10.16	11.51
7	12	17	12.45	8.13	7.76	5.96	11.26	10.96

4	2	1	4.73	12.86	7.63	12.4	4.5	3.04
13	11	5	7.56	7.44	8.61	8.13	4.54	9.08
8	4	4	4.51	7.71	10.07	12.05	7.14	5.23
9	5	9	7.33	9.82	8.86	9.14	7.51	7.67
13	6	6	6	11.07	9.6	9.94	1.27	7.63
7	6	8	7.08	8.45	7.52	9.85	9.92	6.51
8	7	4	3.95	7.74	9.16	12.14	3.94	6.02
10	7	4	6.04	12.07	8.33	10.47	5.1	6.42
9	6	6	6.76	9.67	7.73	10.05	4.91	6.59
0	11	10	7.68	6.75	10.57	7.26	11.25	7.68
11	10	10	10.46	5.94	5.98	4.74	9.87	10.14
8	4	6	3.6	12.8	6.56	9.37	4.9	6.1
9	6	6	7.54	8.86	7.65	9.42	6.17	7.29
9	7	6	4.4	8.27	9.57	9.98	2.92	8.01
7	5	8	6.46	7.86	8.69	8.38	3.03	6.72
9	8	10	8.41	7.63	6.85	6.96	12.01	8.75
6	6	8	5.37	9.52	7.64	9.07	8.72	6.3
6	8	4	6.38	11.36	7.54	9.71	8.54	5.62
5	11	11	11.14	7.28	8.36	4.89	10.68	8.32
5	7	4	8.02	8.62	7.93	8.36	6.36	5.63
6	10	10	10.14	5.99	7.71	6.4	10.25	8.65
6	7	9	6.68	10.25	8.1	6.3	11.2	7.11
8	10	9	9.01	4.62	7.61	6.22	11.25	8.5
6	3	5	4.65	8.05	10.25	9.87	9.22	5.13
8	7	10	9.67	11.41	6.87	7.63	7.31	7.84
7	6	10	10.14	10	12.25	8.86	17.32	7.84
5	4	14	8.58	9.51	8.18	5.96	13.17	8.1
3	10	8	6.6	4.13	7.05	6.55	10.26	6.95
8	5	8	6.44	9.77	7.51	9.45	6.24	6.91
9	13	10	9.25	6.84	7	5.91	4.91	11.19
26	8	13	12.24	1.97	8.04	3.12	8.53	13.57
8	6	6	6.57	6.56	6.64	7.44	8.94	6.61
8	3	5	2.74	10.03	8.24	9.25	3.38	6.02
3	16	8	7.59	5.82	6.95	5.46	14.6	9.39
8	7	10	8.27	9.54	7.03	8.11	6.22	8.31
8	13	18	8.62	9.23	12.35	7.89	12.5	12.65
4	5	11	9.99	5.16	6.64	4.99	8.58	7.28
11	4	6	6.66	7.15	7	7.92	5.92	7.43
10	5	5	5.64	6.94	7.44	9.89	3.03	7.16
9	6	6	8.28	5.58	7.17	7.24	6.59	7.05
3	6	5	7.24	6.32	6.28	7.51	2.36	5.27
9	9	1	5.13	6.7	6.18	7.06	1.39	6.08
12	4	3	10.55	9.87	10.15	8.17	2.75	6.92
10	10	10	7.16	9.26	6.52	6.62	5.81	9.47
9	6	5	6.44	5.13	8.86	7.15	6.29	7.2
3	9	4	9.86	7.97	5.01	5.86	5.68	6.89
11	8	4	6.5	5.98	6.11	7.91	3.03	7.58

8	9	11	7.65	7.11	6.62	5.78	9.36	9.6
16	9	4	8.38	10.32	7.95	10.92	5	9.09
2	5	6	7.08	5.83	6.65	7.3	10.08	4.68
4	5	5	5.48	7.75	6.27	8.52	5.71	4.76
8	7	5	5.16	7.71	6.51	7.27	4.79	5.85
3	4	5	3.75	9.21	7.01	7.36	8.31	4.53
9	13	10	10.7	4.02	6.81	4.59	6.68	11.19
4	3	8	4.74	5.66	7.07	8.21	5.33	4.42
6	4	4	4.42	7.62	8.19	8.6	6.83	4.6
4	13	9	8.16	3.88	6.68	3	9.72	8.68
10	7	11	9.49	5.93	6.88	5.92	6.13	9.49
10	9	4	6.58	4.39	6.45	6.18	6.12	7.86
18	11	9	4.45	5.39	6.19	5.39	4.14	10.59
6	3	4	4.34	7.32	6.35	8.38	5.64	4.35
11	19	25	26.76	7.78	18.27	7.04	18.37	19.98
8	7	5	5.22	9.48	6.5	7.36	4.66	6.34
8	4	0	5.48	7.52	7.45	6.46	4.59	5.21
17	16	20	9.7	3.09	4.63	1.91	8.48	16.38
7	7	4	5.98	6.17	7.44	6.25	5.94	5.58
8	6	3	7.94	4.87	6.73	3.91	8.07	5.89
6	1	1	2.76	9.33	5.62	11.27	1.27	2.97
8	2	4	4.25	6.87	5.59	8.82	4.98	5.45
6	7	10	7.58	6.95	6.99	5.25	11.77	7.19
8	7	6	4.3	7.12	7.24	7.05	5.08	5.93
8	8	9	10.79	3.44	6.6	4.73	8.45	8.25
6	7	13	14.34	0.37	3.54	2.08	7.56	9.23
10	6	6	5.98	5.63	6.75	6.85	6.3	8.2
6	8	8	6.31	8.78	6.11	5.16	3.52	6.58
12	9	9	6.61	4.06	6.14	5.29	7.9	9.55
3	4	5	5.48	7.62	5.79	7.43	8.05	4.29
10	9	14	9.52	9.6	10.45	7.99	14.01	10.87
4	6	8	5.94	5.93	6.24	6.93	8.35	5.65
7	2	4	3.05	9.2	6.47	7.6	2.72	3.6
8	11	10	10.09	3.01	6.47	3	9.14	10.03
6	8	14	6.06	3.89	6.08	6.7	5.56	8.63
11	2	4	3.12	7.65	6.79	9.37	1.89	4.88
6	7	8	8.78	4.51	6.6	5.91	6.75	7.26
8	10	6	9.14	3.68	4.79	4.6	6.16	8.35
9	6	11	8.51	4.22	6.51	6.74	4.58	7.62
3	4	3	3.87	7.35	4.04	5.47	10.2	3.19
11	11	0	4.38	5.21	6.66	7.8	2.31	6.93
3	7	10	9.06	3.17	4.99	2.34	10.19	6.8
6	9	10	6.74	7.14	7.44	3.91	9.37	8.4
8	5	3	3.58	7.91	5.74	7.36	5.04	4.73
8	2	5	6.88	5.66	4.72	7.01	3.16	4.83
3	11	6	10.23	5.09	5.14	3.07	10.95	7.59
3	5	5	3.94	6.22	5.18	9.85	3.52	3.87

10	4	1	4.72	5.96	5.7	6.74	5.07	5.72
7	11	10	12.01	12.94	10.96	8.19	24.17	9.35
3	6	8	6.49	5.63	5.73	3.83	7.73	5.91
5	11	5	8.28	3.55	6.63	4.56	6.41	6.96
8	6	6	7.24	3.16	5.56	4.63	2.53	7.34
6	4	6	6.08	6.87	5.98	6.09	3.17	6.17
5	4	8	5.79	8.58	5.63	4.79	7.72	5.11
4	6	9	4.99	4.89	6.29	6.12	6	6.67
3	5	5	3.86	7.38	5.15	8.82	4.91	4.11
3	2	5	7.37	5.39	4.12	6.09	2.31	4.25
3	8	4	3.22	5.69	3.7	5.85	6.7	4.55
4	4	6	3.77	5.1	5.63	7.62	1.82	5.29
10	8	8	4.51	6.43	6.75	6.53	2.38	8.09
5	6	9	7.25	4.31	4.66	6.49	4.13	6.41
4	5	8	7.2	5.81	6.24	5.06	7.26	5.39
3	5	4	4.36	5.26	5.78	6.3	7.07	4
6	5	9	7.27	4.84	6.29	3.4	7.24	6.83
6	9	9	8.44	3.97	6.16	3.43	8.57	7.62
5	3	4	3.62	7	5.35	6.5	5.56	3.91
7	10	11	8.46	3.06	5.15	3.25	7.35	8.71
10	9	6	7.98	10.31	11.36	13.07	4.66	8.03
3	2	5	5.62	6.16	5.9	5.24	7.61	3.32
6	4	3	5.71	5.77	8.28	4.19	2.48	4.99
4	5	1	2.21	5.55	7.14	6.09	2.6	4.51
7	3	8	6.13	3.44	4.62	4.01	7.56	6.68
6	5	3	5.03	6.25	5.29	6.9	4.06	5.01
8	7	8	4.89	4.22	5.49	5.29	3.99	7.67
4	5	6	5.08	3.32	6.46	4.3	6.75	5.31
2	4	5	6.76	6.93	5.85	5.63	9.1	3.17
5	6	0	4.52	6.58	4.82	6.09	3.7	3.49
7	8	8	5.71	1.29	3.57	5	2.48	6.79
5	18	18	20.17	7.43	6.79	2.28	10.72	12.62
7	0	0	1.85	8.79	3.08	9.27	0	1.91
6	3	1	2.34	6.67	4.44	6.55	5.57	3.48
3	4	4	6.22	5.32	4.74	3.94	9.3	3.77
4	7	17	7.91	2.73	4.86	1.9	9.11	9.53
8	7	4	6.95	4.02	5.07	5.73	2.73	6.93
5	7	9	5.55	2.42	4.39	4.83	5.57	6.66
5	6	1	4.34	5.85	5.24	5.63	3.81	4.5
6	5	1	4.17	5.47	5.21	5.28	5.75	4.46
7	7	8	6.15	2.61	7.56	3.99	4.35	7.46
4	9	6	7.54	1.99	5.77	3.69	5.19	6.8
5	6	10	5.56	3.63	5.67	4.74	4.9	6.96
5	2	4	1.54	9.48	6.73	7.58	1.69	3.42
9	2	3	4.38	6.22	4.55	5.29	6.58	4.38
27	18	22	19.25	24.04	21.07	24.94	10.35	21.59
3	7	5	8.47	4.27	3.3	3.18	7.06	5.76

1	8	9	10.77	3.97	5.26	1.8	9.62	5.64
3	3	4	1.23	9.58	6.11	6.25	4.76	2.81
7	3	5	3.01	6.17	5.68	5.64	2.97	4.41
4	8	5	6.58	3.89	5.67	3.81	6.82	6.22
4	6	8	10.18	3.97	3.32	2.66	6.38	6.82
7	4	3	3.77	4.39	4.04	6.31	2.43	4.96
3	5	8	4.26	5.68	6.06	4.15	7.73	5.21
4	0	4	3.12	7.12	5.48	6.97	0.72	2
5	6	9	7.37	2.65	4.22	2.91	4.1	5.71
4	6	5	4.91	2.03	5.14	4.22	6.27	5.01
7	6	4	5.9	1.74	3.75	3.68	3.74	6.02
12	7	9	6.26	4.01	5.89	3.81	2.1	7.87
5	5	1	5.03	3.52	3.5	4.14	5.43	3.55
6	4	4	2.82	5.46	5.25	5.12	3.82	5.07
6	2	0	3.37	8.87	4.39	4.81	3.16	2.44
13	16	8	6.37	1.77	2.8	1.14	5.05	12.62
5	7	9	8.7	3.06	5.91	3.05	7.61	7.6
3	6	5	4.28	4.54	5.06	4.6	6.41	4.8
1	6	3	5.72	4.93	5.5	5.63	4.35	3.07
3	3	4	3.06	4.22	4.02	4.75	5.98	3.28
5	4	1	3.5	3.92	3.03	5.01	2.36	4.23
3	6	8	8.24	2.1	3.69	2.8	4.01	5.21
4	3	8	5.27	3.68	4.45	3.38	4.59	4.88
4	3	3	6.53	3.94	3.87	4.34	3.03	3.15
4	3	3	4.47	3.27	4.05	3.62	5.72	3.85
3	4	1	3.29	5.8	4.14	5.73	3.03	3.34
6	4	3	2.95	4.85	6.75	4.43	1.89	4.29
4	7	13	6.95	3.38	5.58	3.5	6.05	7.41
4	4	5	4.45	3.78	2.64	5.12	2.67	4.74
8	7	9	7.21	1.64	5.28	0	12.95	7.29
6	6	4	6.48	2.98	3.91	4.52	4.47	5.81
5	3	4	4.19	5.46	4.57	5.29	3.09	4.14
3	6	4	4.59	3.57	4.37	3.25	4.58	4.49
7	6	1	5.71	3.35	4.53	5.16	2.91	4.69
3	5	6	5.02	2.42	4.38	2.98	5.97	4.87
6	2	0	1.85	6.55	2.8	6.94	2.59	2.44
3	4	6	3.73	5.31	4.34	3.7	5.2	4.17
4	6	5	6.84	5.39	4.28	4.56	3.57	4.78
4	2	6	4.84	4.59	5.15	4.96	3.94	4.31
4	3	3	4.95	3.86	4.56	5.91	2.04	3.15
4	0	3	1.26	6.22	3.71	6.37	2.85	2.15
3	3	5	5.62	5.3	4.64	5.96	13.09	4.53
1	2	3	4.97	2.02	1.49	4.16	10.64	1.82
3	2	3	3.54	3.81	4.2	5.86	4.23	2.94
7	5	4	3.96	5.39	4.71	4.27	5.01	5.07
4	3	3	3.41	2.57	5.26	5.66	2.73	3.61
3	7	5	2.72	11.02	4.42	4.1	1.07	4.12

4	8	8	8.13	0.67	6.2	2.3	2.41	7.33
3	4	3	4.8	3.68	2.95	2.78	3.99	3.43
3	3	3	3.75	4.2	5.79	4.21	6.66	3.17
3	7	11	8.26	3.68	5.19	2.68	7.07	6.44
3	4	1	4.17	5.8	3.93	4.51	6.46	2.43
3	5	1	2.86	2.88	4.49	5.28	5.37	3.6
3	3	3	5.24	4.38	2.77	5.06	4.06	3.17
3	2	4	5.44	3.4	3.66	2.67	8.93	3.72
3	6	11	6.68	1.37	3.35	3.18	4.34	6.39
4	9	5	3.81	5.11	3.11	2.85	7.3	5.08
4	6	6	6.57	2.23	5.98	2.7	5.54	5.56
4	6	4	3.77	4.3	5.2	5.24	2.48	4.23
6	4	3	4.76	2.98	5.34	4.84	2.11	4.05
5	1	1	3.2	3.81	3.77	2.7	5.98	3
5	2	8	5.28	4.56	4.31	5.97	4.6	5.07
3	5	4	5.91	2.26	4.14	4.86	1.83	4
5	3	5	3.52	3.89	4.57	4.54	3.45	4.46
3	1	1	3.37	6.28	3.62	4.46	4.28	2.11
5	6	4	5.76	2.57	3.15	2.18	5.56	4.91
5	1	0	0.92	4.31	5.74	5.12	3.33	1.74
3	4	4	5.12	2.7	3.07	3.59	3.26	4.91
3	2	5	2.69	3.03	4.69	4.42	0.66	3.55
5	6	4	4.57	2.9	3.75	4.8	0.78	4.67
4	5	3	3.85	4.64	3.78	4.7	3.69	4.12
3	8	3	10.86	2.02	3.1	2.07	6.59	4.47
4	3	1	1.6	5.85	3	6.48	1.46	2.83
6	1	0	1.2	7.04	3.28	5.7	1.22	1.72
7	2	3	2.55	4.31	5.36	5.48	2.79	3.29
7	2	0	1.7	5.96	4	6.12	0	2.18
4	4	1	2.95	4.67	4.64	4.33	4.05	3.55
1	1	1	1.22	5.88	2.81	4.6	3.03	1.48
8	3	1	2.78	4.89	3.34	4.65	3.93	3.64
3	5	8	5.86	3.47	4.12	2.12	6.41	5.44
3	6	1	4.76	3.92	2.81	4.21	5.37	3.64
4	7	1	4.28	3.97	2.83	2.86	3.94	4.32
5	6	5	6.05	1.95	3.28	2.69	3.43	5.46
4	6	1	4.63	1.88	3.2	4.03	3.69	4.53
3	1	3	1.66	5.58	3.89	5.37	2.41	1.52
0	10	0	5.82	4.48	6.98	6.35	10.44	4.65
2	3	0	2.44	3.55	4.21	4.84	5.01	1.65
4	10	4	6.26	0.7	3.2	1.63	5.36	7.35
6	3	1	3.39	6.92	2.96	5.01	0.66	2.78
4	5	4	5.07	3.35	4.41	3.44	2.48	4.21
3	3	5	3.79	3.65	3.12	2.63	5.01	3.57
4	4	3	4.28	1.66	2.29	2.57	2.18	3.87
10	15	13	6.36	0.75	1.91	0.36	5.52	13.01
5	5	1	2.95	2.9	3.68	4.16	2.48	3.31

7	3	3	4.15	3.19	3.07	4.17	1.89	4.01
0	0	0	0	5.49	2.21	7.6	0.66	0
4	4	3	2.93	1.07	3.85	6.24	0.73	2.93
2	3	5	3.31	4.59	3.5	3.76	5.8	3.15
3	4	3	2.48	2.93	3.95	3.64	2.11	2.98
3	5	6	4.91	2.65	2.41	3.08	6.95	4.63
2	5	5	6	2.76	3.34	2.43	7.48	3.9
3	4	6	5.52	3.01	4.38	2.69	4.7	4.61
2	5	1	2.09	5.34	2.52	3.84	4.3	3.64
2	7	8	4.76	3.06	3.25	2.81	4.52	4.81
3	1	1	1.6	4.81	3.31	5.21	1.7	1.88
5	3	1	3.56	2.61	4	3.82	1.45	3.51
6	3	4	4.66	2.73	4.07	3.89	0.31	3.88
3	4	1	1.01	5.38	2.28	6.33	0.31	2.17
8	13	10	7.14	0	1.39	0.36	5.23	10.75
4	3	1	2.84	3.19	3.82	3.72	2.91	3.06
15	17	15	13.01	13.29	9.2	14.94	12.75	15.41
6	6	3	6.28	2.7	2.48	3.66	0.97	4.56
4	4	10	7.69	0.91	3.46	0.55	2.71	6.01
6	4	4	2	3.57	4.14	3.97	2.53	3.9
3	2	1	2.95	3.5	3.71	4.41	1.58	2.39
3	3	6	3.39	3.8	3.54	3.02	2.77	4.59
3	3	3	3.08	8.44	4.55	7.38	2.79	2.7
5	2	6	2.68	2.98	3.98	3.64	2.18	4.29
3	5	6	5.1	2.41	2.96	2.2	4.16	4.63
8	5	8	5.22	0	5.21	0.37	6.43	6.44
6	2	0	3.85	1.41	5.64	1.11	6.23	1.98
2	2	3	1.23	2.28	3.07	3.87	3.14	1.8
4	4	0	3.33	3.4	1.21	3.17	3.81	3.7
1	7	5	6.98	0.95	3.54	1.46	6.51	5.13
8	2	0	2.4	3.03	2.6	3.84	2.11	3.3
3	3	3	2.63	1.99	3.01	2.6	4.66	3.17
8	1	1	2.86	2.22	5.07	3.85	0.31	2.92
3	7	8	3.86	4.78	2.45	1.61	5.07	5.23
3	4	3	2.97	3.47	3.71	3.06	4.3	2.96
2	1	3	2.59	4.17	2.68	3.43	4.42	1.78
3	5	4	2.8	3.73	2.67	2.06	6.41	3.56
4	1	0	0.71	4.01	2.67	3.54	2.61	1.77
4	1	3	2.55	4.22	2.28	3.98	1.09	2.87
1	1	3	1.75	3.24	4.28	4.43	1.33	1.8
8	5	1	1.64	2.79	3.87	3.25	2	4.64
6	3	0	1.62	3.44	2.46	2.92	1.04	2.93
3	6	6	2.78	4.22	3.26	3.57	2.72	4.21
2	1	3	1.45	2.93	3.66	3.66	3.26	1.78
3	4	3	1.81	7.57	3.98	6.48	4.18	2.49
3	1	6	2.02	3.99	3.21	4.01	1.87	3.17
3	6	4	5.42	1.21	2.45	2.61	1.75	4.72

5	2	4	5.44	1.32	2.92	1.76	3.57	4.12
3	2	4	3.54	2.9	3.45	3.19	3.14	2.79
4	2	1	4.38	1.61	2.44	2.62	3.93	2.57
3	4	3	4.86	3.43	1.7	1.77	4.28	3.22
3	1	3	2.17	4.57	4	4.12	0.97	1.75
2	1	1	1.62	3.26	3.26	4.09	2.49	1.46
13	7	6	7.86	3.85	10.25	8.65	7.26	9.06
4	1	6	2.25	2.1	3.89	3.42	1.94	4.52
1	2	4	2.4	4.3	3.14	3.32	4.34	2.37
26	23	27	23.13	24.68	20.47	20.56	28.87	25.56
3	2	1	4.49	0.99	2.84	1.97	5.25	3.09
4	3	0	3.05	2.49	2.65	4.26	2.06	2.51
3	0	3	2.49	4.08	1.85	4.09	4.3	1.73
5	5	6	5.42	0.75	3.38	1.69	2.73	5.75
4	8	1	3.14	2.05	2.77	1.55	3.7	3.87
3	4	8	6.72	0.7	3.18	0.78	2.53	5.65
0	0	4	0.23	2.75	2.99	2.16	9.87	2.12
3	4	4	2.61	2.9	3.35	2.4	4.71	3.77
2	4	1	4.64	2.28	2.65	1.82	1.46	2.46
4	4	4	4.68	2.36	3.5	3.31	2.79	3.95
2	1	8	5.05	2.31	3.03	1.57	4.71	3.28
5	6	3	6.55	1.66	3.07	1.73	1.8	4.82
7	4	1	2.82	3.01	2.06	3.46	2.06	3.48
7	4	1	1.77	2.03	2.66	2.57	0.73	4.41
2	3	5	2.99	2.31	2.8	1.89	3.86	3.39
3	3	1	2.72	3.55	2.48	3.37	1.7	3.09
3	2	4	3.37	2.26	1.69	3.25	1.04	3.47
3	4	4	4.66	2.61	2.7	1.54	2.84	3.77
3	1	4	1.91	3.65	2.63	5.49	0.36	1.84
4	4	3	3.39	2.06	3.75	2.79	0.61	3.4
1	1	1	5.17	1.02	4.62	1.44	2.12	1.72
3	2	3	2.97	3.47	3.42	1.9	3.38	2.68
5	10	4	4.09	2.03	4.58	1.26	1.09	5.69
2	1	3	1.91	3.37	3.11	3.43	1.15	1.78
4	2	1	3.12	3.6	2.04	3.17	2	2.34
3	3	6	3.22	1.94	2.84	1.88	3.98	4.15
2	2	1	2.91	3.84	3.04	2.94	2.48	1.71
0	3	8	4.89	2.36	2.66	1.46	4.4	3.6
3	1	3	3.14	2.49	2.7	3.42	2.18	2.66
4	3	5	5.14	1.58	2.25	2.69	1.09	4.01
5	3	0	2.25	2.53	2.53	3.08	0.97	2.49
3	2	1	2.34	2.56	3.44	3.02	1.27	2.6
3	0	3	0.78	2.36	3.74	3.51	2.6	1.5
3	3	4	5.35	0.62	2.45	1.27	2.29	3.72
3	7	9	5.89	1.07	2.27	1.27	1.45	5.54
2	4	3	3.42	1.64	3.61	1.21	2.11	2.54
3	2	5	4.97	1.53	2.75	0.75	7.04	3.08

3	6	9	7.21	1.41	2.32	0.63	4.22	5.52
3	2	4	2.82	4.1	2.64	2.86	2.18	3.23
2	4	3	3.14	1.94	2.37	2.88	4.95	2.54
5	8	4	7.54	7.86	5.85	4.16	9.54	6.82
14	15	20	18.68	14.52	13.92	12.91	18.33	16.9
3	6	3	4.55	1.69	2.29	2.16	3.38	3.94
3	1	3	2.08	2.57	2.88	3.69	1.09	1.96
3	2	1	2.38	3.77	2.63	3.61	1.7	1.46
4	2	5	3.52	0.33	2.37	1.77	2.77	3.99
4	5	3	7.51	0.7	2.05	0.59	2.96	4.59
1	5	5	6.21	0	2.34	0.55	4.72	4.39
3	5	8	4.66	1.33	2.4	1.59	2.89	5.21
2	0	3	3.52	2.61	2.26	3.03	1.39	1.75
3	2	3	2.04	1.29	3.11	2.5	3.26	3.17
3	0	1	1.35	4.59	2.24	3.35	0.61	1.39
2	0	1	1.37	2.37	4.06	3.29	0.97	0.97
5	0	13	9.93	5.87	5.63	5.05	8.57	8.87
3	5	5	5.54	2.95	2.5	0.8	2.89	4.11
4	2	1	2.19	1.89	2.12	1.45	3.13	2.57
3	2	4	3.75	1.32	3.26	2.28	1.04	3.23
2	1	1	0.7	3.89	1.92	3.43	1.39	1.46
3	3	3	2.68	3.88	1.3	2.27	3.98	3.19
4	3	3	1.11	2.68	2.01	3.49	0.92	2.91
2	1	1	1.43	1.07	3.68	2.43	0.42	1.22
1	4	4	3.45	0.96	2.02	1.74	1.53	3.58
5	5	5	6.49	5.54	5.89	4.81	8.75	6.37
3	2	1	2.02	3.26	2.97	2.39	1.7	2.13
3	0	4	1.79	1.61	3.23	3.07	1.09	1.81
8	9	11	6.99	1.76	5.66	3.99	7.24	8.67
3	2	1	4.01	0.33	1.53	0.89	2.52	2.39
1	8	4	4.6	3.58	1.64	0.1	3.57	3.67
0	1	1	0.97	5.13	1.57	3.81	0.92	1.27
3	0	0	0	2.36	2.5	3.73	0.61	1.09
4	5	6	5.58	0.62	2.47	0.91	1.09	5.08
3	0	0	1.7	4.59	2.37	2.51	2.14	0.63
1	1	1	1.54	2.61	1.88	3.5	2.43	1.02
3	1	1	1.43	3.35	2.99	2.75	1.7	1.67
2	2	0	0.72	2.95	1.77	3.39	1.04	1.16
3	4	6	4.47	2.02	1.56	0.1	5.84	4.61
0	0	0	0.46	2.76	1.78	4.57	0.31	0
3	1	5	4.65	0.96	1.03	1.93	1.51	3.32
0	2	6	2.61	1.29	3.06	1.25	1.09	3.03
1	3	3	6.09	0.59	2.25	0.83	2.11	3.48
2	1	1	2.65	2.26	2.25	2.85	3.25	1.46
3	3	1	1.28	2.79	2.26	2.93	1.09	2.15
1	4	3	2.85	1.61	2.36	1.37	2.11	3.26
5	1	1	1.2	2.31	2.23	2.84	1.02	2.53

3	1	0	2.19	0.96	1.58	2.79	0.97	0.88
2	0	0	0.55	2.7	3.07	3.27	0.31	0.89
1	6	3	7.18	0.37	1.41	0.41	3.26	3.31
1	0	0	0.23	2.61	1.9	4.11	0.92	0.21
1	0	0	1.89	4.56	2.91	2.35	1.58	0.44
3	0	0	0.49	1.61	2.06	3.92	0.61	0.63
4	4	3	2.89	0.99	5.75	0.75	0.72	3.63
1	1	4	3.71	1.37	3.01	1.45	4.54	1.88
2	4	4	4.78	1.37	1.87	0.8	4.18	2.62
0	2	1	5.54	1.37	1.25	0.33	3.02	1.53
0	3	1	4.18	0.7	0.44	0	7.65	2.25
4	0	1	0.55	3.37	2.76	2.86	0.73	1.83
2	8	8	3.74	0	0.91	0.22	4.56	5.06
1	1	3	1.03	2.2	1.1	3.54	1.7	1.33
3	3	1	4.13	0.62	3.15	1.2	2.18	2.18
2	2	0	0.7	5.13	1.88	2.69	1.09	1.16
4	4	6	2.84	0.37	2.07	1.36	2.12	4.59
2	3	3	3.73	0.33	1.78	1.69	1.02	2.75
2	3	1	4.03	0	2.99	1.58	0.42	2.43
4	4	5	6.98	0.75	0.8	0.89	2.18	4.97
0	1	8	3.5	2.56	2.92	0.88	5.9	2.63
2	9	6	2.93	2.31	2.57	1.99	8.48	5.47
4	3	1	4.84	0.65	1.25	0.34	2.59	2.83
4	7	1	3.25	0.75	1.62	0.43	0.36	4.78
0	2	0	1.28	3.66	1.3	2.54	0.42	0.51
0	1	5	3.58	0.7	1.85	0.12	5.01	1.99
0	2	3	3.19	0	1.54	0.48	6.27	1.38
3	4	6	3.84	0	1.86	0.42	2.35	3.91
17	20	24	15.77	20.13	10.35	14.31	26.73	20.92
4	5	10	5.03	2.67	5.35	2.78	4.05	6.26
3	1	0	4.57	0.29	1.43	1.16	0.92	2.28
8	11	11	6.78	6.71	8.35	6.01	7.76	9.41
4	1	0	1.94	0	2.39	0.92	0.31	2.24
0	0	0	0	0	0	3.05	5.94	0
0	21	0	12.37	8.75	11.78	10.06	10.1	9.79
5	6	6	1.31	0.65	1.32	0.48	3.85	5.54
3	7	3	4.13	0.95	3.45	1.9	4.64	4.68
0	4	8	2.38	0	2.74	2.07	6.62	2.92
1	2	3	5.31	0	0.2	0.1	1.21	2.29
5	8	10	10.03	12.68	9.81	7.6	23.03	7.24
6	9	5	5.33	4.73	5.74	4.31	5.93	6.66
4	5	5	5.73	2.65	2.9	2.63	4.18	4.99
4	0	0	2.43	0	1.66	1.04	3.26	1.75
8	0	0	9.83	5.9	3.58	2.62	3.66	3.75
0	10	0	13.97	2.35	6.19	2.53	5.37	5.58
11	8	6	9.82	7.86	7.66	6.28	3.62	7.98
13	13	14	8.43	2.61	7.69	1.19	5.12	12.51

0	5	8	3.96	3.87	5.26	2.69	7.67	3.18
3	3	0	3.52	3.55	1.74	1.54	5.01	2.09
0	0	0	0	10.91	5.42	7.51	13.54	0
3	2	6	3.65	1.95	3.41	1.87	1.7	4.1
4	3	5	1.88	0	2.72	1.66	1.27	4.48
6	0	0	2.96	4.23	7.6	5.56	10.33	4.03
21	24	0	10.93	8.79	17.58	8.11	8.55	17.89
3	7	3	6.42	0.59	3.4	1.58	3.13	4.66
3	3	9	3.9	1.12	2.83	1.4	4.75	4.76
0	0	4	0	0	6.25	5.55	4.2	0.95
3	0	0	6.81	7.54	3.38	0.6	13.49	2.94
0	0	6	3.19	0	3.27	1.07	3.94	2.75
3	7	5	6.51	0.75	1.58	0.9	1.79	5.06
0	0	0	0	0	11.29	26.25	0	0
5	0	0	0.99	0	3.18	0	0	1.26

ATN.vs.NL	ATN.vs.NL	ATN.vs.NL	IgAN.vs.NL	IgAN.vs.NL	IgAN.vs.NL	IgAN E1.vs	IgAN E1.vs	IgAN E1.vs
0.66	1.58	0.0019	0.2	1.15	0.413	0.51	1.42	0.0172
0.05	1.04	0.7302	-0.16	-1.12	0.3303	-0.11	-1.08	0.4353
0.29	1.23	0.0796	-0.02	-1.01	0.9123	0.27	1.21	0.1041
0.4	1.32	0.01	-0.25	-1.19	0.1899	0.23	1.17	0.142
-0.23	-1.17	0.1487	-0.22	-1.16	0.2124	-0.07	-1.05	0.6262
0.37	1.29	0.0326	0.35	1.27	0.0636	0.11	1.08	0.5426
-1.15	-2.22	0	0.18	1.13	0.4623	-0.49	-1.4	0.0319
1.25	2.38	0	0.57	1.48	0.0706	0.75	1.68	0.0089
-0.04	-1.03	0.8312	-0.1	-1.07	0.5889	-0.04	-1.03	0.8116
-0.9	-1.87	0	-0.38	-1.3	0.0804	-0.3	-1.23	0.1261
1	2	4.00E-04	0.69	1.61	0.0257	0.75	1.69	0.0072
0.41	1.33	0.0329	0.29	1.23	0.1696	0.45	1.36	0.0192
-0.29	-1.22	0.1158	0.16	1.12	0.3838	-0.28	-1.22	0.1168
-0.55	-1.46	0.0038	-0.53	-1.44	0.011	-0.38	-1.3	0.0366
-0.37	-1.29	0.0345	0.03	1.02	0.8776	-0.28	-1.22	0.0939
0.63	1.55	0.0046	-0.32	-1.25	0.2306	0.36	1.29	0.105
1.31	2.48	0	0.51	1.43	0.088	1	2	2.00E-04
-0.32	-1.25	0.1388	0.17	1.13	0.4403	-0.16	-1.12	0.4389
0.28	1.21	0.1464	0.22	1.17	0.2908	0.16	1.12	0.3972
-0.24	-1.18	0.204	-0.08	-1.06	0.7009	-0.23	-1.17	0.2227
0.44	1.36	0.0272	-0.2	-1.15	0.4011	0.25	1.19	0.2152
-0.17	-1.13	0.4278	0.3	1.23	0.1694	0.09	1.07	0.6397
-1.13	-2.19	0	0.05	1.04	0.7987	-0.52	-1.43	0.007
-1.08	-2.12	0	0.13	1.1	0.6038	-0.36	-1.28	0.1348
-1.03	-2.04	2.00E-04	-0.03	-1.02	0.9034	-0.06	-1.05	0.7923
-0.49	-1.4	0.0151	-0.14	-1.1	0.5039	-0.12	-1.08	0.5222
-0.69	-1.61	0.002	0.16	1.11	0.4641	-0.35	-1.28	0.0864
-0.56	-1.48	0.0054	-0.22	-1.17	0.2857	-0.26	-1.2	0.165
0.19	1.14	0.3292	0.39	1.31	0.0575	0.03	1.02	0.8655
-0.29	-1.23	0.1632	0.03	1.02	0.8727	-0.04	-1.03	0.848
-0.04	-1.03	0.8637	-0.29	-1.23	0.2178	-0.08	-1.06	0.703
-0.54	-1.46	0.0134	-0.19	-1.14	0.3979	-0.23	-1.17	0.2592
-0.53	-1.44	0.0176	-0.15	-1.11	0.5121	-0.24	-1.18	0.2511
0.86	1.82	0.1067	0.53	1.44	0.359	1.07	2.1	0.0401
0.77	1.7	7.00E-04	-0.04	-1.03	0.8764	0.7	1.62	0.0019
-0.51	-1.43	0.059	0.28	1.22	0.2977	0.18	1.14	0.4608
0.17	1.13	0.4277	-0.28	-1.21	0.2816	0.03	1.02	0.9083
0.21	1.16	0.3485	0.19	1.14	0.4341	0.13	1.1	0.551
-0.38	-1.3	0.4509	-0.24	-1.18	0.6495	-0.11	-1.08	0.8268
-0.24	-1.18	0.2668	-0.2	-1.15	0.3931	-0.11	-1.08	0.6025
-0.47	-1.39	0.0348	0.04	1.03	0.8576	-0.34	-1.26	0.1151
-0.15	-1.11	0.5194	-0.05	-1.03	0.8479	0.15	1.11	0.4993
-0.02	-1.01	0.9782	0.37	1.29	0.542	0.04	1.03	0.9498

0.32	1.25	0.1962	-0.11	-1.08	0.7075	0.1	1.07	0.6954
0.38	1.3	0.0779	0.17	1.13	0.469	0.17	1.13	0.4233
-0.58	-1.5	0.0419	-0.07	-1.05	0.8186	0.09	1.07	0.7242
-0.95	-1.94	0.001	0.26	1.19	0.345	-0.14	-1.1	0.5876
-0.17	-1.12	0.4711	-0.19	-1.14	0.4387	0.03	1.02	0.8841
-0.44	-1.36	0.0732	-0.12	-1.09	0.6385	-0.48	-1.39	0.047
0.57	1.49	0.0115	-0.28	-1.21	0.3239	0.2	1.15	0.3934
0.38	1.3	0.0885	-0.15	-1.11	0.5771	0.16	1.12	0.4813
1.28	2.43	0	-0.11	-1.08	0.7738	0.92	1.89	0.0035
-0.46	-1.37	0.0738	0.24	1.18	0.3327	-0.05	-1.03	0.8406
2.3	4.93	0	0.15	1.11	0.7367	1.47	2.78	1.00E-04
0.8	1.74	0.0069	0.19	1.14	0.568	0.55	1.46	0.0668
0.47	1.39	0.0519	0.13	1.1	0.6306	0.15	1.11	0.551
1.1	2.15	0.001	0.76	1.69	0.0414	0.37	1.29	0.293
1.99	3.98	0	0.35	1.28	0.3652	1.31	2.48	1.00E-04
1.16	2.23	2.00E-04	0.09	1.07	0.8002	0.66	1.58	0.0406
-0.9	-1.86	0.0027	0.19	1.14	0.5046	-0.55	-1.46	0.0491
-0.47	-1.38	0.0772	-0.08	-1.06	0.7648	-0.13	-1.1	0.582
0.1	1.07	0.6973	-0.01	-1.01	0.9623	0.22	1.17	0.3702
-1.16	-2.23	0.0015	0	1	0.9919	-0.25	-1.19	0.4584
0.44	1.35	0.0885	-0.04	-1.02	0.9046	0.22	1.16	0.4047
-1.79	-3.45	0	-0.32	-1.25	0.2701	-0.09	-1.06	0.7251
0.18	1.13	0.5208	0.13	1.09	0.6759	0.21	1.16	0.4261
-0.43	-1.35	0.1206	0.06	1.04	0.8442	0	-1	0.9994
0.84	1.79	0.0012	0.27	1.21	0.368	0.53	1.44	0.0436
-1.08	-2.11	4.00E-04	0	-1	0.9873	-0.4	-1.32	0.1415
-0.52	-1.44	0.0564	0.02	1.01	0.9448	-0.43	-1.34	0.1063
1.68	3.2	3.00E-04	-0.35	-1.28	0.5137	1.11	2.15	0.0191
-0.43	-1.34	0.1357	-0.16	-1.12	0.5983	-0.27	-1.2	0.327
0.66	1.58	0.0138	-0.31	-1.24	0.3407	0.47	1.38	0.0787
0.23	1.17	0.3909	-0.11	-1.08	0.7027	0.34	1.26	0.1833
-0.04	-1.03	0.8907	-0.1	-1.07	0.7503	0.24	1.18	0.3883
-0.14	-1.1	0.6239	-0.11	-1.08	0.7212	0	1	0.9998
0.68	1.61	0.011	0.08	1.06	0.7985	0.26	1.2	0.3437
-0.1	-1.07	0.7341	0.31	1.24	0.271	0.16	1.11	0.5539
0.09	1.07	0.7384	0.44	1.36	0.1239	0.34	1.27	0.194
-0.03	-1.02	0.8995	-0.11	-1.08	0.6956	-0.1	-1.07	0.6987
-1.36	-2.56	0.0029	-1.26	-2.4	0.0093	-0.42	-1.34	0.3326
-0.64	-1.56	0.0304	0.27	1.21	0.3366	-0.29	-1.22	0.2968
-0.88	-1.84	0.0138	-0.16	-1.12	0.6597	0.03	1.02	0.921
-0.26	-1.2	0.3374	-0.3	-1.23	0.3093	-0.18	-1.13	0.4915
-0.26	-1.19	0.348	-0.47	-1.38	0.1286	-0.3	-1.23	0.2602
0.64	1.55	0.0151	0.21	1.16	0.4764	0.27	1.21	0.3132
2.12	4.34	0	-0.46	-1.37	0.4186	0.82	1.76	0.0923
-0.25	-1.19	0.3813	0.19	1.14	0.4956	-0.14	-1.1	0.6134
-0.88	-1.85	0.0088	-0.34	-1.26	0.3243	-0.22	-1.17	0.4732
-0.81	-1.75	0.0059	-0.2	-1.15	0.4938	-0.58	-1.49	0.0364

0.45	1.36	0.1207	0.05	1.04	0.8727	0.53	1.44	0.0601
-0.46	-1.37	0.1067	0.19	1.14	0.5012	-0.08	-1.06	0.7568
-0.16	-1.12	0.5708	-0.05	-1.04	0.8573	0.07	1.05	0.796
1.1	2.14	7.00E-04	0.91	1.89	0.0097	1.07	2.1	8.00E-04
-1.19	-2.28	0.0739	-0.98	-1.98	0.1495	-0.35	-1.28	0.5947
-0.36	-1.28	0.2206	0.18	1.13	0.5463	-0.26	-1.2	0.35
-0.66	-1.58	0.0402	0.15	1.11	0.644	-0.23	-1.17	0.4485
-0.82	-1.76	0.0422	-0.65	-1.57	0.1271	-0.25	-1.19	0.5166
-0.07	-1.05	0.7911	-0.31	-1.24	0.3202	0.02	1.01	0.9466
0.06	1.04	0.8462	-0.64	-1.56	0.0696	-0.16	-1.12	0.5932
0.75	1.68	0.0139	0.09	1.07	0.7895	0.2	1.15	0.5276
-0.6	-1.52	0.0403	0.12	1.09	0.667	-0.25	-1.19	0.3466
-0.52	-1.43	0.1034	0.16	1.12	0.6028	-0.2	-1.15	0.4914
1.02	2.02	5.00E-04	0.39	1.31	0.248	0.13	1.1	0.668
-0.44	-1.36	0.1975	-0.17	-1.12	0.6464	-0.42	-1.34	0.2103
-0.96	-1.95	0.0035	0.21	1.15	0.4962	-0.43	-1.34	0.1507
0.42	1.33	0.138	0.12	1.08	0.7137	0.04	1.03	0.8898
0.39	1.31	0.3009	0.64	1.56	0.1052	0.71	1.64	0.0494
2.69	6.47	0	0.94	1.92	0.0847	1.52	2.86	0.0024
0.33	1.25	0.2284	-0.21	-1.16	0.5067	0.22	1.16	0.4195
-1.29	-2.44	6.00E-04	-0.27	-1.21	0.462	-0.46	-1.38	0.1723
-0.11	-1.08	0.7097	0.04	1.03	0.9014	0.06	1.05	0.8228
-0.53	-1.44	0.1002	-0.35	-1.27	0.3093	-0.13	-1.09	0.6742
0.05	1.04	0.8682	0.53	1.45	0.0951	0.05	1.04	0.8698
-0.05	-1.04	0.86	0.08	1.06	0.789	-0.11	-1.08	0.6942
-0.42	-1.34	0.1963	0.05	1.04	0.8792	-0.21	-1.16	0.4869
0.08	1.06	0.7867	0.33	1.26	0.288	0.02	1.02	0.9359
0.49	1.41	0.0071	0.18	1.14	0.3746	0.37	1.29	0.0452
0.4	1.32	0.1869	-0.39	-1.31	0.2972	0.19	1.14	0.5434
0.02	1.02	0.9213	0.1	1.07	0.673	0.04	1.03	0.8455
-0.6	-1.52	0.0637	0.22	1.17	0.4759	-0.17	-1.13	0.5548
0.1	1.07	0.7445	-0.04	-1.03	0.9084	0.12	1.09	0.6913
0.32	1.25	0.2934	0.12	1.09	0.7245	0.03	1.02	0.9275
-0.65	-1.57	0.0562	-0.37	-1.29	0.3003	-0.12	-1.09	0.6926
-0.31	-1.24	0.3802	0.03	1.02	0.9392	-0.05	-1.03	0.8837
-0.36	-1.28	0.2653	-0.02	-1.01	0.9492	-0.27	-1.21	0.3768
0.36	1.28	0.5971	0.27	1.21	0.6986	0.29	1.23	0.6653
-1.62	-3.08	0	-0.01	-1.01	0.9729	-0.34	-1.27	0.2674
-0.22	-1.17	0.4759	-0.31	-1.24	0.3592	-0.33	-1.26	0.286
1.82	3.52	0	0.28	1.22	0.5007	0.37	1.29	0.3261
-0.19	-1.14	0.5397	0.02	1.02	0.9481	-0.17	-1.12	0.5897
0.52	1.44	0.0874	-0.09	-1.06	0.8057	0.25	1.19	0.4212
-1.71	-3.26	0	-0.12	-1.09	0.714	-0.28	-1.22	0.3625
0.01	1.01	0.9804	-0.19	-1.14	0.6132	0.21	1.16	0.5013
0.49	1.4	0.1037	0	-1	0.9922	0.05	1.03	0.883
1.46	2.76	1.00E-04	0.63	1.55	0.1287	0.67	1.59	0.0771
0.63	1.54	0.0566	0.8	1.74	0.0211	0.64	1.55	0.0482

-0.47	-1.39	0.1491	-0.47	-1.38	0.1906	-0.24	-1.18	0.4412
-0.74	-1.67	0.0325	-0.13	-1.09	0.7043	0.01	1.01	0.9683
-0.13	-1.09	0.6764	0.09	1.06	0.7814	-0.15	-1.11	0.6216
3.22	9.29	0	0.35	1.28	0.5685	1.61	3.05	0.0036
-0.96	-1.94	0.0102	-0.73	-1.66	0.0605	-0.31	-1.24	0.3621
0.42	1.34	0.0784	-0.12	-1.09	0.6686	0.27	1.21	0.2545
0.07	1.05	0.8433	0.04	1.03	0.9135	0.13	1.09	0.6976
0.44	1.36	0.1547	0.18	1.13	0.5992	0.16	1.11	0.6209
-0.99	-1.99	0.015	-0.53	-1.44	0.2057	-0.33	-1.25	0.3873
-1.12	-2.17	0.0027	0.1	1.07	0.7683	-0.24	-1.18	0.4577
-0.35	-1.28	0.2735	0.16	1.12	0.6198	-0.25	-1.19	0.4163
0.37	1.29	0.3307	0.61	1.53	0.13	0.32	1.25	0.3995
-0.58	-1.49	0.0845	-0.21	-1.16	0.5388	-0.04	-1.03	0.8806
0.79	1.73	0.0174	-0.07	-1.05	0.8676	0.63	1.55	0.0563
0.01	1.01	0.9814	0.26	1.19	0.4341	-0.18	-1.13	0.5729
-0.31	-1.24	0.3746	0.15	1.11	0.6766	-0.22	-1.16	0.5187
0.29	1.22	0.3625	0.04	1.03	0.9181	0.31	1.24	0.3156
-0.23	-1.17	0.5339	0.31	1.24	0.4046	-0.21	-1.15	0.5669
0.15	1.11	0.6502	-0.14	-1.1	0.6925	-0.1	-1.07	0.7489
2.47	5.56	8.00E-04	1.27	2.42	0.0839	1.87	3.65	0.0112
0.38	1.3	0.226	0.18	1.13	0.6122	0.37	1.29	0.2282
-0.79	-1.73	0.0489	-0.36	-1.28	0.3854	-0.04	-1.03	0.9115
1.25	2.37	0	0.41	1.33	0.2168	0.52	1.44	0.0812
-0.05	-1.03	0.8811	0.2	1.15	0.5658	0.08	1.05	0.8105
-0.76	-1.69	0.0907	0.42	1.34	0.3335	-0.24	-1.18	0.5734
-0.37	-1.3	0.273	0.07	1.05	0.8468	-0.41	-1.33	0.2231
-0.79	-1.73	0.0267	0.42	1.34	0.193	-0.36	-1.28	0.2658
0.82	1.77	0.0196	-0.36	-1.29	0.4035	0.89	1.85	0.01
0.68	1.6	0.0591	0.33	1.26	0.4133	0.44	1.36	0.2228
-0.31	-1.24	0.4185	-0.2	-1.15	0.6282	0.15	1.11	0.6668
-0.37	-1.29	0.3485	0.33	1.26	0.4007	0.45	1.37	0.207
-0.22	-1.16	0.5498	-0.36	-1.29	0.3582	-0.26	-1.2	0.4617
0.94	1.92	0.0159	0.17	1.12	0.7041	0.68	1.6	0.0829
0.13	1.1	0.6823	-0.5	-1.41	0.1913	-0.22	-1.16	0.5139
0.53	1.44	0.0984	0.32	1.25	0.3627	-0.19	-1.14	0.5864
-0.49	-1.41	0.1696	-0.13	-1.09	0.7267	-0.27	-1.21	0.425
-0.06	-1.04	0.7594	0.33	1.26	0.0858	0.01	1.01	0.9446
-0.62	-1.54	0.0846	0.17	1.13	0.6337	-0.3	-1.23	0.3882
-0.49	-1.4	0.1505	0.01	1.01	0.9752	-0.48	-1.4	0.1445
-0.06	-1.04	0.851	-0.3	-1.23	0.4147	0.04	1.03	0.8881
0.08	1.05	0.8298	-0.04	-1.03	0.9202	0.1	1.07	0.7729
-0.06	-1.04	0.8595	-0.19	-1.14	0.6066	0.13	1.1	0.6704
-0.13	-1.1	0.7083	-0.64	-1.56	0.122	0.04	1.03	0.9039
-0.37	-1.29	0.281	-0.14	-1.1	0.6913	-0.06	-1.04	0.861
-0.16	-1.12	0.6592	0.31	1.24	0.3947	0.19	1.14	0.5815
1.03	2.04	0.0018	-0.17	-1.12	0.6826	0.08	1.06	0.8225
0.99	1.99	0.0042	0.42	1.34	0.2893	0.38	1.3	0.2962

-1.23	-2.34	0.0115	-0.03	-1.02	0.9494	-0.64	-1.56	0.1666
-0.09	-1.07	0.8069	-0.12	-1.09	0.77	0.11	1.08	0.758
-1.28	-2.44	7.00E-04	-0.61	-1.52	0.0961	-0.26	-1.2	0.3983
-0.31	-1.24	0.3599	0.09	1.07	0.7873	-0.04	-1.03	0.905
-0.67	-1.59	0.0774	0.11	1.08	0.7782	-0.04	-1.03	0.9016
-0.46	-1.38	0.1795	-0.2	-1.15	0.5734	-0.36	-1.29	0.2717
-1.47	-2.76	3.00E-04	-0.64	-1.56	0.097	-0.39	-1.31	0.2517
-0.72	-1.65	0.0497	0.17	1.13	0.6179	-0.28	-1.22	0.3999
-0.56	-1.48	0.1117	-0.09	-1.07	0.7921	-0.34	-1.27	0.3054
0.06	1.05	0.899	-0.06	-1.04	0.9127	0.45	1.36	0.3715
1.05	2.07	0.0045	0.32	1.25	0.4578	0.31	1.24	0.4252
-1.23	-2.34	0.0028	0.41	1.33	0.2414	-0.51	-1.42	0.1555
-0.32	-1.25	0.3552	-0.1	-1.07	0.7866	-0.25	-1.19	0.4585
-1.11	-2.16	0.017	-0.31	-1.24	0.5097	-0.11	-1.08	0.8029
-0.38	-1.3	0.3096	-0.12	-1.09	0.7564	0.02	1.01	0.961
0.28	1.22	0.4202	0.13	1.1	0.7256	0	-1	0.9957
-0.68	-1.61	0.0644	0.06	1.04	0.8705	-0.22	-1.16	0.519
-0.56	-1.47	0.169	0.19	1.14	0.6351	-0.38	-1.3	0.3367
1.09	2.13	0.0025	0.54	1.46	0.1858	0.69	1.61	0.0628
-0.08	-1.05	0.8271	0.05	1.03	0.8992	-0.05	-1.04	0.881
0.62	1.54	0.0834	-0.06	-1.04	0.8832	0.19	1.14	0.6055
0.09	1.06	0.8059	0.65	1.57	0.0774	0.33	1.26	0.3432
0.5	1.42	0.1493	-0.35	-1.27	0.4128	0.24	1.18	0.5013
-1.03	-2.04	0.0129	-0.3	-1.23	0.4597	0.03	1.02	0.9374
0.32	1.25	0.3532	0.53	1.44	0.1395	-0.16	-1.12	0.6523
0.19	1.14	0.5662	0.2	1.15	0.5843	0.43	1.35	0.1748
0.55	1.46	0.1426	0.66	1.58	0.093	0.44	1.36	0.2291
-0.01	-1.01	0.983	-0.59	-1.5	0.2052	0.1	1.07	0.7996
-0.53	-1.45	0.1337	0.03	1.02	0.9431	-0.32	-1.25	0.3315
0.6	1.51	0.0908	0.2	1.15	0.6175	0.2	1.15	0.5832
1.69	3.22	0.0015	-0.22	-1.17	0.7137	1.16	2.23	0.0309
-0.15	-1.11	0.6872	-0.16	-1.12	0.6779	-0.09	-1.07	0.7977
-1.5	-2.82	7.00E-04	0.1	1.07	0.7998	-0.14	-1.1	0.6855
0.49	1.4	0.246	0.1	1.07	0.83	0.3	1.23	0.4705
0.02	1.01	0.9536	0.21	1.16	0.5617	-0.22	-1.16	0.5264
0.15	1.11	0.6713	0.22	1.17	0.5482	0.57	1.48	0.0788
0.93	1.91	0.016	0.09	1.07	0.8356	0.36	1.28	0.377
-0.24	-1.18	0.5252	-0.14	-1.1	0.7211	-0.14	-1.1	0.7001
-0.78	-1.71	0.057	-0.52	-1.44	0.2202	-0.37	-1.29	0.3339
0.18	1.13	0.6162	-0.33	-1.26	0.4138	0.01	1	0.9855
-0.1	-1.07	0.8103	-0.25	-1.19	0.5772	-0.24	-1.18	0.5553
-0.4	-1.32	0.4127	-0.11	-1.08	0.8324	-0.15	-1.11	0.7529
0.31	1.24	0.536	0.2	1.15	0.7126	0.27	1.21	0.5821
0.14	1.1	0.6991	0.43	1.35	0.2576	-0.04	-1.03	0.9186
-0.16	-1.12	0.6657	-0.42	-1.34	0.3173	0.27	1.2	0.4468
0.71	1.64	0.0496	0.39	1.31	0.3274	-0.15	-1.11	0.7041
-0.28	-1.22	0.4558	-0.35	-1.27	0.4034	-0.35	-1.27	0.3546

0.41	1.33	0.2806	0.28	1.21	0.4989	0.15	1.11	0.7047
-0.36	-1.28	0.2881	-0.11	-1.08	0.7513	-0.44	-1.36	0.1907
-0.04	-1.03	0.918	-0.26	-1.2	0.5466	-0.11	-1.08	0.7801
-0.56	-1.47	0.1498	-0.13	-1.09	0.7416	-0.4	-1.32	0.2814
-0.42	-1.34	0.3043	0.07	1.05	0.8661	-0.12	-1.09	0.7577
-0.81	-1.76	0.0545	0.29	1.23	0.4531	-0.05	-1.03	0.9016
1.15	2.22	0.0019	-0.13	-1.1	0.7731	0.53	1.44	0.1781
-0.75	-1.68	0.0612	-0.52	-1.43	0.2162	-0.24	-1.18	0.5123
-0.89	-1.85	0.0234	-0.19	-1.14	0.6189	-0.07	-1.05	0.8302
1.31	2.48	0.0042	0.41	1.33	0.4265	0.97	1.95	0.0367
0.6	1.52	0.1121	0.01	1.01	0.9832	0.19	1.14	0.6226
0.1	1.07	0.8215	-0.42	-1.34	0.403	0.1	1.07	0.8286
-0.15	-1.11	0.8115	0.03	1.02	0.9654	0.17	1.13	0.7826
-0.86	-1.82	0.0305	-0.22	-1.16	0.5782	-0.38	-1.3	0.2937
1.78	3.43	0	0.16	1.12	0.7049	1.26	2.4	6.00E-04
-0.48	-1.39	0.2168	0.28	1.22	0.4489	-0.16	-1.12	0.6499
-0.23	-1.17	0.5796	0.19	1.14	0.6389	0.2	1.15	0.6043
2.07	4.21	0	0.73	1.66	0.2066	1.13	2.19	0.0339
-0.06	-1.04	0.8731	0	-1	0.9954	0.25	1.19	0.4968
0.93	1.9	0.0239	0.31	1.24	0.5058	0.72	1.65	0.0802
-1.83	-3.55	0	-0.31	-1.24	0.418	-1	-2.01	0.0082
-0.94	-1.91	0.0222	-0.36	-1.28	0.3809	-0.6	-1.52	0.1142
0.49	1.41	0.1943	0.39	1.31	0.3486	0.35	1.27	0.3573
-0.62	-1.54	0.1486	0	-1	0.9995	0.07	1.05	0.8533
1.11	2.15	0.0025	-0.29	-1.22	0.5358	0.42	1.33	0.284
2.54	5.81	0	-0.9	-1.86	0.147	0.65	1.57	0.1868
-0.17	-1.12	0.6648	-0.28	-1.21	0.5103	0	1	0.9929
0.24	1.18	0.5522	0.69	1.62	0.0902	0.26	1.2	0.5108
0.3	1.23	0.4668	-0.25	-1.19	0.6007	0.2	1.15	0.6272
-0.42	-1.34	0.2659	0.01	1	0.9869	-0.32	-1.24	0.3889
0.25	1.19	0.4513	0.25	1.19	0.4781	0.33	1.26	0.2915
-0.2	-1.15	0.5914	-0.2	-1.15	0.6166	-0.19	-1.14	0.6165
-1.17	-2.26	0.0076	0.21	1.15	0.5951	-0.23	-1.17	0.5455
1.58	3	1.00E-04	0.08	1.06	0.8703	1	2	0.0209
-0.12	-1.08	0.7657	-0.66	-1.58	0.149	-0.19	-1.14	0.6297
-1.45	-2.73	0.0013	-0.35	-1.27	0.4067	-0.44	-1.36	0.2496
0.5	1.42	0.1799	-0.3	-1.23	0.505	0.12	1.09	0.7466
0.89	1.86	0.0203	-0.26	-1.19	0.5889	0.14	1.1	0.7292
0.3	1.23	0.4284	-0.58	-1.49	0.199	-0.11	-1.08	0.7836
-0.39	-1.31	0.4071	0.38	1.3	0.4239	-0.37	-1.29	0.426
-0.72	-1.65	0.1285	-0.54	-1.46	0.2751	-0.19	-1.14	0.6695
1.7	3.26	4.00E-04	0.4	1.32	0.4724	0.91	1.88	0.0689
0.77	1.7	0.064	0.78	1.72	0.0743	0.8	1.74	0.0482
-0.91	-1.88	0.041	0.06	1.04	0.8866	-0.34	-1.27	0.4006
-0.06	-1.04	0.8863	-0.33	-1.26	0.4732	-0.46	-1.38	0.281
1.53	2.88	9.00E-04	0.63	1.55	0.221	0.6	1.52	0.2128
-1.24	-2.36	0.0033	-0.65	-1.57	0.1209	-0.83	-1.77	0.0332

-0.49	-1.4	0.2446	-0.19	-1.14	0.6679	-0.17	-1.12	0.6748
0.56	1.47	0.095	0.64	1.56	0.0712	0.41	1.33	0.2157
0.71	1.64	0.0879	0.52	1.44	0.2466	0.51	1.43	0.2177
0.75	1.68	0.0583	-0.28	-1.21	0.5578	0.49	1.4	0.2228
0.55	1.47	0.1828	-0.43	-1.35	0.387	0.23	1.18	0.5792
-0.04	-1.03	0.9238	0.13	1.09	0.7867	-0.05	-1.03	0.9138
0.29	1.22	0.4843	0.77	1.7	0.0636	0.24	1.18	0.5476
-0.29	-1.22	0.4799	-0.28	-1.21	0.5193	0.02	1.01	0.9683
-1.07	-2.1	0.0128	-0.24	-1.18	0.5605	-0.74	-1.66	0.0665
0.19	1.14	0.6819	-0.19	-1.14	0.7084	-0.46	-1.38	0.341
-0.78	-1.72	0.0929	-0.07	-1.05	0.8852	-0.59	-1.51	0.1799
-0.94	-1.91	0.0271	-0.56	-1.47	0.1946	-0.44	-1.35	0.2562
-0.48	-1.39	0.2541	-0.04	-1.03	0.9324	0.06	1.04	0.878
0.12	1.09	0.7496	-0.54	-1.46	0.2222	-0.42	-1.34	0.2883
0.45	1.37	0.2508	0.22	1.16	0.6128	0.25	1.19	0.528
-0.48	-1.4	0.2406	-0.25	-1.19	0.5558	-0.14	-1.1	0.7153
1.02	2.03	0.0127	0.53	1.44	0.2553	0.82	1.77	0.0463
1.17	2.25	0.0043	0.21	1.16	0.6601	0.78	1.72	0.0621
-0.76	-1.69	0.0776	0.07	1.05	0.8726	-0.28	-1.22	0.4692
1.26	2.39	0.0022	0	-1	0.9972	0.6	1.52	0.1624
-0.73	-1.66	0.0272	-0.34	-1.26	0.3154	-0.19	-1.14	0.5116
0.08	1.06	0.8487	0.24	1.18	0.5854	0.19	1.14	0.644
0.43	1.35	0.3084	0.43	1.35	0.3464	0.91	1.88	0.0229
-1.19	-2.28	0.0187	-0.17	-1.12	0.7293	0.2	1.15	0.6393
0.55	1.46	0.1941	-0.12	-1.08	0.8108	0.21	1.16	0.6225
-0.4	-1.32	0.3183	-0.15	-1.11	0.7168	-0.33	-1.26	0.4008
-0.11	-1.08	0.7897	-0.28	-1.21	0.5396	0.08	1.06	0.8432
0.23	1.17	0.5891	-0.29	-1.22	0.5555	0.55	1.47	0.1744
0.24	1.18	0.5429	0.27	1.21	0.5202	0.04	1.03	0.911
-0.38	-1.3	0.3848	0.06	1.05	0.8843	-0.29	-1.22	0.5011
0.09	1.07	0.8484	-1.4	-2.64	0.0167	-0.42	-1.34	0.4027
2.82	7.06	0	1.52	2.88	0.0028	1.38	2.61	0.0041
-2.08	-4.24	0	-0.18	-1.13	0.6868	-1.47	-2.76	0.0015
-1.27	-2.4	0.0084	-0.02	-1.01	0.9677	-0.53	-1.45	0.2069
0.61	1.53	0.148	0.41	1.32	0.3804	0.32	1.25	0.4529
1.8	3.48	3.00E-04	0.54	1.46	0.3445	1.15	2.21	0.0267
0.24	1.18	0.5561	-0.47	-1.38	0.3228	-0.1	-1.07	0.8026
0.14	1.11	0.753	-0.8	-1.74	0.1365	-0.18	-1.13	0.6993
-0.34	-1.27	0.4194	0.04	1.02	0.935	-0.11	-1.08	0.7886
-0.24	-1.18	0.5773	0.07	1.05	0.8813	0.04	1.03	0.9295
0.58	1.49	0.1663	-0.43	-1.35	0.3957	0.84	1.79	0.0338
0.91	1.88	0.0302	-0.63	-1.54	0.2415	0.52	1.44	0.2245
0.22	1.17	0.5899	-0.32	-1.24	0.5042	0.24	1.18	0.558
-1.88	-3.67	2.00E-04	0.25	1.19	0.5425	-0.16	-1.12	0.677
-0.23	-1.17	0.6085	0.21	1.16	0.6348	-0.13	-1.1	0.7547
-0.38	-1.3	0.1497	-0.08	-1.06	0.7686	-0.25	-1.19	0.3396
1.29	2.45	0.0021	0.43	1.35	0.3781	0.08	1.06	0.8633

2.31	4.97	0	1.03	2.05	0.052	1.33	2.52	0.006
-1.77	-3.41	7.00E-04	0.54	1.45	0.1989	-0.04	-1.03	0.9184
-0.76	-1.69	0.1187	0.09	1.06	0.8556	0.02	1.01	0.962
0.71	1.64	0.0873	0.09	1.07	0.8449	0.55	1.46	0.1909
1.75	3.37	0	0.55	1.46	0.2798	0.35	1.27	0.4649
-0.67	-1.59	0.1304	-0.53	-1.44	0.255	-0.54	-1.45	0.2025
0.09	1.06	0.8441	0.41	1.33	0.3815	0.43	1.35	0.3172
-1.06	-2.09	0.0345	-0.06	-1.04	0.9016	-0.34	-1.26	0.4627
1.2	2.3	0.007	-0.03	-1.02	0.9525	0.52	1.43	0.2659
0.19	1.14	0.658	-0.81	-1.75	0.1245	0.24	1.18	0.578
0.62	1.53	0.1607	-0.8	-1.74	0.1457	0.07	1.05	0.8863
0.61	1.52	0.2508	0.06	1.04	0.9238	0.56	1.48	0.2854
0.2	1.15	0.6527	-0.18	-1.14	0.7137	-0.24	-1.18	0.6066
-0.73	-1.66	0.1171	0.09	1.06	0.8451	0.07	1.05	0.8707
-0.4	-1.32	0.4523	0.68	1.6	0.1992	-0.09	-1.06	0.8613
2.06	4.16	2.00E-04	0.64	1.56	0.3075	1.07	2.11	0.0646
1.35	2.55	0.0011	0.05	1.04	0.9141	0.86	1.82	0.0442
-0.03	-1.02	0.9444	0.03	1.02	0.9406	0.11	1.08	0.7952
-0.03	-1.02	0.9396	-0.18	-1.13	0.7048	-0.03	-1.02	0.9385
-0.55	-1.46	0.2385	-0.16	-1.12	0.7348	-0.21	-1.15	0.6326
-0.43	-1.35	0.3587	-0.32	-1.25	0.519	-0.65	-1.57	0.169
1.29	2.45	0.013	-0.26	-1.2	0.662	0.38	1.3	0.4861
0.54	1.46	0.2252	0.1	1.07	0.8354	0.37	1.29	0.4075
0.48	1.39	0.28	-0.12	-1.09	0.8059	-0.09	-1.07	0.8379
0.27	1.21	0.5575	-0.08	-1.05	0.8816	0.13	1.09	0.7811
-0.71	-1.64	0.1155	-0.01	-1.01	0.975	-0.41	-1.32	0.3388
-0.55	-1.47	0.2571	0.09	1.06	0.8585	0.55	1.47	0.1951
0.86	1.81	0.0422	-0.02	-1.01	0.9756	0.59	1.51	0.1672
-0.24	-1.18	0.5971	-0.39	-1.31	0.423	-0.82	-1.76	0.0819
3.87	14.61	0	2.19	4.56	0.0014	3.46	10.99	0
0.45	1.37	0.2754	-0.49	-1.4	0.3261	-0.14	-1.1	0.7458
-0.34	-1.26	0.4388	0.03	1.02	0.9449	-0.21	-1.16	0.6126
0.47	1.38	0.3037	0.16	1.12	0.7439	0.39	1.31	0.3856
0.1	1.07	0.8221	-0.58	-1.49	0.2594	-0.21	-1.16	0.6409
0.68	1.61	0.1538	-0.2	-1.15	0.7231	0.52	1.44	0.2739
-1.65	-3.14	0.0014	-0.15	-1.11	0.7381	-1.14	-2.2	0.0163
0	1	0.9942	0.45	1.37	0.3488	0.12	1.09	0.7888
0.49	1.4	0.2295	0.17	1.12	0.7112	-0.14	-1.1	0.7506
-0.02	-1.01	0.9697	-0.09	-1.07	0.8451	0.03	1.02	0.9377
-0.25	-1.19	0.5743	-0.58	-1.49	0.2485	-0.31	-1.24	0.4849
-1.86	-3.64	5.00E-04	-0.1	-1.07	0.8207	-0.7	-1.62	0.1181
-0.06	-1.04	0.9252	-0.12	-1.08	0.854	-0.28	-1.21	0.6404
0.15	1.11	0.7614	-0.81	-1.75	0.1626	-1.19	-2.28	0.0342
-0.64	-1.56	0.1562	-0.54	-1.45	0.2601	-0.46	-1.38	0.2815
-0.1	-1.07	0.8299	0.29	1.22	0.5325	0.11	1.08	0.7899
-0.65	-1.57	0.169	-0.98	-1.97	0.0609	-0.19	-1.14	0.6581
-0.41	-1.33	0.4993	1.05	2.07	0.0829	0.08	1.06	0.8875

1.52	2.87	0.003	-0.81	-1.75	0.1999	1.2	2.3	0.0198
0.67	1.59	0.1661	0.35	1.27	0.5108	0.1	1.08	0.8349
-0.11	-1.08	0.8102	0.02	1.01	0.9666	0.43	1.35	0.2986
1.49	2.8	0.0015	0.49	1.4	0.3646	0.81	1.75	0.0977
-0.14	-1.1	0.7587	0.29	1.22	0.5303	-0.22	-1.16	0.6331
-0.69	-1.62	0.1419	-0.72	-1.64	0.1568	-0.2	-1.15	0.6388
0.02	1.01	0.9649	-0.22	-1.17	0.6416	-0.69	-1.61	0.1375
0.87	1.82	0.0879	0.3	1.23	0.5908	0.39	1.31	0.4537
0.98	1.97	0.0312	-0.77	-1.71	0.179	0.12	1.09	0.8023
0.37	1.29	0.4883	0.72	1.65	0.1825	0.19	1.14	0.7166
1.11	2.16	0.0126	-0.1	-1.08	0.8466	1	2	0.0239
-0.43	-1.35	0.3372	-0.3	-1.23	0.5266	-0.03	-1.02	0.9332
-0.09	-1.07	0.835	-0.63	-1.55	0.2118	0.14	1.1	0.7404
0.29	1.22	0.5763	0.48	1.39	0.3655	0.42	1.34	0.3997
-0.19	-1.14	0.6701	-0.34	-1.26	0.4746	-0.46	-1.37	0.2978
0.23	1.18	0.6347	-0.86	-1.81	0.1327	-0.2	-1.15	0.6964
-0.31	-1.24	0.4997	-0.19	-1.14	0.6851	0	-1	0.9928
-0.36	-1.29	0.4352	0.41	1.33	0.3633	-0.26	-1.2	0.5613
1.28	2.43	0.0095	0.33	1.25	0.5626	0.53	1.44	0.3111
-1.7	-3.26	0.0042	-0.3	-1.23	0.5972	0.12	1.08	0.8207
0.46	1.38	0.3095	-0.33	-1.26	0.5347	-0.21	-1.16	0.6661
-0.62	-1.54	0.2143	-0.55	-1.47	0.2973	0.03	1.02	0.9398
-0.12	-1.08	0.8017	-0.66	-1.58	0.2134	-0.35	-1.27	0.4617
-0.25	-1.19	0.5759	-0.04	-1.03	0.9389	-0.25	-1.19	0.5663
2.13	4.39	0	0.14	1.11	0.8066	0.53	1.44	0.3247
-1.74	-3.34	9.00E-04	-0.21	-1.16	0.6484	-0.98	-1.97	0.0336
-1.69	-3.22	0.0042	0.14	1.1	0.7965	-0.68	-1.6	0.2071
-0.95	-1.93	0.0607	-0.36	-1.28	0.4796	0.01	1	0.9888
-1.5	-2.83	0.0102	-0.19	-1.14	0.7306	-0.58	-1.49	0.286
-0.45	-1.37	0.3425	0.04	1.03	0.9263	0.04	1.03	0.9224
-1.46	-2.75	0.0077	0.26	1.2	0.5837	-0.6	-1.51	0.2123
-0.63	-1.54	0.2007	0.04	1.03	0.9403	-0.47	-1.38	0.3173
1.27	2.42	0.0089	0.69	1.62	0.2001	0.81	1.75	0.1054
0.12	1.09	0.7801	-0.09	-1.06	0.856	-0.45	-1.36	0.3412
0.53	1.44	0.2643	0.4	1.32	0.4285	-0.02	-1.02	0.961
0.99	1.99	0.0331	-0.29	-1.22	0.6083	0.27	1.21	0.5775
0.19	1.14	0.6849	-0.8	-1.74	0.1493	-0.25	-1.19	0.6095
-1.48	-2.79	0.0053	-0.02	-1.02	0.963	-0.46	-1.37	0.3144
-0.04	-1.03	0.9553	-0.11	-1.08	0.8709	0.04	1.03	0.9594
-0.9	-1.86	0.0707	-0.4	-1.32	0.4198	-0.22	-1.16	0.6227
1.64	3.11	0.0018	-0.42	-1.34	0.5062	0.82	1.76	0.1361
-0.54	-1.45	0.2641	0.35	1.28	0.4532	-0.62	-1.54	0.193
0.46	1.38	0.3136	-0.03	-1.02	0.9476	0.3	1.23	0.5104
0.51	1.43	0.3006	0.44	1.36	0.4055	0.28	1.22	0.572
0.56	1.48	0.2646	-0.47	-1.38	0.4284	-0.16	-1.12	0.7615
2.95	7.74	0	0.93	1.91	0.1846	1.5	2.82	0.0251
-0.49	-1.41	0.3458	-0.46	-1.38	0.4025	-0.17	-1.12	0.7398

-0.04	-1.03	0.9296	-0.33	-1.25	0.5407	-0.37	-1.29	0.4633
-3.58	-12	0	-0.67	-1.59	0.2111	-1.71	-3.27	0.0017
-1.01	-2.01	0.0569	-2	-4	0.001	-0.76	-1.69	0.1381
-0.12	-1.08	0.8077	0.27	1.21	0.5773	-0.08	-1.06	0.8623
-0.45	-1.37	0.3761	-0.28	-1.22	0.5934	0.11	1.08	0.807
0.59	1.51	0.1955	-0.14	-1.1	0.7878	-0.23	-1.17	0.638
1.13	2.2	0.02	0.19	1.14	0.7345	0.38	1.3	0.4553
0.91	1.88	0.0563	0.19	1.14	0.7273	0.54	1.45	0.2692
-0.74	-1.67	0.1644	0.34	1.26	0.5077	-0.54	-1.45	0.2917
0.65	1.57	0.2007	0.13	1.09	0.8153	0.15	1.11	0.7803
-1.43	-2.7	0.0088	-0.16	-1.12	0.7502	-0.58	-1.5	0.231
-0.13	-1.09	0.8043	-0.48	-1.39	0.406	0.06	1.04	0.912
0.17	1.13	0.73	-0.49	-1.41	0.376	0.01	1.01	0.9789
-2.14	-4.4	2.00E-04	-0.32	-1.24	0.5164	-1.37	-2.58	0.0067
3.13	8.77	0	0.32	1.24	0.6601	1.39	2.62	0.0394
-0.36	-1.28	0.4711	-0.19	-1.14	0.7114	0.02	1.01	0.9635
-0.18	-1.13	0.5548	-0.15	-1.11	0.6491	-0.59	-1.51	0.0582
0.63	1.55	0.1712	-0.41	-1.33	0.4499	-0.49	-1.41	0.3364
3	8.01	0	0.79	1.73	0.2386	1.99	3.96	9.00E-04
-0.79	-1.73	0.1274	-0.15	-1.11	0.7659	0.07	1.05	0.875
-0.57	-1.49	0.2558	-0.32	-1.24	0.5431	-0.22	-1.17	0.6425
0.15	1.11	0.7637	0.25	1.19	0.6266	0.18	1.13	0.7058
-1.13	-2.19	0.0114	0.14	1.1	0.7279	-0.61	-1.53	0.1295
-0.33	-1.26	0.5138	-0.26	-1.2	0.6253	0.12	1.09	0.7988
1.05	2.08	0.0373	0.14	1.1	0.8043	0.38	1.3	0.4688
2.82	7.04	0	0.31	1.24	0.6577	2.77	6.83	0
1.1	2.14	0.1316	0.42	1.34	0.5634	1.43	2.69	0.0493
-1.27	-2.42	0.0244	-0.68	-1.6	0.2224	-0.37	-1.29	0.4605
0.07	1.05	0.9007	0.05	1.04	0.9239	-1.02	-2.03	0.0789
1.95	3.86	2.00E-04	-0.06	-1.05	0.9187	1.07	2.1	0.0487
-0.61	-1.53	0.2703	-0.34	-1.27	0.5453	-0.46	-1.38	0.3923
0.07	1.05	0.8888	-0.28	-1.21	0.6311	0.22	1.16	0.6692
-0.36	-1.28	0.5374	-0.64	-1.55	0.3064	0.29	1.22	0.6037
1.11	2.16	0.0556	1.28	2.43	0.0328	0.47	1.38	0.4351
0	1	0.999	0.18	1.13	0.7266	0.27	1.21	0.5703
-0.32	-1.25	0.5317	0.25	1.19	0.6211	-0.26	-1.2	0.6042
0.44	1.36	0.4061	0.74	1.67	0.1747	0.33	1.25	0.5408
-1.46	-2.75	0.0223	0.08	1.05	0.9009	-0.36	-1.28	0.5462
-0.59	-1.51	0.2554	-0.01	-1.01	0.9827	-0.73	-1.66	0.1628
-1.11	-2.16	0.0404	-0.45	-1.37	0.3964	-0.13	-1.09	0.787
-0.68	-1.6	0.2475	-0.16	-1.12	0.7858	0.21	1.16	0.6941
-0.58	-1.5	0.3692	0.1	1.07	0.8792	-0.19	-1.14	0.7636
-0.28	-1.21	0.5769	0.2	1.15	0.6864	-0.13	-1.09	0.7926
-0.99	-1.98	0.0735	-0.28	-1.21	0.6008	-0.01	-1	0.9885
-1.55	-2.92	0.0019	0.15	1.11	0.7201	-0.63	-1.55	0.1398
-0.79	-1.73	0.143	-0.05	-1.03	0.9265	-0.35	-1.27	0.4901
0.89	1.86	0.068	-0.66	-1.58	0.2687	-0.06	-1.04	0.9052

1.41	2.65	0.005	-0.14	-1.1	0.8183	0.64	1.56	0.2232
0.16	1.12	0.7435	-0.07	-1.05	0.893	0.05	1.04	0.9157
0.62	1.54	0.2116	-0.46	-1.38	0.4278	-0.06	-1.04	0.9095
1.21	2.32	0.0197	0.77	1.71	0.1715	0.09	1.06	0.8741
-0.82	-1.77	0.129	0.08	1.06	0.8789	-0.08	-1.06	0.8719
-1.08	-2.12	0.0623	-0.31	-1.24	0.5843	-0.31	-1.24	0.5654
-0.14	-1.1	0.7323	-0.97	-1.96	0.0469	0.2	1.15	0.6162
-0.5	-1.42	0.3835	-0.6	-1.52	0.3227	0.09	1.06	0.8747
-0.38	-1.31	0.4687	0.31	1.24	0.5482	-0.09	-1.07	0.854
0.15	1.11	0.474	0.25	1.19	0.2748	0.02	1.01	0.9301
1.02	2.02	0.0608	-0.47	-1.38	0.4576	0.48	1.39	0.3891
-0.44	-1.35	0.389	-0.63	-1.55	0.2488	-0.58	-1.5	0.2483
-0.58	-1.5	0.2626	-0.01	-1.01	0.9868	-0.95	-1.93	0.0749
1.39	2.62	0.0091	-0.54	-1.46	0.3976	0.76	1.7	0.1655
0.83	1.77	0.1632	0.38	1.3	0.5529	0.74	1.67	0.2097
2.55	5.84	0	0.27	1.2	0.6912	1.54	2.91	0.0091
-0.55	-1.47	0.4545	0.17	1.12	0.8192	0.22	1.17	0.7628
0.11	1.08	0.84	0.26	1.2	0.6347	0.43	1.35	0.3885
1.01	2.01	0.1122	0.27	1.2	0.6922	0.46	1.37	0.4774
0.42	1.34	0.3613	-0.4	-1.32	0.4617	0.04	1.03	0.9241
1.48	2.78	0.0045	0.53	1.45	0.3658	0.81	1.75	0.1348
1.67	3.18	9.00E-04	0.06	1.04	0.9238	0.66	1.58	0.2182
-0.28	-1.22	0.5939	-0.23	-1.17	0.6831	-0.56	-1.47	0.2984
-0.44	-1.35	0.4557	-0.32	-1.25	0.597	0.08	1.05	0.8904
0.6	1.51	0.2773	0.29	1.22	0.6287	0.47	1.39	0.3858
-0.26	-1.2	0.6214	0.02	1.02	0.9638	-0.39	-1.31	0.4541
-0.02	-1.02	0.9666	-0.47	-1.38	0.4094	-0.85	-1.8	0.1235
1.36	2.56	0.0096	0.66	1.58	0.2562	0.63	1.55	0.2533
-1.42	-2.68	0.008	-0.65	-1.56	0.2126	-1.05	-2.07	0.0362
0.18	1.13	0.7275	-0.4	-1.32	0.4927	0.35	1.28	0.4873
1.49	2.81	0.009	-0.19	-1.14	0.775	1.35	2.55	0.0179
0.6	1.51	0.2748	0.77	1.7	0.1744	0.74	1.67	0.1664
1.45	2.74	0.0088	0.63	1.55	0.3054	1.63	3.09	0.0026
-0.77	-1.7	0.1749	-0.12	-1.09	0.8315	-0.14	-1.11	0.7837
-0.03	-1.02	0.9513	0.13	1.09	0.8133	-0.5	-1.41	0.3652
0.63	1.55	0.246	0.14	1.1	0.8195	0.55	1.47	0.3034
-0.03	-1.02	0.9583	0.33	1.26	0.53	0.09	1.07	0.8554
1.47	2.77	0.0084	0.61	1.53	0.3202	0.7	1.63	0.2244
-0.15	-1.11	0.767	-0.36	-1.28	0.5127	-0.38	-1.3	0.4578
0.77	1.71	0.1152	-0.54	-1.45	0.3594	-0.23	-1.17	0.6703
-0.38	-1.3	0.5021	-0.25	-1.19	0.6668	-0.21	-1.16	0.6983
-0.35	-1.27	0.5216	-0.22	-1.16	0.697	0.16	1.12	0.7472
-1.56	-2.95	0.0094	-0.57	-1.48	0.3215	0	-1	0.9968
1.72	3.3	0.0015	-0.32	-1.25	0.6266	0.76	1.7	0.1844
1.8	3.47	0.0011	-0.04	-1.03	0.952	0.58	1.5	0.3256
1.23	2.35	0.0391	0.43	1.35	0.5103	1.23	2.35	0.0377
2.13	4.39	2.00E-04	0.9	1.87	0.1638	1.43	2.69	0.0174

2.75	6.72	0	0.89	1.86	0.1797	1.35	2.56	0.0288
-0.02	-1.01	0.9713	0.38	1.3	0.464	-0.07	-1.05	0.8955
0.09	1.06	0.8688	-0.4	-1.32	0.4818	-0.18	-1.13	0.7339
0.79	1.73	0.0664	0.85	1.8	0.0641	0.46	1.37	0.2961
0.5	1.41	0.0551	0.17	1.12	0.5737	0.1	1.07	0.7223
0.97	1.95	0.0576	-0.17	-1.13	0.7714	0.12	1.09	0.825
-0.76	-1.69	0.169	-0.5	-1.42	0.3745	-0.35	-1.27	0.5025
-0.51	-1.43	0.3772	0.02	1.01	0.9728	-0.41	-1.33	0.47
0.84	1.79	0.1289	-0.95	-1.93	0.1496	0.4	1.32	0.4788
2.96	7.76	0	0.53	1.44	0.4441	1.37	2.58	0.0317
2.54	5.81	1.00E-04	-0.02	-1.02	0.9735	1.39	2.61	0.0395
1.34	2.54	0.0111	-0.01	-1.01	0.9887	0.5	1.41	0.3745
0.15	1.11	0.7785	-0.23	-1.17	0.6917	-0.31	-1.24	0.5727
-0.23	-1.17	0.6808	-0.62	-1.54	0.3068	0.26	1.2	0.6224
-1.09	-2.13	0.0632	0.29	1.23	0.5888	-0.52	-1.43	0.3437
-1.01	-2.02	0.104	-0.46	-1.38	0.4585	0.2	1.15	0.7278
0.6	1.51	0.4151	0.21	1.15	0.7787	0.17	1.12	0.8208
2.25	4.75	1.00E-04	1.48	2.8	0.0184	1.24	2.36	0.0421
0.47	1.39	0.4524	0.32	1.25	0.6213	0.39	1.31	0.5362
0.55	1.47	0.3146	-0.54	-1.46	0.3844	0.35	1.27	0.5248
-1.58	-2.98	0.0095	0.05	1.04	0.9232	-0.8	-1.74	0.1494
0.27	1.21	0.6264	0.65	1.57	0.253	-0.47	-1.39	0.4218
-1.33	-2.51	0.0255	-0.41	-1.33	0.4752	-0.68	-1.61	0.2119
-0.61	-1.53	0.3291	-0.93	-1.9	0.1577	0.43	1.35	0.46
0.7	1.63	0.2774	-0.38	-1.3	0.5774	0.2	1.15	0.7648
0.41	1.32	0.3077	0.22	1.16	0.6176	0.26	1.19	0.5174
-0.18	-1.14	0.7479	0.33	1.26	0.5604	0.29	1.23	0.5835
-0.68	-1.6	0.2364	-0.76	-1.69	0.2071	-0.01	-1.01	0.9882
0.69	1.61	0.1562	-0.69	-1.61	0.2294	0.41	1.33	0.4022
1.63	3.1	0.0129	-0.23	-1.17	0.7434	0.53	1.45	0.4323
2.6	6.04	4.00E-04	2.3	4.93	0.0019	1.63	3.1	0.027
-1.45	-2.74	0.0174	0.25	1.19	0.6589	-1.06	-2.09	0.0693
-2.52	-5.74	2.00E-04	-0.74	-1.67	0.2561	-0.57	-1.48	0.3602
2.08	4.23	3.00E-04	-0.02	-1.01	0.9758	1	2	0.0984
-0.37	-1.3	0.5783	0.52	1.44	0.4384	-0.07	-1.05	0.9125
-0.98	-1.97	0.0925	-0.42	-1.34	0.4631	-0.8	-1.74	0.1537
-0.67	-1.59	0.248	0.16	1.12	0.7737	0.06	1.05	0.9046
-1.6	-3.02	0.0102	-0.31	-1.24	0.5929	-0.83	-1.78	0.1458
3.06	8.34	0	2.11	4.33	0.0027	1.78	3.42	0.0104
-2.26	-4.77	8.00E-04	-0.9	-1.86	0.1714	-1.19	-2.29	0.0614
1	2	0.079	-0.57	-1.49	0.3815	-0.55	-1.46	0.3816
0.81	1.76	0.2063	0.13	1.09	0.8507	0.93	1.91	0.1432
2.22	4.65	3.00E-04	0.11	1.08	0.8748	1.06	2.09	0.0953
-0.11	-1.08	0.8378	-0.25	-1.19	0.6674	-0.26	-1.2	0.6339
-0.94	-1.92	0.1151	-0.11	-1.08	0.8549	-0.28	-1.22	0.6076
0.8	1.74	0.195	0.24	1.18	0.711	0.58	1.49	0.3512
-0.9	-1.86	0.1402	-0.31	-1.24	0.6096	-0.25	-1.19	0.6618

-0.34	-1.26	0.6033	-1.05	-2.07	0.1222	-0.68	-1.6	0.2952
-1.8	-3.47	0.0054	-0.41	-1.33	0.5003	-0.16	-1.12	0.7796
2.88	7.38	0	0.41	1.33	0.5676	1.11	2.15	0.1119
-2.47	-5.52	2.00E-04	-0.77	-1.7	0.2063	-1.02	-2.03	0.0809
-0.28	-1.21	0.6504	0.7	1.62	0.2492	0.24	1.18	0.6865
-1.89	-3.7	0.0068	-1.11	-2.16	0.1119	-0.83	-1.78	0.2195
1.41	2.67	0.0348	0.47	1.39	0.5027	2.17	4.49	9.00E-04
1.1	2.15	0.0525	0.06	1.04	0.9237	0.81	1.75	0.1595
2.01	4.02	0.0012	0.68	1.6	0.3163	0.86	1.81	0.186
2.87	7.31	0	1.29	2.44	0.0709	1.15	2.22	0.0986
3.08	8.46	0	1.49	2.82	0.0455	1.19	2.28	0.1101
-1.52	-2.86	0.0201	0.06	1.04	0.9285	-0.06	-1.05	0.9131
2.46	5.52	5.00E-04	0.42	1.33	0.5726	1.11	2.16	0.1256
-1.43	-2.69	0.0184	-0.66	-1.58	0.2656	-1.35	-2.55	0.0223
1.45	2.74	0.0108	-0.27	-1.2	0.6874	1.13	2.2	0.0492
-1.23	-2.35	0.0519	0.69	1.62	0.2317	-0.47	-1.38	0.4313
0.79	1.73	0.1938	-0.7	-1.62	0.3057	0.36	1.28	0.5621
0.89	1.86	0.1281	-1.01	-2.02	0.1333	0.04	1.03	0.9498
0.84	1.79	0.2305	-1.03	-2.04	0.1536	0.58	1.5	0.4093
2.43	5.39	0	0.1	1.07	0.8838	0.16	1.12	0.8023
1.53	2.9	0.0148	1.19	2.28	0.0721	1.33	2.51	0.0356
0.51	1.42	0.4372	0.31	1.24	0.6508	0.39	1.31	0.554
2.68	6.41	1.00E-04	0.8	1.74	0.267	1.15	2.22	0.1002
2.01	4.03	0.0032	0.61	1.53	0.399	1.23	2.35	0.0773
-0.79	-1.73	0.221	0.28	1.21	0.6591	-0.74	-1.67	0.2434
2.77	6.84	1.00E-04	1.26	2.4	0.0868	2.02	4.07	0.0042
1.92	3.77	0.0058	-0.03	-1.02	0.9664	1.16	2.24	0.0999
1.94	3.83	0.0077	0.1	1.07	0.8964	1.15	2.22	0.1169
0.14	1.1	0.8271	0.37	1.29	0.5695	-0.3	-1.23	0.6295
0.78	1.71	0.1178	0	1	0.9999	0.79	1.73	0.1064
1.37	2.59	0.0472	-0.49	-1.41	0.4922	0.26	1.19	0.7167
0.15	1.11	0.7709	0.13	1.1	0.8156	0.41	1.33	0.4342
0.53	1.44	0.4767	-0.52	-1.43	0.4746	0.75	1.68	0.3164
-1.67	-3.18	NA	-1.54	-2.91	NA	-1.69	-3.22	NA
0.15	1.11	0.8377	-0.07	-1.05	0.9279	0.12	1.09	0.8699
0.96	1.94	0.1855	0.58	1.5	0.4246	0.92	1.89	0.2043
0.97	1.96	0.0644	-0.42	-1.34	0.4986	0.69	1.61	0.1937
0.12	1.09	0.8716	-0.45	-1.36	0.5351	0.18	1.14	0.8027
3.16	8.96	0	0.48	1.39	0.518	0.59	1.5	0.4323
0.43	1.34	0.3017	0.7	1.62	0.1063	0.36	1.28	0.3823
0.26	1.19	0.5764	0.17	1.12	0.7335	0.37	1.29	0.4059
0.93	1.91	0.0596	0.03	1.02	0.9556	0.19	1.14	0.7206
0.79	1.73	0.2667	-0.67	-1.59	0.3572	0.46	1.38	0.5163
0.98	1.98	0.187	0.59	1.5	0.4262	0.31	1.24	0.6813
0.98	1.97	0.1759	0.21	1.16	0.7637	0.52	1.43	0.4747
0.56	1.48	0.1954	0.28	1.21	0.5614	0.27	1.21	0.5371
1.71	3.26	0.0193	0.87	1.82	0.237	1.62	3.08	0.0261

0.48	1.39	0.4028	0.44	1.35	0.466	0.8	1.74	0.1513
0.93	1.91	0.1465	0.94	1.92	0.1547	0.27	1.21	0.6756
-2.7	-6.49	1.00E-04	0.24	1.18	0.713	-0.44	-1.36	0.4792
0.65	1.57	0.3487	0.13	1.09	0.8551	0.6	1.52	0.3827
0.08	1.06	0.9115	-1.04	-2.06	0.1474	0.43	1.35	0.5406
-0.22	-1.17	0.755	-0.09	-1.06	0.8988	0.14	1.1	0.8438
0.17	1.12	0.8073	0.12	1.08	0.8617	0.36	1.28	0.6072
1.57	2.97	0.0099	-0.4	-1.32	0.5533	0.88	1.84	0.1578
1.15	2.21	0.0722	0.01	1	0.9924	0.77	1.7	0.2321
-2.96	-7.81	0	-2.76	-6.79	0	0.02	1.01	0.974
1.22	2.33	NA	1.23	2.34	NA	0.87	1.83	NA
0.45	1.36	0.5055	0	-1	0.9981	0.46	1.37	0.496
1.77	3.42	0.0143	0.2	1.15	0.7809	0.58	1.49	0.428
-1.51	-2.85	NA	-1.41	-2.66	NA	-0.8	-1.74	NA
0.69	1.61	0.2672	0.35	1.27	0.5534	1.24	2.36	0.0481

S.a.GN.vs.I	S.a.GN.vs.I	S.a.GN.vs.I	VancATN.v	VancATN.v	VancATN.v	IgAN E0.vs	IgAN E0.vs	IgAN E0.vs
1.22	2.34	0	0.39	1.31	0.0692	-0.46	-1.38	0.0792
0	-1	0.9887	-0.15	-1.11	0.3023	-0.21	-1.16	0.2583
0.2	1.15	0.2802	0.39	1.31	0.0179	-0.32	-1.24	0.1376
-0.12	-1.09	0.5078	0.59	1.51	1.00E-04	-0.65	-1.57	0.0016
-0.39	-1.31	0.0368	-0.11	-1.08	0.4991	0.01	1.01	0.9484
0.43	1.34	0.0236	0.4	1.32	0.0183	-0.02	-1.01	0.9272
-0.6	-1.52	0.0197	-1.05	-2.08	0	1.33	2.51	0
1.3	2.46	0	0.88	1.85	0.0019	-0.68	-1.61	0.0457
-0.04	-1.03	0.8368	-0.14	-1.1	0.4199	-0.06	-1.05	0.761
-0.41	-1.32	0.0699	-0.57	-1.48	0.0055	0.52	1.43	0.0516
1.85	3.61	0	0.91	1.88	0.0011	-0.31	-1.24	0.3568
0.7	1.62	8.00E-04	0.42	1.34	0.0287	-0.12	-1.09	0.6154
-0.14	-1.1	0.5003	-0.3	-1.23	0.1003	0.45	1.37	0.0413
-0.79	-1.73	3.00E-04	-0.28	-1.21	0.1252	0.02	1.02	0.9279
-0.52	-1.43	0.0107	-0.28	-1.22	0.1012	0.4	1.32	0.0621
0.07	1.05	0.7831	0.63	1.55	0.0044	-0.95	-1.94	0.0011
1.41	2.66	0	0.98	1.97	3.00E-04	-0.79	-1.73	0.0143
-0.38	-1.31	0.1127	-0.43	-1.34	0.0498	0.49	1.41	0.0595
0.33	1.26	0.1131	0.41	1.33	0.0272	-0.06	-1.04	0.8112
-0.91	-1.88	2.00E-04	0.02	1.01	0.9251	0.17	1.12	0.4874
0.05	1.04	0.8254	0.1	1.07	0.6274	-0.64	-1.56	0.0135
0.43	1.35	0.048	0.01	1	0.9769	0.47	1.39	0.0661
-0.94	-1.92	1.00E-04	-0.74	-1.67	3.00E-04	1.18	2.27	0
-1.01	-2.02	5.00E-04	-0.74	-1.66	0.0036	1.21	2.32	1.00E-04
-0.99	-1.98	0.0011	-0.67	-1.59	0.0108	0.99	1.99	0.0023
-0.52	-1.44	0.0204	-0.42	-1.34	0.0321	0.35	1.27	0.1571
-0.57	-1.49	0.0191	-0.47	-1.39	0.0262	0.84	1.79	0.0014
-0.88	-1.84	3.00E-04	-0.45	-1.36	0.0226	0.34	1.27	0.1734
0.58	1.5	0.0043	0.23	1.17	0.2358	0.2	1.15	0.3928
-0.75	-1.68	0.0035	-0.23	-1.17	0.2708	0.33	1.26	0.2007
-1	-2	3.00E-04	-0.12	-1.09	0.5558	-0.26	-1.2	0.3394
-0.6	-1.52	0.0151	-0.4	-1.32	0.0598	0.35	1.28	0.1937
-0.19	-1.14	0.4166	-0.19	-1.14	0.3601	0.38	1.3	0.1658
1.07	2.1	0.0562	0.62	1.54	0.2492	-0.34	-1.26	0.5824
1.12	2.18	0	0.71	1.64	0.0018	-0.81	-1.76	0.0058
-0.73	-1.66	0.0188	-0.36	-1.28	0.1739	0.8	1.74	0.0136
0.37	1.29	0.1235	0.1	1.07	0.6538	-0.45	-1.37	0.1141
0.86	1.81	2.00E-04	0.04	1.03	0.8723	-0.02	-1.01	0.9446
0.3	1.23	0.5732	-0.69	-1.61	0.172	0.14	1.1	0.8166
-0.14	-1.11	0.5383	-0.14	-1.1	0.4921	0.04	1.03	0.8831
-0.81	-1.75	0.0025	-0.32	-1.25	0.1366	0.51	1.43	0.0575
0.07	1.05	0.7848	0.12	1.09	0.5951	0.1	1.08	0.7229
0.31	1.24	0.6144	-0.62	-1.54	0.2887	0.39	1.31	0.5579

0.79	1.73	0.0019	0.3	1.23	0.225	-0.42	-1.34	0.1749
0.87	1.83	1.00E-04	-0.13	-1.09	0.5674	-0.2	-1.15	0.4414
-1.08	-2.11	0.0015	-0.23	-1.17	0.4108	0.51	1.43	0.1378
-1.01	-2.02	0.0018	-0.37	-1.29	0.1628	1.21	2.31	3.00E-04
-0.33	-1.26	0.208	-0.14	-1.1	0.546	-0.03	-1.02	0.9196
-0.43	-1.35	0.1137	-0.46	-1.37	0.0628	0.32	1.25	0.2887
0.21	1.16	0.4173	0.34	1.26	0.1477	-0.85	-1.81	0.0049
0.09	1.07	0.72	0.43	1.34	0.0547	-0.53	-1.44	0.0675
1.37	2.58	0	0.77	1.7	0.0163	-1.39	-2.62	5.00E-04
-0.14	-1.1	0.6068	-0.37	-1.29	0.1412	0.7	1.62	0.0205
1.01	2.01	0.0137	2	3.99	0	-2.16	-4.46	0
0.39	1.31	0.2466	0.94	1.91	0.0014	-0.61	-1.52	0.098
0.52	1.43	0.051	0.36	1.29	0.1358	-0.34	-1.26	0.2625
1.73	3.32	0	1.61	3.06	0	-0.35	-1.27	0.3834
1.43	2.7	1.00E-04	1.68	3.2	0	-1.64	-3.12	0
0.94	1.92	0.0067	0.73	1.65	0.0246	-1.07	-2.09	0.0068
-0.89	-1.86	0.007	-1.15	-2.22	2.00E-04	1.09	2.13	0.0019
-0.43	-1.34	0.1452	-0.22	-1.16	0.3841	0.39	1.31	0.23
0.11	1.08	0.7108	-0.02	-1.02	0.9236	-0.11	-1.08	0.7233
-0.73	-1.66	0.0569	-0.79	-1.72	0.0245	1.16	2.24	0.0068
0.2	1.15	0.491	0.2	1.15	0.4444	-0.47	-1.39	0.1461
-0.52	-1.44	0.0827	-1.31	-2.48	0	1.47	2.77	2.00E-04
0.36	1.29	0.2225	0.16	1.12	0.5609	-0.05	-1.04	0.8795
-0.72	-1.64	0.0274	-0.04	-1.03	0.8814	0.49	1.4	0.1452
0.78	1.71	0.0065	0.82	1.76	0.0016	-0.57	-1.48	0.0777
-0.51	-1.43	0.1	-0.55	-1.47	0.0487	1.07	2.11	0.0028
-0.39	-1.31	0.1923	-0.55	-1.46	0.0452	0.54	1.46	0.0999
1.24	2.37	0.0138	1.87	3.65	1.00E-04	-2.03	-4.08	4.00E-04
-0.59	-1.5	0.069	-0.27	-1.21	0.3296	0.27	1.2	0.4437
0.3	1.23	0.3226	0.35	1.27	0.2027	-0.97	-1.96	0.006
0.36	1.29	0.2042	0.23	1.17	0.3749	-0.34	-1.27	0.3076
-0.33	-1.25	0.3373	-0.07	-1.05	0.8097	-0.06	-1.04	0.8651
0.6	1.51	0.0372	0.04	1.03	0.8821	0.03	1.02	0.9326
0.47	1.39	0.1199	0.44	1.35	0.1144	-0.6	-1.52	0.0756
0.21	1.15	0.4829	0.21	1.16	0.4293	0.41	1.33	0.2179
0.63	1.55	0.0272	0.01	1.01	0.9679	0.35	1.27	0.2889
-0.06	-1.04	0.8258	0	-1	0.997	-0.08	-1.06	0.8081
-2.78	-6.89	0	-0.95	-1.93	0.033	0.1	1.07	0.8612
-0.35	-1.28	0.2642	-0.47	-1.39	0.1002	0.91	1.88	0.0083
-2.66	-6.33	0	-0.88	-1.84	0.0137	0.72	1.65	0.0921
-0.41	-1.33	0.182	-0.34	-1.26	0.2201	-0.04	-1.03	0.9031
-0.65	-1.57	0.0443	-0.19	-1.14	0.475	-0.21	-1.16	0.5487
0.7	1.62	0.0141	0.43	1.34	0.1116	-0.42	-1.34	0.1929
1.45	2.73	0.0043	1.13	2.19	0.0188	-2.58	-5.97	0
-0.51	-1.42	0.1178	-0.31	-1.24	0.2702	0.44	1.36	0.1892
-1.48	-2.79	3.00E-04	-0.71	-1.63	0.0316	0.55	1.46	0.1832
-1.21	-2.32	5.00E-04	-0.63	-1.55	0.0251	0.61	1.52	0.086

0.29	1.22	0.3668	0.55	1.46	0.0521	-0.39	-1.31	0.2715
-0.35	-1.28	0.2532	-0.14	-1.1	0.5986	0.65	1.57	0.0546
-0.49	-1.41	0.134	0.01	1.01	0.9674	0.11	1.08	0.7608
0.94	1.91	0.0088	1.54	2.91	0	-0.18	-1.14	0.6236
0.4	1.32	0.5534	-1.92	-3.79	0.0041	0.2	1.15	0.7784
-0.51	-1.42	0.1285	-0.4	-1.32	0.169	0.54	1.45	0.1239
-0.66	-1.58	0.0633	-0.39	-1.31	0.2086	0.81	1.75	0.0339
-1.65	-3.13	5.00E-04	-0.44	-1.36	0.2592	0.16	1.12	0.7385
-0.37	-1.3	0.2479	-0.11	-1.08	0.6843	-0.24	-1.18	0.5024
-1.12	-2.18	0.004	-0.07	-1.05	0.8096	-0.7	-1.63	0.0741
0.56	1.47	0.1007	0.52	1.44	0.0905	-0.65	-1.57	0.0852
-0.91	-1.88	0.0082	-0.47	-1.38	0.0997	0.72	1.65	0.0368
0.21	1.16	0.5015	-0.56	-1.48	0.0762	0.68	1.6	0.07
0.67	1.59	0.041	0.67	1.59	0.0248	-0.63	-1.55	0.0772
-0.84	-1.79	0.0322	-0.52	-1.43	0.1297	0.28	1.21	0.508
-0.62	-1.53	0.0745	-0.93	-1.9	0.0044	1.17	2.25	0.0022
-0.41	-1.33	0.2444	-0.1	-1.07	0.7411	-0.3	-1.23	0.3879
0.5	1.42	0.2133	0.88	1.84	0.015	0.25	1.19	0.5646
2.67	6.38	0	2.05	4.13	0	-1.75	-3.37	0.0017
0.02	1.01	0.9547	0.36	1.28	0.1818	-0.54	-1.45	0.1239
-2.05	-4.13	0	-0.87	-1.83	0.0147	1.02	2.02	0.0229
0.08	1.06	0.7986	0.11	1.08	0.7105	0.15	1.11	0.6794
-0.79	-1.73	0.0318	-0.37	-1.29	0.2363	0.18	1.13	0.6464
0.42	1.34	0.1988	-0.13	-1.1	0.6756	0.48	1.4	0.1906
-0.36	-1.28	0.2927	-0.26	-1.2	0.3886	0.13	1.1	0.7051
-0.49	-1.4	0.179	-0.21	-1.16	0.4931	0.47	1.38	0.2273
-0.1	-1.07	0.7642	-0.13	-1.1	0.6653	0.25	1.19	0.4797
0.68	1.61	5.00E-04	0.39	1.31	0.0344	-0.31	-1.24	0.177
0.66	1.58	0.0407	0.74	1.67	0.0103	-0.79	-1.73	0.0498
0.02	1.01	0.9475	0.18	1.13	0.4034	0.08	1.06	0.7729
0.17	1.13	0.5874	-0.24	-1.18	0.4304	0.82	1.77	0.0299
0.37	1.29	0.2726	0.12	1.09	0.7084	-0.14	-1.11	0.714
0.4	1.32	0.2278	0.15	1.11	0.6206	-0.2	-1.15	0.5913
-1.34	-2.53	0.0013	-0.3	-1.23	0.3496	0.28	1.21	0.5009
-0.9	-1.86	0.0317	-0.67	-1.59	0.0665	0.34	1.26	0.4262
-0.59	-1.51	0.1051	-0.27	-1.21	0.3868	0.34	1.26	0.3863
0.43	1.35	0.538	-0.2	-1.15	0.7636	-0.09	-1.06	0.9028
-0.94	-1.92	0.0129	-1.36	-2.57	2.00E-04	1.61	3.06	2.00E-04
-1.12	-2.17	0.0048	-0.48	-1.4	0.1301	-0.09	-1.07	0.8147
1.44	2.71	1.00E-04	1.46	2.76	0	-1.54	-2.9	2.00E-04
-0.46	-1.37	0.2104	-0.11	-1.08	0.7344	0.22	1.16	0.5743
0.03	1.02	0.9263	0.6	1.51	0.0464	-0.61	-1.53	0.1162
-1.53	-2.89	3.00E-04	-1.1	-2.14	0.0018	1.58	3	4.00E-04
0.7	1.62	0.037	0.07	1.05	0.8239	-0.2	-1.14	0.639
0.53	1.45	0.1031	0.33	1.26	0.271	-0.49	-1.41	0.1926
1.9	3.75	0	1.01	2.01	0.0069	-0.83	-1.78	0.053
0.84	1.79	0.0158	0.57	1.49	0.0806	0.17	1.13	0.6484

-0.3	-1.23	0.3962	-0.14	-1.1	0.646	0.01	1.01	0.9834
-0.79	-1.73	0.0398	-0.25	-1.19	0.4369	0.61	1.52	0.1416
-0.32	-1.25	0.3546	-0.15	-1.11	0.6176	0.22	1.16	0.5582
2.96	7.77	0	2.77	6.84	0	-2.86	-7.28	0
-1.26	-2.39	0.0031	-0.44	-1.35	0.2092	0.22	1.17	0.632
0.42	1.34	0.1121	0.26	1.2	0.2762	-0.54	-1.45	0.0789
-0.29	-1.22	0.4561	0.44	1.36	0.1665	-0.03	-1.02	0.9478
0.25	1.19	0.479	0.3	1.23	0.3435	-0.26	-1.2	0.4958
-1.41	-2.66	0.0025	-0.37	-1.3	0.3273	0.46	1.38	0.3497
-1.56	-2.95	4.00E-04	-0.83	-1.78	0.0185	1.22	2.32	0.0046
-1.21	-2.32	0.003	-0.53	-1.44	0.1063	0.51	1.43	0.1798
1.56	2.95	0	-0.11	-1.08	0.7787	0.24	1.18	0.5956
-0.19	-1.14	0.5767	-0.3	-1.23	0.3489	0.37	1.29	0.3672
0.17	1.12	0.6632	0.78	1.71	0.0187	-0.85	-1.81	0.0427
0.01	1	0.9852	-0.36	-1.28	0.2762	0.25	1.19	0.5059
-0.01	-1.01	0.9834	-0.29	-1.22	0.4103	0.46	1.38	0.2696
-0.36	-1.28	0.3438	0.3	1.23	0.3386	-0.25	-1.19	0.5206
0.59	1.5	0.1067	-0.27	-1.2	0.4674	0.54	1.45	0.2128
-0.59	-1.51	0.1327	-0.08	-1.06	0.8068	-0.29	-1.22	0.4733
2.43	5.37	0.001	2.09	4.26	0.0045	-1.2	-2.3	0.1084
0.54	1.45	0.1089	0.23	1.17	0.4655	-0.2	-1.15	0.5962
-1.43	-2.69	0.0025	-0.16	-1.12	0.668	0.43	1.35	0.3729
1.21	2.32	1.00E-04	1.02	2.03	4.00E-04	-0.83	-1.78	0.0165
0.09	1.06	0.8045	0.09	1.06	0.7798	0.25	1.19	0.5317
-2	-3.99	3.00E-04	-0.2	-1.15	0.6407	1.18	2.27	0.0219
-0.1	-1.07	0.7795	-0.31	-1.24	0.3625	0.44	1.36	0.2794
-0.21	-1.15	0.5643	-0.36	-1.28	0.2769	1.21	2.31	0.0029
0.03	1.02	0.946	0.93	1.9	0.0075	-1.19	-2.27	0.01
0.67	1.6	0.0854	0.31	1.24	0.4049	-0.35	-1.28	0.4176
0.11	1.08	0.7843	-0.21	-1.15	0.58	0.11	1.08	0.8097
-1	-1.99	0.0322	0.09	1.07	0.8048	0.7	1.63	0.1277
-0.88	-1.84	0.0425	0.12	1.09	0.7219	-0.15	-1.11	0.7411
0.89	1.85	0.0364	0.84	1.78	0.0331	-0.77	-1.71	0.1054
-0.38	-1.3	0.3216	0.14	1.1	0.6612	-0.63	-1.55	0.1342
0.7	1.63	0.0398	0.01	1.01	0.9786	-0.21	-1.15	0.5928
-1.22	-2.32	0.0054	-0.35	-1.27	0.3207	0.36	1.29	0.4021
0.44	1.36	0.0237	0.13	1.09	0.4937	0.39	1.31	0.0841
0.92	1.89	0.0084	-0.69	-1.61	0.055	0.79	1.73	0.0625
-0.94	-1.92	0.0192	-0.53	-1.44	0.1161	0.5	1.41	0.2193
-0.19	-1.14	0.5954	0.16	1.11	0.6177	-0.24	-1.18	0.5658
0.41	1.33	0.2664	-0.06	-1.04	0.8662	-0.11	-1.08	0.7915
-0.39	-1.31	0.3082	0.19	1.14	0.5437	-0.13	-1.09	0.7544
0.07	1.05	0.8592	-0.13	-1.1	0.7061	-0.5	-1.42	0.2744
0.37	1.29	0.2751	-0.24	-1.18	0.4773	0.23	1.17	0.5863
0.5	1.42	0.167	0.21	1.16	0.5411	0.47	1.39	0.2681
0.61	1.53	0.1035	0.79	1.72	0.0196	-1.2	-2.29	0.0054
0.88	1.84	0.0208	0.8	1.74	0.0228	-0.57	-1.49	0.1717

-1.22	-2.33	0.0194	-1.7	-3.25	7.00E-04	1.2	2.29	0.0338
-0.71	-1.64	0.1094	0.16	1.12	0.6479	-0.03	-1.02	0.9534
-0.73	-1.65	0.0551	-1.09	-2.13	0.0026	0.68	1.6	0.1373
-0.26	-1.2	0.4802	-0.23	-1.18	0.4841	0.41	1.33	0.3189
-2.13	-4.37	0	-0.35	-1.28	0.3288	0.78	1.72	0.082
0.01	1	0.9855	-0.56	-1.48	0.1046	0.26	1.2	0.5349
-1.48	-2.79	9.00E-04	-0.96	-1.94	0.0103	0.82	1.77	0.0912
-0.89	-1.85	0.0317	-0.62	-1.54	0.0821	0.89	1.86	0.0364
-0.92	-1.9	0.0252	-0.57	-1.48	0.104	0.47	1.38	0.2701
0.51	1.42	0.3417	0.07	1.05	0.8936	-0.12	-1.09	0.8354
0.95	1.94	0.0179	1.02	2.03	0.0054	-0.73	-1.66	0.1036
-0.82	-1.77	0.0513	-0.56	-1.48	0.1244	1.64	3.11	4.00E-04
-0.52	-1.44	0.1829	-0.33	-1.26	0.3305	0.22	1.17	0.5979
-1.4	-2.65	0.0068	-0.32	-1.25	0.4573	0.81	1.75	0.1425
-1.2	-2.3	0.0099	-0.33	-1.25	0.3745	0.26	1.2	0.5666
0.75	1.68	0.0364	0.32	1.25	0.3595	-0.15	-1.11	0.7294
-0.04	-1.03	0.915	-0.5	-1.42	0.1581	0.74	1.67	0.0876
-0.19	-1.14	0.6531	-0.71	-1.64	0.0836	0.75	1.69	0.1145
1.05	2.07	0.0075	0.68	1.61	0.0689	-0.55	-1.46	0.2027
-0.36	-1.28	0.3643	-0.52	-1.43	0.1584	0.12	1.09	0.7708
0.62	1.53	0.1163	0.4	1.32	0.278	-0.69	-1.61	0.1291
0.76	1.69	0.0389	0.15	1.11	0.686	0.56	1.47	0.1854
0.81	1.75	0.0278	0.42	1.34	0.233	-0.85	-1.81	0.0618
-0.13	-1.1	0.74	-0.85	-1.8	0.0351	0.73	1.66	0.1389
-0.06	-1.04	0.8764	0.03	1.02	0.9296	0.21	1.16	0.5966
0.91	1.88	0.0065	-0.16	-1.12	0.6425	0.01	1	0.9894
1.09	2.13	0.0042	0.41	1.33	0.2786	0.12	1.08	0.7884
0.58	1.5	0.1562	0.05	1.04	0.8989	-0.58	-1.49	0.2594
-0.54	-1.45	0.1702	-0.43	-1.35	0.2092	0.56	1.47	0.1863
-0.2	-1.15	0.6412	0.85	1.81	0.012	-0.4	-1.32	0.3566
1.22	2.33	0.0325	1.81	3.51	6.00E-04	-1.91	-3.76	0.0025
0.23	1.18	0.541	-0.15	-1.11	0.6885	-0.02	-1.01	0.9704
-1.21	-2.31	0.0086	-0.55	-1.47	0.1409	1.59	3.01	0.0014
1.3	2.46	0.0022	0.72	1.64	0.0806	-0.39	-1.31	0.445
-0.37	-1.29	0.3586	0.02	1.01	0.9625	0.19	1.14	0.6451
0.62	1.54	0.08	0.62	1.53	0.0582	0.07	1.05	0.8571
0.68	1.6	0.1131	0.47	1.38	0.2437	-0.84	-1.79	0.0794
-0.37	-1.29	0.3818	-0.07	-1.05	0.8475	0.1	1.07	0.8338
-1.42	-2.68	0.0034	-0.44	-1.36	0.2543	0.25	1.19	0.6105
-0.15	-1.11	0.7139	-0.01	-1.01	0.9668	-0.51	-1.42	0.2574
-1.32	-2.5	0.0096	-0.51	-1.42	0.2261	-0.15	-1.11	0.7639
-1.62	-3.08	0.0052	-0.19	-1.14	0.6883	0.29	1.23	0.6107
-1.05	-2.07	0.0647	-0.18	-1.13	0.7253	-0.11	-1.08	0.8469
-0.17	-1.13	0.6811	0.47	1.39	0.1796	0.29	1.22	0.5023
-0.17	-1.13	0.6789	0.01	1.01	0.968	-0.26	-1.2	0.5871
-0.02	-1.01	0.9622	0.23	1.17	0.5514	-0.32	-1.25	0.4653
-1.12	-2.17	0.0179	-0.03	-1.02	0.9369	-0.06	-1.04	0.8964

0.62	1.54	0.1233	0.69	1.61	0.0595	-0.13	-1.09	0.7747
-1.01	-2.02	0.0146	-0.21	-1.16	0.5246	0.25	1.19	0.548
0.42	1.34	0.2936	-0.59	-1.51	0.1543	-0.22	-1.16	0.6495
-0.48	-1.39	0.2533	-0.78	-1.71	0.0502	0.43	1.34	0.3569
-0.5	-1.42	0.2619	-0.29	-1.22	0.4719	0.49	1.4	0.3111
0.18	1.13	0.6532	-0.63	-1.55	0.1245	1.11	2.15	0.022
0.46	1.38	0.2855	1.19	2.28	0.0013	-1.29	-2.44	0.0072
-0.58	-1.49	0.1797	-0.86	-1.82	0.033	0.24	1.18	0.6338
-0.33	-1.26	0.402	-0.8	-1.75	0.0356	0.7	1.62	0.1323
1.48	2.79	0.0022	1.37	2.58	0.0026	-0.89	-1.86	0.0979
-0.01	-1	0.99	0.62	1.53	0.1018	-0.59	-1.51	0.2056
0	1	0.9921	0.31	1.24	0.4787	-0.52	-1.44	0.3451
-0.18	-1.14	0.7809	0.73	1.65	0.2483	0.18	1.13	0.7983
-0.53	-1.44	0.2038	-0.84	-1.8	0.0322	0.65	1.57	0.1733
1.25	2.38	0.0017	1.38	2.59	2.00E-04	-1.62	-3.07	3.00E-04
-0.6	-1.51	0.1687	-0.2	-1.15	0.5909	0.76	1.7	0.0896
-0.41	-1.33	0.3657	-0.2	-1.14	0.6275	0.42	1.34	0.3806
1.89	3.71	5.00E-04	2.76	6.76	0	-1.34	-2.54	0.0215
-0.04	-1.03	0.9216	-0.12	-1.09	0.7565	0.06	1.04	0.8989
0.94	1.92	0.0331	0.58	1.49	0.1734	-0.62	-1.53	0.2116
-2.42	-5.34	0	-1.69	-3.24	1.00E-04	1.52	2.86	0.0028
-0.7	-1.62	0.1079	-0.61	-1.53	0.114	0.58	1.5	0.2372
1.06	2.08	0.0059	0.4	1.32	0.2881	-0.11	-1.08	0.8129
-0.44	-1.36	0.3392	-0.24	-1.18	0.5617	0.62	1.54	0.2197
0.74	1.67	0.0744	0.73	1.66	0.0529	-1.39	-2.63	0.0038
1.62	3.07	0.001	1.9	3.72	0	-3.43	-10.81	0
-0.11	-1.08	0.789	0.24	1.18	0.5037	-0.11	-1.08	0.8176
-0.42	-1.34	0.3759	0.3	1.23	0.4548	0.45	1.37	0.3243
0.57	1.49	0.1945	0.79	1.72	0.0482	-0.55	-1.47	0.2866
0.06	1.04	0.8764	-0.74	-1.67	0.0641	0.43	1.35	0.3433
0.76	1.69	0.0237	0.41	1.33	0.2014	0	1	0.9929
0.26	1.2	0.5014	-0.3	-1.23	0.4332	0	-1	0.9988
-1.23	-2.35	0.011	-0.96	-1.95	0.0224	1.38	2.6	0.0056
1.4	2.64	0.002	1.55	2.94	2.00E-04	-1.5	-2.83	0.0039
-0.22	-1.16	0.6151	0.29	1.22	0.4382	-0.54	-1.45	0.2873
-1.8	-3.48	5.00E-04	-0.85	-1.8	0.0384	1.1	2.15	0.0361
0.19	1.14	0.6621	0.27	1.21	0.4787	-0.8	-1.74	0.0953
0.36	1.29	0.4105	0.79	1.73	0.04	-1.15	-2.22	0.0206
-0.55	-1.46	0.231	0.13	1.09	0.7331	-0.87	-1.83	0.0736
0.79	1.73	0.0868	-0.61	-1.52	0.2097	0.77	1.71	0.1575
-1.34	-2.54	0.0131	-0.15	-1.11	0.7335	0.18	1.13	0.7557
1.8	3.49	4.00E-04	1.29	2.45	0.0086	-1.3	-2.47	0.0215
1.15	2.22	0.0072	1.01	2.01	0.012	0.02	1.01	0.9682
-0.45	-1.37	0.3234	-0.54	-1.46	0.1982	0.97	1.96	0.0598
-0.97	-1.96	0.0529	-0.5	-1.41	0.2535	-0.27	-1.21	0.5987
1.59	3.01	0.0012	1.13	2.19	0.0164	-0.89	-1.86	0.0963
-1.32	-2.5	0.0047	-1.28	-2.42	0.0024	0.59	1.51	0.2467

-0.39	-1.31	0.3892	-0.16	-1.12	0.6902	0.3	1.23	0.5485
1.48	2.79	0	0.2	1.15	0.5587	0.08	1.06	0.8284
0.91	1.88	0.0388	0.56	1.48	0.1799	-0.19	-1.14	0.7024
0.41	1.33	0.355	0.52	1.44	0.1941	-1.03	-2.04	0.042
-0.69	-1.61	0.1836	0.59	1.5	0.1535	-0.98	-1.97	0.0643
-0.68	-1.6	0.1845	0	1	0.9996	0.17	1.12	0.7478
0.65	1.57	0.1285	0.08	1.06	0.8514	0.48	1.4	0.2997
-0.02	-1.02	0.9588	0.08	1.06	0.8348	0.01	1	0.9899
-0.76	-1.69	0.0925	-1.03	-2.04	0.0154	0.83	1.77	0.102
-1.05	-2.07	0.0588	-0.5	-1.41	0.314	-0.38	-1.31	0.4954
0.16	1.12	0.7159	-0.34	-1.27	0.4339	0.71	1.64	0.1877
-1.7	-3.26	0.001	-0.55	-1.46	0.167	0.38	1.3	0.4642
-1.14	-2.2	0.0229	0.27	1.2	0.4802	0.44	1.36	0.3746
-0.55	-1.47	0.2255	-0.07	-1.05	0.8599	-0.67	-1.59	0.1747
0.47	1.38	0.2698	0.07	1.05	0.8559	-0.23	-1.17	0.623
0.13	1.1	0.7462	-0.6	-1.52	0.1481	0.23	1.17	0.6428
1	2	0.0238	0.93	1.91	0.024	-0.5	-1.41	0.3028
1.13	2.19	0.0102	1.01	2.01	0.0147	-0.96	-1.94	0.0558
-0.25	-1.19	0.5578	-0.64	-1.56	0.1223	0.82	1.77	0.0988
1.05	2.07	0.0196	1.29	2.45	0.0015	-1.26	-2.39	0.0149
-1.36	-2.56	9.00E-04	-0.67	-1.59	0.0391	0.39	1.31	0.3336
0.48	1.4	0.2621	-0.56	-1.47	0.215	0.16	1.12	0.7465
-0.53	-1.45	0.3048	0.27	1.2	0.5351	-0.01	-1	0.9907
-0.95	-1.93	0.0736	-0.38	-1.31	0.4029	1.02	2.03	0.08
0.79	1.73	0.074	0.65	1.57	0.114	-0.66	-1.58	0.2039
-0.63	-1.55	0.1683	-0.39	-1.31	0.3264	0.25	1.19	0.6064
-0.37	-1.29	0.4314	0.49	1.4	0.1968	-0.17	-1.12	0.7426
0.58	1.49	0.1952	0.26	1.2	0.5432	-0.52	-1.44	0.3306
0.62	1.53	0.1404	-0.74	-1.67	0.1029	0.03	1.02	0.9477
-0.58	-1.5	0.2334	-0.66	-1.58	0.1436	0.44	1.36	0.388
-0.84	-1.79	0.1368	0.31	1.24	0.5135	-1.49	-2.81	0.0178
1.99	3.96	1.00E-04	2.19	4.57	0	-1.3	-2.46	0.0097
-3.27	-9.64	0	-1.9	-3.74	1.00E-04	1.91	3.75	9.00E-04
-0.22	-1.16	0.6328	-0.75	-1.68	0.0896	1.25	2.37	0.023
1.13	2.19	0.0092	-0.02	-1.02	0.9604	-0.21	-1.15	0.6773
1.93	3.82	3.00E-04	2	4.01	1.00E-04	-1.26	-2.39	0.0309
-0.83	-1.78	0.0987	0.27	1.2	0.5062	-0.7	-1.63	0.1713
0.13	1.09	0.7977	0.37	1.3	0.4024	-0.94	-1.92	0.1058
-0.47	-1.38	0.3175	-0.26	-1.2	0.5336	0.38	1.3	0.4521
0.16	1.11	0.7285	-0.15	-1.11	0.7177	0.31	1.24	0.5477
0.12	1.09	0.7972	0.83	1.77	0.0399	-1	-2.01	0.0612
0.45	1.37	0.3426	0.77	1.71	0.069	-1.54	-2.9	0.0058
0.06	1.04	0.8933	0.46	1.38	0.242	-0.54	-1.45	0.2989
-1.68	-3.2	0.0016	-1.03	-2.04	0.0209	2.13	4.37	1.00E-04
0.26	1.2	0.5637	-0.16	-1.12	0.7054	0.44	1.36	0.3946
-1.17	-2.25	4.00E-04	-0.21	-1.16	0.419	0.3	1.23	0.3558
1.02	2.03	0.0276	0.8	1.74	0.0689	-0.86	-1.82	0.0854

2.11	4.31	0	1.4	2.64	0.0041	-1.28	-2.43	0.013
-0.4	-1.32	0.3989	-1.03	-2.05	0.028	2.31	4.95	1.00E-04
-0.71	-1.63	0.1754	-0.32	-1.25	0.4847	0.84	1.8	0.1323
0.76	1.7	0.0887	0.66	1.58	0.1148	-0.62	-1.54	0.2236
1.13	2.19	0.0185	1.21	2.32	0.0063	-1.21	-2.31	0.0169
-1.13	-2.19	0.0266	-0.3	-1.23	0.4708	0.14	1.1	0.796
0.79	1.72	0.0829	0.27	1.21	0.5336	0.32	1.25	0.5433
-2.18	-4.54	3.00E-04	-1.55	-2.92	0.0033	1	2	0.0841
0.43	1.34	0.4081	0.84	1.79	0.0653	-1.23	-2.35	0.0255
0.47	1.38	0.3029	0.21	1.16	0.6193	-1	-2	0.0788
-0.01	-1	0.9901	0.67	1.59	0.125	-1.42	-2.67	0.0147
-0.49	-1.41	0.4097	0.9	1.86	0.0855	-0.55	-1.47	0.3712
0.3	1.23	0.5368	-0.16	-1.12	0.7285	-0.39	-1.31	0.4817
-0.34	-1.27	0.4741	0.01	1	0.9897	0.82	1.76	0.1288
-0.42	-1.34	0.4586	-0.72	-1.65	0.187	1.08	2.12	0.0723
1.76	3.38	0.003	2.98	7.87	0	-1.42	-2.67	0.0235
1.12	2.18	0.0138	1.17	2.25	0.0053	-1.29	-2.45	0.0125
0.45	1.37	0.3148	0.08	1.05	0.8606	0.07	1.05	0.9008
-0.34	-1.26	0.4833	-0.77	-1.71	0.0993	-0.14	-1.11	0.7824
0.28	1.21	0.5425	-0.49	-1.41	0.2819	0.39	1.31	0.481
-0.83	-1.78	0.1184	-0.18	-1.13	0.6972	0.11	1.08	0.8419
0.45	1.36	0.439	0.7	1.62	0.1927	-1.55	-2.93	0.013
0.39	1.31	0.4253	0.43	1.35	0.3364	-0.44	-1.35	0.415
-0.37	-1.29	0.4781	-0.37	-1.29	0.4384	-0.6	-1.51	0.2675
0.59	1.5	0.2246	0.13	1.1	0.7789	-0.35	-1.27	0.5339
-0.75	-1.69	0.1274	-0.67	-1.6	0.1314	0.7	1.62	0.1863
-0.84	-1.79	0.1222	-0.04	-1.03	0.9349	0.64	1.56	0.258
0.7	1.63	0.1296	0.92	1.9	0.0272	-0.87	-1.83	0.0949
-0.75	-1.68	0.1447	-0.15	-1.11	0.7399	-0.15	-1.11	0.7798
4.59	24.1	0	3.87	14.67	0	-1.68	-3.21	0.0045
0.02	1.01	0.9743	0.34	1.27	0.4159	-0.94	-1.92	0.078
-0.63	-1.55	0.1997	-0.32	-1.25	0.4606	0.37	1.29	0.4751
0.46	1.38	0.3471	0.43	1.35	0.347	-0.3	-1.24	0.5743
-0.73	-1.65	0.1702	-0.13	-1.09	0.7749	-0.68	-1.6	0.2266
0.87	1.83	0.0833	0.6	1.52	0.2123	-0.88	-1.84	0.1336
-1.28	-2.42	0.0161	-1.25	-2.38	0.0104	1.49	2.82	0.0108
0.4	1.32	0.4153	0.13	1.09	0.7867	0.45	1.37	0.4108
-0.3	-1.23	0.5365	0.03	1.02	0.945	-0.32	-1.25	0.5123
-0.28	-1.21	0.5687	-0.19	-1.14	0.6637	-0.07	-1.05	0.8865
-1.21	-2.32	0.0256	-0.82	-1.76	0.0879	-0.32	-1.25	0.5642
-1.05	-2.06	0.0436	-1.34	-2.54	0.0066	1.76	3.39	0.0033
0.83	1.77	0.1804	-0.27	-1.21	0.649	-0.06	-1.04	0.9308
1.13	2.19	0.0253	-0.96	-1.95	0.0824	-0.96	-1.94	0.1247
-0.43	-1.35	0.3668	-0.87	-1.82	0.0609	0.1	1.07	0.8504
0.21	1.16	0.6476	0.26	1.2	0.5411	0.38	1.3	0.4645
-0.89	-1.86	0.0883	-0.6	-1.52	0.1967	-0.33	-1.26	0.5777
-1.06	-2.08	0.104	-0.02	-1.01	0.9793	1.46	2.76	0.0293

0.14	1.1	0.8088	1.39	2.62	0.0069	-2.33	-5.02	3.00E-04
0.44	1.36	0.4033	0.29	1.22	0.5602	-0.32	-1.25	0.5683
0.59	1.51	0.192	-0.32	-1.25	0.4888	0.13	1.09	0.8109
1.27	2.42	0.0121	1.12	2.17	0.02	-1	-2	0.0687
0.41	1.32	0.3825	-0.74	-1.67	0.1308	0.43	1.35	0.4155
-0.01	-1.01	0.9817	-0.46	-1.38	0.3095	-0.02	-1.01	0.9719
-0.32	-1.25	0.511	-0.57	-1.49	0.2148	-0.24	-1.18	0.6498
1.46	2.75	0.005	0.41	1.33	0.4315	-0.57	-1.48	0.3393
0.4	1.32	0.4356	0.86	1.82	0.0584	-1.75	-3.37	0.0034
1.16	2.23	0.0289	0.73	1.66	0.1502	0.36	1.28	0.5475
0.87	1.83	0.077	0.9	1.86	0.0477	-1.22	-2.33	0.0287
-0.89	-1.86	0.0828	-0.31	-1.24	0.4762	0.13	1.1	0.8076
-0.96	-1.94	0.0735	-0.23	-1.18	0.6004	-0.54	-1.45	0.336
1.01	2.01	0.0482	0.23	1.17	0.6521	0.19	1.14	0.7394
-0.33	-1.25	0.4948	-0.23	-1.18	0.5912	-0.15	-1.11	0.7737
-0.95	-1.94	0.0994	-0.27	-1.21	0.5932	-1.09	-2.13	0.0761
-0.31	-1.24	0.5243	-0.03	-1.02	0.9376	0.11	1.08	0.8361
-0.07	-1.05	0.8884	-0.84	-1.79	0.0893	0.77	1.71	0.1445
1.21	2.31	0.0223	1.06	2.09	0.0335	-0.95	-1.94	0.1005
-0.54	-1.45	0.3503	-1.17	-2.25	0.0389	1.41	2.65	0.0362
-0.11	-1.08	0.8347	0.42	1.33	0.3615	-0.79	-1.73	0.1633
-1.71	-3.27	0.0046	-0.36	-1.28	0.4606	0.07	1.05	0.9037
-1.76	-3.39	0.0033	-0.09	-1.07	0.8425	-0.54	-1.45	0.3555
-0.33	-1.26	0.5033	-0.15	-1.11	0.7374	0.22	1.16	0.6864
1.44	2.71	0.0079	1	2	0.0563	-1.99	-3.97	7.00E-04
-1.74	-3.34	0.0019	-1.06	-2.08	0.0252	1.53	2.89	0.0098
-1.55	-2.93	0.0114	-1.34	-2.53	0.0192	1.83	3.55	0.0053
-0.81	-1.76	0.1278	-0.63	-1.54	0.1946	0.59	1.51	0.3195
-2.61	-6.1	1.00E-04	-1.22	-2.33	0.0326	1.31	2.47	0.0478
-0.14	-1.1	0.773	-0.23	-1.17	0.6134	0.5	1.41	0.372
-0.51	-1.43	0.3267	-1.33	-2.51	0.0131	1.72	3.29	0.0046
-0.23	-1.18	0.6405	-0.25	-1.19	0.5879	0.66	1.58	0.2431
1.39	2.63	0.0066	1.17	2.25	0.0164	-0.58	-1.5	0.2951
0.29	1.22	0.5451	-0.16	-1.12	0.7224	-0.21	-1.16	0.6931
0.4	1.32	0.4447	0.55	1.47	0.241	-0.13	-1.09	0.8186
0.24	1.18	0.6511	0.88	1.84	0.0614	-1.28	-2.43	0.0282
-0.09	-1.06	0.8697	0.15	1.11	0.7439	-0.99	-1.99	0.0985
-0.98	-1.97	0.0669	-1.53	-2.88	0.004	1.46	2.74	0.0149
0.19	1.14	0.7819	-0.12	-1.09	0.8625	-0.07	-1.05	0.9122
0	-1	0.9988	-1.22	-2.33	0.018	0.5	1.41	0.3976
1.42	2.67	0.0117	1.87	3.65	3.00E-04	-2.06	-4.18	0.0014
-1.82	-3.53	0.0023	-0.7	-1.63	0.15	0.89	1.85	0.1059
-0.33	-1.26	0.5384	0.26	1.2	0.577	-0.5	-1.41	0.372
0.83	1.77	0.1092	0.4	1.32	0.4232	-0.07	-1.05	0.8966
-0.21	-1.15	0.7229	0.49	1.4	0.3316	-1.03	-2.04	0.0989
2.73	6.62	0	3.88	14.7	0	-2.02	-4.05	0.0029
-0.63	-1.55	0.2672	-0.29	-1.23	0.5664	0.03	1.02	0.9591

-0.78	-1.72	0.1659	-0.04	-1.03	0.9327	-0.28	-1.22	0.6315
-2.69	-6.46	0	-3.61	-12.21	0	2.92	7.55	0
-2.17	-4.52	5.00E-04	-1.04	-2.05	0.0502	-0.99	-1.98	0.1424
0.54	1.46	0.2523	-0.24	-1.18	0.6215	0.39	1.31	0.4841
-0.62	-1.54	0.2647	-0.27	-1.21	0.5844	0.17	1.12	0.7805
1	2	0.0328	0.49	1.4	0.2891	-0.73	-1.66	0.1922
1.37	2.58	0.0075	0.57	1.48	0.2638	-0.94	-1.93	0.1034
0.7	1.63	0.1774	0.68	1.6	0.1652	-0.72	-1.65	0.2034
0.08	1.06	0.8829	-0.09	-1.06	0.861	1.08	2.11	0.0729
0.56	1.47	0.3088	0.63	1.55	0.2116	-0.52	-1.43	0.3847
-1.27	-2.41	0.0267	-1.21	-2.31	0.0225	1.27	2.41	0.0407
-1.01	-2.01	0.0943	-0.12	-1.08	0.8261	-0.34	-1.27	0.5845
-1.78	-3.44	0.0044	-0.04	-1.03	0.9414	-0.66	-1.58	0.2733
-2.56	-5.92	0	-1.4	-2.64	0.0065	1.82	3.54	0.0041
2.68	6.39	1.00E-04	3.67	12.75	0	-2.82	-7.05	0
-0.33	-1.26	0.5282	-0.22	-1.16	0.6525	0.17	1.12	0.7772
-0.21	-1.15	0.5355	0.04	1.03	0.8804	0.03	1.02	0.9364
-1.2	-2.29	0.0447	0.26	1.2	0.5853	-1.05	-2.06	0.071
1.74	3.34	0.0065	2.66	6.3	0	-2.21	-4.62	4.00E-04
-0.55	-1.46	0.3083	-0.03	-1.02	0.9469	0.64	1.56	0.2889
-1.06	-2.08	0.0631	-0.75	-1.68	0.1443	0.26	1.19	0.6678
-0.04	-1.03	0.9457	0.51	1.43	0.2764	0.1	1.07	0.8573
-1.2	-2.3	0.0149	-1.28	-2.43	0.0048	1.27	2.42	0.0125
-0.59	-1.5	0.2917	0.17	1.13	0.7204	0.07	1.05	0.9062
0.73	1.66	0.1845	0.9	1.86	0.0784	-0.91	-1.88	0.1284
3.05	8.26	0	3.1	8.58	0	-2.5	-5.66	2.00E-04
1.44	2.71	0.0499	0.59	1.51	0.4155	-0.68	-1.6	0.3667
-0.32	-1.24	0.5611	-0.98	-1.97	0.072	0.59	1.51	0.3711
0.22	1.17	0.69	0.24	1.18	0.6458	-0.01	-1.01	0.9834
1.82	3.52	0.001	1.53	2.9	0.0037	-2.01	-4.04	0.0013
-0.68	-1.6	0.2479	-0.14	-1.1	0.7901	0.27	1.2	0.6806
0.67	1.59	0.2039	0.24	1.18	0.6362	-0.35	-1.27	0.5786
-1.7	-3.26	0.0095	-0.33	-1.26	0.5721	-0.27	-1.21	0.6873
1.36	2.56	0.0243	1.41	2.65	0.0137	0.17	1.13	0.7849
0.4	1.32	0.4429	-0.03	-1.02	0.9457	0.18	1.13	0.7555
0.28	1.21	0.5968	-0.74	-1.68	0.1689	0.58	1.49	0.3281
1.43	2.7	0.0058	0.7	1.62	0.1779	0.3	1.23	0.6164
-0.36	-1.28	0.5643	-0.73	-1.66	0.2301	1.53	2.89	0.0292
-1.35	-2.55	0.0245	-0.44	-1.35	0.3929	0.58	1.5	0.3342
-1.29	-2.44	0.0287	-1.14	-2.2	0.0354	0.66	1.58	0.2971
-0.47	-1.39	0.4389	0.43	1.35	0.4215	0.52	1.43	0.4393
-0.88	-1.84	0.1918	-0.02	-1.01	0.9778	0.68	1.6	0.3403
-0.27	-1.2	0.6216	0.15	1.11	0.746	0.48	1.4	0.4028
-0.18	-1.13	0.7369	-0.86	-1.82	0.1106	0.71	1.63	0.2682
-0.58	-1.5	0.2182	-1.22	-2.32	0.0099	1.7	3.25	0.0023
-0.88	-1.84	0.1295	-0.35	-1.28	0.4907	0.74	1.67	0.2315
-0.38	-1.3	0.5197	0.72	1.64	0.1472	-1.56	-2.94	0.0125

0.86	1.81	0.1253	1.07	2.1	0.0368	-1.55	-2.92	0.0118
0.02	1.01	0.9709	-0.16	-1.11	0.7603	-0.24	-1.18	0.691
0.46	1.38	0.3932	0.03	1.02	0.9601	-1.09	-2.12	0.0789
1.07	2.09	0.0563	0.74	1.67	0.1678	-0.44	-1.36	0.453
-1.36	-2.57	0.0242	-1.01	-2.02	0.0653	0.9	1.87	0.1435
-0.61	-1.52	0.3049	-1.14	-2.2	0.0501	0.77	1.7	0.2468
-0.23	-1.18	0.6112	0.05	1.04	0.9019	-0.82	-1.77	0.1254
-0.68	-1.6	0.2706	0.26	1.2	0.6362	-0.1	-1.07	0.8846
0.32	1.25	0.5486	-0.42	-1.34	0.4297	0.7	1.62	0.246
0.46	1.38	0.0418	0.3	1.23	0.1454	0.1	1.07	0.7096
1.16	2.23	0.0411	0.57	1.49	0.3019	-1.48	-2.8	0.0235
-0.83	-1.78	0.1414	-0.6	-1.52	0.2403	-0.19	-1.14	0.7516
0.01	1.01	0.9811	-1	-2	0.0657	0.57	1.49	0.3398
0.59	1.5	0.3232	1.46	2.76	0.0056	-1.93	-3.81	0.0031
0.98	1.97	0.1126	1.1	2.14	0.0588	-0.45	-1.37	0.499
1.32	2.5	0.0379	2.3	4.92	1.00E-04	-2.28	-4.86	4.00E-04
0.94	1.92	0.1957	0.03	1.02	0.9631	0.72	1.65	0.3098
0.8	1.74	0.127	0.55	1.46	0.2758	0.15	1.11	0.8002
-0.1	-1.07	0.8822	0.34	1.26	0.6038	-0.74	-1.67	0.2909
-0.17	-1.13	0.7428	0.22	1.16	0.6403	-0.82	-1.76	0.1571
1.33	2.51	0.0168	0.84	1.79	0.1237	-0.94	-1.92	0.1134
0.14	1.1	0.8126	1.28	2.42	0.0133	-1.61	-3.05	0.0078
-0.58	-1.49	0.3203	0.01	1.01	0.9784	0.06	1.04	0.929
-1.07	-2.11	0.0939	0.65	1.57	0.2199	0.12	1.08	0.8633
0.87	1.82	0.1278	0.68	1.6	0.21	-0.31	-1.24	0.6214
-0.72	-1.64	0.2193	-0.11	-1.08	0.8346	0.28	1.22	0.6416
-1.13	-2.19	0.0628	0.02	1.02	0.9644	-0.45	-1.36	0.4735
0.75	1.69	0.1973	1.08	2.12	0.0423	-0.7	-1.62	0.2409
-2.38	-5.22	1.00E-04	-1.46	-2.76	0.0062	0.78	1.71	0.2165
-1.17	-2.25	0.062	0.22	1.16	0.677	-0.58	-1.49	0.357
0.44	1.35	0.4919	0.22	1.16	0.7265	-1.68	-3.2	0.0121
0.75	1.68	0.1916	0.46	1.38	0.4029	0.17	1.13	0.7779
0.14	1.1	0.8334	1.88	3.67	5.00E-04	-0.82	-1.77	0.1872
-1.17	-2.25	0.0586	-0.82	-1.76	0.1495	0.65	1.57	0.3169
-0.45	-1.36	0.4472	-0.31	-1.24	0.5709	0.16	1.12	0.7907
0.89	1.85	0.1141	0.94	1.92	0.0735	-0.49	-1.41	0.4333
-0.18	-1.13	0.7576	-0.57	-1.49	0.2991	0.36	1.29	0.5444
1.29	2.44	0.0298	1.02	2.03	0.0744	-0.86	-1.81	0.1725
-0.55	-1.47	0.3303	-0.31	-1.24	0.5451	-0.21	-1.16	0.7323
-0.86	-1.81	0.1611	0.45	1.36	0.3763	-1.31	-2.49	0.0332
-1.04	-2.06	0.0978	-0.22	-1.17	0.6888	0.13	1.09	0.8465
-0.81	-1.75	0.1799	-0.18	-1.13	0.7324	0.13	1.09	0.8401
-0.48	-1.39	0.4068	-1.03	-2.04	0.0695	1	1.99	0.1477
0.62	1.54	0.319	1.25	2.38	0.0258	-2.04	-4.11	0.0019
0.24	1.18	0.7058	1.71	3.26	0.0019	-1.84	-3.57	0.0047
0.67	1.59	0.2997	0.84	1.79	0.1686	-0.81	-1.75	0.2284
2.52	5.73	0	1.53	2.89	0.0107	-1.23	-2.34	0.0526

1.99	3.97	0.0016	2.37	5.17	1.00E-04	-1.86	-3.62	0.0035
-0.3	-1.23	0.594	0.14	1.1	0.7846	0.4	1.32	0.4946
0.64	1.56	0.2195	-0.17	-1.12	0.7513	-0.49	-1.4	0.4351
1.07	2.1	0.0181	0.68	1.61	0.1146	0.06	1.04	0.9108
0.48	1.4	0.0923	0.36	1.29	0.1661	-0.33	-1.26	0.3023
0.52	1.44	0.3519	0.78	1.71	0.1317	-1.14	-2.2	0.0651
-1.3	-2.46	0.034	-0.8	-1.75	0.1436	0.26	1.19	0.6934
-0.77	-1.71	0.2147	-0.97	-1.96	0.1058	0.53	1.45	0.4191
0.54	1.45	0.3679	0.98	1.97	0.0738	-1.79	-3.45	0.0087
1.73	3.32	0.0081	2.34	5.06	1.00E-04	-2.43	-5.38	2.00E-04
2.19	4.56	0.0013	2.13	4.38	0.0012	-2.56	-5.91	3.00E-04
0.74	1.67	0.208	1.46	2.74	0.0053	-1.35	-2.55	0.0319
-0.81	-1.75	0.1843	-0.67	-1.59	0.24	-0.38	-1.3	0.5481
0.26	1.2	0.6482	0.28	1.21	0.6074	-0.39	-1.31	0.5601
-1.46	-2.76	0.021	-0.98	-1.98	0.0891	1.39	2.61	0.0333
-1.14	-2.2	0.081	-1.25	-2.39	0.0469	0.55	1.46	0.4354
0.46	1.37	0.5324	0.5	1.41	0.4941	-0.39	-1.31	0.6031
1.45	2.74	0.0222	1.87	3.64	0.0016	-0.76	-1.7	0.2117
0.81	1.75	0.2107	0.64	1.56	0.3015	-0.15	-1.11	0.8306
-0.69	-1.61	0.279	0.35	1.28	0.5272	-1.1	-2.14	0.0974
-1.03	-2.05	0.0896	-1.03	-2.04	0.0738	1.63	3.09	0.0154
0.66	1.58	0.2525	0.44	1.36	0.4238	0.38	1.3	0.5429
-1.26	-2.39	0.0423	-0.26	-1.2	0.6276	0.92	1.89	0.1743
-1.42	-2.68	0.0343	-0.8	-1.74	0.2055	-0.32	-1.25	0.6581
-0.01	-1.01	0.9831	0.72	1.65	0.2672	-1.09	-2.13	0.1312
0.78	1.71	0.0591	0.4	1.32	0.3107	-0.19	-1.14	0.6934
-0.31	-1.24	0.6088	-0.11	-1.08	0.8506	0.52	1.43	0.4211
-1.1	-2.14	0.0785	-0.71	-1.63	0.215	-0.08	-1.06	0.9023
0.71	1.64	0.1709	0.95	1.94	0.0458	-1.38	-2.6	0.0234
1.05	2.07	0.1279	1.06	2.09	0.113	-1.87	-3.65	0.0096
2.29	4.89	0.002	2.36	5.14	0.0013	-0.29	-1.23	0.6924
-1.38	-2.59	0.0302	-1.29	-2.44	0.0323	1.7	3.26	0.0115
-1.68	-3.2	0.0132	-1.36	-2.57	0.0365	1.78	3.43	0.0163
0.31	1.24	0.6441	1.93	3.82	8.00E-04	-2.1	-4.29	0.0016
-0.14	-1.1	0.8409	-1.03	-2.04	0.1323	0.9	1.86	0.2164
-0.49	-1.4	0.4041	-1.37	-2.58	0.0222	0.55	1.47	0.4097
-0.5	-1.42	0.4063	-0.55	-1.47	0.3342	0.83	1.78	0.2019
-1.29	-2.45	0.0411	-1.22	-2.33	0.0414	1.28	2.44	0.0647
3.29	9.81	0	3.06	8.33	0	-0.95	-1.93	0.1511
-2.29	-4.9	8.00E-04	-2.88	-7.34	0	1.36	2.56	0.0664
-0.26	-1.2	0.6834	0.57	1.49	0.3261	-1.57	-2.97	0.0204
0	1	0.9975	0.88	1.84	0.1694	-0.69	-1.61	0.3349
0.99	1.99	0.137	1.55	2.92	0.0132	-2.11	-4.31	0.0021
0.12	1.09	0.8314	-0.7	-1.63	0.2232	-0.14	-1.1	0.8299
-1.03	-2.04	0.1029	-0.39	-1.31	0.4853	0.83	1.78	0.2162
0.49	1.4	0.4561	0.95	1.93	0.1179	-0.55	-1.47	0.4203
-0.98	-1.97	0.1253	-0.14	-1.1	0.8041	0.59	1.5	0.3948

-1.01	-2.01	0.1401	-1.12	-2.17	0.0939	-0.72	-1.64	0.325
-1.83	-3.56	0.0057	-1.39	-2.62	0.0264	1.39	2.61	0.053
1.86	3.63	0.0081	1.98	3.95	0.0035	-2.47	-5.54	5.00E-04
-1.65	-3.14	0.0109	-2.5	-5.64	1.00E-04	1.7	3.24	0.0194
-0.33	-1.26	0.6105	-1.27	-2.41	0.0524	0.98	1.97	0.147
-1.66	-3.17	0.0179	-1.73	-3.32	0.0127	0.78	1.72	0.2982
0.31	1.24	0.6622	1.65	3.13	0.0133	-0.94	-1.92	0.1907
1.29	2.44	0.0295	0.29	1.22	0.6285	-1.04	-2.06	0.1134
1.77	3.41	0.0066	1.28	2.44	0.0443	-1.33	-2.51	0.05
2.05	4.13	0.0034	1.39	2.61	0.0459	-1.58	-3	0.0208
3.63	12.38	0	2.4	5.27	0.0011	-1.59	-3	0.0305
-1.24	-2.35	0.0622	-0.51	-1.43	0.4021	1.57	2.97	0.0277
2.59	6.02	3.00E-04	2.77	6.84	1.00E-04	-2.05	-4.14	0.005
-0.92	-1.89	0.1316	-1.25	-2.38	0.0347	0.77	1.7	0.2674
0.67	1.59	0.292	0.71	1.63	0.2384	-1.72	-3.3	0.0104
-0.92	-1.89	0.1556	-0.87	-1.83	0.1585	1.93	3.8	0.0049
0.47	1.38	0.4724	1.36	2.57	0.0211	-1.49	-2.81	0.035
-0.41	-1.33	0.5369	0.5	1.41	0.4051	-1.91	-3.75	0.0064
-0.69	-1.61	0.3413	0.35	1.28	0.62	-1.87	-3.66	0.0115
0.99	1.99	0.132	1.99	3.98	8.00E-04	-2.33	-5.03	5.00E-04
2.06	4.17	0.0014	1.11	2.16	0.0823	-0.34	-1.27	0.609
1.55	2.94	0.0193	1.12	2.17	0.0834	-0.2	-1.15	0.7777
1.89	3.7	0.0073	2.09	4.26	0.002	-1.88	-3.68	0.0073
0.35	1.28	0.6259	2.48	5.58	2.00E-04	-1.4	-2.64	0.0521
-1.47	-2.77	0.0307	-1.47	-2.77	0.0272	1.07	2.09	0.1308
3.09	8.51	0	2.02	4.06	0.0043	-1.51	-2.85	0.0334
2.55	5.87	3.00E-04	0.99	1.99	0.1633	-1.95	-3.85	0.0076
1.37	2.59	0.0628	1.92	3.8	0.0081	-1.84	-3.59	0.0128
0.65	1.57	0.3194	0.42	1.34	0.4982	0.23	1.18	0.7385
0.44	1.36	0.419	0.97	1.96	0.0466	-0.78	-1.71	0.1908
-0.01	-1.01	0.9897	0.67	1.59	0.3414	-1.87	-3.65	0.0113
0.29	1.22	0.6074	0.54	1.45	0.3009	-0.02	-1.02	0.9693
-0.27	-1.2	0.716	0.68	1.6	0.3597	-1.05	-2.07	0.1488
0.1	1.07	NA	-1.67	-3.19	NA	0.13	1.09	NA
0.01	1.01	0.9851	-0.01	-1.01	0.9898	-0.22	-1.16	0.7693
1.87	3.67	0.0098	2.38	5.21	8.00E-04	-0.37	-1.3	0.6166
1.09	2.13	0.048	1.14	2.2	0.0269	-1.39	-2.62	0.0305
0.66	1.58	0.3634	0.21	1.16	0.7709	-0.56	-1.48	0.4176
1.49	2.8	0.0468	2.2	4.58	0.0029	-2.69	-6.44	2.00E-04
1.47	2.77	4.00E-04	-0.03	-1.02	0.9466	0.27	1.21	0.5667
0.42	1.34	0.3817	0.57	1.48	0.1984	-0.09	-1.06	0.8703
0.55	1.47	0.3095	0.76	1.7	0.1268	-0.9	-1.87	0.1282
0.98	1.97	0.1759	0.51	1.43	0.4716	-1.46	-2.75	0.0493
0.34	1.26	0.6458	0.33	1.26	0.6555	-0.4	-1.32	0.588
0.45	1.36	0.5246	0.47	1.38	0.518	-0.77	-1.7	0.2524
-0.55	-1.47	0.2799	0.31	1.24	0.4785	-0.29	-1.22	0.5777
1.28	2.42	0.0819	2.06	4.18	0.0046	-0.84	-1.79	0.2629

1.22	2.33	0.0359	0.17	1.13	0.7689	-0.04	-1.03	0.9497
1.29	2.44	0.0502	0.45	1.37	0.4864	0.01	1.01	0.9859
0.47	1.38	0.4664	-2.72	-6.59	1.00E-04	2.93	7.65	1.00E-04
0	-1	0.9968	0.76	1.69	0.273	-0.52	-1.43	0.4831
-0.23	-1.17	0.7515	0.93	1.9	0.1845	-1.12	-2.17	0.1323
0.25	1.19	0.723	-0.12	-1.09	0.8625	0.13	1.1	0.837
0.11	1.08	0.8686	0.36	1.29	0.5997	-0.05	-1.04	0.9326
0.71	1.64	0.2785	1.2	2.3	0.0506	-1.97	-3.93	0.0047
1.31	2.49	0.0459	1.32	2.5	0.0369	-1.14	-2.2	0.1077
-0.58	-1.5	0.3322	-2.13	-4.37	7.00E-04	0.2	1.15	0.7884
1.56	2.96	NA	0.81	1.76	NA	0	1	NA
0.5	1.41	0.4424	0.39	1.31	0.56	-0.45	-1.36	0.4501
0.66	1.58	0.3663	1.53	2.88	0.0353	-1.57	-2.97	0.0352
-1.41	-2.66	NA	-1.51	-2.85	NA	0.1	1.07	NA
0.35	1.28	0.5488	0.78	1.72	0.2095	-0.34	-1.26	0.5279

Log2 FC, Fold change, P value								
IgAN E1.vs	IgAN E1.vs	IgAN E1.vs	S.a.GN.vs.A	S.a.GN.vs.A	S.a.GN.vs.A	VancATN.v	VancATN.v	VancATN.v
-0.15	-1.11	0.5263	0.56	1.48	0.0264	-0.27	-1.2	0.2706
-0.16	-1.12	0.3344	-0.05	-1.04	0.7769	-0.2	-1.15	0.2375
-0.03	-1.02	0.8935	-0.09	-1.07	0.6645	0.1	1.07	0.6094
-0.17	-1.13	0.3314	-0.53	-1.44	0.0104	0.19	1.14	0.2694
0.16	1.12	0.3932	-0.15	-1.11	0.4678	0.13	1.09	0.4995
-0.26	-1.2	0.1859	0.06	1.04	0.776	0.03	1.02	0.8598
0.66	1.58	0.0198	0.55	1.46	0.0766	0.1	1.07	0.7471
-0.51	-1.42	0.108	0.05	1.03	0.8922	-0.37	-1.29	0.2419
0	-1	0.9865	0	-1	0.9896	-0.1	-1.07	0.6107
0.6	1.52	0.0141	0.49	1.41	0.0657	0.33	1.26	0.1851
-0.24	-1.18	0.4357	0.86	1.81	0.0087	-0.09	-1.06	0.7787
0.03	1.02	0.8831	0.28	1.22	0.2252	0.01	1.01	0.9704
0.01	1.01	0.969	0.15	1.11	0.5069	-0.01	-1.01	0.9589
0.17	1.13	0.437	-0.24	-1.18	0.3366	0.27	1.21	0.2197
0.09	1.06	0.6608	-0.15	-1.11	0.5334	0.09	1.06	0.6625
-0.27	-1.2	0.2897	-0.56	-1.47	0.0478	0	1	0.9982
-0.31	-1.24	0.2907	0.1	1.07	0.7479	-0.33	-1.26	0.2629
0.16	1.12	0.5227	-0.06	-1.05	0.8158	-0.11	-1.08	0.6764
-0.12	-1.08	0.5914	0.06	1.04	0.8145	0.13	1.1	0.5312
0.02	1.01	0.9422	-0.67	-1.59	0.0142	0.26	1.2	0.2362
-0.19	-1.14	0.3956	-0.39	-1.31	0.1262	-0.34	-1.27	0.1425
0.27	1.2	0.2747	0.6	1.52	0.0186	0.18	1.13	0.4757
0.61	1.53	0.0153	0.19	1.14	0.5	0.39	1.31	0.1349
0.72	1.65	0.0167	0.07	1.05	0.8471	0.35	1.27	0.2652
0.96	1.95	0.0017	0.04	1.03	0.9104	0.36	1.28	0.2577
0.37	1.29	0.1054	-0.04	-1.03	0.8878	0.07	1.05	0.7812
0.33	1.26	0.194	0.11	1.08	0.6921	0.21	1.16	0.4191
0.3	1.23	0.1916	-0.32	-1.24	0.2565	0.12	1.08	0.6311
-0.16	-1.12	0.4832	0.39	1.31	0.0928	0.04	1.03	0.8652
0.26	1.19	0.2856	-0.45	-1.37	0.1161	0.07	1.05	0.7892
-0.04	-1.03	0.8605	-0.96	-1.95	0.0016	-0.09	-1.06	0.7199
0.31	1.24	0.2129	-0.06	-1.04	0.8327	0.14	1.1	0.5817
0.29	1.22	0.2552	0.34	1.26	0.225	0.34	1.26	0.1906
0.21	1.16	0.7081	0.21	1.15	0.7289	-0.24	-1.18	0.6808
-0.07	-1.05	0.7763	0.35	1.28	0.1793	-0.06	-1.04	0.8141
0.7	1.62	0.0218	-0.21	-1.16	0.544	0.15	1.11	0.6308
-0.15	-1.11	0.5552	0.19	1.14	0.477	-0.08	-1.05	0.7662
-0.08	-1.06	0.7597	0.65	1.57	0.0136	-0.17	-1.13	0.5059
0.27	1.21	0.6301	0.68	1.6	0.248	-0.31	-1.24	0.5819
0.13	1.1	0.5926	0.09	1.07	0.7315	0.09	1.07	0.7063
0.14	1.1	0.599	-0.33	-1.26	0.2727	0.15	1.11	0.5681
0.31	1.24	0.2555	0.22	1.17	0.4487	0.27	1.21	0.3115
0.05	1.04	0.9344	0.32	1.25	0.6252	-0.61	-1.52	0.3454

-0.22	-1.16	0.4326	0.48	1.39	0.0986	-0.02	-1.01	0.9416
-0.2	-1.15	0.4007	0.5	1.41	0.0475	-0.51	-1.42	0.0467
0.68	1.6	0.0354	-0.49	-1.41	0.1993	0.36	1.28	0.2784
0.81	1.76	0.0121	-0.06	-1.04	0.8763	0.58	1.5	0.0797
0.2	1.15	0.4491	-0.17	-1.12	0.5792	0.03	1.02	0.915
-0.04	-1.03	0.8928	0.01	1.01	0.9789	-0.01	-1.01	0.9604
-0.38	-1.3	0.1483	-0.36	-1.28	0.2097	-0.24	-1.18	0.3596
-0.22	-1.17	0.3836	-0.29	-1.22	0.3101	0.04	1.03	0.8622
-0.36	-1.29	0.2831	0.09	1.06	0.81	-0.51	-1.43	0.1363
0.41	1.33	0.1555	0.32	1.25	0.3171	0.09	1.06	0.766
-0.83	-1.77	0.0309	-1.29	-2.45	0.0023	-0.31	-1.24	0.4173
-0.25	-1.19	0.4443	-0.41	-1.33	0.2599	0.14	1.1	0.6747
-0.32	-1.25	0.2431	0.05	1.03	0.8741	-0.11	-1.08	0.6965
-0.73	-1.66	0.053	0.63	1.55	0.0977	0.51	1.43	0.1529
-0.68	-1.61	0.045	-0.56	-1.48	0.126	-0.31	-1.24	0.3501
-0.5	-1.41	0.1538	-0.22	-1.16	0.5629	-0.43	-1.35	0.2176
0.35	1.27	0.3148	0	1	0.9916	-0.25	-1.19	0.489
0.33	1.26	0.2688	0.04	1.03	0.9029	0.25	1.19	0.4189
0.12	1.09	0.6745	0.01	1	0.9866	-0.12	-1.09	0.6767
0.91	1.88	0.0257	0.43	1.35	0.3398	0.37	1.3	0.3777
-0.22	-1.17	0.446	-0.24	-1.18	0.4618	-0.24	-1.18	0.4232
1.7	3.25	0	1.26	2.4	0.0016	0.47	1.39	0.2385
0.04	1.03	0.9048	0.19	1.14	0.5804	-0.02	-1.01	0.9561
0.43	1.35	0.1683	-0.28	-1.22	0.4444	0.39	1.31	0.2159
-0.31	-1.24	0.2843	-0.06	-1.04	0.8445	-0.02	-1.02	0.9368
0.68	1.6	0.0499	0.57	1.48	0.1349	0.53	1.44	0.1382
0.1	1.07	0.759	0.13	1.1	0.6999	-0.02	-1.02	0.9422
-0.57	-1.49	0.2647	-0.43	-1.35	0.4228	0.19	1.14	0.7059
0.16	1.12	0.6305	-0.16	-1.12	0.6582	0.16	1.11	0.6401
-0.19	-1.14	0.5287	-0.35	-1.28	0.2863	-0.31	-1.24	0.312
0.11	1.08	0.7029	0.14	1.1	0.6714	0.01	1	0.9842
0.29	1.22	0.3913	-0.29	-1.22	0.4542	-0.03	-1.02	0.9291
0.14	1.1	0.6658	0.74	1.67	0.0279	0.18	1.13	0.5794
-0.42	-1.34	0.1679	-0.21	-1.16	0.5191	-0.25	-1.19	0.4128
0.25	1.19	0.4238	0.3	1.23	0.3747	0.3	1.24	0.3339
0.25	1.19	0.4198	0.54	1.45	0.1009	-0.08	-1.06	0.7999
-0.07	-1.05	0.8253	-0.03	-1.02	0.9251	0.03	1.02	0.9156
0.94	1.92	0.0657	-1.43	-2.69	0.0181	0.41	1.33	0.4285
0.36	1.28	0.2939	0.29	1.22	0.4334	0.17	1.13	0.6237
0.91	1.89	0.0221	-1.78	-3.43	0.001	0	1	0.9907
0.08	1.06	0.7966	-0.15	-1.11	0.6662	-0.07	-1.05	0.819
-0.05	-1.03	0.8864	-0.4	-1.32	0.2793	0.06	1.05	0.8393
-0.37	-1.29	0.2175	0.06	1.04	0.8399	-0.21	-1.16	0.4743
-1.3	-2.46	0.0106	-0.67	-1.59	0.2067	-0.99	-1.98	0.0501
0.11	1.08	0.733	-0.26	-1.2	0.4755	-0.06	-1.05	0.8462
0.66	1.58	0.0819	-0.6	-1.51	0.1959	0.18	1.13	0.6526
0.23	1.17	0.4995	-0.41	-1.33	0.3134	0.17	1.13	0.618

0.08	1.06	0.799	-0.16	-1.11	0.6594	0.1	1.07	0.7466
0.38	1.3	0.2398	0.1	1.07	0.7737	0.32	1.25	0.3315
0.23	1.17	0.472	-0.33	-1.26	0.3688	0.17	1.13	0.5968
-0.03	-1.02	0.9346	-0.16	-1.12	0.666	0.44	1.36	0.1895
0.84	1.79	0.2394	1.59	3.01	0.0282	-0.73	-1.66	0.3067
0.1	1.07	0.7774	-0.15	-1.11	0.6998	-0.04	-1.03	0.9002
0.44	1.35	0.2354	0	-1	0.9988	0.27	1.21	0.466
0.57	1.48	0.2101	-0.83	-1.78	0.1172	0.38	1.3	0.4119
0.09	1.07	0.7721	-0.3	-1.23	0.4091	-0.04	-1.03	0.9037
-0.22	-1.16	0.5275	-1.18	-2.27	0.0054	-0.13	-1.09	0.7064
-0.55	-1.46	0.114	-0.19	-1.14	0.6017	-0.22	-1.17	0.5101
0.35	1.27	0.3005	-0.31	-1.24	0.4326	0.13	1.1	0.7005
0.31	1.24	0.3875	0.73	1.66	0.0534	-0.05	-1.03	0.9043
-0.88	-1.84	0.0087	-0.34	-1.27	0.325	-0.34	-1.27	0.2844
0.02	1.01	0.959	-0.4	-1.32	0.3647	-0.08	-1.06	0.844
0.54	1.45	0.1532	0.35	1.27	0.4029	0.04	1.03	0.9266
-0.38	-1.3	0.2443	-0.82	-1.77	0.0295	-0.52	-1.43	0.1215
0.32	1.25	0.4301	0.11	1.08	0.7975	0.49	1.41	0.2316
-1.18	-2.26	0.0217	-0.02	-1.01	0.9682	-0.65	-1.57	0.2
-0.11	-1.08	0.7208	-0.31	-1.24	0.3706	0.03	1.02	0.9177
0.82	1.77	0.0532	-0.76	-1.69	0.1444	0.42	1.33	0.344
0.18	1.13	0.604	0.2	1.14	0.5993	0.22	1.17	0.5211
0.4	1.32	0.269	-0.26	-1.2	0.5314	0.16	1.12	0.673
0	-1	0.996	0.37	1.29	0.3238	-0.19	-1.14	0.6127
-0.06	-1.04	0.8546	-0.3	-1.23	0.4223	-0.21	-1.15	0.5509
0.2	1.15	0.5807	-0.07	-1.05	0.8712	0.2	1.15	0.5853
-0.06	-1.04	0.8669	-0.18	-1.13	0.6297	-0.21	-1.16	0.5428
-0.13	-1.09	0.5358	0.19	1.14	0.3877	-0.1	-1.07	0.6207
-0.22	-1.16	0.5312	0.26	1.2	0.4717	0.34	1.27	0.2999
0.02	1.01	0.9368	-0.01	-1	0.9839	0.16	1.11	0.5327
0.43	1.34	0.2461	0.77	1.71	0.0444	0.36	1.28	0.3333
0.02	1.01	0.9558	0.27	1.2	0.4847	0.01	1.01	0.9684
-0.29	-1.23	0.4036	0.08	1.06	0.8303	-0.17	-1.12	0.6325
0.52	1.44	0.1705	-0.69	-1.61	0.1395	0.35	1.27	0.376
0.26	1.2	0.5145	-0.59	-1.5	0.2082	-0.36	-1.29	0.3913
0.08	1.06	0.8211	-0.24	-1.18	0.5685	0.08	1.06	0.8201
-0.07	-1.05	0.928	0.07	1.05	0.9244	-0.56	-1.48	0.4353
1.28	2.43	0.0024	0.68	1.61	0.1479	0.26	1.2	0.5701
-0.11	-1.08	0.7674	-0.9	-1.86	0.0408	-0.26	-1.2	0.4793
-1.45	-2.72	1.00E-04	-0.38	-1.3	0.3072	-0.35	-1.28	0.3028
0.03	1.02	0.9381	-0.26	-1.2	0.524	0.09	1.06	0.808
-0.27	-1.21	0.4282	-0.49	-1.4	0.2083	0.08	1.05	0.8188
1.42	2.68	9.00E-04	0.17	1.13	0.7341	0.61	1.52	0.1863
0.21	1.15	0.5803	0.69	1.62	0.0737	0.06	1.05	0.8646
-0.44	-1.36	0.2004	0.04	1.03	0.9012	-0.15	-1.11	0.6507
-0.79	-1.73	0.0462	0.44	1.36	0.2642	-0.46	-1.37	0.2413
0.01	1.01	0.9765	0.22	1.16	0.569	-0.05	-1.04	0.8859

0.24	1.18	0.5318	0.17	1.13	0.6719	0.33	1.26	0.3794
0.75	1.68	0.0507	-0.05	-1.04	0.9066	0.49	1.4	0.2151
-0.02	-1.01	0.9547	-0.2	-1.15	0.6204	-0.02	-1.02	0.9454
-1.61	-3.04	0.004	-0.26	-1.2	0.6503	-0.44	-1.36	0.4155
0.65	1.57	0.1237	-0.3	-1.24	0.5343	0.52	1.43	0.2236
-0.15	-1.11	0.5799	0	-1	0.9954	-0.16	-1.11	0.5664
0.06	1.04	0.8732	-0.36	-1.28	0.4096	0.37	1.3	0.3141
-0.28	-1.22	0.4176	-0.19	-1.14	0.613	-0.14	-1.1	0.6822
0.67	1.59	0.147	-0.42	-1.34	0.4302	0.62	1.54	0.1824
0.88	1.84	0.0344	-0.44	-1.36	0.3832	0.29	1.22	0.5163
0.1	1.07	0.7857	-0.86	-1.82	0.0576	-0.18	-1.13	0.6454
-0.05	-1.04	0.9014	1.19	2.28	0.0056	-0.48	-1.4	0.2759
0.53	1.44	0.1557	0.38	1.3	0.3522	0.28	1.21	0.47
-0.16	-1.11	0.6673	-0.62	-1.54	0.137	-0.01	-1.01	0.9743
-0.18	-1.14	0.6115	0	-1	0.9982	-0.36	-1.29	0.3312
0.09	1.07	0.8188	0.3	1.23	0.4775	0.03	1.02	0.9504
0.02	1.01	0.9512	-0.65	-1.57	0.1197	0.01	1.01	0.973
0.02	1.02	0.9557	0.82	1.76	0.0555	-0.04	-1.03	0.9293
-0.25	-1.19	0.5009	-0.74	-1.67	0.0871	-0.23	-1.17	0.5458
-0.61	-1.52	0.4189	-0.05	-1.03	0.9475	-0.38	-1.3	0.609
-0.01	-1.01	0.9776	0.16	1.12	0.6693	-0.15	-1.11	0.679
0.75	1.68	0.0942	-0.64	-1.56	0.2307	0.63	1.55	0.165
-0.72	-1.65	0.0216	-0.04	-1.03	0.9121	-0.23	-1.17	0.4569
0.13	1.09	0.7362	0.14	1.1	0.7342	0.14	1.1	0.7111
0.52	1.43	0.3018	-1.24	-2.36	0.0409	0.56	1.47	0.2681
-0.03	-1.02	0.9336	0.27	1.21	0.5154	0.07	1.05	0.8626
0.43	1.35	0.2916	0.58	1.5	0.1781	0.43	1.35	0.2942
0.07	1.05	0.8602	-0.79	-1.73	0.0751	0.11	1.08	0.78
-0.24	-1.18	0.5527	-0.01	-1	0.989	-0.37	-1.29	0.3645
0.46	1.38	0.2762	0.42	1.34	0.3619	0.1	1.07	0.8182
0.82	1.77	0.0565	-0.63	-1.54	0.227	0.46	1.38	0.2972
-0.05	-1.03	0.9118	-0.66	-1.58	0.1688	0.34	1.26	0.4054
-0.26	-1.2	0.5446	-0.05	-1.04	0.9049	-0.11	-1.08	0.8043
-0.35	-1.27	0.3557	-0.51	-1.43	0.2256	0.01	1.01	0.9821
-0.72	-1.64	0.0582	0.17	1.13	0.6424	-0.52	-1.43	0.1653
0.22	1.16	0.5916	-0.73	-1.65	0.1378	0.14	1.11	0.7272
0.07	1.05	0.7439	0.5	1.41	0.0283	0.18	1.14	0.3986
0.32	1.25	0.4296	1.54	2.9	2.00E-04	-0.07	-1.05	0.8664
0.01	1	0.9886	-0.46	-1.37	0.3164	-0.04	-1.03	0.9117
0.1	1.08	0.775	-0.13	-1.1	0.7468	0.22	1.16	0.5548
0.02	1.02	0.9528	0.34	1.26	0.4242	-0.14	-1.1	0.7391
0.19	1.14	0.6037	-0.33	-1.26	0.4386	0.25	1.19	0.5006
0.18	1.13	0.6638	0.2	1.15	0.6435	0	-1	0.9995
0.31	1.24	0.4174	0.74	1.67	0.0663	0.13	1.1	0.7387
0.35	1.27	0.3899	0.66	1.58	0.1181	0.37	1.29	0.3635
-0.95	-1.93	0.0133	-0.42	-1.33	0.294	-0.24	-1.18	0.4993
-0.61	-1.53	0.1153	-0.11	-1.08	0.7825	-0.19	-1.14	0.6132

0.59	1.5	0.2828	0.01	1.01	0.9901	-0.47	-1.39	0.4168
0.2	1.15	0.6302	-0.62	-1.54	0.2075	0.26	1.19	0.5431
1.02	2.03	0.0147	0.56	1.47	0.2328	0.2	1.15	0.6641
0.28	1.21	0.4762	0.05	1.03	0.9093	0.08	1.06	0.8435
0.63	1.55	0.1386	-1.45	-2.74	0.0105	0.32	1.25	0.4683
0.1	1.07	0.8032	0.47	1.38	0.261	-0.1	-1.07	0.8063
1.08	2.11	0.0173	-0.01	-1.01	0.9786	0.51	1.42	0.2817
0.44	1.35	0.295	-0.17	-1.13	0.7204	0.1	1.07	0.825
0.22	1.16	0.5872	-0.36	-1.29	0.4399	-0.01	-1.01	0.9838
0.38	1.3	0.4968	0.44	1.36	0.4528	0	1	0.9954
-0.74	-1.66	0.0765	-0.09	-1.07	0.8249	-0.03	-1.02	0.9486
0.72	1.65	0.1196	0.41	1.32	0.4295	0.67	1.59	0.1566
0.07	1.05	0.8559	-0.2	-1.15	0.6477	-0.01	-1.01	0.9706
1.01	2.01	0.0514	-0.29	-1.22	0.6222	0.79	1.73	0.1317
0.4	1.32	0.3432	-0.82	-1.76	0.1127	0.05	1.04	0.9039
-0.28	-1.22	0.4786	0.47	1.39	0.2447	0.03	1.02	0.929
0.47	1.38	0.2627	0.64	1.56	0.1461	0.18	1.13	0.676
0.18	1.13	0.6947	0.37	1.29	0.4555	-0.15	-1.11	0.7512
-0.4	-1.32	0.3054	-0.04	-1.03	0.9225	-0.41	-1.33	0.3038
0.03	1.02	0.9491	-0.29	-1.22	0.5224	-0.44	-1.36	0.2915
-0.43	-1.35	0.2893	-0.01	-1.01	0.9856	-0.23	-1.17	0.5773
0.24	1.18	0.5542	0.67	1.59	0.1126	0.06	1.04	0.8929
-0.27	-1.2	0.4982	0.3	1.23	0.454	-0.09	-1.06	0.8269
1.06	2.08	0.0203	0.9	1.86	0.0683	0.18	1.14	0.7053
-0.48	-1.39	0.2308	-0.38	-1.3	0.3813	-0.29	-1.22	0.4695
0.24	1.18	0.5134	0.72	1.65	0.0589	-0.35	-1.28	0.3689
-0.1	-1.07	0.8053	0.55	1.46	0.1943	-0.14	-1.1	0.7396
0.11	1.08	0.8113	0.59	1.51	0.2068	0.06	1.04	0.8971
0.21	1.15	0.6117	-0.01	-1	0.9911	0.1	1.07	0.8162
-0.4	-1.32	0.3192	-0.8	-1.74	0.0831	0.26	1.2	0.4954
-0.53	-1.44	0.3523	-0.47	-1.38	0.4334	0.12	1.09	0.8269
0.06	1.04	0.8921	0.38	1.3	0.3857	0	1	0.9966
1.35	2.56	0.0048	0.29	1.22	0.6048	0.94	1.92	0.0583
-0.18	-1.14	0.6948	0.82	1.76	0.0834	0.23	1.17	0.6166
-0.24	-1.18	0.5482	-0.39	-1.31	0.384	0	-1	0.992
0.42	1.34	0.2632	0.48	1.39	0.238	0.47	1.38	0.2146
-0.58	-1.49	0.183	-0.25	-1.19	0.5812	-0.46	-1.38	0.2853
0.1	1.07	0.8171	-0.13	-1.09	0.7877	0.17	1.12	0.6949
0.41	1.33	0.3801	-0.64	-1.56	0.2385	0.33	1.26	0.4802
-0.17	-1.12	0.672	-0.32	-1.25	0.4676	-0.19	-1.14	0.6386
-0.14	-1.1	0.7614	-1.22	-2.33	0.0274	-0.41	-1.33	0.3886
0.25	1.19	0.6465	-1.22	-2.33	0.0544	0.21	1.16	0.7059
-0.04	-1.02	0.9489	-1.36	-2.57	0.027	-0.49	-1.4	0.3869
-0.18	-1.13	0.6687	-0.32	-1.25	0.4981	0.33	1.26	0.4174
0.43	1.35	0.3026	-0.01	-1.01	0.9861	0.18	1.13	0.6789
-0.86	-1.82	0.0432	-0.73	-1.66	0.1121	-0.48	-1.4	0.2423
-0.06	-1.04	0.8854	-0.83	-1.78	0.1104	0.25	1.19	0.556

-0.26	-1.2	0.5378	0.21	1.16	0.6352	0.28	1.21	0.498
-0.08	-1.06	0.8439	-0.65	-1.57	0.1602	0.15	1.11	0.7015
-0.07	-1.05	0.8795	0.46	1.38	0.313	-0.55	-1.47	0.2403
0.16	1.12	0.721	0.08	1.06	0.8726	-0.22	-1.16	0.6382
0.3	1.23	0.514	-0.09	-1.06	0.8661	0.13	1.1	0.7786
0.77	1.7	0.1015	0.99	1.99	0.0432	0.19	1.14	0.7079
-0.63	-1.54	0.1265	-0.69	-1.61	0.1281	0.03	1.02	0.9325
0.51	1.43	0.2572	0.18	1.13	0.7259	-0.11	-1.08	0.8164
0.82	1.76	0.0601	0.56	1.47	0.2422	0.08	1.06	0.8591
-0.34	-1.27	0.4822	0.17	1.13	0.7317	0.06	1.04	0.9013
-0.41	-1.33	0.3372	-0.61	-1.52	0.201	0.01	1.01	0.9733
-0.01	-1	0.9917	-0.1	-1.07	0.8582	0.21	1.16	0.6734
0.33	1.25	0.6346	-0.03	-1.02	0.9642	0.88	1.84	0.1996
0.48	1.4	0.2852	0.33	1.26	0.499	0.02	1.01	0.9691
-0.52	-1.43	0.1812	-0.53	-1.44	0.2096	-0.4	-1.32	0.2973
0.32	1.24	0.4727	-0.12	-1.08	0.8133	0.28	1.22	0.5282
0.42	1.34	0.3487	-0.19	-1.14	0.7165	0.03	1.02	0.9489
-0.95	-1.93	0.0784	-0.18	-1.13	0.7429	0.68	1.61	0.1742
0.31	1.24	0.47	0.02	1.01	0.966	-0.06	-1.04	0.8962
-0.21	-1.15	0.6419	0.02	1.01	0.9739	-0.35	-1.27	0.4386
0.82	1.77	0.104	-0.59	-1.5	0.3378	0.13	1.1	0.8053
0.34	1.26	0.4778	0.24	1.18	0.6394	0.32	1.25	0.497
-0.14	-1.1	0.7316	0.57	1.48	0.1828	-0.09	-1.06	0.8358
0.7	1.62	0.1449	0.19	1.14	0.7279	0.39	1.31	0.4321
-0.69	-1.61	0.0911	-0.37	-1.29	0.3936	-0.37	-1.29	0.3525
-1.89	-3.69	1.00E-04	-0.92	-1.89	0.0497	-0.64	-1.56	0.1308
0.17	1.13	0.6949	0.05	1.04	0.9079	0.41	1.33	0.3402
0.02	1.01	0.9651	-0.66	-1.58	0.2027	0.06	1.04	0.8981
-0.1	-1.07	0.8264	0.27	1.2	0.5842	0.48	1.4	0.287
0.11	1.08	0.8033	0.49	1.4	0.2893	-0.31	-1.24	0.5018
0.09	1.06	0.8102	0.51	1.43	0.1774	0.16	1.12	0.6607
0.02	1.01	0.9656	0.47	1.38	0.3019	-0.1	-1.07	0.8297
0.95	1.93	0.0514	-0.06	-1.04	0.9194	0.21	1.16	0.6861
-0.59	-1.5	0.184	-0.18	-1.13	0.6928	-0.03	-1.02	0.946
-0.07	-1.05	0.8765	-0.1	-1.07	0.8357	0.41	1.33	0.3536
1	2.01	0.0454	-0.35	-1.27	0.5587	0.6	1.51	0.2503
-0.38	-1.3	0.3733	-0.32	-1.25	0.4906	-0.23	-1.17	0.585
-0.75	-1.68	0.0871	-0.53	-1.44	0.2591	-0.1	-1.07	0.8118
-0.4	-1.32	0.3514	-0.84	-1.79	0.088	-0.17	-1.12	0.6955
0.02	1.02	0.9661	1.19	2.27	0.0276	-0.21	-1.16	0.7017
0.53	1.44	0.3178	-0.62	-1.54	0.3036	0.57	1.48	0.2863
-0.79	-1.73	0.1226	0.1	1.07	0.8496	-0.41	-1.33	0.4149
0.03	1.02	0.938	0.39	1.31	0.4023	0.24	1.18	0.5787
0.57	1.48	0.257	0.46	1.38	0.3958	0.37	1.29	0.4743
-0.4	-1.32	0.4091	-0.91	-1.88	0.097	-0.44	-1.35	0.3748
-0.92	-1.9	0.0673	0.06	1.04	0.9038	-0.4	-1.32	0.4183
0.41	1.33	0.3975	-0.08	-1.06	0.8804	-0.04	-1.03	0.9383

0.32	1.25	0.4952	0.1	1.07	0.8481	0.33	1.26	0.4908
-0.15	-1.11	0.6961	0.92	1.9	0.013	-0.36	-1.28	0.3526
-0.2	-1.15	0.6665	0.2	1.15	0.676	-0.15	-1.11	0.7491
-0.26	-1.2	0.5473	-0.34	-1.26	0.4786	-0.23	-1.17	0.6067
-0.32	-1.25	0.4922	-1.24	-2.37	0.0244	0.03	1.02	0.9398
0	-1	0.9921	-0.64	-1.55	0.258	0.04	1.03	0.9324
-0.04	-1.03	0.9234	0.36	1.28	0.4473	-0.21	-1.16	0.6562
0.3	1.23	0.5062	0.26	1.2	0.5905	0.36	1.29	0.4216
0.33	1.26	0.5013	0.31	1.24	0.5571	0.04	1.03	0.9334
-0.66	-1.58	0.2255	-1.24	-2.37	0.0384	-0.69	-1.61	0.2075
0.19	1.14	0.7278	0.94	1.92	0.0798	0.44	1.36	0.406
0.5	1.41	0.2986	-0.77	-1.7	0.1918	0.39	1.31	0.4286
0.53	1.45	0.2484	-0.66	-1.58	0.2364	0.74	1.67	0.1066
-0.54	-1.46	0.2256	-0.67	-1.59	0.177	-0.19	-1.14	0.6662
-0.2	-1.15	0.645	0.02	1.01	0.968	-0.38	-1.3	0.4041
0.34	1.27	0.4586	0.62	1.53	0.2055	-0.12	-1.09	0.8091
-0.2	-1.15	0.643	-0.02	-1.01	0.9637	-0.09	-1.07	0.8316
-0.39	-1.31	0.3751	-0.03	-1.02	0.9416	-0.16	-1.12	0.7126
0.47	1.39	0.3264	0.5	1.42	0.3327	0.11	1.08	0.8267
-0.65	-1.57	0.1448	-0.2	-1.15	0.663	0.04	1.03	0.9294
0.53	1.45	0.1524	-0.63	-1.55	0.174	0.06	1.04	0.8791
0.11	1.08	0.8176	0.4	1.32	0.4065	-0.64	-1.56	0.2048
0.48	1.39	0.2922	-0.97	-1.96	0.0817	-0.17	-1.12	0.7282
1.39	2.62	0.011	0.24	1.18	0.6951	0.81	1.75	0.1559
-0.34	-1.26	0.4767	0.24	1.18	0.6128	0.11	1.08	0.8147
0.07	1.05	0.8717	-0.23	-1.17	0.6635	0.01	1.01	0.9829
0.19	1.14	0.6837	-0.26	-1.19	0.6223	0.6	1.51	0.1827
0.32	1.25	0.4905	0.35	1.27	0.4869	0.03	1.02	0.9557
-0.2	-1.15	0.6612	0.37	1.29	0.426	-0.98	-1.98	0.0483
0.09	1.07	0.8491	-0.2	-1.15	0.7159	-0.28	-1.21	0.5923
-0.51	-1.42	0.3594	-0.93	-1.9	0.1277	0.22	1.16	0.6848
-1.44	-2.71	0.0024	-0.83	-1.78	0.0897	-0.63	-1.55	0.1667
0.62	1.53	0.2972	-1.19	-2.28	0.0884	0.18	1.13	0.7714
0.73	1.66	0.1767	1.05	2.07	0.0615	0.51	1.43	0.3545
-0.29	-1.22	0.5362	0.52	1.43	0.2754	-0.64	-1.55	0.1961
-0.65	-1.57	0.2139	0.13	1.1	0.802	0.2	1.15	0.688
-0.34	-1.27	0.4618	-1.07	-2.09	0.0489	0.03	1.02	0.9499
-0.32	-1.25	0.5351	-0.02	-1.01	0.9746	0.23	1.17	0.65
0.23	1.18	0.6231	-0.13	-1.09	0.8104	0.08	1.06	0.8647
0.28	1.21	0.5655	0.4	1.32	0.44	0.09	1.06	0.8598
0.27	1.2	0.545	-0.45	-1.37	0.3751	0.25	1.19	0.5735
-0.39	-1.31	0.3984	-0.46	-1.37	0.3612	-0.14	-1.1	0.7588
0.01	1.01	0.9757	-0.16	-1.12	0.7498	0.24	1.18	0.5915
1.71	3.28	0.0018	0.2	1.15	0.7569	0.85	1.8	0.1461
0.09	1.07	0.853	0.49	1.4	0.3479	0.06	1.04	0.9031
0.14	1.1	0.6539	-0.78	-1.72	0.0325	0.17	1.13	0.5735
-1.21	-2.32	0.0123	-0.27	-1.21	0.5698	-0.49	-1.41	0.2763

-0.98	-1.97	0.0354	-0.21	-1.15	0.6623	-0.92	-1.89	0.0508
1.73	3.31	0.0022	1.37	2.58	0.0237	0.74	1.66	0.2229
0.78	1.72	0.1454	0.05	1.04	0.9299	0.44	1.35	0.4277
-0.17	-1.12	0.7117	0.05	1.03	0.9184	-0.06	-1.04	0.9034
-1.4	-2.65	0.0034	-0.62	-1.54	0.1986	-0.54	-1.45	0.2247
0.13	1.09	0.8028	-0.47	-1.38	0.4201	0.37	1.29	0.4671
0.34	1.26	0.4909	0.7	1.62	0.1745	0.19	1.14	0.7125
0.72	1.65	0.1967	-1.12	-2.18	0.0911	-0.48	-1.4	0.4253
-0.68	-1.6	0.1629	-0.77	-1.71	0.1468	-0.36	-1.28	0.4564
0.04	1.03	0.9284	0.28	1.21	0.5862	0.02	1.02	0.9646
-0.55	-1.46	0.2689	-0.62	-1.54	0.2516	0.05	1.04	0.9154
-0.05	-1.03	0.9369	-1.1	-2.15	0.0847	0.29	1.22	0.6147
-0.44	-1.36	0.3939	0.1	1.07	0.8591	-0.37	-1.29	0.4833
0.8	1.74	0.1188	0.39	1.31	0.4923	0.73	1.66	0.1552
0.31	1.24	0.5991	-0.02	-1.01	0.9745	-0.32	-1.25	0.6047
-0.98	-1.98	0.0874	-0.3	-1.23	0.6099	0.92	1.89	0.0812
-0.49	-1.4	0.2686	-0.23	-1.17	0.6265	-0.18	-1.13	0.6817
0.14	1.1	0.774	0.48	1.4	0.3453	0.11	1.08	0.8302
0	1	1	-0.31	-1.24	0.5681	-0.74	-1.67	0.1582
0.34	1.27	0.5148	0.82	1.77	0.1252	0.06	1.04	0.9188
-0.22	-1.16	0.6896	-0.4	-1.32	0.5023	0.26	1.19	0.6323
-0.91	-1.88	0.1107	-0.84	-1.8	0.1632	-0.59	-1.51	0.2945
-0.17	-1.13	0.7267	-0.15	-1.11	0.7749	-0.11	-1.08	0.8244
-0.57	-1.48	0.2586	-0.84	-1.8	0.1303	-0.85	-1.8	0.1049
-0.14	-1.1	0.7814	0.31	1.24	0.5566	-0.14	-1.1	0.7879
0.31	1.24	0.5514	-0.04	-1.03	0.9419	0.04	1.03	0.943
1.11	2.15	0.0343	-0.29	-1.22	0.6401	0.52	1.43	0.3439
-0.27	-1.2	0.5585	-0.16	-1.11	0.7512	0.06	1.05	0.8869
-0.58	-1.5	0.2762	-0.52	-1.43	0.3672	0.09	1.06	0.8594
-0.41	-1.33	0.4068	0.72	1.65	0.1425	0.01	1	0.9906
-0.59	-1.51	0.2157	-0.44	-1.35	0.3938	-0.11	-1.08	0.8081
0.12	1.09	0.8	-0.29	-1.23	0.5945	0.02	1.01	0.9701
-0.08	-1.06	0.8767	-0.01	-1	0.9899	-0.04	-1.03	0.9355
-0.31	-1.24	0.5413	-0.83	-1.77	0.1512	-0.23	-1.17	0.6525
-0.16	-1.12	0.7596	0.19	1.14	0.7259	-0.08	-1.06	0.8725
0.51	1.43	0.3889	0.37	1.29	0.5577	0.4	1.32	0.507
0.12	1.09	0.82	0.4	1.32	0.4729	0.12	1.09	0.8174
-0.63	-1.54	0.1847	-0.79	-1.73	0.1308	-0.46	-1.37	0.3277
0.05	1.03	0.919	-0.26	-1.2	0.6294	-0.18	-1.13	0.7271
-0.06	-1.04	0.9101	-0.96	-1.94	0.1074	-0.56	-1.48	0.2987
1.16	2.24	0.0501	0.82	1.76	0.2005	0.52	1.44	0.4044
-0.22	-1.17	0.7333	0.88	1.84	0.1887	-0.22	-1.16	0.7412
-1.34	-2.53	0.0282	0.98	1.97	0.0835	-1.12	-2.17	0.0652
0.18	1.13	0.7339	0.21	1.16	0.7048	-0.23	-1.17	0.6757
0.21	1.16	0.6727	0.31	1.24	0.5588	0.36	1.28	0.4724
0.45	1.37	0.3898	-0.25	-1.19	0.6801	0.05	1.03	0.9319
0.5	1.41	0.4538	-0.65	-1.57	0.3581	0.4	1.32	0.5515

-0.32	-1.25	0.5562	-1.38	-2.6	0.0243	-0.13	-1.1	0.807
-0.56	-1.48	0.2981	-0.23	-1.17	0.6888	-0.38	-1.3	0.483
0.54	1.46	0.2691	0.7	1.62	0.1785	-0.21	-1.16	0.6887
-0.68	-1.6	0.176	-0.21	-1.16	0.683	-0.37	-1.29	0.4534
-0.08	-1.05	0.8848	0.55	1.46	0.3034	-0.6	-1.51	0.2769
0.49	1.4	0.3542	0.68	1.61	0.2187	0.23	1.17	0.671
-0.71	-1.63	0.1732	-0.34	-1.27	0.5292	-0.59	-1.51	0.2527
-0.48	-1.39	0.392	0.6	1.51	0.286	-0.45	-1.37	0.4175
-0.86	-1.81	0.0955	-0.58	-1.49	0.2865	-0.11	-1.08	0.8146
-0.17	-1.13	0.7648	0.79	1.73	0.1737	0.37	1.29	0.5146
-0.11	-1.08	0.8127	-0.25	-1.19	0.6304	-0.22	-1.16	0.6507
0.39	1.31	0.4291	-0.47	-1.38	0.4202	0.12	1.08	0.8194
0.23	1.17	0.6363	-0.86	-1.82	0.1393	-0.14	-1.1	0.7809
0.13	1.1	0.8113	0.72	1.65	0.2015	-0.06	-1.04	0.9225
-0.27	-1.21	0.5889	-0.14	-1.1	0.7916	-0.05	-1.03	0.9232
-0.43	-1.35	0.4407	-1.19	-2.28	0.0565	-0.51	-1.42	0.3693
0.3	1.23	0.5498	-0.01	-1.01	0.9897	0.27	1.21	0.595
0.1	1.07	0.8467	0.3	1.23	0.5961	-0.48	-1.39	0.3995
-0.76	-1.69	0.16	-0.07	-1.05	0.8952	-0.22	-1.16	0.6764
1.82	3.53	0.0044	1.17	2.24	0.0868	0.53	1.45	0.4312
-0.67	-1.59	0.2025	-0.57	-1.49	0.3091	-0.05	-1.03	0.925
0.66	1.58	0.2331	-1.09	-2.12	0.1021	0.27	1.2	0.6409
-0.23	-1.17	0.6691	-1.64	-3.12	0.0106	0.03	1.02	0.962
0	-1	0.9988	-0.08	-1.06	0.8885	0.1	1.07	0.8397
-1.61	-3.04	0.0028	-0.7	-1.62	0.1966	-1.14	-2.2	0.0298
0.76	1.69	0.2001	0	-1	0.9957	0.68	1.6	0.2583
1.01	2.01	0.1205	0.14	1.1	0.8446	0.35	1.28	0.6002
0.95	1.94	0.085	0.13	1.1	0.83	0.32	1.25	0.5818
0.92	1.9	0.1516	-1.11	-2.15	0.1232	0.28	1.22	0.6693
0.49	1.41	0.3469	0.31	1.24	0.5875	0.22	1.16	0.6855
0.86	1.82	0.1593	0.95	1.93	0.1394	0.13	1.1	0.8388
0.16	1.11	0.7799	0.39	1.31	0.5035	0.37	1.3	0.4991
-0.47	-1.38	0.3682	0.12	1.09	0.8212	-0.1	-1.07	0.8416
-0.57	-1.49	0.2759	0.16	1.12	0.759	-0.29	-1.22	0.577
-0.55	-1.47	0.3035	-0.13	-1.1	0.8091	0.02	1.02	0.9655
-0.72	-1.65	0.1661	-0.75	-1.68	0.182	-0.12	-1.08	0.8162
-0.44	-1.36	0.4165	-0.28	-1.21	0.628	-0.04	-1.03	0.9435
1.02	2.03	0.0826	0.5	1.41	0.4389	-0.05	-1.03	0.9393
0.08	1.05	0.9105	0.23	1.18	0.7244	-0.08	-1.06	0.9021
0.68	1.6	0.2187	0.9	1.86	0.1199	-0.32	-1.25	0.5951
-0.82	-1.77	0.14	-0.22	-1.16	0.6997	0.23	1.17	0.6639
-0.08	-1.06	0.8818	-1.28	-2.43	0.0501	-0.17	-1.12	0.7706
-0.16	-1.12	0.7512	-0.79	-1.73	0.1694	-0.2	-1.15	0.6938
-0.23	-1.17	0.6717	0.31	1.24	0.5779	-0.11	-1.08	0.8351
-0.72	-1.65	0.2079	-0.77	-1.7	0.2125	-0.07	-1.05	0.8961
-1.46	-2.74	0.0218	-0.22	-1.17	0.722	0.93	1.9	0.1103
0.33	1.26	0.5741	-0.13	-1.1	0.8323	0.2	1.15	0.7345

-0.32	-1.25	0.5663	-0.74	-1.67	0.2306	0	1	0.9969
1.87	3.66	0.008	0.89	1.86	0.2316	-0.03	-1.02	0.9729
0.25	1.19	0.6754	-1.16	-2.24	0.0882	-0.03	-1.02	0.9656
0.04	1.02	0.9474	0.66	1.58	0.2251	-0.12	-1.09	0.8248
0.56	1.48	0.3109	-0.17	-1.13	0.7858	0.18	1.13	0.7558
-0.82	-1.77	0.1215	0.41	1.33	0.4202	-0.1	-1.07	0.8355
-0.75	-1.68	0.1619	0.23	1.17	0.6645	-0.57	-1.48	0.2902
-0.37	-1.3	0.4713	-0.21	-1.16	0.7038	-0.24	-1.18	0.6469
0.2	1.15	0.7391	0.82	1.76	0.1825	0.65	1.57	0.2683
-0.51	-1.42	0.3723	-0.09	-1.07	0.8716	-0.02	-1.01	0.9752
0.85	1.8	0.1636	0.16	1.12	0.8051	0.22	1.17	0.7254
0.19	1.14	0.7478	-0.88	-1.84	0.1806	0.02	1.01	0.9792
-0.16	-1.12	0.7756	-1.95	-3.87	0.0033	-0.21	-1.15	0.7106
0.77	1.7	0.2339	-0.43	-1.34	0.5507	0.74	1.67	0.2572
-1.74	-3.35	0.0061	-0.46	-1.37	0.4658	0.54	1.45	0.3493
0.38	1.3	0.49	0.02	1.02	0.9713	0.14	1.1	0.8049
-0.41	-1.33	0.2532	-0.03	-1.02	0.9396	0.22	1.17	0.5189
-1.12	-2.18	0.0404	-1.83	-3.56	0.0034	-0.37	-1.3	0.4723
-1.02	-2.02	0.0585	-1.26	-2.4	0.0321	-0.34	-1.27	0.5033
0.86	1.82	0.127	0.24	1.18	0.7019	0.76	1.69	0.1855
0.35	1.28	0.5326	-0.49	-1.4	0.4454	-0.17	-1.13	0.7689
0.03	1.02	0.9505	-0.19	-1.14	0.7548	0.37	1.29	0.4954
0.52	1.43	0.309	-0.07	-1.05	0.9091	-0.15	-1.11	0.7889
0.45	1.37	0.417	-0.26	-1.2	0.6789	0.5	1.42	0.3695
-0.67	-1.59	0.2268	-0.32	-1.25	0.5799	-0.16	-1.11	0.7728
-0.04	-1.03	0.9357	0.23	1.17	0.6855	0.29	1.22	0.5957
0.33	1.26	0.6576	0.34	1.26	0.651	-0.5	-1.42	0.5006
0.9	1.87	0.1474	0.96	1.94	0.1428	0.29	1.23	0.6517
-1.09	-2.12	0.0867	0.16	1.12	0.7994	0.17	1.13	0.7671
-0.88	-1.84	0.1012	-0.14	-1.1	0.8045	-0.42	-1.33	0.426
0.15	1.11	0.8098	-0.07	-1.05	0.9128	0.47	1.38	0.4453
0.14	1.11	0.8022	0.6	1.52	0.3112	0.17	1.12	0.7696
0.65	1.57	0.3036	-1.34	-2.54	0.0576	0.03	1.02	0.9609
-0.64	-1.56	0.303	0.25	1.19	0.6959	0.29	1.23	0.6242
0.27	1.21	0.6211	0.4	1.32	0.4947	-0.03	-1.02	0.9511
0.06	1.04	0.915	0.6	1.52	0.3145	-0.42	-1.34	0.4927
-0.12	-1.09	0.8382	0.99	1.99	0.0833	0.25	1.19	0.6549
1.1	2.15	0.111	1.1	2.14	0.1244	0.73	1.66	0.2999
-0.13	-1.1	0.8264	-0.76	-1.69	0.2554	0.16	1.12	0.791
0.98	1.98	0.0978	-0.17	-1.13	0.7968	-0.03	-1.02	0.9674
0.89	1.85	0.1592	0.21	1.15	0.7613	1.11	2.16	0.078
0.39	1.31	0.5762	-0.3	-1.23	0.6806	0.56	1.48	0.4196
0.15	1.11	0.7855	0.01	1.01	0.9807	0.43	1.35	0.4343
0.98	1.97	0.1021	0.8	1.75	0.2068	0.13	1.09	0.8445
0.92	1.89	0.1015	0.96	1.95	0.1024	0.33	1.26	0.5769
0.44	1.36	0.464	-0.09	-1.06	0.8925	0.44	1.35	0.4713
-0.96	-1.94	0.0867	-1.27	-2.42	0.0383	-0.18	-1.13	0.7392

-0.76	-1.7	0.1563	-0.55	-1.46	0.3336	-0.33	-1.26	0.5233
-0.11	-1.08	0.8421	-0.14	-1.1	0.8096	-0.32	-1.25	0.5751
-0.68	-1.6	0.2272	-0.16	-1.12	0.7815	-0.6	-1.51	0.2926
-1.12	-2.18	0.0544	-0.15	-1.11	0.7992	-0.47	-1.39	0.3999
0.74	1.67	0.2117	-0.54	-1.46	0.4237	-0.19	-1.14	0.7637
0.77	1.71	0.2254	0.48	1.39	0.4817	-0.06	-1.04	0.9336
0.35	1.27	0.4593	-0.09	-1.06	0.8616	0.19	1.14	0.6818
0.59	1.5	0.3488	-0.17	-1.13	0.7971	0.76	1.69	0.2251
0.29	1.22	0.6204	0.7	1.63	0.2475	-0.03	-1.02	0.9557
-0.14	-1.1	0.5845	0.31	1.24	0.2336	0.15	1.11	0.5373
-0.54	-1.45	0.3611	0.15	1.11	0.8083	-0.44	-1.36	0.454
-0.15	-1.11	0.8009	-0.4	-1.32	0.5304	-0.17	-1.12	0.7779
-0.36	-1.29	0.5511	0.59	1.51	0.3257	-0.41	-1.33	0.5029
-0.63	-1.54	0.2698	-0.8	-1.74	0.1911	0.07	1.05	0.8922
-0.09	-1.06	0.8905	0.15	1.11	0.8149	0.27	1.21	0.6604
-1	-2.01	0.071	-1.23	-2.34	0.0429	-0.25	-1.19	0.6414
0.78	1.71	0.2836	1.5	2.82	0.035	0.59	1.5	0.4171
0.32	1.25	0.5667	0.69	1.62	0.2373	0.44	1.36	0.4392
-0.55	-1.46	0.4168	-1.11	-2.15	0.1187	-0.67	-1.59	0.326
-0.38	-1.3	0.4695	-0.6	-1.51	0.2979	-0.2	-1.15	0.6965
-0.67	-1.59	0.2241	-0.14	-1.11	0.7972	-0.64	-1.55	0.2493
-1.01	-2.01	0.0626	-1.52	-2.88	0.0121	-0.39	-1.31	0.4499
-0.27	-1.21	0.6513	-0.29	-1.23	0.6471	0.3	1.23	0.6149
0.51	1.43	0.4205	-0.64	-1.56	0.363	1.09	2.13	0.0796
-0.12	-1.09	0.8363	0.27	1.21	0.6581	0.08	1.06	0.8864
-0.13	-1.1	0.8246	-0.46	-1.37	0.4763	0.15	1.11	0.7966
-0.83	-1.77	0.1747	-1.11	-2.16	0.0907	0.04	1.03	0.9387
-0.73	-1.66	0.1939	-0.6	-1.52	0.3099	-0.27	-1.21	0.6163
0.37	1.29	0.5461	-0.96	-1.95	0.1682	-0.04	-1.03	0.9484
0.17	1.12	0.7654	-1.35	-2.55	0.0432	0.03	1.02	0.9534
-0.14	-1.1	0.8106	-1.05	-2.07	0.1061	-1.27	-2.42	0.0435
0.14	1.1	0.8086	0.15	1.11	0.8062	-0.14	-1.1	0.8186
0.18	1.13	0.7487	-1.32	-2.49	0.0427	0.42	1.34	0.4375
0.62	1.54	0.3159	-0.4	-1.32	0.5608	-0.05	-1.03	0.9415
-0.46	-1.38	0.4474	-0.41	-1.33	0.5192	-0.28	-1.21	0.6496
-0.08	-1.05	0.8965	0.26	1.2	0.6661	0.31	1.24	0.5848
0.12	1.09	0.8354	-0.15	-1.11	0.8133	-0.55	-1.46	0.373
-0.76	-1.7	0.198	-0.18	-1.13	0.7685	-0.45	-1.36	0.444
-0.23	-1.17	0.6931	-0.4	-1.32	0.5208	-0.16	-1.12	0.7829
-1	-2	0.0776	-1.63	-3.1	0.0105	-0.33	-1.26	0.5444
0.17	1.12	0.7906	-0.66	-1.58	0.3373	0.16	1.12	0.8035
0.51	1.43	0.3871	-0.46	-1.38	0.4897	0.17	1.12	0.7849
1.56	2.95	0.0156	1.09	2.12	0.1146	0.53	1.45	0.4376
-0.96	-1.94	0.0967	-1.1	-2.15	0.076	-0.47	-1.39	0.3985
-1.22	-2.32	0.0396	-1.55	-2.94	0.0156	-0.09	-1.06	0.8695
0	-1	0.9968	-0.56	-1.48	0.3966	-0.39	-1.31	0.5336
-0.71	-1.63	0.2225	0.39	1.31	0.5033	-0.6	-1.52	0.2999

-1.4	-2.63	0.0169	-0.76	-1.7	0.2011	-0.38	-1.3	0.4897
-0.05	-1.03	0.9332	-0.28	-1.22	0.6491	0.16	1.11	0.7847
-0.26	-1.2	0.6523	0.56	1.47	0.3405	-0.25	-1.19	0.6675
-0.33	-1.26	0.482	0.28	1.21	0.5666	-0.11	-1.08	0.8193
-0.4	-1.32	0.1774	-0.02	-1.01	0.9592	-0.13	-1.1	0.6473
-0.85	-1.8	0.1373	-0.44	-1.36	0.4536	-0.19	-1.14	0.7277
0.41	1.33	0.5049	-0.54	-1.46	0.4274	-0.05	-1.03	0.9403
0.1	1.07	0.8755	-0.26	-1.2	0.706	-0.46	-1.37	0.4955
-0.44	-1.36	0.4636	-0.3	-1.23	0.6326	0.13	1.1	0.8167
-1.59	-3.01	0.0072	-1.22	-2.34	0.0462	-0.62	-1.53	0.2688
-1.15	-2.23	0.0825	-0.35	-1.27	0.6028	-0.41	-1.33	0.53
-0.85	-1.8	0.1399	-0.6	-1.52	0.3158	0.11	1.08	0.8324
-0.45	-1.37	0.4486	-0.96	-1.94	0.1441	-0.81	-1.76	0.1889
0.49	1.41	0.4186	0.49	1.41	0.4426	0.51	1.42	0.4086
0.57	1.49	0.3789	-0.37	-1.29	0.6024	0.11	1.08	0.873
1.21	2.32	0.0693	-0.12	-1.09	0.8633	-0.24	-1.18	0.7339
-0.43	-1.35	0.5667	-0.14	-1.1	0.8542	-0.1	-1.07	0.8984
-1.01	-2.01	0.0874	-0.8	-1.74	0.1982	-0.38	-1.3	0.5019
-0.09	-1.06	0.8992	0.34	1.26	0.6262	0.17	1.13	0.7995
-0.2	-1.15	0.7354	-1.24	-2.36	0.0643	-0.2	-1.15	0.7385
0.78	1.72	0.2506	0.54	1.46	0.4463	0.55	1.46	0.4251
-0.75	-1.68	0.2436	0.39	1.31	0.539	0.17	1.12	0.7828
0.64	1.56	0.3311	0.07	1.05	0.9224	1.07	2.1	0.1006
1.04	2.05	0.1183	-0.81	-1.76	0.2647	-0.19	-1.14	0.7878
-0.51	-1.42	0.4649	-0.72	-1.65	0.3165	0.01	1.01	0.9841
-0.15	-1.11	0.737	0.37	1.29	0.414	0	-1	0.9914
0.48	1.39	0.437	-0.13	-1.09	0.8481	0.08	1.05	0.9033
0.67	1.59	0.2832	-0.42	-1.34	0.5432	-0.03	-1.02	0.9629
-0.28	-1.21	0.6024	0.02	1.02	0.9693	0.26	1.2	0.6152
-1.1	-2.14	0.1127	-0.58	-1.5	0.4092	-0.57	-1.49	0.4023
-0.96	-1.95	0.1934	-0.3	-1.24	0.6823	-0.23	-1.17	0.7524
0.39	1.31	0.5665	0.08	1.06	0.9126	0.17	1.12	0.809
1.95	3.87	0.007	0.84	1.79	0.2613	1.16	2.24	0.116
-1.08	-2.11	0.0697	-1.77	-3.41	0.0071	-0.15	-1.11	0.7947
0.3	1.23	0.6749	0.24	1.18	0.7475	-0.66	-1.58	0.3696
0.18	1.13	0.7896	0.49	1.4	0.4723	-0.39	-1.31	0.5645
0.74	1.66	0.2435	0.17	1.12	0.8035	0.12	1.09	0.8561
0.76	1.7	0.2677	0.3	1.23	0.6768	0.37	1.3	0.5968
-1.28	-2.44	0.046	0.23	1.18	0.7091	0	-1	0.9972
1.06	2.09	0.1463	-0.04	-1.03	0.9611	-0.62	-1.54	0.4066
-1.55	-2.92	0.0179	-1.26	-2.4	0.06	-0.43	-1.35	0.4873
0.12	1.08	0.8613	-0.81	-1.75	0.2565	0.07	1.05	0.9218
-1.16	-2.23	0.066	-1.22	-2.34	0.0647	-0.67	-1.59	0.277
-0.15	-1.11	0.8094	0.23	1.18	0.7124	-0.59	-1.51	0.3553
0.66	1.57	0.3168	-0.09	-1.06	0.9027	0.55	1.46	0.4104
-0.22	-1.17	0.7345	-0.31	-1.24	0.6495	0.15	1.11	0.8116
0.65	1.57	0.3299	-0.08	-1.06	0.9085	0.76	1.69	0.256

-0.35	-1.27	0.6253	-0.67	-1.59	0.3573	-0.78	-1.72	0.277
1.64	3.11	0.0176	-0.04	-1.03	0.9597	0.41	1.33	0.575
-1.78	-3.42	0.0092	-1.02	-2.03	0.1373	-0.9	-1.87	0.1745
1.44	2.72	0.0432	0.81	1.76	0.2742	-0.03	-1.02	0.968
0.52	1.43	0.4347	-0.05	-1.03	0.9441	-0.99	-1.98	0.1638
1.06	2.08	0.1529	0.22	1.17	0.7646	0.16	1.11	0.834
0.75	1.68	0.2644	-1.11	-2.15	0.1267	0.23	1.17	0.7362
-0.3	-1.23	0.6223	0.19	1.14	0.7628	-0.81	-1.76	0.1945
-1.15	-2.22	0.0756	-0.24	-1.18	0.7154	-0.72	-1.65	0.2557
-1.72	-3.29	0.01	-0.82	-1.77	0.2166	-1.48	-2.8	0.0251
-1.89	-3.71	0.0096	0.55	1.46	0.4435	-0.68	-1.61	0.3391
1.45	2.74	0.0372	0.28	1.22	0.704	1	2.01	0.1571
-1.35	-2.55	0.06	0.12	1.09	0.8611	0.31	1.24	0.6548
0.07	1.05	0.9157	0.51	1.42	0.4723	0.17	1.13	0.8022
-0.32	-1.25	0.5857	-0.79	-1.72	0.2203	-0.75	-1.68	0.2208
0.77	1.7	0.2678	0.32	1.25	0.6632	0.37	1.29	0.6062
-0.43	-1.35	0.5088	-0.33	-1.25	0.6318	0.57	1.48	0.3678
-0.85	-1.81	0.1863	-1.3	-2.46	0.0588	-0.39	-1.31	0.5348
-0.26	-1.2	0.7208	-1.53	-2.89	0.0393	-0.49	-1.41	0.5035
-2.27	-4.81	4.00E-04	-1.44	-2.71	0.0251	-0.44	-1.35	0.4488
-0.21	-1.16	0.7475	0.53	1.44	0.4235	-0.42	-1.34	0.52
-0.12	-1.09	0.8617	1.04	2.06	0.1392	0.61	1.52	0.3791
-1.53	-2.89	0.0228	-0.79	-1.73	0.241	-0.59	-1.51	0.3605
-0.78	-1.72	0.2613	-1.66	-3.15	0.022	0.47	1.38	0.4829
0.05	1.03	0.9477	-0.68	-1.6	0.3547	-0.68	-1.61	0.3484
-0.75	-1.68	0.2666	0.32	1.24	0.6403	-0.75	-1.68	0.2682
-0.75	-1.69	0.2882	0.64	1.56	0.3659	-0.92	-1.9	0.1967
-0.79	-1.73	0.2858	-0.57	-1.48	0.4473	-0.01	-1.01	0.9849
-0.44	-1.36	0.5179	0.51	1.42	0.4646	0.29	1.22	0.6725
0.01	1.01	0.9787	-0.34	-1.26	0.5622	0.19	1.14	0.7148
-1.12	-2.17	0.1244	-1.38	-2.61	0.0608	-0.71	-1.63	0.3289
0.25	1.19	0.6629	0.13	1.1	0.8279	0.39	1.31	0.508
0.22	1.16	0.7725	-0.8	-1.74	0.2757	0.15	1.11	0.8388
-0.02	-1.01	NA	1.77	3.4	NA	-0.01	-1	NA
-0.03	-1.02	0.9677	-0.14	-1.1	0.8531	-0.16	-1.12	0.8301
-0.04	-1.03	0.9555	0.92	1.89	0.2177	1.42	2.68	0.0507
-0.28	-1.22	0.6147	0.12	1.09	0.8348	0.17	1.12	0.7555
0.07	1.05	0.9276	0.54	1.45	0.4431	0.1	1.07	0.894
-2.58	-5.96	5.00E-04	-1.68	-3.2	0.0227	-0.97	-1.95	0.1807
-0.07	-1.05	0.8826	1.05	2.07	0.0251	-0.45	-1.37	0.3369
0.11	1.08	0.8192	0.17	1.12	0.7529	0.31	1.24	0.5306
-0.75	-1.68	0.1766	-0.38	-1.3	0.5098	-0.17	-1.12	0.7508
-0.33	-1.26	0.6574	0.19	1.14	0.8028	-0.28	-1.21	0.7083
-0.68	-1.6	0.3622	-0.64	-1.56	0.3781	-0.65	-1.57	0.381
-0.46	-1.38	0.5049	-0.53	-1.45	0.4279	-0.51	-1.43	0.4601
-0.29	-1.22	0.5478	-1.12	-2.17	0.0428	-0.25	-1.19	0.6077
-0.08	-1.06	0.9105	-0.43	-1.35	0.5668	0.36	1.28	0.6311

0.32	1.25	0.5979	0.74	1.67	0.2388	-0.31	-1.24	0.6251
-0.66	-1.58	0.3389	0.36	1.28	0.6041	-0.48	-1.39	0.4865
2.26	4.78	0.0017	3.17	8.98	0	-0.02	-1.01	0.9771
-0.05	-1.03	0.9502	-0.65	-1.57	0.3792	0.11	1.08	0.8813
0.35	1.28	0.6344	-0.31	-1.24	0.6816	0.85	1.8	0.2489
0.36	1.29	0.5919	0.47	1.38	0.4749	0.1	1.07	0.8837
0.19	1.14	0.7724	-0.06	-1.04	0.9251	0.19	1.14	0.7636
-0.7	-1.62	0.2824	-0.86	-1.82	0.2028	-0.37	-1.29	0.5668
-0.38	-1.3	0.5743	0.17	1.12	0.8056	0.17	1.13	0.7931
2.98	7.9	0	2.38	5.21	0.001	0.84	1.78	0.2573
-0.35	-1.28	NA	0.34	1.27	NA	-0.41	-1.33	NA
0.01	1.01	0.9867	0.05	1.03	0.9352	-0.06	-1.04	0.9285
-1.2	-2.29	0.1084	-1.11	-2.16	0.1364	-0.25	-1.19	0.7386
0.71	1.64	NA	0.1	1.07	NA	0	-1	NA
0.55	1.46	0.3373	-0.34	-1.26	0.5307	0.09	1.07	0.8716

IgAN E1.vs	IgAN E1.vs	IgAN E1.vs	S.a.GN.vs.I	S.a.GN.vs.I	S.a.GN.vs.I	VancATN.v	VancATN.v	VancATN.v
0.31	1.24	0.2389	1.03	2.04	2.00E-04	0.2	1.15	0.4623
0.05	1.03	0.8019	0.16	1.12	0.431	0.01	1	0.9726
0.29	1.22	0.1684	0.22	1.17	0.3267	0.41	1.33	0.0499
0.48	1.39	0.0208	0.12	1.09	0.597	0.84	1.79	0
0.14	1.11	0.4649	-0.17	-1.12	0.4544	0.11	1.08	0.5714
-0.24	-1.18	0.2518	0.08	1.06	0.7235	0.05	1.04	0.7974
-0.67	-1.59	0.0163	-0.78	-1.72	0.0099	-1.23	-2.35	0
0.18	1.13	0.6095	0.73	1.66	0.0443	0.32	1.24	0.3618
0.06	1.04	0.7695	0.06	1.04	0.7863	-0.04	-1.03	0.8643
0.09	1.06	0.7308	-0.02	-1.02	0.9362	-0.18	-1.13	0.4787
0.07	1.05	0.8429	1.17	2.25	9.00E-04	0.22	1.17	0.5089
0.15	1.11	0.5194	0.4	1.32	0.1073	0.13	1.09	0.5903
-0.45	-1.36	0.0415	-0.3	-1.23	0.2049	-0.46	-1.38	0.0358
0.15	1.11	0.5259	-0.27	-1.2	0.3192	0.25	1.19	0.2911
-0.31	-1.24	0.1366	-0.55	-1.46	0.0212	-0.31	-1.24	0.1425
0.69	1.61	0.0191	0.39	1.31	0.2189	0.95	1.94	0.001
0.49	1.4	0.1362	0.89	1.86	0.009	0.47	1.38	0.1554
-0.33	-1.26	0.1906	-0.56	-1.47	0.0492	-0.6	-1.51	0.0224
-0.06	-1.04	0.7953	0.11	1.08	0.6566	0.19	1.14	0.4099
-0.15	-1.11	0.5237	-0.83	-1.78	0.003	0.1	1.07	0.6788
0.45	1.36	0.0851	0.25	1.19	0.3798	0.3	1.23	0.2583
-0.21	-1.15	0.4026	0.13	1.1	0.6125	-0.3	-1.23	0.2395
-0.57	-1.49	0.0151	-0.99	-1.98	3.00E-04	-0.79	-1.73	0.0011
-0.49	-1.41	0.0931	-1.15	-2.22	6.00E-04	-0.87	-1.83	0.0042
-0.03	-1.02	0.9167	-0.95	-1.94	0.0064	-0.63	-1.55	0.0457
0.02	1.01	0.9301	-0.39	-1.31	0.1491	-0.28	-1.22	0.2453
-0.51	-1.42	0.0411	-0.73	-1.66	0.0096	-0.63	-1.55	0.0136
-0.04	-1.03	0.8773	-0.66	-1.58	0.0202	-0.23	-1.17	0.359
-0.36	-1.28	0.126	0.19	1.14	0.4294	-0.16	-1.12	0.4842
-0.07	-1.05	0.7676	-0.78	-1.72	0.0079	-0.26	-1.2	0.3006
0.21	1.16	0.4202	-0.7	-1.63	0.0298	0.17	1.12	0.5313
-0.04	-1.03	0.883	-0.41	-1.33	0.1606	-0.21	-1.16	0.4307
-0.09	-1.06	0.7369	-0.04	-1.03	0.8851	-0.04	-1.03	0.8742
0.55	1.46	0.3629	0.54	1.46	0.3893	0.1	1.07	0.8752
0.74	1.67	0.0112	1.16	2.24	1.00E-04	0.75	1.69	0.0105
-0.1	-1.07	0.7424	-1.01	-2.01	0.0044	-0.64	-1.56	0.0424
0.3	1.23	0.29	0.64	1.56	0.0315	0.37	1.3	0.1893
-0.06	-1.04	0.8289	0.67	1.59	0.0172	-0.16	-1.11	0.5787
0.13	1.1	0.8202	0.54	1.45	0.3755	-0.45	-1.36	0.4483
0.09	1.07	0.7251	0.05	1.04	0.8512	0.05	1.04	0.837
-0.38	-1.3	0.15	-0.85	-1.8	0.0056	-0.36	-1.29	0.1696
0.2	1.15	0.4798	0.12	1.09	0.7002	0.17	1.13	0.5526
-0.34	-1.26	0.6121	-0.06	-1.05	0.925	-1	-2	0.1344

0.2	1.15	0.5177	0.9	1.86	0.0049	0.4	1.32	0.1958
0	-1	0.9973	0.7	1.62	0.01	-0.3	-1.23	0.2722
0.16	1.12	0.6252	-1.01	-2.01	0.0098	-0.16	-1.12	0.64
-0.39	-1.31	0.2017	-1.27	-2.41	6.00E-04	-0.63	-1.54	0.0483
0.23	1.17	0.4183	-0.14	-1.1	0.6649	0.06	1.04	0.8402
-0.36	-1.28	0.2284	-0.31	-1.24	0.3358	-0.33	-1.26	0.2658
0.48	1.39	0.1208	0.49	1.41	0.1368	0.62	1.53	0.0451
0.31	1.24	0.2922	0.24	1.18	0.4483	0.57	1.49	0.0462
1.02	2.03	0.0104	1.47	2.78	4.00E-04	0.88	1.83	0.0304
-0.29	-1.22	0.3087	-0.38	-1.3	0.225	-0.61	-1.52	0.0401
1.33	2.51	0.0034	0.86	1.82	0.0766	1.85	3.6	0
0.35	1.28	0.3367	0.2	1.15	0.6225	0.74	1.67	0.0409
0.02	1.01	0.9607	0.38	1.31	0.2295	0.23	1.17	0.4455
-0.39	-1.31	0.3446	0.98	1.97	0.0173	0.86	1.81	0.0278
0.96	1.95	0.0176	1.08	2.12	0.0113	1.33	2.51	9.00E-04
0.57	1.48	0.1566	0.85	1.8	0.0428	0.63	1.55	0.1141
-0.74	-1.67	0.0269	-1.09	-2.12	0.0041	-1.34	-2.54	2.00E-04
-0.05	-1.04	0.8609	-0.35	-1.27	0.3169	-0.14	-1.1	0.6575
0.23	1.18	0.4527	0.12	1.09	0.7283	-0.01	-1.01	0.9716
-0.25	-1.19	0.5364	-0.73	-1.66	0.0995	-0.79	-1.73	0.0587
0.25	1.19	0.4425	0.24	1.18	0.5027	0.24	1.18	0.4735
0.23	1.17	0.4773	-0.21	-1.15	0.5683	-1	-1.99	0.0063
0.09	1.06	0.7905	0.24	1.18	0.5059	0.03	1.02	0.9199
-0.06	-1.04	0.8607	-0.77	-1.71	0.0387	-0.09	-1.07	0.7687
0.26	1.2	0.4233	0.51	1.42	0.1393	0.54	1.46	0.0895
-0.39	-1.31	0.2345	-0.51	-1.42	0.1631	-0.55	-1.46	0.1054
-0.44	-1.36	0.166	-0.41	-1.33	0.243	-0.57	-1.48	0.085
1.46	2.75	0.0111	1.6	3.02	0.0077	2.22	4.66	1.00E-04
-0.11	-1.08	0.7465	-0.43	-1.35	0.2572	-0.11	-1.08	0.7428
0.78	1.72	0.0267	0.62	1.53	0.1071	0.66	1.58	0.0645
0.45	1.37	0.166	0.48	1.39	0.1742	0.35	1.27	0.2969
0.35	1.27	0.3303	-0.22	-1.17	0.5798	0.03	1.02	0.9308
0.11	1.08	0.7491	0.71	1.64	0.0462	0.15	1.11	0.6628
0.18	1.13	0.5982	0.39	1.31	0.2856	0.36	1.28	0.3034
-0.16	-1.12	0.6192	-0.11	-1.08	0.7566	-0.1	-1.07	0.745
-0.1	-1.07	0.7524	0.19	1.14	0.5749	-0.43	-1.35	0.1919
0.01	1.01	0.9681	0.05	1.03	0.8903	0.11	1.08	0.7313
0.84	1.79	0.1157	-1.52	-2.88	0.0144	0.31	1.24	0.5631
-0.56	-1.47	0.0888	-0.62	-1.54	0.0849	-0.74	-1.67	0.028
0.19	1.14	0.6342	-2.5	-5.66	0	-0.72	-1.64	0.0932
0.12	1.09	0.715	-0.11	-1.08	0.7666	-0.03	-1.02	0.9259
0.17	1.12	0.6345	-0.19	-1.14	0.6357	0.28	1.21	0.4286
0.06	1.04	0.8621	0.49	1.4	0.157	0.21	1.16	0.5201
1.28	2.43	0.033	1.91	3.76	0.0019	1.59	3.01	0.0076
-0.33	-1.26	0.3107	-0.71	-1.63	0.059	-0.51	-1.42	0.1329
0.12	1.09	0.7602	-1.14	-2.21	0.0141	-0.37	-1.29	0.3623
-0.38	-1.3	0.2673	-1.02	-2.02	0.0116	-0.43	-1.35	0.2089

0.48	1.39	0.1782	0.24	1.18	0.5368	0.5	1.41	0.1608
-0.27	-1.2	0.3974	-0.54	-1.46	0.13	-0.33	-1.26	0.3087
0.12	1.09	0.7132	-0.44	-1.36	0.2546	0.07	1.05	0.8476
0.16	1.11	0.6752	0.02	1.01	0.9592	0.63	1.54	0.0866
0.63	1.55	0.3826	1.39	2.61	0.0598	-0.94	-1.92	0.1987
-0.44	-1.36	0.1941	-0.69	-1.61	0.0735	-0.58	-1.5	0.0959
-0.37	-1.3	0.3026	-0.81	-1.75	0.0482	-0.54	-1.45	0.1481
0.41	1.32	0.3952	-0.99	-1.99	0.0695	0.21	1.16	0.658
0.33	1.26	0.3425	-0.06	-1.04	0.8737	0.2	1.15	0.575
0.48	1.4	0.22	-0.48	-1.39	0.3008	0.57	1.49	0.148
0.11	1.08	0.7862	0.46	1.38	0.2566	0.43	1.35	0.2634
-0.38	-1.3	0.2505	-1.04	-2.05	0.008	-0.59	-1.51	0.0818
-0.37	-1.29	0.3057	0.05	1.03	0.8947	-0.73	-1.65	0.0531
-0.25	-1.19	0.5002	0.28	1.22	0.4621	0.28	1.22	0.4339
-0.26	-1.19	0.5349	-0.68	-1.6	0.1385	-0.35	-1.28	0.3952
-0.63	-1.55	0.0741	-0.82	-1.77	0.0373	-1.13	-2.19	0.0027
-0.08	-1.05	0.83	-0.52	-1.44	0.1954	-0.22	-1.16	0.5534
0.07	1.05	0.8691	-0.14	-1.1	0.7647	0.24	1.18	0.5805
0.58	1.49	0.3121	1.73	3.32	0.0029	1.1	2.15	0.0501
0.43	1.35	0.2181	0.23	1.17	0.5484	0.57	1.49	0.1012
-0.19	-1.14	0.643	-1.78	-3.42	6.00E-04	-0.6	-1.52	0.1632
0.02	1.02	0.9446	0.04	1.03	0.9112	0.07	1.05	0.8492
0.22	1.17	0.563	-0.45	-1.36	0.3054	-0.02	-1.02	0.9506
-0.48	-1.4	0.1818	-0.11	-1.08	0.7716	-0.67	-1.59	0.0731
-0.2	-1.15	0.5774	-0.44	-1.36	0.2643	-0.34	-1.27	0.3452
-0.26	-1.2	0.4818	-0.54	-1.45	0.2009	-0.26	-1.2	0.4857
-0.31	-1.24	0.3816	-0.43	-1.35	0.2642	-0.47	-1.38	0.199
0.18	1.13	0.4271	0.5	1.41	0.038	0.21	1.15	0.37
0.57	1.49	0.1555	1.05	2.07	0.0117	1.13	2.19	0.0039
-0.06	-1.04	0.8266	-0.08	-1.06	0.7734	0.08	1.06	0.7675
-0.4	-1.32	0.2653	-0.05	-1.03	0.8959	-0.46	-1.38	0.2029
0.16	1.12	0.6724	0.41	1.33	0.3143	0.16	1.12	0.6854
-0.09	-1.07	0.8101	0.28	1.22	0.4788	0.03	1.02	0.9283
0.24	1.18	0.5379	-0.97	-1.96	0.0421	0.07	1.05	0.871
-0.08	-1.06	0.851	-0.93	-1.9	0.0526	-0.7	-1.63	0.1075
-0.25	-1.19	0.5054	-0.57	-1.49	0.1775	-0.25	-1.19	0.5122
0.02	1.02	0.9735	0.16	1.12	0.8306	-0.47	-1.39	0.5185
-0.33	-1.26	0.3792	-0.93	-1.9	0.0318	-1.35	-2.55	0.0013
-0.02	-1.01	0.9677	-0.8	-1.75	0.0794	-0.17	-1.13	0.6651
0.09	1.06	0.8437	1.16	2.23	0.0098	1.18	2.27	0.0054
-0.19	-1.14	0.6189	-0.48	-1.39	0.2584	-0.13	-1.09	0.7379
0.34	1.26	0.3891	0.12	1.09	0.7776	0.69	1.61	0.0742
-0.16	-1.12	0.6776	-1.41	-2.66	0.0029	-0.98	-1.97	0.0189
0.4	1.32	0.3217	0.89	1.85	0.0338	0.26	1.2	0.5282
0.05	1.03	0.8986	0.54	1.45	0.1782	0.34	1.26	0.3743
0.04	1.03	0.9263	1.27	2.42	0.0041	0.38	1.3	0.3929
-0.16	-1.12	0.6642	0.04	1.03	0.9107	-0.23	-1.17	0.5529

0.23	1.17	0.5706	0.17	1.12	0.7016	0.32	1.25	0.42
0.14	1.1	0.7095	-0.66	-1.58	0.1384	-0.12	-1.09	0.7637
-0.24	-1.18	0.5164	-0.41	-1.33	0.3093	-0.24	-1.18	0.5136
1.26	2.39	0.0477	2.61	6.09	1.00E-04	2.42	5.36	1.00E-04
0.43	1.34	0.3298	-0.53	-1.44	0.2968	0.3	1.23	0.5009
0.39	1.31	0.2022	0.54	1.45	0.0991	0.38	1.3	0.2157
0.09	1.06	0.8285	-0.33	-1.26	0.4671	0.4	1.32	0.3119
-0.03	-1.02	0.9452	0.07	1.05	0.874	0.11	1.08	0.7638
0.21	1.15	0.662	-0.88	-1.84	0.1029	0.16	1.11	0.7404
-0.34	-1.26	0.3835	-1.66	-3.16	7.00E-04	-0.93	-1.91	0.0245
-0.41	-1.33	0.2684	-1.38	-2.59	0.0026	-0.69	-1.61	0.0748
-0.29	-1.22	0.5135	0.95	1.93	0.0333	-0.72	-1.65	0.1176
0.16	1.12	0.6652	0.02	1.01	0.9694	-0.09	-1.06	0.825
0.7	1.62	0.0974	0.24	1.18	0.6132	0.84	1.79	0.0451
-0.43	-1.35	0.2481	-0.25	-1.19	0.5324	-0.61	-1.53	0.1126
-0.37	-1.29	0.3668	-0.16	-1.11	0.7168	-0.43	-1.35	0.2937
0.27	1.21	0.4788	-0.4	-1.32	0.37	0.26	1.2	0.4985
-0.51	-1.43	0.2253	0.28	1.21	0.5156	-0.58	-1.49	0.1816
0.04	1.03	0.9238	-0.45	-1.36	0.3288	0.06	1.04	0.8786
0.59	1.51	0.4271	1.15	2.22	0.1224	0.82	1.76	0.2748
0.19	1.14	0.6092	0.36	1.28	0.3667	0.06	1.04	0.8859
0.32	1.25	0.4878	-1.07	-2.1	0.0481	0.2	1.15	0.6692
0.11	1.08	0.7612	0.8	1.74	0.0303	0.61	1.52	0.084
-0.12	-1.09	0.7516	-0.11	-1.08	0.7922	-0.11	-1.08	0.7811
-0.66	-1.58	0.1813	-2.42	-5.36	1.00E-04	-0.62	-1.54	0.2105
-0.47	-1.39	0.2382	-0.17	-1.12	0.692	-0.37	-1.29	0.3557
-0.78	-1.72	0.0391	-0.62	-1.54	0.1239	-0.78	-1.71	0.0421
1.25	2.38	0.0059	0.39	1.31	0.4428	1.29	2.45	0.0047
0.11	1.08	0.7933	0.35	1.27	0.4513	-0.02	-1.01	0.9673
0.35	1.28	0.4321	0.31	1.24	0.5222	-0.01	-1.01	0.9809
0.12	1.09	0.7806	-1.33	-2.51	0.0106	-0.24	-1.18	0.5891
0.1	1.07	0.8181	-0.51	-1.43	0.3107	0.49	1.4	0.2662
0.51	1.43	0.2846	0.72	1.64	0.1536	0.67	1.59	0.1635
0.28	1.22	0.5071	0.12	1.09	0.7961	0.64	1.56	0.1276
-0.51	-1.42	0.2099	0.38	1.3	0.3458	-0.31	-1.24	0.4375
-0.14	-1.1	0.7335	-1.09	-2.13	0.0286	-0.22	-1.16	0.6095
-0.32	-1.25	0.1461	0.11	1.08	0.6436	-0.21	-1.16	0.3457
-0.47	-1.38	0.2556	0.75	1.68	0.0737	-0.86	-1.82	0.0426
-0.49	-1.41	0.2164	-0.95	-1.94	0.0372	-0.54	-1.46	0.1803
0.34	1.27	0.396	0.1	1.07	0.8159	0.45	1.37	0.2597
0.14	1.1	0.7463	0.45	1.37	0.3139	-0.02	-1.02	0.9608
0.32	1.25	0.4219	-0.2	-1.15	0.6542	0.38	1.3	0.3445
0.68	1.6	0.1322	0.71	1.63	0.141	0.5	1.42	0.273
0.09	1.06	0.8293	0.51	1.43	0.2175	-0.1	-1.07	0.8175
-0.13	-1.09	0.7577	0.19	1.14	0.6554	-0.1	-1.07	0.8026
0.25	1.19	0.5821	0.78	1.72	0.0928	0.95	1.94	0.0283
-0.04	-1.03	0.9267	0.46	1.38	0.3018	0.38	1.3	0.3668

-0.61	-1.53	0.2643	-1.19	-2.28	0.0447	-1.67	-3.18	0.0039
0.23	1.17	0.608	-0.59	-1.51	0.2476	0.28	1.22	0.5271
0.34	1.27	0.4005	-0.12	-1.09	0.7926	-0.48	-1.4	0.2786
-0.13	-1.1	0.7357	-0.36	-1.28	0.4101	-0.33	-1.26	0.415
-0.15	-1.11	0.7241	-2.23	-4.7	1.00E-04	-0.46	-1.38	0.2884
-0.16	-1.12	0.6932	0.21	1.16	0.627	-0.36	-1.29	0.3918
0.25	1.19	0.5613	-0.84	-1.78	0.1074	-0.31	-1.24	0.5
-0.46	-1.37	0.2554	-1.07	-2.09	0.0227	-0.8	-1.74	0.0578
-0.25	-1.19	0.5445	-0.83	-1.78	0.079	-0.48	-1.39	0.2593
0.5	1.42	0.3915	0.57	1.48	0.3574	0.13	1.09	0.8308
-0.01	-1	0.9914	0.64	1.55	0.1811	0.71	1.63	0.1156
-0.91	-1.88	0.0269	-1.23	-2.35	0.0087	-0.97	-1.96	0.0212
-0.15	-1.11	0.7161	-0.43	-1.34	0.353	-0.24	-1.18	0.5721
0.2	1.15	0.6966	-1.1	-2.14	0.0633	-0.02	-1.01	0.9769
0.14	1.1	0.75	-1.08	-2.11	0.0404	-0.21	-1.15	0.6448
-0.14	-1.1	0.7506	0.62	1.53	0.154	0.18	1.13	0.6663
-0.27	-1.21	0.5002	-0.1	-1.07	0.8221	-0.56	-1.47	0.1853
-0.57	-1.49	0.2206	-0.39	-1.31	0.435	-0.91	-1.87	0.0598
0.15	1.11	0.7387	0.51	1.42	0.2652	0.14	1.1	0.7516
-0.1	-1.07	0.814	-0.41	-1.33	0.3769	-0.57	-1.48	0.1954
0.25	1.19	0.5816	0.68	1.6	0.1546	0.46	1.38	0.314
-0.32	-1.25	0.432	0.11	1.08	0.7912	-0.5	-1.41	0.2285
0.59	1.5	0.2021	1.15	2.23	0.0137	0.77	1.7	0.0936
0.33	1.26	0.4644	0.17	1.12	0.7317	-0.54	-1.46	0.2591
-0.69	-1.61	0.0944	-0.59	-1.5	0.1843	-0.5	-1.41	0.2237
0.23	1.18	0.548	0.71	1.64	0.0765	-0.36	-1.28	0.3872
-0.22	-1.16	0.6134	0.43	1.35	0.3275	-0.25	-1.19	0.5589
0.69	1.61	0.1737	1.17	2.25	0.0242	0.64	1.56	0.2107
-0.35	-1.27	0.3879	-0.56	-1.48	0.2133	-0.46	-1.38	0.2674
0	1	0.9999	-0.4	-1.32	0.418	0.65	1.57	0.1198
1.38	2.6	0.0299	1.44	2.71	0.029	2.03	4.09	0.0012
0.07	1.05	0.8683	0.4	1.32	0.3901	0.02	1.01	0.9671
-0.24	-1.18	0.576	-1.3	-2.47	0.0112	-0.65	-1.57	0.1442
0.2	1.15	0.6882	1.2	2.3	0.0185	0.62	1.53	0.2168
-0.43	-1.35	0.2996	-0.58	-1.49	0.2076	-0.19	-1.14	0.6367
0.35	1.27	0.3836	0.4	1.32	0.3422	0.39	1.31	0.3229
0.26	1.2	0.5929	0.59	1.5	0.2509	0.38	1.3	0.4432
0	1	0.995	-0.22	-1.17	0.6493	0.07	1.05	0.8722
0.15	1.11	0.7493	-0.9	-1.86	0.1079	0.08	1.06	0.8726
0.34	1.26	0.4501	0.18	1.14	0.7052	0.32	1.25	0.484
0.01	1.01	0.987	-1.07	-2.11	0.0633	-0.26	-1.2	0.6038
-0.04	-1.03	0.941	-1.52	-2.86	0.0194	-0.09	-1.06	0.8814
0.08	1.05	0.8944	-1.25	-2.37	0.0506	-0.37	-1.3	0.526
-0.47	-1.39	0.277	-0.61	-1.52	0.2032	0.04	1.03	0.9242
0.69	1.61	0.1313	0.25	1.19	0.619	0.44	1.35	0.3507
-0.54	-1.46	0.2363	-0.42	-1.33	0.3981	-0.17	-1.12	0.709
0	-1	0.9965	-0.77	-1.71	0.1559	0.32	1.24	0.4924

-0.13	-1.1	0.7697	0.34	1.27	0.4707	0.41	1.33	0.3554
-0.33	-1.25	0.4259	-0.9	-1.87	0.0578	-0.1	-1.07	0.8099
0.15	1.11	0.7513	0.68	1.6	0.1657	-0.33	-1.26	0.5076
-0.27	-1.2	0.5513	-0.35	-1.27	0.4757	-0.65	-1.57	0.1702
-0.19	-1.14	0.6849	-0.57	-1.49	0.2659	-0.35	-1.28	0.4531
-0.34	-1.27	0.4414	-0.11	-1.08	0.8091	-0.92	-1.89	0.0505
0.66	1.58	0.1806	0.6	1.51	0.2553	1.32	2.5	0.0057
0.28	1.21	0.5517	-0.06	-1.04	0.9088	-0.35	-1.27	0.484
0.12	1.08	0.7835	-0.14	-1.1	0.761	-0.62	-1.53	0.1781
0.55	1.47	0.3105	1.07	2.09	0.0569	0.95	1.94	0.0762
0.18	1.13	0.7018	-0.01	-1.01	0.9773	0.61	1.52	0.1934
0.52	1.43	0.3466	0.43	1.34	0.465	0.74	1.67	0.1795
0.15	1.11	0.8355	-0.21	-1.16	0.7688	0.7	1.62	0.3196
-0.16	-1.12	0.7155	-0.31	-1.24	0.5232	-0.63	-1.55	0.1827
1.1	2.14	0.0153	1.09	2.13	0.0227	1.21	2.32	0.0074
-0.45	-1.36	0.2952	-0.88	-1.84	0.071	-0.48	-1.4	0.2666
0	1	0.9968	-0.61	-1.52	0.2422	-0.39	-1.31	0.4123
0.4	1.32	0.5092	1.16	2.24	0.0574	2.03	4.07	4.00E-04
0.25	1.19	0.5792	-0.04	-1.03	0.937	-0.12	-1.09	0.8014
0.41	1.33	0.4068	0.63	1.55	0.223	0.26	1.2	0.598
-0.69	-1.62	0.1295	-2.11	-4.31	3.00E-04	-1.38	-2.61	0.0054
-0.25	-1.19	0.6006	-0.34	-1.27	0.5042	-0.26	-1.19	0.5885
-0.04	-1.03	0.9331	0.67	1.6	0.1378	0.02	1.01	0.9666
0.07	1.05	0.8788	-0.44	-1.36	0.4078	-0.24	-1.18	0.6284
0.7	1.63	0.1569	1.03	2.04	0.0467	1.02	2.03	0.0373
1.55	2.93	0.0156	2.51	5.71	1.00E-04	2.79	6.93	0
0.28	1.21	0.5464	0.16	1.12	0.7426	0.52	1.43	0.2591
-0.43	-1.35	0.3377	-1.12	-2.17	0.0334	-0.4	-1.32	0.3851
0.45	1.37	0.3835	0.82	1.77	0.1257	1.03	2.05	0.0401
-0.32	-1.25	0.4671	0.05	1.04	0.9063	-0.74	-1.67	0.1121
0.08	1.06	0.8282	0.51	1.42	0.2049	0.16	1.11	0.6859
0.02	1.01	0.9662	0.47	1.38	0.3256	-0.1	-1.07	0.8394
-0.43	-1.35	0.3302	-1.44	-2.71	0.0073	-1.17	-2.25	0.0155
0.91	1.88	0.0851	1.32	2.49	0.0161	1.47	2.77	0.0046
0.47	1.39	0.3513	0.44	1.36	0.4153	0.95	1.93	0.0546
-0.1	-1.07	0.8389	-1.45	-2.73	0.012	-0.5	-1.42	0.3092
0.42	1.34	0.3867	0.48	1.4	0.3501	0.57	1.48	0.2417
0.4	1.32	0.4399	0.62	1.54	0.2497	1.05	2.07	0.0349
0.47	1.39	0.3413	0.03	1.02	0.9533	0.71	1.63	0.1508
-0.75	-1.68	0.1645	0.41	1.33	0.4396	-0.99	-1.98	0.0749
0.35	1.28	0.5221	-0.8	-1.74	0.1979	0.39	1.31	0.4798
0.51	1.43	0.3806	1.4	2.65	0.0172	0.89	1.86	0.1211
0.02	1.01	0.9735	0.37	1.29	0.4476	0.22	1.17	0.6275
-0.4	-1.32	0.4067	-0.51	-1.43	0.3298	-0.61	-1.52	0.2247
-0.13	-1.1	0.8013	-0.64	-1.56	0.2675	-0.17	-1.12	0.7517
-0.03	-1.02	0.9555	0.95	1.94	0.0886	0.49	1.41	0.3638
-0.18	-1.13	0.7132	-0.67	-1.6	0.2172	-0.63	-1.55	0.2159

0.02	1.01	0.9665	-0.2	-1.15	0.7012	0.03	1.02	0.9568
-0.23	-1.17	0.5565	0.84	1.79	0.0321	-0.44	-1.36	0.2726
-0.01	-1.01	0.9835	0.38	1.3	0.4475	0.04	1.03	0.9357
0.77	1.7	0.1317	0.69	1.61	0.2022	0.8	1.74	0.1162
0.66	1.58	0.2157	-0.26	-1.2	0.6654	1.01	2.02	0.0544
-0.17	-1.13	0.7383	-0.8	-1.75	0.1644	-0.13	-1.09	0.809
-0.52	-1.44	0.2526	-0.12	-1.09	0.8001	-0.69	-1.61	0.1434
0.29	1.23	0.5377	0.26	1.2	0.6157	0.36	1.28	0.4543
-0.49	-1.41	0.307	-0.51	-1.43	0.3242	-0.78	-1.72	0.1177
-0.27	-1.21	0.638	-0.86	-1.82	0.1728	-0.3	-1.23	0.6011
-0.53	-1.44	0.3147	0.23	1.17	0.6661	-0.28	-1.21	0.5965
0.12	1.09	0.8017	-1.14	-2.21	0.0529	0.01	1.01	0.9833
0.09	1.07	0.84	-1.1	-2.15	0.0501	0.3	1.23	0.5153
0.12	1.09	0.8098	-0.01	-1	0.9904	0.48	1.39	0.3359
0.03	1.02	0.9495	0.25	1.19	0.6148	-0.14	-1.11	0.7657
0.11	1.08	0.8141	0.39	1.31	0.4408	-0.35	-1.28	0.486
0.3	1.23	0.5416	0.48	1.39	0.3498	0.41	1.32	0.4033
0.57	1.48	0.2631	0.92	1.89	0.079	0.8	1.74	0.1136
-0.35	-1.27	0.4567	-0.32	-1.25	0.5243	-0.71	-1.64	0.146
0.61	1.52	0.2551	1.05	2.08	0.0535	1.29	2.45	0.0117
0.14	1.1	0.7057	-1.02	-2.03	0.0291	-0.33	-1.26	0.4078
-0.05	-1.04	0.9137	0.25	1.19	0.6265	-0.8	-1.74	0.1247
0.48	1.4	0.3141	-0.96	-1.95	0.0947	-0.16	-1.12	0.7498
0.37	1.29	0.4836	-0.78	-1.72	0.1956	-0.22	-1.16	0.6918
0.33	1.26	0.5349	0.91	1.88	0.0917	0.77	1.71	0.1359
-0.18	-1.13	0.712	-0.48	-1.39	0.3676	-0.24	-1.18	0.619
0.35	1.28	0.4735	-0.09	-1.06	0.8709	0.76	1.7	0.1144
0.84	1.79	0.1049	0.87	1.82	0.1133	0.55	1.46	0.3038
-0.23	-1.17	0.6294	0.34	1.27	0.4863	-1.01	-2.02	0.0503
-0.35	-1.27	0.488	-0.64	-1.56	0.2452	-0.72	-1.65	0.1688
0.98	1.97	0.1243	0.56	1.48	0.4084	1.71	3.27	0.0061
-0.14	-1.1	0.7886	0.46	1.38	0.3928	0.67	1.59	0.1911
-1.29	-2.44	0.0158	-3.09	-8.53	0	-1.73	-3.31	0.0021
-0.52	-1.43	0.3046	-0.2	-1.15	0.7061	-0.73	-1.66	0.1571
-0.08	-1.06	0.8691	0.72	1.65	0.1531	-0.43	-1.35	0.4135
0.6	1.52	0.31	1.39	2.62	0.021	1.46	2.75	0.0115
0.36	1.29	0.4854	-0.36	-1.29	0.5362	0.73	1.66	0.1527
0.62	1.54	0.2924	0.93	1.9	0.1281	1.17	2.26	0.0415
-0.14	-1.1	0.7682	-0.5	-1.42	0.3487	-0.29	-1.22	0.553
-0.03	-1.02	0.9511	0.09	1.06	0.8654	-0.22	-1.17	0.6633
1.27	2.41	0.0151	0.55	1.47	0.342	1.26	2.39	0.0172
1.15	2.21	0.0414	1.08	2.11	0.0699	1.4	2.63	0.0125
0.55	1.47	0.2804	0.38	1.3	0.4938	0.78	1.72	0.1238
-0.42	-1.33	0.3735	-1.93	-3.81	9.00E-04	-1.28	-2.43	0.0118
-0.35	-1.27	0.4925	0.05	1.03	0.9269	-0.38	-1.3	0.46
-0.16	-1.12	0.6086	-1.08	-2.12	0.004	-0.13	-1.09	0.6915
-0.35	-1.27	0.5184	0.59	1.51	0.2701	0.37	1.29	0.4758

0.3	1.23	0.5781	1.07	2.1	0.0486	0.37	1.29	0.4981
-0.58	-1.5	0.2198	-0.94	-1.92	0.075	-1.57	-2.97	0.0027
-0.07	-1.05	0.901	-0.79	-1.73	0.1788	-0.41	-1.33	0.4508
0.45	1.37	0.3747	0.67	1.59	0.2093	0.57	1.48	0.2677
-0.2	-1.15	0.7201	0.59	1.5	0.2849	0.67	1.59	0.1987
-0.01	-1.01	0.9811	-0.61	-1.52	0.3071	0.23	1.17	0.6643
0.02	1.01	0.9667	0.38	1.3	0.4696	-0.13	-1.1	0.7959
-0.28	-1.21	0.6113	-2.12	-4.35	0.0012	-1.48	-2.8	0.013
0.55	1.47	0.331	0.46	1.37	0.4478	0.87	1.83	0.1183
1.04	2.06	0.0633	1.28	2.42	0.0286	1.02	2.03	0.0713
0.87	1.82	0.144	0.8	1.74	0.2049	1.47	2.77	0.0111
0.51	1.42	0.4103	-0.55	-1.46	0.4134	0.84	1.79	0.1695
-0.06	-1.04	0.9208	0.48	1.4	0.4007	0.02	1.02	0.9693
-0.02	-1.01	0.9668	-0.43	-1.35	0.4333	-0.08	-1.06	0.8704
-0.77	-1.71	0.1903	-1.1	-2.15	0.0803	-1.4	-2.64	0.0219
0.43	1.35	0.5041	1.12	2.17	0.0884	2.33	5.04	1.00E-04
0.81	1.75	0.1265	1.07	2.09	0.0518	1.12	2.17	0.0323
0.08	1.05	0.8832	0.42	1.34	0.4332	0.04	1.03	0.9369
0.14	1.11	0.7799	-0.16	-1.12	0.7742	-0.59	-1.51	0.2818
-0.05	-1.03	0.9276	0.43	1.35	0.4221	-0.33	-1.26	0.5415
-0.33	-1.26	0.5587	-0.51	-1.43	0.4026	0.14	1.1	0.7969
0.64	1.56	0.3185	0.71	1.63	0.2876	0.96	1.94	0.1317
0.27	1.2	0.6202	0.29	1.22	0.6153	0.33	1.26	0.5426
0.03	1.02	0.9586	-0.25	-1.19	0.6814	-0.25	-1.19	0.6613
0.2	1.15	0.7141	0.66	1.58	0.2486	0.21	1.16	0.7123
-0.39	-1.31	0.4389	-0.74	-1.67	0.1881	-0.66	-1.58	0.2076
0.47	1.38	0.3682	-0.93	-1.9	0.1286	-0.12	-1.09	0.8195
0.61	1.52	0.2504	0.72	1.65	0.195	0.94	1.92	0.0711
-0.43	-1.35	0.4473	-0.36	-1.29	0.5446	0.24	1.18	0.6535
1.27	2.41	0.0335	2.4	5.29	1.00E-04	1.69	3.22	0.0042
0.35	1.27	0.5273	0.5	1.42	0.3818	0.83	1.78	0.1222
-0.24	-1.18	0.6288	-0.66	-1.58	0.2373	-0.35	-1.27	0.4953
0.23	1.17	0.6731	0.3	1.23	0.6012	0.26	1.2	0.6261
0.37	1.29	0.5182	-0.15	-1.11	0.8141	0.45	1.36	0.4302
0.72	1.65	0.2192	1.07	2.1	0.0768	0.8	1.74	0.1755
-0.98	-1.98	0.0754	-1.12	-2.18	0.0606	-1.09	-2.13	0.0521
-0.33	-1.26	0.538	-0.05	-1.04	0.926	-0.33	-1.25	0.5451
-0.3	-1.23	0.5503	-0.47	-1.38	0.3986	-0.14	-1.1	0.7858
0.12	1.09	0.8095	-0.19	-1.14	0.7423	-0.1	-1.07	0.8498
0.26	1.2	0.6344	-0.64	-1.55	0.3094	-0.24	-1.18	0.6772
-0.6	-1.51	0.2609	-0.94	-1.92	0.1081	-1.24	-2.36	0.0289
-0.17	-1.12	0.8079	0.94	1.92	0.1735	-0.16	-1.12	0.8155
-0.38	-1.3	0.5658	1.94	3.83	0.002	-0.16	-1.12	0.8109
0.07	1.05	0.8939	0.11	1.08	0.8537	-0.33	-1.26	0.555
-0.17	-1.13	0.7349	-0.07	-1.05	0.8943	-0.02	-1.02	0.9616
0.78	1.72	0.1697	0.08	1.06	0.8928	0.38	1.3	0.5223
-0.97	-1.96	0.1419	-2.11	-4.32	0.0027	-1.07	-2.1	0.1073

2.01	4.03	0.0019	0.95	1.93	0.1734	2.2	4.58	7.00E-04
-0.24	-1.18	0.6762	0.1	1.07	0.8739	-0.06	-1.04	0.9225
0.41	1.33	0.418	0.57	1.49	0.2893	-0.34	-1.27	0.5328
0.32	1.25	0.5704	0.79	1.73	0.1752	0.63	1.55	0.26
-0.51	-1.42	0.3352	0.11	1.08	0.831	-1.03	-2.04	0.064
0.51	1.43	0.3586	0.7	1.63	0.2255	0.25	1.19	0.6589
-0.47	-1.38	0.3988	-0.1	-1.07	0.8613	-0.35	-1.28	0.5227
0.09	1.06	0.8824	1.16	2.24	0.0538	0.11	1.08	0.8538
0.9	1.86	0.1473	1.18	2.26	0.0652	1.64	3.11	0.0062
-0.53	-1.44	0.3712	0.44	1.35	0.4629	0.01	1.01	0.9824
1.11	2.15	0.046	0.97	1.96	0.0989	1	2	0.0744
0.26	1.2	0.6123	-0.6	-1.51	0.3139	-0.01	-1.01	0.9789
0.77	1.71	0.1579	-0.33	-1.25	0.603	0.4	1.32	0.4819
-0.06	-1.04	0.9143	0.53	1.44	0.3629	-0.25	-1.19	0.6693
-0.12	-1.09	0.8231	0.01	1.01	0.9845	0.1	1.08	0.8439
0.66	1.58	0.2886	-0.1	-1.07	0.8828	0.58	1.5	0.3524
0.19	1.14	0.7185	-0.12	-1.09	0.8346	0.16	1.12	0.7645
-0.67	-1.59	0.194	-0.48	-1.39	0.3812	-1.25	-2.38	0.024
0.2	1.15	0.7406	0.88	1.84	0.1468	0.74	1.67	0.2085
0.41	1.33	0.4985	-0.24	-1.18	0.7138	-0.88	-1.83	0.1787
0.12	1.09	0.8375	0.22	1.16	0.7222	0.74	1.67	0.1899
0.59	1.5	0.3083	-1.16	-2.23	0.088	0.19	1.14	0.7441
0.31	1.24	0.5969	-1.1	-2.15	0.1033	0.56	1.48	0.3312
-0.22	-1.16	0.6811	-0.29	-1.23	0.605	-0.11	-1.08	0.8319
0.38	1.31	0.5383	1.29	2.45	0.0395	0.85	1.81	0.1649
-0.77	-1.7	0.1567	-1.53	-2.89	0.0137	-0.85	-1.8	0.1243
-0.82	-1.76	0.184	-1.69	-3.23	0.0122	-1.48	-2.78	0.0214
0.36	1.29	0.5118	-0.46	-1.37	0.4571	-0.27	-1.21	0.638
-0.38	-1.3	0.542	-2.41	-5.33	6.00E-04	-1.02	-2.03	0.1152
0	-1	0.9979	-0.19	-1.14	0.7433	-0.28	-1.21	0.6107
-0.86	-1.81	0.1198	-0.77	-1.71	0.1879	-1.59	-3	0.0078
-0.51	-1.42	0.3595	-0.27	-1.21	0.6393	-0.29	-1.22	0.5995
0.12	1.08	0.8367	0.7	1.63	0.2244	0.48	1.4	0.3886
-0.36	-1.28	0.5207	0.37	1.3	0.5036	-0.07	-1.05	0.892
-0.43	-1.35	0.4511	-0.01	-1.01	0.9886	0.15	1.11	0.7861
0.56	1.48	0.3505	0.53	1.45	0.4029	1.17	2.24	0.0467
0.55	1.47	0.3644	0.72	1.64	0.2599	0.95	1.94	0.1116
-0.44	-1.35	0.4184	-0.96	-1.94	0.1113	-1.51	-2.84	0.0119
0.15	1.11	0.8208	0.31	1.24	0.6319	-0.01	-1.01	0.9866
0.18	1.13	0.7428	0.4	1.32	0.4884	-0.82	-1.76	0.1742
1.24	2.36	0.0601	1.84	3.59	0.0059	2.29	4.89	3.00E-04
-0.97	-1.96	0.075	-2.17	-4.49	8.00E-04	-1.05	-2.08	0.0576
0.34	1.26	0.5457	-0.3	-1.23	0.6289	0.29	1.23	0.6005
-0.16	-1.12	0.7841	0.39	1.31	0.5106	-0.04	-1.03	0.9452
0.3	1.23	0.6368	0.26	1.2	0.7006	0.96	1.94	0.1253
0.56	1.47	0.4263	1.79	3.46	0.0106	2.94	7.69	0
0.3	1.23	0.6256	-0.17	-1.12	0.7992	0.17	1.12	0.7835

-0.04	-1.03	0.9489	-0.46	-1.37	0.4806	0.29	1.22	0.6279
-1.04	-2.06	0.0982	-2.02	-4.07	0.0036	-2.94	-7.69	0
1.24	2.36	0.0612	-0.18	-1.13	0.8083	0.96	1.95	0.153
-0.35	-1.27	0.5178	0.27	1.21	0.6174	-0.51	-1.42	0.3604
0.4	1.32	0.49	-0.34	-1.26	0.5967	0.01	1.01	0.9846
-0.09	-1.06	0.8784	1.14	2.21	0.0453	0.63	1.55	0.2649
0.19	1.14	0.747	1.18	2.26	0.0489	0.38	1.3	0.5245
0.35	1.27	0.5439	0.51	1.43	0.393	0.49	1.4	0.398
-0.88	-1.83	0.1335	-0.26	-1.2	0.6635	-0.42	-1.34	0.4585
0.02	1.01	0.9802	0.43	1.34	0.4976	0.5	1.42	0.3998
-0.42	-1.34	0.4628	-1.11	-2.15	0.0844	-1.05	-2.07	0.085
0.53	1.45	0.3909	-0.53	-1.45	0.4364	0.36	1.28	0.5666
0.5	1.42	0.4033	-1.29	-2.45	0.0648	0.45	1.37	0.4552
-1.05	-2.07	0.073	-2.25	-4.75	9.00E-04	-1.08	-2.12	0.0684
1.07	2.1	0.1358	2.36	5.13	9.00E-04	3.36	10.24	0
0.21	1.16	0.7077	-0.14	-1.11	0.8141	-0.03	-1.02	0.9625
-0.44	-1.36	0.2459	-0.06	-1.04	0.8833	0.19	1.14	0.5987
-0.08	-1.06	0.8976	-0.78	-1.72	0.2451	0.67	1.59	0.2539
1.19	2.29	0.0666	0.95	1.93	0.1672	1.86	3.64	0.0033
0.22	1.17	0.6881	-0.4	-1.32	0.5193	0.12	1.09	0.8315
0.1	1.07	0.8688	-0.74	-1.67	0.2512	-0.43	-1.35	0.4756
-0.07	-1.05	0.9024	-0.29	-1.22	0.6366	0.26	1.2	0.6373
-0.75	-1.69	0.1125	-1.34	-2.53	0.0145	-1.42	-2.68	0.0059
0.38	1.3	0.5104	-0.33	-1.26	0.6078	0.43	1.35	0.4584
0.24	1.18	0.6969	0.59	1.51	0.3517	0.76	1.69	0.2099
2.46	5.49	2.00E-04	2.73	6.64	1.00E-04	2.79	6.9	0
1.01	2.01	0.1783	1.01	2.02	0.1754	0.17	1.13	0.8192
0.31	1.24	0.6143	0.36	1.29	0.5734	-0.3	-1.23	0.6455
-1.07	-2.1	0.1013	0.17	1.13	0.7907	0.19	1.14	0.7602
1.13	2.19	0.0778	1.88	3.68	0.0038	1.6	3.03	0.0116
-0.12	-1.08	0.8549	-0.34	-1.26	0.6152	0.2	1.15	0.7458
0.49	1.41	0.4237	0.95	1.93	0.1327	0.52	1.43	0.4027
0.93	1.9	0.1627	-1.07	-2.1	0.1417	0.31	1.24	0.6523
-0.82	-1.76	0.2046	0.07	1.05	0.9082	0.12	1.09	0.8432
0.09	1.06	0.8741	0.21	1.16	0.7188	-0.22	-1.16	0.7102
-0.52	-1.43	0.3744	0.02	1.01	0.9721	-1	-2	0.1015
-0.42	-1.33	0.4818	0.69	1.62	0.2344	-0.04	-1.03	0.9414
-0.43	-1.35	0.5182	-0.44	-1.35	0.5296	-0.81	-1.75	0.2373
-0.72	-1.64	0.2349	-1.34	-2.53	0.0437	-0.43	-1.34	0.4747
0.32	1.25	0.5813	-0.83	-1.78	0.2112	-0.69	-1.61	0.2776
0.37	1.3	0.5571	-0.31	-1.24	0.6507	0.59	1.51	0.3492
-0.29	-1.22	0.6807	-0.98	-1.97	0.1805	-0.12	-1.08	0.868
-0.33	-1.26	0.5574	-0.47	-1.39	0.4394	-0.05	-1.04	0.9289
0.27	1.21	0.6408	0.1	1.07	0.8734	-0.58	-1.49	0.3561
-0.78	-1.72	0.1167	-0.73	-1.66	0.1709	-1.37	-2.58	0.0105
-0.3	-1.23	0.6133	-0.83	-1.78	0.202	-0.3	-1.23	0.6116
0.6	1.51	0.3554	0.28	1.22	0.6825	1.38	2.6	0.0276

0.78	1.72	0.216	1	2	0.128	1.21	2.32	0.0515
0.12	1.09	0.8323	0.09	1.07	0.8826	-0.08	-1.06	0.8896
0.4	1.32	0.5253	0.92	1.9	0.1529	0.49	1.4	0.442
-0.69	-1.61	0.271	0.29	1.22	0.6357	-0.03	-1.02	0.9582
-0.16	-1.12	0.7834	-1.44	-2.72	0.0303	-1.09	-2.13	0.0793
0	1	0.9937	-0.29	-1.22	0.6627	-0.82	-1.77	0.2143
1.17	2.25	0.0263	0.73	1.66	0.1941	1.02	2.03	0.0556
0.69	1.61	0.2935	-0.08	-1.05	0.9125	0.86	1.81	0.1882
-0.41	-1.33	0.4846	0	1	0.9956	-0.73	-1.66	0.2233
-0.23	-1.17	0.3718	0.21	1.16	0.437	0.05	1.04	0.8376
0.95	1.93	0.1541	1.63	3.09	0.0155	1.04	2.06	0.1168
0.05	1.03	0.9378	-0.2	-1.15	0.7604	0.03	1.02	0.9629
-0.94	-1.92	0.1242	0.02	1.01	0.9723	-0.99	-1.98	0.1097
1.31	2.47	0.0498	1.13	2.19	0.1041	2.01	4.02	0.0021
0.36	1.29	0.5836	0.6	1.52	0.3785	0.72	1.65	0.2717
1.27	2.42	0.0564	1.05	2.07	0.1333	2.03	4.09	0.0018
0.06	1.04	0.9355	0.78	1.71	0.2647	-0.13	-1.1	0.8527
0.17	1.13	0.7688	0.54	1.45	0.3702	0.29	1.22	0.625
0.19	1.14	0.7875	-0.37	-1.29	0.6179	0.07	1.05	0.9209
0.44	1.36	0.451	0.22	1.17	0.7243	0.62	1.53	0.2913
0.28	1.21	0.6524	0.8	1.74	0.2018	0.31	1.24	0.6191
0.6	1.52	0.3385	0.09	1.06	0.8999	1.22	2.33	0.0475
-0.33	-1.26	0.5991	-0.35	-1.27	0.5965	0.24	1.18	0.6934
0.4	1.32	0.5441	-0.75	-1.69	0.2907	0.97	1.96	0.1279
0.19	1.14	0.7638	0.58	1.5	0.3676	0.39	1.31	0.5274
-0.41	-1.33	0.494	-0.74	-1.67	0.2569	-0.13	-1.1	0.8265
-0.38	-1.3	0.5572	-0.67	-1.59	0.3346	0.49	1.4	0.4283
-0.03	-1.02	0.9563	0.09	1.07	0.8864	0.42	1.34	0.4824
-0.4	-1.32	0.5028	-1.74	-3.34	0.0117	-0.82	-1.76	0.1927
0.75	1.68	0.2258	-0.77	-1.7	0.2742	0.61	1.53	0.3275
1.54	2.9	0.0214	0.62	1.54	0.3815	0.4	1.32	0.5649
-0.03	-1.02	0.9584	-0.02	-1.01	0.9735	-0.31	-1.24	0.6145
1	2	0.1034	-0.5	-1.41	0.4753	1.25	2.37	0.0409
-0.02	-1.02	0.968	-1.05	-2.07	0.1263	-0.7	-1.62	0.2824
-0.63	-1.55	0.317	-0.58	-1.49	0.3796	-0.44	-1.36	0.4814
0.42	1.34	0.5048	0.75	1.69	0.2429	0.81	1.75	0.1923
-0.24	-1.18	0.6791	-0.51	-1.42	0.4199	-0.91	-1.88	0.1431
0.09	1.07	0.8863	0.68	1.6	0.3022	0.41	1.33	0.5234
-0.02	-1.01	0.974	-0.19	-1.14	0.7681	0.05	1.03	0.9372
0.31	1.24	0.6286	-0.32	-1.25	0.6493	0.99	1.98	0.1151
0.04	1.03	0.951	-0.79	-1.73	0.2616	0.03	1.02	0.9628
0.38	1.3	0.5295	-0.59	-1.5	0.3867	0.04	1.03	0.9514
0.57	1.48	0.3621	0.09	1.07	0.8914	-0.46	-1.38	0.4848
1.08	2.12	0.1097	0.94	1.92	0.1853	1.57	2.96	0.0187
0.62	1.54	0.3608	0.28	1.22	0.6937	1.74	3.35	0.0073
0.8	1.75	0.227	0.24	1.18	0.7316	0.41	1.33	0.5434
0.52	1.43	0.4232	1.61	3.06	0.0126	0.63	1.54	0.3353

0.46	1.38	0.489	1.1	2.14	0.105	1.48	2.78	0.0215
-0.45	-1.37	0.4399	-0.69	-1.61	0.2762	-0.25	-1.19	0.672
0.23	1.17	0.7203	1.04	2.06	0.0971	0.24	1.18	0.7101
-0.39	-1.31	0.4345	0.23	1.17	0.6596	-0.16	-1.12	0.7419
-0.07	-1.05	0.8305	0.32	1.25	0.3571	0.2	1.15	0.5417
0.29	1.22	0.6482	0.7	1.62	0.2882	0.95	1.93	0.1269
0.15	1.11	0.8057	-0.8	-1.74	0.2491	-0.3	-1.23	0.6398
-0.43	-1.35	0.5075	-0.79	-1.73	0.2512	-0.99	-1.98	0.1419
1.35	2.55	0.0503	1.49	2.8	0.036	1.92	3.79	0.0045
0.84	1.79	0.2261	1.2	2.3	0.0881	1.81	3.51	0.0068
1.41	2.66	0.0516	2.21	4.63	0.0024	2.15	4.45	0.0026
0.51	1.42	0.438	0.75	1.68	0.2654	1.47	2.76	0.0192
-0.08	-1.06	0.9028	-0.58	-1.5	0.3965	-0.44	-1.35	0.503
0.89	1.85	0.1731	0.89	1.85	0.1916	0.9	1.86	0.1686
-0.81	-1.75	0.1899	-1.76	-3.38	0.0105	-1.28	-2.42	0.0473
0.66	1.58	0.322	-0.67	-1.6	0.3515	-0.79	-1.73	0.2655
-0.04	-1.03	0.957	0.25	1.19	0.7359	0.29	1.23	0.695
-0.24	-1.19	0.701	-0.03	-1.02	0.9625	0.38	1.3	0.5396
0.06	1.05	0.9268	0.48	1.4	0.4942	0.32	1.25	0.6445
0.89	1.86	0.1771	-0.14	-1.11	0.8404	0.89	1.86	0.1787
-0.85	-1.8	0.1776	-1.09	-2.13	0.1065	-1.08	-2.11	0.0951
-1.13	-2.18	0.0822	0.01	1	0.9923	-0.21	-1.16	0.7303
-0.28	-1.21	0.668	-0.85	-1.8	0.221	0.15	1.11	0.8129
1.36	2.56	0.0496	-0.49	-1.41	0.5072	0.13	1.09	0.8578
0.58	1.49	0.4242	0.37	1.29	0.6193	1.1	2.15	0.126
0.04	1.03	0.9342	0.56	1.47	0.2509	0.18	1.14	0.6997
-0.04	-1.03	0.9509	-0.64	-1.56	0.3373	-0.44	-1.35	0.4902
0.75	1.68	0.2464	-0.34	-1.26	0.6346	0.05	1.04	0.9379
1.1	2.14	0.072	1.4	2.64	0.0265	1.64	3.12	0.0064
0.77	1.7	0.2969	1.29	2.44	0.0831	1.29	2.45	0.0756
-0.67	-1.59	0.3708	-0.01	-1.01	0.989	0.06	1.04	0.9339
-1.31	-2.48	0.0449	-1.62	-3.08	0.0188	-1.53	-2.9	0.0212
0.17	1.13	0.8055	-0.94	-1.91	0.2054	-0.62	-1.53	0.3944
1.02	2.03	0.1377	0.33	1.26	0.6531	1.95	3.87	0.0034
-0.6	-1.51	0.4072	-0.66	-1.58	0.3681	-1.56	-2.94	0.0342
-0.38	-1.3	0.566	-0.07	-1.05	0.922	-0.95	-1.93	0.1659
-0.1	-1.07	0.8731	-0.66	-1.58	0.3222	-0.71	-1.64	0.2688
-0.52	-1.43	0.4304	-0.98	-1.97	0.1628	-0.91	-1.88	0.1802
-0.34	-1.26	0.6231	1.18	2.26	0.0808	0.94	1.92	0.1513
-0.3	-1.23	0.6802	-1.39	-2.63	0.0602	-1.98	-3.94	0.0077
0.02	1.02	0.9727	0.31	1.24	0.6724	1.14	2.21	0.0957
0.8	1.74	0.2551	-0.13	-1.09	0.8647	0.75	1.68	0.2887
0.95	1.93	0.1767	0.88	1.85	0.2237	1.44	2.71	0.0389
-0.01	-1.01	0.9875	0.37	1.29	0.573	-0.45	-1.37	0.4958
-0.18	-1.13	0.7819	-0.92	-1.89	0.1873	-0.29	-1.22	0.6582
0.33	1.26	0.6297	0.24	1.18	0.7337	0.71	1.63	0.2995
0.06	1.04	0.9275	-0.67	-1.59	0.3467	0.17	1.12	0.8

0.37	1.29	0.6118	0.05	1.03	0.9513	-0.06	-1.05	0.9301
0.25	1.19	0.7026	-1.42	-2.68	0.05	-0.98	-1.97	0.1642
0.69	1.62	0.3439	1.45	2.73	0.0499	1.57	2.97	0.0295
-0.25	-1.19	0.7107	-0.88	-1.84	0.2218	-1.73	-3.31	0.0173
-0.46	-1.38	0.4812	-1.03	-2.04	0.1402	-1.97	-3.91	0.0052
0.28	1.21	0.707	-0.56	-1.47	0.4588	-0.62	-1.54	0.4054
1.69	3.24	0.0167	-0.16	-1.12	0.8274	1.17	2.25	0.1015
0.75	1.68	0.2594	1.23	2.34	0.069	0.23	1.17	0.7357
0.18	1.13	0.7997	1.09	2.13	0.1206	0.6	1.52	0.3833
-0.14	-1.1	0.8512	0.76	1.69	0.2922	0.1	1.07	0.8906
-0.3	-1.23	0.6857	2.14	4.4	0.0039	0.9	1.87	0.2221
-0.12	-1.09	0.8581	-1.29	-2.45	0.0733	-0.57	-1.48	0.4077
0.7	1.62	0.3478	2.17	4.52	0.0033	2.36	5.13	0.0012
-0.7	-1.62	0.3096	-0.26	-1.2	0.7069	-0.6	-1.51	0.3853
1.4	2.64	0.0382	0.94	1.91	0.1907	0.98	1.97	0.1588
-1.16	-2.23	0.0755	-1.61	-3.05	0.0204	-1.56	-2.95	0.0199
1.06	2.08	0.1385	1.16	2.24	0.1115	2.06	4.16	0.003
1.05	2.07	0.1413	0.6	1.52	0.4164	1.51	2.85	0.0324
1.61	3.05	0.0299	0.34	1.27	0.6459	1.38	2.6	0.0634
0.06	1.05	0.9282	0.89	1.85	0.2167	1.89	3.72	0.0051
0.14	1.1	0.8393	0.87	1.83	0.2037	-0.08	-1.05	0.9109
0.08	1.06	0.9103	1.25	2.37	0.0846	0.81	1.75	0.2535
0.35	1.27	0.6342	1.08	2.12	0.1386	1.29	2.44	0.07
0.62	1.54	0.3958	-0.26	-1.19	0.7322	1.87	3.65	0.0088
-1.02	-2.03	0.1451	-1.75	-3.36	0.0165	-1.75	-3.36	0.0152
0.76	1.7	0.2905	1.83	3.55	0.0114	0.76	1.7	0.2928
1.19	2.29	0.1056	2.59	6	4.00E-04	1.02	2.03	0.167
1.05	2.07	0.1567	1.28	2.42	0.0861	1.83	3.55	0.0135
-0.67	-1.59	0.3356	0.28	1.21	0.6979	0.05	1.04	0.9384
0.79	1.73	0.1792	0.44	1.36	0.4845	0.97	1.96	0.0984
0.75	1.68	0.3135	0.48	1.4	0.5174	1.16	2.23	0.1177
0.28	1.21	0.6483	0.16	1.12	0.8053	0.41	1.33	0.5013
1.27	2.41	0.0822	0.26	1.19	0.7188	1.2	2.3	0.0987
-0.15	-1.11	NA	1.64	3.11	NA	-0.13	-1.1	NA
0.19	1.14	0.8007	0.08	1.06	0.9131	0.06	1.04	0.9387
0.33	1.26	0.6557	1.29	2.44	0.0846	1.8	3.47	0.0146
1.11	2.15	0.0862	1.51	2.85	0.0223	1.56	2.95	0.0142
0.63	1.55	0.3664	1.1	2.15	0.1052	0.66	1.58	0.3435
0.11	1.08	0.8809	1.01	2.01	0.1705	1.72	3.29	0.0192
-0.34	-1.27	0.4731	0.77	1.71	0.1123	-0.73	-1.66	0.1375
0.2	1.15	0.7025	0.26	1.2	0.6478	0.4	1.32	0.4481
0.15	1.11	0.8003	0.52	1.44	0.407	0.73	1.66	0.2196
1.13	2.19	0.128	1.64	3.12	0.0274	1.18	2.27	0.1118
-0.28	-1.21	0.7019	-0.25	-1.19	0.7305	-0.25	-1.19	0.7286
0.31	1.24	0.6483	0.24	1.18	0.7162	0.26	1.19	0.7023
0	-1	0.9932	-0.83	-1.78	0.1522	0.04	1.03	0.9432
0.75	1.69	0.3138	0.41	1.33	0.5847	1.2	2.29	0.1098

0.36	1.29	0.5682	0.78	1.72	0.2307	-0.27	-1.2	0.6828
-0.67	-1.59	0.342	0.35	1.27	0.6248	-0.49	-1.4	0.4864
-0.68	-1.6	0.329	0.23	1.17	0.7439	-2.96	-7.76	0
0.47	1.39	0.5217	-0.13	-1.1	0.8598	0.63	1.54	0.3959
1.47	2.77	0.0473	0.81	1.76	0.2758	1.97	3.91	0.0078
0.23	1.17	0.7272	0.33	1.26	0.5983	-0.04	-1.03	0.9566
0.24	1.18	0.6999	-0.01	-1	0.9922	0.25	1.19	0.691
1.28	2.43	0.0697	1.11	2.16	0.1266	1.61	3.04	0.0221
0.76	1.69	0.285	1.31	2.48	0.0703	1.31	2.49	0.0624
2.78	6.87	1.00E-04	2.18	4.53	0.0026	0.63	1.55	0.3903
-0.36	-1.28	NA	0.34	1.26	NA	-0.41	-1.33	NA
0.46	1.37	0.4401	0.5	1.41	0.3815	0.39	1.31	0.5083
0.37	1.3	0.6169	0.46	1.37	0.5393	1.32	2.5	0.0763
0.61	1.53	NA	0	1	NA	-0.1	-1.07	NA
0.89	1.85	0.1015	0	1	0.9949	0.43	1.35	0.4234

S.a.GN.vs.I	S.a.GN.vs.I	S.a.GN.vs.I	VancATN.v	VancATN.v	VancATN.v	VancATN.v	VancATN.v	VancATN.v
0.72	1.64	0.0048	-0.12	-1.08	0.6352	-0.83	-1.78	0.0012
0.11	1.08	0.5484	-0.04	-1.03	0.8157	-0.15	-1.11	0.4207
-0.07	-1.05	0.7524	0.12	1.09	0.5141	0.19	1.14	0.3656
-0.35	-1.28	0.0852	0.36	1.29	0.0361	0.72	1.65	4.00E-04
-0.31	-1.24	0.1304	-0.03	-1.02	0.8633	0.28	1.21	0.1785
0.32	1.25	0.1302	0.29	1.23	0.1316	-0.03	-1.02	0.9025
-0.11	-1.08	0.6972	-0.56	-1.48	0.0436	-0.45	-1.37	0.1397
0.55	1.47	0.1018	0.14	1.1	0.6644	-0.41	-1.33	0.2195
0	1	0.9981	-0.1	-1.07	0.6163	-0.1	-1.07	0.6478
-0.11	-1.08	0.6707	-0.27	-1.2	0.2567	-0.16	-1.12	0.5386
1.1	2.14	8.00E-04	0.16	1.11	0.6183	-0.94	-1.92	0.0039
0.25	1.19	0.2761	-0.02	-1.02	0.9123	-0.27	-1.21	0.237
0.15	1.11	0.5234	-0.02	-1.01	0.9271	-0.17	-1.12	0.4748
-0.42	-1.33	0.0935	0.1	1.07	0.64	0.52	1.43	0.0379
-0.24	-1.18	0.3021	0	1	0.9973	0.24	1.18	0.3058
-0.29	-1.23	0.3032	0.27	1.2	0.2872	0.56	1.48	0.0471
0.41	1.33	0.191	-0.02	-1.01	0.9451	-0.43	-1.35	0.1721
-0.22	-1.17	0.4103	-0.27	-1.2	0.2871	-0.04	-1.03	0.8791
0.17	1.13	0.4628	0.25	1.19	0.2395	0.08	1.06	0.7322
-0.68	-1.6	0.0109	0.25	1.19	0.2566	0.93	1.9	5.00E-04
-0.2	-1.15	0.4404	-0.15	-1.11	0.5251	0.05	1.03	0.8504
0.34	1.26	0.1699	-0.09	-1.06	0.7076	-0.43	-1.35	0.0886
-0.42	-1.34	0.1194	-0.22	-1.17	0.3492	0.2	1.14	0.4798
-0.65	-1.57	0.0439	-0.37	-1.3	0.1973	0.28	1.21	0.4014
-0.92	-1.9	0.0055	-0.6	-1.52	0.0424	0.32	1.25	0.3492
-0.41	-1.33	0.105	-0.3	-1.23	0.177	0.1	1.07	0.6912
-0.22	-1.16	0.4257	-0.12	-1.09	0.6261	0.1	1.07	0.7266
-0.62	-1.54	0.0205	-0.19	-1.14	0.4077	0.43	1.35	0.1157
0.55	1.47	0.0181	0.2	1.15	0.3797	-0.35	-1.28	0.1253
-0.71	-1.64	0.0112	-0.19	-1.14	0.4217	0.52	1.43	0.0692
-0.92	-1.89	0.0023	-0.05	-1.03	0.8508	0.87	1.83	0.0042
-0.37	-1.3	0.1763	-0.17	-1.13	0.487	0.2	1.15	0.4725
0.05	1.03	0.8606	0.05	1.03	0.8504	0	-1	0.9979
0	-1	0.994	-0.45	-1.37	0.4301	-0.45	-1.36	0.4595
0.42	1.34	0.1043	0.01	1.01	0.9626	-0.41	-1.33	0.1172
-0.91	-1.88	0.007	-0.54	-1.46	0.0678	0.37	1.29	0.2935
0.34	1.27	0.2042	0.07	1.05	0.7712	-0.27	-1.2	0.322
0.73	1.65	0.0053	-0.1	-1.07	0.712	-0.82	-1.77	0.002
0.41	1.33	0.4848	-0.58	-1.5	0.3015	-0.99	-1.98	0.0929
-0.04	-1.03	0.8846	-0.04	-1.03	0.877	0	1	0.9971
-0.47	-1.39	0.1129	0.01	1.01	0.9581	0.49	1.4	0.1059
-0.08	-1.06	0.7744	-0.03	-1.02	0.9076	0.05	1.04	0.8582
0.27	1.21	0.6821	-0.66	-1.58	0.3046	-0.93	-1.91	0.1618

0.7	1.62	0.0162	0.2	1.15	0.4753	-0.5	-1.41	0.0842
0.7	1.63	0.0053	-0.3	-1.23	0.2375	-1	-2	1.00E-04
-1.17	-2.25	0.0015	-0.32	-1.25	0.3051	0.85	1.8	0.0235
-0.87	-1.83	0.0142	-0.23	-1.17	0.4454	0.64	1.56	0.0772
-0.36	-1.29	0.2109	-0.17	-1.12	0.5135	0.19	1.14	0.5125
0.05	1.03	0.8796	0.02	1.02	0.9323	-0.02	-1.02	0.9422
0.01	1.01	0.9593	0.14	1.1	0.6021	0.12	1.09	0.6732
-0.07	-1.05	0.8167	0.27	1.2	0.2929	0.33	1.26	0.2391
0.45	1.37	0.2133	-0.15	-1.11	0.6684	-0.6	-1.52	0.1022
-0.09	-1.07	0.7604	-0.32	-1.25	0.2586	-0.23	-1.17	0.4661
-0.47	-1.38	0.2781	0.52	1.43	0.1774	0.99	1.98	0.0205
-0.16	-1.12	0.6667	0.39	1.31	0.2339	0.55	1.46	0.13
0.37	1.29	0.2131	0.22	1.16	0.4372	-0.15	-1.11	0.6024
1.36	2.58	5.00E-04	1.25	2.37	8.00E-04	-0.12	-1.09	0.7484
0.12	1.09	0.7486	0.37	1.29	0.2845	0.25	1.19	0.5032
0.28	1.22	0.4548	0.07	1.05	0.8523	-0.22	-1.16	0.5682
-0.34	-1.27	0.358	-0.6	-1.52	0.0883	-0.26	-1.2	0.5115
-0.29	-1.22	0.3706	-0.08	-1.06	0.7703	0.21	1.15	0.5323
-0.12	-1.08	0.7112	-0.25	-1.19	0.3977	-0.13	-1.09	0.6865
-0.48	-1.4	0.2559	-0.54	-1.45	0.1732	-0.06	-1.04	0.8978
-0.01	-1.01	0.9647	-0.01	-1.01	0.9614	0	-1	0.9998
-0.43	-1.35	0.1932	-1.22	-2.34	3.00E-04	-0.79	-1.73	0.0353
0.15	1.11	0.6536	-0.05	-1.04	0.8603	-0.2	-1.15	0.5443
-0.72	-1.64	0.0436	-0.04	-1.03	0.8967	0.68	1.6	0.0589
0.25	1.19	0.4273	0.28	1.22	0.3196	0.04	1.03	0.9016
-0.11	-1.08	0.7447	-0.16	-1.11	0.6337	-0.04	-1.03	0.9104
0.04	1.03	0.9151	-0.12	-1.09	0.7023	-0.16	-1.12	0.6492
0.14	1.1	0.8014	0.76	1.7	0.1352	0.63	1.54	0.2468
-0.32	-1.25	0.3737	0	-1	0.9931	0.32	1.25	0.382
-0.17	-1.12	0.6153	-0.12	-1.09	0.6934	0.05	1.03	0.8891
0.02	1.02	0.9389	-0.11	-1.08	0.7163	-0.13	-1.09	0.6834
-0.57	-1.49	0.1238	-0.32	-1.24	0.3417	0.25	1.19	0.5035
0.6	1.51	0.0667	0.04	1.03	0.8971	-0.56	-1.47	0.09
0.21	1.16	0.5332	0.17	1.13	0.5795	-0.04	-1.03	0.9125
0.05	1.04	0.8752	0.05	1.04	0.8574	0	1	0.9935
0.29	1.22	0.3608	-0.33	-1.26	0.2856	-0.62	-1.54	0.0595
0.03	1.02	0.9138	0.1	1.07	0.7413	0.06	1.04	0.8474
-2.37	-5.15	1.00E-04	-0.53	-1.44	0.2917	1.84	3.58	0.0021
-0.07	-1.05	0.8544	-0.19	-1.14	0.5759	-0.12	-1.09	0.7418
-2.69	-6.47	0	-0.91	-1.88	0.0223	1.78	3.45	0.001
-0.23	-1.18	0.5003	-0.16	-1.11	0.6227	0.08	1.06	0.8242
-0.35	-1.27	0.3334	0.11	1.08	0.7262	0.46	1.38	0.204
0.43	1.35	0.1763	0.15	1.11	0.6084	-0.27	-1.21	0.3853
0.63	1.55	0.2469	0.31	1.24	0.5498	-0.32	-1.25	0.5555
-0.37	-1.3	0.3011	-0.17	-1.13	0.589	0.2	1.15	0.5901
-1.26	-2.39	0.0043	-0.48	-1.4	0.1942	0.78	1.71	0.0883
-0.64	-1.56	0.1034	-0.06	-1.04	0.8631	0.58	1.5	0.1433

-0.24	-1.18	0.4964	0.02	1.02	0.9429	0.26	1.2	0.4591
-0.27	-1.21	0.4265	-0.06	-1.04	0.8445	0.21	1.16	0.5411
-0.56	-1.48	0.1185	-0.06	-1.04	0.8522	0.51	1.42	0.1665
-0.14	-1.1	0.7188	0.47	1.39	0.1583	0.61	1.52	0.1008
0.75	1.69	0.2964	-1.57	-2.97	0.028	-2.32	-5.01	0.0014
-0.24	-1.18	0.5152	-0.14	-1.1	0.6805	0.1	1.07	0.7862
-0.44	-1.35	0.2715	-0.16	-1.12	0.6482	0.27	1.21	0.4975
-1.4	-2.64	0.0067	-0.19	-1.14	0.6656	1.21	2.31	0.0205
-0.39	-1.31	0.2714	-0.13	-1.1	0.6787	0.26	1.2	0.4726
-0.96	-1.95	0.0236	0.09	1.06	0.8006	1.05	2.07	0.0137
0.36	1.28	0.3447	0.32	1.25	0.3568	-0.03	-1.02	0.93
-0.66	-1.58	0.0837	-0.21	-1.16	0.514	0.45	1.36	0.2549
0.42	1.33	0.2484	-0.36	-1.28	0.322	-0.77	-1.71	0.0401
0.54	1.45	0.1456	0.54	1.45	0.1183	0	-1	0.9986
-0.42	-1.34	0.336	-0.1	-1.07	0.8021	0.32	1.25	0.4671
-0.19	-1.14	0.6249	-0.5	-1.42	0.1785	-0.31	-1.24	0.4506
-0.45	-1.36	0.2439	-0.14	-1.1	0.6823	0.31	1.24	0.4307
-0.21	-1.16	0.6313	0.17	1.12	0.677	0.38	1.3	0.3886
1.16	2.23	0.0325	0.53	1.44	0.3092	-0.63	-1.54	0.2402
-0.2	-1.15	0.5613	0.14	1.1	0.6423	0.34	1.27	0.3206
-1.58	-2.99	0.0015	-0.41	-1.33	0.3188	1.17	2.26	0.0207
0.02	1.01	0.9597	0.04	1.03	0.8957	0.03	1.02	0.9444
-0.67	-1.59	0.1007	-0.24	-1.18	0.4933	0.42	1.34	0.3095
0.37	1.29	0.314	-0.18	-1.14	0.6104	-0.56	-1.47	0.1432
-0.24	-1.18	0.5186	-0.14	-1.11	0.6727	0.1	1.07	0.7978
-0.27	-1.21	0.4998	0	-1	0.9993	0.27	1.21	0.5041
-0.13	-1.09	0.7387	-0.16	-1.11	0.653	-0.03	-1.02	0.9358
0.32	1.25	0.1472	0.02	1.02	0.9048	-0.29	-1.22	0.1847
0.47	1.39	0.1884	0.56	1.47	0.0928	0.08	1.06	0.808
-0.03	-1.02	0.9255	0.14	1.1	0.5777	0.16	1.12	0.552
0.35	1.27	0.3374	-0.06	-1.05	0.8532	-0.41	-1.33	0.2632
0.25	1.19	0.5118	-0.01	-1	0.9877	-0.25	-1.19	0.5064
0.37	1.3	0.319	0.13	1.09	0.7238	-0.25	-1.19	0.5084
-1.22	-2.32	0.0069	-0.18	-1.13	0.6282	1.04	2.05	0.0231
-0.85	-1.8	0.063	-0.62	-1.54	0.1295	0.22	1.17	0.6376
-0.32	-1.25	0.4323	0	1	0.9969	0.32	1.25	0.4342
0.13	1.1	0.8542	-0.5	-1.41	0.4901	-0.63	-1.55	0.3884
-0.6	-1.51	0.1528	-1.02	-2.03	0.0117	-0.42	-1.34	0.3566
-0.79	-1.73	0.0706	-0.16	-1.11	0.6729	0.63	1.55	0.1547
1.07	2.09	0.0095	1.09	2.13	0.0046	0.03	1.02	0.9437
-0.29	-1.22	0.4733	0.06	1.04	0.8656	0.35	1.28	0.3882
-0.22	-1.16	0.5816	0.35	1.27	0.3038	0.57	1.48	0.1416
-1.25	-2.38	0.0061	-0.82	-1.76	0.039	0.43	1.35	0.3689
0.49	1.4	0.1941	-0.14	-1.1	0.7017	-0.63	-1.54	0.1014
0.49	1.4	0.1865	0.29	1.22	0.4078	-0.2	-1.15	0.5848
1.23	2.35	0.0028	0.33	1.26	0.4117	-0.9	-1.86	0.0267
0.21	1.15	0.5829	-0.06	-1.04	0.861	-0.27	-1.21	0.4803

-0.06	-1.04	0.8781	0.1	1.07	0.7904	0.16	1.12	0.6933
-0.8	-1.74	0.0554	-0.26	-1.2	0.4711	0.54	1.45	0.2062
-0.18	-1.13	0.6527	0	-1	0.99	0.17	1.13	0.6641
1.35	2.54	0.0219	1.16	2.24	0.0384	-0.18	-1.14	0.7487
-0.95	-1.93	0.0419	-0.13	-1.09	0.7496	0.82	1.77	0.0817
0.15	1.11	0.6128	-0.01	-1.01	0.9781	-0.15	-1.11	0.5992
-0.42	-1.33	0.3268	0.31	1.24	0.3874	0.73	1.66	0.0814
0.09	1.07	0.8125	0.14	1.1	0.6904	0.05	1.03	0.8972
-1.09	-2.13	0.0336	-0.05	-1.03	0.912	1.04	2.06	0.0437
-1.32	-2.5	0.0056	-0.59	-1.51	0.1369	0.73	1.66	0.1423
-0.96	-1.95	0.031	-0.28	-1.21	0.4592	0.69	1.61	0.1346
1.24	2.36	0.0036	-0.43	-1.35	0.3285	-1.67	-3.18	2.00E-04
-0.15	-1.11	0.7	-0.25	-1.19	0.4837	-0.1	-1.07	0.7959
-0.46	-1.38	0.2658	0.14	1.11	0.6899	0.61	1.52	0.1436
0.18	1.14	0.638	-0.18	-1.13	0.6325	-0.36	-1.28	0.3648
0.21	1.16	0.6137	-0.07	-1.05	0.8672	-0.28	-1.21	0.5125
-0.67	-1.59	0.1033	-0.01	-1.01	0.9784	0.66	1.58	0.1114
0.79	1.73	0.0583	-0.06	-1.04	0.884	-0.85	-1.81	0.0448
-0.49	-1.4	0.2608	0.02	1.02	0.9502	0.51	1.42	0.2414
0.56	1.47	0.4567	0.22	1.17	0.7665	-0.33	-1.26	0.6551
0.17	1.12	0.6457	-0.14	-1.1	0.6945	-0.31	-1.24	0.4148
-1.39	-2.62	0.0066	-0.12	-1.09	0.7779	1.27	2.41	0.0139
0.69	1.61	0.0418	0.5	1.41	0.1191	-0.19	-1.14	0.5614
0.01	1.01	0.9756	0.01	1.01	0.9697	0	1	0.9965
-1.76	-3.38	0.0029	0.04	1.03	0.9347	1.8	3.48	0.0024
0.31	1.24	0.4599	0.1	1.07	0.7949	-0.2	-1.15	0.6229
0.15	1.11	0.7056	0	1	0.9955	-0.15	-1.11	0.7124
-0.86	-1.82	0.0509	0.04	1.03	0.9157	0.9	1.87	0.0419
0.23	1.17	0.5883	-0.13	-1.1	0.7468	-0.36	-1.29	0.4029
-0.05	-1.03	0.918	-0.36	-1.29	0.3885	-0.32	-1.25	0.4846
-1.45	-2.73	0.0034	-0.36	-1.28	0.383	1.09	2.13	0.0311
-0.62	-1.53	0.1971	0.38	1.31	0.3394	1	2	0.0334
0.2	1.15	0.6546	0.15	1.11	0.7207	-0.05	-1.04	0.9105
-0.16	-1.12	0.7029	0.36	1.28	0.3418	0.52	1.43	0.2163
0.89	1.86	0.0247	0.2	1.15	0.6189	-0.7	-1.62	0.0774
-0.95	-1.93	0.048	-0.07	-1.05	0.8528	0.87	1.83	0.0716
0.43	1.35	0.0533	0.11	1.08	0.5938	-0.32	-1.25	0.1548
1.21	2.32	0.0024	-0.4	-1.32	0.336	-1.61	-3.05	1.00E-04
-0.46	-1.38	0.3039	-0.05	-1.04	0.8987	0.41	1.33	0.3656
-0.24	-1.18	0.5546	0.11	1.08	0.7534	0.35	1.27	0.3851
0.31	1.24	0.45	-0.16	-1.12	0.691	-0.47	-1.39	0.2649
-0.53	-1.44	0.2089	0.06	1.04	0.8702	0.59	1.5	0.1641
0.03	1.02	0.9506	-0.18	-1.13	0.6619	-0.2	-1.15	0.6418
0.43	1.34	0.267	-0.18	-1.13	0.6327	-0.61	-1.52	0.1244
0.32	1.24	0.4341	0.02	1.02	0.9526	-0.29	-1.22	0.472
0.53	1.45	0.2082	0.7	1.63	0.0697	0.17	1.13	0.6667
0.5	1.42	0.2319	0.42	1.34	0.2838	-0.08	-1.06	0.8463

-0.58	-1.5	0.3148	-1.06	-2.08	0.0595	-0.48	-1.39	0.431
-0.82	-1.77	0.0877	0.05	1.04	0.894	0.87	1.83	0.0697
-0.46	-1.38	0.2705	-0.82	-1.77	0.0417	-0.36	-1.28	0.4284
-0.23	-1.17	0.5852	-0.2	-1.15	0.6054	0.03	1.02	0.9445
-2.08	-4.24	1.00E-04	-0.31	-1.24	0.447	1.77	3.42	0.0015
0.37	1.29	0.3628	-0.2	-1.15	0.6172	-0.57	-1.49	0.174
-1.09	-2.13	0.0255	-0.56	-1.48	0.1822	0.53	1.44	0.3018
-0.61	-1.52	0.1848	-0.34	-1.27	0.4062	0.27	1.2	0.5722
-0.58	-1.5	0.2017	-0.23	-1.17	0.571	0.36	1.28	0.4491
0.06	1.04	0.9153	-0.38	-1.3	0.4998	-0.44	-1.36	0.4555
0.64	1.56	0.1495	0.71	1.64	0.0866	0.07	1.05	0.8713
-0.32	-1.25	0.5037	-0.06	-1.04	0.8963	0.26	1.2	0.5864
-0.28	-1.21	0.5283	-0.09	-1.06	0.8258	0.19	1.14	0.671
-1.3	-2.46	0.0209	-0.22	-1.16	0.6578	1.08	2.12	0.0572
-1.22	-2.32	0.0149	-0.34	-1.27	0.4057	0.87	1.83	0.0892
0.75	1.69	0.0651	0.32	1.25	0.4223	-0.44	-1.35	0.2776
0.18	1.13	0.6714	-0.29	-1.22	0.4804	-0.46	-1.38	0.2844
0.19	1.14	0.7007	-0.34	-1.26	0.4753	-0.52	-1.43	0.2948
0.36	1.29	0.39	-0.01	-1	0.9878	-0.37	-1.29	0.3868
-0.31	-1.24	0.4797	-0.47	-1.38	0.2567	-0.16	-1.12	0.7321
0.42	1.34	0.3298	0.21	1.15	0.6174	-0.22	-1.16	0.6147
0.43	1.35	0.2893	-0.18	-1.14	0.6466	-0.61	-1.53	0.1417
0.57	1.48	0.1632	0.18	1.13	0.6466	-0.39	-1.31	0.3386
-0.16	-1.12	0.7167	-0.87	-1.83	0.0495	-0.71	-1.64	0.1394
0.1	1.07	0.8231	0.19	1.14	0.6384	0.09	1.07	0.8337
0.48	1.39	0.1915	-0.59	-1.51	0.1185	-1.07	-2.1	0.0064
0.65	1.57	0.122	-0.04	-1.03	0.9285	-0.69	-1.61	0.1065
0.48	1.4	0.2917	-0.05	-1.03	0.9134	-0.53	-1.45	0.2517
-0.21	-1.16	0.6291	-0.11	-1.08	0.7836	0.1	1.07	0.8197
-0.4	-1.32	0.3921	0.65	1.57	0.0913	1.05	2.08	0.0193
0.06	1.04	0.9209	0.65	1.57	0.2501	0.59	1.51	0.3215
0.32	1.25	0.4508	-0.05	-1.04	0.8949	-0.38	-1.3	0.3855
-1.07	-2.09	0.0324	-0.41	-1.33	0.3327	0.65	1.57	0.204
1	2	0.0343	0.41	1.33	0.3684	-0.59	-1.5	0.2053
-0.15	-1.11	0.7412	0.24	1.18	0.5529	0.38	1.3	0.387
0.06	1.04	0.8831	0.05	1.03	0.8923	-0.01	-1.01	0.9829
0.32	1.25	0.4901	0.11	1.08	0.7988	-0.21	-1.16	0.6543
-0.23	-1.17	0.6259	0.07	1.05	0.8681	0.3	1.23	0.5256
-1.05	-2.07	0.0472	-0.08	-1.05	0.8667	0.97	1.97	0.068
-0.15	-1.11	0.7292	-0.02	-1.01	0.9582	0.13	1.1	0.768
-1.08	-2.12	0.0507	-0.27	-1.21	0.5694	0.81	1.75	0.1515
-1.47	-2.78	0.0186	-0.04	-1.03	0.9365	1.43	2.7	0.023
-1.32	-2.5	0.0308	-0.45	-1.37	0.4209	0.87	1.83	0.16
-0.14	-1.1	0.7716	0.51	1.42	0.2103	0.65	1.57	0.1549
-0.44	-1.35	0.3297	-0.25	-1.19	0.536	0.19	1.14	0.6864
0.13	1.09	0.7897	0.38	1.3	0.3934	0.25	1.19	0.6021
-0.77	-1.7	0.1377	0.32	1.25	0.4573	1.09	2.12	0.0336

0.48	1.39	0.2895	0.54	1.46	0.192	0.07	1.05	0.8741
-0.57	-1.49	0.2138	0.23	1.17	0.5569	0.8	1.74	0.0795
0.53	1.44	0.2441	-0.48	-1.4	0.299	-1.01	-2.02	0.0346
-0.08	-1.06	0.8637	-0.38	-1.3	0.4038	-0.3	-1.23	0.5462
-0.38	-1.31	0.4363	-0.17	-1.12	0.7103	0.22	1.16	0.6656
0.23	1.17	0.6147	-0.58	-1.5	0.2021	-0.81	-1.75	0.0922
-0.06	-1.04	0.8957	0.66	1.58	0.1056	0.72	1.65	0.1091
-0.34	-1.26	0.4797	-0.63	-1.54	0.1702	-0.29	-1.22	0.568
-0.26	-1.2	0.554	-0.73	-1.66	0.0856	-0.47	-1.39	0.3128
0.51	1.43	0.3128	0.4	1.32	0.4067	-0.11	-1.08	0.8206
-0.2	-1.15	0.6832	0.42	1.34	0.3188	0.62	1.54	0.1892
-0.09	-1.07	0.8648	0.22	1.16	0.6629	0.31	1.24	0.5647
-0.36	-1.28	0.6114	0.55	1.47	0.4176	0.91	1.88	0.1957
-0.15	-1.11	0.7491	-0.47	-1.38	0.2999	-0.32	-1.25	0.5202
-0.01	-1	0.9868	0.11	1.08	0.7711	0.12	1.09	0.7746
-0.43	-1.35	0.3658	-0.03	-1.02	0.9351	0.4	1.32	0.4105
-0.61	-1.52	0.2172	-0.39	-1.31	0.3801	0.22	1.16	0.6713
0.77	1.7	0.1787	1.63	3.1	0.0019	0.86	1.82	0.1084
-0.29	-1.22	0.5289	-0.37	-1.29	0.3908	-0.08	-1.06	0.8689
0.22	1.17	0.6389	-0.14	-1.11	0.7497	-0.37	-1.29	0.4464
-1.41	-2.66	0.0146	-0.69	-1.61	0.1639	0.72	1.65	0.2337
-0.1	-1.07	0.8468	-0.01	-1.01	0.9807	0.08	1.06	0.8657
0.71	1.64	0.0942	0.06	1.04	0.8934	-0.66	-1.57	0.1255
-0.51	-1.43	0.3079	-0.31	-1.24	0.4986	0.2	1.15	0.6956
0.32	1.25	0.4749	0.32	1.25	0.4484	0	-1	0.994
0.97	1.95	0.0657	1.24	2.37	0.0107	0.28	1.21	0.5642
-0.12	-1.08	0.8033	0.24	1.18	0.5639	0.35	1.28	0.4391
-0.68	-1.6	0.1844	0.04	1.03	0.9313	0.72	1.65	0.1631
0.37	1.29	0.4472	0.58	1.5	0.1942	0.21	1.16	0.651
0.38	1.3	0.3989	-0.42	-1.34	0.3517	-0.8	-1.74	0.0903
0.42	1.34	0.2517	0.07	1.05	0.8374	-0.35	-1.28	0.3445
0.45	1.36	0.313	-0.11	-1.08	0.7933	-0.56	-1.48	0.2152
-1.01	-2.01	0.0558	-0.74	-1.67	0.1178	0.27	1.2	0.6299
0.4	1.32	0.3962	0.56	1.47	0.206	0.15	1.11	0.7392
-0.03	-1.02	0.9483	0.48	1.39	0.2719	0.51	1.42	0.2847
-1.35	-2.56	0.0152	-0.41	-1.33	0.3861	0.95	1.93	0.0983
0.06	1.04	0.896	0.15	1.11	0.7336	0.09	1.06	0.8539
0.22	1.17	0.6493	0.65	1.57	0.139	0.43	1.35	0.3608
-0.44	-1.36	0.3799	0.24	1.18	0.5897	0.68	1.6	0.1746
1.16	2.24	0.0284	-0.24	-1.18	0.6672	-1.4	-2.64	0.0103
-1.15	-2.22	0.05	0.04	1.03	0.9409	1.19	2.28	0.0435
0.89	1.86	0.098	0.38	1.3	0.4663	-0.51	-1.43	0.3362
0.35	1.28	0.4381	0.21	1.16	0.6274	-0.14	-1.11	0.7498
-0.11	-1.08	0.8301	-0.2	-1.15	0.6761	-0.09	-1.07	0.861
-0.51	-1.43	0.3589	-0.04	-1.02	0.9435	0.48	1.39	0.3975
0.98	1.98	0.0634	0.53	1.44	0.306	-0.46	-1.38	0.3756
-0.5	-1.41	0.3462	-0.45	-1.37	0.3531	0.04	1.03	0.9377

-0.22	-1.17	0.6554	0.01	1	0.9893	0.23	1.17	0.6491
1.07	2.1	0.004	-0.21	-1.16	0.5816	-1.28	-2.43	8.00E-04
0.39	1.31	0.4072	0.05	1.03	0.9138	-0.34	-1.27	0.4713
-0.08	-1.05	0.8747	0.04	1.03	0.9345	0.11	1.08	0.8165
-0.92	-1.9	0.0966	0.35	1.28	0.4438	1.28	2.42	0.0202
-0.63	-1.55	0.2579	0.05	1.03	0.9238	0.68	1.6	0.2253
0.4	1.32	0.389	-0.16	-1.12	0.7223	-0.57	-1.48	0.2372
-0.04	-1.03	0.9373	0.06	1.05	0.8815	0.1	1.07	0.8299
-0.02	-1.01	0.968	-0.29	-1.22	0.5542	-0.27	-1.21	0.6096
-0.59	-1.5	0.3368	-0.03	-1.02	0.9537	0.56	1.47	0.3674
0.76	1.69	0.1465	0.25	1.19	0.6203	-0.5	-1.42	0.3283
-1.27	-2.41	0.0246	-0.11	-1.08	0.8073	1.15	2.23	0.043
-1.2	-2.29	0.0256	0.21	1.15	0.6277	1.4	2.64	0.0085
-0.13	-1.09	0.8025	0.36	1.28	0.4347	0.48	1.4	0.3364
0.22	1.17	0.6367	-0.17	-1.13	0.6999	-0.4	-1.32	0.4094
0.27	1.21	0.5565	-0.46	-1.38	0.3227	-0.74	-1.67	0.1339
0.18	1.13	0.6982	0.11	1.08	0.8028	-0.07	-1.05	0.8781
0.35	1.28	0.4484	0.23	1.17	0.6047	-0.13	-1.09	0.786
0.03	1.02	0.9556	-0.36	-1.29	0.4431	-0.39	-1.31	0.443
0.45	1.37	0.3527	0.69	1.61	0.1205	0.24	1.18	0.6033
-1.16	-2.24	0.0085	-0.47	-1.39	0.1978	0.69	1.61	0.1333
0.3	1.23	0.5324	-0.75	-1.68	0.1307	-1.04	-2.06	0.0427
-1.44	-2.72	0.0073	-0.64	-1.56	0.1597	0.8	1.74	0.1523
-1.15	-2.22	0.0431	-0.59	-1.5	0.2472	0.56	1.48	0.3378
0.58	1.5	0.2367	0.44	1.36	0.3412	-0.14	-1.1	0.7737
-0.3	-1.23	0.5547	-0.06	-1.05	0.8882	0.24	1.18	0.6478
-0.44	-1.36	0.3813	0.41	1.33	0.3412	0.85	1.8	0.0859
0.03	1.02	0.9563	-0.29	-1.23	0.5241	-0.32	-1.25	0.5183
0.57	1.48	0.2229	-0.78	-1.72	0.1161	-1.35	-2.56	0.0079
-0.29	-1.23	0.585	-0.37	-1.3	0.4637	-0.08	-1.06	0.8888
-0.42	-1.34	0.4988	0.73	1.66	0.1847	1.15	2.22	0.0574
0.6	1.52	0.2423	0.81	1.75	0.094	0.2	1.15	0.6829
-1.8	-3.49	0.0072	-0.44	-1.36	0.4498	1.36	2.58	0.0473
0.32	1.25	0.5384	-0.22	-1.16	0.6704	-0.54	-1.45	0.3144
0.81	1.75	0.0916	-0.34	-1.27	0.4871	-1.15	-2.22	0.0211
0.79	1.73	0.1522	0.86	1.81	0.0995	0.07	1.05	0.898
-0.73	-1.65	0.1843	0.37	1.29	0.4224	1.09	2.14	0.0423
0.31	1.24	0.5791	0.55	1.47	0.2802	0.25	1.19	0.6459
-0.36	-1.28	0.4834	-0.15	-1.11	0.7479	0.21	1.16	0.6889
0.12	1.09	0.8102	-0.19	-1.14	0.69	-0.31	-1.24	0.5426
-0.72	-1.65	0.1474	-0.02	-1.01	0.9702	0.7	1.63	0.1599
-0.07	-1.05	0.8904	0.25	1.19	0.5912	0.32	1.25	0.5277
-0.17	-1.13	0.7253	0.23	1.17	0.6065	0.4	1.32	0.4128
-1.51	-2.86	0.008	-0.87	-1.82	0.0804	0.65	1.57	0.2821
0.39	1.31	0.4374	-0.03	-1.02	0.95	-0.43	-1.34	0.4078
-0.92	-1.89	0.0107	0.04	1.03	0.9045	0.96	1.94	0.0082
0.94	1.92	0.0706	0.72	1.64	0.1508	-0.22	-1.17	0.6515

0.77	1.71	0.1213	0.07	1.05	0.8931	-0.71	-1.63	0.1582
-0.36	-1.28	0.4918	-0.99	-1.99	0.0553	-0.63	-1.55	0.2612
-0.73	-1.65	0.1986	-0.34	-1.27	0.5044	0.38	1.3	0.5073
0.22	1.16	0.6522	0.11	1.08	0.8042	-0.1	-1.08	0.8284
0.78	1.72	0.1366	0.86	1.82	0.0805	0.08	1.06	0.8725
-0.59	-1.51	0.2953	0.24	1.18	0.625	0.83	1.78	0.139
0.36	1.28	0.4679	-0.15	-1.11	0.7493	-0.51	-1.43	0.3092
-1.84	-3.59	0.004	-1.21	-2.31	0.0373	0.64	1.55	0.3473
-0.09	-1.07	0.8652	0.32	1.25	0.516	0.42	1.34	0.4422
0.23	1.17	0.6402	-0.02	-1.02	0.9639	-0.25	-1.19	0.6136
-0.07	-1.05	0.8973	0.6	1.52	0.2237	0.67	1.59	0.2122
-1.06	-2.08	0.0973	0.33	1.26	0.558	1.39	2.62	0.0282
0.54	1.45	0.3237	0.08	1.06	0.8845	-0.46	-1.38	0.3997
-0.41	-1.33	0.433	-0.06	-1.04	0.8964	0.35	1.27	0.5101
-0.33	-1.26	0.5935	-0.63	-1.55	0.2953	-0.3	-1.23	0.6425
0.68	1.61	0.263	1.9	3.74	6.00E-04	1.22	2.33	0.0321
0.26	1.2	0.5867	0.31	1.24	0.4863	0.05	1.04	0.9157
0.34	1.27	0.4924	-0.03	-1.02	0.9435	-0.38	-1.3	0.4558
-0.31	-1.24	0.5639	-0.74	-1.67	0.1535	-0.43	-1.35	0.4438
0.48	1.4	0.346	-0.29	-1.22	0.5809	-0.77	-1.7	0.1484
-0.18	-1.13	0.7623	0.47	1.39	0.3758	0.66	1.58	0.2629
0.07	1.05	0.9109	0.32	1.25	0.5853	0.25	1.19	0.6853
0.02	1.01	0.9693	0.06	1.04	0.8999	0.04	1.03	0.9374
-0.28	-1.21	0.6301	-0.28	-1.21	0.6047	0	-1	0.996
0.46	1.37	0.3909	0	1	0.9954	-0.45	-1.37	0.3983
-0.35	-1.27	0.5266	-0.27	-1.2	0.5985	0.08	1.06	0.8881
-1.39	-2.63	0.015	-0.59	-1.5	0.233	0.8	1.75	0.1752
0.11	1.08	0.8205	0.33	1.26	0.4644	0.22	1.16	0.6521
0.07	1.05	0.9104	0.67	1.59	0.2037	0.61	1.52	0.2851
1.13	2.19	0.0238	0.42	1.33	0.3986	-0.72	-1.64	0.1442
0.16	1.11	0.767	0.48	1.4	0.3181	0.33	1.25	0.5284
-0.42	-1.34	0.4388	-0.11	-1.08	0.8285	0.31	1.24	0.5691
0.07	1.05	0.894	0.04	1.03	0.9412	-0.03	-1.02	0.9493
-0.51	-1.43	0.3771	0.08	1.06	0.875	0.59	1.51	0.3065
0.35	1.27	0.5196	0.08	1.05	0.885	-0.27	-1.21	0.6145
-0.14	-1.1	0.817	-0.11	-1.08	0.8454	0.03	1.02	0.9629
0.28	1.21	0.609	0	1	0.9957	-0.27	-1.21	0.6157
-0.16	-1.12	0.7601	0.17	1.12	0.7337	0.33	1.26	0.5381
-0.31	-1.24	0.5599	-0.22	-1.17	0.6484	0.09	1.06	0.8753
-0.9	-1.87	0.1283	-0.5	-1.42	0.348	0.4	1.32	0.518
-0.35	-1.27	0.5525	-0.64	-1.56	0.2524	-0.3	-1.23	0.6262
1.11	2.15	0.0997	0.01	1	0.9923	-1.1	-2.14	0.1023
2.32	4.98	2.00E-04	0.22	1.17	0.7302	-2.09	-4.27	6.00E-04
0.03	1.02	0.9501	-0.4	-1.32	0.4445	-0.44	-1.35	0.4371
0.1	1.07	0.8464	0.15	1.11	0.7595	0.05	1.03	0.927
-0.7	-1.62	0.2223	-0.41	-1.33	0.4365	0.29	1.22	0.6212
-1.14	-2.21	0.1001	-0.1	-1.07	0.8788	1.04	2.06	0.1349

-1.06	-2.09	0.0846	0.19	1.14	0.731	1.25	2.37	0.0422
0.34	1.26	0.5613	0.19	1.14	0.7378	-0.15	-1.11	0.7934
0.16	1.11	0.7505	-0.76	-1.69	0.1311	-0.91	-1.88	0.0841
0.47	1.38	0.3855	0.31	1.24	0.547	-0.16	-1.12	0.766
0.62	1.54	0.2379	-0.52	-1.44	0.3386	-1.14	-2.21	0.0397
0.19	1.14	0.7135	-0.26	-1.2	0.615	-0.45	-1.37	0.4045
0.37	1.29	0.5157	0.11	1.08	0.8353	-0.25	-1.19	0.6532
1.07	2.1	0.0592	0.02	1.02	0.9687	-1.05	-2.07	0.0662
0.28	1.21	0.6204	0.74	1.67	0.1502	0.46	1.38	0.3945
0.97	1.96	0.0975	0.54	1.46	0.3382	-0.42	-1.34	0.4564
-0.14	-1.1	0.7906	-0.1	-1.08	0.8248	0.03	1.02	0.9532
-0.86	-1.81	0.1233	-0.28	-1.21	0.5725	0.58	1.5	0.3073
-1.1	-2.14	0.0551	-0.38	-1.3	0.4497	0.72	1.65	0.2194
0.59	1.51	0.2858	-0.19	-1.14	0.7353	-0.78	-1.71	0.1694
0.13	1.09	0.809	0.22	1.17	0.6563	0.09	1.07	0.8615
-0.76	-1.69	0.2286	-0.08	-1.05	0.8936	0.68	1.6	0.2829
-0.31	-1.24	0.5657	-0.03	-1.02	0.9509	0.28	1.21	0.608
0.19	1.14	0.7232	-0.58	-1.49	0.2955	-0.77	-1.71	0.1829
0.68	1.61	0.2293	0.54	1.45	0.3219	-0.15	-1.11	0.7929
-0.65	-1.57	0.2932	-1.29	-2.44	0.0363	-0.63	-1.55	0.3371
0.1	1.07	0.865	0.62	1.54	0.2361	0.52	1.44	0.3508
-1.75	-3.35	0.0063	-0.39	-1.31	0.4652	1.35	2.55	0.0385
-1.41	-2.66	0.0283	0.25	1.19	0.6333	1.67	3.18	0.0092
-0.08	-1.06	0.8881	0.11	1.08	0.8361	0.18	1.13	0.7406
0.91	1.88	0.1189	0.47	1.38	0.4081	-0.44	-1.36	0.4409
-0.76	-1.7	0.2206	-0.08	-1.06	0.8848	0.68	1.61	0.2786
-0.87	-1.83	0.1923	-0.66	-1.58	0.3007	0.22	1.16	0.7553
-0.82	-1.77	0.1565	-0.63	-1.55	0.2364	0.19	1.14	0.7572
-2.03	-4.08	0.0035	-0.64	-1.56	0.3117	1.39	2.62	0.0503
-0.19	-1.14	0.7323	-0.27	-1.21	0.5911	-0.09	-1.06	0.8725
0.09	1.06	0.8848	-0.73	-1.66	0.2257	-0.81	-1.76	0.197
0.23	1.18	0.6804	0.22	1.16	0.6849	-0.02	-1.01	0.9768
0.59	1.5	0.28	0.36	1.29	0.4831	-0.22	-1.17	0.6778
0.73	1.66	0.1793	0.28	1.22	0.5961	-0.45	-1.37	0.4034
0.42	1.34	0.4648	0.58	1.49	0.2817	0.16	1.12	0.777
-0.03	-1.02	0.9578	0.6	1.52	0.2475	0.63	1.55	0.2608
0.16	1.12	0.7803	0.4	1.32	0.457	0.24	1.18	0.675
-0.52	-1.44	0.377	-1.07	-2.1	0.0689	-0.55	-1.46	0.3954
0.16	1.11	0.8132	-0.16	-1.12	0.8139	-0.32	-1.25	0.6314
0.22	1.16	0.6846	-1	-2	0.0781	-1.22	-2.33	0.0394
0.6	1.52	0.3093	1.05	2.07	0.0559	0.45	1.36	0.4262
-1.2	-2.29	0.0658	-0.08	-1.06	0.8838	1.11	2.16	0.0899
-0.63	-1.55	0.2726	-0.04	-1.03	0.9355	0.59	1.51	0.3099
0.54	1.46	0.3343	0.12	1.09	0.83	-0.42	-1.34	0.4496
-0.04	-1.03	0.9452	0.65	1.57	0.2573	0.7	1.62	0.2591
1.23	2.35	0.0633	2.38	5.21	1.00E-04	1.15	2.22	0.0613
-0.46	-1.38	0.4549	-0.13	-1.09	0.8245	0.34	1.26	0.5925

-0.42	-1.34	0.5017	0.32	1.25	0.5624	0.74	1.67	0.228
-0.98	-1.97	0.1617	-1.9	-3.73	0.0072	-0.92	-1.89	0.2185
-1.42	-2.67	0.0352	-0.28	-1.21	0.643	1.14	2.2	0.0954
0.63	1.54	0.2425	-0.16	-1.11	0.7712	-0.78	-1.72	0.1535
-0.73	-1.66	0.2195	-0.38	-1.3	0.4803	0.35	1.27	0.5704
1.23	2.35	0.0228	0.72	1.65	0.178	-0.52	-1.43	0.3147
0.98	1.98	0.0776	0.19	1.14	0.7375	-0.8	-1.74	0.1504
0.16	1.12	0.7676	0.14	1.1	0.7953	-0.03	-1.02	0.9596
0.62	1.53	0.3019	0.45	1.37	0.4297	-0.16	-1.12	0.7792
0.41	1.33	0.4926	0.49	1.4	0.3878	0.08	1.06	0.8945
-0.68	-1.61	0.2778	-0.62	-1.54	0.2942	0.06	1.04	0.9281
-1.07	-2.09	0.0993	-0.17	-1.13	0.767	0.89	1.86	0.1719
-1.79	-3.47	0.0068	-0.05	-1.04	0.9288	1.74	3.35	0.0089
-1.2	-2.29	0.0803	-0.03	-1.02	0.96	1.17	2.24	0.0906
1.29	2.44	0.0544	2.28	4.87	3.00E-04	1	2	0.1063
-0.36	-1.28	0.538	-0.24	-1.18	0.6568	0.12	1.08	0.8429
0.38	1.31	0.3223	0.64	1.55	0.0723	0.25	1.19	0.5005
-0.71	-1.63	0.2801	0.75	1.68	0.1794	1.46	2.75	0.0215
-0.25	-1.19	0.6886	0.67	1.59	0.2186	0.92	1.89	0.124
-0.62	-1.54	0.2865	-0.1	-1.07	0.8435	0.52	1.43	0.3803
-0.84	-1.79	0.1758	-0.52	-1.44	0.3564	0.31	1.24	0.6263
-0.22	-1.16	0.7081	0.33	1.26	0.5286	0.55	1.47	0.3402
-0.59	-1.5	0.2856	-0.67	-1.59	0.1969	-0.08	-1.06	0.8896
-0.71	-1.64	0.2377	0.05	1.04	0.9251	0.76	1.69	0.208
0.35	1.28	0.5557	0.52	1.43	0.3564	0.16	1.12	0.7775
0.28	1.21	0.6274	0.33	1.26	0.5372	0.05	1.04	0.9225
0.01	1.01	0.9915	-0.83	-1.78	0.264	-0.84	-1.79	0.2609
0.05	1.04	0.9299	-0.61	-1.52	0.315	-0.66	-1.58	0.2986
1.24	2.37	0.0572	1.26	2.4	0.0445	0.02	1.01	0.9779
0.75	1.68	0.1904	0.46	1.38	0.3979	-0.28	-1.22	0.6131
-0.22	-1.17	0.7331	0.32	1.25	0.5958	0.54	1.45	0.4012
0.46	1.37	0.4313	0.03	1.02	0.9639	-0.43	-1.35	0.4606
-2	-3.99	0.004	-0.62	-1.54	0.3261	1.37	2.59	0.0514
0.89	1.86	0.167	0.94	1.92	0.128	0.05	1.03	0.9384
0.12	1.09	0.8244	-0.31	-1.24	0.5765	-0.43	-1.35	0.4569
0.54	1.45	0.3591	-0.48	-1.4	0.4233	-1.02	-2.03	0.0967
1.11	2.16	0.0514	0.37	1.3	0.5106	-0.73	-1.66	0.1874
0	-1	0.9949	-0.37	-1.3	0.5764	-0.37	-1.29	0.5949
-0.62	-1.54	0.3472	0.29	1.22	0.6253	0.91	1.89	0.1646
-1.16	-2.23	0.0674	-1.01	-2.01	0.0885	0.15	1.11	0.8267
-0.68	-1.61	0.2938	0.22	1.16	0.711	0.9	1.87	0.1637
-0.69	-1.61	0.3372	0.17	1.13	0.8015	0.86	1.82	0.2297
-0.14	-1.1	0.8153	0.28	1.22	0.6024	0.42	1.34	0.474
-0.17	-1.13	0.7676	-0.85	-1.81	0.1474	-0.68	-1.6	0.2801
0.05	1.03	0.928	-0.59	-1.5	0.2745	-0.64	-1.55	0.2651
-0.53	-1.44	0.4042	0	-1	0.9947	0.53	1.44	0.4107
-0.32	-1.25	0.6211	0.78	1.72	0.1655	1.1	2.14	0.0759

0.21	1.16	0.7174	0.43	1.35	0.4351	0.22	1.16	0.71
-0.03	-1.02	0.956	-0.21	-1.16	0.7128	-0.18	-1.13	0.7709
0.52	1.43	0.3846	0.09	1.06	0.8838	-0.44	-1.35	0.4688
0.98	1.97	0.1126	0.65	1.57	0.2755	-0.32	-1.25	0.5855
-1.28	-2.43	0.0476	-0.93	-1.91	0.1206	0.35	1.28	0.6073
-0.3	-1.23	0.6451	-0.83	-1.78	0.1938	-0.53	-1.45	0.4319
-0.44	-1.35	0.3852	-0.15	-1.11	0.742	0.28	1.22	0.5758
-0.76	-1.7	0.2474	0.17	1.13	0.7758	0.94	1.91	0.1557
0.41	1.33	0.4848	-0.32	-1.25	0.58	-0.73	-1.66	0.2249
0.44	1.36	0.0854	0.28	1.22	0.2385	-0.16	-1.12	0.5323
0.68	1.6	0.2641	0.1	1.07	0.873	-0.59	-1.5	0.338
-0.25	-1.19	0.6929	-0.02	-1.01	0.9741	0.23	1.17	0.7176
0.96	1.94	0.1183	-0.05	-1.04	0.9359	-1.01	-2.01	0.1045
-0.17	-1.13	0.7816	0.7	1.62	0.2141	0.87	1.83	0.151
0.24	1.18	0.7129	0.36	1.28	0.5613	0.12	1.09	0.8517
-0.22	-1.17	0.7243	0.76	1.69	0.1782	0.98	1.97	0.1089
0.72	1.65	0.3131	-0.19	-1.14	0.7942	-0.91	-1.88	0.2019
0.37	1.29	0.514	0.11	1.08	0.834	-0.25	-1.19	0.6521
-0.56	-1.47	0.4363	-0.12	-1.09	0.8617	0.44	1.35	0.5435
-0.22	-1.16	0.705	0.17	1.13	0.7407	0.39	1.31	0.4955
0.52	1.44	0.3704	0.03	1.02	0.9578	-0.49	-1.41	0.4017
-0.52	-1.43	0.4127	0.61	1.53	0.2656	1.13	2.19	0.0662
-0.02	-1.01	0.9754	0.57	1.49	0.3364	0.59	1.51	0.3493
-1.15	-2.22	0.0918	0.58	1.49	0.3301	1.73	3.31	0.0101
0.39	1.31	0.5193	0.21	1.15	0.7249	-0.19	-1.14	0.7584
-0.33	-1.25	0.6108	0.28	1.22	0.6289	0.61	1.53	0.3382
-0.29	-1.22	0.6749	0.87	1.83	0.1503	1.16	2.23	0.0772
0.13	1.09	0.8386	0.46	1.37	0.4239	0.33	1.26	0.5834
-1.33	-2.52	0.05	-0.41	-1.33	0.502	0.92	1.89	0.187
-1.52	-2.86	0.021	-0.14	-1.1	0.81	1.38	2.61	0.0375
-0.91	-1.88	0.1614	-1.13	-2.19	0.0724	-0.22	-1.17	0.7456
0.01	1.01	0.9864	-0.28	-1.21	0.6349	-0.29	-1.22	0.6418
-1.49	-2.82	0.0198	0.25	1.19	0.6421	1.74	3.34	0.0063
-1.02	-2.03	0.1231	-0.67	-1.59	0.2801	0.35	1.28	0.6084
0.05	1.03	0.9398	0.19	1.14	0.7617	0.14	1.1	0.8315
0.34	1.26	0.5753	0.39	1.31	0.4941	0.05	1.04	0.9303
-0.27	-1.2	0.663	-0.67	-1.59	0.2682	-0.4	-1.32	0.5373
0.59	1.5	0.3499	0.32	1.24	0.6028	-0.27	-1.21	0.6631
-0.17	-1.13	0.7831	0.07	1.05	0.9069	0.24	1.18	0.7017
-0.63	-1.55	0.3428	0.67	1.59	0.2436	1.3	2.47	0.0435
-0.83	-1.77	0.2226	-0.01	-1.01	0.9879	0.82	1.76	0.2304
-0.97	-1.96	0.1305	-0.35	-1.27	0.5527	0.63	1.54	0.341
-0.47	-1.39	0.4457	-1.03	-2.04	0.0952	-0.55	-1.47	0.404
-0.15	-1.11	0.8216	0.48	1.4	0.4126	0.63	1.55	0.3204
-0.34	-1.26	0.6151	1.13	2.18	0.0574	1.46	2.76	0.0229
-0.56	-1.48	0.3958	-0.39	-1.31	0.5331	0.17	1.13	0.8008
1.09	2.13	0.0663	0.11	1.08	0.8598	-0.99	-1.98	0.0975

0.63	1.55	0.3164	1.02	2.02	0.0867	0.38	1.3	0.5275
-0.24	-1.18	0.7035	0.21	1.15	0.718	0.44	1.36	0.4748
0.82	1.76	0.1637	0.01	1.01	0.9864	-0.81	-1.75	0.1724
0.62	1.53	0.2143	0.23	1.17	0.6351	-0.39	-1.31	0.4294
0.39	1.31	0.2293	0.27	1.2	0.3727	-0.12	-1.09	0.7092
0.41	1.32	0.5107	0.66	1.58	0.2533	0.25	1.19	0.6716
-0.95	-1.93	0.152	-0.46	-1.37	0.4565	0.49	1.41	0.4691
-0.36	-1.28	0.5958	-0.56	-1.47	0.3993	-0.2	-1.15	0.7778
0.14	1.1	0.8282	0.57	1.49	0.3327	0.44	1.35	0.4845
0.37	1.29	0.5755	0.97	1.96	0.1088	0.61	1.52	0.3335
0.8	1.75	0.2456	0.75	1.68	0.2663	-0.06	-1.04	0.9322
0.24	1.18	0.6967	0.96	1.94	0.0908	0.72	1.64	0.2291
-0.5	-1.42	0.4484	-0.36	-1.28	0.5671	0.14	1.1	0.8339
0	-1	0.9998	0.01	1.01	0.9813	0.01	1.01	0.9822
-0.95	-1.93	0.1696	-0.47	-1.38	0.4701	0.48	1.39	0.4982
-1.34	-2.53	0.053	-1.45	-2.74	0.031	-0.12	-1.08	0.8727
0.29	1.22	0.6965	0.33	1.26	0.6562	0.04	1.03	0.9555
0.21	1.16	0.7397	0.63	1.54	0.2971	0.41	1.33	0.5111
0.42	1.34	0.5401	0.26	1.19	0.702	-0.17	-1.12	0.8087
-1.04	-2.05	0.122	0	1	0.9986	1.04	2.05	0.1234
-0.24	-1.18	0.7279	-0.23	-1.17	0.7266	0.01	1.01	0.9906
1.13	2.19	0.0823	0.91	1.88	0.1485	-0.22	-1.16	0.7246
-0.58	-1.49	0.3992	0.43	1.34	0.4881	1	2	0.1363
-1.85	-3.61	0.0084	-1.23	-2.34	0.0669	0.62	1.54	0.3941
-0.21	-1.16	0.7706	0.52	1.44	0.4523	0.73	1.66	0.3068
0.52	1.44	0.2524	0.14	1.1	0.744	-0.38	-1.3	0.4061
-0.61	-1.52	0.3496	-0.4	-1.32	0.5105	0.21	1.15	0.7576
-1.09	-2.13	0.1021	-0.7	-1.62	0.2611	0.39	1.31	0.5723
0.3	1.23	0.5941	0.54	1.46	0.3039	0.24	1.18	0.6621
0.52	1.43	0.472	0.53	1.44	0.4536	0.01	1.01	0.9906
0.66	1.58	0.3784	0.73	1.66	0.3244	0.07	1.05	0.9229
-0.32	-1.24	0.6543	-0.23	-1.17	0.7404	0.09	1.06	0.8998
-1.11	-2.16	0.1244	-0.79	-1.73	0.2612	0.32	1.25	0.6653
-0.69	-1.62	0.3096	0.93	1.91	0.1188	1.62	3.08	0.0139
-0.06	-1.05	0.9289	-0.96	-1.94	0.1873	-0.89	-1.86	0.2278
0.31	1.24	0.6395	-0.57	-1.48	0.3964	-0.88	-1.84	0.2016
-0.57	-1.48	0.384	-0.62	-1.53	0.3225	-0.05	-1.04	0.9407
-0.46	-1.38	0.5088	-0.39	-1.31	0.5621	0.07	1.05	0.9211
1.52	2.86	0.0218	1.28	2.43	0.046	-0.24	-1.18	0.706
-1.1	-2.14	0.1348	-1.68	-3.21	0.0219	-0.58	-1.5	0.4348
0.28	1.22	0.6897	1.12	2.17	0.0914	0.84	1.79	0.2196
-0.93	-1.9	0.1907	-0.05	-1.04	0.9392	0.88	1.84	0.2182
-0.07	-1.05	0.9224	0.49	1.4	0.4507	0.55	1.47	0.4133
0.38	1.3	0.5463	-0.44	-1.36	0.4882	-0.83	-1.77	0.2085
-0.74	-1.67	0.2757	-0.11	-1.08	0.8613	0.63	1.55	0.3581
-0.09	-1.06	0.8966	0.38	1.3	0.562	0.47	1.38	0.4936
-0.73	-1.66	0.2893	0.11	1.08	0.8652	0.84	1.79	0.2241

-0.33	-1.25	0.6566	-0.44	-1.35	0.5461	-0.11	-1.08	0.8814
-1.68	-3.19	0.0171	-1.23	-2.35	0.0681	0.44	1.36	0.5451
0.75	1.68	0.2935	0.88	1.83	0.208	0.12	1.09	0.8611
-0.63	-1.55	0.3754	-1.47	-2.78	0.039	-0.84	-1.8	0.2568
-0.57	-1.48	0.4086	-1.51	-2.84	0.0298	-0.94	-1.92	0.197
-0.83	-1.78	0.2609	-0.9	-1.87	0.2225	-0.07	-1.05	0.9281
-1.86	-3.62	0.0091	-0.52	-1.44	0.4349	1.34	2.52	0.0634
0.48	1.4	0.438	-0.52	-1.43	0.4124	-1	-2	0.1218
0.91	1.88	0.177	0.43	1.34	0.5215	-0.49	-1.4	0.4655
0.89	1.86	0.2055	0.23	1.18	0.7393	-0.66	-1.58	0.3476
2.44	5.42	0.001	1.21	2.31	0.1017	-1.23	-2.35	0.089
-1.17	-2.25	0.0965	-0.45	-1.36	0.5008	0.72	1.65	0.3129
1.48	2.78	0.0436	1.66	3.17	0.02	0.19	1.14	0.7942
0.43	1.35	0.534	0.1	1.07	0.8835	-0.33	-1.26	0.6332
-0.47	-1.38	0.4703	-0.43	-1.34	0.4879	0.04	1.03	0.9517
-0.45	-1.37	0.5198	-0.4	-1.32	0.5531	0.05	1.03	0.9459
0.11	1.08	0.8764	1	2	0.1174	0.89	1.86	0.1798
-0.45	-1.36	0.5259	0.46	1.38	0.483	0.91	1.88	0.1926
-1.27	-2.41	0.0879	-0.23	-1.17	0.7547	1.04	2.05	0.1634
0.83	1.77	0.2393	1.83	3.55	0.005	1	2	0.1233
0.73	1.66	0.2649	-0.21	-1.16	0.745	-0.95	-1.93	0.1554
1.17	2.24	0.0984	0.73	1.66	0.2907	-0.44	-1.35	0.5319
0.74	1.67	0.2993	0.94	1.92	0.1695	0.2	1.15	0.768
-0.88	-1.84	0.2314	1.25	2.38	0.068	2.13	4.37	0.003
-0.73	-1.66	0.32	-0.73	-1.66	0.3134	0	-1	0.9971
1.06	2.09	0.1225	0	-1	0.9986	-1.07	-2.09	0.1238
1.39	2.63	0.0523	-0.17	-1.12	0.8149	-1.56	-2.95	0.0306
0.22	1.17	0.7638	0.78	1.71	0.2942	0.55	1.47	0.4585
0.95	1.93	0.1738	0.73	1.65	0.2848	-0.22	-1.17	0.7485
-0.35	-1.27	0.5422	0.18	1.13	0.732	0.53	1.44	0.3555
-0.26	-1.2	0.722	0.41	1.33	0.5755	0.68	1.6	0.3628
-0.12	-1.09	0.843	0.13	1.1	0.8192	0.25	1.19	0.6785
-1.01	-2.02	0.1663	-0.06	-1.05	0.9318	0.95	1.93	0.1946
1.78	3.45	NA	0.01	1.01	NA	-1.77	-3.41	NA
-0.11	-1.08	0.8852	-0.13	-1.09	0.8617	-0.02	-1.02	0.9751
0.96	1.94	0.197	1.46	2.76	0.0439	0.51	1.42	0.4874
0.4	1.32	0.4907	0.45	1.37	0.4124	0.05	1.03	0.9313
0.47	1.39	0.5009	0.03	1.02	0.9662	-0.44	-1.36	0.5286
0.9	1.86	0.2296	1.61	3.05	0.0305	0.71	1.64	0.3396
1.11	2.16	0.0166	-0.39	-1.31	0.4118	-1.5	-2.83	0.0017
0.05	1.04	0.9186	0.2	1.15	0.685	0.14	1.11	0.7824
0.37	1.29	0.5364	0.58	1.49	0.2998	0.21	1.16	0.7175
0.51	1.43	0.4907	0.05	1.04	0.9451	-0.46	-1.38	0.535
0.03	1.02	0.9649	0.03	1.02	0.9718	-0.01	-1	0.9935
-0.07	-1.05	0.9168	-0.05	-1.04	0.9428	0.02	1.01	0.9757
-0.83	-1.77	0.1365	0.04	1.03	0.9325	0.87	1.82	0.1189
-0.34	-1.27	0.6451	0.44	1.36	0.5534	0.79	1.73	0.2928

0.42	1.34	0.4966	-0.63	-1.55	0.3084	-1.05	-2.07	0.0996
1.02	2.02	0.1474	0.18	1.13	0.7958	-0.84	-1.79	0.2323
0.91	1.88	0.1902	-2.28	-4.85	0.0015	-3.19	-9.11	0
-0.6	-1.52	0.4128	0.15	1.11	0.8321	0.76	1.69	0.3044
-0.66	-1.58	0.377	0.5	1.41	0.4967	1.15	2.22	0.1198
0.1	1.08	0.8732	-0.26	-1.2	0.6967	-0.37	-1.29	0.5734
-0.25	-1.19	0.6929	0.01	1.01	0.9908	0.25	1.19	0.6841
-0.17	-1.12	0.8072	0.33	1.25	0.6159	0.49	1.41	0.4684
0.55	1.46	0.4286	0.55	1.47	0.4091	0.01	1	0.9939
-0.6	-1.52	0.3542	-2.15	-4.43	0.0014	-1.55	-2.92	0.0285
0.69	1.62	NA	-0.06	-1.04	NA	-0.75	-1.68	NA
0.04	1.03	0.9491	-0.07	-1.05	0.9153	-0.1	-1.07	0.8611
0.08	1.06	0.9101	0.95	1.93	0.2033	0.86	1.82	0.2474
-0.61	-1.53	NA	-0.71	-1.64	NA	-0.1	-1.07	NA
-0.88	-1.84	0.1019	-0.46	-1.37	0.4252	0.43	1.34	0.4257

					Flag of top proteins (P value < 0.01 and Fold c			
flag.ATN.v	flag.IgAN E	flag.IgAN E	flag.S.a.GN	flag.VancA	flag.IgAN.v	flag.IgAN E	flag.S.a.GN	flag.VancA
0	0	0	1	0	0	0	0	0
0	0	0	0	0	0	0	0	0
0	0	0	0	0	0	0	0	0
0	0	0	0	0	0	0	0	0
0	0	0	0	0	0	0	0	0
0	0	0	0	0	0	0	0	0
1	0	0	0	1	1	0	0	0
1	0	0	1	0	0	0	0	0
0	0	0	0	0	0	0	0	0
0	0	0	0	0	0	0	0	0
1	0	0	1	0	0	0	0	0
0	0	0	0	0	0	0	0	0
0	0	0	0	0	0	0	0	0
0	0	0	0	0	0	0	0	0
0	0	0	0	0	0	0	0	0
0	0	0	0	0	0	0	0	0
1	0	1	1	0	0	0	0	0
0	0	0	0	0	0	0	0	0
0	0	0	0	0	0	0	0	0
0	0	0	0	0	0	0	0	0
0	0	0	0	0	0	0	0	0
0	0	0	0	0	0	0	0	0
1	0	0	0	0	1	0	0	0
1	0	0	1	0	1	0	0	0
1	0	0	0	0	0	0	0	0
0	0	0	0	0	0	0	0	0
0	0	0	0	0	0	0	0	0
0	0	0	0	0	0	0	0	0
0	0	0	0	0	0	0	0	0
0	0	0	0	0	0	0	0	0
0	0	0	1	0	0	0	0	0
0	0	0	0	0	0	0	0	0
0	0	0	0	0	0	0	0	0
0	0	0	0	0	0	0	0	0
0	0	0	1	0	0	0	0	0
0	0	0	0	0	0	0	0	0
0	0	0	0	0	0	0	0	0
0	0	0	0	0	0	0	0	0
0	0	0	0	0	0	0	0	0
0	0	0	0	0	0	0	0	0
0	0	0	0	0	0	0	0	0
0	0	0	0	0	0	0	0	0
0	0	0	0	0	0	0	0	0

0	0	0	0	0	0	0	0	0
0	0	0	0	0	0	0	0	0
0	0	0	1	0	0	0	0	0
0	0	0	1	0	1	0	0	0
0	0	0	0	0	0	0	0	0
0	0	0	0	0	0	0	0	0
0	0	0	0	0	0	0	0	0
0	0	0	0	0	0	0	0	0
1	0	0	1	0	1	0	0	0
0	0	0	0	0	0	0	0	0
1	0	1	0	1	1	0	1	0
0	0	0	0	0	0	0	0	0
0	0	0	0	0	0	0	0	0
1	0	0	1	1	0	0	0	0
1	0	1	1	1	1	0	0	0
1	0	0	0	0	1	0	0	0
0	0	0	0	1	1	0	0	0
0	0	0	0	0	0	0	0	0
0	0	0	0	0	0	0	0	0
1	0	0	0	0	1	0	0	0
0	0	0	0	0	0	0	0	0
1	0	0	0	1	1	1	1	0
0	0	0	0	0	0	0	0	0
0	0	0	0	0	0	0	0	0
0	0	0	0	0	0	0	0	0
1	0	0	0	0	1	0	0	0
0	0	0	0	0	0	0	0	0
1	0	0	0	1	1	0	0	0
0	0	0	0	0	0	0	0	0
0	0	0	0	0	0	0	0	0
0	0	0	0	0	0	0	0	0
0	0	0	0	0	0	0	0	0
0	0	0	0	0	0	0	0	0
0	0	0	0	0	0	0	0	0
0	0	0	0	0	0	0	0	0
0	0	0	0	0	0	0	0	0
0	0	0	0	0	0	0	0	0
1	1	0	1	0	0	0	0	0
0	0	0	0	0	0	0	0	0
0	0	0	1	0	0	0	1	0
0	0	0	0	0	0	0	0	0
0	0	0	0	0	0	0	0	0
0	0	0	0	0	0	0	0	0
1	0	0	1	0	1	0	0	0
0	0	0	0	0	0	0	0	0
0	0	0	1	0	0	0	0	0
0	0	0	1	0	0	0	0	0

0	0	0	0	0	0	0	0	0
0	0	0	0	0	0	0	0	0
0	0	0	0	0	0	0	0	0
1	0	1	0	1	0	0	0	0
0	0	0	0	1	0	0	0	0
0	0	0	0	0	0	0	0	0
0	0	0	0	0	0	0	0	0
0	0	0	1	0	0	0	0	0
0	0	0	0	0	0	0	0	0
0	0	0	1	0	0	0	1	0
0	0	0	0	0	0	0	0	0
0	0	0	0	0	0	0	0	0
0	0	0	0	0	0	0	0	0
1	0	0	0	0	0	0	0	0
0	0	0	0	0	0	0	0	0
0	0	0	0	0	1	0	0	0
0	0	0	0	0	0	0	0	0
0	0	0	0	0	0	0	0	0
1	0	1	1	1	1	0	0	0
0	0	0	0	0	0	0	0	0
1	0	0	1	0	0	0	0	0
0	0	0	0	0	0	0	0	0
0	0	0	0	0	0	0	0	0
0	0	0	0	0	0	0	0	0
0	0	0	0	0	0	0	0	0
0	0	0	0	0	0	0	0	0
0	0	0	0	0	0	0	0	0
0	0	0	0	0	0	0	0	0
0	0	0	0	0	0	0	0	0
0	0	0	0	0	0	0	0	0
0	0	0	0	0	0	0	0	0
0	0	0	0	0	0	0	0	0
0	0	0	0	0	0	0	0	0
0	0	0	1	0	0	0	0	0
0	0	0	0	0	0	0	0	0
0	0	0	0	0	0	0	0	0
0	0	0	0	0	0	0	0	0
1	0	0	0	1	1	1	0	0
0	0	0	1	0	0	0	0	0
1	0	0	1	1	1	1	0	0
0	0	0	0	0	0	0	0	0
0	0	0	0	0	0	0	0	0
1	0	0	1	1	1	1	0	0
0	0	0	0	0	0	0	0	0
0	0	0	0	0	0	0	0	0
1	0	0	1	1	0	0	0	0
0	0	0	0	0	0	0	0	0

0	0	0	0	0	0	0	0	0
0	0	0	0	0	0	0	0	0
0	0	0	0	0	0	0	0	0
1	0	1	1	1	1	1	0	0
0	0	0	1	0	0	0	0	0
0	0	0	0	0	0	0	0	0
0	0	0	0	0	0	0	0	0
0	0	0	0	0	0	0	0	0
0	0	0	1	0	0	0	0	0
1	0	0	1	0	1	0	0	0
0	0	0	1	0	0	0	0	0
0	0	0	1	0	0	0	1	0
0	0	0	0	0	0	0	0	0
0	0	0	0	0	0	0	0	0
0	0	0	0	0	0	0	0	0
0	0	0	0	0	0	0	0	0
0	0	0	0	0	0	0	0	0
0	0	0	0	0	0	0	0	0
0	0	0	0	0	0	0	0	0
1	0	0	1	1	0	0	0	0
0	0	0	0	0	0	0	0	0
0	0	0	1	0	0	0	0	0
1	0	0	1	1	0	0	0	0
0	0	0	0	0	0	0	0	0
0	0	0	1	0	0	0	0	0
0	0	0	0	0	0	0	0	0
0	0	0	0	0	1	0	0	0
0	0	0	0	0	1	0	0	0
0	0	0	0	0	0	0	0	0
0	0	0	0	0	0	0	0	0
0	0	0	0	0	0	0	0	0
0	0	0	0	0	0	0	0	0
0	0	0	0	0	0	0	0	0
0	0	0	0	0	0	0	0	0
0	0	0	0	0	0	0	0	0
0	0	0	1	0	0	0	0	0
0	0	0	0	0	0	0	0	0
0	0	0	0	0	0	0	1	0
0	0	0	0	0	0	0	0	0
0	0	0	0	0	0	0	0	0
0	0	0	0	0	0	0	0	0
0	0	0	0	0	0	0	0	0
0	0	0	0	0	0	0	0	0
0	0	0	0	0	0	0	0	0
0	0	0	0	0	0	0	0	0
1	0	0	0	0	1	0	0	0
0	0	0	0	0	0	0	0	0

0	0	0	0	1	0	0	0	0
0	0	0	0	0	0	0	0	0
1	0	0	0	1	0	0	0	0
0	0	0	0	0	0	0	0	0
0	0	0	1	0	0	0	0	0
0	0	0	0	0	0	0	0	0
1	0	0	1	0	0	0	0	0
0	0	0	0	0	0	0	0	0
0	0	0	0	0	0	0	0	0
0	0	0	0	0	0	0	0	0
1	0	0	0	1	0	0	0	0
1	0	0	0	0	1	0	0	0
0	0	0	0	0	0	0	0	0
0	0	0	1	0	0	0	0	0
0	0	0	1	0	0	0	0	0
0	0	0	0	0	0	0	0	0
0	0	0	0	0	0	0	0	0
0	0	0	0	0	0	0	0	0
1	0	0	1	0	0	0	0	0
0	0	0	0	0	0	0	0	0
0	0	0	0	0	0	0	0	0
0	0	0	0	0	0	0	0	0
0	0	0	0	0	0	0	0	0
0	0	0	0	0	0	0	0	0
0	0	0	0	0	0	0	0	0
0	0	0	0	0	0	0	0	0
0	0	0	1	0	0	0	0	0
0	0	0	0	0	0	0	0	0
0	0	0	0	0	0	0	0	0
0	0	0	0	0	0	0	0	0
1	0	0	0	1	1	0	0	0
0	0	0	0	0	0	0	0	0
1	0	0	1	0	1	1	0	0
0	0	0	1	0	0	0	0	0
0	0	0	0	0	0	0	0	0
0	0	0	0	0	0	0	0	0
0	0	0	0	0	0	0	0	0
0	0	0	0	0	0	0	0	0
0	0	0	1	0	0	0	0	0
0	0	0	0	0	0	0	0	0
0	0	0	1	0	0	0	0	0
0	0	0	1	0	0	0	0	0
0	0	0	0	0	0	0	0	0
0	0	0	0	0	0	0	0	0
0	0	0	0	0	0	0	0	0
0	0	0	0	0	0	0	0	0
0	0	0	0	0	0	0	0	0

0	0	0	0	0	0	0	0	0
0	0	0	0	0	0	0	0	0
0	0	0	0	0	0	0	0	0
0	0	0	0	0	0	0	0	0
0	0	0	0	0	0	0	0	0
0	0	0	0	0	0	0	0	0
1	0	0	0	1	1	0	0	0
0	0	0	0	0	0	0	0	0
0	0	0	0	0	0	0	0	0
1	0	0	1	1	0	0	0	0
0	0	0	0	0	0	0	0	0
0	0	0	0	0	0	0	0	0
0	0	0	0	0	0	0	0	0
0	0	0	0	0	0	0	0	0
1	0	1	1	1	1	0	0	0
0	0	0	0	0	0	0	0	0
0	0	0	0	0	0	0	0	0
1	0	0	1	1	0	0	0	0
0	0	0	0	0	0	0	0	0
0	0	0	0	0	0	0	0	0
1	0	1	1	1	1	0	0	0
0	0	0	0	0	0	0	0	0
0	0	0	1	0	0	0	0	0
0	0	0	0	0	0	0	0	0
1	0	0	0	0	1	0	0	0
1	0	0	1	1	1	1	0	0
0	0	0	0	0	0	0	0	0
0	0	0	0	0	0	0	0	0
0	0	0	0	0	0	0	0	0
0	0	0	0	0	0	0	0	0
0	0	0	0	0	0	0	0	0
0	0	0	0	0	0	0	0	0
1	0	0	0	0	1	0	0	0
1	0	0	1	1	1	0	0	0
0	0	0	0	0	0	0	0	0
1	0	0	1	0	0	0	0	0
0	0	0	0	0	0	0	0	0
0	0	0	0	0	0	0	0	0
0	0	0	0	0	0	0	0	0
0	0	0	0	0	0	0	0	0
0	0	0	0	0	0	0	0	0
1	0	0	1	1	0	0	0	0
0	0	0	1	0	0	0	0	0
0	0	0	0	0	0	0	0	0
0	0	0	0	0	0	0	0	0
1	0	0	1	0	0	0	0	0
1	0	0	1	1	0	0	0	0

0	0	0	0	0	0	0	0	0
0	0	0	1	0	0	0	0	0
0	0	0	0	0	0	0	0	0
0	0	0	0	0	0	0	0	0
0	0	0	0	0	0	0	0	0
0	0	0	0	0	0	0	0	0
0	0	0	0	0	0	0	0	0
0	0	0	0	0	0	0	0	0
0	0	0	0	0	0	0	0	0
0	0	0	0	0	0	0	0	0
0	0	0	0	0	0	0	0	0
0	0	0	1	0	0	0	0	0
0	0	0	0	0	0	0	0	0
0	0	0	0	0	0	0	0	0
0	0	0	0	0	0	0	0	0
0	0	0	0	0	0	0	0	0
0	0	0	0	0	0	0	0	0
1	0	0	0	0	0	0	0	0
0	0	0	0	0	0	0	0	0
1	0	0	0	1	0	0	0	0
0	0	0	1	0	0	0	0	0
0	0	0	0	0	0	0	0	0
0	0	0	0	0	0	0	0	0
0	0	0	0	0	0	0	0	0
0	0	0	0	0	0	0	0	0
0	0	0	0	0	0	0	0	0
0	0	0	0	0	0	0	0	0
0	0	0	0	0	0	0	0	0
0	0	0	0	0	0	0	0	0
0	0	0	0	0	0	0	0	0
0	0	0	0	0	0	0	0	0
1	1	1	1	1	1	1	0	0
1	0	1	1	1	1	0	0	0
1	0	0	0	0	0	0	0	0
0	0	0	1	0	0	0	0	0
1	0	0	1	1	0	0	0	0
0	0	0	0	0	0	0	0	0
0	0	0	0	0	0	0	0	0
0	0	0	0	0	0	0	0	0
0	0	0	0	0	0	0	0	0
0	0	0	0	0	0	0	0	0
0	0	0	0	0	1	0	0	0
0	0	0	0	0	0	0	0	0
1	0	0	1	0	1	1	0	0
0	0	0	0	0	0	0	0	0
0	0	0	1	0	0	0	0	0
1	0	0	0	0	0	0	0	0

1	0	1	1	1	0	0	0	0
1	0	0	0	0	1	1	0	0
0	0	0	0	0	0	0	0	0
0	0	0	0	0	0	0	0	0
1	0	0	0	1	0	1	0	0
0	0	0	0	0	0	0	0	0
0	0	0	0	0	0	0	0	0
0	0	0	1	1	0	0	0	0
1	0	0	0	0	0	0	0	0
0	0	0	0	0	0	0	0	0
0	0	0	0	0	0	0	0	0
0	0	0	0	0	0	0	0	0
0	0	0	0	0	0	0	0	0
0	0	0	0	0	0	0	0	0
0	0	0	0	0	0	0	0	0
1	0	0	1	1	0	0	0	0
1	0	0	0	1	0	0	0	0
0	0	0	0	0	0	0	0	0
0	0	0	0	0	0	0	0	0
0	0	0	0	0	0	0	0	0
0	0	0	0	0	0	0	0	0
0	0	0	0	0	0	0	0	0
0	0	0	0	0	0	0	0	0
0	0	0	0	0	0	0	0	0
0	0	0	0	0	0	0	0	0
0	0	0	0	0	0	0	0	0
0	0	0	0	0	0	0	0	0
0	0	0	0	0	0	0	0	0
0	0	0	0	0	0	0	0	0
1	1	1	1	1	1	0	0	0
0	0	0	0	0	0	0	0	0
0	0	0	0	0	0	0	0	0
0	0	0	0	0	0	0	0	0
0	0	0	0	0	0	0	0	0
0	0	0	0	0	0	0	0	0
1	0	0	0	0	0	0	0	0
0	0	0	0	0	0	0	0	0
0	0	0	0	0	0	0	0	0
0	0	0	0	0	0	0	0	0
0	0	0	0	0	0	0	0	0
1	0	0	0	1	1	0	0	0
0	0	0	0	0	0	0	0	0
0	0	0	0	0	0	0	0	0
0	0	0	0	0	0	0	0	0
0	0	0	0	0	0	0	0	0
0	0	0	0	0	0	0	0	0
0	0	0	0	0	0	0	0	0

1	0	0	0	1	1	0	0	0
0	0	0	0	0	0	0	0	0
0	0	0	0	0	0	0	0	0
1	0	0	0	0	0	0	0	0
0	0	0	0	0	0	0	0	0
0	0	0	0	0	0	0	0	0
0	0	0	0	0	0	0	0	0
0	0	0	1	0	0	0	0	0
0	0	0	0	0	1	0	0	0
0	0	0	0	0	0	0	0	0
0	0	0	0	0	0	0	0	0
0	0	0	0	0	0	0	0	0
0	0	0	0	0	0	0	0	0
0	0	0	0	0	0	0	0	0
0	0	0	0	0	0	0	0	0
0	0	0	0	0	0	0	0	0
0	0	0	0	0	0	0	0	0
0	0	0	0	0	0	0	0	0
1	0	0	0	0	0	0	0	0
1	0	0	0	0	0	1	0	0
0	0	0	0	0	0	0	0	0
0	0	0	1	0	0	0	0	0
0	0	0	1	0	0	0	0	0
0	0	0	0	0	0	0	0	0
1	0	0	1	0	1	1	0	0
1	0	0	1	0	1	0	0	0
1	0	0	0	0	1	0	0	0
0	0	0	0	0	0	0	0	0
0	0	0	1	0	0	0	0	0
0	0	0	0	0	0	0	0	0
1	0	0	0	0	1	0	0	0
0	0	0	0	0	0	0	0	0
1	0	0	1	0	0	0	0	0
0	0	0	0	0	0	0	0	0
0	0	0	0	0	0	0	0	0
0	0	0	0	0	0	0	0	0
0	0	0	0	0	0	0	0	0
1	0	0	0	1	0	0	0	0
0	0	0	0	0	0	0	0	0
0	0	0	0	0	0	0	0	0
1	0	0	0	1	1	0	0	0
0	0	0	1	0	0	0	0	0
0	0	0	0	0	0	0	0	0
0	0	0	0	0	0	0	0	0
0	0	0	0	0	0	0	0	0
1	0	0	1	1	1	0	0	0
0	0	0	0	0	0	0	0	0

0	0	0	0	0	0	0	0	0
1	0	1	1	1	1	1	0	0
0	1	0	1	0	0	0	0	0
0	0	0	0	0	0	0	0	0
0	0	0	0	0	0	0	0	0
0	0	0	0	0	0	0	0	0
0	0	0	1	0	0	0	0	0
0	0	0	0	0	0	0	0	0
0	0	0	0	0	0	0	0	0
0	0	0	0	0	0	0	0	0
1	0	0	0	0	0	0	0	0
0	0	0	0	0	0	0	0	0
0	0	0	1	0	0	0	1	0
1	0	1	1	1	1	0	0	0
1	0	0	1	1	1	1	0	0
0	0	0	0	0	0	0	0	0
0	0	0	0	0	0	0	0	0
0	0	0	0	0	0	0	1	0
1	0	1	1	1	1	0	0	0
0	0	0	0	0	0	0	0	0
0	0	0	0	0	0	0	0	0
0	0	0	0	0	0	0	0	0
0	0	0	0	1	0	0	0	0
0	0	0	0	0	0	0	0	0
0	0	0	0	0	0	0	0	0
1	0	1	1	1	1	0	0	0
0	0	0	0	0	0	0	0	0
0	0	0	0	0	0	0	0	0
0	0	0	0	0	0	0	0	0
1	0	0	1	1	1	0	0	0
0	0	0	0	0	0	0	0	0
0	0	0	0	0	0	0	0	0
0	0	0	1	0	0	0	0	0
0	0	0	0	0	0	0	0	0
0	0	0	0	0	0	0	0	0
0	0	0	0	0	0	0	0	0
0	0	0	1	0	0	0	0	0
0	0	0	0	0	0	0	0	0
0	0	0	0	0	0	0	0	0
0	0	0	0	0	0	0	0	0
0	0	0	0	0	0	0	0	0
0	0	0	0	0	0	0	0	0
0	0	0	0	0	0	0	0	0
0	0	0	0	0	0	0	0	0
1	0	0	0	1	1	0	0	0
0	0	0	0	0	0	0	0	0
0	0	0	0	0	0	0	0	0

1	0	0	0	0	0	0	0	0
0	0	0	0	0	0	0	0	0
0	0	0	0	0	0	0	0	0
0	0	0	0	0	0	0	0	0
0	0	0	0	0	0	0	0	0
0	0	0	0	0	0	0	0	0
0	0	0	0	0	0	0	0	0
0	0	0	0	0	0	0	0	0
0	0	0	0	0	0	0	0	0
0	0	0	0	0	0	0	0	0
0	0	0	0	0	0	0	0	0
0	0	0	0	0	0	0	0	0
0	0	0	0	0	0	0	0	0
1	0	0	0	1	1	0	0	0
0	0	0	0	0	0	0	0	0
1	0	1	0	1	1	0	0	0
0	0	0	0	0	0	0	0	0
0	0	0	0	0	0	0	0	0
0	0	0	0	0	0	0	0	0
0	0	0	0	0	0	0	0	0
1	0	0	0	0	0	0	0	0
1	0	0	0	0	1	0	0	0
0	0	0	0	0	0	0	0	0
0	0	0	0	0	0	0	0	0
0	0	0	0	0	0	0	0	0
0	0	0	0	0	0	0	0	0
0	0	0	0	0	0	0	0	0
1	0	0	0	0	0	0	0	0
1	0	0	1	1	0	0	0	0
0	0	0	0	0	0	0	0	0
1	0	0	0	0	0	0	0	0
0	0	0	0	0	0	0	0	0
1	0	1	0	1	0	0	0	0
0	0	0	0	0	0	0	0	0
0	0	0	0	0	0	0	0	0
0	0	0	0	0	0	0	0	0
0	0	0	0	0	0	0	0	0
1	0	0	0	0	0	0	0	0
0	0	0	0	0	0	0	0	0
0	0	0	0	0	0	0	0	0
0	0	0	0	0	0	0	0	0
0	0	0	0	0	0	0	0	0
1	0	0	0	0	0	0	0	0
1	0	0	0	0	1	0	0	0
1	0	0	0	1	1	0	0	0
0	0	0	0	0	0	0	0	0
1	0	0	1	0	0	0	0	0

1	0	0	1	1	1	0	0	0
0	0	0	0	0	0	0	0	0
0	0	0	0	0	0	0	0	0
0	0	0	0	0	0	0	0	0
0	0	0	0	0	0	0	0	0
0	0	0	0	0	0	0	0	0
0	0	0	0	0	0	0	0	0
0	0	0	0	0	0	0	0	0
0	0	0	0	0	1	0	0	0
1	0	0	1	1	1	1	0	0
1	0	0	1	1	1	0	0	0
0	0	0	0	1	0	0	0	0
0	0	0	0	0	0	0	0	0
0	0	0	0	0	0	0	0	0
0	0	0	0	0	0	0	0	0
0	0	0	0	0	0	0	0	0
0	0	0	0	0	0	0	0	0
1	0	0	0	1	0	0	0	0
0	0	0	0	0	0	0	0	0
0	0	0	0	0	0	0	0	0
1	0	0	0	0	0	0	0	0
0	0	0	0	0	0	0	0	0
0	0	0	0	0	0	0	0	0
0	0	0	0	0	0	0	0	0
0	0	0	0	0	0	0	0	0
0	0	0	0	0	0	0	0	0
0	0	0	0	0	0	0	0	0
0	0	0	0	0	0	0	0	0
0	0	0	0	0	0	0	0	0
0	0	0	0	0	1	0	0	0
1	1	0	1	1	0	0	0	0
0	0	0	0	0	0	0	0	0
1	0	0	0	0	0	1	0	0
1	0	0	0	1	1	0	1	0
0	0	0	0	0	0	0	0	0
0	0	0	0	0	0	0	0	0
0	0	0	0	0	0	0	0	0
0	0	0	0	0	0	0	0	0
1	1	0	1	1	0	0	0	0
1	0	0	1	1	0	0	0	0
0	0	0	0	0	0	0	0	0
0	0	0	0	0	0	0	0	0
1	0	0	0	0	1	0	0	0
0	0	0	0	0	0	0	0	0
0	0	0	0	0	0	0	0	0
0	0	0	0	0	0	0	0	0
0	0	0	0	0	0	0	0	0

0	0	0	0	0	0	0	0	0
1	0	0	1	0	0	0	0	0
1	0	0	1	1	1	1	0	0
1	0	0	0	1	0	0	0	0
0	0	0	0	0	0	0	0	0
1	0	0	0	0	0	0	0	0
0	0	1	0	0	0	0	0	0
0	0	0	0	0	0	0	0	0
1	0	0	1	0	0	0	0	0
1	0	0	1	0	0	1	0	0
1	0	0	1	1	0	1	0	0
0	0	0	0	0	0	0	0	0
1	0	0	1	1	1	0	0	0
0	0	0	0	0	0	0	0	0
0	0	0	0	0	0	0	0	0
0	0	0	0	0	1	0	0	0
0	0	0	0	0	0	0	0	0
0	0	0	0	0	1	0	0	0
0	0	0	0	0	0	0	0	0
1	0	0	0	1	1	1	0	0
0	0	0	1	0	0	0	0	0
0	0	0	0	0	0	0	0	0
1	0	0	1	1	1	0	0	0
1	0	0	0	1	0	0	0	0
0	0	0	0	0	0	0	0	0
1	0	1	1	1	0	0	0	0
1	0	0	1	0	1	0	0	0
1	0	0	0	1	0	0	0	0
0	0	0	0	0	0	0	0	0
0	0	0	0	0	0	0	0	0
0	0	0	0	0	0	0	0	0
0	0	0	0	0	0	0	0	0
0	0	0	0	0	0	0	0	0
0	0	0	0	0	0	0	0	0
0	0	0	0	0	0	0	0	0
0	0	0	1	1	0	0	0	0
0	0	0	0	0	0	0	0	0
0	0	0	0	0	0	0	0	0
1	0	0	0	1	1	1	0	0
0	0	0	1	0	0	0	0	0
0	0	0	0	0	0	0	0	0
0	0	0	0	0	0	0	0	0
0	0	0	0	0	0	0	0	0
0	0	0	0	0	0	0	0	0
0	0	0	0	0	0	0	0	0
0	0	0	0	0	0	0	0	0
0	0	0	0	1	0	0	0	0

0	0	0	0	0	0	0	0	0
0	0	0	0	0	0	0	0	0
1	0	0	0	1	1	1	1	0
0	0	0	0	0	0	0	0	0
0	0	0	0	0	0	0	0	0
0	0	0	0	0	0	0	0	0
0	0	0	0	0	0	0	0	0
1	0	0	0	0	1	0	0	0
0	0	0	0	0	0	0	0	0
1	1	0	0	1	0	1	1	0
0	0	0	0	0	0	0	0	0
0	0	0	0	0	0	0	0	0
0	0	0	0	0	0	0	0	0
0	0	0	0	0	0	0	0	0
0	0	0	0	0	0	0	0	0

change > 2)					
flag.IgAN E	flag.S.a.GN	flag.VancA	flag.S.a.GN	flag.VancA	flag.VancATN.vs.S.a.GN
0	1	0	0	0	0
0	0	0	0	0	0
0	0	0	0	0	0
0	0	0	0	0	0
0	0	0	0	0	0
0	0	0	0	0	0
0	0	1	0	0	0
0	0	0	0	0	0
0	0	0	0	0	0
0	0	0	0	0	0
0	1	0	1	0	0
0	0	0	0	0	0
0	0	0	0	0	0
0	0	0	0	0	0
0	0	0	0	0	0
0	0	0	0	0	0
0	0	0	0	0	0
0	0	0	0	0	0
0	0	0	0	0	0
0	0	0	0	0	0
0	0	0	0	0	0
0	0	0	0	0	0
0	0	0	0	0	0
0	1	0	0	0	0
0	0	0	0	0	0
0	0	0	0	0	0
0	0	0	0	0	0
0	0	0	0	0	0
0	0	0	0	0	0
0	0	0	0	0	0
0	0	0	0	0	0
0	0	0	0	0	0
0	0	0	0	0	0
0	0	0	0	0	0
0	1	0	0	0	0
0	1	0	0	0	0
0	0	0	0	0	0
0	0	0	0	0	0
0	0	0	0	0	0
0	0	0	0	0	0
0	0	0	0	0	0
0	0	0	0	0	0
0	0	0	0	0	0

0	0	0	0	0	0
0	0	0	0	0	1
0	1	0	1	0	0
0	1	0	0	0	0
0	0	0	0	0	0
0	0	0	0	0	0
0	0	0	0	0	0
0	0	0	0	0	0
0	1	0	0	0	0
0	0	0	0	0	0
1	0	1	0	0	0
0	0	0	0	0	0
0	0	0	0	0	0
0	0	0	1	1	0
0	0	1	0	0	0
0	0	0	0	0	0
0	1	1	0	0	0
0	0	0	0	0	0
0	0	0	0	0	0
0	0	0	0	0	0
0	0	0	0	0	0
0	0	0	0	1	0
0	0	0	0	0	0
0	0	0	0	0	0
0	0	0	0	0	0
0	0	0	0	0	0
0	0	0	0	0	0
0	1	1	0	0	0
0	0	0	0	0	0
0	0	0	0	0	0
0	0	0	0	0	0
0	0	0	0	0	0
0	0	0	0	0	0
0	0	0	0	0	0
0	0	0	0	0	0
0	0	0	0	0	0
0	0	0	0	0	0
0	0	0	1	0	1
0	0	0	0	0	0
0	1	0	1	0	1
0	0	0	0	0	0
0	0	0	0	0	0
0	0	0	0	0	0
0	1	1	0	0	0
0	0	0	0	0	0
0	0	0	1	0	0
0	0	0	0	0	0

0	0	0	0	0	0
0	0	0	0	0	0
0	0	0	0	0	0
0	0	0	0	0	0
0	0	0	0	0	1
0	0	0	0	0	0
0	0	0	0	0	0
0	0	0	1	0	0
0	0	0	0	0	0
0	0	0	0	0	0
0	0	0	0	0	0
0	1	0	0	0	0
0	0	0	0	0	0
0	0	0	0	0	0
0	0	0	0	0	0
0	0	1	0	0	0
0	0	0	0	0	0
0	0	0	0	0	0
0	1	0	0	0	0
0	0	0	0	0	0
0	1	0	1	0	0
0	0	0	0	0	0
0	0	0	0	0	0
0	0	0	0	0	0
0	0	0	0	0	0
0	0	0	0	0	0
0	0	0	0	0	0
0	0	0	0	0	0
0	0	1	0	0	0
0	0	0	0	0	0
0	0	0	0	0	0
0	0	0	0	0	0
0	0	0	0	0	0
0	0	0	1	0	0
0	0	0	0	0	0
0	0	0	0	0	0
0	0	0	0	0	0
0	0	1	0	0	0
0	0	0	0	0	0
0	1	1	1	1	0
0	0	0	0	0	0
0	0	0	0	0	0
0	1	0	1	0	0
0	0	0	0	0	0
0	0	0	0	0	0
0	1	0	1	0	0
0	0	0	0	0	0

0	0	0	0	0	0
0	0	0	0	0	0
0	0	0	0	0	0
0	1	1	0	0	0
0	0	0	0	0	0
0	0	0	0	0	0
0	0	0	0	0	0
0	0	0	0	0	0
0	0	0	0	0	0
0	1	0	1	0	0
0	1	0	0	0	0
0	0	0	1	0	1
0	0	0	0	0	0
0	0	0	0	0	0
0	0	0	0	0	0
0	0	0	0	0	0
0	0	0	0	0	0
0	0	0	0	0	0
0	0	0	0	0	0
0	0	0	0	0	0
0	0	0	0	0	0
0	0	0	1	0	0
0	0	0	0	0	0
0	0	0	0	0	0
0	1	0	1	0	1
0	0	0	0	0	0
0	0	0	0	0	0
1	0	1	0	0	0
0	0	0	0	0	0
0	0	0	0	0	0
0	0	0	1	0	0
0	0	0	0	0	0
0	0	0	0	0	0
0	0	0	0	0	0
0	0	0	0	0	0
0	0	0	0	0	0
0	0	0	0	0	0
0	0	0	1	0	1
0	0	0	0	0	0
0	0	0	0	0	0
0	0	0	0	0	0
0	0	0	0	0	0
0	0	0	0	0	0
0	0	0	0	0	0
0	0	0	0	0	0
0	0	0	0	0	0
0	0	0	0	0	0

0	0	1	0	0	0
0	0	0	0	0	0
0	0	0	0	0	0
0	0	0	0	0	0
0	1	0	1	0	1
0	0	0	0	0	0
0	0	0	0	0	0
0	0	0	0	0	0
0	0	0	0	0	0
0	0	0	0	0	0
0	0	0	0	0	0
0	1	0	0	0	0
0	0	0	0	0	0
0	0	0	0	0	0
0	0	0	0	0	0
0	0	0	0	0	0
0	0	0	0	0	0
0	0	0	0	0	0
0	0	0	0	0	0
0	0	0	0	0	0
0	0	0	0	0	0
0	0	0	0	0	0
0	0	0	0	0	0
0	0	0	0	0	0
0	0	0	0	0	0
0	0	0	0	0	1
0	0	0	0	0	0
0	0	0	0	0	0
0	0	0	0	0	0
0	0	0	0	0	0
0	0	1	0	0	0
0	0	0	0	0	0
0	0	0	0	0	0
0	0	0	0	0	0
0	0	0	0	0	0
0	0	0	0	0	0
0	0	0	0	0	0
0	0	0	0	0	0
0	0	0	0	0	0
0	0	0	0	0	0
0	0	0	0	0	0
0	0	0	0	0	0
0	0	0	0	0	0
0	0	0	0	0	0
0	0	0	0	0	0
0	0	0	0	0	0
0	0	0	0	0	0

0	0	0	0	0	0
0	0	0	0	0	0
0	0	0	0	0	0
0	0	0	0	0	0
0	0	0	0	0	0
0	0	0	0	0	0
0	0	1	0	0	0
0	0	0	0	0	0
0	0	0	0	0	0
0	0	0	0	0	0
0	0	0	0	0	0
0	0	0	0	0	0
0	0	0	0	0	0
0	0	0	0	0	0
0	0	1	0	0	0
0	0	0	0	0	0
0	0	0	0	0	0
0	0	1	0	1	0
0	0	0	0	0	0
0	0	0	0	0	0
0	1	1	0	0	0
0	0	0	0	0	0
0	0	0	0	0	0
0	0	0	0	0	0
0	0	0	0	0	0
0	1	1	0	0	0
0	0	0	0	0	0
0	0	0	0	0	0
0	0	0	0	0	0
0	0	0	0	0	0
0	0	0	0	0	0
0	0	0	0	0	0
0	1	0	0	0	0
0	0	1	0	0	0
0	0	0	0	0	0
0	0	0	0	0	0
0	0	0	0	0	0
0	0	0	0	0	0
0	0	0	0	0	0
0	0	0	0	0	0
0	0	0	0	0	0
0	0	0	0	0	0
0	0	0	0	0	0
0	0	0	0	0	0
0	0	0	0	0	0
0	0	0	0	0	0
0	0	0	0	0	0

0	0	0	0	0	0
0	0	0	1	0	1
0	0	0	0	0	0
0	0	0	0	0	0
0	0	0	0	0	0
0	0	0	0	0	0
0	0	0	0	0	0
0	0	0	0	0	0
0	0	0	0	0	0
0	0	0	0	0	0
0	0	0	0	0	0
0	0	0	0	0	0
0	0	0	0	0	1
0	0	0	0	0	0
0	0	0	0	0	0
0	0	0	0	0	0
0	0	0	0	0	0
0	0	0	0	0	0
0	0	0	0	0	0
0	0	0	0	0	0
0	0	0	1	0	0
0	0	0	0	0	0
0	0	0	1	0	0
0	0	0	0	0	0
0	0	0	0	0	0
0	0	0	0	0	0
0	0	0	0	0	0
0	0	0	0	0	0
0	0	0	0	0	1
0	0	0	0	0	0
0	0	1	0	0	0
0	0	0	0	0	0
0	1	1	1	0	0
0	0	0	0	0	0
0	0	0	0	0	0
0	0	0	0	0	0
0	0	0	0	0	0
0	0	0	0	0	0
0	0	0	0	0	0
0	0	0	0	0	0
0	0	0	0	0	0
0	0	0	0	0	0
0	0	0	0	0	0
0	1	0	1	0	0
0	0	0	0	0	0
0	1	0	0	0	0
0	0	0	0	0	0

0	0	0	0	0	0
0	0	1	0	0	0
0	0	0	0	0	0
0	0	0	0	0	0
0	0	0	0	0	0
0	0	0	0	0	0
0	0	0	0	0	0
0	1	0	1	0	0
0	0	0	0	0	0
0	0	0	0	0	0
0	0	0	0	0	0
0	0	0	0	0	0
0	0	0	0	0	0
0	0	0	0	0	0
0	0	0	0	0	0
0	0	1	0	1	0
0	0	0	0	0	0
0	0	0	0	0	0
0	0	0	0	0	0
0	0	0	0	0	0
0	0	0	0	0	0
0	0	0	0	0	0
0	0	0	0	0	0
0	0	0	0	0	0
0	0	0	0	0	0
0	0	0	0	0	0
0	0	0	0	0	0
0	0	0	0	0	0
0	0	0	0	0	0
0	1	1	0	0	0
0	0	0	0	0	0
0	0	0	0	0	0
0	0	0	0	0	0
0	0	0	0	0	0
0	0	0	0	0	0
0	0	0	0	0	0
0	0	0	0	0	0
0	0	0	0	0	0
0	0	0	0	0	0
0	0	0	0	0	0
0	0	0	0	0	0
0	0	0	0	0	0
0	1	0	1	0	1
0	0	0	0	0	0
0	0	0	0	0	0
0	0	0	0	0	0
0	1	0	0	0	0

1	0	1	0	0	0
0	0	0	0	0	0
0	0	0	0	0	0
0	0	0	0	0	0
0	0	0	0	0	0
0	0	0	0	0	0
0	0	0	0	0	0
0	0	0	0	0	0
0	0	1	0	0	0
0	0	0	0	0	0
0	0	0	0	0	0
0	0	0	0	0	0
0	0	0	0	0	0
0	0	0	0	0	0
0	0	0	0	0	0
0	0	0	0	0	0
0	0	0	0	0	0
0	0	0	0	0	0
0	0	0	0	0	0
0	0	0	0	0	0
0	0	0	0	0	0
0	0	0	1	0	0
0	0	0	0	0	1
0	0	0	0	0	0
0	0	0	0	0	0
0	0	0	0	0	0
0	0	0	0	0	0
0	0	0	0	0	0
0	1	0	1	0	0
0	0	0	0	0	0
0	0	1	0	0	0
0	0	0	0	0	0
0	0	0	0	0	0
0	0	0	0	0	0
0	0	0	0	0	0
0	0	0	0	0	0
0	0	0	0	0	0
0	0	0	0	0	0
0	0	0	0	0	0
0	0	0	0	0	0
0	1	1	0	0	0
0	1	0	0	0	0
0	0	0	0	0	0
0	0	0	0	0	0
0	0	0	0	0	0
0	0	1	0	1	0
0	0	0	0	0	0

0	0	0	0	0	0
0	1	1	0	1	0
0	0	0	0	0	0
0	0	0	0	0	0
0	0	0	0	0	0
0	0	0	0	0	0
0	0	0	0	0	0
0	0	0	0	0	0
0	0	0	0	0	0
0	0	0	0	0	0
0	0	0	0	0	0
0	0	0	0	0	0
0	0	0	1	0	1
0	1	0	0	0	0
0	1	1	0	1	0
0	0	0	0	0	0
0	0	0	0	0	0
0	0	0	0	0	0
0	0	1	0	0	0
0	0	0	0	0	0
0	0	0	0	0	0
0	0	0	0	0	0
0	0	1	0	0	0
0	0	0	0	0	0
0	0	0	0	0	0
1	1	1	0	0	0
0	0	0	0	0	0
0	0	0	0	0	0
0	0	0	0	0	0
0	1	0	0	0	0
0	0	0	0	0	0
0	0	0	0	0	0
0	0	0	1	0	0
0	0	0	0	0	0
0	0	0	0	0	0
0	0	0	0	0	0
0	0	0	0	0	0
0	0	0	0	0	0
0	0	0	0	0	0
0	0	0	0	0	0
0	0	0	0	0	0
0	0	0	0	0	0
0	0	0	0	0	0
0	0	0	0	0	0
0	0	0	0	0	0
0	0	0	0	0	0
0	0	0	0	0	0

0	0	0	0	0	0
0	0	0	0	0	0
0	0	0	0	0	0
0	0	0	0	0	0
0	0	0	0	0	0
0	0	0	0	0	0
0	0	0	0	0	0
0	0	0	0	0	0
0	0	0	0	0	0
0	0	0	0	0	0
0	0	0	0	0	0
0	0	0	0	0	0
0	0	0	0	0	0
0	0	1	0	0	0
0	0	0	0	0	0
0	0	1	0	0	0
0	0	0	0	0	0
0	0	0	0	0	0
0	0	0	0	0	0
0	0	0	0	0	0
0	0	0	0	0	0
0	0	0	0	0	0
0	0	0	0	0	0
0	0	0	0	0	0
0	0	0	0	0	0
0	0	0	0	0	0
0	0	0	0	0	0
0	0	0	0	0	0
0	0	0	0	0	0
0	0	0	0	0	0
0	0	0	0	0	0
0	0	0	0	0	0
0	0	0	0	0	1
0	0	0	0	0	0
0	0	0	0	0	0
0	0	0	0	0	0
0	0	0	0	0	0
0	0	0	0	0	0
0	0	0	0	0	0
0	0	0	0	0	0
0	0	0	0	0	0
0	0	0	0	0	0
0	0	0	0	0	0
0	0	0	0	0	0
0	0	1	0	0	0
0	0	0	0	0	0
0	0	0	0	0	0

0	0	0	0	0	0
0	0	0	0	0	0
0	0	0	0	0	0
0	0	0	0	0	0
0	0	0	0	0	0
0	0	0	0	0	0
0	0	0	0	0	0
0	0	0	0	0	0
0	0	1	0	0	0
0	0	1	0	0	0
0	1	1	0	0	0
0	0	0	0	0	0
0	0	0	0	0	0
0	0	0	0	0	0
0	0	0	0	0	0
0	0	0	0	0	0
0	0	0	0	0	0
0	0	0	0	0	0
0	0	0	0	0	0
0	0	0	0	0	0
0	0	0	0	0	0
0	0	0	0	0	0
0	0	0	0	0	0
0	0	0	1	0	0
0	0	0	0	0	0
0	0	0	0	0	0
0	0	0	0	0	0
0	0	0	0	0	0
0	0	1	0	0	0
0	0	0	0	0	0
0	0	0	0	0	0
0	0	0	0	0	0
0	0	0	0	0	0
0	0	1	0	0	0
0	0	0	0	0	0
0	0	0	0	0	0
0	0	0	0	0	0
0	0	0	0	0	0
0	0	0	0	0	0
0	0	1	0	0	0
0	0	0	0	0	0
0	0	0	0	0	0
0	0	0	0	0	0
0	0	0	0	0	0
0	0	0	0	0	0
0	0	0	0	0	0
0	0	0	0	0	0

0	0	0	0	0	0
0	0	0	0	0	0
0	0	0	0	0	0
0	0	0	0	0	0
0	0	1	0	0	0
0	0	0	0	0	0
0	0	0	1	0	0
0	0	0	0	0	0
0	0	0	0	0	0
0	0	0	0	0	0
0	1	0	1	0	0
0	0	0	0	0	0
0	1	1	0	0	0
0	0	0	0	0	0
0	0	0	0	0	0
0	0	0	0	0	0
0	0	1	0	0	0
0	0	0	0	0	0
0	0	0	0	0	0
0	0	1	0	1	0
0	0	0	0	0	0
0	0	0	0	0	0
0	0	0	0	0	0
0	0	1	0	0	1
0	0	0	0	0	0
0	0	0	0	0	0
0	1	0	0	0	0
0	0	0	0	0	0
0	0	0	0	0	0
0	0	0	0	0	0
0	0	0	0	0	0
0	0	0	0	0	0
0	0	0	0	0	0
0	0	0	0	0	0
0	0	0	0	0	0
0	0	0	0	0	0
0	0	0	0	0	0
0	0	0	0	0	0
0	0	0	0	0	0
0	0	0	0	0	1
0	0	0	0	0	0
0	0	0	0	0	0
0	0	0	0	0	0
0	0	0	0	0	0
0	0	0	0	0	0
0	0	0	0	0	0
0	0	0	0	0	0

0	0	0	0	0	0
0	0	0	0	0	0
0	0	1	0	1	1
0	0	0	0	0	0
0	0	1	0	0	0
0	0	0	0	0	0
0	0	0	0	0	0
0	0	0	0	0	0
0	0	0	0	0	0
1	1	0	0	1	0
0	0	0	0	0	0
0	0	0	0	0	0
0	0	0	0	0	0
0	0	0	0	0	0
0	0	0	0	0	0
